# Projectability disentanglement for accurate and automated electronic-structure Hamiltonians

**DOI:** 10.1038/s41524-023-01146-w

**Published:** 2023-11-03

**Authors:** Junfeng Qiao, Giovanni Pizzi, Nicola Marzari

**Affiliations:** 1 Theory and Simulations of Materials (THEOS), and National Centre for Computational Design and Discovery of Novel Materials (MARVEL), École Polytechnique Fédérale de Lausanne, 1015 Lausanne, Switzerland; 2 Laboratory for Materials Simulations (LMS), Paul Scherrer Institut (PSI), CH-5232 Villigen PSI, Switzerland

**Keywords:** Electronic structure, Computational methods

## Abstract

Maximally-localized Wannier functions (MLWFs) are broadly used to characterize the electronic structure of materials. Generally, one can construct MLWFs describing isolated bands (e.g. valence bands of insulators) or entangled bands (e.g. valence and conduction bands of insulators, or metals). Obtaining accurate and compact MLWFs often requires chemical intuition and trial and error, a challenging step even for experienced researchers and a roadblock for high-throughput calculations. Here, we present an automated approach, projectability-disentangled Wannier functions (PDWFs), that constructs MLWFs spanning the occupied bands and their complement for the empty states, providing a tight-binding picture of optimized atomic orbitals in crystals. Key to the algorithm is a projectability measure for each Bloch state onto atomic orbitals, determining if that state should be kept identically, discarded, or mixed into the disentanglement. We showcase the accuracy on a test set of 200 materials, and the reliability by constructing 21,737 Wannier Hamiltonians.

## Introduction

In periodic crystals, the electronic structure is usually described using one-particle Bloch wavefunctions. While choosing a basis set that is also periodic to describe these wavefunctions can often be beneficial, an alternative approach is to adopt localized orbitals in real space. One such choice of orbitals are Wannier functions (WFs), that can be obtained by Fourier transforming the periodic wavefunctions from reciprocal to real space. WFs are not unique, as they depend on the choice of the gauge (i.e., the choice of the phases of the wavefunctions) at each point in the Brillouin zone (BZ). Maximally-localized Wannier functions (MLWFs)^1–4^ are obtained by a gauge choice that is optimized to provide the most localized set of WFs, i.e., those that minimize the sum of their quadratic spread in real space^1^. Having a very localized representation of the electronic structure not only provides an insightful analysis of chemical bonding in solids, but also brings a formal connection between the MLWF centers and the modern theory of electric polarization^5^. Moreover, the real-space locality of MLWF allows for accurate and fast interpolation of physical operators^6^, enabling calculations of material properties that require dense samplings of the BZ, such as Fermi surface, orbital magnetization^7^, anomalous Hall conductivity^8,9^, and spin Hall conductivity^10^, to name a few. Practically, one obtains MLWFs starting from a set of Bloch wavefunctions, calculated e.g., from density-functional theory (DFT). Often, these Bloch states are projected onto some localized orbitals (usually chosen by the user) to generate initial guesses for MLWFs. In an insulator, by minimizing the spread functional^1^ which measures localization, one can obtain a set of MLWFs, i.e., “Wannierize” a material. The Wannierization contains an additional disentanglement step^2^ if the target Bloch states are not isolated from other band manifolds. For such entangled bands—metals or the conduction bands of insulators—one needs to first identify the relevant Bloch states that will be used to construct MLWFs, and then mix or “disentangle” these from all the Bloch states^2^. Practically, the choices for the initial projections and states to be disentangled substantially influence the shape and the quality of the final MLWFs.

In recent years, a lot of effort has been devoted to obtaining high-quality MLWFs and automate the Wannierization procedure. Focus of the research can be categorized into the following classes: (a) minimization algorithms, such as: the symmetry-adapted WF method that adds constraints to impose the symmetries of the resulting WFs^11^; the simultaneous diagonalization algorithm that directly minimizes the spread functional for an isolated (or “Γ-only”) system^12^; the partly-occupied WF method, where the total spread is directly minimized in one step^13,14^, rather than performing a two-step minimization for its gauge-invariant and gauge-dependent parts as in the standard procedure^2^; or the variational formulation, that combines single-step optimization with manifold optimization to make the minimization algorithm more robust^15^; (b) different forms of the spread functional, such as the selectively localized WFs (SLWFs) for which only a subset of WFs of interest are localized and a penalty term is added to constrain the position of the WF centers^16^, or the spread-balanced WF method, that adds a penalty term to distribute the spread as uniformly as possible among all WFs^17^; (c) targeting a subset of orbitals, e.g. SLWF for a subset of MLWFs^16^ or the optimized projection functions method where starting projections for the Wannierization are generated from a larger group of initial ones^18^; (d) matrix manifold algorithms instead of projection methods to construct a smooth gauge in a non-iterative way^19,20^; (e) basis-vector decomposition of the density matrix, e.g. the selected columns of the density matrix (SCDM) algorithm^21,22^, that starts from the density matrix of the system and uses QR decomposition with column pivoting (QRCP) to automatically generate an optimal set of basis vectors from the columns of the density matrix.

At the same time, high-throughput (HT) calculations have become increasingly popular for materials discovery and design. Calculations and results managed by workflow engines are collected into databases of original calculations, such as the Materials Project^23^, AFLOW^24^, OQMD^25^, CMR^26^, and the Materials Cloud^27^, or aggregated, as in NOMAD^28^. Thanks to recent research advances on Wannierization algorithms, it starts now to be possible to run HT Wannierizations for many materials and generate tight-binding (TB) models that reliably describe their physics. So far, several attempts have been made in this direction. Reference^29^ gathered 195 Wannier TB Hamiltonians and applied post-processing symmetrization to study strained III-V semiconductor materials. Reference^30^ implemented the SCDM algorithm and designed a protocol to determine automatically the remaining free parameters of the algorithm; this protocol, implemented into automated workflows, was verified to work well for band interpolations on a set of 200 structures (metals, or valence and conduction bands of insulators) and 81 insulators (valence bands only). Reference^31^ accumulated a Wannier TB Hamiltonian database of 1771 materials using the standard hydrogenic orbital projections. However, there are still several challenges for an accurate and automated HT Wannierization, some of which might be more relevant depending on the research goal and the specific property to compute: MLWFs should be able to faithfully represent the original band structure, often (e.g., for transport properties) at least for those bands close to the Fermi energy; MLWFs should resemble the physically intuitive atomic orbitals for solids that would enter into Bloch sums; the algorithm should be fully and reliably automated and the implementation should be efficient for HT calculations.

To overcome the challenges mentioned above, in this paper we present an approach for automated Wannierization. First, we choose physically-inspired orbitals as initial projectors for MLWFs, that is, the pseudo-atomic orbitals (PAOs) from pseudopotentials^32^. Then, for each state $\left\vert n{{{\bf{k}}}}\right\rangle$ (*n* is the band index, **k** is the Bloch quasi-momentum) we decide if it should be dropped, kept identically, or thrown into the disentanglement algorithm depending on the value of its projectability onto the chosen set of PAOs, replacing the standard disentanglement and frozen manifolds based only on energy windows. This approach naturally and powerfully targets the TB picture of atomic orbitals in crystals, as it will also become apparent from our results. Moreover, we fully automate this approach and implement it in the form of open-source AiiDA^33–35^ workflows. To assess its effectiveness and precision, we compare the quality of the band interpolation and the locality of the Wannier Hamiltonians generated with the present approach, which we name as projectability-disentangled Wannier functions (PDWFs), with the results from the SCDM algorithm^30^. Statistics from 200 materials demonstrate that PDWFs are more localized and more atomic-like, and the band interpolation is accurate at the meV scale. Furthermore, to demonstrate the reliability and automation of our method and workflows, we carry out a large-scale high-throughput Wannierization of 21,737 materials from the Materials Cloud^27,36^.

To set the context for the following paragraphs, here we briefly summarize the notations for WFs; a detailed description can be found in refs. ^1–3^. WFs $\left\vert {w}_{n{{{\bf{R}}}}}\right\rangle$ are unitary transformations of Bloch wavefunctions $\left\vert {\psi }_{m{{{\bf{k}}}}}\right\rangle$, given by

1 $$\left\vert {w}_{n{{{\bf{R}}}}}\right\rangle =\frac{V}{{(2\pi )}^{3}}{\int}_{{{{\rm{BZ}}}}}{{{\rm{d}}}}{{{\bf{k}}}}{{{{\rm{e}}}}}^{-{{{\rm{i}}}}{{{\bf{k}}}}\cdot {{{\bf{R}}}}}\mathop{\sum }\limits_{m=1}^{J\,{{\mathrm{or}}}\,{J}_{{{{\bf{k}}}}}}\left\vert {\psi }_{m{{{\bf{k}}}}}\right\rangle {U}_{mn{{{\bf{k}}}}},$$

where **k** and **R** are the Bloch quasi-momentum in the BZ and a real-space lattice vector, respectively; *m* is the band index, and *n* is the Wannier-function index (running from 1 to the number of WFs *J*). For an isolated group of bands, *J* is equal to the number of bands, and the *U*_*mn***k**_ are unitary matrices; for entangled bands, the number of bands considered at each *k*-point is *J*_**k**_ ≥ *J*, and the *U*_*mn***k**_ are semi-unitary rectangular matrices. MLWFs are the minimizers of the quadratic spread functional^1^

2 $$\Omega =\mathop{\sum }\limits_{n=1}^{J}\left[\langle {w}_{n{{{\bf{0}}}}}| {{{{\bf{r}}}}}^{2}| {w}_{n{{{\bf{0}}}}}\rangle -{\left\vert \langle {w}_{n{{{\bf{0}}}}}| {{{\bf{r}}}}| {w}_{n{{{\bf{0}}}}}\rangle \right\vert }^{2}\right].$$

Since Eq. (2) is a minimization problem with multiple local minima, initial guesses for *U*_*mn***k**_ substantially influence the optimization path and the final minimum obtained. In order to target the most localized and chemically appealing solution, ref. ^1^ used hydrogenic wavefunctions $\left\vert {g}_{n}\right\rangle$ (i.e., analytic solutions of the isolated hydrogenic Schrödinger equation) to provide a set of sensible initial guesses $\left\vert {\phi }_{n{{{\bf{k}}}}}\right\rangle$, after projection on the space defined by the relevant Bloch states:

3 $$\left\vert {\phi }_{n{{{\bf{k}}}}}\right\rangle =\mathop{\sum }\limits_{m=1}^{J\,{{\mathrm{or}}}\,{J}_{{{{\bf{k}}}}}}\left\vert {\psi }_{m{{{\bf{k}}}}}\right\rangle \langle {\psi }_{m{{{\bf{k}}}}}| {g}_{n}\rangle .$$

The projection matrices *A*_*mn***k**_ = 〈*ψ*_*m***k**_∣*g*_*n*_〉, after Löwdin orthonormalization^37^, form the initial guesses for *U*_*mn***k**_. We underline that while the gauge of Bloch wavefunctions $\left\vert {\psi }_{m{{{\bf{k}}}}}\right\rangle$ is arbitrary, Eq. (3) is invariant to such gauge freedom: suppose $\left\vert {\psi }_{i{{{\bf{k}}}}}^{{\prime} }\right\rangle$ are also solutions of the electronic structure problem, then $\left\vert {\psi }_{i{{{\bf{k}}}}}^{{\prime} }\right\rangle$ are related to $\left\vert {\psi }_{m{{{\bf{k}}}}}\right\rangle$ by some unitary matrices $\left\vert {\psi }_{i{{{\bf{k}}}}}^{{\prime} }\right\rangle ={\sum }_{m}\left\vert {\psi }_{m{{{\bf{k}}}}}\right\rangle {U}_{mi{{{\bf{k}}}}}$; thus $\left\vert {\phi }_{n{{{\bf{k}}}}}\right\rangle ={\sum }_{m}\left\vert {\psi }_{m{{{\bf{k}}}}}\right\rangle \langle {\psi }_{m{{{\bf{k}}}}}| {g}_{n}\rangle ={\sum }_{m}{\sum }_{i}\left\vert {\psi }_{i{{{\bf{k}}}}}^{{\prime} }\right\rangle {U}_{im{{{\bf{k}}}}}^{* }{U}_{mi{{{\bf{k}}}}}\langle {\psi }_{i{{{\bf{k}}}}}^{{\prime} }| {g}_{n}\rangle ={\sum }_{i}\left\vert {\psi }_{i{{{\bf{k}}}}}^{{\prime} }\right\rangle \langle {\psi }_{i{{{\bf{k}}}}}^{{\prime} }| {g}_{n}\rangle$ does not depend on the gauge of Bloch wavefunctions, where superscript * denotes conjugate transpose. For entangled bands, the “standard” disentanglement approach^2^ uses energy windows to choose the disentanglement and frozen manifolds: (a) an (outer) disentanglement window that includes a large set of Bloch states, which can be mixed together to obtain a smaller disentangled manifold; (b) an (inner) frozen window that specifies a smaller set of Bloch states (often states around Fermi energy) which are kept unchanged in the final disentangled manifold.

Since in the following sections the present results are compared with SCDM, we also summarize the SCDM procedure here. The SCDM method^21^ starts from the real-space density matrix $\langle {{{\bf{r}}}}| {P}_{{{{\bf{k}}}}}| {{{{\bf{r}}}}}^{{\prime} }\rangle$ where ${P}_{{{{\bf{k}}}}}=\mathop{\sum }\nolimits_{m = 1}^{{J}_{{{{\bf{k}}}}}}\left\vert {\psi }_{m{{{\bf{k}}}}}\right\rangle \left\langle {\psi }_{m{{{\bf{k}}}}}\right\vert$, and uses QR factorization with column pivoting (QRCP) to decompose $\langle {{{\bf{r}}}}| {P}_{{{{\bf{k}}}}}| {{{{\bf{r}}}}}^{{\prime} }\rangle$ into a set of localized real-space orbitals, thanks to the near-sightedness principle^38,39^ stating that the matrix elements $\langle {{{\bf{r}}}}| {P}_{{{{\bf{k}}}}}| {{{{\bf{r}}}}}^{{\prime} }\rangle$ decay exponentially with the distance between two points **r** and ${{{{\bf{r}}}}}^{{\prime} }$ in insulating systems. While storing the full $\langle {{{\bf{r}}}}| {P}_{{{{\bf{k}}}}}| {{{{\bf{r}}}}}^{{\prime} }\rangle$ is memory intensive (it has size *N*_**r**_ × *N*_**r**_, where *N*_**r**_ is the number of real-space grid points), one can equivalently decompose the matrix formed by the real-space Bloch wavefunctions ${\Psi }_{{{{\bf{k}}}}}^{* }={[{\psi }_{1{{{\bf{k}}}}},\ldots ,{\psi }_{{J}_{{{{\bf{k}}}}}{{{\bf{k}}}}}]}^{* }$, which has a smaller size *J*_**k**_ × *N*_**r**_. For periodic systems, often the choice of columns in the QRCP algorithm can be performed using the wavefunctions at the Γ point only (Ψ_Γ_)^40^, and the same column selection is then used for all other *k*-points. For entangled bands, since the density matrix is not continuous across the *k*-points, one can construct a quasi-density matrix (or equivalently a matrix of wavefunctions) $\mathop{\sum }\nolimits_{m = 1}^{{J}_{{{{\bf{k}}}}}}\left\vert {\psi }_{m{{{\bf{k}}}}}\right\rangle f({\varepsilon }_{m{{{\bf{k}}}}})\left\langle {\psi }_{m{{{\bf{k}}}}}\right\vert$, where *f*(*ε*_*m***k**_) is a smooth function of the energy eigenvalues *ε*_*m***k**_, specifying the target energy window for the constructed MLWFs. Often the complementary error function $\frac{1}{2}\,{{{\rm{erfc}}}}(\frac{\varepsilon -\mu }{\sigma })$ is chosen as *f*(*ε*), and the choice of *μ* and *σ* determines the shape of MLWFs, as well as band-interpolation quality. Using projectability, defined later in Eq. (5), *μ* and *σ* can be automatically chosen, thus automating the Wannierization process^30^.

## Results

### Pseudo-atomic-orbital projections

In addition to the hydrogenic orbitals discussed above, alternative starting guesses for the Wannierization can be used. For instance, in pseudopotential plane-wave methods, PAOs are localized orbitals originating from the pseudopotential generation procedure^32^. In this procedure, for each element, atomic wavefunctions of an isolated atom are pseudized to remove the radial nodes and are localized functions around the atom; spherical harmonics with well-defined angular-momentum character (*s*, *p*, *d*, or *f*) are chosen for their angular dependency. Then, the PAOs are summed over lattice points with appropriate phases to obtain Bloch sums, Fourier transformed to a plane-wave basis, Löwdin-orthonormalized, and finally taken as the projectors for initial projections. PAOs are commonly used for analyzing the orbital contributions to band structures, as the basis set for non-iterative construction of TB Hamiltonians^32^, or as projectors in DFT+Hubbard calculations^41^.

In order to understand the contribution of each orbital $\left\vert {g}_{n}\right\rangle$ to a Bloch state $\left\vert {\psi }_{m{{{\bf{k}}}}}\right\rangle$, we define a measure of projectability as the square of the inner product between $\left\vert {\psi }_{m{{{\bf{k}}}}}\right\rangle$ and $\left\vert {g}_{n}\right\rangle$:

4 $${p}_{nm{{{\bf{k}}}}}={\left\vert \langle {g}_{n}| {\psi }_{m{{{\bf{k}}}}}\rangle \right\vert }^{2};$$

the projectability of $\left\vert {\psi }_{m{{{\bf{k}}}}}\right\rangle$ onto all PAOs is then defined as

5 $${p}_{m{{{\bf{k}}}}}=\mathop{\sum}\limits_{n}{p}_{nm{{{\bf{k}}}}}.$$

**Fig. 1:**
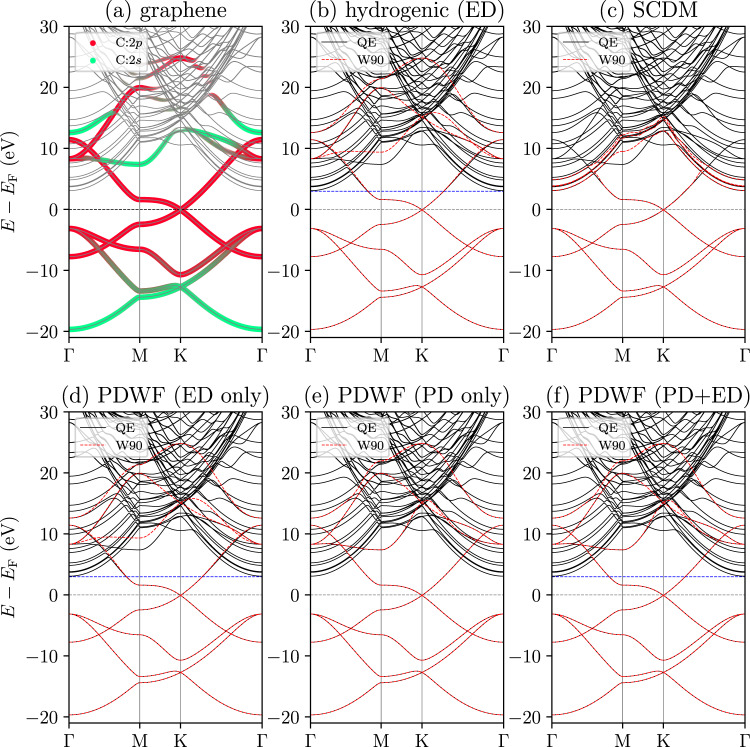
Comparisons of graphene band structures interpolated using different methods. **a** DFT band structure, shown as grey lines. The colored dots represent the projectabilities onto carbon 2*s* (green) and 2*p* (red) orbitals. The size of each dot is proportional to the total projectability *p*_*m***k**_ of the band *m* at *k*-point **k**; see Eq. (5). For a detailed plot of total projectability, see Supplementary Fig. 1. Comparisons of the original and the Wannier-interpolated bands for **b** hydrogenic projections with energy disentanglement (ED), **c** SCDM, **d** PAO with ED, **e** PAO with projectability disentanglement (PD), and **f** PAO with PD+ED. The Fermi energy *E*_F_ (horizontal black dashed line) is at zero; the horizontal blue dashed line denotes the top of the inner energy window, where applicable.

If the projectors $\left\vert {g}_{n}\right\rangle$ are complete for $\left\vert {\psi }_{m{{{\bf{k}}}}}\right\rangle$, then *p*_*m***k**_ = 1. The band projectability is a very useful criterion to identify the orbital character of the bands; this is exemplified in Fig. 1a, where we show the projectability of the bands of graphene onto 2*s* and 2*p* PAOs for carbon. It is immediately apparent how one can easily identify states in the conduction manifold that have a strong 2*p* and 2*s* component.

Compared with the hydrogenic projections, which is the method used by default in Wannier90 ^4^ and its interface code to Quantum ESPRESSO ^42^ (called pw2wannier90.x), PAOs are better adapted to each element since they come exactly from the pseudopotential used in the actual solid-state calculation. Moreover, in pseudopotentials with semicore states, the PAOs for semicores are nodeless and those for valence wavefunctions have at least one radial node (so as to be orthogonal to the semicore states with same angular momentum); thus band projectability can clearly differentiate semicore from valence, making PAOs more convenient than the hydrogenic orbitals, for which the user would need to manually set the correct radial functions for both semicore and valence projectors. For these reasons, we use in this work the PAOs as initial and more accurate projections. If needed, higher energy orbitals not included in the pseudopotential file can be constructed, for example, using solutions of Schrödinger equation under confinement potential^43,44^ (see also discussion in Section “Additional PAOs for high-energy/accuracy interpolation”).

### Projectability disentanglement

As mentioned, the standard disentanglement approach selects the disentanglement and frozen manifolds via two energy windows^2^. We refer to this as energy disentanglement (ED). However, since bands have dispersions across the BZ, a fixed window for all *k*-points might not be an optimal choice. Taking the graphene band structure (Fig. 1a) as an example, the bands with large projectability are mixed with many free-electron bands with zero projectability (grey bands in the conduction region). In this case, one is faced with several options for the outer and inner energy windows, each with different shortcomings: (a) If the inner window includes free-electron bands, the final MLWFs are mixtures of 2*s*, 2*p* atomic orbitals and free-electron bands, delocalizing the resulting MLWFs; (b) if the outer window excludes both the free-electron bands and the atomic-orbital states inside free-electron bands, the WFs lack the anti-bonding part of the bonding/anti-bonding closure^13^, again degrading the localization of WF; (c) if the upper bound of the inner window is set to its maximal allowed value, i.e. the blue dashed line positioned at the minimum of free-electron bands in Fig. 1b, and all the DFT eigenstates are included in the outer window, the disentanglement algorithm^2^ will extract an optimally smooth manifold, at the expense of decreasing the chemical representability of the atomic-orbital bands in the free-electron region; in other words, the MLWFs obtained lose the information of the TB atomic orbitals in this chemical environment (see Fig. 1b).

The graphene case highlights the limitations of the standard ED. Instead, we propose here to select the disentanglement and frozen manifolds based on the projectability *p*_*m***k**_ of each state on the chosen PAOs (i.e., states are selected irrespective of their energy, but rather based on their chemical representativeness). Specifically, we select states based on two thresholds ${p}_{\min }$ and ${p}_{\max }$: (a) If *p*_*m***k**_ < ${p}_{\min }$, the state *ψ*_*m***k**_ is discarded. (b) If *p*_*m***k**_≥${p}_{\max }$, the state *ψ*_*m***k**_ is kept identically. Crucially, all states for which ${p}_{\min }$ ≤*p*_*m***k**_ < ${p}_{\max }$ are thrown in the disentanglement algorithm. Optimal numerical values for ${p}_{\min }$ and ${p}_{\max }$ are discussed later. In the case of graphene, ${p}_{\max }$ identifies the fully atomic-orbital states inside the free-electron bands, while ${p}_{\min }$ removes the fully free-electron bands from the disentanglement process, preventing the mixing of atomic and free-electron states. The two thresholds ${p}_{\min }$ and ${p}_{\max }$ constitute the parameters of the disentanglement process, replacing the four defining energy windows (the lower and upper bounds of the outer and inner energy windows). We note that projectability disentanglement is different from partly-occupied WF^13,14^ in that the latter uses an energy window to select frozen states and minimizes the total spread functional directly, while projectability disentanglement selects the localized states using projectability instead of a constant energy window across *k*-points. In fact, one can combine projectability disentanglement with a variational formulation^15^ to construct MLWFs by minimizing directly the total spread functional.

Ideally, if PAOs were always a complete set to describe valence and near-Fermi-energy conduction bands, the PD would select the most relevant Bloch states and accurately interpolate these DFT bands. However, since the PAOs are fixed orbitals from isolated single-atom calculations for each element, if the chemical environment in the crystal structure is significantly different from that of pseudopotential generation, then the total projectability *p*_*m***k**_ might be smaller than 1 for bands around the conduction band minimum (CBM) or even for valence bands. In such cases, one solution is to increase the number of PAOs, i.e., adding more projectors with higher angular momentum, as we will discuss in Section “Additional PAOs for high-energy/accuracy interpolation”. However, since one almost always wants to correctly reproduce valence bands (plus possibly the bottom of the conduction) but at the same time keep the Wannier Hamiltonian small for computational reasons, we suggest to additionally freeze all the states that sit below the Fermi energy in metals (or below the CBM for insulators) and also those a few eV above (typically, 2 eV or so). Such a combination of PD+ED gives accurate interpolation of bands below and around the Fermi energy (or band edges for insulators), as well as maximally restoring the atomic-orbital picture.

We stress here that, even if we call the resulting Wannier functions PDWFs for clarity, our optimal suggestion is to always also freeze the states in the energy window mentioned above, as we discuss in the next sections.

### Comparisons of four prototypical materials

We choose four prototypical materials to discuss the present method: graphene, silicon, copper, and strontium vanadate (SrVO_3_). Graphene is a difficult case where atomic-orbital states highly mix with free-electron bands; silicon tests the Wannierization of both valence and conduction bands of an insulator; copper is a test on a metal; and SrVO_3_ represents the class of (metallic) perovskites. We compare the shapes, centers, and spreads of the resulting MLWFs using the five methods mentioned earlier: hydrogenic projection with ED (i.e., the standard approach), SCDM, PAO projection with ED, PAO projection with PD, and PAO projection with PD+ED.

**Fig. 2:**
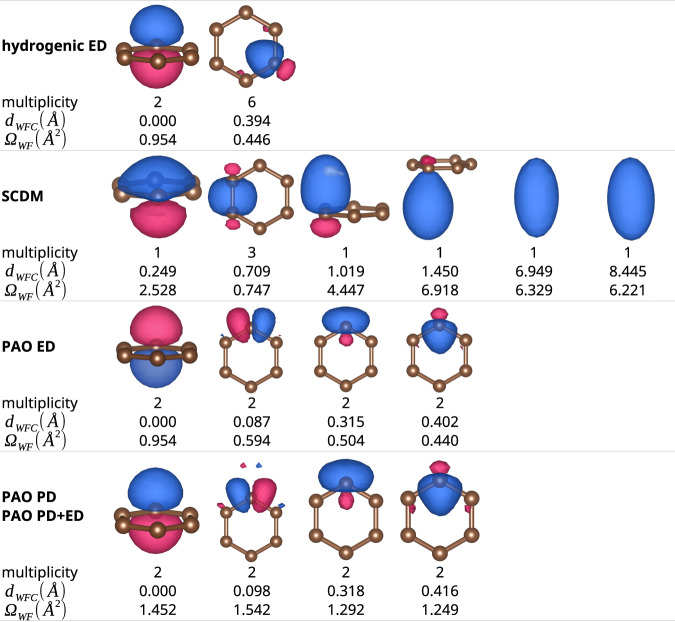
Graphene MLWFs: shapes, centers, and spreads obtained using different methods. *d*_WFC_ is the distance of the WF center from the nearest-neighbor atom, and Ω_WF_ is the MLWF spread. The multiplicity is the number of equivalent MLWFs, i.e. having the same *d*_WFC_, Ω_WF_, and shape, but different orientations.

In the case of graphene, the original and interpolated band structures for the five methods discussed are shown in Fig. 1b–f. The blue dashed lines in Fig. 1b, d, f indicate the top of the inner energy window, which is set optimally (and manually) to just below the free-electron bands, to freeze as much as possible the atomic-orbital states but exclude any free-electron state. For PD and PD+ED, we choose ${p}_{\max }$ = 0.85 and ${p}_{\min }$ = 0.02 (we will discuss later on the choice of these thresholds). Comparing Fig. 1d and Fig. 1b, one sees that ED produces similar bands irrespective of using hydrogenic or PAO projection. However, as shown in Fig. 2 (first and third row), the MLWFs for the two cases fall into slightly different minima: MLWFs from hydrogenic projection with ED are *p*_*z*_ and hybridized *s* ± *p* orbitals pointing towards the center of the hexagon, while MLWFs from PAO with ED are *p*_*z*_, *p*_*x*_, and *s* ± *p*_*y*_. This is due to the fact that the PAO projections guide the minimization towards spherical harmonics, while the hydrogenic projections are farther away from such local minimum and the optimization algorithm happens to escape and converge to a better minimum. A possible future work is to introduce more advanced optimization algorithms to improve the convergence of maximal localization. Both the PAO with PD and PAO with PD+ED cases reach the same set of MLWFs, *p*_*z*_, *p*_*x*_, and *s* ± *p*_*y*_, but with larger spreads than the PAO with ED, since the PD and PD+ED freeze more states, giving thus less freedom for maximal localization. Nevertheless, the interpolated bands of the PAO with PD and PAO with PD+ED cases can much better reproduce the atomic-orbital states inside the free-electron bands. Finally, compared to other cases, SCDM includes some free-electron bands, some of which can be even reproduced by the Wannier interpolation. However, in order to follow those free-electron bands, abrupt changes of character and band derivative are needed in the conduction band. As required by Nyquist–Shannon sampling theorem^45^, this results in a denser **k**-space sampling needed to obtain a good interpolation quality. Moreover, the MLWFs are much more delocalized and do not resemble atomic orbitals: as shown in Fig. 2, the last two MLWFs for SCDM are floating away from the graphene 2D lattice, blurring the TB picture of atomic orbitals in solids.

**Fig. 3:**
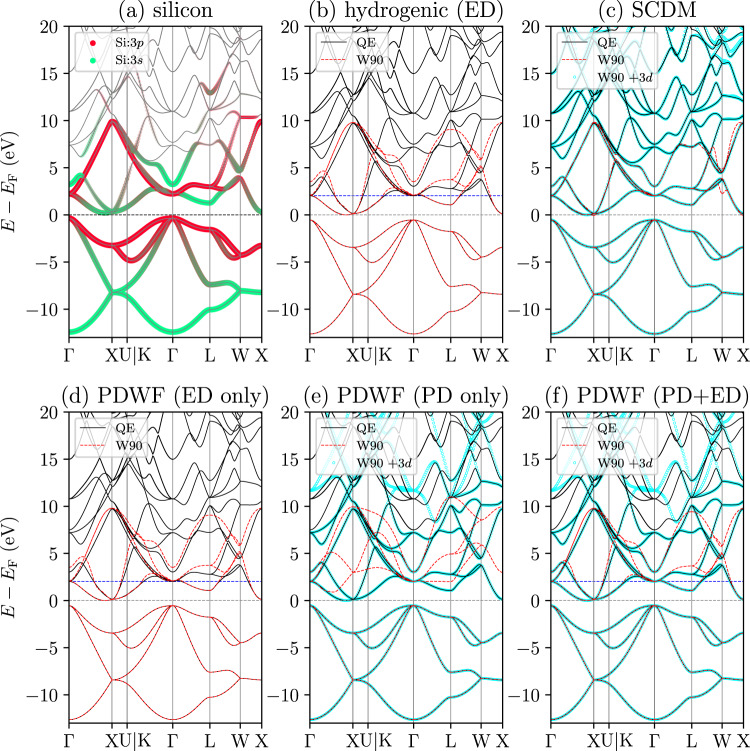
Comparisons of silicon band structures interpolated using different methods. **a** DFT band structure, shown as grey lines. The colored dots represent the projectabilities of silicon 3*s* (green) and 3*p* (red) orbitals. The size of the dot is proportional to the total projectability *p*_*m***k**_ of the band *m* at *k*-point **k**. For a detailed plot of total projectability, see Supplementary Fig. 2. Comparisons of the original and the Wannier-interpolated bands for **b** hydrogenic projections with ED, **c** SCDM, **d** PAO with ED, **e** PAO with PD, and **f** PAO with PD+ED. The CBM (horizontal black dashed line) is at zero; the horizontal blue dashed line denotes the top of the inner energy window, i.e., CBM + 2 eV, where applicable. Note in **c**, **e**, and **f**, the cyan lines with circle markers show the interpolated bands obtained including also 3d orbitals, and consequently increasing the dimensionality of the disentangled manifold. These additional states are beneficial because of the presence of an intrinsic *d* component at the bottom of the conduction manifold, and lead to more accurate band interpolations.

For silicon, the SCDM method obtains four front-bonding and four back-bonding MLWFs, while all other cases lead to atom-centered *s* and *p* MLWFs, as shown in Supplementary Fig. 3. While overall the SCDM bands (Fig. 3c) seem to reproduce relatively better the higher conduction bands, they fail to correctly reproduce the bottom of the conduction band near the X point, induce more wiggles around X and W, and have much larger spreads. Due to the low projectability of Bloch states around X (*p*_*m***k**_ around 0.83), the CBM is not correctly reproduced in the PAO with PD, as these are not frozen in PD with the current choice of ${p}_{\max }$ = 0.95 and ${p}_{\min }$ = 0.01. To explicitly freeze the CBM, ${p}_{\max }$ would need to be lowered below 0.83. However, such kind of decrease will also result in freezing some high-energy conduction bands, degrading the localization. PD+ED overcomes this by explicitly freezing the near-Fermi-energy and low-projectability states at the CBM, but still only freezing those atomic-orbital states in the high-energy conduction bands that possess high projectability (see Fig. 3f), thus improving band interpolation. We note that the lower projectability of silicon CBM is intrinsic to the material—its CBM also includes 3*d* character. Therefore, by adding *d* PAOs, the CBM projectability increases (from 0.83 to 0.99) and one can restore a high-quality band-structure interpolation within the PD method: as shown in Fig. 3e, the low-energy conduction bands are correctly reproduced once we regenerate a silicon pseudopotential including 3*d* PAOs. Therefore, PD is sufficient to obtain an accurate band interpolation if enough PAOs are included (we will also discuss this later in Section “Additional PAOs for high-energy/accuracy interpolation”). For completeness, we show the SCDM interpolation using the regenerated pseudopotential in Fig. 3c: the added *d* PAOs help select a larger manifold thanks to the increased projectability, enabling SCDM to reproduce higher conduction bands, as well as fixing the wrong interpolation at the W point. Moreover, additional PAOs can also benefit ED, since the frozen window can be enlarged to reproduce more states. In general, adding more PAOs improves interpolation quality in cases where the target bands have low projectability, at the price of increased computational cost. PD+ED is a better option for reaching a good interpolation accuracy while keeping the size of the corresponding TB model small.

Results for copper and SrVO_3_ are only shown in the SI (Supplementary Figs. 4, 6, 7 and 9), since the conclusions are the same: PD+ED consistently provides the best interpolation quality among all methods we consider, while not requiring to increase the size of the Hamiltonian model, and results in WFs that resemble atomic orbitals or their hybridization.

### High-throughput verification on 200 materials

In this section we discuss the applicability of the present PDWF method to obtain, in a fully automated way and without user input, WFs for any material. In order to assess quantitatively its performance, we compare it to SCDM, that can also be fully automated (see ref. ^30^).

In all results that follow, we exclude semicore orbitals in both methods, since these low-energy states correspond to almost flat bands and do not play any role in the chemistry of the materials. We compare quantitatively the band interpolation quality between the two methods and the corresponding WF centers and spreads on the 200-structure set used in ref. ^30^ for both occupied and unoccupied bands, totalling 6818 MLWFs for each method. In accordance with refs. ^30,46^, the band interpolation quality is measured by the average band distance,

6 $${\eta }_{\nu }=\sqrt{\frac{\mathop{\sum}\limits_{n{{{\bf{k}}}}}{\tilde{f}}_{n{{{\bf{k}}}}}{({\varepsilon }_{n{{{\bf{k}}}}}^{{{\mbox{DFT}}}}-{\varepsilon }_{n{{{\bf{k}}}}}^{{{\mbox{Wan}}}})}^{2}}{\mathop{\sum}\limits_{n{{{\bf{k}}}}}{\tilde{f}}_{n{{{\bf{k}}}}}}},$$

and the max band distance,

7 $${\eta }_{\nu }^{\max }=\mathop{\max }\limits_{n{{{\bf{k}}}}}\left({\tilde{f}}_{n{{{\bf{k}}}}}\left\vert {\varepsilon }_{n{{{\bf{k}}}}}^{\,{{\mbox{DFT}}}\,}-{\varepsilon }_{n{{{\bf{k}}}}}^{\,{{\mbox{Wan}}}\,}\right\vert \right),$$

where ${\tilde{f}}_{n{{{\bf{k}}}}}=\sqrt{{f}_{n{{{\bf{k}}}}}^{\,{{\mbox{DFT}}}\,}({E}_{{{{\rm{F}}}}}+\nu ,\sigma ){f}_{n{{{\bf{k}}}}}^{\,{{\mbox{Wan}}}\,}({E}_{{{{\rm{F}}}}}+\nu ,\sigma )}$ and *f*(*E*_F_ + *ν*, *σ*) is the Fermi-Dirac distribution. Here *E*_F_ + *ν* and *σ* are fictitious Fermi levels and smearing widths which we choose for comparing a specific range of bands. Since the Wannier TB model describes the low-energy valence electrons, it is expected that the band interpolation deviates from the original in the higher conduction band region. Therefore, the higher *ν* is, the larger *η*_*ν*_ is expected to be. In the following paragraphs, we will use *η*_0_ and *η*_2_ to compare bands below *E*_F_ and *E*_F_ + 2 eV, respectively; *σ* is always fixed at 0.1 eV.

In the Supplementary Section 2.1, we provide comparisons between the Wannier-interpolated bands and the DFT bands for both PDWF and SCDM, their respective band distances, and the Hamiltonian decay plots for each of the 200 materials. We discuss these properties in the following.

### Projectability thresholds and automation

**Fig. 4:**
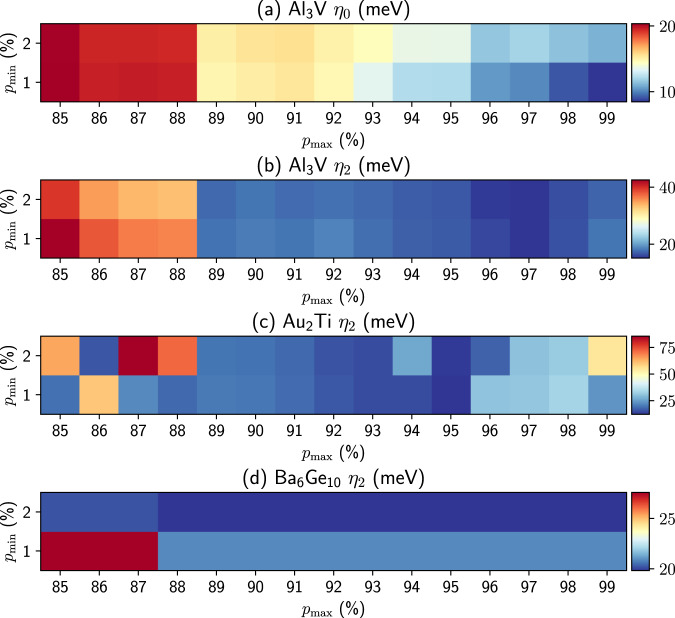
Quality of band interpolations: band distances for different choices of ${p}_{\min }$ and ${p}_{\max }$. **a** *η*_0_ of Al_3_V, **b** *η*_2_ of Al_3_V, **c** *η*_2_ of Au_2_Ti, and **d** *η*_2_ of Ba_6_Ge_10_. Note the color scale is different for each plot.

For PDWF, we set the maximum of the inner window to the Fermi energy + 2 eV for metals, or to the CBM + 2 eV for insulators, to fully reproduce states around Fermi energy or the band edges. We also specify the two additional parameters ${p}_{\min }$ and ${p}_{\max }$. From our tests, in most cases ${p}_{\max }$ = 0.95 and ${p}_{\min }$ = 0.01 already produce very good results. However, since chemical environments vary across different crystal structures, the two parameters are not universal and influence the quality of band interpolation. Figure 4 shows the variation of band distances w.r.t. ${p}_{\min }$ and ${p}_{\max }$ for several materials. For Al_3_V (Fig. 4a, b), *η*_0_ and *η*_2_ reach a minimum at two different sets of parameters, i.e., ${p}_{\max }$ = 0.99, ${p}_{\min }$ = 0.01 and ${p}_{\max }$ = 0.97, ${p}_{\min }$ = 0.01, respectively. In some cases, the variation of *η* w.r.t. ${p}_{\max }$ and ${p}_{\min }$ can be non-monotonic and display multiple local minima: For instance, in Au_2_Ti (Fig. 4c) at ${p}_{\min }$ = 0.01, *η*_2_ decreases from ${p}_{\max }$ = 0.90 to 0.95 but increases from ${p}_{\max }$ = 0.95 to 0.98 and finally reaches a local minimum at ${p}_{\max }$ = 0.99. In other cases, *η* can be quite stable and largely independent of the parameters: e.g., for Ba_6_Ge_10_ (Fig. 4d), *η*_2_ reaches the same minimum for ${p}_{\max }$ = 0.99 to 0.88.

Therefore, we implement an iterative optimization workflow to automatically find the optimal values for ${p}_{\max }$ and ${p}_{\min }$, in order to fully automate the Wannierization procedure. The workflow is released as part of the aiida-wannier90-workflows package^47^. First, we run a QE band structure workflow to get the reference DFT bands for calculating *η*_2_; in addition, the DFT bands are also used to calculate the band gap of the material. Second, we run an optimization workflow with the following settings: The maximum of the inner window is set to Fermi energy + 2 eV for metals and CBM + 2 eV for insulators, respectively; ${p}_{\max }$ and ${p}_{\min }$ are set to the defaults of 0.95 and 0.01, respectively. Third, if the average band distance *η*_2_ is less than a threshold (set to 10 meV here), the workflow stops; otherwise, the workflow iterates on a mesh of ${p}_{\max }$ and ${p}_{\min }$, i.e. ${p}_{\max }$ decreasing from 0.99 to 0.80 with step size −0.01, and ${p}_{\min }$ = 0.01 or 0.02, until *η*_2_≤ threshold. If *η*_2_ is still larger than the threshold after exhausting all the parameter combinations, the workflow will output the minimum-*η*_2_ calculation.

### Band distance

**Fig. 5:**
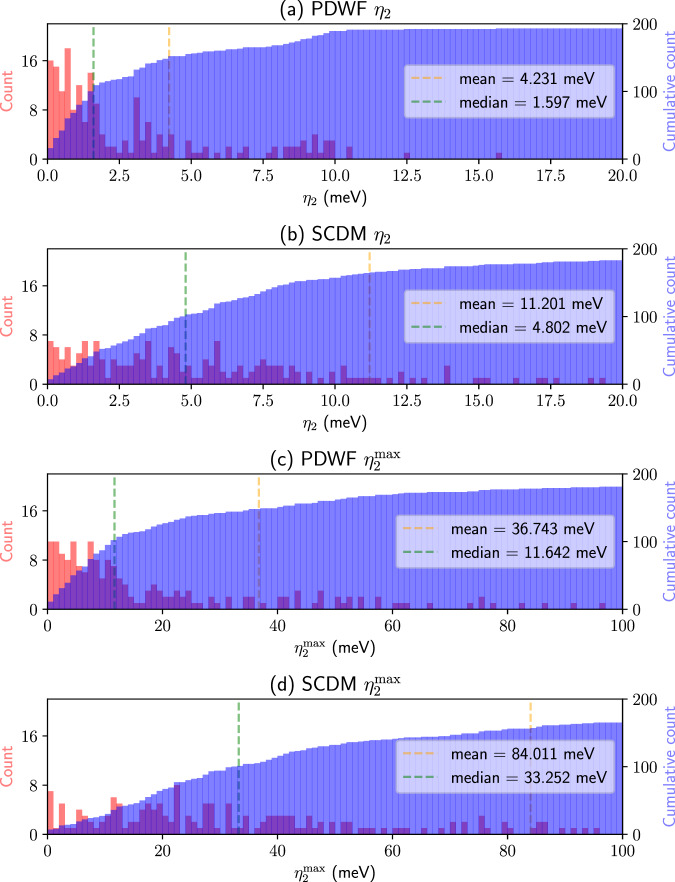
Histogram (red) and cumulative histogram (blue) of the band distances *η*_2_ and ${\eta }_{2}^{\max }$ for 200 reference structures. **a** *η*_2_ of PDWF, **b** *η*_2_ of SCDM, **c** ${\eta }_{2}^{\max }$ of PDWF, and **d** ${\eta }_{2}^{\max }$ of SCDM. The orange (green) vertical line is the mean (median) of the band distance for the 200 structures; their values are shown in the right of each panel; PDWF provides approximately an improvement by a factor of 3.

To compare quantitatively the band interpolation quality of SCDM and PDWF, we Wannierize the 200 structures mentioned earlier and calculate their band distances with respect to the corresponding DFT bands. We choose *η*_2_ and ${\eta }_{2}^{\max }$ to compare near-Fermi-energy bands. The histograms of the band distances for the 200 structures are shown in Fig. 5. To directly compare SCDM and PDWF, the mean and median value of *η* of the 200 calculations are shown as vertical lines in each panel. For PDWF, the mean *η*_2_ is 4.231 meV, to be compared with 11.201 meV for SCDM. For ${\eta }_{2}^{\max }$ (that is a more stringent test of the quality of interpolation) the PDWF method also performs better, with a ${\eta }_{2}^{\max }=$ 36.743 meV vs. 84.011 meV for SCDM. We can also observe this trend in Fig. 5: For *η*_2_ and ${\eta }_{2}^{\max }$, the PDWF histogram bins are much more clustered towards *η* = 0. Note that in the cumulative histograms of *η*_2_, at *η* = 20 meV, the PDWF cumulative count is closer to the total number of calculations (200). This indicates that the PDWF has a higher success rate in reducing the interpolation error below 20 meV. Similarly, for ${\eta }_{2}^{\max }$, PDWF has a higher success rate in reducing the interpolation error under 100 meV (to get a better overview of *η* and ${\eta }^{\max }$, we further show the same histograms of *η* in a wider range 0 meV to 100 meV, and ${\eta }^{\max }$ in range 0 meV to 500 meV, in Supplementary Figs. 11 and 12). To reduce the effect of major outliers, we can also compare the interpolation accuracy of successful calculations, i.e., excluding the outlier calculations which have significantly large band distances. As shown in Supplementary Table 1, the ${\eta }_{2}^{\le 20}$, i.e., the average of all the calculations for which *η*_2_≤ 20 meV, indicates that PDWF (2.922 meV) is twice as good as SCDM (5.280 meV), and also has a higher success rate: for ${\eta }_{2}^{\le 20}$, 193/200 = 96.5% of the structures have *η*_2_≤ 20 meV, while for SCDM it is 183/200 = 91.5%. More details are listed in Supplementary Table 1.

In summary, PDWF provides more accurate and robust interpolations, especially for bands around the Fermi energy or the band gap edges, which are the most relevant bands for many applications. Last but not least, a higher energy range can be accurately interpolated by increasing the number of PAOs (see Section “Additional PAOs for high-energy/accuracy interpolation”).

### MLWF centers

**Fig. 6:**
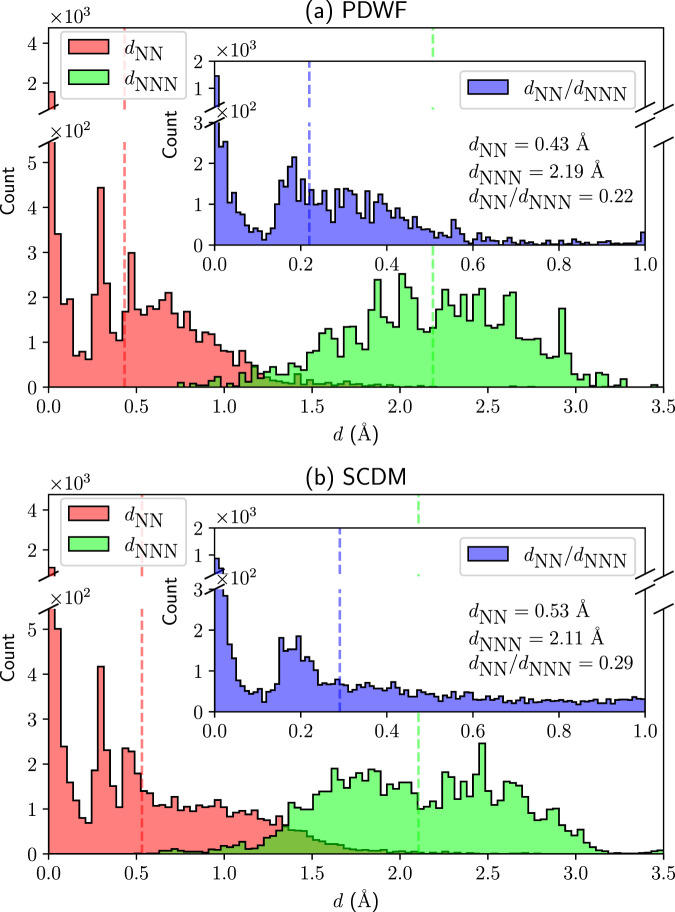
Histogram of the distances of the WF centers from the NN atom (red, *d*_NN_) and NNN atom (green, *d*_NNN_), for 200 reference structures. **a** PDWF and **b** SCDM. The inset of each panel shows the histogram of the ratio of *d*_NN_/*d*_NNN_. The numbers in the lower right of each inset are the averages over all the 6818 MLWFs; PDWF provides MLWFs that are both closer to the NN atom and further away from the NNN atom.

Since we are aiming at restoring a tight-binding atomic-orbital picture with PDWF, we compare the distance of the WF centers from the nearest-neighboring (NN) and next-nearest-neighboring (NNN) atoms, again both for SCDM and PDWF. For each method, we compute *d*_NN_ and *d*_NNN_, i.e., the average distance of all the 6818 MLWFs from the respective NN and NNN atoms. If *d*_NN_ is 0, then the atomic-orbital picture is strictly preserved. However, this is unlikely to happen since there is no constraint on the WF centers during both the disentanglement and the localization, and the final PDWFs, resembling atomic orbitals, are optimized according to the chemical environment. Still, if a WF center is much closer to the NN atom than to the NNN atom, then one can still assign it to the NN atom, preserving the atomic-orbital picture. Figure 6 shows the histograms for *d*_NN_ and *d*_NNN_ for the two methods. The PDWF average *d*_NN_ = 0.43 Å is smaller than the SCDM *d*_NN_ = 0.53 Å, and correspondingly the PDWF *d*_NNN_ = 2.19 Å is instead larger than the SCDM *d*_NNN_ = 2.11 Å. This can also be observed in Fig. 6: The overlap of the *d*_NN_ and *d*_NNN_ histograms is smaller for PDWF than for SCDM. To further understand the overlaps, we plot the histogram of the ratio *d*_NN_/*d*_NNN_ of each MLWF in the insets of Fig. 6. For a MLWF, if *d*_NN_/*d*_NNN_ = 1, then the MLWF is a bonding orbital centered between two atoms; while if *d*_NN_/*d*_NNN_ ≪ 1, then it can be regarded as an (almost) atomic orbital. The histogram of the ratio of SCDM has a long tail extending towards 1.0, i.e., there are a large number of SCDM MLWFs sitting close to bond centers; on the contrary, the vast majority of the PDWF MLWFs are closer to the NN atom.

We can further compare the effect of maximal localization on the WF centers. The WFs from the projection matrices *A*_*mn***k**_ are strictly atom-centered, i.e. *d*_NN_ = 0. The inset of Supplementary Fig 13a shows the histogram of the initial WFs, i.e., after disentanglement and before maximal localization, and the final MLWFs, i.e., after maximal localization, for PDWF. If one chooses *d*_NN_≤ 0.1 Å as the criterion for atom-centered MLWFs, then 5594/6818 = 82.0% of the initial WFs and 2045/6818 = 30.0% of the final MLWFs are atom-centered. The disentanglement and maximal localization improve the band interpolation, but since there is no constraint on the WF center in the spread functional Eq. (2), many of the final MLWF centers are not atom-centered. As a comparison, for SCDM, 955/6818 = 14.0% of the initial WFs and 1823/6818 = 26.7% of the final MLWFs are atom-centered. For completeness, the statistics and histograms of initial and final *d*_NN_, *d*_NNN_, and *d*_NN_/*d*_NNN_ are shown in Supplementary Table 2 and Supplementary Fig. 13.

In summary, for PDWF, most of the initial WFs (after disentanglement and before maximal localization) are atom-centered; many drift a bit away from atom centers during the localization, but the MLWFs are still much closer to the NN than to NNN atoms. For SCDM, most of the initial WFs are away from atom centers, and maximal localization pushes some of the WFs back to atoms, but there is still a large number of MLWFs for which an atom representing the WF center cannot be clearly identified. To exactly fix the MLWFs to atomic positions, one needs to add constraints to the spread functional^16^, at the cost of potentially having worse interpolators. However, this is beyond the scope of the current work, and here we rely on the atom-centered PAO projectors to guide the MLWFs towards the atomic positions, so that the final MLWFs are optimally localized and atom-centered.

### MLWF spreads

**Fig. 7:**
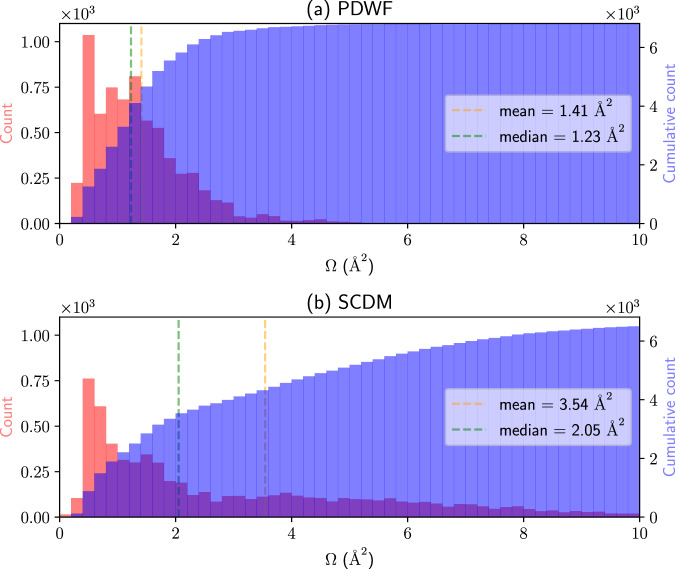
Histogram (red) and cumulative histogram (blue) of WF spreads for 200 reference structures. **a** PDWF and **b** SCDM. The orange (green) vertical line is the mean (median) spread of the 6818 MLWFs, their values are shown in the right of each panel. The long tail of MLWF spreads obtained with SCDM is absent in PDWF.

Next, we investigate the spread distributions of SCDM and PDWF. Usually, we want localized MLWFs to restore the TB atomic orbitals. Figure 7 shows the histograms of the spread distributions for the two methods. The SCDM spreads have a long tail extending over 10 Å^2^ in Fig. 7b, due to its inclusion of free-electron states in the density matrix, thus resulting in more delocalized MLWFs as discussed earlier (see e.g. Fig. 2). On the contrary, the PDWF selects and freezes atomic-orbital states from the remaining bands, leading to much more localized MLWFs, thus much more clustered in a narrow range of 0 Å^2^ to 4 Å^2^, and already at 5 Å^2^ the cumulative histogram almost reaches the total number of MLWFs (see Fig. 7a). This can be interpreted as follows: The PAO initial projections guide the spread minimization toward the (local) minimum resembling spherical harmonics, whereas the SCDM-decomposed basis vectors are designed to be mathematical objects spanning as much as possible the density matrix, but result in WFs for which it is harder to assign definite orbital characters.

We can further compare the average initial (after disentanglement but before maximal localization) and final (after disentanglement and maximal localization) spreads between the two methods, as shown in Supplementary Table 3 and corresponding histograms in Supplementary Fig. 14. Maximal localization is needed to bring SCDM spreads, from the initial Ω^*i*^ = 30.82 Å^2^ to the final Ω^*f*^ = 3.54 Å^2^ ; For PDWF, the initial Ω^*i*^ = 2.72 Å^2^ is already quite localized, and much better than the final Ω^*f*^ for SCDM; localization then brings it to an optimal Ω^*f*^ = 1.41 Å^2^.

### Hamiltonian decay

**Fig. 8:**
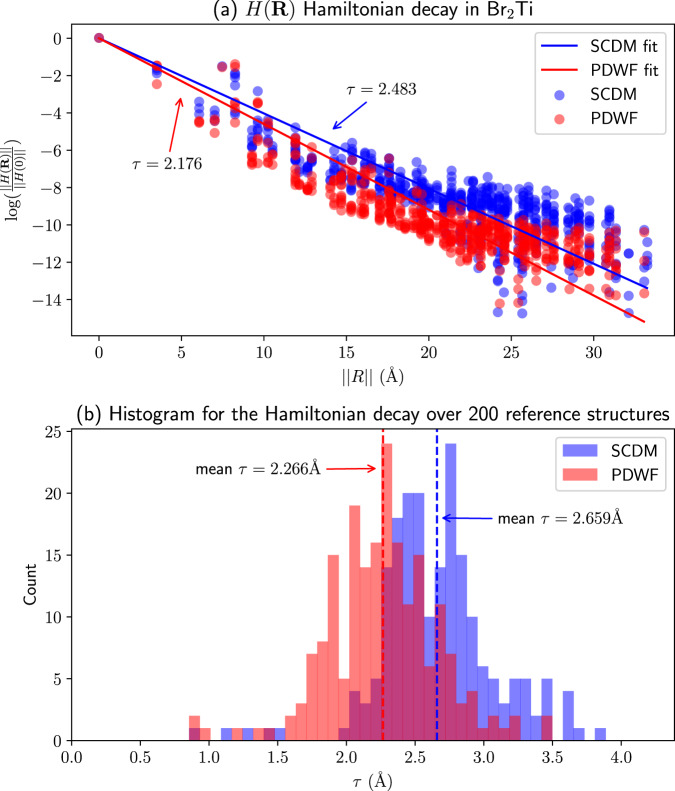
Exponential decay of the Hamiltonian *H*(**R**) in the basis of MLWFs. **a** Exponential-form fitting of Frobenius norm of the Hamiltonian $\left\Vert H({{{\bf{R}}}})\right\Vert$ w.r.t. to the 2-norm of lattice vector $\left\Vert {{{\bf{R}}}}\right\Vert$ for the case of Br_2_Ti, for PDWF (red) and SCDM (blue). The *τ* reported are the fitted decay lengths of the PDWF and SCDM Hamiltonians, respectively. **b** Histogram of decay lengths *τ* for the 200 reference materials, obtained using PDWF (red) and SCDM (blue). The vertical lines indicate the mean *τ* of PDWF and SCDM, respectively.

Finally, we compare the decay length of the Wannier gauge Hamiltonian between the two methods in Fig. 8. Thanks to the localization of MLWFs, the expectation values of quantum mechanical operators in the MLWF basis, such as the Hamiltonian *H*(**R**), decay rapidly with respect to the lattice vector **R** (exponentially in insulators^48,49^ and properly disentangled metals). To compare this decay for the Hamiltonian matrix elements, we approximate the Frobenius norm of the Hamiltonian as

8 $$\left\Vert H({{{\bf{R}}}})\right\Vert =\left\Vert H({{{\bf{0}}}})\right\Vert \exp \left(-\frac{\left\Vert {{{\bf{R}}}}\right\Vert }{\tau }\right),$$

where *τ* measures the decay length. Then *τ* is fitted by least squares to the calculated $\left\Vert H({{{\bf{R}}}})\right\Vert$; as shown in Fig. 8a, the Hamiltonian of PDWF decays faster than SCDM for Br_2_Ti, which is selected here to represent the general trend between PDWF and SCDM Hamiltonians. Figure 8b shows the histogram of *τ* for the 200 materials; the mean *τ* are 2.266 Å for PDWF and 2.659 Å for SCDM, respectively, indicating that the PDWF Hamiltonian decays faster than SCDM, consistent with the better band interpolation of PDWF discussed in Fig. 5.

### High-throughput Wannierization

**Fig. 9:**
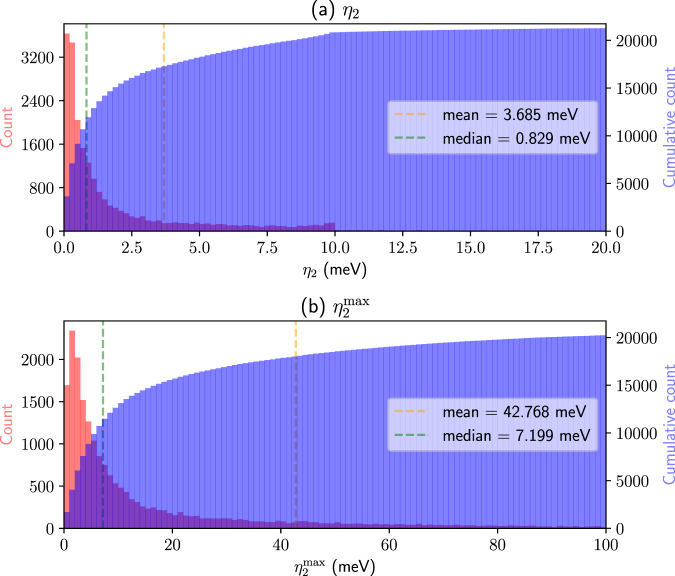
Histogram (red) and cumulative histogram (blue) of the PDWF band distances for 21,737 non-magnetic structures obtained from the Materials Cloud MC3D database^36^. **a** Average band distance *η*_2_ and **b** max band distance ${\eta }_{2}^{\max }$. The orange (green) vertical line is the mean (median) of the band distance for the 21,737 structures; their values are shown in the right of each panel.

Based on the above verification, we run a HT Wannierization using PDWF for 21,737 materials, selected from the non-magnetic materials of the MC3D database^36^. Figure 9 shows the band distance histograms for *η*_2_ and ${\eta }_{2}^{\max }$. Overall, the statistics follow the same trend as the 200 materials set in Fig. 5: the average *η*_2_ and average ${\eta }_{2}^{\max }$ are 3.685 meV and 42.768 meV, respectively. Note in Fig. 9a the *η*_2_ is not truncated at 10 meV, but rather due to the automated optimization workflow: results that have *η*_2_ larger than a threshold (10 meV) are further optimized with respect to ${p}_{\min }$ and ${p}_{\max }$, thus improving the average band distance *η*_2_. In Supplementary Table 4 we show several other statistics for the band distances. The accurate interpolation quality of PDWF can be assessed, for instance, from the number of systems with *η*_2_≤ 20 meV, that are ≈ 97.8% of all the calculations (21259/21737); the corresponding band distance calculated on these 21259 calculations is ${\eta }_{2}^{\le 20}$ = 2.118 meV. This remarkable result show how automated and reliable Wannierizations can now be deployed automatically both for individual calculation and for HT application.

### Additional PAOs for high-energy/accuracy interpolation

Based on the HT Wannierization results, one can identify cases where the interpolation quality can be further improved by increasing the number of PAOs. Typically, the number of PAOs is determined during pseudopotential generation, and they are usually the orbitals describing low-energy valence electrons. In some cases, the bonding/anti-bonding combinations of these PAOs are not sufficient to span the space of target conduction bands, leading to a loss of interpolation quality. We use silicon as an example to illustrate the difficulties of accurately describing its CBM^50^, which is not located at any high-symmetry *k*-point, but along the Γ − X line. The common choice of one *s* and three *p* hydrogenic or PAOs projectors per atom results in oscillations in the Wannier-interpolated bands at the meV level. To remedy this, one can use a larger set of PAOs, e.g., by regenerating a silicon pseudopotential including *d* PAOs as discussed in Section “Comparisons of four prototypical materials” for silicon. However, generating a new pseudopotential requires extensive testing and validation, therefore another solution could be using a set of PAOs different from the pseudopotential ones. To compare this second approach, we test here also PAOs obtained from the OpenMX code^44^, and Wannierize silicon using one *s*, three *p*, and five *d* PAOs per atom using ED. This provides a much better description of the CBM, as shown in Supplementary Fig. 17 Moreover, the additional *d* orbitals allow to raise the inner energy window and better reproduce a larger number of conduction bands, as shown in Supplementary Fig. 18, which might be beneficial for some applications. For completeness, we also show the WF spreads and shapes of *d* orbitals in Supplementary Fig. 19. However, there are some caveats to this approach. When using external PAOs, ideally one should generate them using the same pseudization scheme as the pseudopotentials used in the DFT calculations. The PAOs from OpenMX are instead generated using a different scheme, resulting in lower projectabilities (smaller than one even for the valence bands, as shown in Supplementary Fig. 21). In such case, PD cannot reproduce the original bands (see Supplementary Fig. 20b), thus ED (with a higher inner energy window) is needed to obtain accurate interpolation (see Supplementary Fig. 18d). In comparison, the pseudopotential PAOs which we regenerated with 3*d* orbitals (as discussed in Section “Comparisons of four prototypical materials” for silicon) are better projectors for the wavefunctions. Indeed, the first 12 bands have projectabilities almost equal to 1, and as a consequence PD itself already provides accurate band interpolation (all the low-energy conduction states are frozen since their projectabilities are high, see Supplementary Fig. 20a). Moreover, we mention that when adding additional projectors one needs to make sure that they have the correct number of radial nodes: e.g., the gold pseudopotential from SSSP^46^ contains 5*s* + 5*p* semicore states, and 6*s* + 5*d* orbitals for valence electrons. If one wants to add an additional 6*p* orbital, it is important to ensure that the 6*p* orbital has one radial node, such that it is orthogonal to the nodeless 5*p* semicore state; Otherwise, the Bloch wavefunctions would project onto the 5*p* semicore state, and PD would only disentangle the 5*p* semicore states instead of the 6*p* orbitals contributing to bands above the Fermi energy. In summary, including more projectors can further improve the interpolation quality, but at the expense of increasing the number of orbitals in the model. The combination of PD and ED enables to improve the interpolation quality of low-projectability states while keeping the TB model size small. Automatic checks could be implemented in the future in the AiiDA workflows to detect whether the projectability drops below a certain threshold, and in that case either raise a warning or automatically add more projectors.

## Discussions

We present an automated method for the automated, robust, and reliable construction of tight-binding models based on MLWFs. The approach applies equally well to metals, insulators and semiconductors, providing in all cases atomic-like orbitals that span both the occupied states, and the empty ones whose character remains orbital-like and and not free-electron-like. The method is based on the band projectability onto pseudo-atomic orbitals to select which states are kept identically, dropped, or passed on to the established disentanglement procedure. We augment such projectability-based selection with an additional energy window to guarantee that all states around the Fermi level or the conduction band edge are well reproduced, showing that such a combination enables accurate interpolation even when minimal sets of initial atomic orbitals are chosen. This results in compact Wannier tight-binding models that provide accurate band interpolations while preserving the picture of atomic orbitals in crystals. We refer to the method collectively as projectability-disentangled Wannier functions (PDWF).

The Wannierization process is implemented as fully automated AiiDA workflows. We compare PDWFs with the other method that is also fully automated, namely SCDM. We show with a detailed study of 200 structures that PDWFs lead to more accurate band interpolations (with errors with respect to the original bands at the meV scale), and are more atom-centered and more localized than those originating from SCDM. The high accuracy in band interpolations, the target atomic orbitals obtained, and the low computational cost make PDWFs an ideal choice for automated or high-throughput Wannierization, which we demonstrate by performing the Wannierization of 21,737 non-magnetic structures from the Materials Cloud MC3D database.

## Methods

### Code implementation

We implement the PAO projection in the pw2wannier90.x executable inside Quantum ESPRESSO (QE)^42,51^; the PD and PD+ED methods are implemented on top of the Wannier90 code^4^. In terms of the practical implementation, computing PAO projections is more efficient in both computational time and memory than the SCDM QR decomposition with column pivoting (QRCP) algorithm, since the *A*_*mn***k**_ matrices (i.e., the inner products of Bloch wavefunctions with PAOs) can be evaluated in the plane-wave *G* vector space, rather than requiring a Fourier transform and decomposition of very large real-space wavefunction matrices. Furthermore, since the HT Wannierization can be computationally intensive, we implement a “*k*-pool parallelization strategy” inside pw2wannier90.x, similarly to the main pw.x code of QE, to efficiently utilize many-core architectures by parallelizing over “pools” of processors for the almost trivially-parallel computations at each *k*-point. Test results show that *k* − pool parallelization significantly improves the efficiency of pw2wannier90.x (benchmarks are shown in Supplementary Fig. 10).

### DFT calculations

The DFT calculations are carried out using QE, with the SSSP efficiency (version 1.1, PBE functional) library^46^ for pseudopotentials and its recommended energy cutoffs. The HT calculations are managed with the AiiDA infrastructure^33–35^ which submits QE and Wannier90 calculations to remote clusters, parses, and stores the results into a database, while also orchestrating all sequences of simulations and workflows. The automated AiiDA workflows are open-source and hosted on GitHub^47^. The workflows accept a crystal structure as input and provide the Wannier-interpolated band structure, the real-space MLWFs, and a number of additional quantities as output. Semicore states from pseudopotentials are automatically detected and excluded from the Wannierizations, except for a few cases where some semicore states overlap with valence states; in such cases, all the semicore states are Wannierized, otherwise the band interpolation quality would be degraded, especially for SCDM. A regular *k*-point mesh is used for the Wannier calculations, with a *k*-point spacing of 0.2 Å^−1^, as selected by the protocol in ref. ^30^.

### Visualizations

MLWFs are rendered with VESTA^52^. Figures are generated by matplotlib^53^.

### Supplementary information

Supplementary Information

## Data Availability

All data generated for this work can be obtained from the Materials Cloud Archive (10.24435/materialscloud:v4-e9).

## Code availability

All codes used for this work are open-source; the latest stable versions can be downloaded at http://www.wannier.org/, https://www.quantum-espresso.org/, https://www.aiida.net/, and https://github.com/aiidateam/aiida-wannier90-workflows. The modifications to the codes mentioned above implemented for this work will become available in the next releases of Quantum ESPRESSO (pw2wannier90.x) and Wannier90.
