# Supplementary material for: Projectability disentanglement for accurate and automated electronic-structure Hamiltonians
Source: NPJ Comput Mater. 2023 Nov 3;9(1):208. doi: 10.1038/s41524-023-01146-w (PMC11041802; doi:10.1038/s41524-023-01146-w)

Supplementary Information:

Projectability disentanglement for accurate and automated electronic-structure Hamiltonians

Junfeng Qiao[1], Giovanni Pizzi[1,2] and Nicola Marzari[1,2]

[1]Theory and Simulations of Materials (THEOS), and National Centre for Computational Design and Discovery of Novel Materials (MARVEL), École Polytechnique Fédérale de Lausanne, 1015 Lausanne, Switzerland

[2]Laboratory for Materials Simulations (LMS), Paul Scherrer Institut (PSI), CH-5232 Villigen PSI, Switzerland

September 12, 2023

Contents

1 Supplementary Discussion **3**
1.1 Comparison . . . 3
1.1.1 Graphene . . . 3
1.1.2 Silicon . . . 4
1.1.3 Copper . . . 6
1.1.4 Strontium vanadate . . . 10
1.2 `pw2wannier90.x` parallelization . . . 14
1.3 Band distance . . . 15
1.4 MLWF centers . . . 19
1.5 MLWF spreads . . . 21
1.6 High-throughput Wannierization . . . 23
1.7 Additional PAOs . . . 26

Supplementary References **31**

2 Supplementary Figures **32**
2.1 Band structure and Hamiltonian decay . . . 32

1 Supplementary Discussion

1.1 Comparison

1.1.1 Graphene

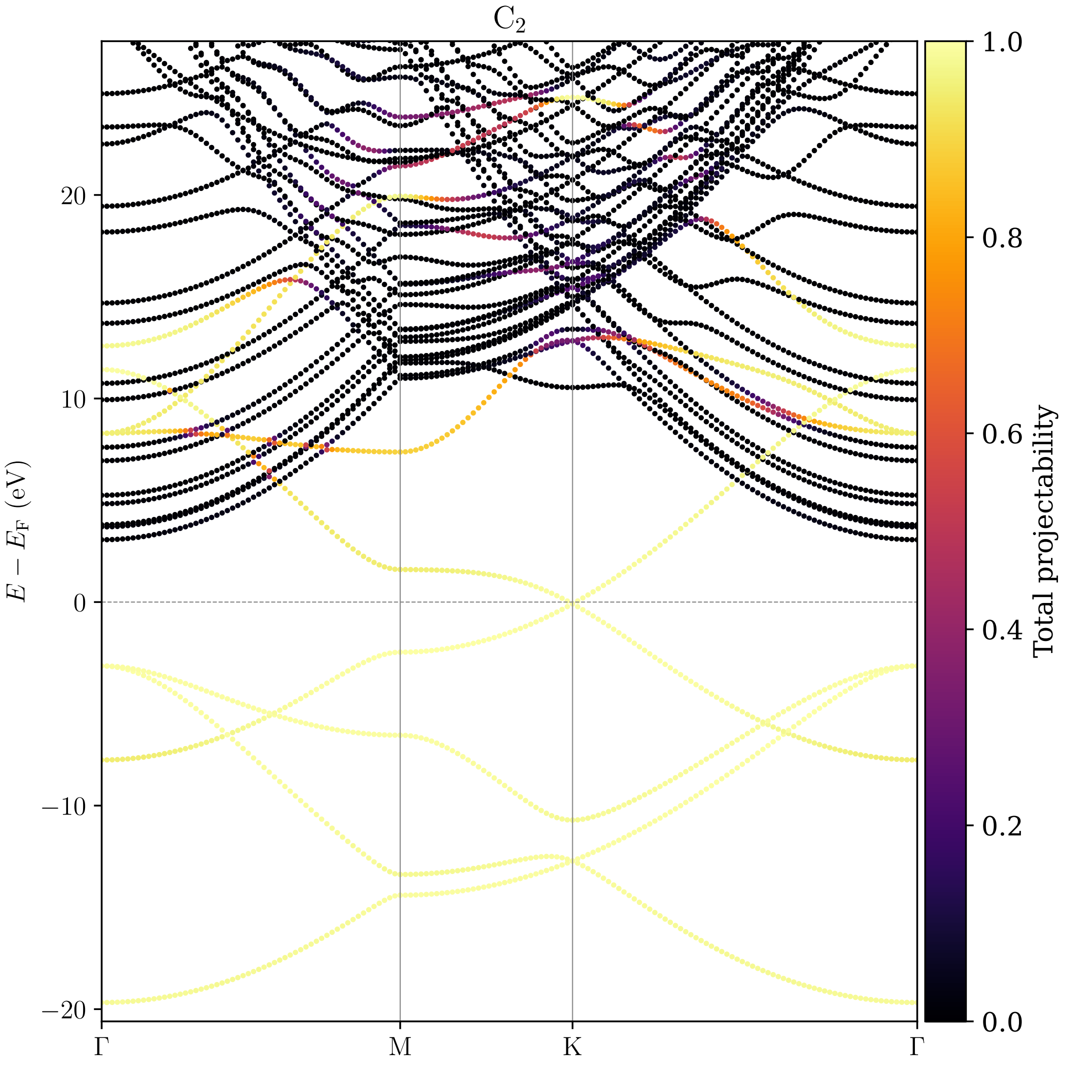

Supplementary Figure 1: Graphene band structure projectability.

1.1.2 Silicon

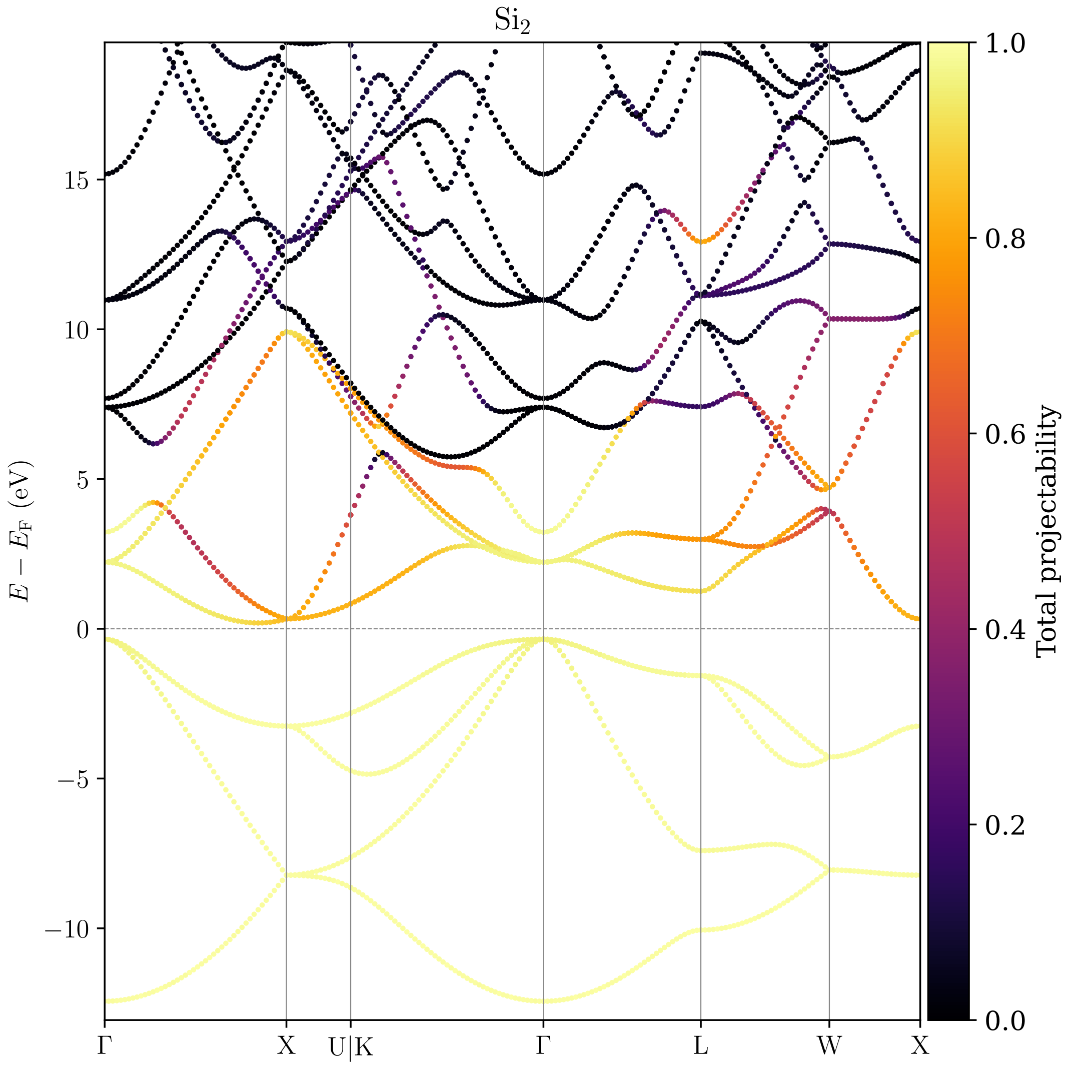

Supplementary Figure 2: Silicon band structure projectability.

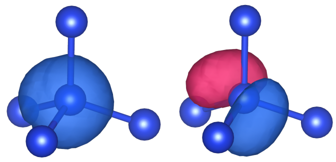

	multiplicity	2	6		
**hydrogenic ED**	$d_{WFC}(\AA)$	0.000	0.000		
	$\Omega_{WF}(\AA^2)$	1.849	2.546		
**PAO ED**	$d_{WFC}(\AA)$	0.000	0.000		
	$\Omega_{WF}(\AA^2)$	1.849	2.546		
**PAO PD**	$d_{WFC}(\AA)$	0.000	0.000		
	$\Omega_{WF}(\AA^2)$	1.320	1.885		
**PAO PD+ED**	$d_{WFC}(\AA)$	0.000	0.000		
	$\Omega_{WF}(\AA^2)$	1.798	2.597		

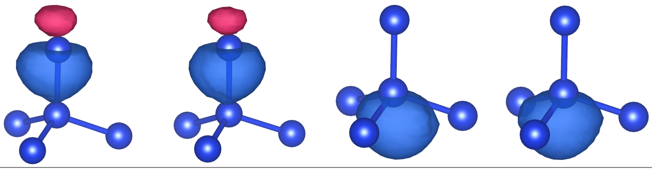

	multiplicity	1	3	3	1
**SCDM**	$d_{WFC}(\AA)$	0.566	0.638	0.833	0.836
	$\Omega_{WF}(\AA^2)$	5.201	4.921	4.507	4.586

Supplementary Figure 3: Silicon MLWF shapes, centers, and spreads for hydrogenic with ED, SCDM, PAO with ED, PAO with PD, and PAO with PD+ED. d_{WFC} is the distance of WF centers from the nearest-neighbor atom, and Ω_{WF} is the spread of each WF.

1.1.3 Copper

Here we test the five methods with a metal, copper. For PD and PD+ED, we choose $p_{\text{max}} = 0.95$, $p_{\text{min}} = 0.01$. As shown in Fig. 4, all the methods can accurately interpolate occupied bands. At first glance, the SCDM reproduces better the overall band structure. However, in the higher energy range for the bands at the X point at about 17 eV, the SCDM has an unphysical large discontinuity of bands along different directions, leading to its much larger spreads and irregular shapes in Fig. 6. For all other four methods, although the high-energy conduction bands do not overlap with DFT bands (as expected), they are still smoothly connected throughout the BZ. Thanks to the freezing of some high projectability states in the high-energy region, the PD and the PD+ED can reproduce better the high-energy unoccupied bands.

Apart from SCDM, MLWFs of all the other four methods are strictly atom-centered orbitals, and perfectly preserve the multiplicities of s, p, and d orbitals. Note the PD and PD+ED reach the same set of MLWFs, since the PAOs are complete for all the bands inside the inner energy window. The high projectability ensures the freezing of all the bands in the inner energy window, thus in this case PD is enough.

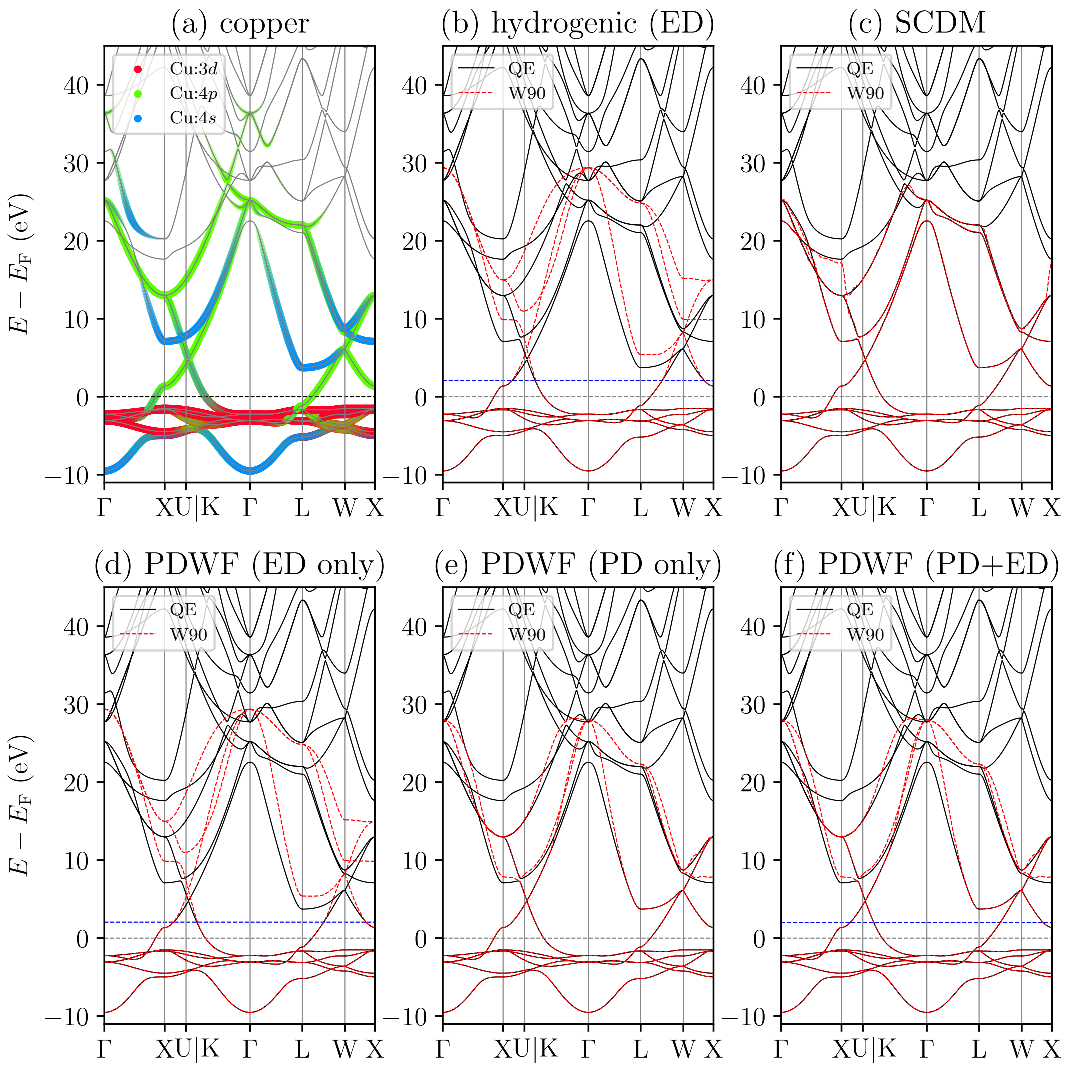

Supplementary Figure 4: (a) Band structures of copper, shown as grey lines. The colored dots represent the projectability of copper $3d$ (red), $4p$ (green), and $4s$ (blue) orbitals. The size of the dot is proportional to the total projectability $p_{m\mathbf{k}}$ of the band m at kpoint $\mathbf{k}$. For a detailed plot of total projectability, see Fig. 5. Comparisons of QE and Wannier-interpolated bands for (b) hydrogenic with ED, (c) SCDM, (d) PAO with ED, (e) PAO with PD, and (f) PAO with PD+ED. The Fermi energy E_F (horizontal black dashed line) is at zero. The horizontal blue dashed line denotes the top of inner energy window, which equals $E_F + 2\,\text{eV}$.

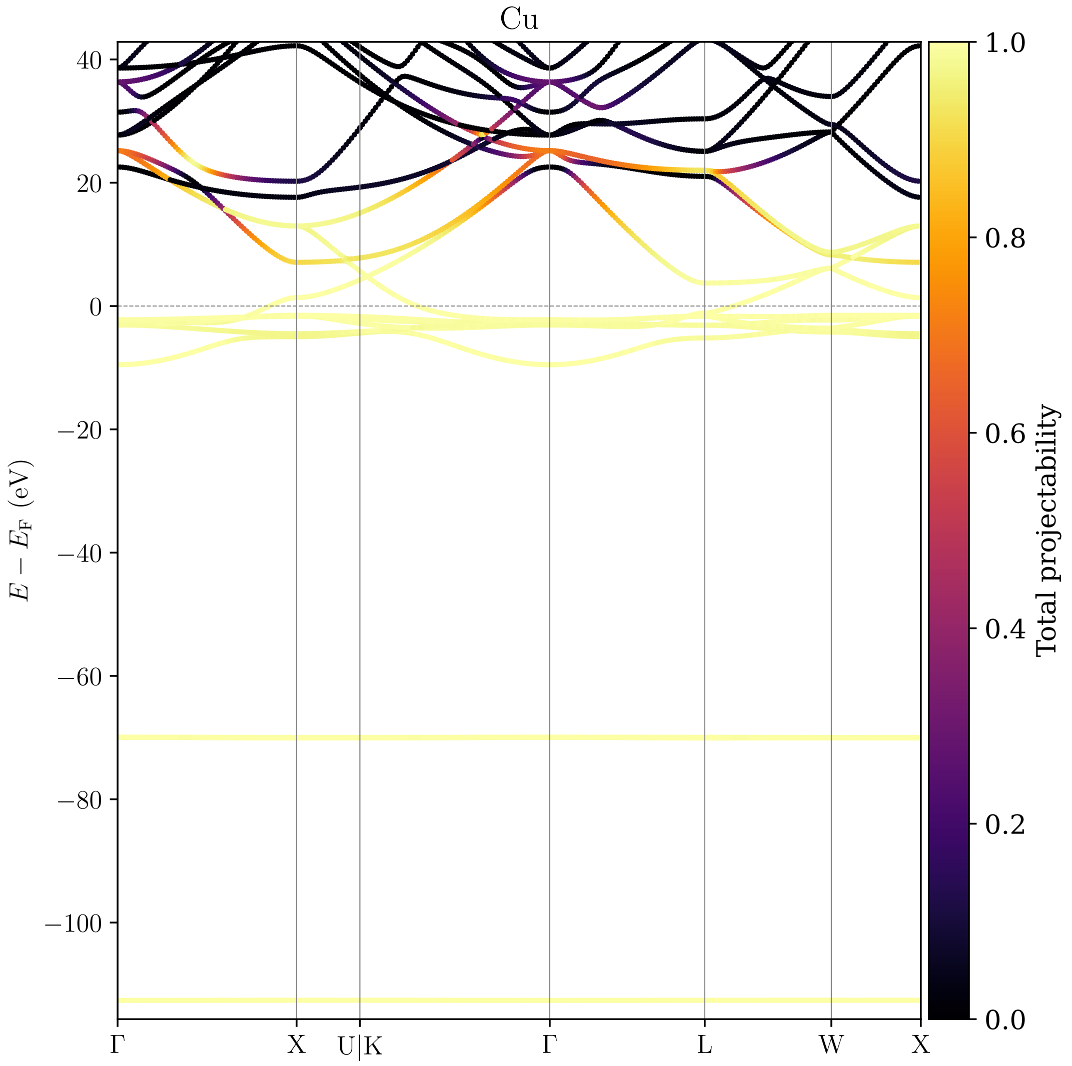

Supplementary Figure 5: Copper band structure projectability.

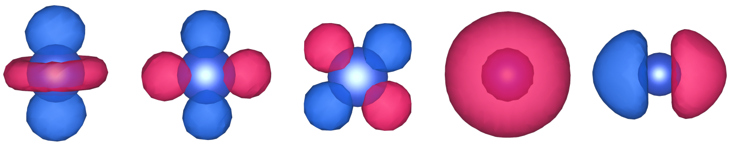

		d_{z^2}	$d_{x^2-y^2}$	d_{xy}, d_{xz}, d_{yz}	s	p_x, p_y, p_z
	character	d_{z^2}	$d_{x^2-y^2}$	d_{xy}, d_{xz}, d_{yz}	s	p_x, p_y, p_z
	multiplicity	1	1	3	1	3
**hydrogenic ED**	$d_{WFC}(Å)$	0.000	0.000	0.000	0.000	0.000
	$\Omega_{WF}(Å^2)$	0.370	0.370	0.364	1.227	1.271
**PAO ED**	$d_{WFC}(Å)$	0.000	0.000	0.000	0.000	0.000
	$\Omega_{WF}(Å^2)$	0.370	0.370	0.364	1.227	1.271
**PAO PD**	$d_{WFC}(Å)$	0.000	0.000	0.000	0.000	0.000
	$\Omega_{WF}(Å^2)$	0.340	0.340	0.336	1.531	2.000
**PAO PD+ED**	$d_{WFC}(Å)$	0.000	0.000	0.000	0.000	0.000
	$\Omega_{WF}(Å^2)$	0.340	0.340	0.336	1.531	2.000

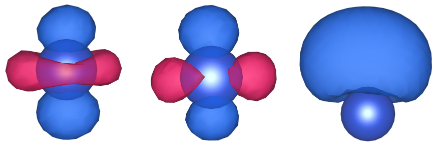

**SCDM**	$d_{WFC}(Å)$	0.005	0.030	0.760
	$\Omega_{WF}(Å^2)$	0.339	0.421	5.123
	$d_{WFC}(Å)$	0.021		0.997
	$\Omega_{WF}(Å^2)$	0.402		4.790
	$d_{WFC}(Å)$	0.023		1.063
	$\Omega_{WF}(Å^2)$	0.409		5.005
	$d_{WFC}(Å)$	0.029		1.296
	$\Omega_{WF}(Å^2)$	0.453		4.932

Supplementary Figure 6: Copper MLWF shapes, centers, and spreads for hydrogenic with ED, SCDM, PAO with ED, PAO with PD, and PAO with PD+ED. For SCDM, all the 9 MLWFs are listed since their d_{WFC}, Ω_{WF}, and shapes are different. The 9 MLWFs are roughly categorized into 3 groups.

1.1.4 Strontium vanadate

Here we test a perovskite, $SrVO_3$. For PAO with PD and PAO with PD+ED, we choose $p_{\max} = 0.95$, $p_{\min} = 0.01$. Figure 7 shows the band structures. As shown in Fig. 7a, a large portion of conduction bands have high projectability; in such cases, it is relatively safe to increase the maximum inner energy window. However, in HT calculations, it is difficult to tune the inner energy window in an automated fashion. With the help of PD or PD+ED, the high-projectability states are automatically frozen, leading to overall much better interpolated bands when comparing Figs. 7e and 7f with Figs. 7b and 7d.

Figure 9 shows the MLWF shapes of the five methods. The MLWFs of both the hydrogenic with ED and PAO with ED are orbitals with the characteristic s, p, and d shapes and are strictly atom-centered. For PAO with PD and PAO with PD+ED, although few MLWFs hybridize and slightly drift away from the atom centers, most of the MLWFs still resemble atomic orbitals. The SCDM MLWFs again have much larger spreads and irregular shapes.

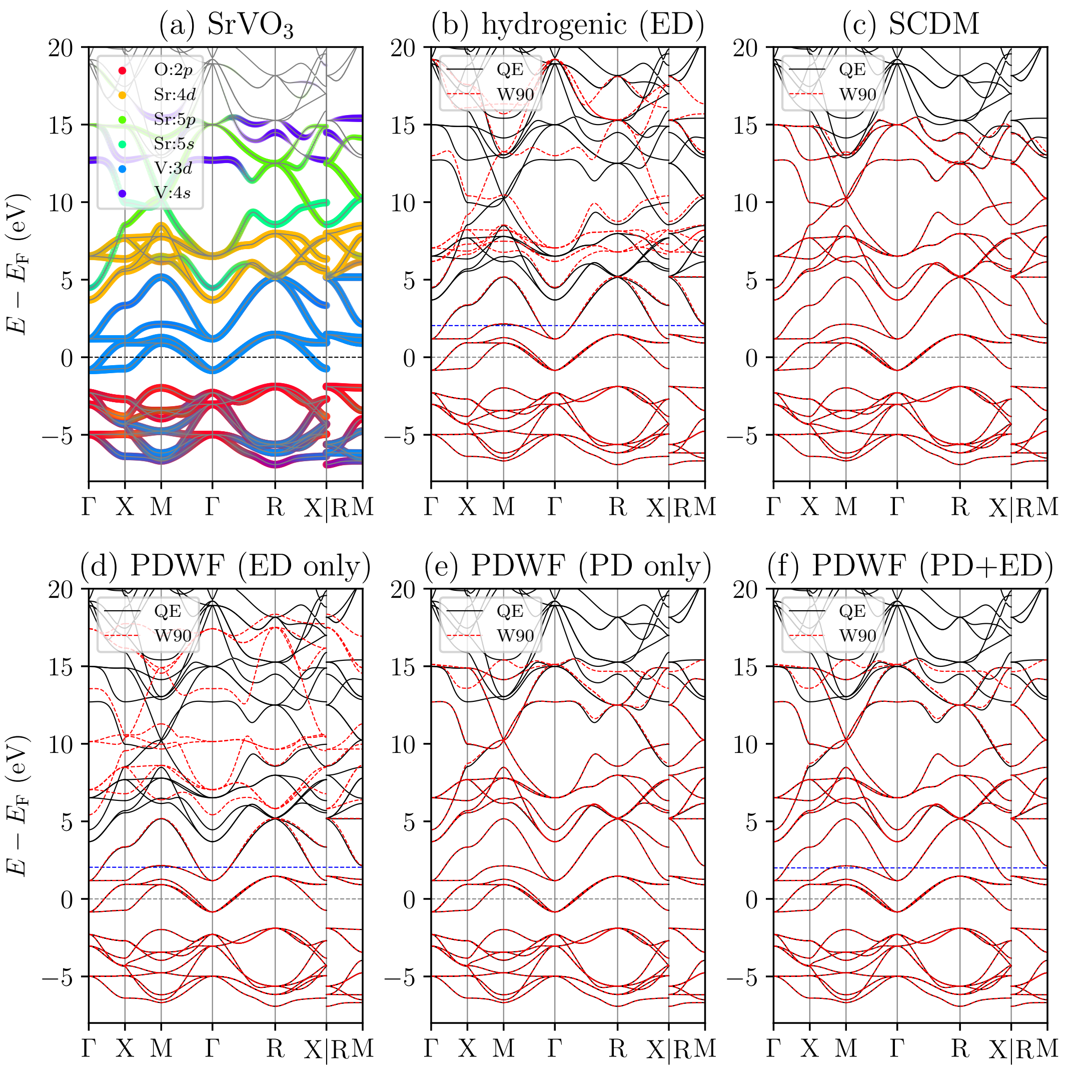

Supplementary Figure 7: (a) Band structures of $SrVO_3$, shown as grey lines. The colored dots represent the projectability of oxygen $2p$ (red), strontium $4d$ (orange), strontium $5p$ (green), strontium $5s$ (cyan), vanadium $3d$ (blue), and vanadium $4s$ (purple) orbitals, respectively. The size of the dot is proportional to the total projectability $p_{m\mathbf{k}}$ of the band m at kpoint $\mathbf{k}$. For a detailed plot of total projectability, see Fig. 8. Comparisons of QE and Wannier-interpolated bands for (b) hydrogenic with ED, (c) SCDM, (d) PAO with ED, (e) PAO with PD, and (f) PAO with PD+ED. The Fermi energy E_F (horizontal black dashed line) is at zero. The horizontal blue dashed line denotes the maximum inner energy window, which equals $E_F + 2\,\text{eV}$.

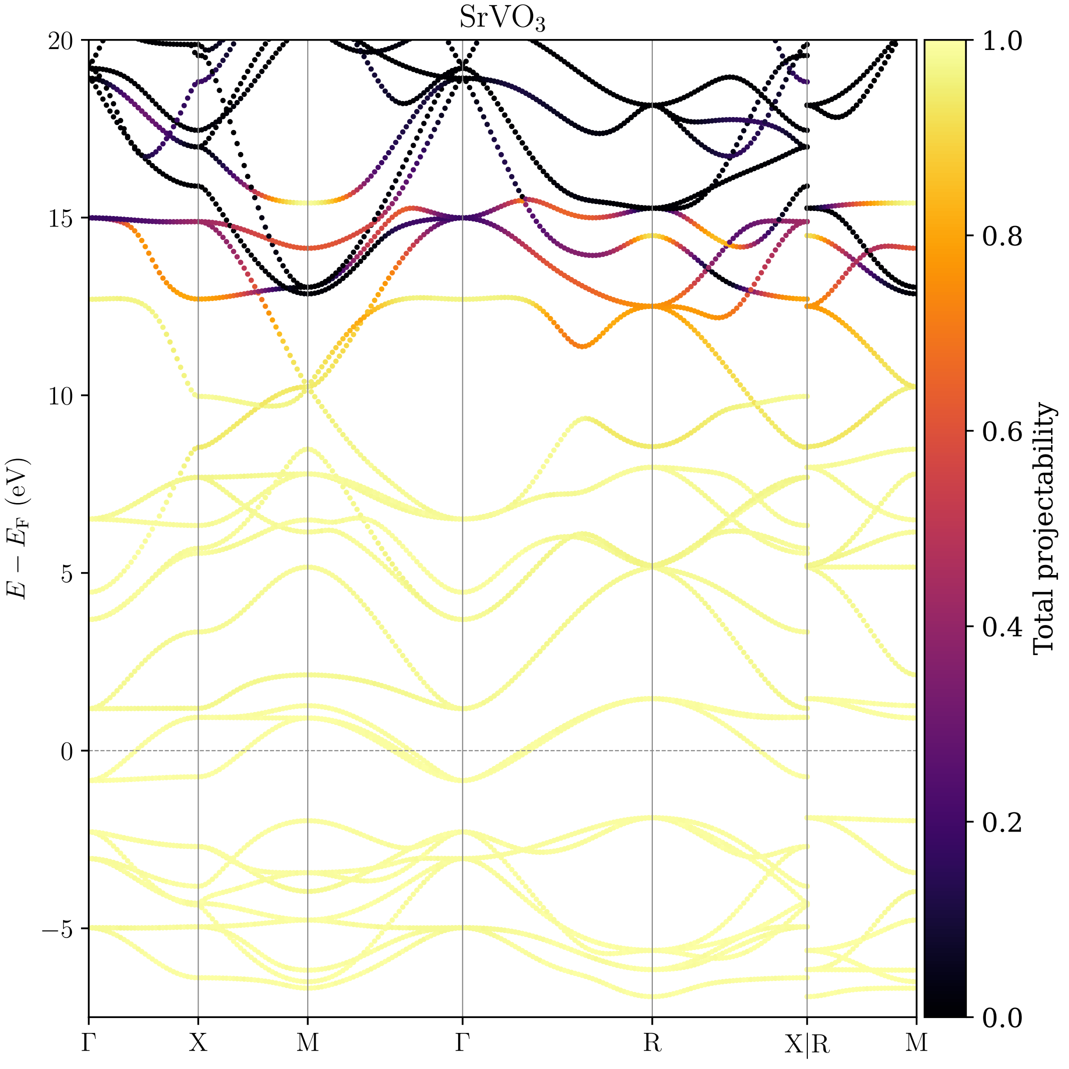

Supplementary Figure 8: $SrVO_3$ band structure projectability.

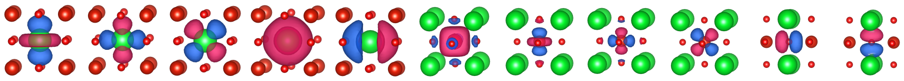

	character	d_{z^2}	$d_{x^2-y^2}$	d_{xy}, d_{xz}, d_{yz}	s	p_x, p_y, p_z	hybrid	d_{z^2}	$d_{x^2-y^2}$	d_{xy}, d_{xz}, d_{yz}	$p(O-V)$	$p(O-Sr)$
	multiplicity	1	1	3	1	3	1	1	1	3	3	6
**hydrogenic ED**	$d_{WFC}(Å)$	0.000	0.000	0.000	0.000	0.000	0.000	0.000	0.000	0.000	0.000	0.000
	$\Omega_{WF}(Å^2)$	1.287	1.287	1.395	2.648	2.717	1.758	0.527	0.527	0.660	0.626	0.646

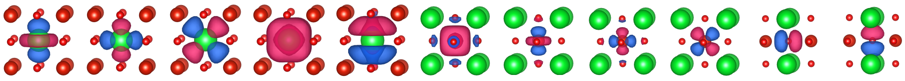

	character	d_{z^2}	$d_{x^2-y^2}$	d_{xy}, d_{xz}, d_{yz}	hybrid	p_x, p_y, p_z	hybrid	d_{z^2}	$d_{x^2-y^2}$	d_{xy}, d_{xz}, d_{yz}	$p(O-V)$	$p(O-Sr)$
	multiplicity	1	1	3	1	3	1	1	1	3	3	6
**PAO ED**	$d_{WFC}(Å)$	0.000	0.000	0.000	0.000	0.000	0.000	0.000	0.000	0.000	0.000	0.000
	$\Omega_{WF}(Å^2)$	1.022	1.022	2.178	2.850	2.792	1.778	0.528	0.528	0.643	0.618	0.642

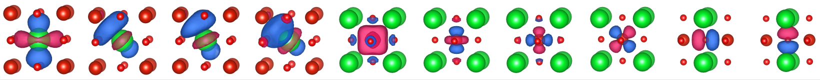

	character	d_{z^2}	hybrid	hybrid	hybrid	hybrid	d_{z^2}	$d_{x^2-y^2}$	d_{xy}, d_{xz}, d_{yz}	$p(O-V)$	$p(O-Sr)$
	multiplicity	1	2	2	4	1	1	1	3	3	6
**PAO PD**	$d_{WFC}(Å)$	0.113	0.922	0.776	0.930	0.000	0.000	0.000	0.000	0.000	0.000
	$\Omega_{WF}(Å^2)$	2.042	2.266	2.249	2.215	3.095	0.558	0.558	0.648	0.628	0.587
**PAO PD+ED**	$d_{WFC}(Å)$	0.113	0.923	0.778	0.929	0.000	0.000	0.000	0.000	0.000	0.000
	$\Omega_{WF}(Å^2)$	2.043	2.267	2.250	2.215	3.097	0.559	0.558	0.648	0.632	0.587

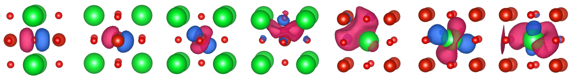

**SCDM**	$d_{WFC}(Å)$	0.020	0.012	0.005	0.681	1.382	0.325	0.225
	$\Omega_{WF}(Å^2)$	0.825	0.825	0.693	17.260	9.450	3.717	6.906
	$d_{WFC}(Å)$	0.024	0.006	0.012	0.807	1.370	0.406	
	$\Omega_{WF}(Å^2)$	0.849	0.793	0.776	16.208	8.097	4.249	
	$d_{WFC}(Å)$	0.037	0.013	0.012			0.106	
	$\Omega_{WF}(Å^2)$	0.888	0.789	0.682			3.285	
	$d_{WFC}(Å)$		0.017	0.010			0.137	
	$\Omega_{WF}(Å^2)$		0.691	0.718			3.369	
	$d_{WFC}(Å)$		0.011	0.002			0.107	
	$\Omega_{WF}(Å^2)$		0.788	0.604			4.414	
	$d_{WFC}(Å)$		0.016					
	$\Omega_{WF}(Å^2)$		0.693					

Supplementary Figure 9: $SrVO_3$ MLWF shapes, centers, and spreads for hydrogenic with ED, SCDM, PAO with ED, PAO with PD, and PAO with PD+ED. For SCDM, all the 24 MLWFs are listed since their d_{WFC}, Ω_{WF}, and shapes are different. The 24 MLWFs are roughly categorized into 7 groups.

1.2 `pw2wannier90.x` parallelization

Since HT calculations are expensive, to improve their efficiency and fully utilize many-core CPUs we implement $k-$pool parallelization[1, 2] for `pw2wannier90.x`. Figure 10 shows the $k-$pool parallelization efficiency of our `pw2wannier90.x` implementation, tested on a $3 \times 3 \times 3$ silicon supercell. The speedup is defined as T_{n_k}/T_1 where T_{n_k} is the wallclock time of a calculation with the number of $k-$pools equals n_k. Almost all the time is spent in computing `mmn` matrices, and the $k-$pool implementation performs well at reducing the wallclock time, e.g. comparing $n_k = 16$ with $n_k = 1$, the total wallclock time is reduced by 12 times.

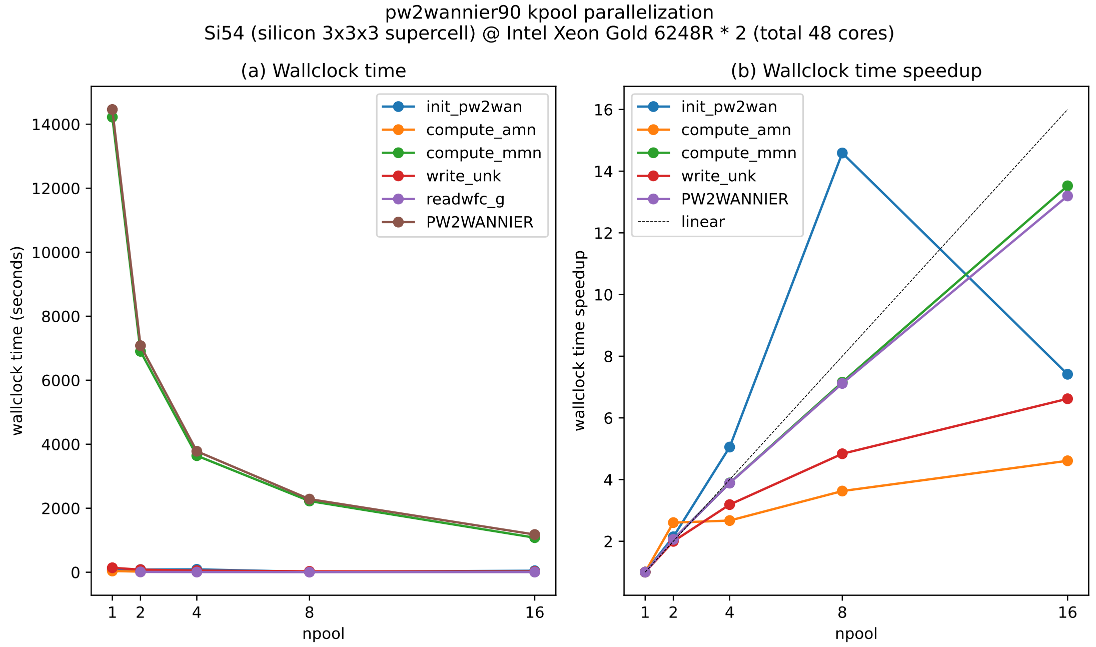

Supplementary Figure 10: $k-$pool parallelization efficiency of `pw2wannier90.x` implementation. (a) Wallclock time and (b) wallclock time speedup for initialization (`init_pw2wan`), computation of `amn` matrices using PAO (`compute_amn`), computation of `mmn` matrices (`compute_mmn`), writing of `unk` matrices (`write_unk`), reading of wavefunctions (`readwfc_g`), and the total time of pw2wannier90 (`PW2WANNIER`).

1.3 Band distance

To allow a better overview of the band-distance histograms, Figs. 11 and 12 show the zoom out of the corresponding Fig. 5 in the main text. As discussed in the main text, the PDWF η_0 and η_2 are much more clustered towards $\eta = 0$. The conclusions are the same for $\eta_0^{\max}$ and $\eta_2^{\max}$, as shown in Fig. 12. Notice that in Figs. 11 and 12, for η_0, η_2, $\eta_0^{\max}$, and $\eta_2^{\max}$, the SCDM cumulative histograms do not reach 200 at the rightmost position of horizontal axes, i.e., there are some outliers with $\eta > 100\,\mathrm{meV}$ or $\eta^{\max} > 500\,\mathrm{meV}$. To allow comparison of successful calculations, i.e., excluding outlier calculations, we listed the $\eta^{\leq 20}$ (i.e., the average of all the calculations for which $\eta \leq 20\,\mathrm{meV}$), $\eta^{\leq 100}$, $\eta^{\max,\leq 100}$ (the average of all the calculations for which $\eta^{\max} \leq 100\,\mathrm{meV}$), and $\eta^{\max,\leq 500}$ in Table 1. N in Table 1 denotes the number of successful calculations, which participate in the averagings of $\eta^{\leq 20}$, $\eta^{\leq 100}$, $\eta^{\max,\leq 100}$, or $\eta^{\max,\leq 500}$. As expected, SCDM is relatively better at interpolating high-energy conduction bands, while PDWF is more accurate at occupied and near-Fermi-energy bands.

For $\eta_4^{\leq 20}$, although PDWF (4.939 meV) is still more accurate than SCDM (around 5.892 meV), the SCDM outperforms PDWF with a higher success rate (171/200 = 85.5% compared with 108/200 = 54%). However, as we are not freezing all the states below $E_\mathrm{F} + 4\,\mathrm{eV}$, this is expected and in most practical cases it is not an issue since we usually focus on low-energy physics. In Table 1, we also compare $\eta_0^{\max,\leq 100}$, $\eta_2^{\max,\leq 100}$, $\eta_4^{\max,\leq 100}$, and extending the comparison range, i.e., $\eta_0^{\leq 100}$, $\eta_2^{\leq 100}$, $\eta_4^{\leq 100}$, $\eta_0^{\max,\leq 500}$, $\eta_2^{\max,\leq 500}$, $\eta_4^{\max,\leq 500}$, obtaining the same conclusions.

Supplementary Table 1: Average of band distances for PDWF and SCDM projections. η_ν is the average of average band distance of the 200 structures below $E_\text{F} + \nu$, where $\nu = 0$, 2, or 4 eV. $\eta_\nu^{\leq 20}$ is the average of structures with $\eta_\nu \leq$ 20 meV. η_ν^{max} is the average of max band distance of the 200 structures. $\eta_\nu^{\text{max},\leq 100}$ is the average of structures with $\eta_\nu^{\text{max}} \leq 100$ meV. N is the number of structures participating in the averagings.

Type	PDWF		SCDM	
	η (meV)	N	η (meV)	N
η_0	2.685	200	10.278	200
η_2	4.231	200	11.201	200
η_4	34.888	200	16.456	200
$\eta_0^{\leq 20}$	1.902	196	5.094	185
$\eta_2^{\leq 20}$	2.922	193	5.280	183
$\eta_4^{\leq 20}$	4.939	108	5.892	171
$\eta_0^{\leq 100}$	2.685	200	6.953	198
$\eta_2^{\leq 100}$	4.231	200	7.714	198
$\eta_4^{\leq 100}$	22.701	179	9.621	195
η_0^{max}	20.392	200	73.238	200
η_2^{max}	36.743	200	84.011	200
η_4^{max}	294.971	200	139.696	200
$\eta_0^{\text{max},\leq 100}$	12.360	193	30.933	171
$\eta_2^{\text{max},\leq 100}$	19.024	181	32.155	165
$\eta_4^{\text{max},\leq 100}$	18.739	83	35.592	146
$\eta_0^{\text{max},\leq 500}$	20.392	200	51.389	197
$\eta_2^{\text{max},\leq 500}$	32.038	198	56.346	196
$\eta_4^{\text{max},\leq 500}$	132.687	152	78.399	190

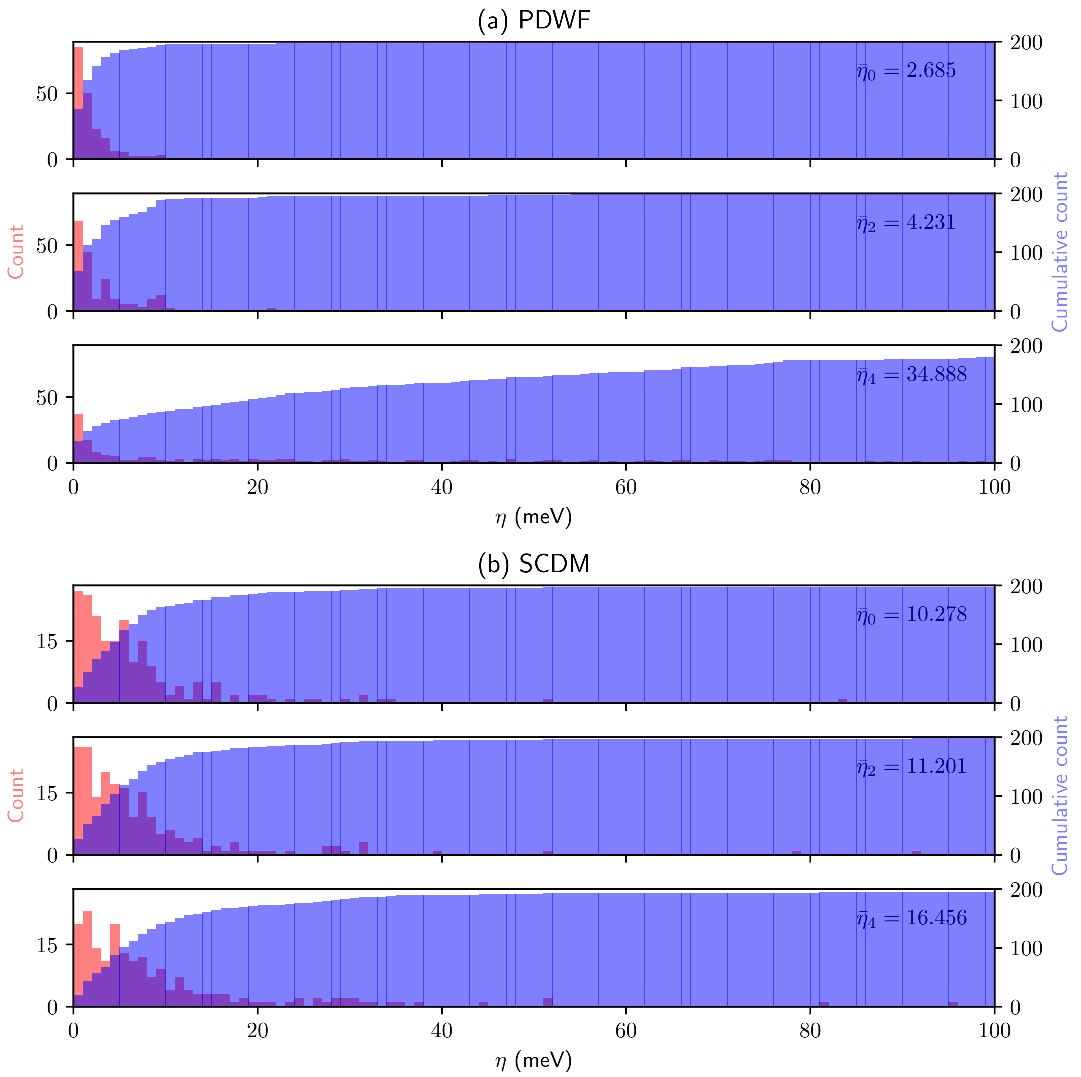

Supplementary Figure 11: Histograms (red) and cumulative histograms (blue) of band distances η_0, η_2, and η_4 for 200 structures, using (a) PDWF and (b) SCDM. The top, center, and bottom plots in each panel are η_0, η_2, and η_4, respectively. The $\bar{\eta}$ at the top right of each panel is the mean value for the 200 structures.

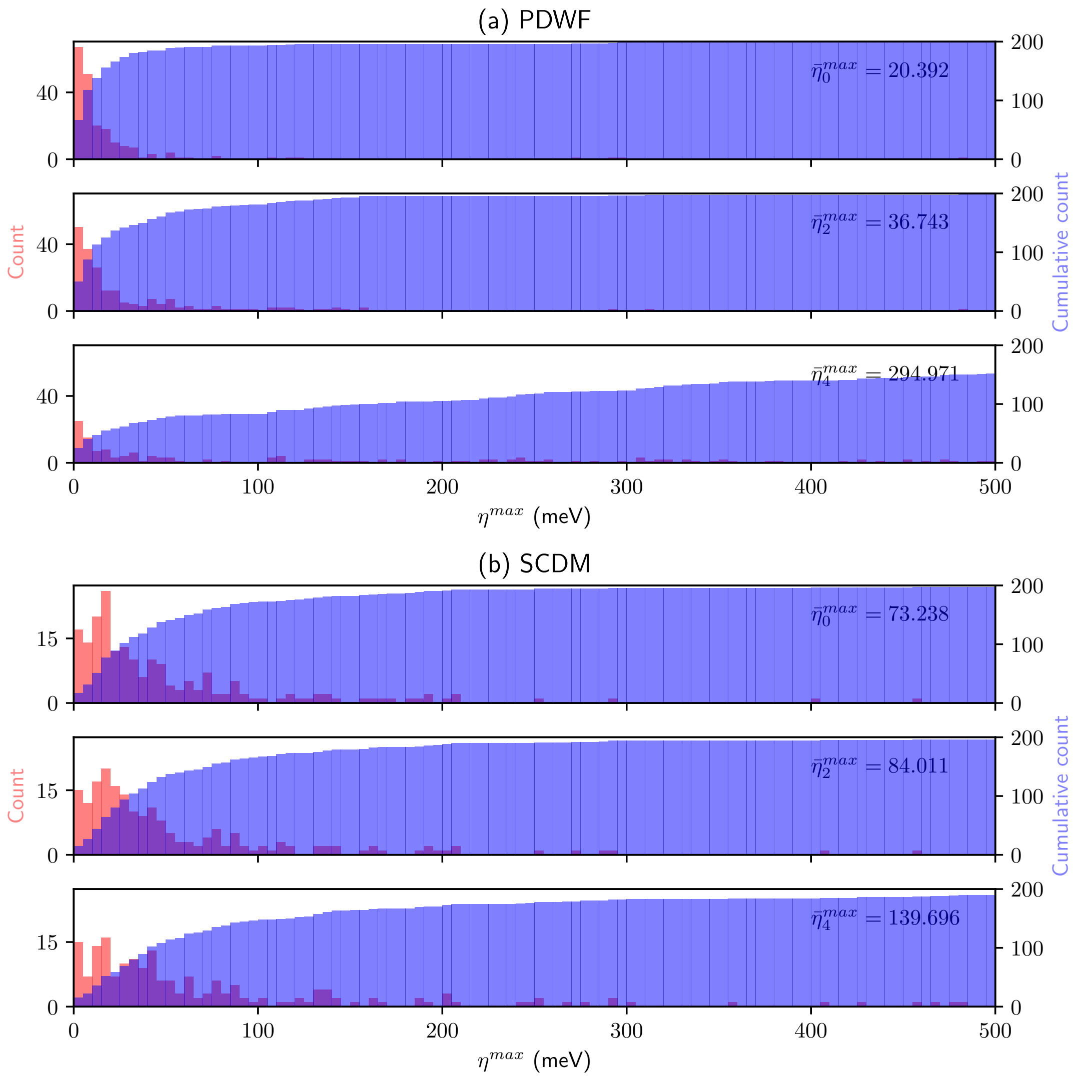

Supplementary Figure 12: Histograms (red) and cumulative histograms (blue) of band distances $\eta_0^{\max}$, $\eta_2^{\max}$, and $\eta_4^{\max}$ for 200 structures, using (a) PDWF and (b) SCDM. The top, center, and bottom plots for each panel are $\eta_0^{\max}$, $\eta_2^{\max}$ and $\eta_4^{\max}$, respectively. The $\bar{\eta}^{\max}$ at the top right of each panel is the mean value for the 200 structures. This is a zoom-out of Fig. 11.

1.4 MLWF centers

We list in Table 2 the average distance of the WF centers from their nearest neighboring (NN) atom and their next-nearest neighboring (NNN) atom, for the initial WFs (i.e. after disentanglement but before maximal localization) and the final MLWFs (i.e. after maximal localization). The corresponding histograms are shown in Fig. 13. For PDWF, the initial WFs are mostly atom-centered ($d^i_{\mathrm{NN}} = 0.07\,\text{Å}$). The maximal localization pushes the final MLWFs slightly further away ($d^f_{\mathrm{NN}} = 0.43\,\text{Å}$). Inspecting the ratio of $d^{i(f)}_{\mathrm{NN}}/d^{i(f)}_{\mathrm{NNN}}$, both the initial WFs and final MLWFs are still much closer to NN than NNN atoms. For SCDM, both initial WFs and final MLWFs are farther away from NN atoms than PDWF.

Supplementary Table 2: The average distance of WF center from the NN or NNN atom of the 200 structures for PDWF and SCDM methods. d_{NN} and d_{NNN} refer to the distance of the WF center from its NN and NNN atoms, respectively. d^i and d^f are the averages of the initial and the final WF center distances of the 200 structures (in total 6818 WFs), respectively. $d^{i(f)}_{\mathrm{NN}}/d^{i(f)}_{\mathrm{NNN}}$ is the average of the ratio of each WF.

d (Å)	PDWF	SCDM
d^i_{NN}	0.07	0.51
d^i_{NNN}	2.36	2.11
d^f_{NN}	0.43	0.53
d^f_{NNN}	2.19	2.11
$d^i_{\mathrm{NN}}/d^i_{\mathrm{NNN}}$	0.03	0.28
$d^f_{\mathrm{NN}}/d^f_{\mathrm{NNN}}$	0.22	0.29

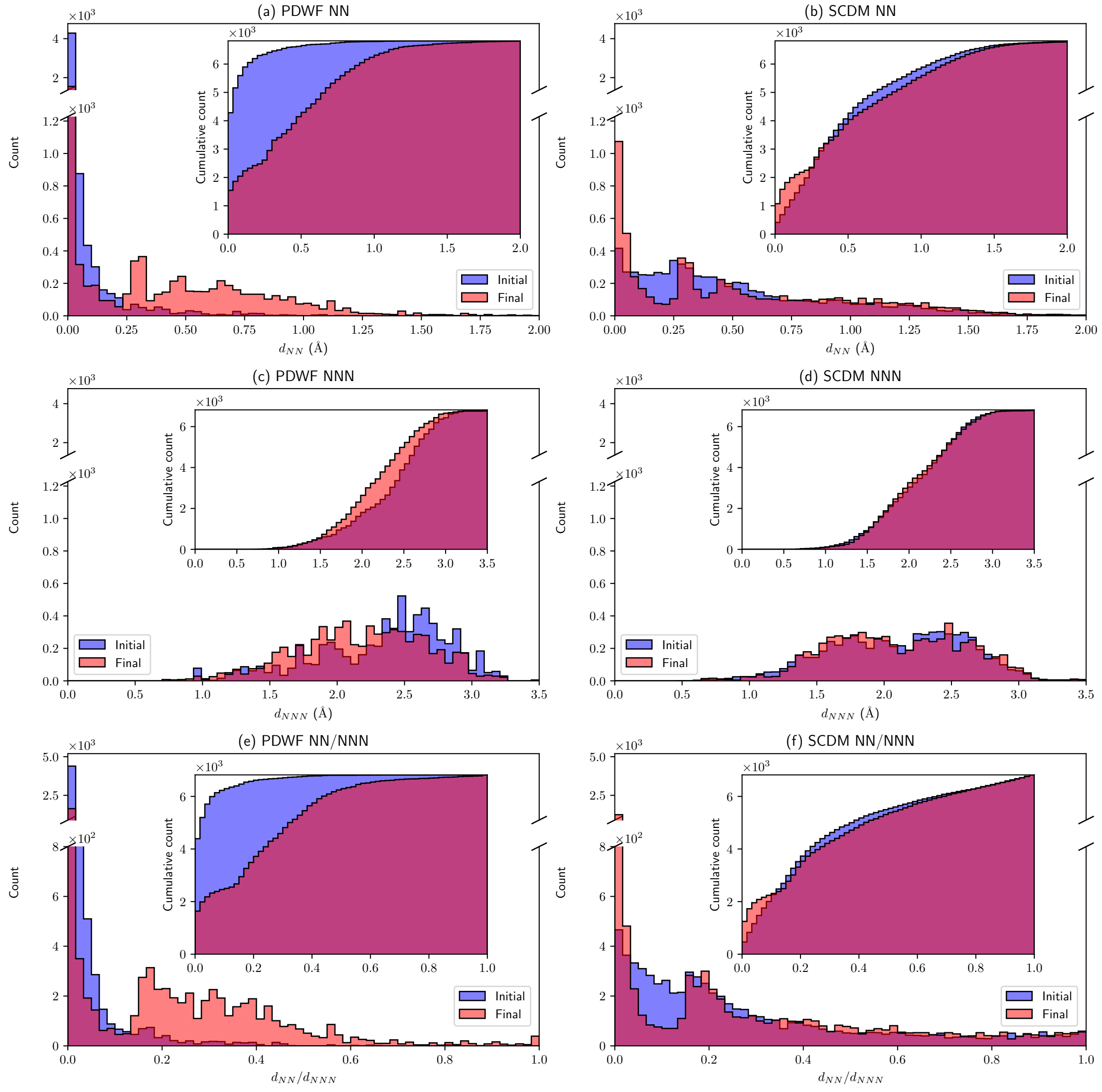

Supplementary Figure 13: Histograms of the distance of WF centers from their (a) NN atom using PDWF, (b) NN atom using SCDM, (c) NNN atom using PDWF, (d) NNN atom using SCDM; (e) the ratio $d_{\mathrm{NN}}/d_{\mathrm{NNN}}$ using PDWF, and (f) the ratio $d_{\mathrm{NN}}/d_{\mathrm{NNN}}$ using SCDM, of the 200 structures. The insets show the cumulative histogram of each panel. Note that in each histogram the deep red area is the overlap of the initial (red) and the final (blue).

1.5 MLWF spreads

Table 3 and Fig. 14 shows both the initial and final spreads of WFs. The average spreads excluding outliers, i.e., $\Omega^{i(f),\leq 10}$ and $\Omega^{i(f),\leq 20}$ are also listed in Table 3. In all cases, PDWF MLWFs are more localized than SCDM MLWFs.

Supplementary Table 3: Average of WF spreads of the 200 structures for PDWF and SCDM projections. Ω^i and Ω^f are the averages of initial (after disentanglement, before maximal localization) and final (after disentanglement and maximal localization) WF spreads of the 200 structures (in total 6818 WFs), respectively. $\Omega^{i,\leq 10}$ is the average of WFs with $\Omega^i \leq 10\,\text{Å}^2$. N is the number of WFs participating in the averaging.

Type	PDWF		SCDM	
	Ω (Å^2)	N	Ω (Å^2)	N
Ω^i	2.72	6818	30.82	6818
Ω^f	1.41	6818	3.54	6818
$\Omega^{i,\leq 10}$	1.99	6626	3.83	5062
$\Omega^{f,\leq 10}$	1.38	6808	2.91	6501
$\Omega^{i,\leq 20}$	2.14	6708	4.68	5599
$\Omega^{f,\leq 20}$	1.40	6814	3.31	6765

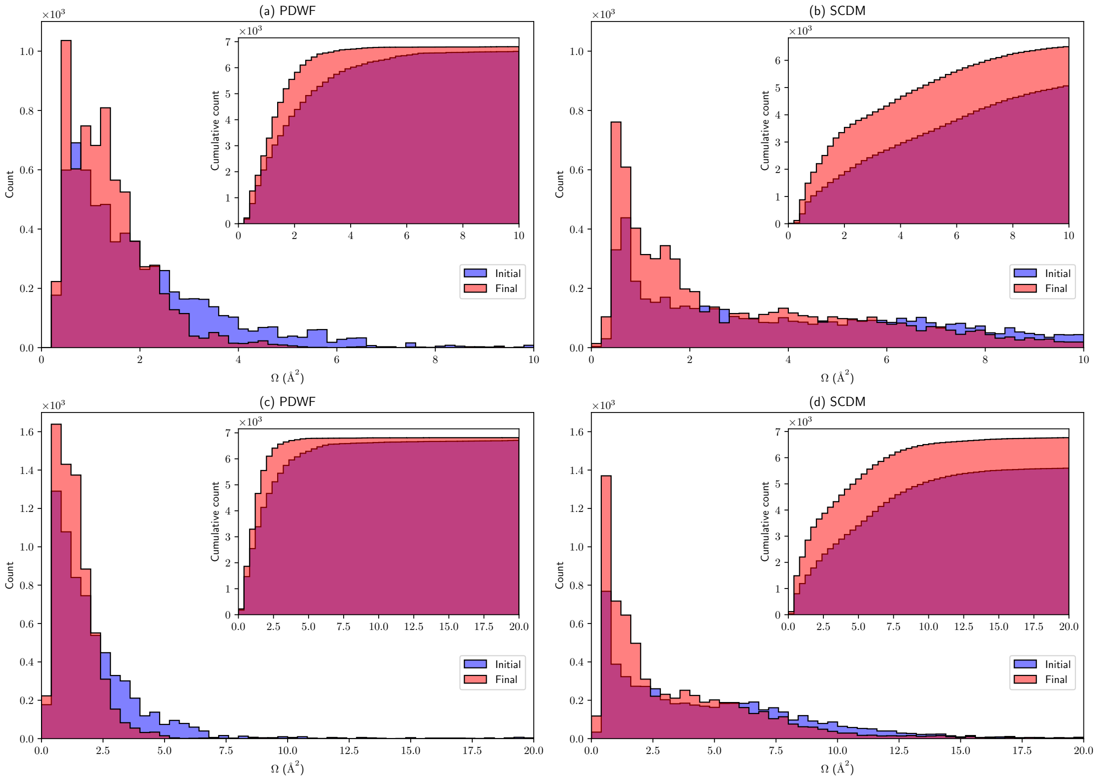

Supplementary Figure 14: Histogram of WF spreads for 200 structures using (a) PDWF with Ω in the range 0 Å^2 to 10 Å^2, (b) SCDM with Ω in the range 0 Å^2 to 10 Å^2, (c) PDWF with Ω in the range 0 Å^2 to 20 Å^2, and (d) SCDM with Ω in the range 0 Å^2 to 20 Å^2. The insets show the cumulative histogram of each panel. Note in each histogram, the deep red area is the overlap of the initial (red) and the final (blue).

1.6 High-throughput Wannierization

Supplementary Table 4: Average band distance for 21 737 structures from Materials Cloud. η_ν is the average band distance of all the structures below $E_\mathrm{F} + \nu$, where $\nu = 0$, 2, or 4 eV. $\eta_\nu^{\leq 20}$ is the average of structures with $\eta_\nu \leq 20$ meV. $\eta_\nu^{\max}$ is the max distance of all the structures. $\eta_\nu^{\max,\leq 100}$ is the average of structures with $\eta_\nu^{\max} \leq 100$ meV. N is the number of structures participating in the averagings.

Type	η (meV)	N
η_0	2.003	21737
η_2	3.685	21737
η_4	38.437	21737
$\eta_0^{\leq 20}$	1.129	21445
$\eta_2^{\leq 20}$	2.118	21259
$\eta_4^{\leq 20}$	4.054	11911
$\eta_0^{\leq 100}$	1.654	21703
$\eta_2^{\leq 100}$	2.885	21650
$\eta_4^{\leq 100}$	22.748	19100
$\eta_0^{\max}$	25.329	21737
$\eta_2^{\max}$	42.768	21737
$\eta_4^{\max}$	297.723	21737
$\eta_0^{\max,\leq 100}$	7.939	20980
$\eta_2^{\max,\leq 100}$	15.145	20217
$\eta_4^{\max,\leq 100}$	20.864	9769
$\eta_0^{\max,\leq 500}$	13.492	21567
$\eta_2^{\max,\leq 500}$	24.805	21453
$\eta_4^{\max,\leq 500}$	128.216	16593

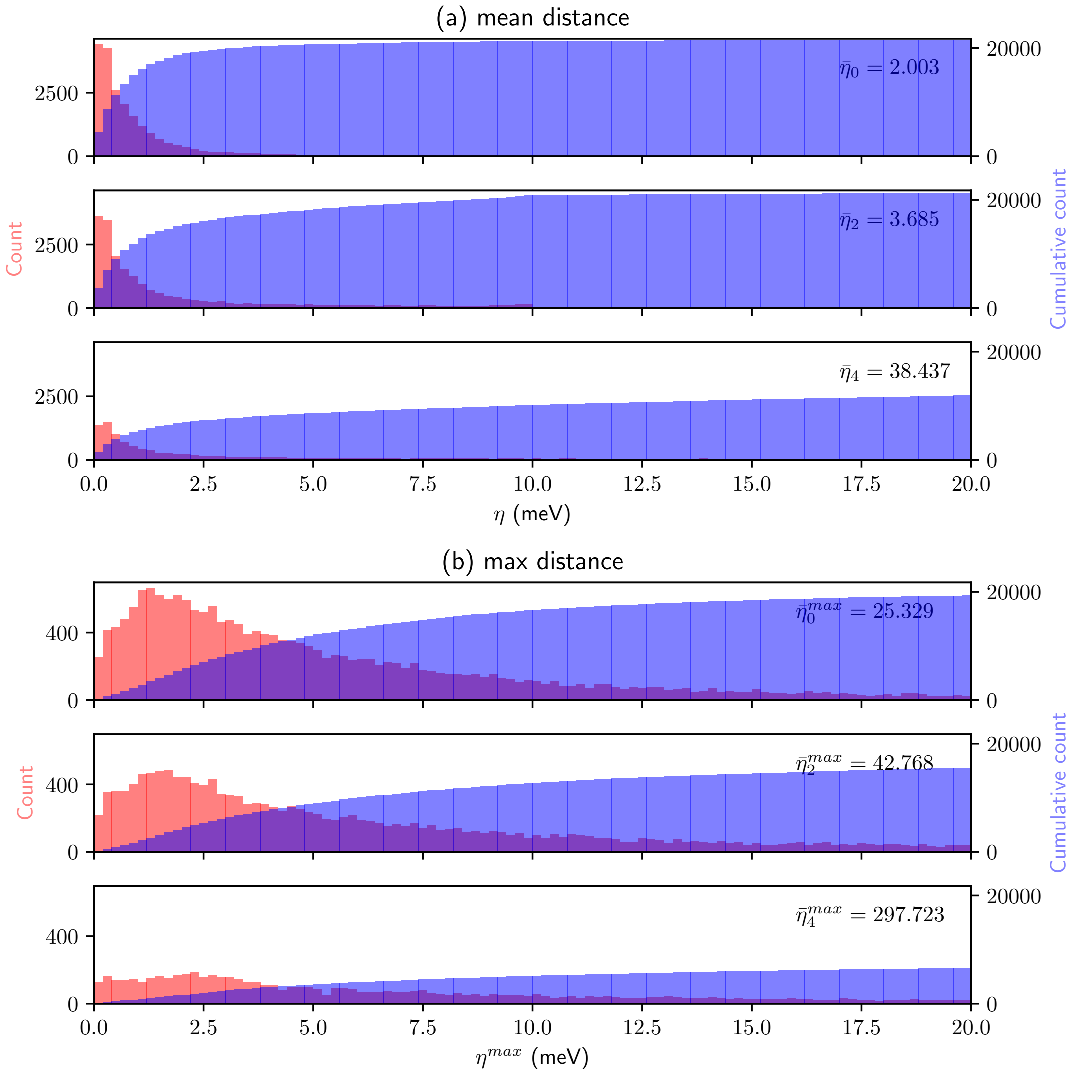

Supplementary Figure 15: Histograms (red) and cumulative histograms (blue) of band distances of 21 737 structures, for (a) η_0, η_2, and η_4, and (b) $\eta_0^{\max}$, $\eta_2^{\max}$, and $\eta_4^{\max}$. The $\bar{\eta}$ or $\bar{\eta}^{\max}$ at the top right of each panel is the mean value for the 21 737 structures.

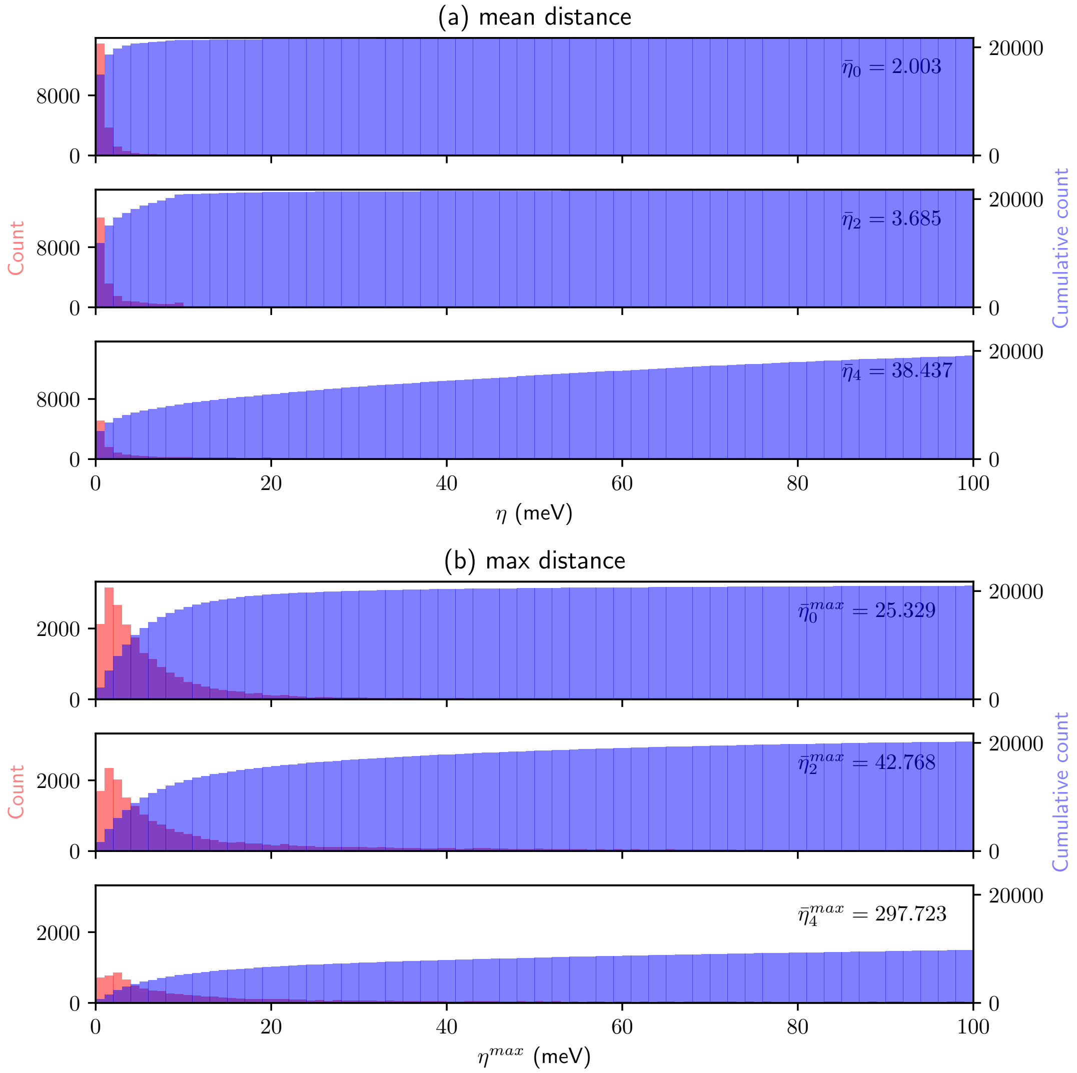

Supplementary Figure 16: Histograms (red) and cumulative histograms (blue) of band distances of 21 737 structures, for (a) η_0, η_2, and η_4, and (b) $\eta_0^{\max}$, $\eta_2^{\max}$, and $\eta_4^{\max}$. The $\bar{\eta}$ or $\bar{\eta}^{\max}$ at the top right of each panel is the mean value for the 21 737 structures. This is a zoom-out of Fig. 15.

1.7 Additional PAOs

Usually, pseudopotentials are pseudized to accurately reproduce all-electron calculations at specified energies. The targets are often the states below and a few eV above Fermi energy. Most often the pseudopotentials also contains a set of PAOs which normally are good basis vectors that can span the valence bands and a few conduction bands of solids, as indicated by their projectability. However, in some cases where the chemical environment of solid is significantly different from that of atoms, the projectability of states around the Fermi energy on all available PAOs is low. In other cases, one may want to Wannierize higher conduction bands. To tackle these difficulties, we can add additional higher-angular-momentum projectors to satisfy these needs. Take silicon as an example, to Wannierize both valence and conduction bands, one s and three p projectors for each silicon atom are used to generate 8 MLWFs, however, they cannot accurately interpolate bands 5 eV above E_{F}, see Fig. 18a. If 10 additional localized projectors, e.g., five d PAOs on each silicon atom, are introduced, the higher conduction bands can be accurately described (also with a higher inner energy window), see Fig. 18d.

In Fig. 18, the PAOs from the `OpenMX` package [3] (PAO `DFT_DATA19`) are used to generate 18 MLWFs. The `OpenMX` PAOs are the solutions of the Schrödinger equation in a confined potential, thus they are always localized and can be alternatively used as atomic projectors for MLWFs. This is similar to the inclusion of polarization orbitals in the LCAO method[4]. Comparing Fig. 18a and Fig. 18b, the s and p PAOs from the QE pseudopotential and from the `OpenMX` PAOs essentially have the same quality of band interpolation. The inclusion of d PAOs allows raising the maximum inner energy window, thus resulting in a much more accurate interpolation of bands, as shown in Figs. 18c and 18d. Figures 3 and 19 show the MLWF shapes for pseudopotential PAOs and `OpenMX` PAOs, respectively. The ten additional d MLWFs are clearly visible in Fig. 19. When raising the maximum inner energy window to CBM + 10 eV, the MLWFs are mixtures of atomic orbitals and high-energy conduction bands, losing the physical picture of TB atomic orbitals. Instead, in the ED (maximum inner energy window = CBM + 2 eV), PD, or PD+ED cases, the MLWFs still preserve the s, p, or d characters similar to the original `OpenMX` PAO projectors.

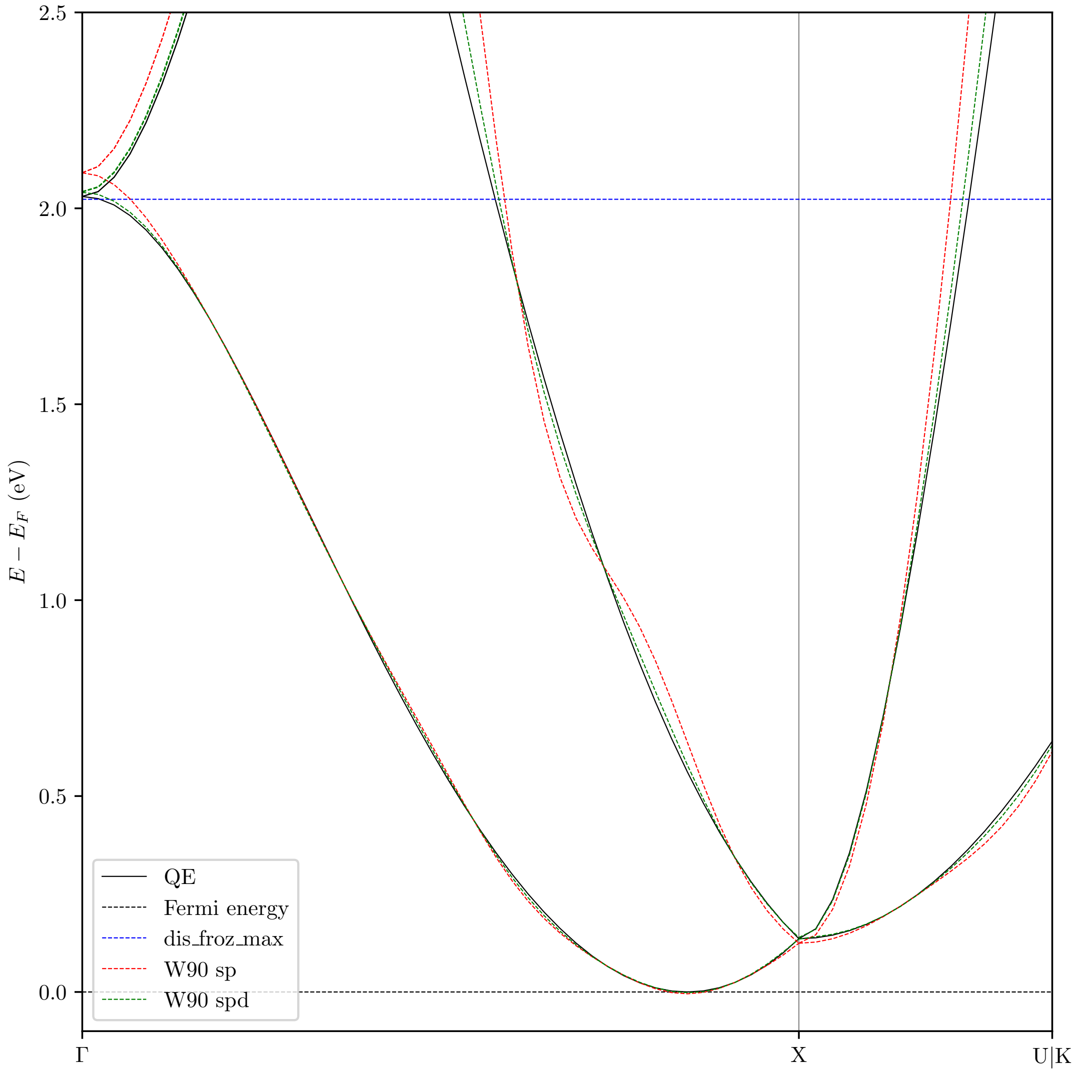

Supplementary Figure 17: Comparison of QE bands (black lines) and Wannier-interpolated bands using `OpenMX` s, p projections (red dashed lines) and `OpenMX` s, p, d projections (green dashed lines). Results using QE s, p projections are not shown since they exactly overlap with that from `OpenMX` s, p projections. The MLWFs are obtained using `OpenMX` PAOs and ED with the maximum of the inner energy window (blue dashed line) set to CBM + 2 eV (actually slightly larger than CBM + 2 eV since in the `AiiDA` workflow the CBM is automatically computed based on the eigenenergies only on the regular $k-$point mesh of the non-self-consistent calculation, that does not include the exact $k-$point where the CBM is located). The CBM (horizontal black dashed line) is at zero. Note that the interpolation quality can be further improved by increasing the $k-$point sampling density.

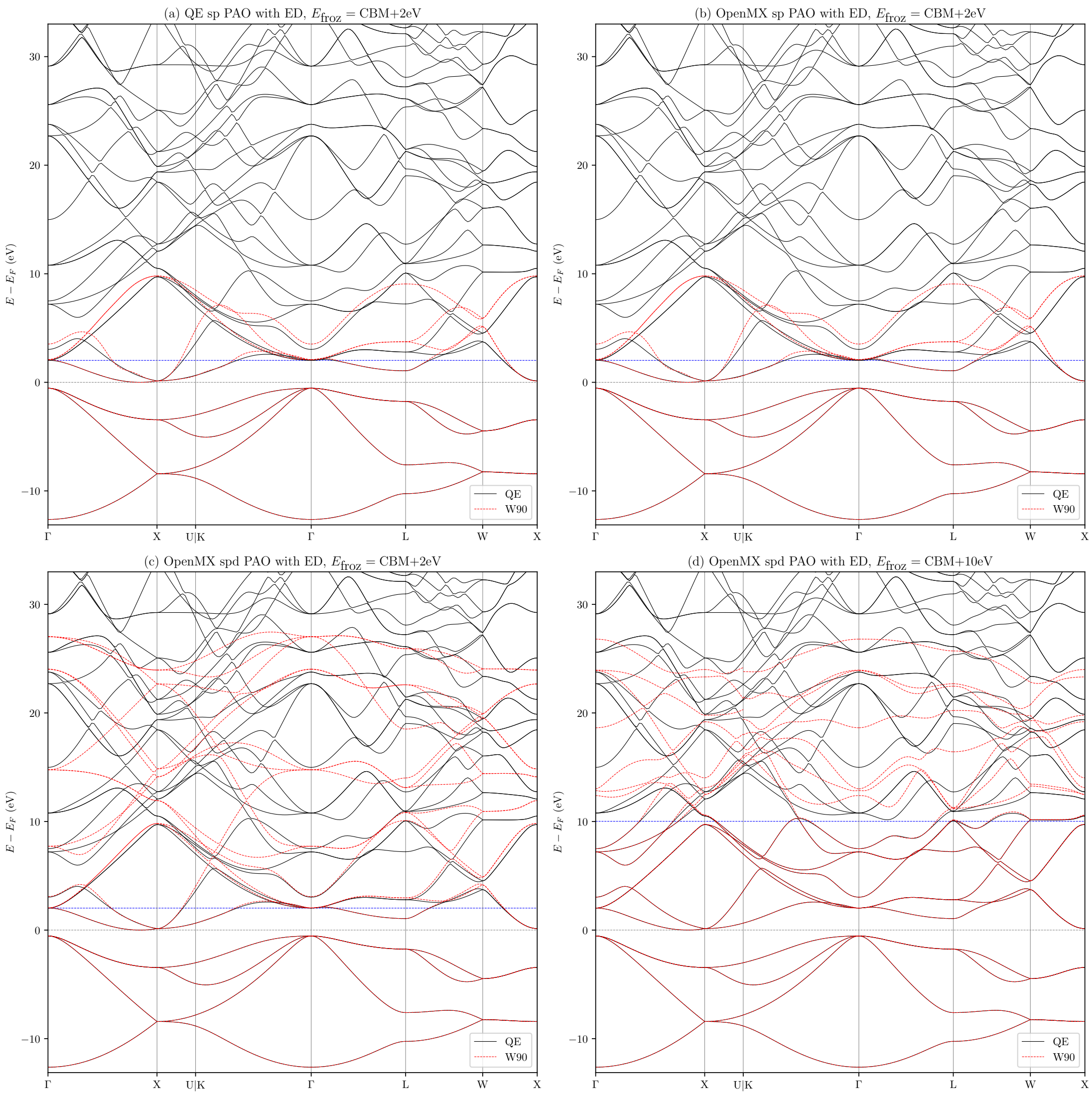

Supplementary Figure 18: Comparisons between QE bands (black lines) and Wannier-interpolated bands (red dashed lines) using (a) QE pseudopotential PAOs with ED, where the maximum inner energy window is set as CBM + 2 eV, (b) `OpenMX` s, p PAOs with ED, where the maximum inner energy window is set as CBM + 2 eV, (c) `OpenMX` s, p, d PAOs with ED, where the maximum inner energy window is set as CBM + 2 eV, and (d) `OpenMX` s, p, d PAOs with ED, where the maximum inner energy window is set as CBM + 10 eV. The Fermi energy E_{F} (horizontal black dashed line) is at zero. The horizontal blue dashed line denotes the maximum inner energy window.

		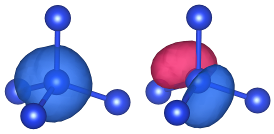				
	multiplicity	2	6			
**sp ED**	$d_{WFC}(\AA)$<br>$\Omega_{WF}(\AA^2)$	0.000<br>1.849	0.000<br>2.546			
**sp PD**	$d_{WFC}(\AA)$<br>$\Omega_{WF}(\AA^2)$	0.000<br>1.060	0.000<br>1.256			
**sp PD+ED**	$d_{WFC}(\AA)$<br>$\Omega_{WF}(\AA^2)$	0.000<br>1.844	0.000<br>2.550			
		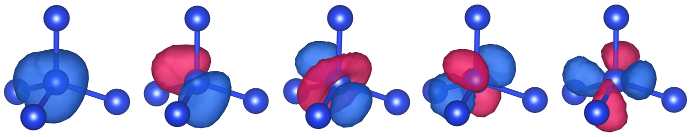				
	multiplicity	2	6	2	2	6
	character	s	p_x, p_y, p_z	d_{z^2}	$d_{x^2-y^2}$	d_{xy}, d_{xz}, d_{yz}
**spd ED**	$d_{WFC}(\AA)$<br>$\Omega_{WF}(\AA^2)$	0.000<br>1.101	0.000<br>1.411	0.000<br>1.451	0.000<br>1.451	0.000<br>1.557
**spd PD**	$d_{WFC}(\AA)$<br>$\Omega_{WF}(\AA^2)$	0.000<br>1.121	0.000<br>1.278	0.000<br>1.352	0.000<br>1.352	0.000<br>1.327
**spd PD+ED**	$d_{WFC}(\AA)$<br>$\Omega_{WF}(\AA^2)$	0.000<br>1.101	0.000<br>1.412	0.000<br>1.454	0.000<br>1.454	0.000<br>1.556
		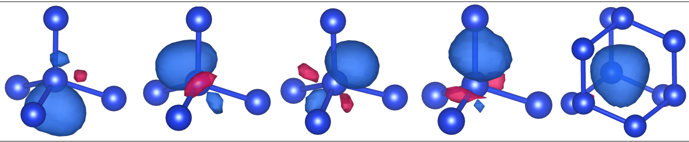				
**spd ED CBM + 10 eV**	$d_{WFC}(\AA)$<br>$\Omega_{WF}(\AA^2)$	0.905<br>1.082	0.722<br>0.928	0.752<br>1.141	0.995<br>1.235	1.793<br>1.459
	$d_{WFC}(\AA)$<br>$\Omega_{WF}(\AA^2)$	0.807<br>0.985	0.77<br>1.055	0.738<br>1.053	0.952<br>1.214	1.877<br>1.428
	$d_{WFC}(\AA)$<br>$\Omega_{WF}(\AA^2)$	0.835<br>1.103	0.798<br>0.988	0.749<br>1.125		
	$d_{WFC}(\AA)$<br>$\Omega_{WF}(\AA^2)$	0.917<br>1.112	0.735<br>0.932	0.752<br>1.043		
	$d_{WFC}(\AA)$<br>$\Omega_{WF}(\AA^2)$	0.827<br>1.110	0.787<br>1.022			

Supplementary Figure 19: Silicon MLWF shapes, centers, and spreads for `OpenMX` s+p or `OpenMX` s+p+d PAOs as starting projections, with ED, PD, and PD+ED disentanglements, where for ED and PD+ED the maximum of the inner energy window is set at CBM + 2 eV. In addition, we also show the results for ED disentanglement when the maximum of the inner energy window is set as CBM + 10 eV. In this latter case, all the 18 MLWFs are listed (and roughly categorized into five groups) since their d_{WFC}, Ω_{WF}, and shapes are different; as discussed in main text Section II B, including the higher-energy states breaks the atomic-like picture of these WFs.

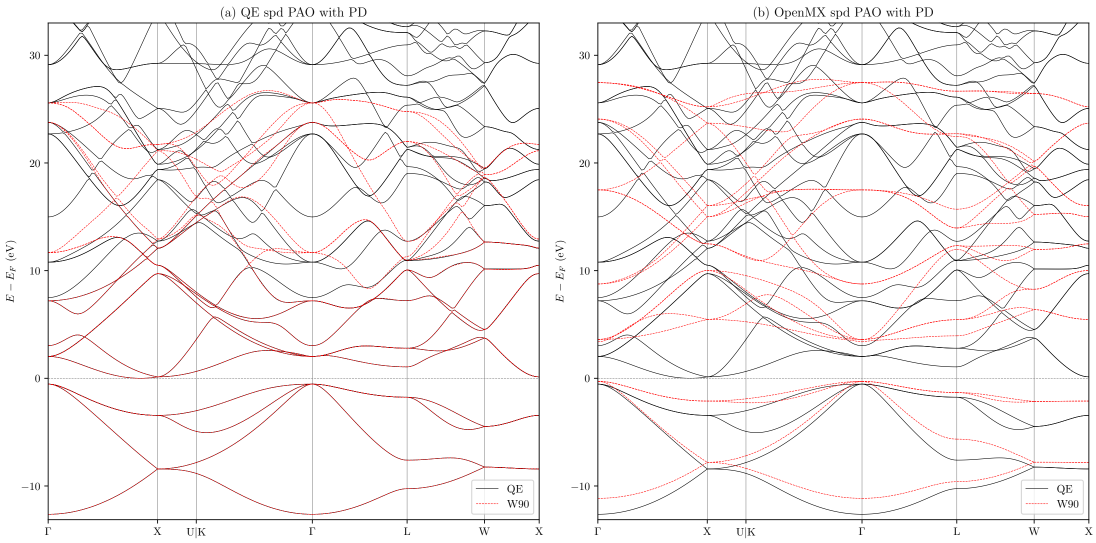

Supplementary Figure 20: Comparisons between QE bands (black lines) and Wannier-interpolated bands (red dashed lines) using (a) a newly generated custom pseudopotential including $3d$ PAOs with PD, (b) `OpenMX` s, p, d PAOs with PD. The Fermi energy E_{F} (horizontal black dashed line) is at zero.

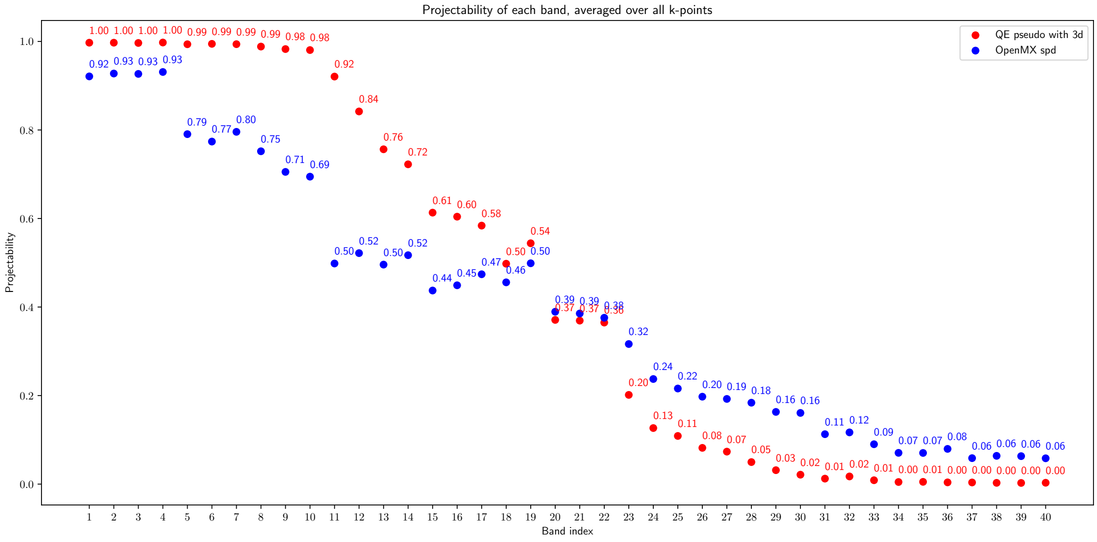

Supplementary Figure 21: Band projectabilities using the orbitals from our newly generated custom pseudopotential including $3d$ PAOs (red) and `OpenMX` s, p, d PAOs (blue).

Supplementary References

[1] Giannozzi, P. *et al.* QUANTUM ESPRESSO: a modular and open-source software project for quantum simulations of materials. *J. Phys.: Condens. Matter* **21**, 395502 (2009).

[2] Giannozzi, P. *et al.* Quantum ESPRESSO toward the exascale. *J. Chem. Phys.* **152**, 154105 (2020).

[3] Ozaki, T. & Kino, H. Numerical atomic basis orbitals from H to Kr. *Phys. Rev. B* **69**, 195113 (2004).

[4] Hariharan, P. C. & Pople, J. A. The influence of polarization functions on molecular orbital hydrogenation energies. *Theor. Chim. Acta* **28**, 213–222 (1973).

[5] Pizzi, G. *et al.* Wannier90 as a community code: new features and applications. *J. Phys.: Condens. Matter* **32**, 165902 (2020).

2 Supplementary Figures

2.1 Band structure and Hamiltonian decay

In this section, we show on each page the band structures, band distances, and Hamiltonian decay for each of the 200 structures.

On each page, the left (right) column shows the band structure comparison between `Quantum ESPRESSO`, labeled QE, and Wannier-interpolated bands from PDWF (SCDM) method. The Fermi energy E_{F} is at zero. The horizontal blue dashed line denotes the `dis_froz_max` in the PDWF method (note in the PDWF method some high-projectability states which are higher than the `dis_froz_max` are also frozen, but they are not explicitly shown in the figure). The title shows the structural formula and a description of the calculation, and the UUID of the corresponding `AiiDA` calculations for the QE band structure (PW) and Wannier-interpolated bands (W90). In general, there are two types of description: (a) "exclude semicore": the semicore states are excluded from Wannierization, this is the default setting; (b) "INCLUDE semicore": the semicore states are included in Wannierization, since the semicore states overlap with valence states, Wannierization quality would be worse if the semicore states were excluded.

The table at the bottom left shows the band distances between the band structures. All the η are in meV units. The last column of the table shows the Hamiltonian decay τ in Å units.

Finally, the bottom figure shows the Hamiltonian decay and its logarithmic fitting for both PDWF and SCDM methods. Note since the minimal-distance replica selection method is used in the Wannier interpolation [5], when plotting the figures, the $\mathbf{R}$ vector (shown on the x axis) already contains the translation vector $\mathbf{T}$, which is chosen to minimize the distance between two WF centers $\mathbf{r}_m$ (of $|w_{m\mathbf{0}}\rangle$) and $\mathbf{r}_n$ (of $|w_{n\mathbf{R}}\rangle$):

$$\arg\min_{\mathbf{T}} |\mathbf{r}_m - (\mathbf{r}_n + \mathbf{R} + \mathbf{T})| . \quad (1)$$

List of 200 structures (Hill formula, in alphabetic order)

1. Ag_2Hf_2 34
2. Ag_2Sc 35
3. Ag_2Ti_4 36
4. Ag_2Y 37
5. Ag_4Sc_2 38
6. AgMg 39
7. AgSc 40
8. AgY 41
9. $Al_{12}Ni_4$ 42
10. $Al_{18}Co_4$ 43
11. $Al_{24}W_6$ 44
12. Al_2Mo_6 45
13. Al_2Nb_6 46
14. Al_2Os 47
15. Al_3Nb 48
16. Al_3Ta 49
17. $Al_4Mn_2O_8$ 50
18. $Al_4O_8Zn_2$ 51
19. Al_4Sc_2 52
20. Al_4Y_2 53
21. Al_8Zr_4 54
22. AlCo 55
23. AlFe 56
24. $AlHfNi_2$ 57
25. AlIr 58
26. $AlRe_2$ 59
27. Ar_2 60
28. As_2Ni_2 61
29. $As_4O_{16}Y_4$ 62
30. $As_8Si_4Zn_4$ 63
31. AsB 64
32. Au_2Hf_2 65
33. Au_2Nb 66
34. Au_2Pb_4 67
35. Au_2Sc 68
36. Au_2Y 69
37. Au_4V_2 70
38. AuMn 71
39. $B_{16}Mg_4$ 72
40. B_2Cr 73
41. B_2Hf 74
42. B_2Mn 75
43. B_2Ta 76
44. B_2Ti 77
45. B_2Y 78
46. B_4Co_4 79
47. B_4Ni_4 80
48. B_4Re_2 81
49. B_4V_6 82
50. B_6Si 83
51. $B_8H_{16}O_{16}$ 84
52. BaS 85
53. BaTe 86
54. BeO_4S 87
55. Bi_2Na_6 88
56. Bi_4Cl_{12} 89
57. BiNa 90
58. $Br_{18}W_3$ 91
59. Br_2Fe 92
60. Br_2Ti 93
61. Br_2V 94
62. Br_4Ca_2 95
63. Br_6Ni_3 96
64. Br_6Zr_2 97
65. BrLi 98
66. BrNa 99
67. $C_2Cd_2O_6$ 100
68. $C_2Cu_2O_6$ 101
69. $C_2N_2Na_2$ 102
70. $C_3Ag_3N_3$ 103
71. C_4Mg_2 104
72. $C_4O_{12}Sr_4$ 105
73. C_6Mg_4 106
74. CBe_2 107
75. CTa_2 108
76. CTi 109
77. $Ca_4Cr_4O_{12}$ 110
78. Ca_4Mg_8 111
79. CaI_2 112
80. CaO 113
81. $CdCl_2$ 114
82. $Cl_2O_2Ti_2$ 115
83. Cl_2Ti 116
84. Cl_2V 117
85. $Cl_4Li_4O_{16}$ 118
86. Cl_6Ni_3 119
87. Cl_6Rh_2 120
88. Cl_8Hf_2 121
89. Cl_8Sr_4 122
90. Co_2F_4 123
91. $Co_2O_8W_2$ 124
92. Co_4S_8 125
93. Co_4Sc_8 126
94. CoHf 127
95. $CoSi_2$ 128
96. Cr_2F_4 129
97. Cr_6Ga_2 130
98. Cr_6Si_2 131
99. Cs_4O_4 132
100. CsH 133
101. Cu_3Pt 134
102. Cu_4P_8 135
103. Cu_4Sb_2 136
104. Cu_4Sc_2 137
105. Cu_4Te_2 138
106. F_2Ti 139
107. F_2Xe 140
108. $F_4H_2K_2$ 141
109. F_4Ni_2 142
110. F_4Sn 143
111. F_8V_2 144
112. FLi 145
113. FNa 146
114. Fe_4N 147
115. FeO 148
116. FeTi 149
117. Ga_2Mo_6 150
118. Ga_4Y_4 151
119. GaRu 152
120. Ge_4Ir_4 153
121. Ge_4Pt_4 154
122. Ge_4Ru_4 155
123. $H_8O_{16}W_4$ 156
124. HK 157
125. HLi 158
126. He 159
127. $Hf_{10}Si_6$ 160
128. Hf_2I_6 161
129. Hf_4Ni_4 162
130. Hf_4Si_2 163
131. Hf_4Si_4 164
132. Hf_4Si_8 165
133. Hf_6Si_4 166
134. HfIr 167
135. HfOs 168
136. HfRh 169
137. Hg_3O_3 170
138. INa 171
139. In_3Pd_2 172
140. InP 173
141. InPd 174
142. $IrSn_2$ 175
143. IrY 176
144. K_2O 177
145. K_2O_2 178
146. $K_4O_6S_2$ 179
147. Kr_2 180
148. Li_2Te 181
149. $Li_4O_6Si_2$ 182
150. $Mg_2O_{10}Ti_4$ 183
151. $Mg_4O_{12}Se_4$ 184
152. MgTl 185
153. Mo_4S_6 186
154. Mo_6Si_2 187
155. $N_2Na_2O_6$ 188
156. NNb 189
157. Na_2O 190
158. Na_2Se 191
159. Na_2Te 192
160. Na_6O_6 193
161. Ne 194
162. Ni_3O_3 195
163. Ni_4Zr_4 196
164. NiPt 197
165. NiSc 198
166. $NiSi_2$ 199
167. O_2Pb_2 200
168. O_2Pd_2 201
169. O_2Rb 202
170. O_2Rb_2 203
171. O_2Sr 204
172. O_4Rb_4 205
173. OsTi 206
174. Pd_2Y_2 207
175. Pd_4S_8 208
176. PdSc 209
177. Pt_3Sc 210
178. Pt_3Y 211
179. Pt_4Si_4 212
180. Pt_4Y_4 213
181. PtS_2 214
182. Re_2Si_4 215
183. Rh_8Si_4 216
184. RhSc 217
185. RhY 218
186. Ru_4S_8 219
187. RuTi 220
188. RuZr 221
189. S_2Ta 222
190. S_2Zr 223
191. Se_2Sn 224
192. Se_4Tl_4 225
193. SeSr 226
194. Si_2Ta_4 227
195. Si_2Zr_4 228
196. Si_6Ta_3 229
197. Si_6Y_{10} 230
198. Te_2Zr 231
199. W 232
200. Zn_2 233

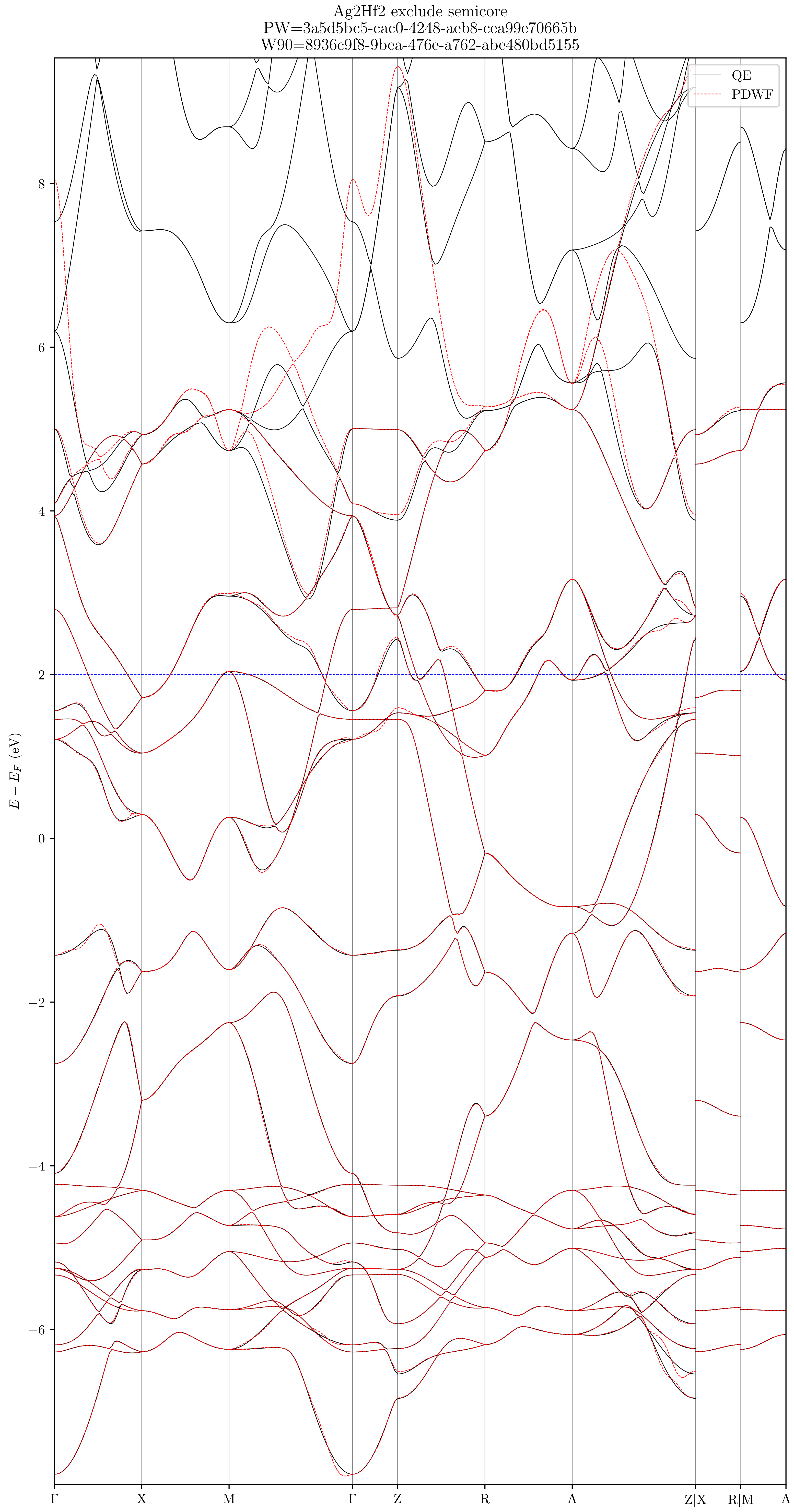

PDWF band distance (meV) and τ ($\AA$)

η_0	η_2	η_4	$\eta_0^{\max}$	$\eta_2^{\max}$	$\eta_4^{\max}$	τ
8.2	8.8	11.9	106.0	106.0	107.6	2.572

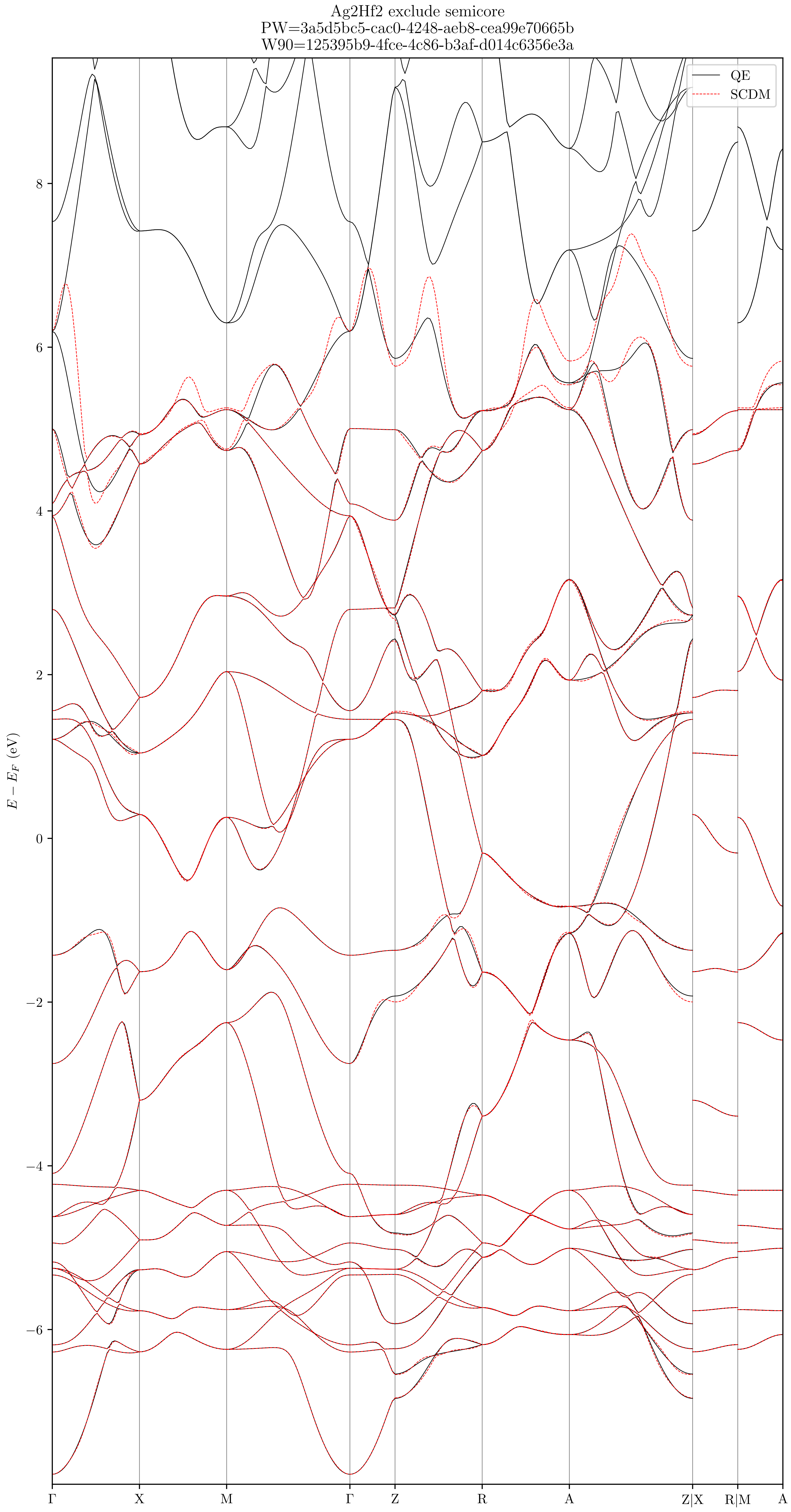

SCDM band distance (meV) and τ ($\AA$)

η_0	η_2	η_4	$\eta_0^{\max}$	$\eta_2^{\max}$	$\eta_4^{\max}$	τ
8.4	8.4	9.1	72.6	72.6	72.6	2.522

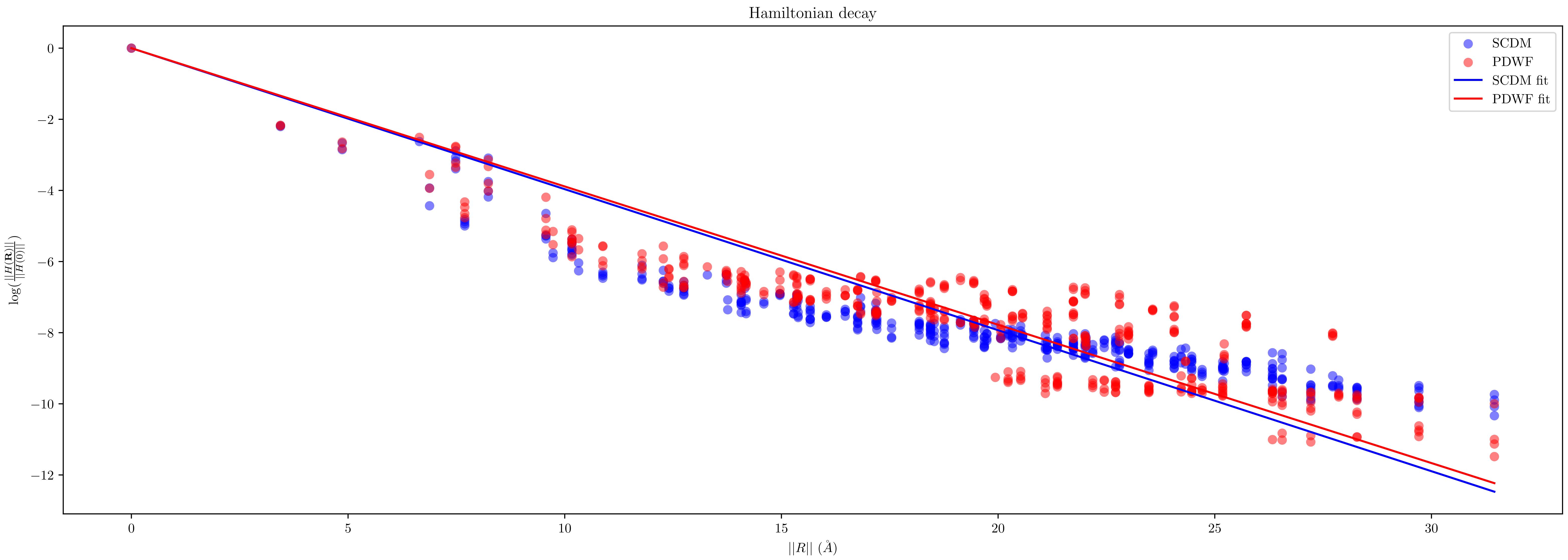

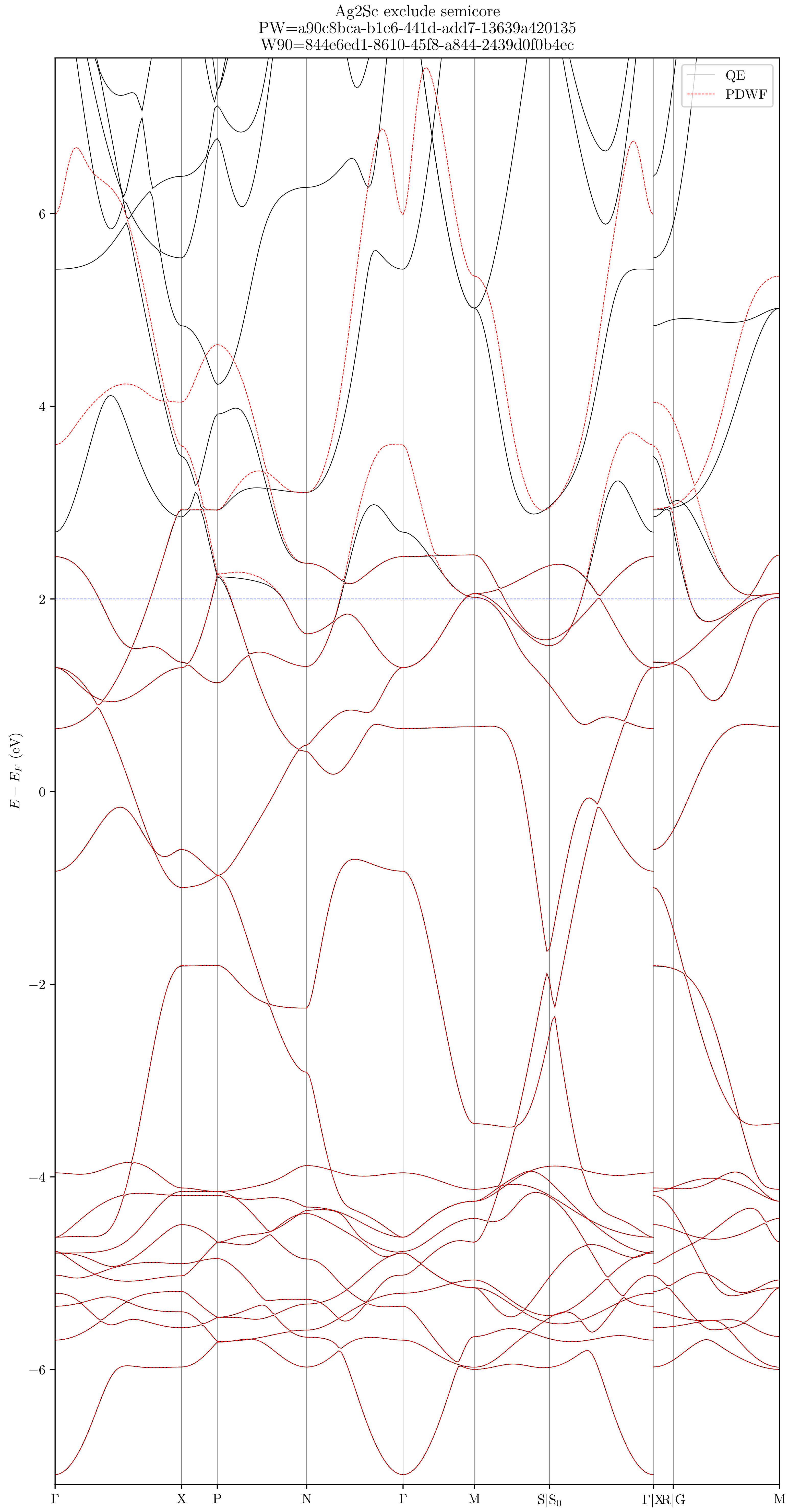

PDWF band distance (meV) and τ ($\AA$)

η_0	η_2	η_4	$\eta_0^{\max}$	$\eta_2^{\max}$	$\eta_4^{\max}$	τ
0.6	1.7	104.0	5.2	11.5	897.3	2.253

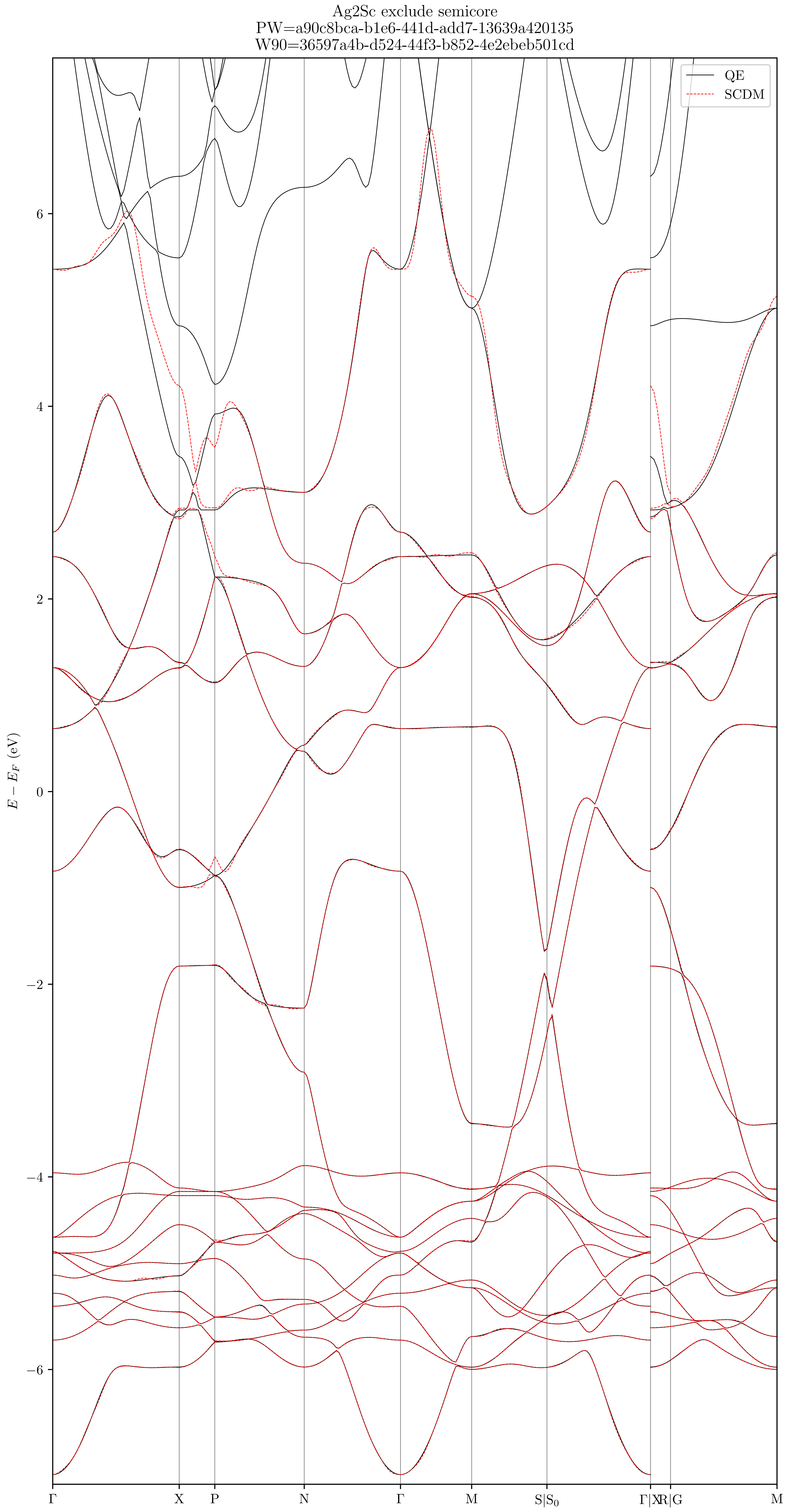

SCDM band distance (meV) and τ ($\AA$)

η_0	η_2	η_4	$\eta_0^{\max}$	$\eta_2^{\max}$	$\eta_4^{\max}$	τ
6.1	6.3	26.8	194.6	194.8	465.3	2.865

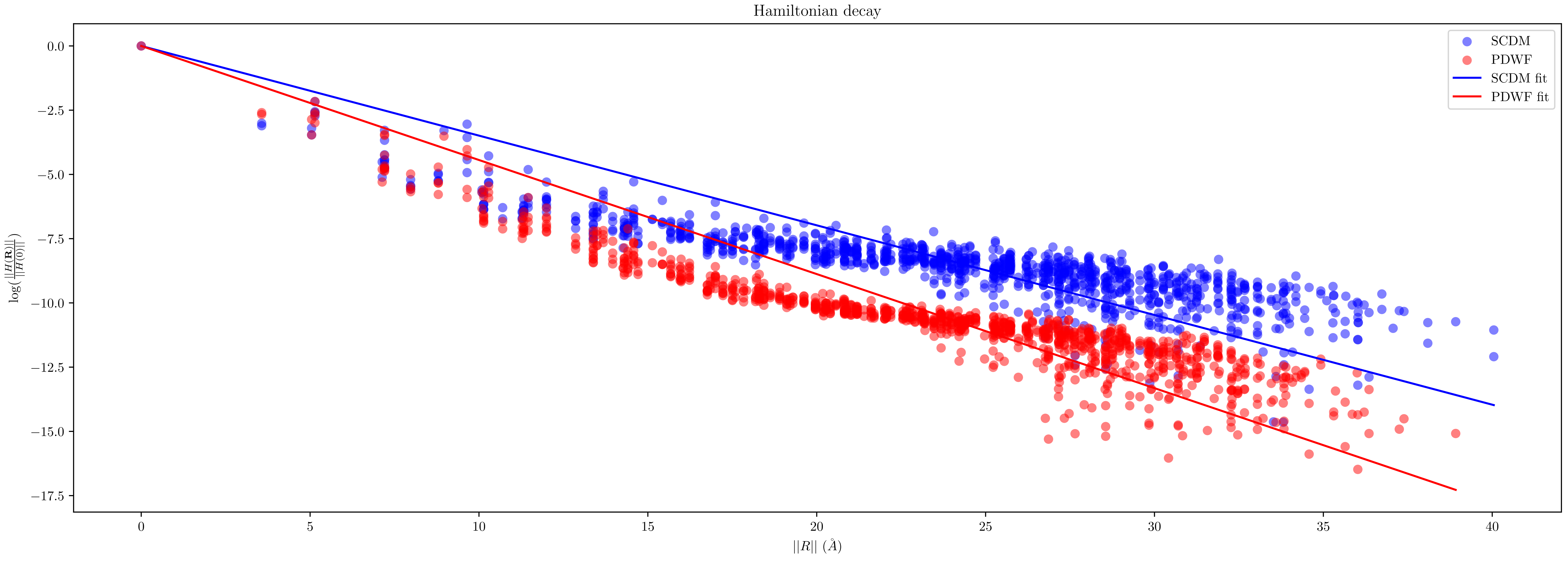

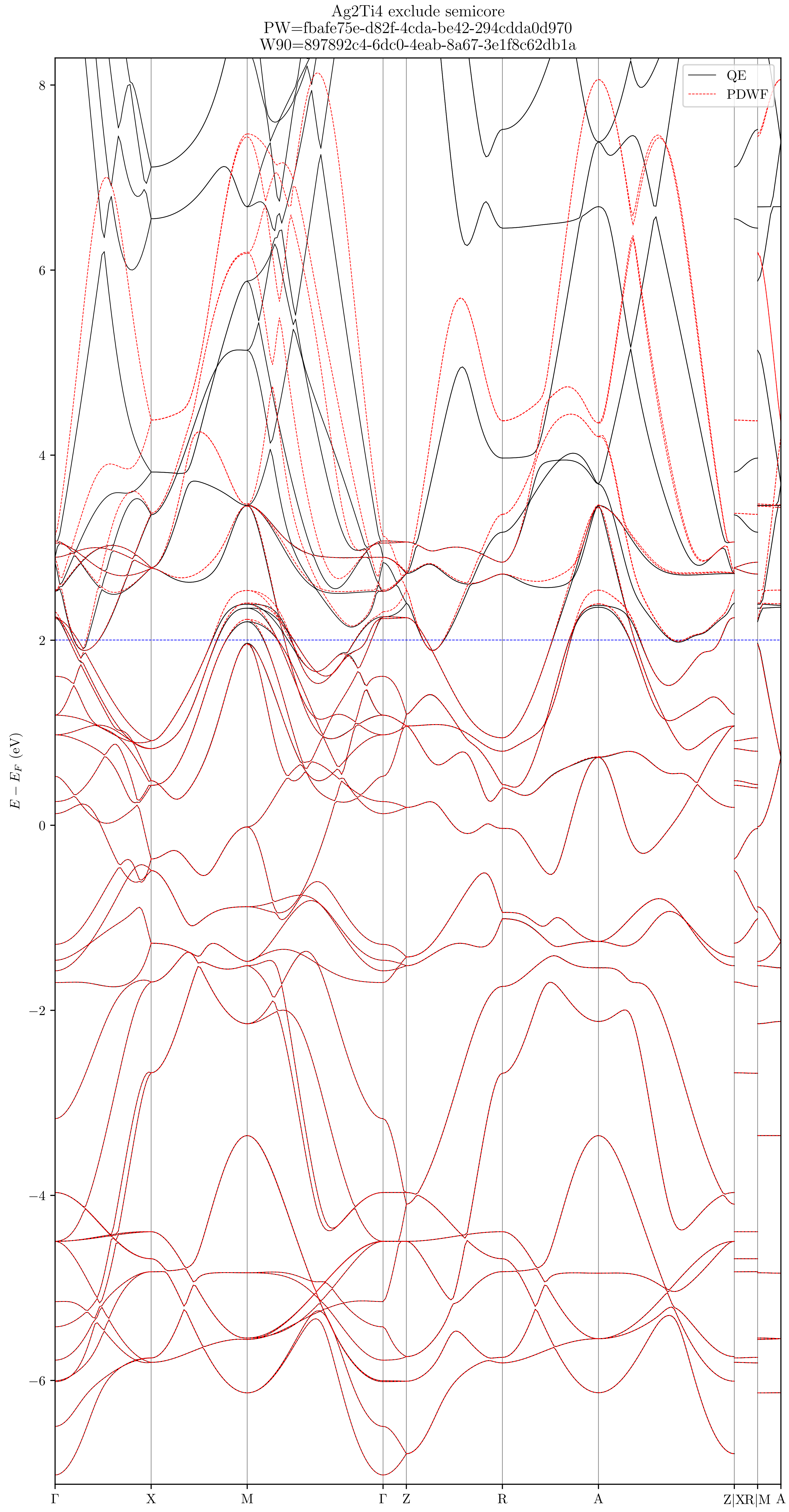

PDWF band distance (meV) and τ ($\AA$)

η_0	η_2	η_4	$\eta_0^{\max}$	$\eta_2^{\max}$	$\eta_4^{\max}$	τ
0.6	3.3	61.1	3.7	23.3	510.1	2.247

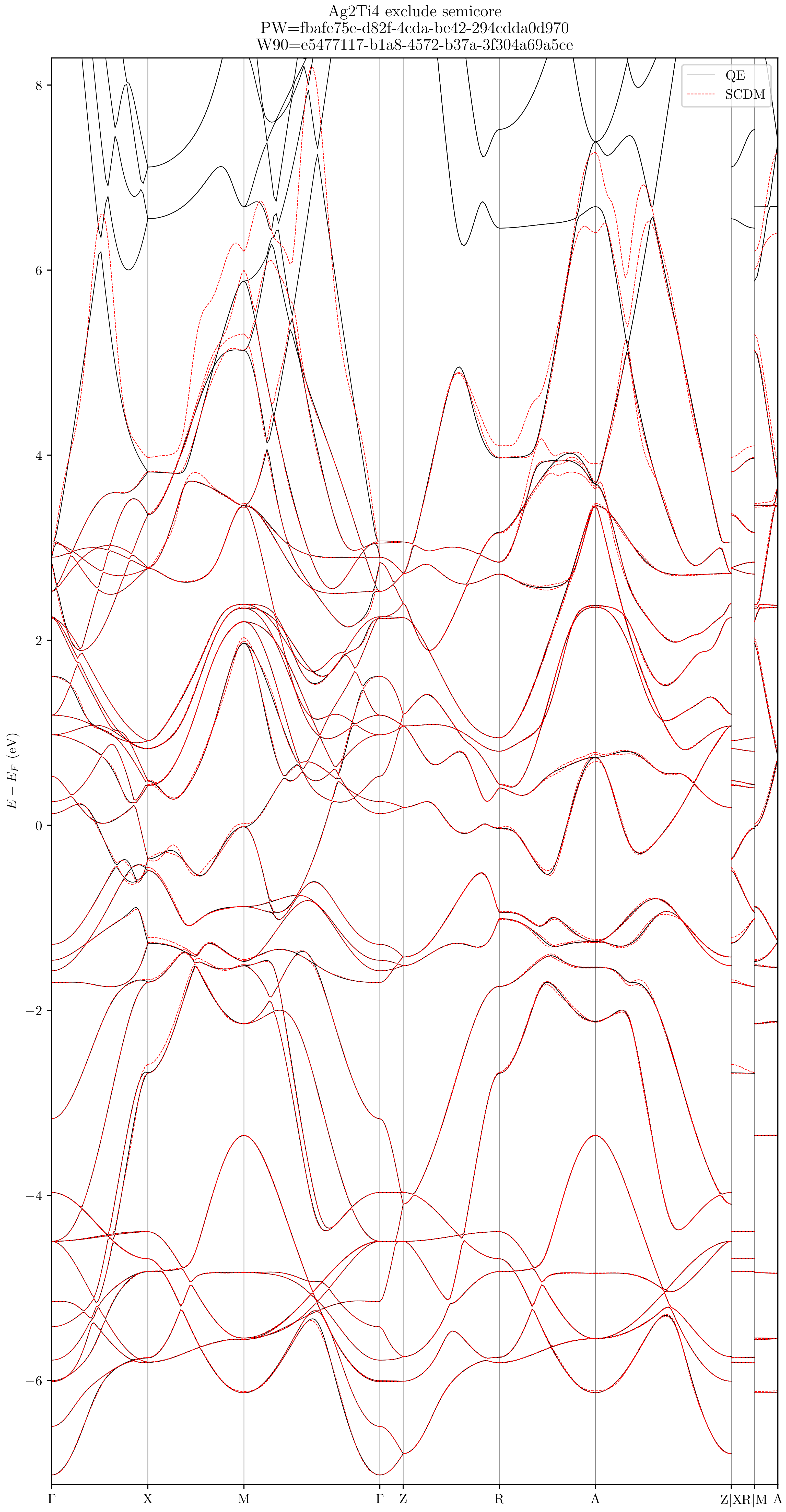

SCDM band distance (meV) and τ ($\AA$)

η_0	η_2	η_4	$\eta_0^{\max}$	$\eta_2^{\max}$	$\eta_4^{\max}$	τ
10.4	10.4	16.7	91.6	91.6	185.0	2.802

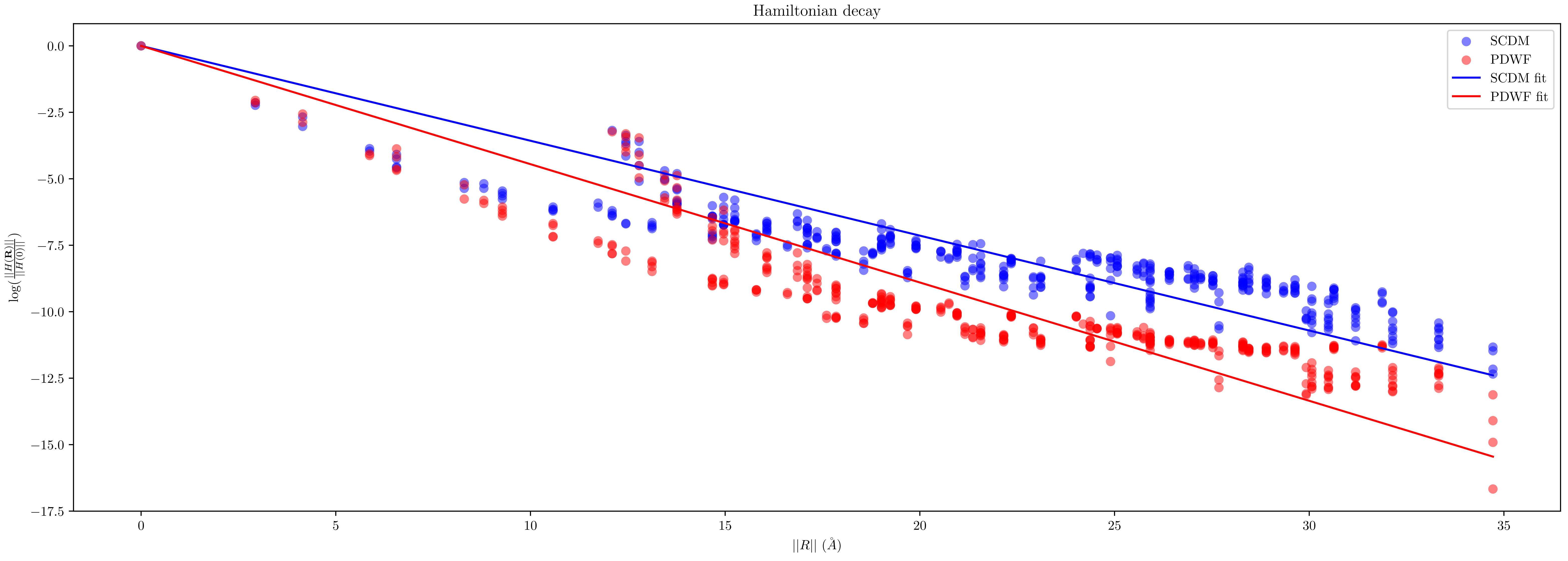

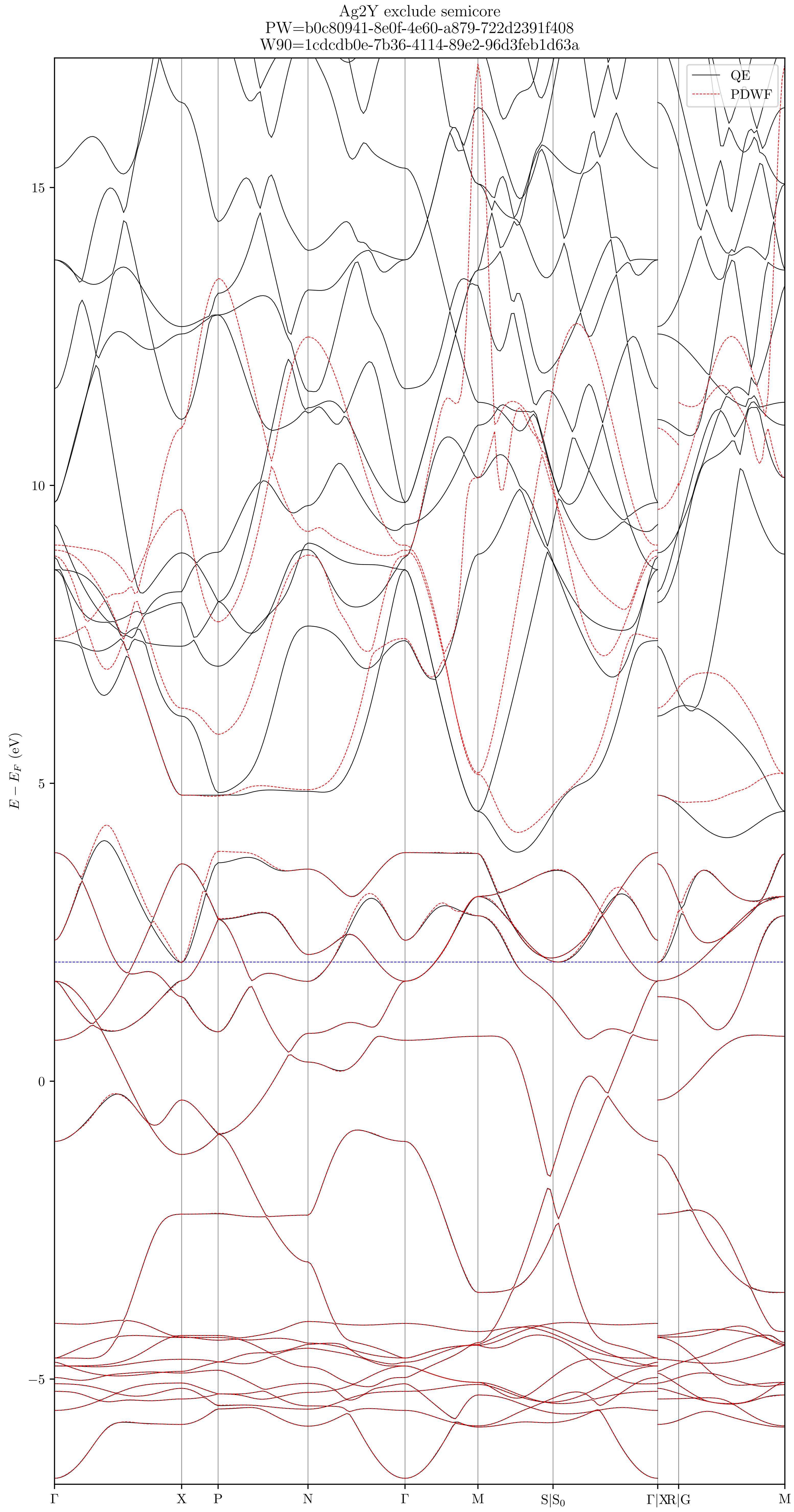

PDWF band distance (meV) and τ ($\AA$)

η_0	η_2	η_4	$\eta_0^{\max}$	$\eta_2^{\max}$	$\eta_4^{\max}$	τ
2.3	3.0	23.0	32.5	35.0	237.6	2.687

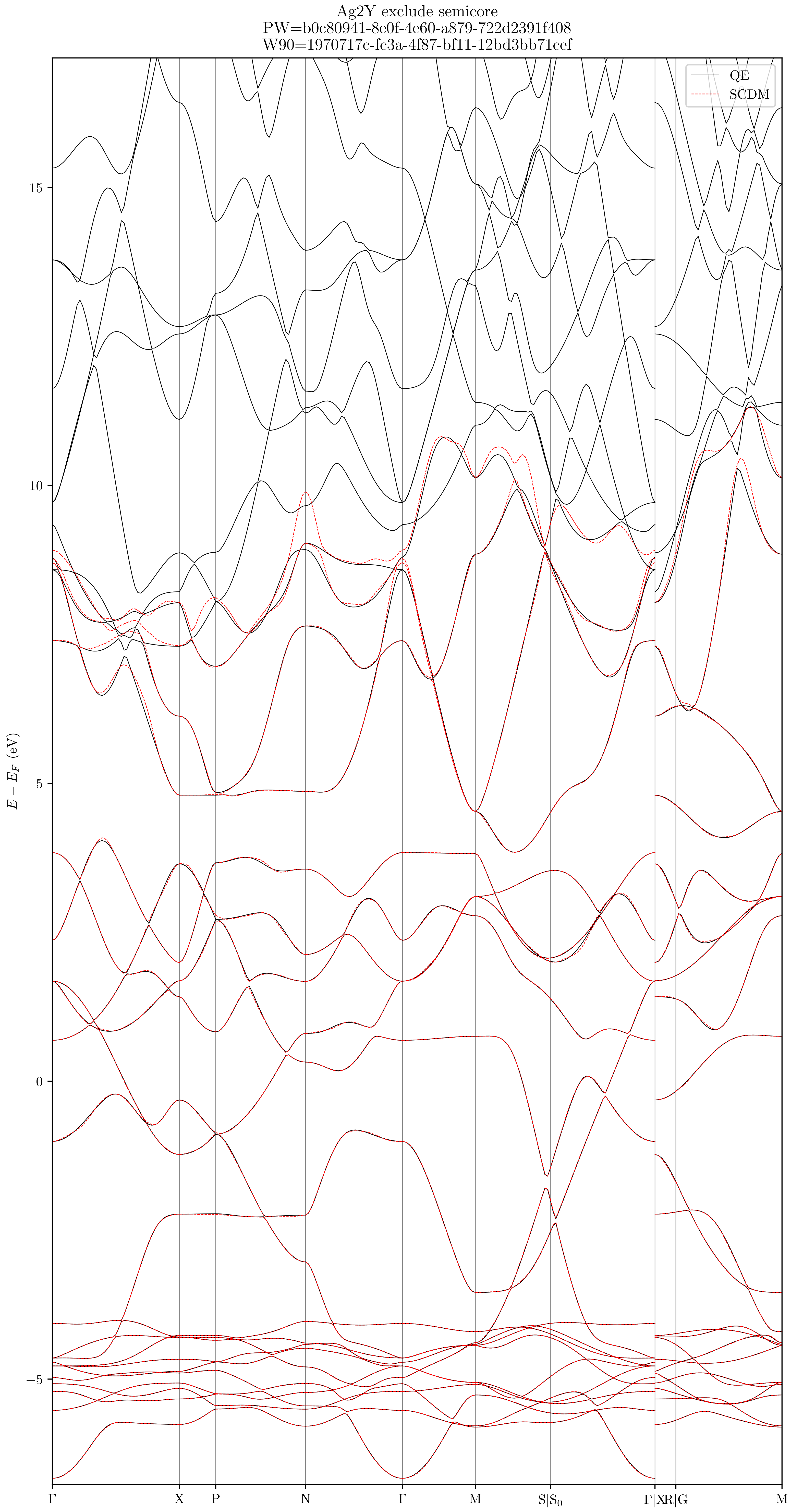

SCDM band distance (meV) and τ ($\AA$)

η_0	η_2	η_4	$\eta_0^{\max}$	$\eta_2^{\max}$	$\eta_4^{\max}$	τ
3.6	4.4	6.2	32.2	45.1	67.5	3.061

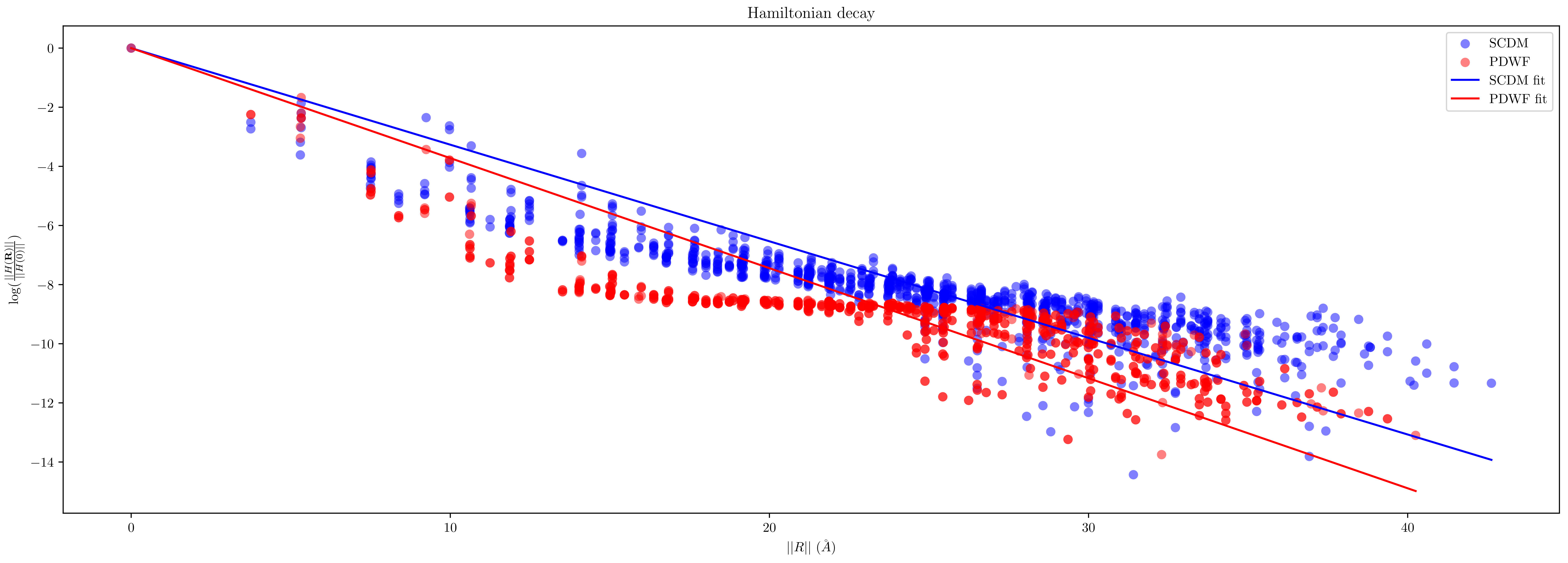

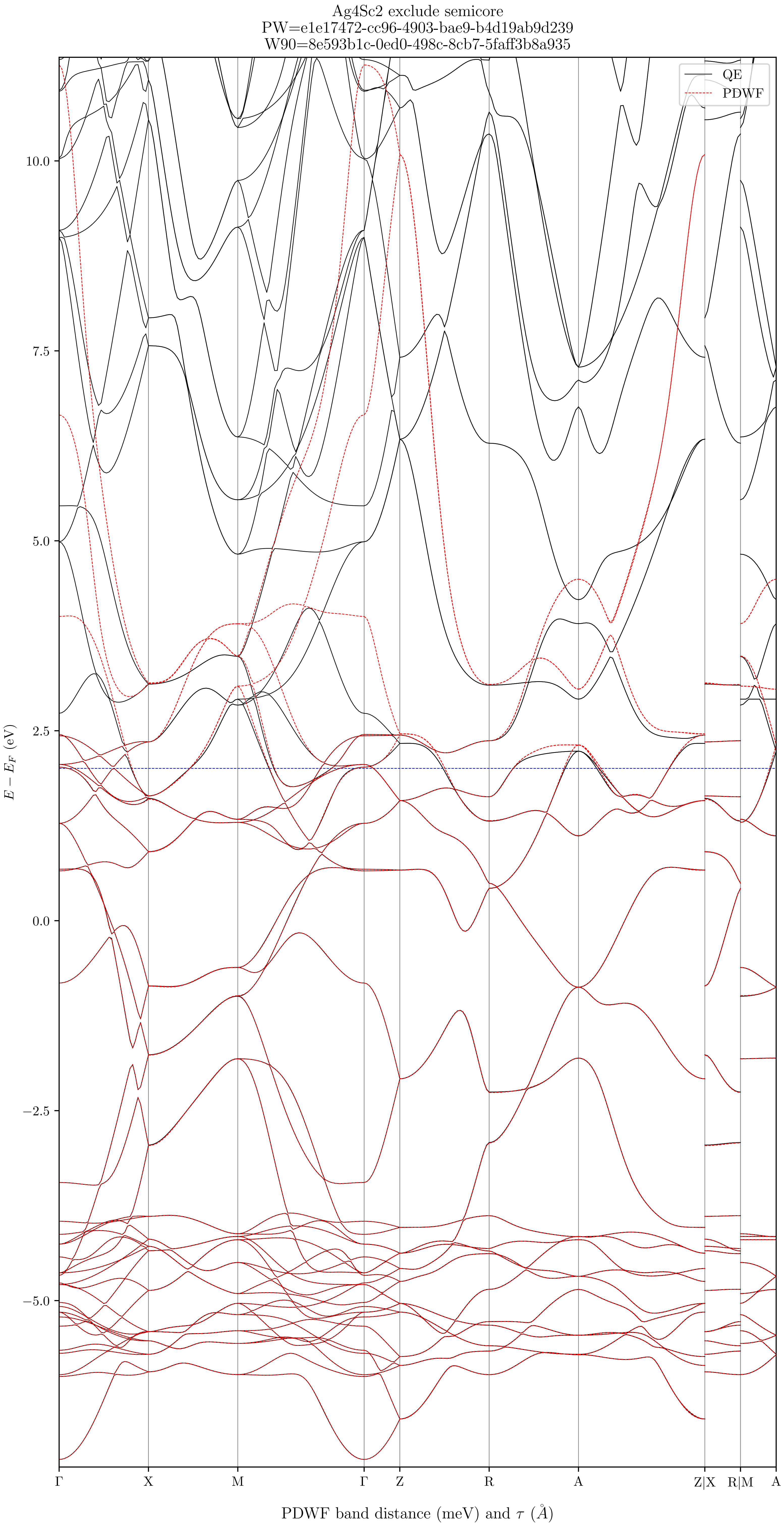

η_0	η_2	η_4	$\eta_0^{\max}$	$\eta_2^{\max}$	$\eta_4^{\max}$	τ
1.4	3.8	96.3	13.1	24.9	1020.0	1.839

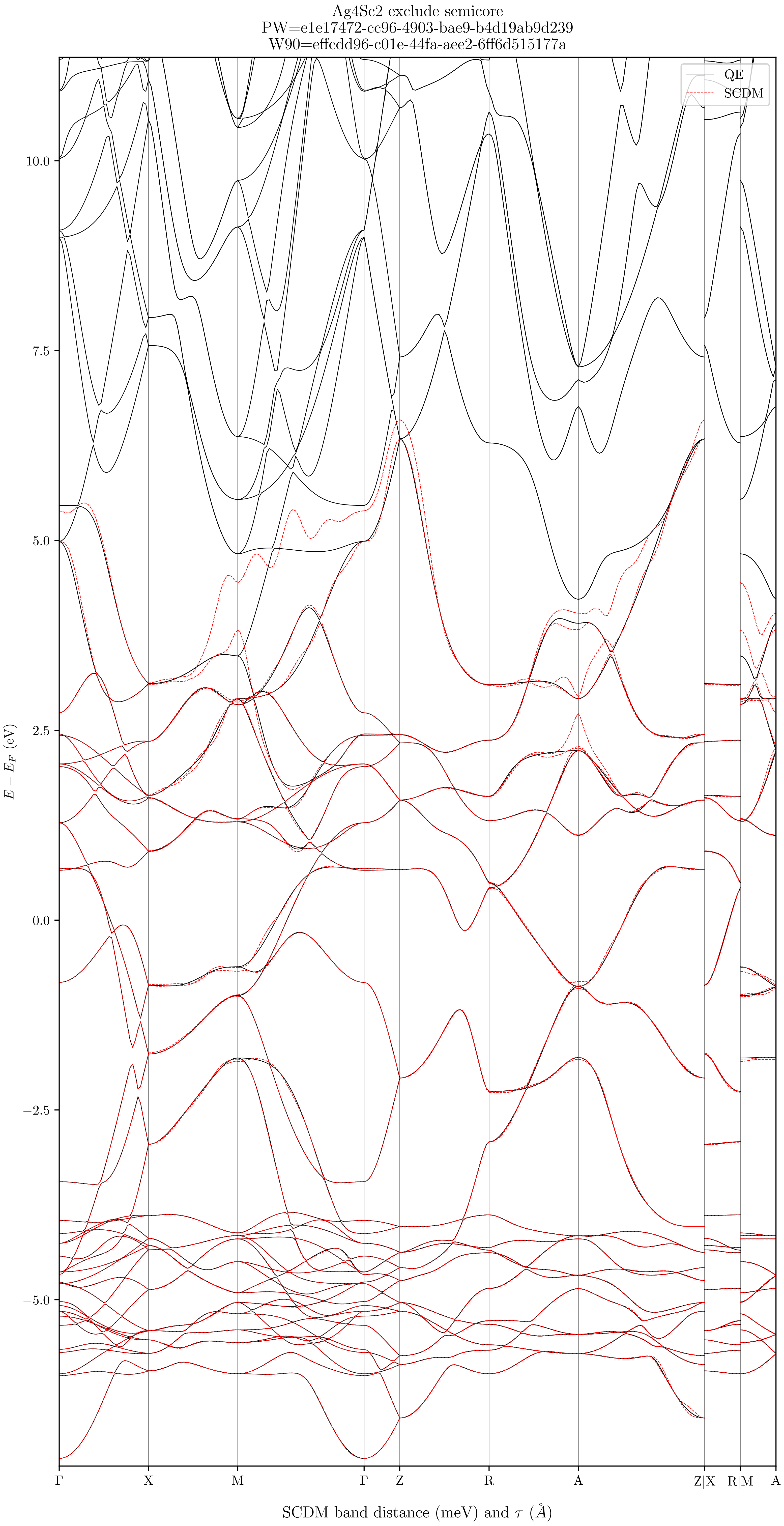

η_0	η_2	η_4	$\eta_0^{\max}$	$\eta_2^{\max}$	$\eta_4^{\max}$	τ
6.6	7.8	32.9	71.9	79.5	596.9	2.559

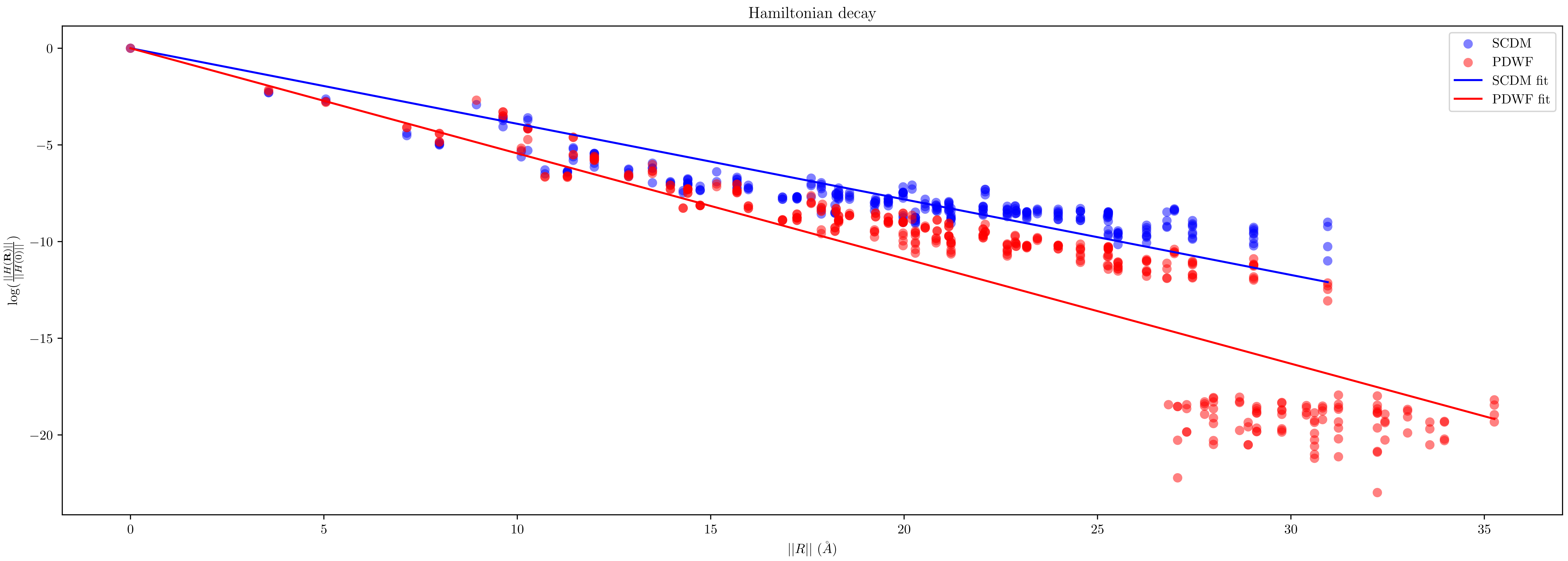

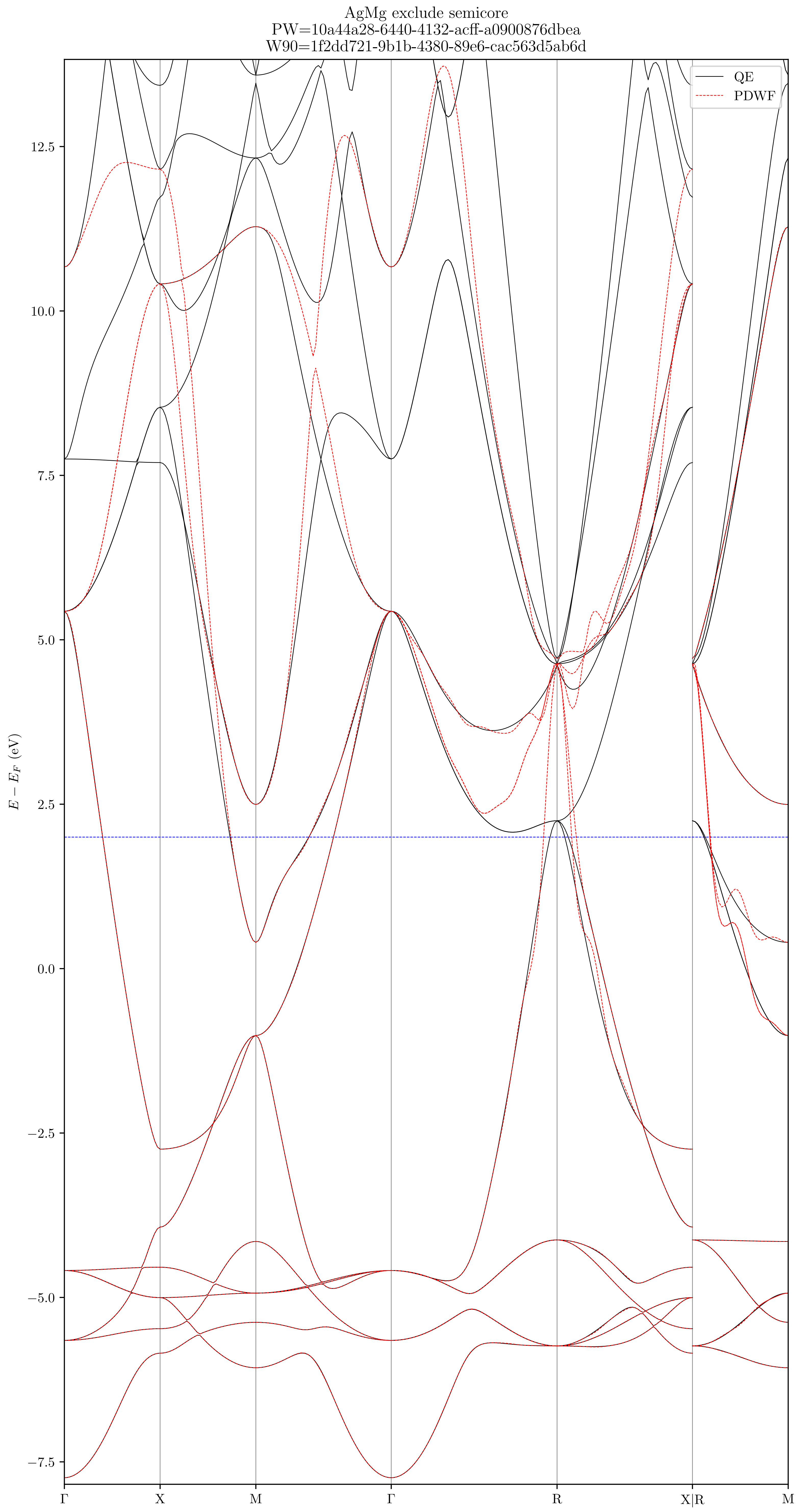

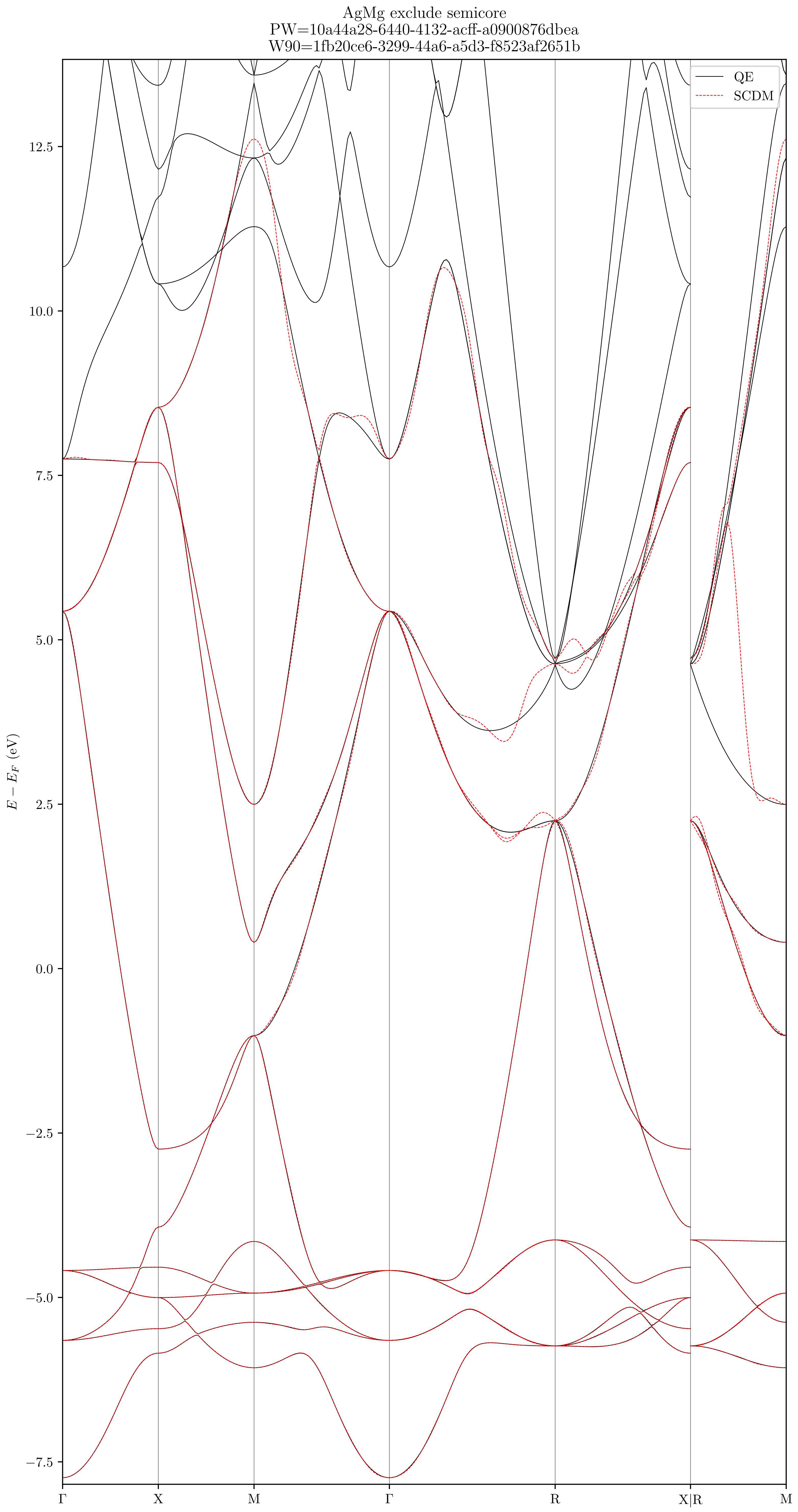

PDWF band distance (meV) and τ (Å)

η_0	η_2	η_4	$\eta_0^{\max}$	$\eta_2^{\max}$	$\eta_4^{\max}$	τ
18.3	54.7	226.5	299.4	501.7	1635.0	2.114

SCDM band distance (meV) and τ (Å)

η_0	η_2	η_4	$\eta_0^{\max}$	$\eta_2^{\max}$	$\eta_4^{\max}$	τ
6.5	13.1	37.7	70.9	107.9	798.2	2.504

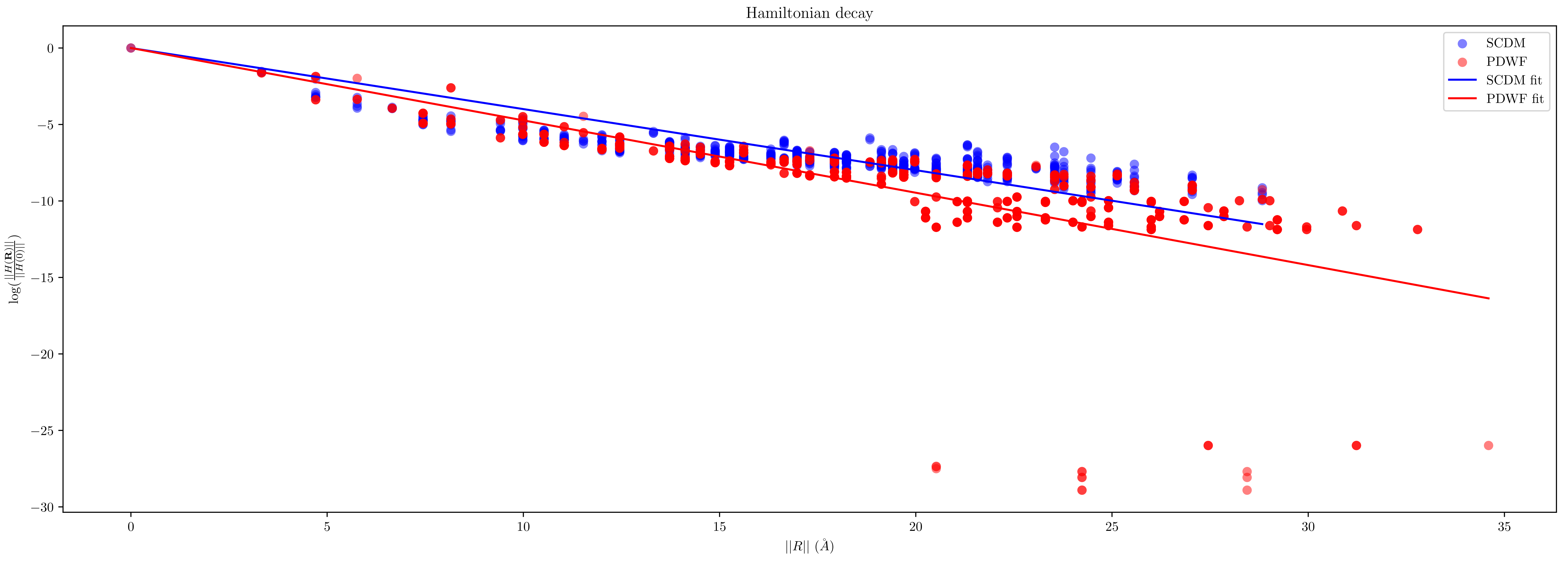

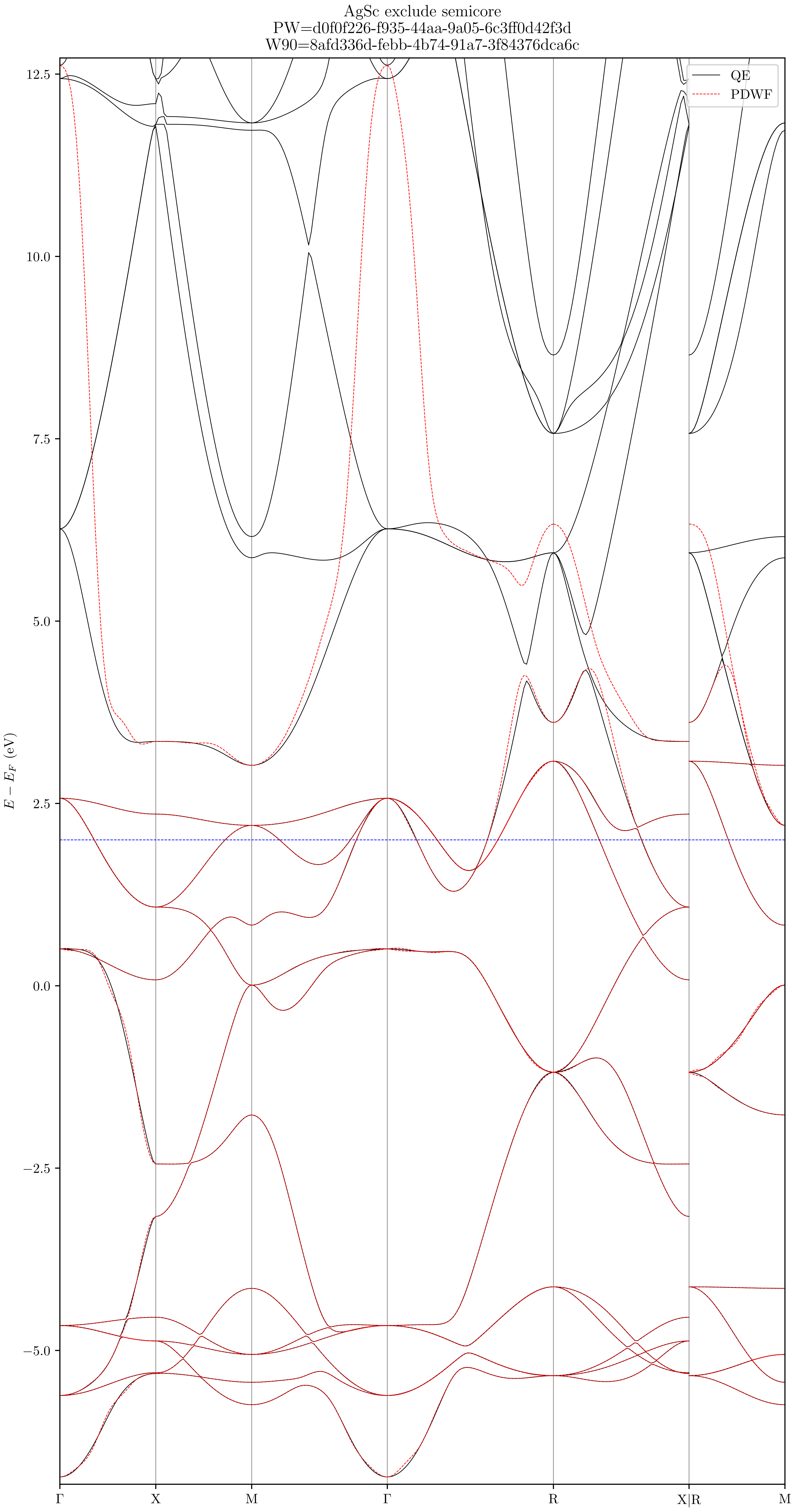

PDWF band distance (meV) and τ ($\AA$)

η_0	η_2	η_4	$\eta_0^{\max}$	$\eta_2^{\max}$	$\eta_4^{\max}$	τ
9.6	9.0	32.3	119.2	119.2	332.7	2.266

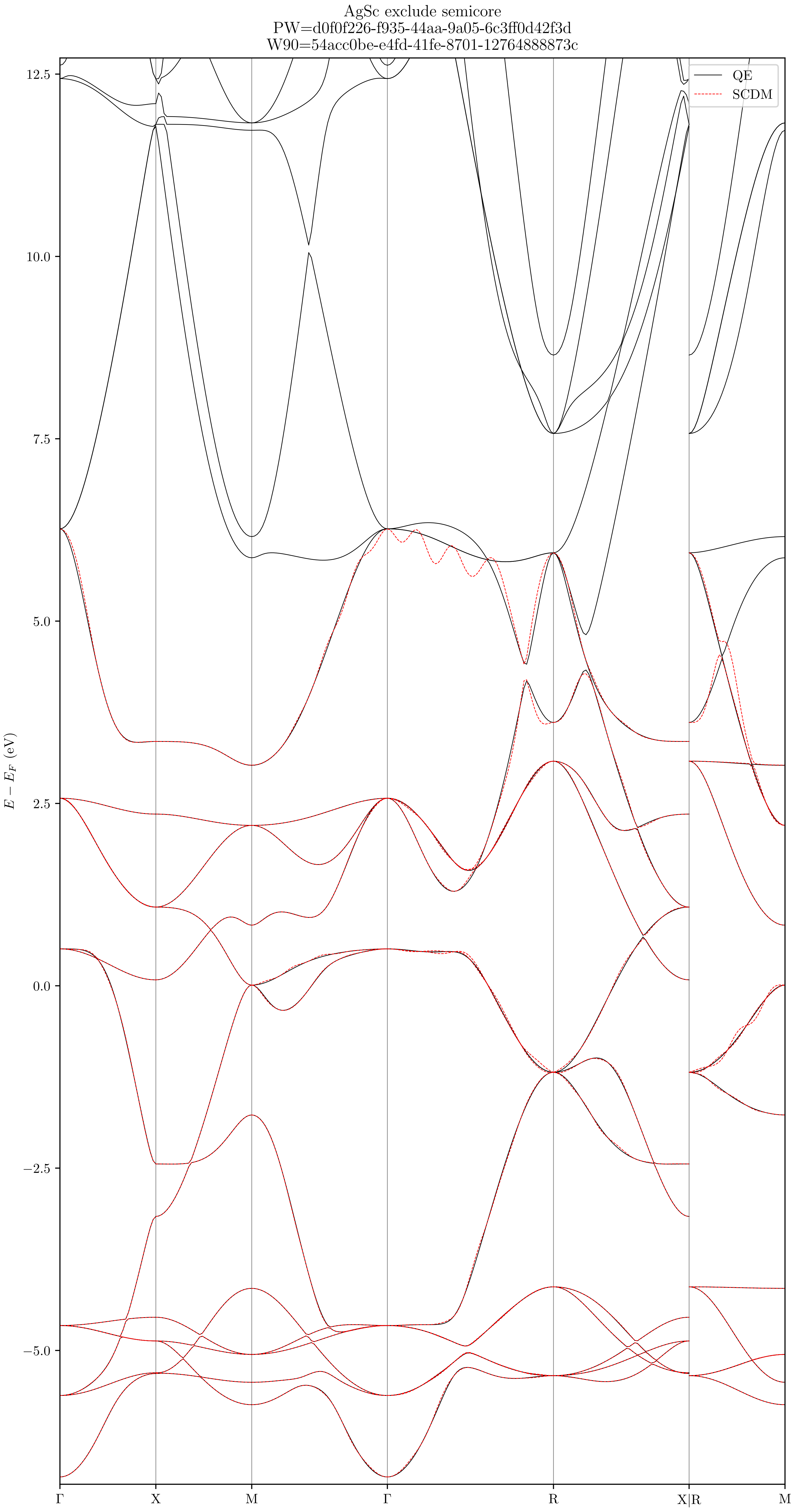

SCDM band distance (meV) and τ ($\AA$)

η_0	η_2	η_4	$\eta_0^{\max}$	$\eta_2^{\max}$	$\eta_4^{\max}$	τ
9.4	9.4	15.5	85.4	85.5	240.6	2.401

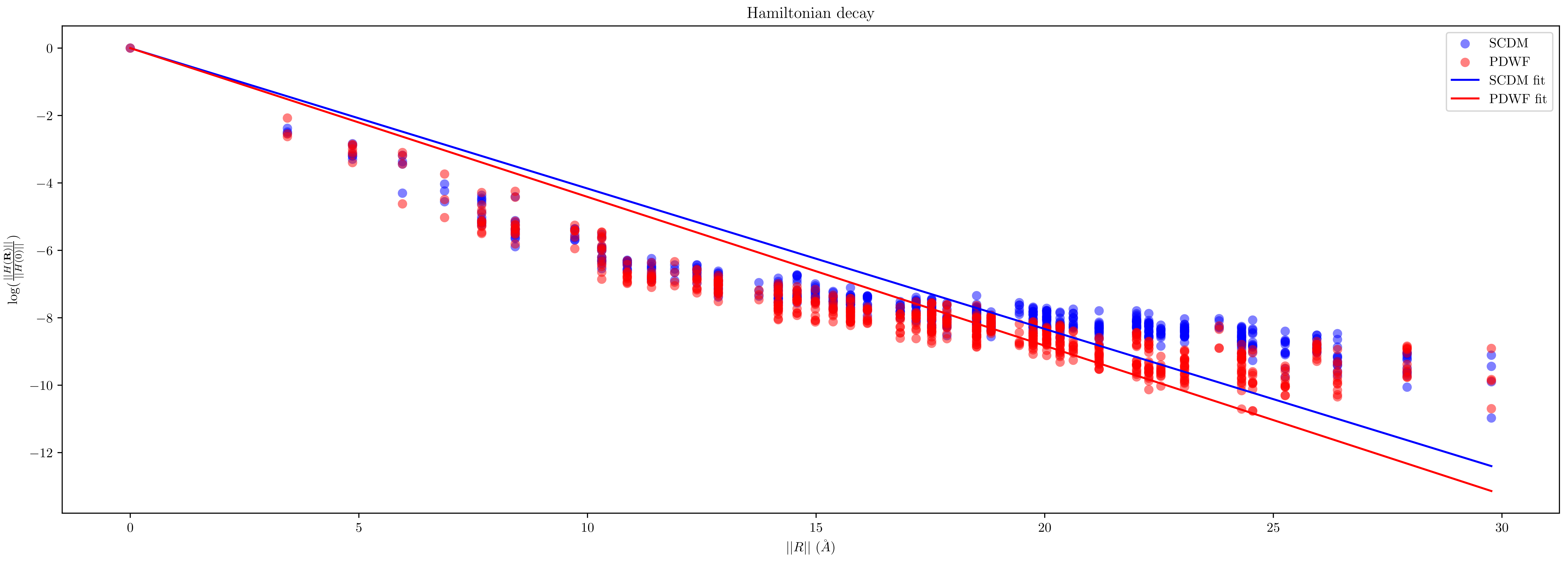

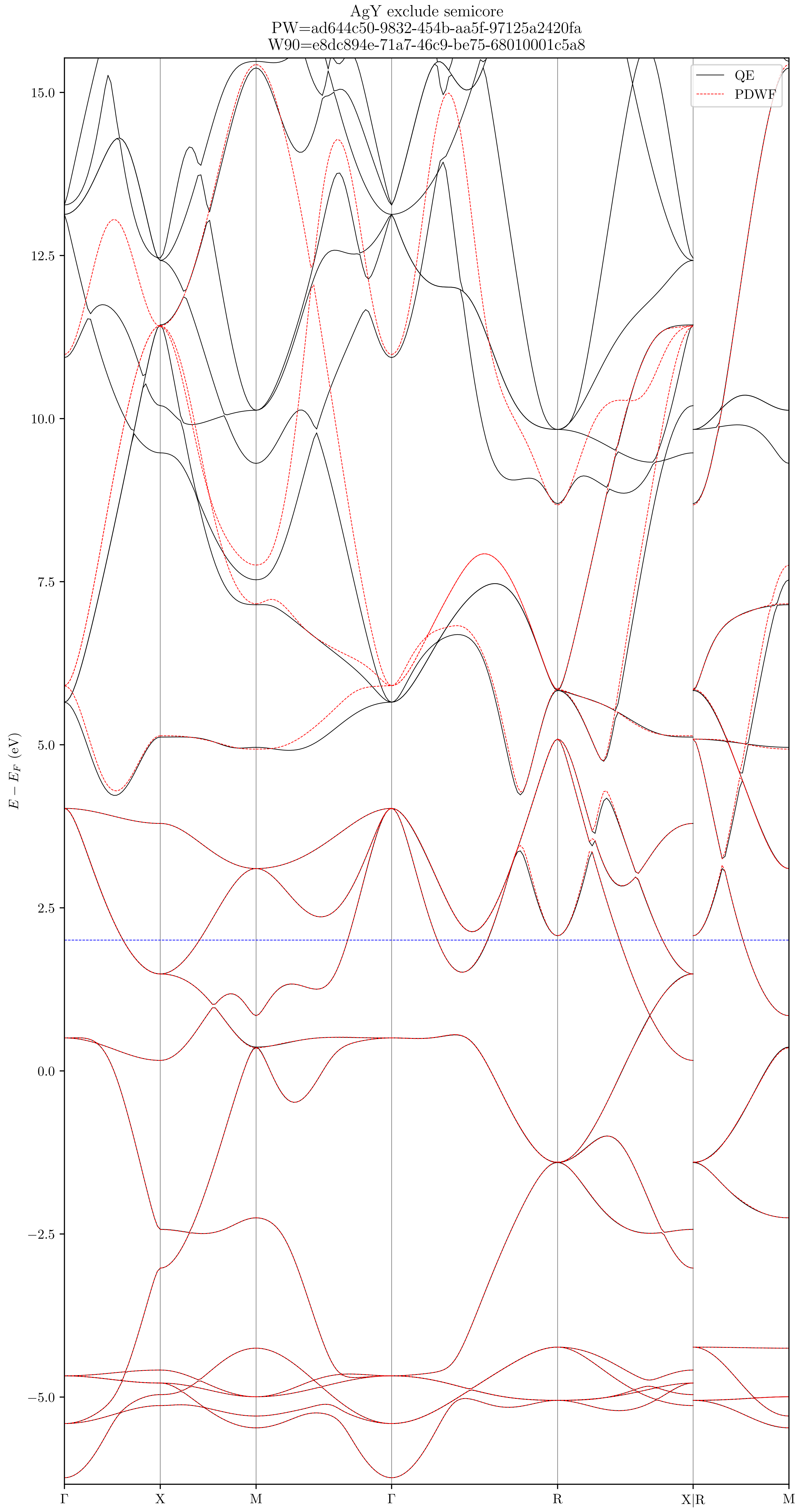

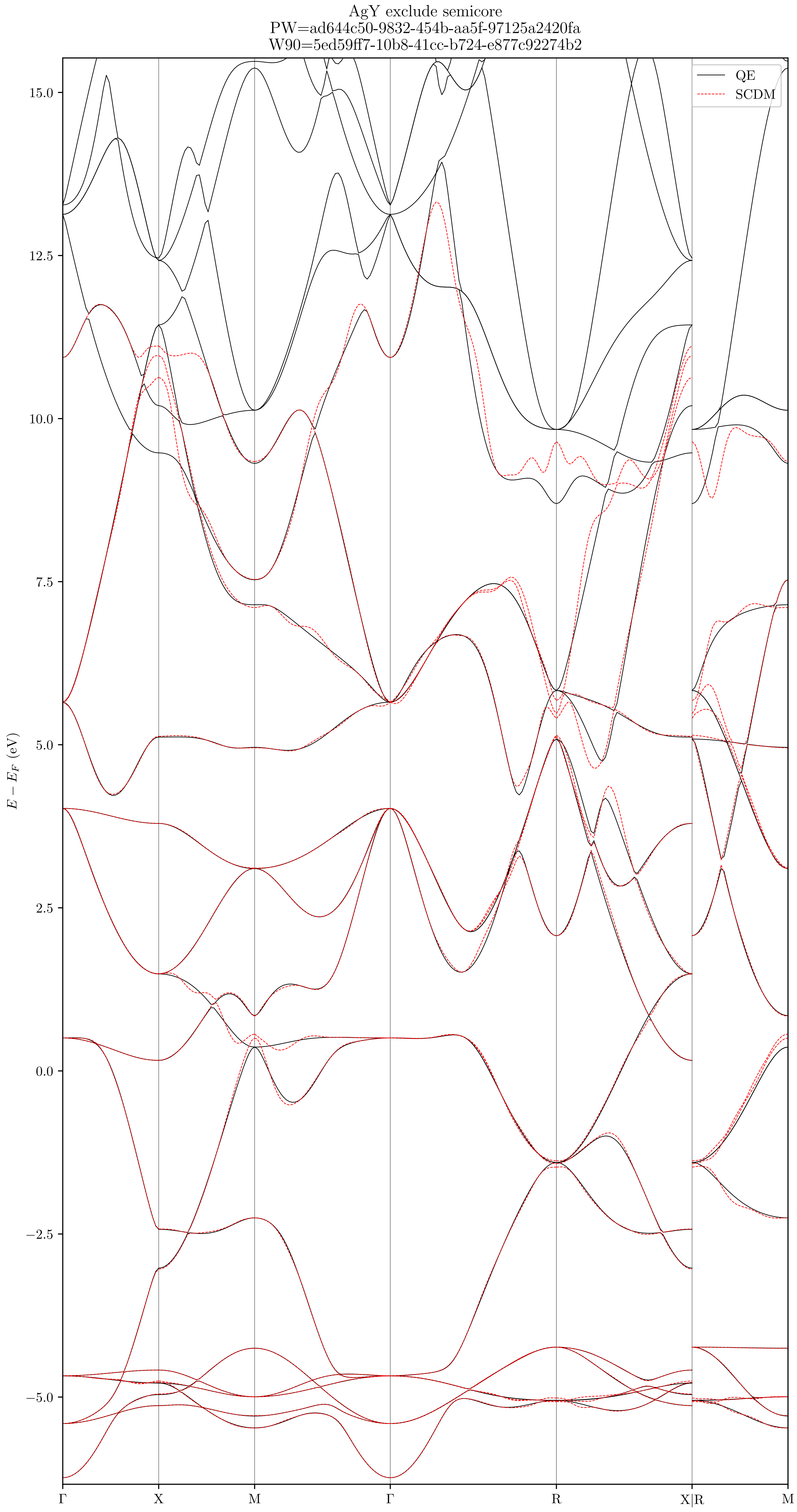

PDWF band distance (meV) and τ ($\AA$)

η_0	η_2	η_4	$\eta_0^{\max}$	$\eta_2^{\max}$	$\eta_4^{\max}$	τ
1.9	2.4	8.4	11.6	13.5	114.8	2.024

SCDM band distance (meV) and τ ($\AA$)

η_0	η_2	η_4	$\eta_0^{\max}$	$\eta_2^{\max}$	$\eta_4^{\max}$	τ
19.0	27.3	29.5	127.7	199.6	201.4	2.568

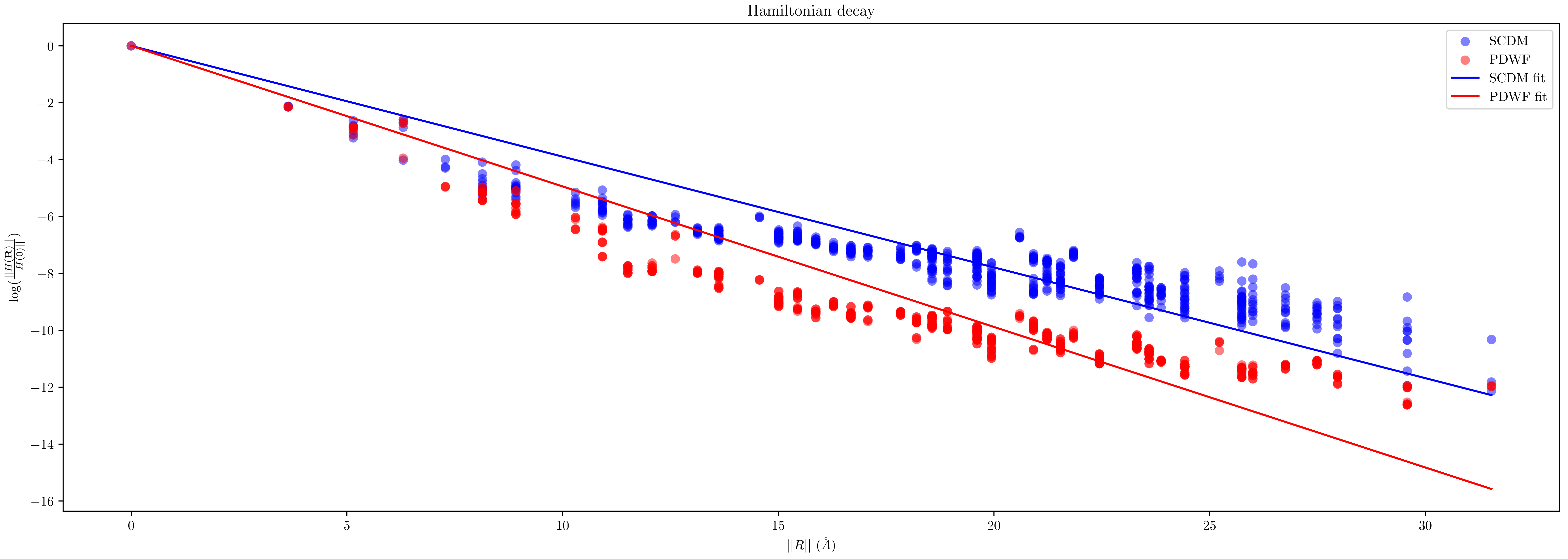

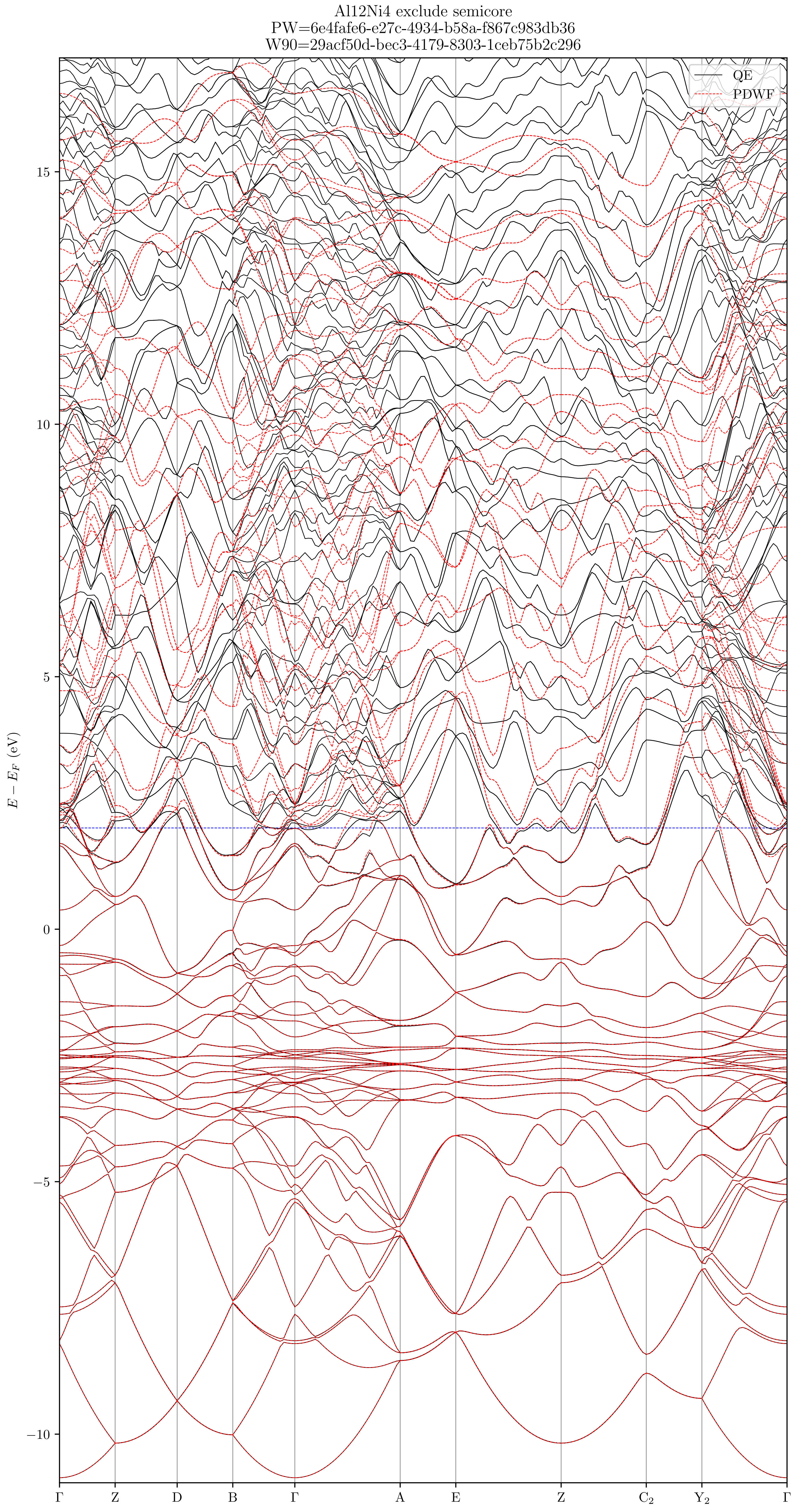

PDWF band distance (meV) and τ ($\AA$)

η_0	η_2	η_4	$\eta_0^{\max}$	$\eta_2^{\max}$	$\eta_4^{\max}$	τ
2.4	9.4	107.3	25.5	156.0	585.9	2.710

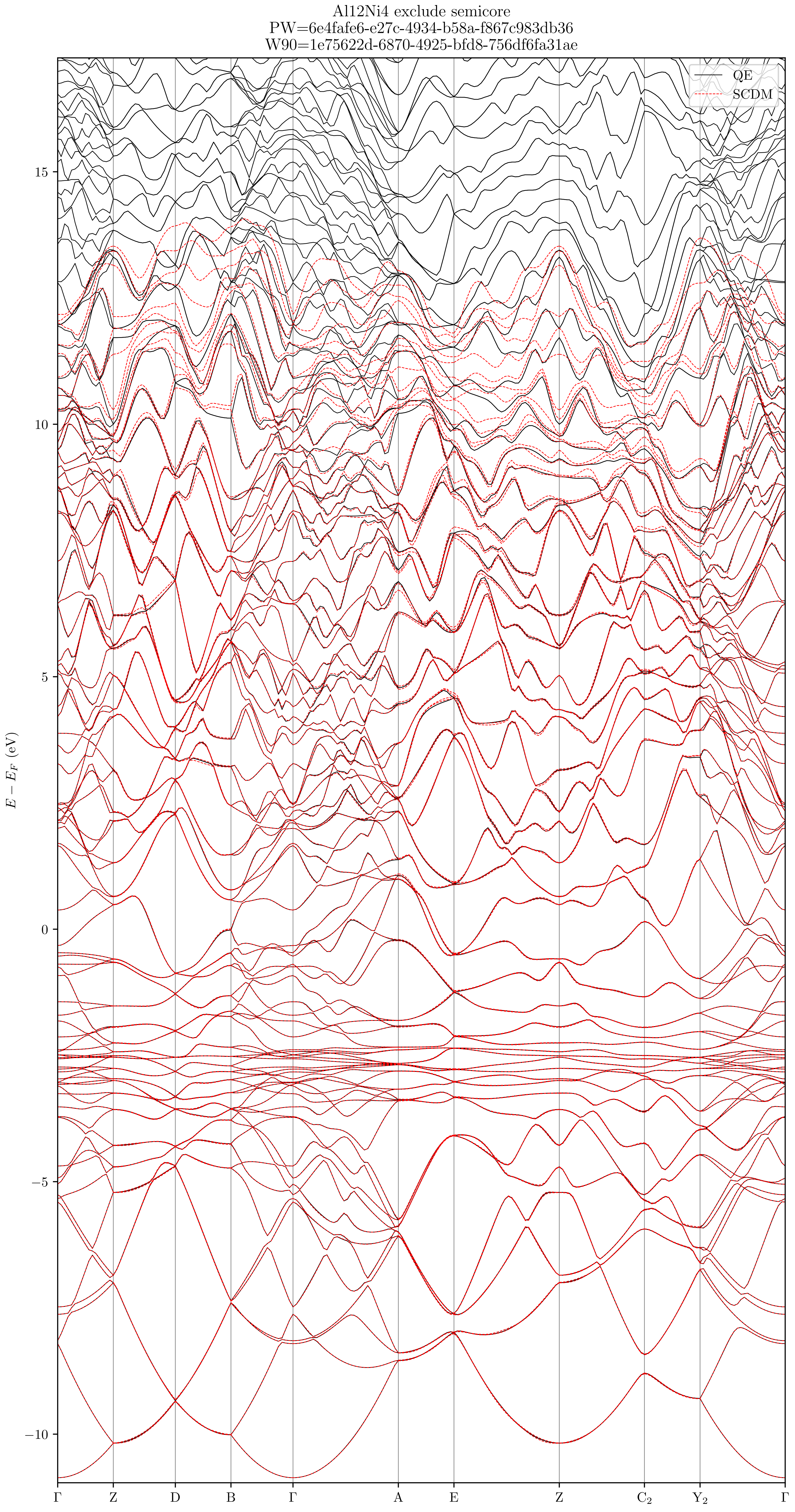

SCDM band distance (meV) and τ ($\AA$)

η_0	η_2	η_4	$\eta_0^{\max}$	$\eta_2^{\max}$	$\eta_4^{\max}$	τ
5.3	6.1	7.3	35.2	39.0	44.3	3.313

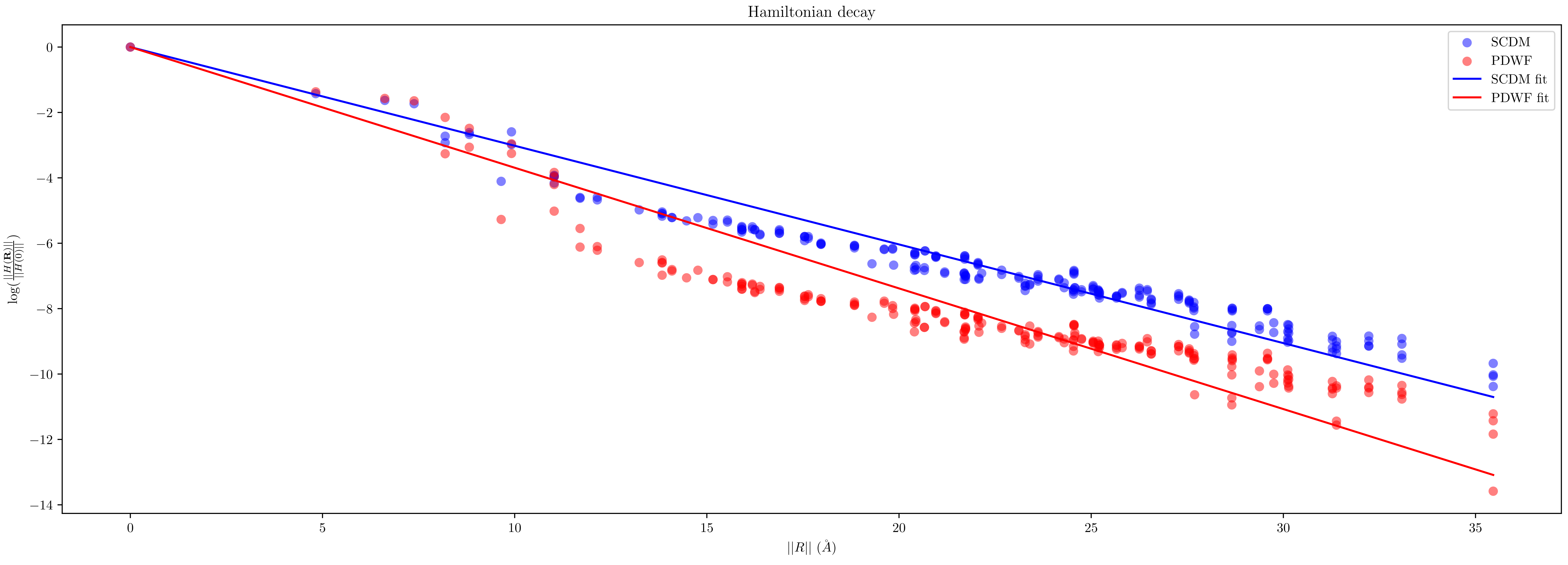

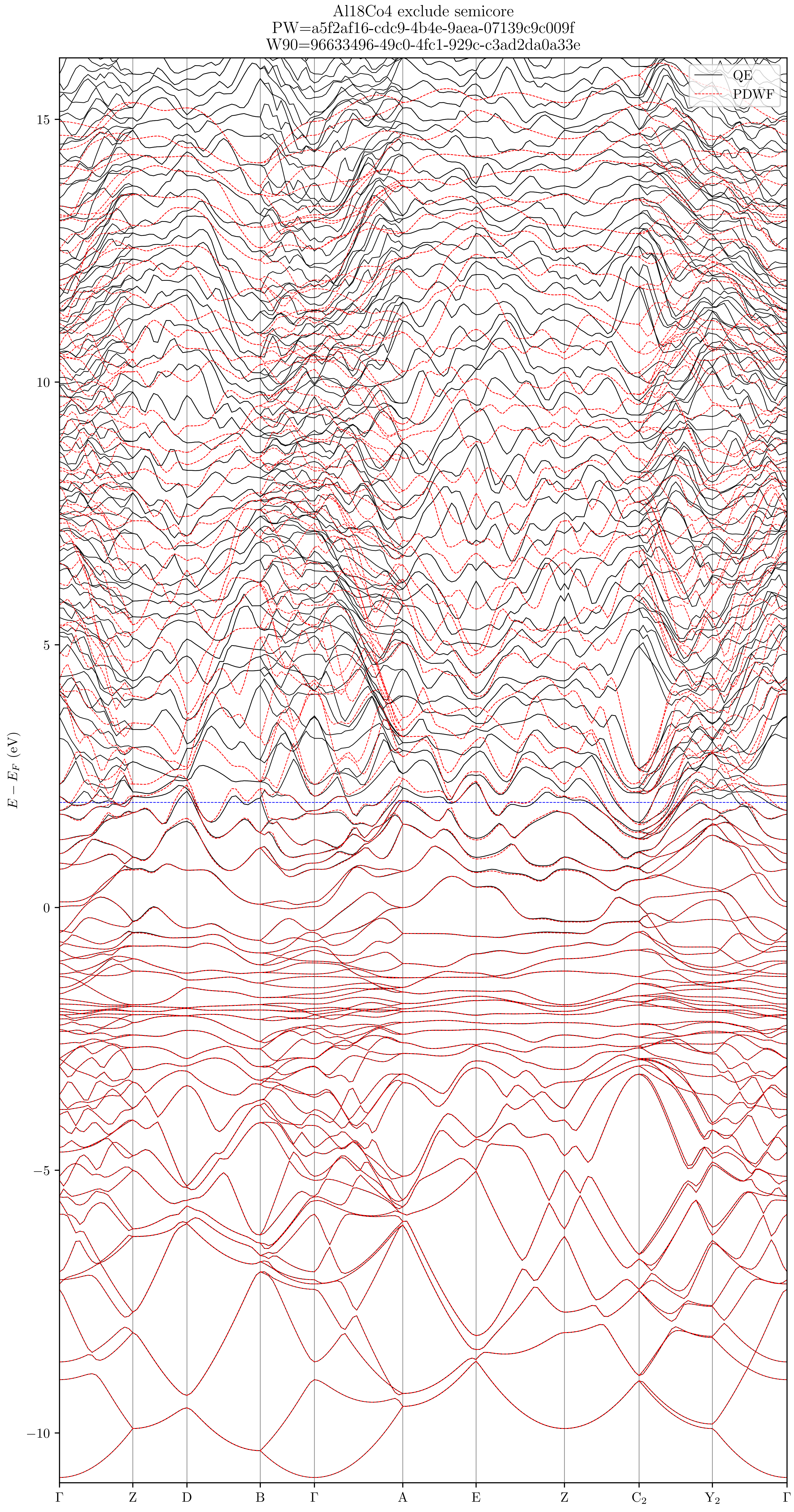

PDWF band distance (meV) and τ ($\AA$)

η_0	η_2	η_4	$\eta_0^{\max}$	$\eta_2^{\max}$	$\eta_4^{\max}$	τ
2.1	9.9	122.5	15.7	96.3	686.7	2.751

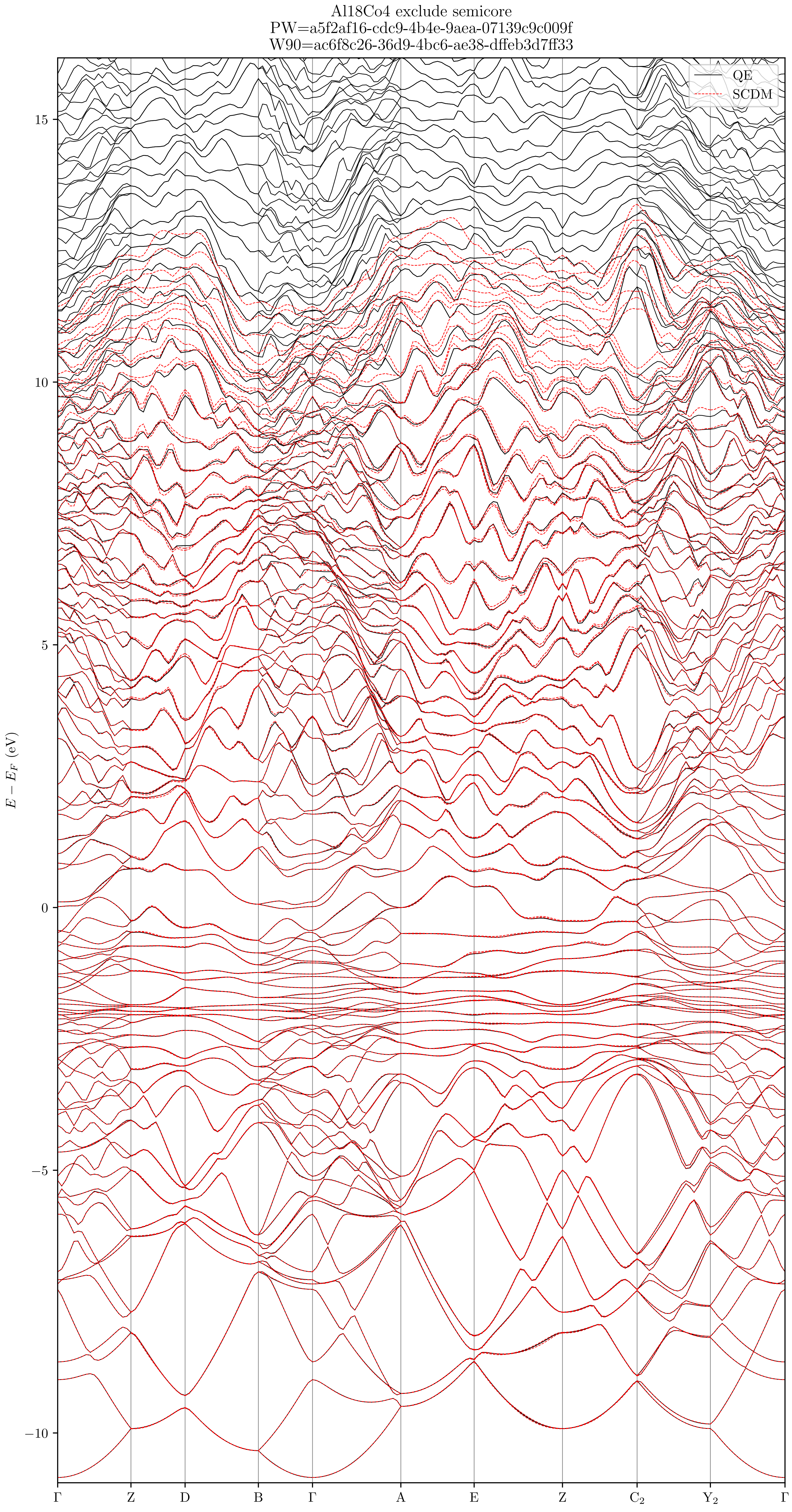

SCDM band distance (meV) and τ ($\AA$)

η_0	η_2	η_4	$\eta_0^{\max}$	$\eta_2^{\max}$	$\eta_4^{\max}$	τ
5.2	5.9	7.1	34.3	34.3	50.8	3.399

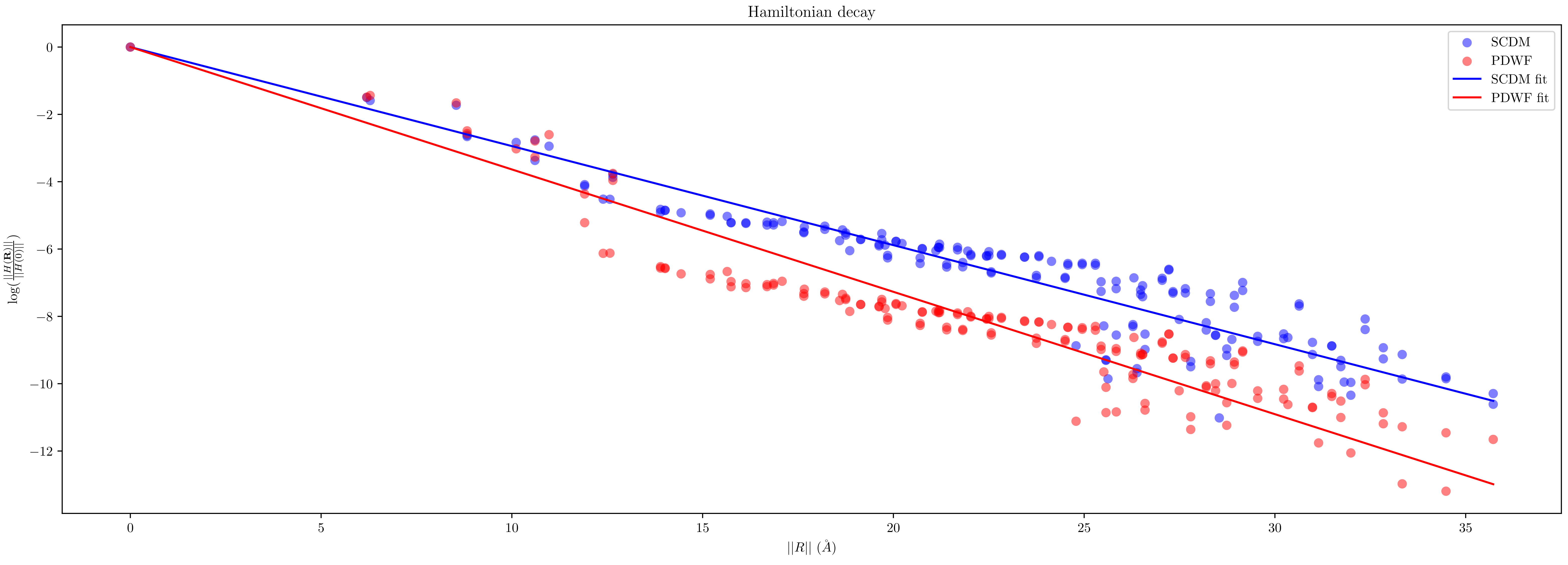

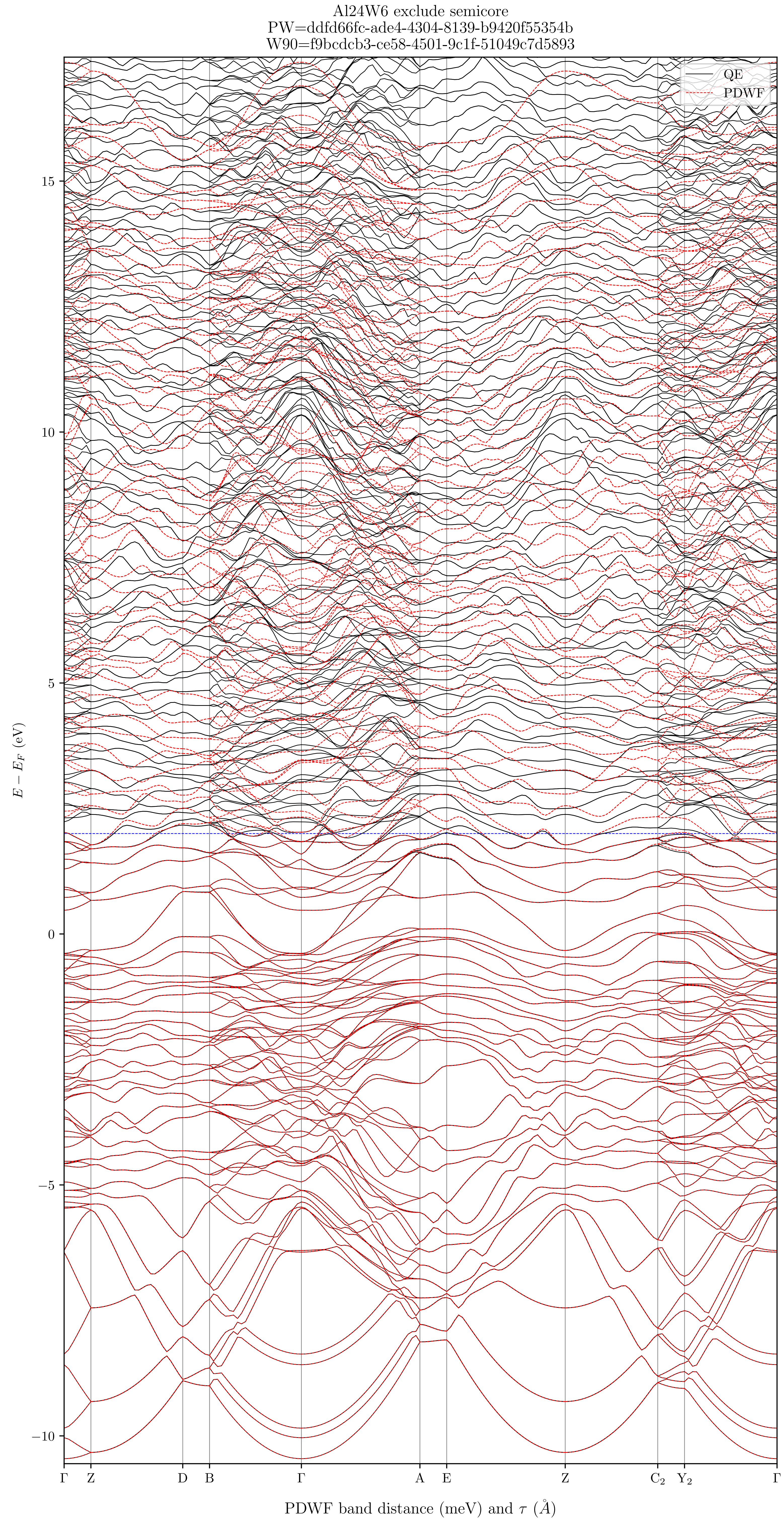

η_0	η_2	η_4	$\eta_0^{\max}$	$\eta_2^{\max}$	$\eta_4^{\max}$	τ
0.9	7.7	166.4	5.6	37.9	636.6	2.753

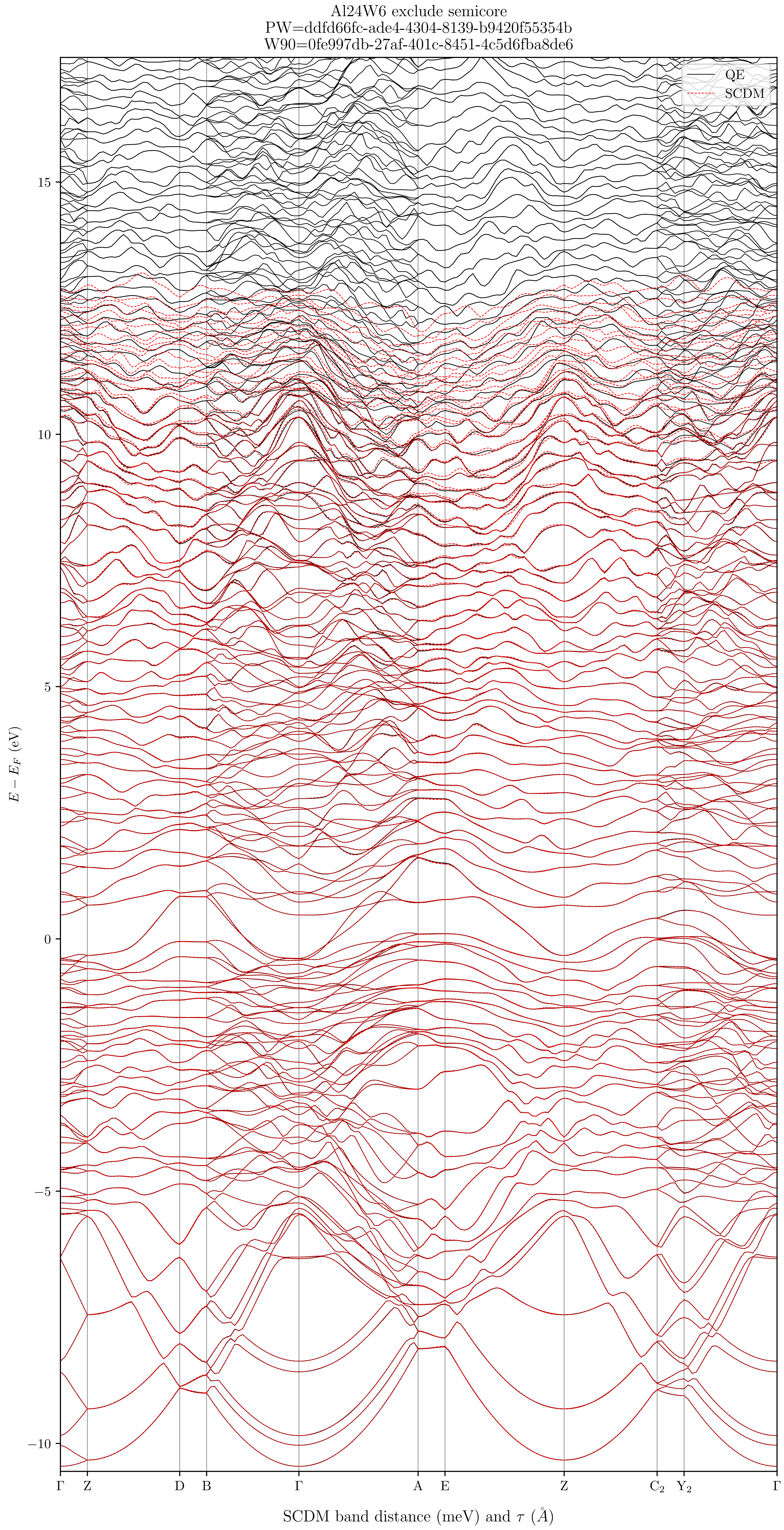

η_0	η_2	η_4	$\eta_0^{\max}$	$\eta_2^{\max}$	$\eta_4^{\max}$	τ
2.9	3.0	3.2	15.8	18.9	19.1	3.643

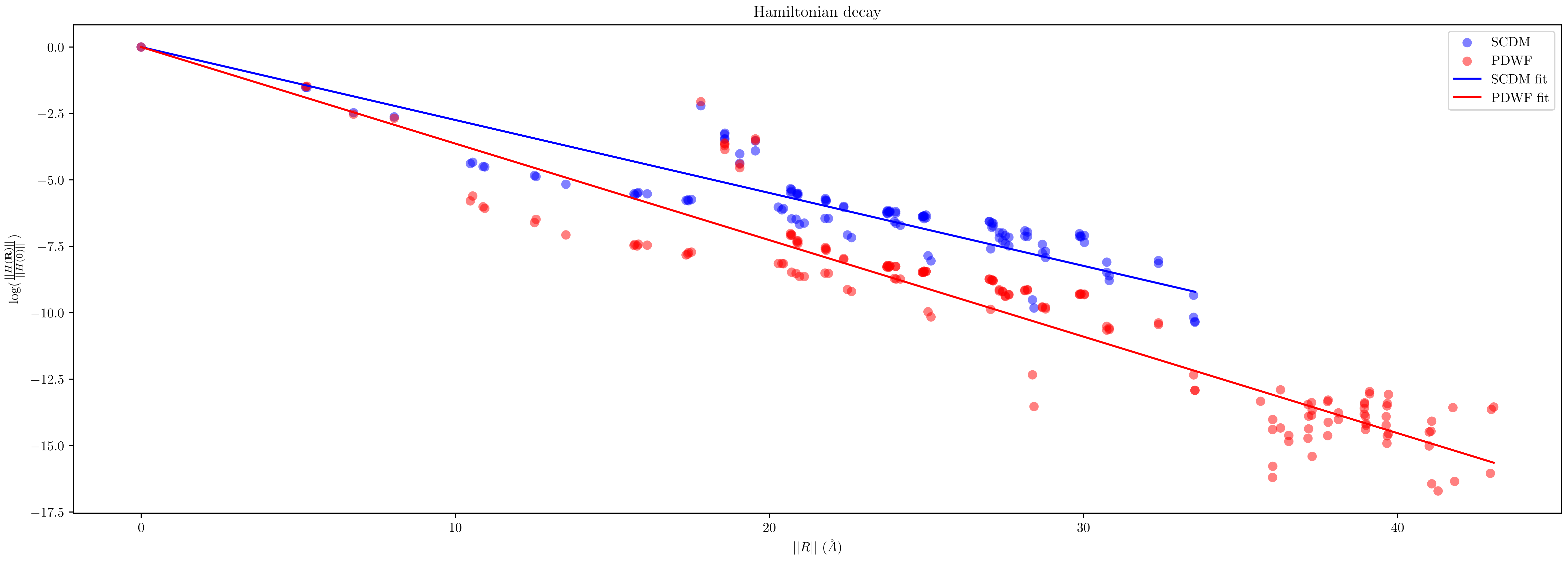

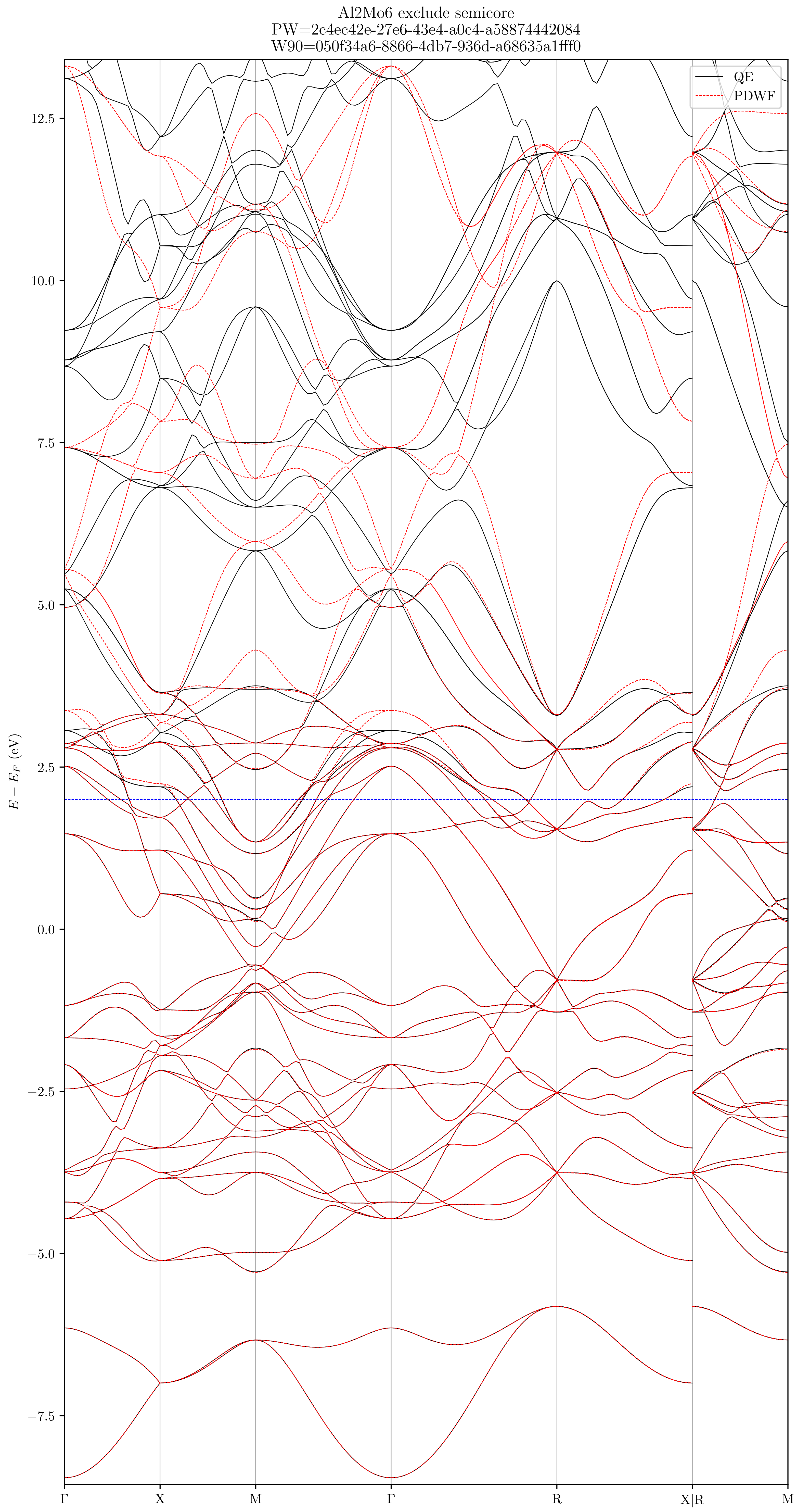

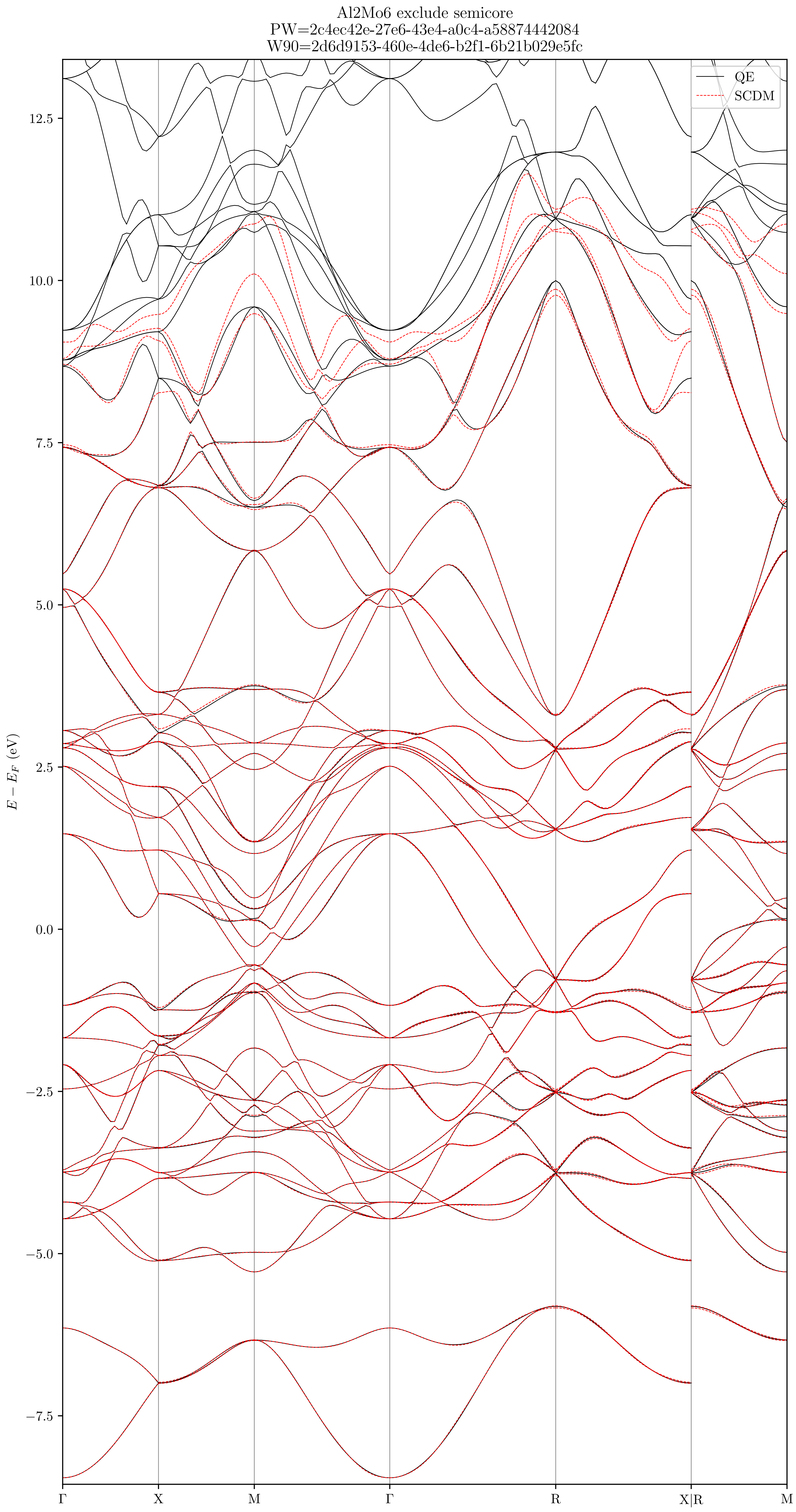

PDWF band distance (meV) and τ ($\AA$)

η_0	η_2	η_4	$\eta_0^{\max}$	$\eta_2^{\max}$	$\eta_4^{\max}$	τ
2.4	3.1	37.8	18.3	18.3	309.5	2.313

SCDM band distance (meV) and τ ($\AA$)

η_0	η_2	η_4	$\eta_0^{\max}$	$\eta_2^{\max}$	$\eta_4^{\max}$	τ
7.9	7.5	7.8	47.6	47.6	58.7	2.742

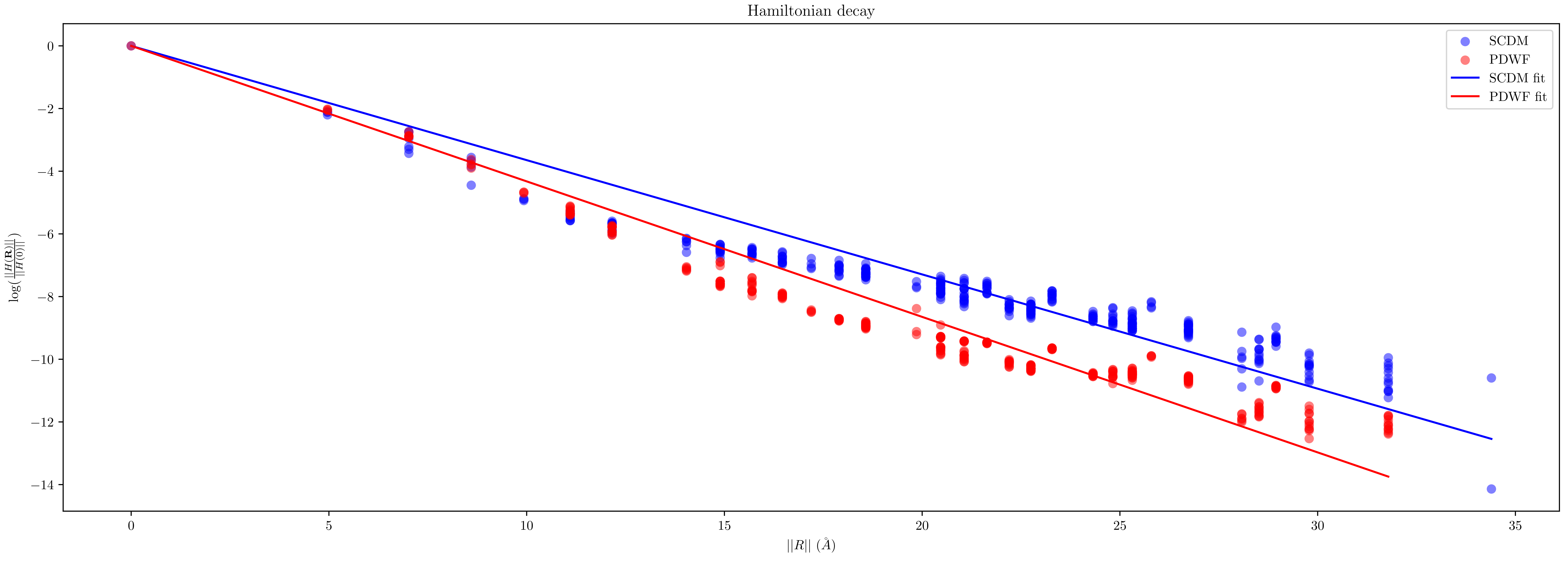

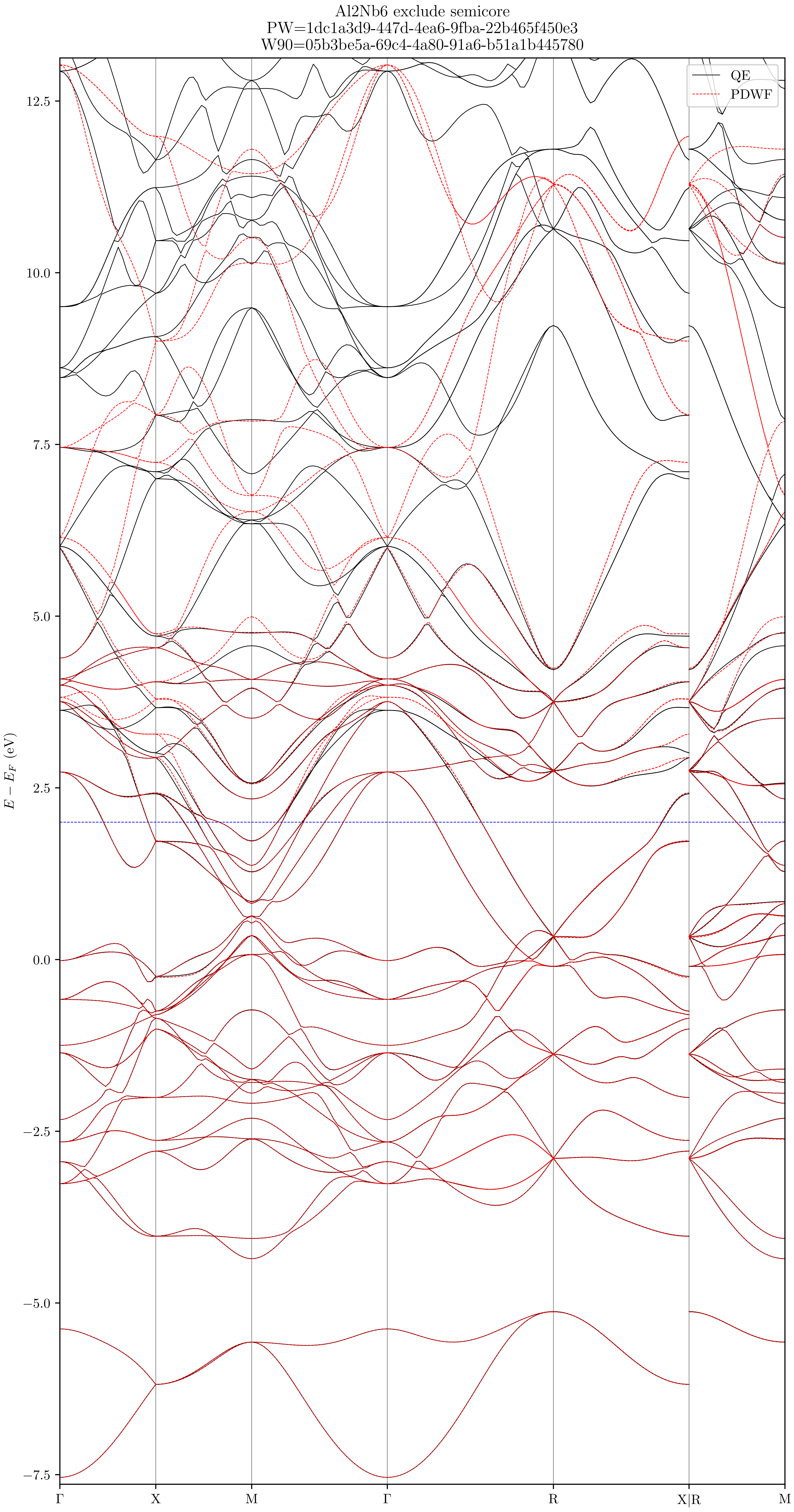

PDWF band distance (meV) and τ ($\AA$)

η_0	η_2	η_4	$\eta_0^{\max}$	$\eta_2^{\max}$	$\eta_4^{\max}$	τ
2.3	3.0	28.7	15.7	17.4	273.0	2.489

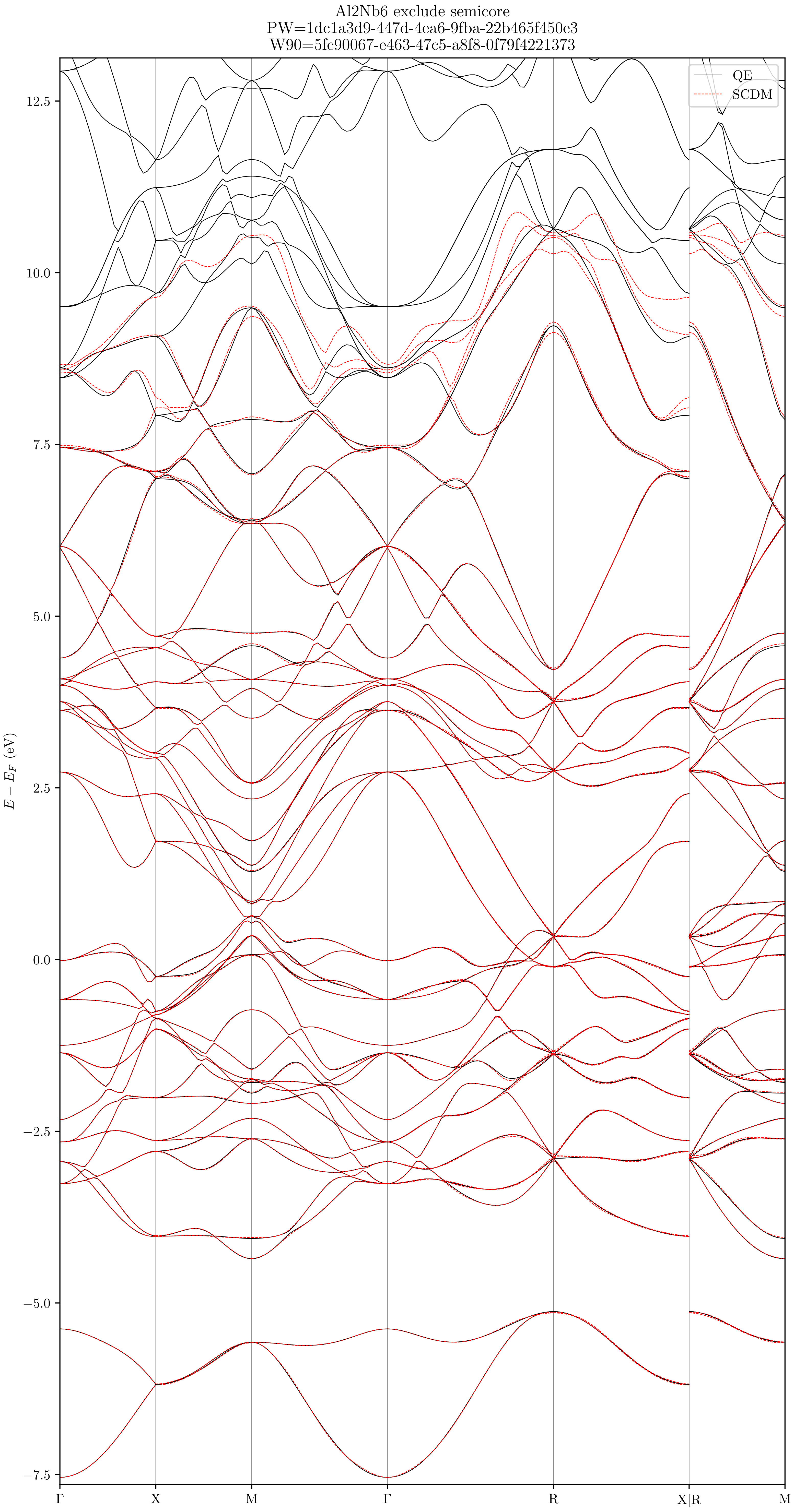

SCDM band distance (meV) and τ ($\AA$)

η_0	η_2	η_4	$\eta_0^{\max}$	$\eta_2^{\max}$	$\eta_4^{\max}$	τ
7.8	7.4	7.0	49.2	49.2	49.2	2.815

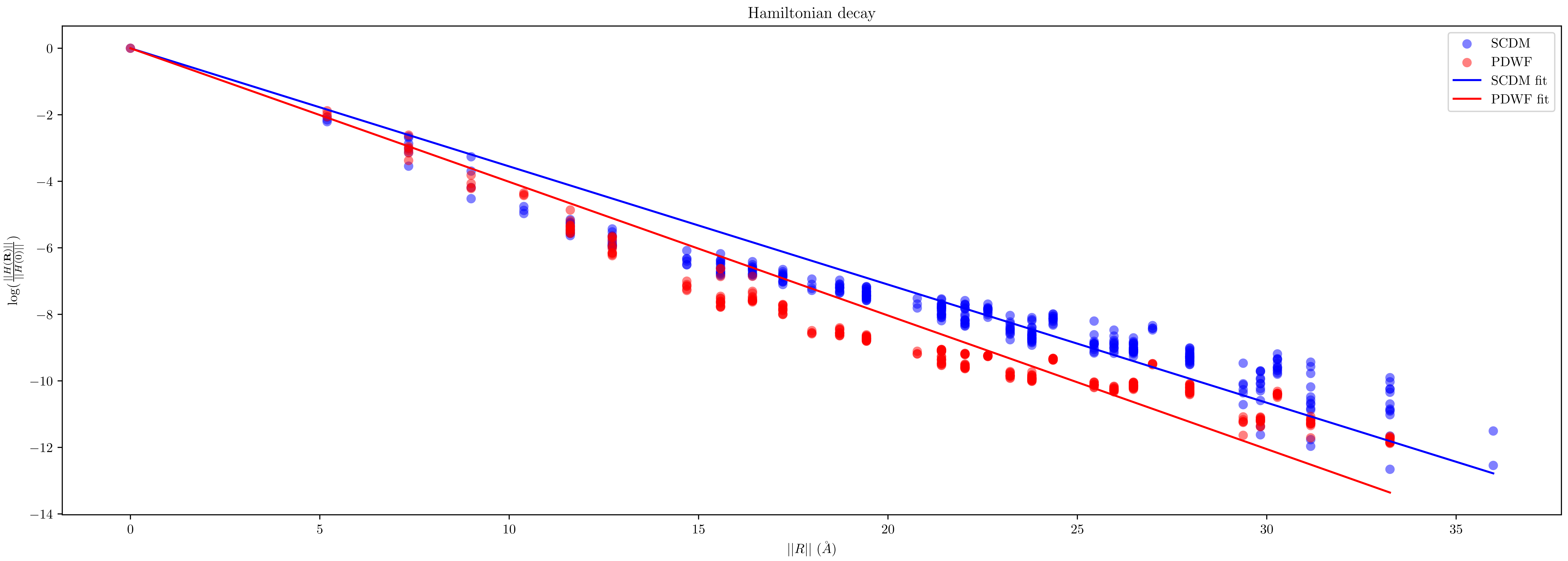

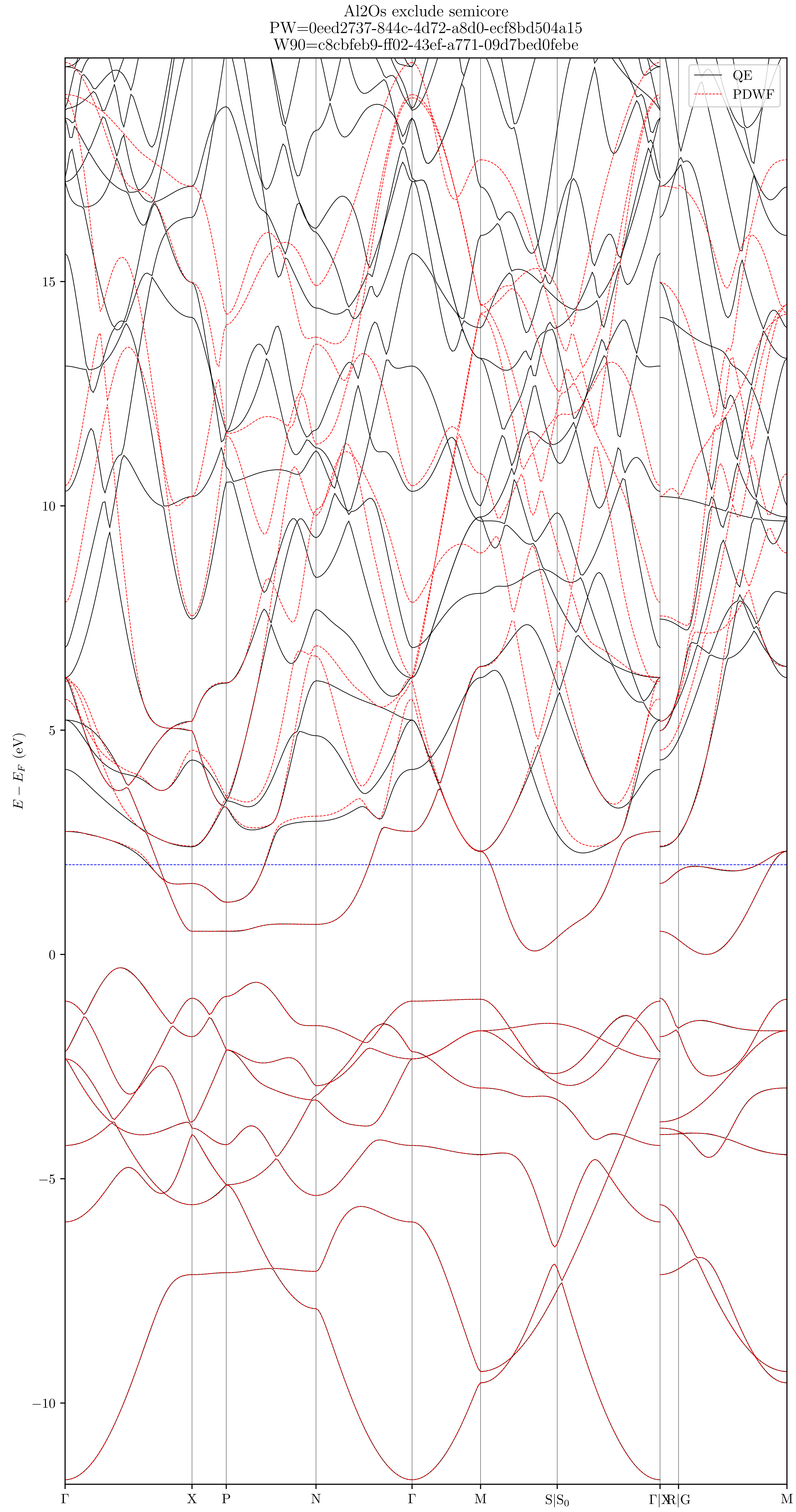

PDWF band distance (meV) and τ ($\AA$)

η_0	η_2	η_4	$\eta_0^{\max}$	$\eta_2^{\max}$	$\eta_4^{\max}$	τ
3.0	3.4	52.3	14.1	14.1	647.4	2.537

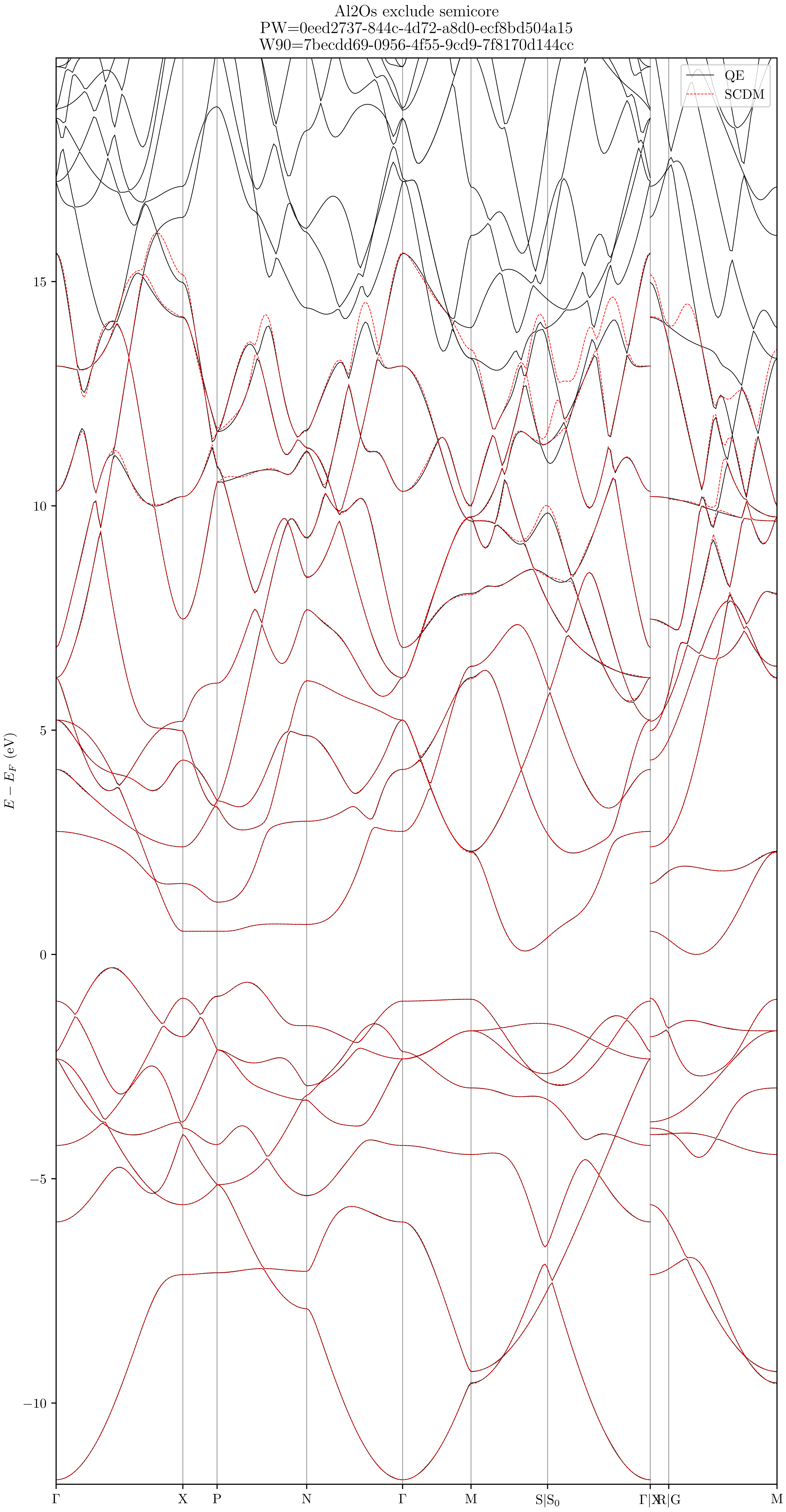

SCDM band distance (meV) and τ ($\AA$)

η_0	η_2	η_4	$\eta_0^{\max}$	$\eta_2^{\max}$	$\eta_4^{\max}$	τ
4.5	4.4	4.9	21.0	21.0	27.6	2.733

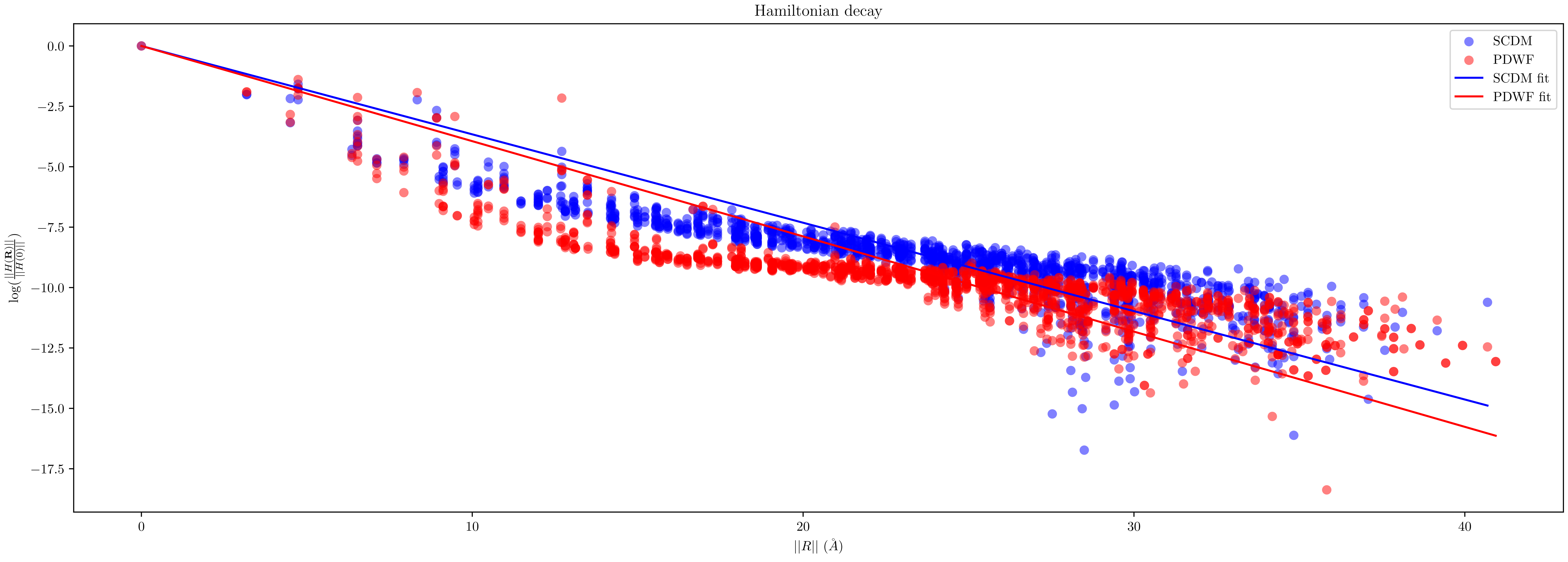

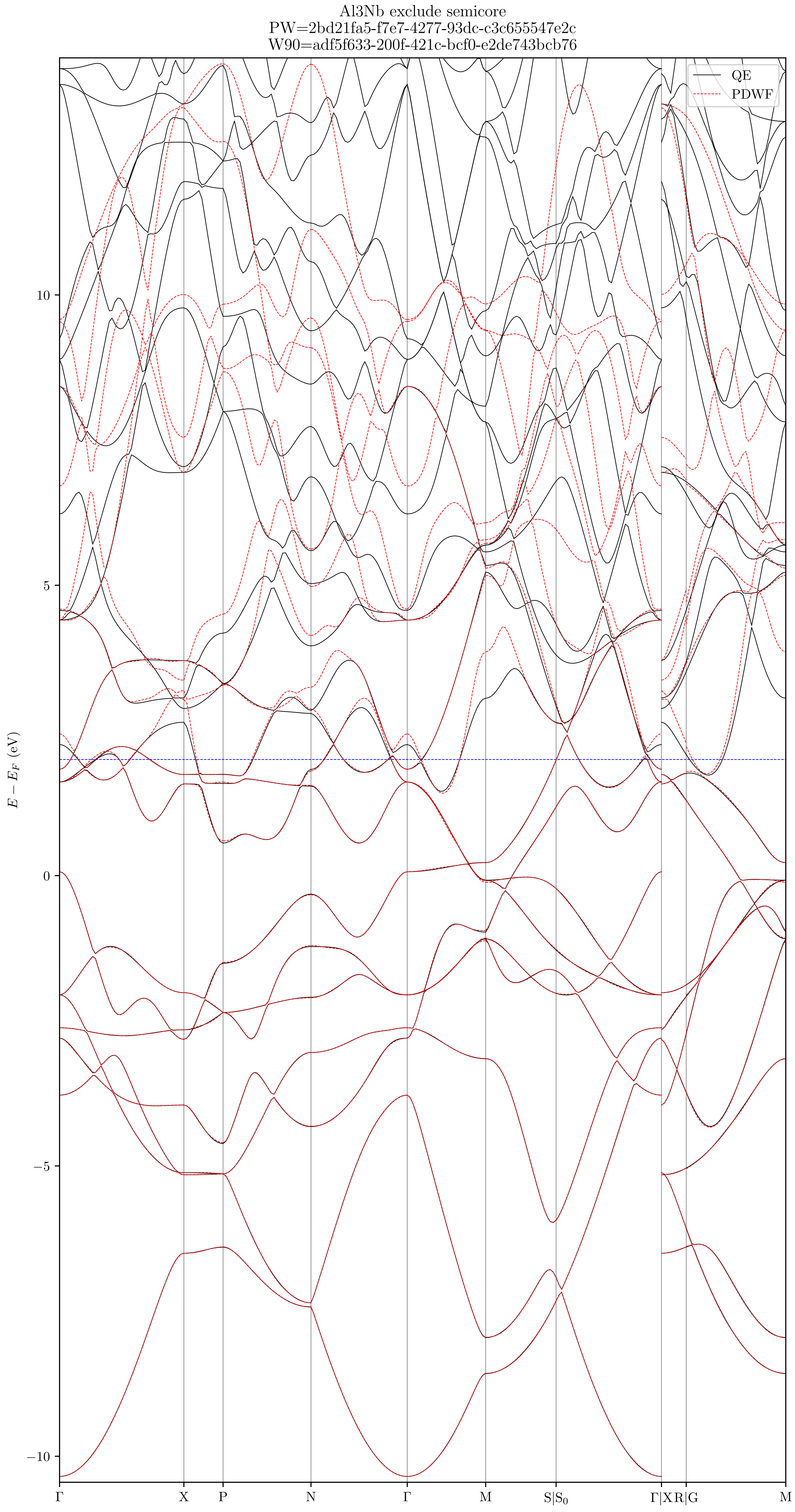

PDWF band distance (meV) and τ ($\AA$)

η_0	η_2	η_4	$\eta_0^{\max}$	$\eta_2^{\max}$	$\eta_4^{\max}$	τ
6.1	9.6	86.3	30.1	57.4	714.8	2.659

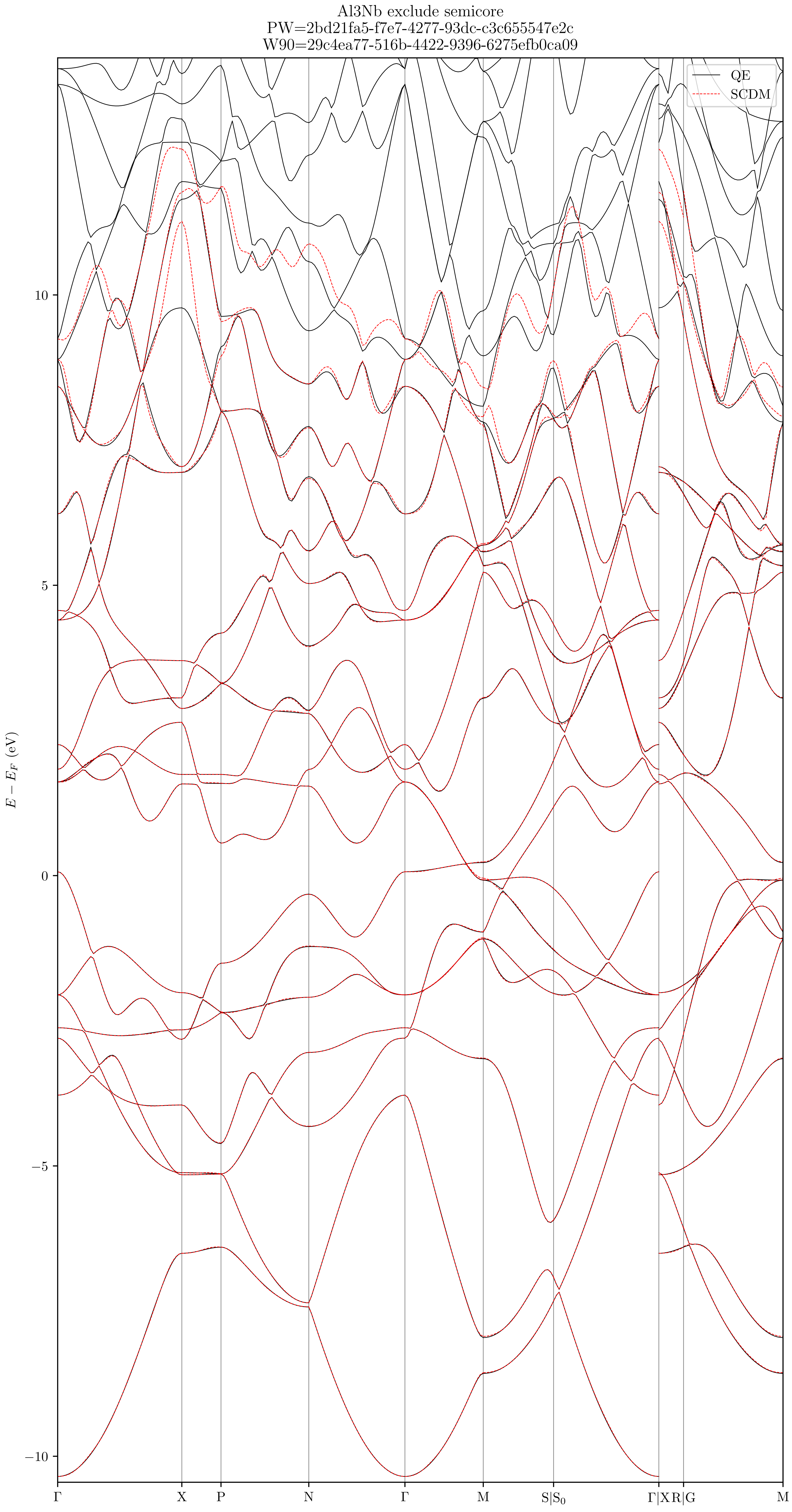

SCDM band distance (meV) and τ ($\AA$)

η_0	η_2	η_4	$\eta_0^{\max}$	$\eta_2^{\max}$	$\eta_4^{\max}$	τ
6.0	5.9	6.6	24.5	34.4	34.4	2.872

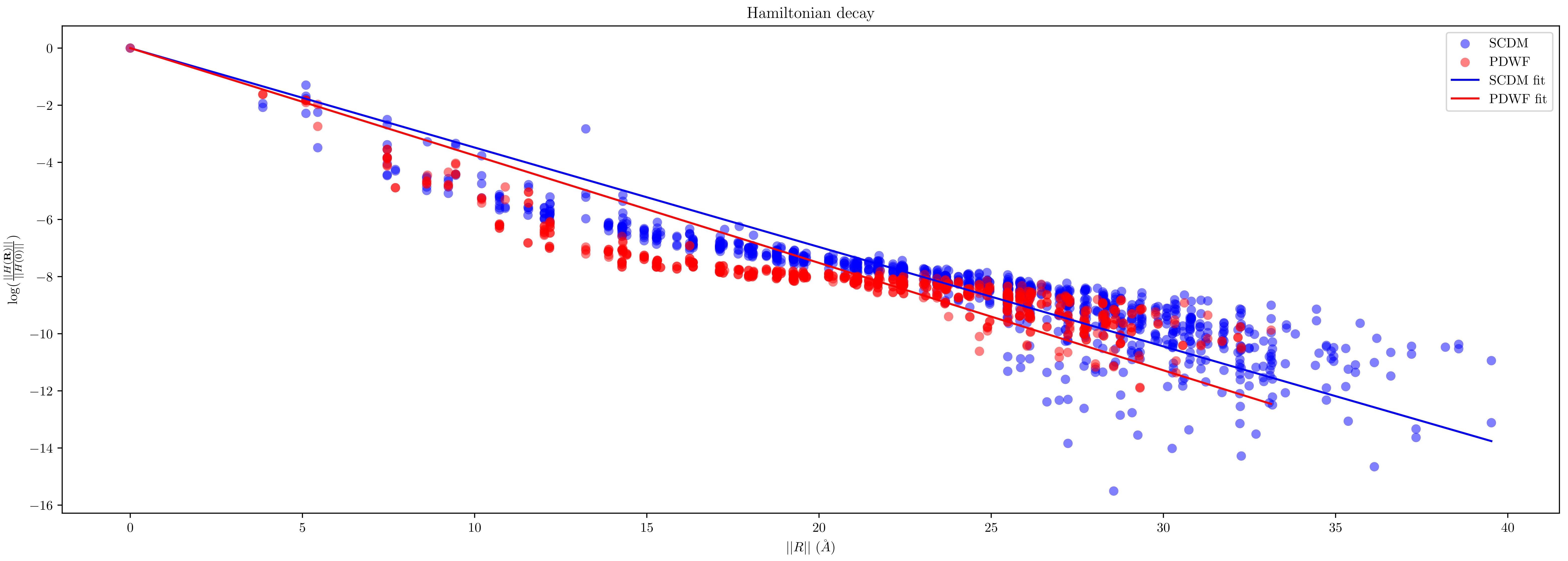

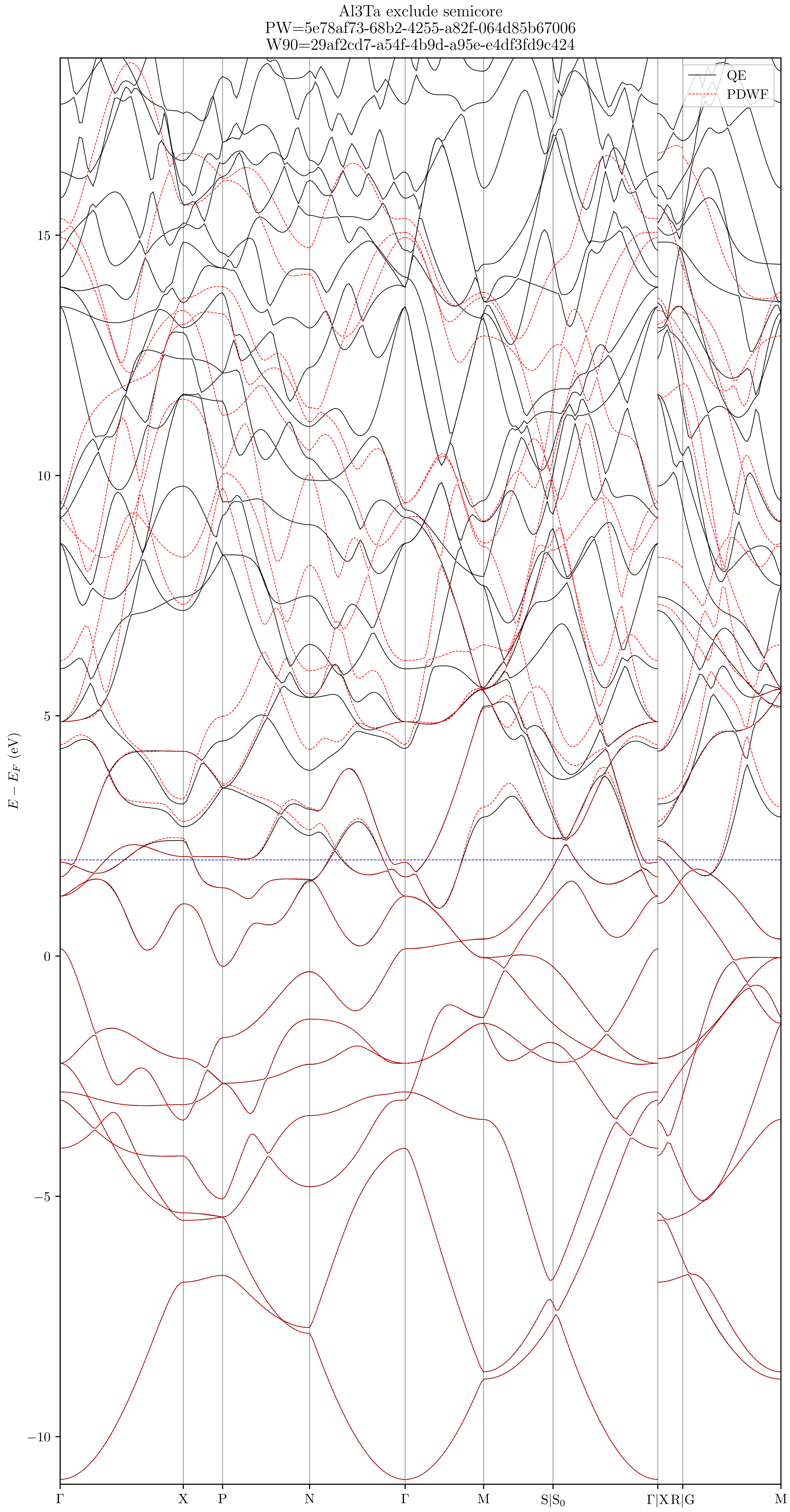

PDWF band distance (meV) and τ ($\AA$)

η_0	η_2	η_4	$\eta_0^{\max}$	$\eta_2^{\max}$	$\eta_4^{\max}$	τ
2.8	4.9	50.3	13.9	46.6	473.0	2.514

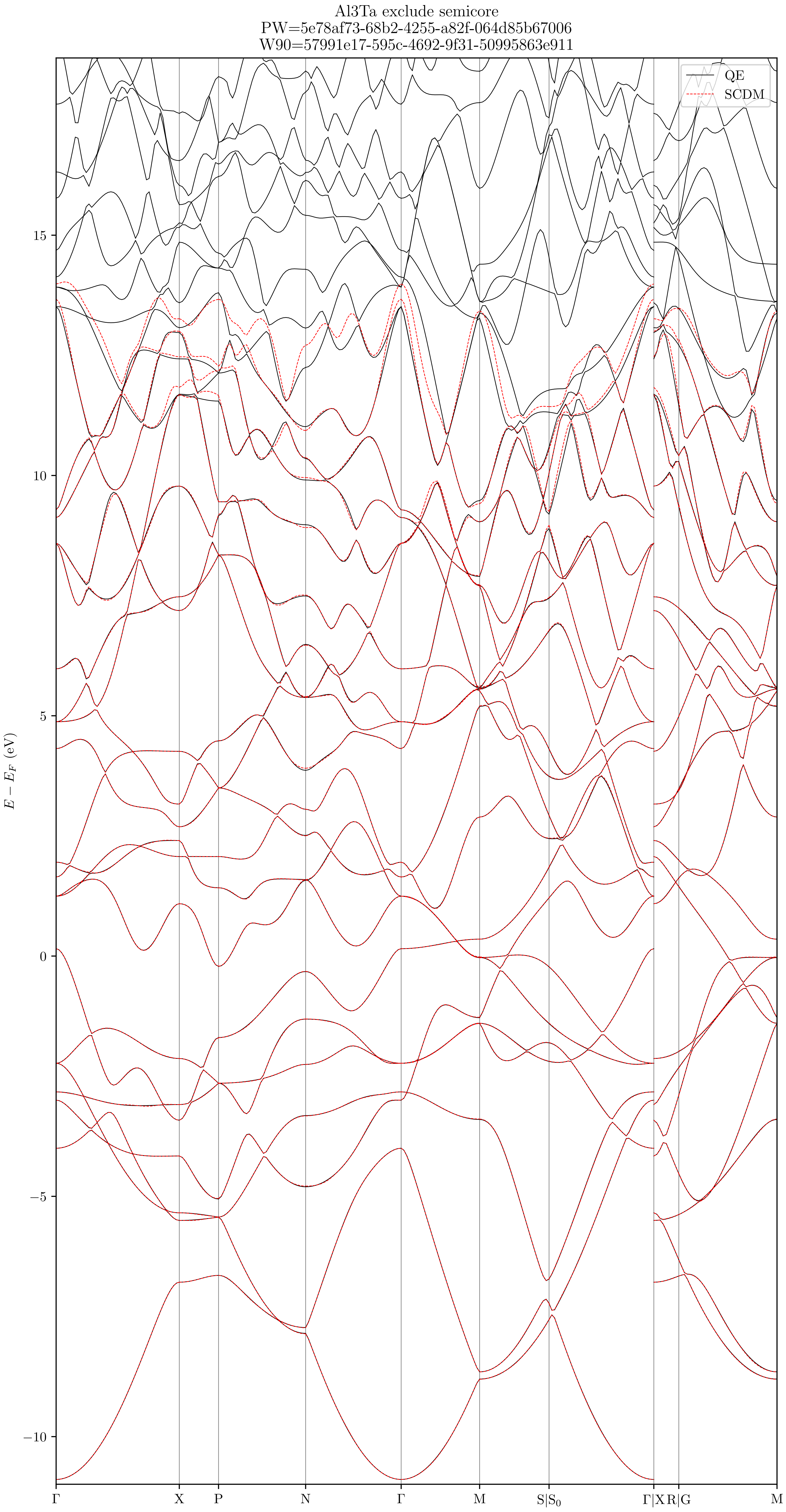

SCDM band distance (meV) and τ ($\AA$)

η_0	η_2	η_4	$\eta_0^{\max}$	$\eta_2^{\max}$	$\eta_4^{\max}$	τ
4.5	4.5	5.0	15.0	15.0	32.8	2.782

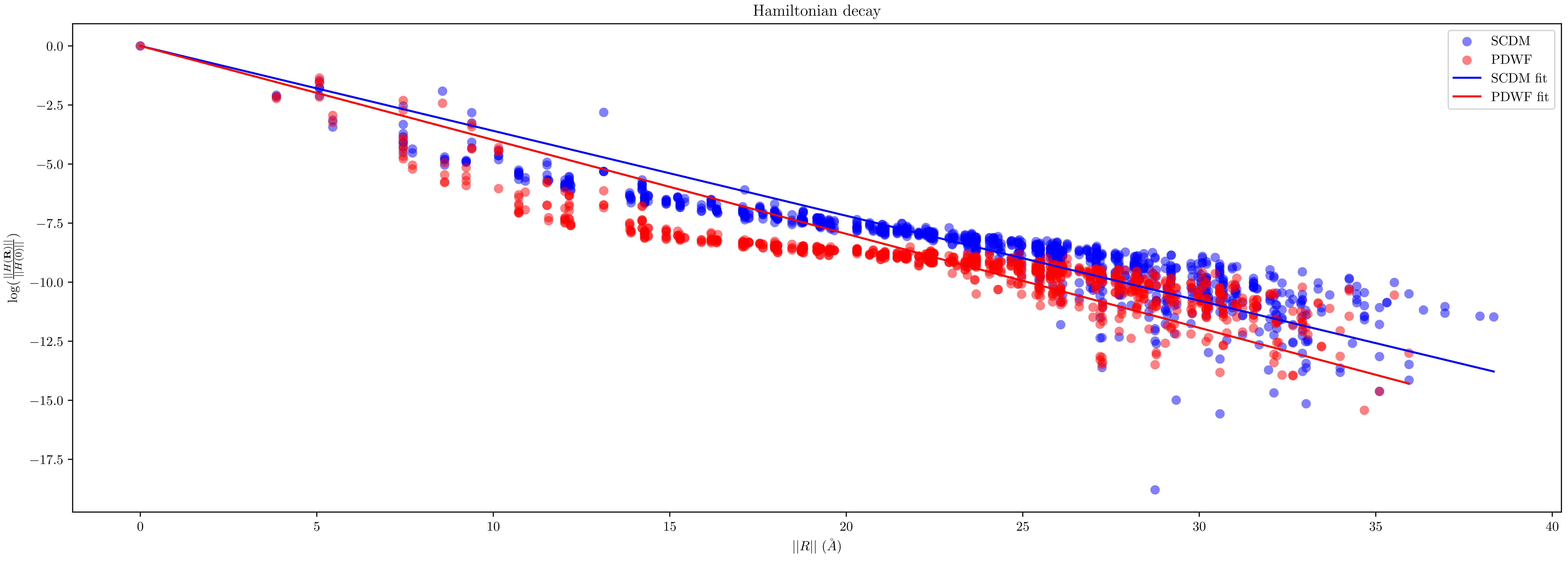

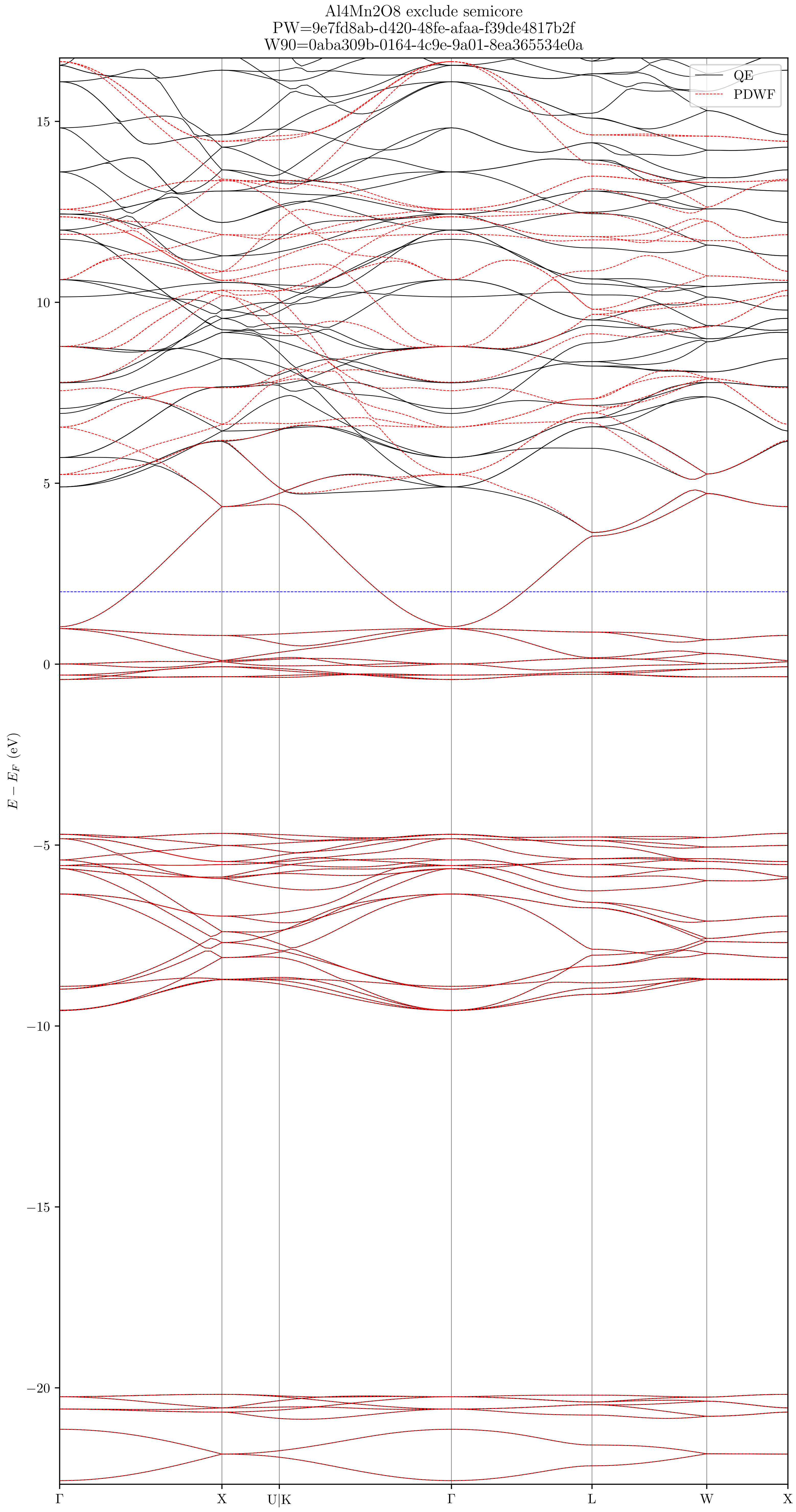

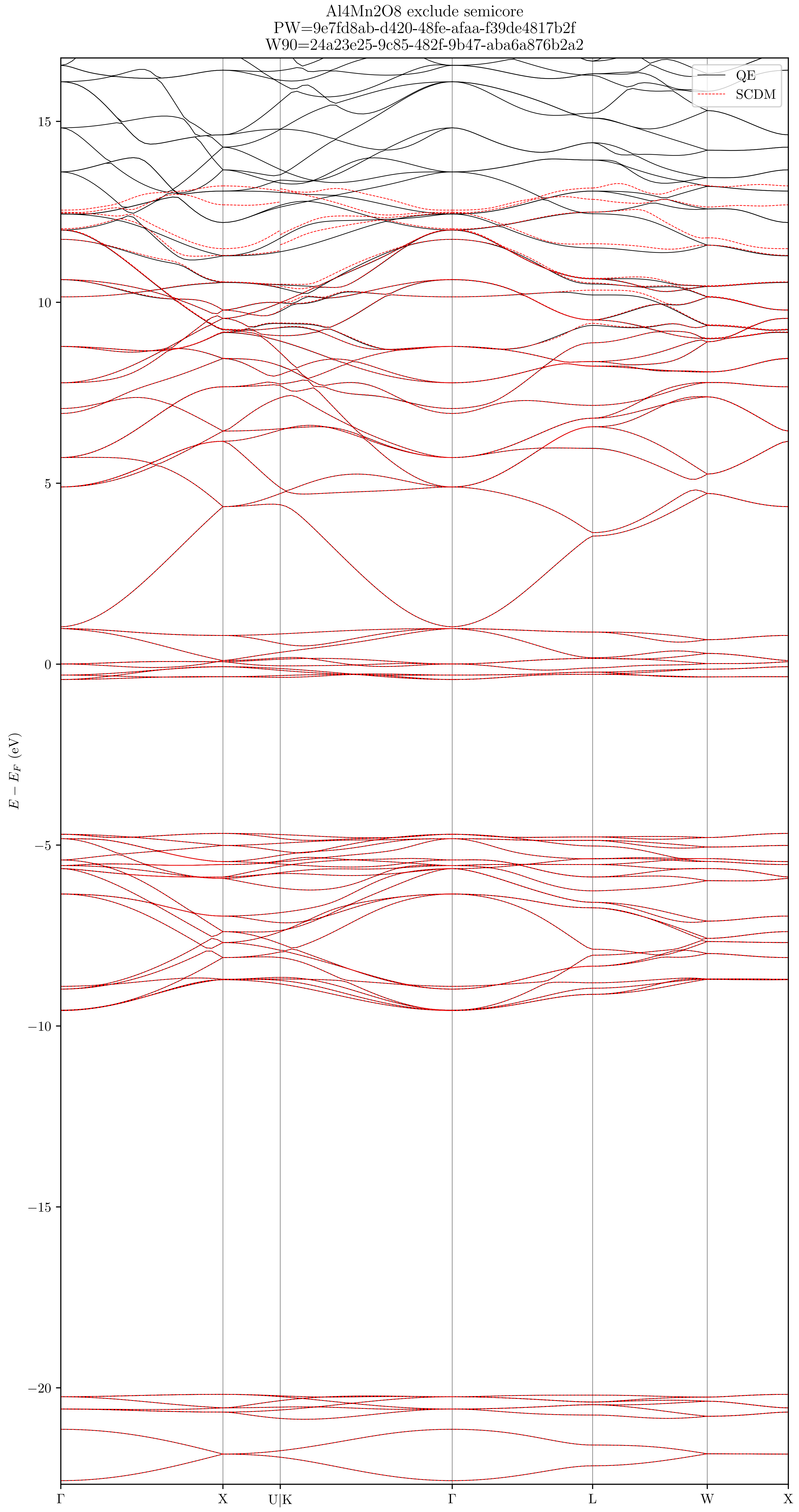

PDWF band distance (meV) and τ ($\AA$)

η_0	η_2	η_4	$\eta_0^{\max}$	$\eta_2^{\max}$	$\eta_4^{\max}$	τ
0.6	0.7	0.9	3.7	4.2	9.6	2.418

SCDM band distance (meV) and τ ($\AA$)

η_0	η_2	η_4	$\eta_0^{\max}$	$\eta_2^{\max}$	$\eta_4^{\max}$	τ
0.3	0.3	0.3	2.4	2.4	2.5	2.401

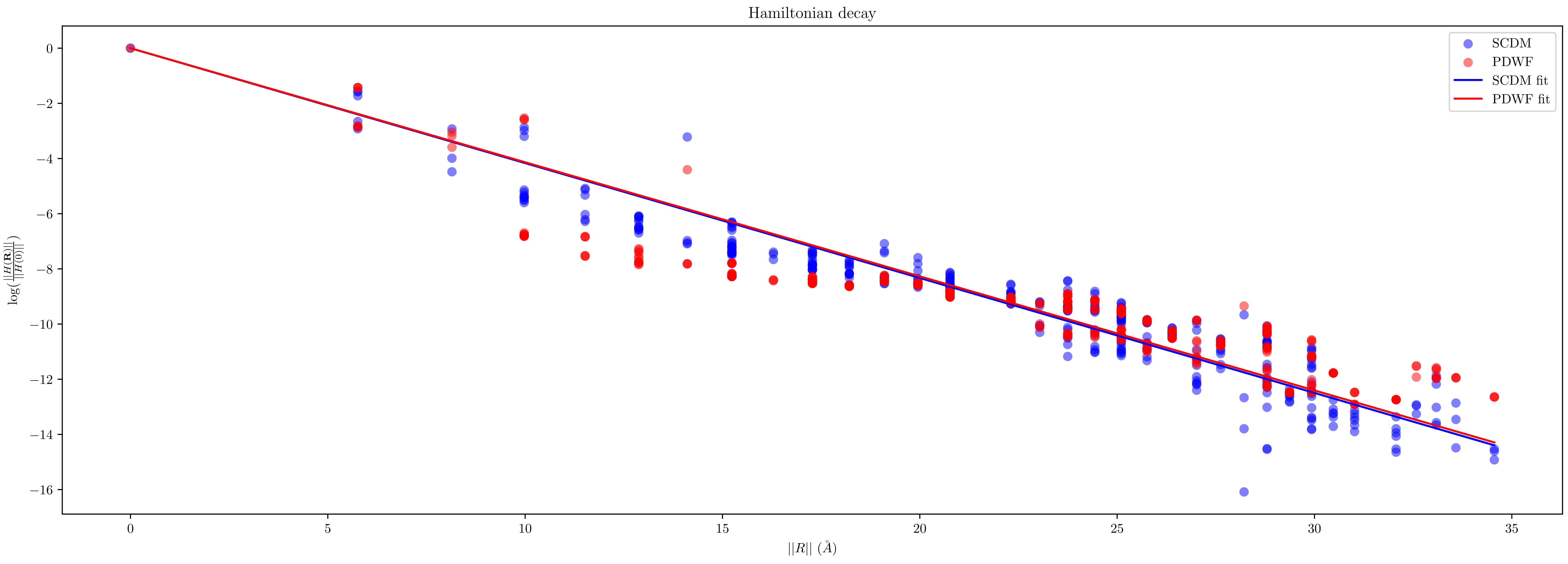

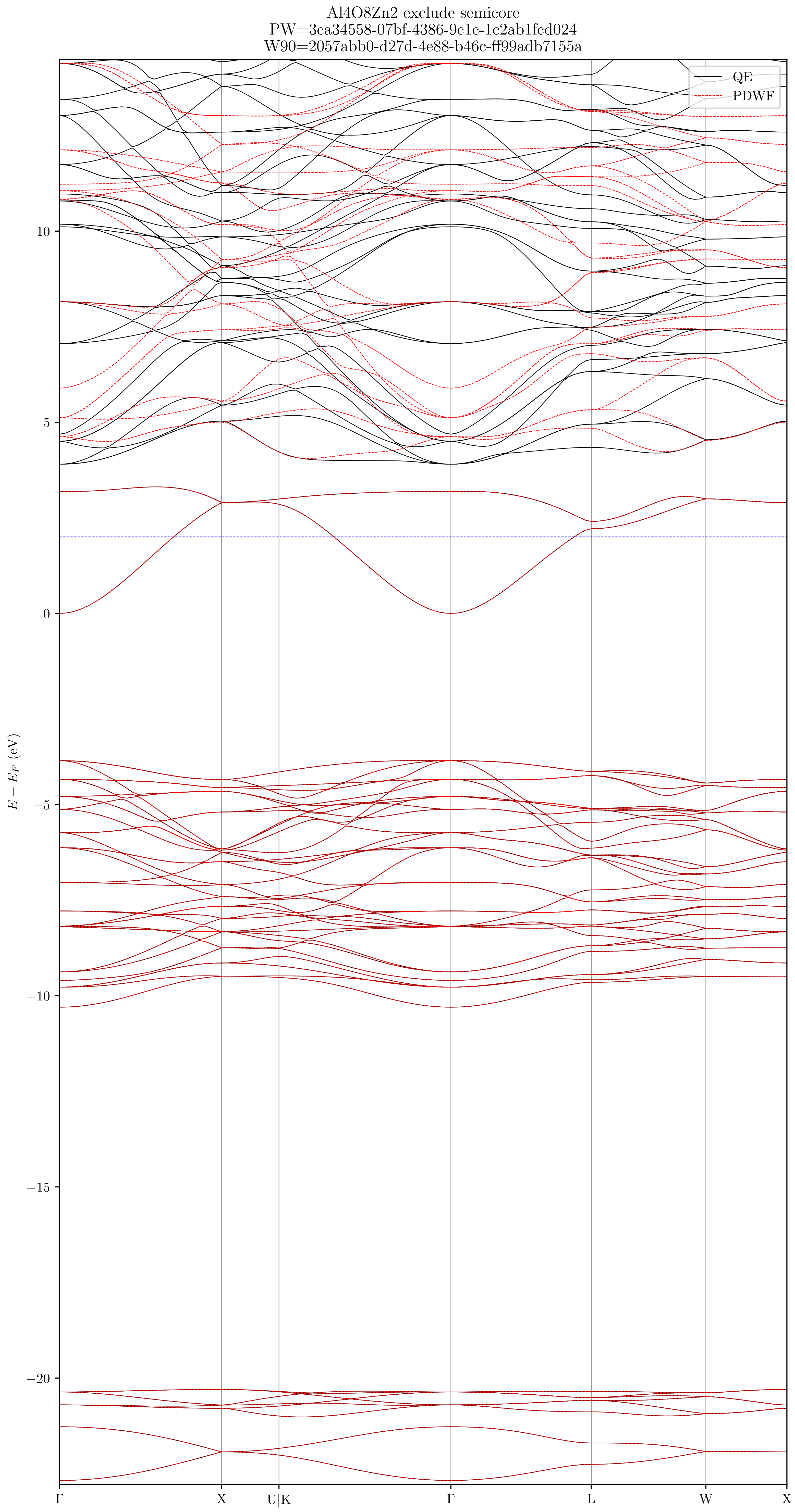

PDWF band distance (meV) and τ ($\AA$)

η_0	η_2	η_4	$\eta_0^{\max}$	$\eta_2^{\max}$	$\eta_4^{\max}$	τ
0.6	0.6	0.6	5.5	5.5	5.5	2.392

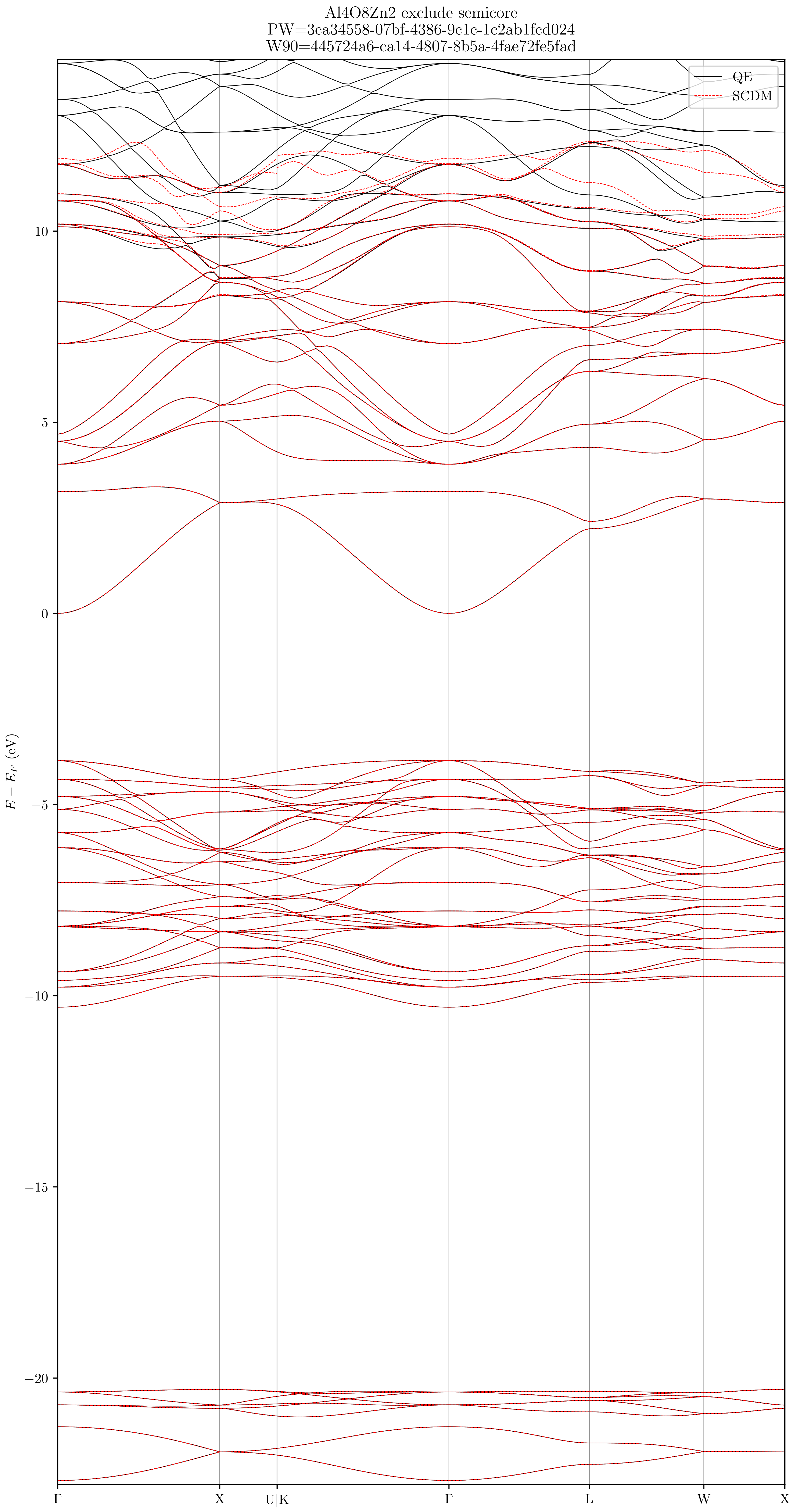

SCDM band distance (meV) and τ ($\AA$)

η_0	η_2	η_4	$\eta_0^{\max}$	$\eta_2^{\max}$	$\eta_4^{\max}$	τ
0.4	0.4	0.4	2.3	2.3	2.3	2.534

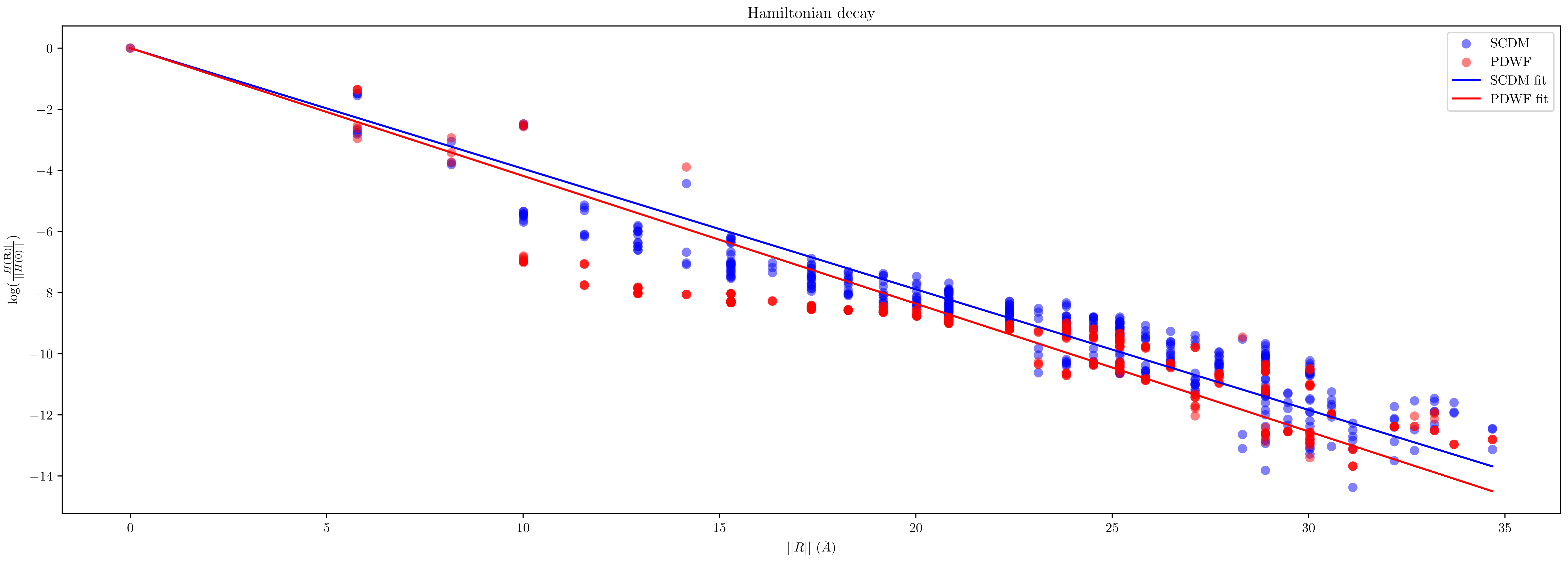

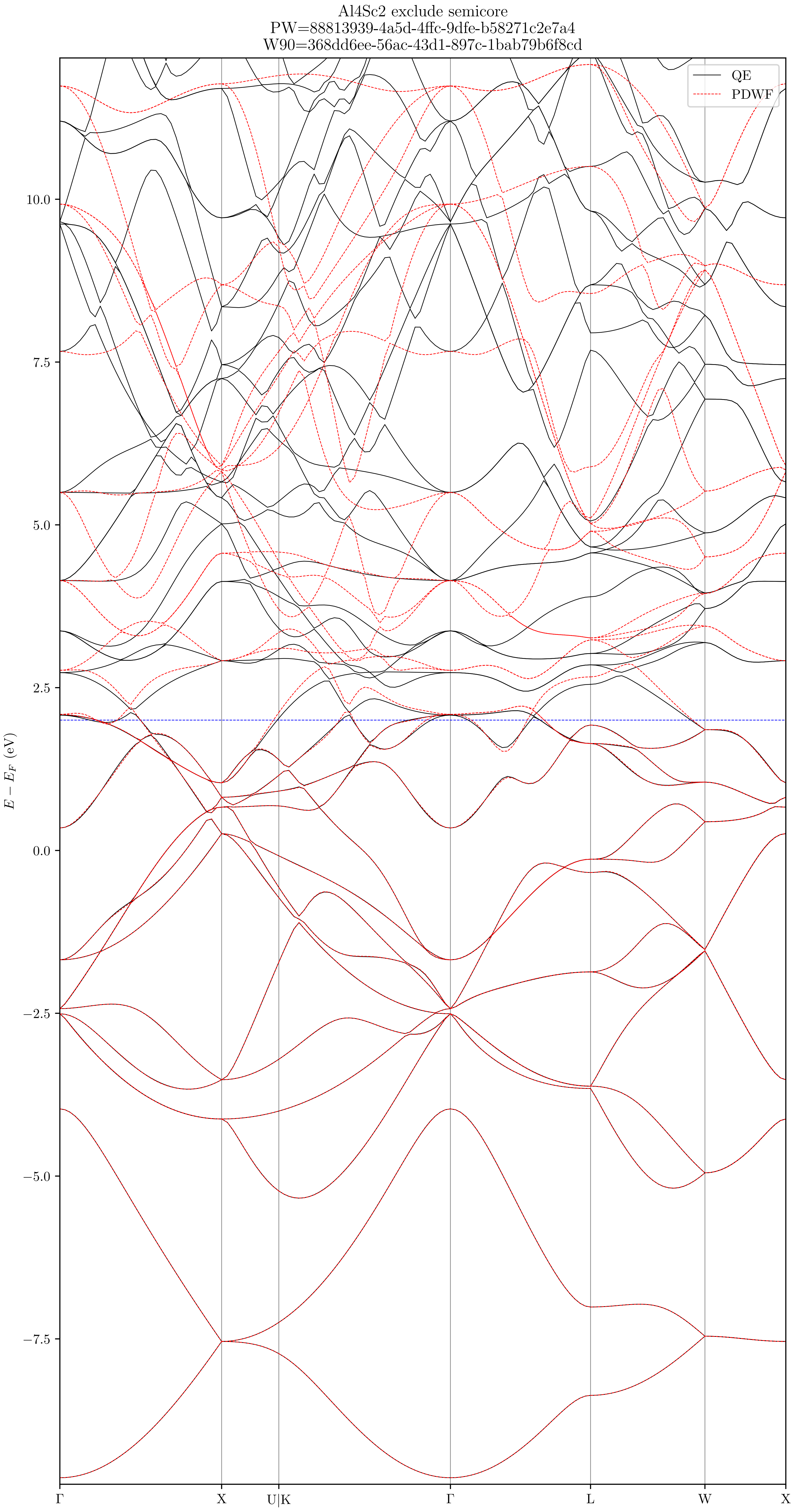

PDWF band distance (meV) and τ ($\AA$)

η_0	η_2	η_4	$\eta_0^{\max}$	$\eta_2^{\max}$	$\eta_4^{\max}$	τ
2.1	8.4	177.7	16.0	62.3	707.7	2.516

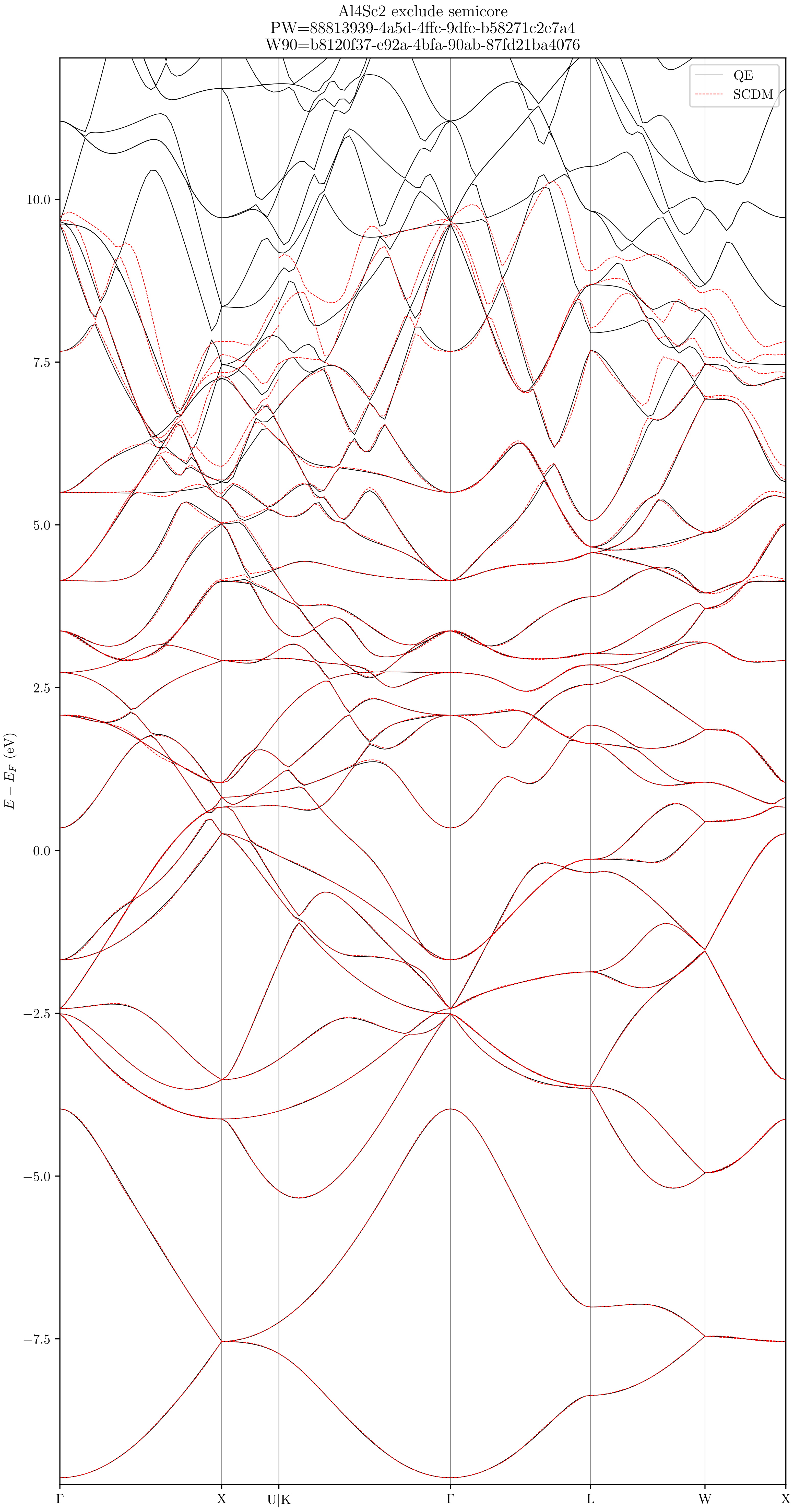

SCDM band distance (meV) and τ ($\AA$)

η_0	η_2	η_4	$\eta_0^{\max}$	$\eta_2^{\max}$	$\eta_4^{\max}$	τ
7.0	7.7	8.2	29.2	55.8	56.1	3.170

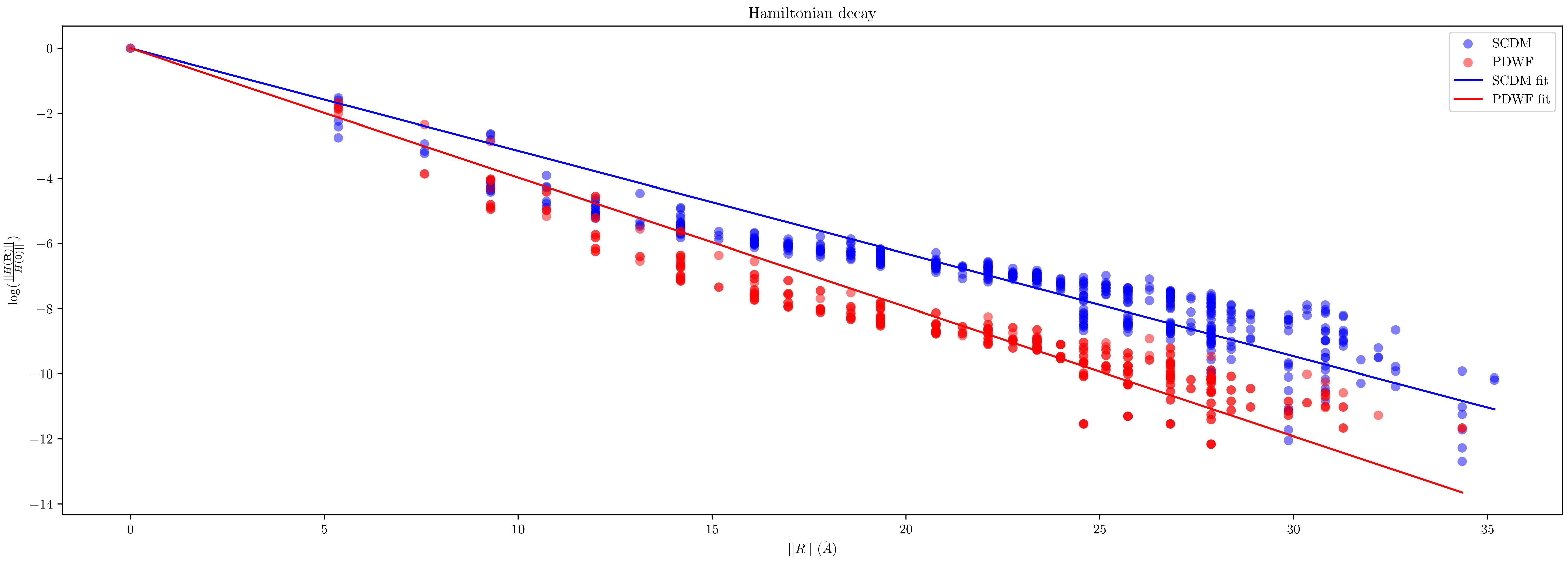

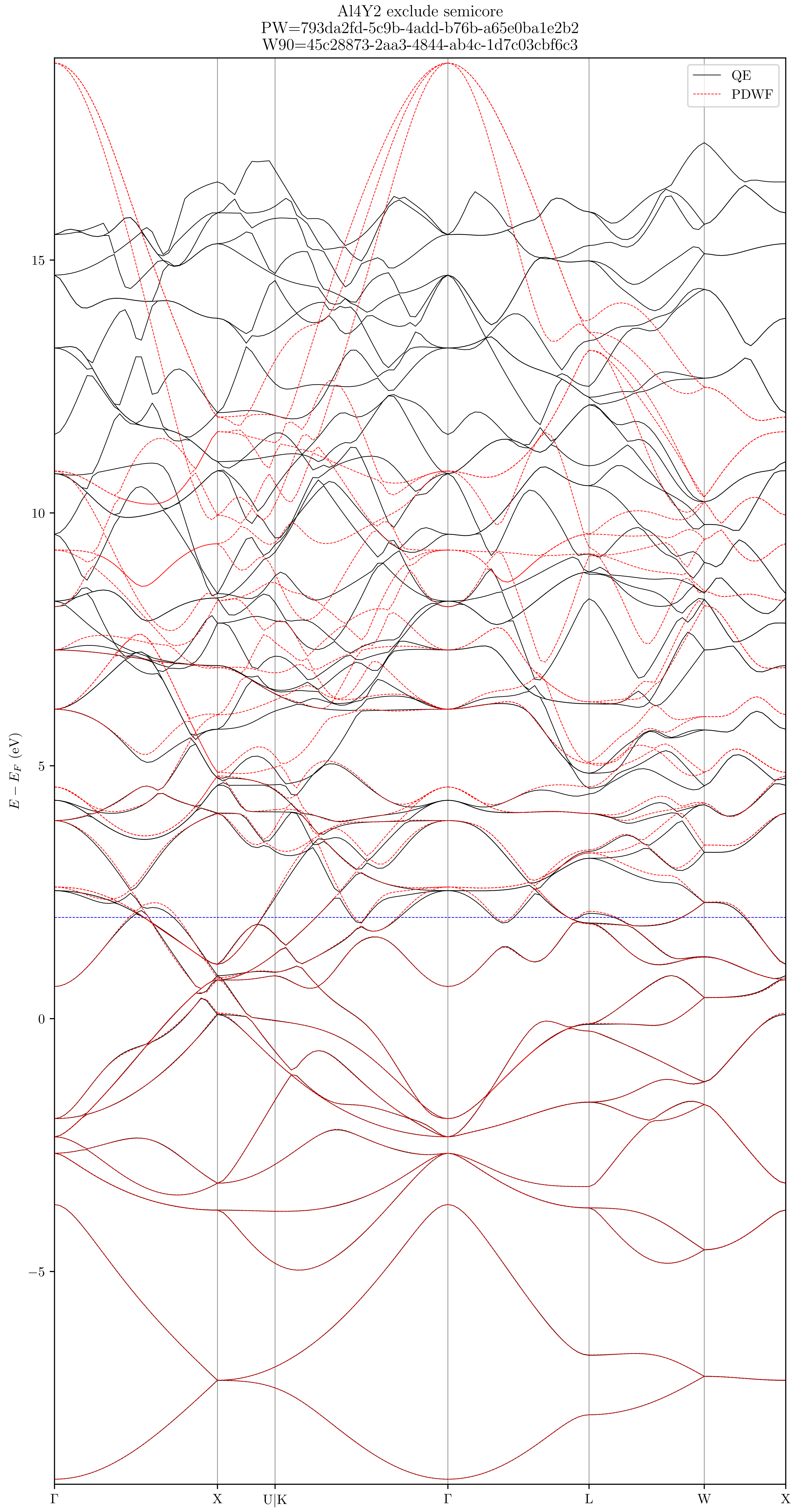

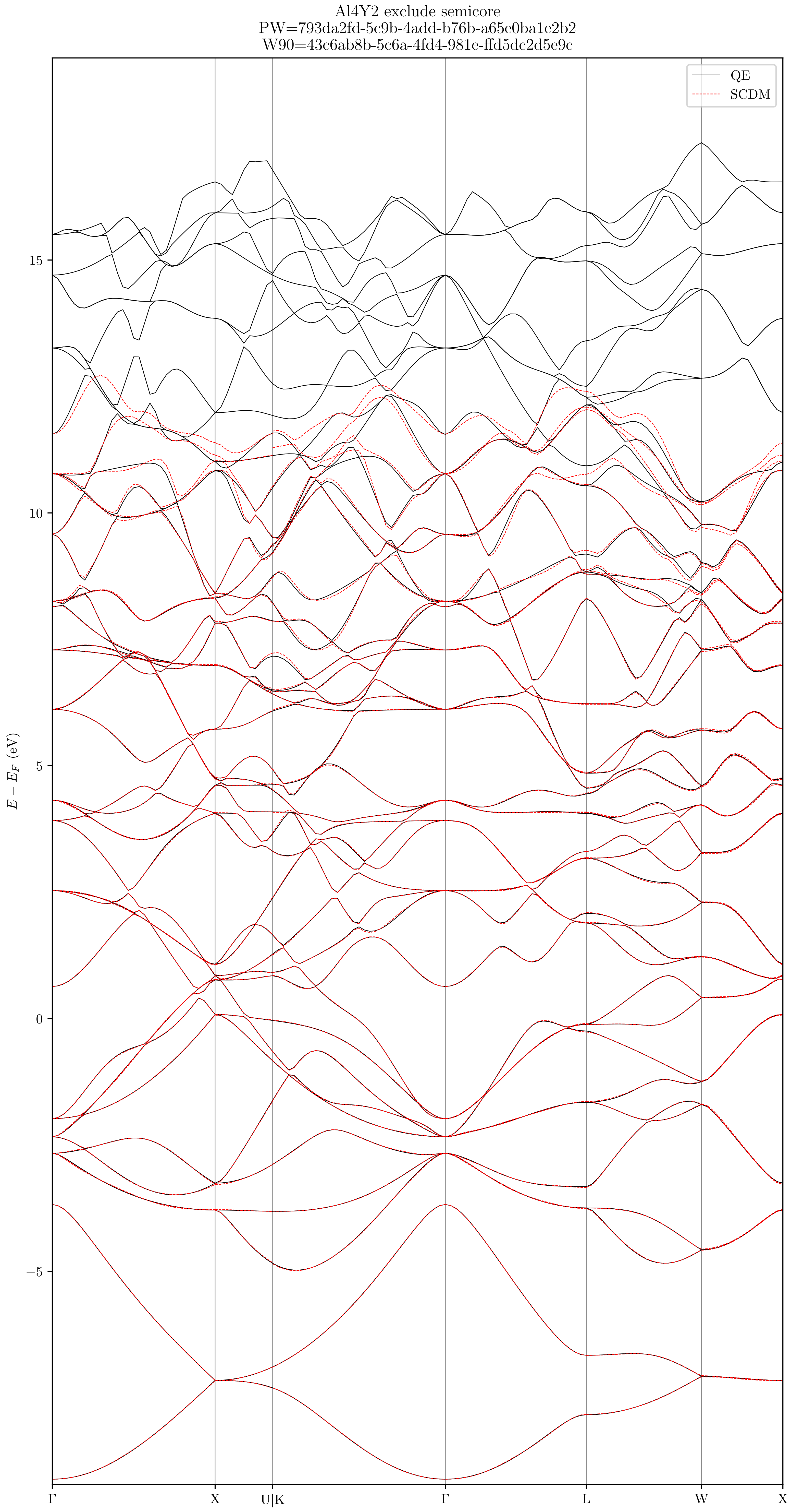

PDWF band distance (meV) and τ ($\AA$)

η_0	η_2	η_4	η_0^{max}	η_2^{max}	η_4^{max}	τ
4.1	8.6	41.3	20.2	47.4	250.3	2.517

SCDM band distance (meV) and τ ($\AA$)

η_0	η_2	η_4	η_0^{max}	η_2^{max}	η_4^{max}	τ
6.9	7.0	7.4	27.8	27.8	32.0	2.889

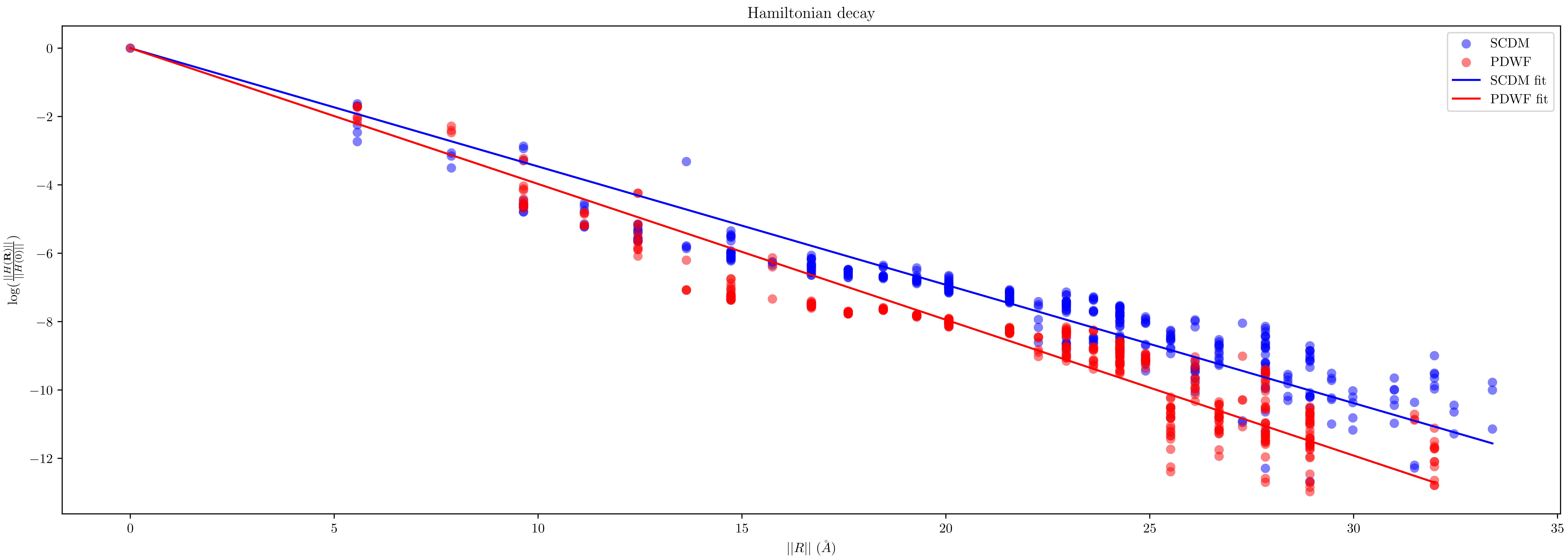

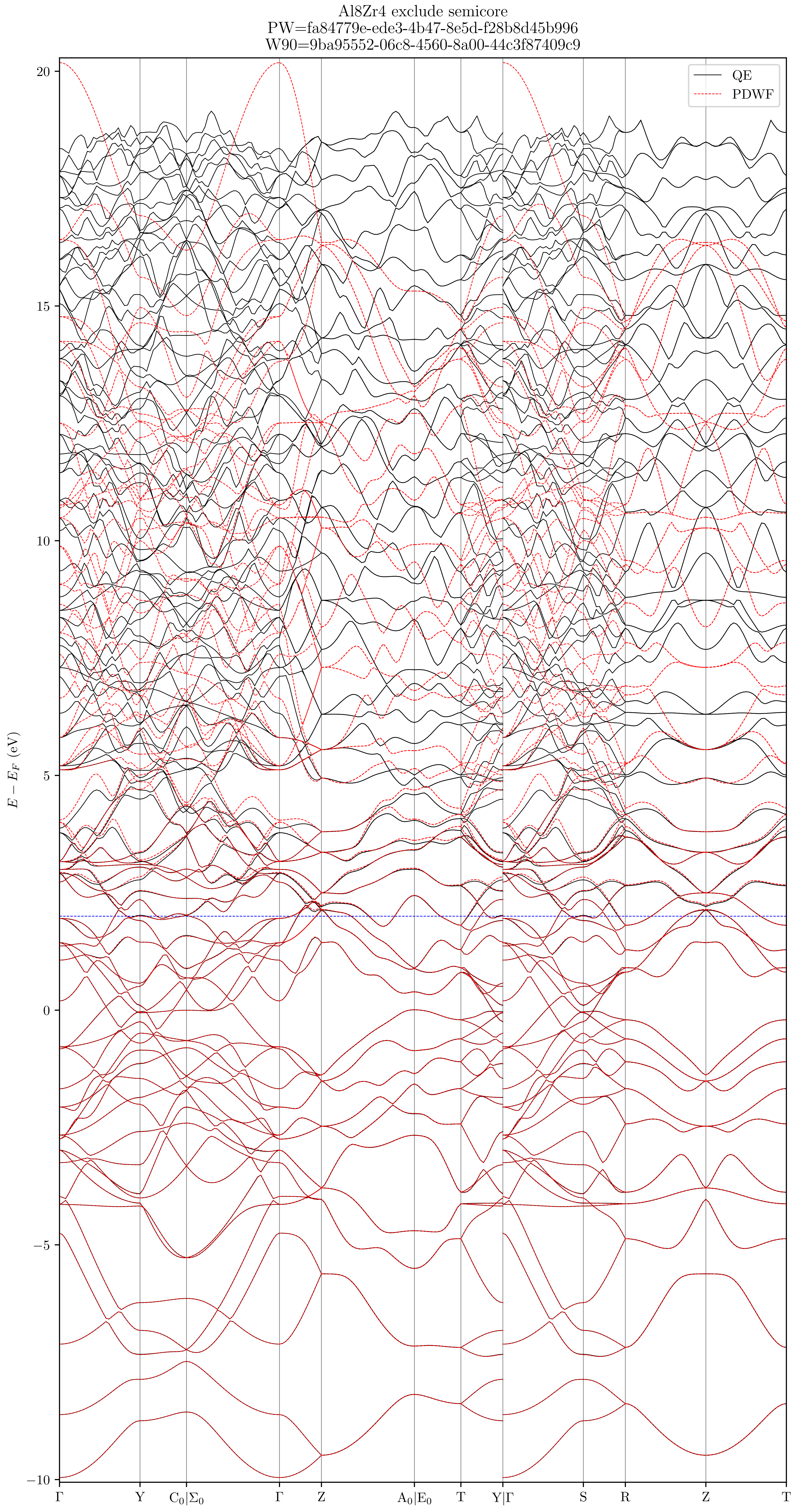

PDWF band distance (meV) and τ ($\AA$)

η_0	η_2	η_4	$\eta_0^{\max}$	$\eta_2^{\max}$	$\eta_4^{\max}$	τ
2.0	2.6	20.2	12.6	19.1	169.4	2.623

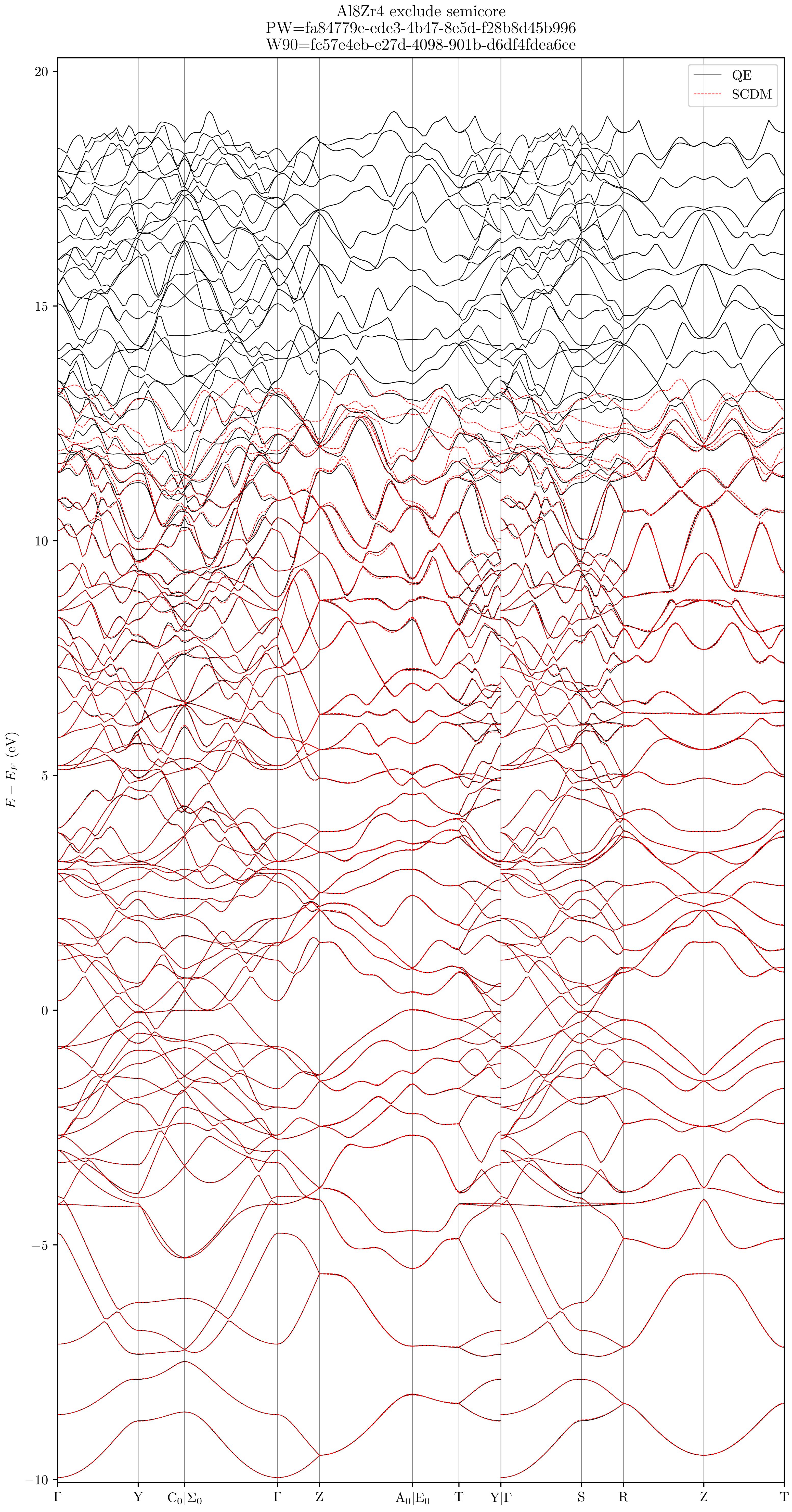

SCDM band distance (meV) and τ ($\AA$)

η_0	η_2	η_4	$\eta_0^{\max}$	$\eta_2^{\max}$	$\eta_4^{\max}$	τ
4.2	4.5	4.6	19.0	20.9	27.2	3.066

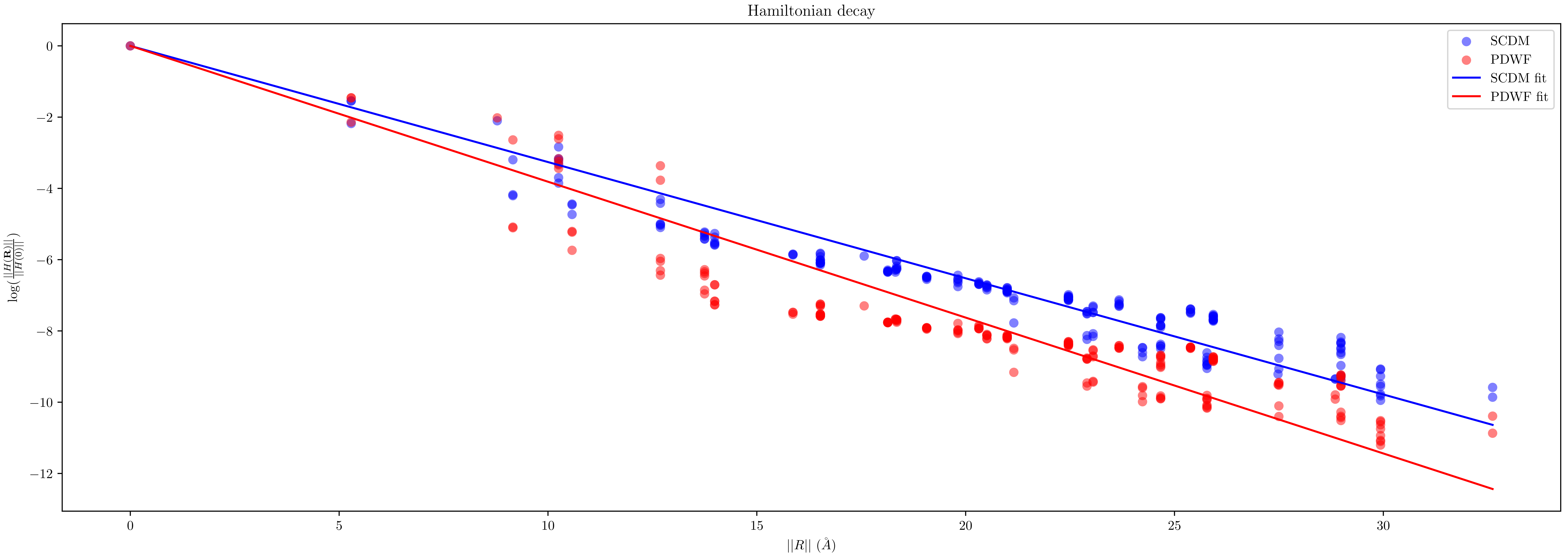

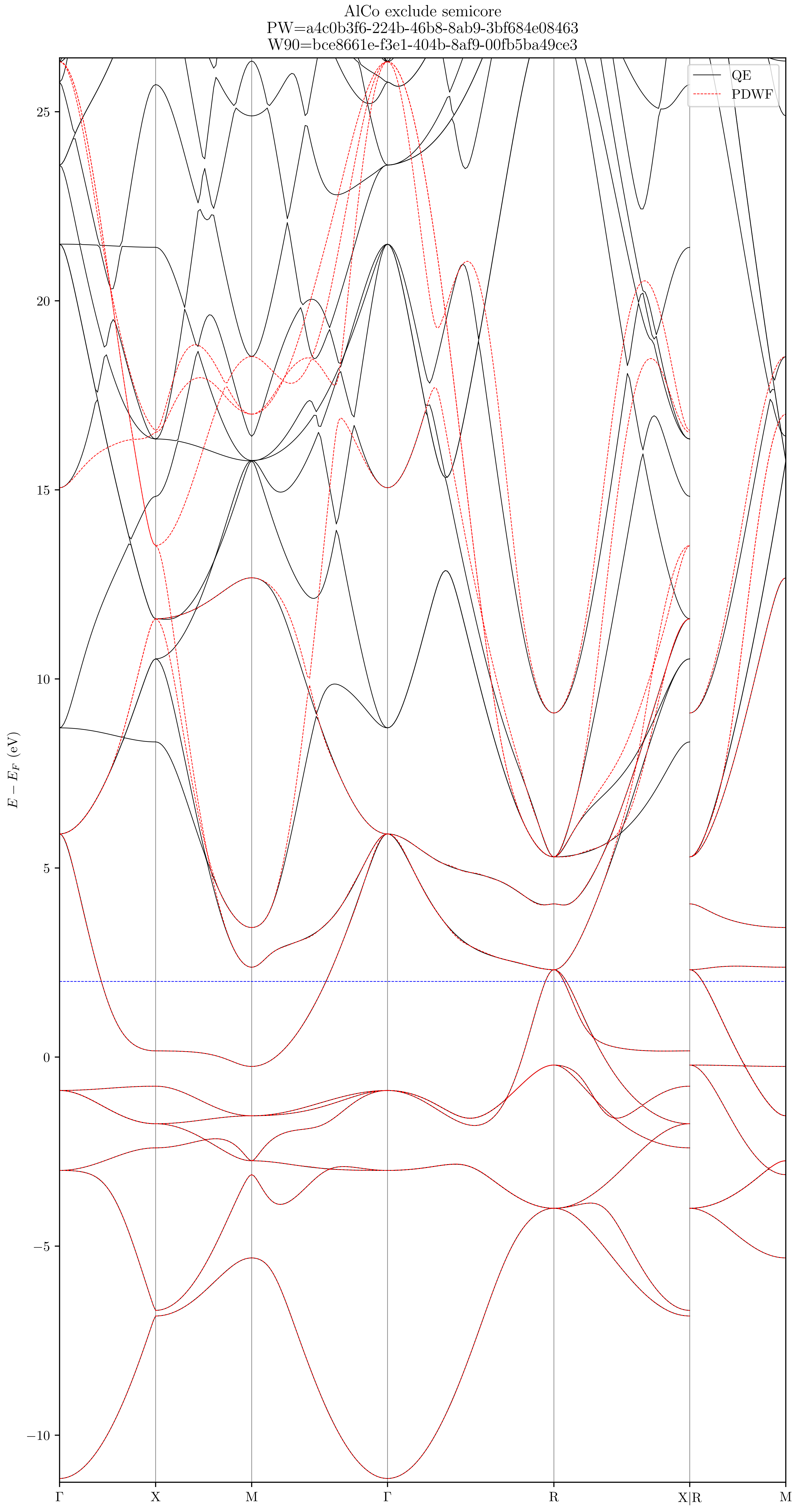

PDWF band distance (meV) and τ ($\AA$)

η_0	η_2	η_4	$\eta_0^{\max}$	$\eta_2^{\max}$	$\eta_4^{\max}$	τ
1.6	1.9	4.3	11.1	13.0	51.0	1.586

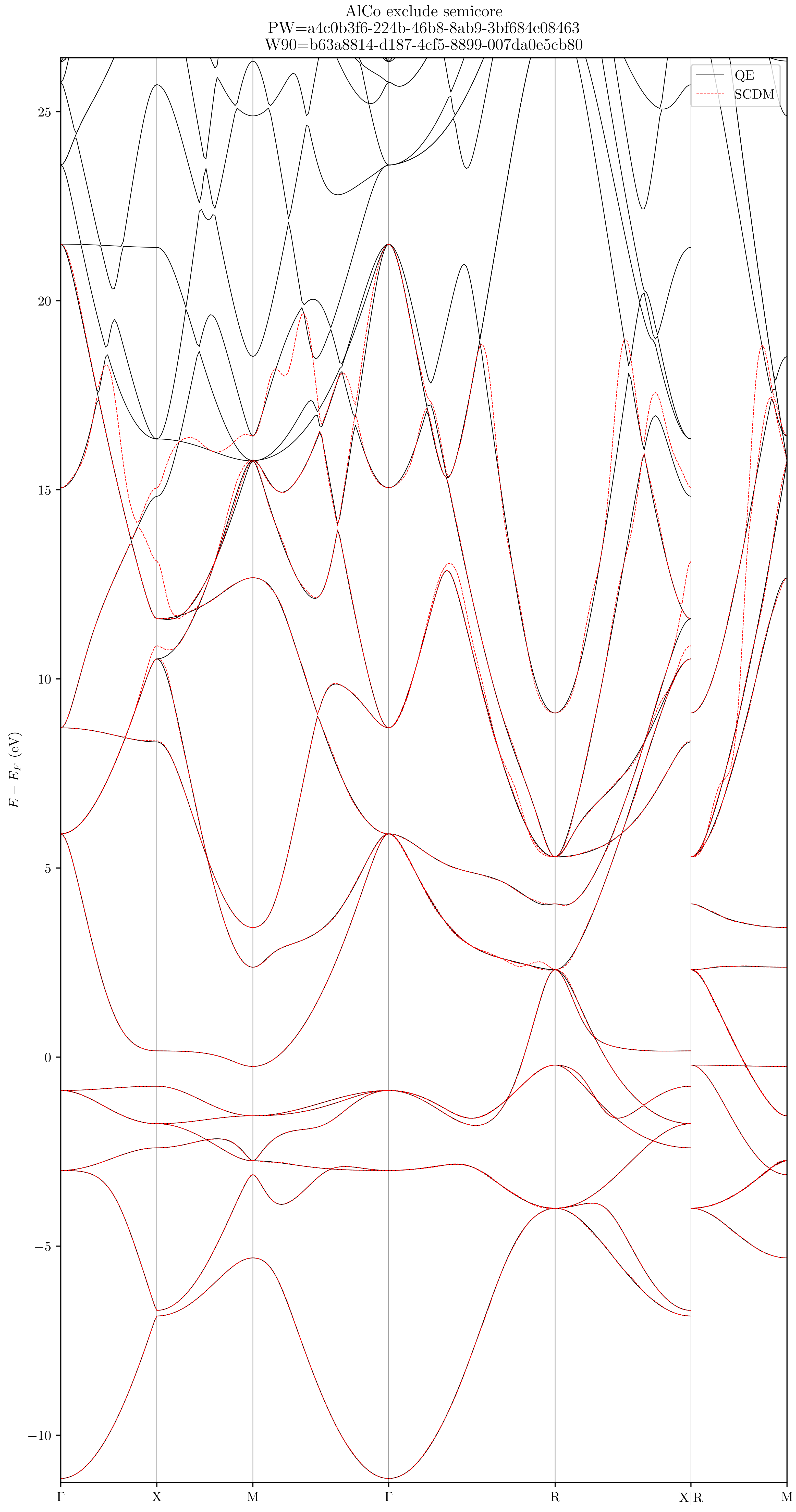

SCDM band distance (meV) and τ ($\AA$)

η_0	η_2	η_4	$\eta_0^{\max}$	$\eta_2^{\max}$	$\eta_4^{\max}$	τ
5.8	6.5	11.9	32.9	41.8	161.2	2.445

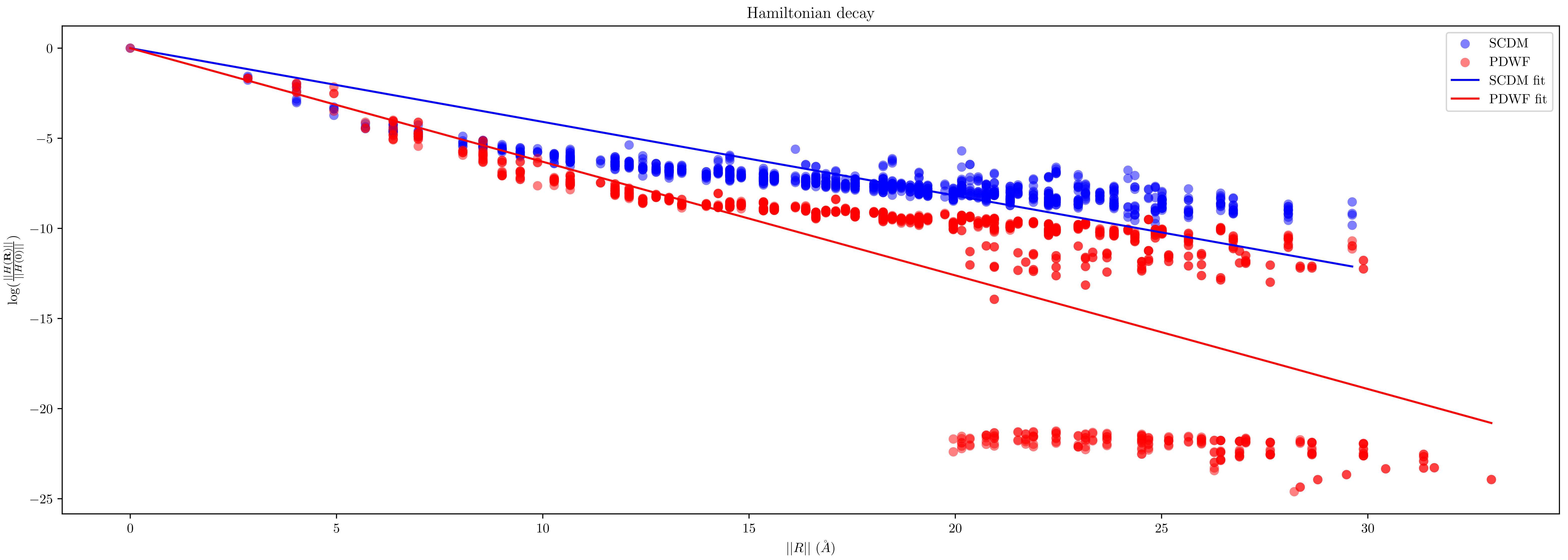

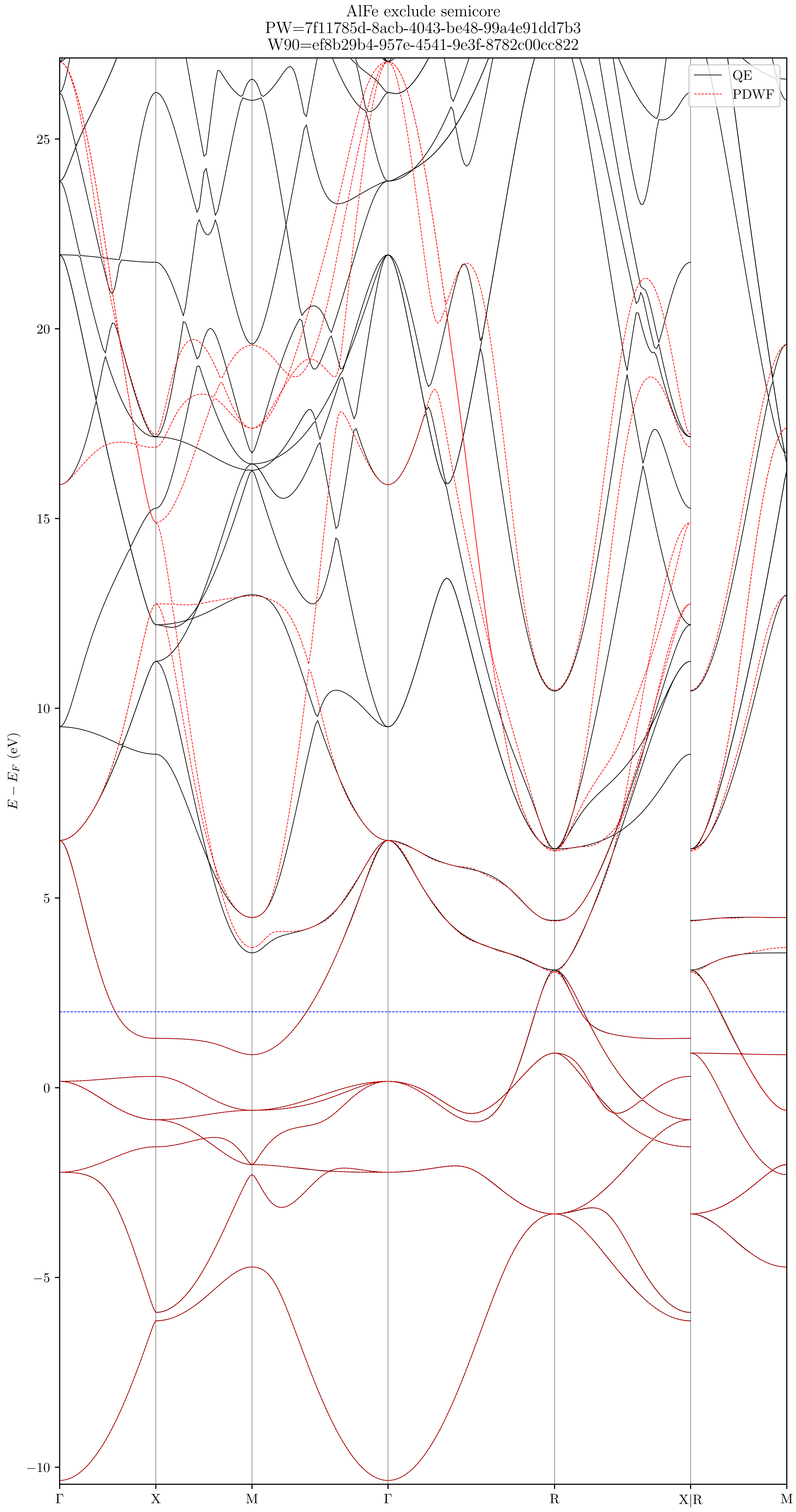

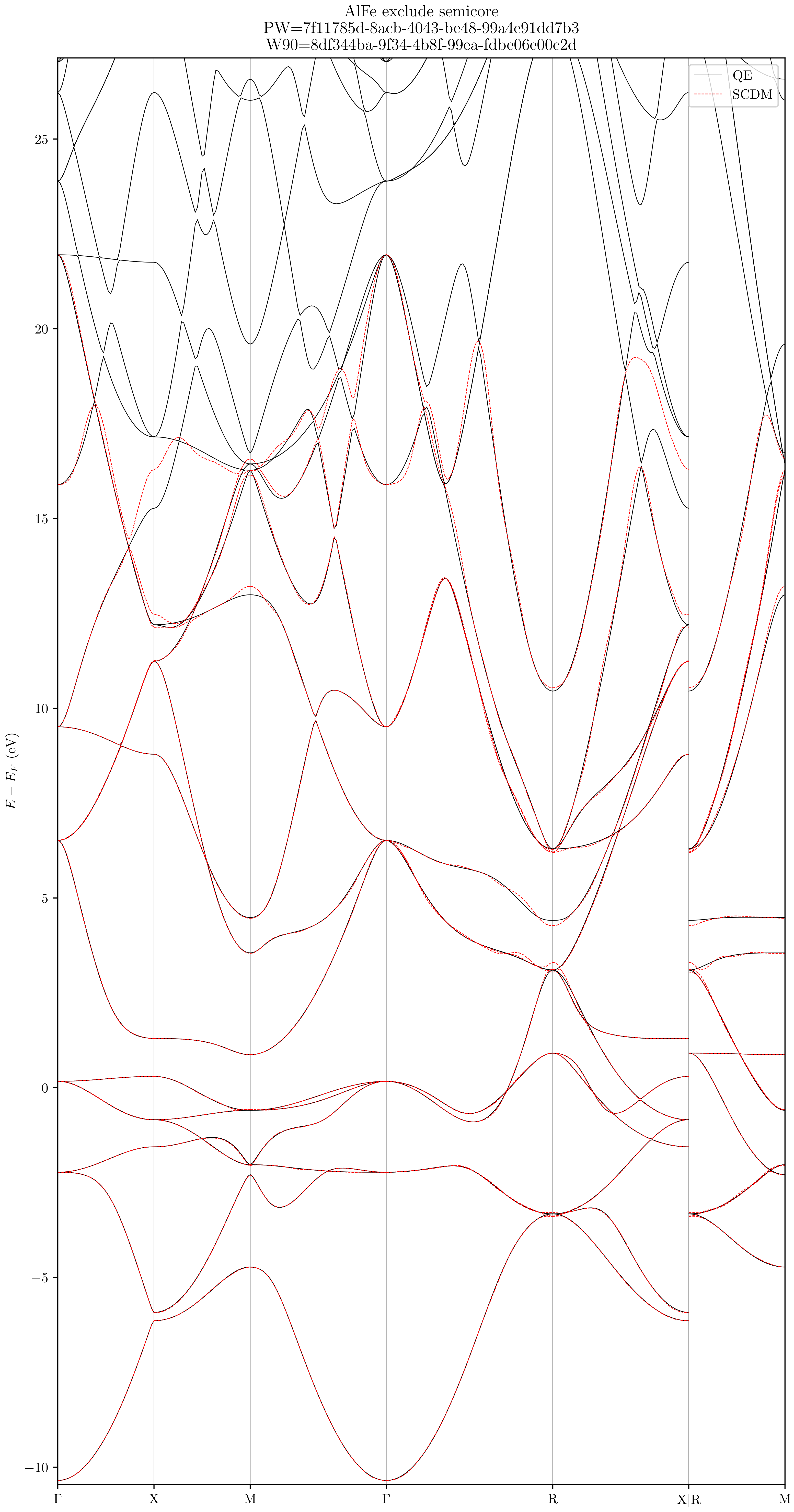

PDWF band distance (meV) and τ ($\AA$)

η_0	η_2	η_4	$\eta_0^{\max}$	$\eta_2^{\max}$	$\eta_4^{\max}$	τ
3.1	4.0	17.9	11.9	28.3	150.9	1.913

SCDM band distance (meV) and τ ($\AA$)

η_0	η_2	η_4	$\eta_0^{\max}$	$\eta_2^{\max}$	$\eta_4^{\max}$	τ
11.2	10.9	19.2	65.9	65.9	204.2	2.296

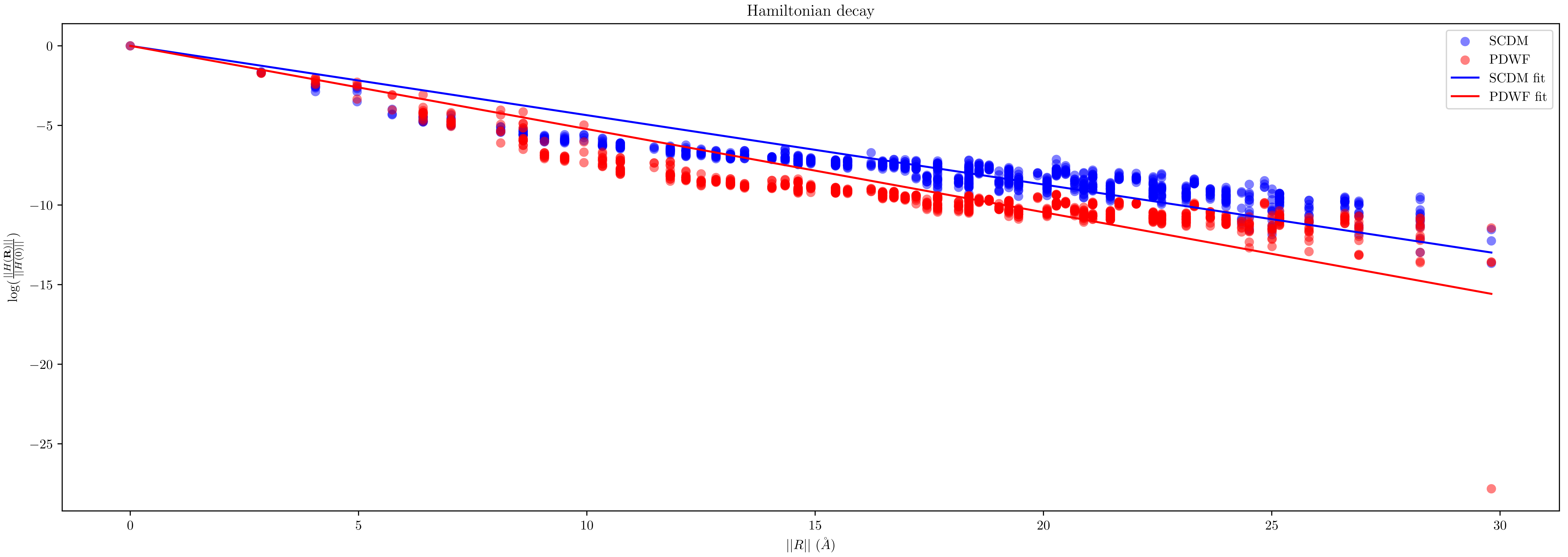

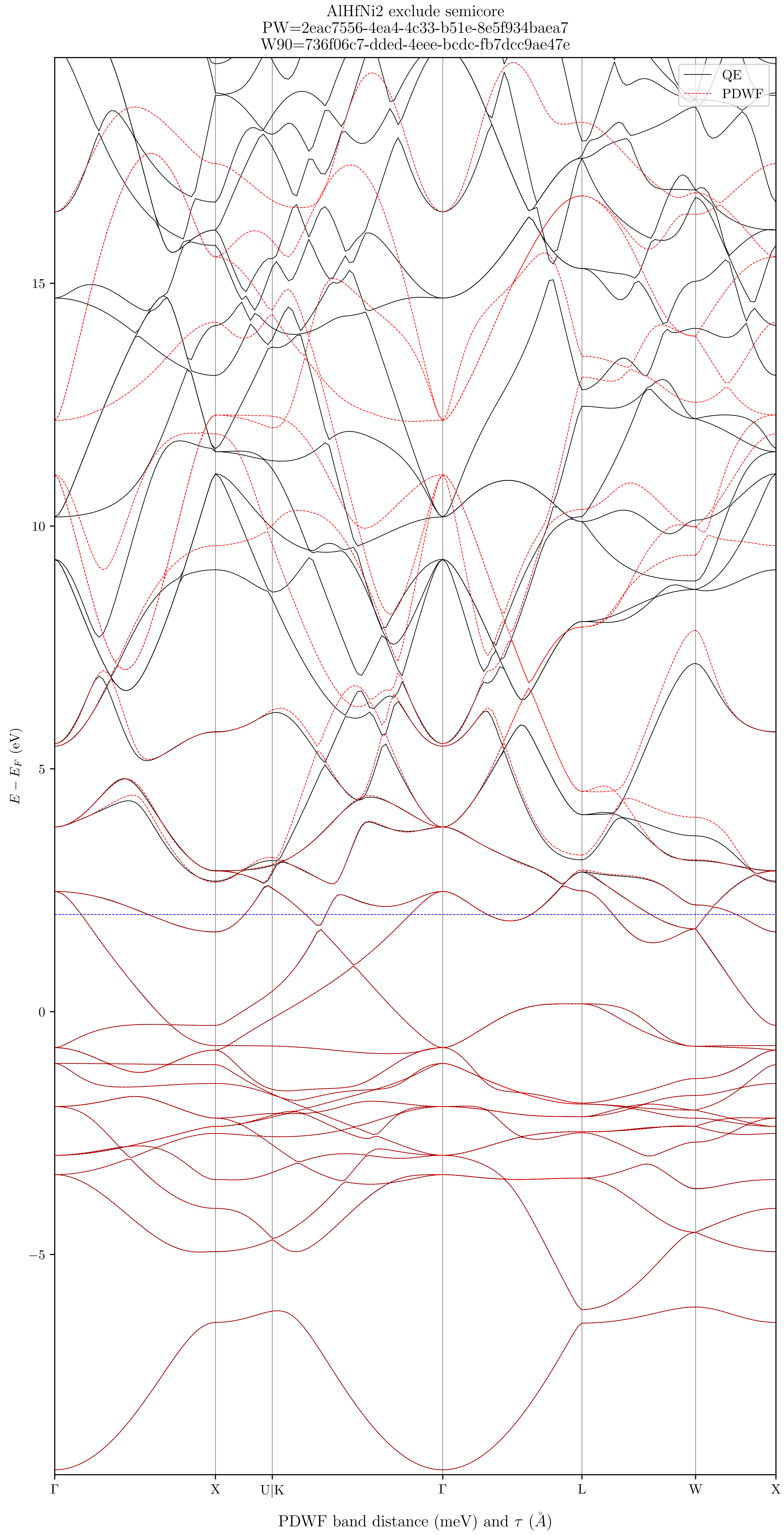

η_0	η_2	η_4	$\eta_0^{\max}$	$\eta_2^{\max}$	$\eta_4^{\max}$	τ
1.1	1.5	29.8	7.8	13.3	305.1	2.174

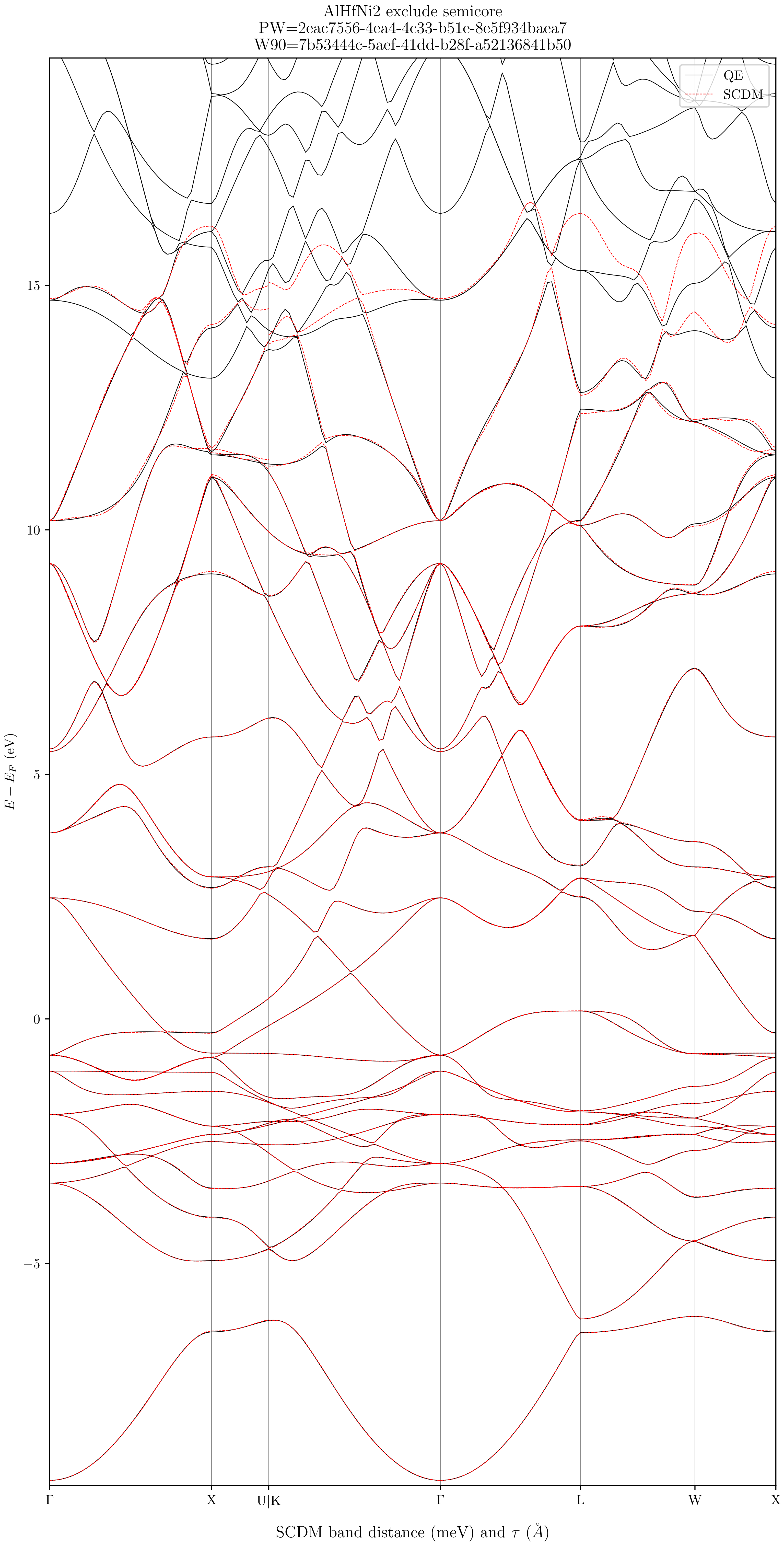

η_0	η_2	η_4	$\eta_0^{\max}$	$\eta_2^{\max}$	$\eta_4^{\max}$	τ
3.3	3.3	3.8	18.5	18.5	23.9	2.475

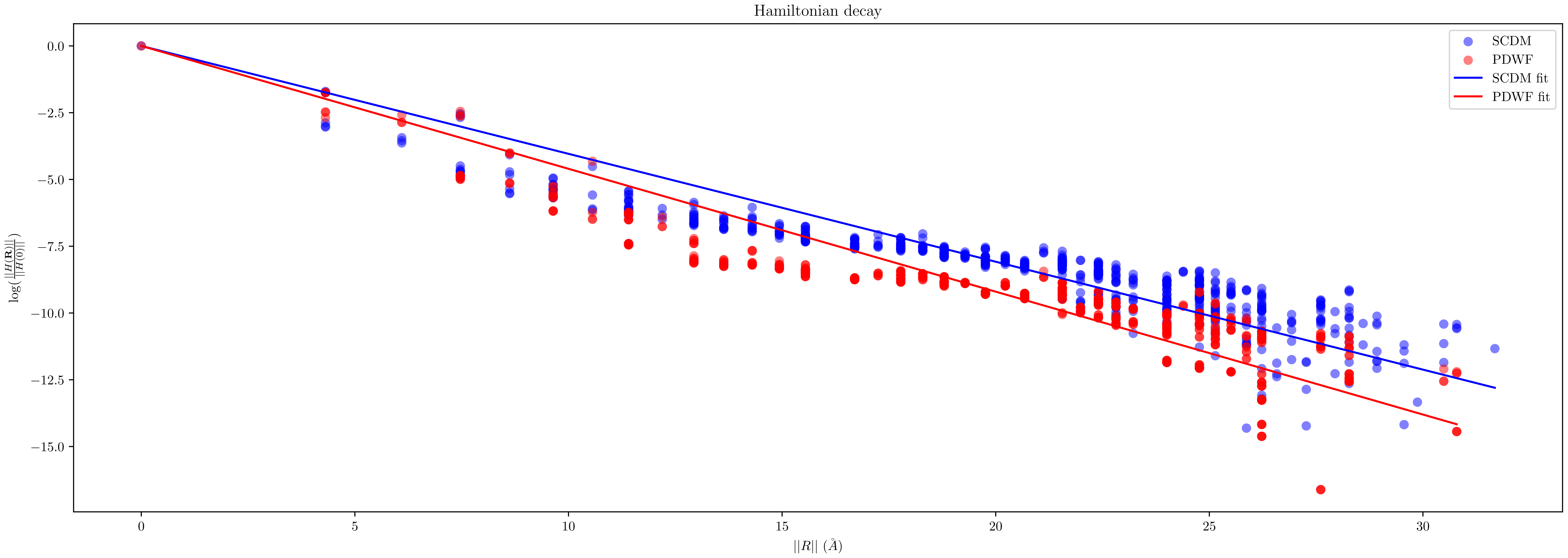

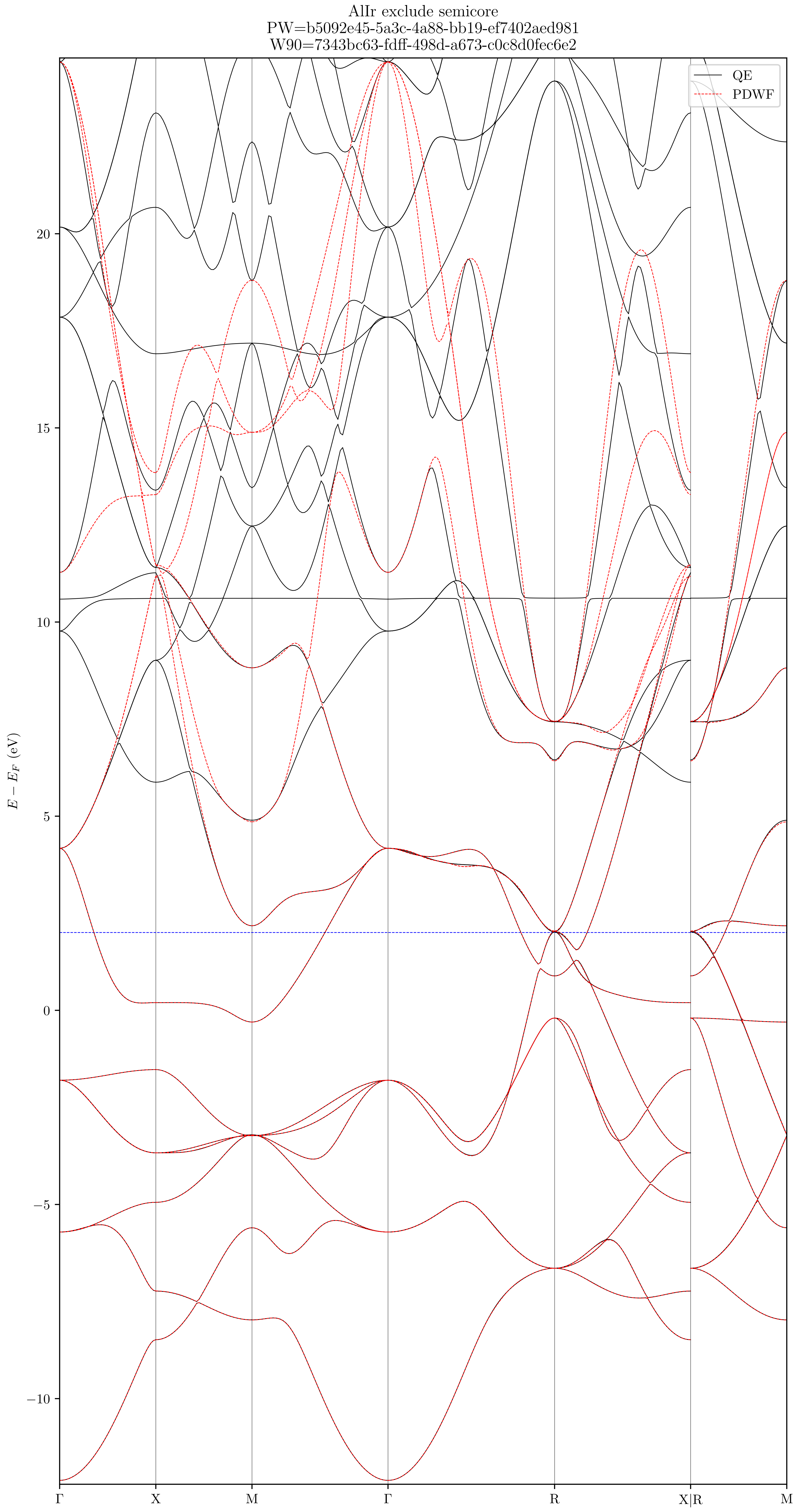

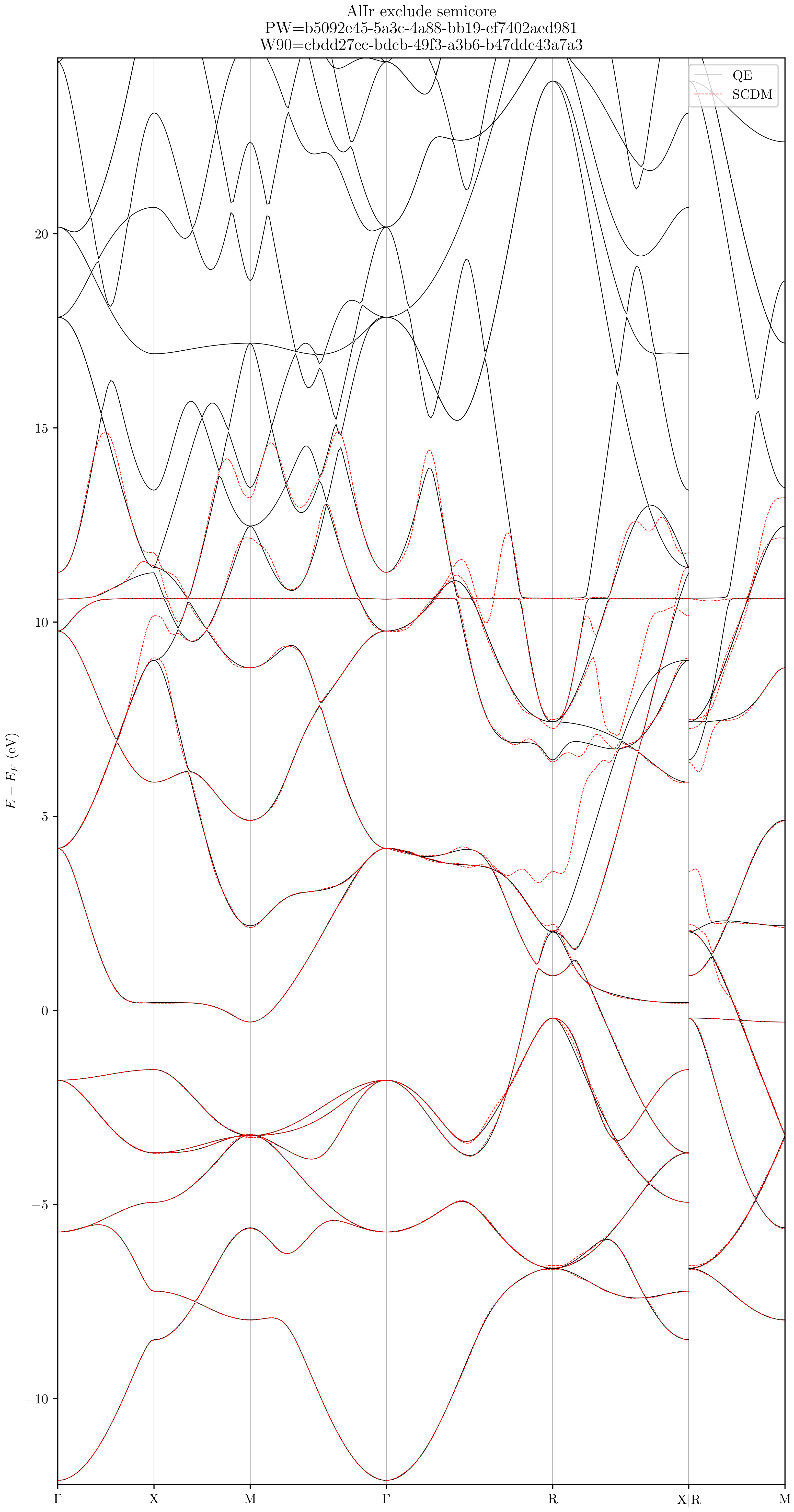

PDWF band distance (meV) and τ ($\AA$)

η_0	η_2	η_4	$\eta_0^{\max}$	$\eta_2^{\max}$	$\eta_4^{\max}$	τ
3.1	4.4	6.2	17.1	24.1	47.4	1.973

SCDM band distance (meV) and τ ($\AA$)

η_0	η_2	η_4	$\eta_0^{\max}$	$\eta_2^{\max}$	$\eta_4^{\max}$	τ
20.9	27.1	140.1	173.9	274.6	1564.9	2.330

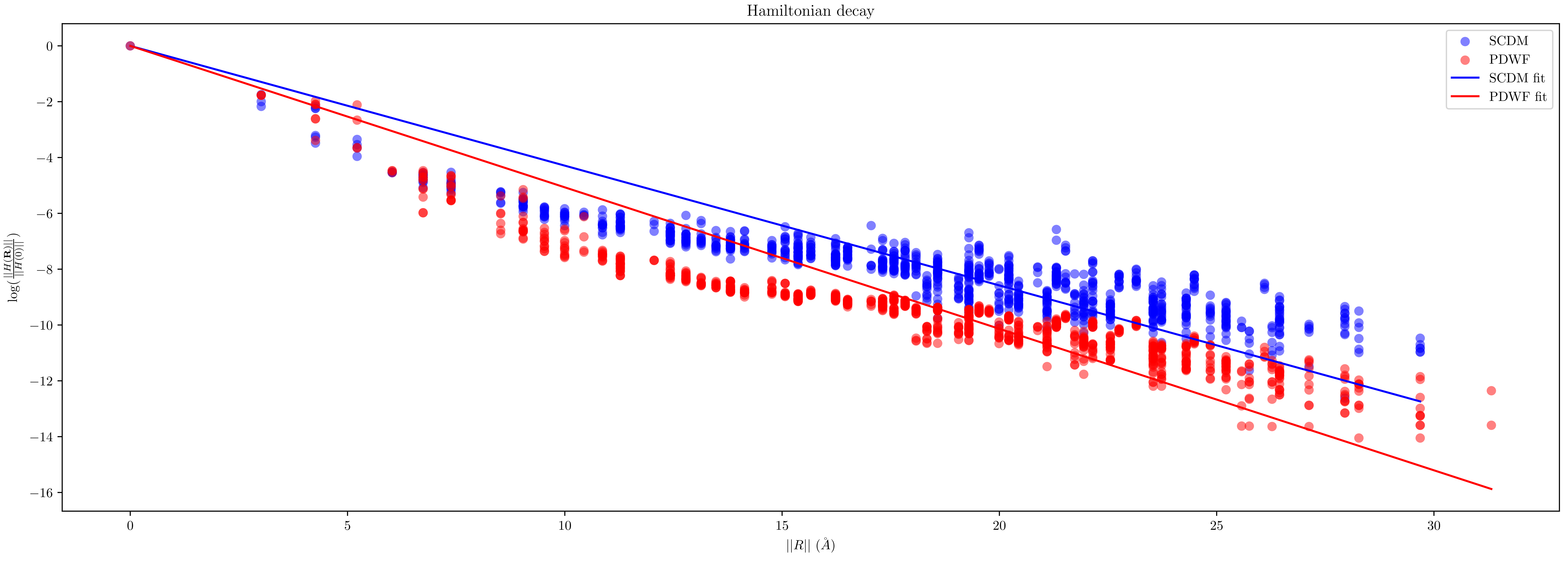

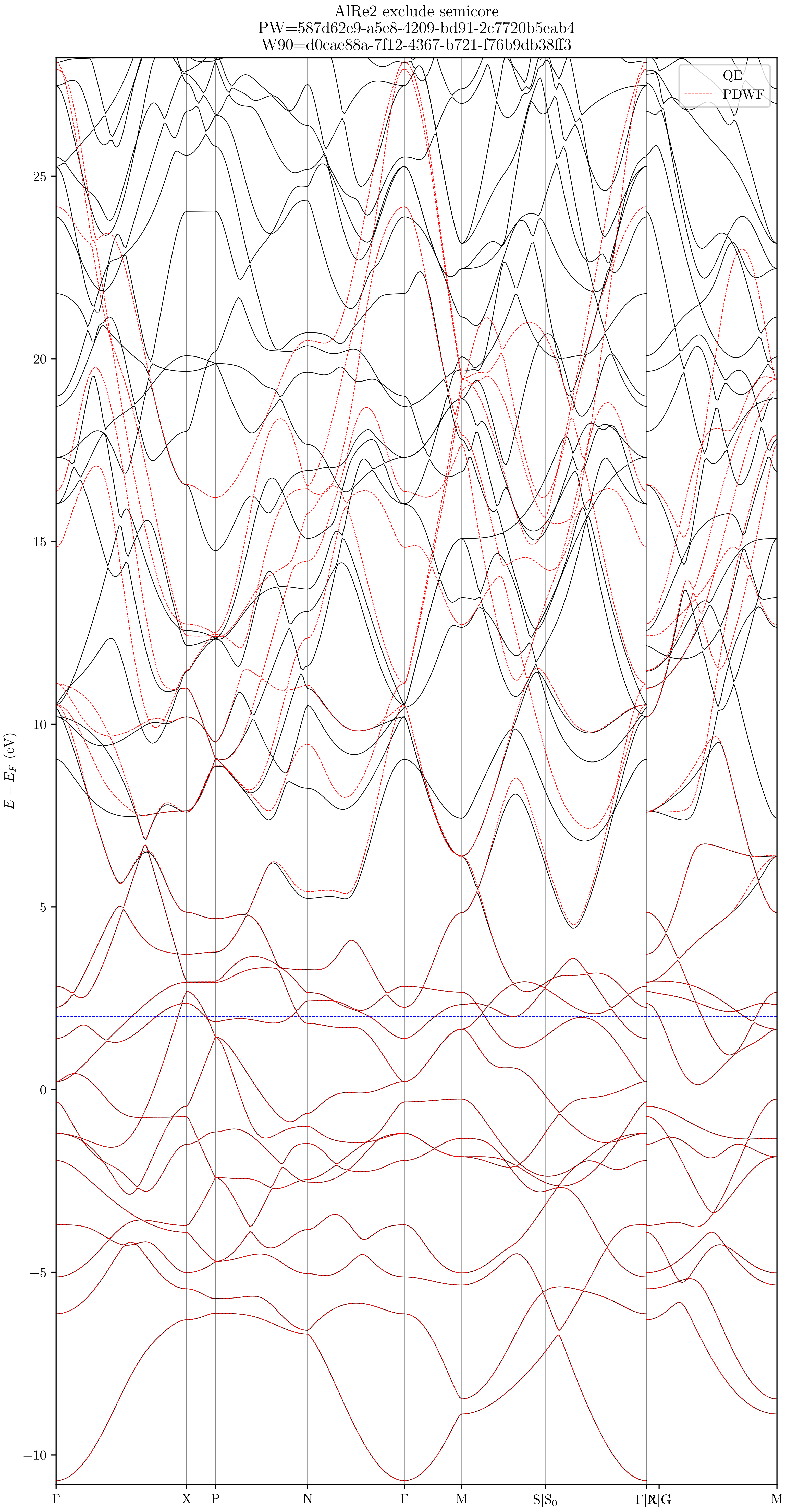

PDWF band distance (meV) and τ ($\AA$)

η_0	η_2	η_4	$\eta_0^{\max}$	$\eta_2^{\max}$	$\eta_4^{\max}$	τ
1.5	1.6	1.8	8.0	8.0	17.4	2.460

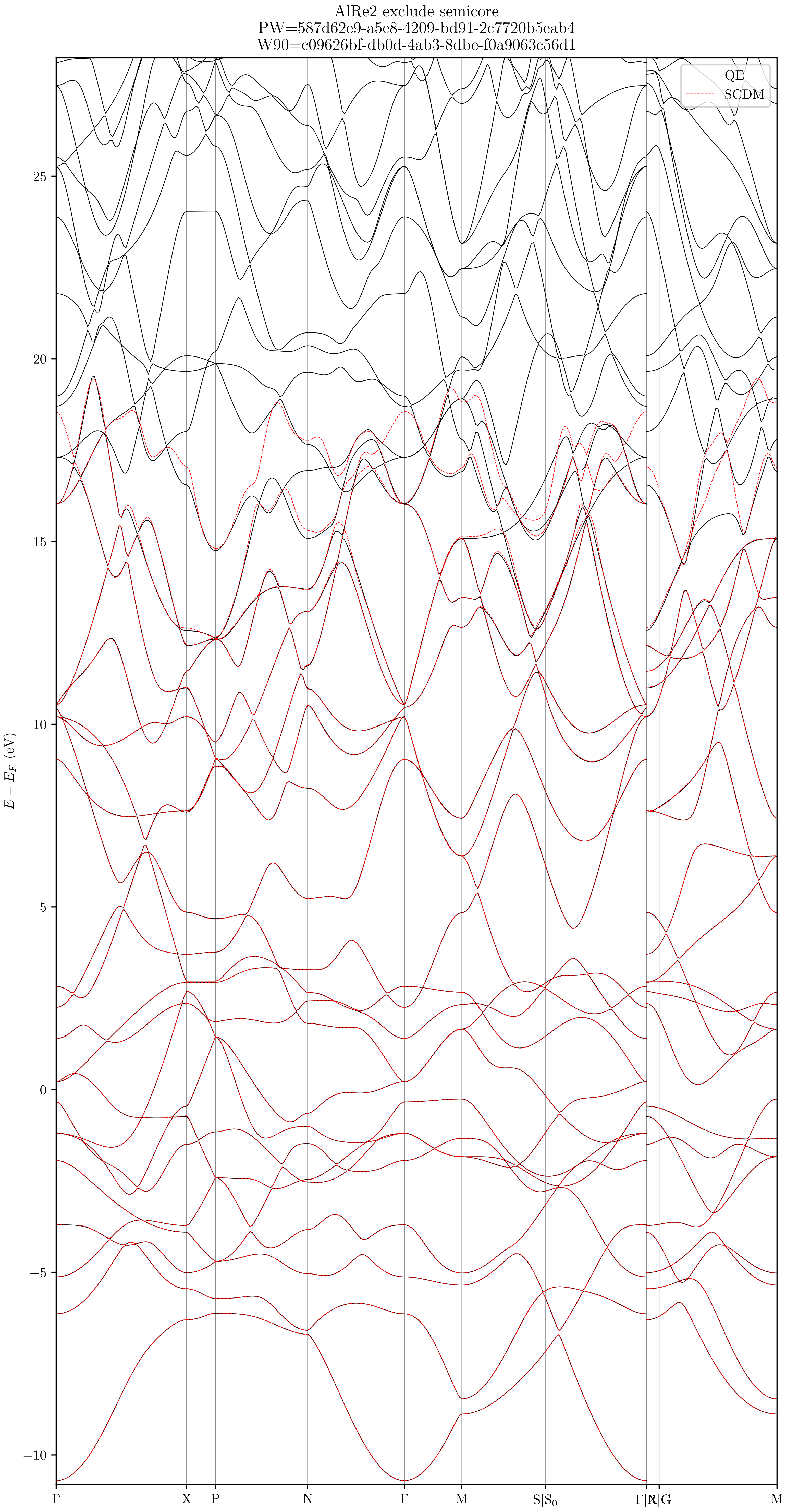

SCDM band distance (meV) and τ ($\AA$)

η_0	η_2	η_4	$\eta_0^{\max}$	$\eta_2^{\max}$	$\eta_4^{\max}$	τ
2.7	2.7	2.6	17.9	17.9	17.9	2.781

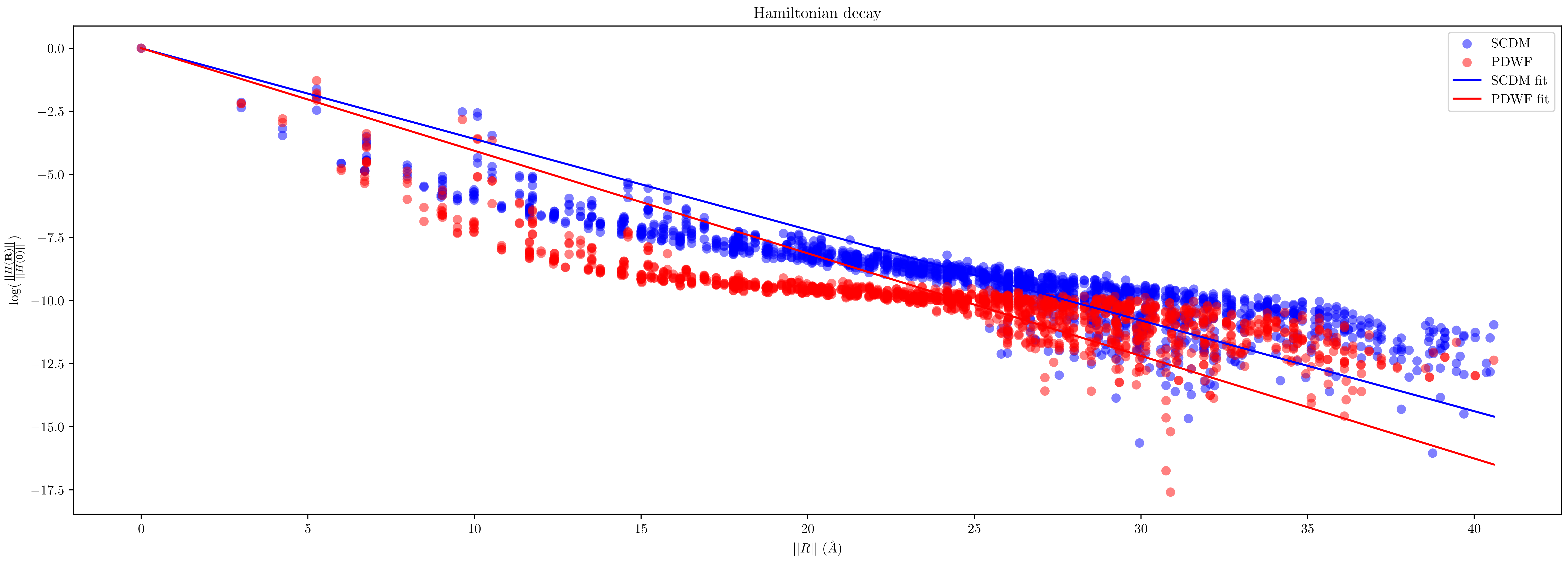

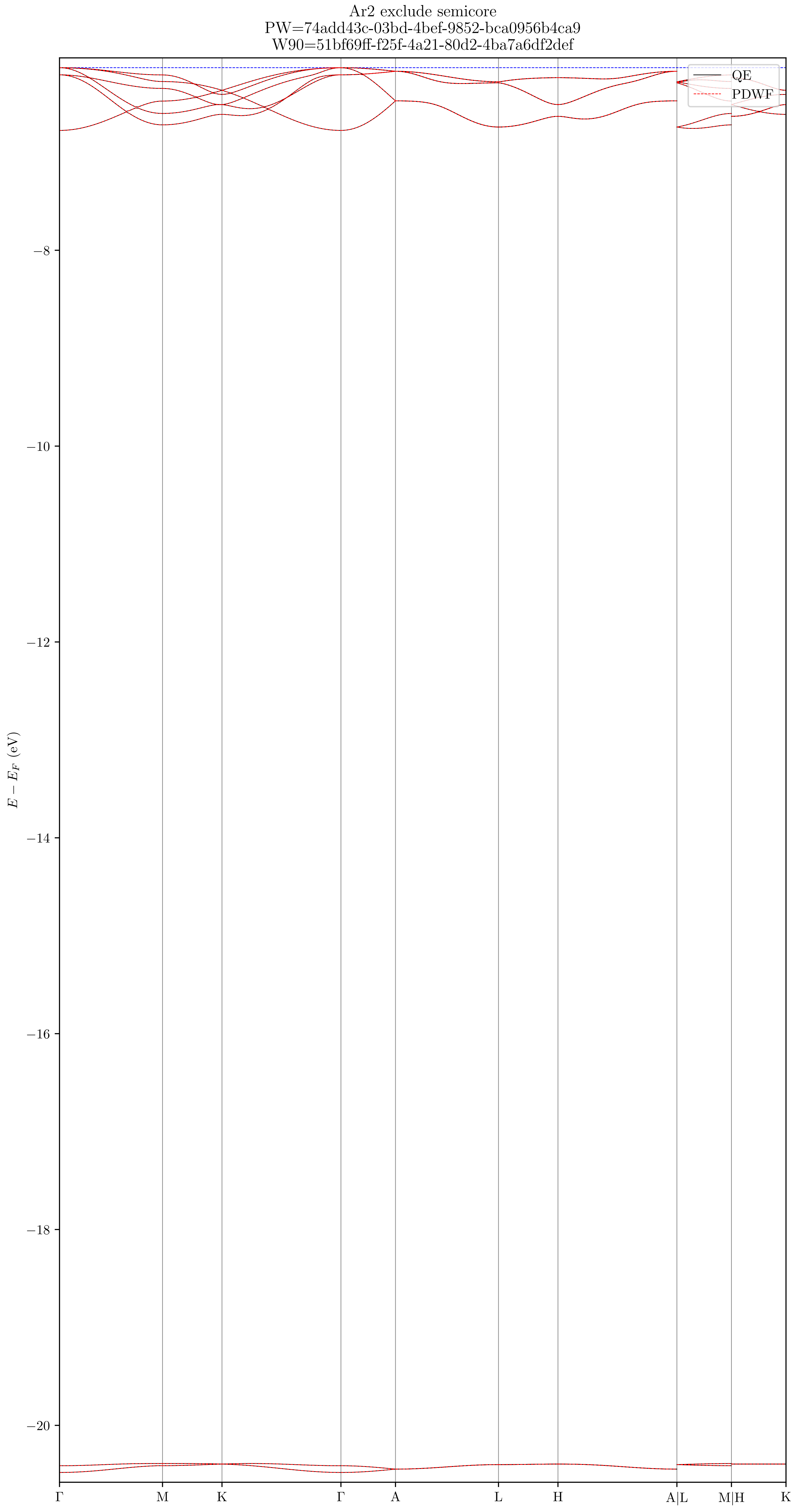

PDWF band distance (meV) and τ ($\AA$)

η_0	η_2	η_4	$\eta_0^{\max}$	$\eta_2^{\max}$	$\eta_4^{\max}$	τ
0.0	0.0	0.0	0.0	0.0	0.0	1.214

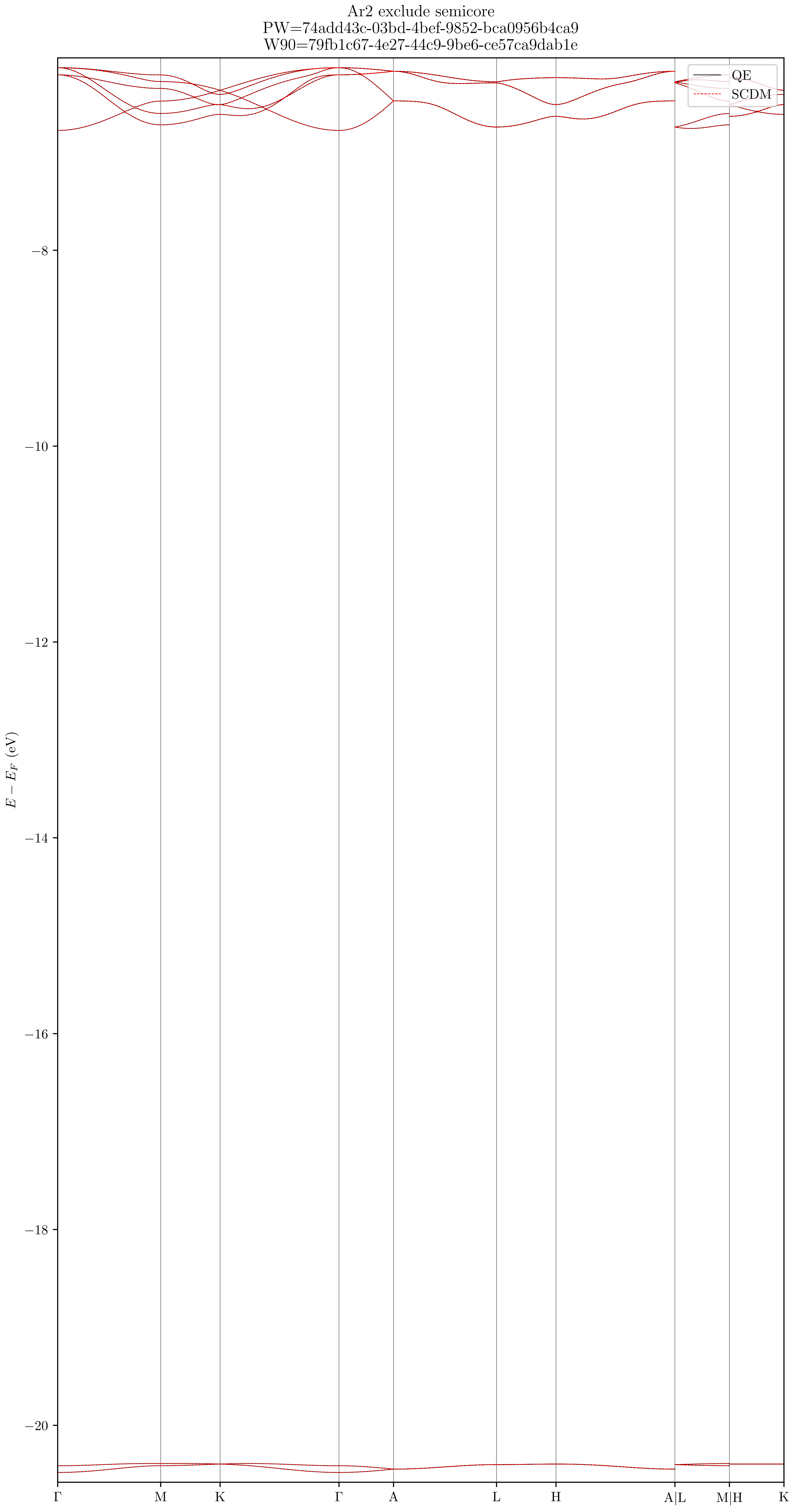

SCDM band distance (meV) and τ ($\AA$)

η_0	η_2	η_4	$\eta_0^{\max}$	$\eta_2^{\max}$	$\eta_4^{\max}$	τ
0.0	0.0	0.0	0.0	0.0	0.0	1.279

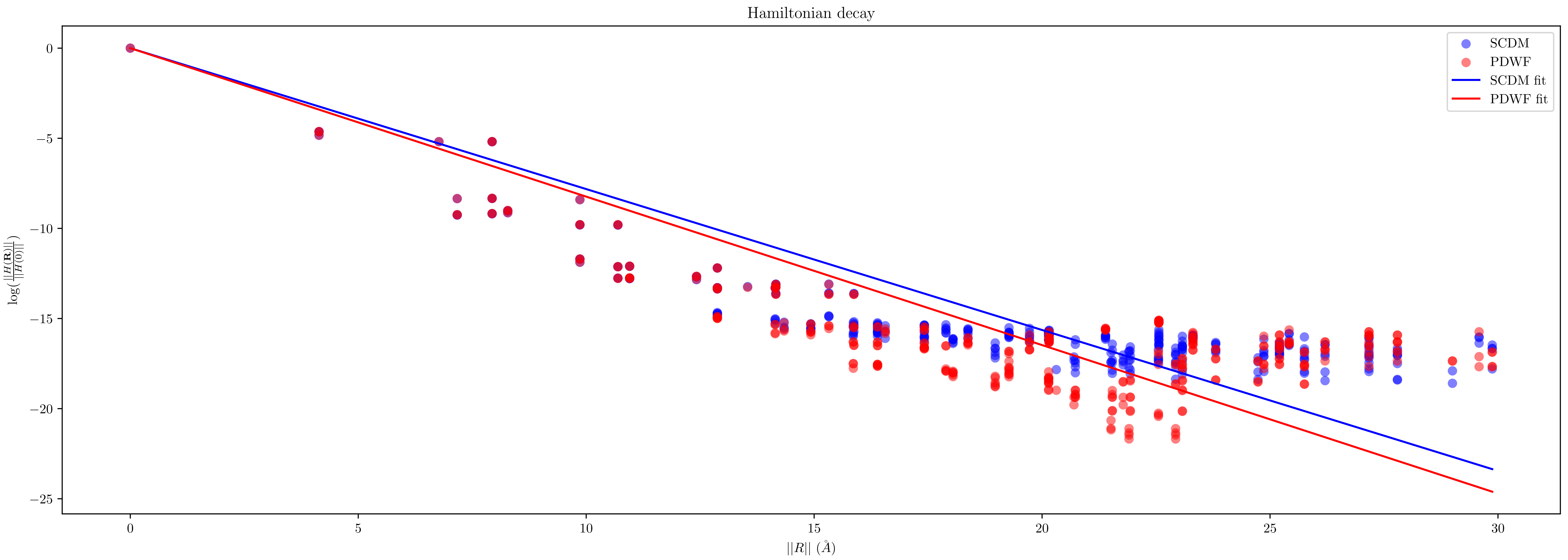

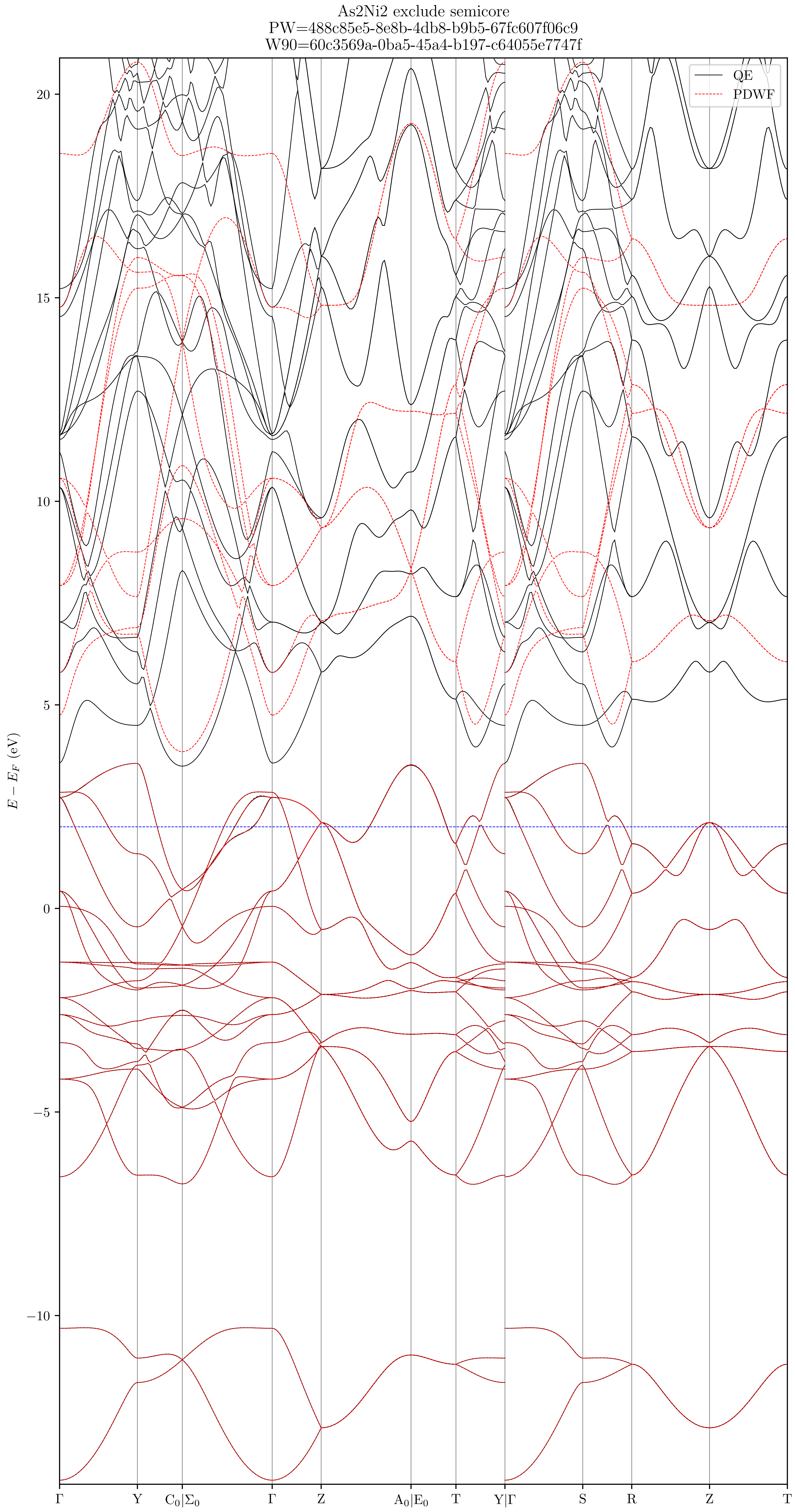

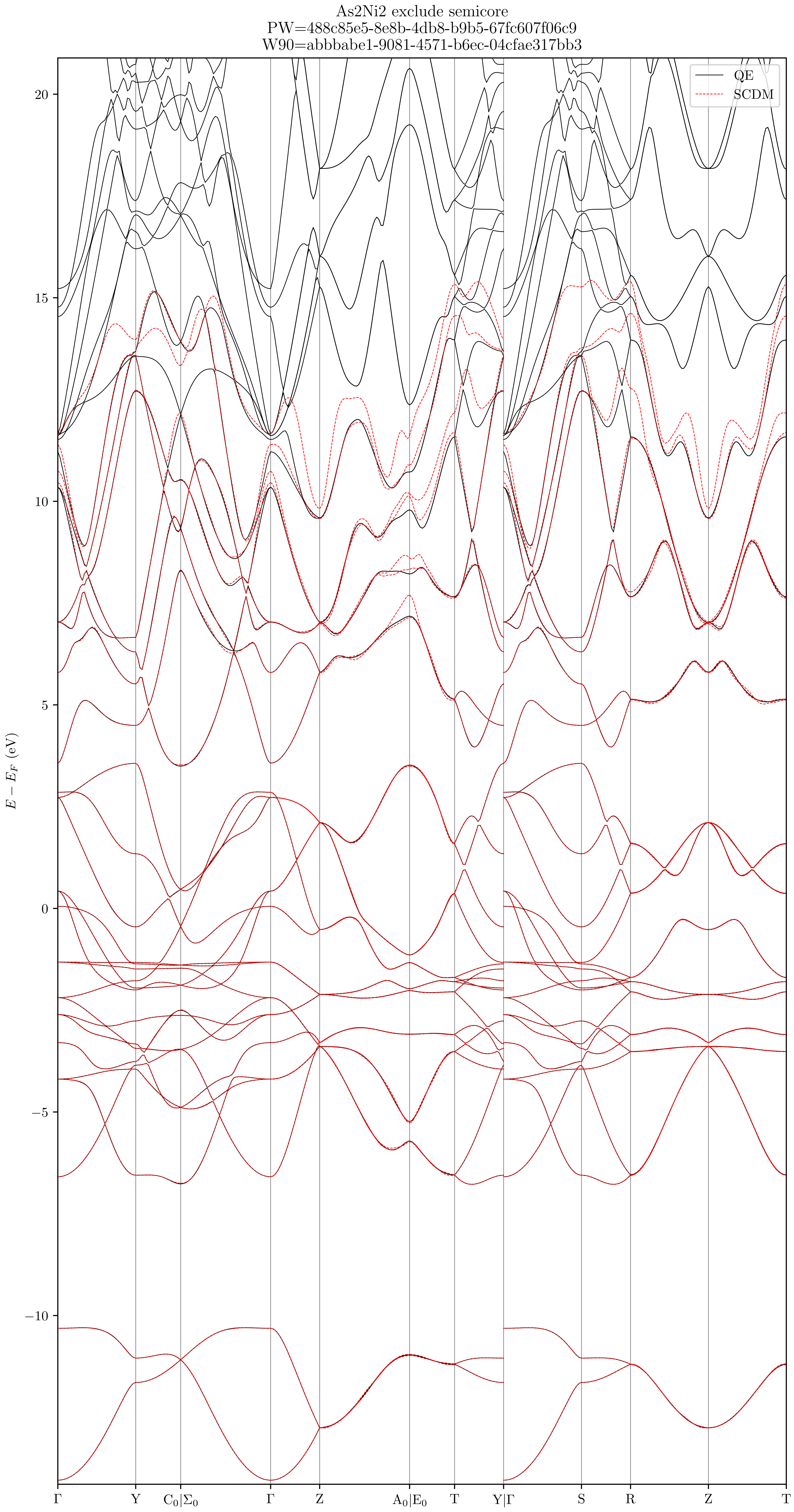

PDWF band distance (meV) and τ ($\AA$)

η_0	η_2	η_4	$\eta_0^{\max}$	$\eta_2^{\max}$	$\eta_4^{\max}$	τ
1.2	1.5	19.5	9.8	11.9	320.3	2.065

SCDM band distance (meV) and τ ($\AA$)

η_0	η_2	η_4	$\eta_0^{\max}$	$\eta_2^{\max}$	$\eta_4^{\max}$	τ
5.3	5.7	5.9	51.6	51.6	51.6	2.585

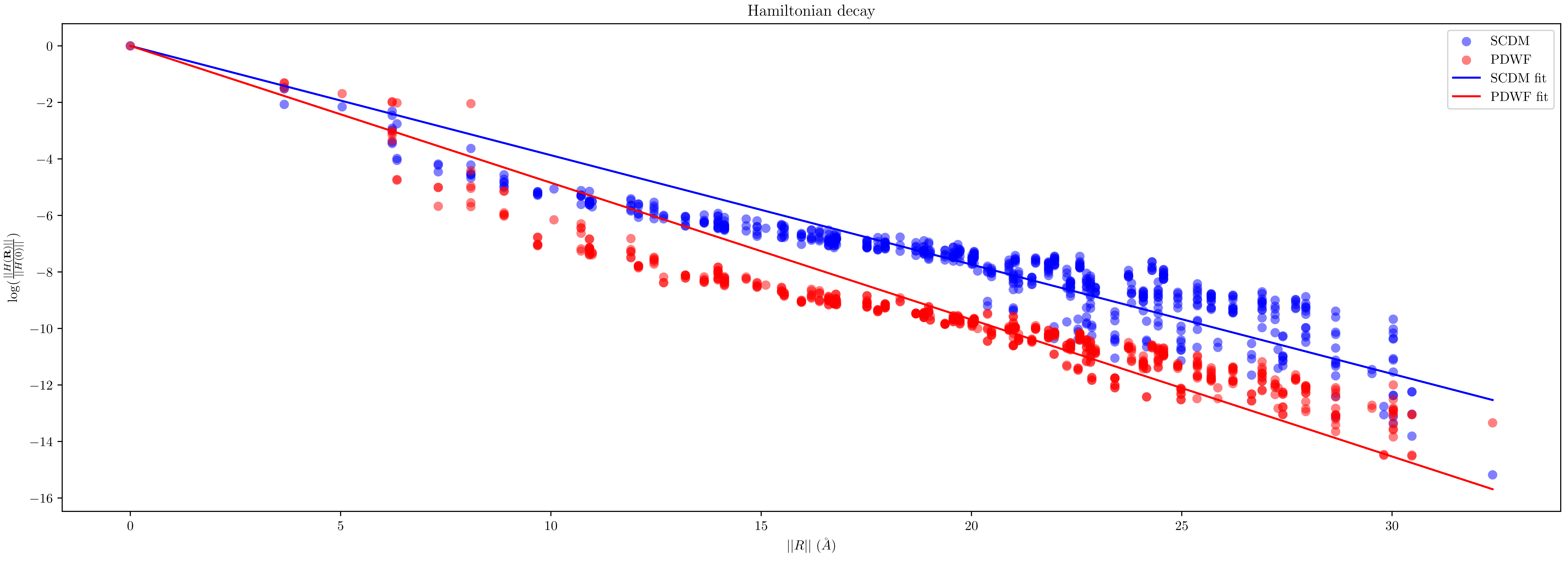

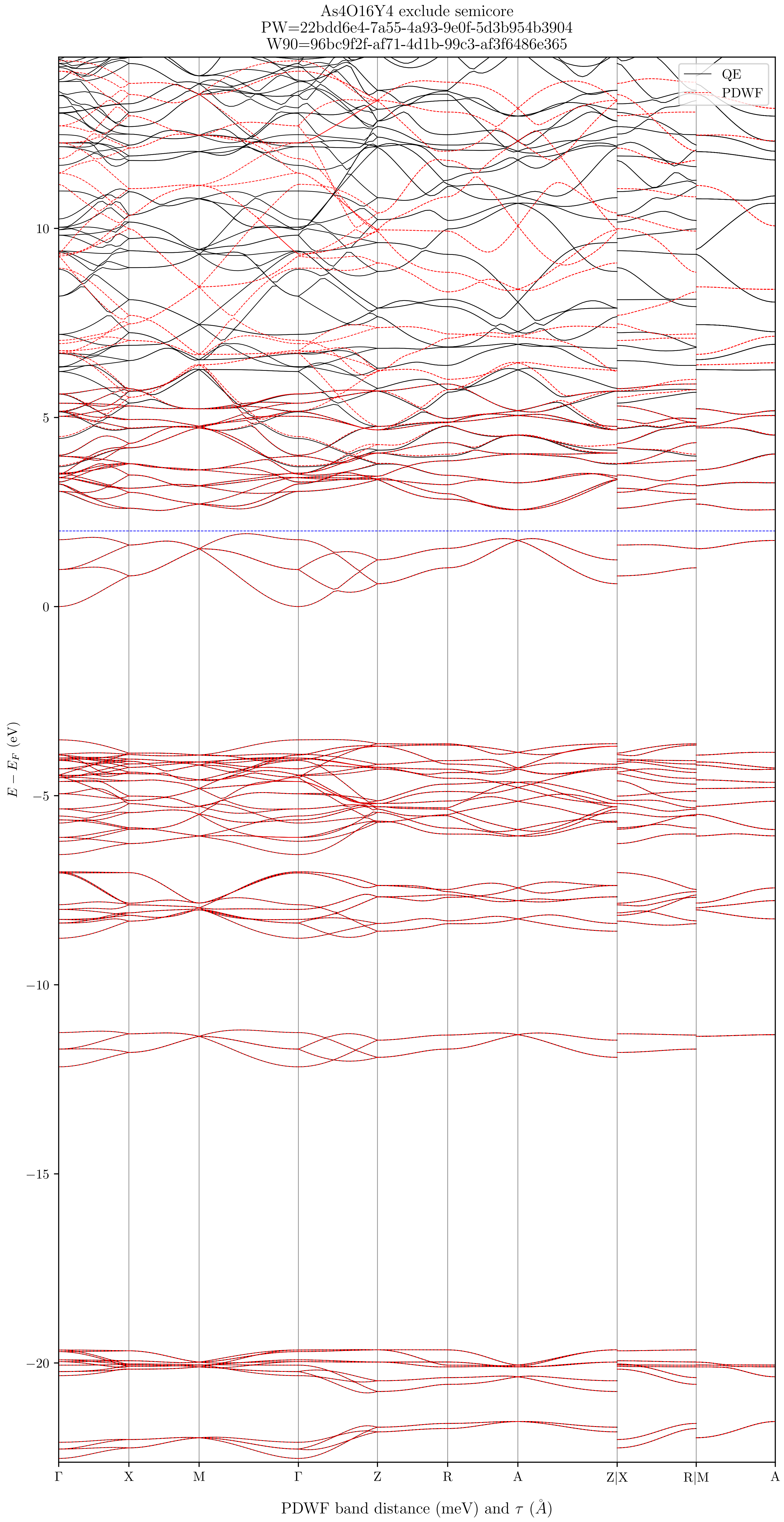

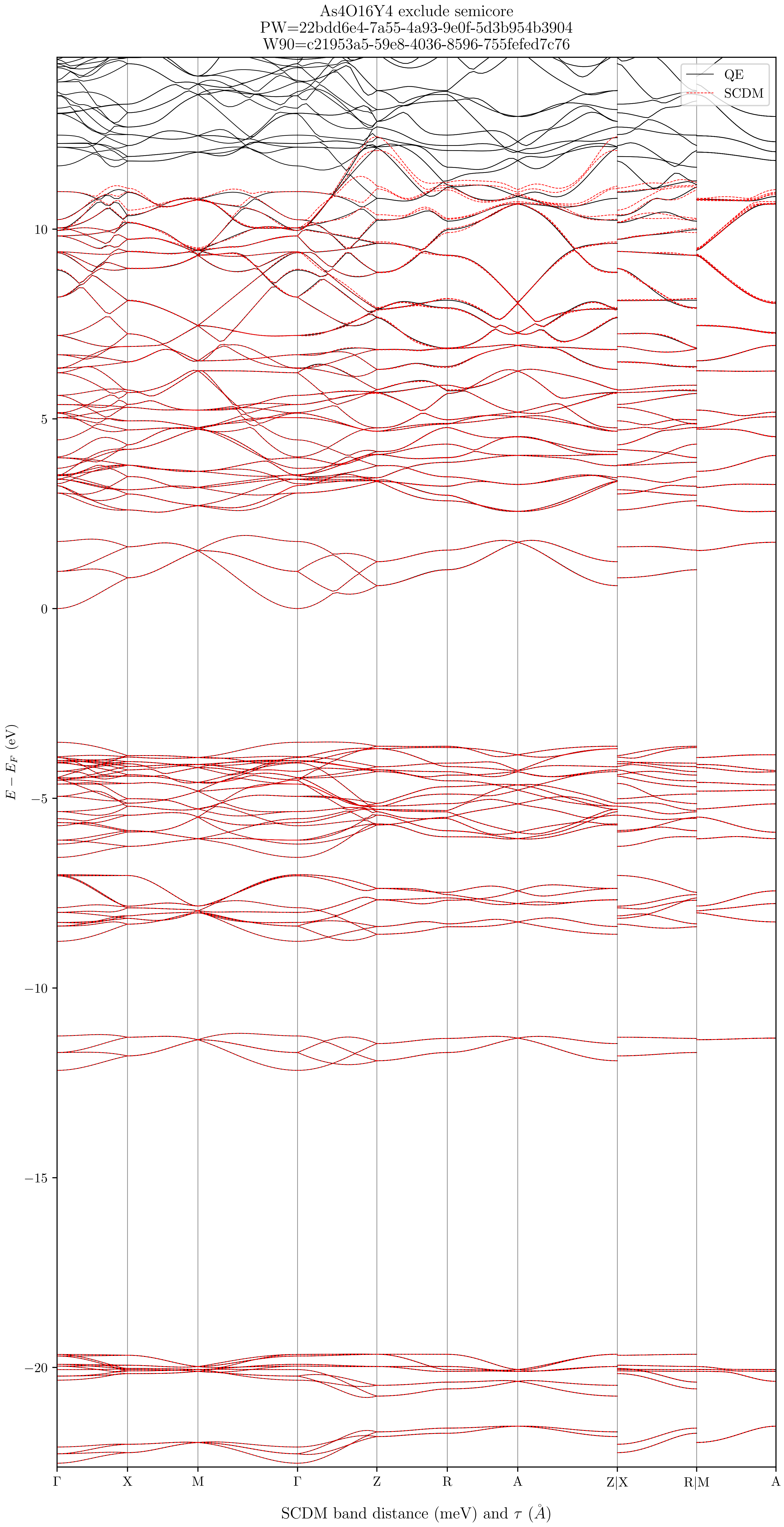

η_0	η_2	η_4	$\eta_0^{\max}$	$\eta_2^{\max}$	$\eta_4^{\max}$	τ
0.5	0.5	0.9	4.7	4.7	9.9	2.726

η_0	η_2	η_4	$\eta_0^{\max}$	$\eta_2^{\max}$	$\eta_4^{\max}$	τ
1.2	1.2	1.4	9.8	9.8	10.0	3.050

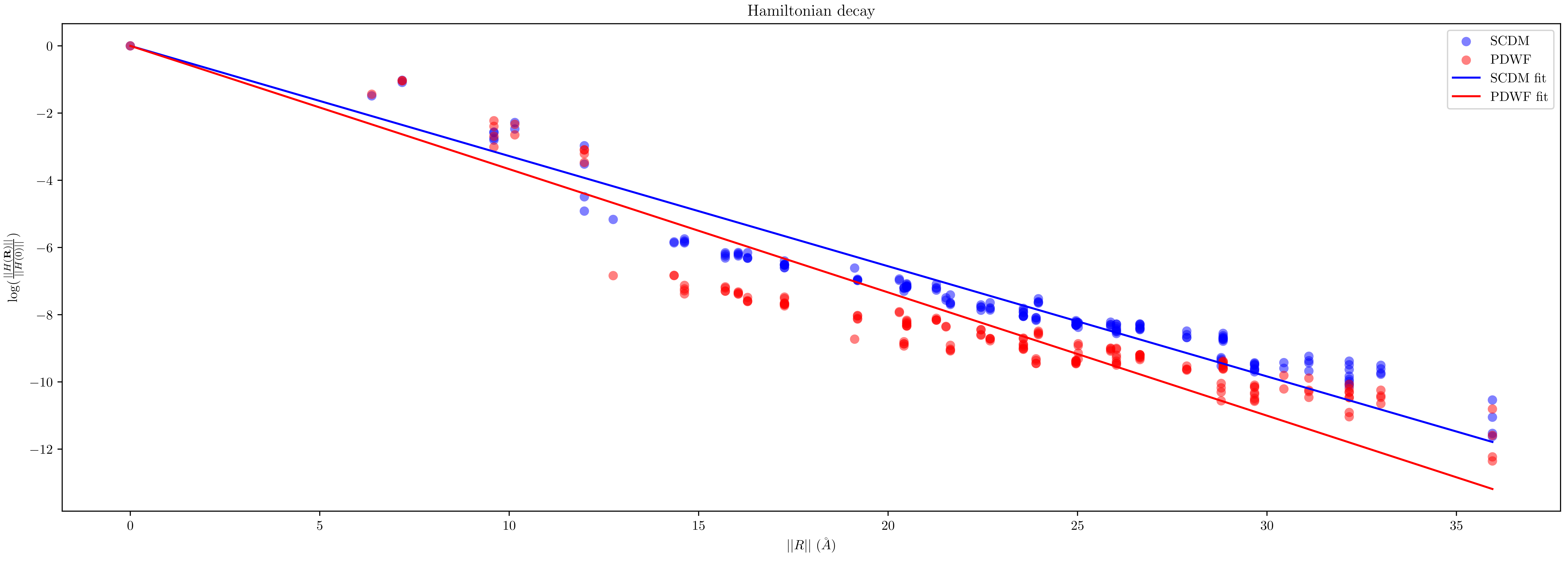

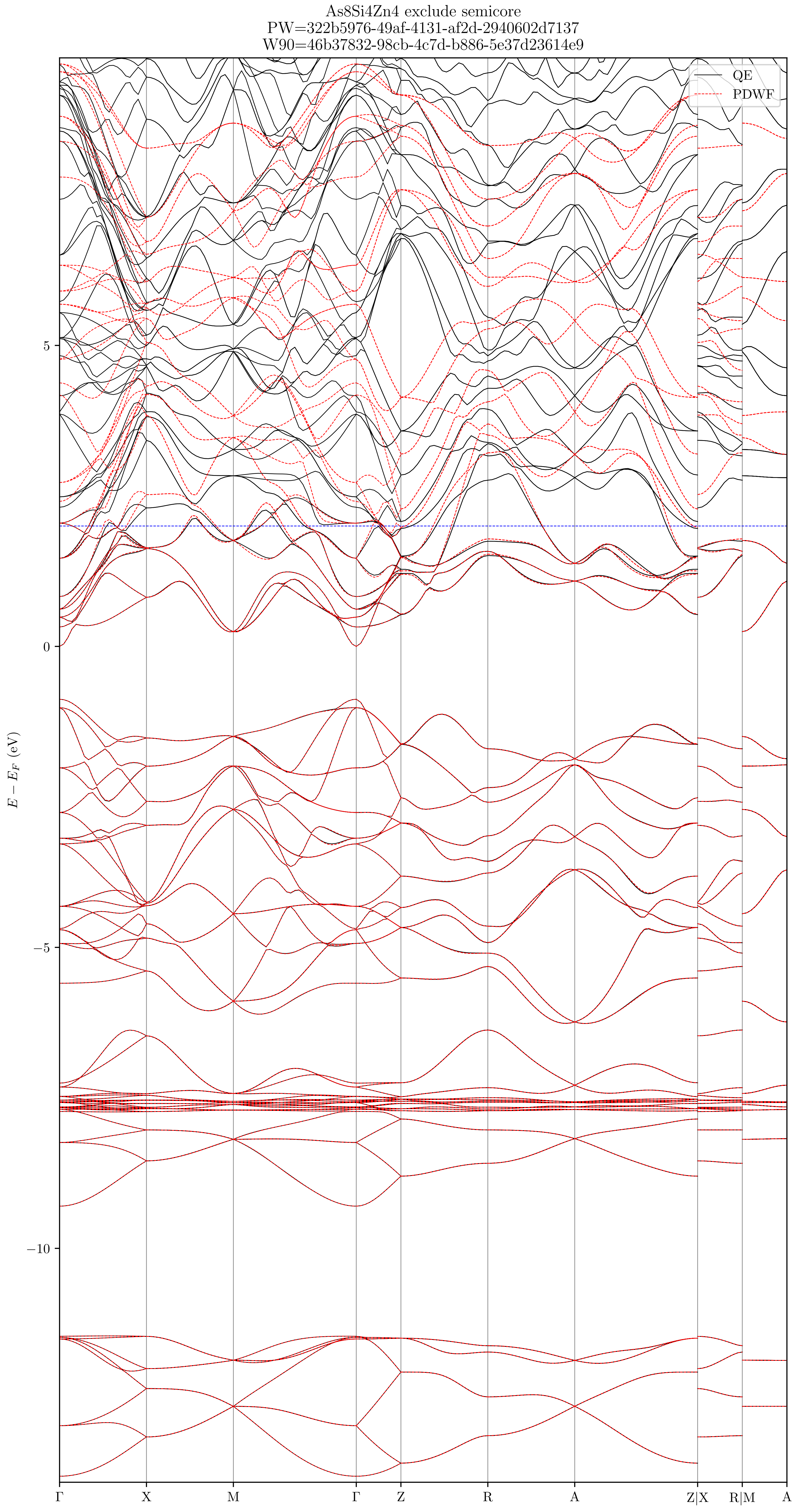

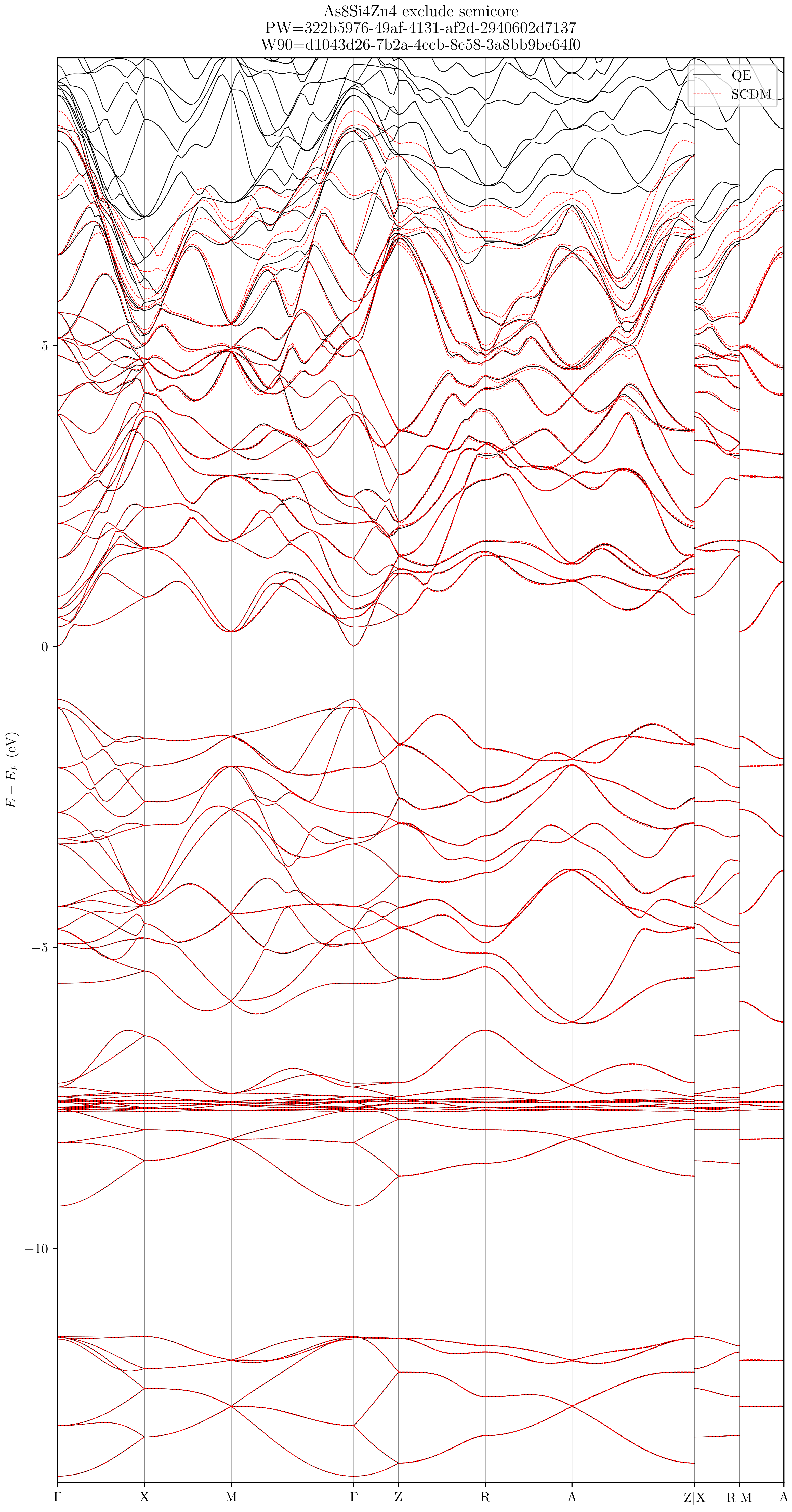

PDWF band distance (meV) and τ ($\AA$)

η_0	η_2	η_4	$\eta_0^{\max}$	$\eta_2^{\max}$	$\eta_4^{\max}$	τ
1.8	4.8	105.6	18.8	54.5	591.3	3.046

SCDM band distance (meV) and τ ($\AA$)

η_0	η_2	η_4	$\eta_0^{\max}$	$\eta_2^{\max}$	$\eta_4^{\max}$	τ
3.6	4.4	6.0	25.7	28.0	56.2	3.609

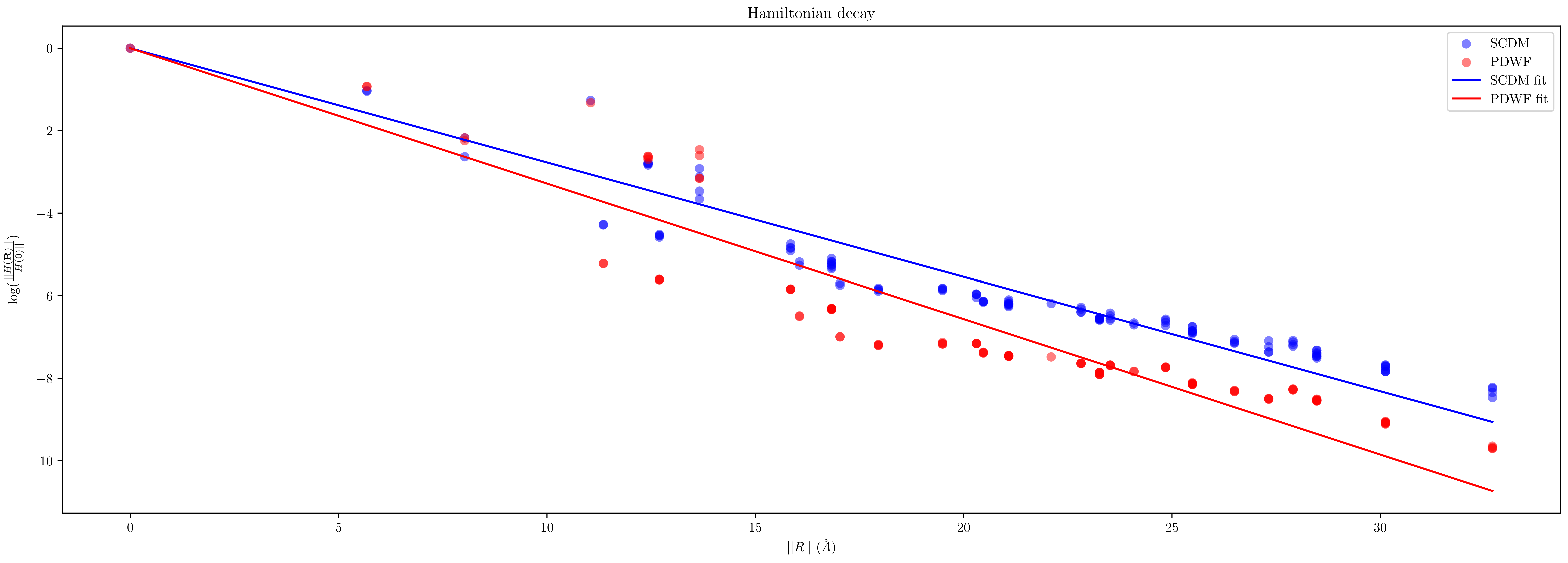

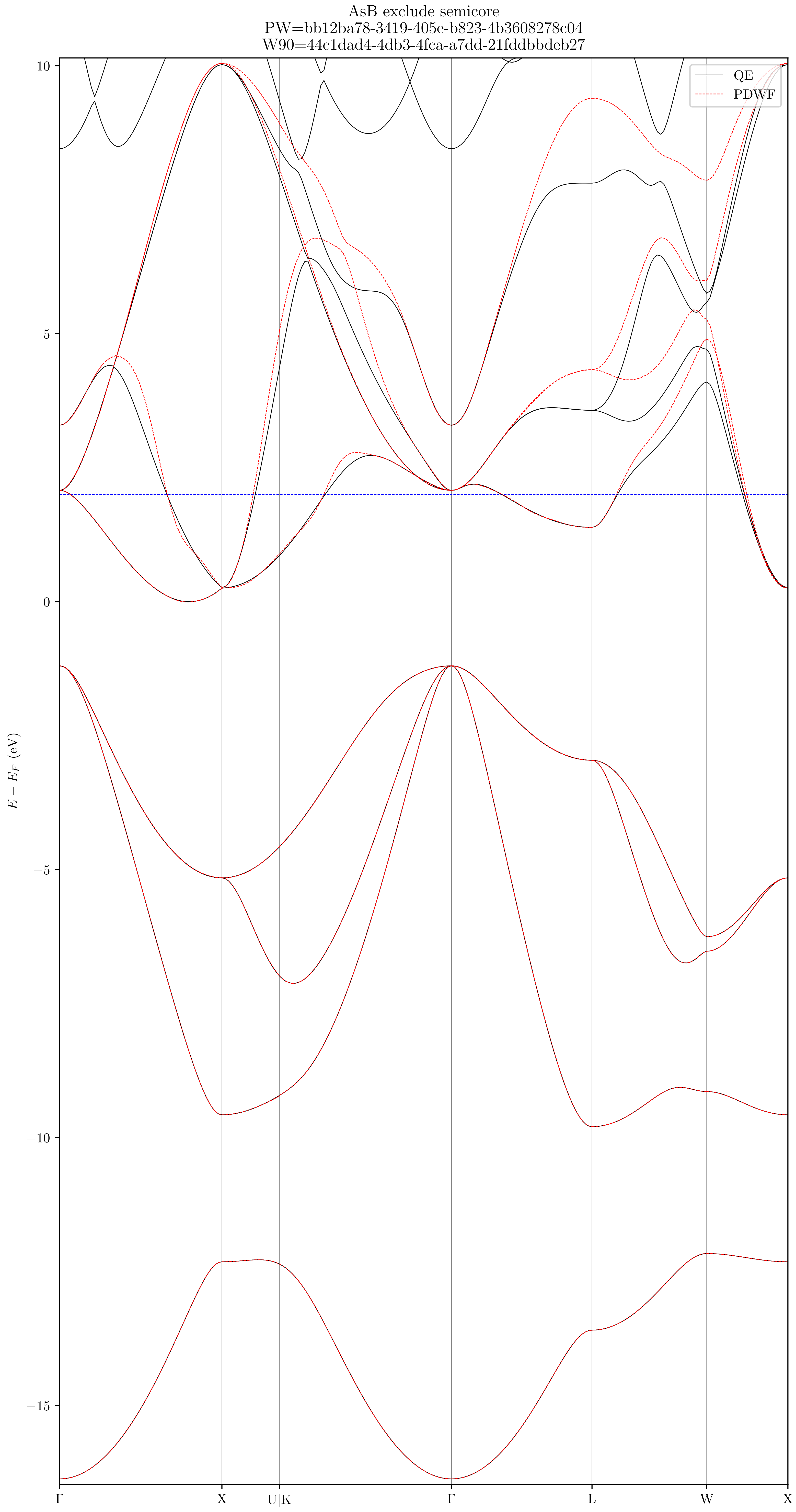

PDWF band distance (meV) and τ ($\AA$)

η_0	η_2	η_4	$\eta_0^{\max}$	$\eta_2^{\max}$	$\eta_4^{\max}$	τ
1.7	9.7	55.9	8.0	109.1	436.9	2.209

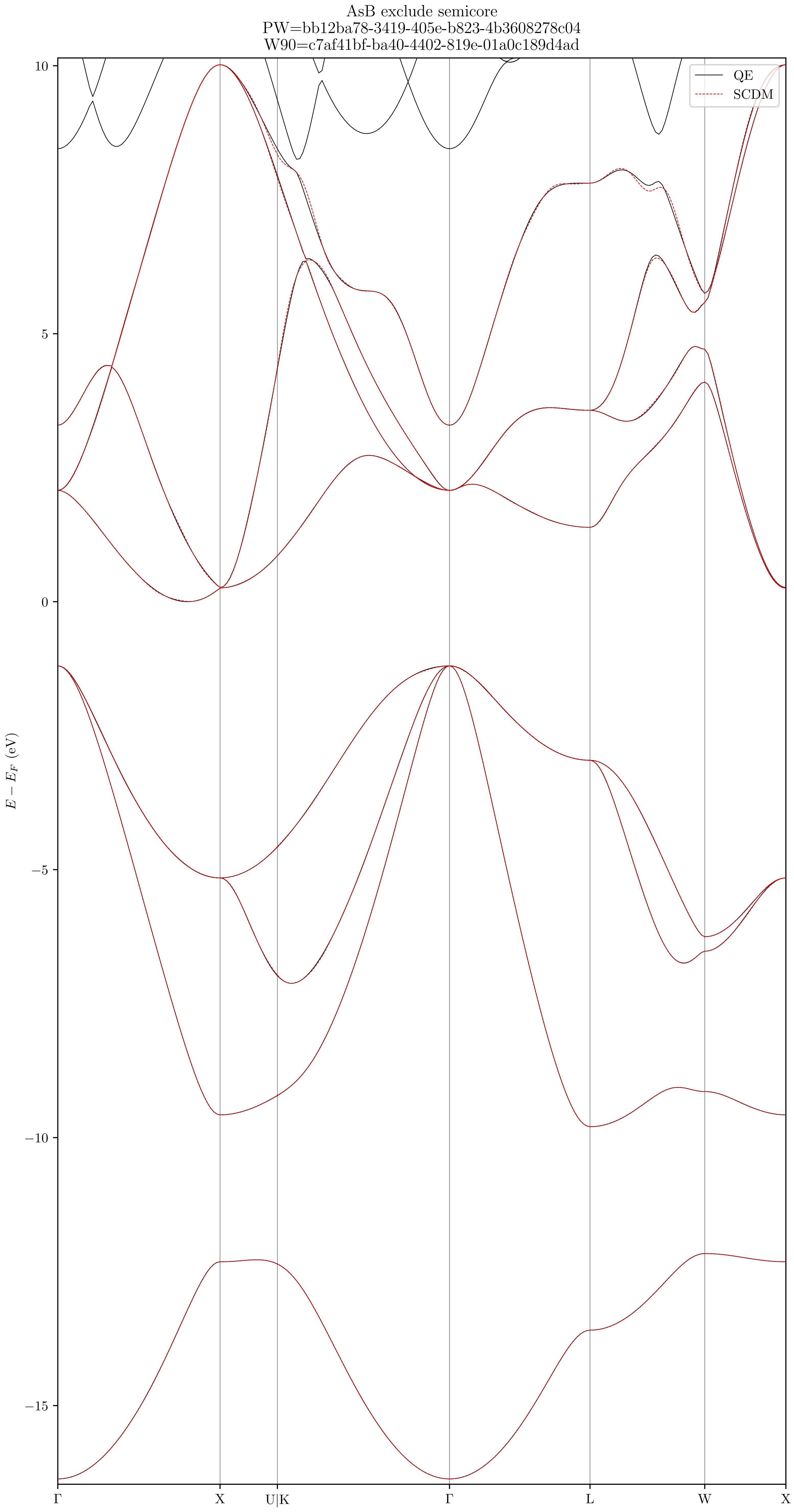

SCDM band distance (meV) and τ ($\AA$)

η_0	η_2	η_4	$\eta_0^{\max}$	$\eta_2^{\max}$	$\eta_4^{\max}$	τ
3.9	4.3	4.8	19.2	19.2	27.6	2.309

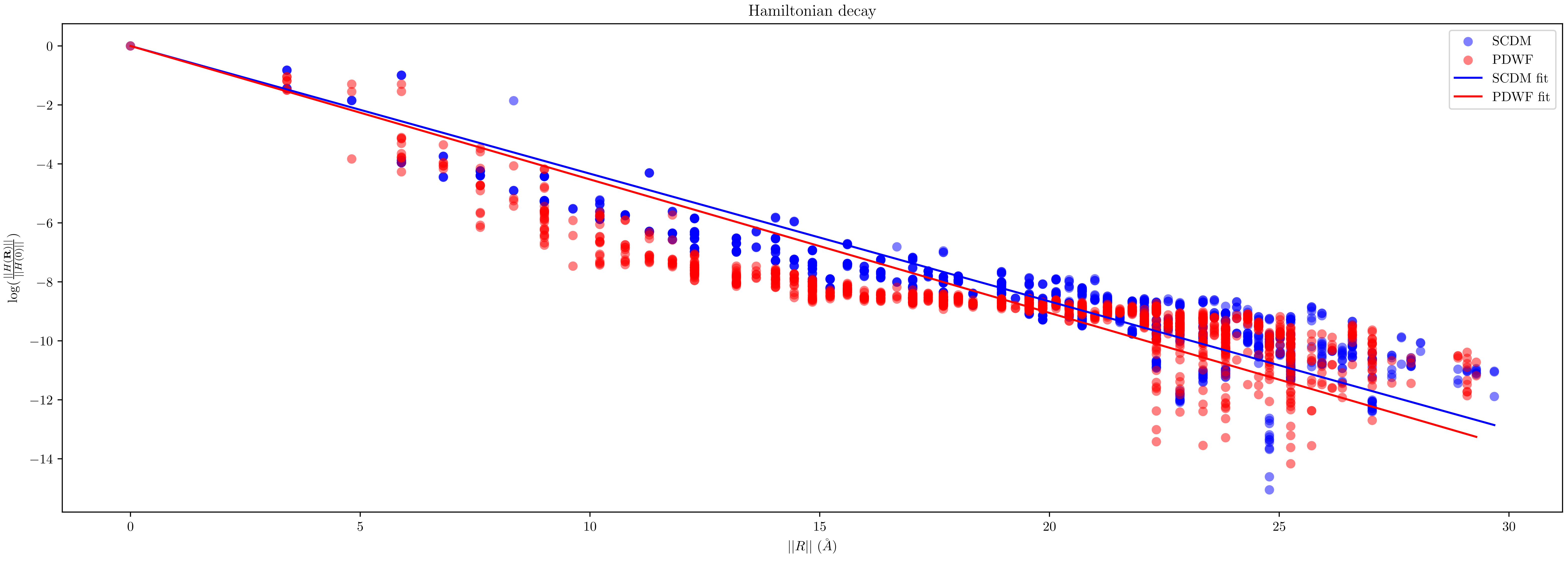

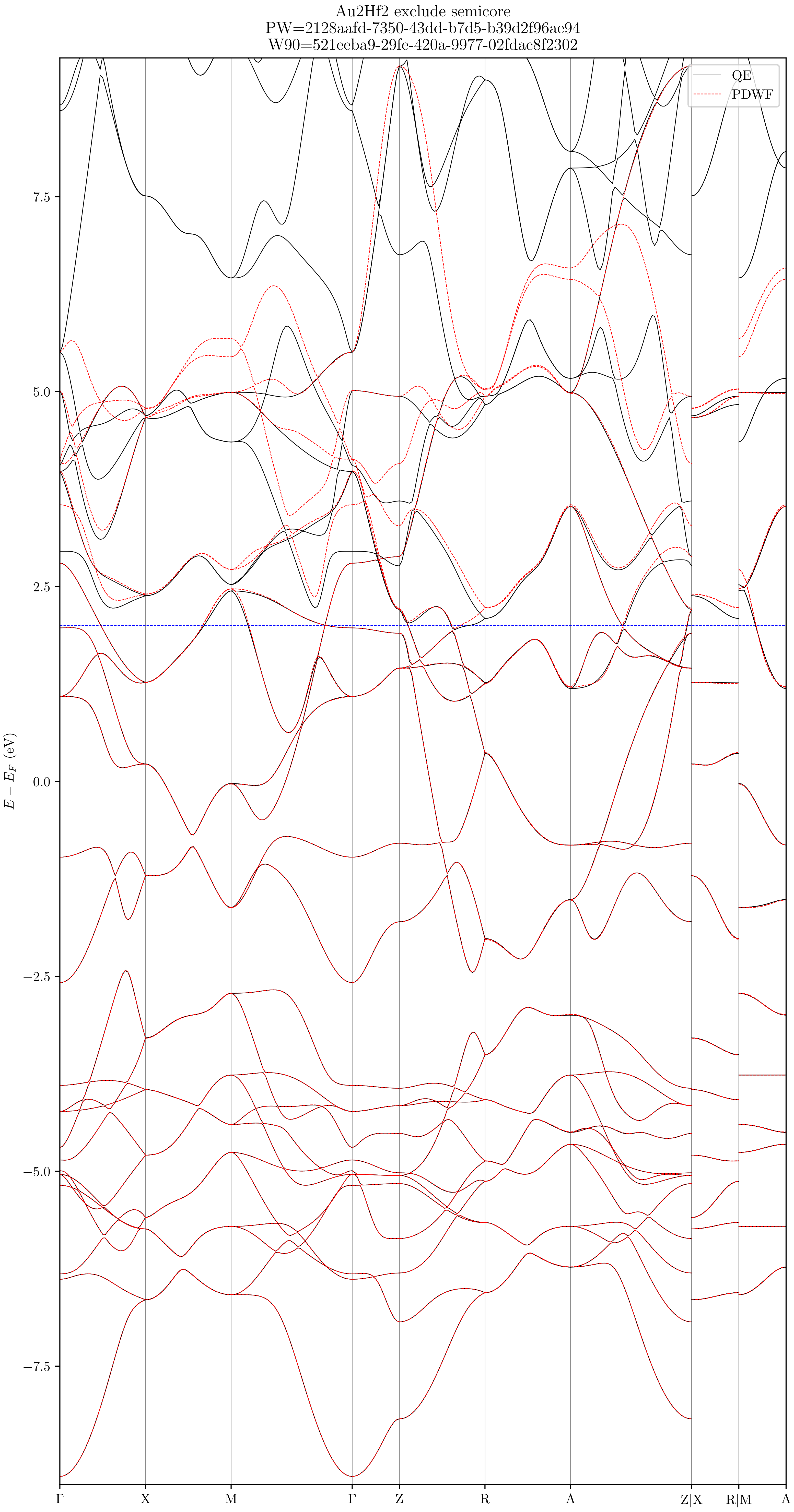

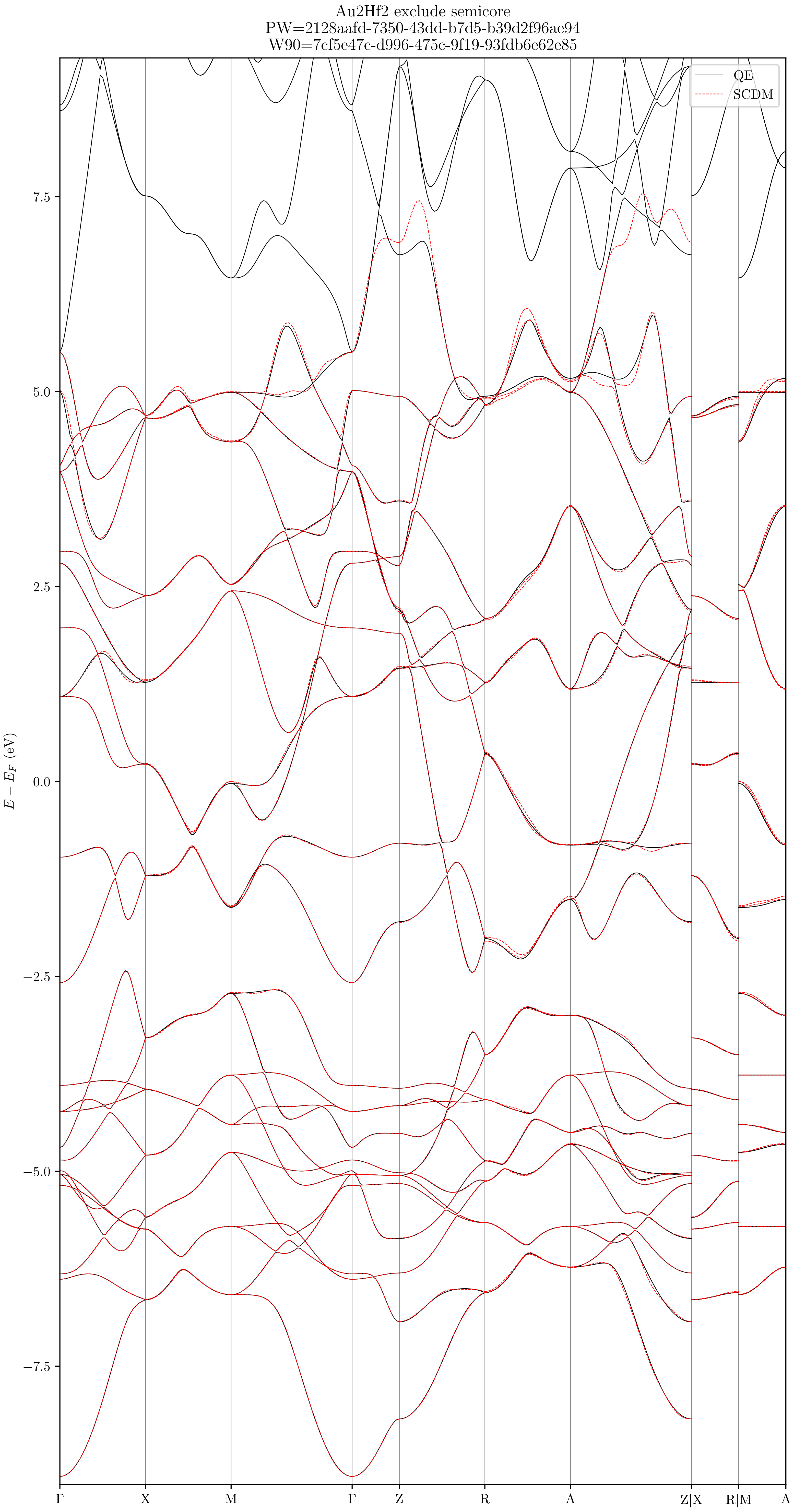

PDWF band distance (meV) and τ ($\AA$)

η_0	η_2	η_4	$\eta_0^{\max}$	$\eta_2^{\max}$	$\eta_4^{\max}$	τ
2.1	6.3	62.5	13.2	42.0	705.6	2.075

SCDM band distance (meV) and τ ($\AA$)

η_0	η_2	η_4	$\eta_0^{\max}$	$\eta_2^{\max}$	$\eta_4^{\max}$	τ
9.4	9.9	11.0	72.1	72.1	81.8	2.463

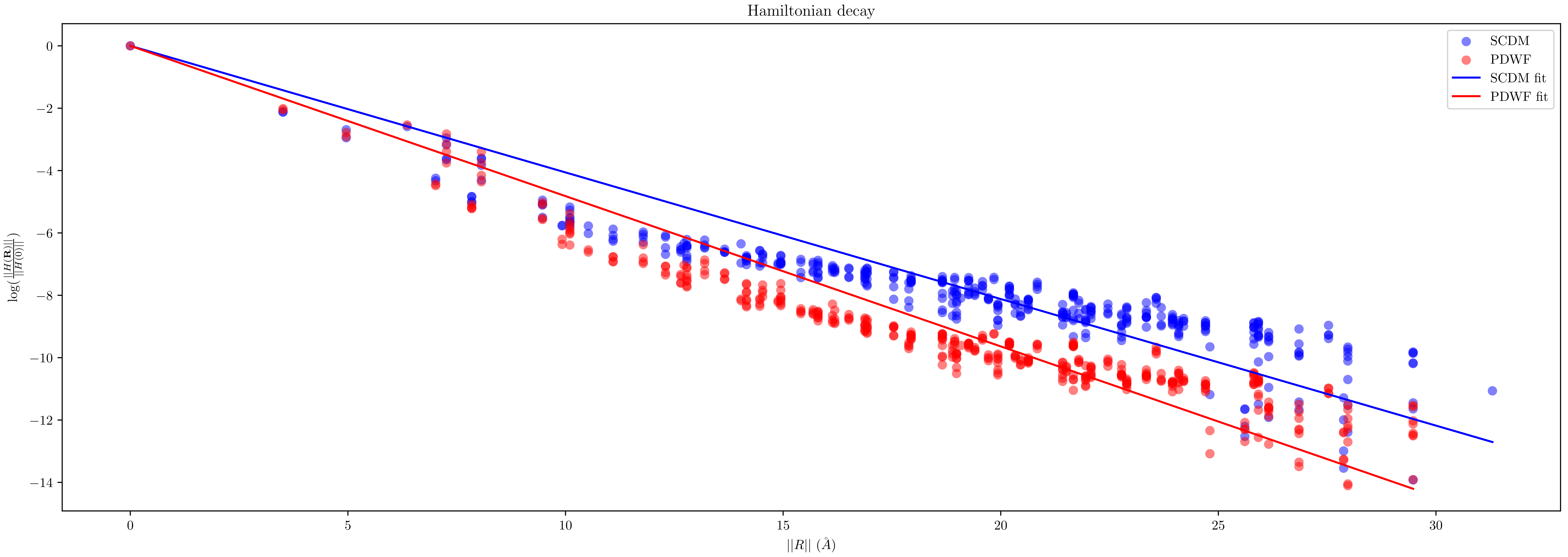

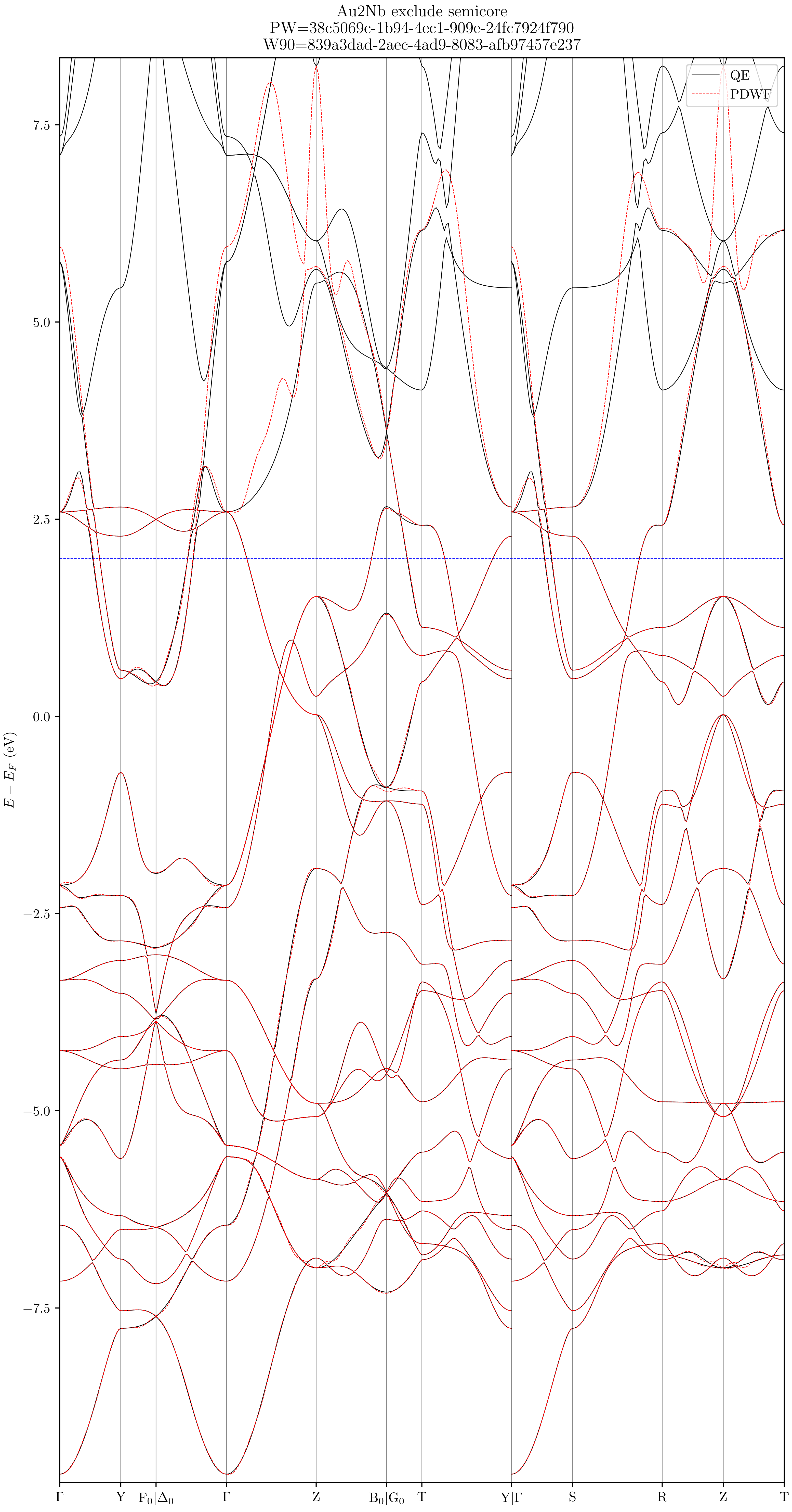

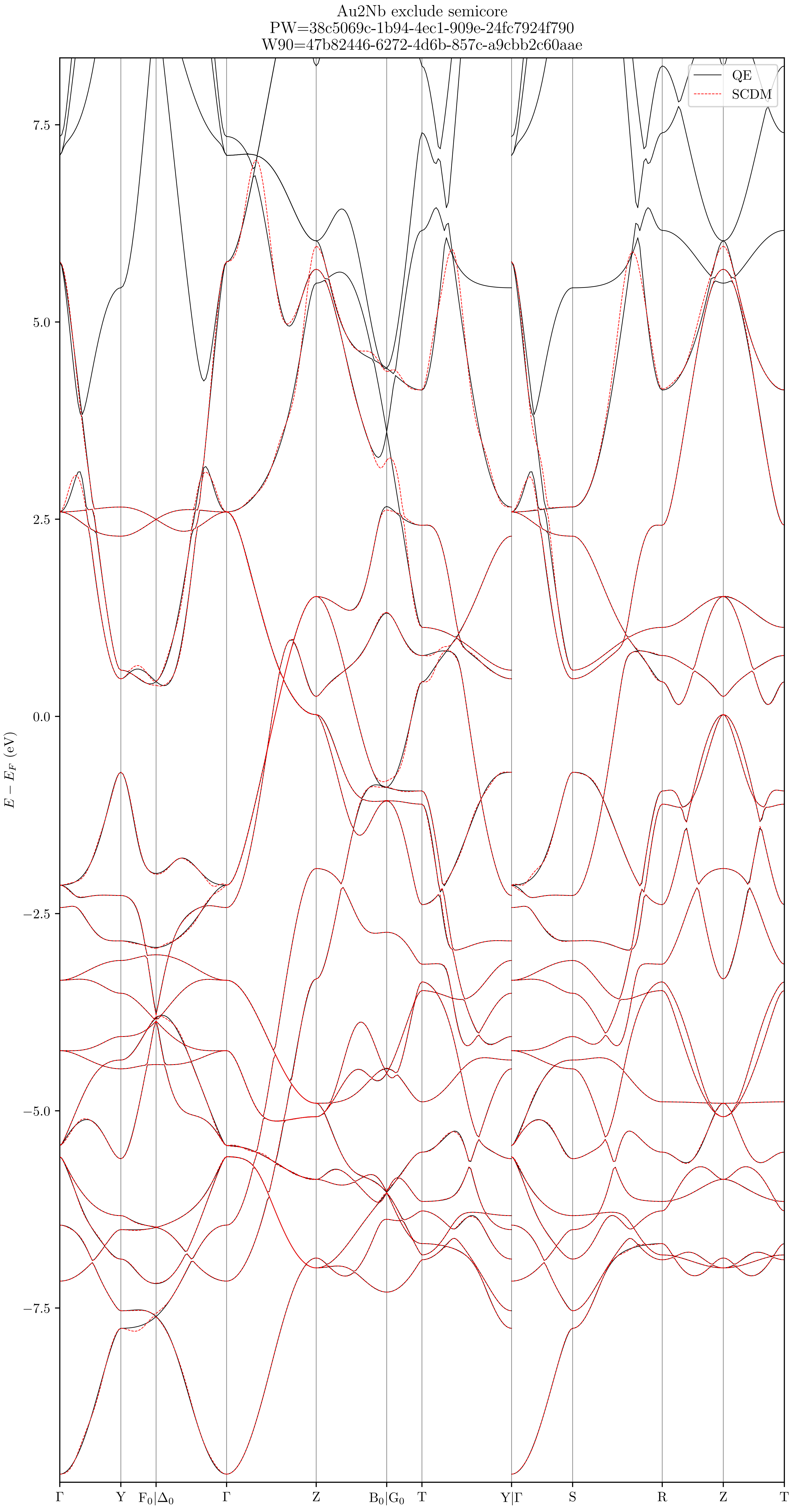

PDWF band distance (meV) and τ ($\AA$)

η_0	η_2	η_4	$\eta_0^{\max}$	$\eta_2^{\max}$	$\eta_4^{\max}$	τ
8.3	8.7	32.7	76.8	77.6	597.8	2.186

SCDM band distance (meV) and τ ($\AA$)

η_0	η_2	η_4	$\eta_0^{\max}$	$\eta_2^{\max}$	$\eta_4^{\max}$	τ
7.2	8.2	18.8	80.2	80.2	355.8	2.388

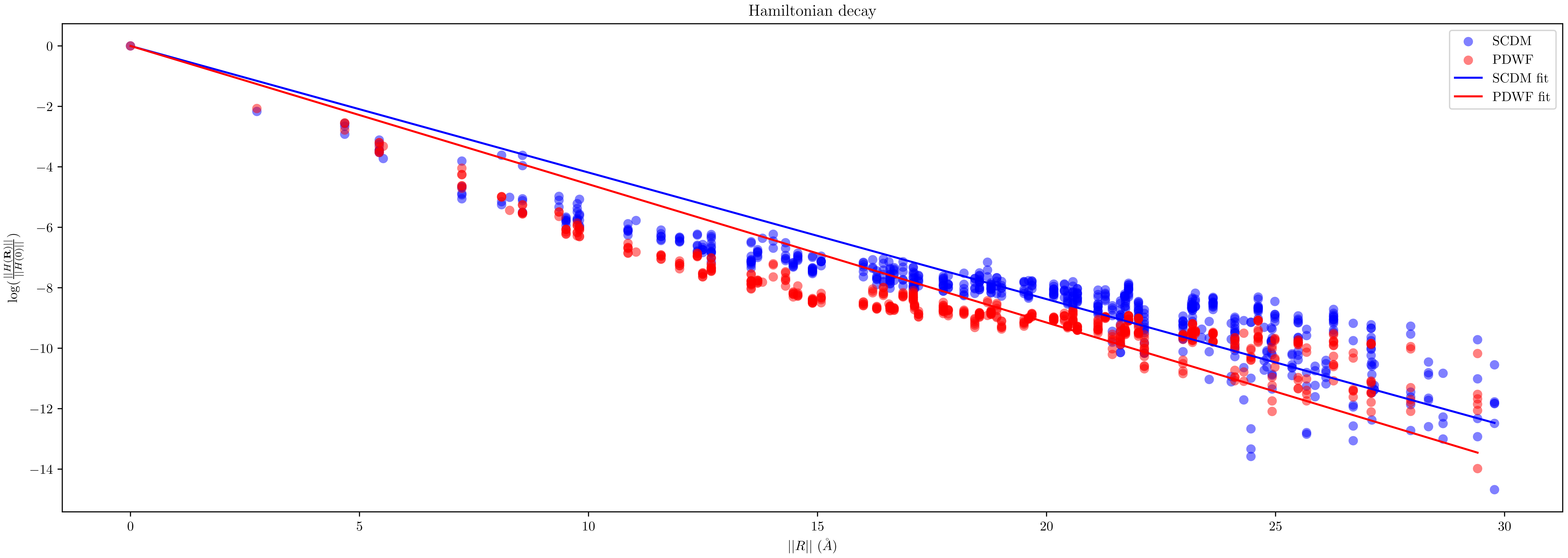

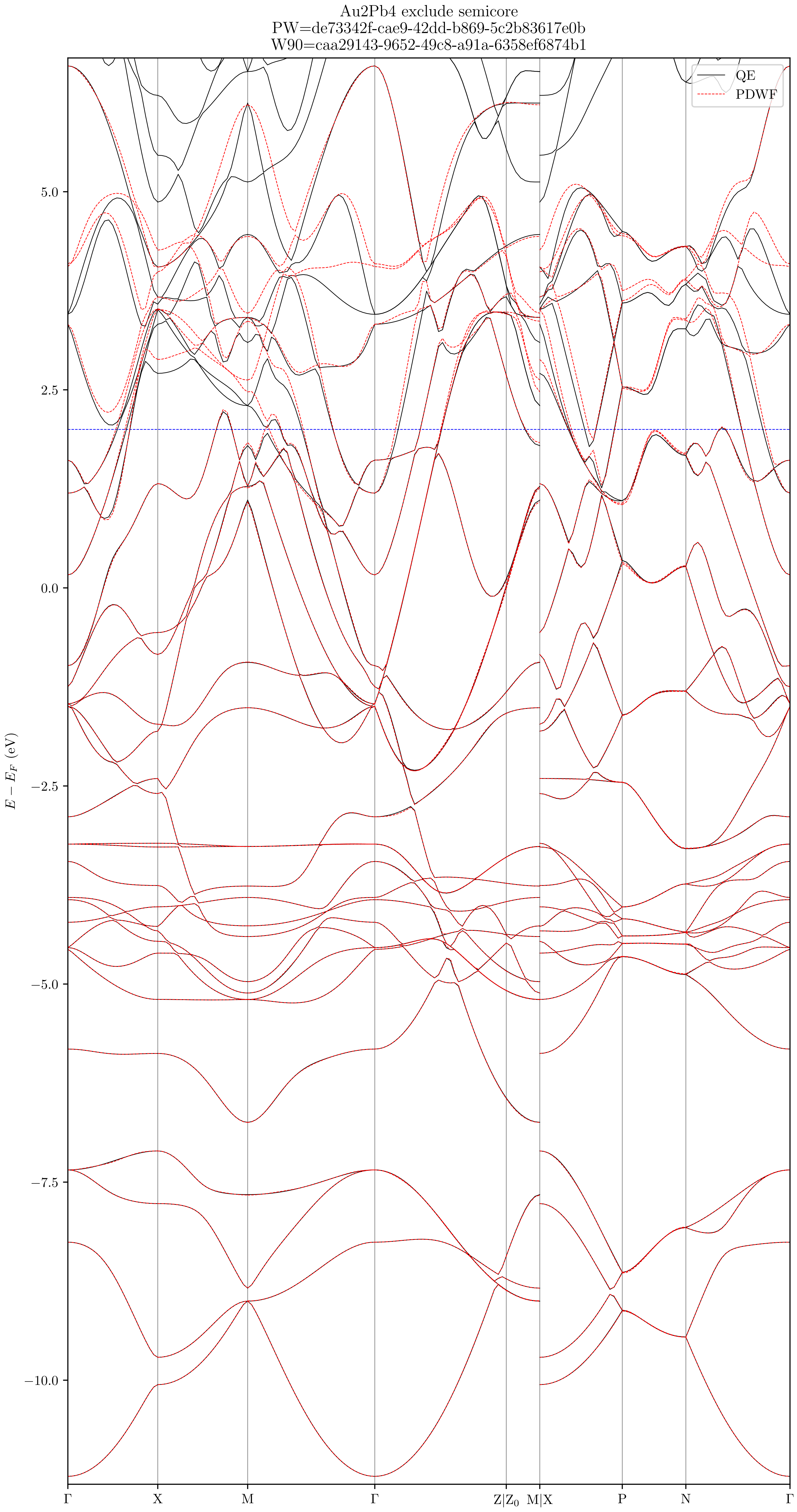

PDWF band distance (meV) and τ ($\AA$)

η_0	η_2	η_4	$\eta_0^{\max}$	$\eta_2^{\max}$	$\eta_4^{\max}$	τ
3.4	9.9	70.2	26.2	141.9	355.7	2.579

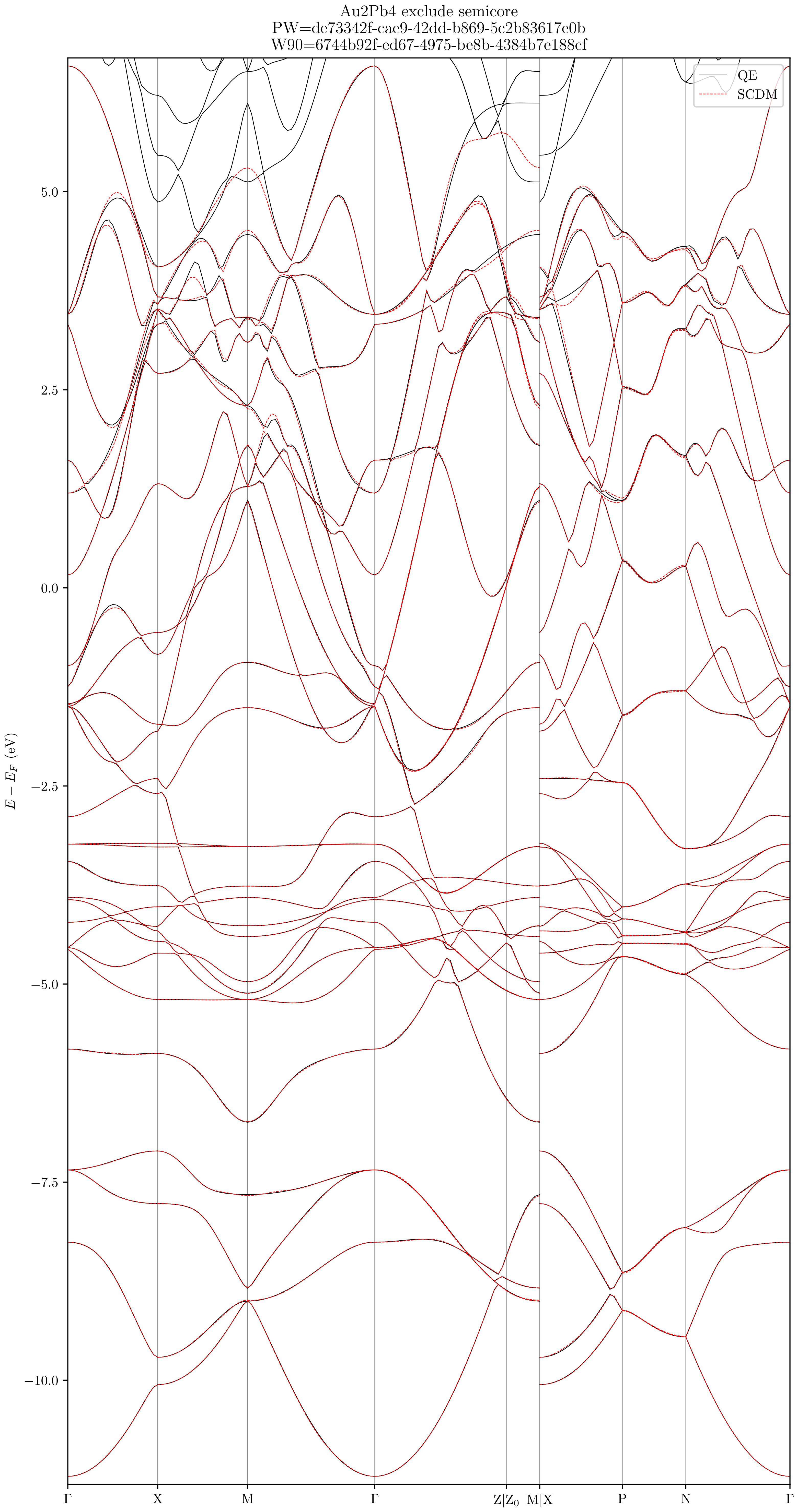

SCDM band distance (meV) and τ ($\AA$)

η_0	η_2	η_4	$\eta_0^{\max}$	$\eta_2^{\max}$	$\eta_4^{\max}$	τ
5.2	7.4	13.1	39.1	70.0	135.3	2.698

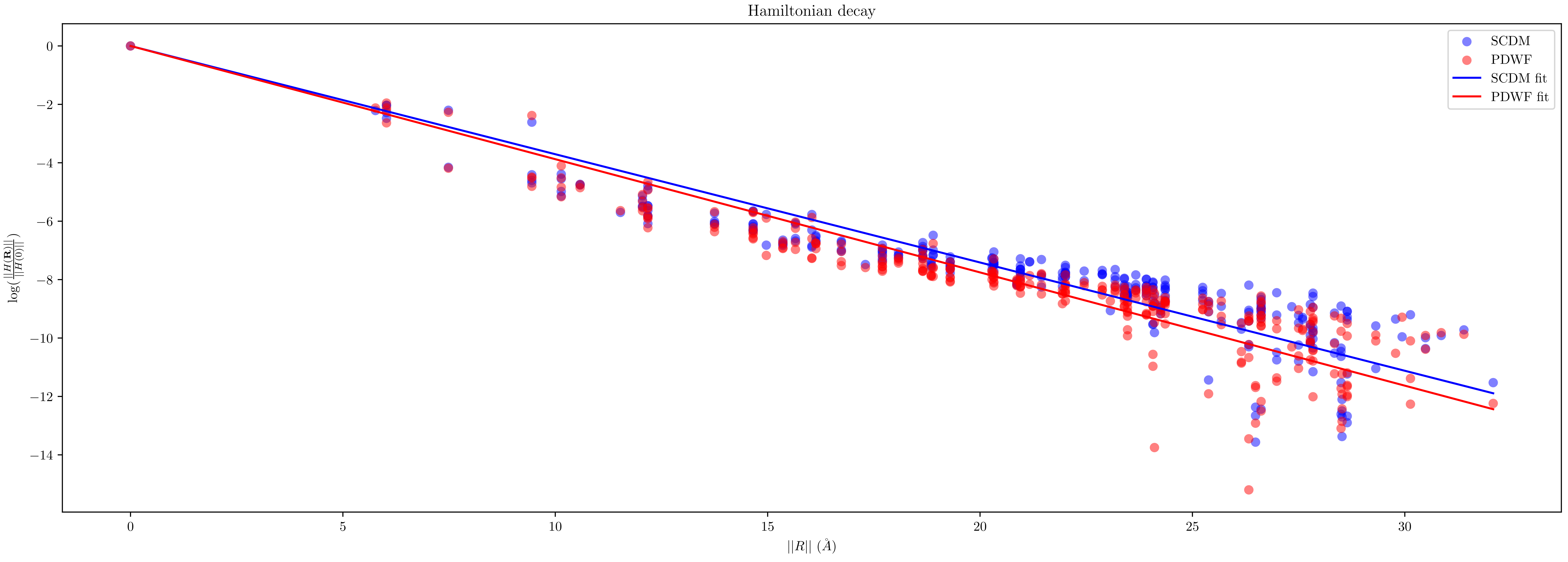

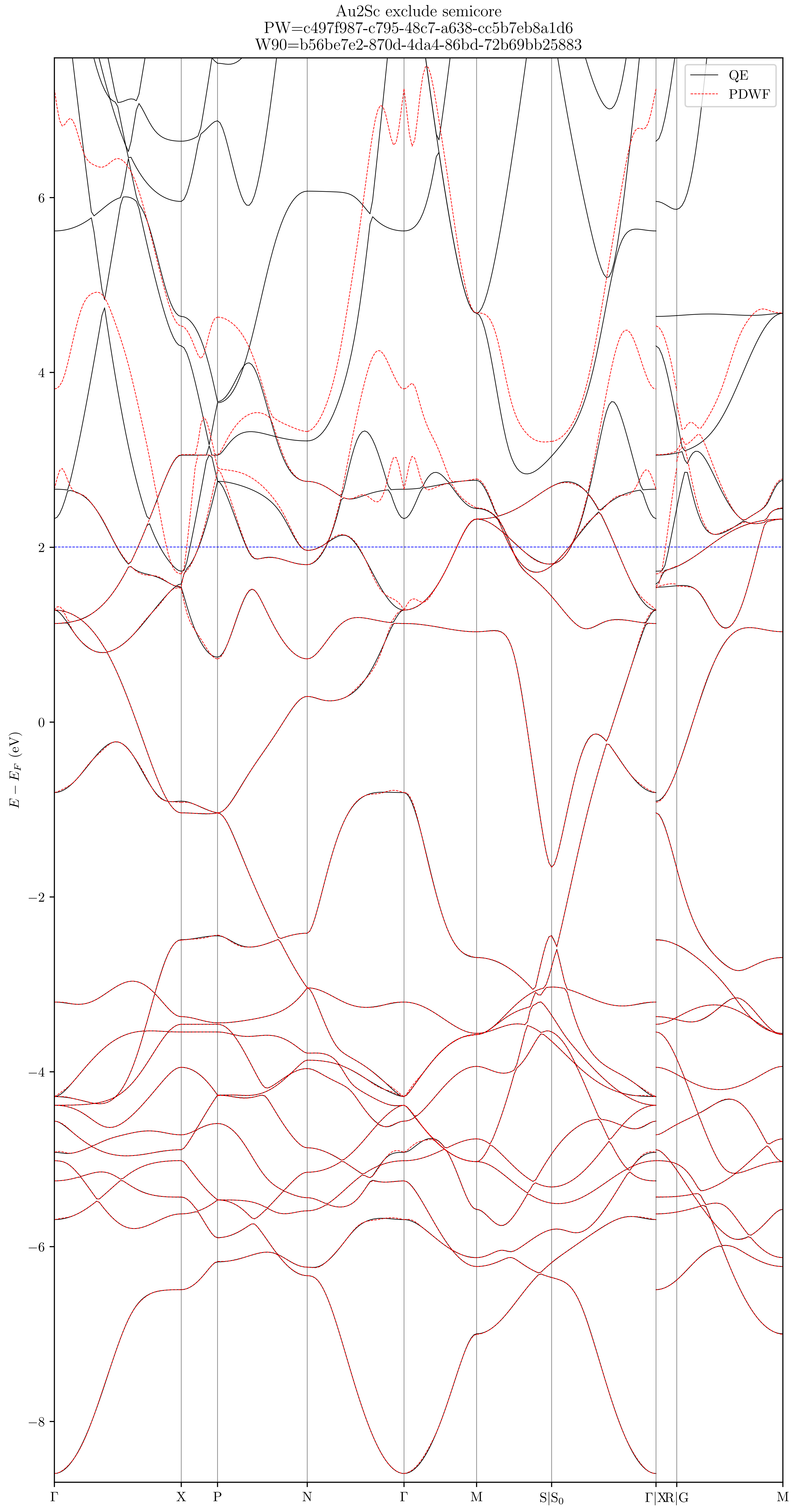

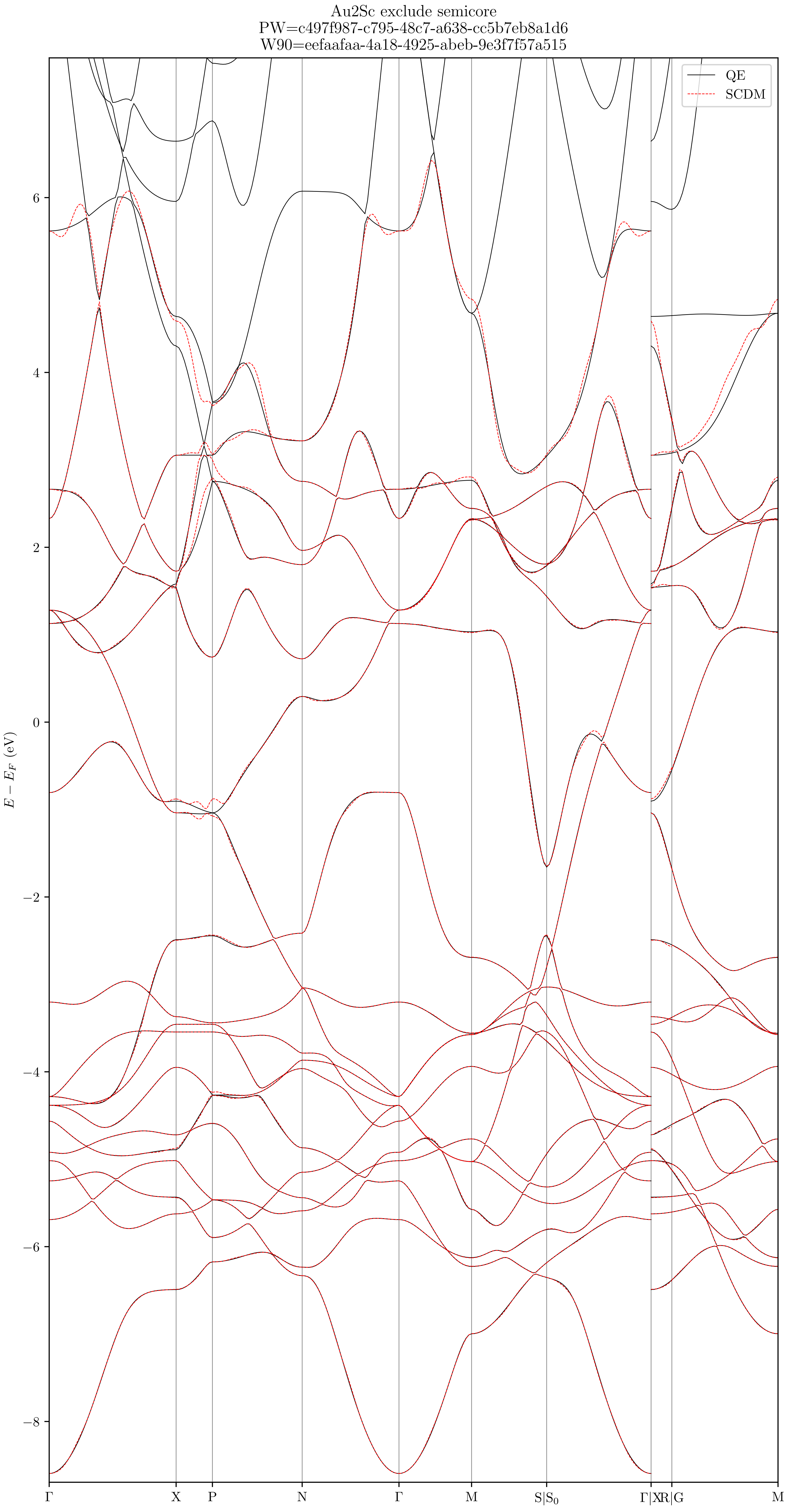

PDWF band distance (meV) and τ ($\AA$)

η_0	η_2	η_4	$\eta_0^{\max}$	$\eta_2^{\max}$	$\eta_4^{\max}$	τ
3.3	9.3	119.2	31.1	117.8	1073.3	2.528

SCDM band distance (meV) and τ ($\AA$)

η_0	η_2	η_4	$\eta_0^{\max}$	$\eta_2^{\max}$	$\eta_4^{\max}$	τ
7.7	7.8	21.6	159.6	159.6	481.1	2.727

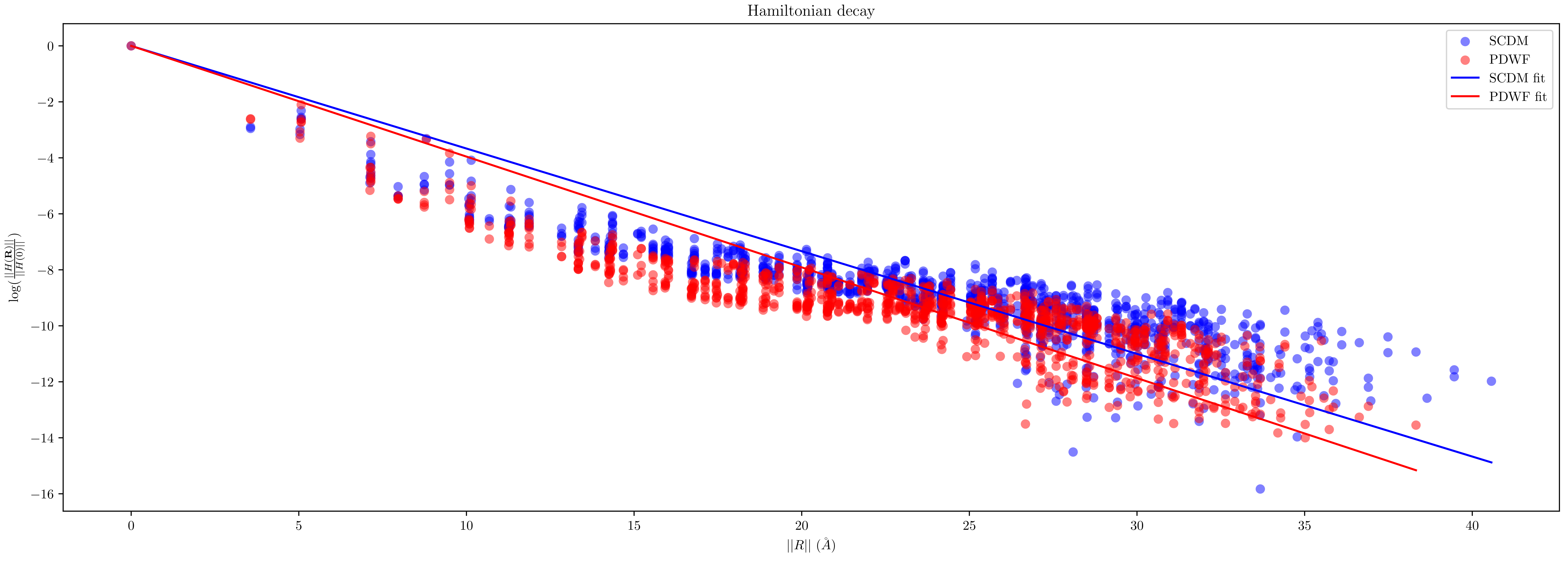

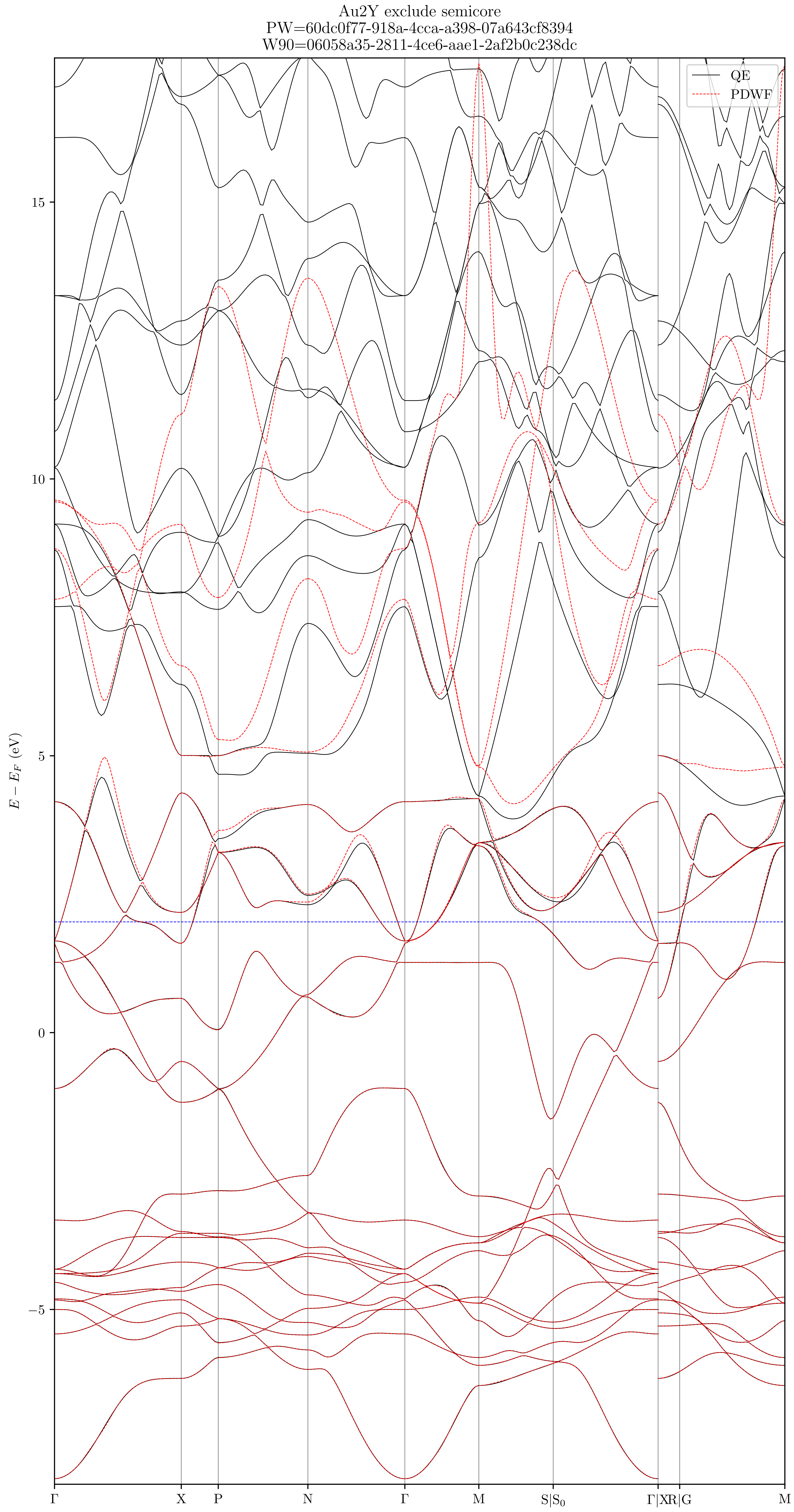

PDWF band distance (meV) and τ ($\AA$)

η_0	η_2	η_4	$\eta_0^{\max}$	$\eta_2^{\max}$	$\eta_4^{\max}$	τ
2.1	3.0	23.7	18.4	51.0	255.8	2.614

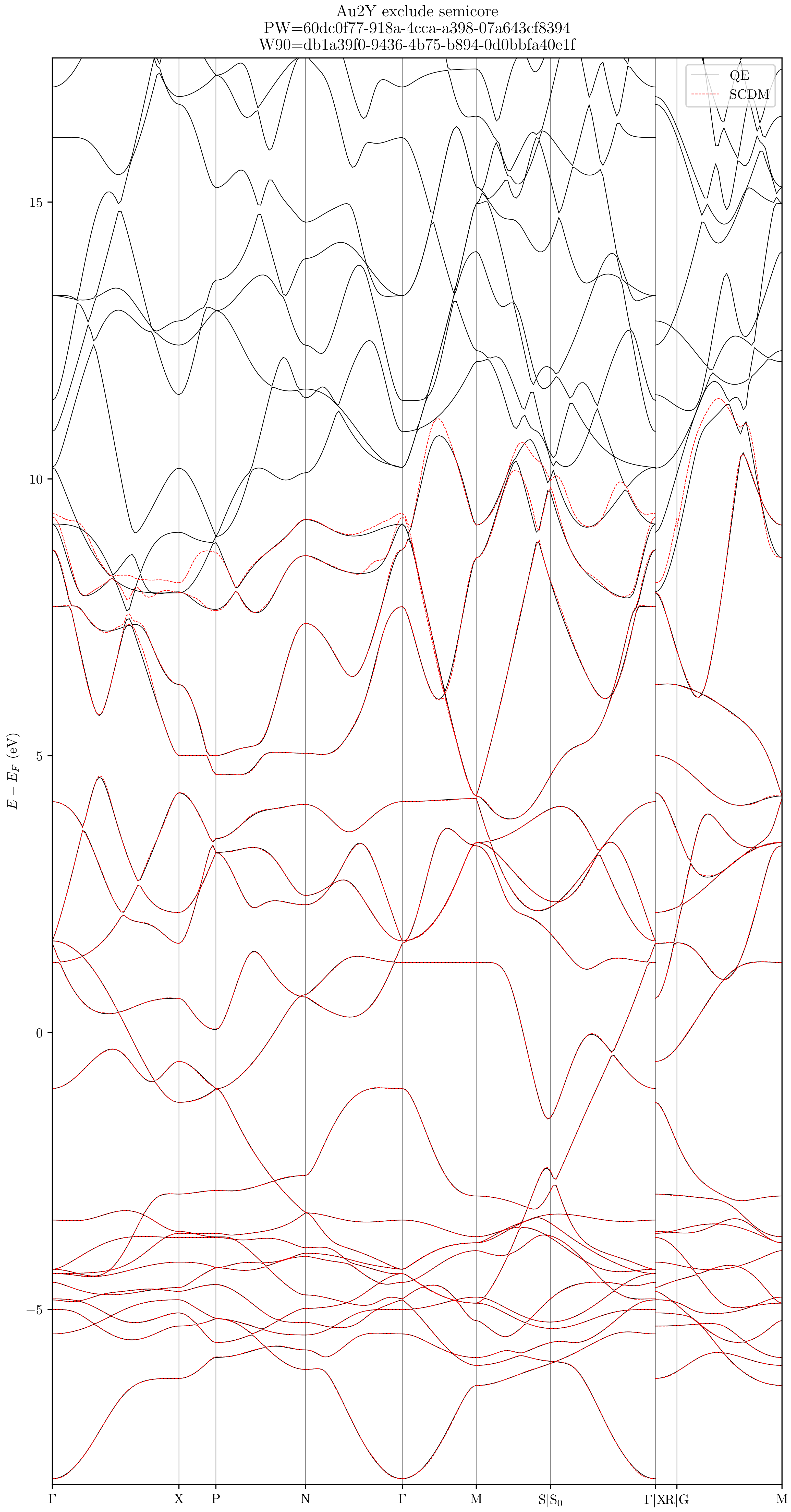

SCDM band distance (meV) and τ ($\AA$)

η_0	η_2	η_4	$\eta_0^{\max}$	$\eta_2^{\max}$	$\eta_4^{\max}$	τ
3.1	3.6	4.4	24.4	28.2	42.0	2.833

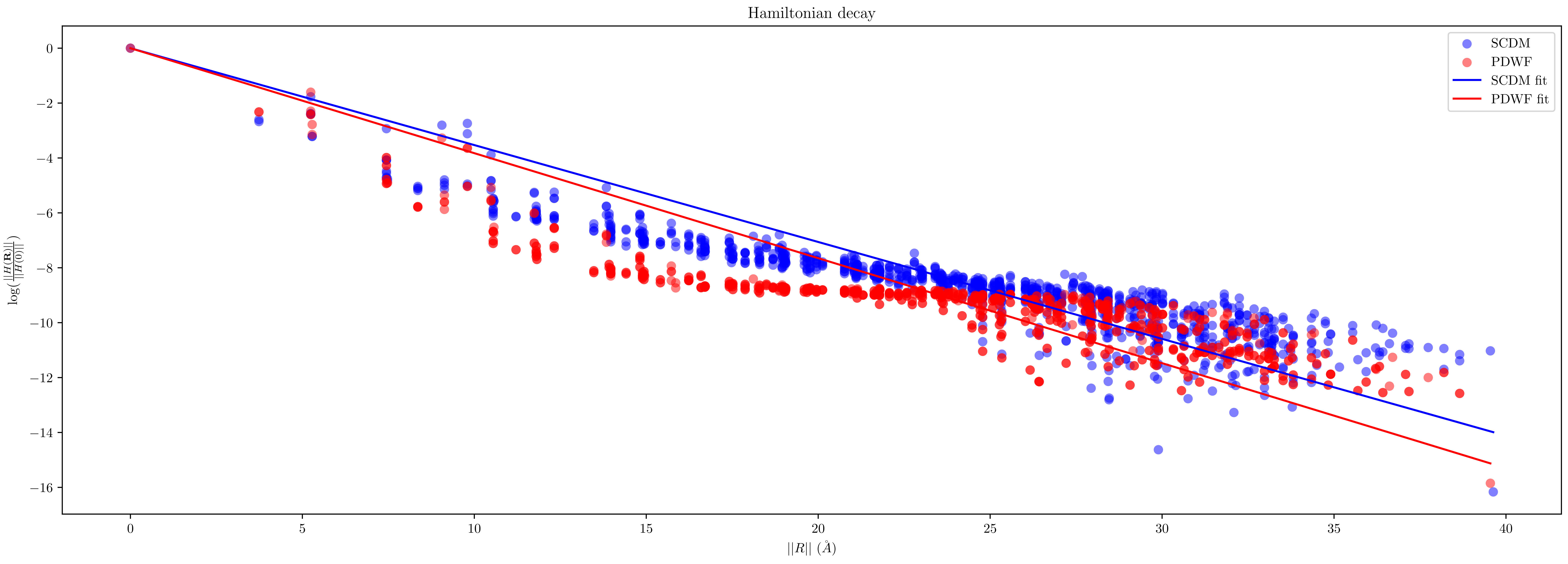

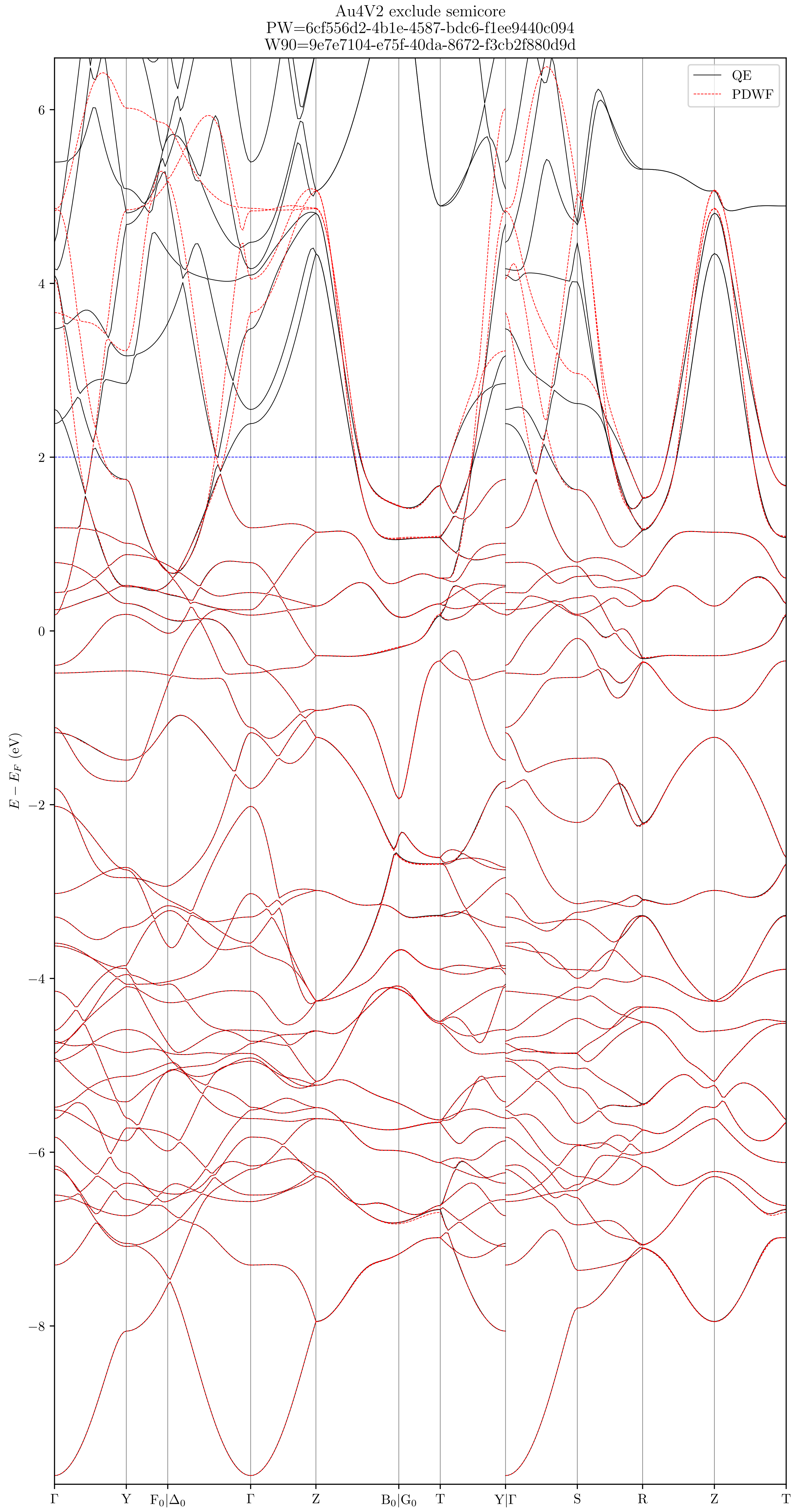

PDWF band distance (meV) and τ ($\AA$)

η_0	η_2	η_4	η_0^{max}	η_2^{max}	η_4^{max}	τ
2.8	6.6	112.8	33.0	111.8	1256.5	2.118

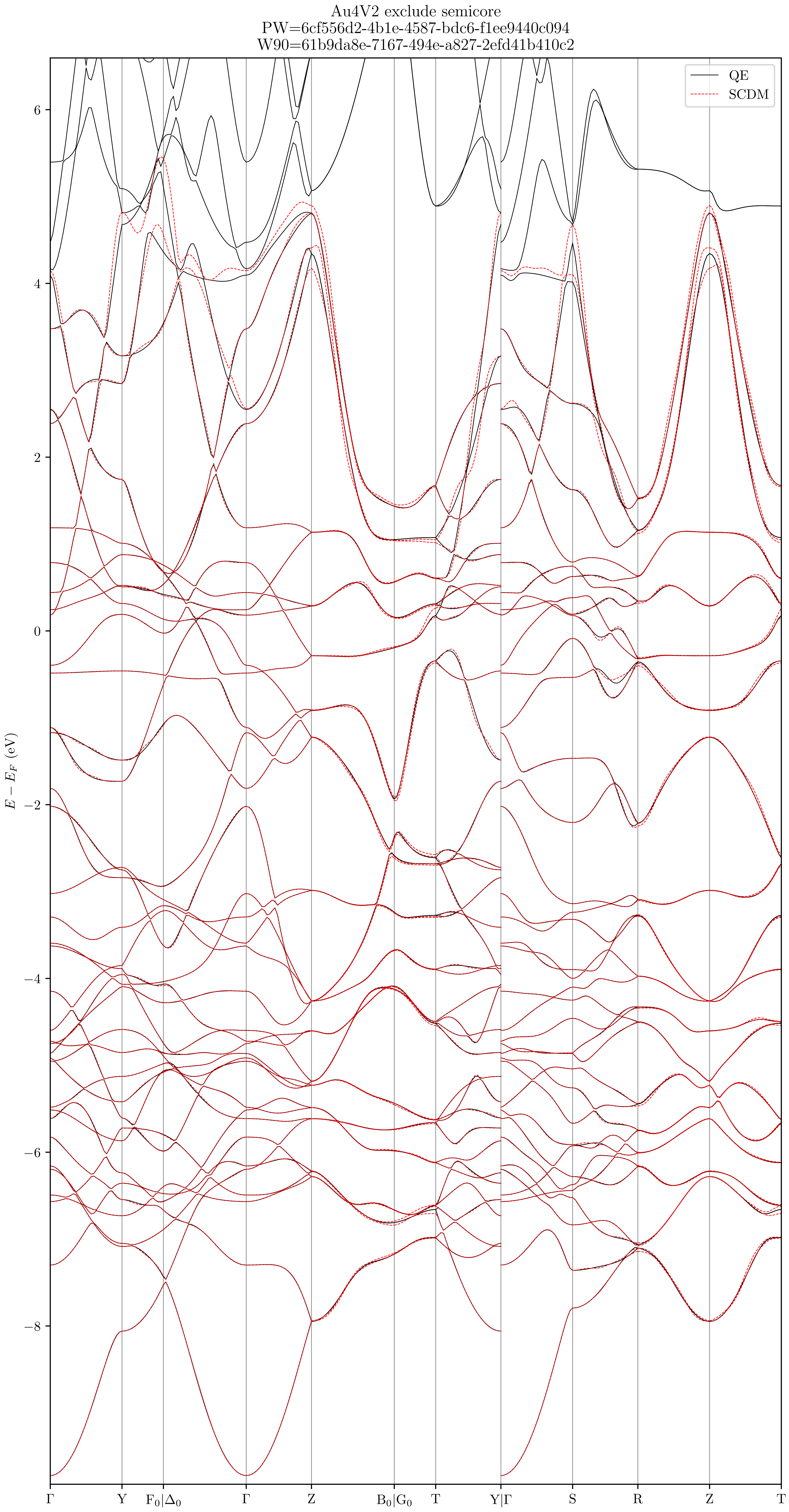

SCDM band distance (meV) and τ ($\AA$)

η_0	η_2	η_4	η_0^{max}	η_2^{max}	η_4^{max}	τ
8.2	10.3	18.5	73.8	110.5	252.0	2.560

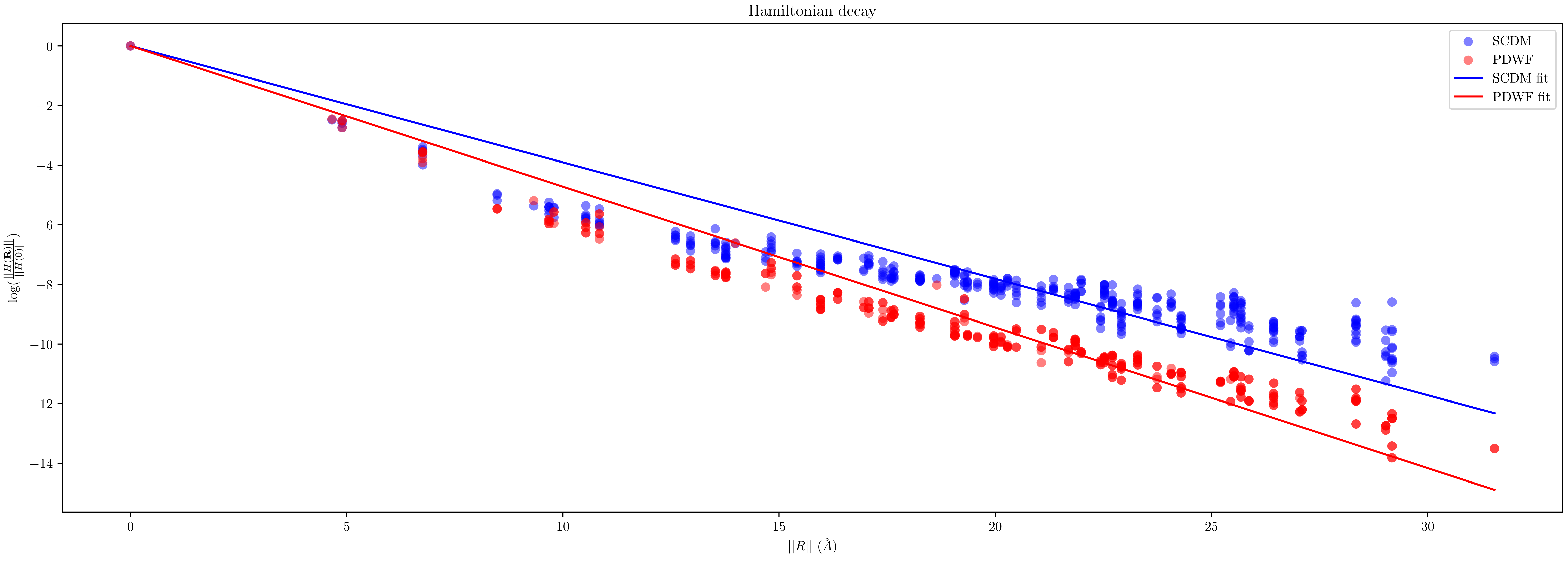

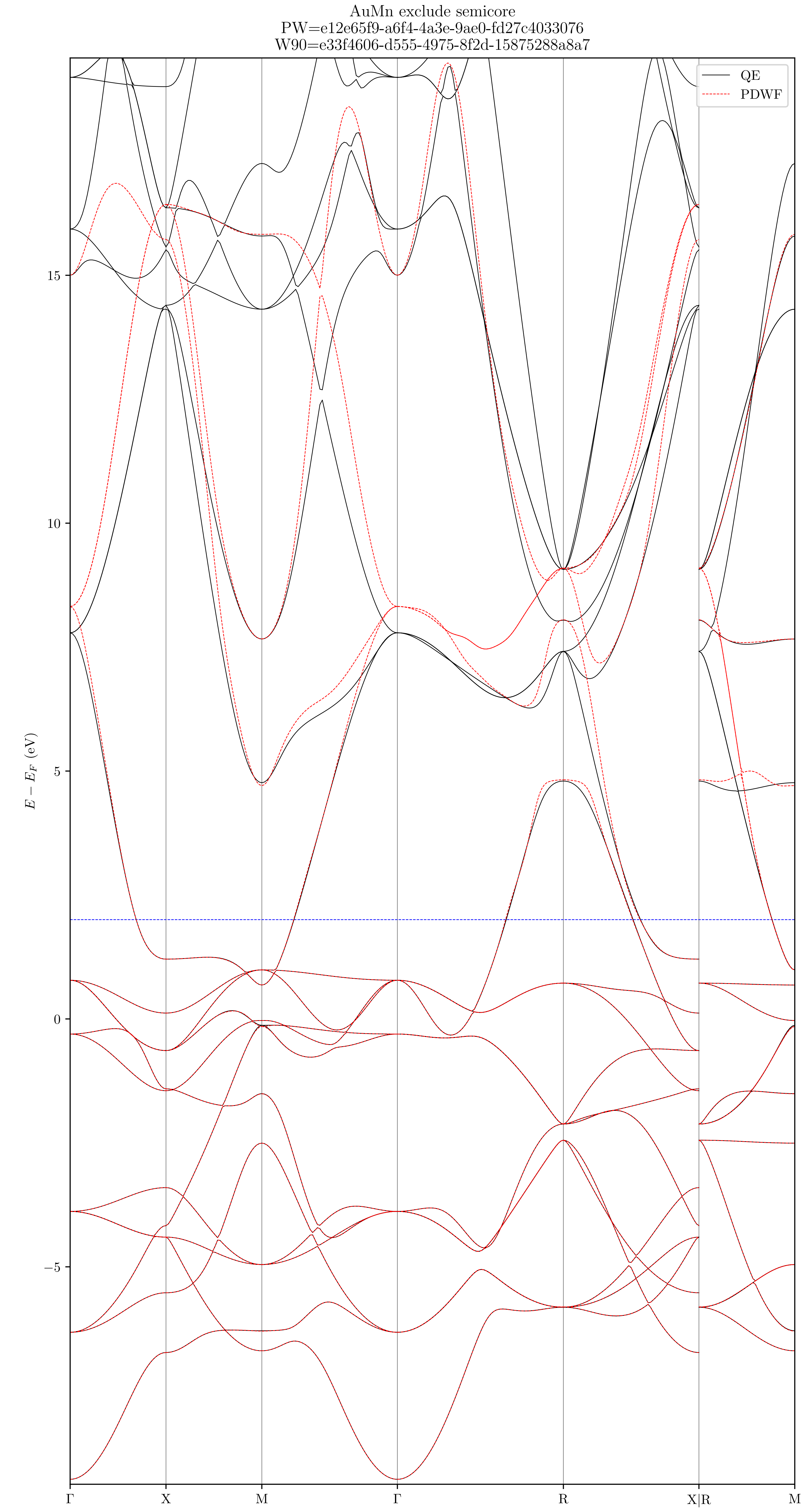

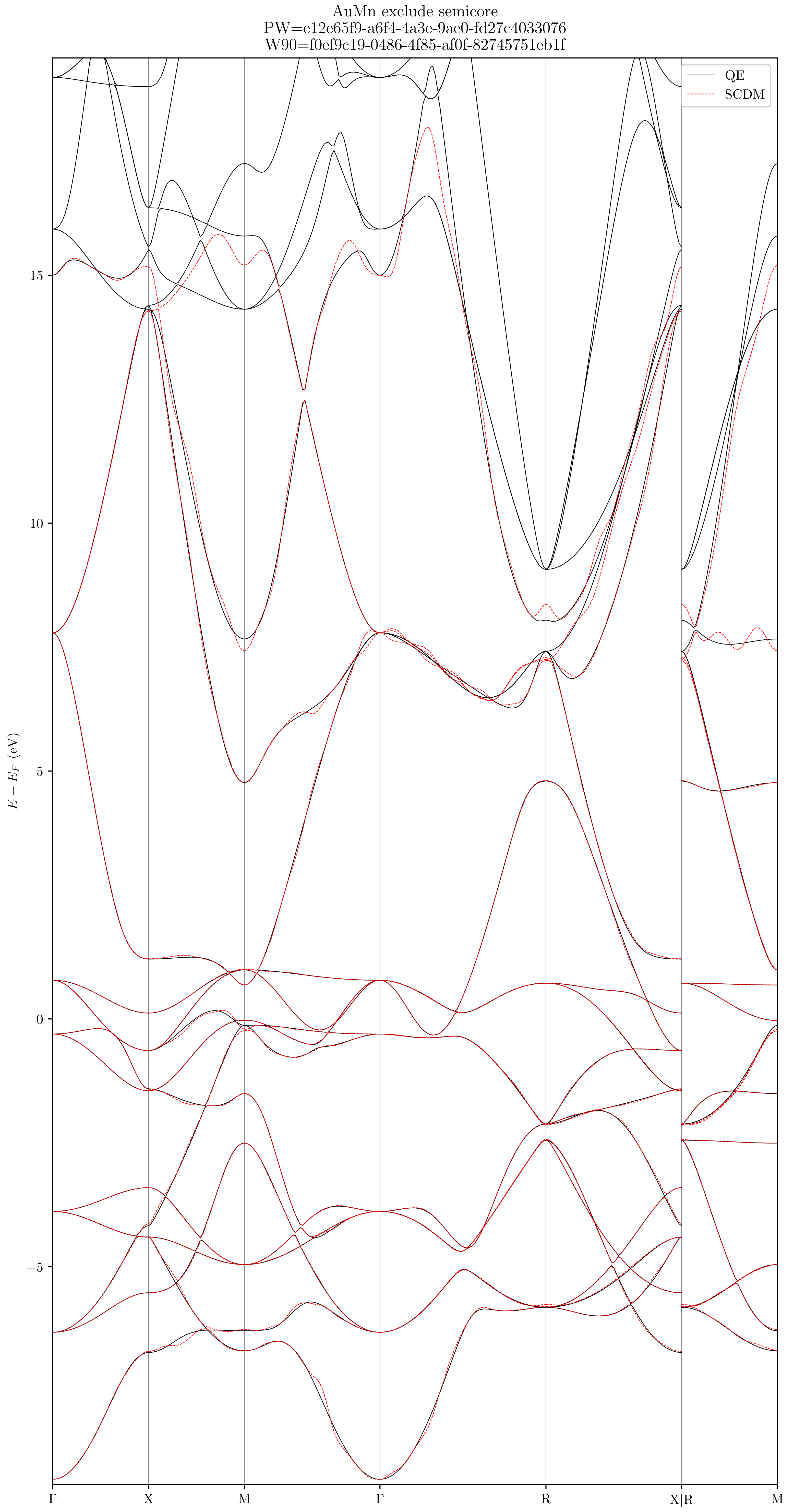

PDWF band distance (meV) and τ ($\AA$)

η_0	η_2	η_4	$\eta_0^{\max}$	$\eta_2^{\max}$	$\eta_4^{\max}$	τ
2.3	2.7	13.5	19.0	28.9	248.1	1.881

SCDM band distance (meV) and τ ($\AA$)

η_0	η_2	η_4	$\eta_0^{\max}$	$\eta_2^{\max}$	$\eta_4^{\max}$	τ
21.6	20.0	20.2	253.1	253.1	253.1	2.449

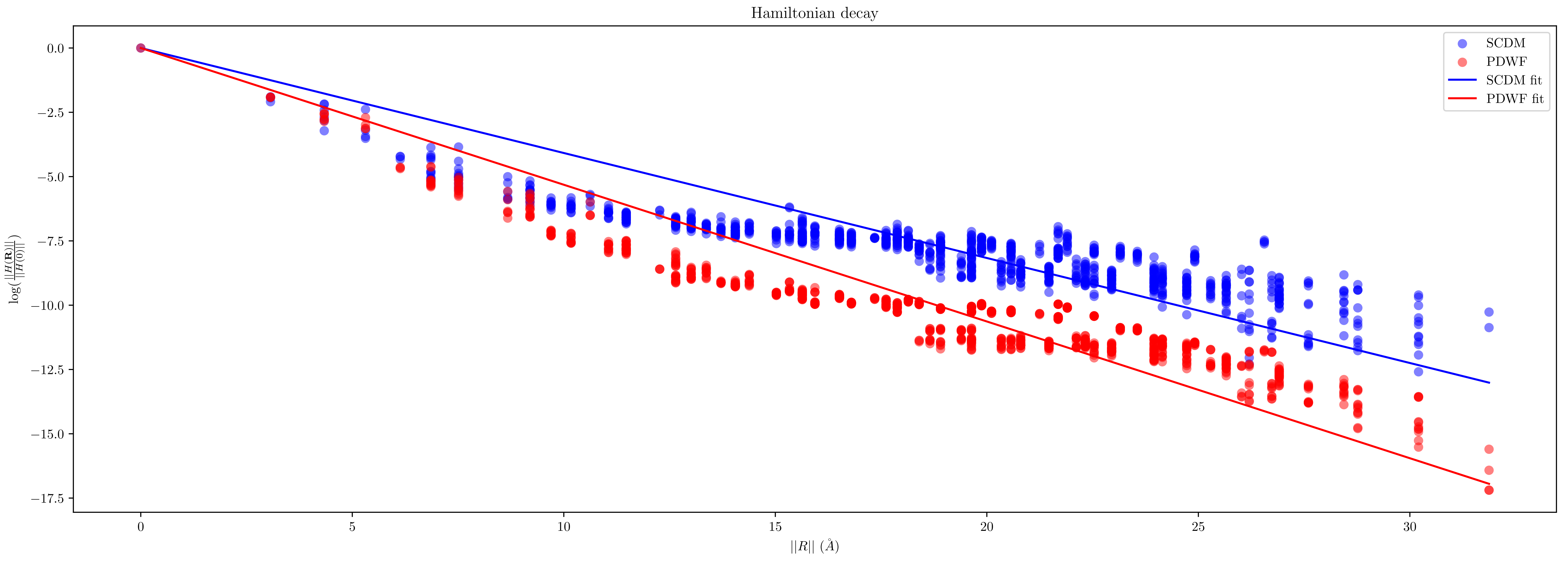

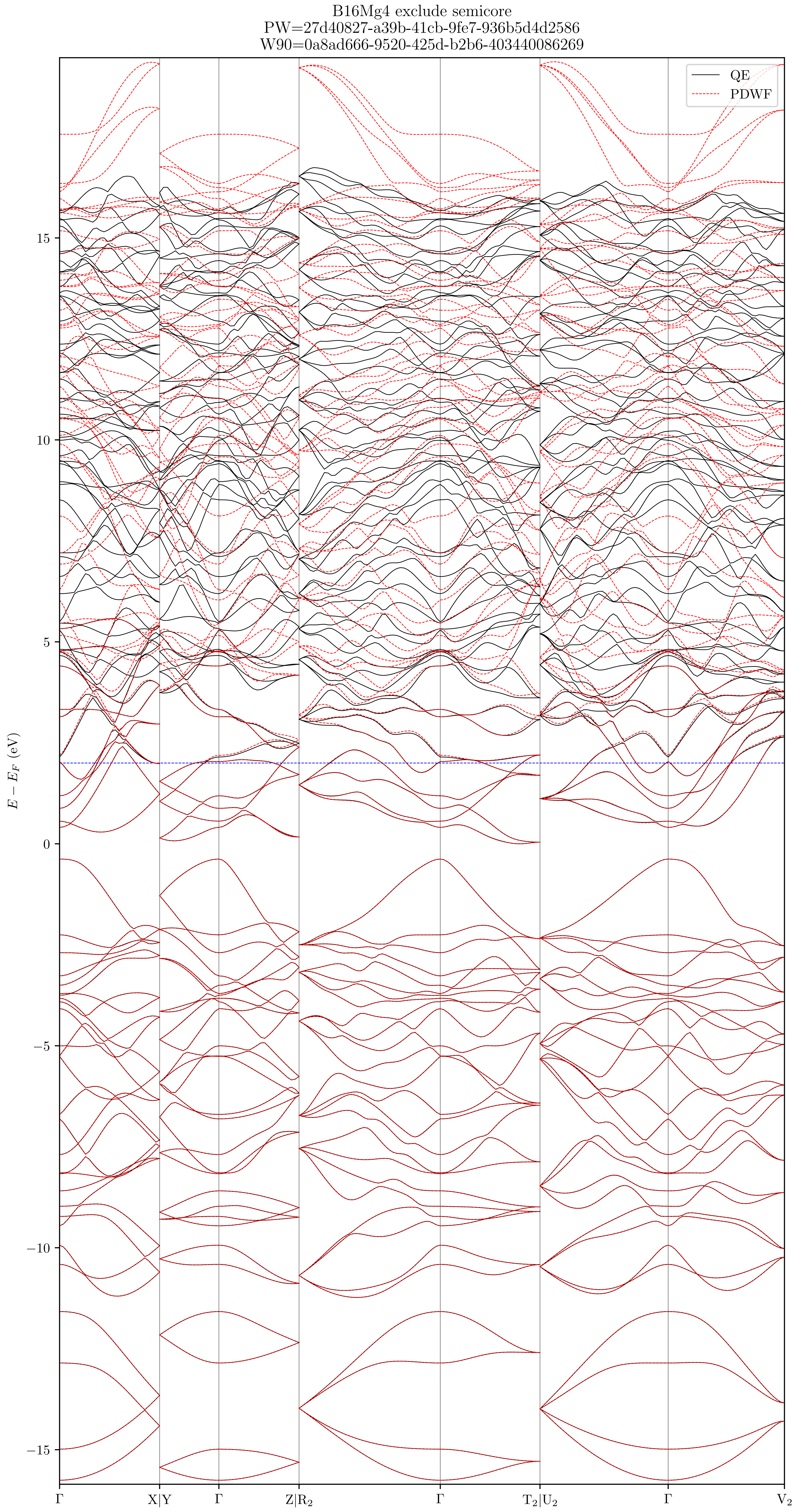

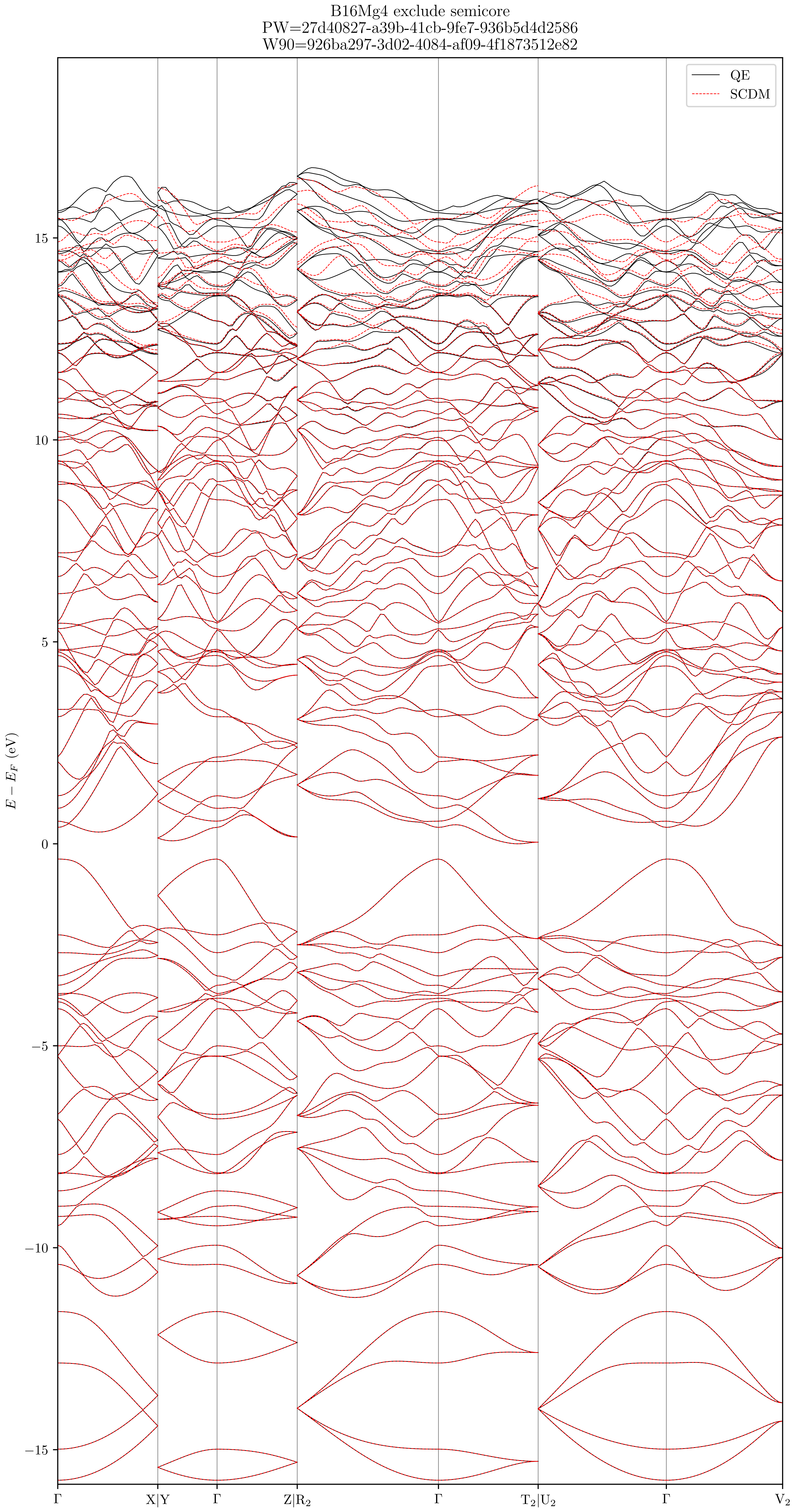

PDWF band distance (meV) and τ ($\AA$)

η_0	η_2	η_4	$\eta_0^{\max}$	$\eta_2^{\max}$	$\eta_4^{\max}$	τ
1.5	1.9	19.8	8.5	10.1	208.5	2.591

SCDM band distance (meV) and τ ($\AA$)

η_0	η_2	η_4	$\eta_0^{\max}$	$\eta_2^{\max}$	$\eta_4^{\max}$	τ
1.1	1.2	1.3	6.7	6.7	8.0	2.743

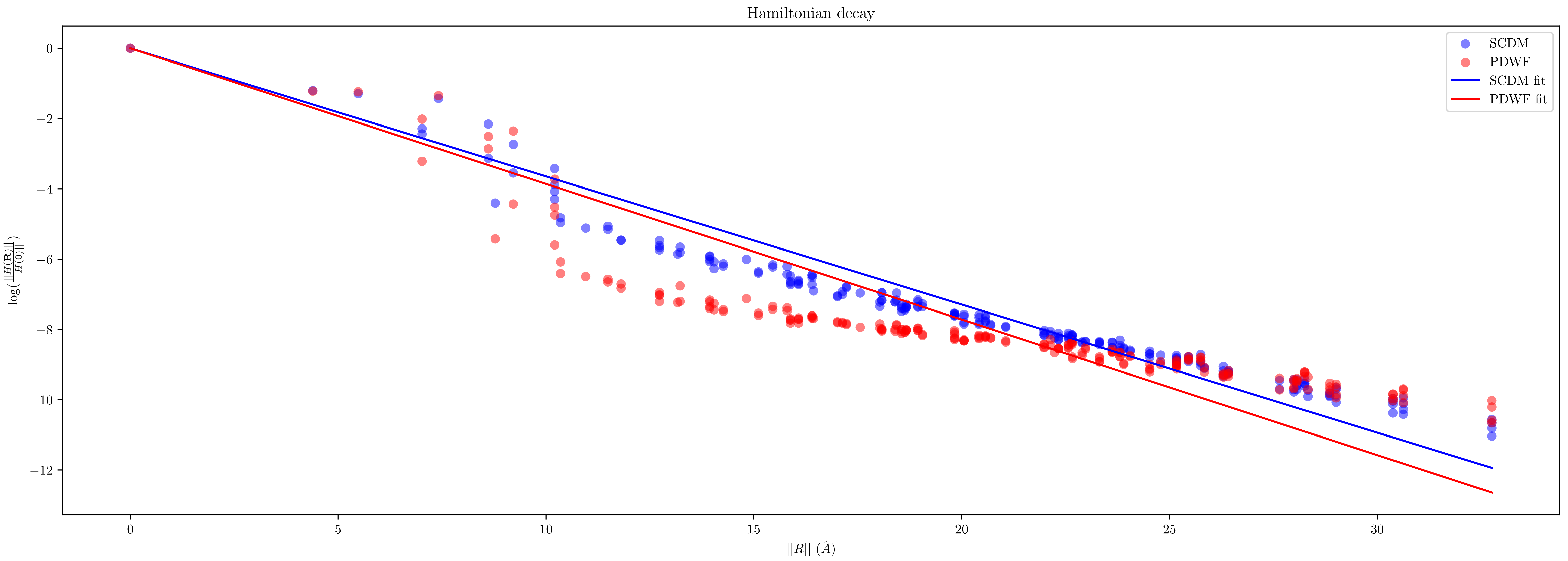

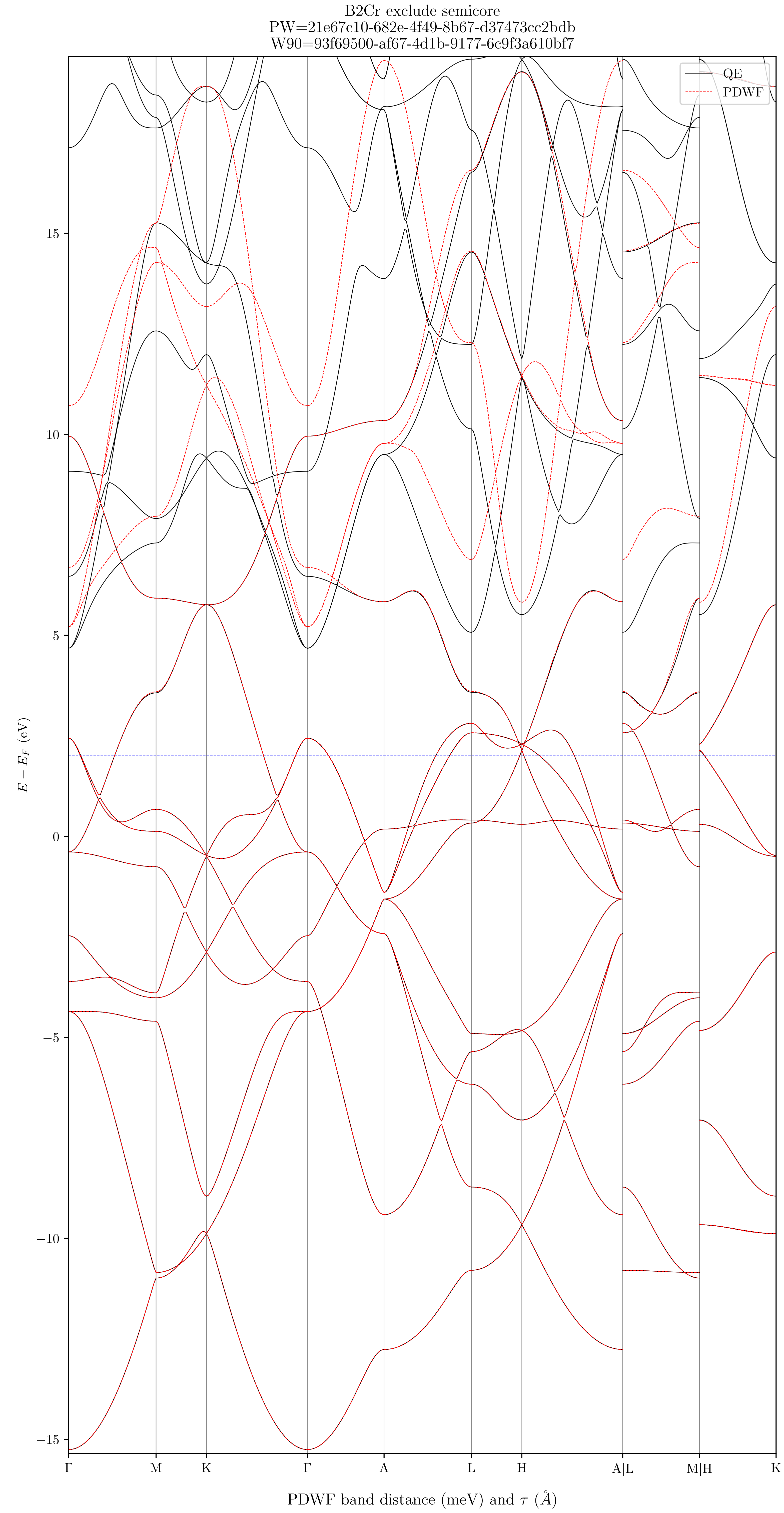

η_0	η_2	η_4	$\eta_0^{\max}$	$\eta_2^{\max}$	$\eta_4^{\max}$	τ
1.6	1.6	3.8	11.5	11.5	44.9	1.925

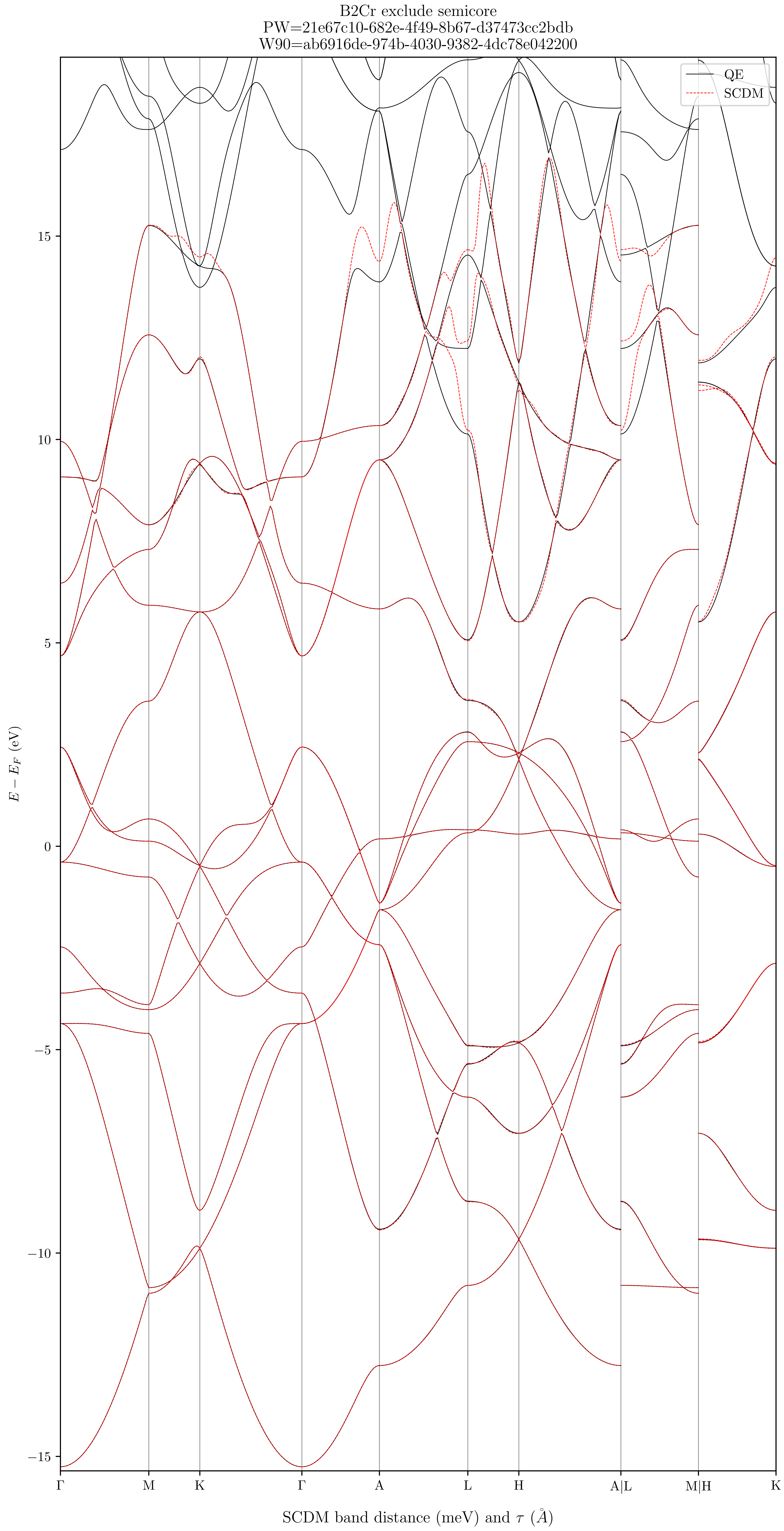

η_0	η_2	η_4	$\eta_0^{\max}$	$\eta_2^{\max}$	$\eta_4^{\max}$	τ
6.0	5.5	6.2	42.9	42.9	42.9	2.124

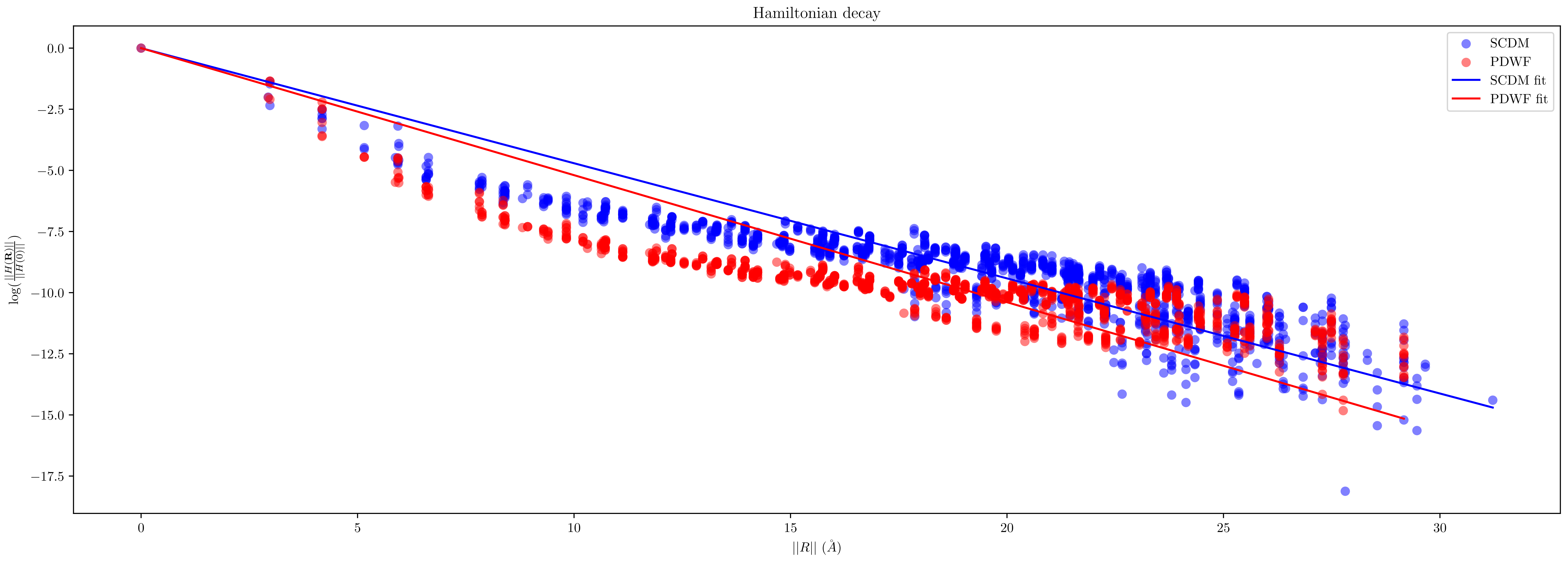

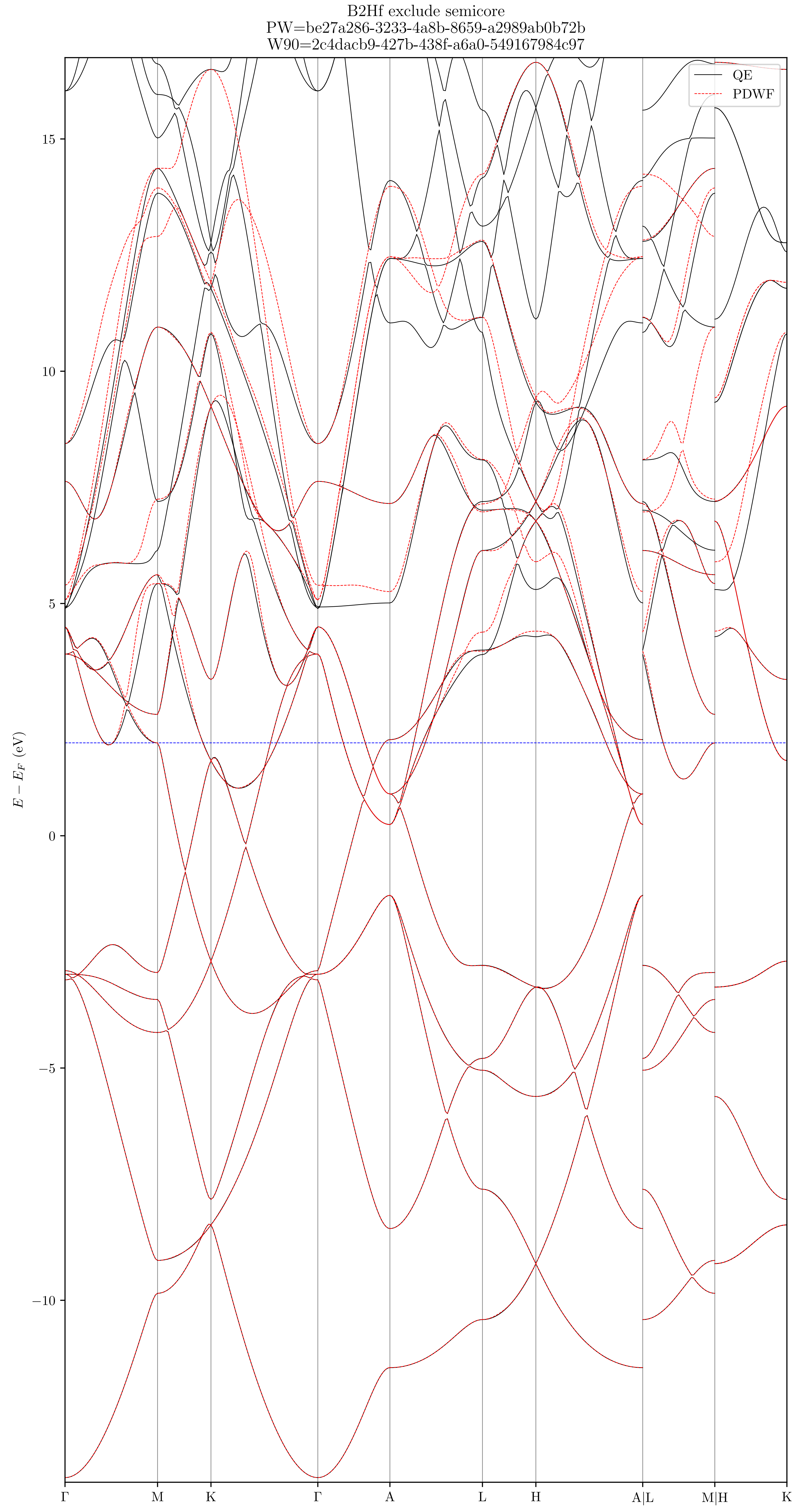

PDWF band distance (meV) and τ (Å)

η_0	η_2	η_4	$\eta_0^{\max}$	$\eta_2^{\max}$	$\eta_4^{\max}$	τ
3.3	3.9	31.6	14.4	21.7	510.7	2.095

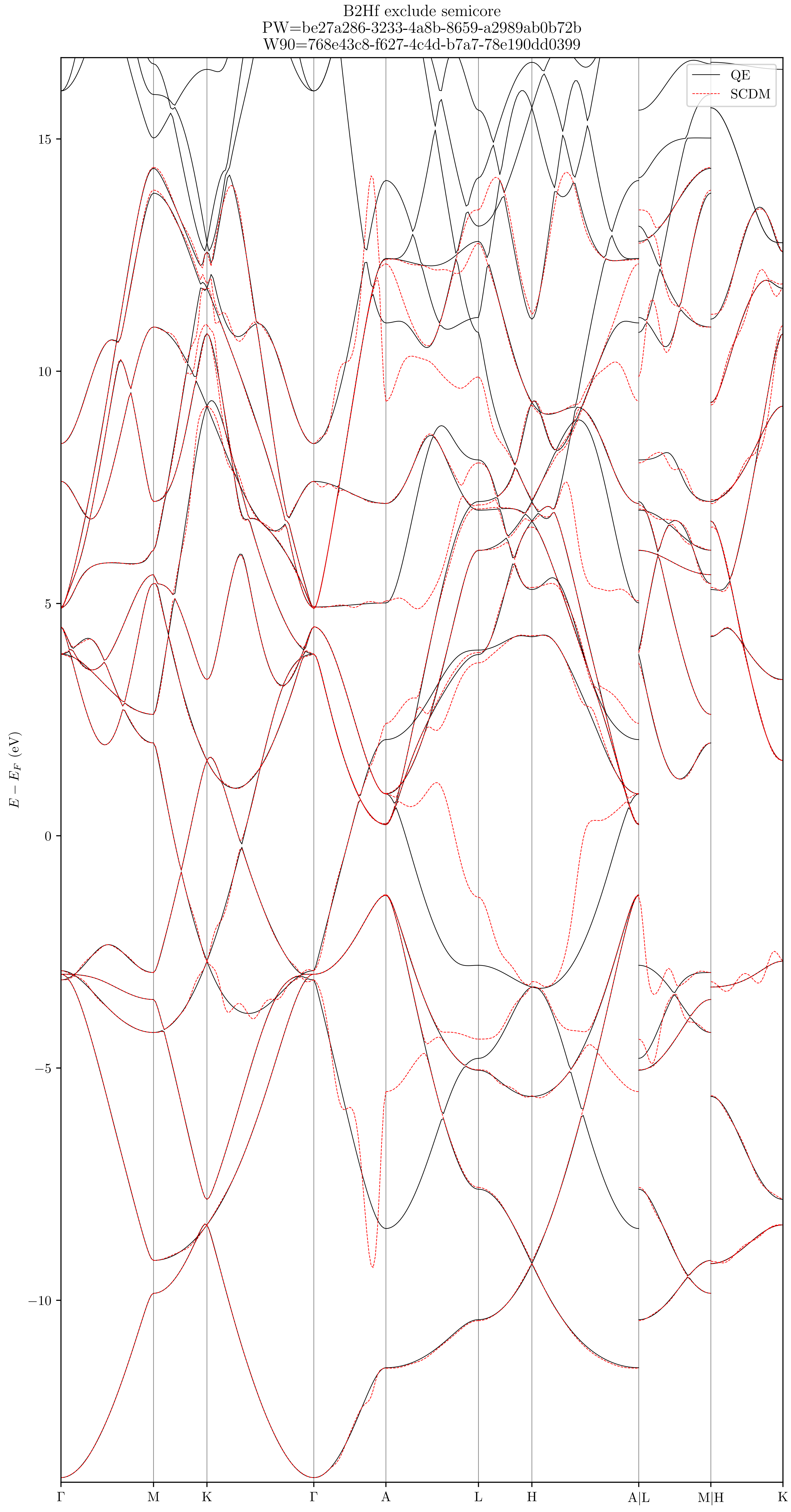

SCDM band distance (meV) and τ (Å)

η_0	η_2	η_4	$\eta_0^{\max}$	$\eta_2^{\max}$	$\eta_4^{\max}$	τ
549.5	586.3	530.4	2946.9	3327.4	3327.6	2.509

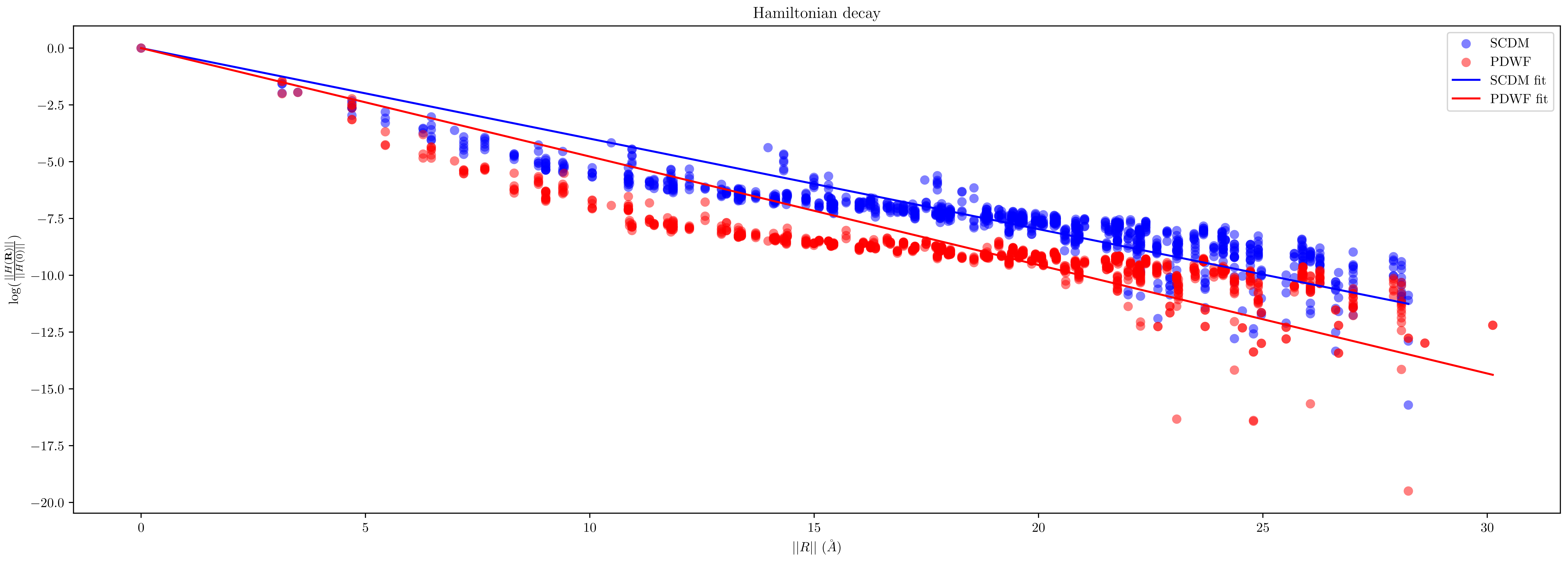

B2Mn exclude semicore
PW=1f23b5c4-a17a-4b9c-96f2-6976ab734028
W90=7dd1daef-69ae-447e-9741-5a7e8af9039e

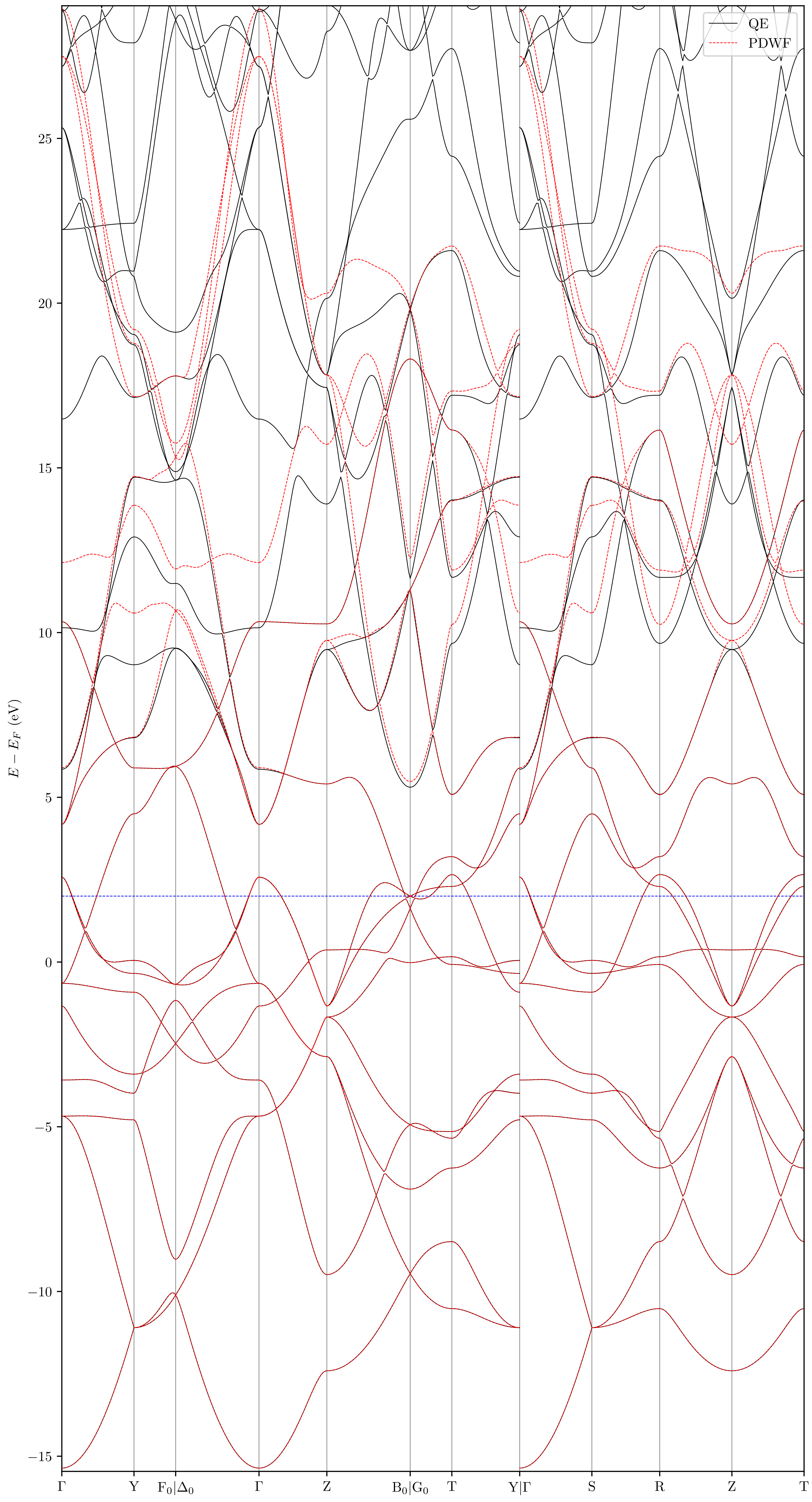

PDWF band distance (meV) and τ ($\AA$)

η_0	η_2	η_4	$\eta_0^{\max}$	$\eta_2^{\max}$	$\eta_4^{\max}$	τ
1.4	1.4	1.4	6.4	7.3	7.3	1.910

B2Mn exclude semicore
PW=1f23b5c4-a17a-4b9c-96f2-6976ab734028
W90=6bee2cd5-24c1-40a3-be43-42b0ceec2d4f

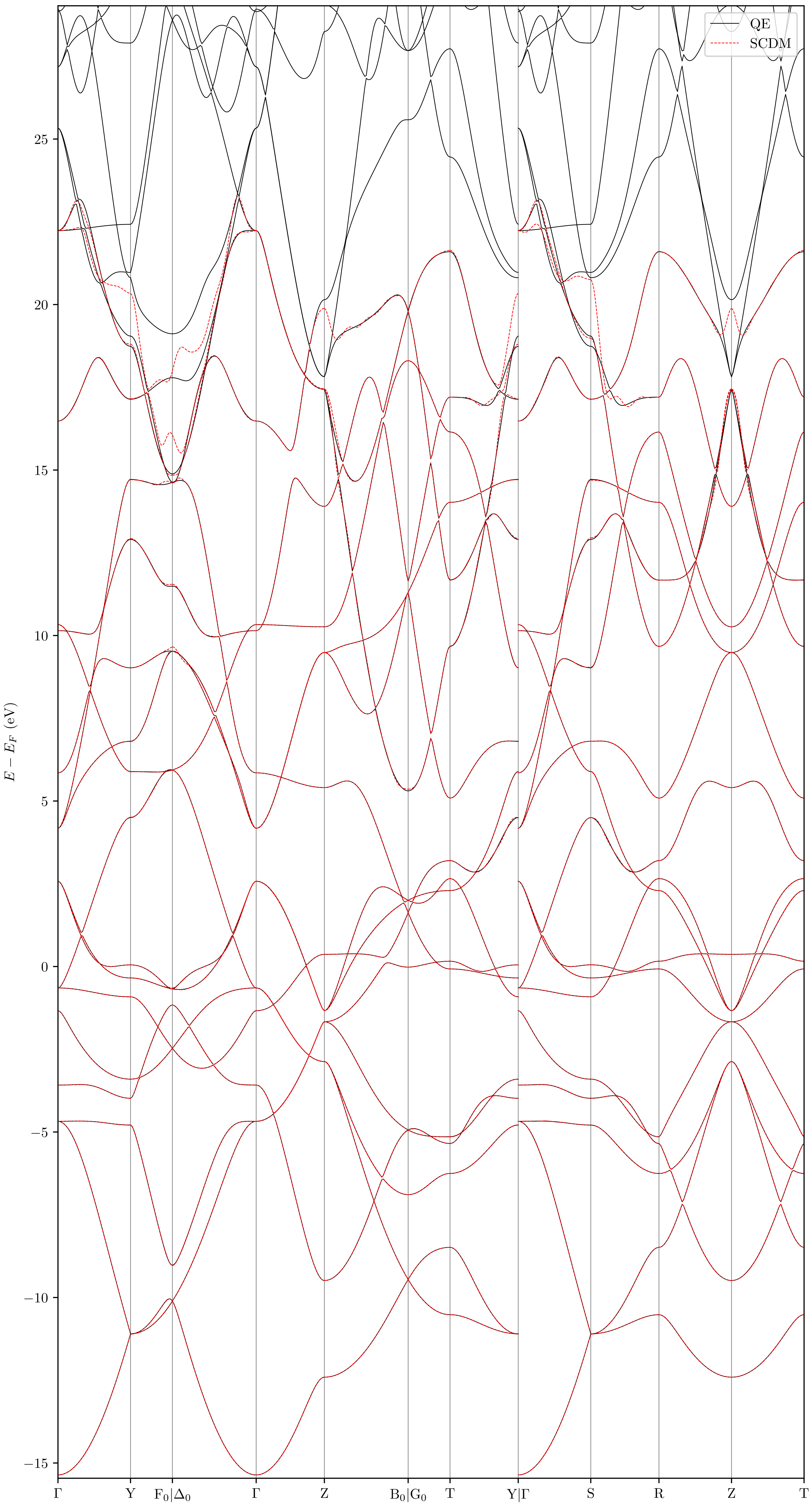

SCDM band distance (meV) and τ ($\AA$)

η_0	η_2	η_4	$\eta_0^{\max}$	$\eta_2^{\max}$	$\eta_4^{\max}$	τ
2.6	2.4	3.1	31.8	31.9	37.6	2.048

Hamiltonian decay

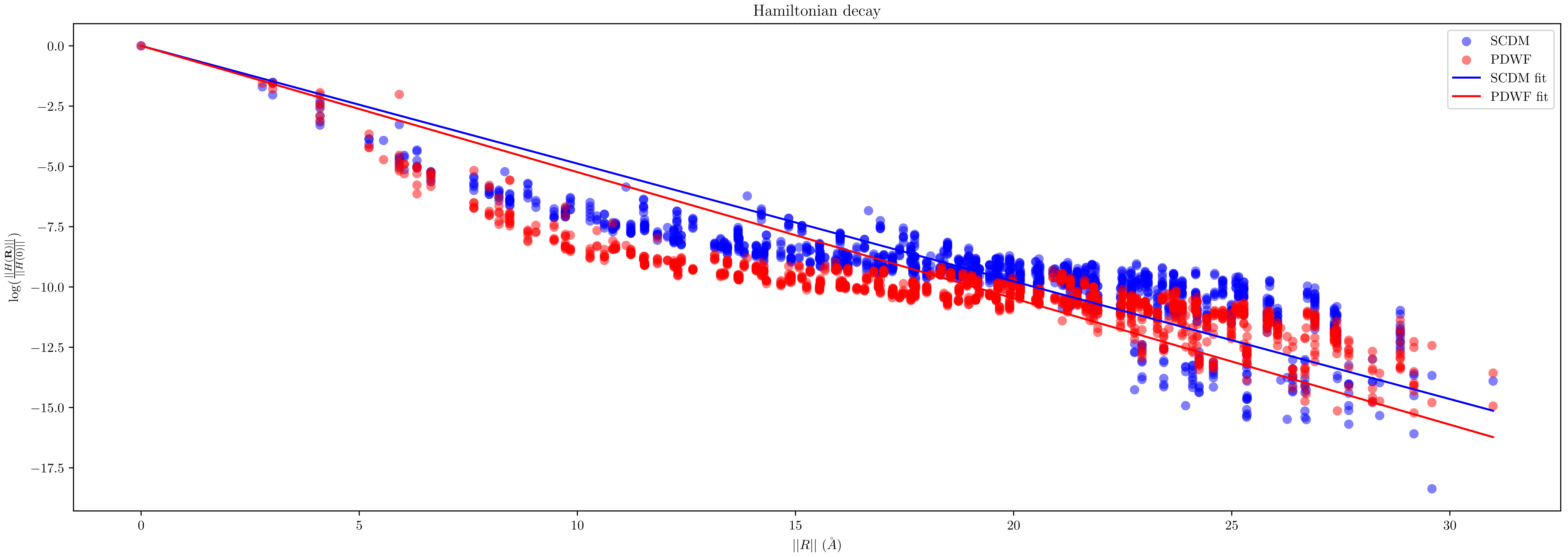

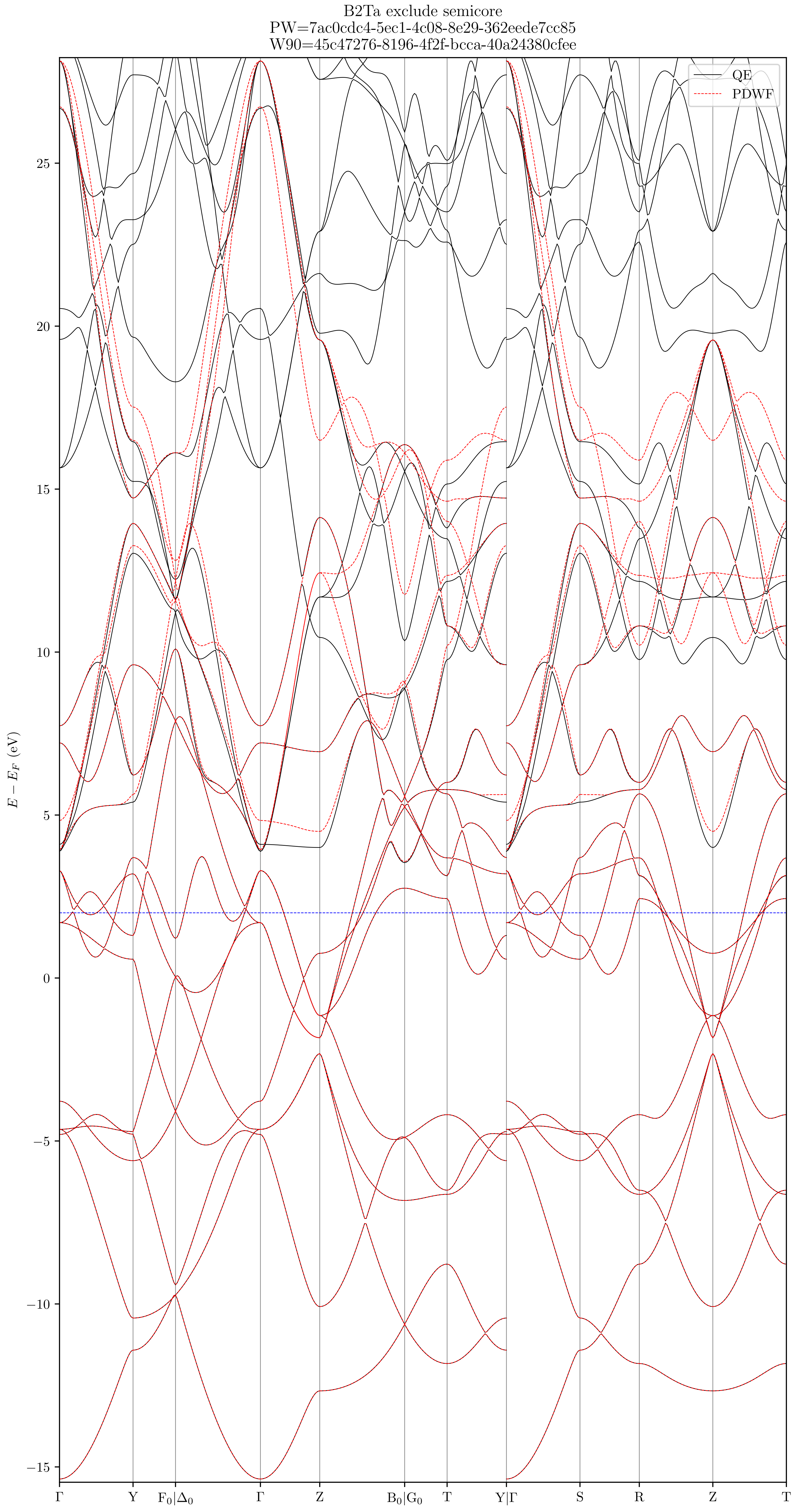

PDWF band distance (meV) and τ (Å)

η_0	η_2	η_4	η_0^{max}	η_2^{max}	η_4^{max}	τ
1.1	1.2	11.7	7.2	8.0	34.4	1.897

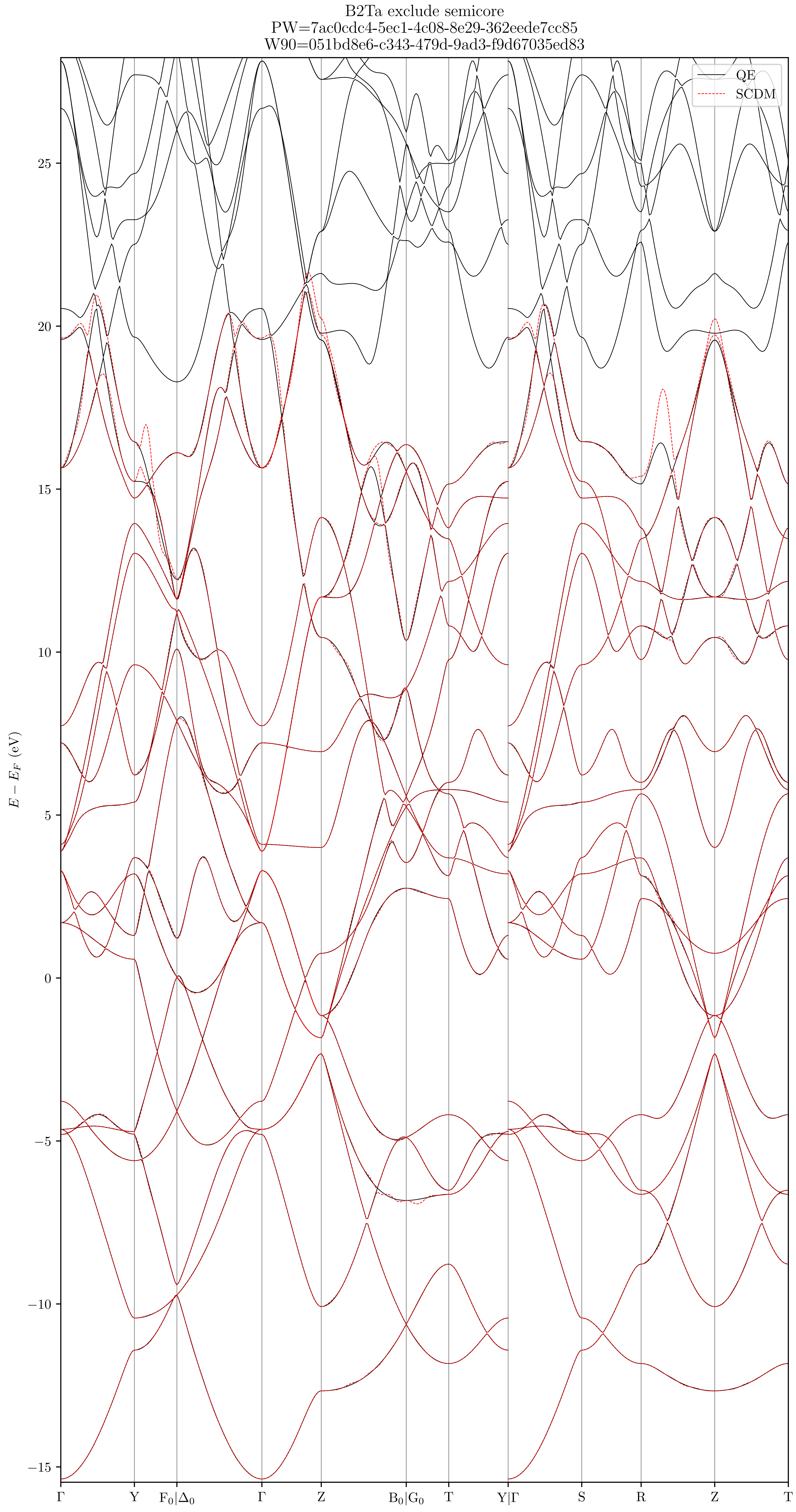

SCDM band distance (meV) and τ (Å)

η_0	η_2	η_4	η_0^{max}	η_2^{max}	η_4^{max}	τ
13.4	13.9	14.3	138.7	138.7	138.7	2.409

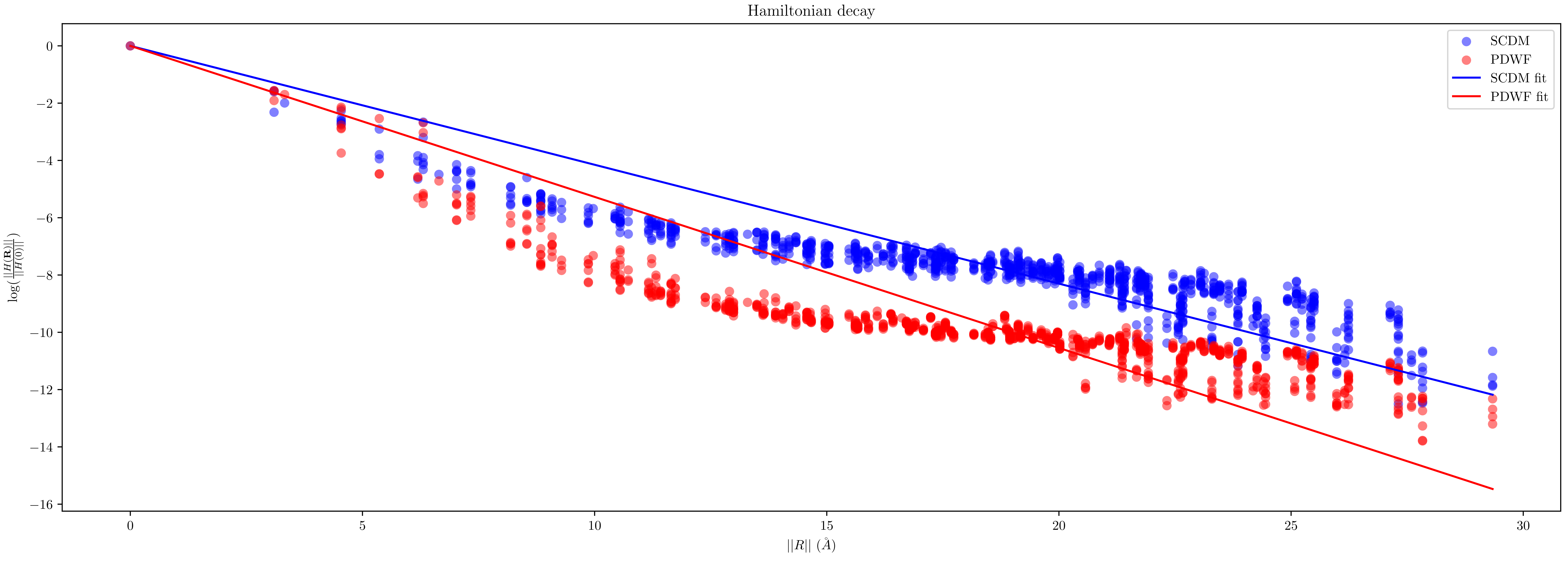

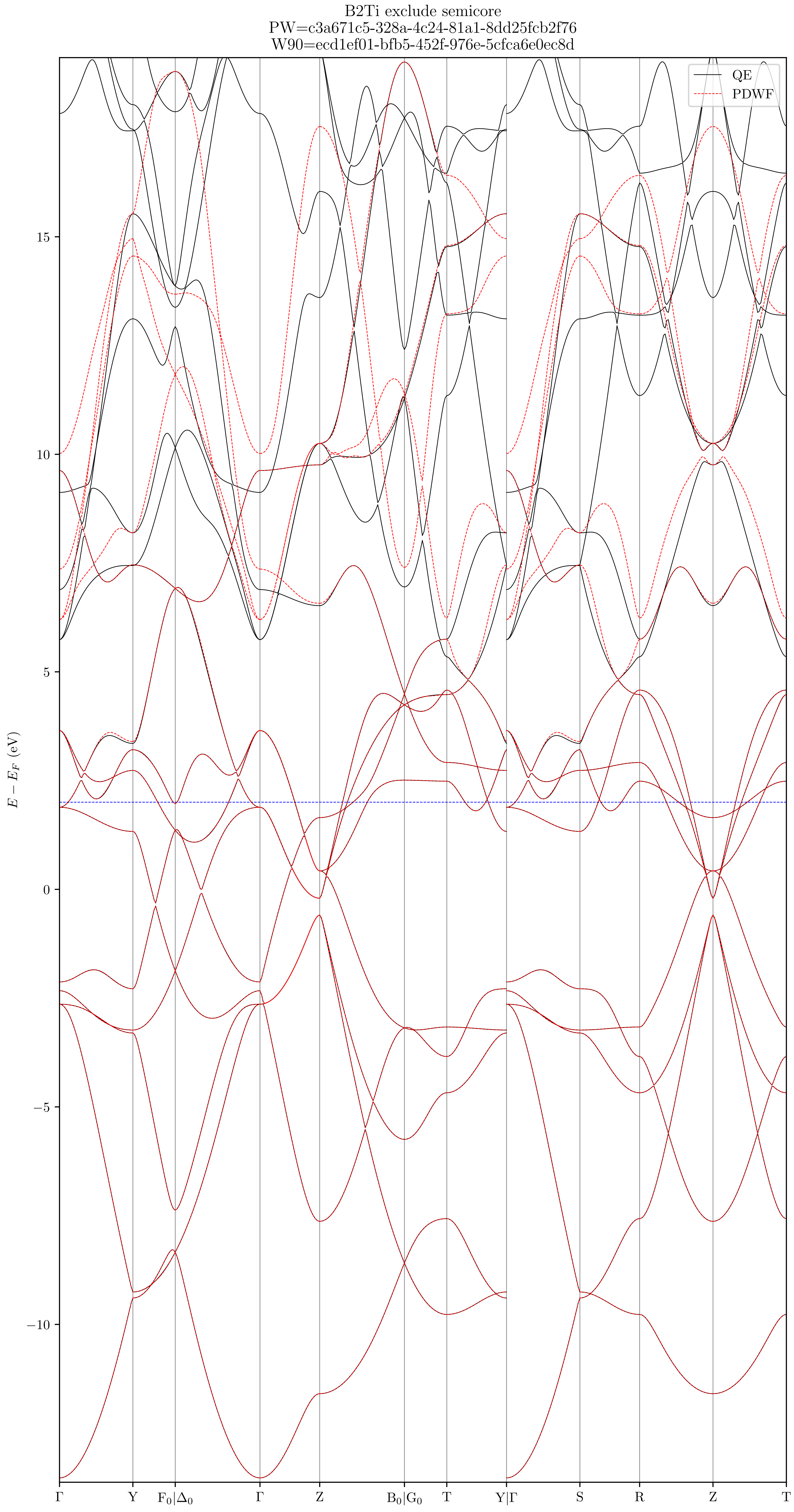

PDWF band distance (meV) and τ ($\AA$)

η_0	η_2	η_4	$\eta_0^{\max}$	$\eta_2^{\max}$	$\eta_4^{\max}$	τ
1.5	1.5	6.9	12.5	12.5	74.7	1.931

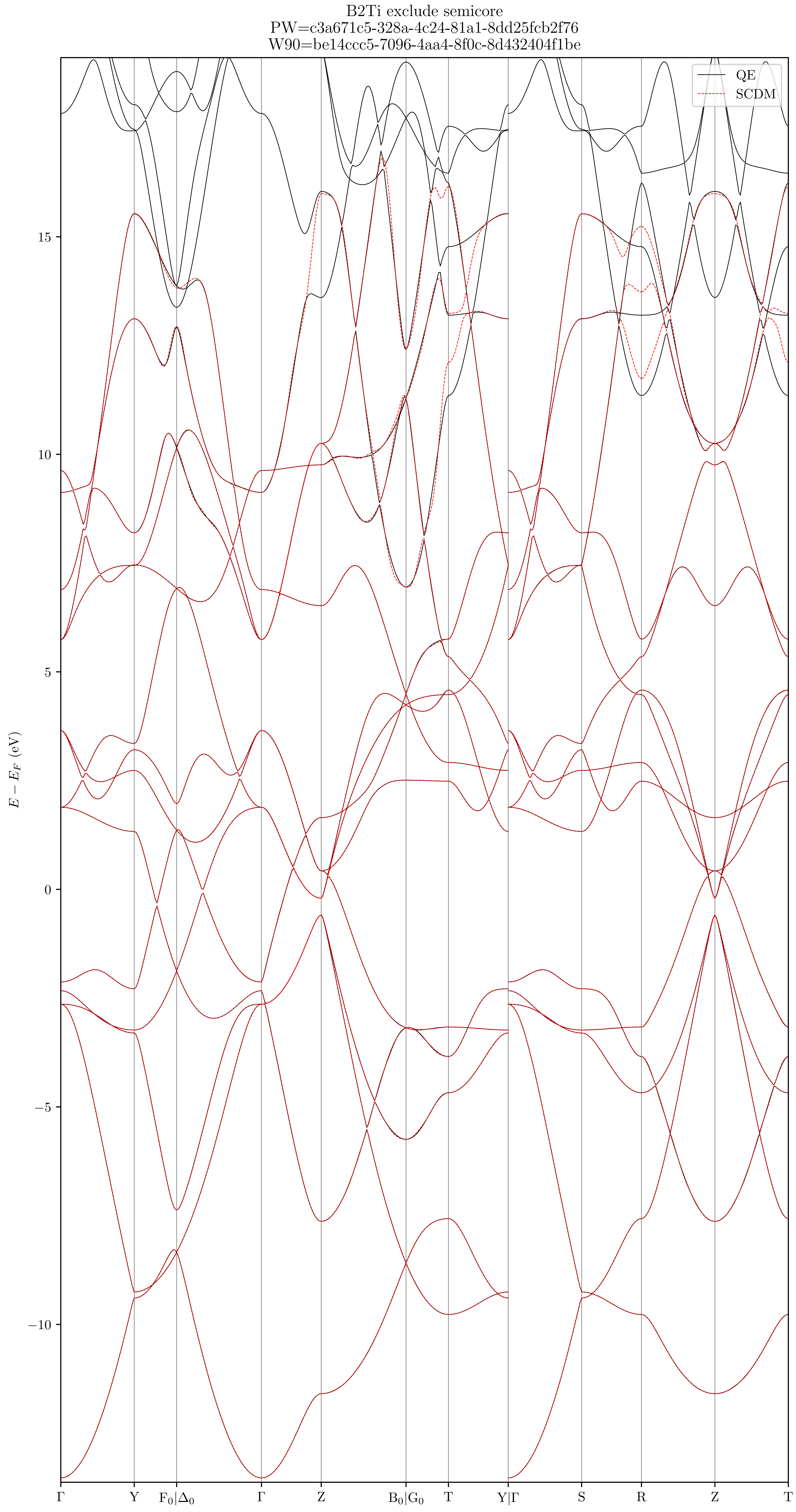

SCDM band distance (meV) and τ ($\AA$)

η_0	η_2	η_4	$\eta_0^{\max}$	$\eta_2^{\max}$	$\eta_4^{\max}$	τ
5.7	5.4	4.8	31.9	31.9	31.9	2.152

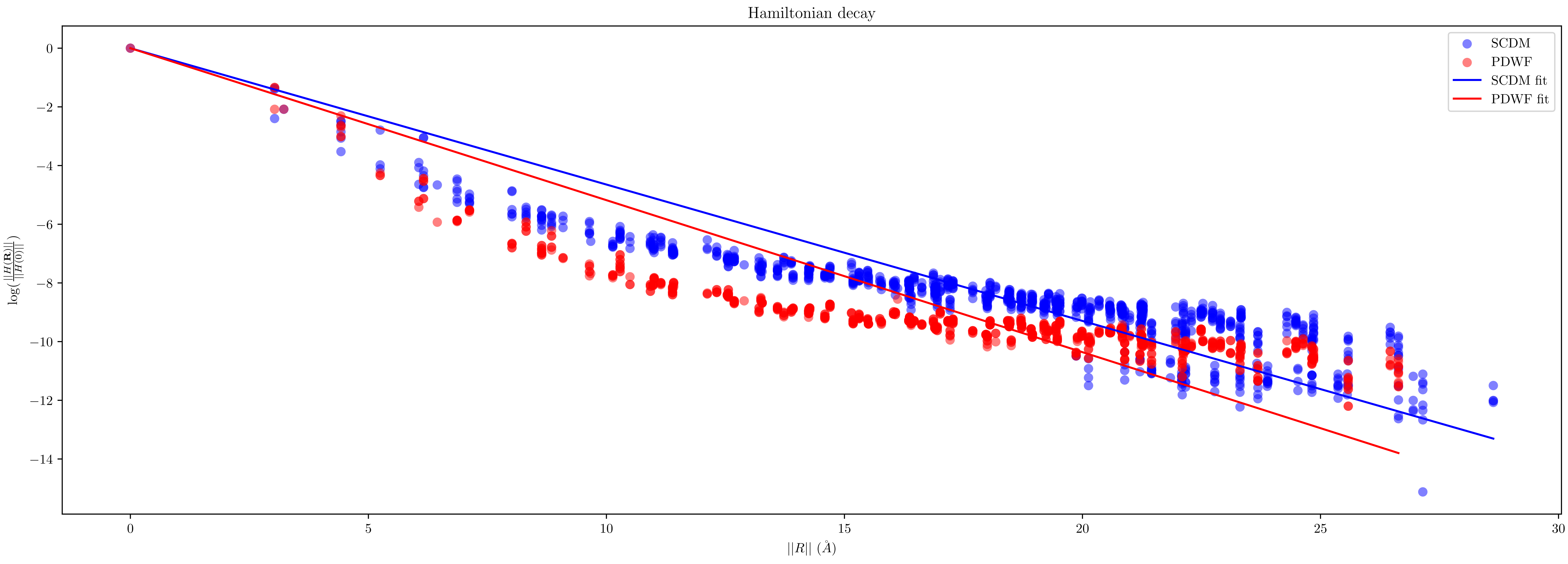

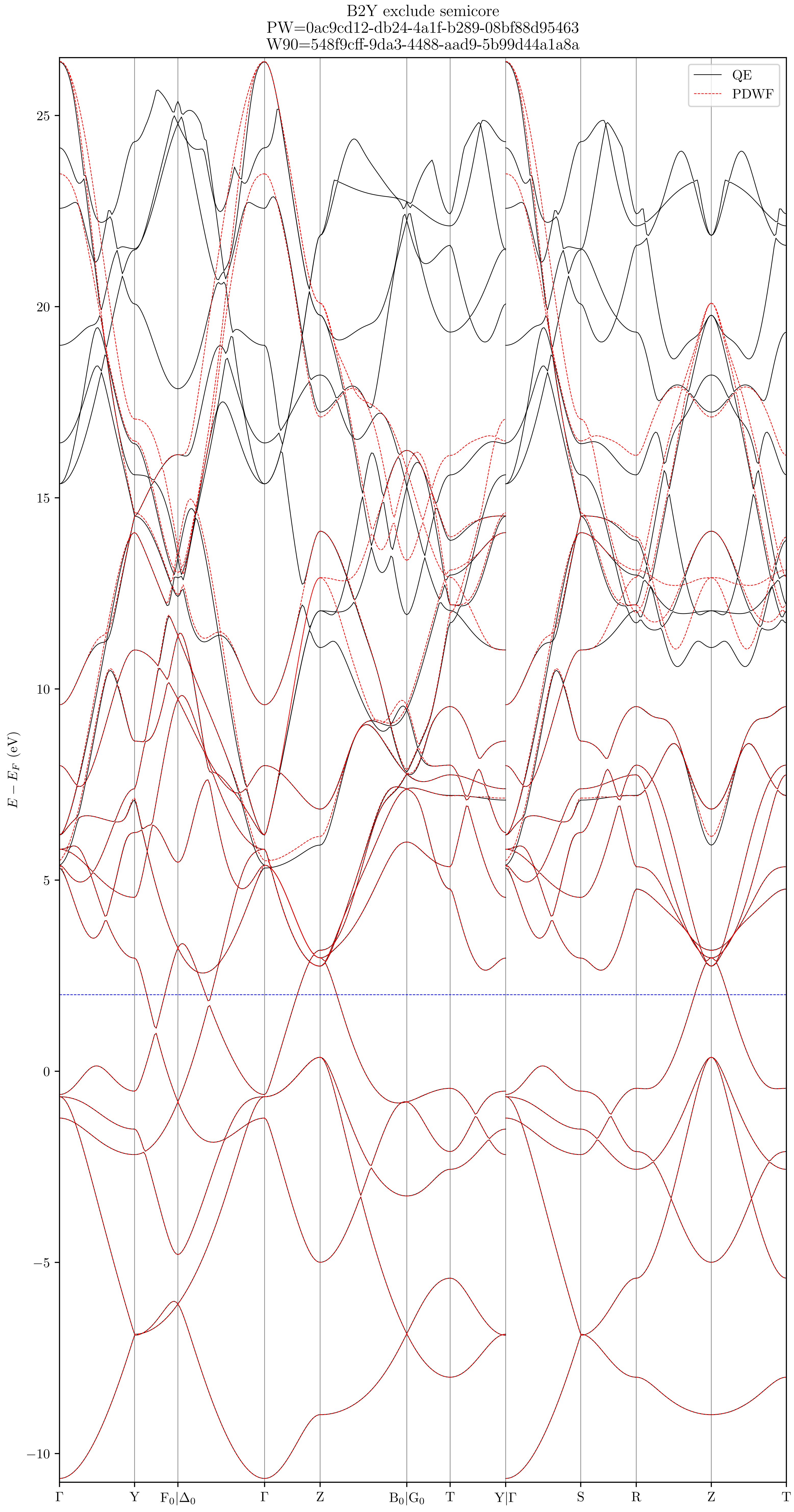

PDWF band distance (meV) and τ (Å)

η_0	η_2	η_4	η_0^{max}	η_2^{max}	η_4^{max}	τ
1.6	1.7	2.0	7.0	9.3	10.3	2.084

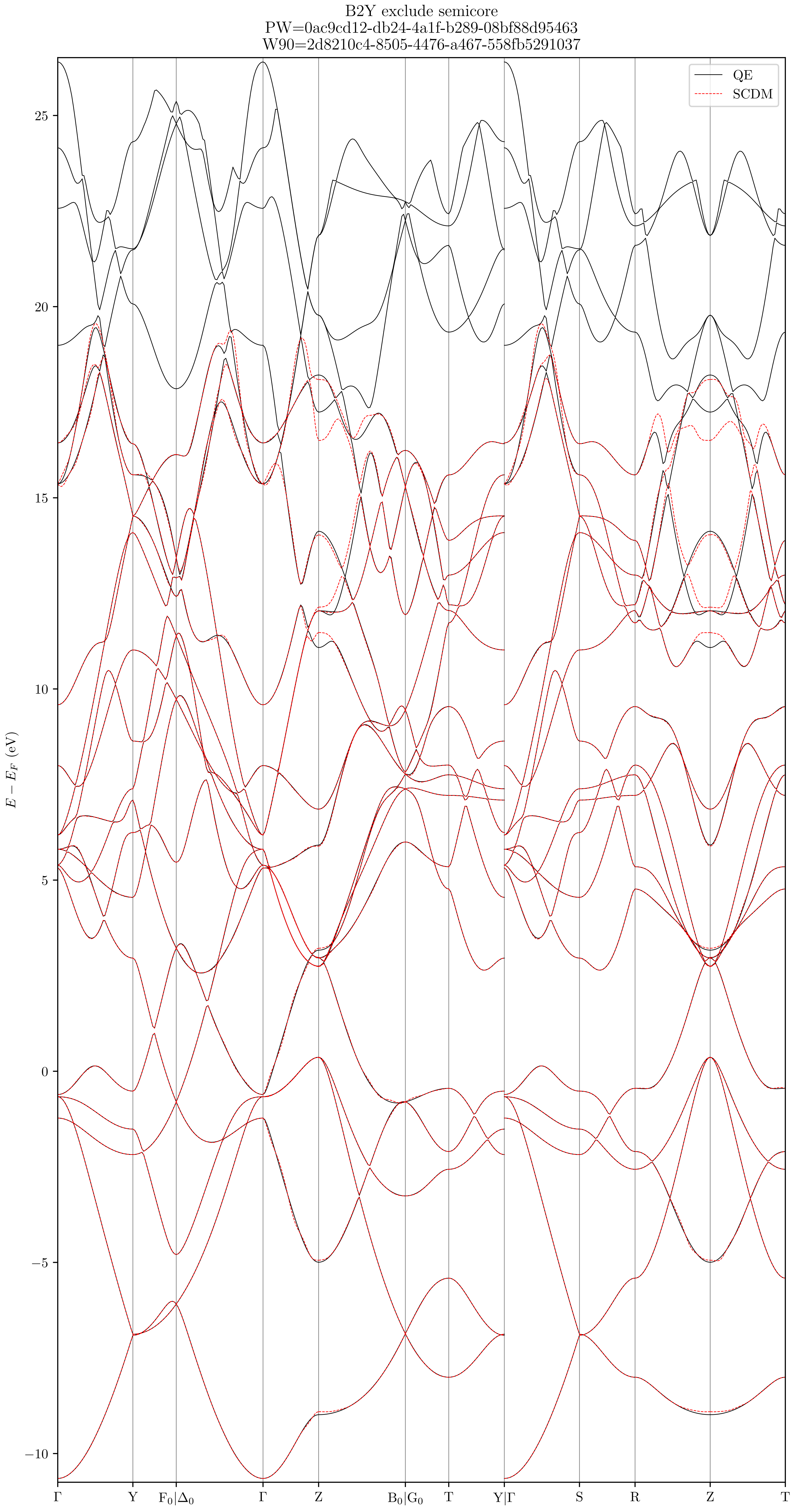

SCDM band distance (meV) and τ (Å)

η_0	η_2	η_4	η_0^{max}	η_2^{max}	η_4^{max}	τ
15.1	15.4	15.2	110.3	110.3	110.3	2.451

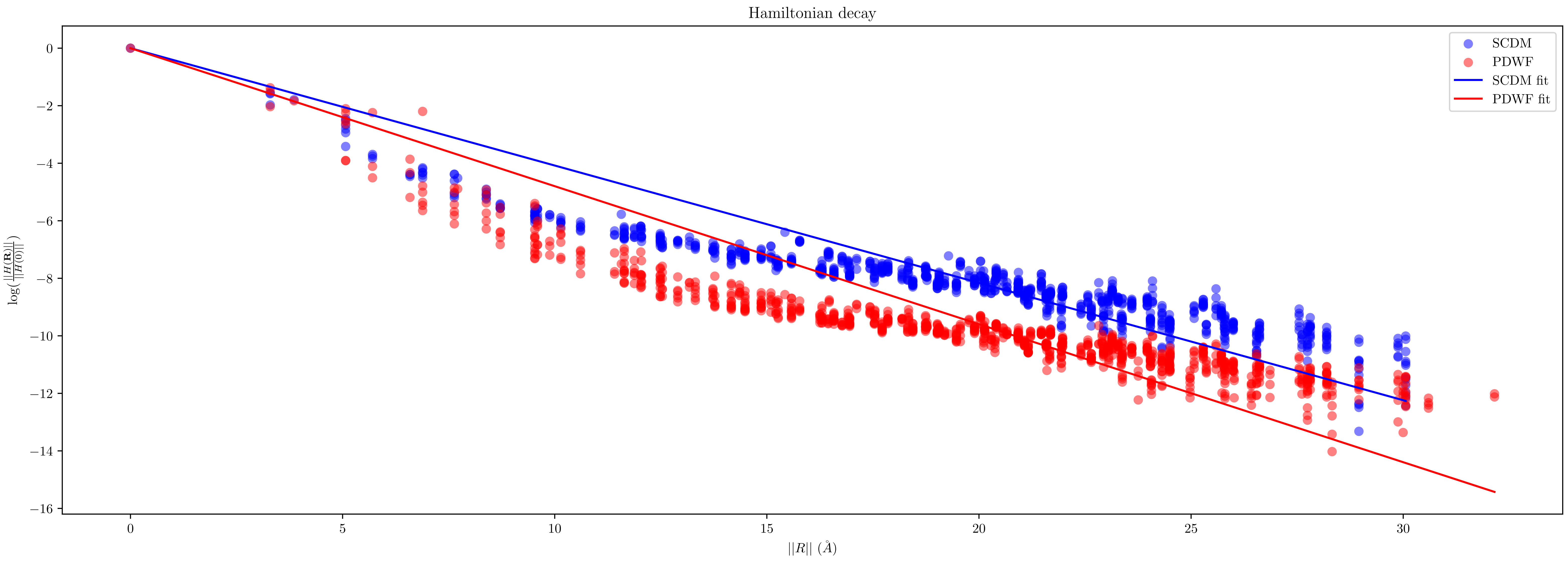

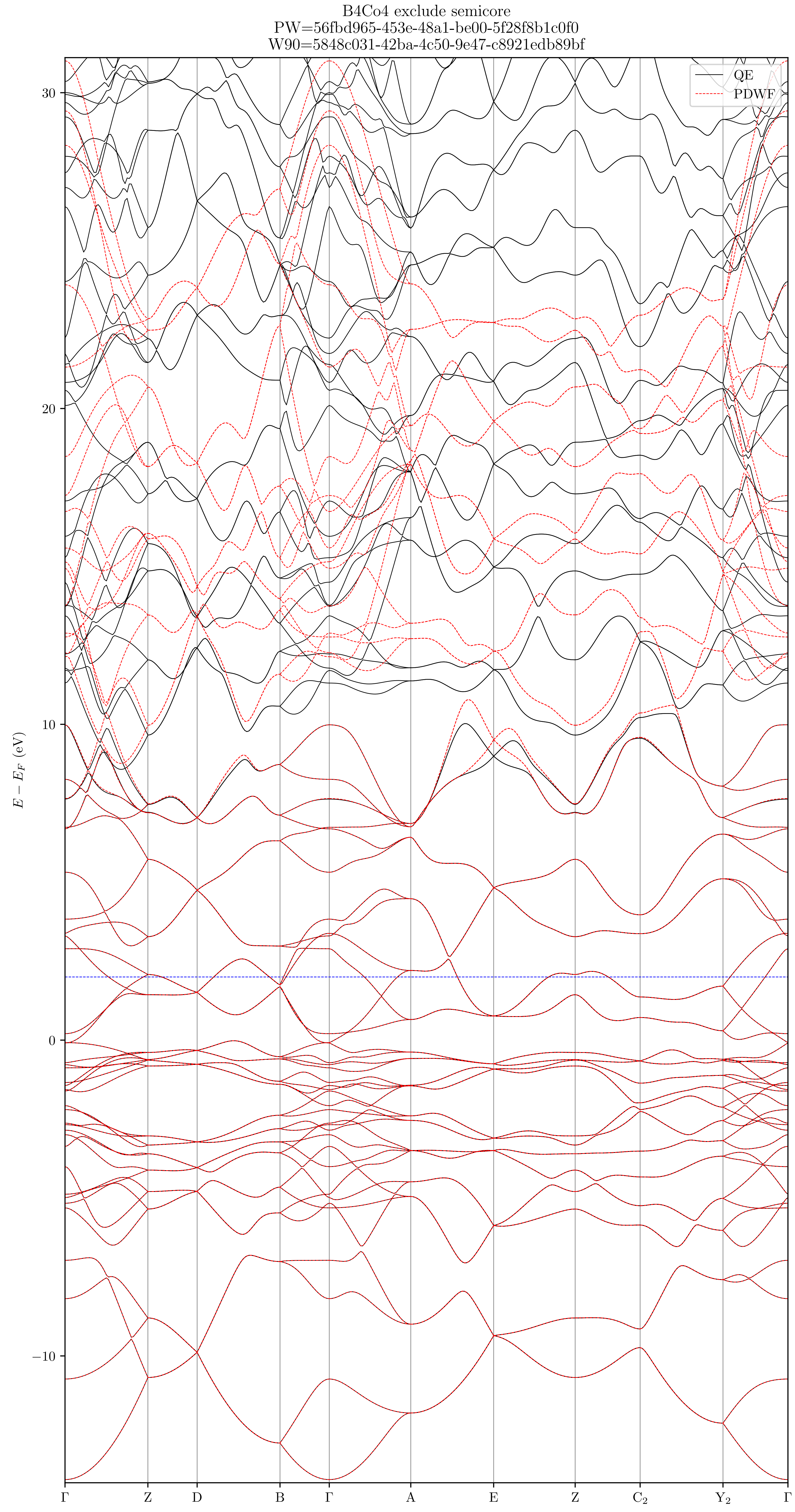

PDWF band distance (meV) and τ ($\AA$)

η_0	η_2	η_4	$\eta_0^{\max}$	$\eta_2^{\max}$	$\eta_4^{\max}$	τ
0.6	0.6	0.7	4.4	4.4	5.1	2.029

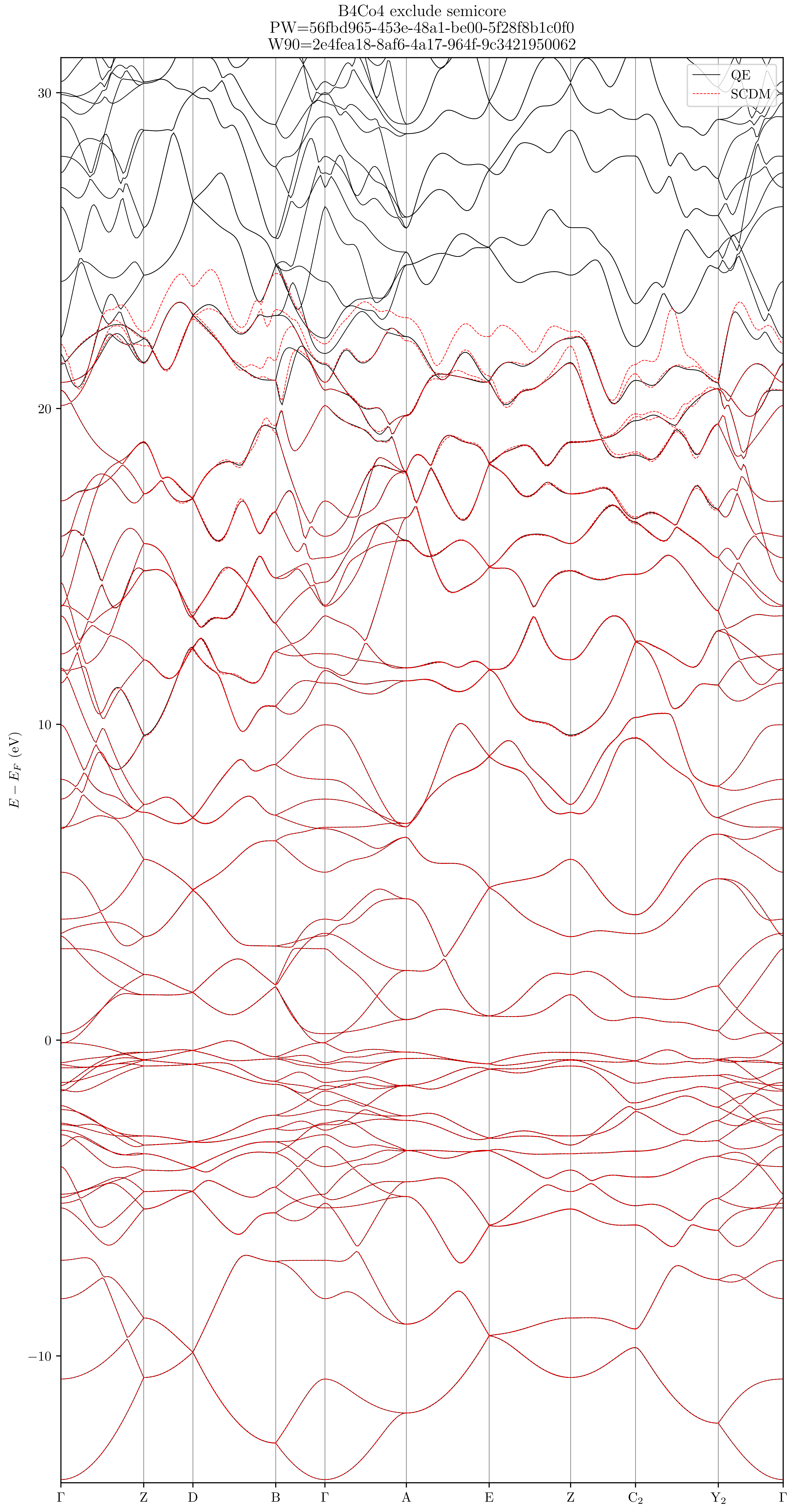

SCDM band distance (meV) and τ ($\AA$)

η_0	η_2	η_4	$\eta_0^{\max}$	$\eta_2^{\max}$	$\eta_4^{\max}$	τ
2.3	2.2	2.2	16.6	16.6	16.6	2.464

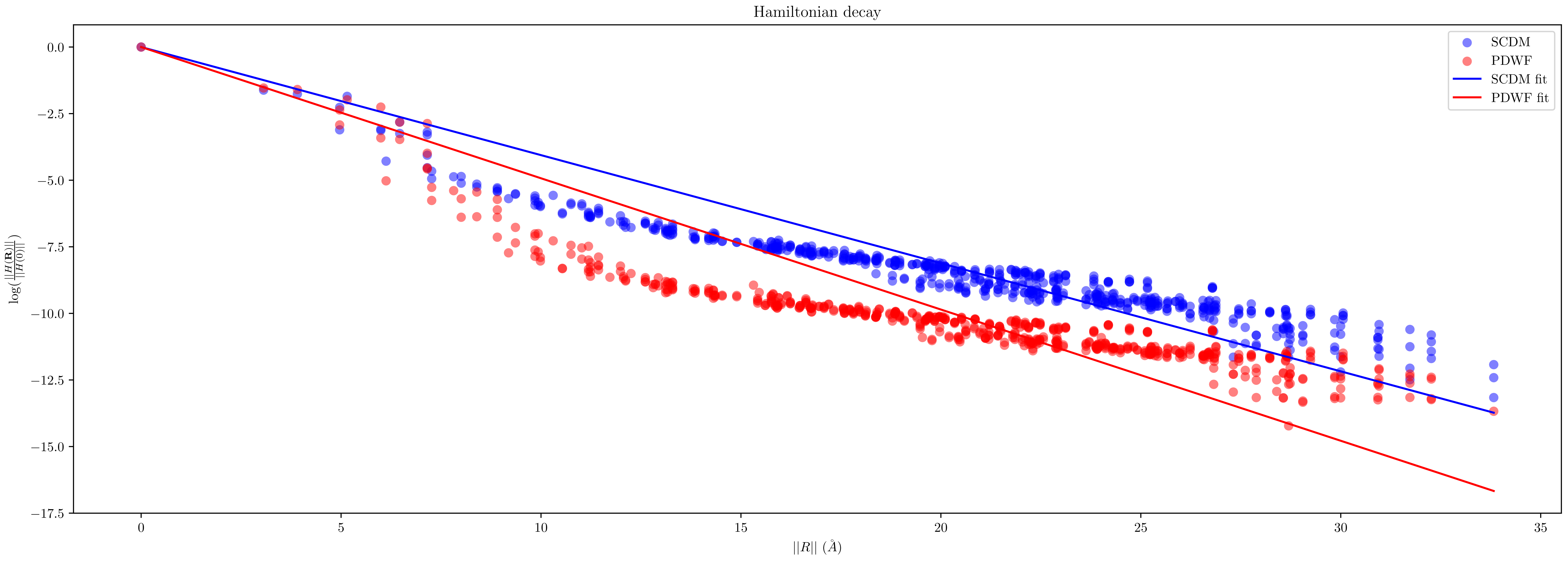

B4Ni4 exclude semicore
PW=81b84c87-f49c-46a1-9c0d-da21157cf47c
W90=77e39593-a244-4a39-9fc1-14092ca7a3b5

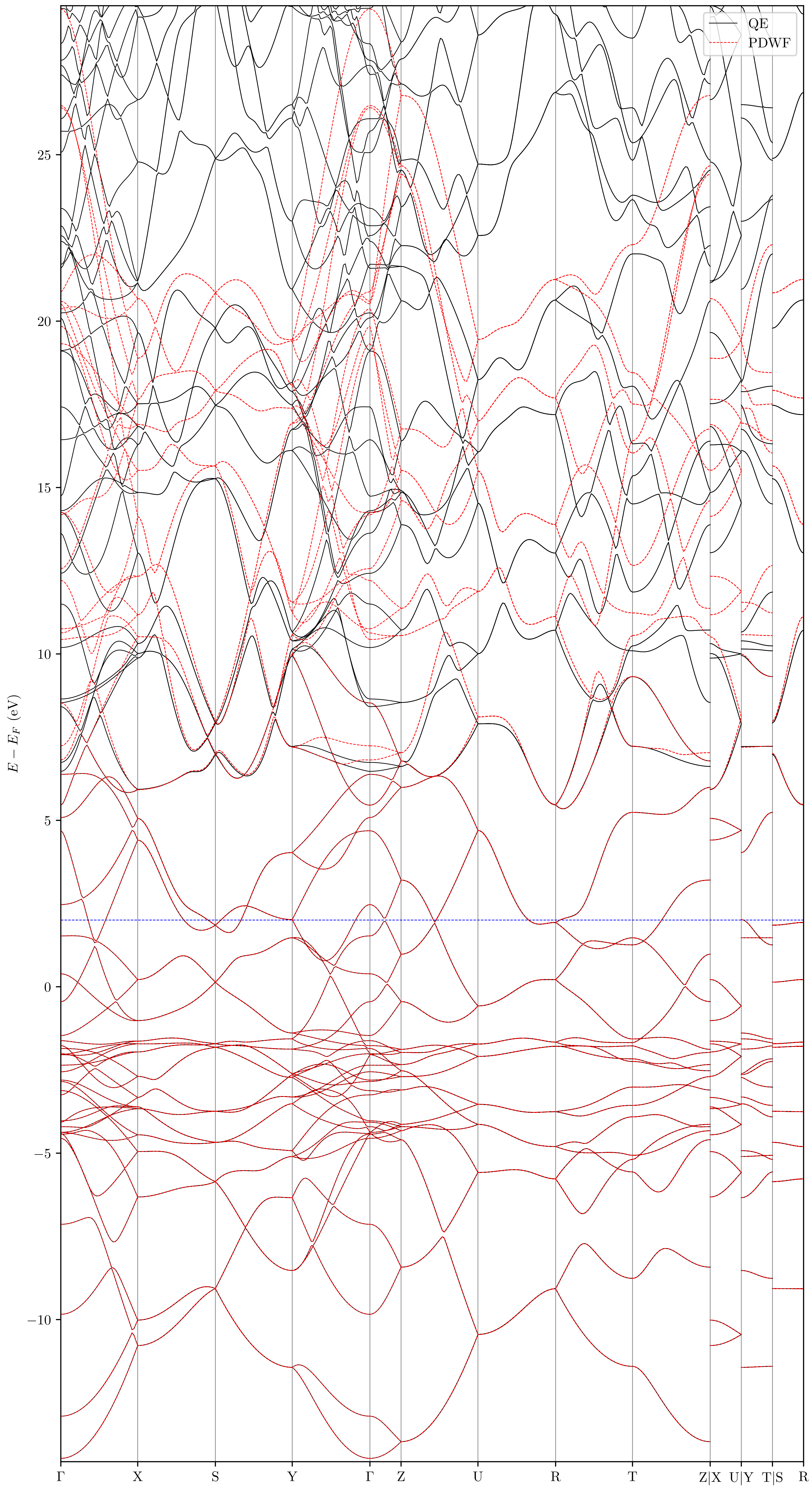

PDWF band distance (meV) and τ ($\AA$)

η_0	η_2	η_4	$\eta_0^{\max}$	$\eta_2^{\max}$	$\eta_4^{\max}$	τ
0.9	1.0	1.0	5.5	6.4	8.2	2.104

B4Ni4 exclude semicore
PW=81b84c87-f49c-46a1-9c0d-da21157cf47c
W90=4203a12d-503a-482b-b04a-75bd52191c64

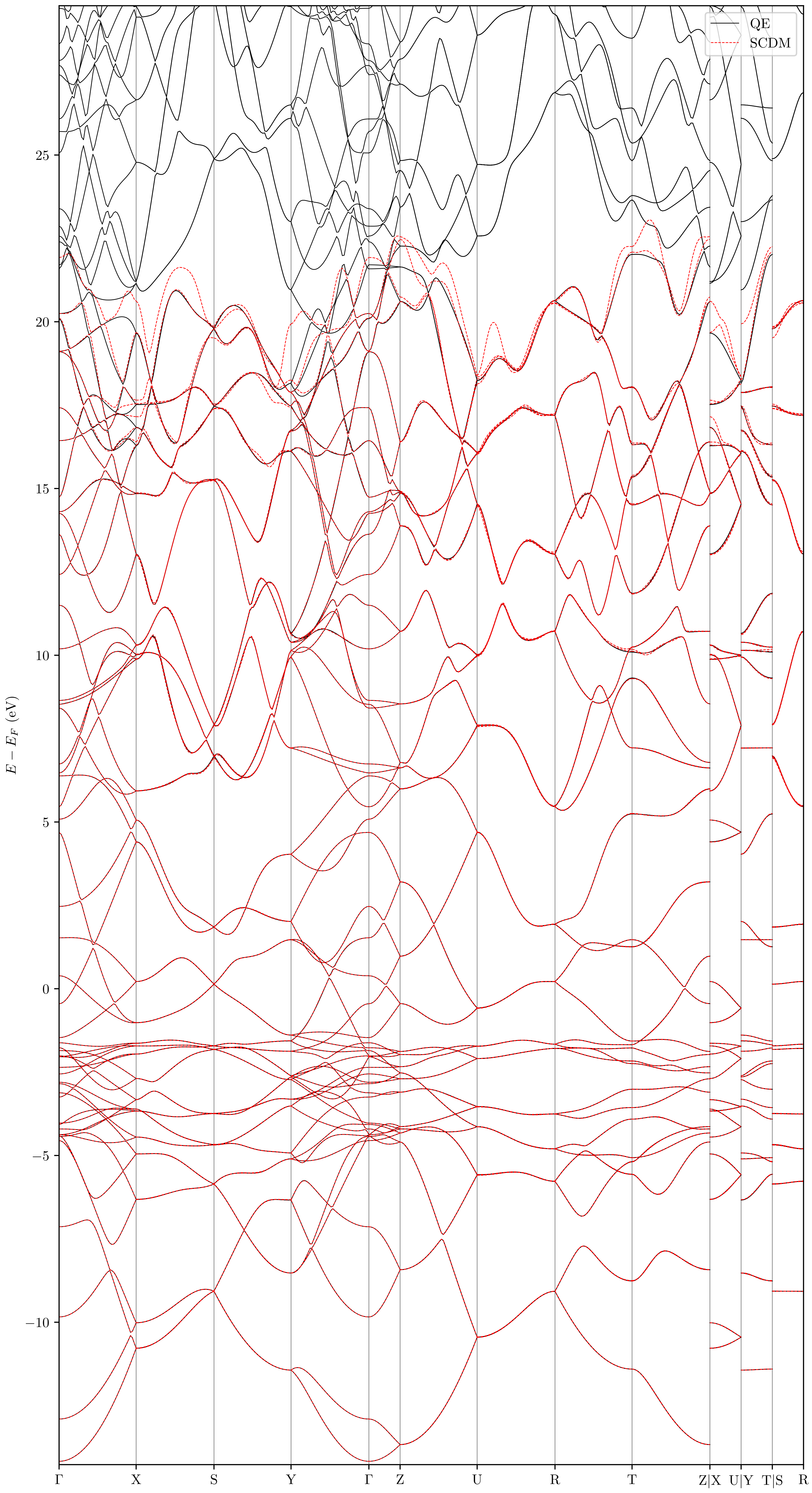

SCDM band distance (meV) and τ ($\AA$)

η_0	η_2	η_4	$\eta_0^{\max}$	$\eta_2^{\max}$	$\eta_4^{\max}$	τ
3.2	3.2	3.3	19.5	22.2	22.3	2.511

Hamiltonian decay

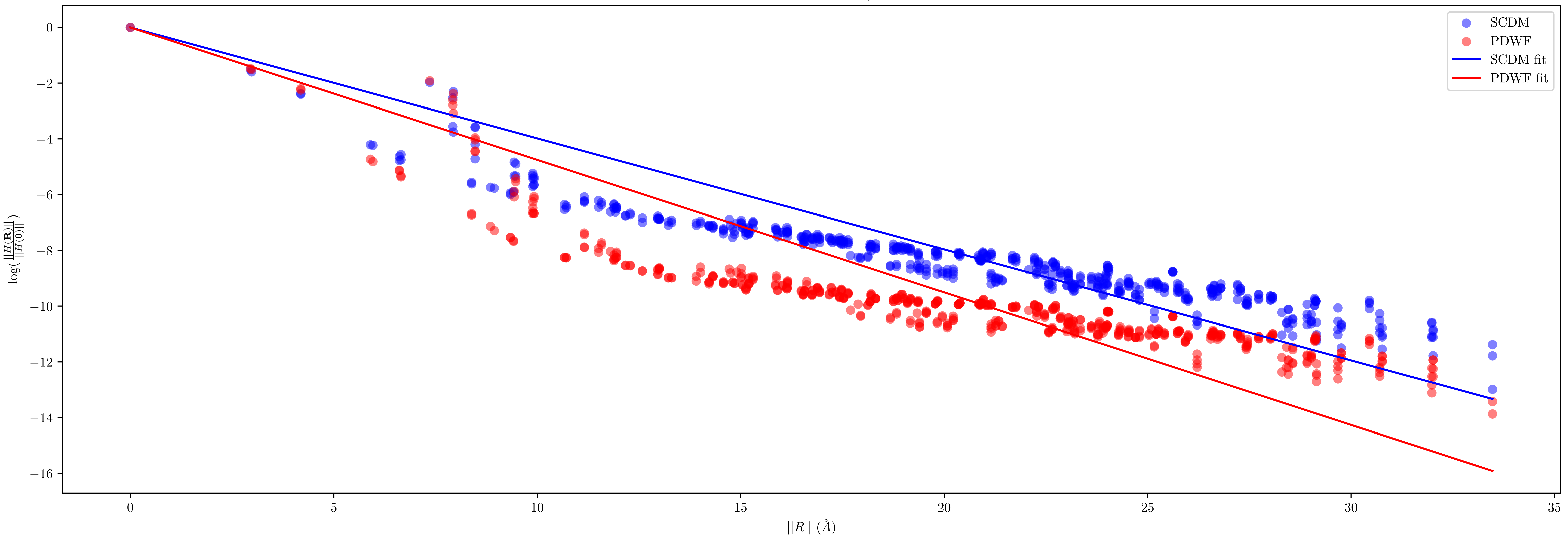

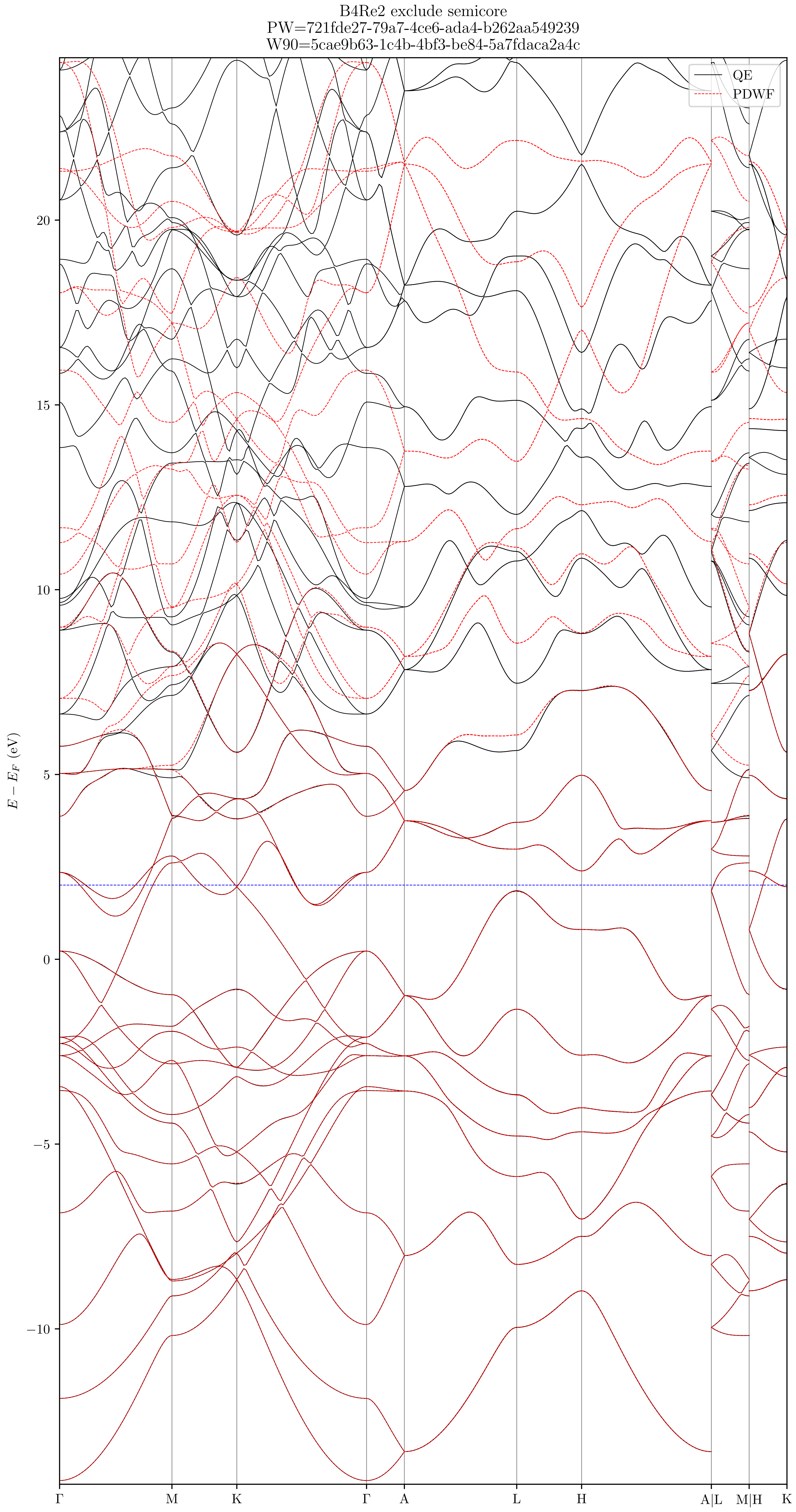

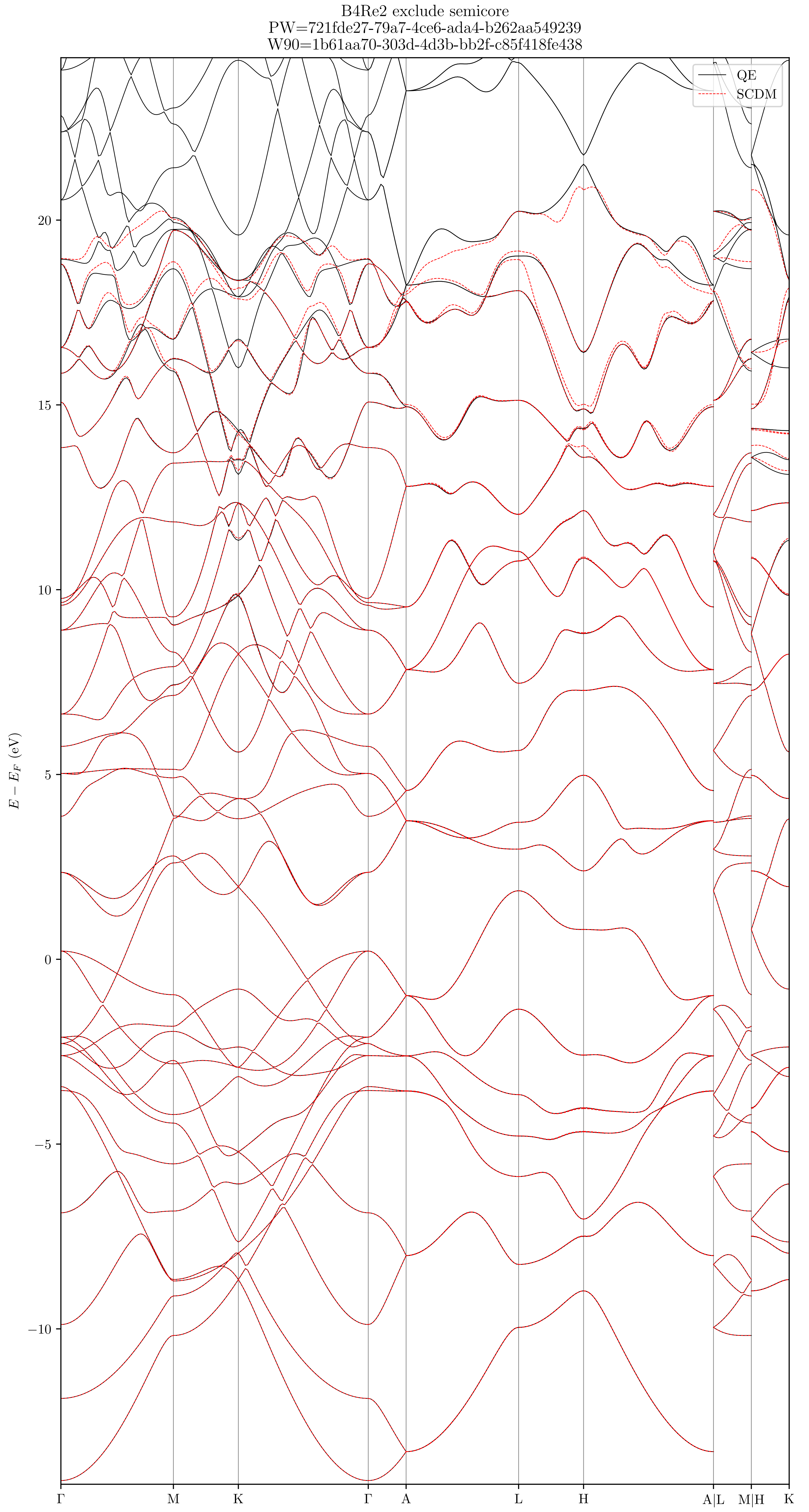

PDWF band distance (meV) and τ ($\AA$)

η_0	η_2	η_4	η_0^{max}	η_2^{max}	η_4^{max}	τ
3.1	3.3	3.4	22.3	22.3	22.3	2.256

SCDM band distance (meV) and τ ($\AA$)

η_0	η_2	η_4	η_0^{max}	η_2^{max}	η_4^{max}	τ
3.0	2.9	2.8	22.9	22.9	22.9	2.352

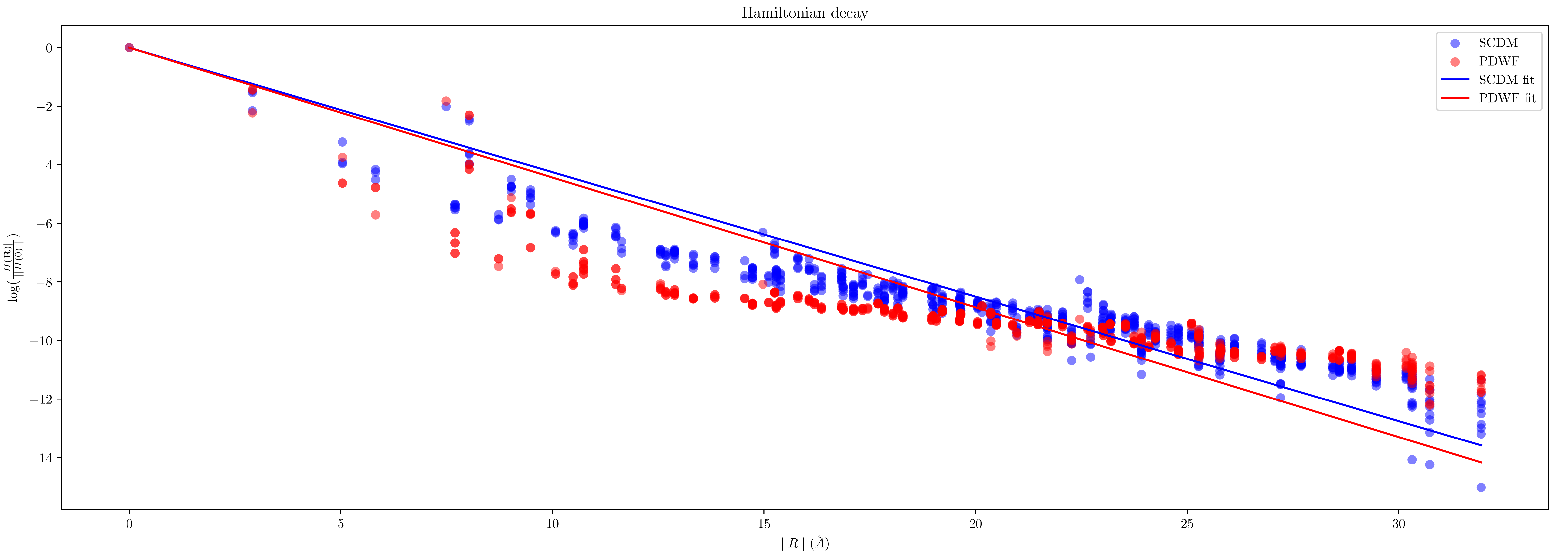

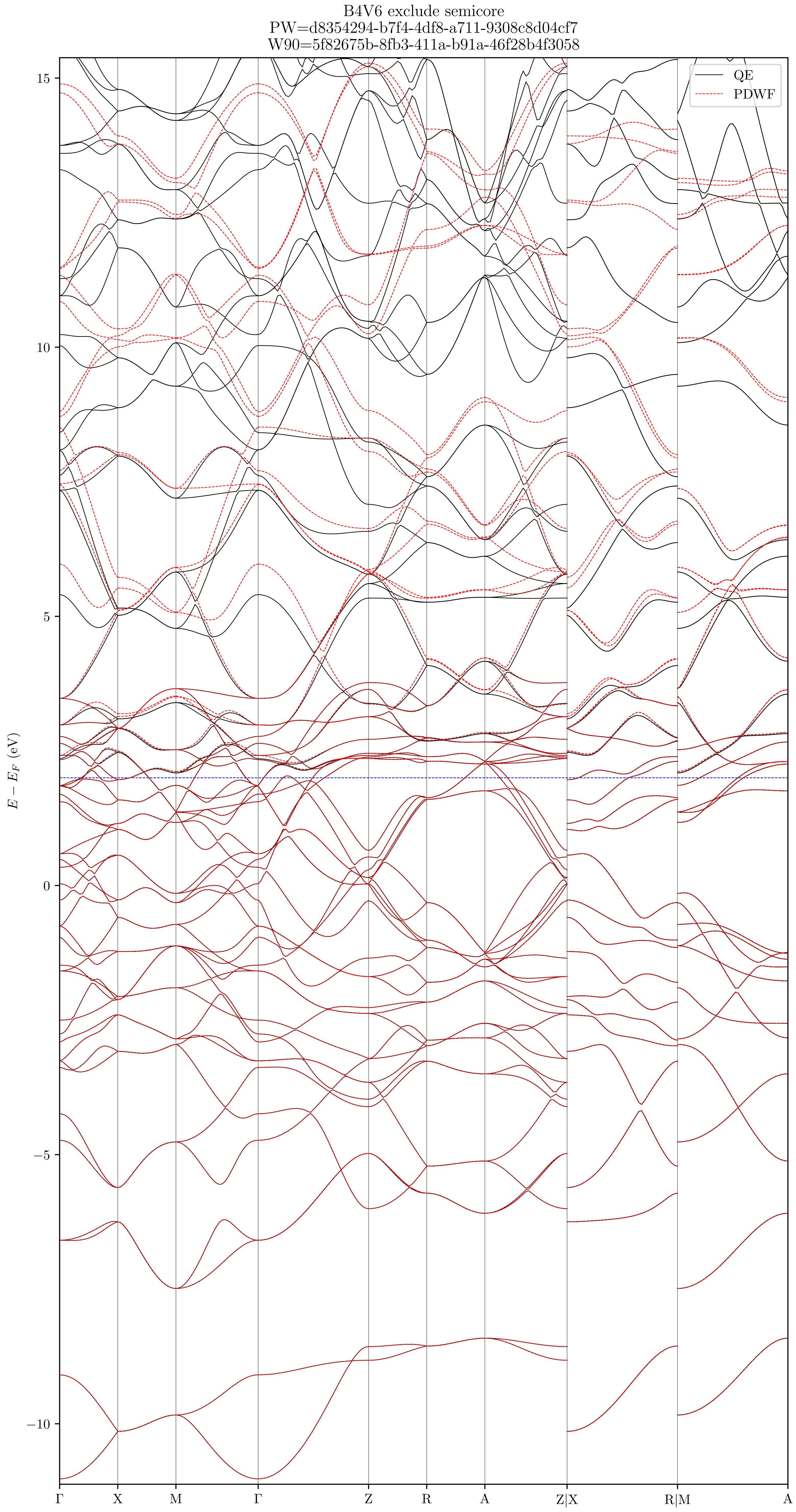

PDWF band distance (meV) and τ ($\AA$)

η_0	η_2	η_4	$\eta_0^{\max}$	$\eta_2^{\max}$	$\eta_4^{\max}$	τ
0.4	1.2	16.2	2.4	7.1	134.5	1.925

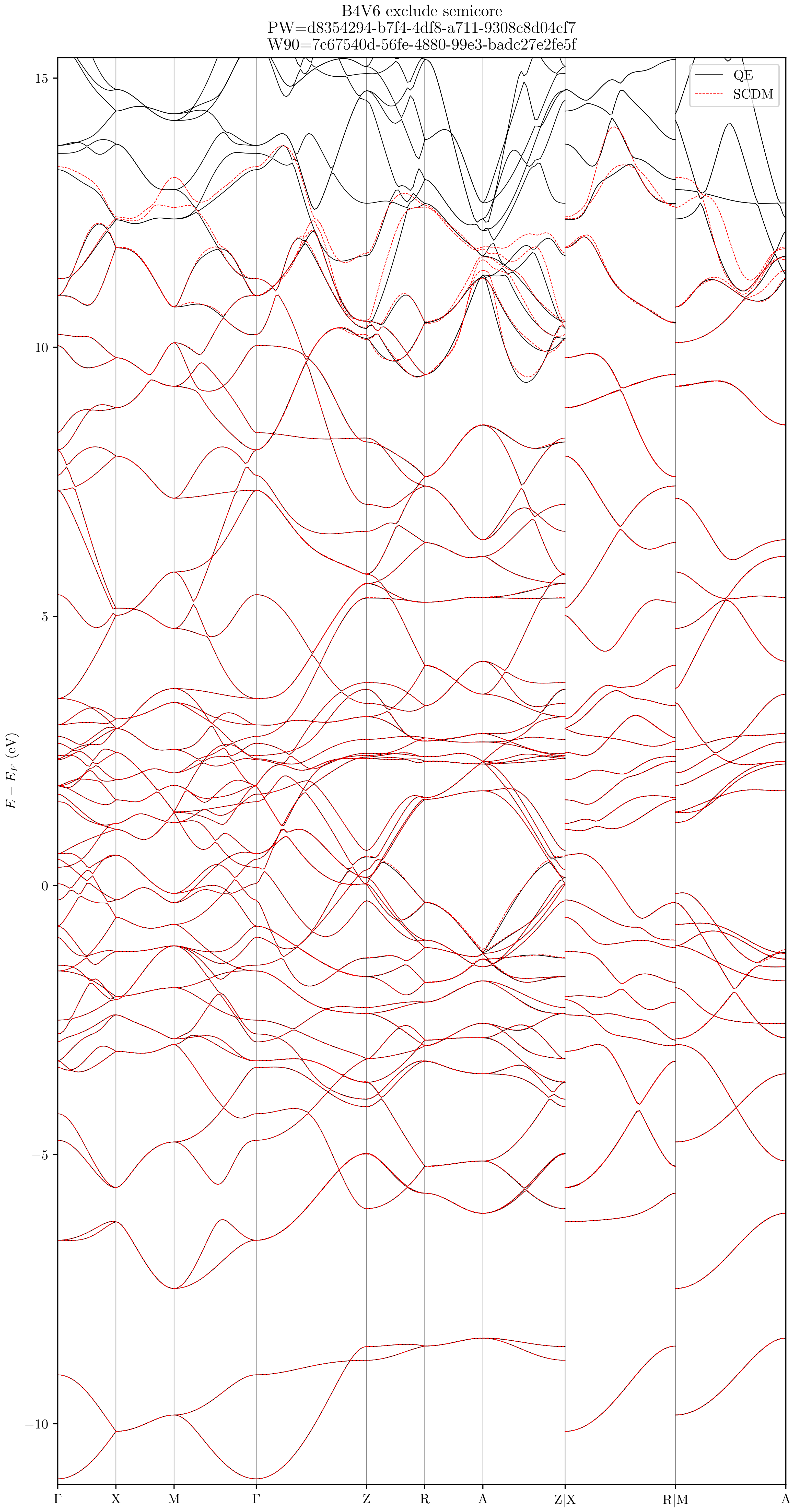

SCDM band distance (meV) and τ ($\AA$)

η_0	η_2	η_4	$\eta_0^{\max}$	$\eta_2^{\max}$	$\eta_4^{\max}$	τ
4.4	4.2	3.7	60.9	61.5	61.5	2.412

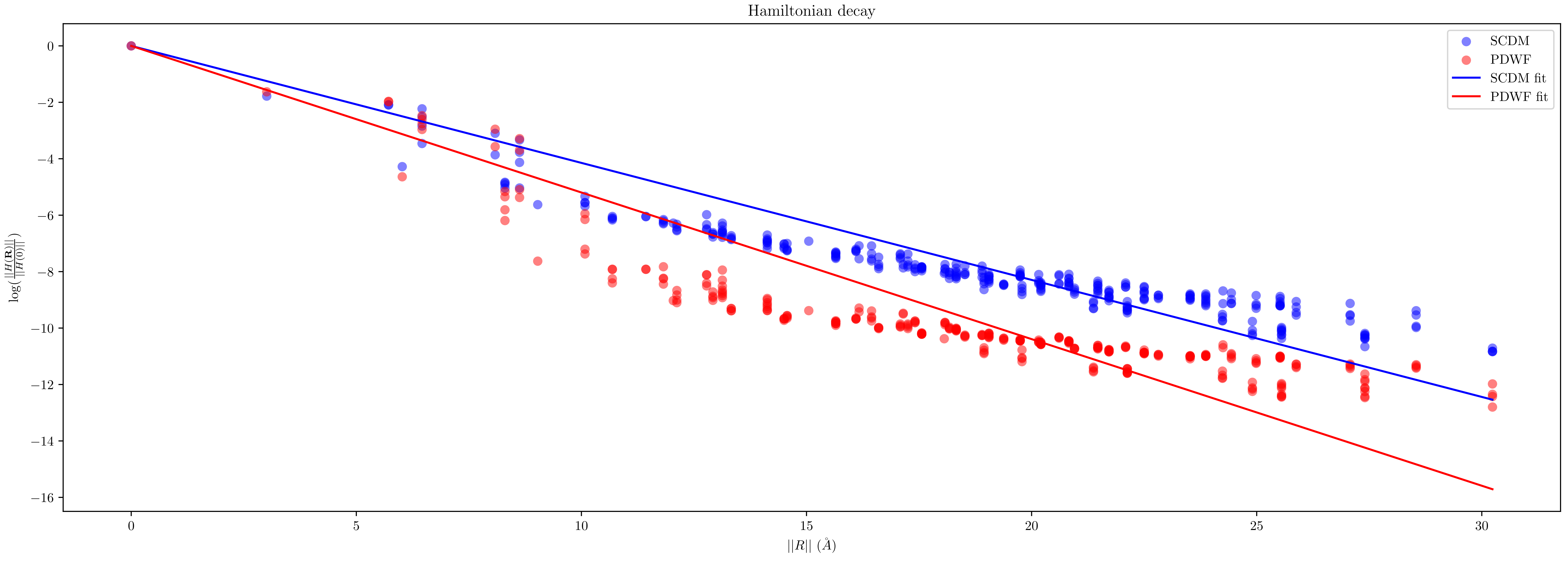

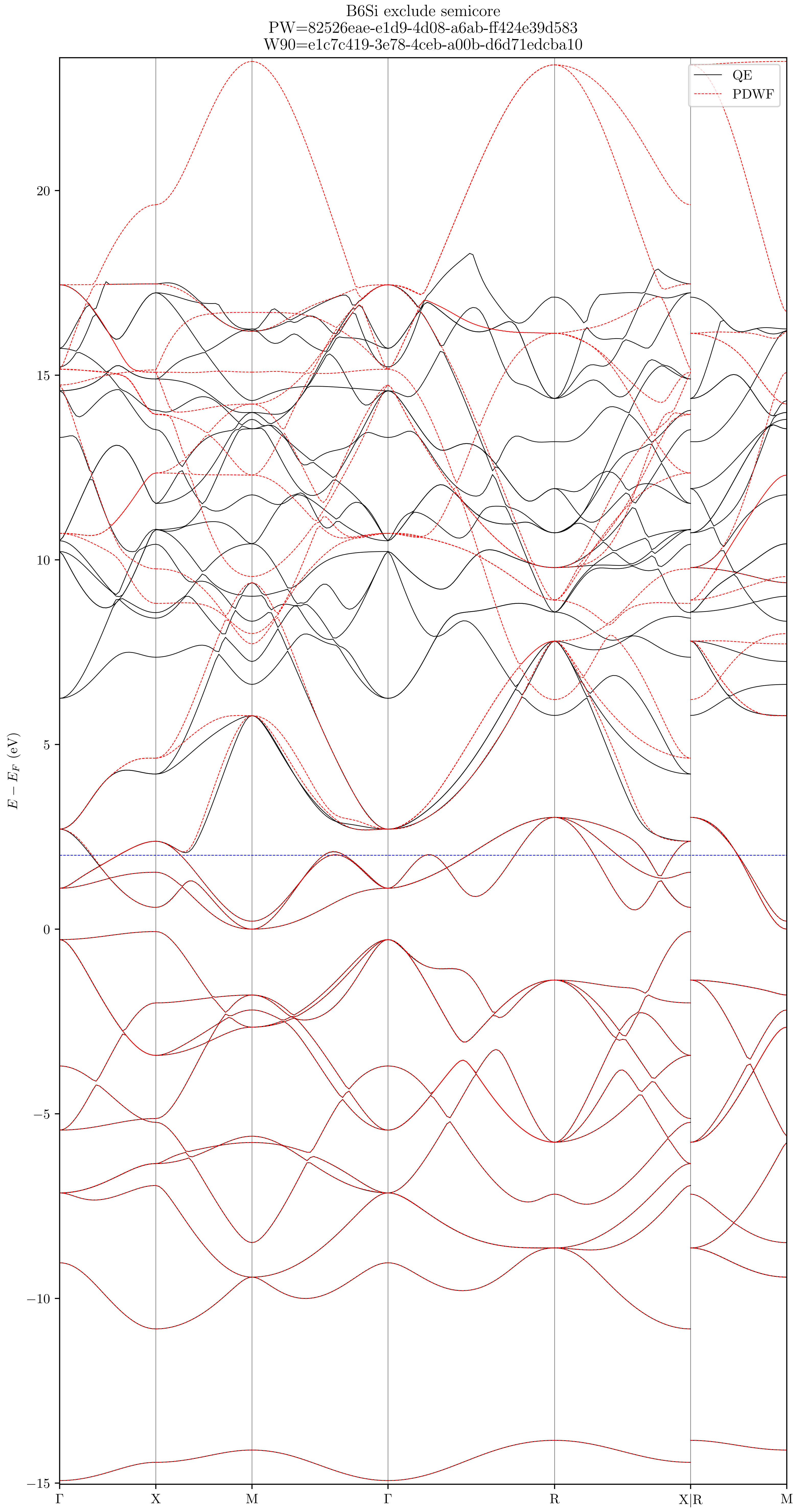

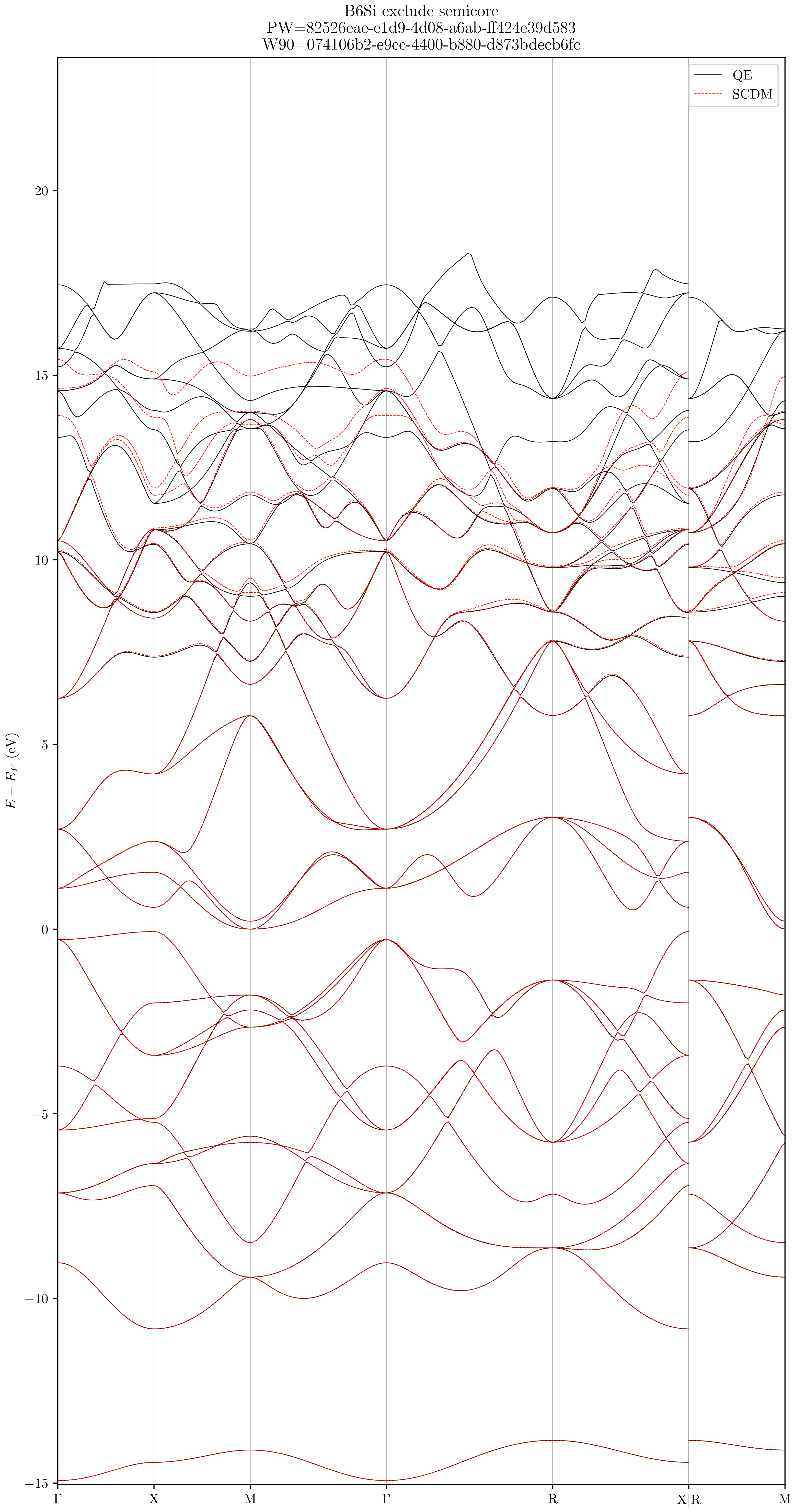

PDWF band distance (meV) and τ ($\AA$)

η_0	η_2	η_4	$\eta_0^{\max}$	$\eta_2^{\max}$	$\eta_4^{\max}$	τ
1.9	2.4	30.6	16.9	22.0	334.2	2.145

SCDM band distance (meV) and τ ($\AA$)

η_0	η_2	η_4	$\eta_0^{\max}$	$\eta_2^{\max}$	$\eta_4^{\max}$	τ
3.6	3.4	3.3	19.2	19.2	19.2	2.337

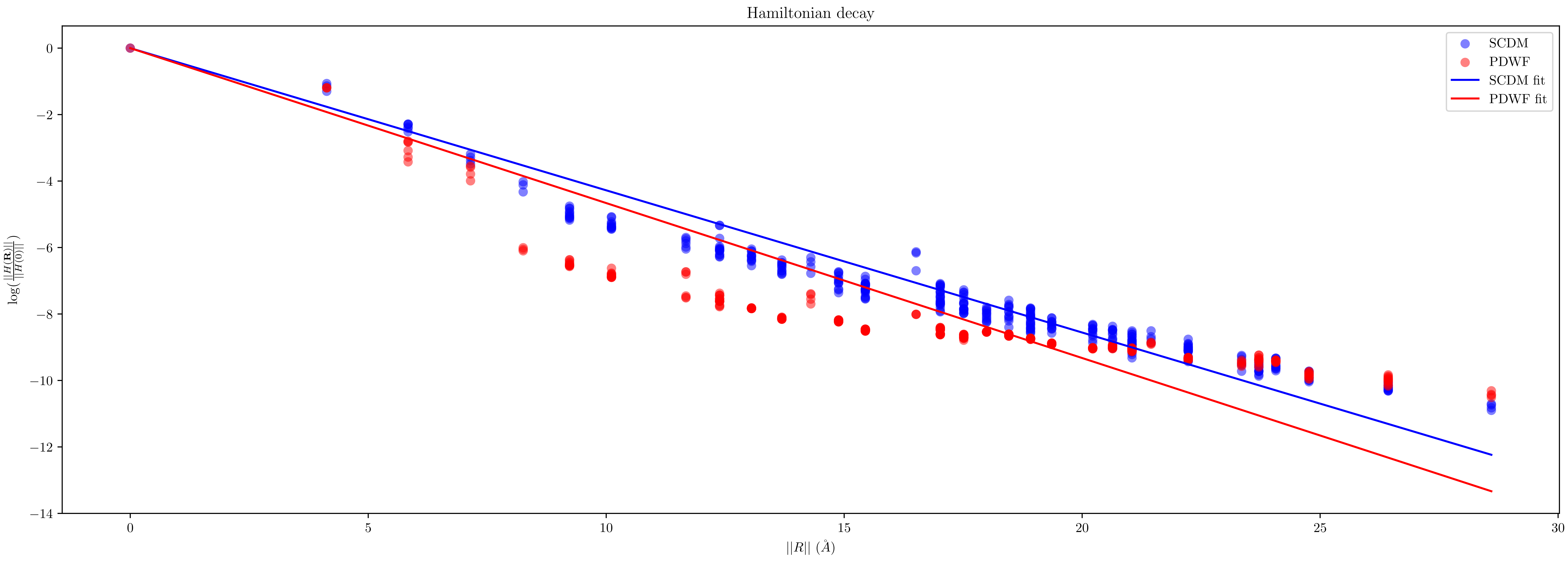

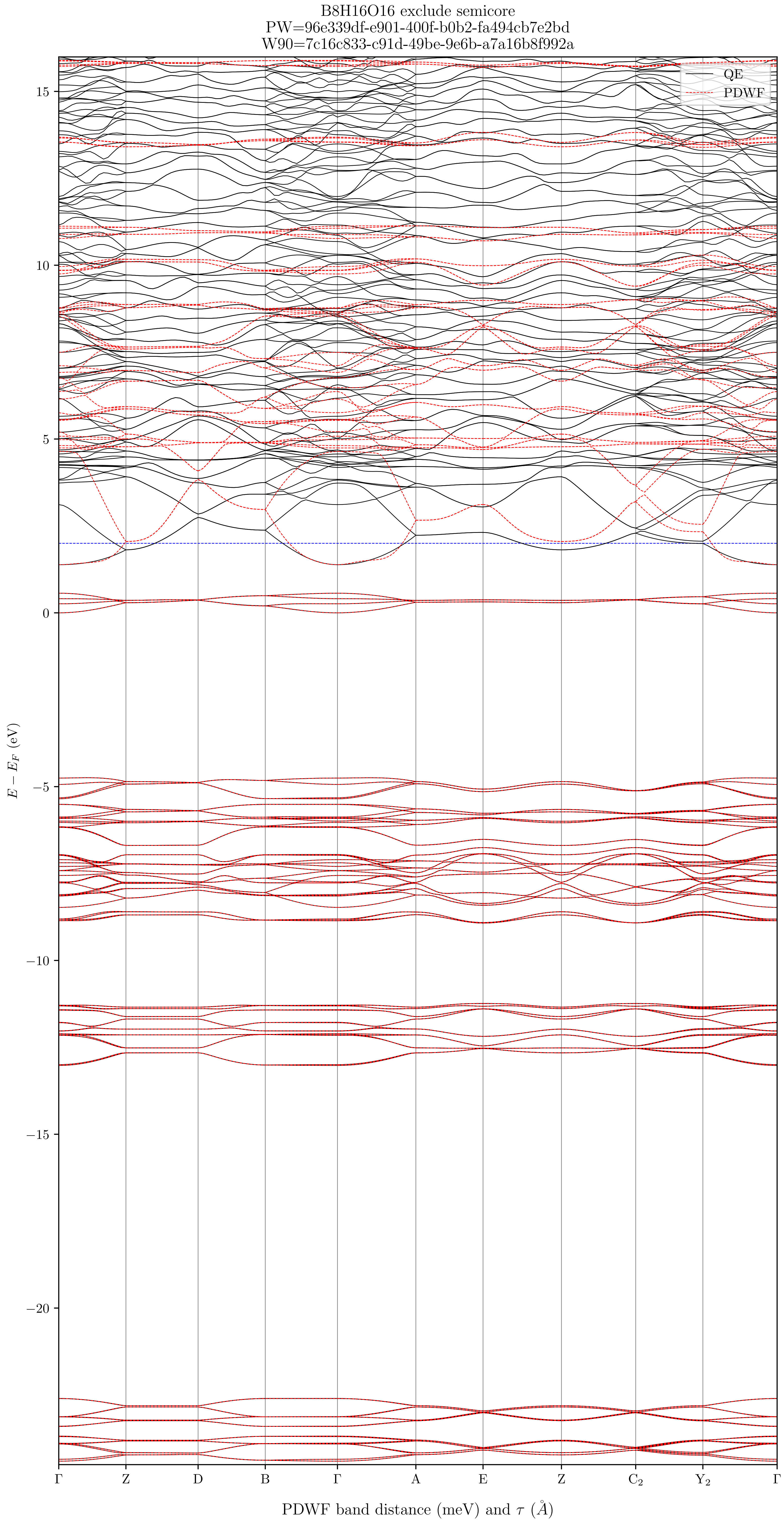

PDWF band distance (meV) and τ ($\AA$)

η_0	η_2	η_4	$\eta_0^{\max}$	$\eta_2^{\max}$	$\eta_4^{\max}$	τ
0.3	0.3	13.7	2.3	2.3	142.7	2.703

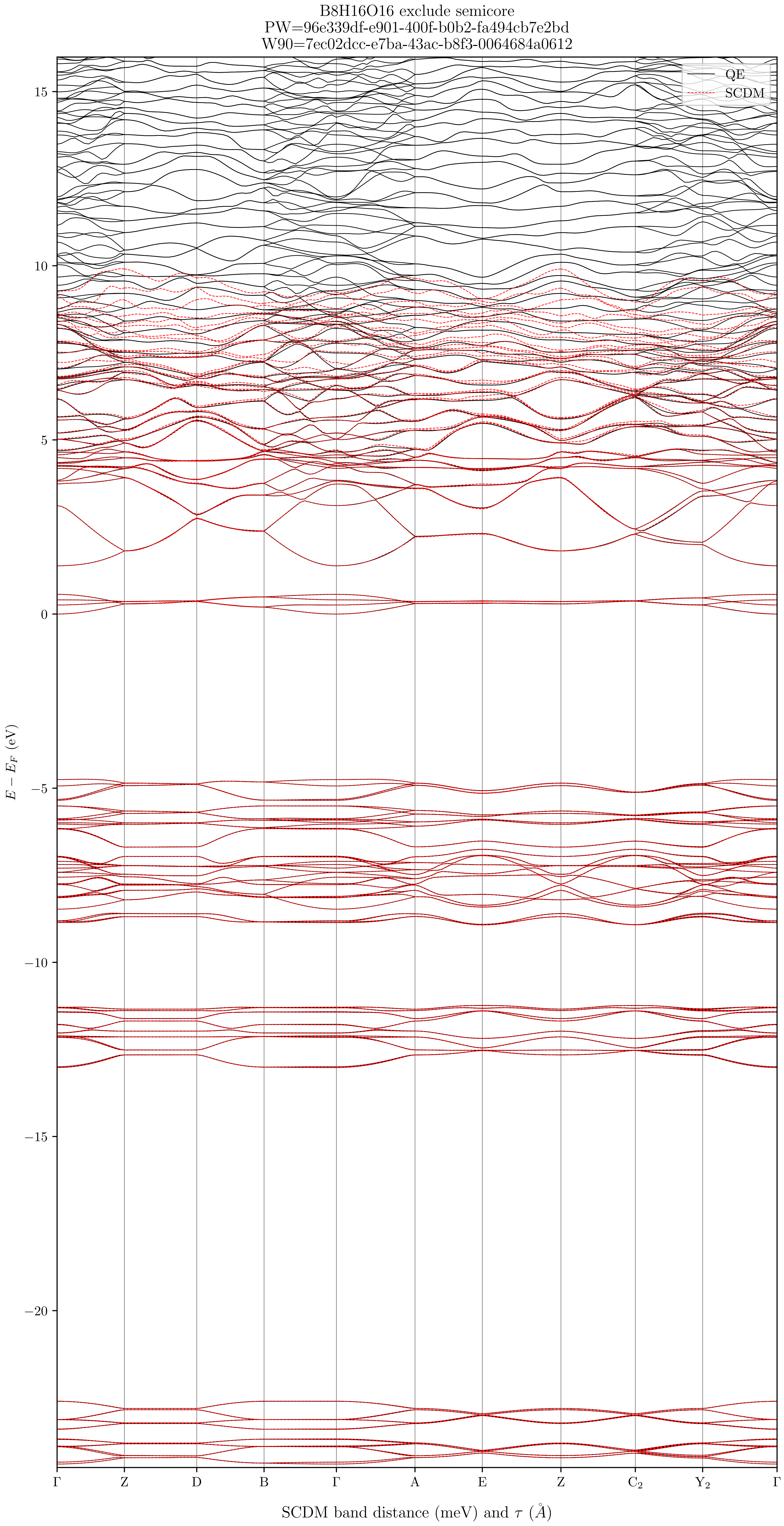

SCDM band distance (meV) and τ ($\AA$)

η_0	η_2	η_4	$\eta_0^{\max}$	$\eta_2^{\max}$	$\eta_4^{\max}$	τ
1.3	1.4	1.8	7.1	7.1	16.4	3.653

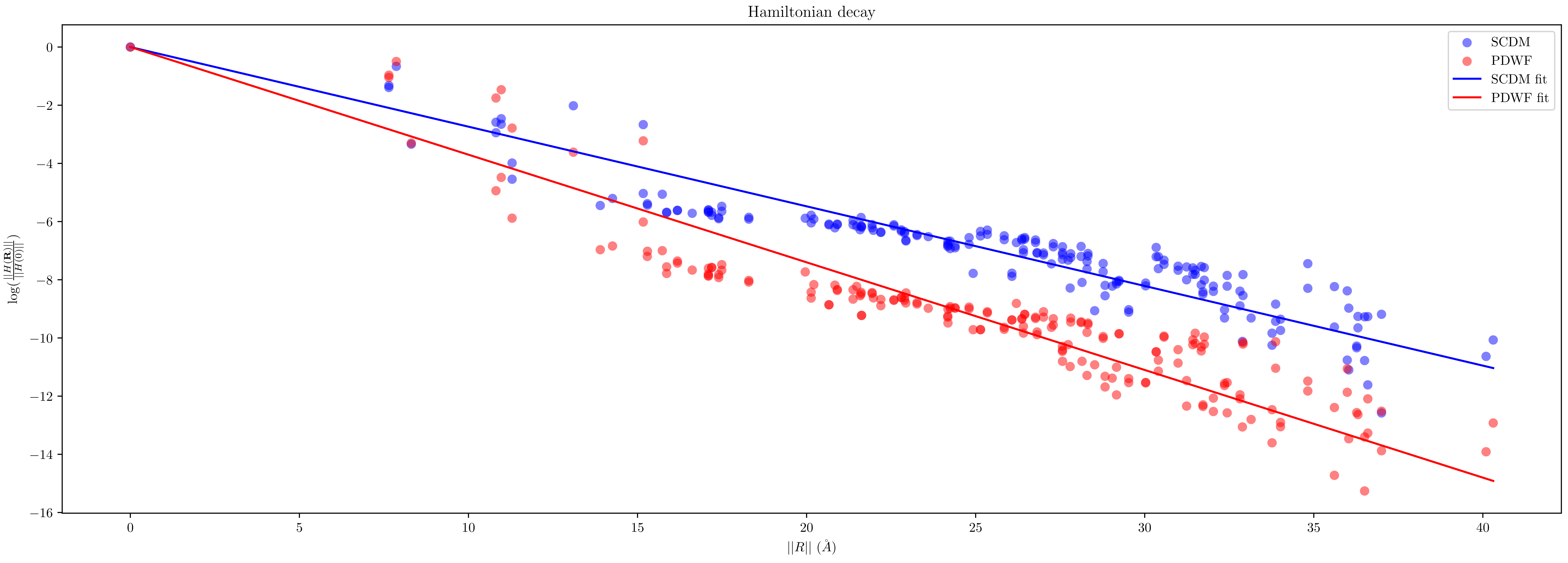

BaS exclude semicore
PW=07ed0f70-fd9c-4611-917f-978083c8dcb0
W90=4fcedb8f-3ed3-4b77-a7eb-4d6881165dc1

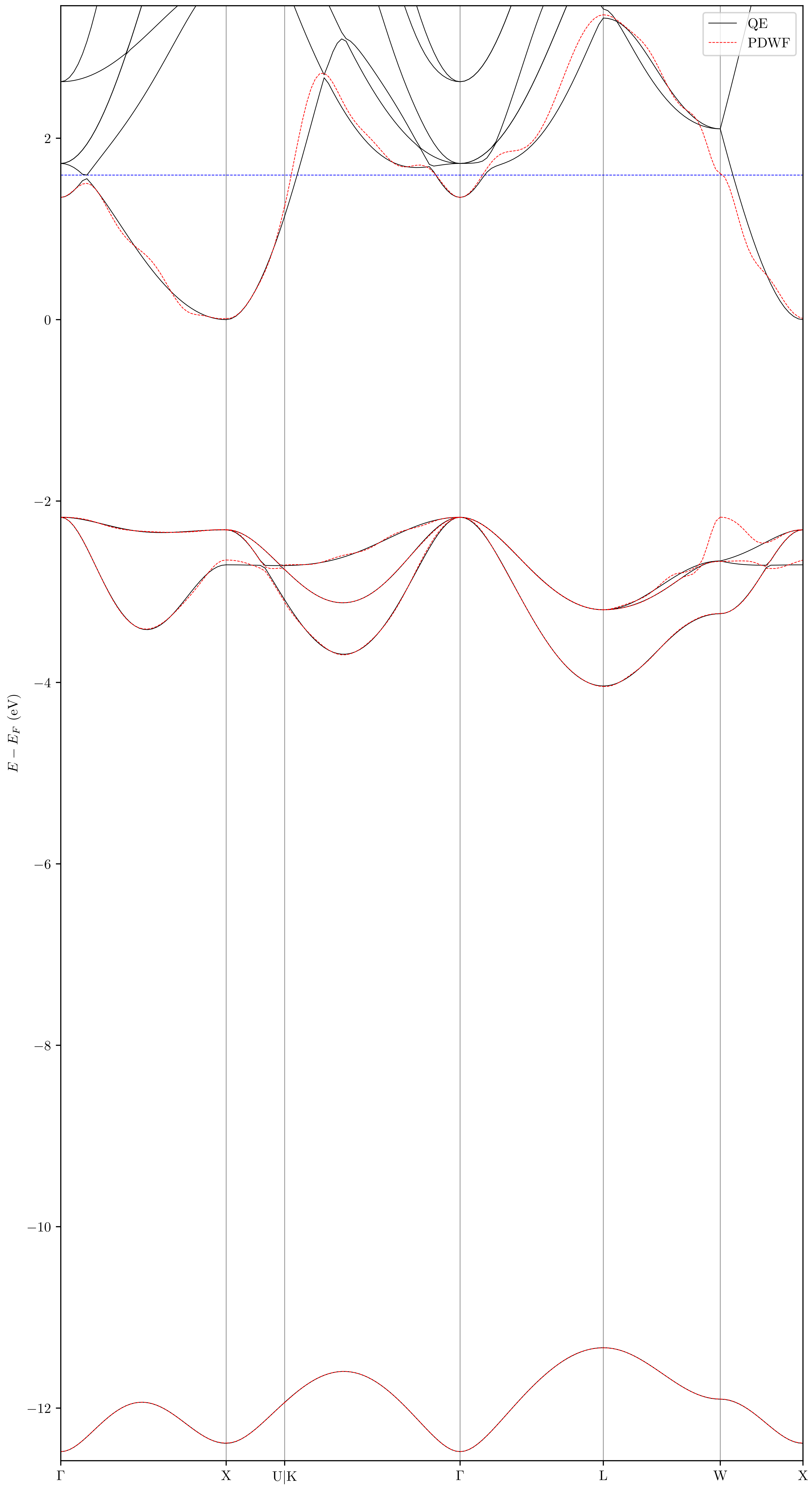

PDWF band distance (meV) and τ (Å)

η_0	η_2	η_4	$\eta_0^{\max}$	$\eta_2^{\max}$	$\eta_4^{\max}$	τ
46.0	46.2	69.9	483.1	483.1	492.2	2.922

BaS exclude semicore
PW=07ed0f70-fd9c-4611-917f-978083c8dcb0
W90=0a20abf7-c4f4-4e02-8ae1-ce427b152796

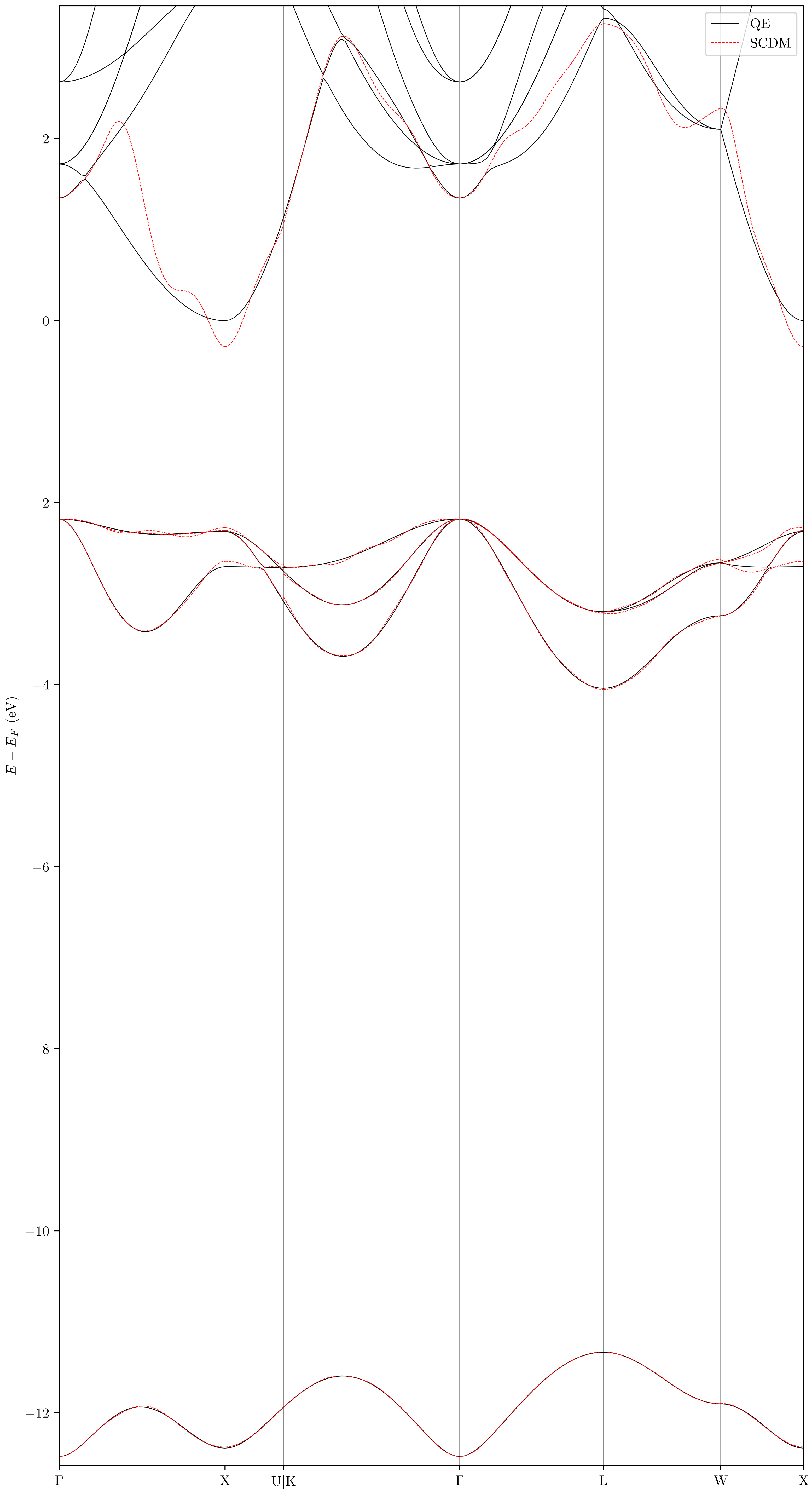

SCDM band distance (meV) and τ (Å)

η_0	η_2	η_4	$\eta_0^{\max}$	$\eta_2^{\max}$	$\eta_4^{\max}$	τ
15.1	39.7	151.4	60.7	287.4	1194.8	2.992

Hamiltonian decay

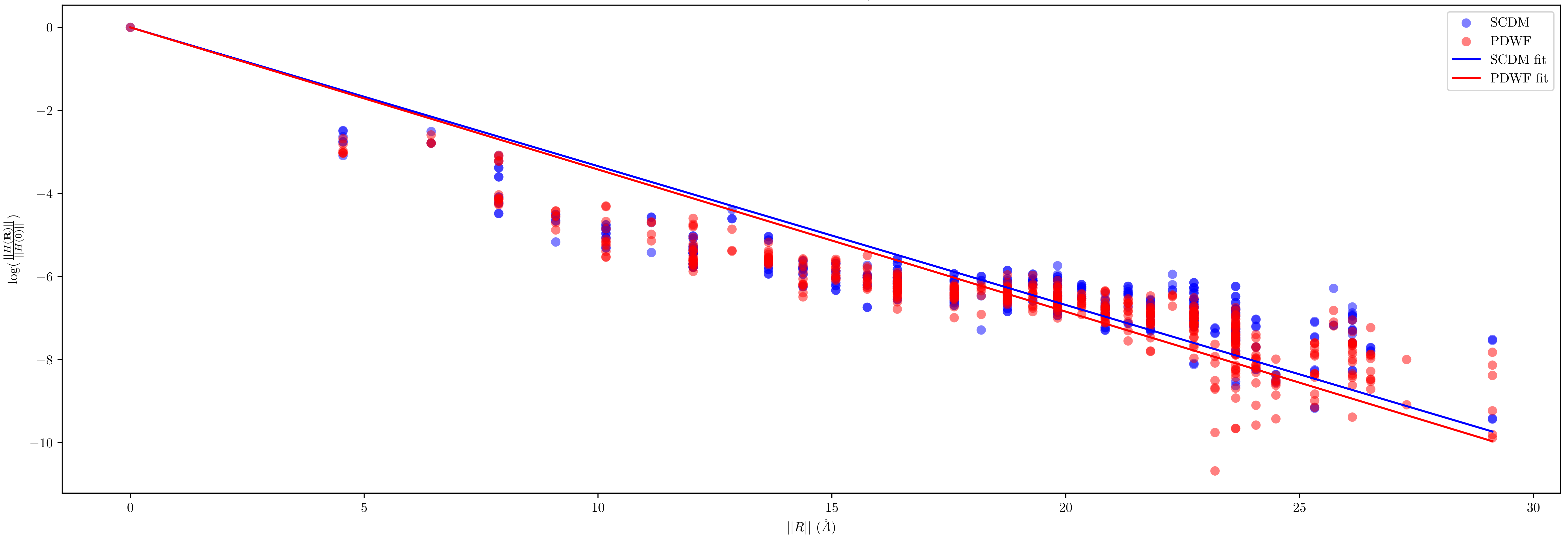

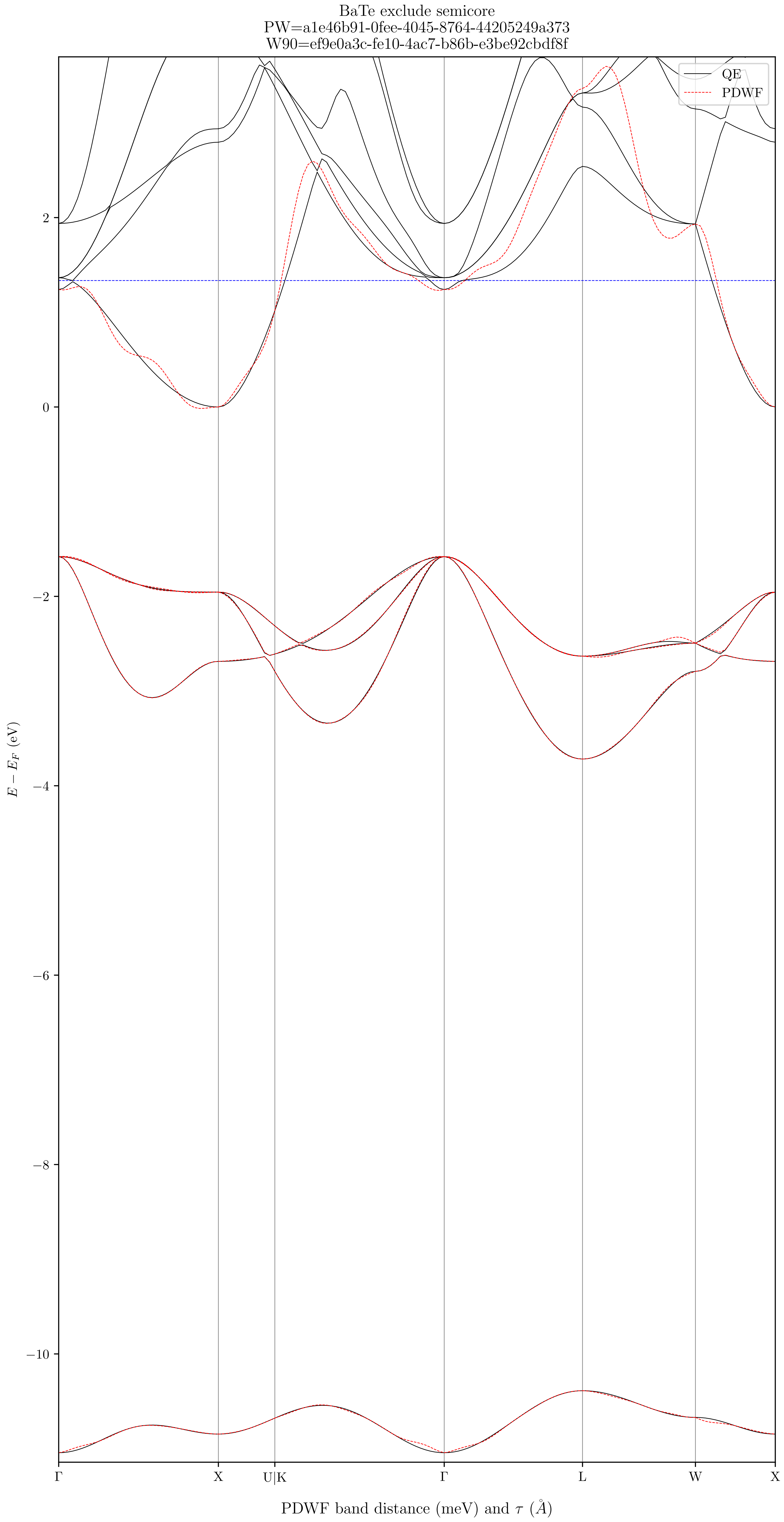

η_0	η_2	η_4	$\eta_0^{\max}$	$\eta_2^{\max}$	$\eta_4^{\max}$	τ
9.0	21.1	128.2	50.2	130.9	798.0	2.931

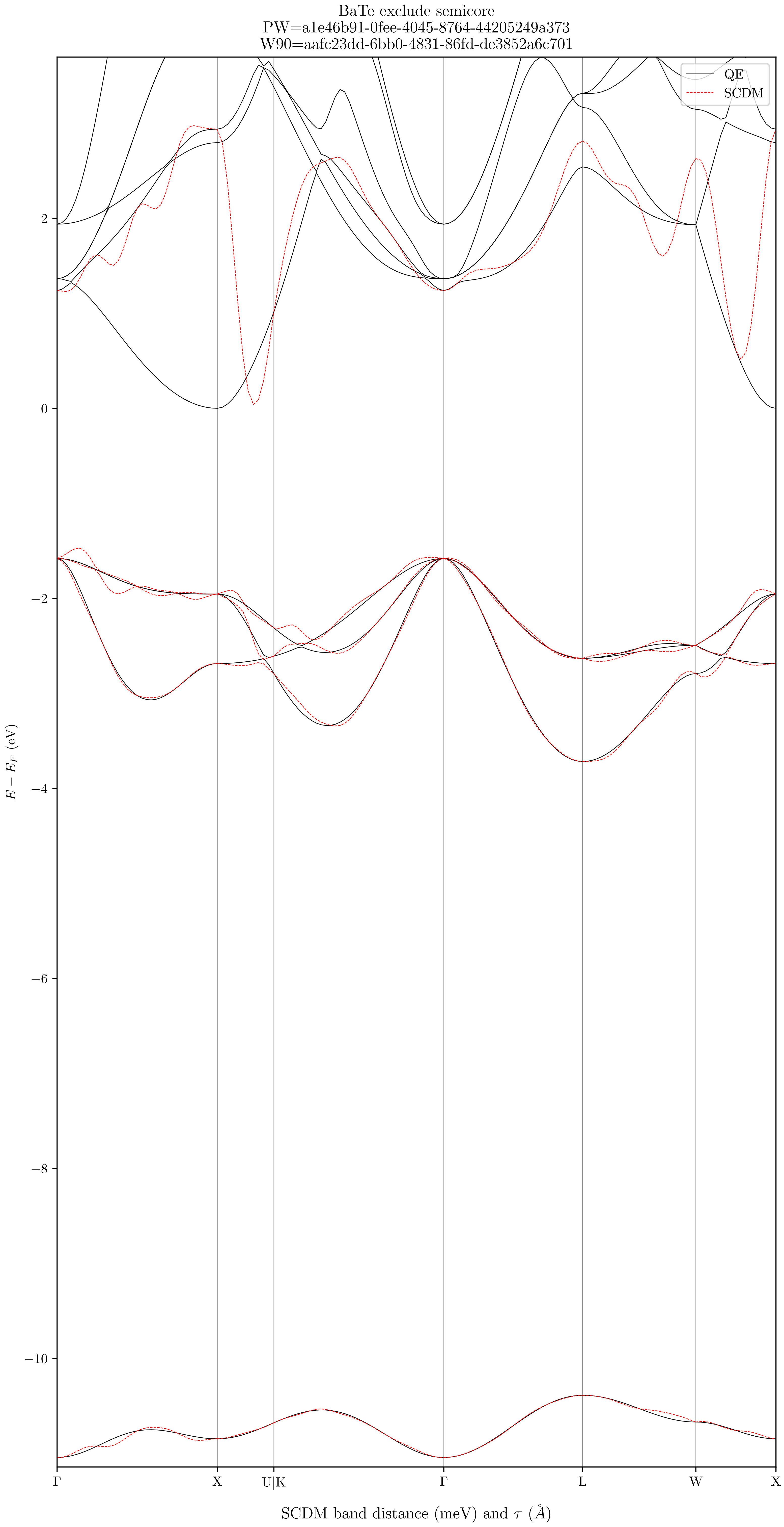

η_0	η_2	η_4	$\eta_0^{\max}$	$\eta_2^{\max}$	$\eta_4^{\max}$	τ
34.1	91.7	470.8	180.9	853.7	2884.7	3.444

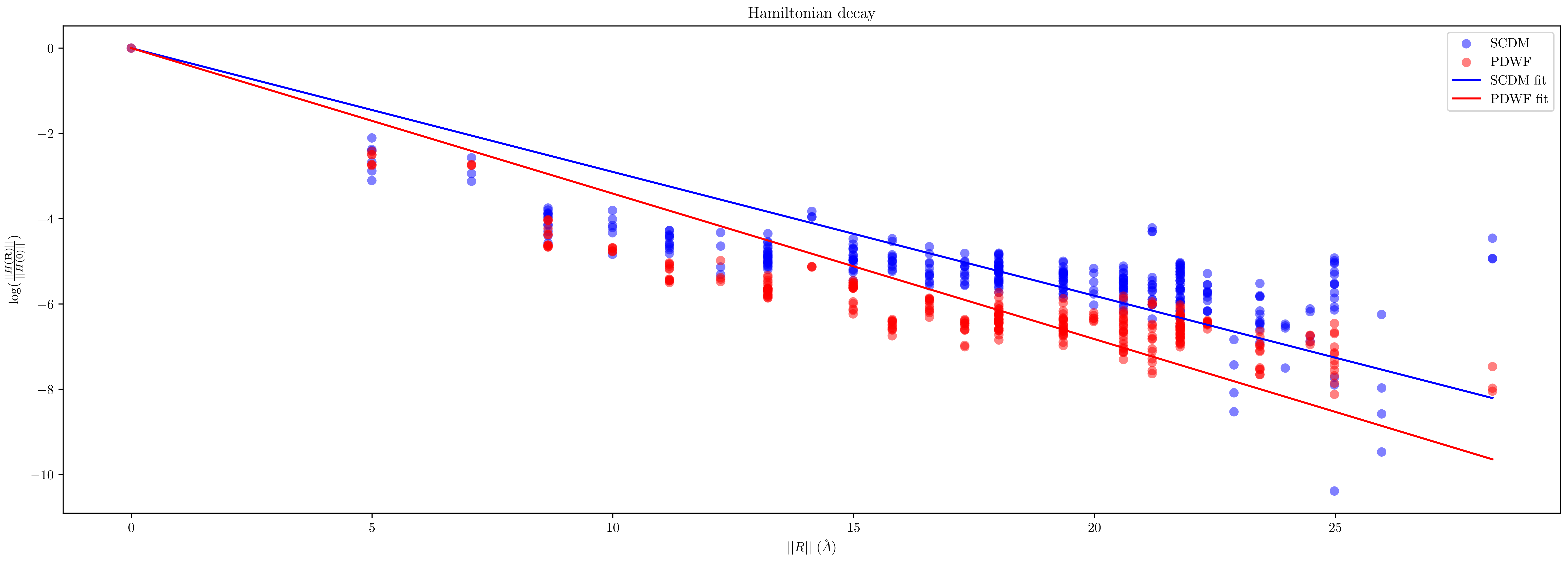

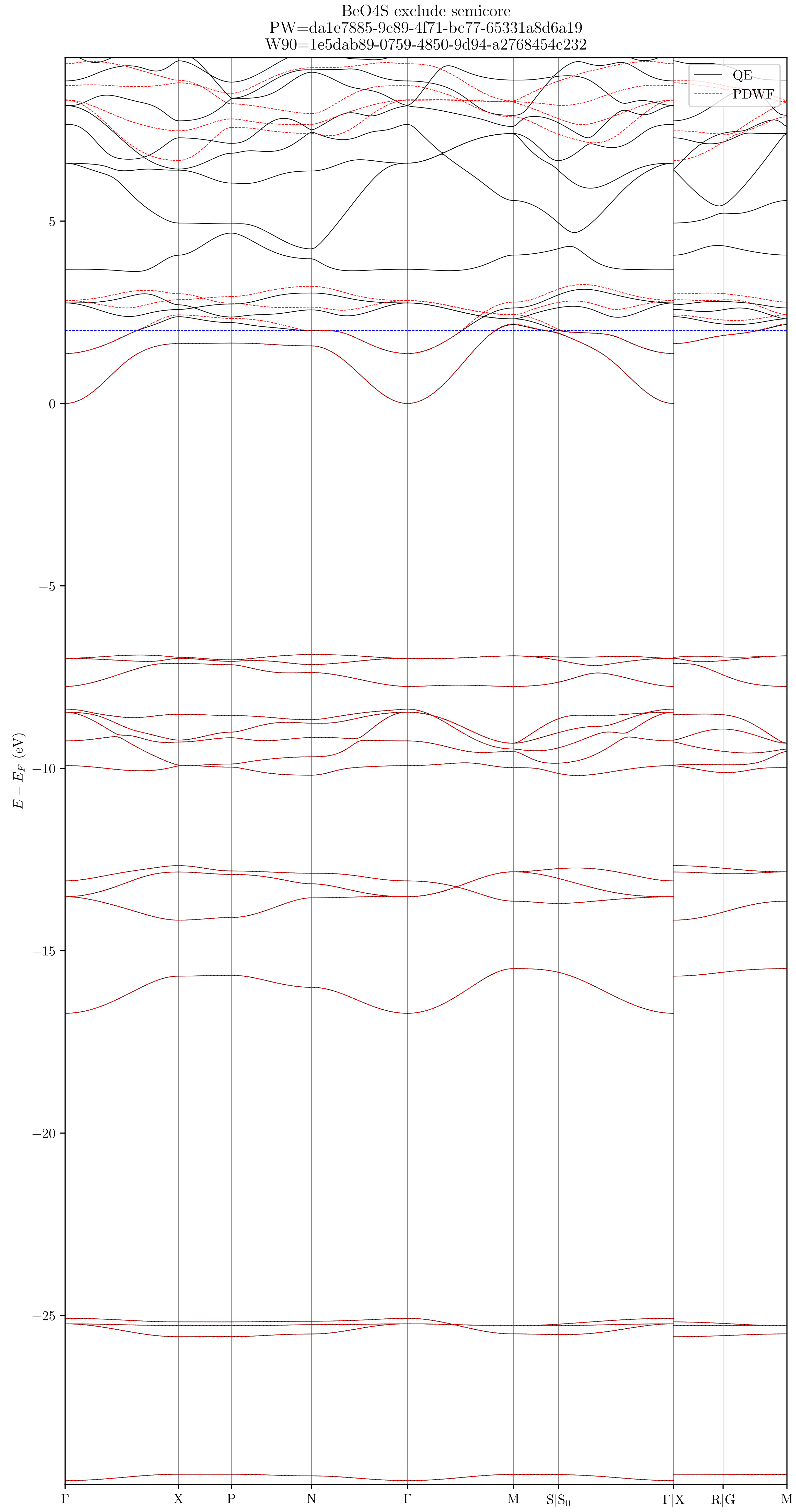

PDWF band distance (meV) and τ ($\AA$)

η_0	η_2	η_4	η_0^{max}	η_2^{max}	η_4^{max}	τ
0.2	0.2	1.4	1.4	1.4	4.8	2.291

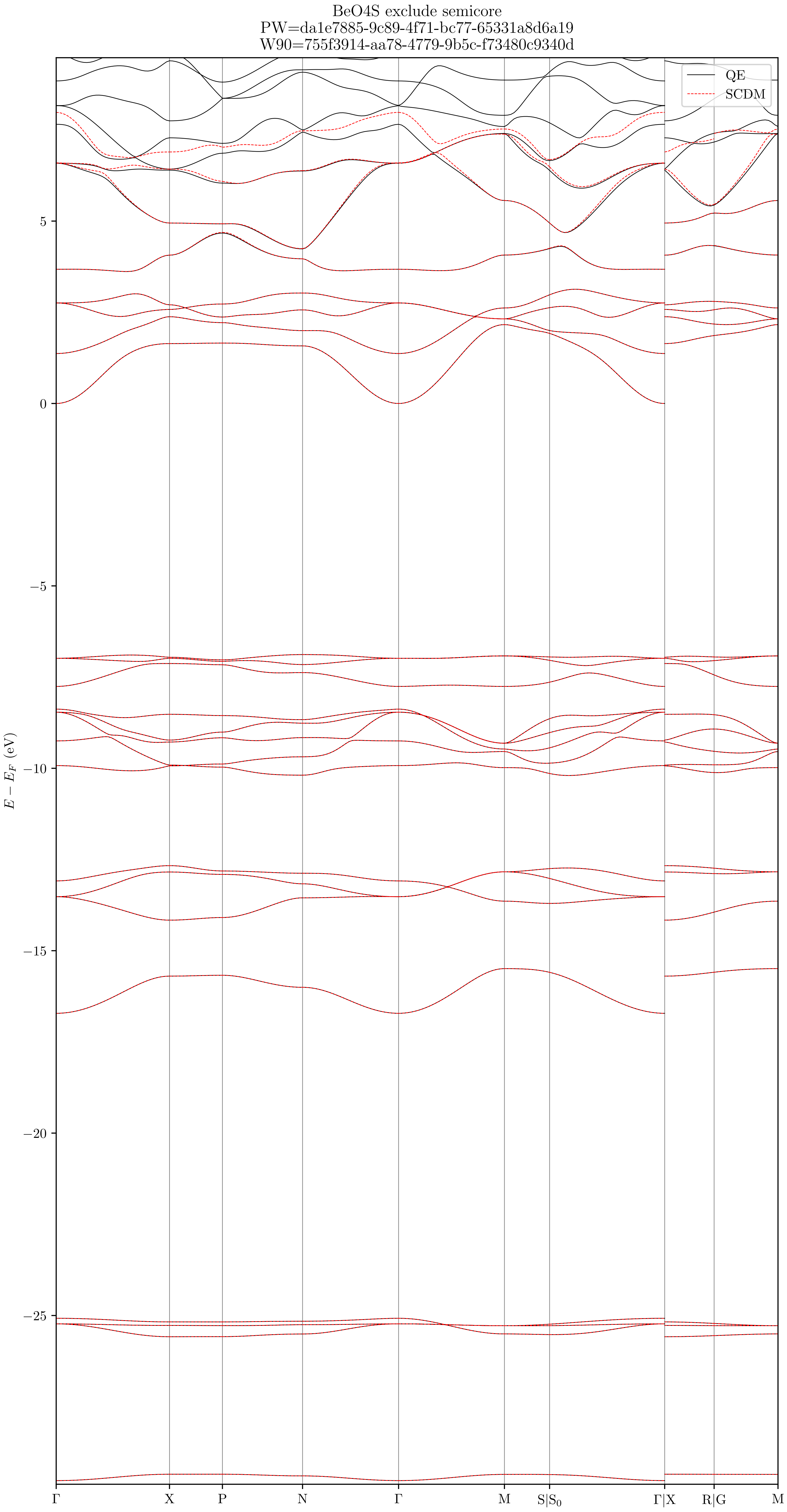

SCDM band distance (meV) and τ ($\AA$)

η_0	η_2	η_4	η_0^{max}	η_2^{max}	η_4^{max}	τ
0.1	0.1	0.1	0.6	0.6	1.1	2.196

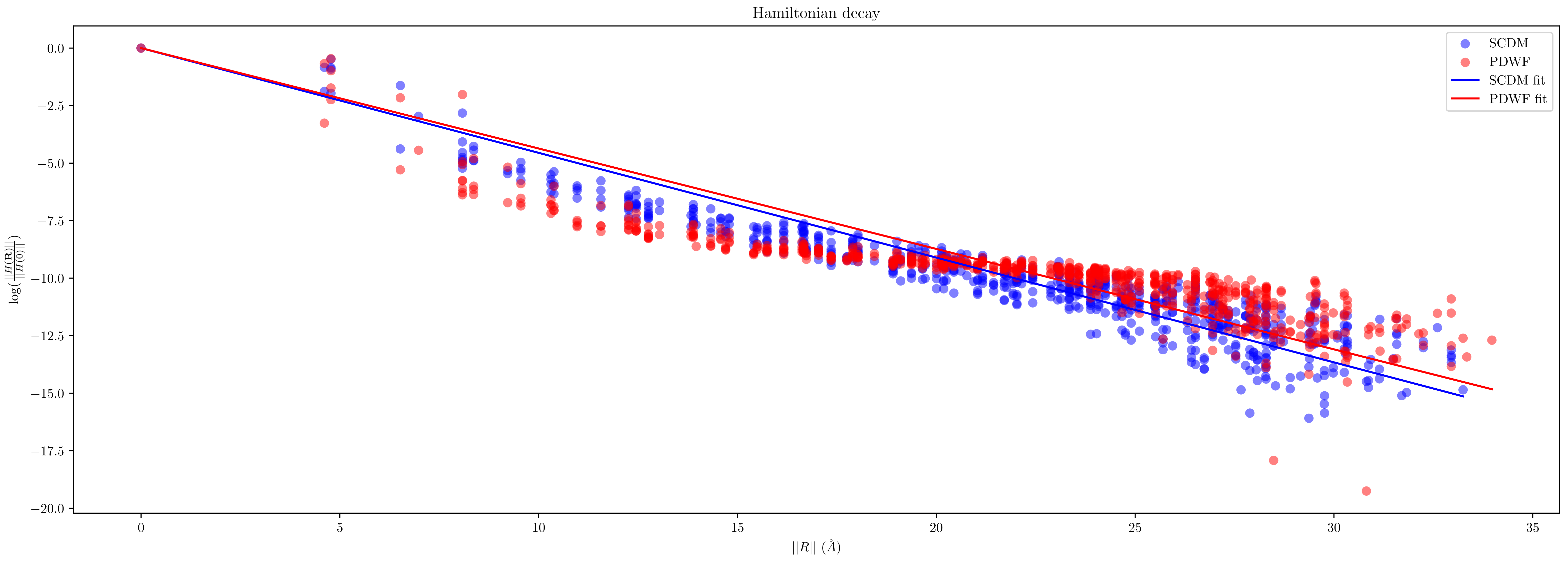

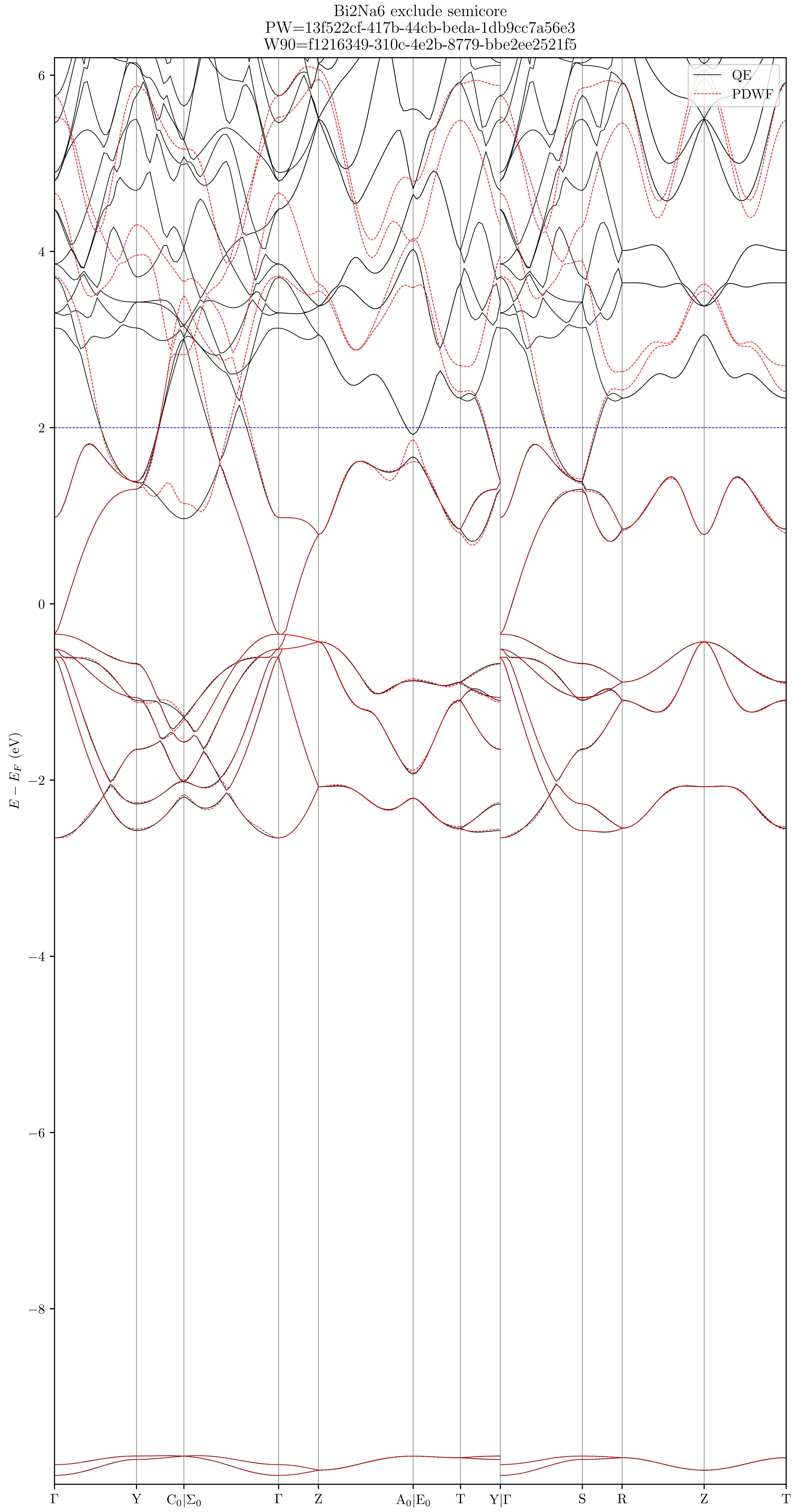

PDWF band distance (meV) and τ ($\AA$)

η_0	η_2	η_4	η_0^{max}	η_2^{max}	η_4^{max}	τ
7.6	21.8	254.3	45.0	312.6	1652.3	3.480

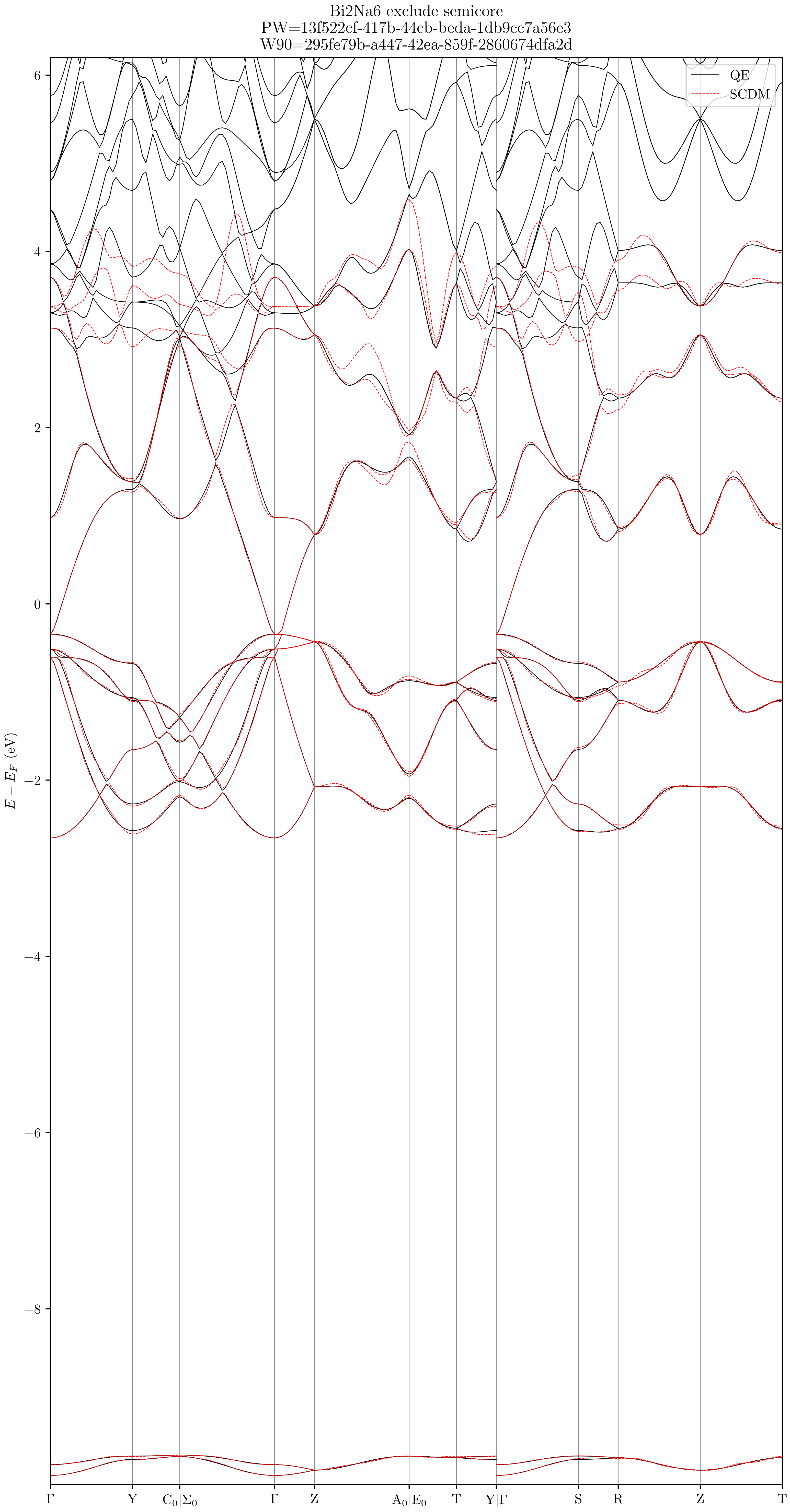

SCDM band distance (meV) and τ ($\AA$)

η_0	η_2	η_4	η_0^{max}	η_2^{max}	η_4^{max}	τ
13.0	21.2	95.1	55.9	163.8	564.3	3.728

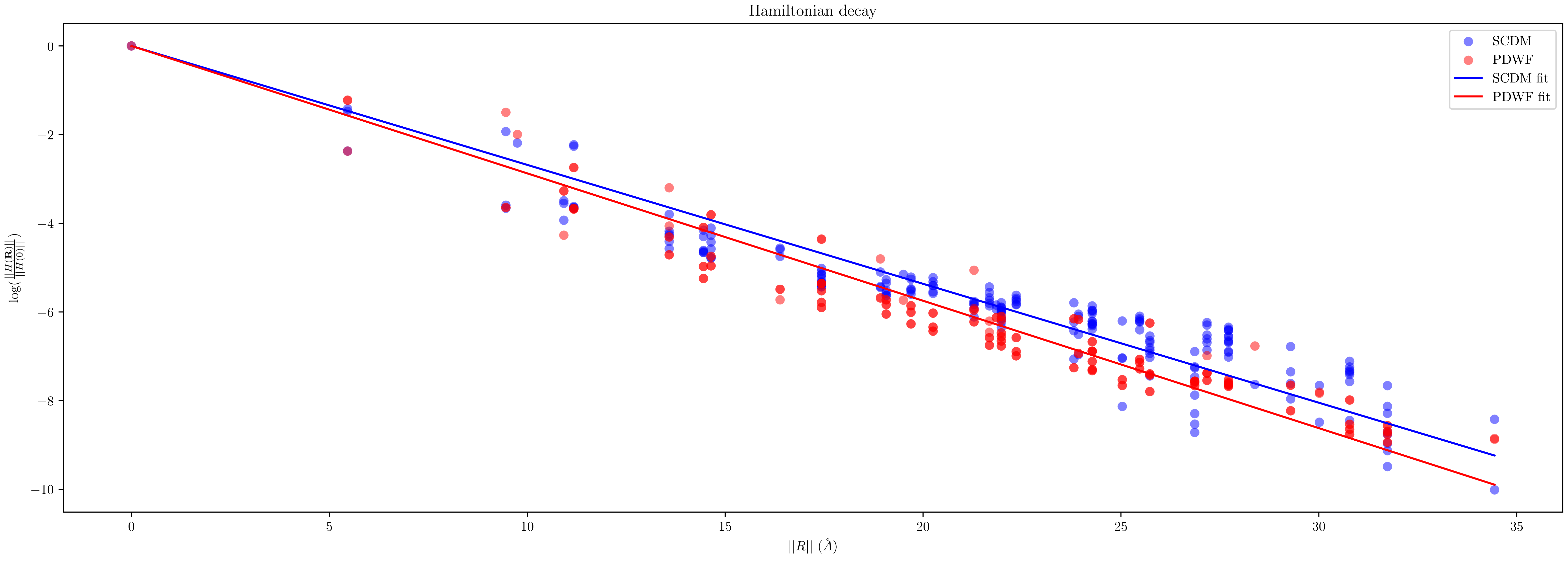

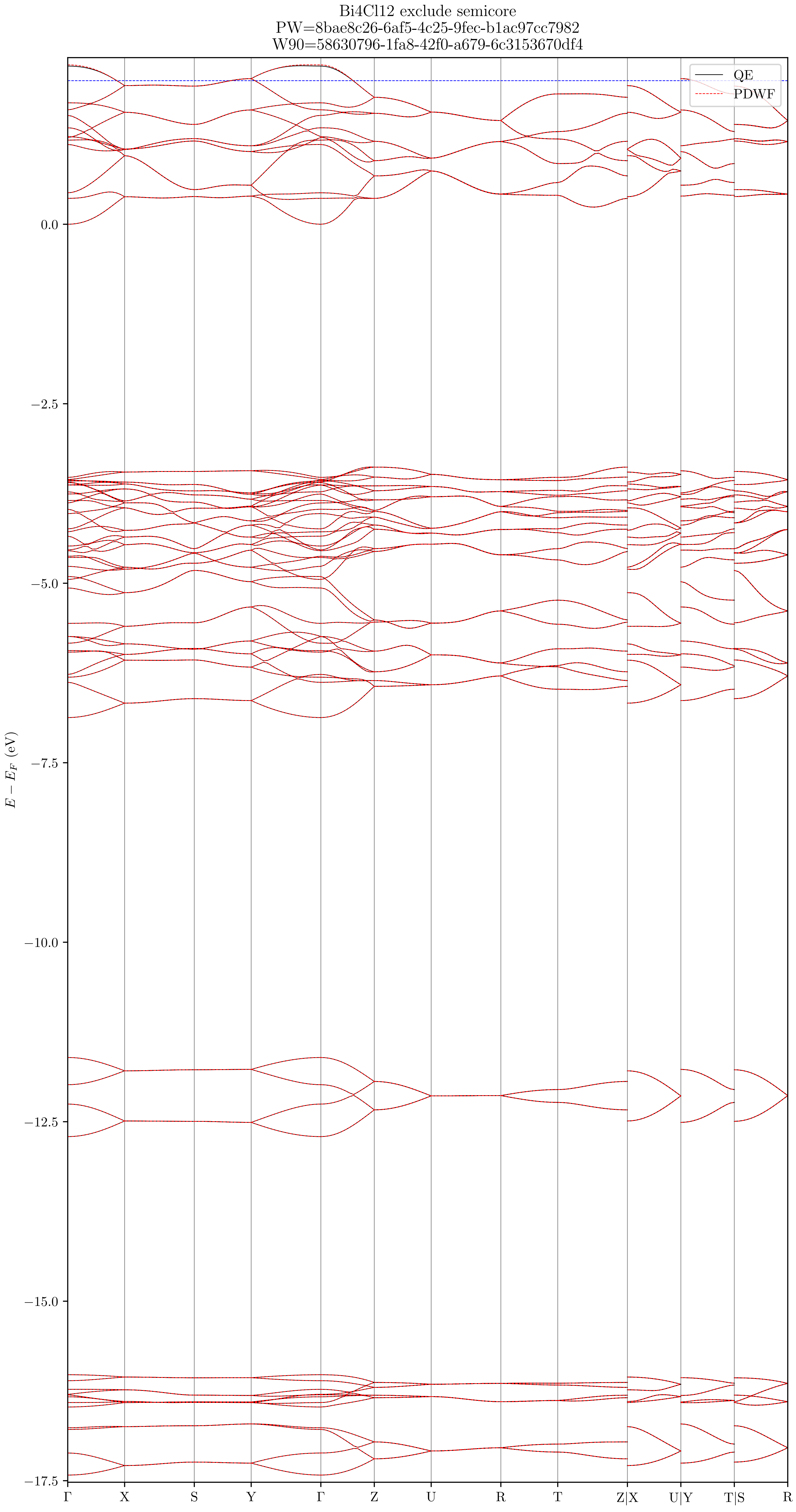

PDWF band distance (meV) and τ ($\AA$)

η_0	η_2	η_4	$\eta_0^{\max}$	$\eta_2^{\max}$	$\eta_4^{\max}$	τ
0.1	0.1	0.6	0.5	0.5	16.3	2.151

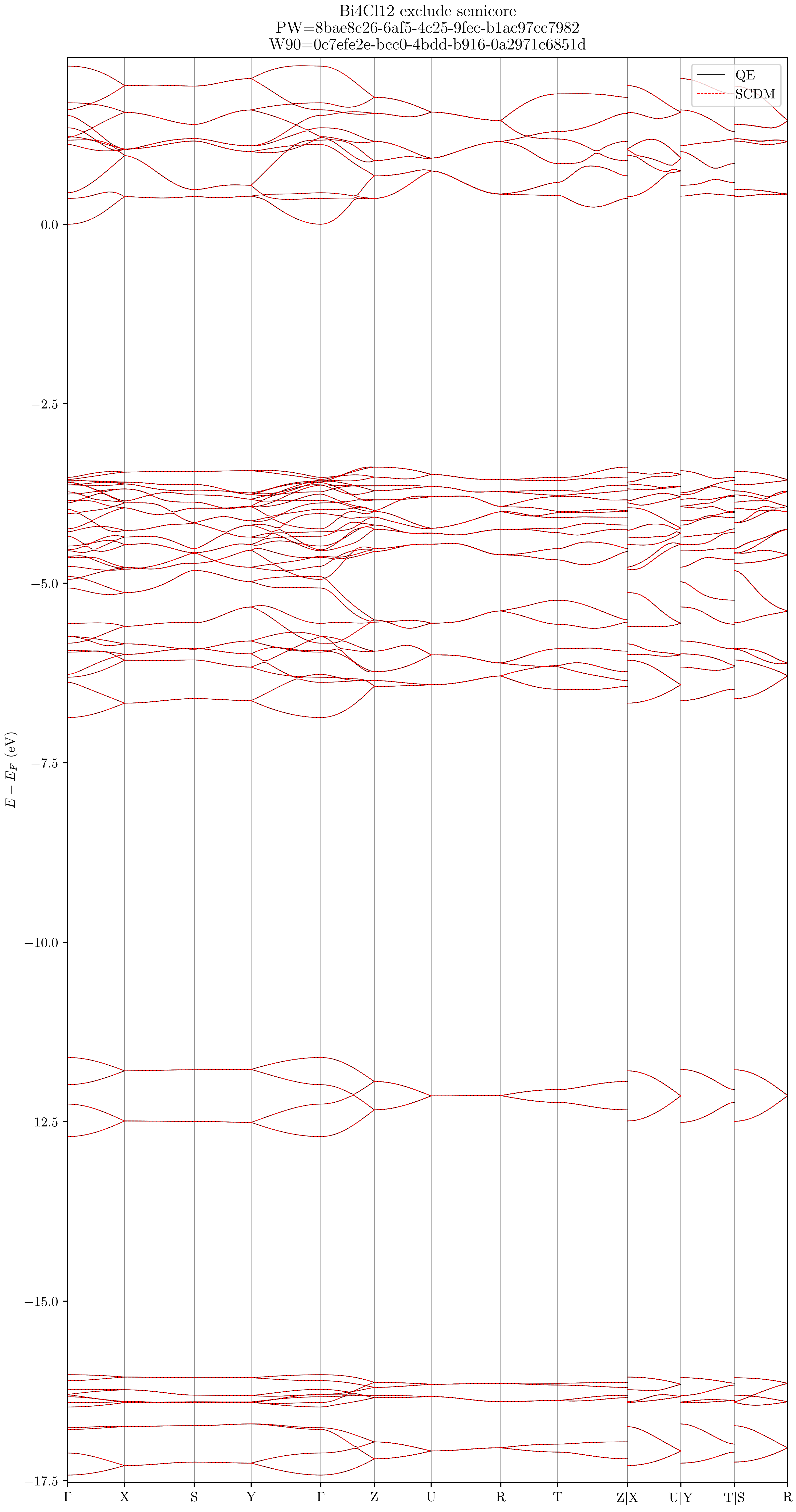

SCDM band distance (meV) and τ ($\AA$)

η_0	η_2	η_4	$\eta_0^{\max}$	$\eta_2^{\max}$	$\eta_4^{\max}$	τ
0.1	0.1	0.1	0.5	0.5	0.7	2.127

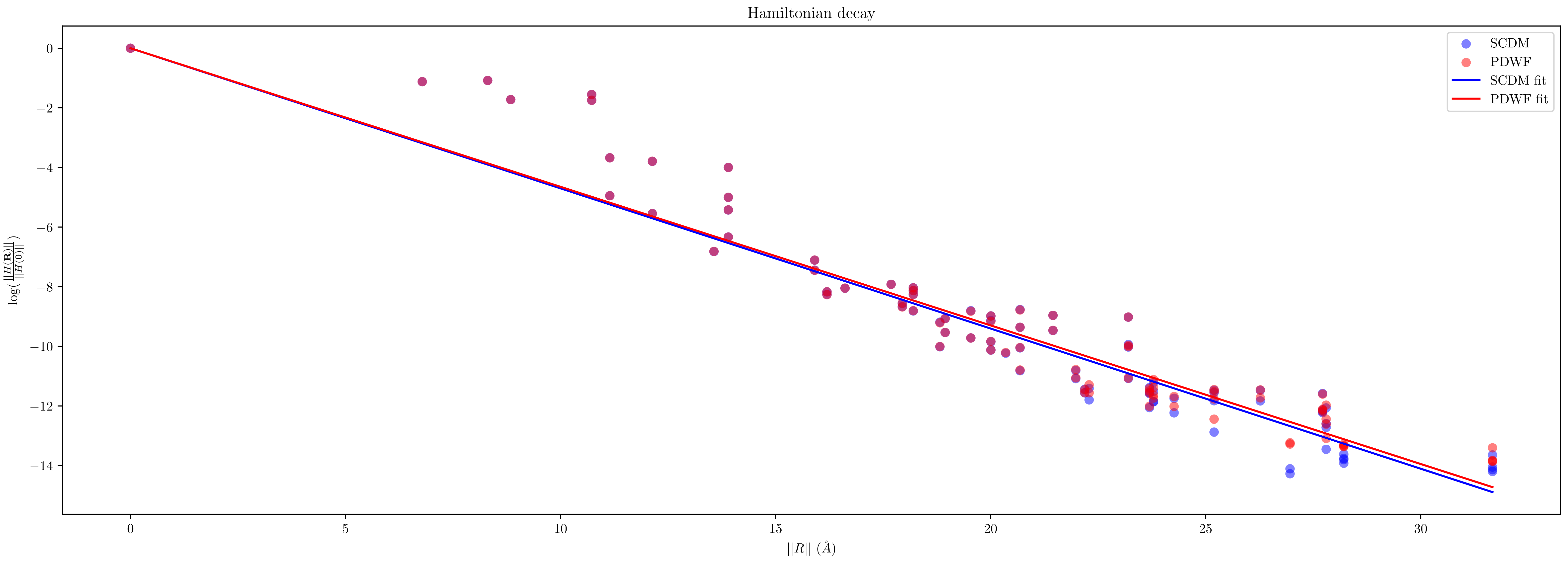

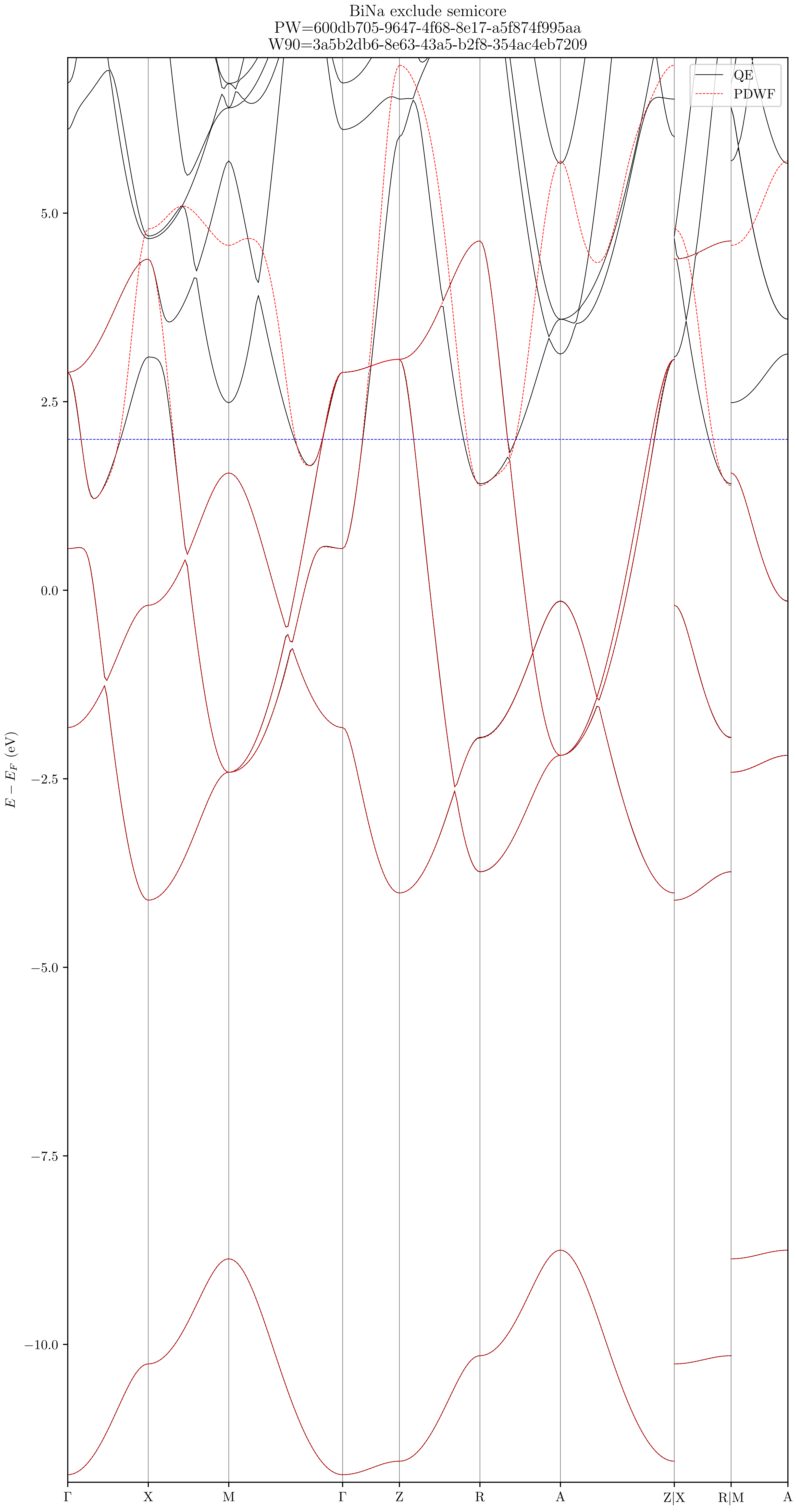

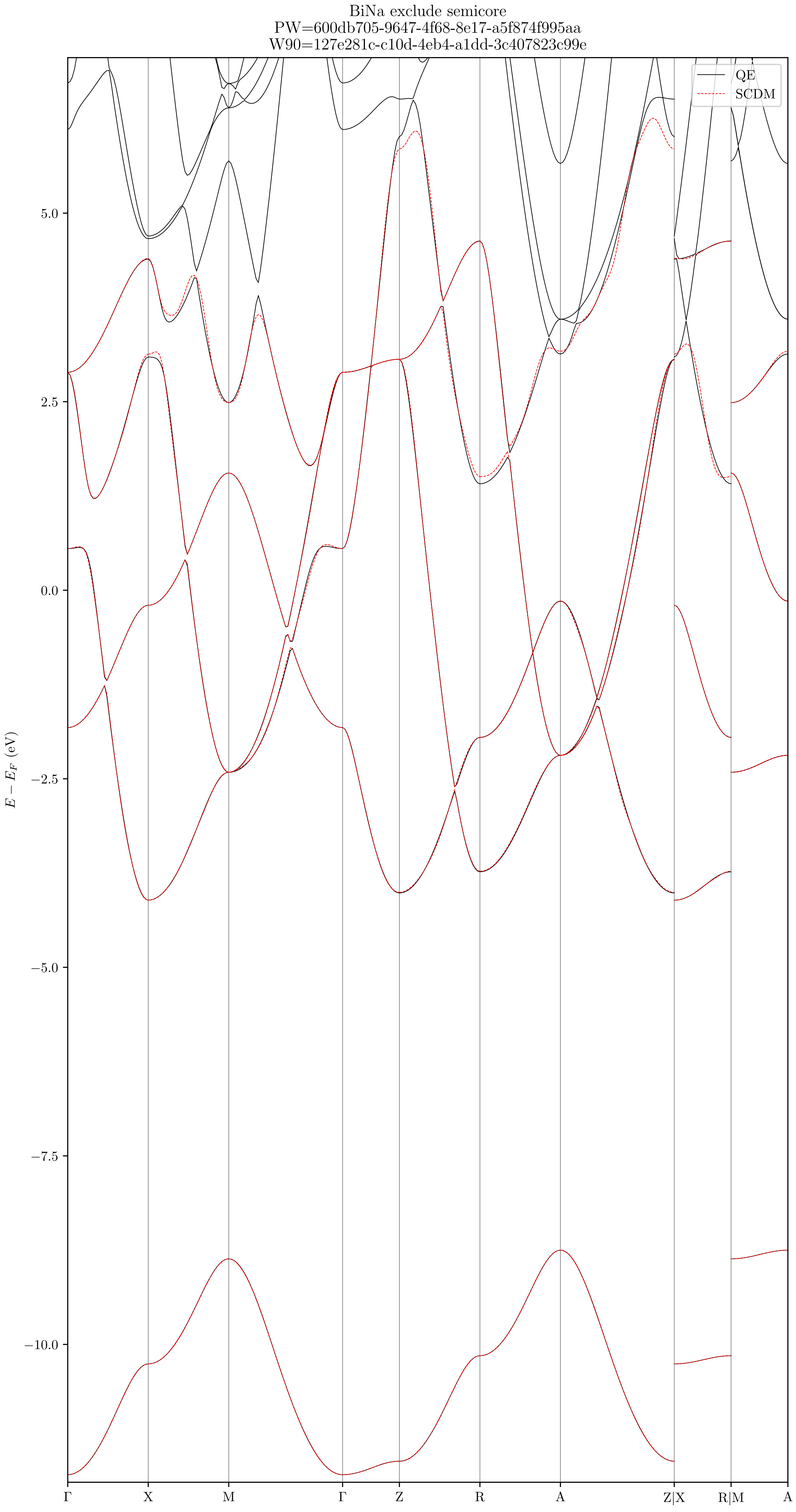

PDWF band distance (meV) and τ ($\AA$)

η_0	η_2	η_4	$\eta_0^{\max}$	$\eta_2^{\max}$	$\eta_4^{\max}$	τ
3.9	15.6	156.5	21.7	144.7	1028.0	2.085

SCDM band distance (meV) and τ ($\AA$)

η_0	η_2	η_4	$\eta_0^{\max}$	$\eta_2^{\max}$	$\eta_4^{\max}$	τ
8.4	17.0	30.8	43.2	103.8	300.5	2.710

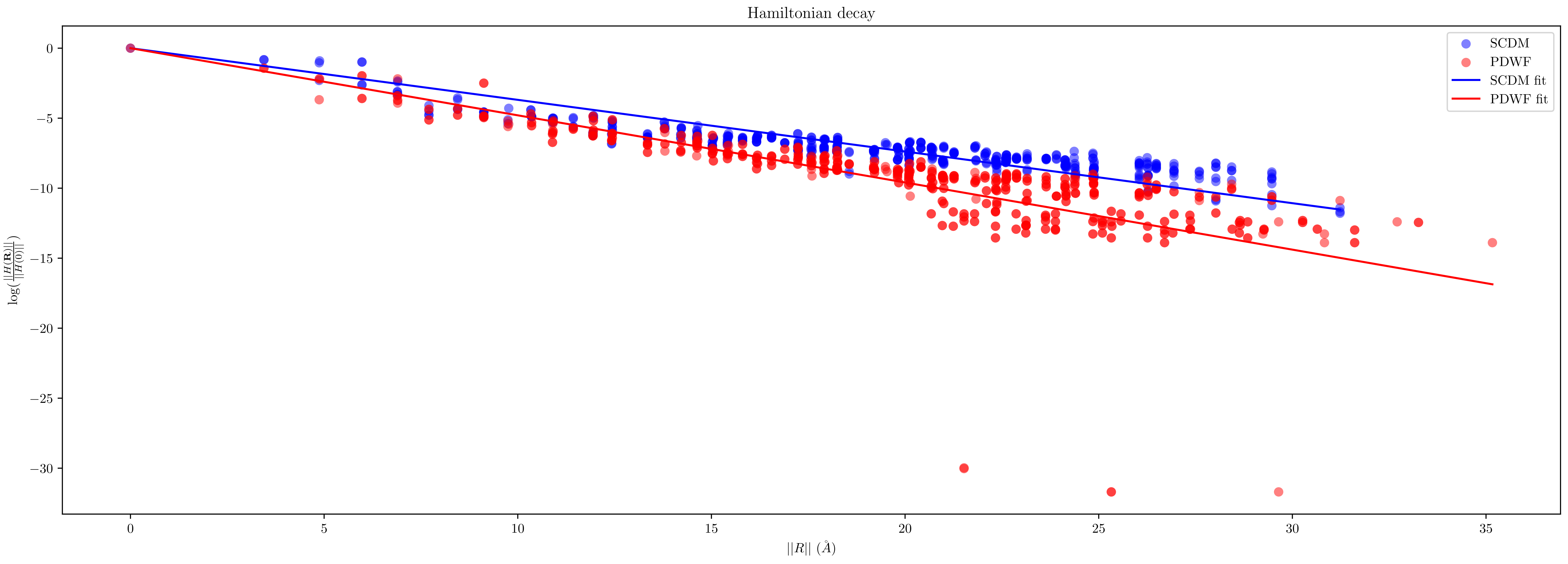

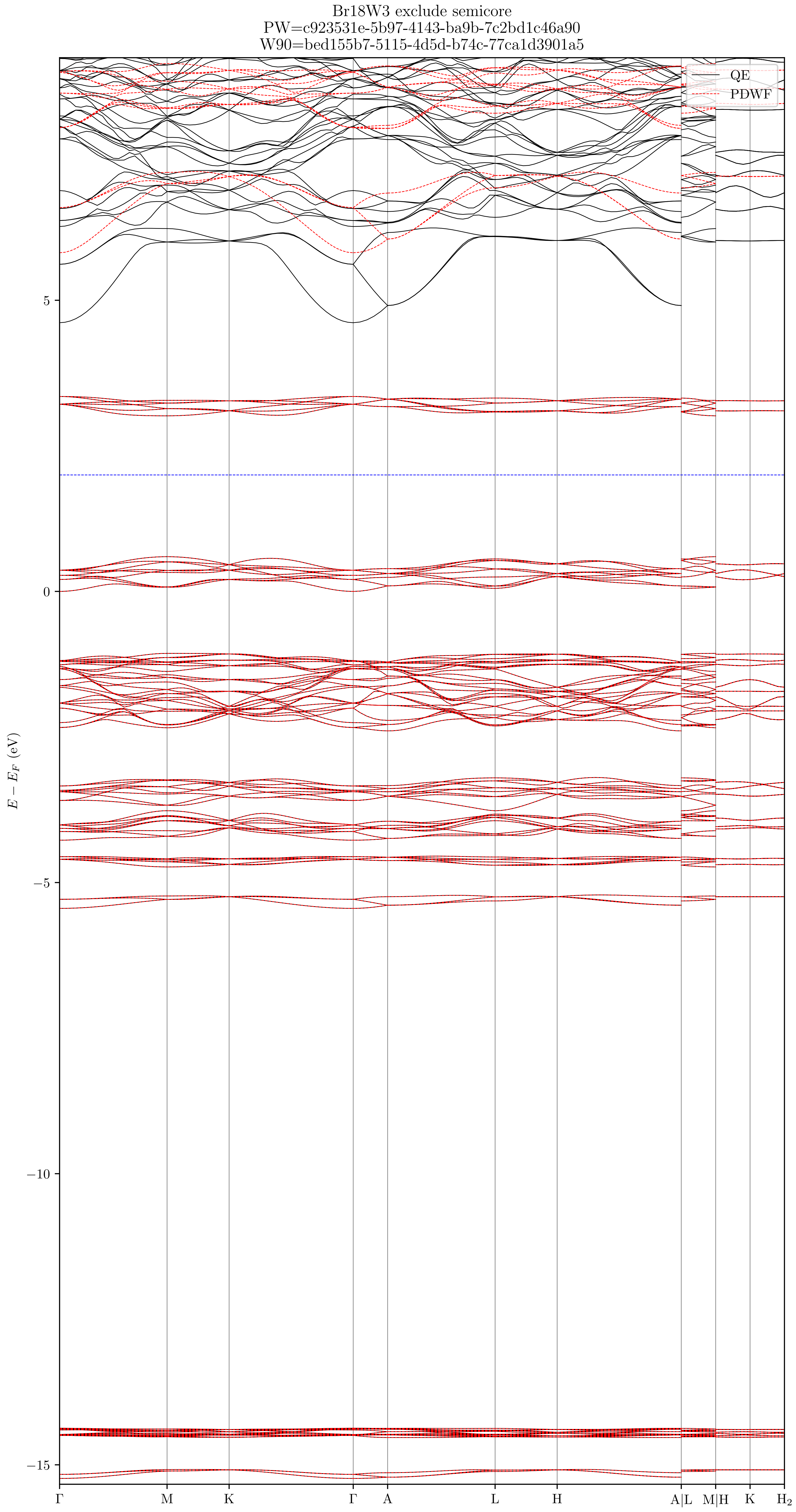

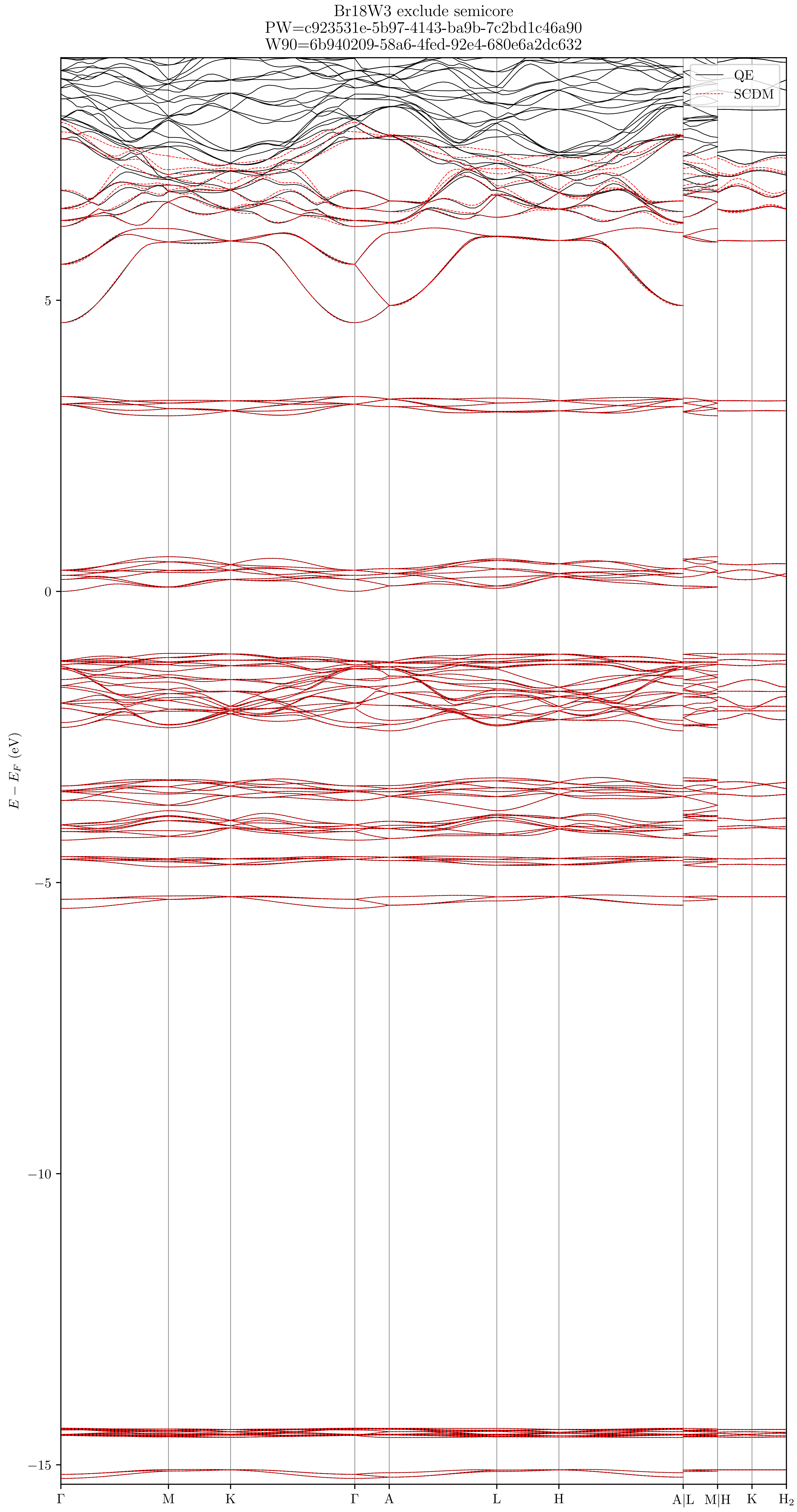

PDWF band distance (meV) and τ ($\AA$)

η_0	η_2	η_4	$\eta_0^{\max}$	$\eta_2^{\max}$	$\eta_4^{\max}$	τ
0.1	0.1	0.1	0.7	0.7	0.7	2.916

SCDM band distance (meV) and τ ($\AA$)

η_0	η_2	η_4	$\eta_0^{\max}$	$\eta_2^{\max}$	$\eta_4^{\max}$	τ
0.8	0.8	0.9	5.8	5.8	9.8	3.887

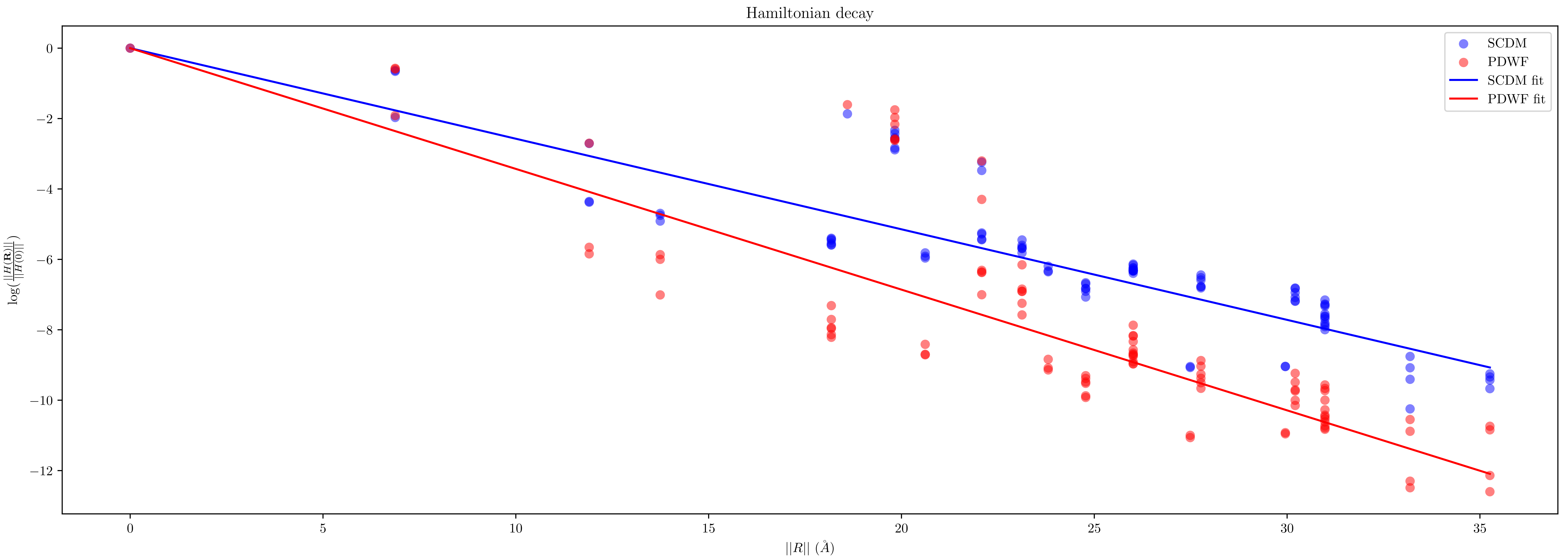

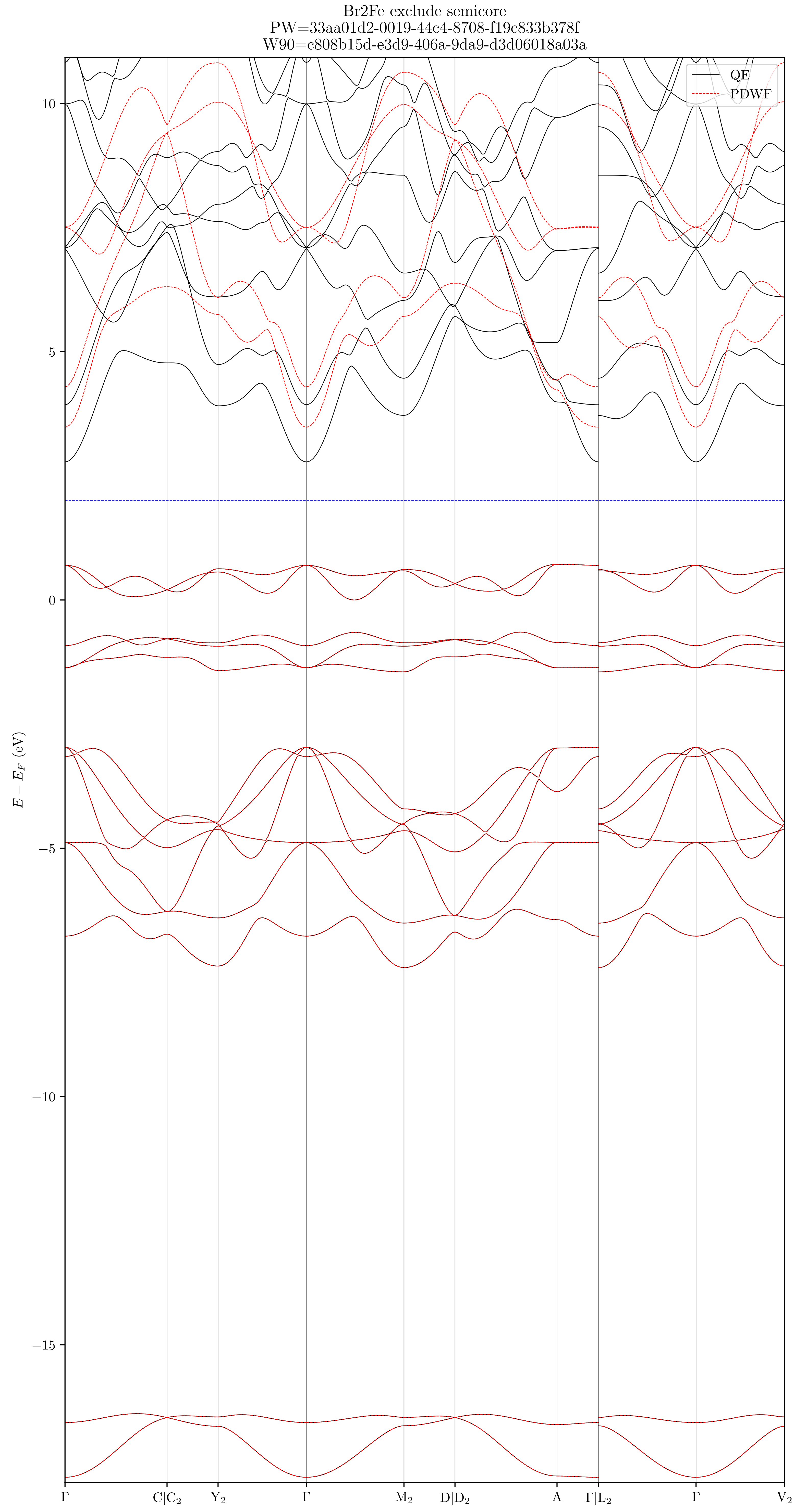

PDWF band distance (meV) and τ ($\AA$)

η_0	η_2	η_4	$\eta_0^{\max}$	$\eta_2^{\max}$	$\eta_4^{\max}$	τ
0.7	0.7	71.3	4.5	4.5	686.9	2.209

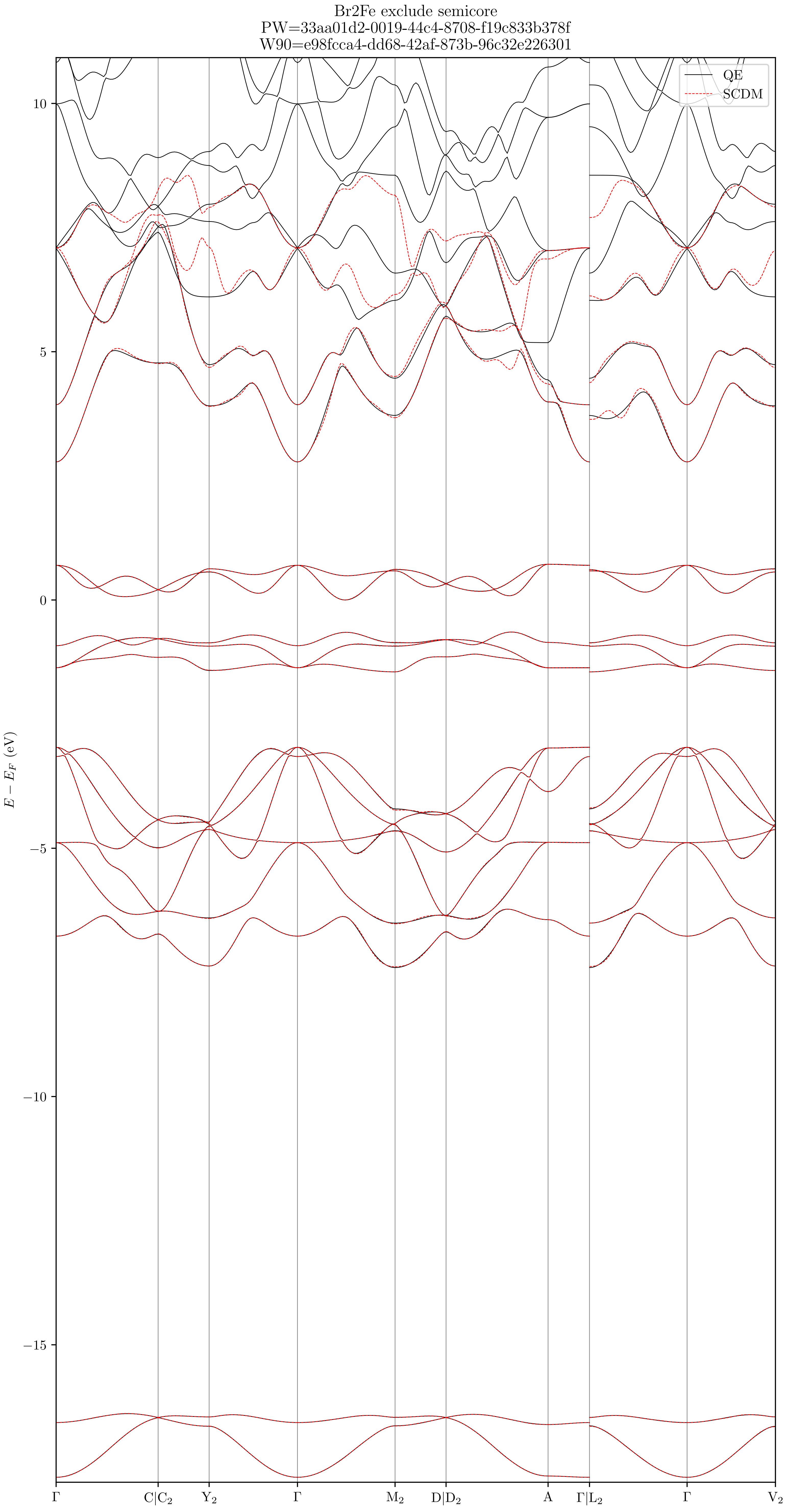

SCDM band distance (meV) and τ ($\AA$)

η_0	η_2	η_4	$\eta_0^{\max}$	$\eta_2^{\max}$	$\eta_4^{\max}$	τ
4.1	3.9	7.3	25.7	25.7	95.4	2.928

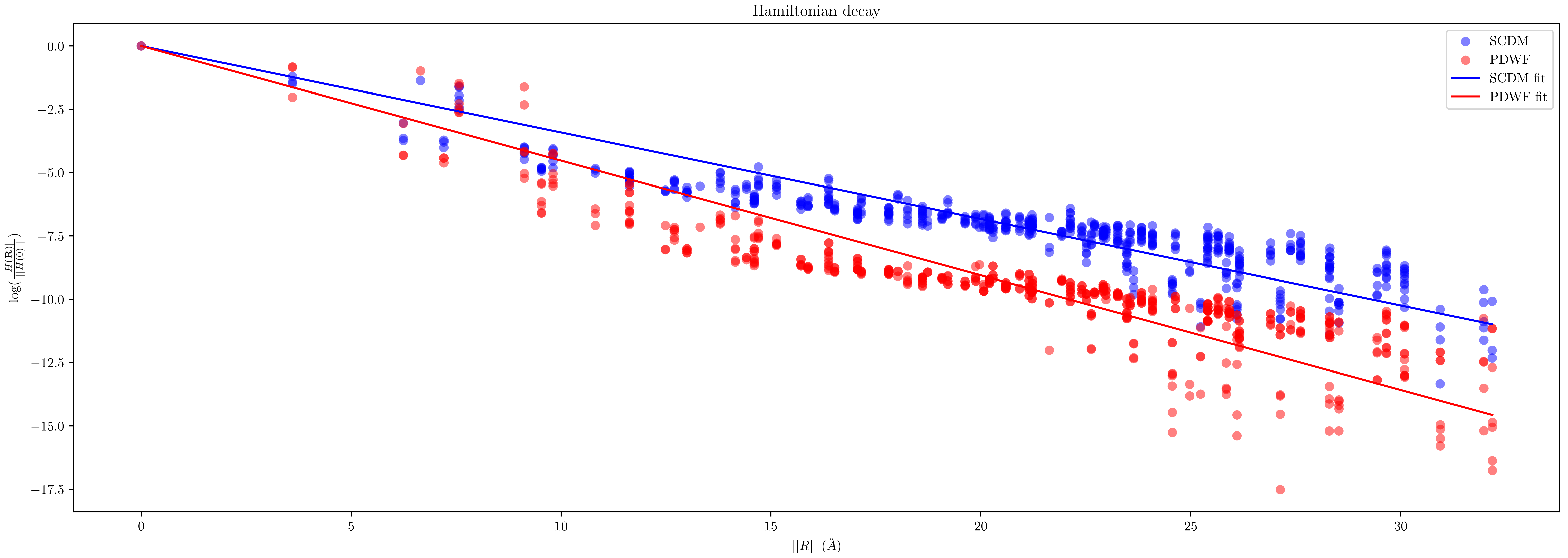

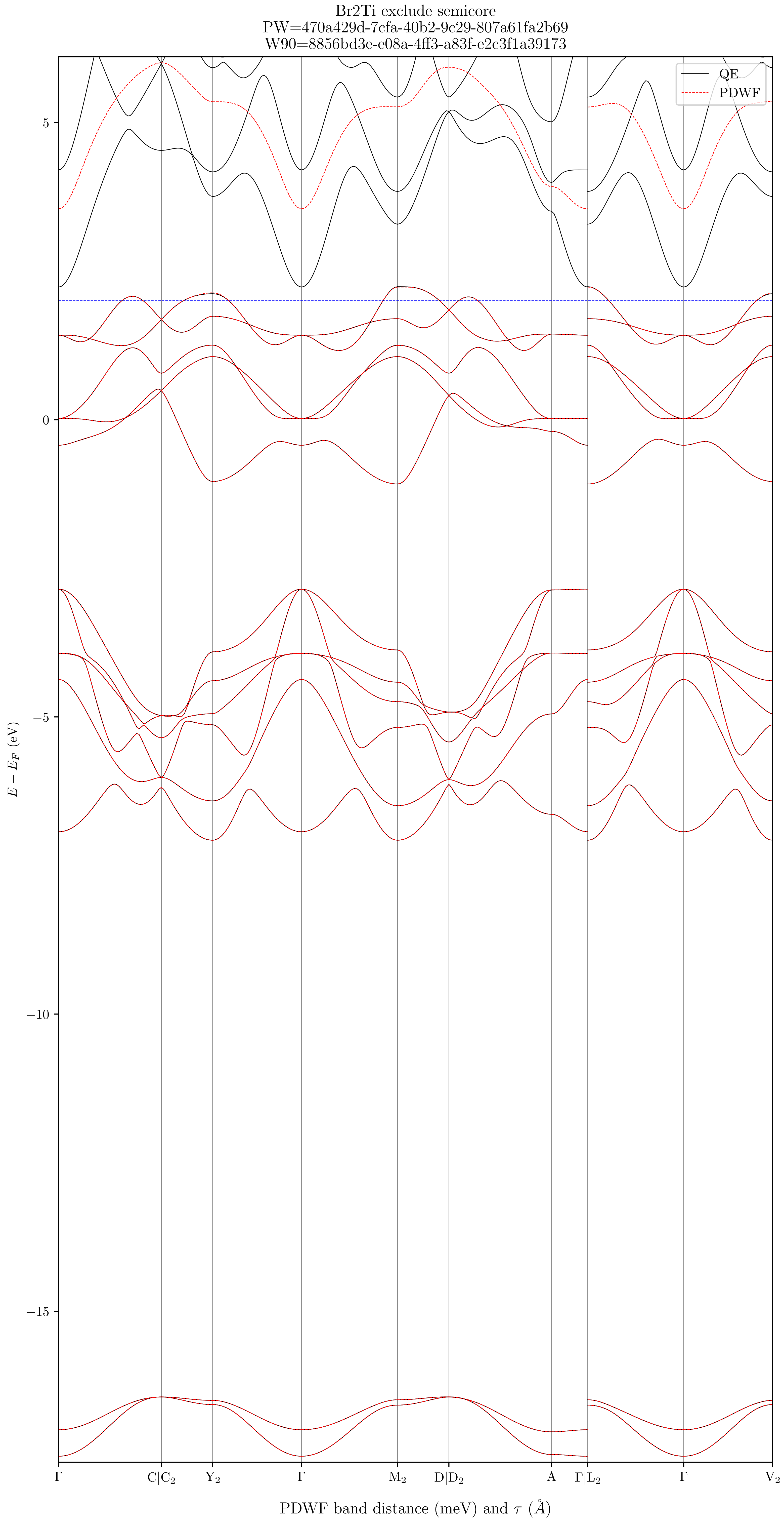

η_0	η_2	η_4	$\eta_0^{\max}$	$\eta_2^{\max}$	$\eta_4^{\max}$	τ
0.3	1.2	152.2	1.5	3.6	1308.5	2.176

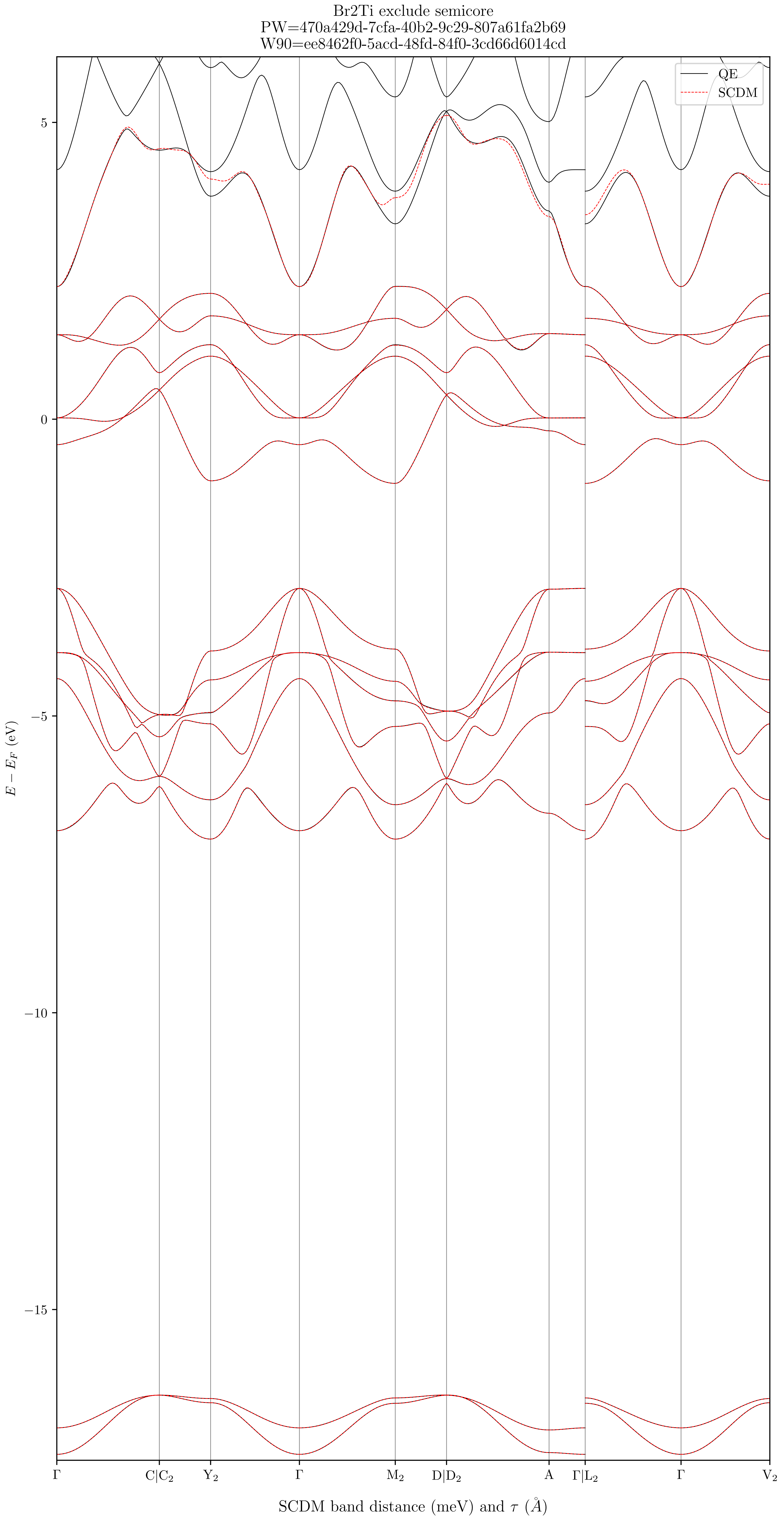

η_0	η_2	η_4	$\eta_0^{\max}$	$\eta_2^{\max}$	$\eta_4^{\max}$	τ
1.4	1.4	24.6	9.8	9.8	427.5	2.483

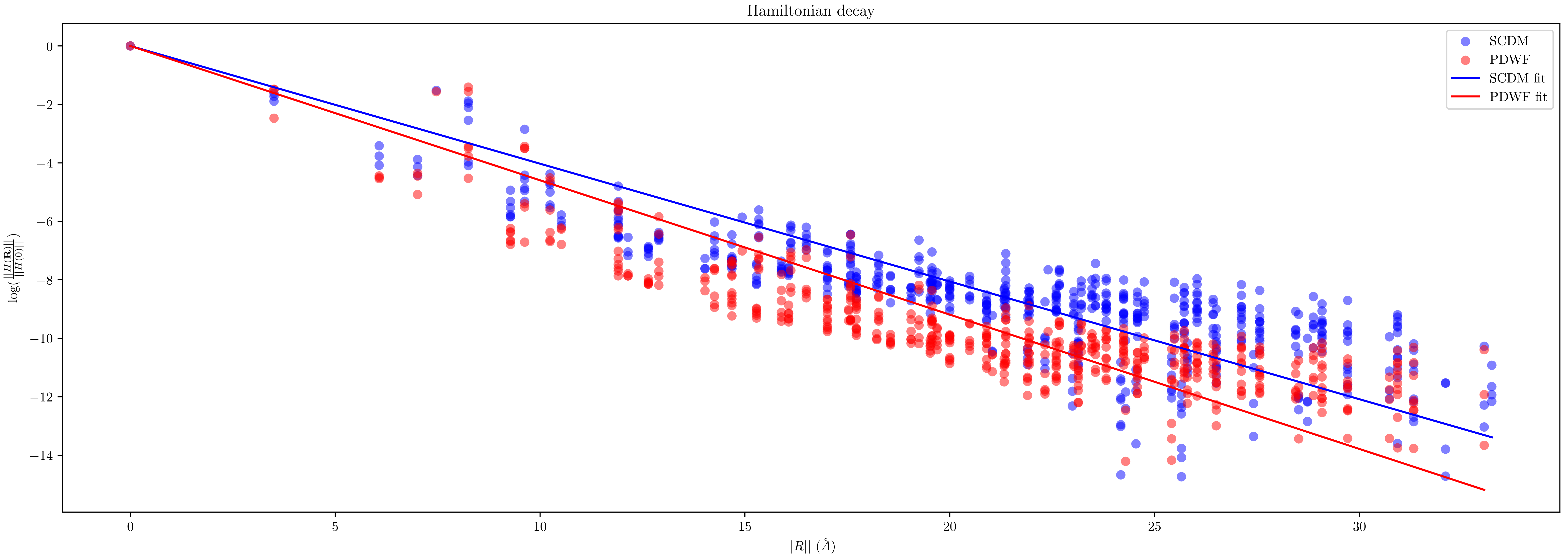

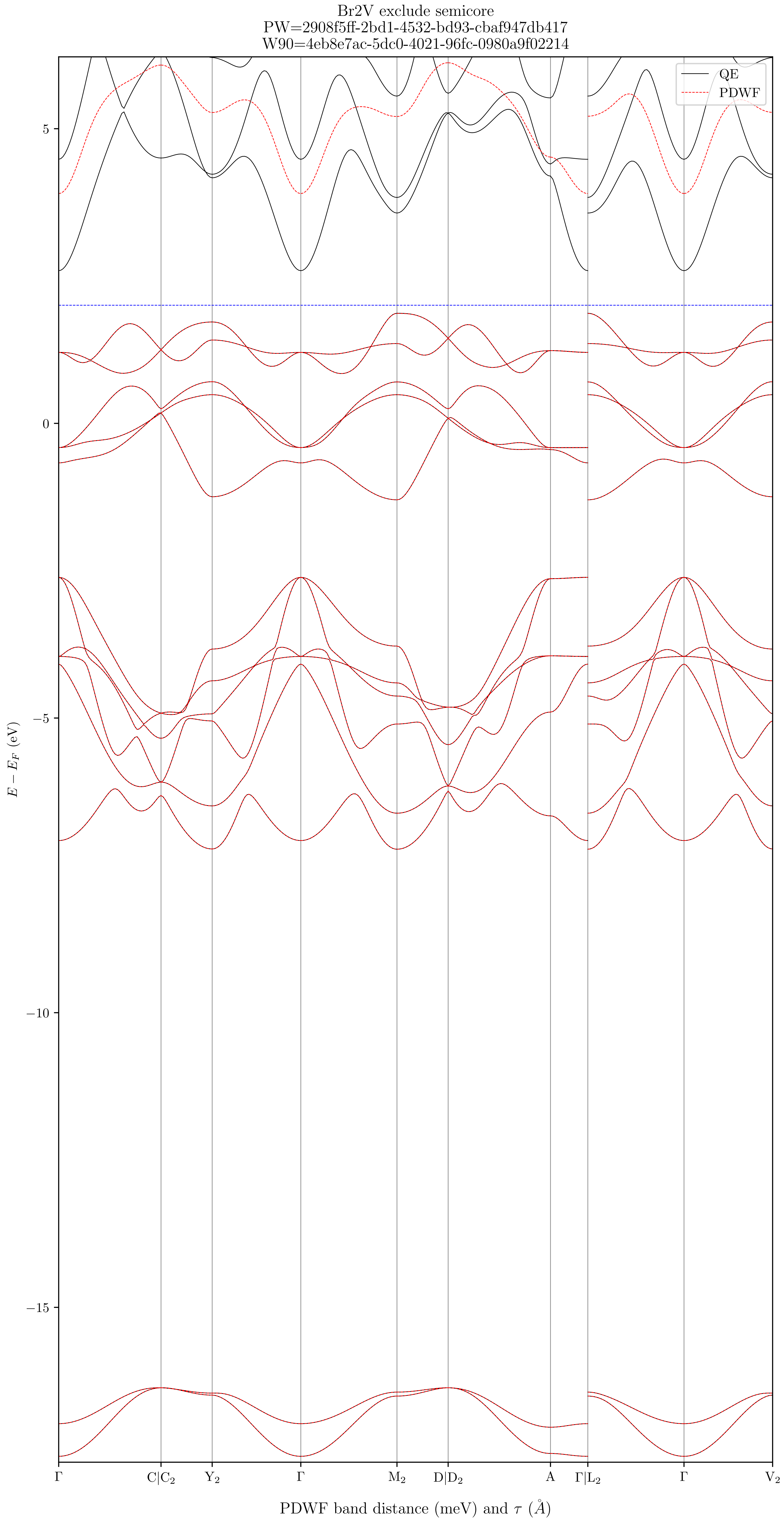

η_0	η_2	η_4	η_0^{max}	η_2^{max}	η_4^{max}	τ
0.2	0.4	113.2	1.3	2.3	1119.6	1.787

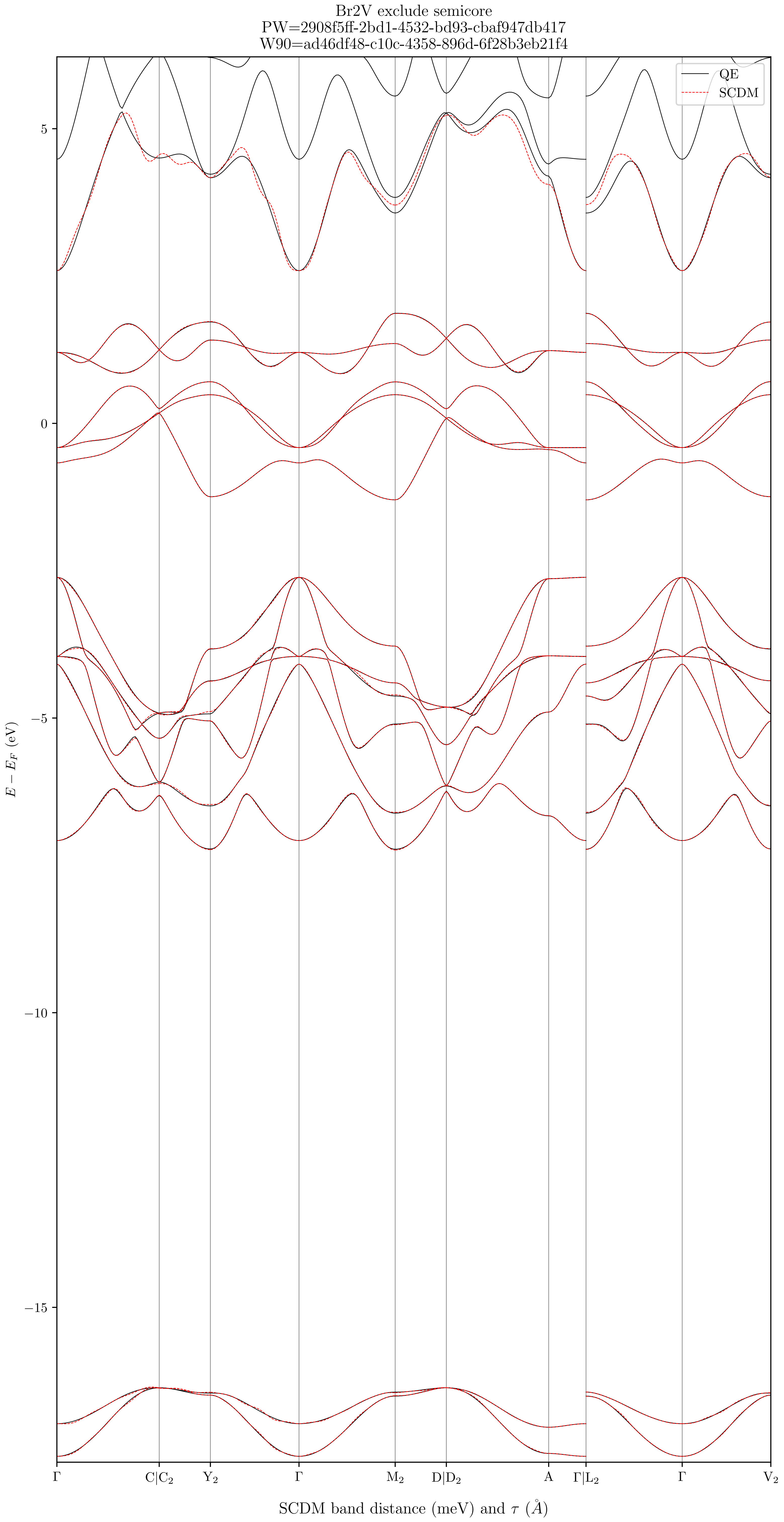

η_0	η_2	η_4	η_0^{max}	η_2^{max}	η_4^{max}	τ
7.6	6.8	16.3	46.0	46.0	201.3	2.864

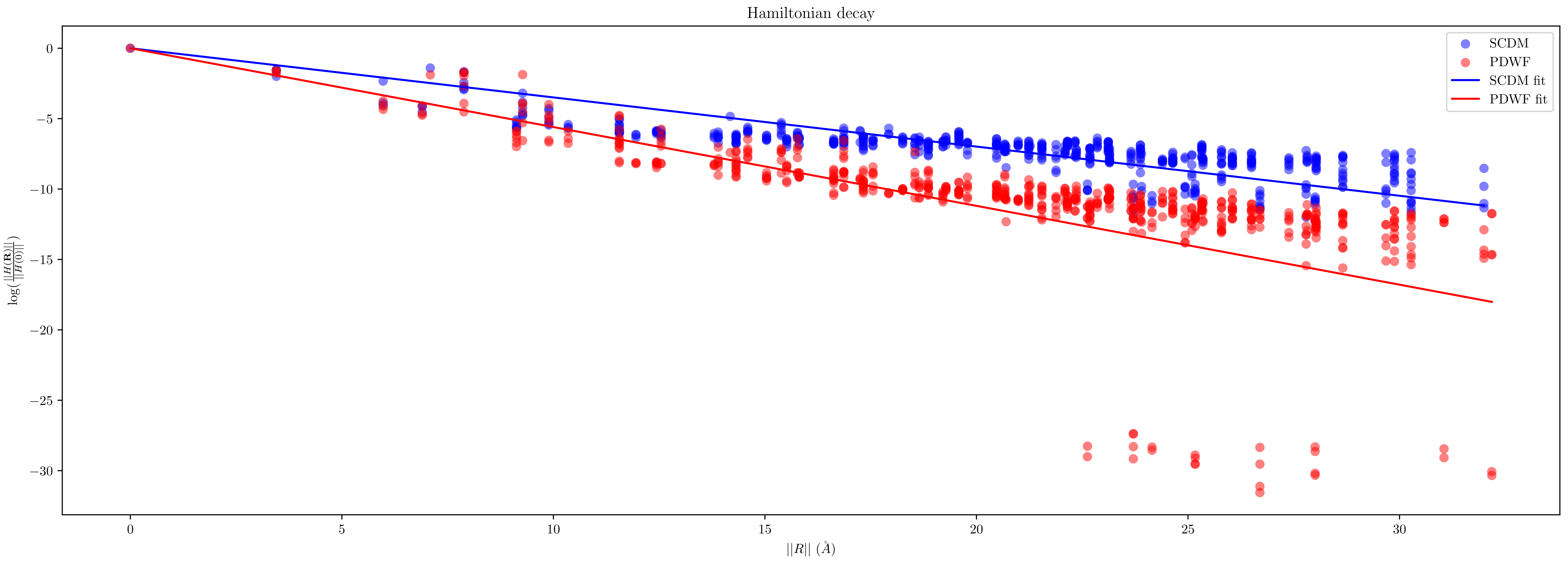

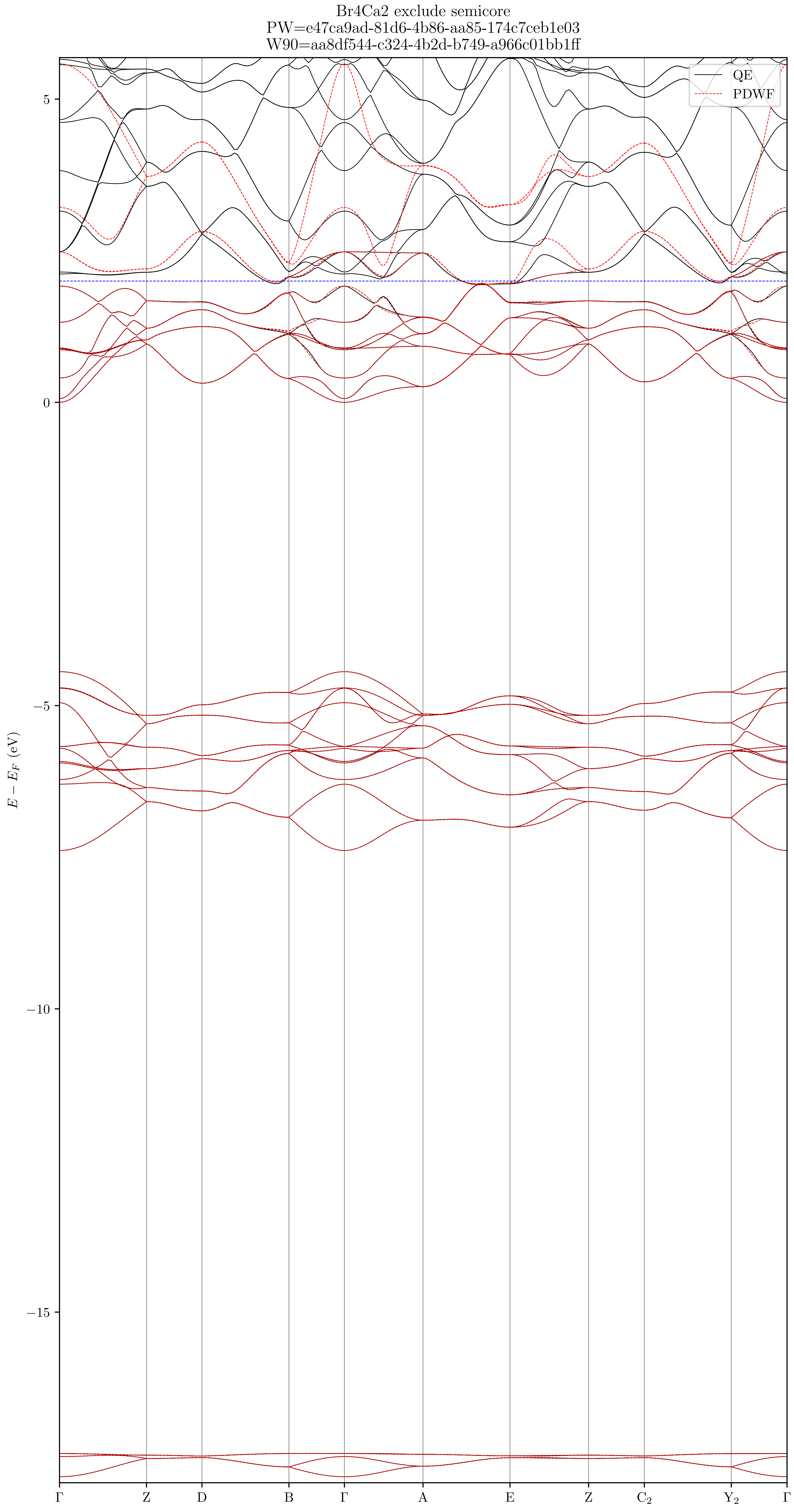

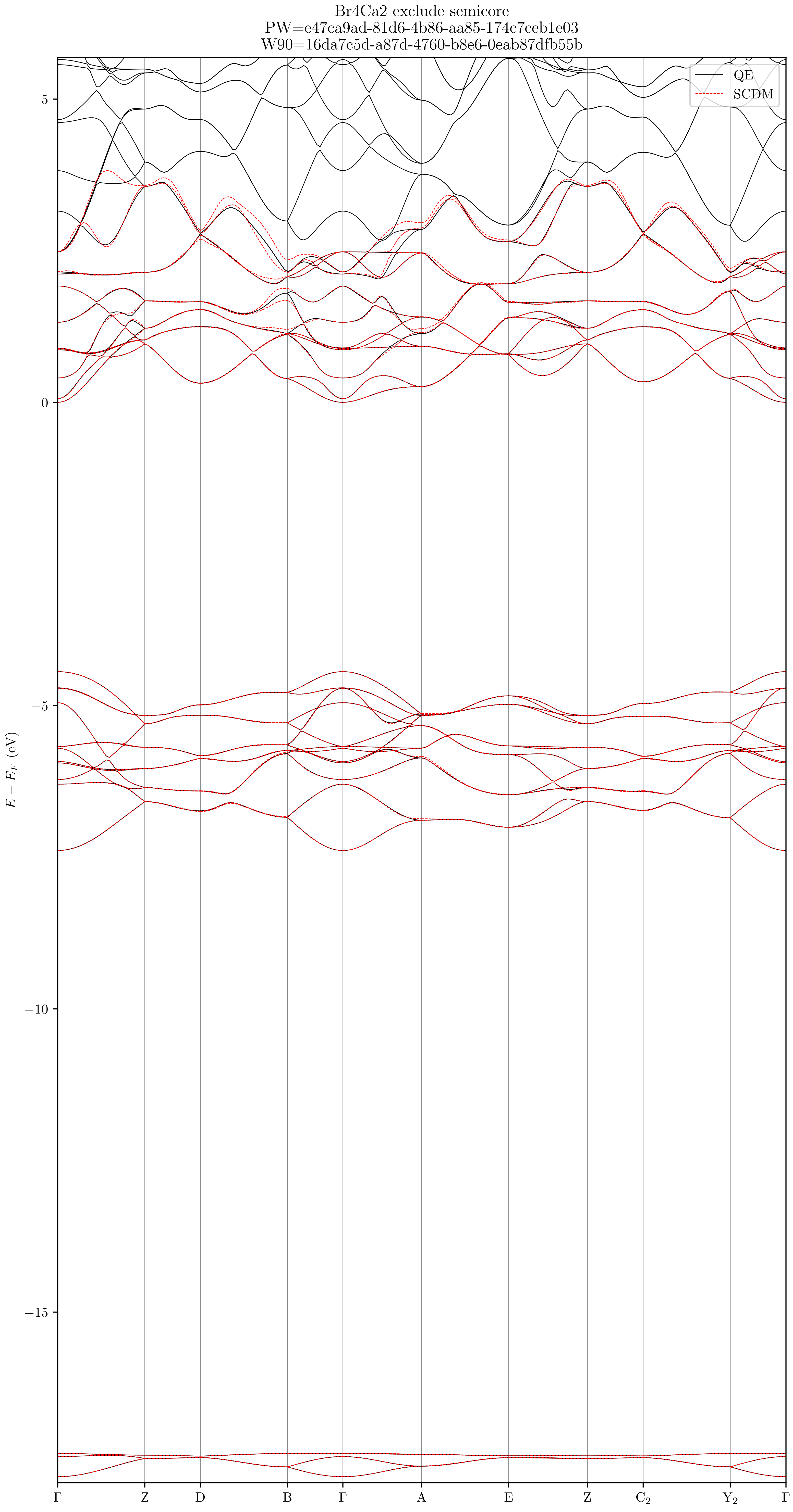

PDWF band distance (meV) and τ ($\AA$)

η_0	η_2	η_4	$\eta_0^{\max}$	$\eta_2^{\max}$	$\eta_4^{\max}$	τ
0.8	0.8	1.7	4.9	4.9	30.4	2.500

SCDM band distance (meV) and τ ($\AA$)

η_0	η_2	η_4	$\eta_0^{\max}$	$\eta_2^{\max}$	$\eta_4^{\max}$	τ
3.3	3.3	3.4	27.3	27.3	27.3	2.896

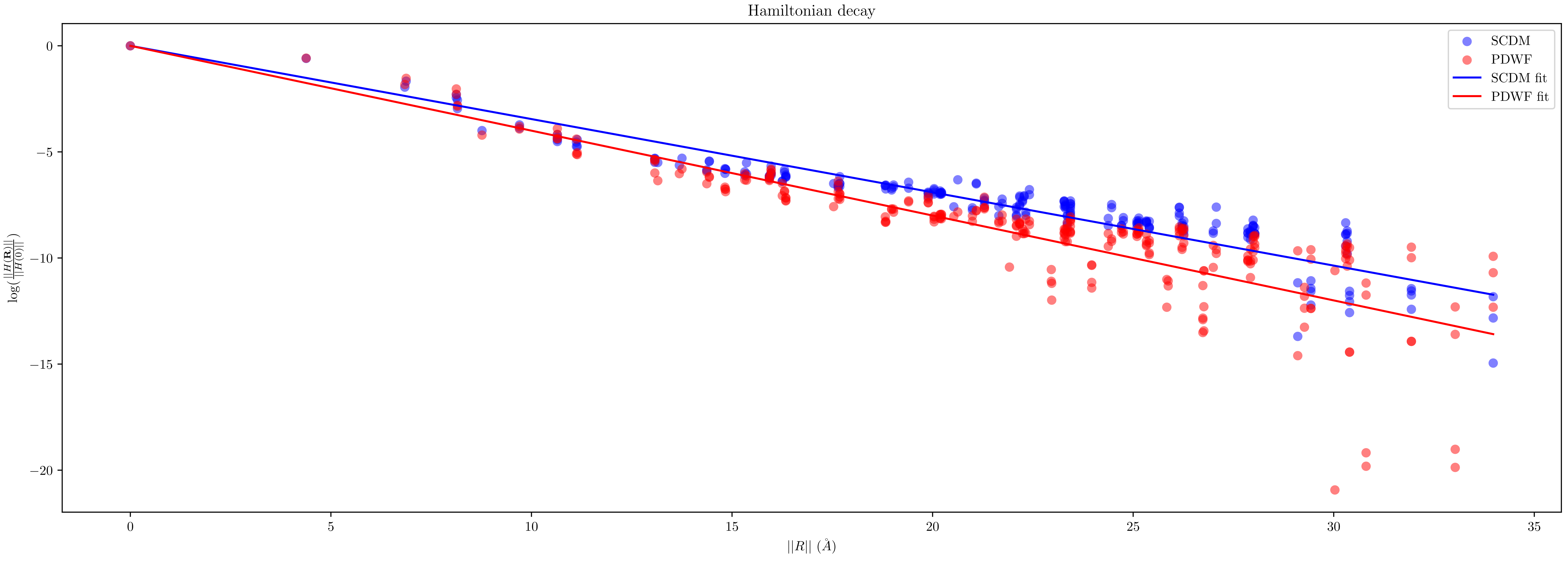

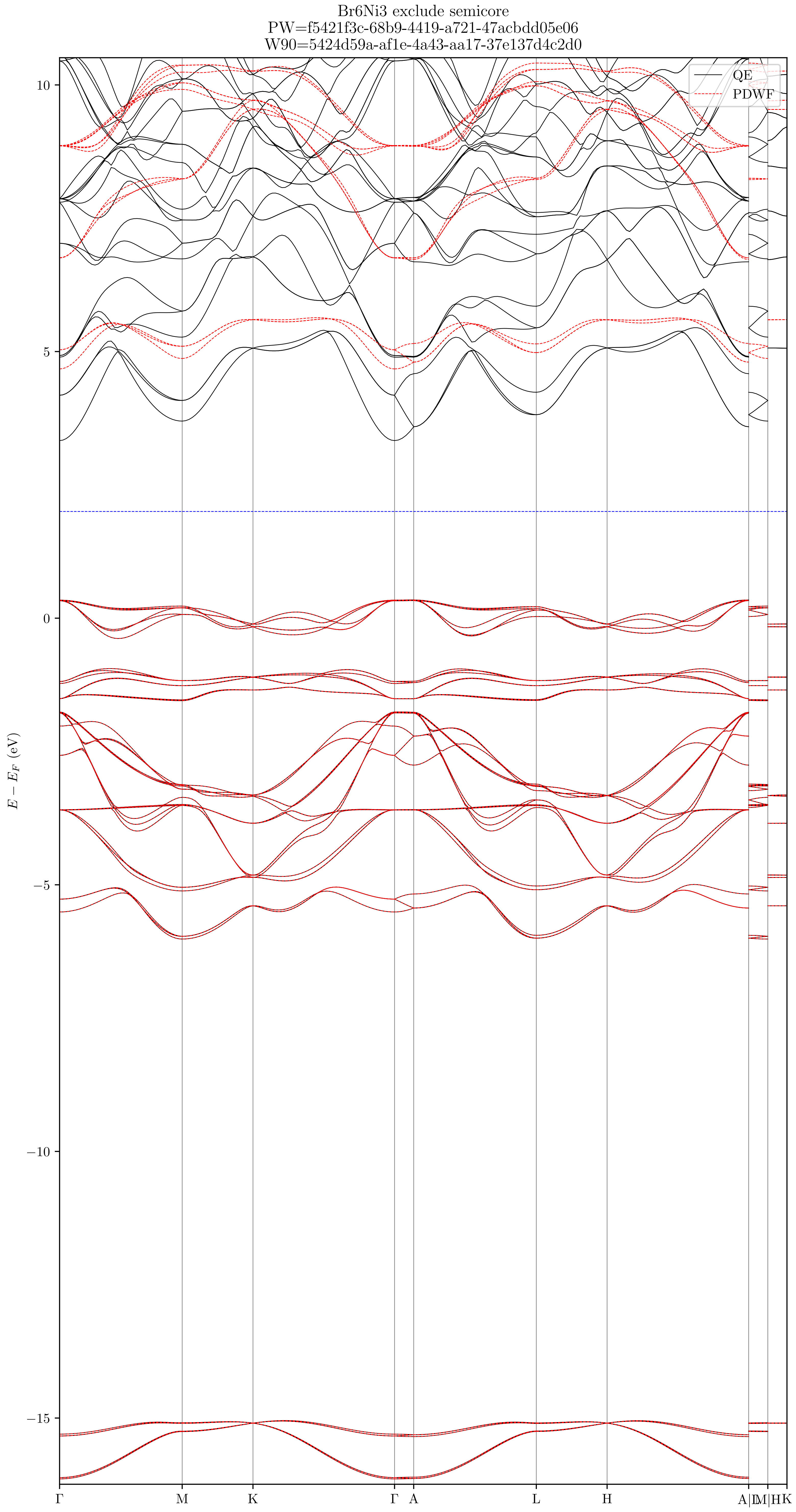

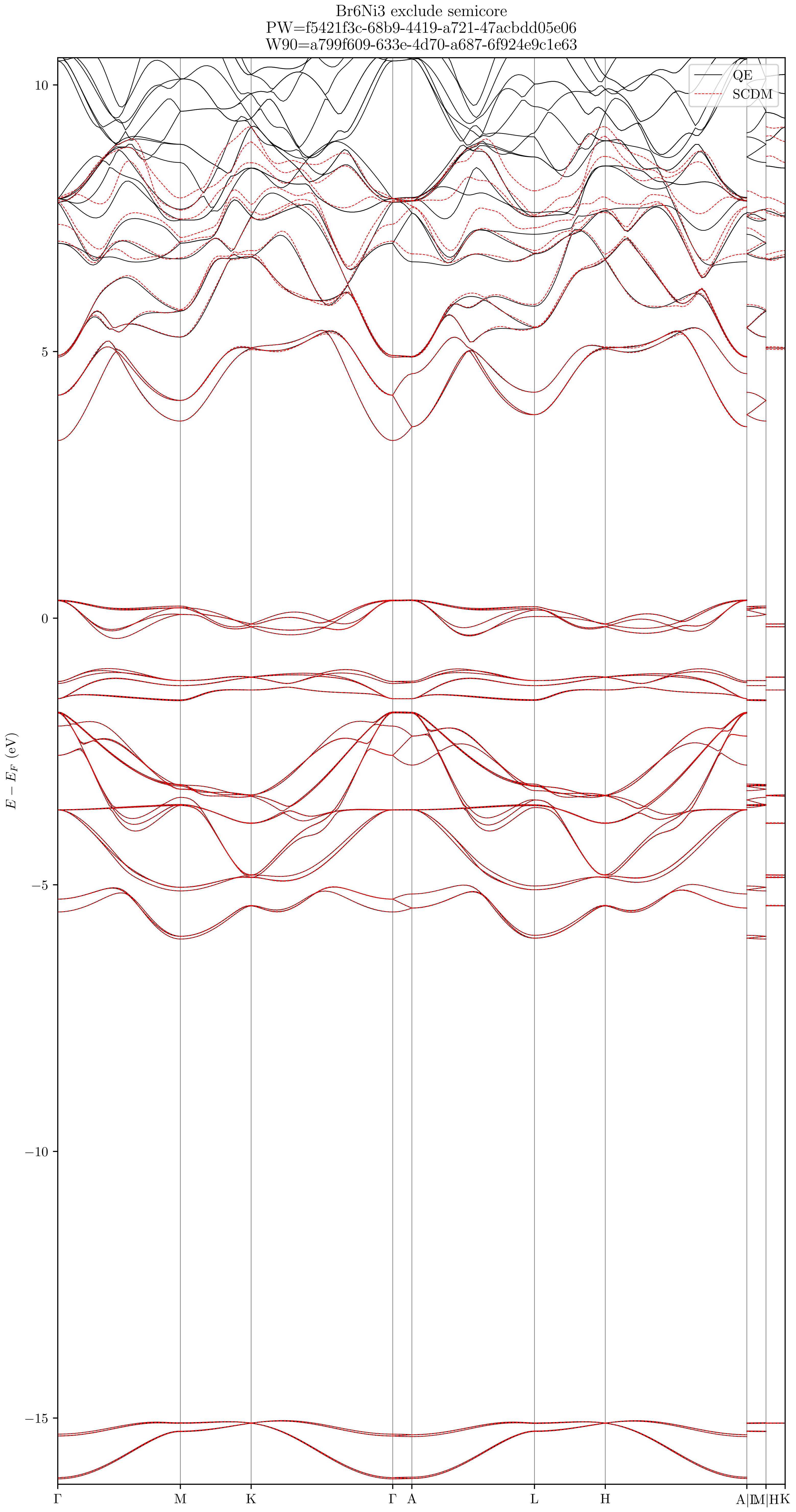

PDWF band distance (meV) and τ ($\AA$)

η_0	η_2	η_4	$\eta_0^{\max}$	$\eta_2^{\max}$	$\eta_4^{\max}$	τ
0.3	0.3	15.2	1.6	1.6	46.2	2.504

SCDM band distance (meV) and τ ($\AA$)

η_0	η_2	η_4	$\eta_0^{\max}$	$\eta_2^{\max}$	$\eta_4^{\max}$	τ
1.8	1.7	1.8	11.4	11.4	11.4	3.427

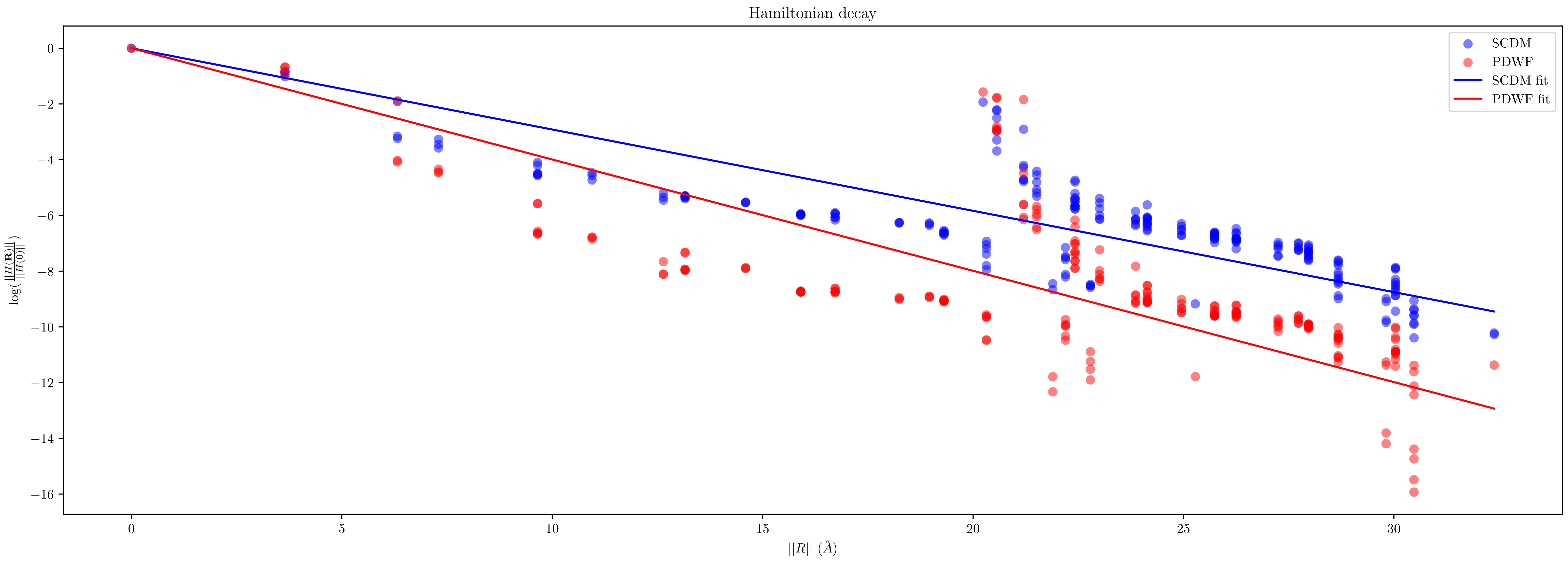

Br6Zr2 exclude semicore
PW=16beb9da-db31-4975-84ac-c0d4097a5f0c
W90=d77f11d3-6e40-46ce-af4f-2da9a0a631f1

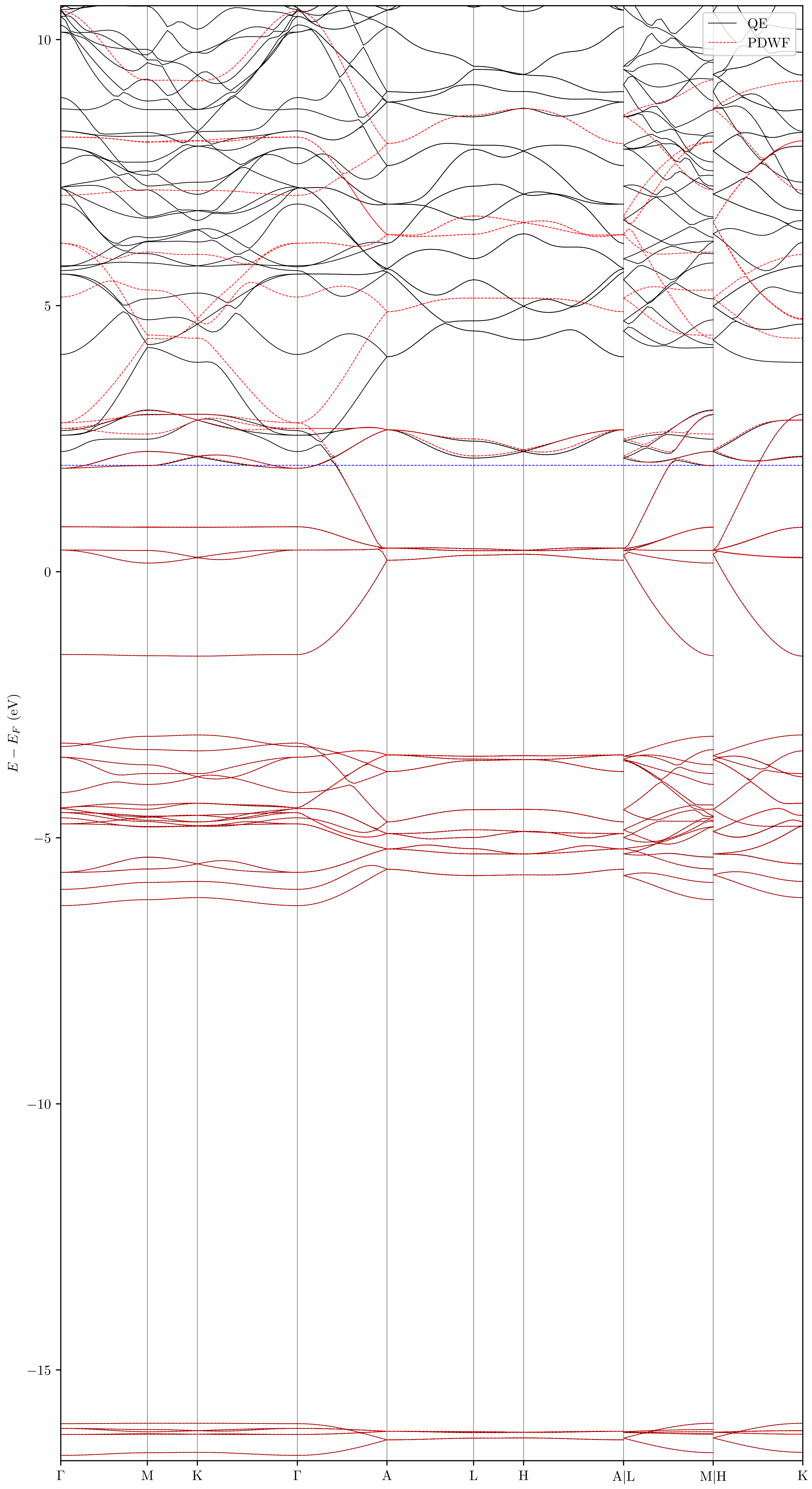

Br6Zr2 exclude semicore
PW=16beb9da-db31-4975-84ac-c0d4097a5f0c
W90=ad0ac761-af46-4e8c-a946-5f069cd6818f

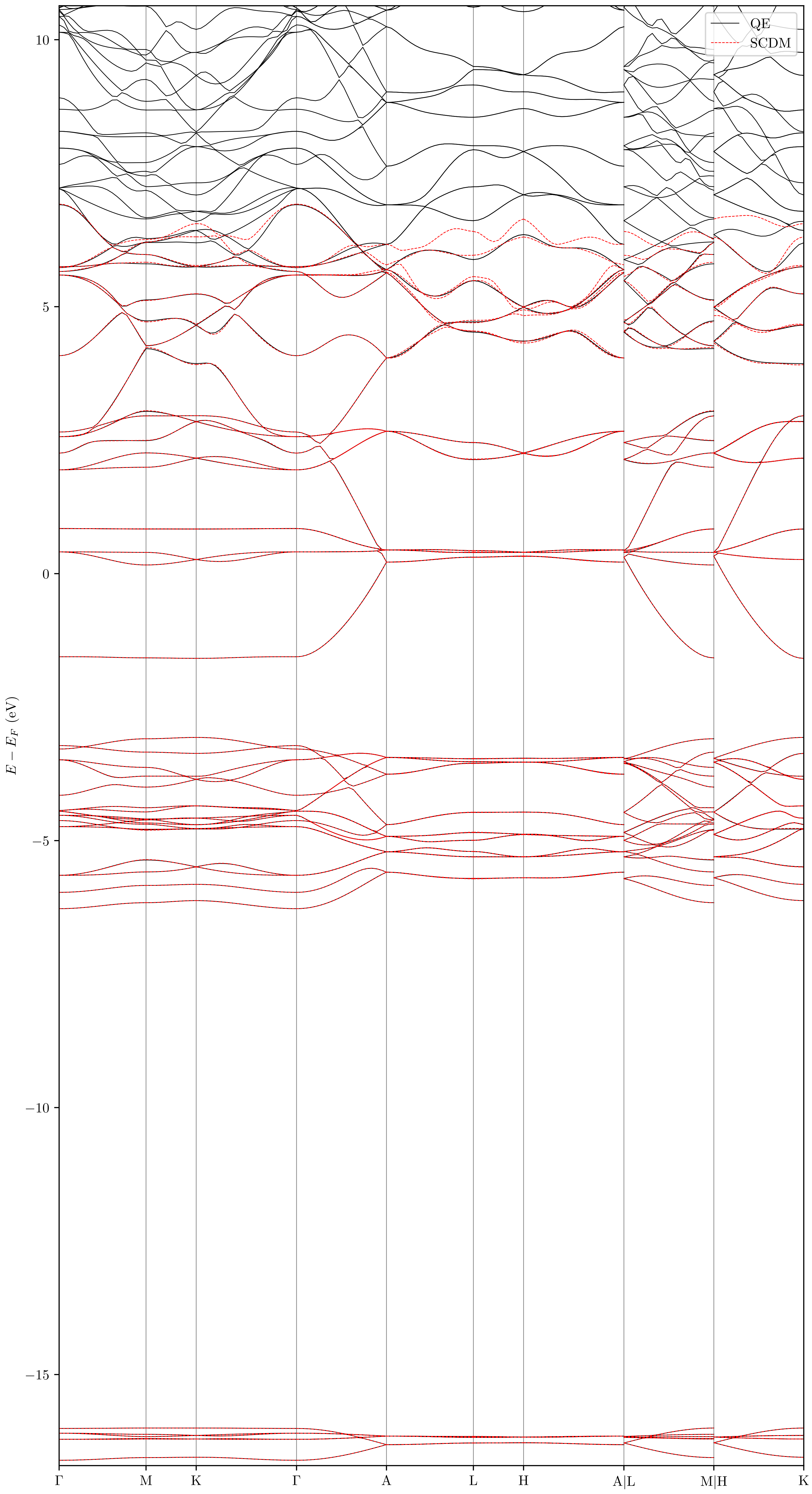

PDWF band distance (meV) and τ ($\AA$)

η_0	η_2	η_4	$\eta_0^{\max}$	$\eta_2^{\max}$	$\eta_4^{\max}$	τ
1.0	3.1	47.9	5.1	14.3	739.9	2.981

SCDM band distance (meV) and τ ($\AA$)

η_0	η_2	η_4	$\eta_0^{\max}$	$\eta_2^{\max}$	$\eta_4^{\max}$	τ
1.7	1.7	2.4	12.4	12.4	17.1	3.220

Hamiltonian decay

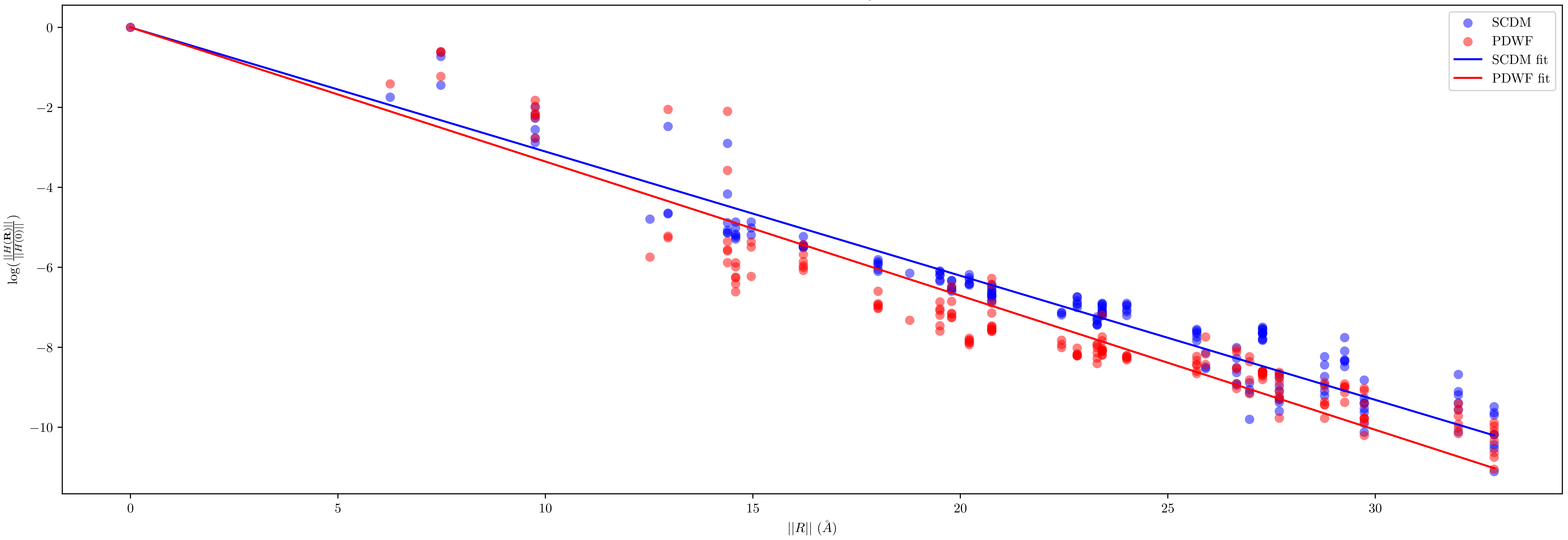

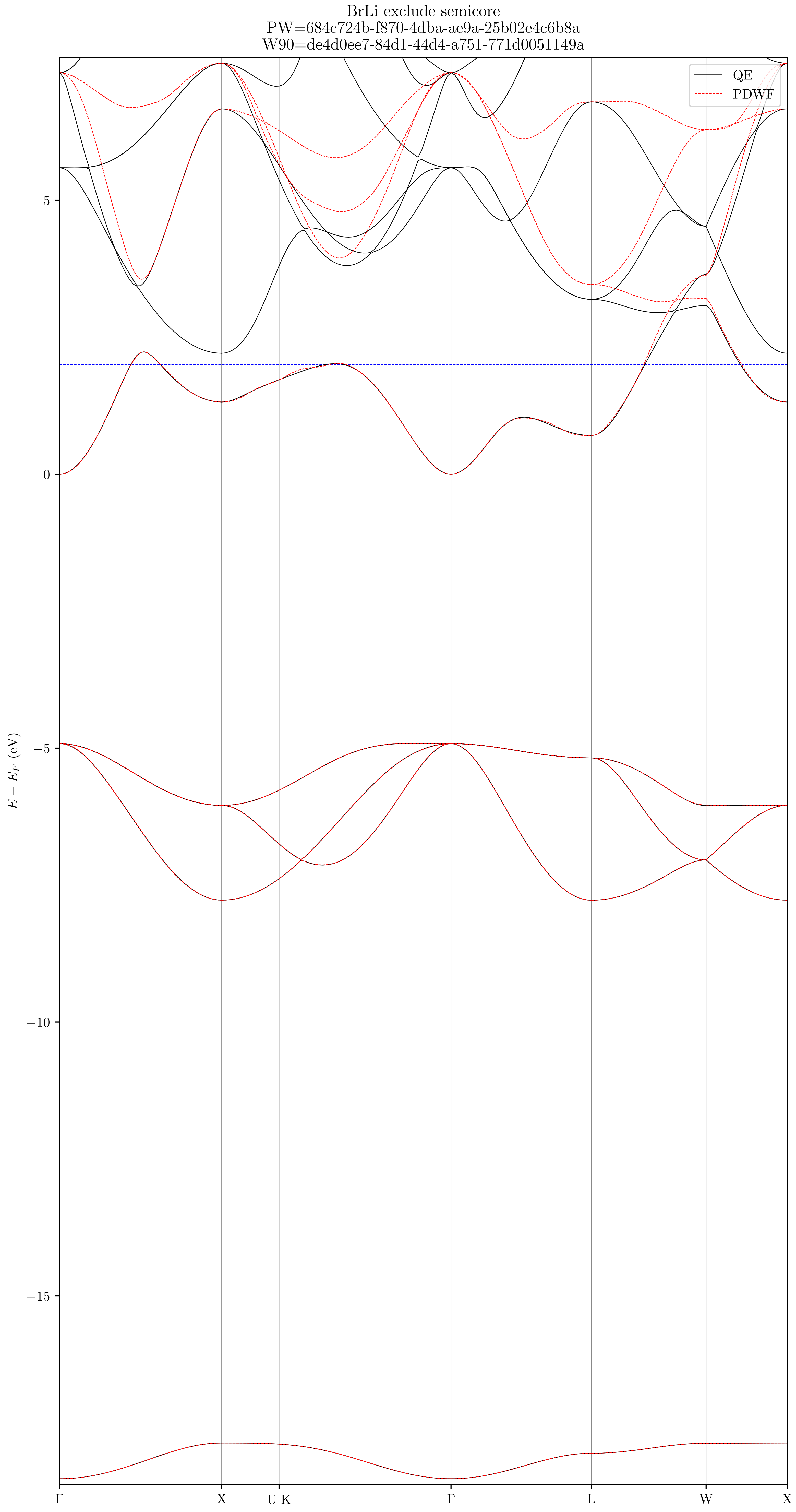

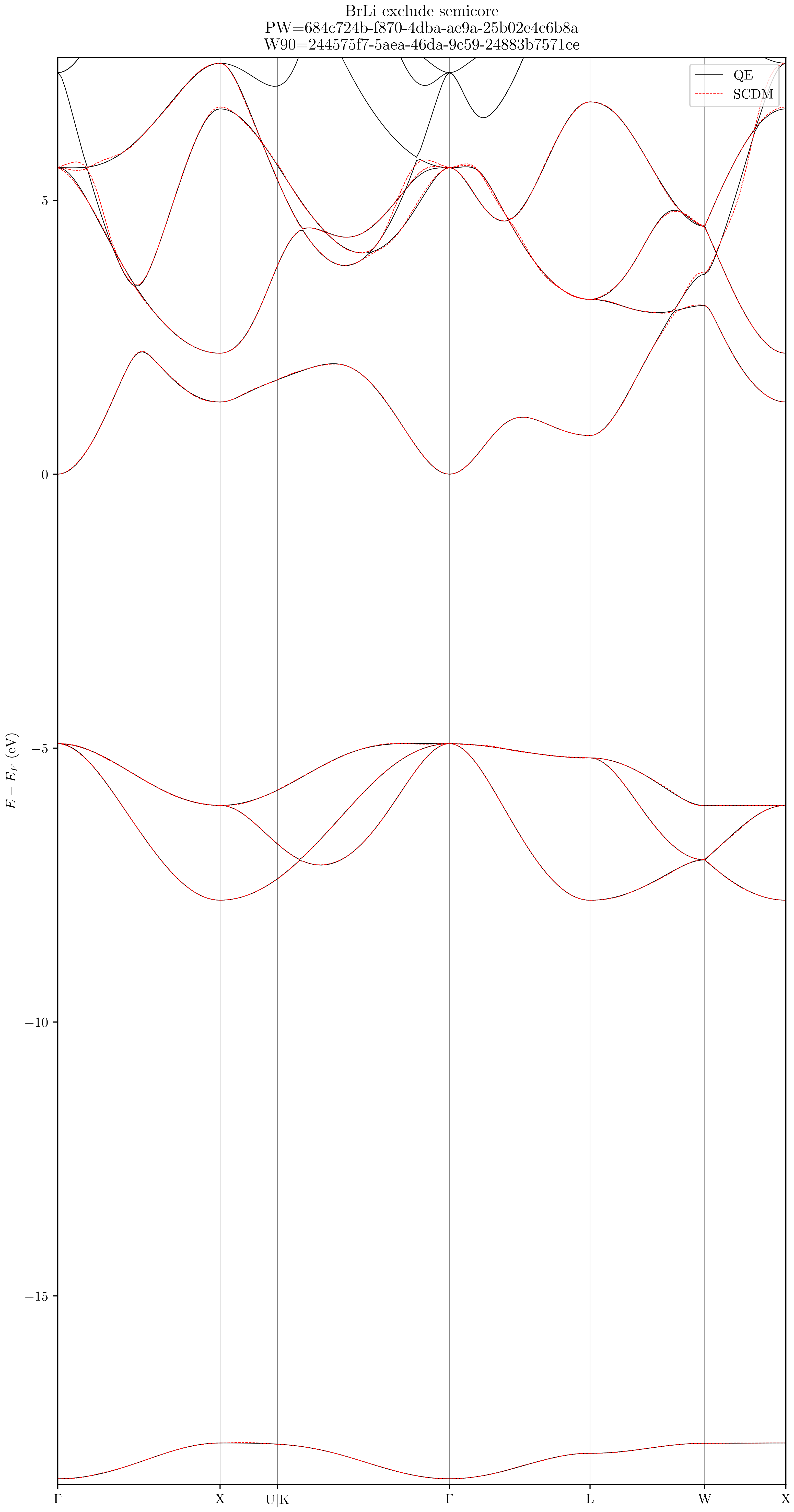

PDWF band distance (meV) and τ ($\AA$)

η_0	η_2	η_4	$\eta_0^{\max}$	$\eta_2^{\max}$	$\eta_4^{\max}$	τ
1.6	1.6	4.1	9.6	9.6	28.7	2.191

SCDM band distance (meV) and τ ($\AA$)

η_0	η_2	η_4	$\eta_0^{\max}$	$\eta_2^{\max}$	$\eta_4^{\max}$	τ
3.9	3.9	4.1	16.7	16.7	16.7	2.482

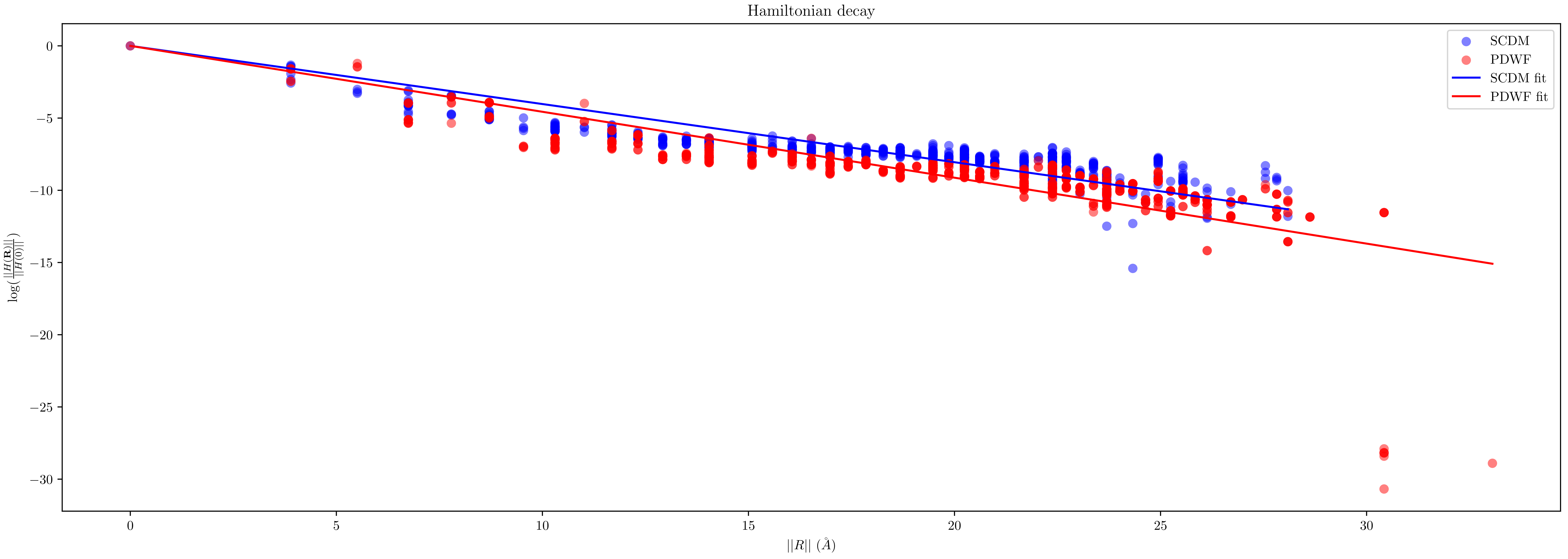

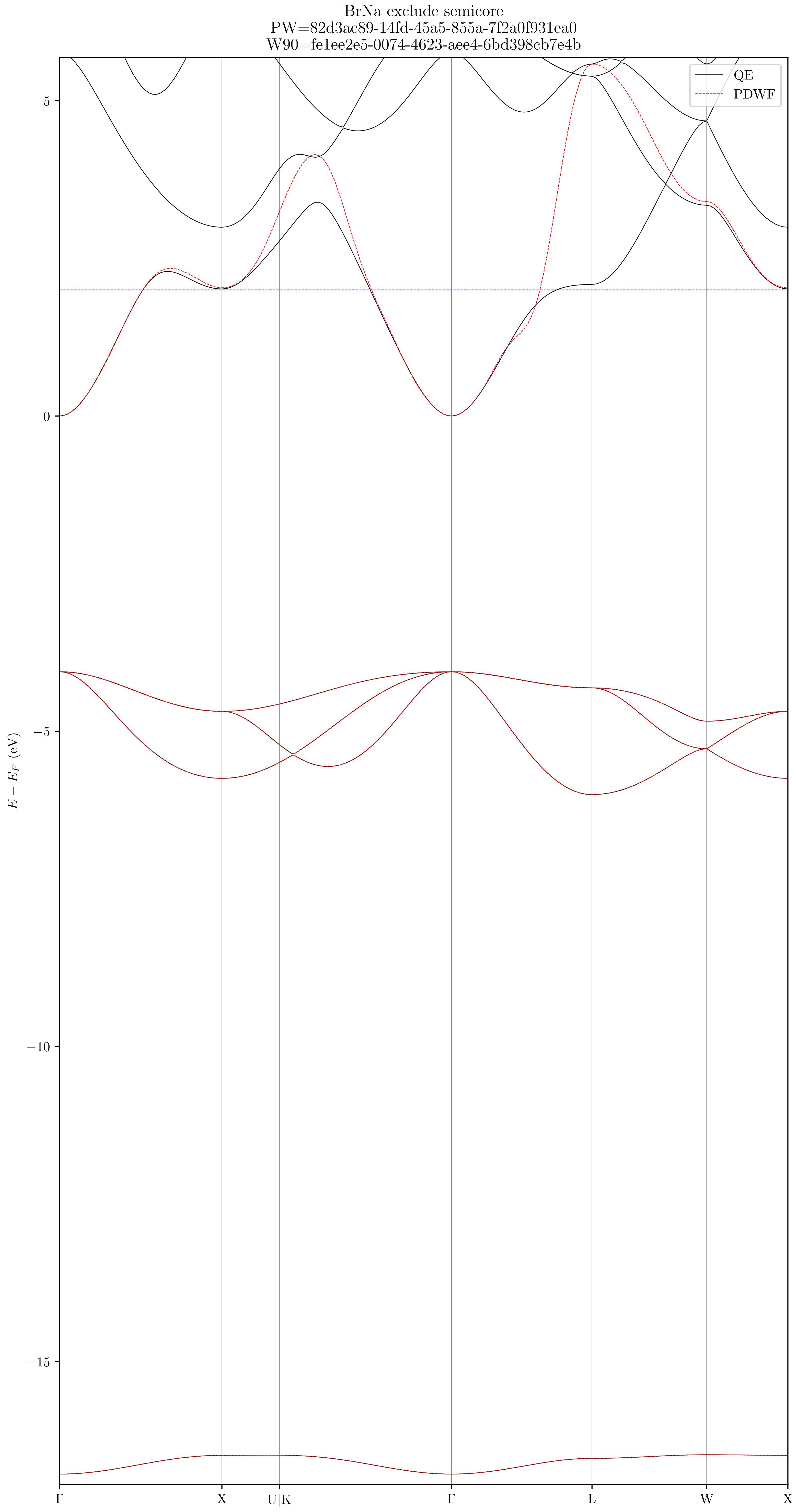

PDWF band distance (meV) and τ ($\AA$)

η_0	η_2	η_4	$\eta_0^{\max}$	$\eta_2^{\max}$	$\eta_4^{\max}$	τ
0.6	0.6	14.4	3.3	3.3	149.6	2.077

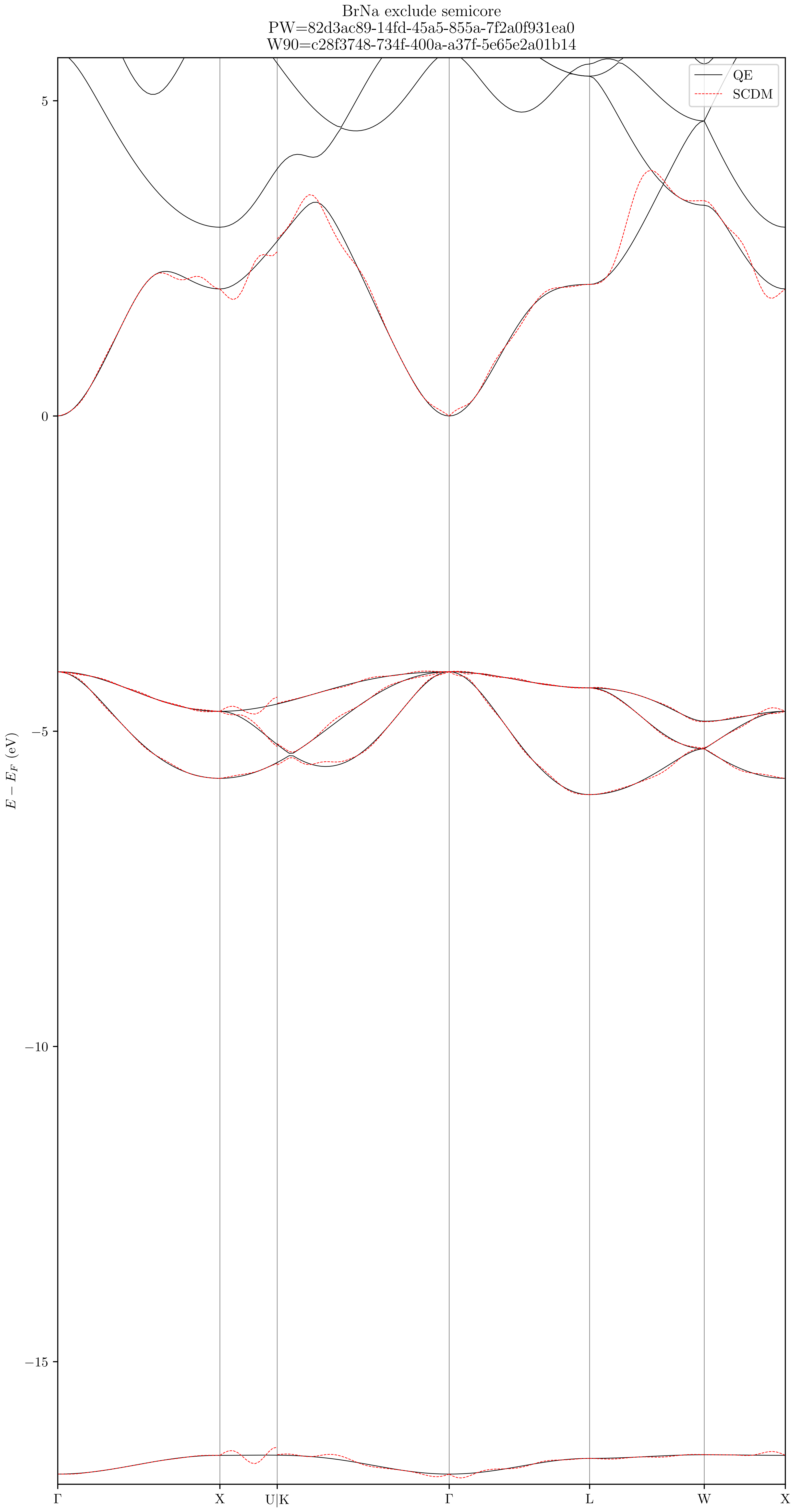

SCDM band distance (meV) and τ ($\AA$)

η_0	η_2	η_4	$\eta_0^{\max}$	$\eta_2^{\max}$	$\eta_4^{\max}$	τ
23.4	23.5	27.8	134.4	134.4	134.4	3.480

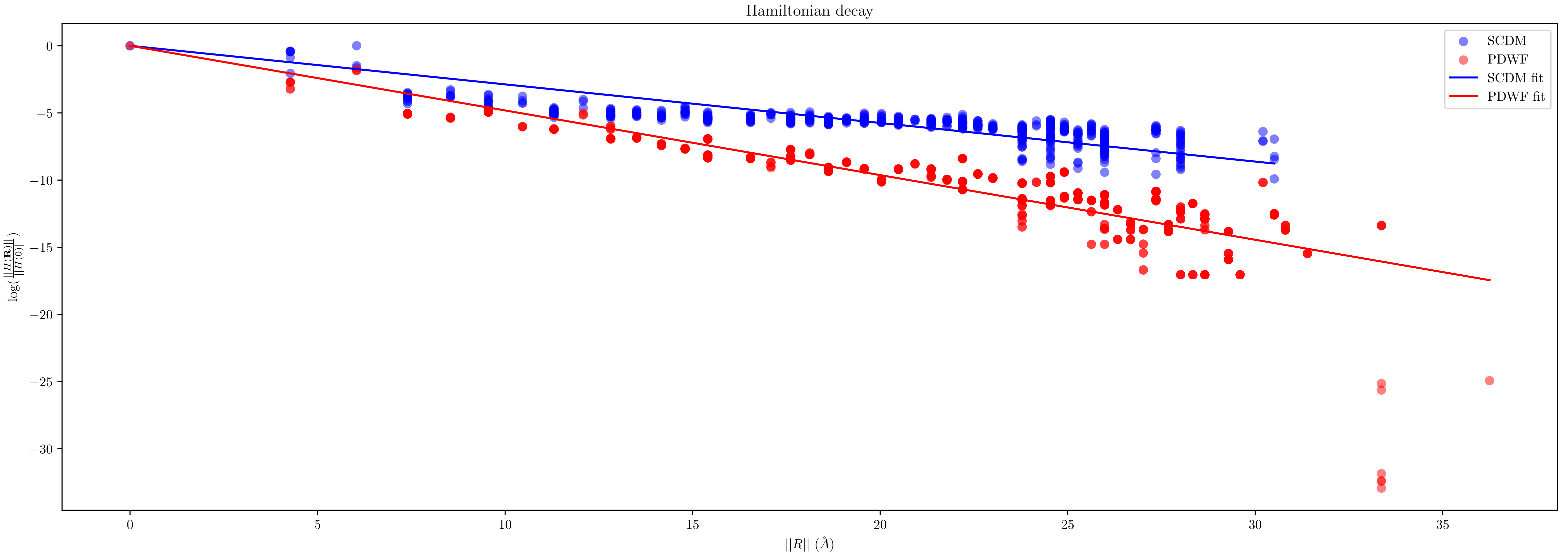

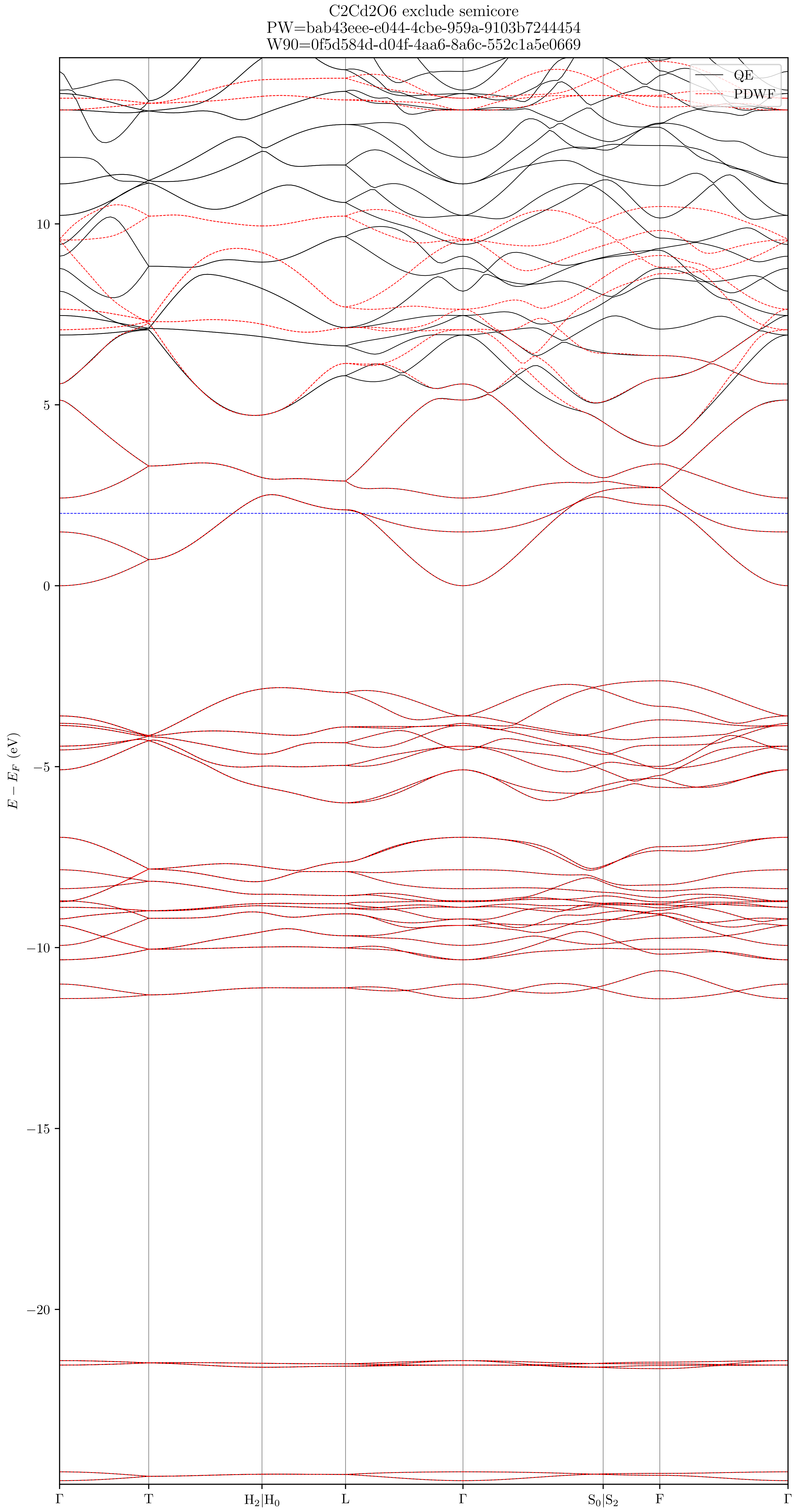

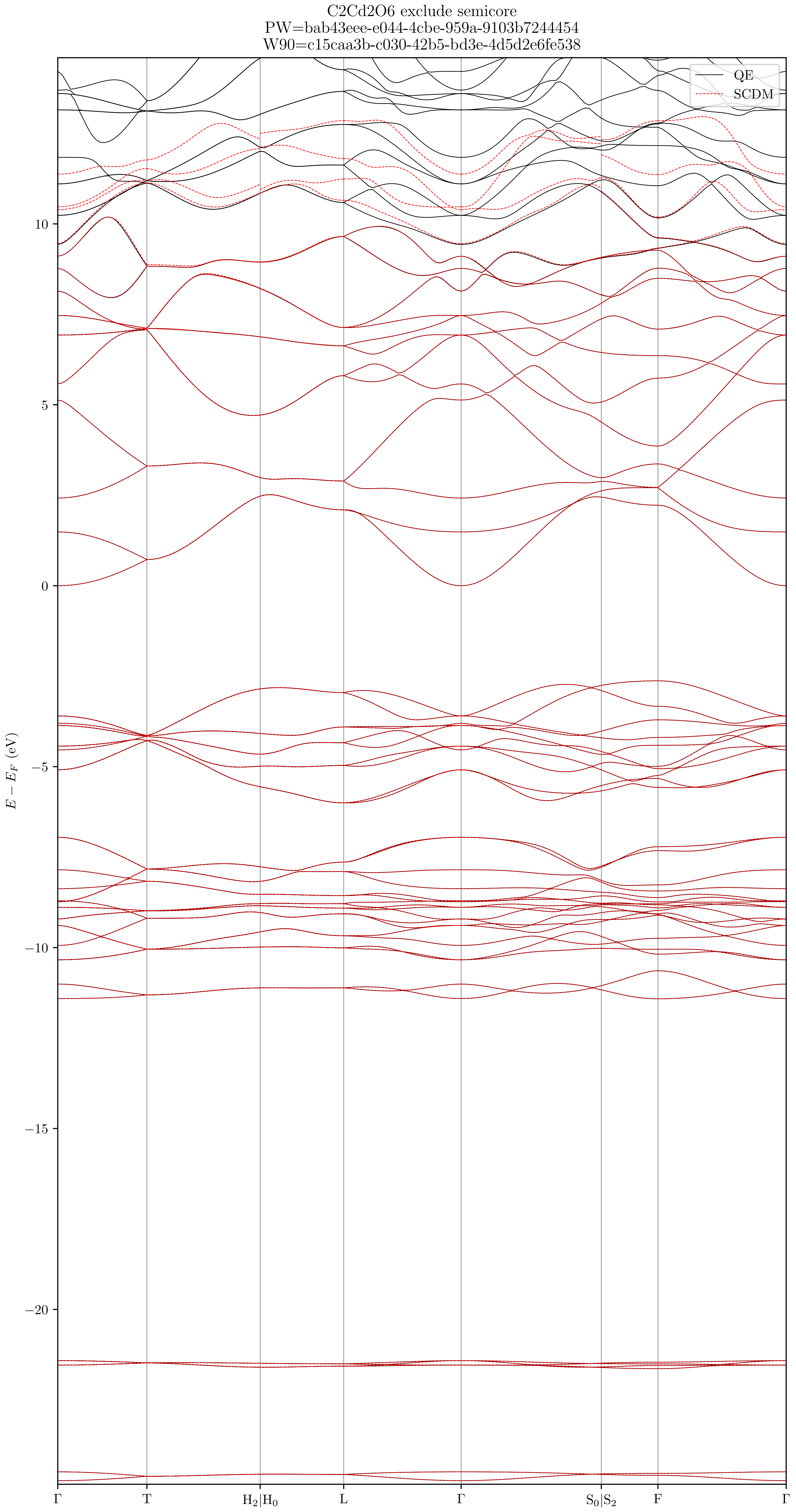

PDWF band distance (meV) and τ ($\AA$)

η_0	η_2	η_4	$\eta_0^{\max}$	$\eta_2^{\max}$	$\eta_4^{\max}$	τ
0.3	0.3	0.4	2.0	2.0	3.8	2.719

SCDM band distance (meV) and τ ($\AA$)

η_0	η_2	η_4	$\eta_0^{\max}$	$\eta_2^{\max}$	$\eta_4^{\max}$	τ
0.3	0.3	0.3	2.1	2.1	2.7	2.755

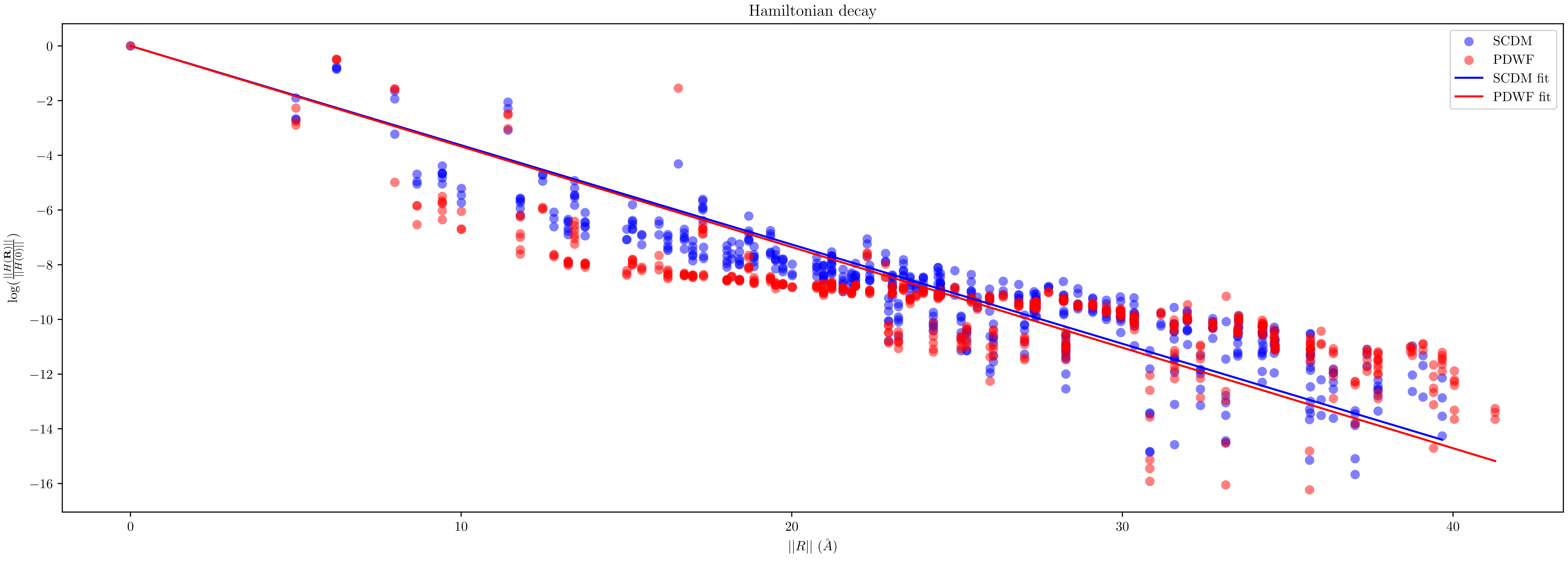

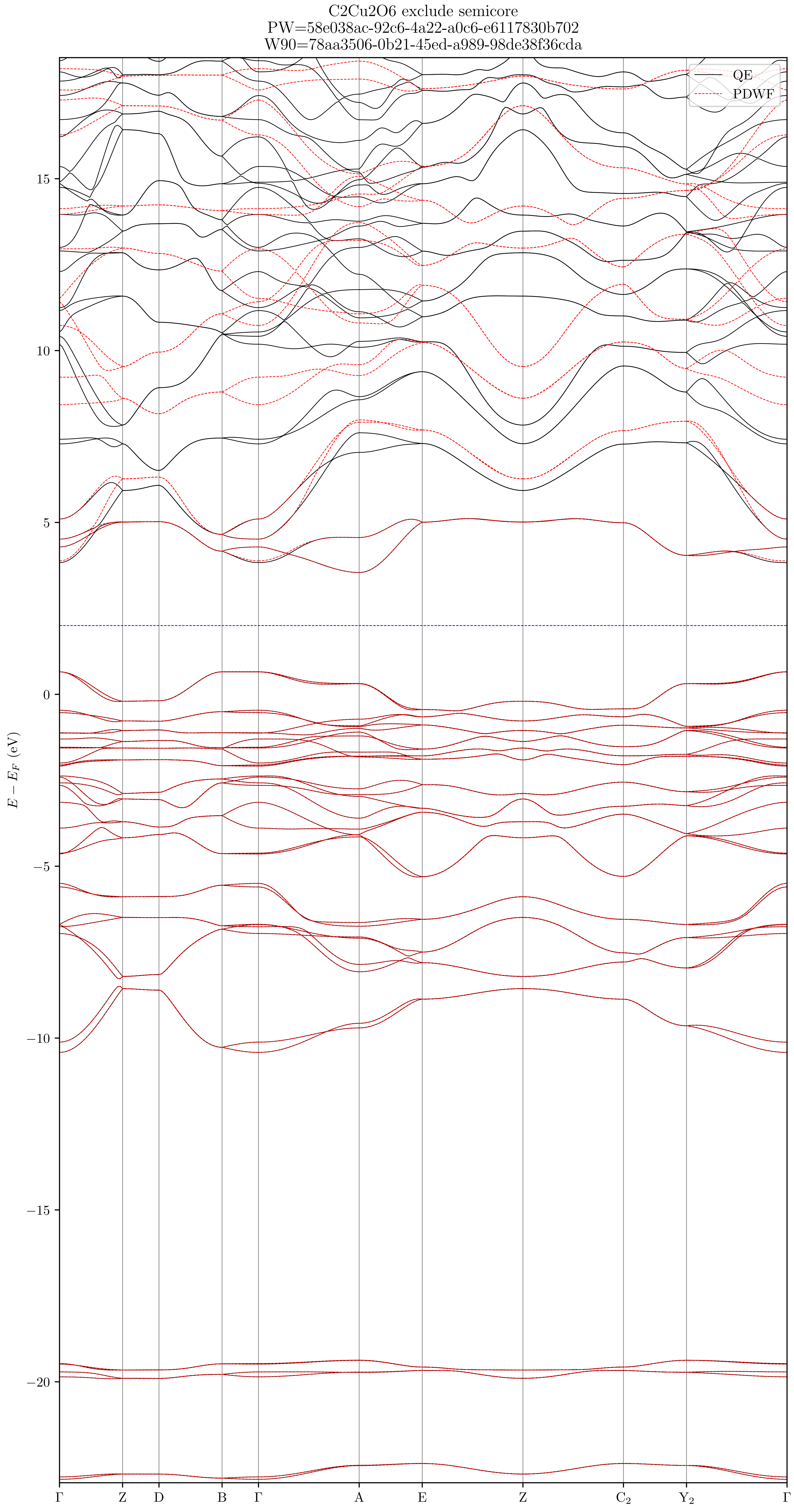

PDWF band distance (meV) and τ ($\mathring{A}$)

η_0	η_2	η_4	$\eta_0^{\max}$	$\eta_2^{\max}$	$\eta_4^{\max}$	τ
0.5	0.5	3.0	4.1	4.1	44.5	2.295

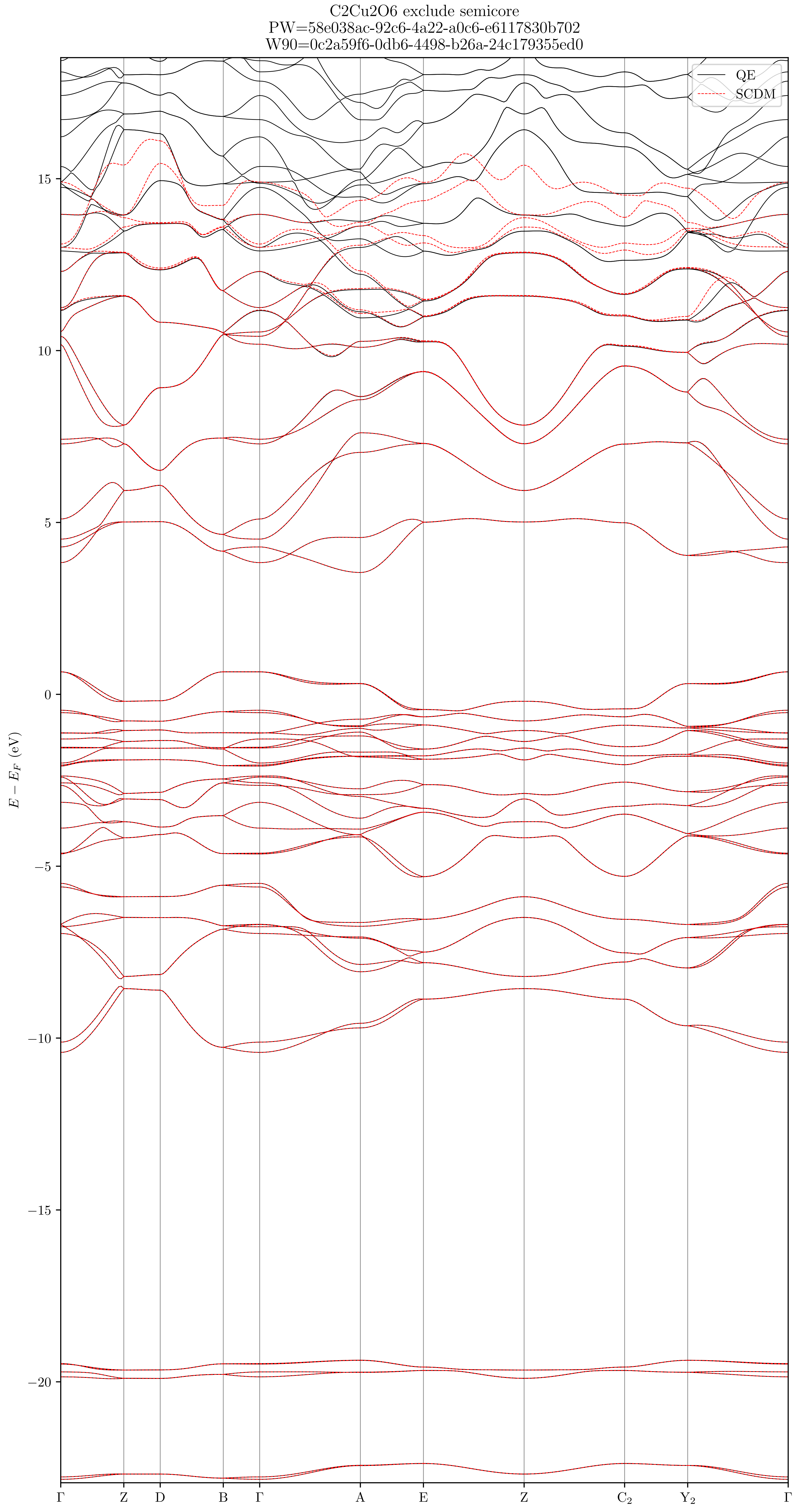

SCDM band distance (meV) and τ ($\mathring{A}$)

η_0	η_2	η_4	$\eta_0^{\max}$	$\eta_2^{\max}$	$\eta_4^{\max}$	τ
0.5	0.5	0.5	3.3	3.3	3.7	2.315

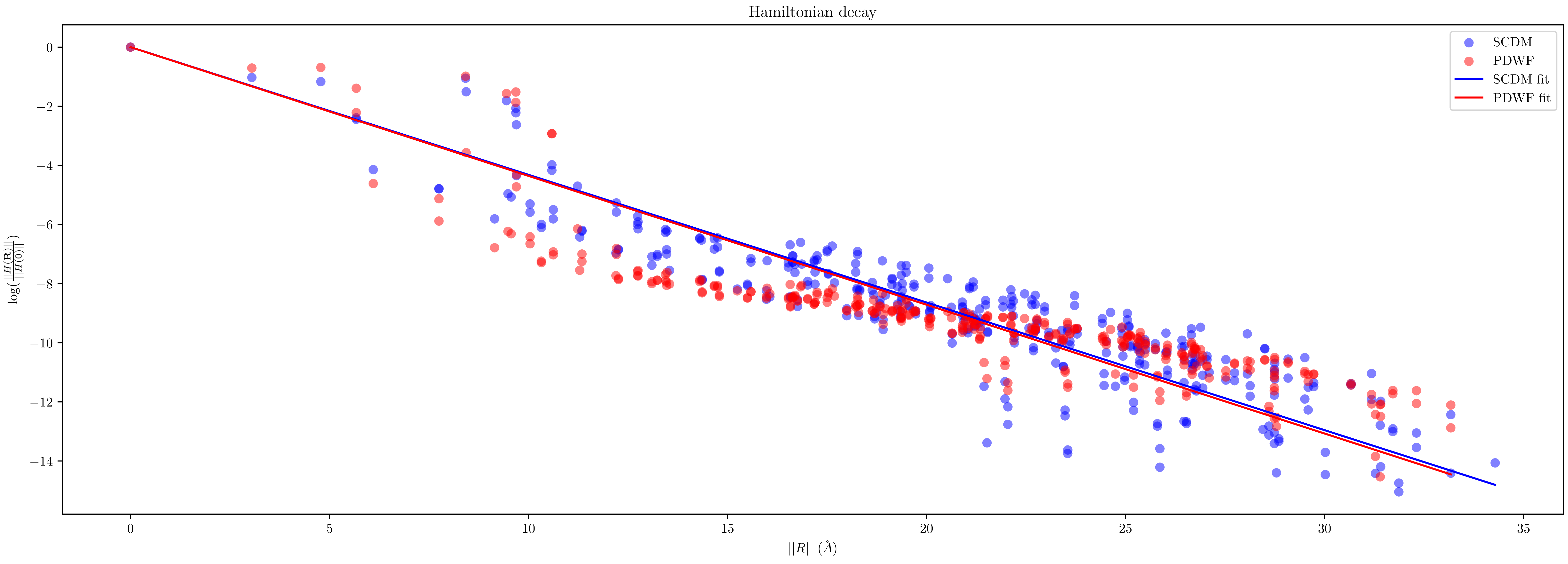

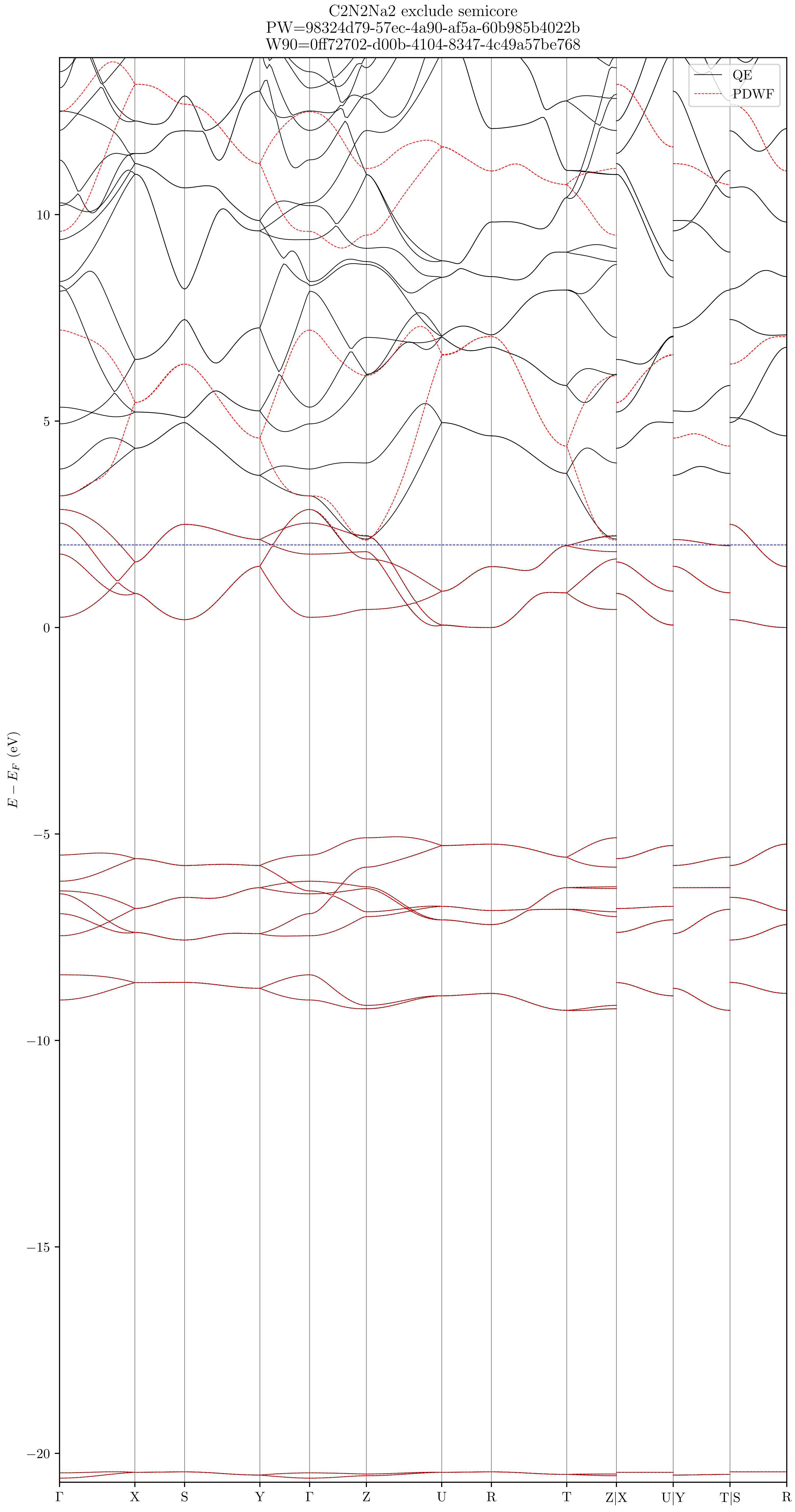

PDWF band distance (meV) and τ ($\AA$)

η_0	η_2	η_4	$\eta_0^{\max}$	$\eta_2^{\max}$	$\eta_4^{\max}$	τ
0.9	0.9	1.2	5.2	5.2	9.6	2.347

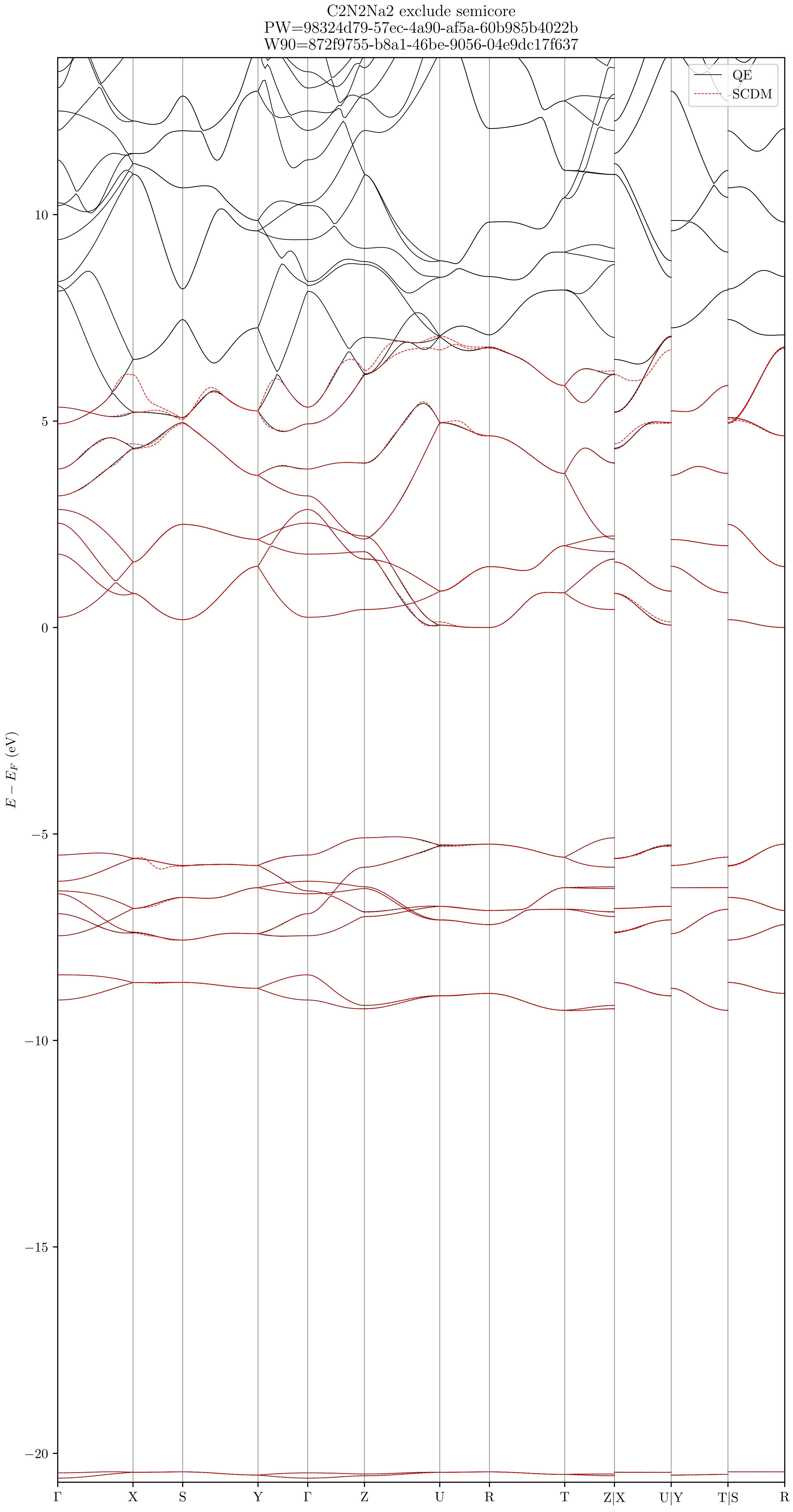

SCDM band distance (meV) and τ ($\AA$)

η_0	η_2	η_4	$\eta_0^{\max}$	$\eta_2^{\max}$	$\eta_4^{\max}$	τ
8.3	8.3	9.9	167.7	167.7	167.7	2.783

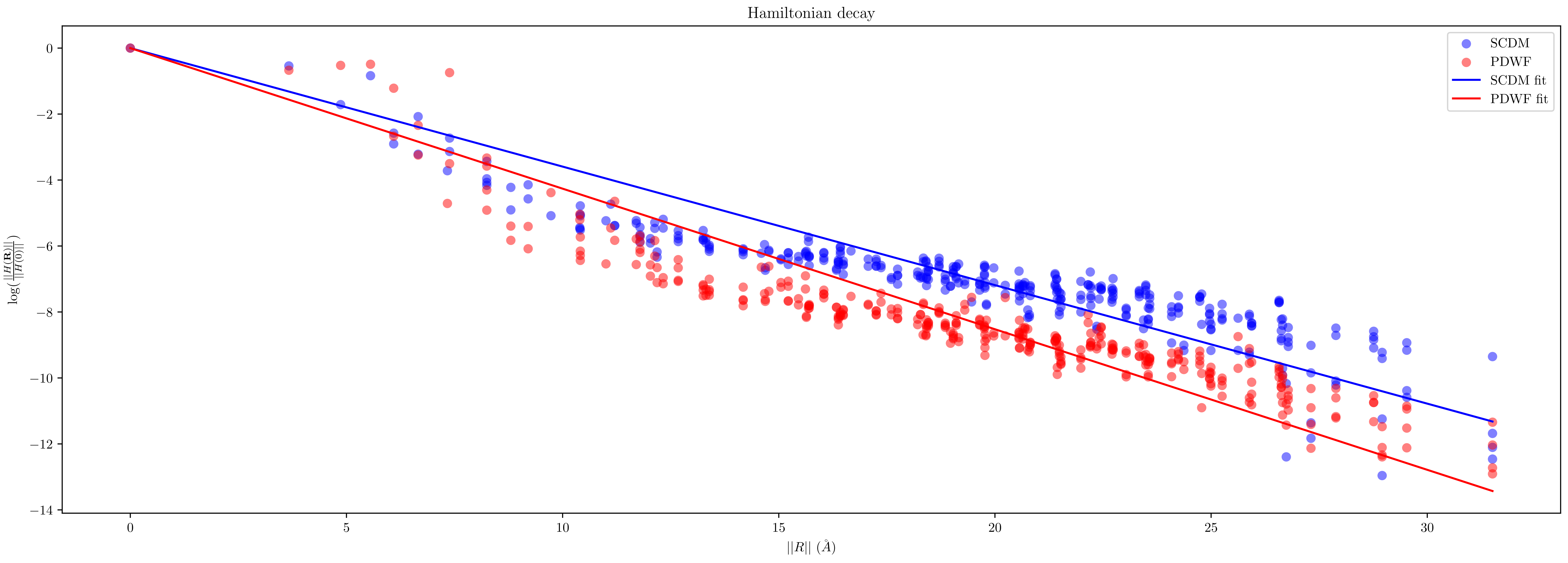

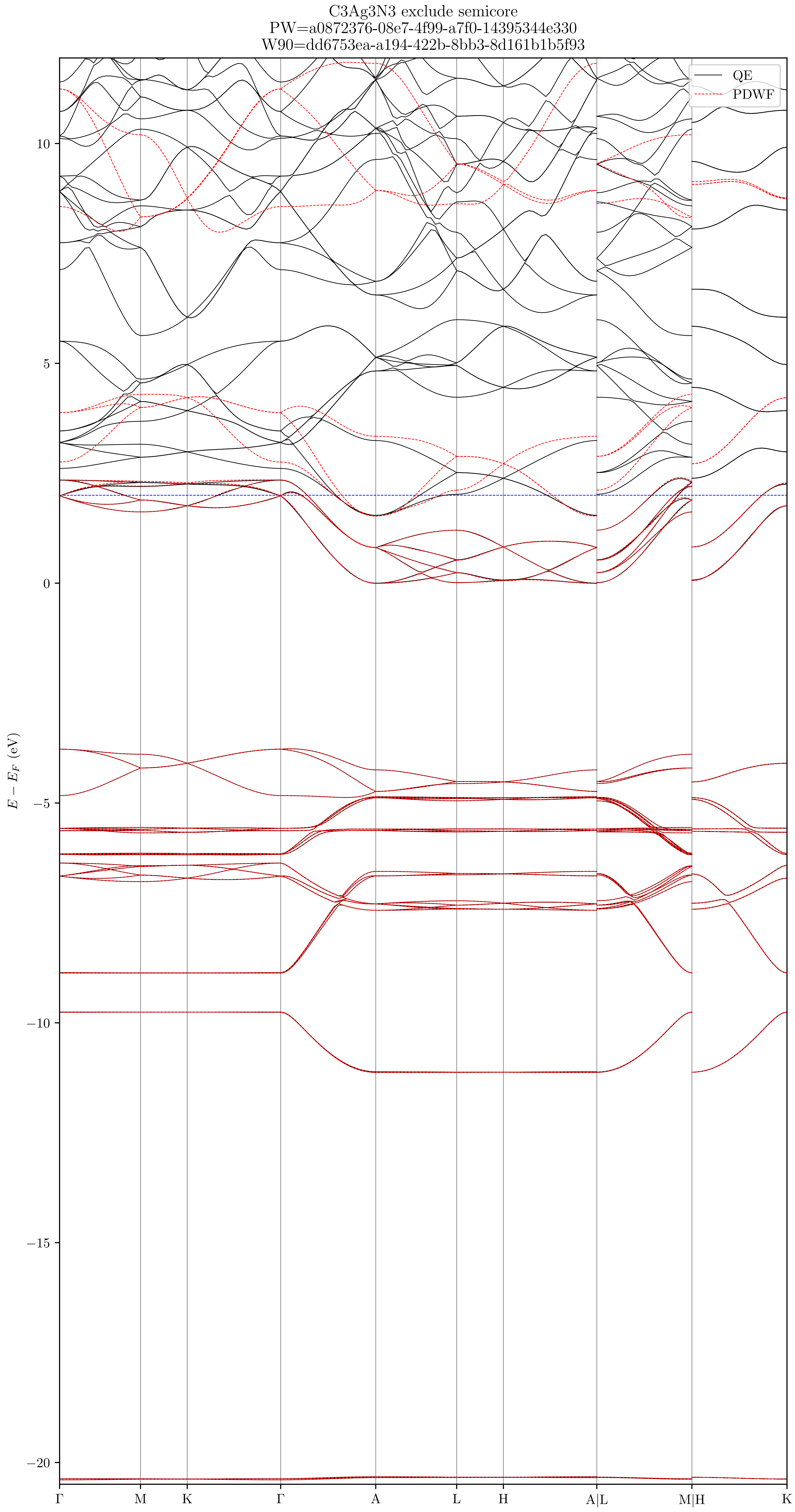

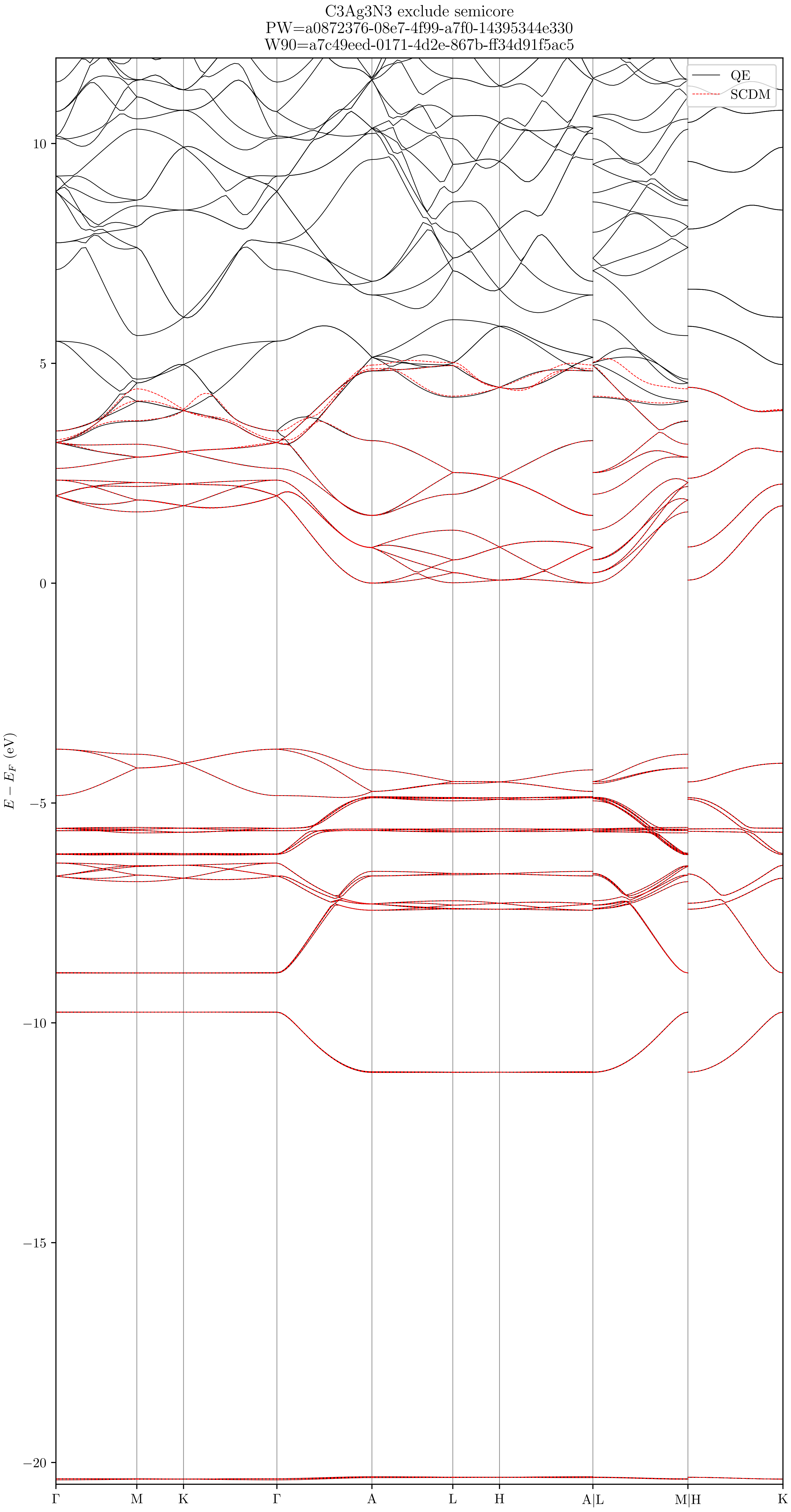

PDWF band distance (meV) and τ ($\AA$)

η_0	η_2	η_4	$\eta_0^{\max}$	$\eta_2^{\max}$	$\eta_4^{\max}$	τ
0.9	1.7	16.0	5.2	12.6	157.9	2.839

SCDM band distance (meV) and τ ($\AA$)

η_0	η_2	η_4	$\eta_0^{\max}$	$\eta_2^{\max}$	$\eta_4^{\max}$	τ
0.7	0.8	1.4	4.2	6.1	9.2	2.940

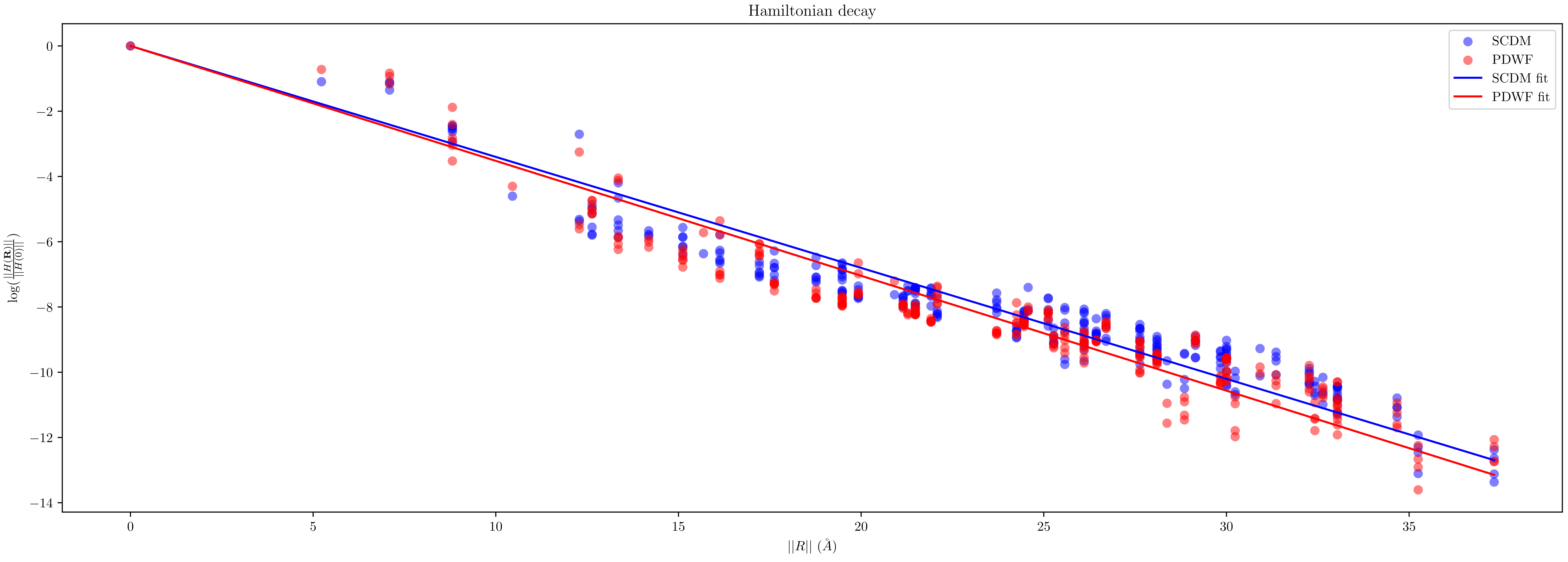

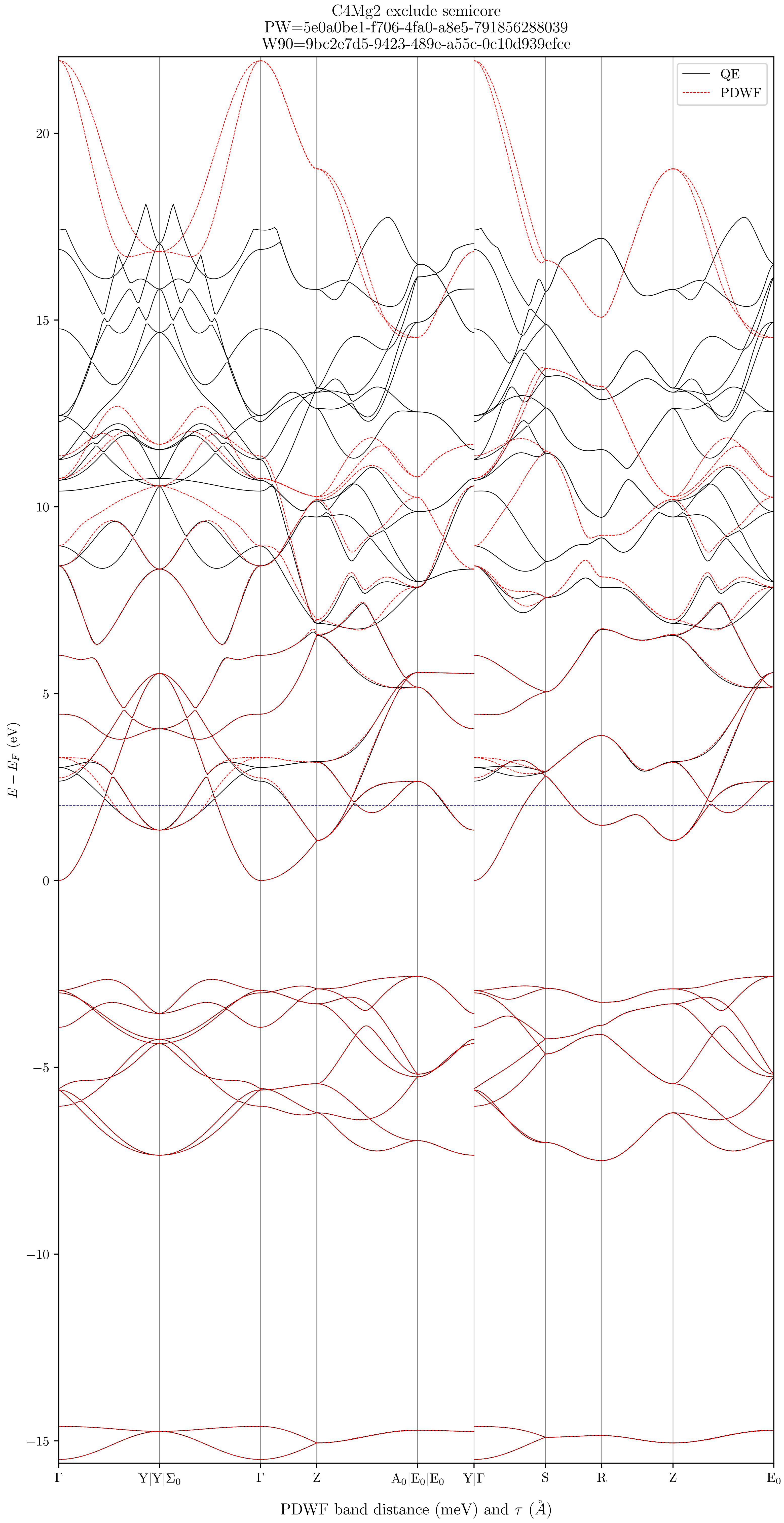

η_0	η_2	η_4	$\eta_0^{\max}$	$\eta_2^{\max}$	$\eta_4^{\max}$	τ
1.7	2.0	42.0	10.7	11.4	308.6	2.314

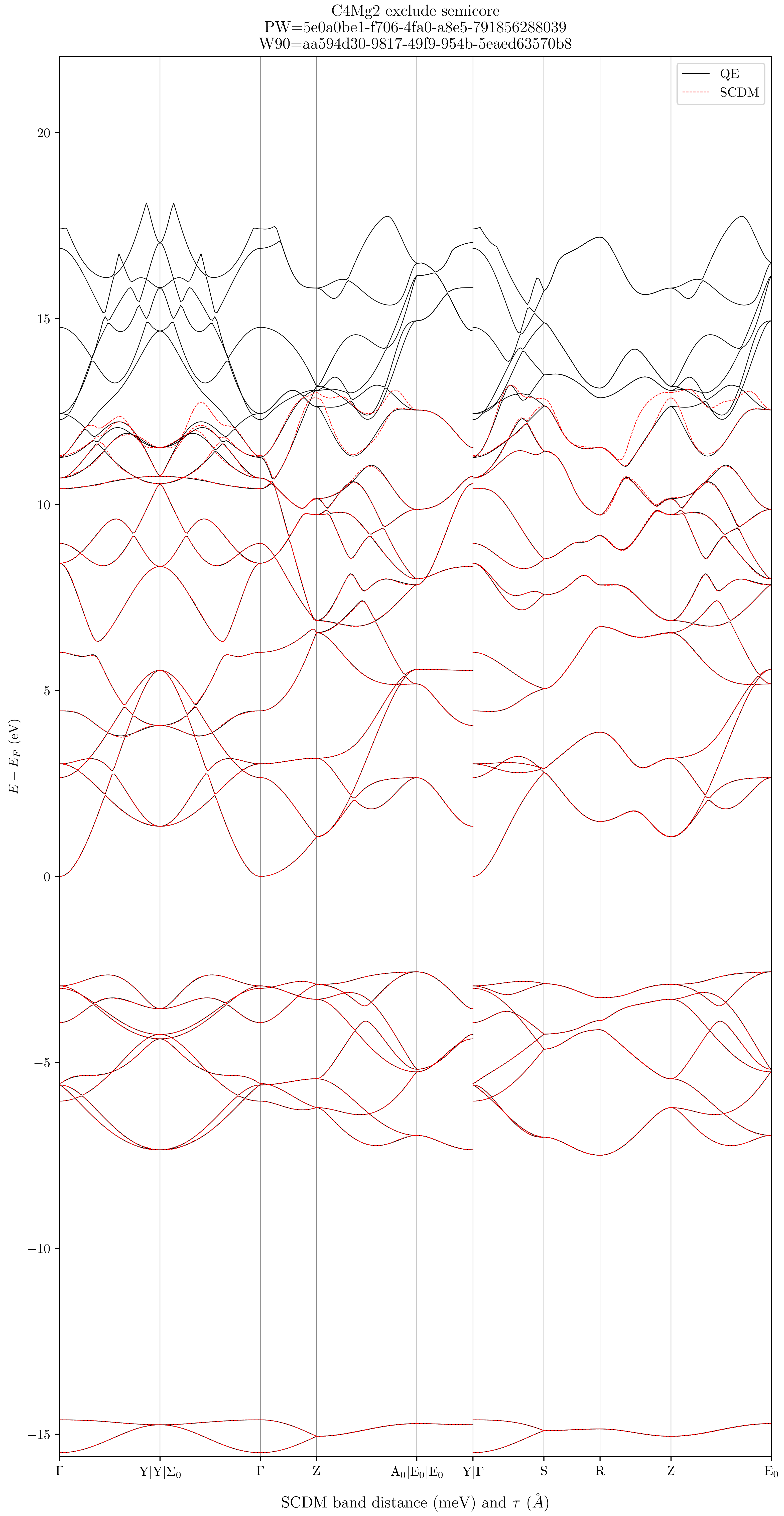

η_0	η_2	η_4	$\eta_0^{\max}$	$\eta_2^{\max}$	$\eta_4^{\max}$	τ
2.9	3.0	3.1	16.1	16.1	16.1	2.517

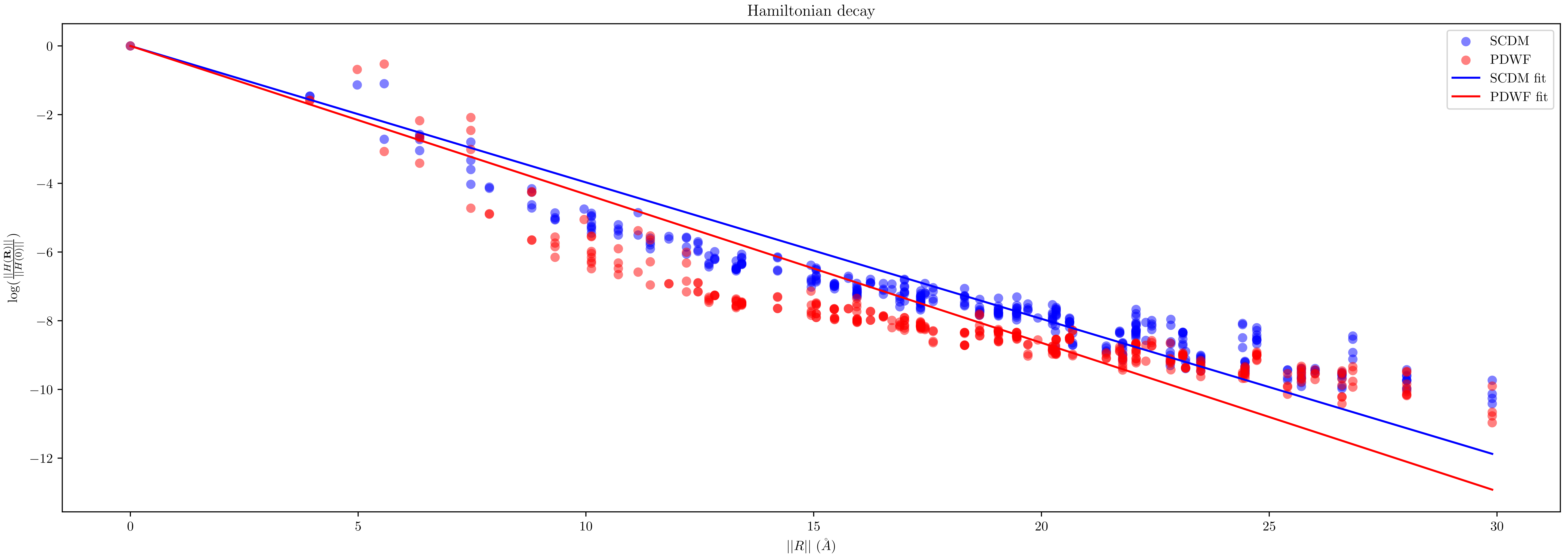

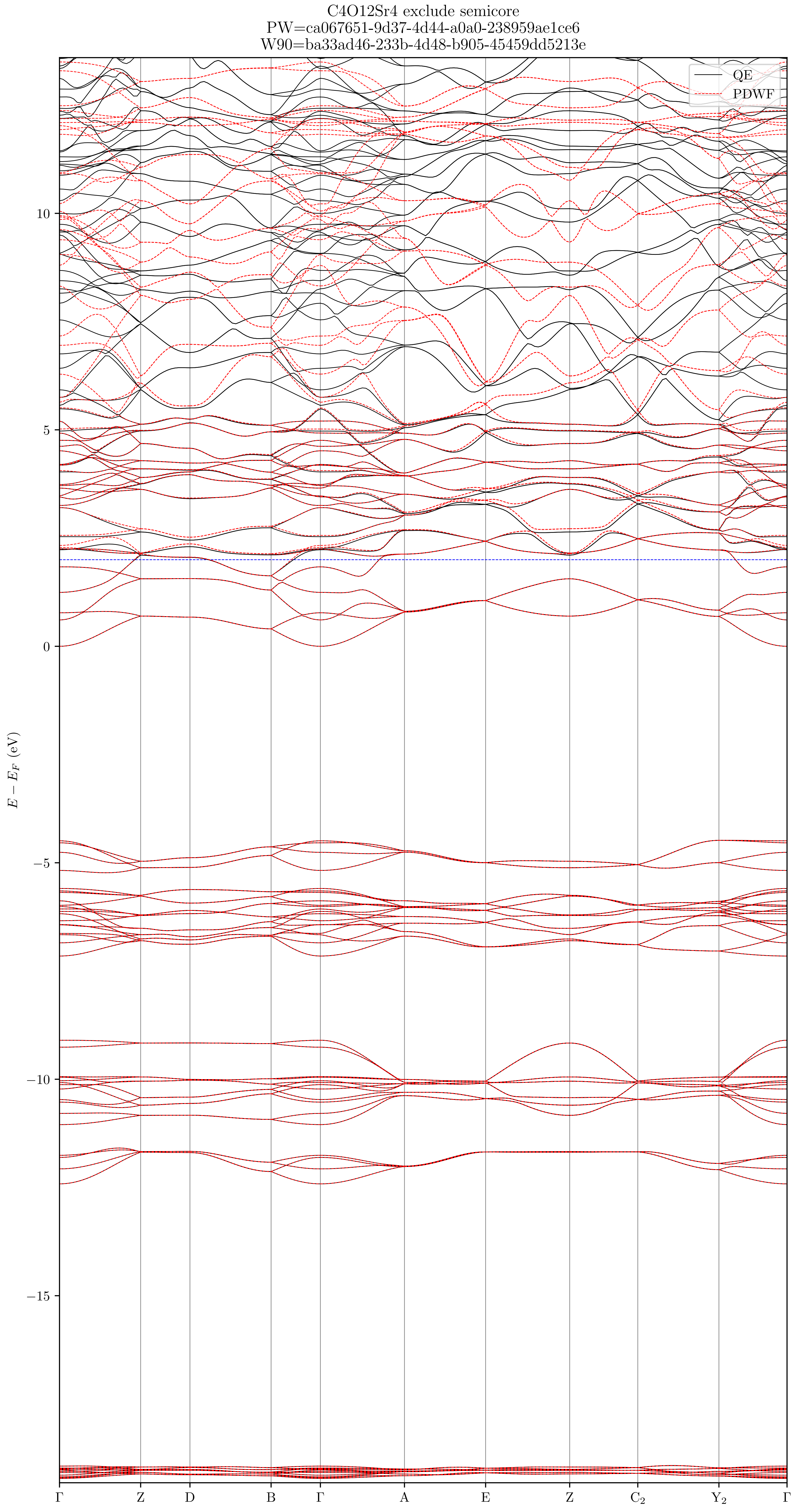

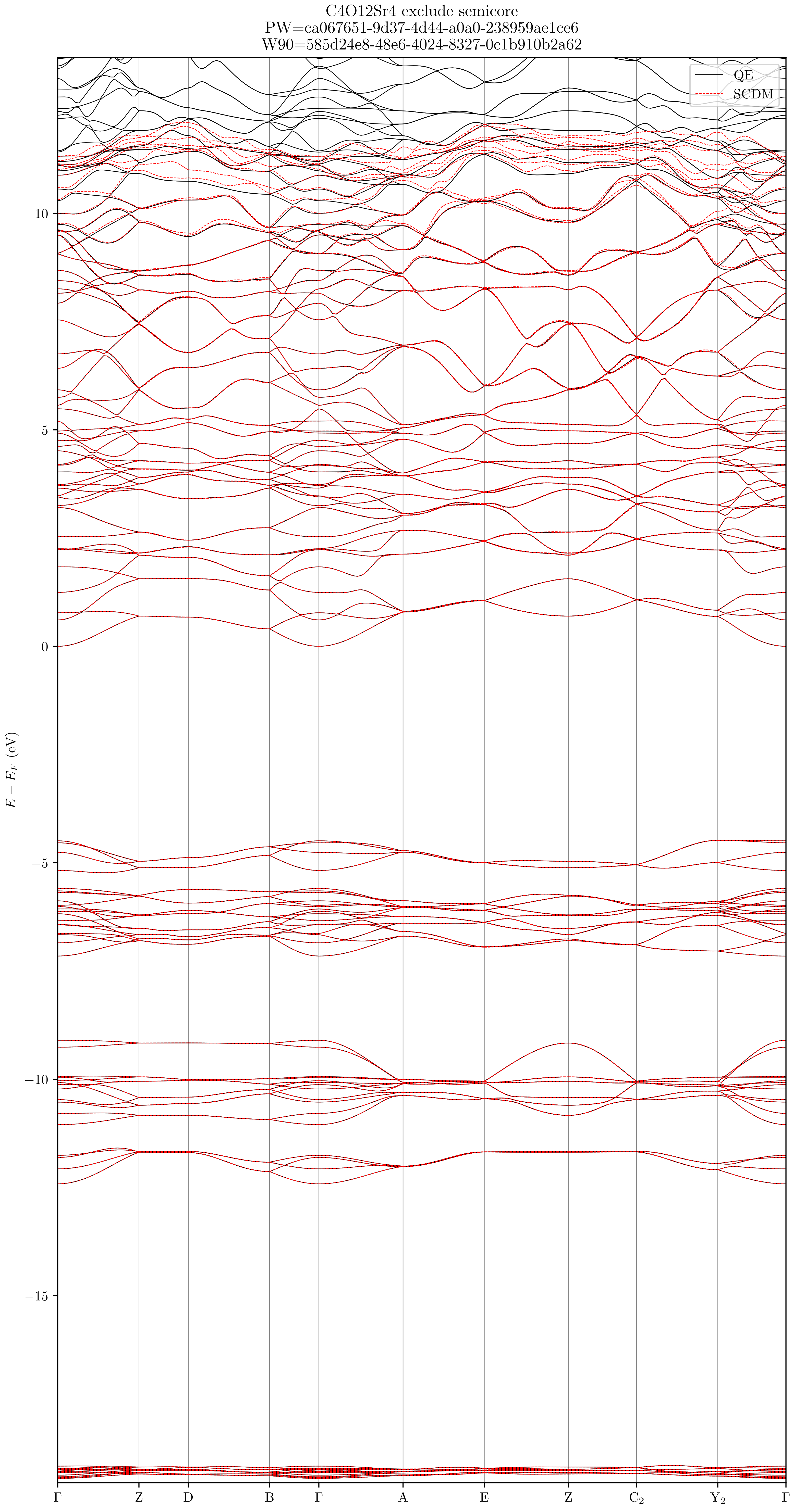

PDWF band distance (meV) and τ ($\AA$)

η_0	η_2	η_4	$\eta_0^{\max}$	$\eta_2^{\max}$	$\eta_4^{\max}$	τ
0.3	0.3	0.3	1.9	1.9	1.9	2.481

SCDM band distance (meV) and τ ($\AA$)

η_0	η_2	η_4	$\eta_0^{\max}$	$\eta_2^{\max}$	$\eta_4^{\max}$	τ
1.2	1.2	1.2	7.9	7.9	7.9	3.182

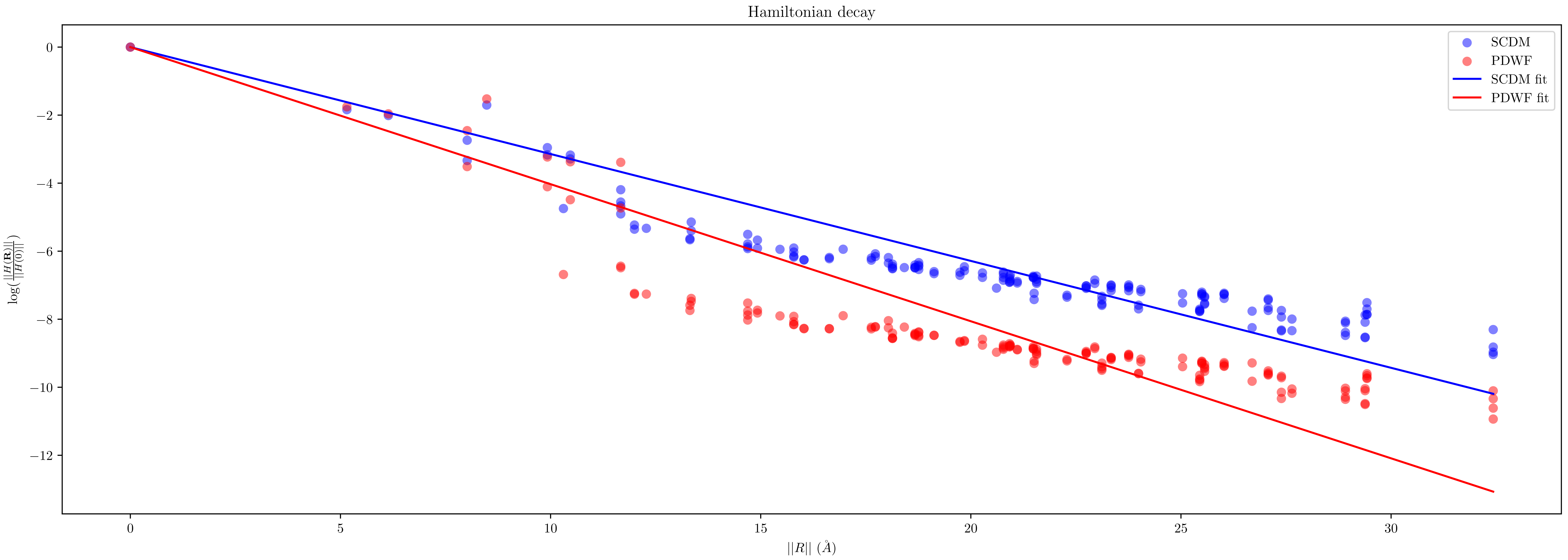

C6Mg4 exclude semicore
PW=5bfd3dcb-c54d-4110-bbbb-76a35bfa9516
W90=7b182dfb-4eff-43af-b151-ba1e22211e5d

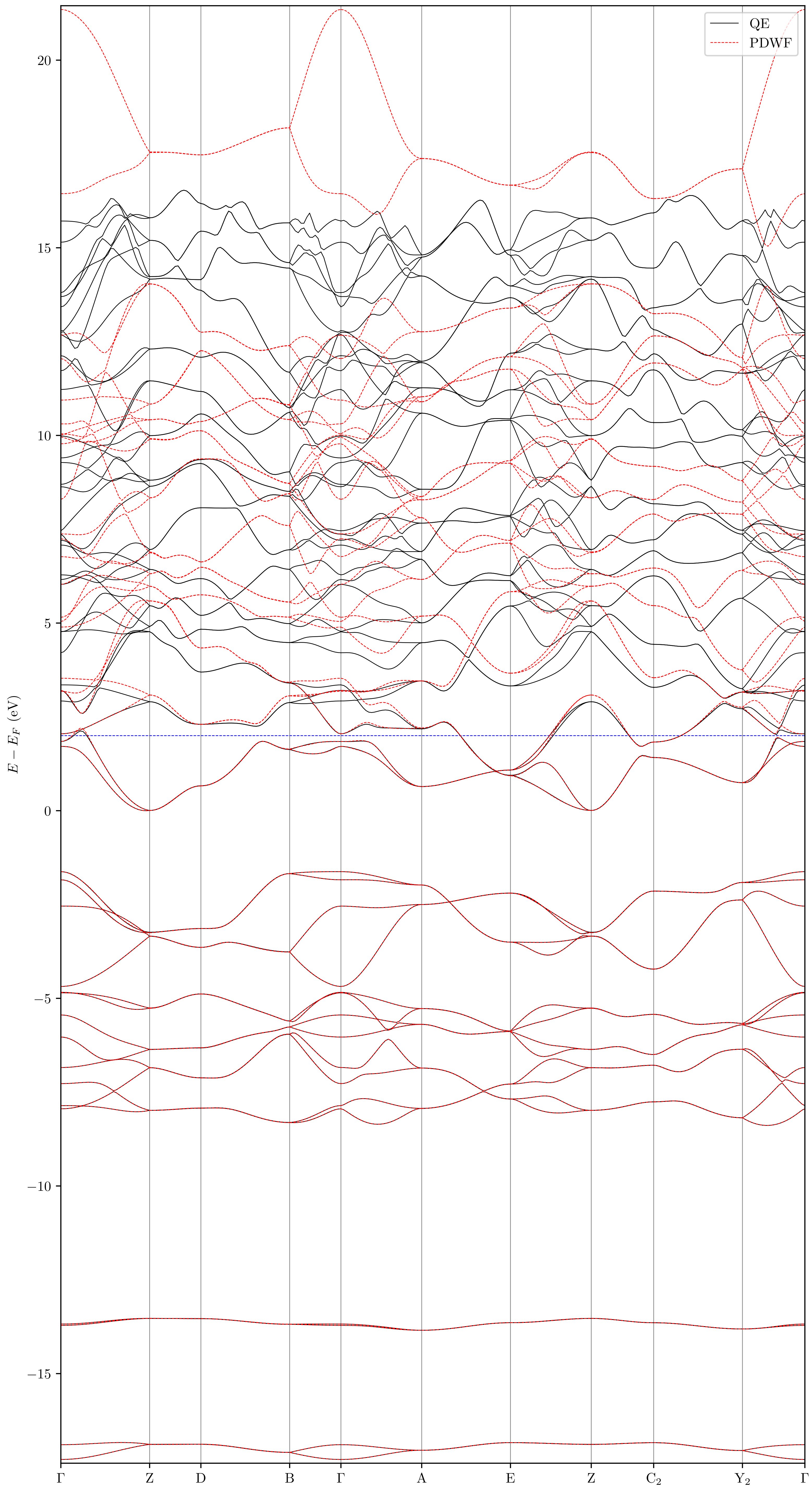

PDWF band distance (meV) and τ ($\AA$)

η_0	η_2	η_4	$\eta_0^{\max}$	$\eta_2^{\max}$	$\eta_4^{\max}$	τ
1.8	2.0	33.9	12.2	12.2	295.4	2.416

C6Mg4 exclude semicore
PW=5bfd3dcb-c54d-4110-bbbb-76a35bfa9516
W90=aadc0a0c-bbca-43b3-8835-19d1f7e17d51

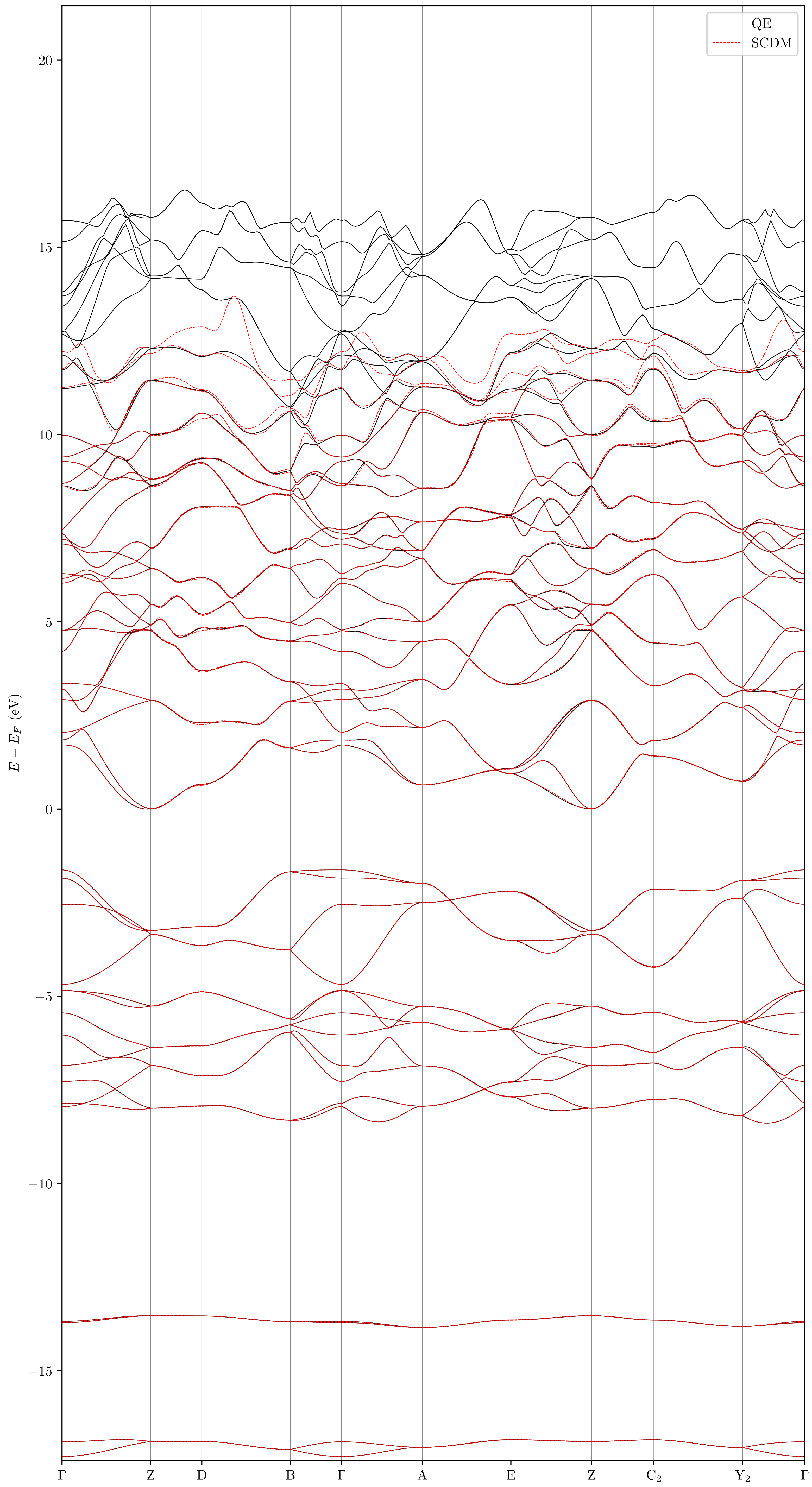

SCDM band distance (meV) and τ ($\AA$)

η_0	η_2	η_4	$\eta_0^{\max}$	$\eta_2^{\max}$	$\eta_4^{\max}$	τ
2.8	3.5	4.4	15.8	34.6	53.1	2.824

Hamiltonian decay

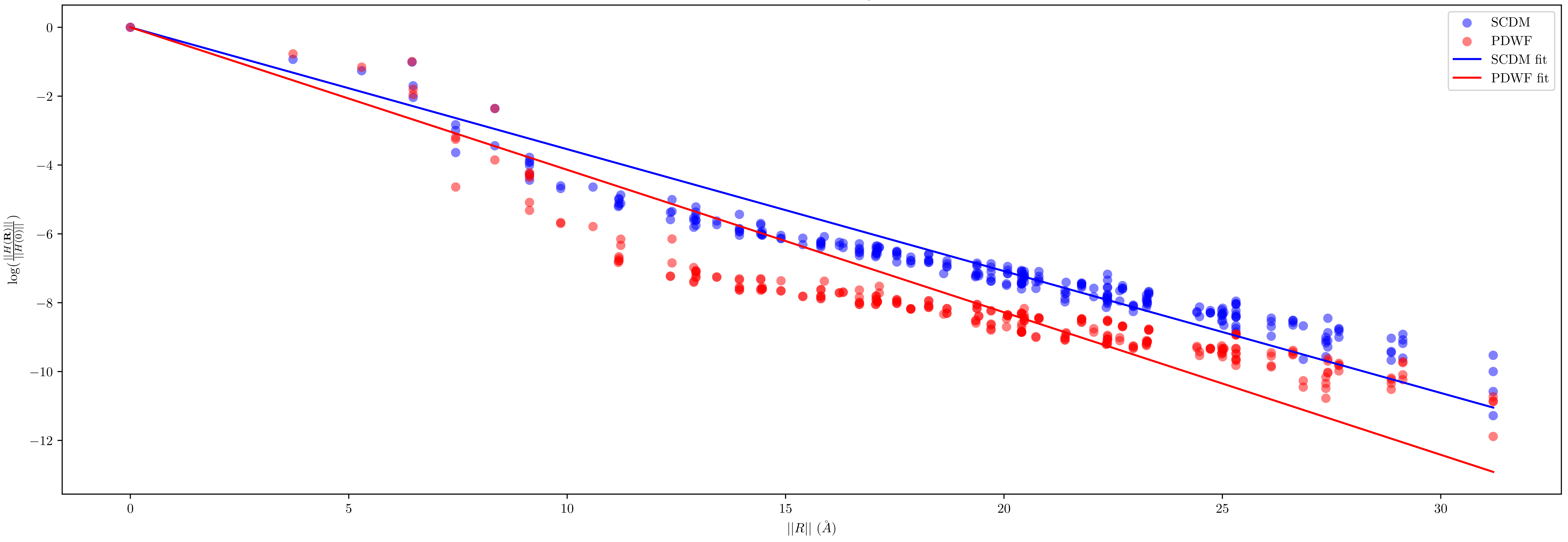

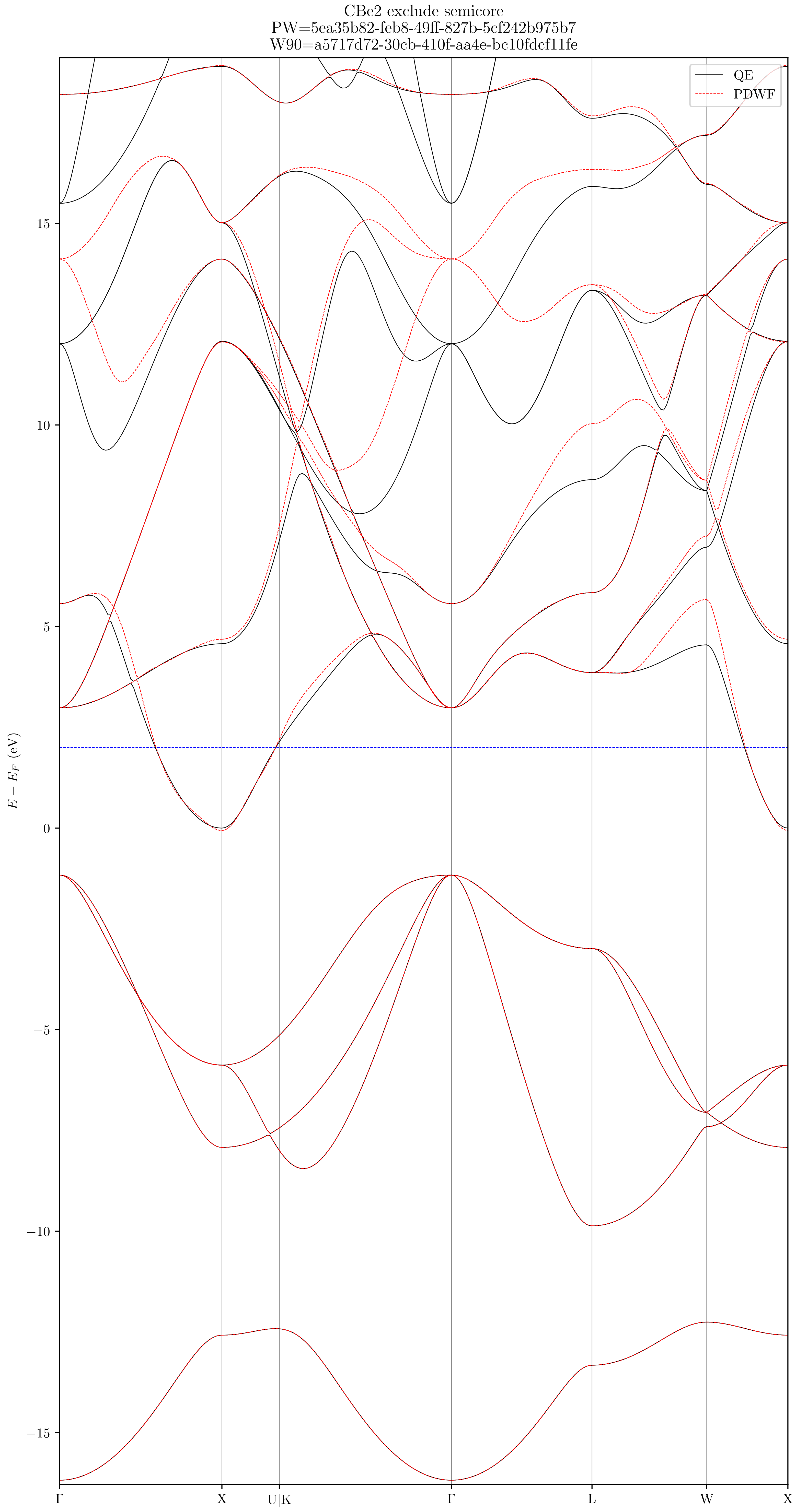

PDWF band distance (meV) and τ ($\AA$)

η_0	η_2	η_4	$\eta_0^{\max}$	$\eta_2^{\max}$	$\eta_4^{\max}$	τ
1.7	7.8	37.5	6.3	60.1	346.2	2.051

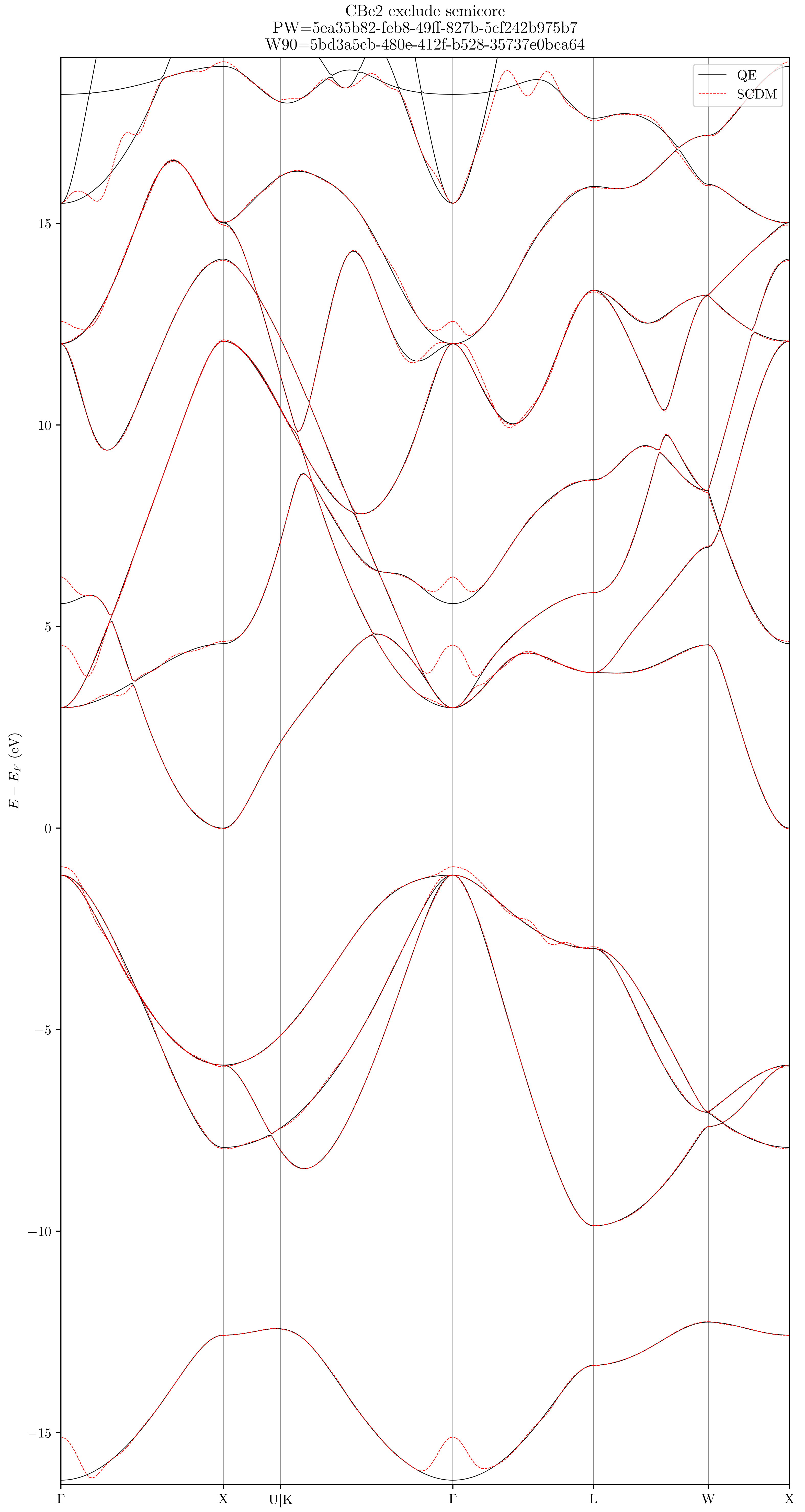

SCDM band distance (meV) and τ ($\AA$)

η_0	η_2	η_4	$\eta_0^{\max}$	$\eta_2^{\max}$	$\eta_4^{\max}$	τ
129.2	126.4	122.5	1074.0	1074.0	1074.0	2.532

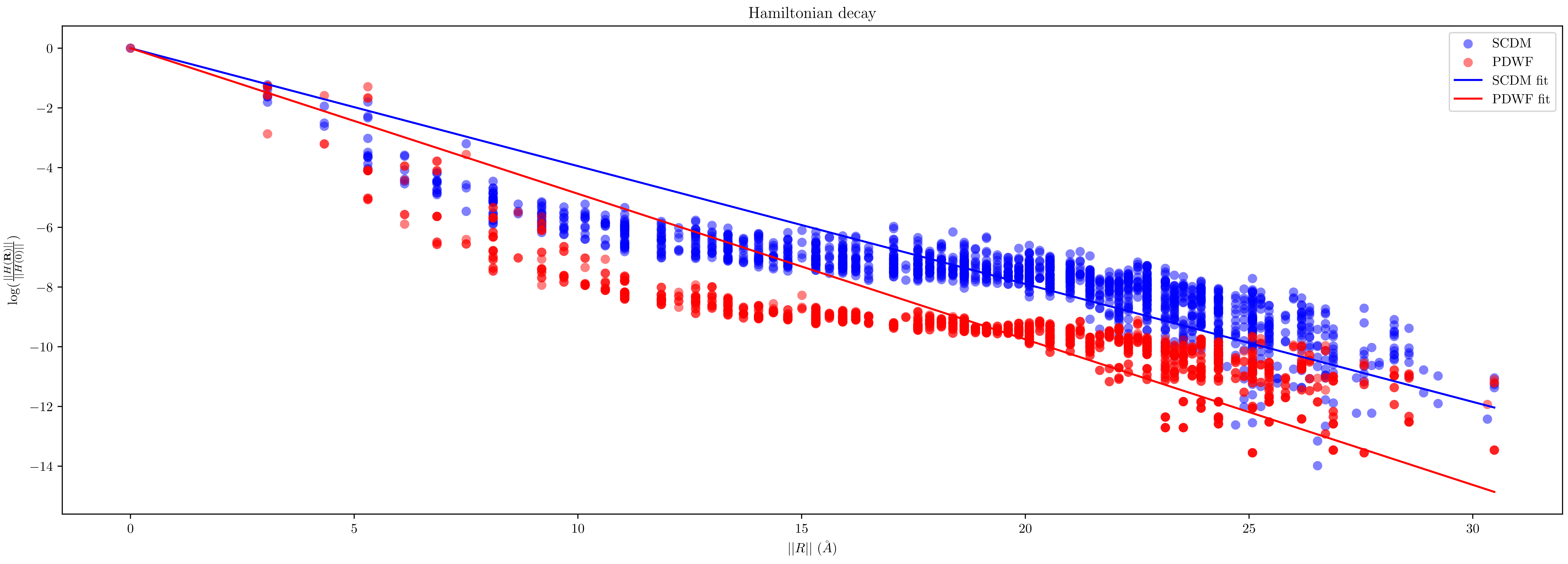

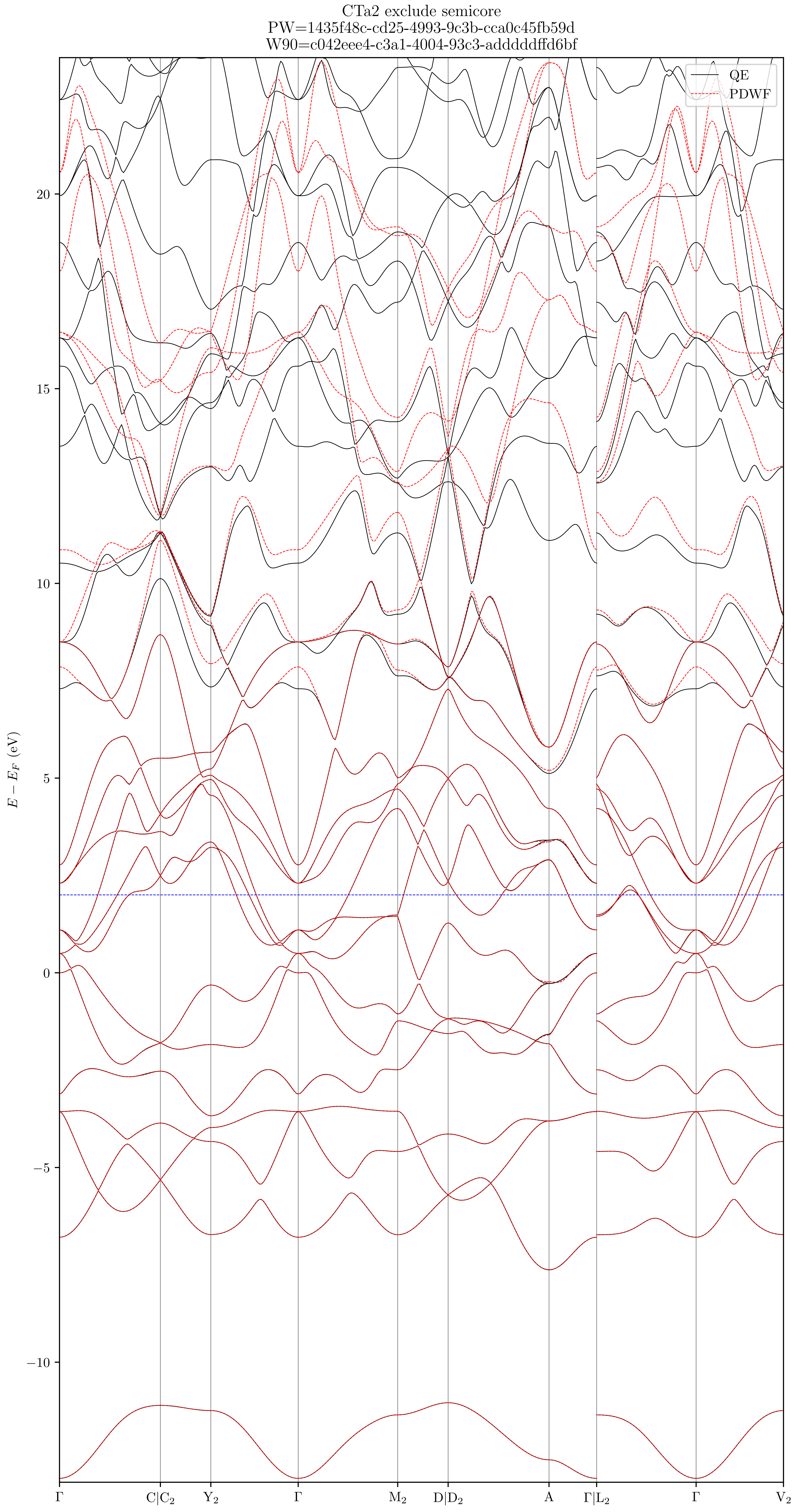

PDWF band distance (meV) and τ ($\AA$)

η_0	η_2	η_4	$\eta_0^{\max}$	$\eta_2^{\max}$	$\eta_4^{\max}$	τ
4.1	4.2	5.0	40.4	43.6	43.6	1.994

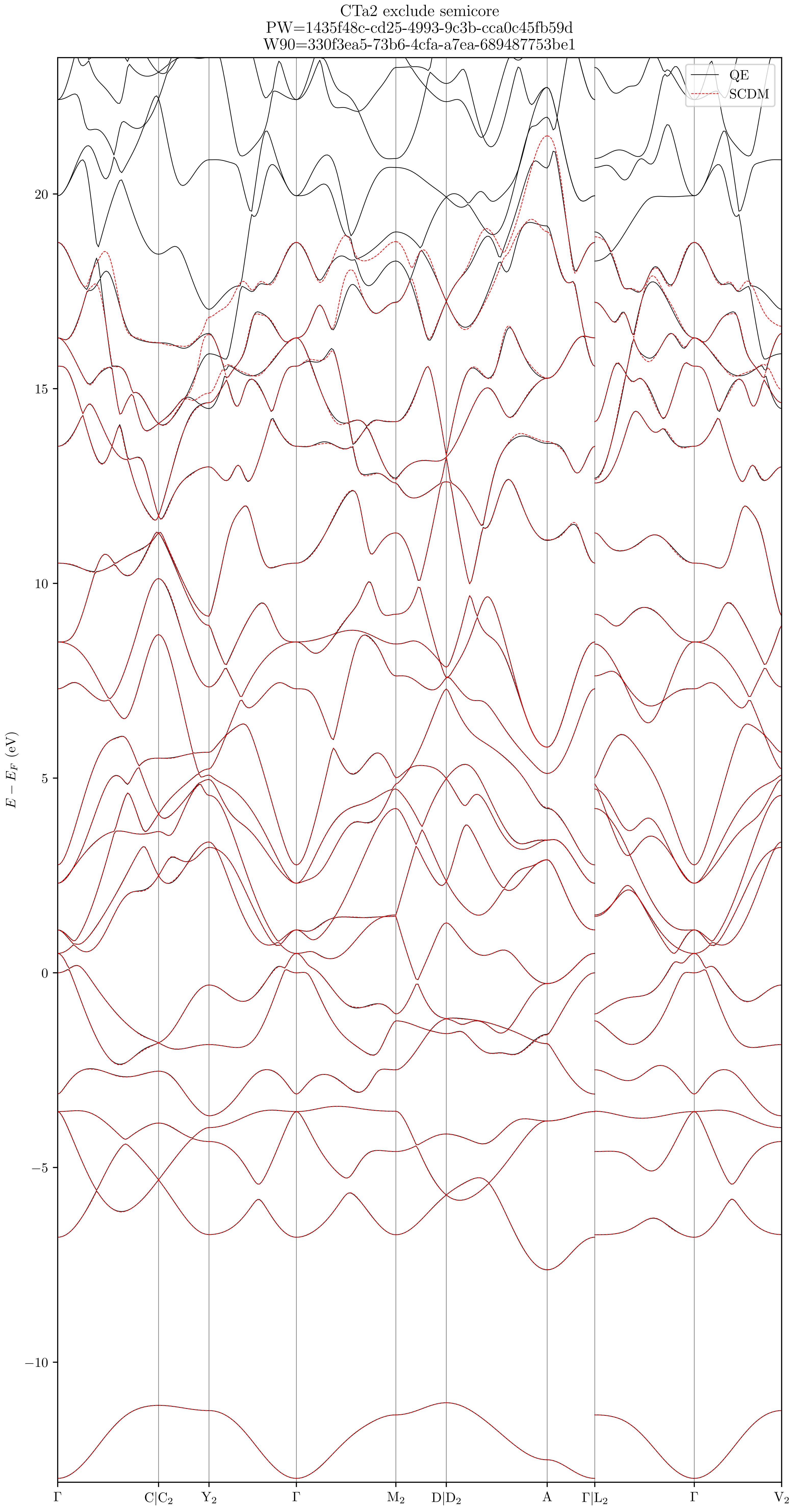

SCDM band distance (meV) and τ ($\AA$)

η_0	η_2	η_4	$\eta_0^{\max}$	$\eta_2^{\max}$	$\eta_4^{\max}$	τ
4.3	4.3	4.4	28.5	28.5	28.5	2.228

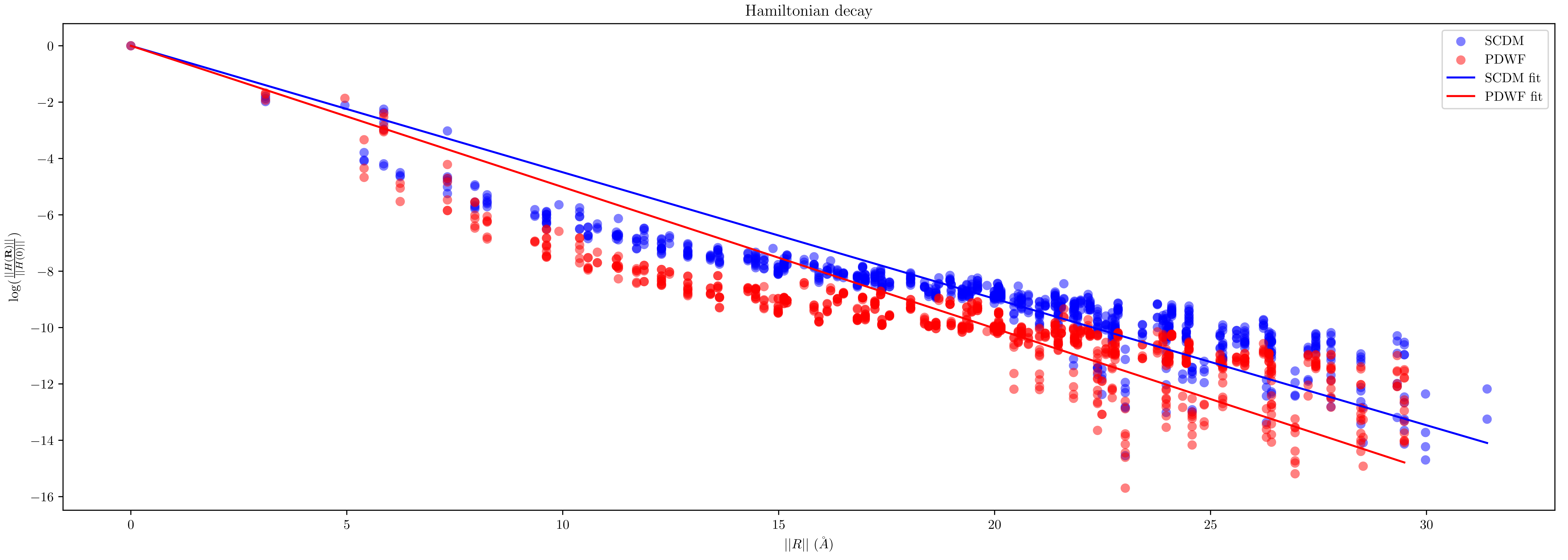

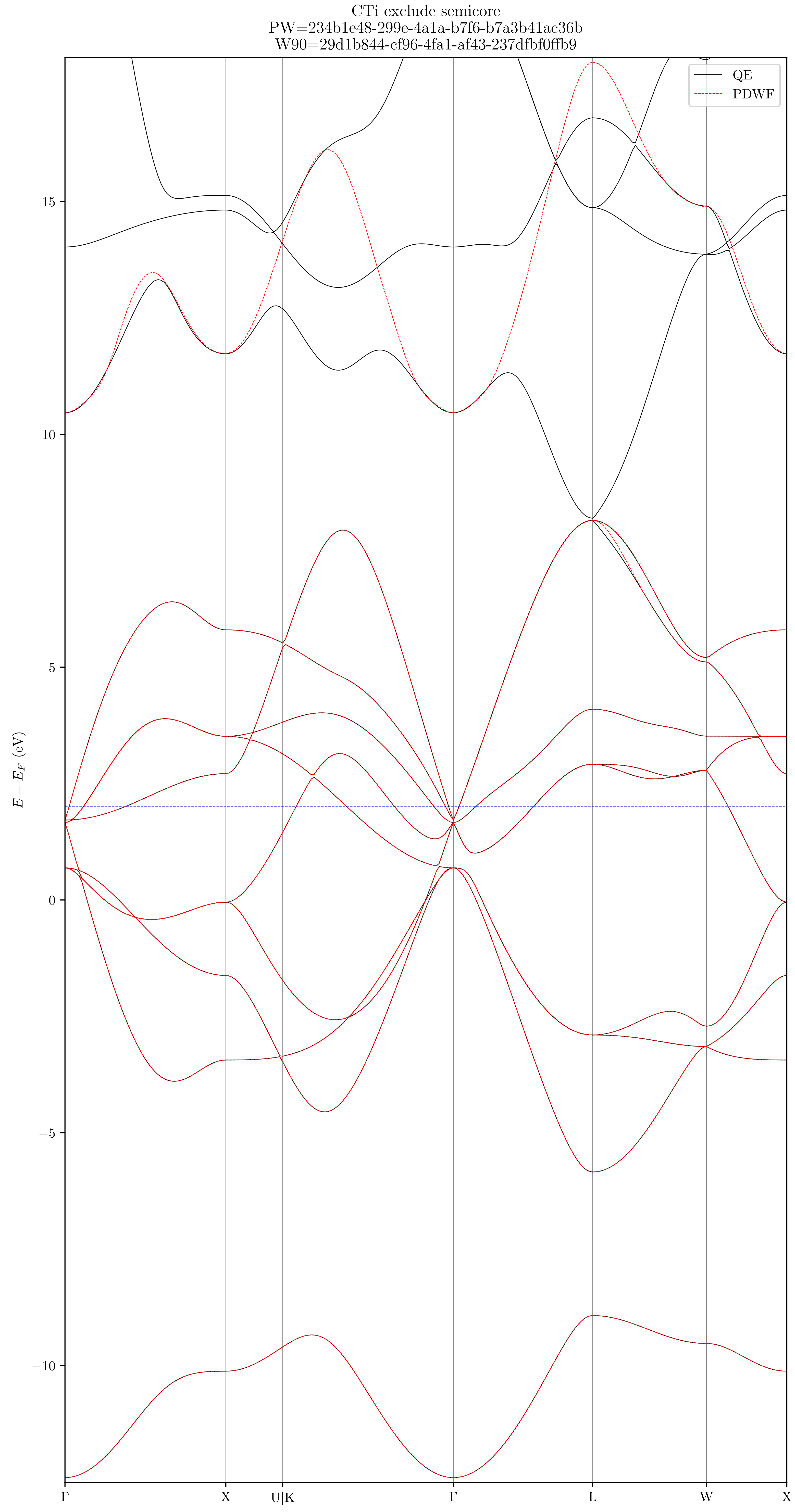

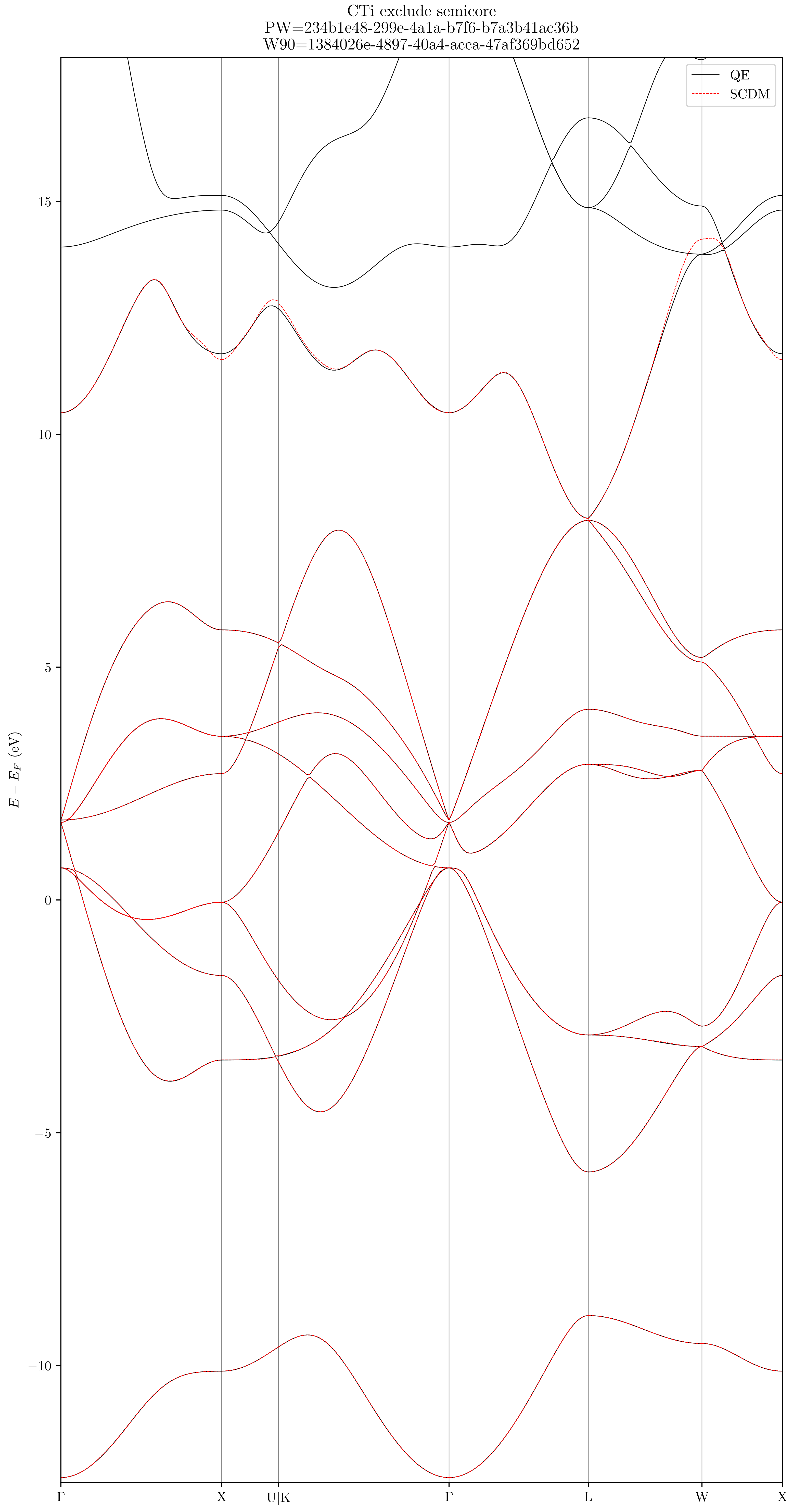

PDWF band distance (meV) and τ ($\AA$)

η_0	η_2	η_4	$\eta_0^{\max}$	$\eta_2^{\max}$	$\eta_4^{\max}$	τ
0.7	0.6	0.7	2.5	2.6	5.1	1.759

SCDM band distance (meV) and τ ($\AA$)

η_0	η_2	η_4	$\eta_0^{\max}$	$\eta_2^{\max}$	$\eta_4^{\max}$	τ
2.3	2.0	1.7	13.0	13.0	13.0	2.043

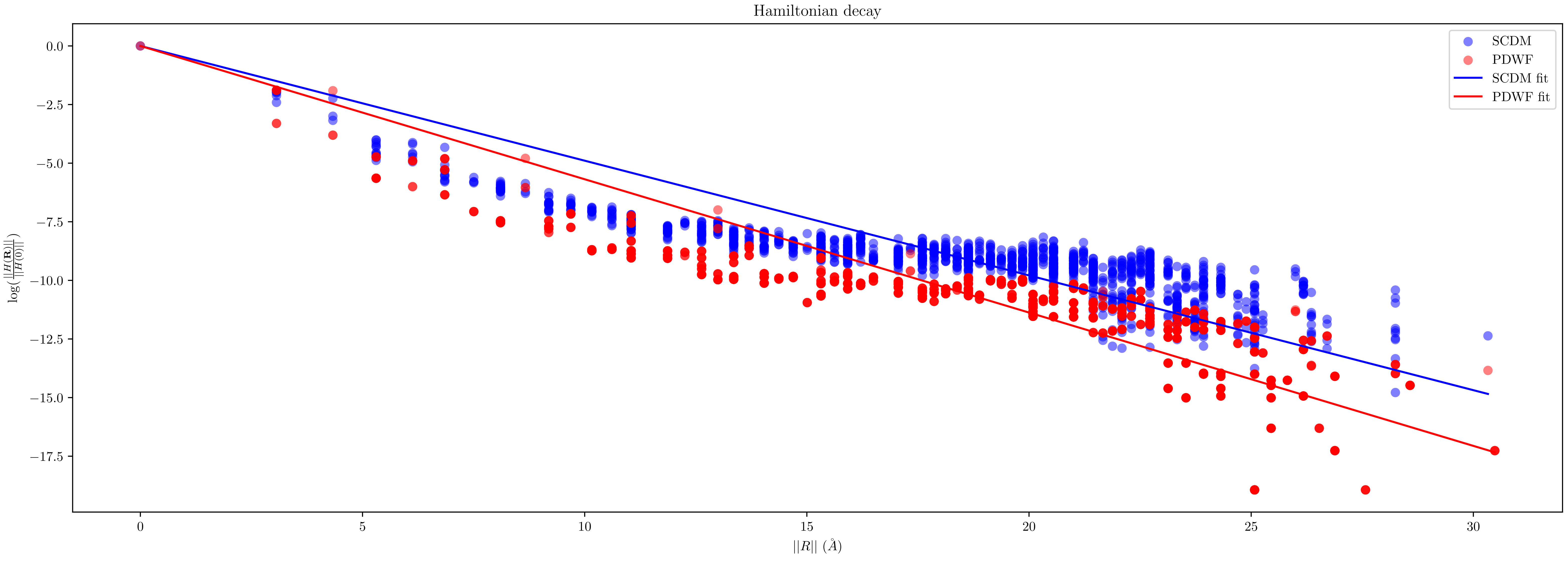

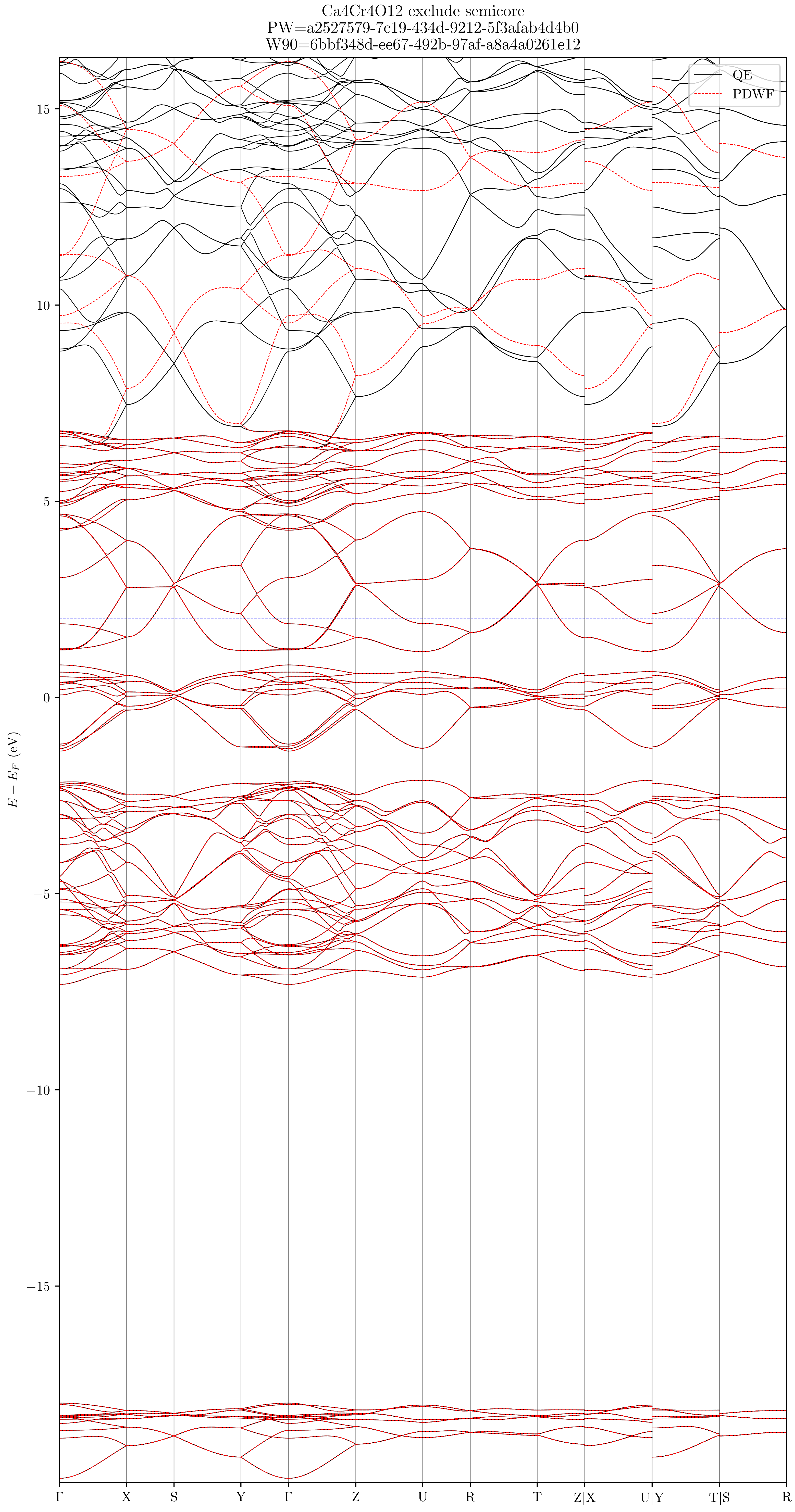

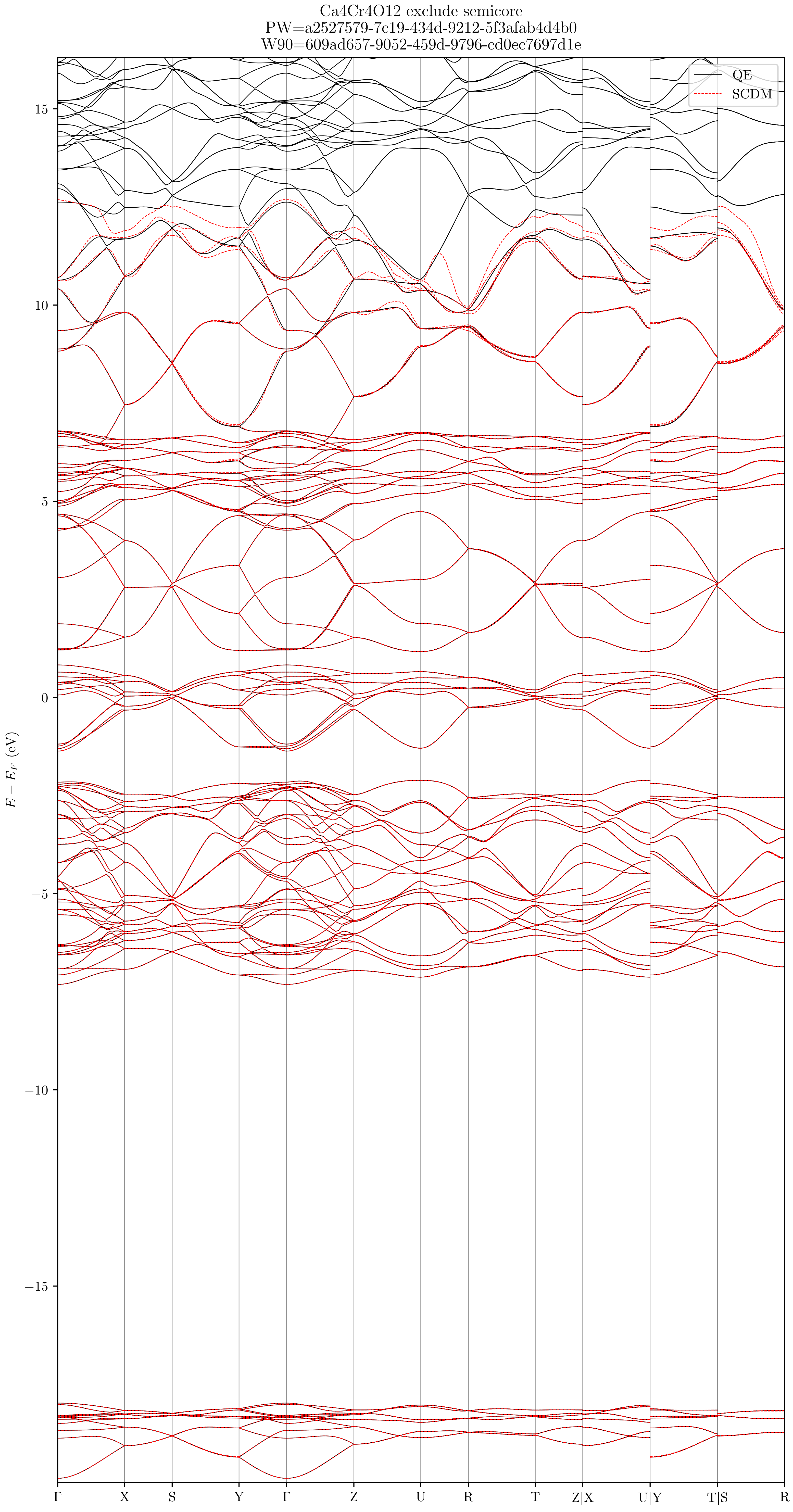

PDWF band distance (meV) and τ ($\AA$)

η_0	η_2	η_4	$\eta_0^{\max}$	$\eta_2^{\max}$	$\eta_4^{\max}$	τ
0.1	0.1	0.1	0.9	0.9	0.9	1.751

SCDM band distance (meV) and τ ($\AA$)

η_0	η_2	η_4	$\eta_0^{\max}$	$\eta_2^{\max}$	$\eta_4^{\max}$	τ
1.9	1.7	1.7	17.8	17.8	17.8	2.253

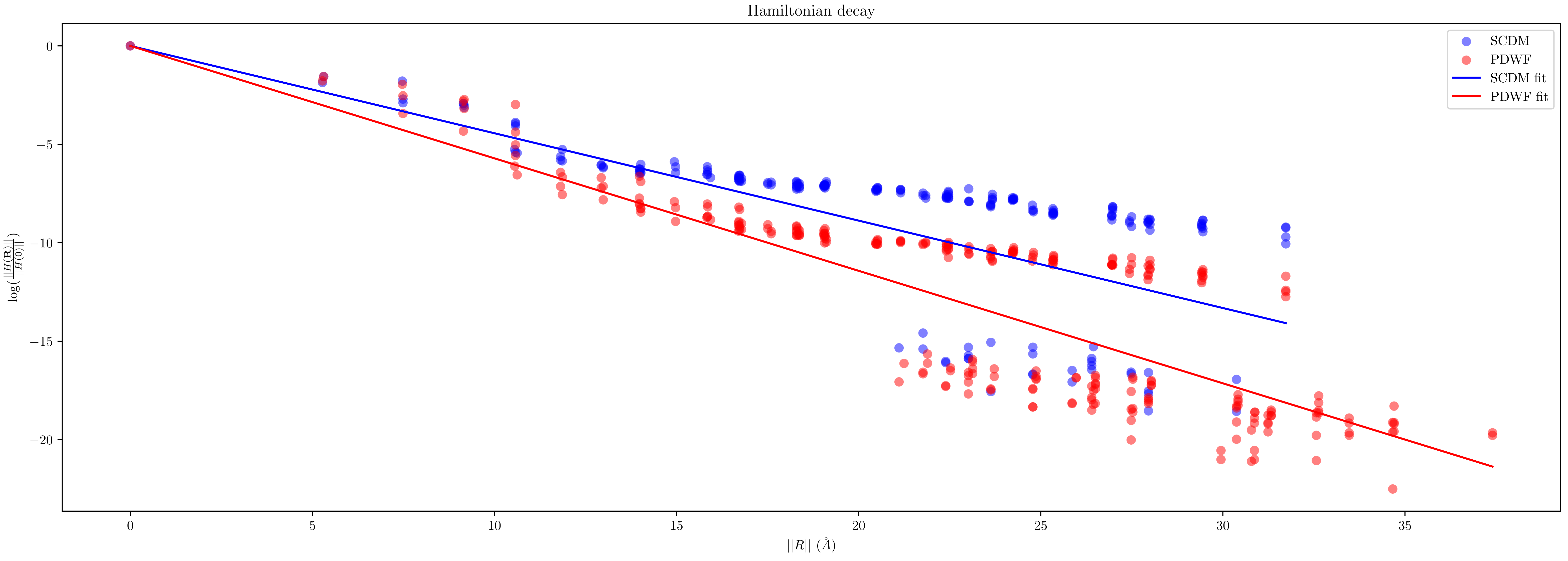

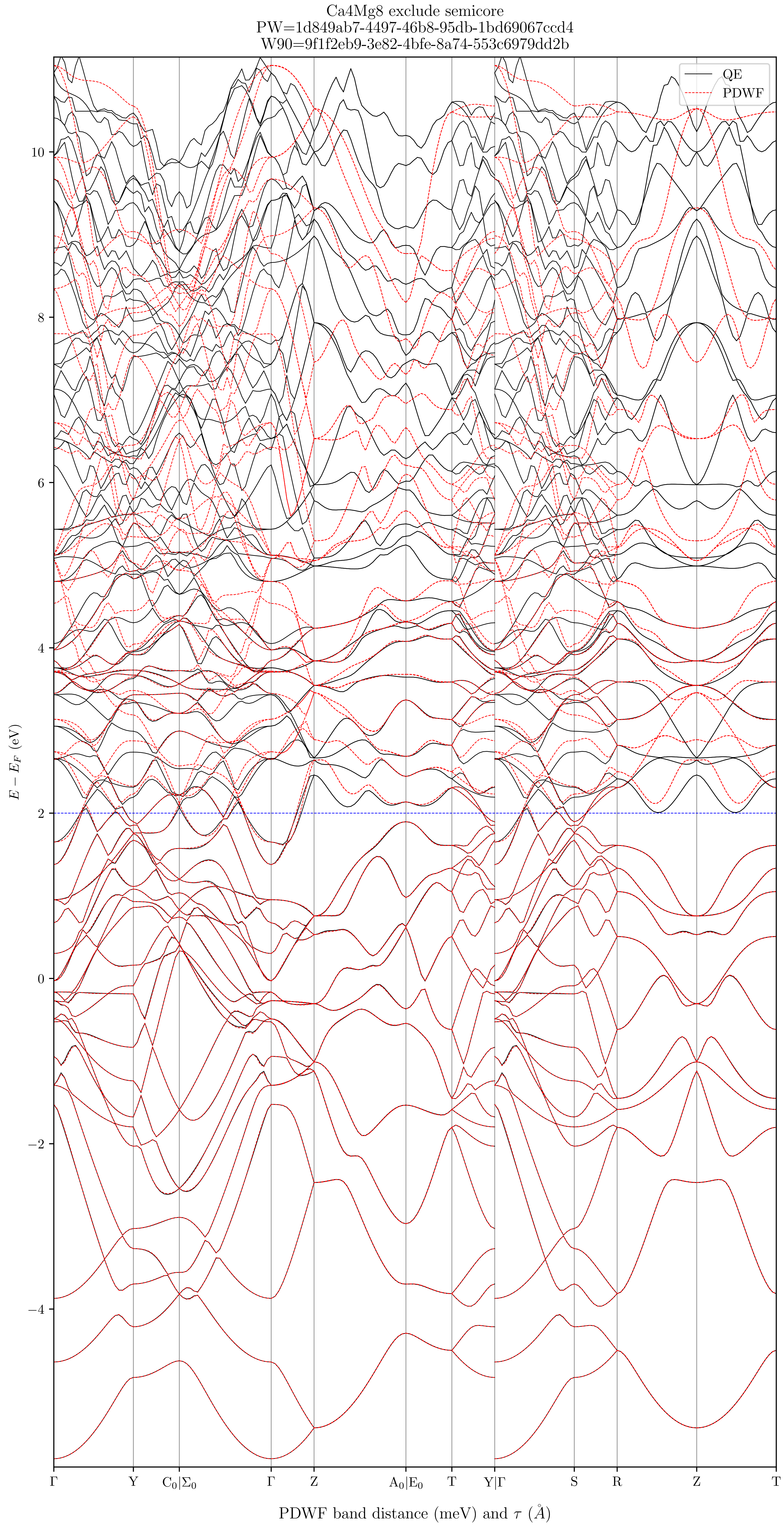

η_0	η_2	η_4	$\eta_0^{\max}$	$\eta_2^{\max}$	$\eta_4^{\max}$	τ
3.0	9.4	102.8	21.1	41.1	789.6	3.225

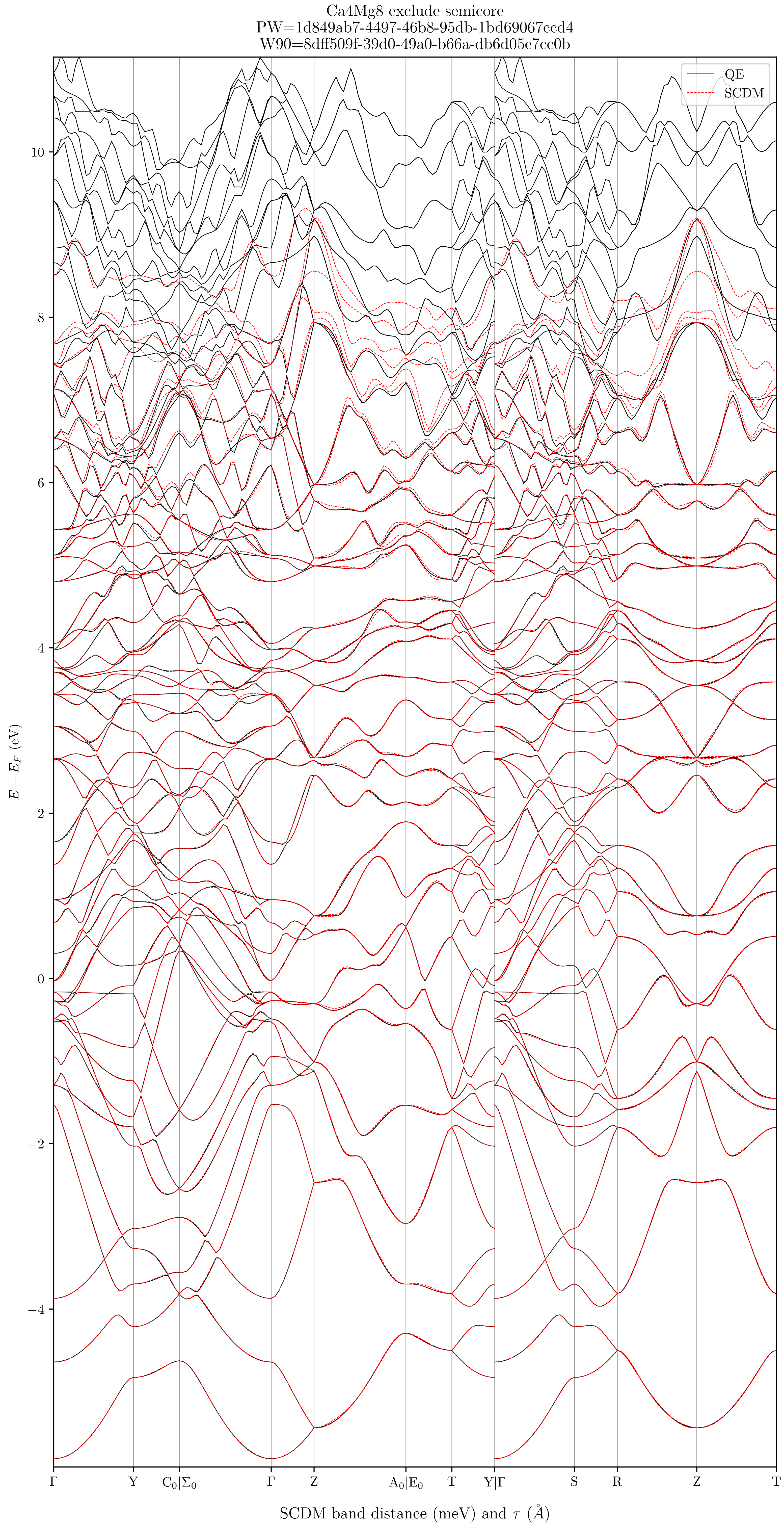

η_0	η_2	η_4	$\eta_0^{\max}$	$\eta_2^{\max}$	$\eta_4^{\max}$	τ
5.6	5.7	6.1	28.0	28.8	39.4	3.597

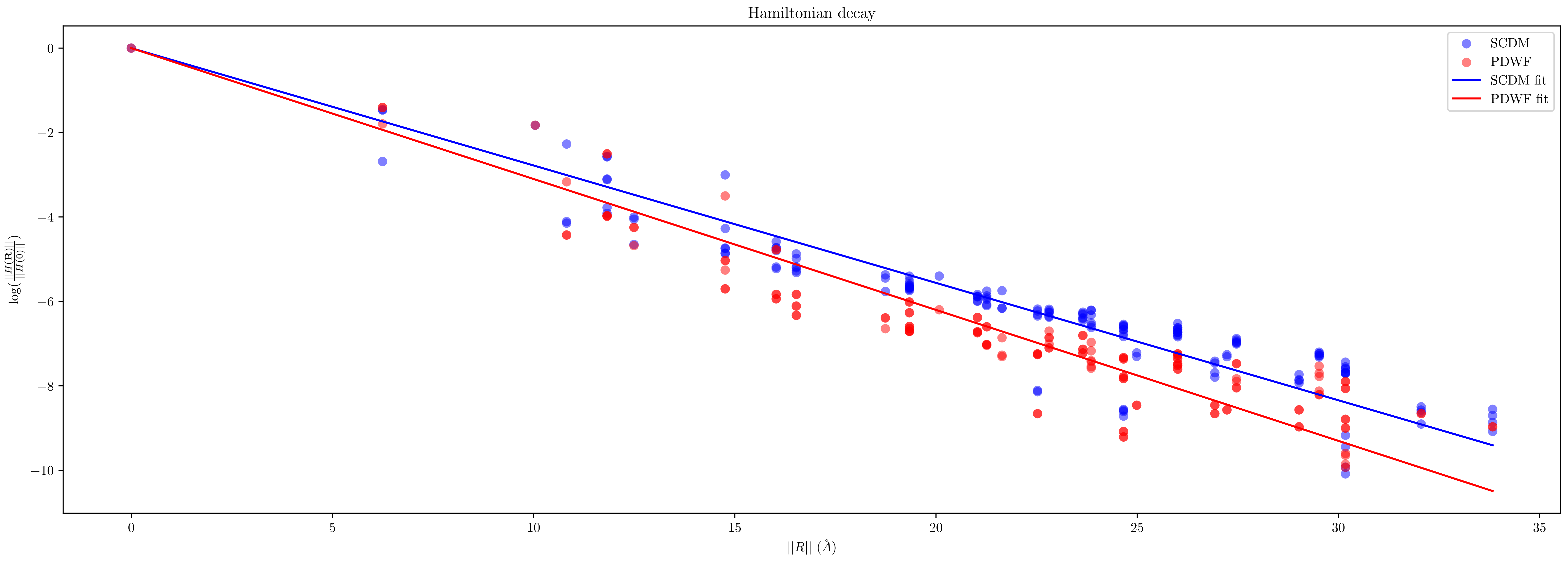

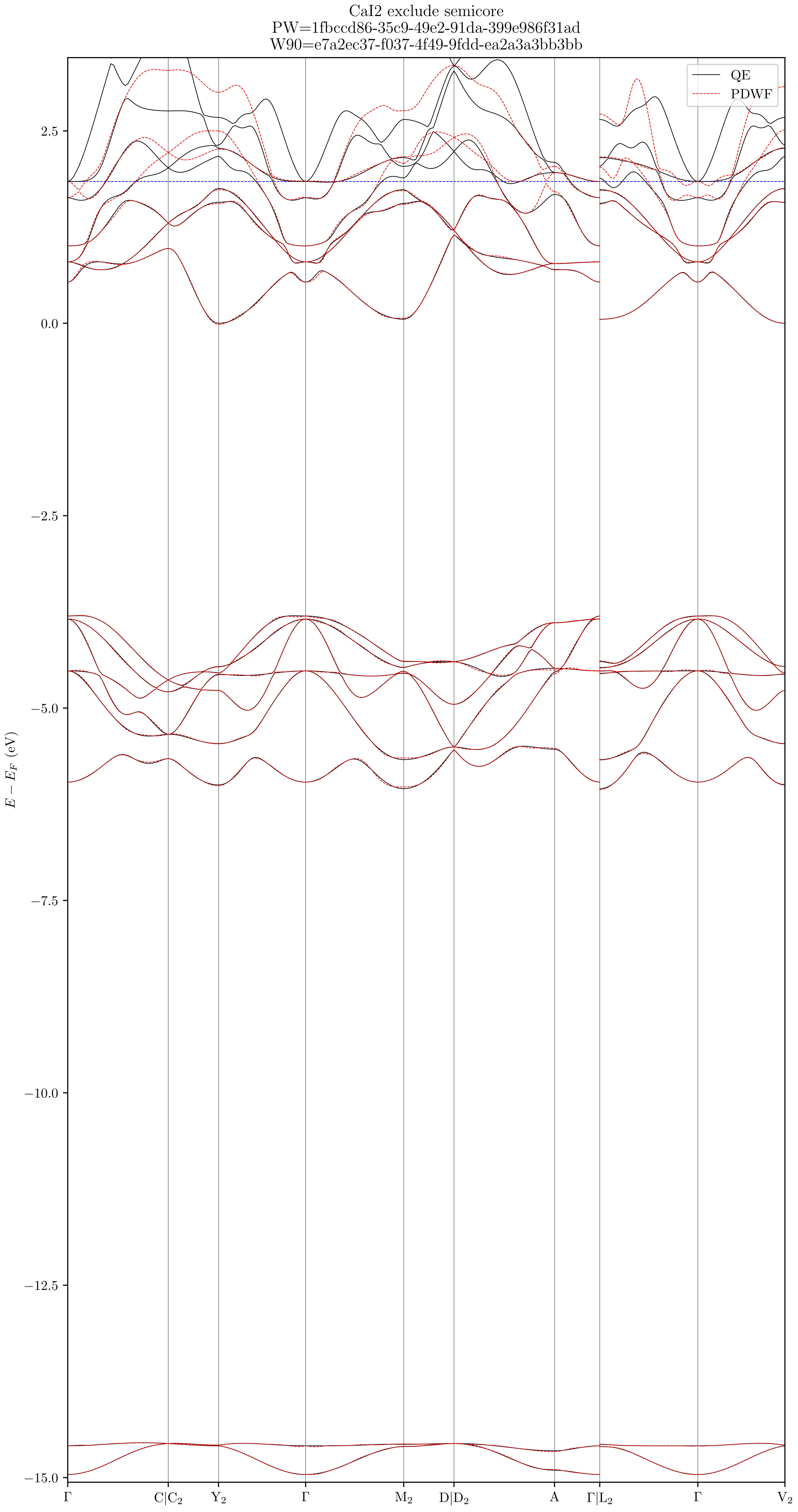

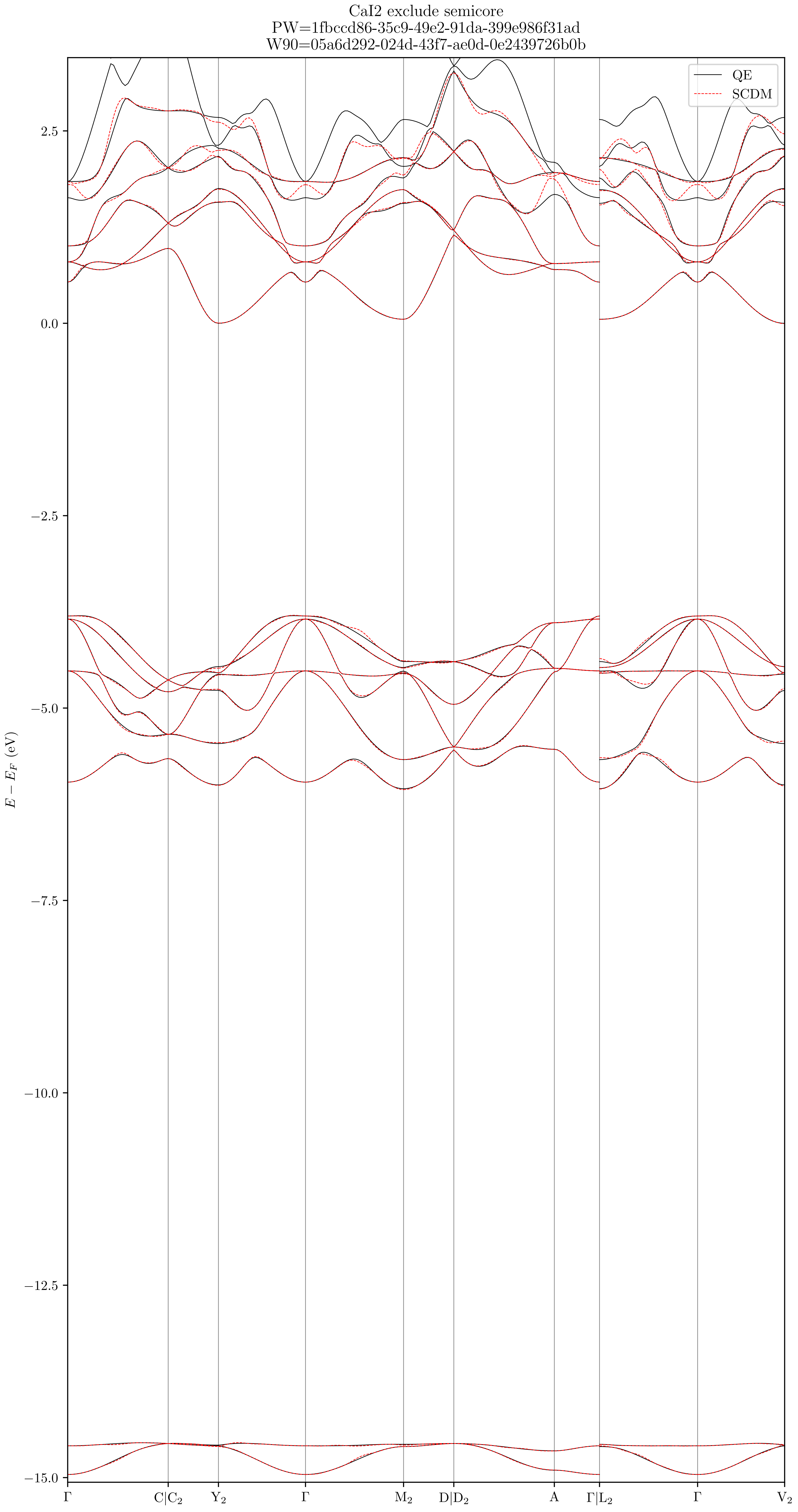

PDWF band distance (meV) and τ ($\AA$)

η_0	η_2	η_4	$\eta_0^{\max}$	$\eta_2^{\max}$	$\eta_4^{\max}$	τ
5.4	5.4	8.2	26.3	26.3	46.1	2.797

SCDM band distance (meV) and τ ($\AA$)

η_0	η_2	η_4	$\eta_0^{\max}$	$\eta_2^{\max}$	$\eta_4^{\max}$	τ
7.7	7.7	11.1	62.5	62.5	62.5	3.184

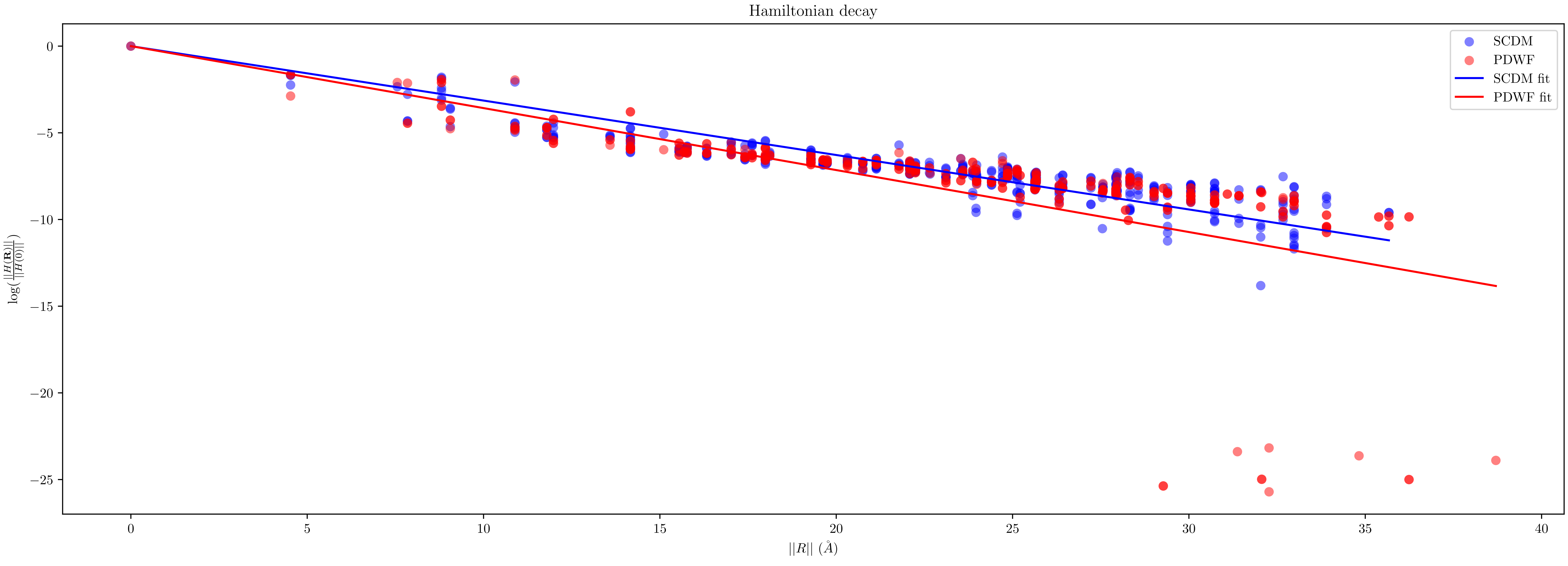

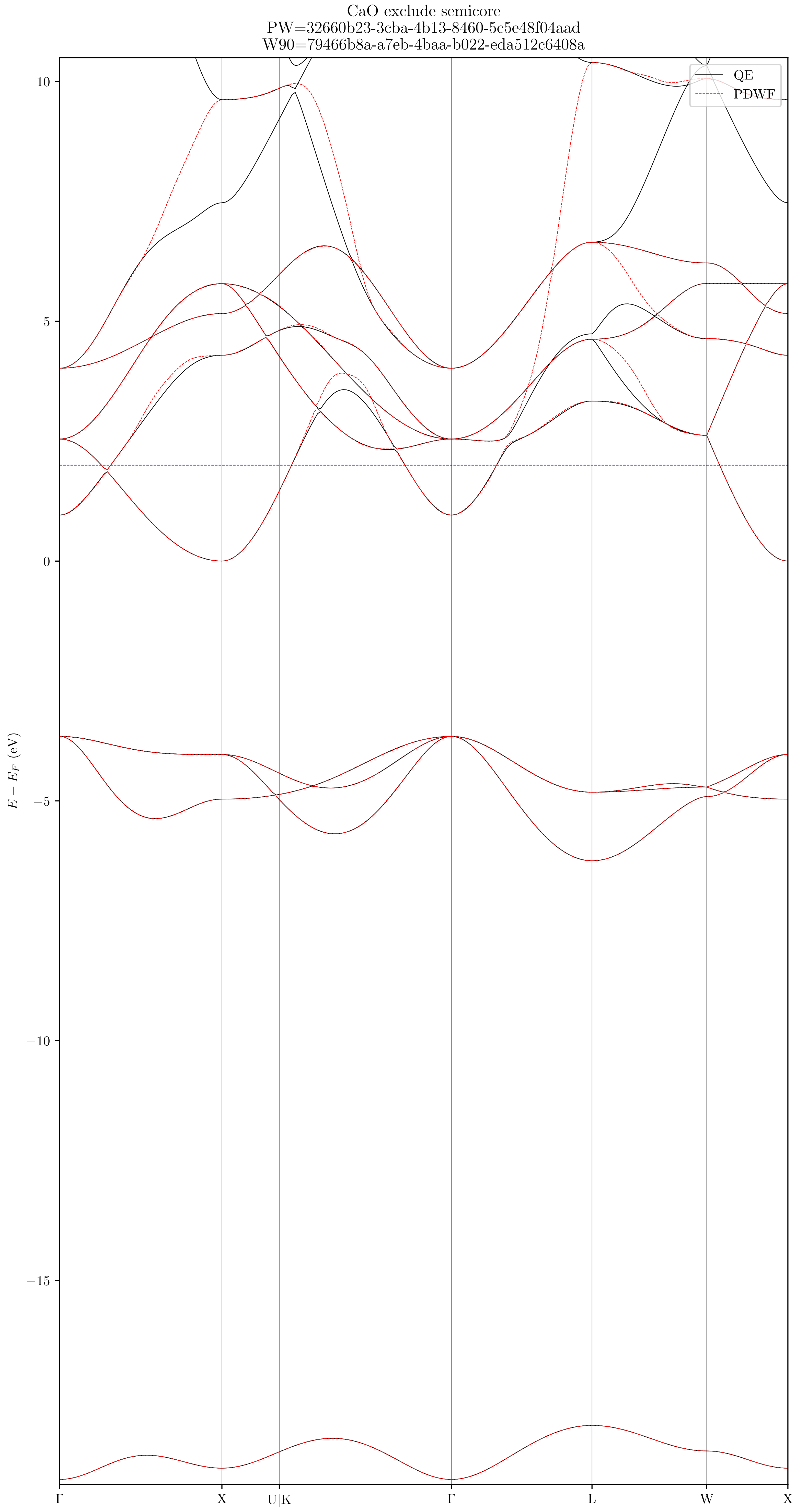

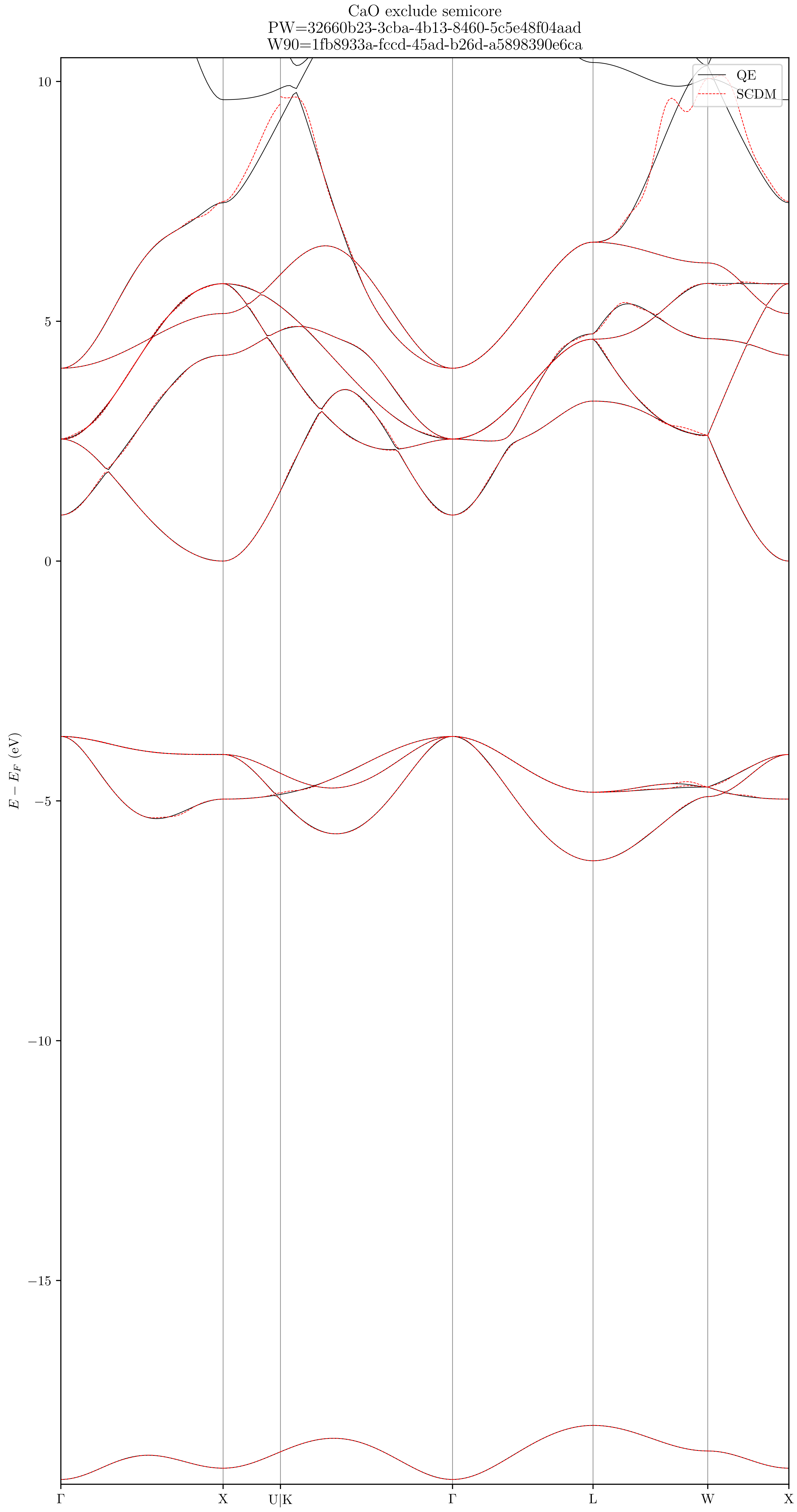

PDWF band distance (meV) and τ ($\AA$)

η_0	η_2	η_4	$\eta_0^{\max}$	$\eta_2^{\max}$	$\eta_4^{\max}$	τ
1.4	1.4	2.4	8.5	8.5	21.7	2.098

SCDM band distance (meV) and τ ($\AA$)

η_0	η_2	η_4	$\eta_0^{\max}$	$\eta_2^{\max}$	$\eta_4^{\max}$	τ
7.5	7.4	8.2	60.6	60.6	60.6	2.470

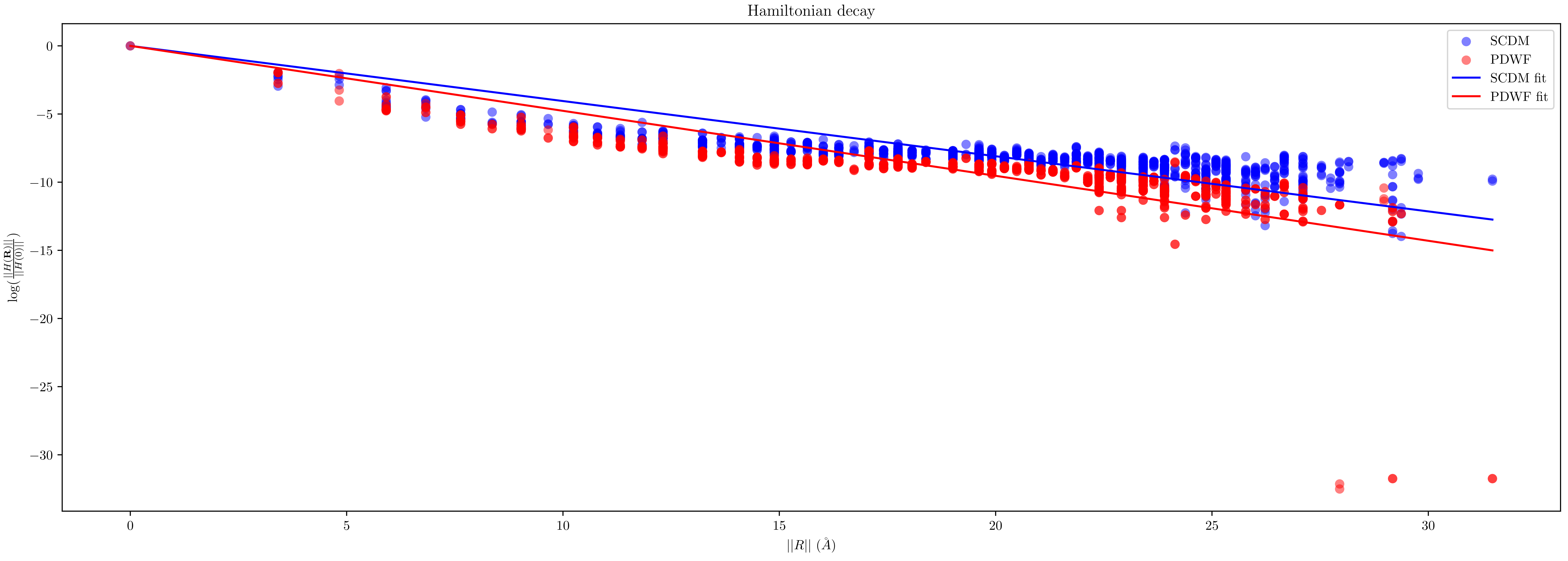

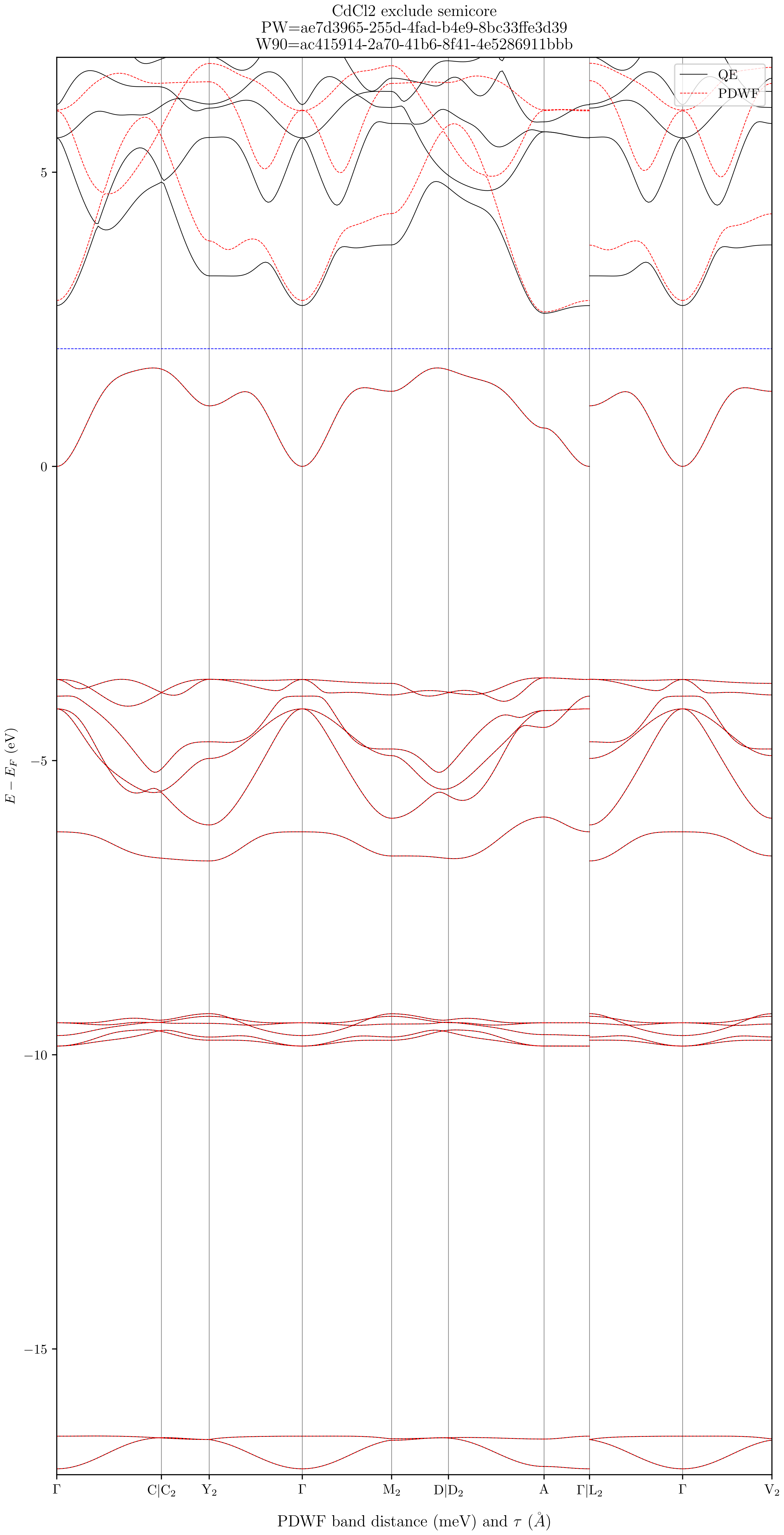

η_0	η_2	η_4	$\eta_0^{\max}$	$\eta_2^{\max}$	$\eta_4^{\max}$	τ
0.4	0.4	0.5	3.0	3.0	3.0	2.226

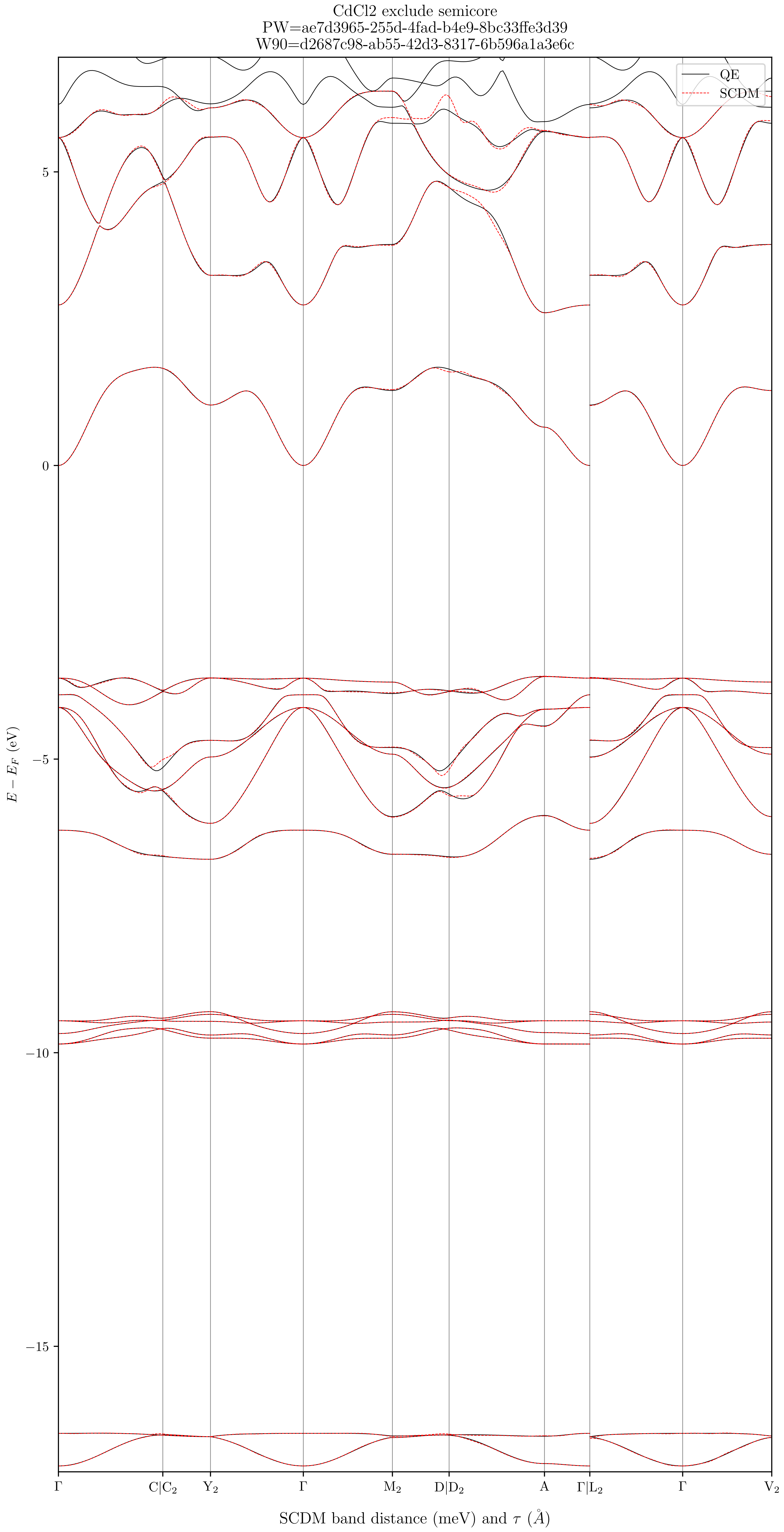

η_0	η_2	η_4	$\eta_0^{\max}$	$\eta_2^{\max}$	$\eta_4^{\max}$	τ
7.8	7.8	7.9	142.4	142.4	142.4	2.715

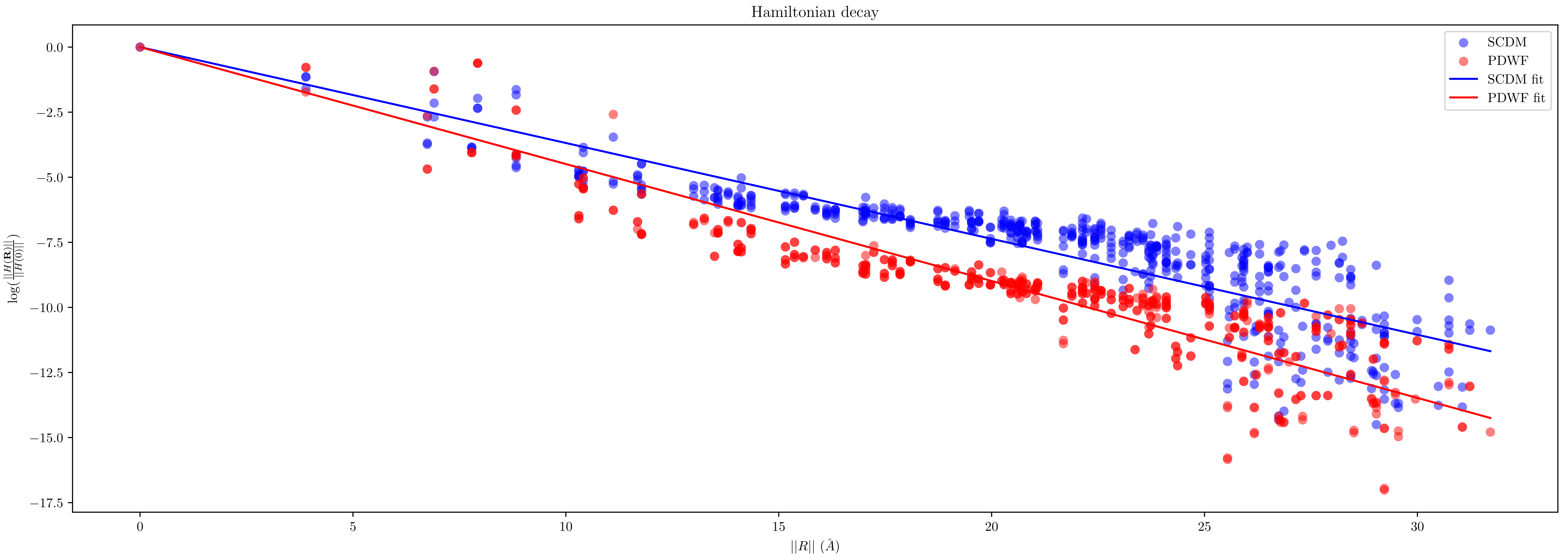

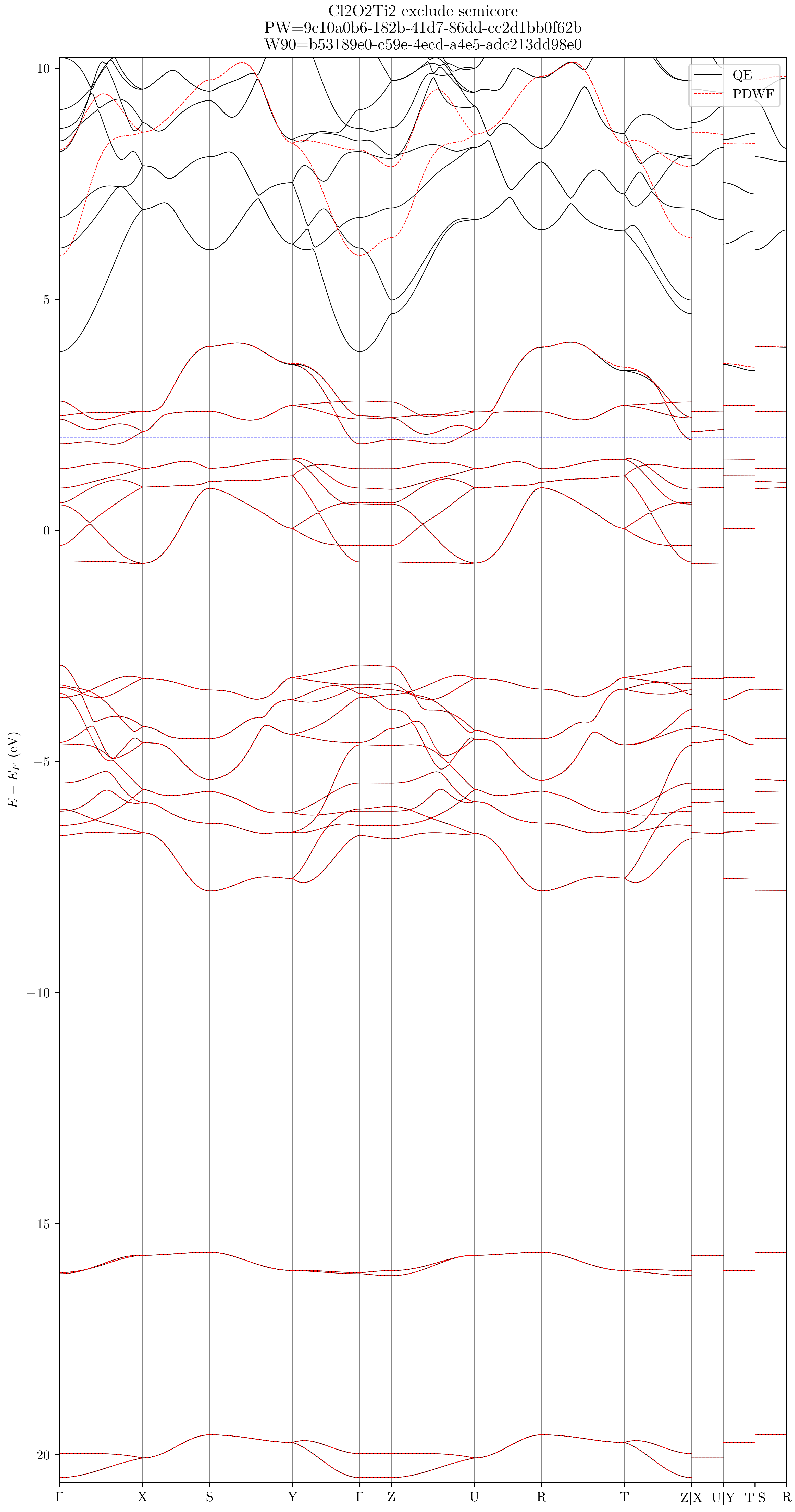

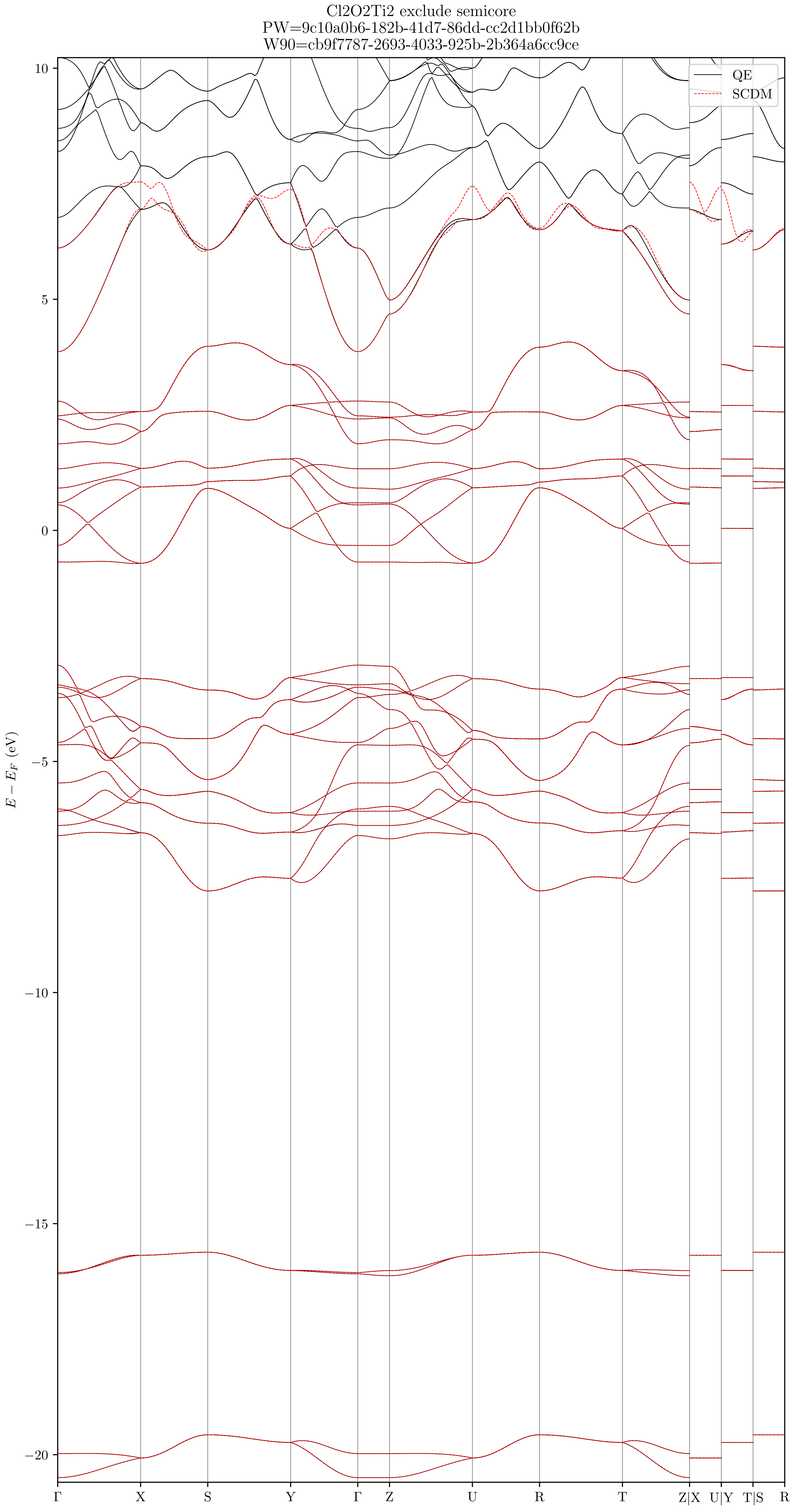

PDWF band distance (meV) and τ ($\AA$)

η_0	η_2	η_4	$\eta_0^{\max}$	$\eta_2^{\max}$	$\eta_4^{\max}$	τ
0.1	0.2	5.5	1.4	1.7	83.7	1.767

SCDM band distance (meV) and τ ($\AA$)

η_0	η_2	η_4	$\eta_0^{\max}$	$\eta_2^{\max}$	$\eta_4^{\max}$	τ
1.1	1.0	1.0	8.6	8.6	12.9	2.617

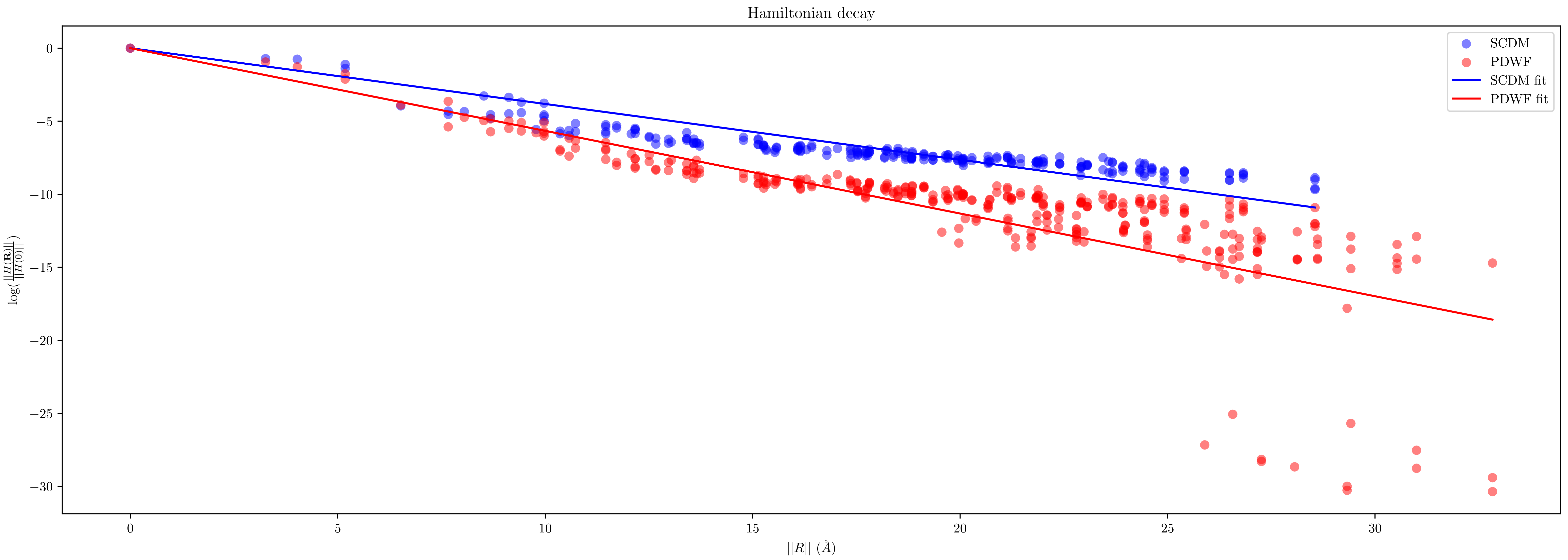

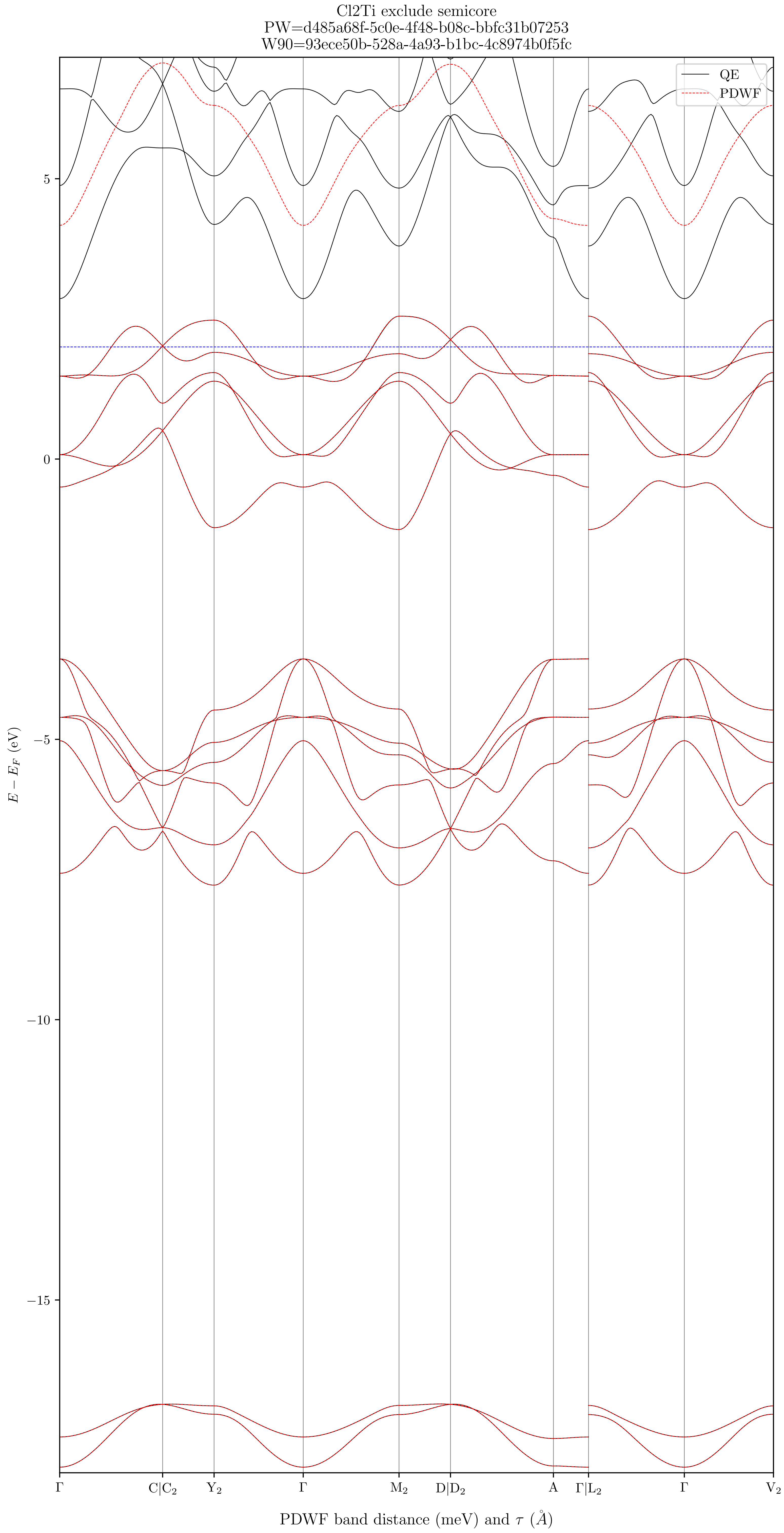

η_0	η_2	η_4	$\eta_0^{\max}$	$\eta_2^{\max}$	$\eta_4^{\max}$	τ
0.1	0.3	74.6	0.7	3.2	495.8	2.031

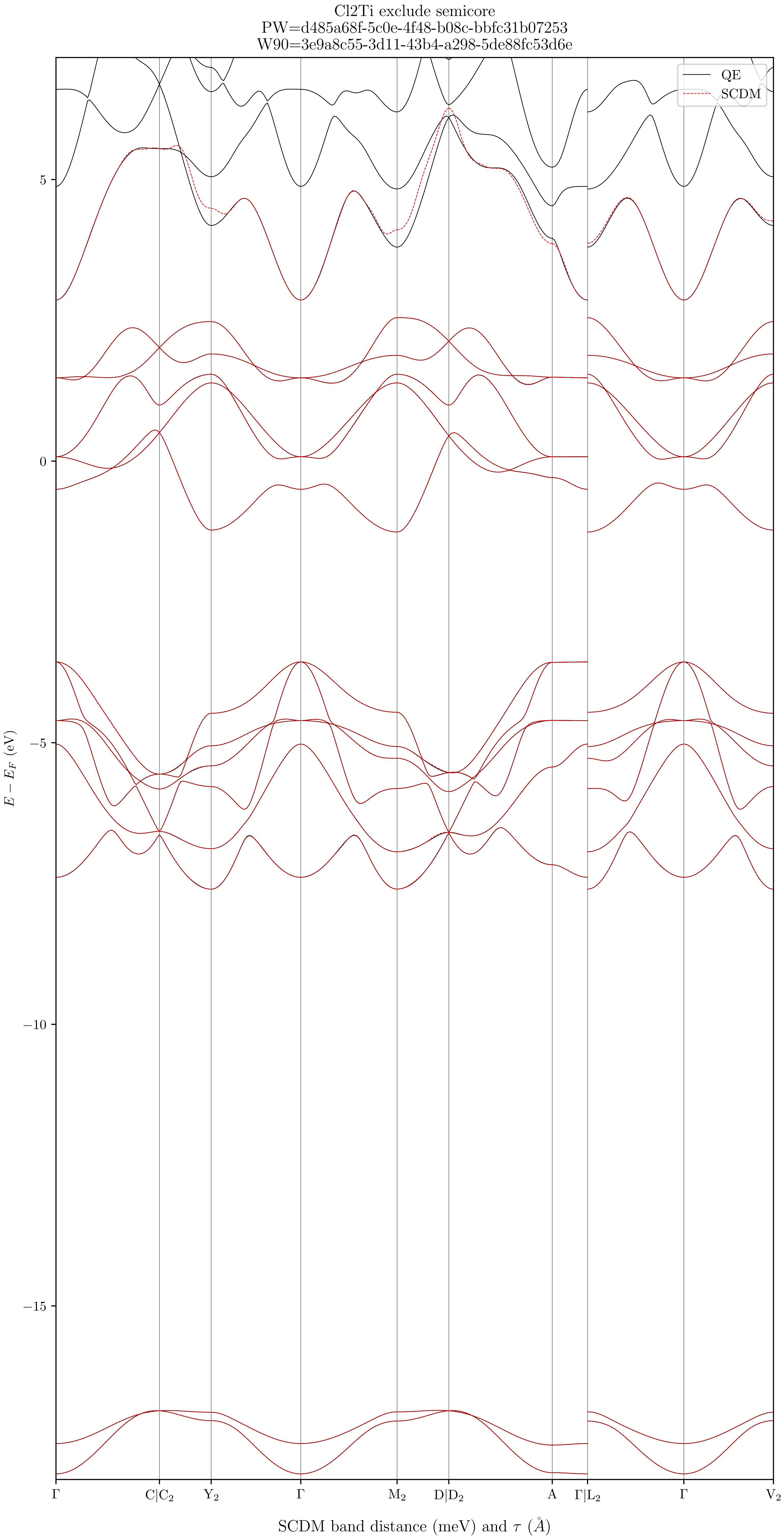

η_0	η_2	η_4	$\eta_0^{\max}$	$\eta_2^{\max}$	$\eta_4^{\max}$	τ
1.7	1.6	9.4	17.4	17.4	139.5	2.465

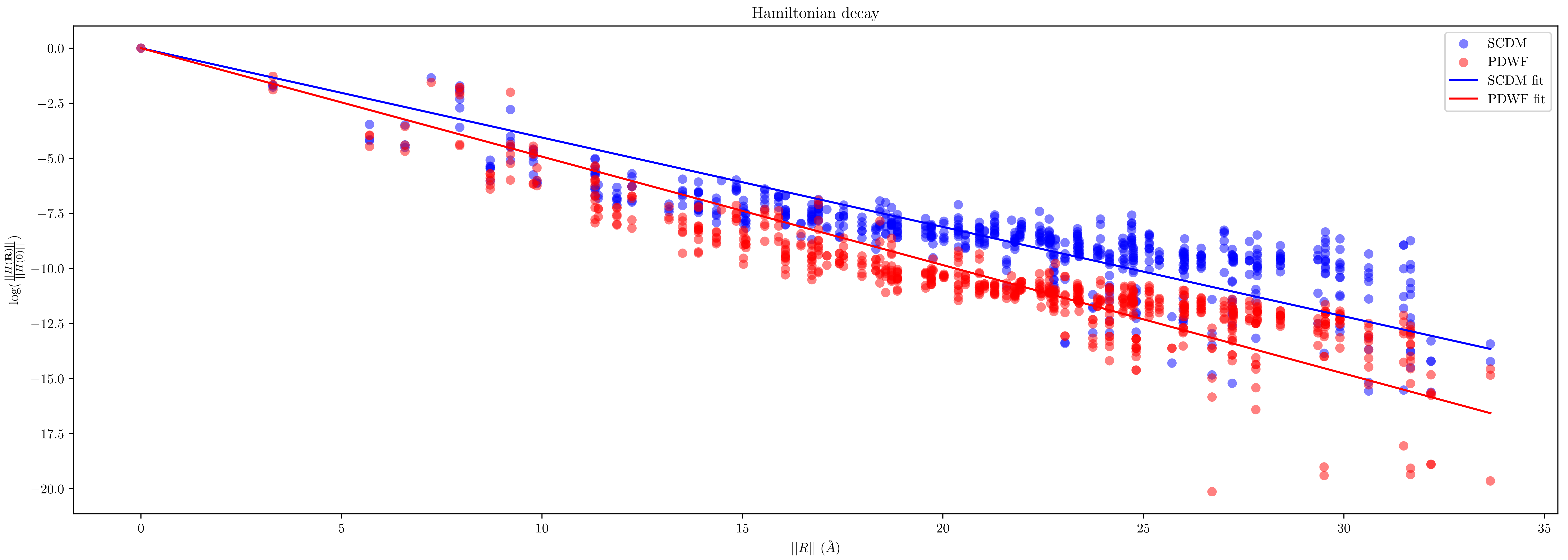

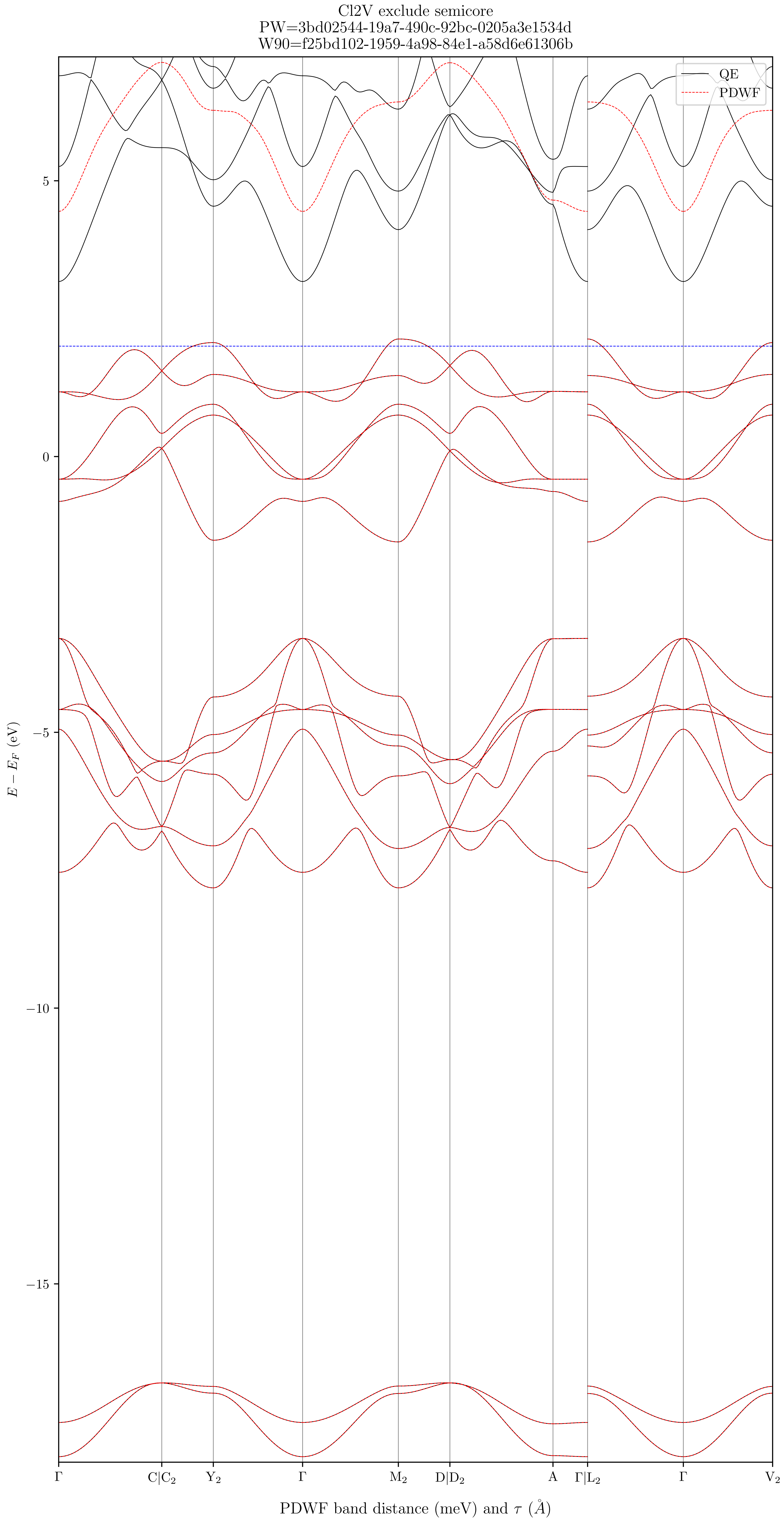

η_0	η_2	η_4	$\eta_0^{\max}$	$\eta_2^{\max}$	$\eta_4^{\max}$	τ
0.2	0.1	36.2	1.0	1.0	137.0	1.925

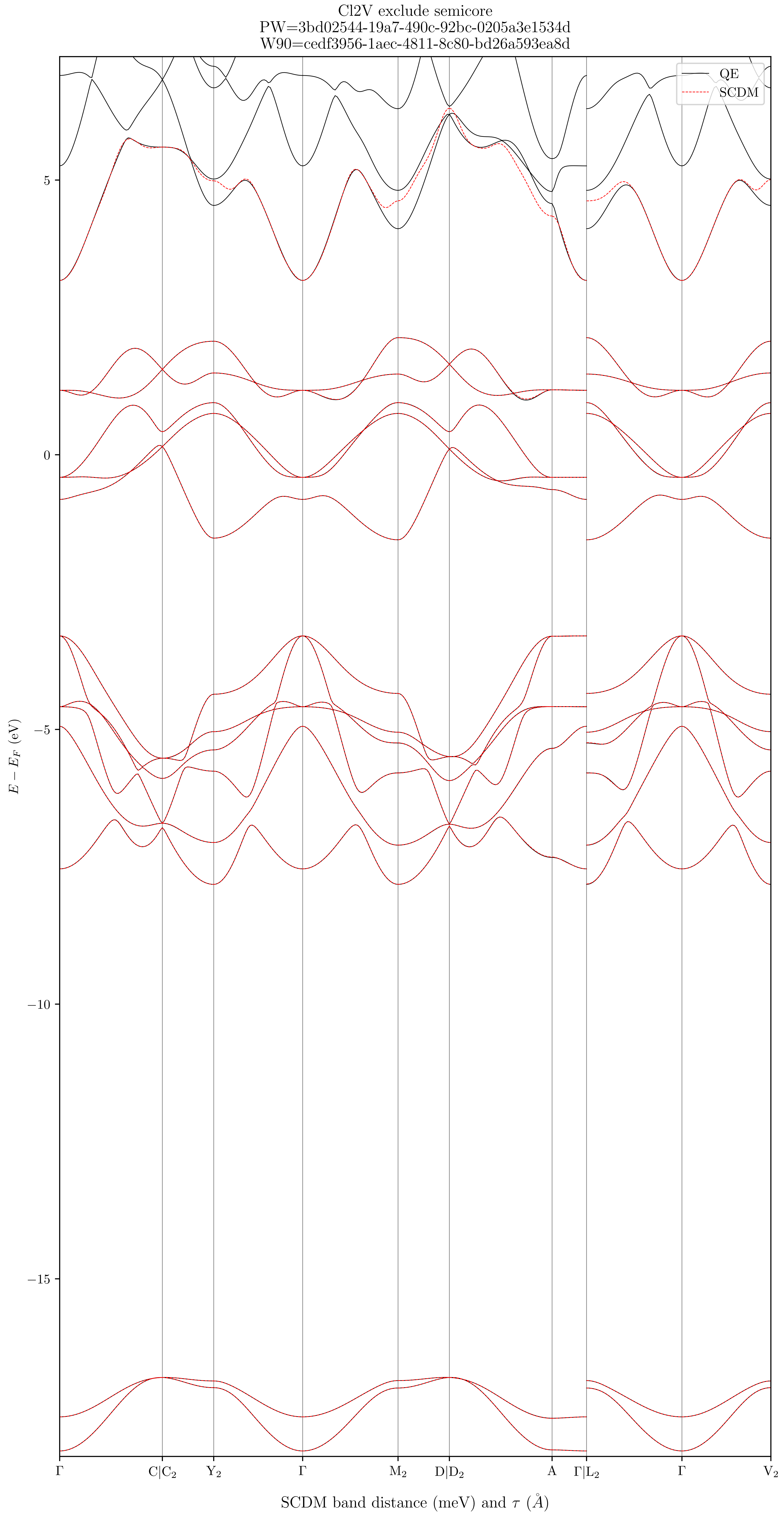

η_0	η_2	η_4	$\eta_0^{\max}$	$\eta_2^{\max}$	$\eta_4^{\max}$	τ
1.2	1.6	4.5	10.0	20.9	39.8	2.479

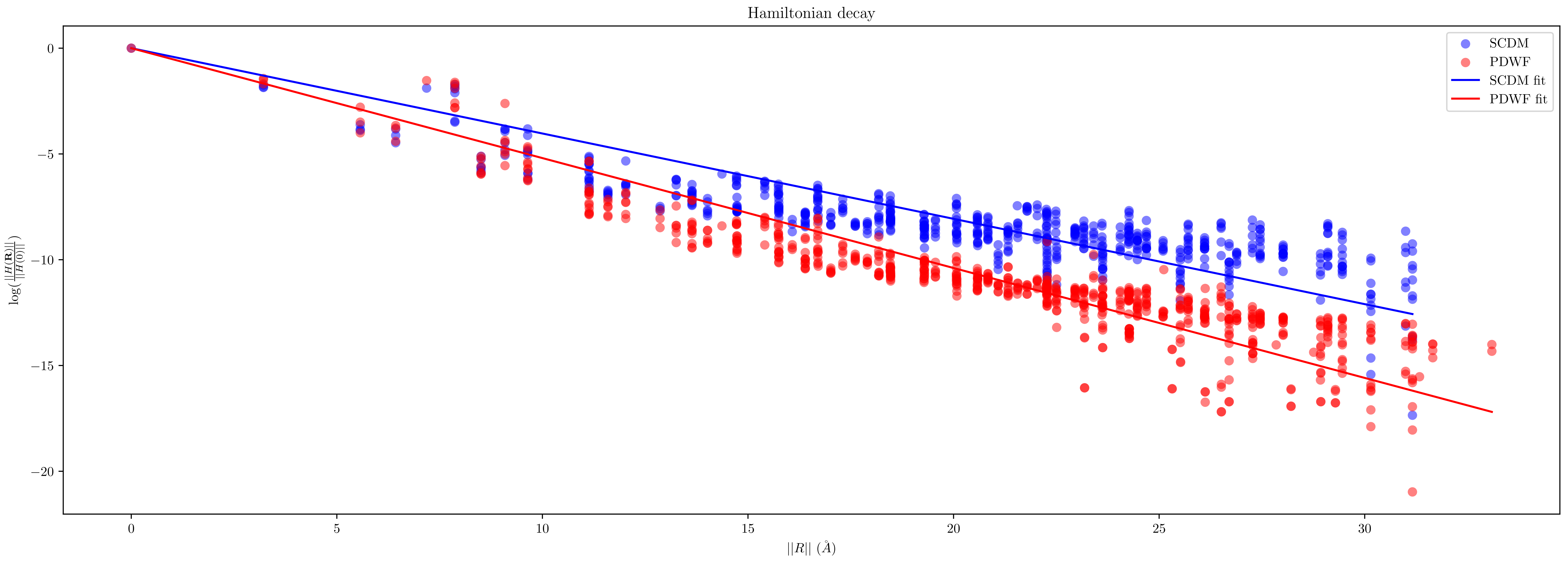

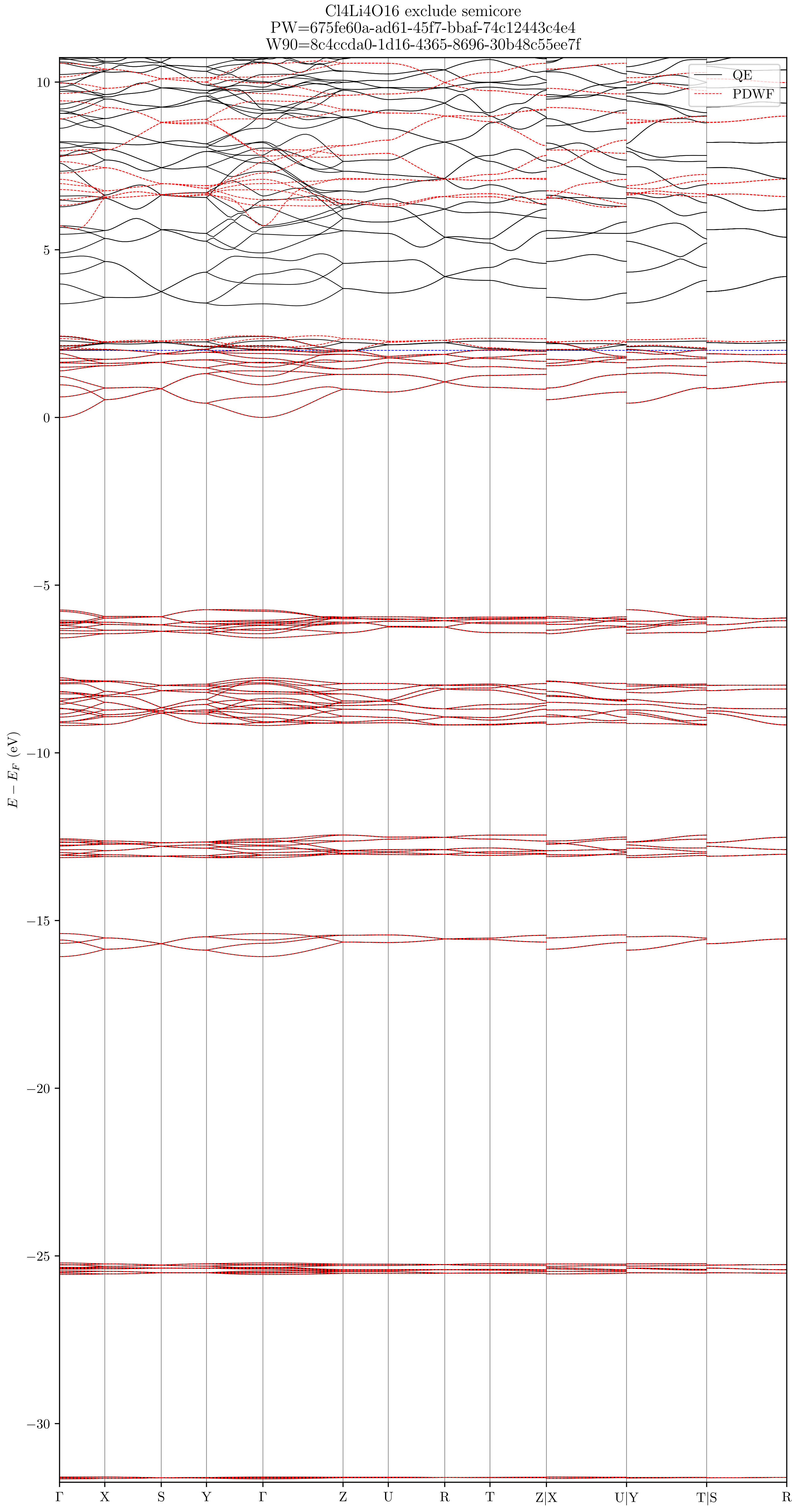

PDWF band distance (meV) and τ ($\AA$)

η_0	η_2	η_4	$\eta_0^{\max}$	$\eta_2^{\max}$	$\eta_4^{\max}$	τ
0.2	0.2	0.2	1.6	1.6	1.6	2.397

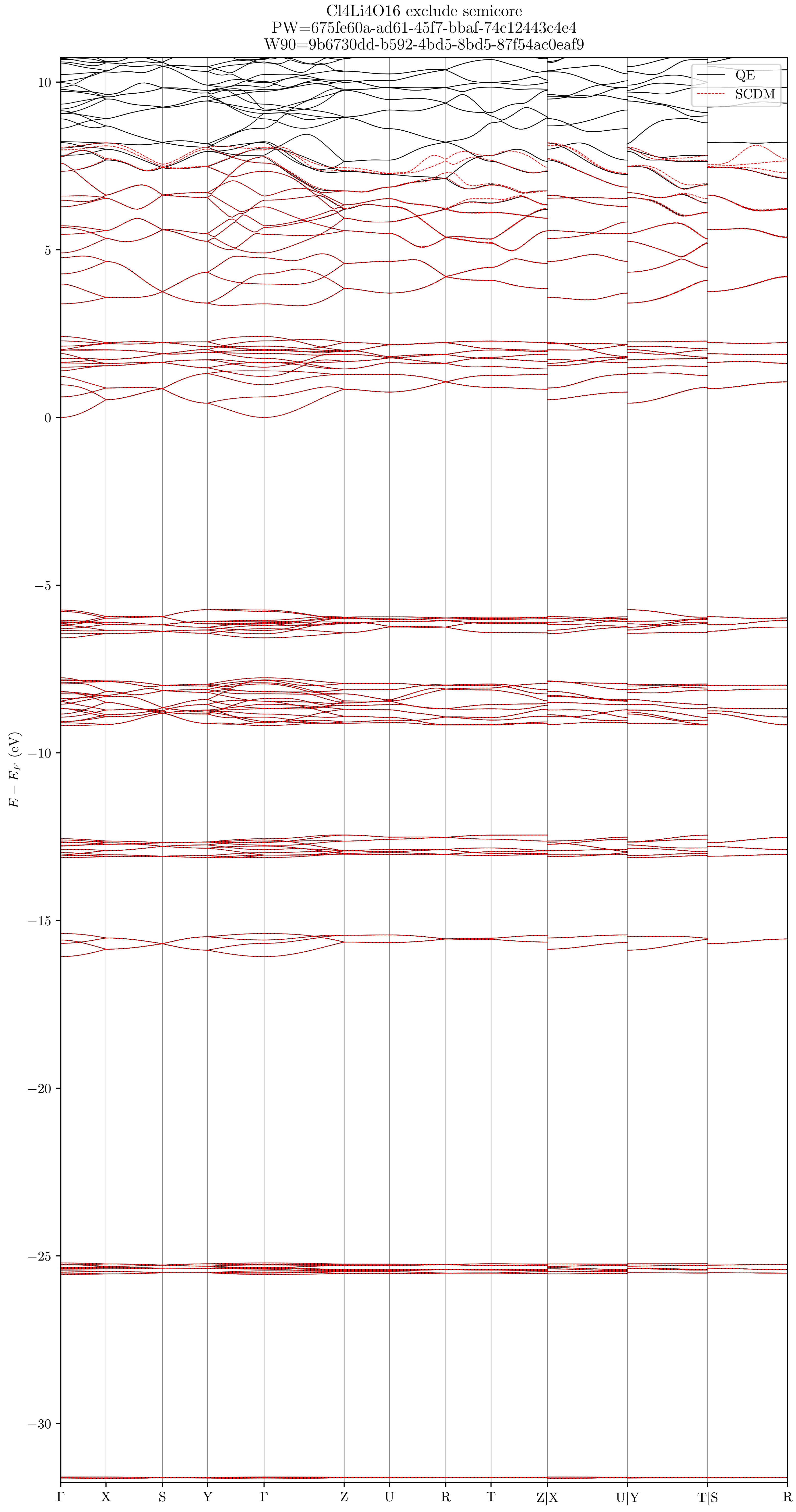

SCDM band distance (meV) and τ ($\AA$)

η_0	η_2	η_4	$\eta_0^{\max}$	$\eta_2^{\max}$	$\eta_4^{\max}$	τ
0.2	0.2	0.3	2.0	2.0	3.6	2.584

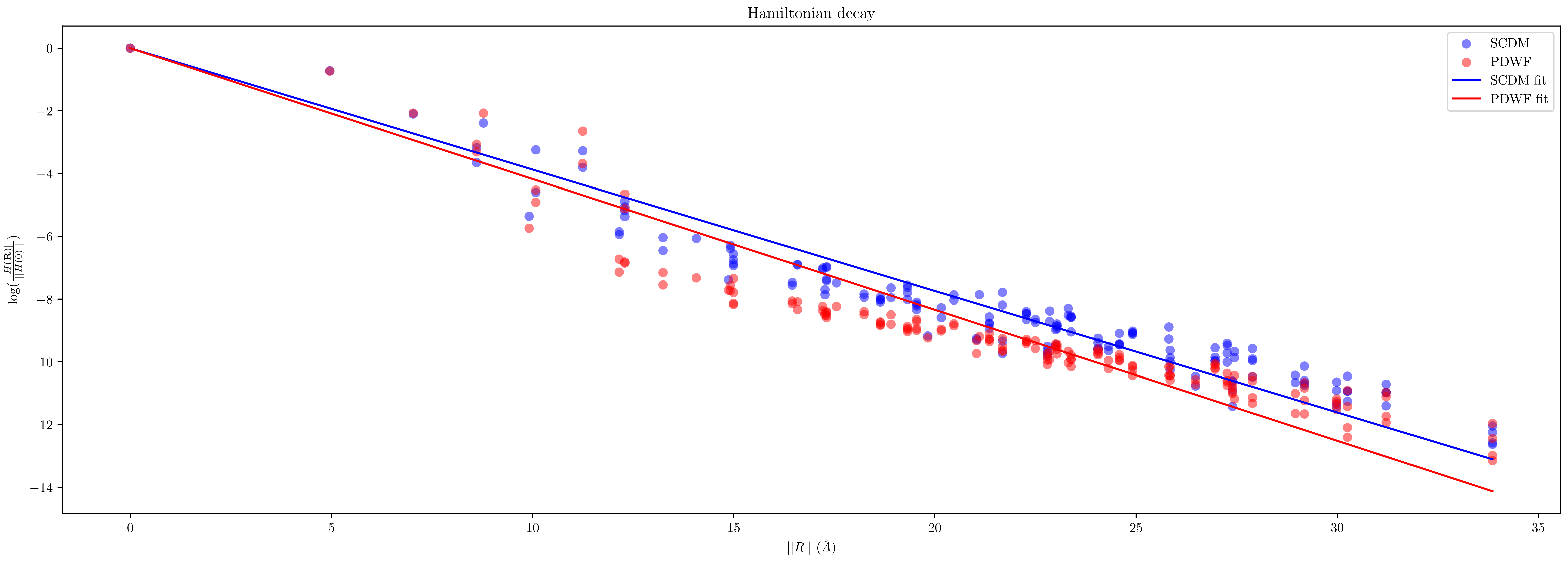

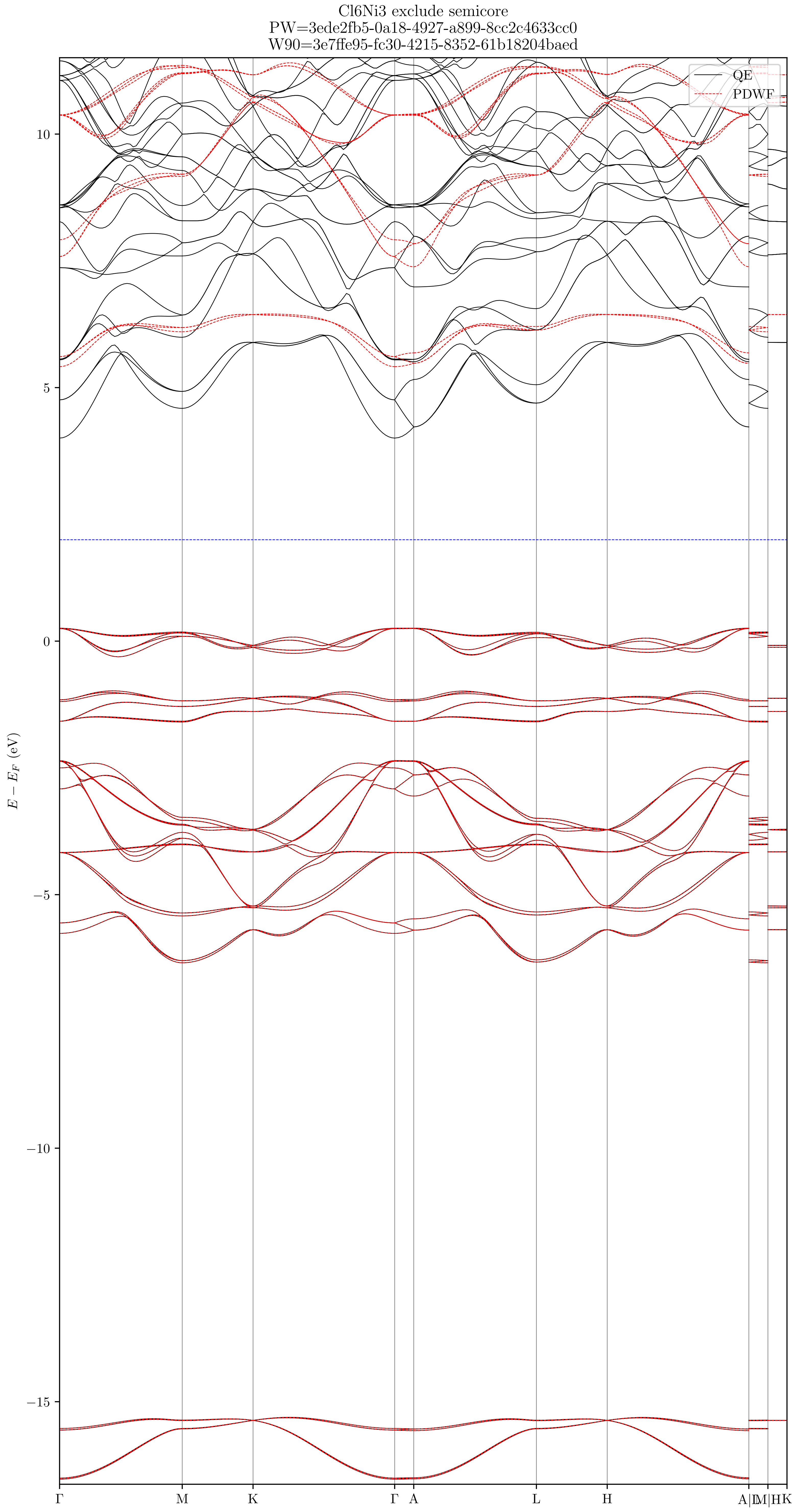

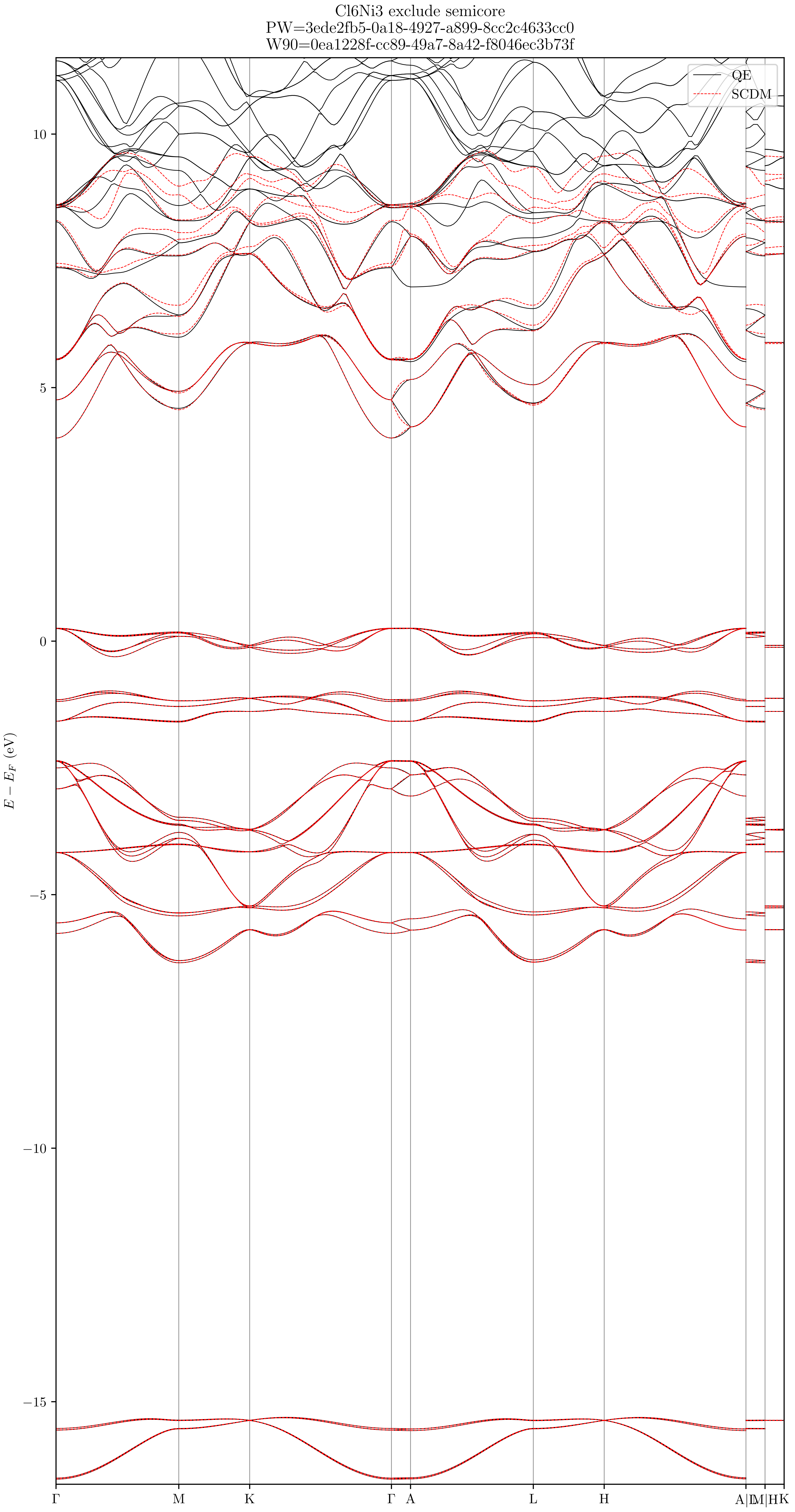

PDWF band distance (meV) and τ ($\AA$)

η_0	η_2	η_4	$\eta_0^{\max}$	$\eta_2^{\max}$	$\eta_4^{\max}$	τ
0.2	0.2	1.5	2.0	2.0	2.0	2.432

SCDM band distance (meV) and τ ($\AA$)

η_0	η_2	η_4	$\eta_0^{\max}$	$\eta_2^{\max}$	$\eta_4^{\max}$	τ
1.2	1.2	1.3	11.7	11.7	11.7	3.262

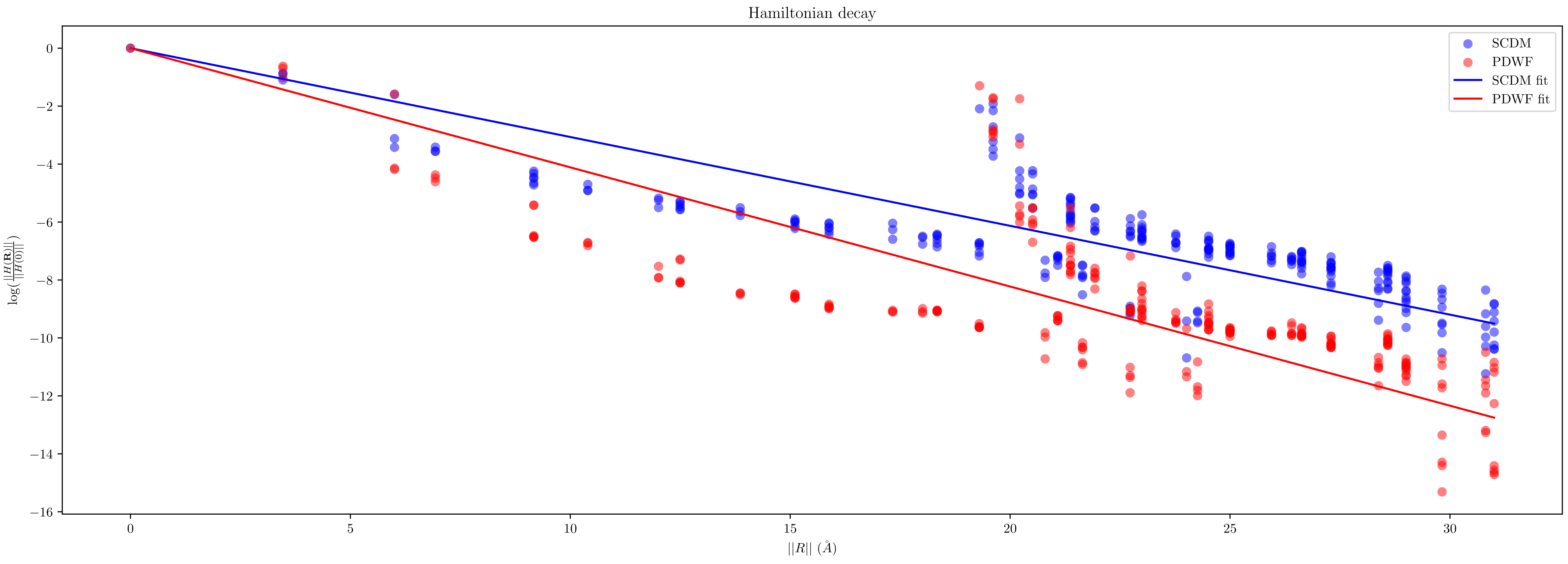

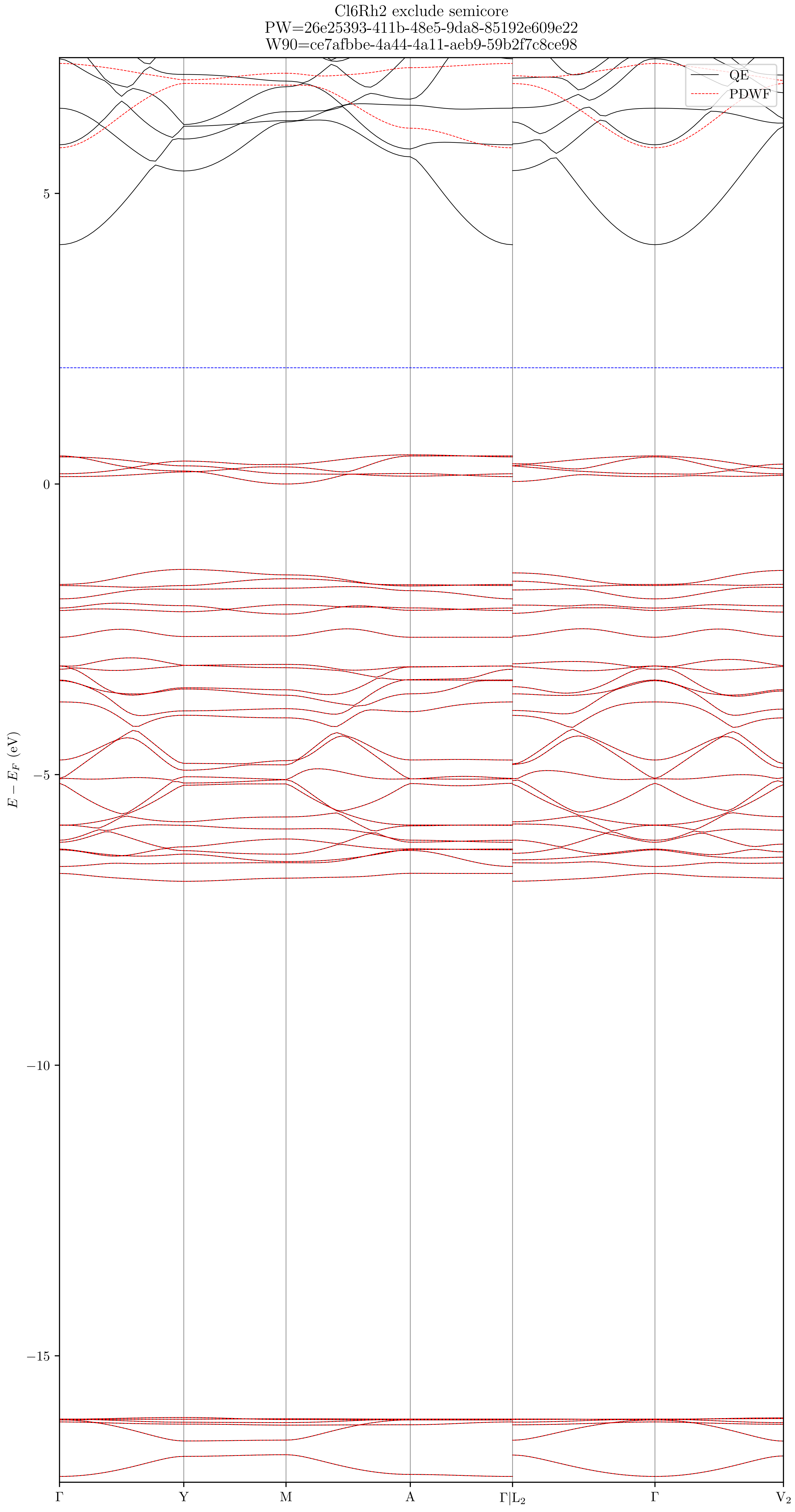

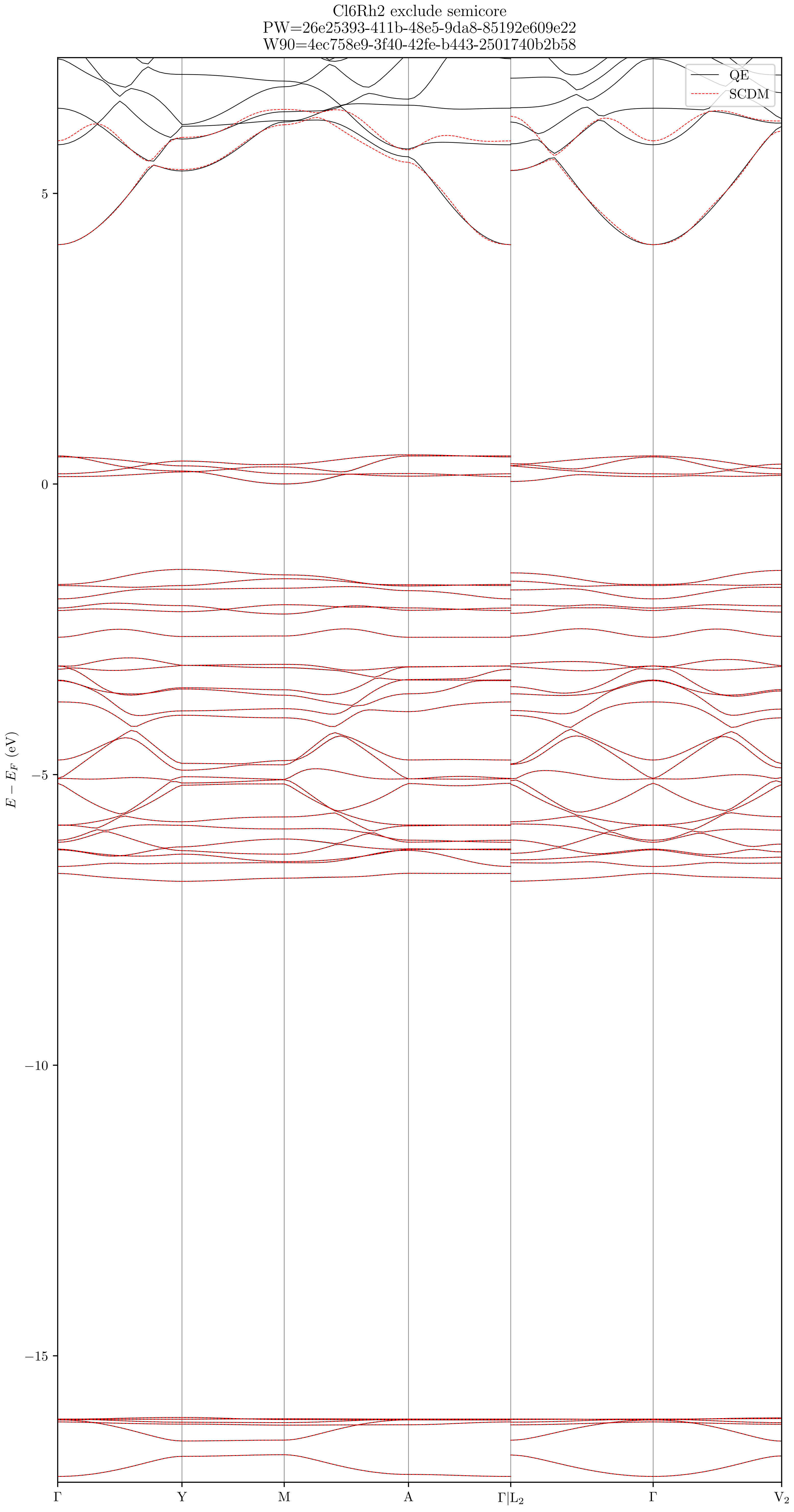

PDWF band distance (meV) and τ ($\AA$)

η_0	η_2	η_4	$\eta_0^{\max}$	$\eta_2^{\max}$	$\eta_4^{\max}$	τ
0.1	0.1	0.1	0.9	0.9	0.9	2.099

SCDM band distance (meV) and τ ($\AA$)

η_0	η_2	η_4	$\eta_0^{\max}$	$\eta_2^{\max}$	$\eta_4^{\max}$	τ
0.6	0.6	0.6	4.8	4.8	4.8	2.703

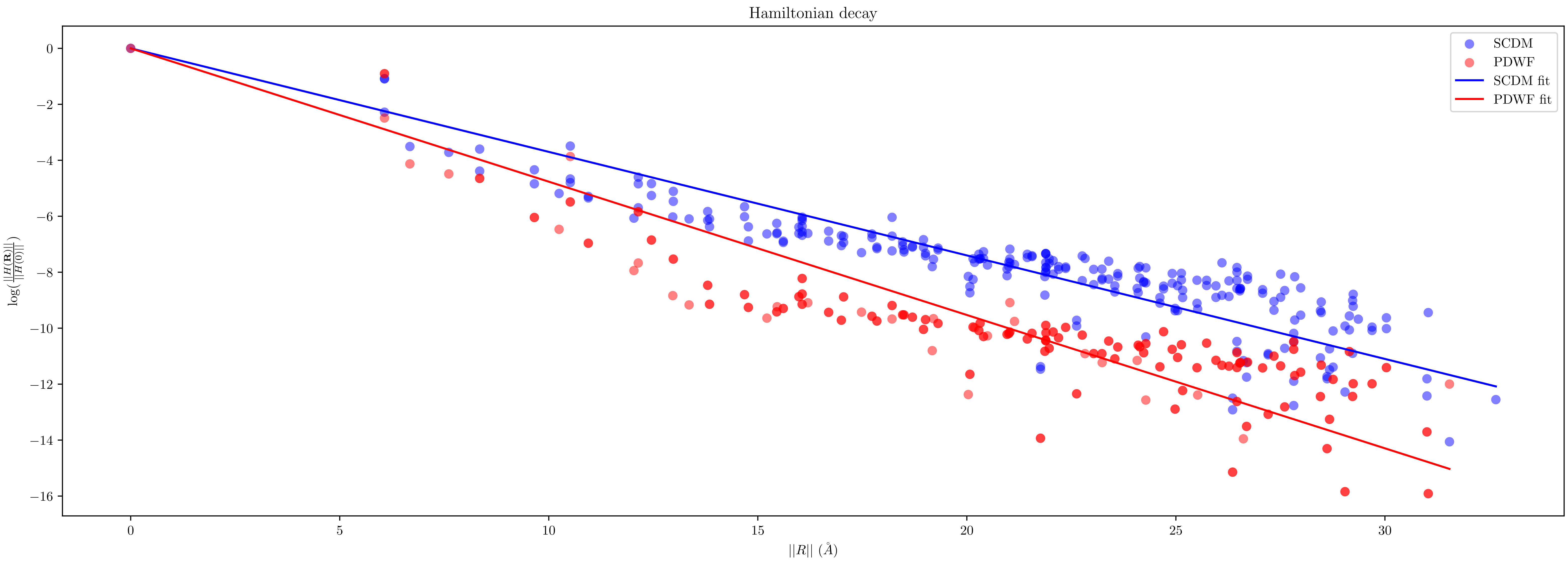

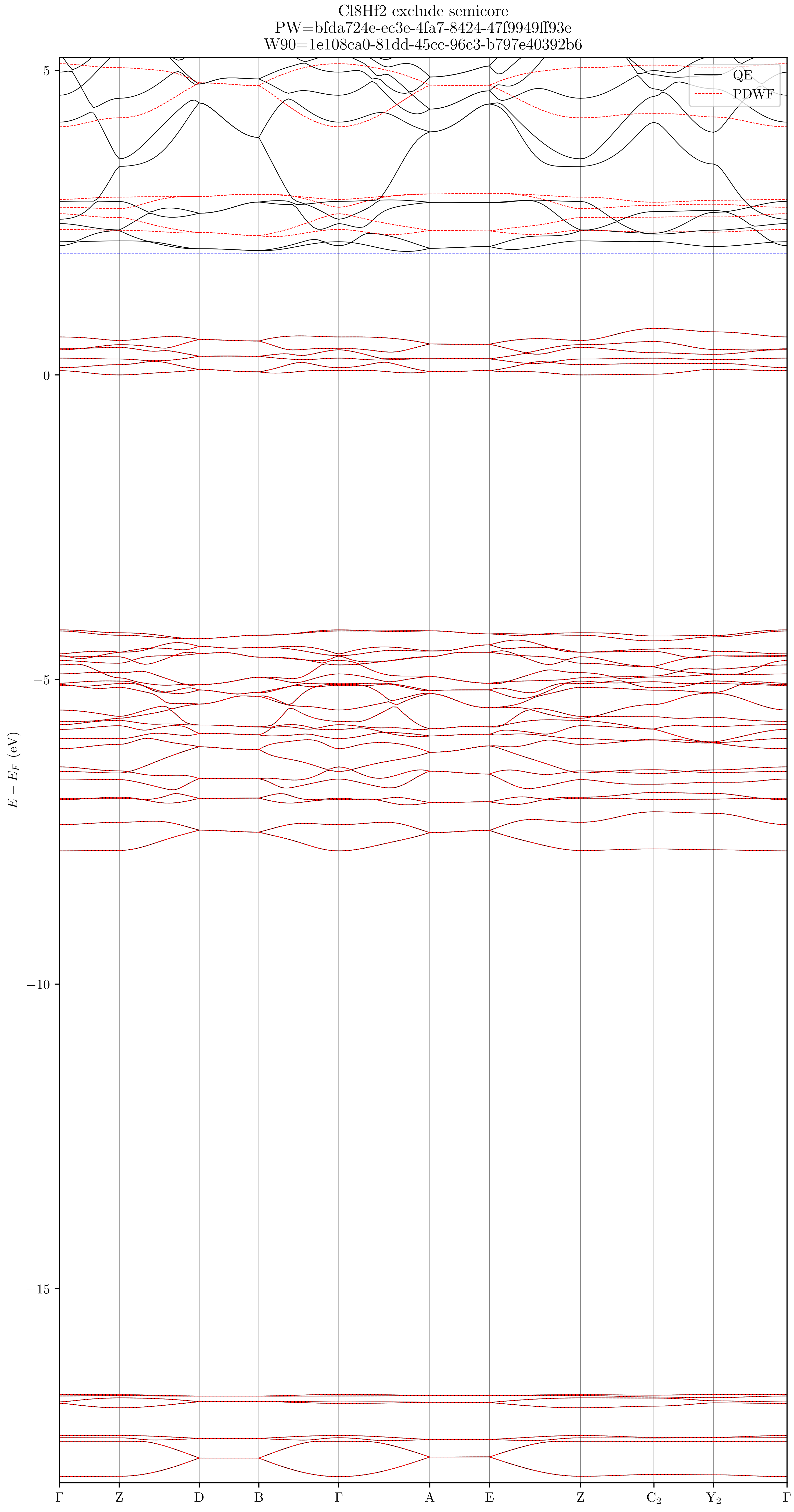

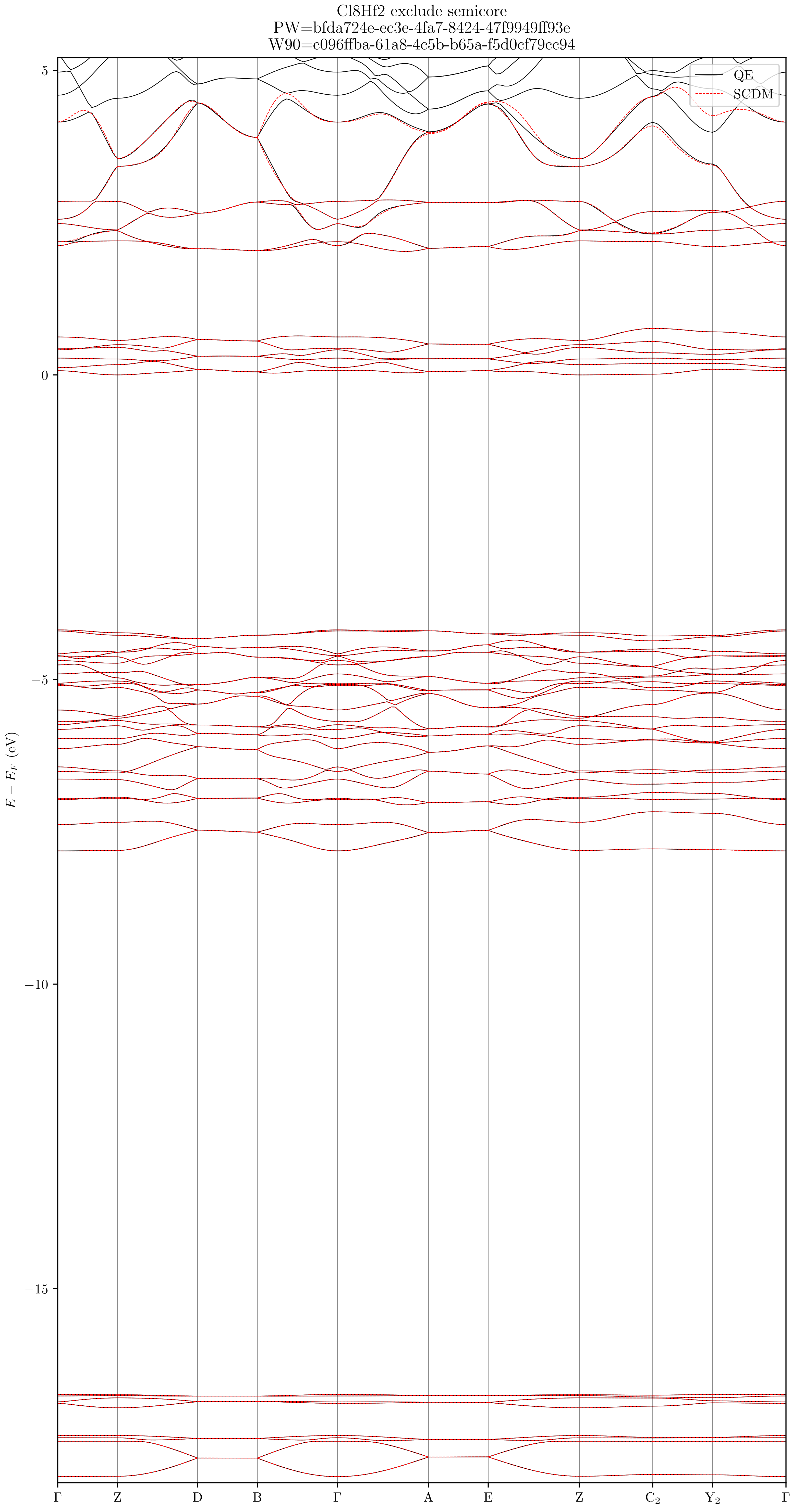

PDWF band distance (meV) and τ ($\AA$)

η_0	η_2	η_4	$\eta_0^{\max}$	$\eta_2^{\max}$	$\eta_4^{\max}$	τ
0.1	0.7	76.6	0.6	0.6	460.3	2.080

SCDM band distance (meV) and τ ($\AA$)

η_0	η_2	η_4	$\eta_0^{\max}$	$\eta_2^{\max}$	$\eta_4^{\max}$	τ
0.5	0.5	1.6	5.1	5.1	27.1	2.522

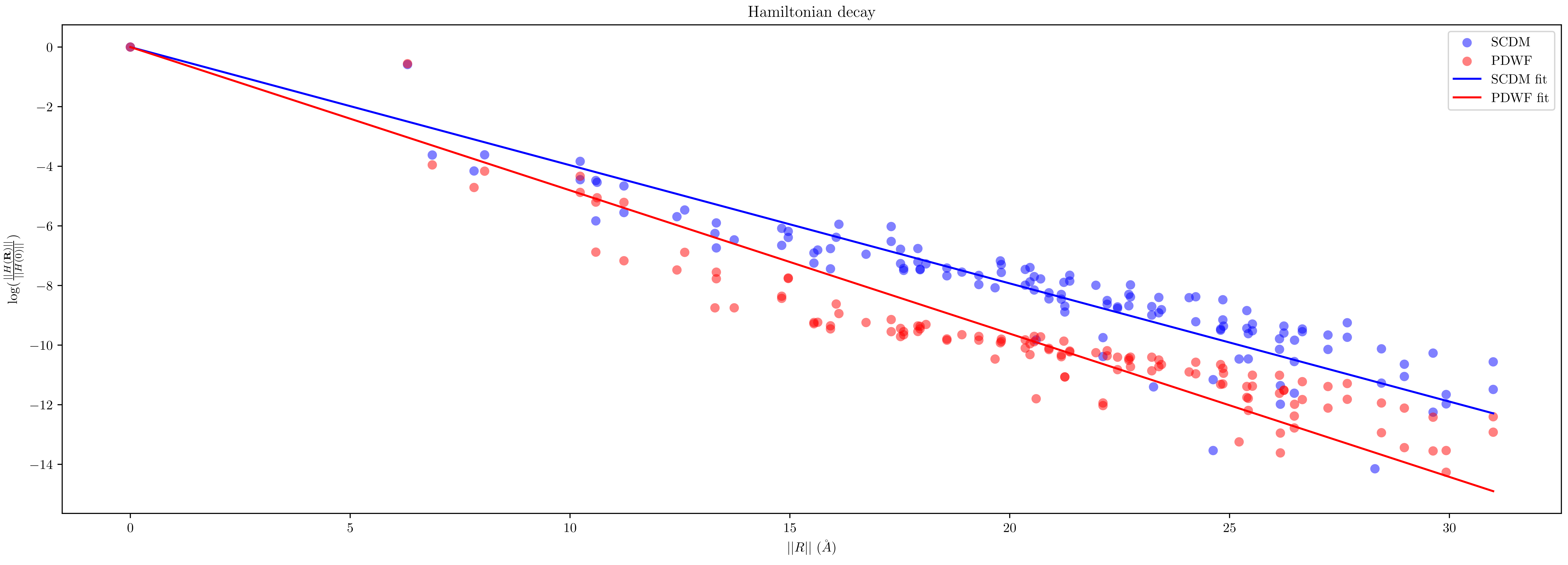

Cl8Sr4 exclude semicore
PW=5b3b9fff-736c-47a7-b3c7-1b5fe6dc92cf
W90=12397093-56f9-4a3a-a10c-80333e64d9be

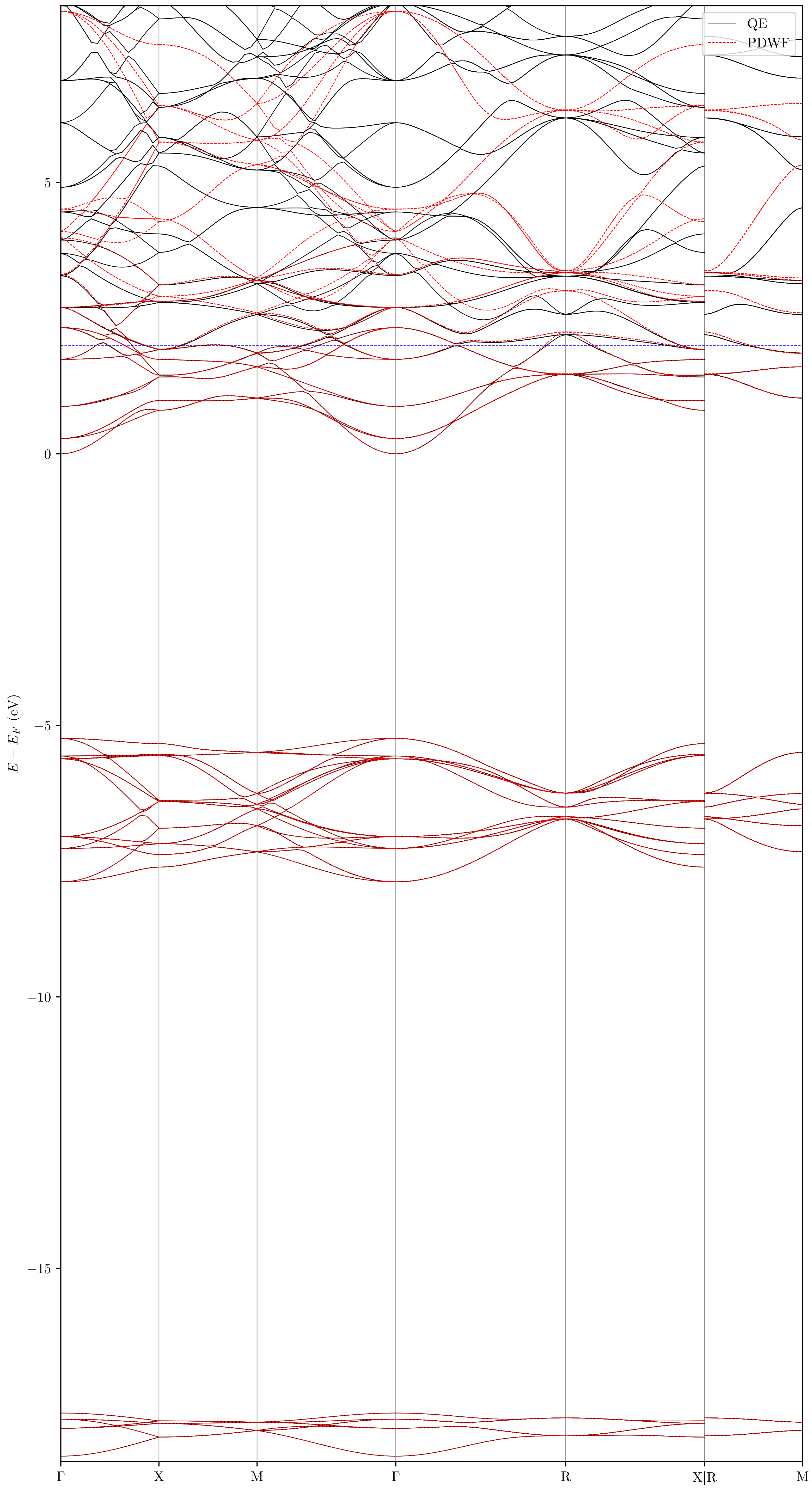

Cl8Sr4 exclude semicore
PW=5b3b9fff-736c-47a7-b3c7-1b5fe6dc92cf
W90=8d096c0d-332e-4df3-8800-ba6850f0c805

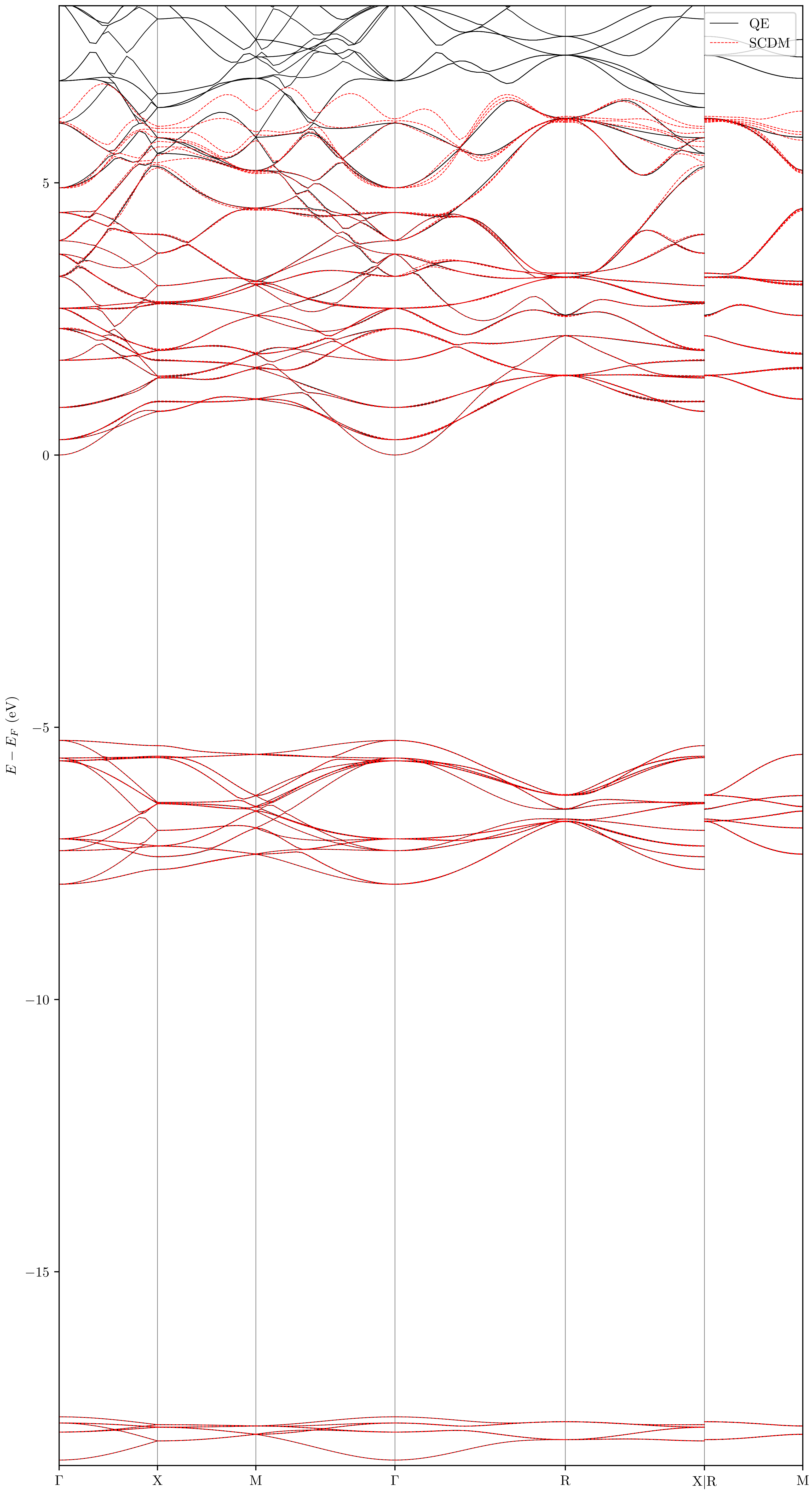

PDWF band distance (meV) and τ ($\AA$)

η_0	η_2	η_4	$\eta_0^{\max}$	$\eta_2^{\max}$	$\eta_4^{\max}$	τ
0.6	0.6	7.1	4.0	4.0	44.6	2.641

SCDM band distance (meV) and τ ($\AA$)

η_0	η_2	η_4	$\eta_0^{\max}$	$\eta_2^{\max}$	$\eta_4^{\max}$	τ
2.4	2.4	4.1	13.4	13.4	23.8	3.261

Hamiltonian decay

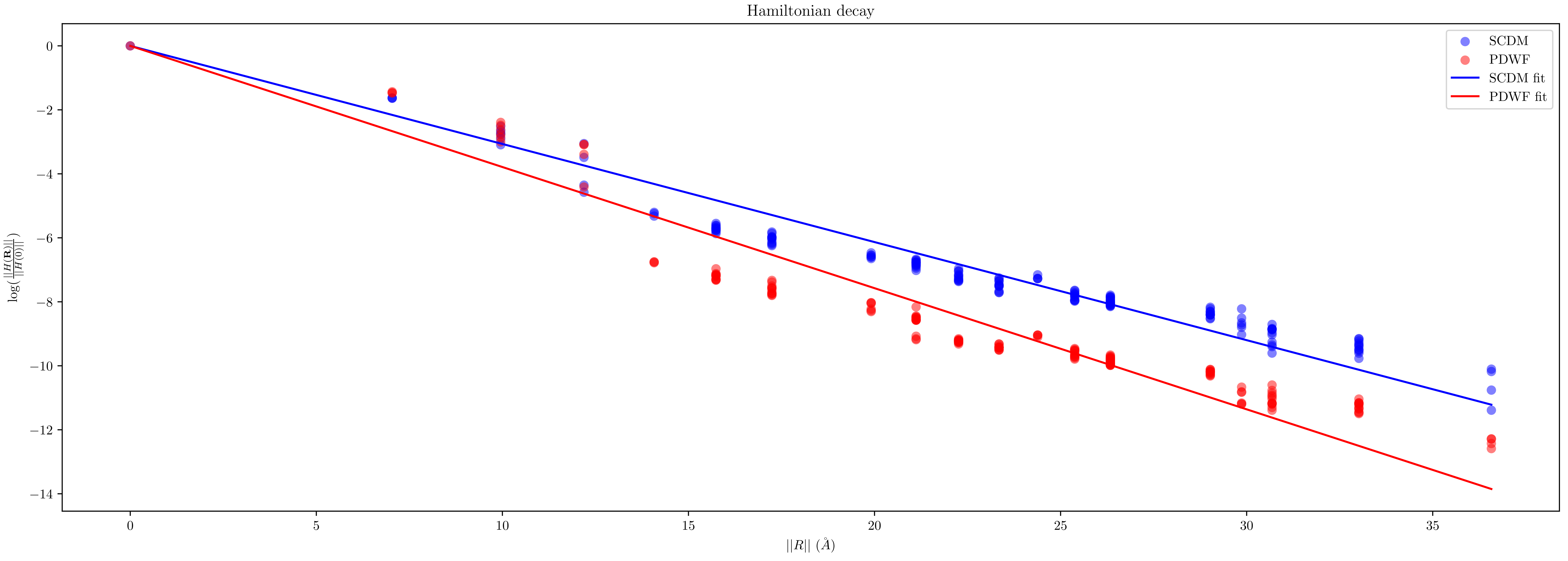

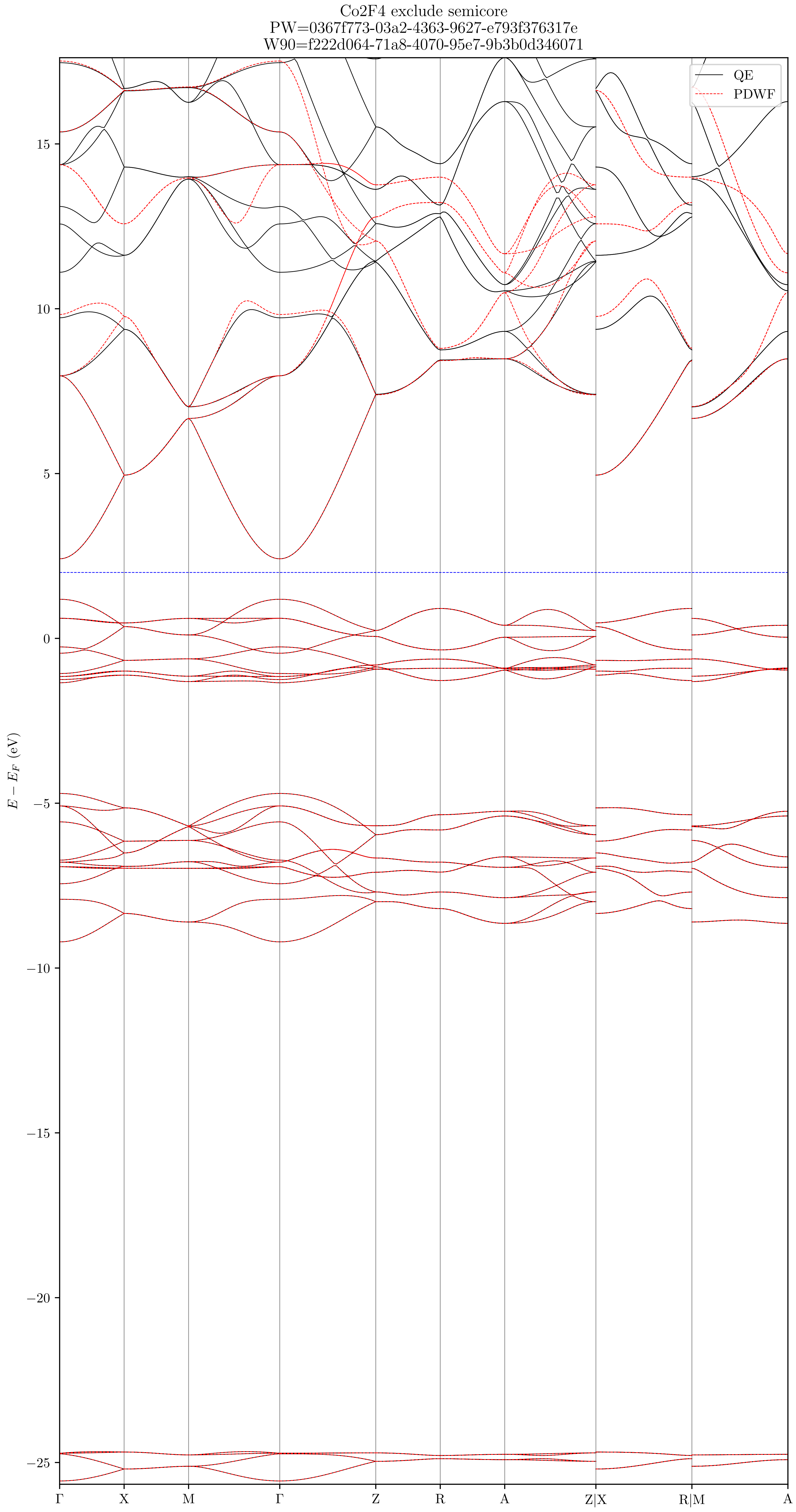

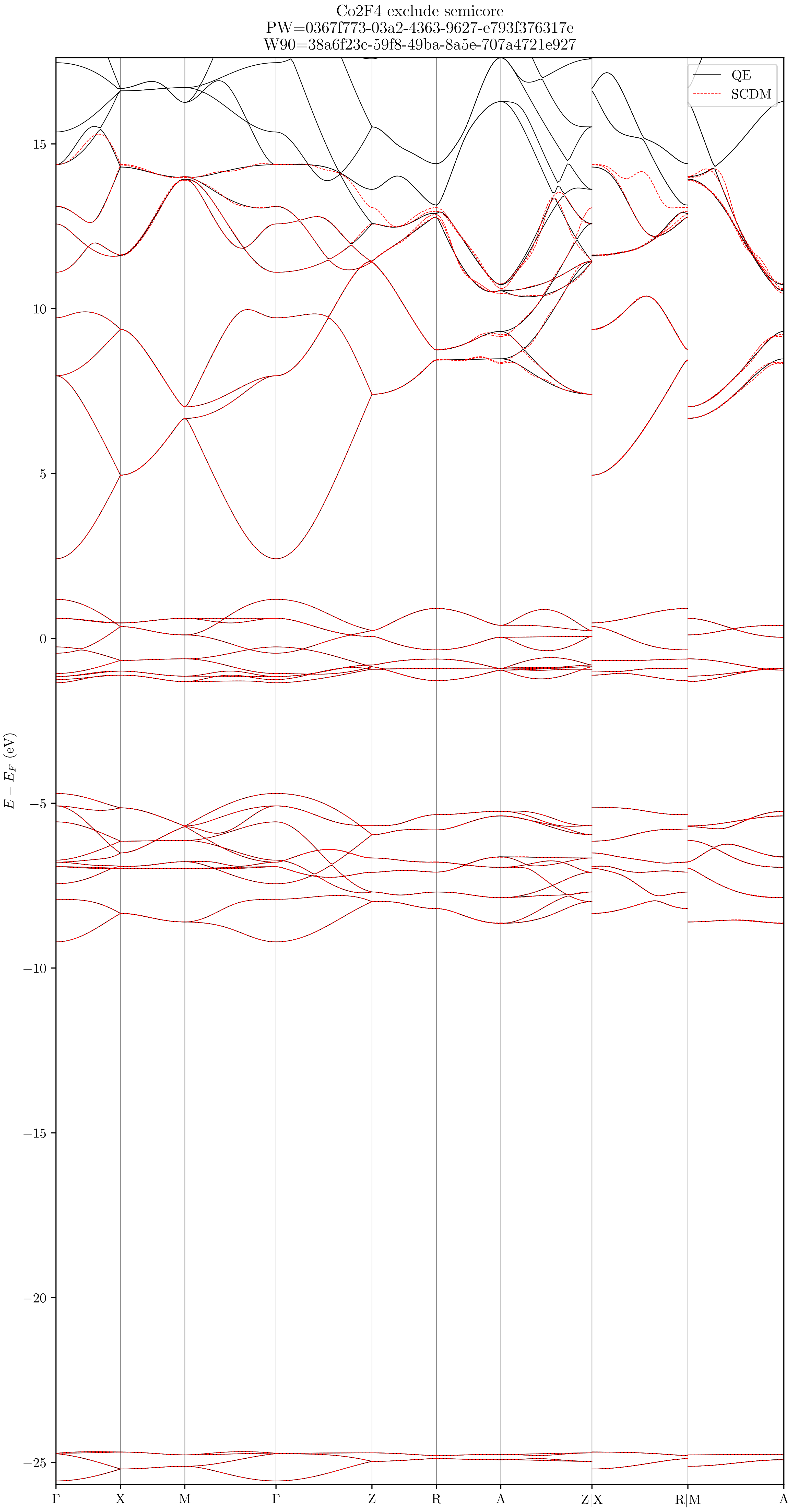

PDWF band distance (meV) and τ ($\AA$)

η_0	η_2	η_4	$\eta_0^{\max}$	$\eta_2^{\max}$	$\eta_4^{\max}$	τ
0.5	0.5	0.5	3.5	3.5	3.5	2.056

SCDM band distance (meV) and τ ($\AA$)

η_0	η_2	η_4	$\eta_0^{\max}$	$\eta_2^{\max}$	$\eta_4^{\max}$	τ
1.8	1.7	1.7	12.4	12.4	12.4	2.374

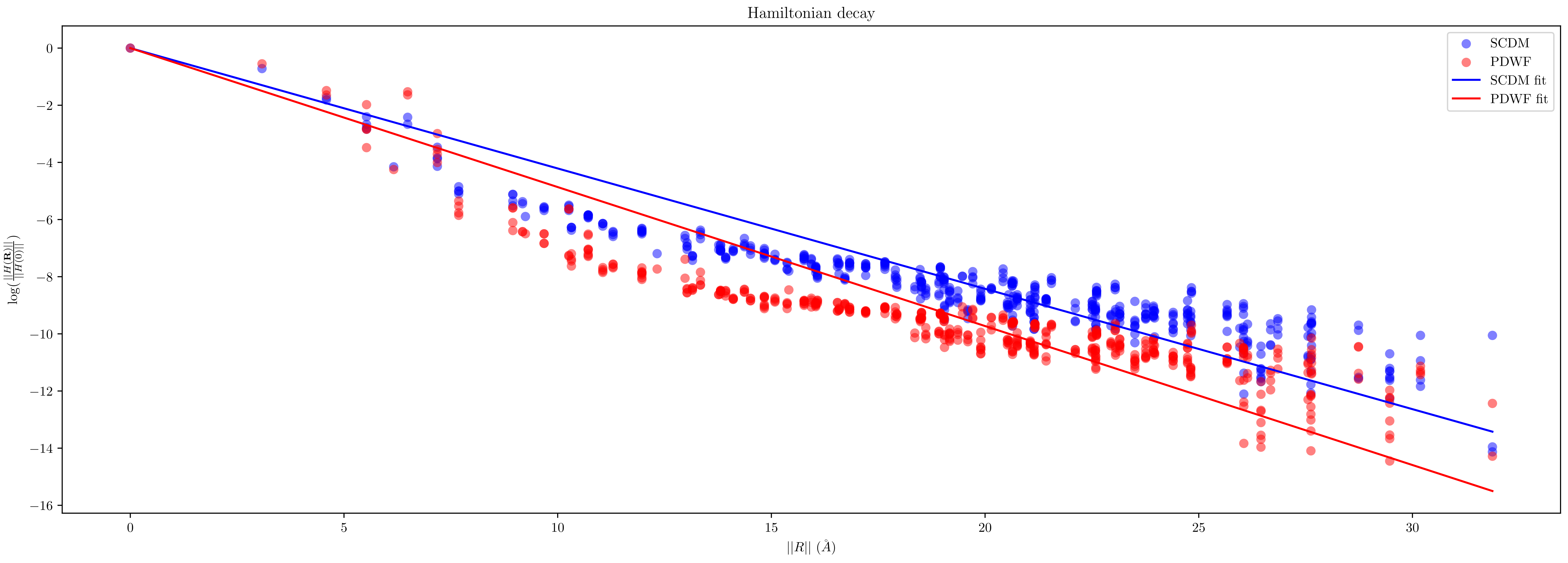

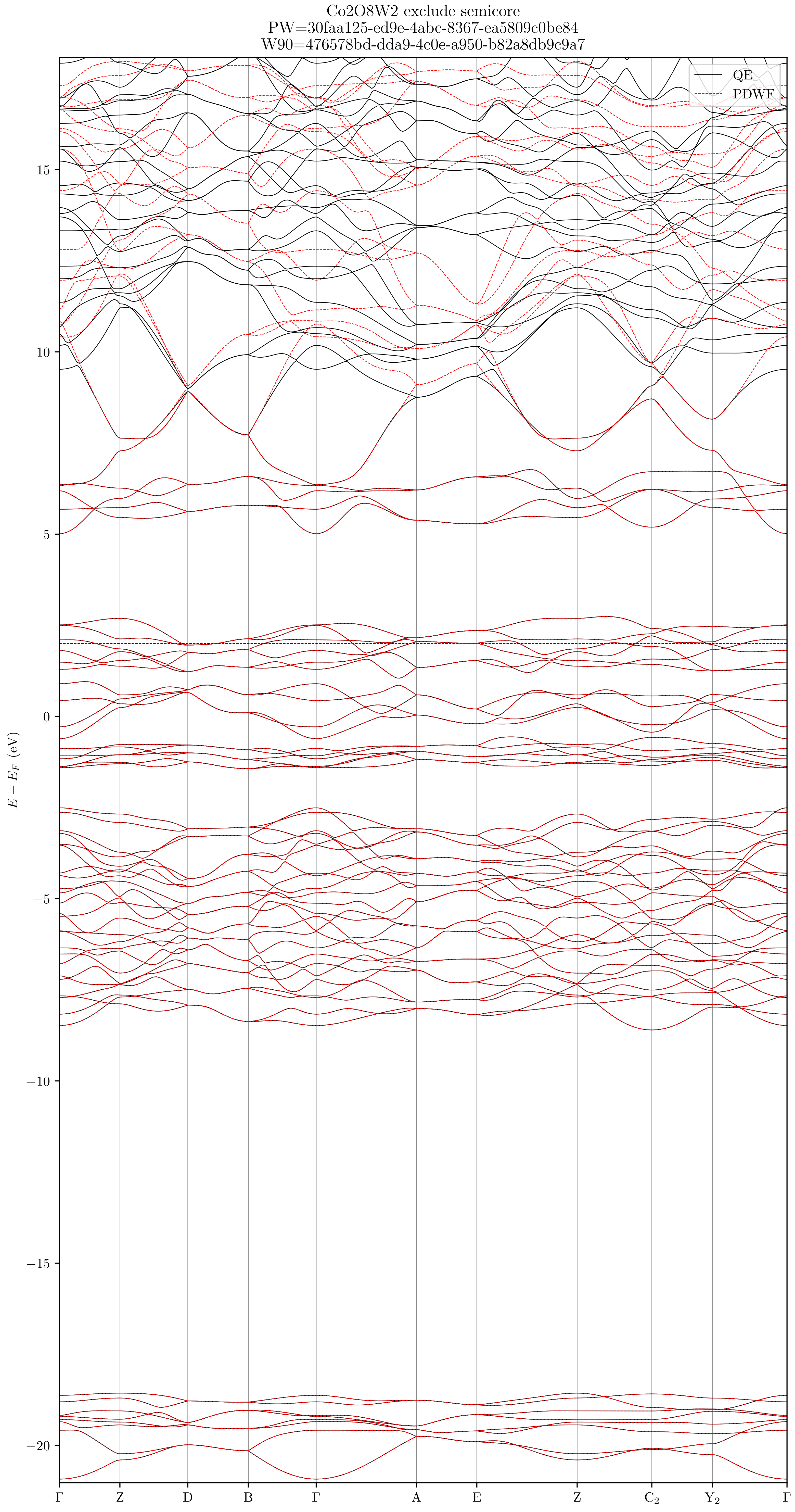

PDWF band distance (meV) and τ ($\AA$)

η_0	η_2	η_4	$\eta_0^{\max}$	$\eta_2^{\max}$	$\eta_4^{\max}$	τ
0.3	0.3	0.3	1.9	1.9	1.9	2.164

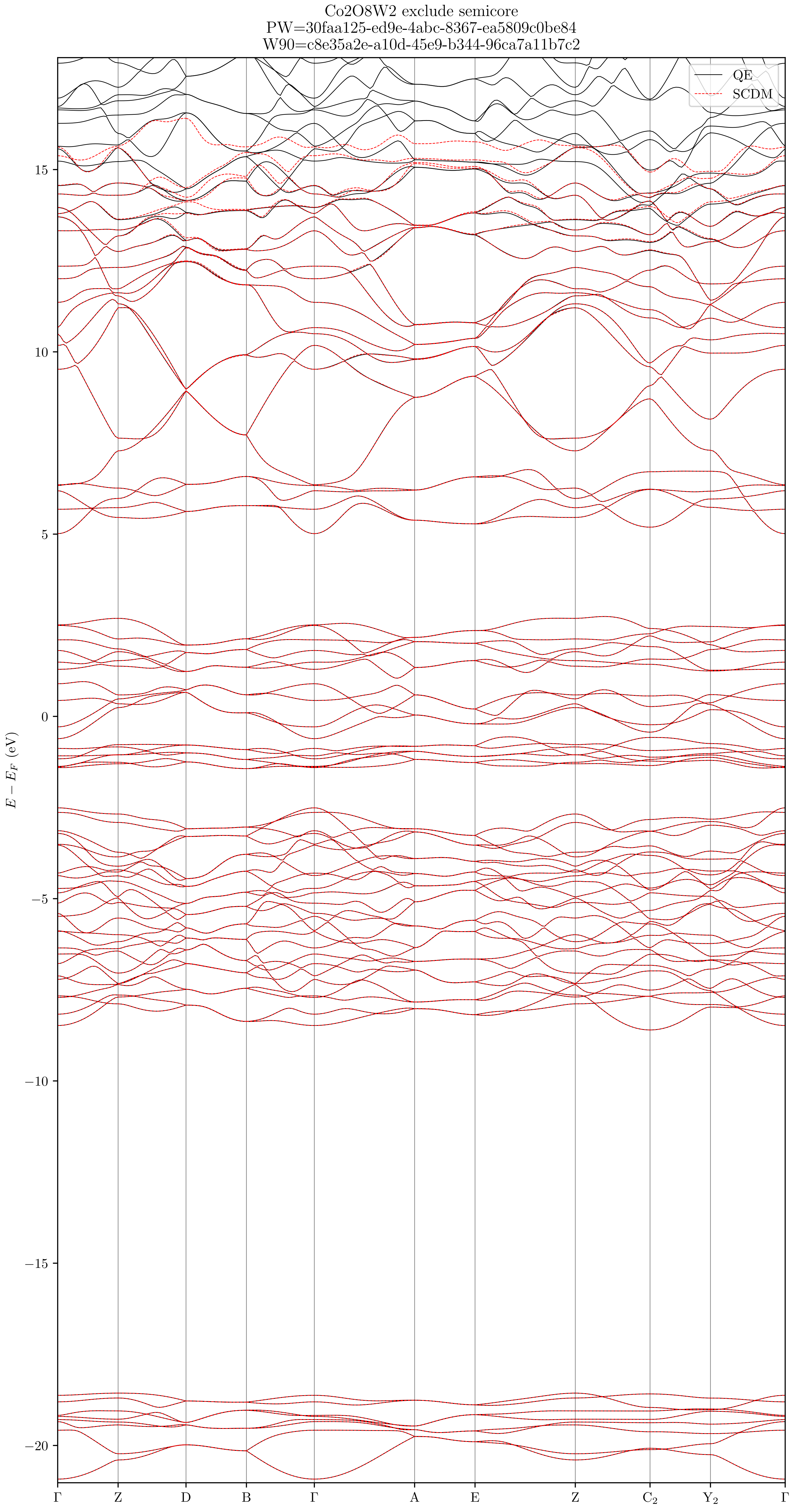

SCDM band distance (meV) and τ ($\AA$)

η_0	η_2	η_4	$\eta_0^{\max}$	$\eta_2^{\max}$	$\eta_4^{\max}$	τ
0.7	0.7	0.7	6.0	6.0	6.0	2.394

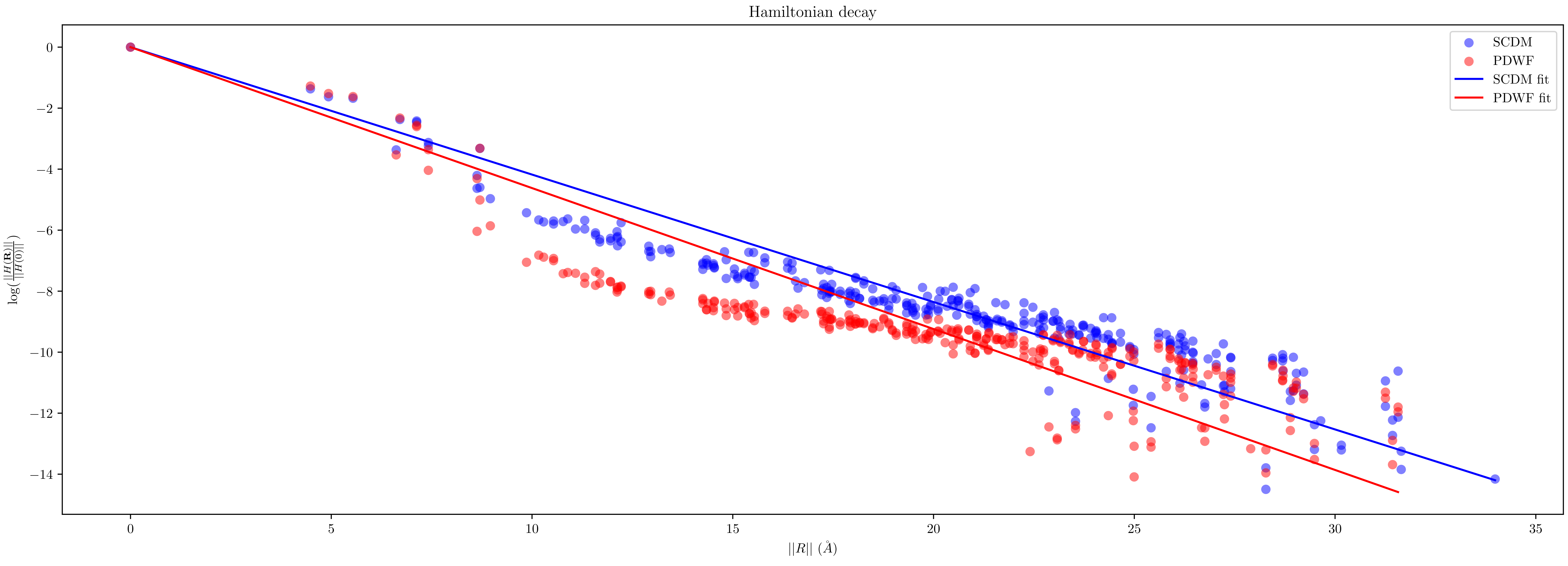

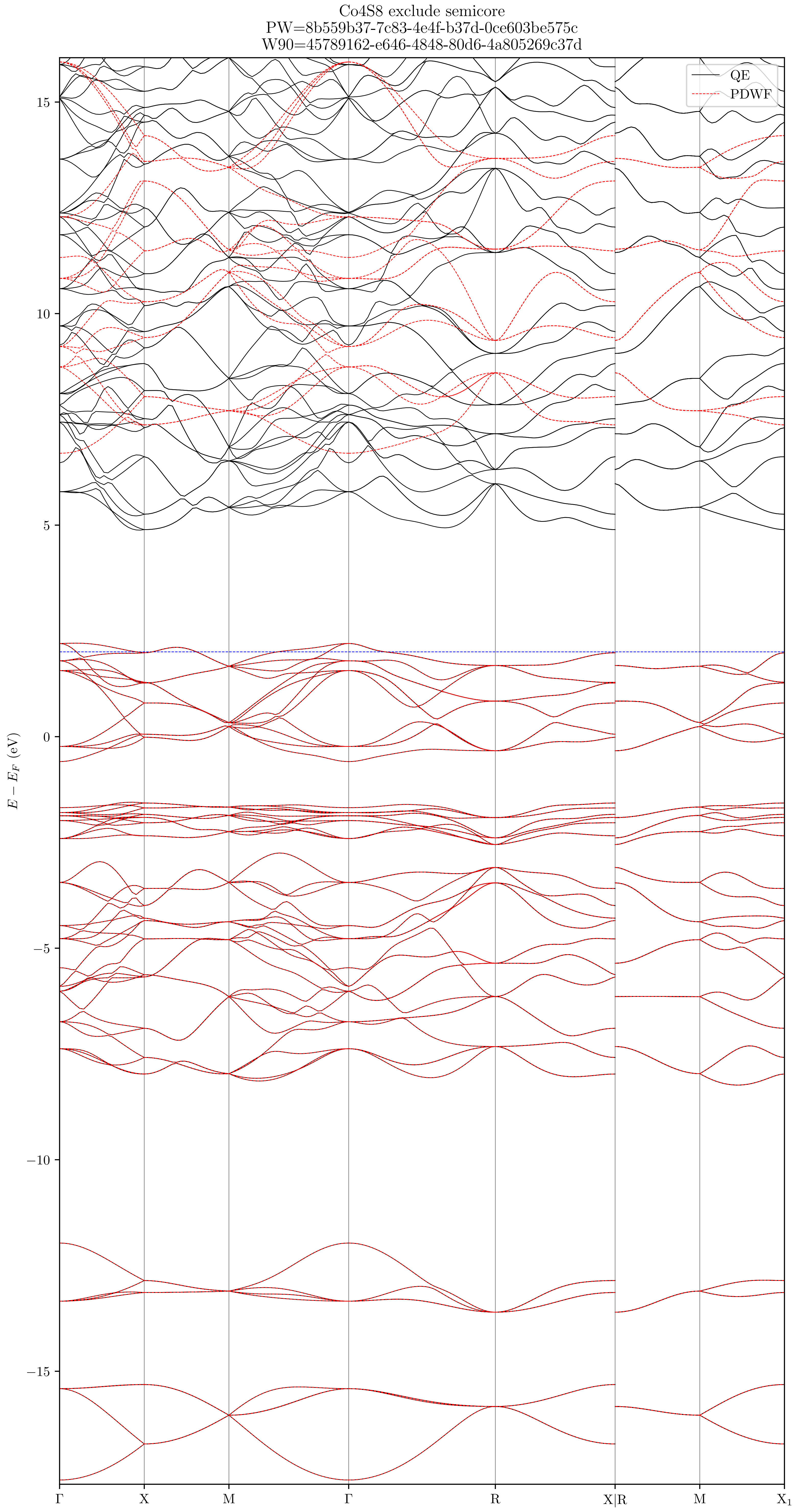

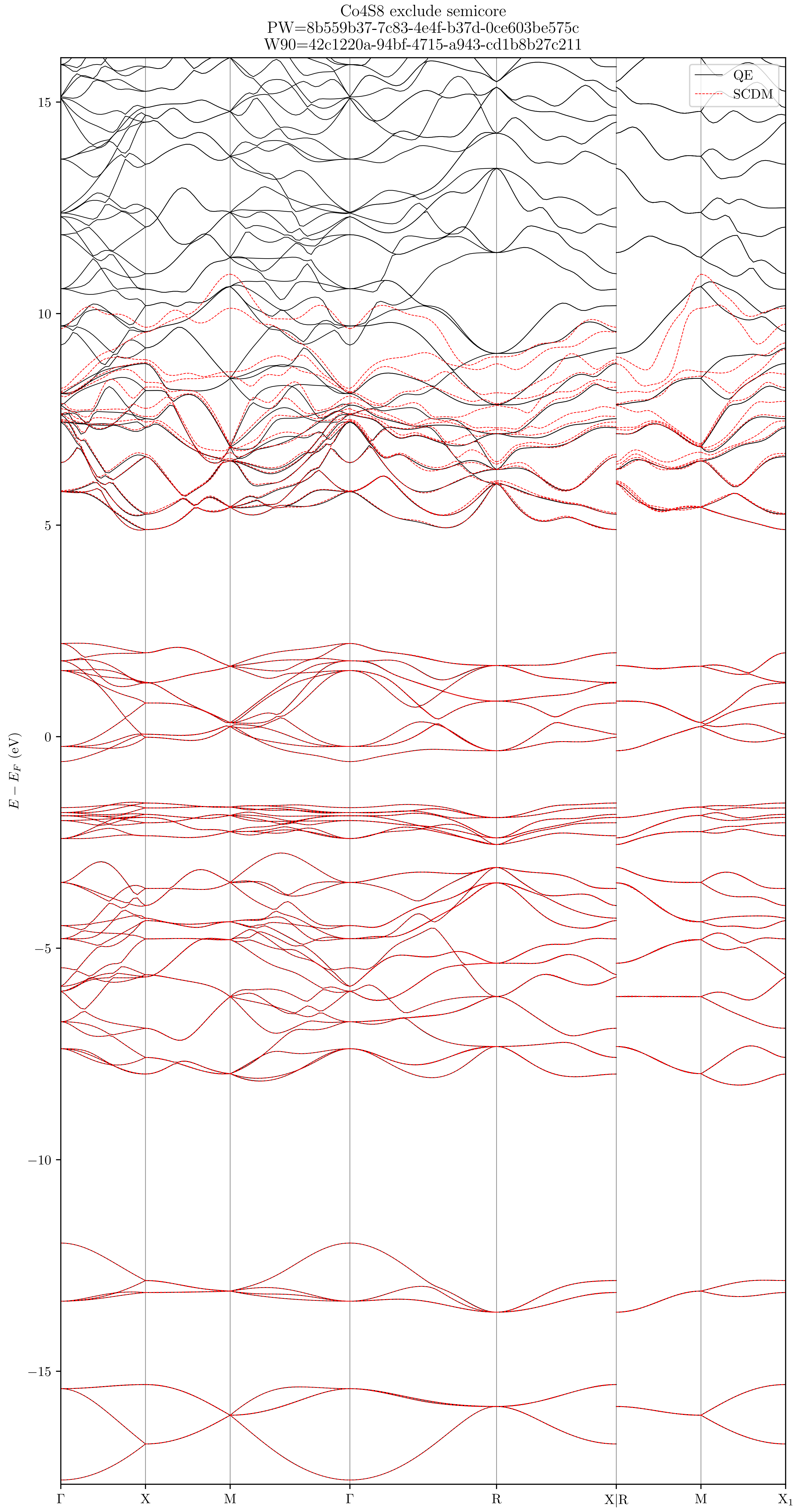

PDWF band distance (meV) and τ (Å)

η_0	η_2	η_4	$\eta_0^{\max}$	$\eta_2^{\max}$	$\eta_4^{\max}$	τ
0.3	0.3	0.4	1.8	4.6	4.7	2.057

SCDM band distance (meV) and τ (Å)

η_0	η_2	η_4	$\eta_0^{\max}$	$\eta_2^{\max}$	$\eta_4^{\max}$	τ
2.5	2.6	2.6	18.4	18.4	18.4	2.768

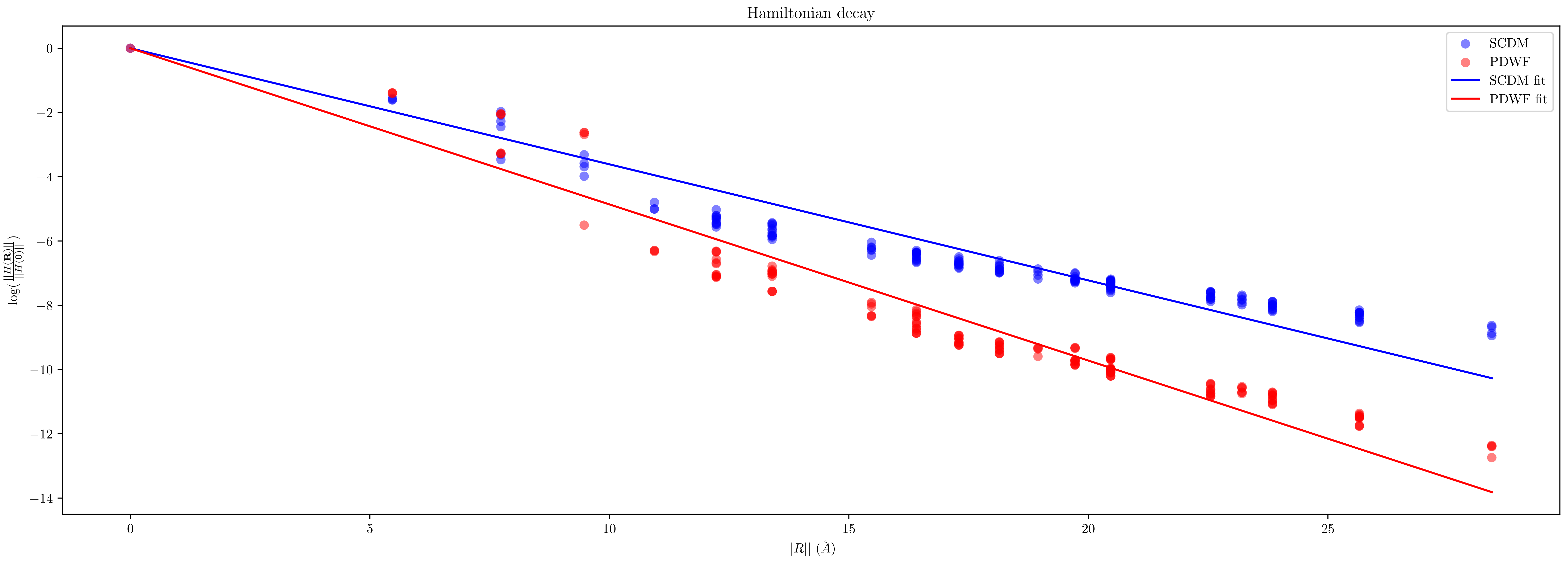

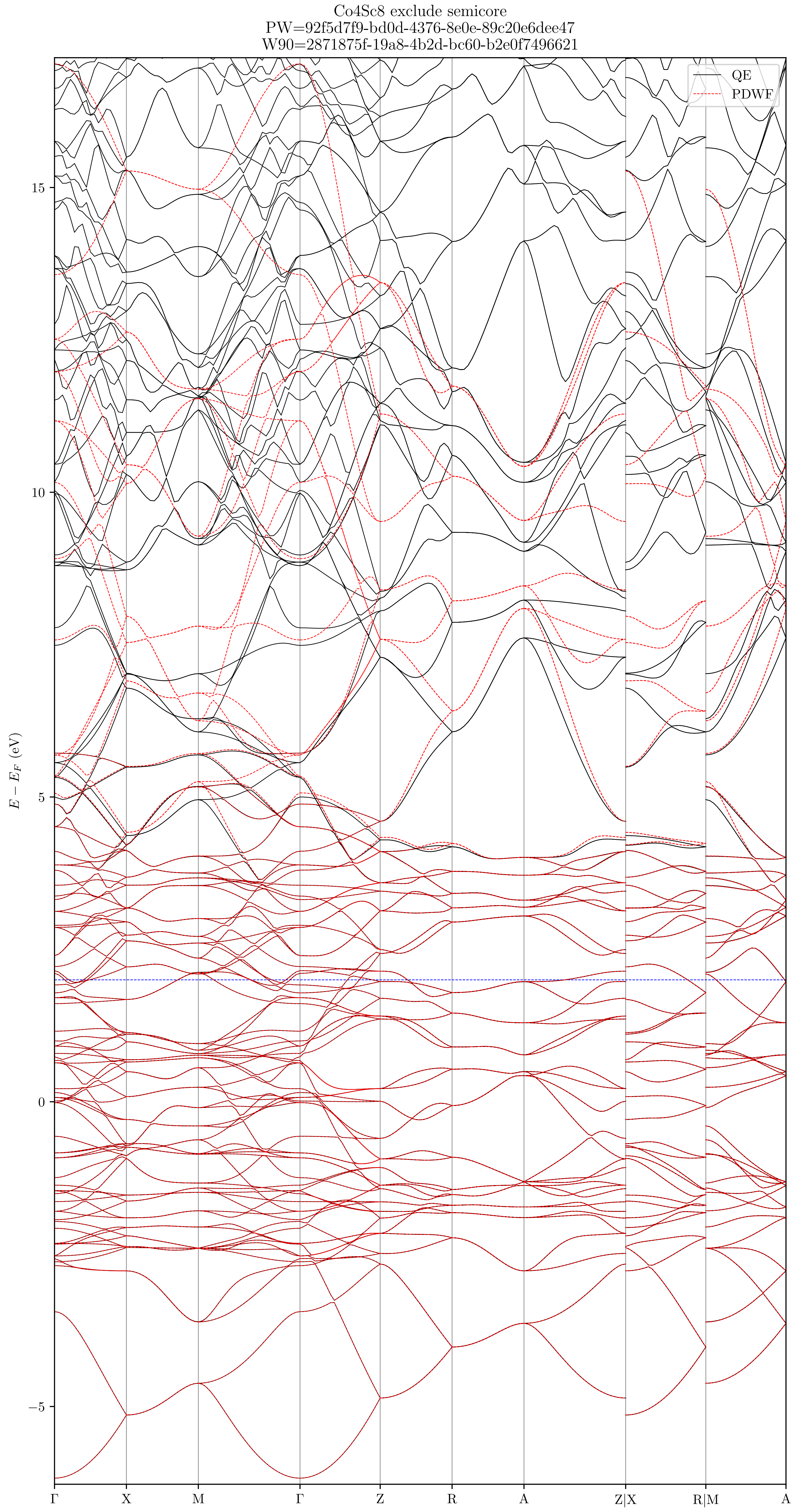

PDWF band distance (meV) and τ ($\AA$)

η_0	η_2	η_4	$\eta_0^{\max}$	$\eta_2^{\max}$	$\eta_4^{\max}$	τ
0.5	0.6	2.1	5.1	5.1	19.6	2.231

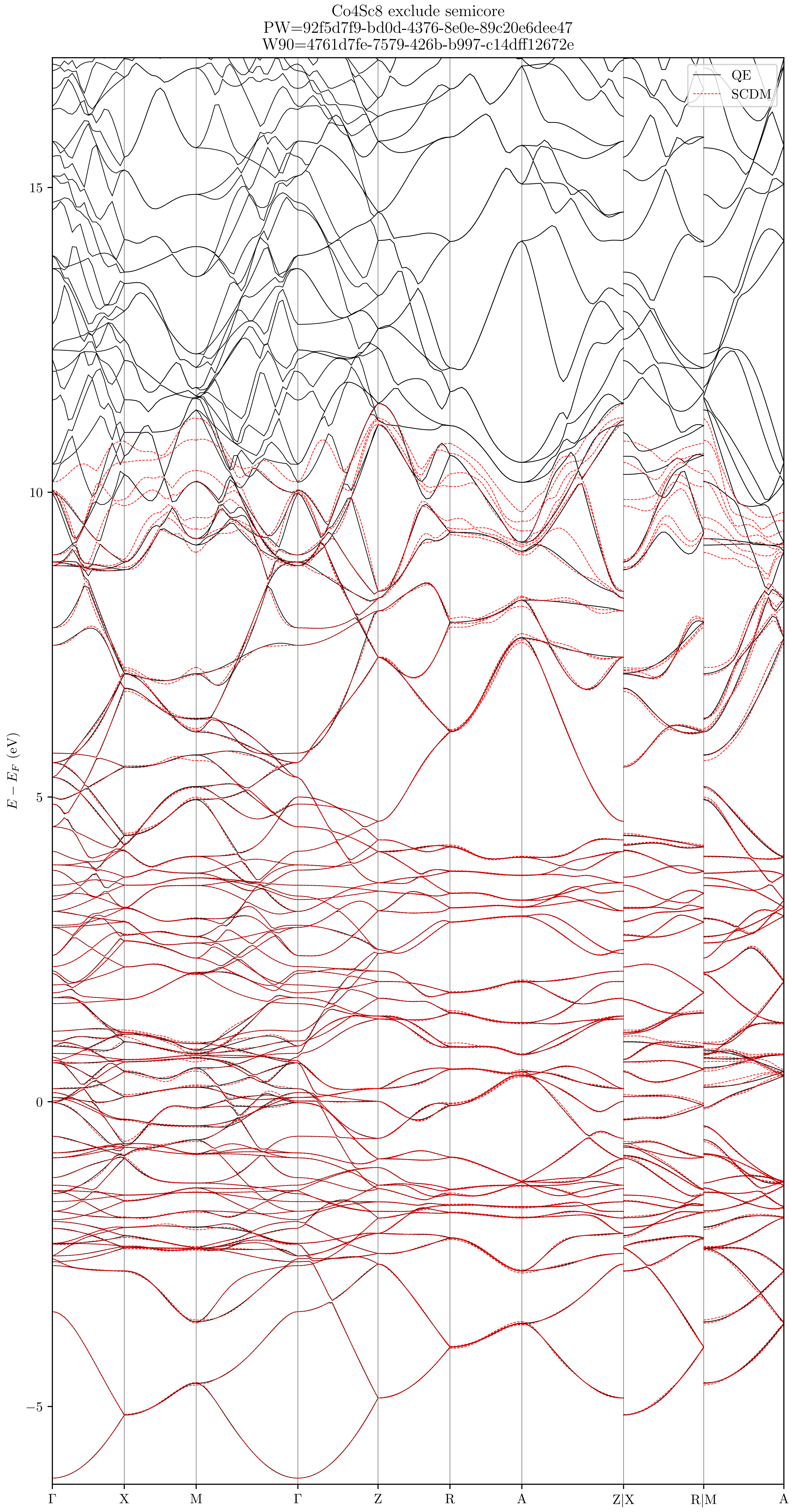

SCDM band distance (meV) and τ ($\AA$)

η_0	η_2	η_4	$\eta_0^{\max}$	$\eta_2^{\max}$	$\eta_4^{\max}$	τ
9.2	12.3	10.8	67.6	102.3	102.4	3.017

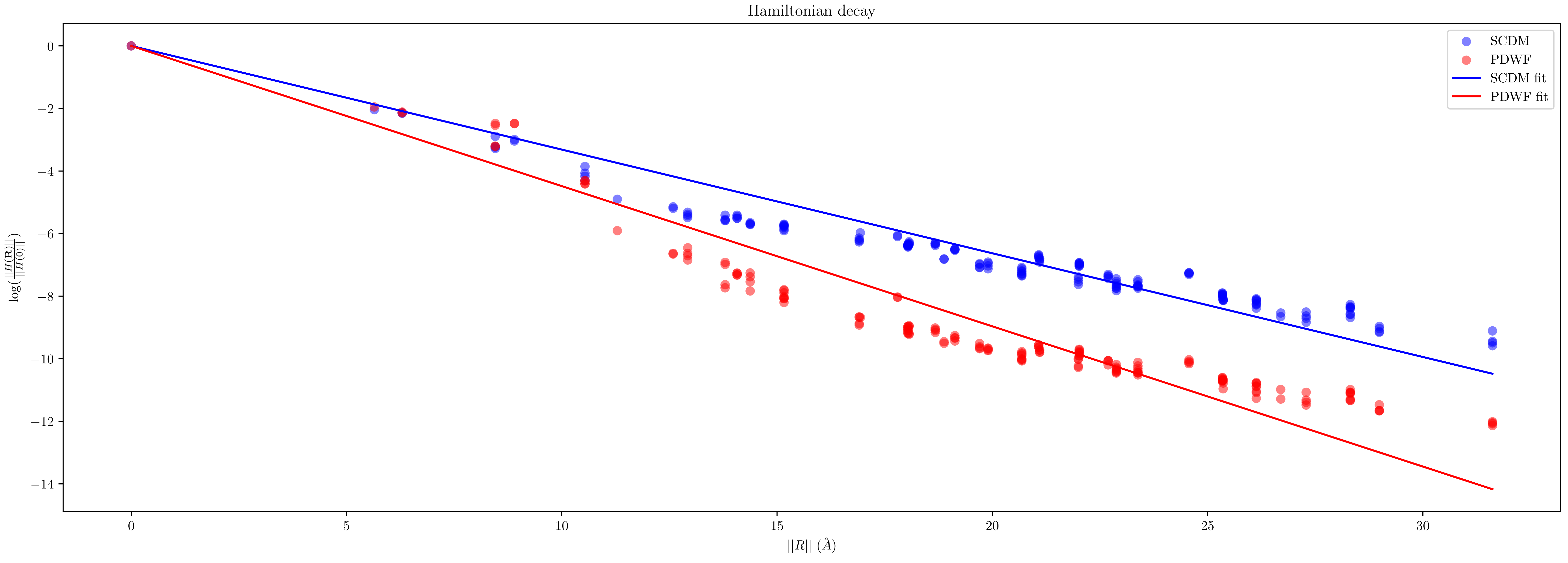

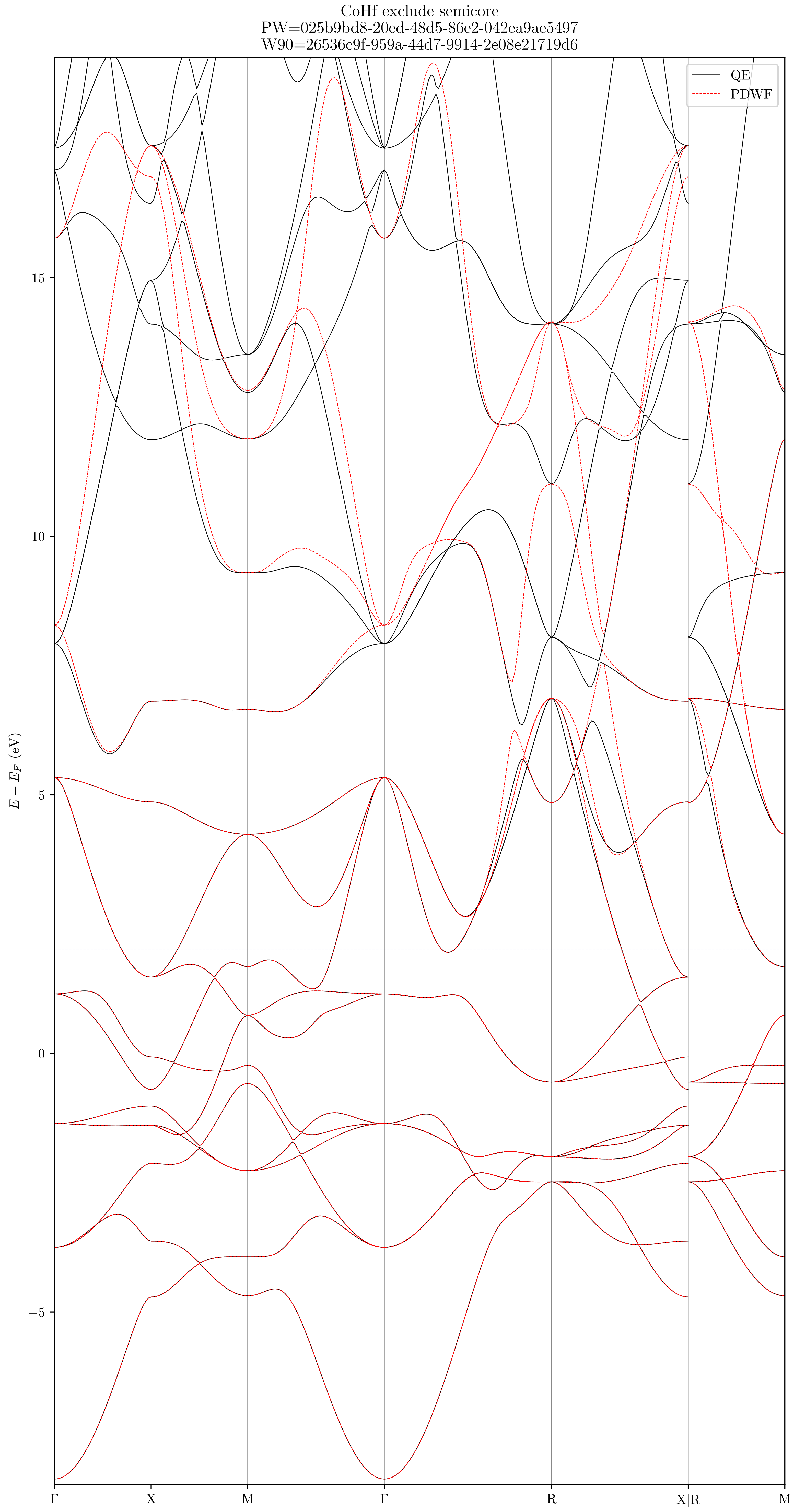

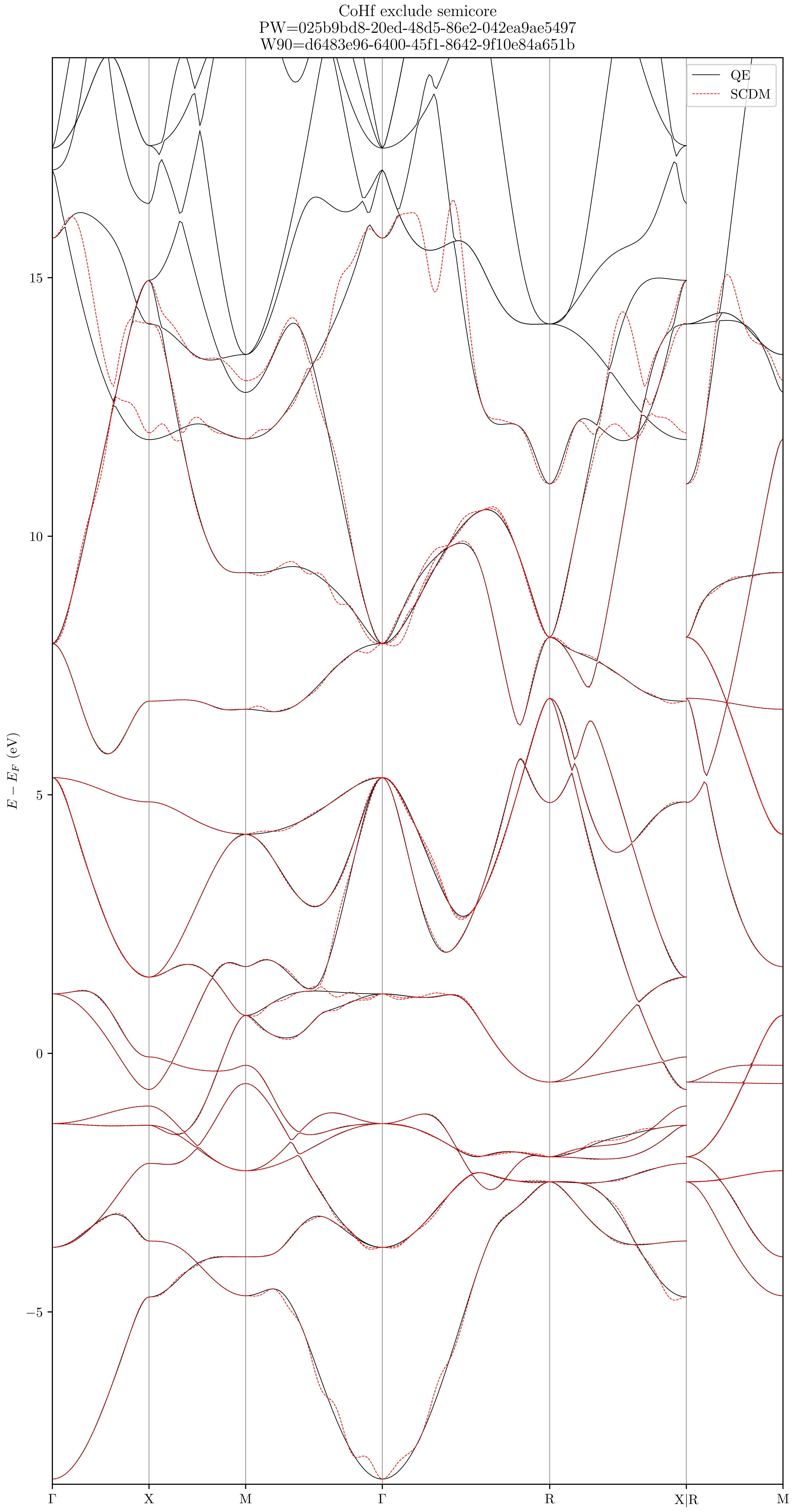

PDWF band distance (meV) and τ ($\AA$)

η_0	η_2	η_4	$\eta_0^{\max}$	$\eta_2^{\max}$	$\eta_4^{\max}$	τ
1.4	1.6	9.6	9.8	10.2	110.1	1.835

SCDM band distance (meV) and τ ($\AA$)

η_0	η_2	η_4	$\eta_0^{\max}$	$\eta_2^{\max}$	$\eta_4^{\max}$	τ
29.3	28.3	30.2	290.4	290.4	290.4	2.494

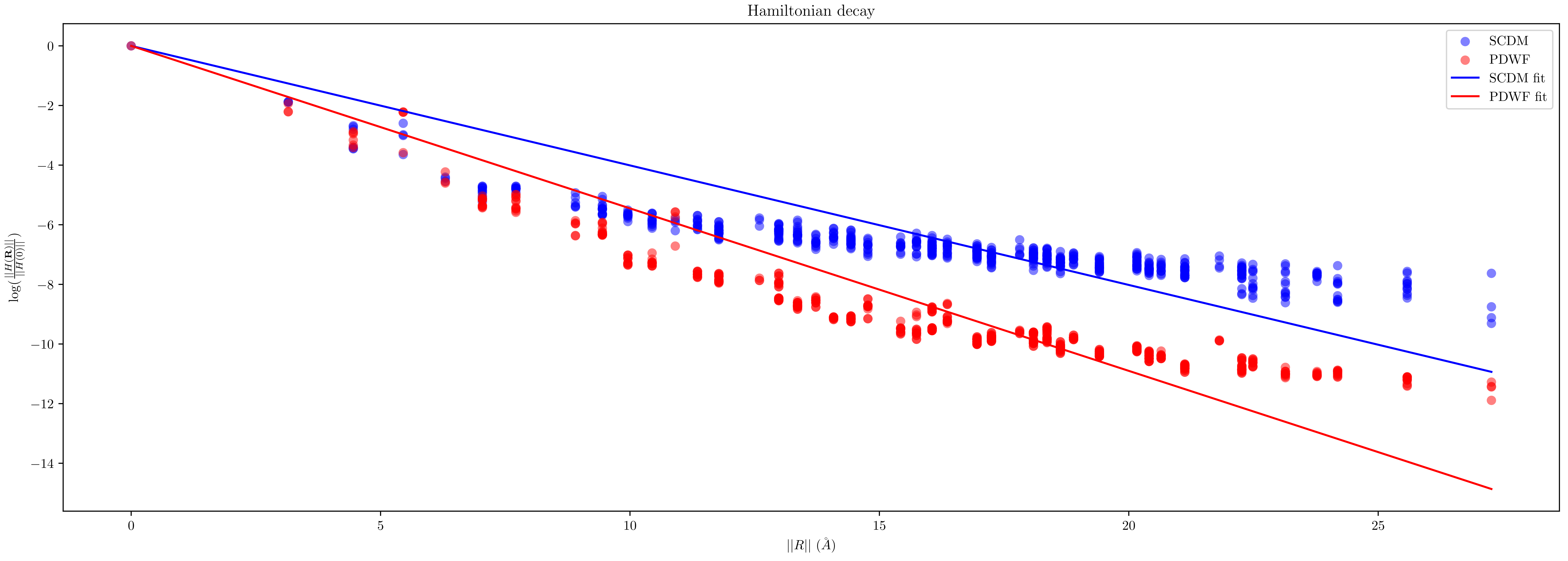

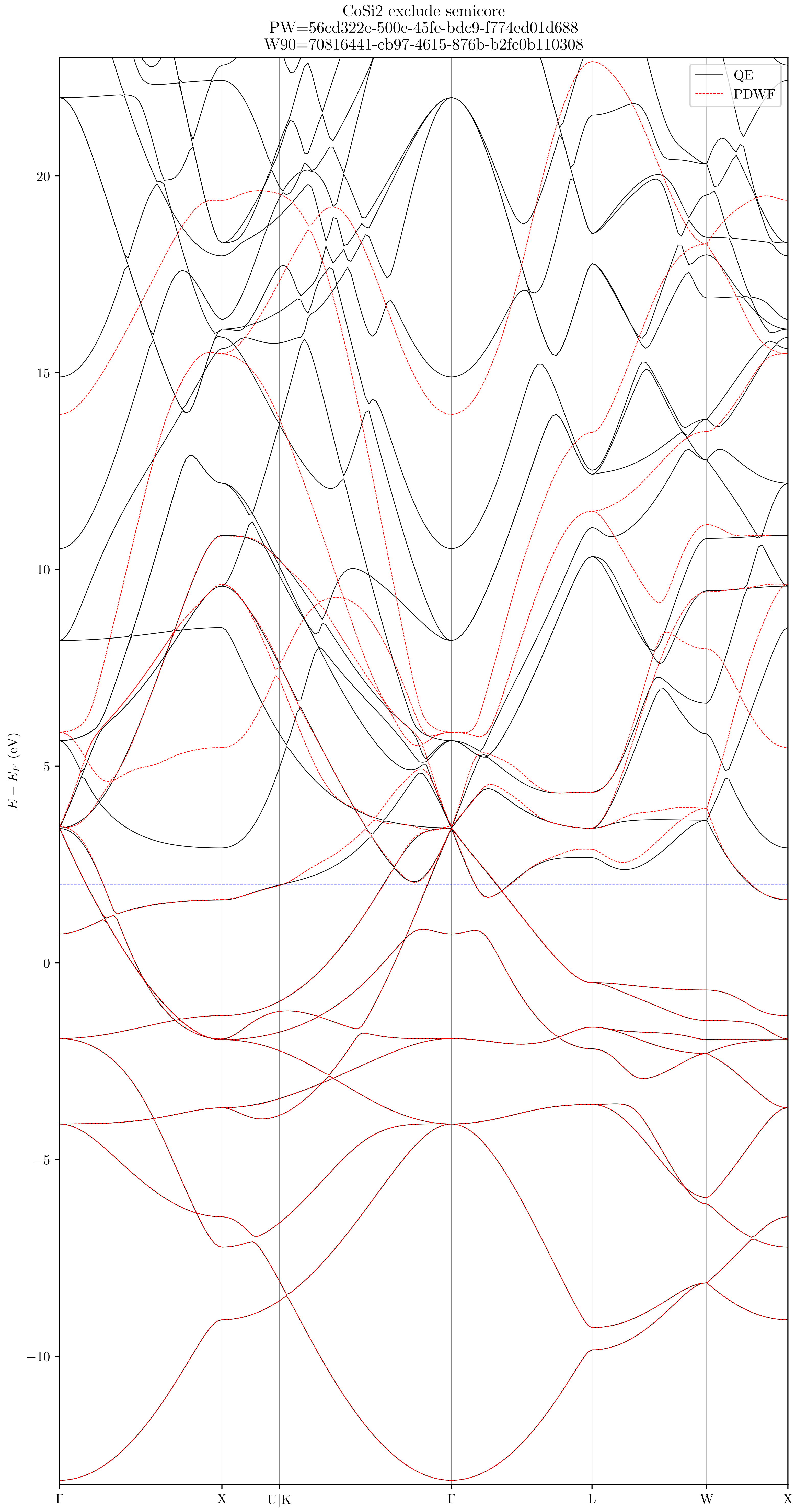

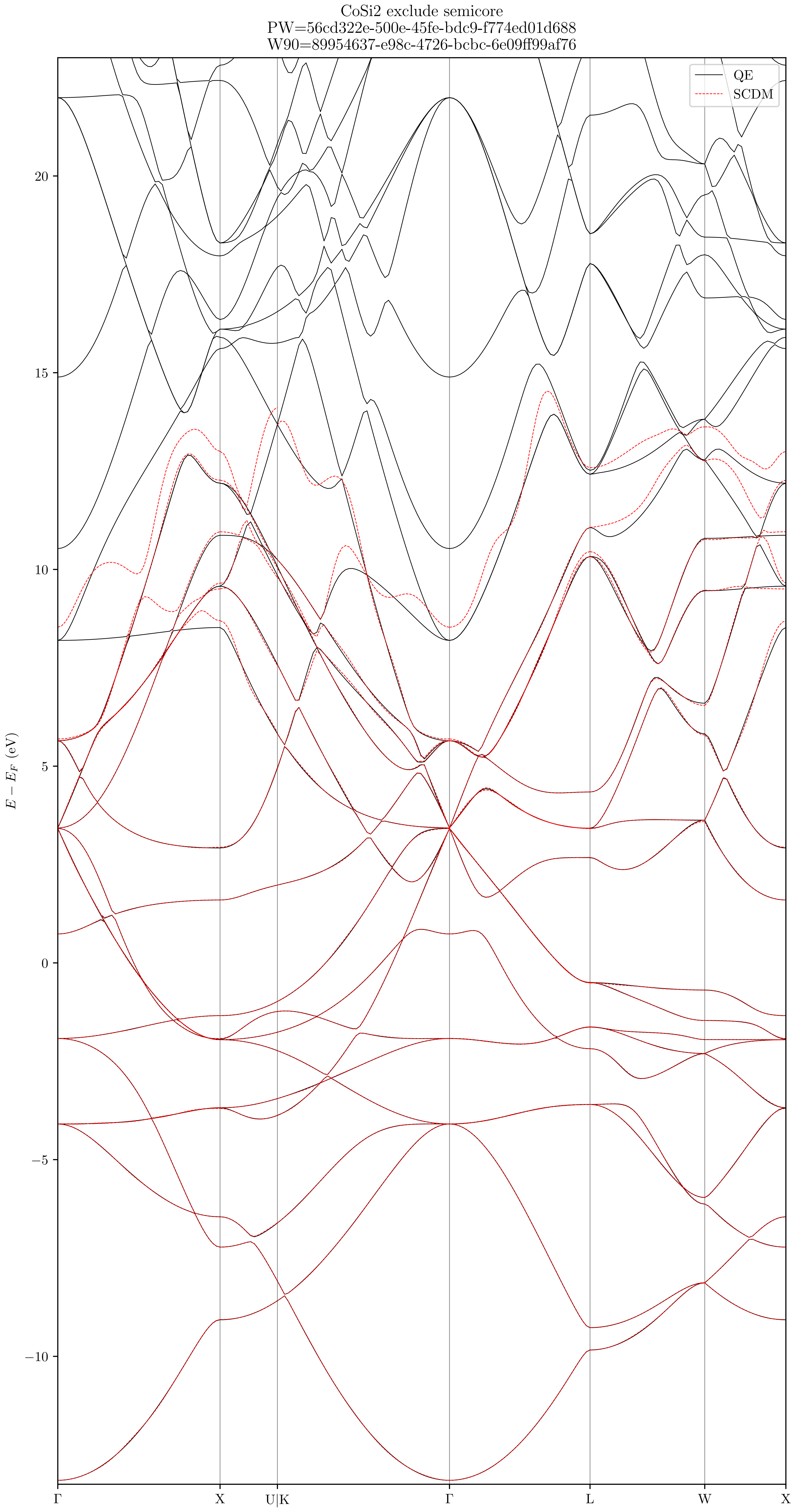

PDWF band distance (meV) and τ ($\AA$)

η_0	η_2	η_4	$\eta_0^{\max}$	$\eta_2^{\max}$	$\eta_4^{\max}$	τ
2.7	5.0	58.1	13.4	25.0	384.0	2.396

SCDM band distance (meV) and τ ($\AA$)

η_0	η_2	η_4	$\eta_0^{\max}$	$\eta_2^{\max}$	$\eta_4^{\max}$	τ
4.6	4.8	6.1	19.1	22.6	35.9	2.603

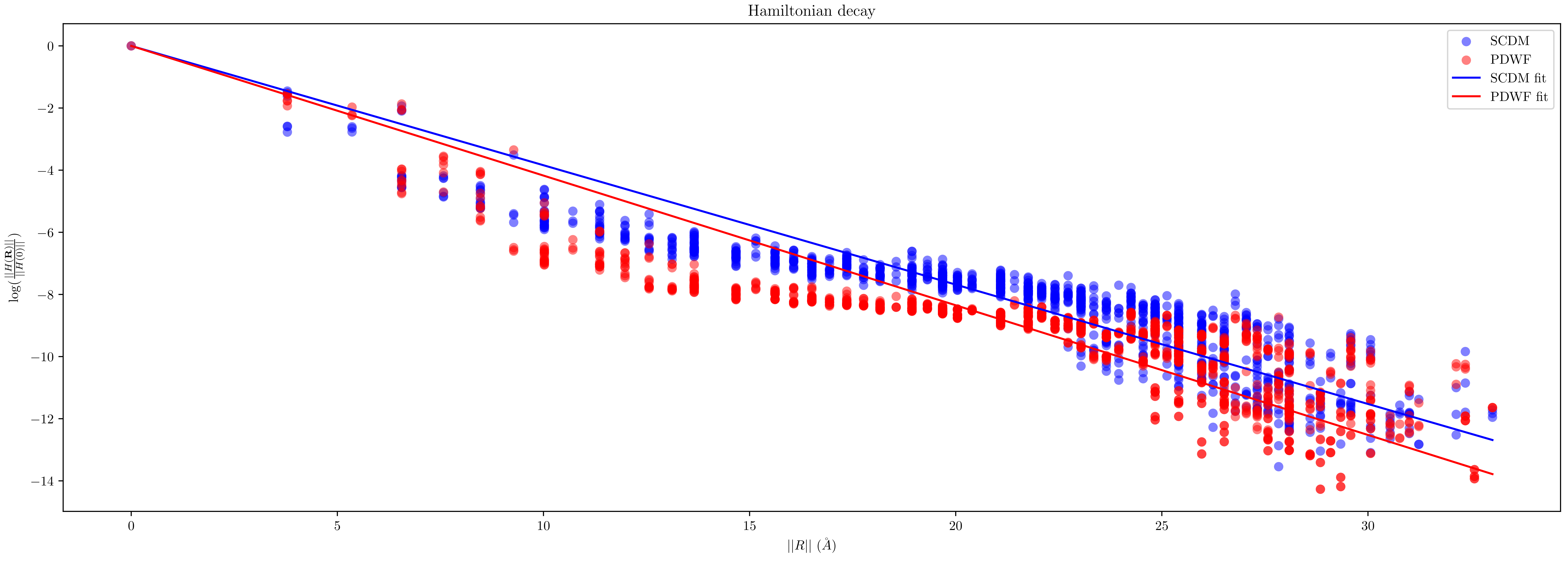

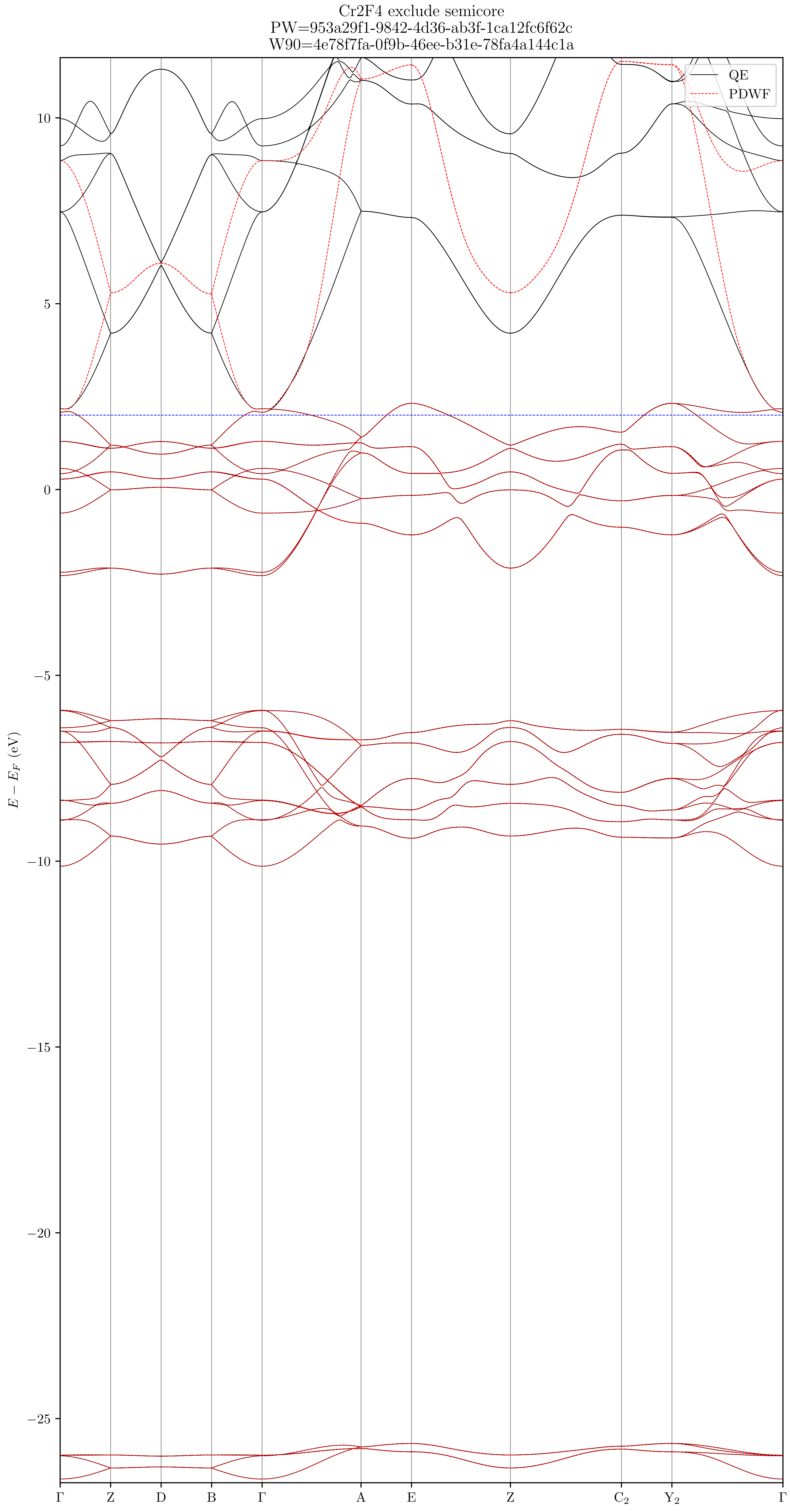

PDWF band distance (meV) and τ ($\AA$)

η_0	η_2	η_4	$\eta_0^{\max}$	$\eta_2^{\max}$	$\eta_4^{\max}$	τ
0.1	0.2	17.4	0.6	2.1	515.4	1.868

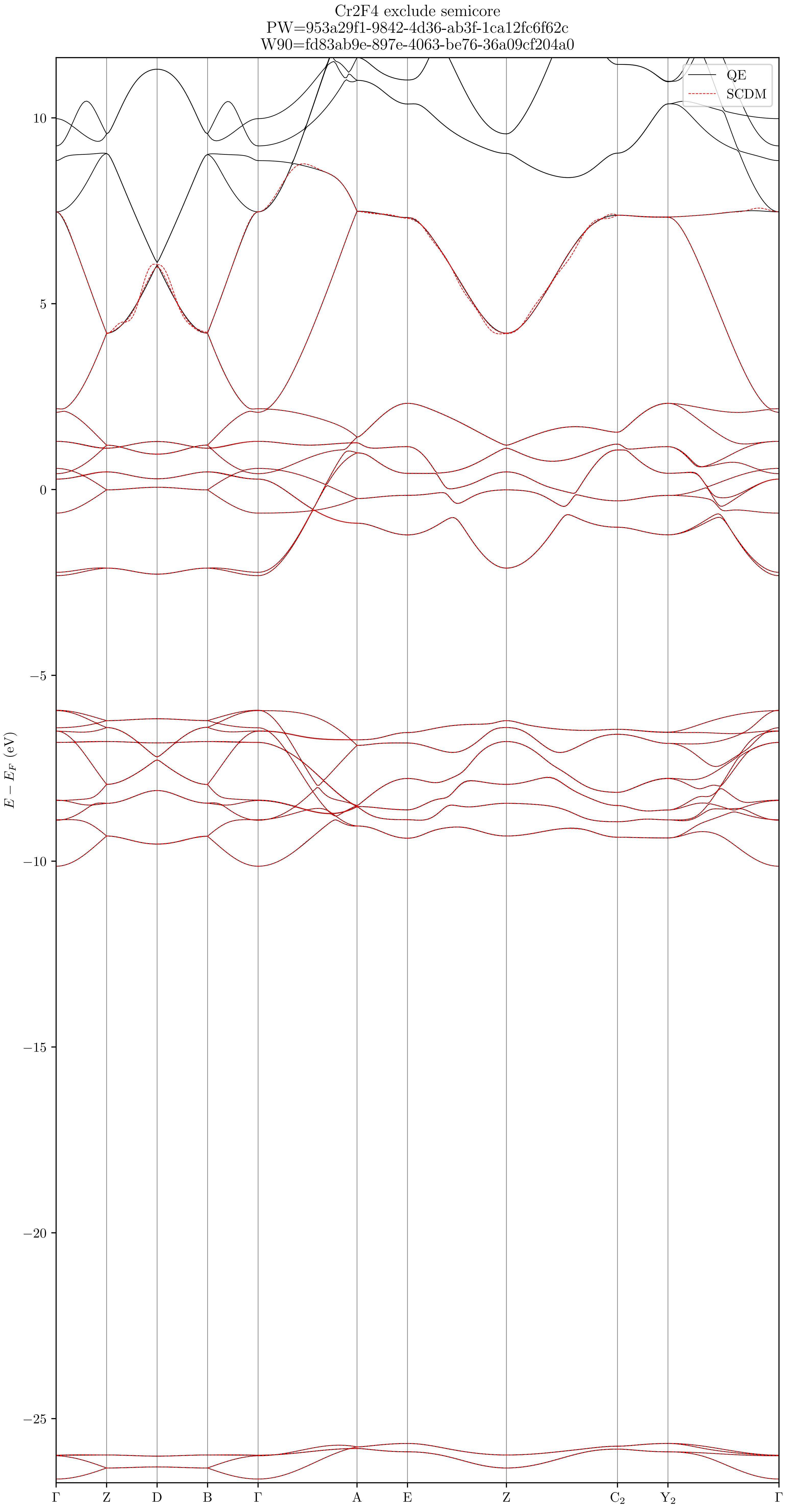

SCDM band distance (meV) and τ ($\AA$)

η_0	η_2	η_4	$\eta_0^{\max}$	$\eta_2^{\max}$	$\eta_4^{\max}$	τ
1.1	1.1	1.4	11.9	11.9	13.0	2.354

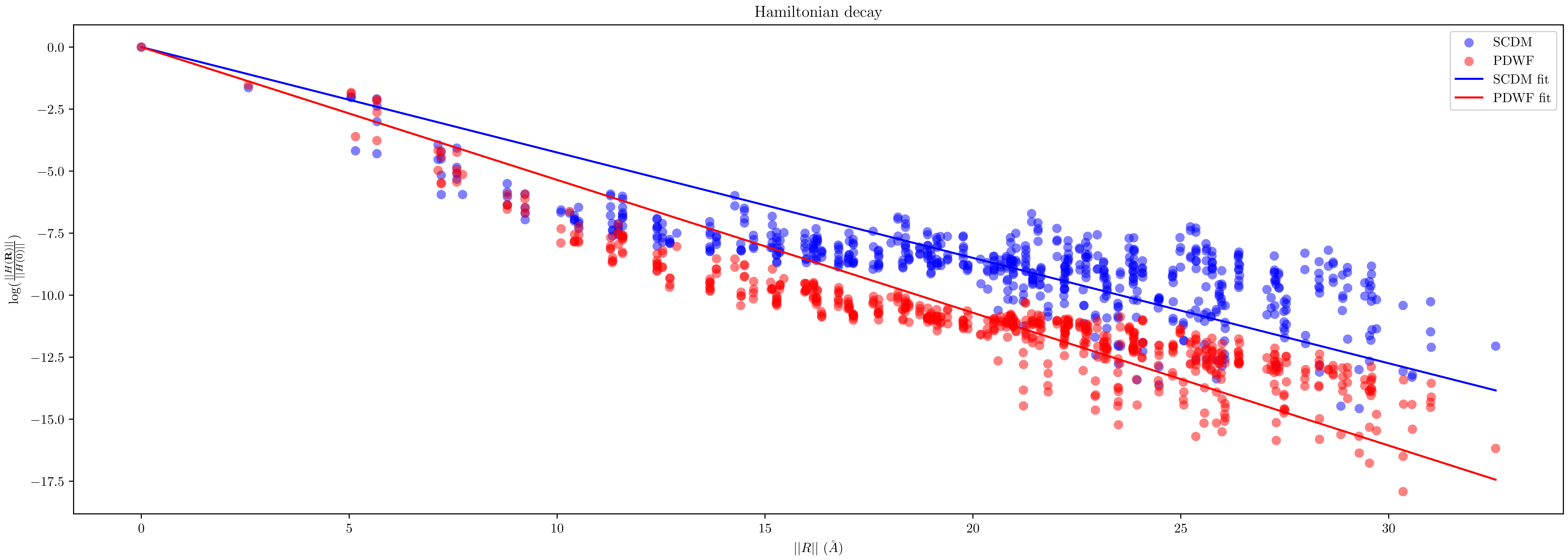

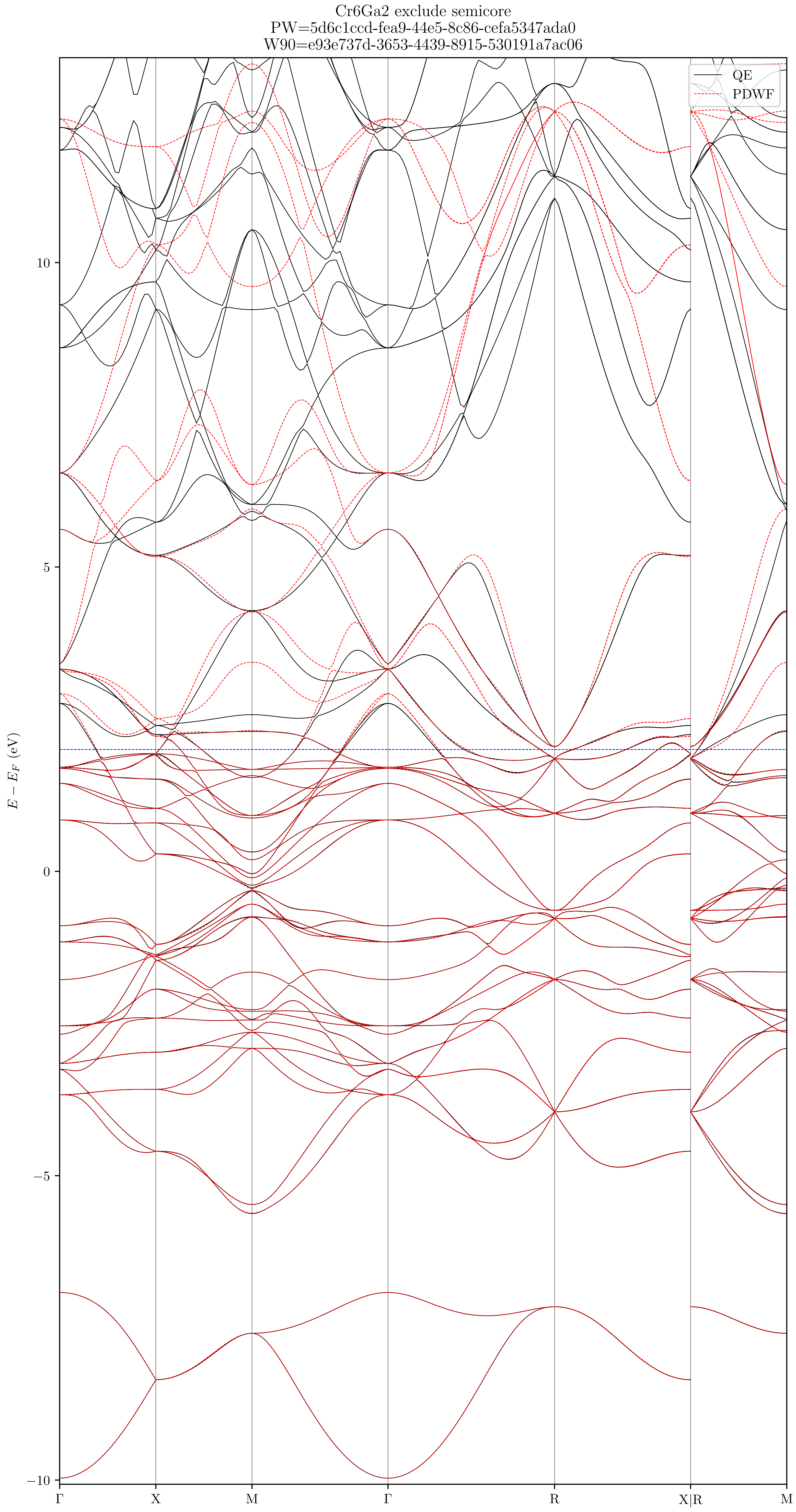

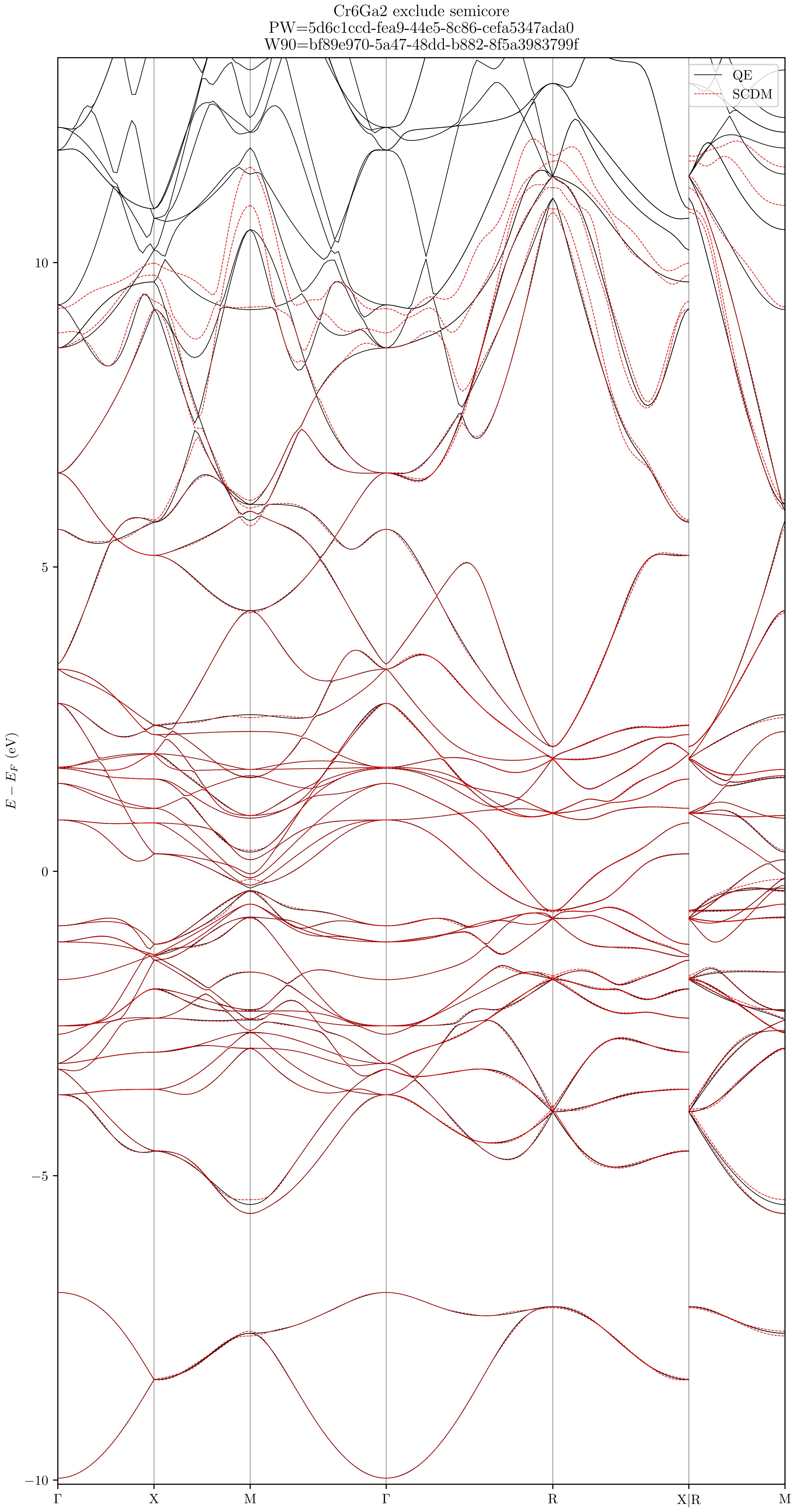

PDWF band distance (meV) and τ (Å)

η_0	η_2	η_4	$\eta_0^{\max}$	$\eta_2^{\max}$	$\eta_4^{\max}$	τ
2.3	3.5	65.3	17.5	18.5	859.6	2.253

SCDM band distance (meV) and τ (Å)

η_0	η_2	η_4	$\eta_0^{\max}$	$\eta_2^{\max}$	$\eta_4^{\max}$	τ
13.6	11.5	11.4	122.4	140.3	140.3	2.560

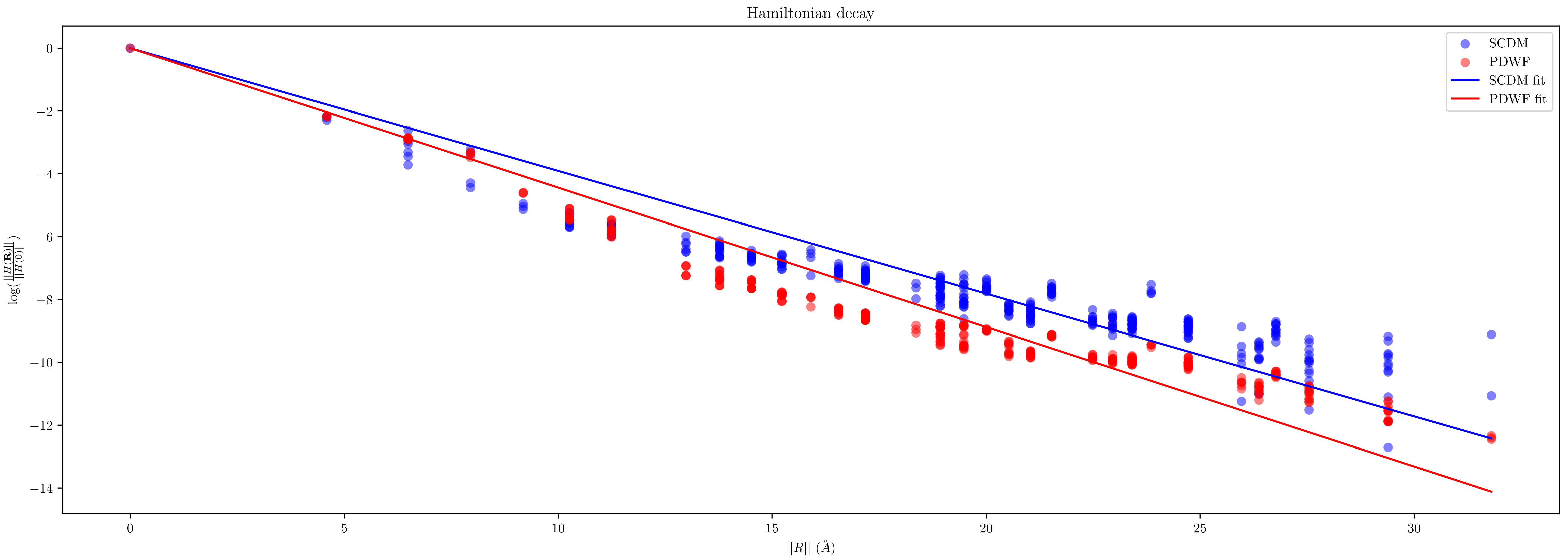

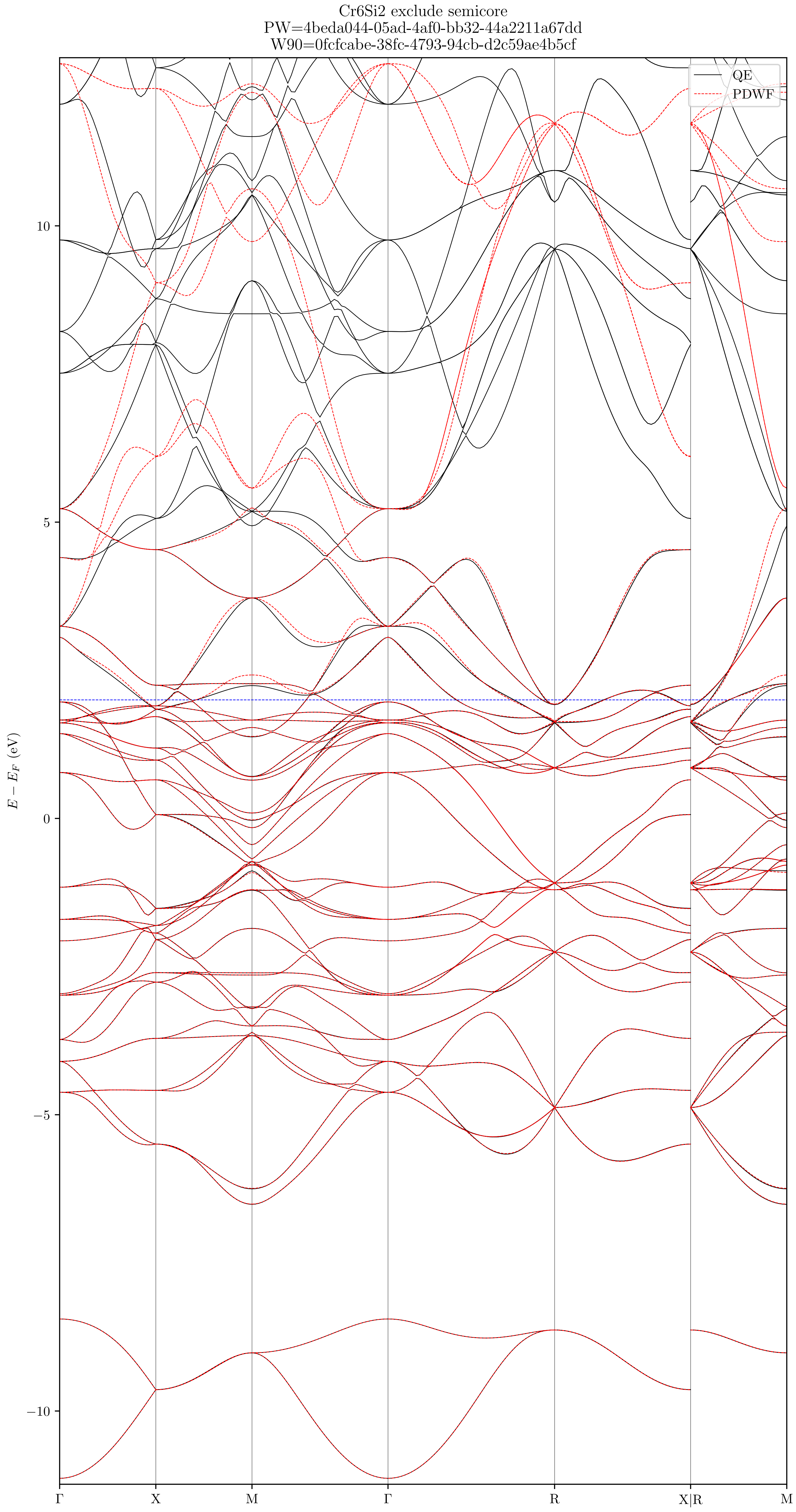

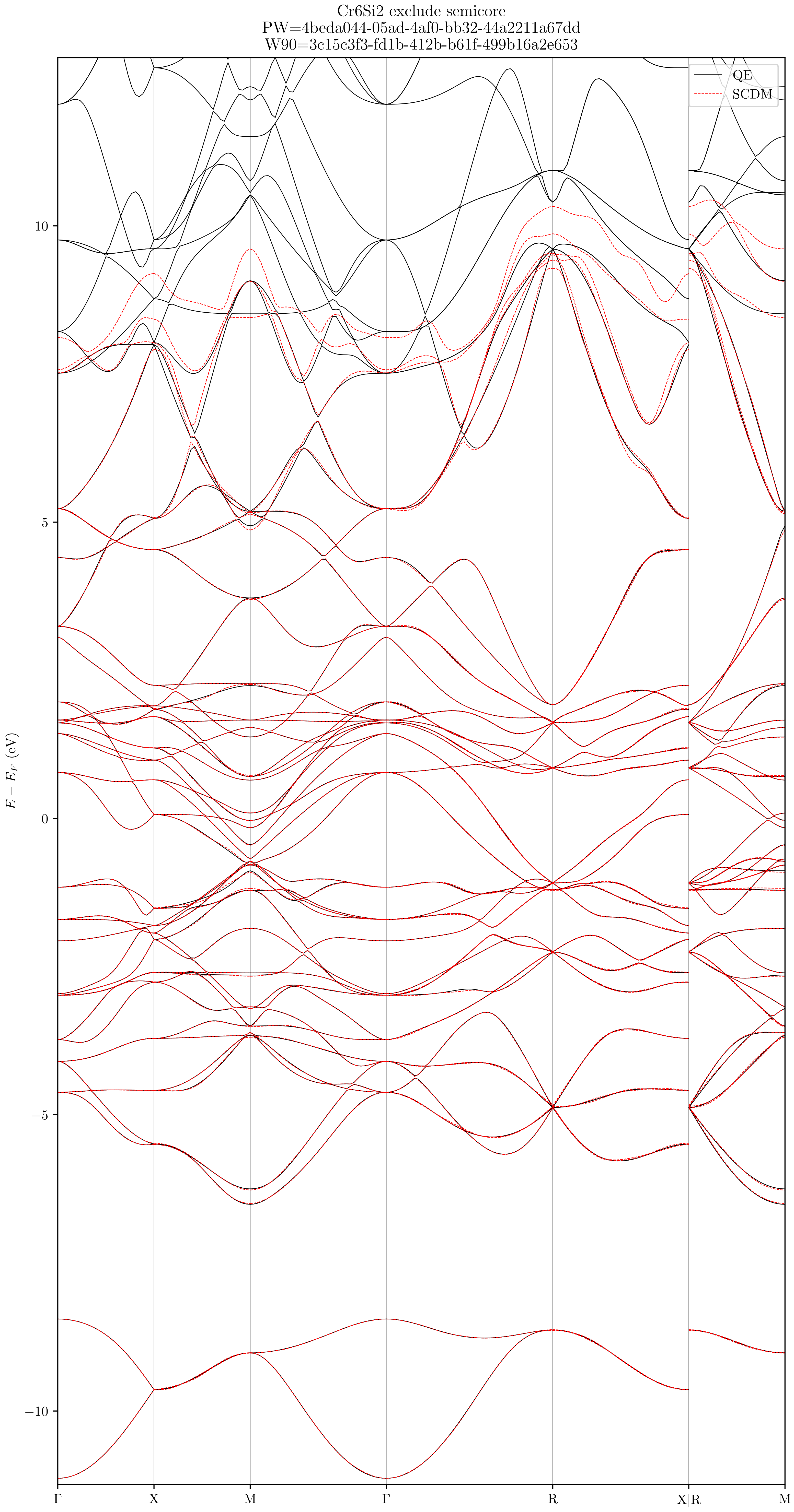

PDWF band distance (meV) and τ ($\AA$)

η_0	η_2	η_4	$\eta_0^{\max}$	$\eta_2^{\max}$	$\eta_4^{\max}$	τ
2.7	4.3	17.2	27.3	35.2	319.2	2.236

SCDM band distance (meV) and τ ($\AA$)

η_0	η_2	η_4	$\eta_0^{\max}$	$\eta_2^{\max}$	$\eta_4^{\max}$	τ
6.8	5.9	6.1	42.0	42.0	42.0	2.475

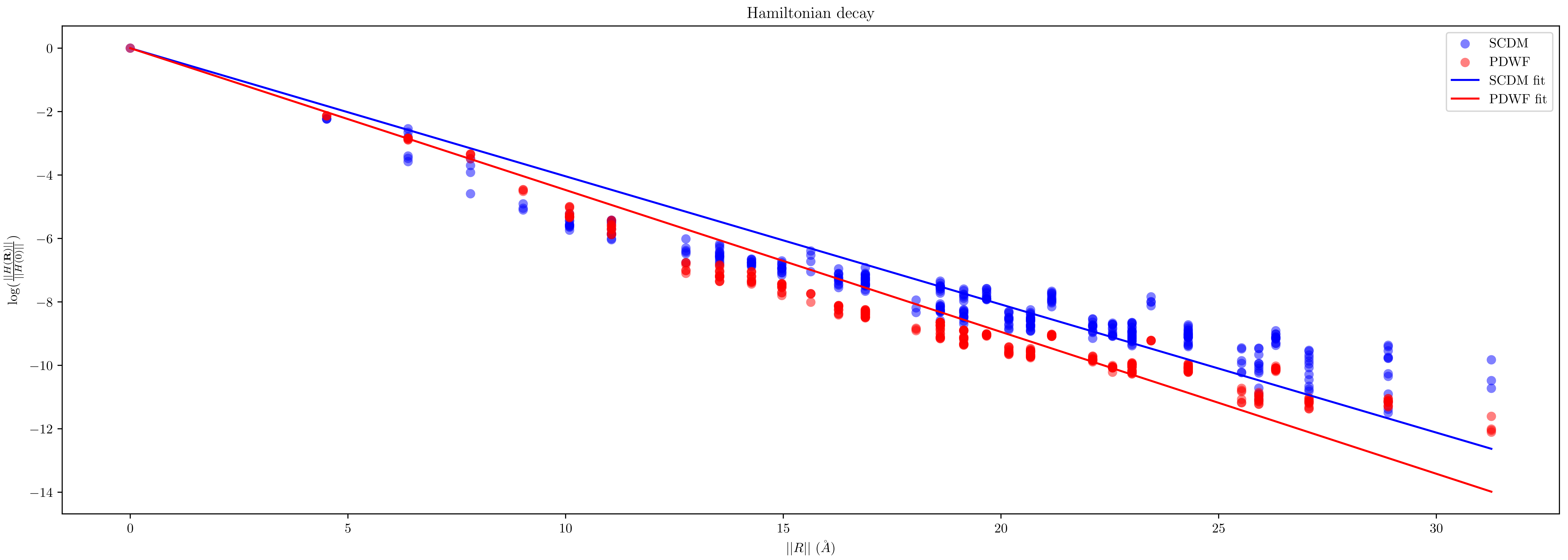

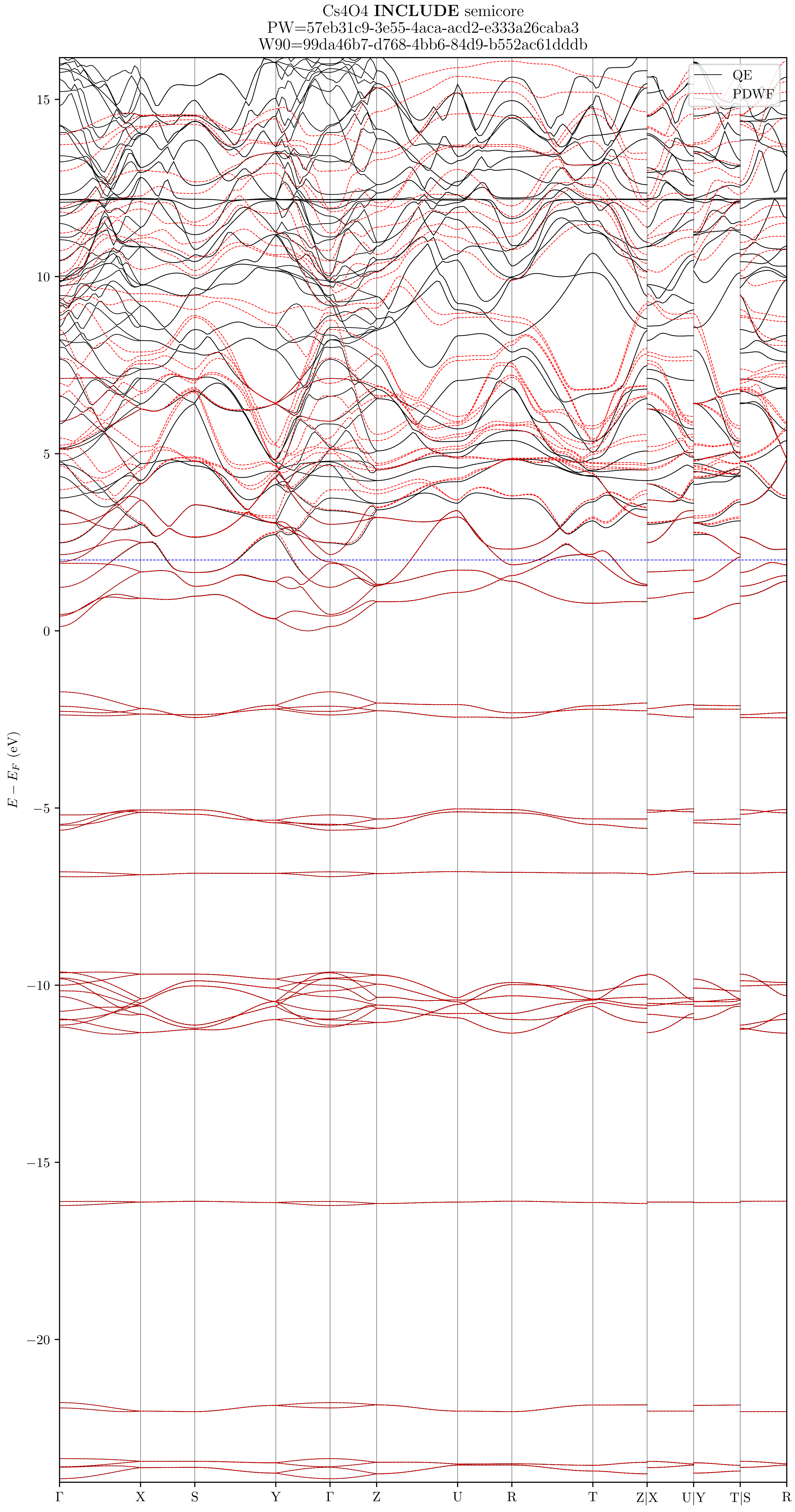

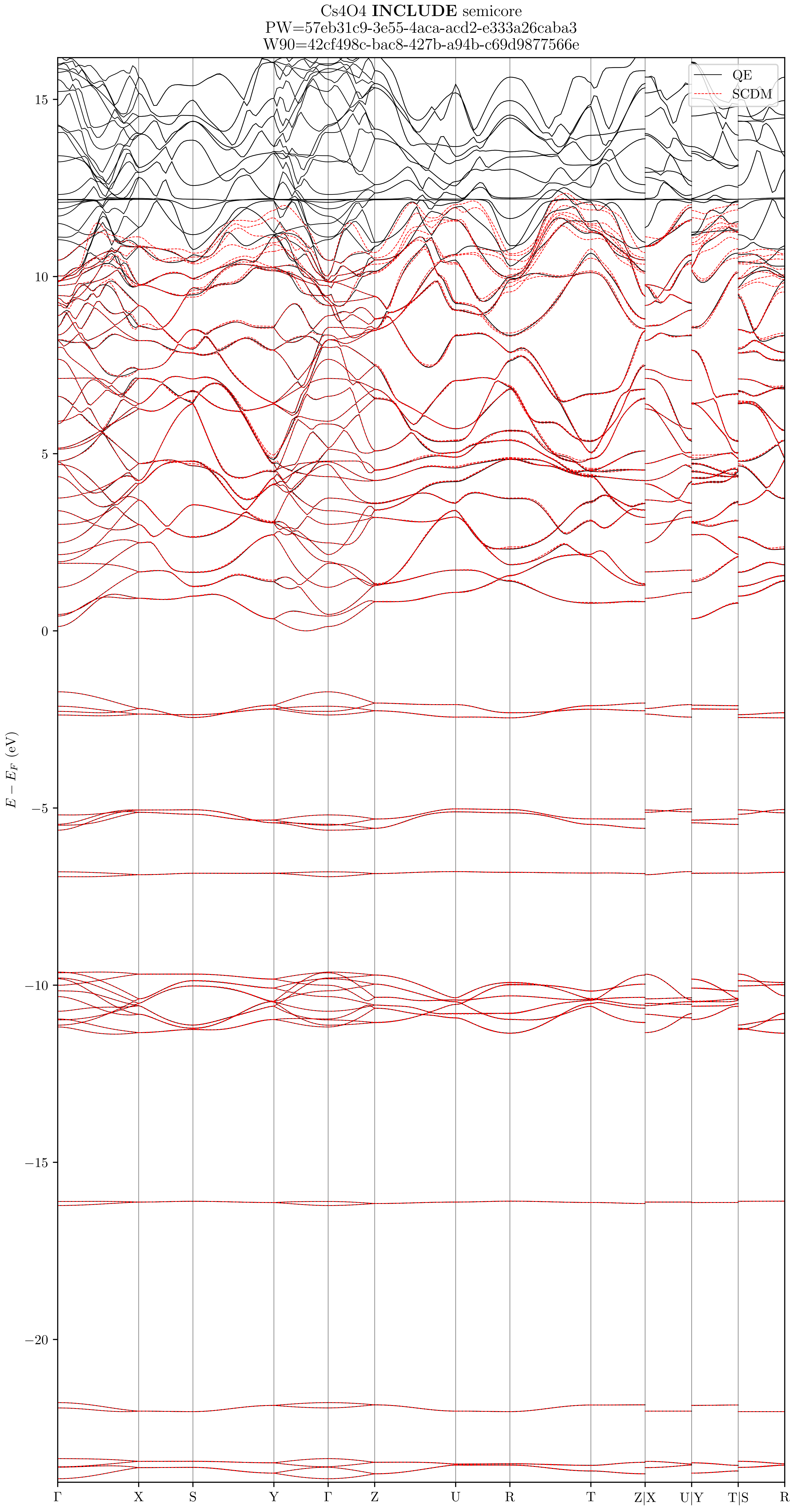

PDWF band distance (meV) and τ ($\AA$)

η_0	η_2	η_4	$\eta_0^{\max}$	$\eta_2^{\max}$	$\eta_4^{\max}$	τ
1.1	1.3	7.8	8.0	11.1	113.7	2.683

SCDM band distance (meV) and τ ($\AA$)

η_0	η_2	η_4	$\eta_0^{\max}$	$\eta_2^{\max}$	$\eta_4^{\max}$	τ
3.3	3.5	5.9	22.4	22.4	62.6	3.266

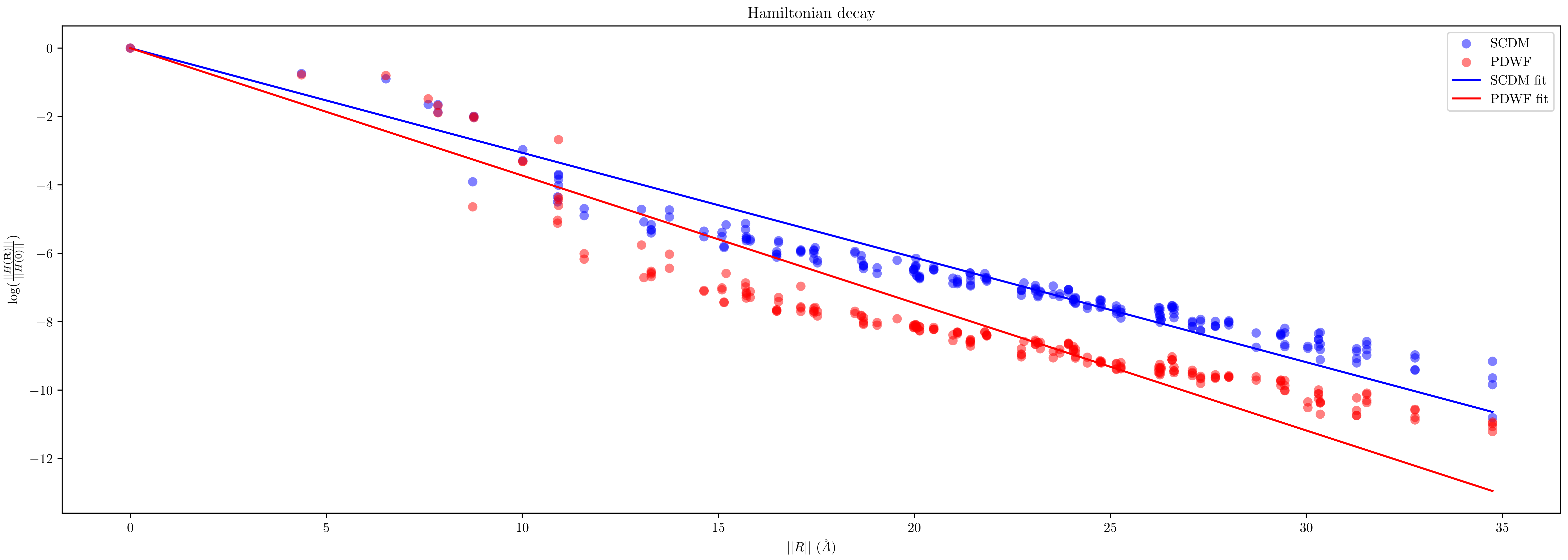

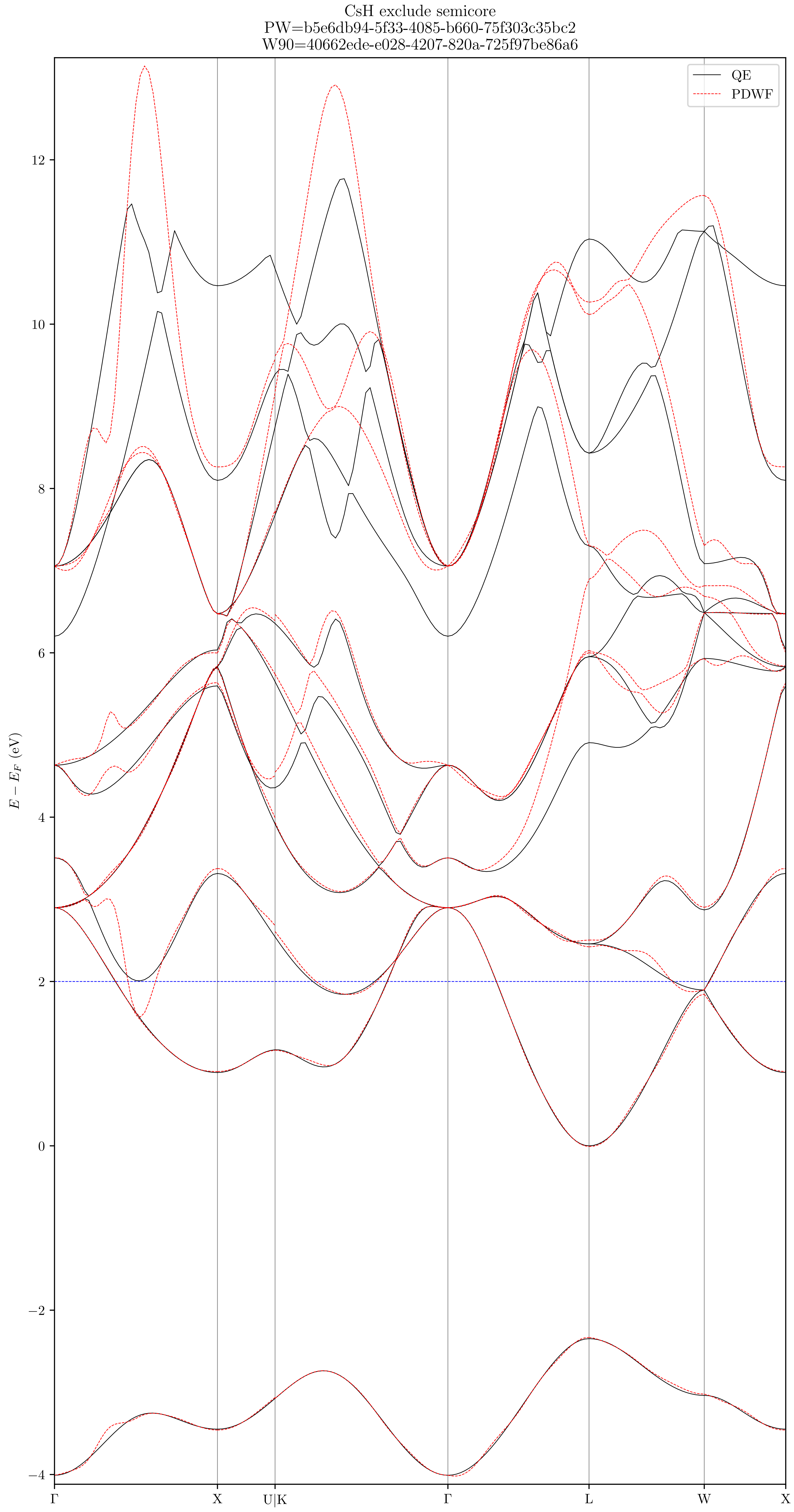

PDWF band distance (meV) and τ ($\AA$)

η_0	η_2	η_4	$\eta_0^{\max}$	$\eta_2^{\max}$	$\eta_4^{\max}$	τ
22.7	20.6	65.8	121.4	121.4	617.0	2.821

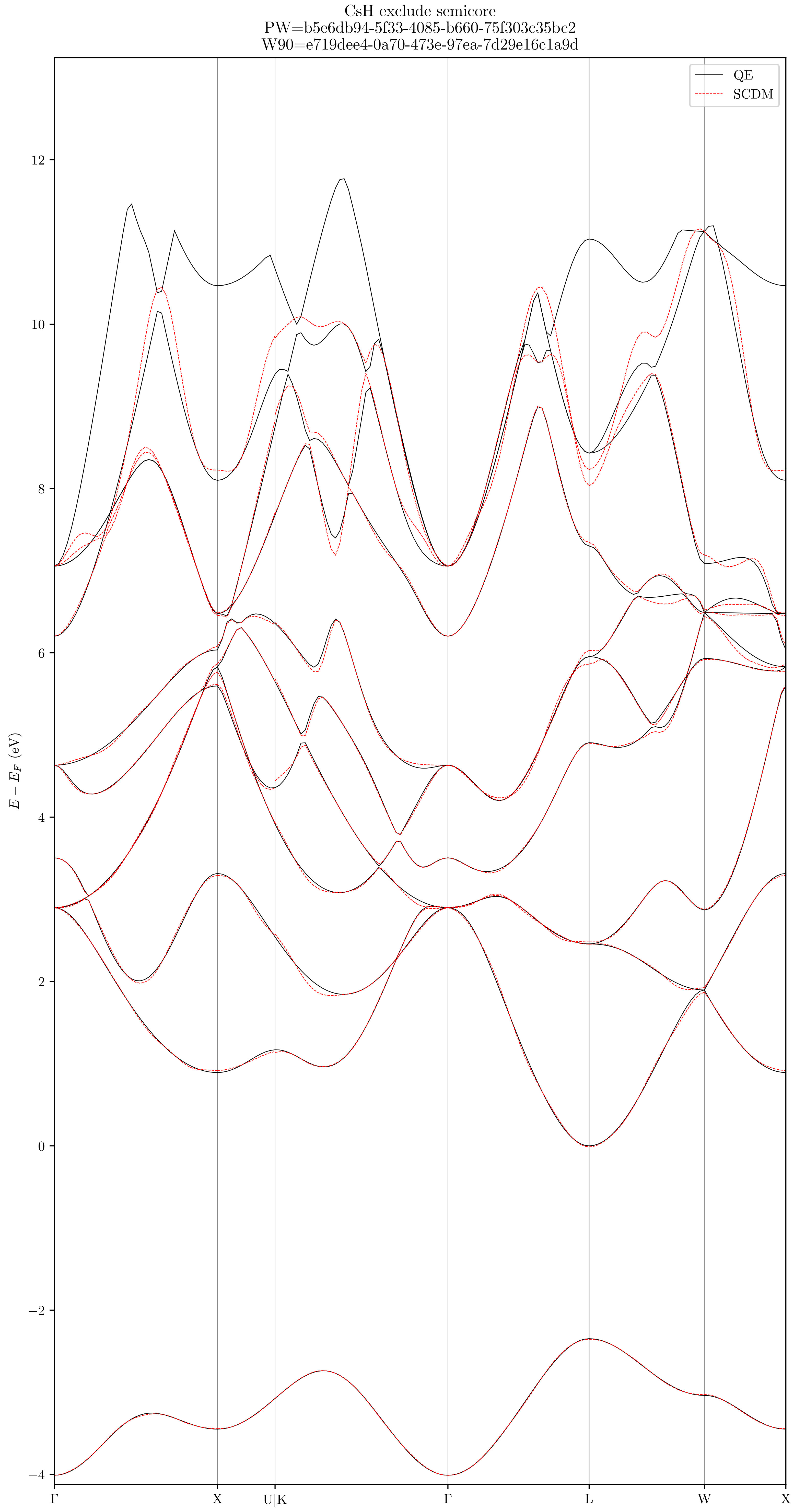

SCDM band distance (meV) and τ ($\AA$)

η_0	η_2	η_4	$\eta_0^{\max}$	$\eta_2^{\max}$	$\eta_4^{\max}$	τ
4.8	9.8	16.7	14.2	31.1	90.3	2.871

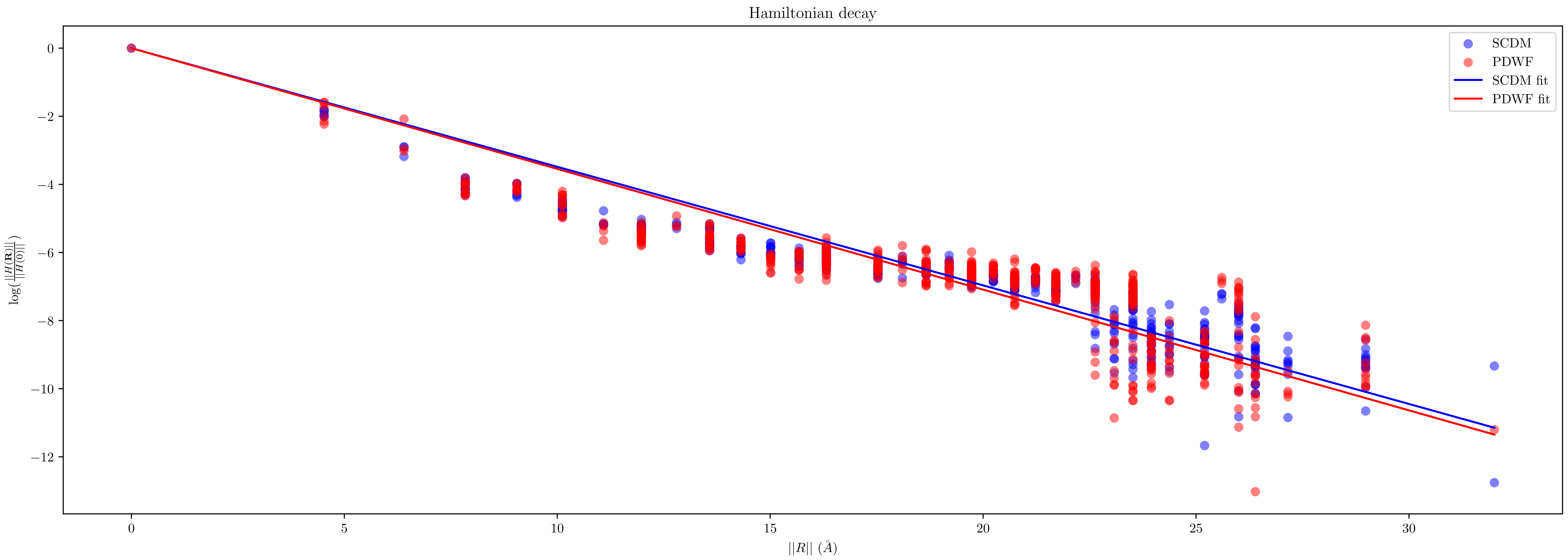

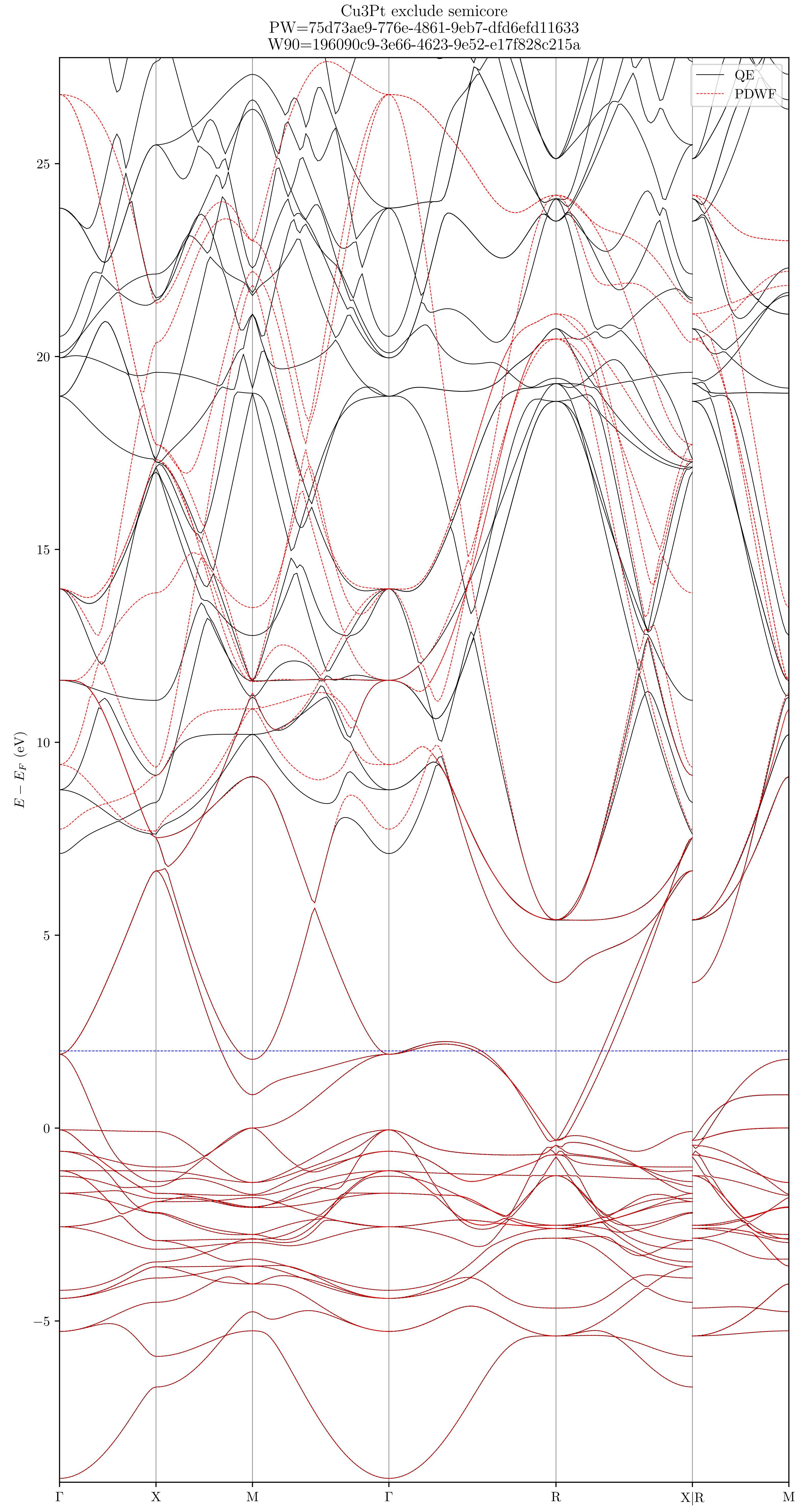

PDWF band distance (meV) and τ ($\AA$)

η_0	η_2	η_4	$\eta_0^{\max}$	$\eta_2^{\max}$	$\eta_4^{\max}$	τ
0.9	1.1	1.2	8.8	8.8	10.5	2.058

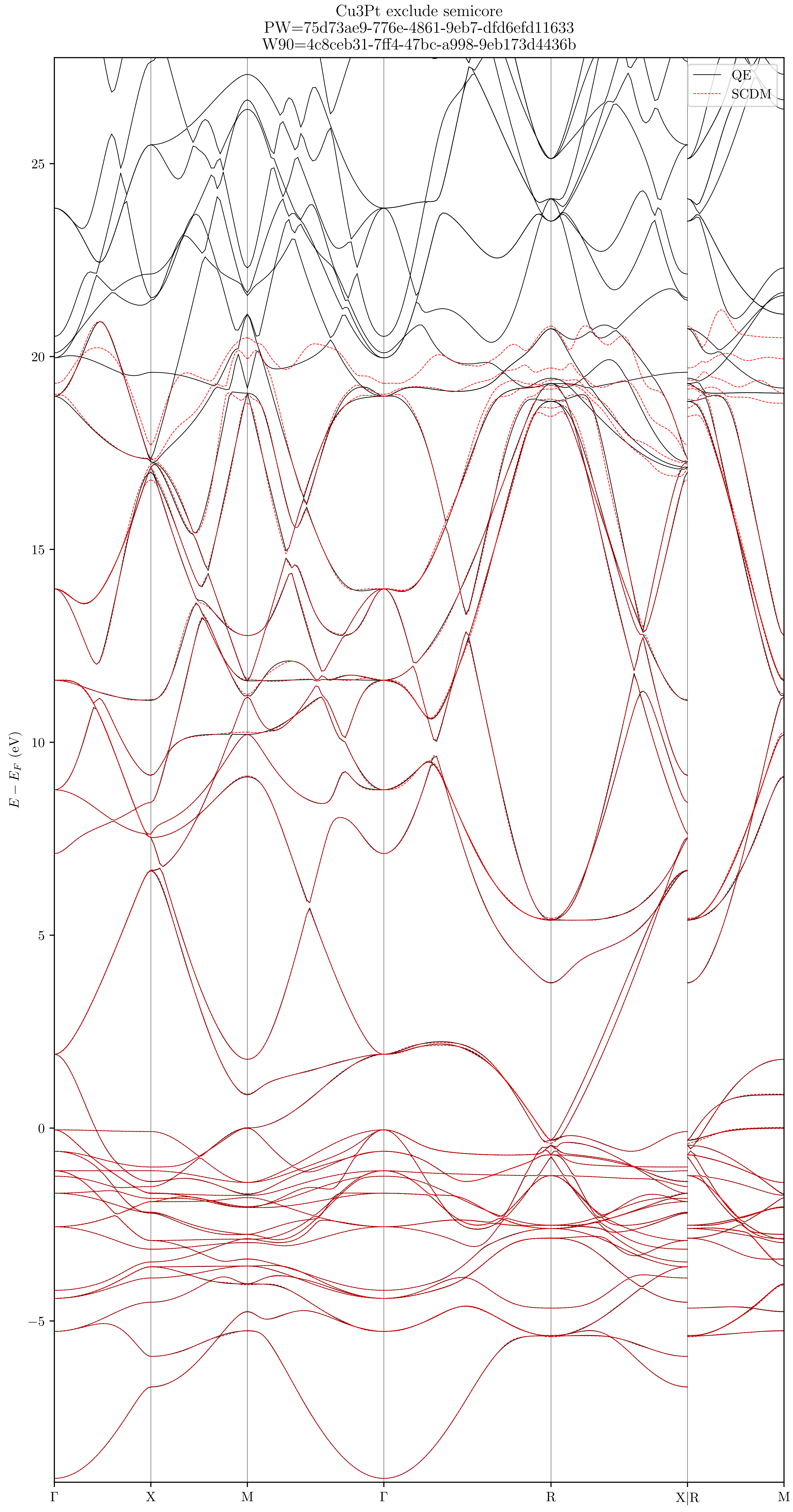

SCDM band distance (meV) and τ ($\AA$)

η_0	η_2	η_4	$\eta_0^{\max}$	$\eta_2^{\max}$	$\eta_4^{\max}$	τ
5.1	5.5	5.8	82.3	84.7	84.7	2.495

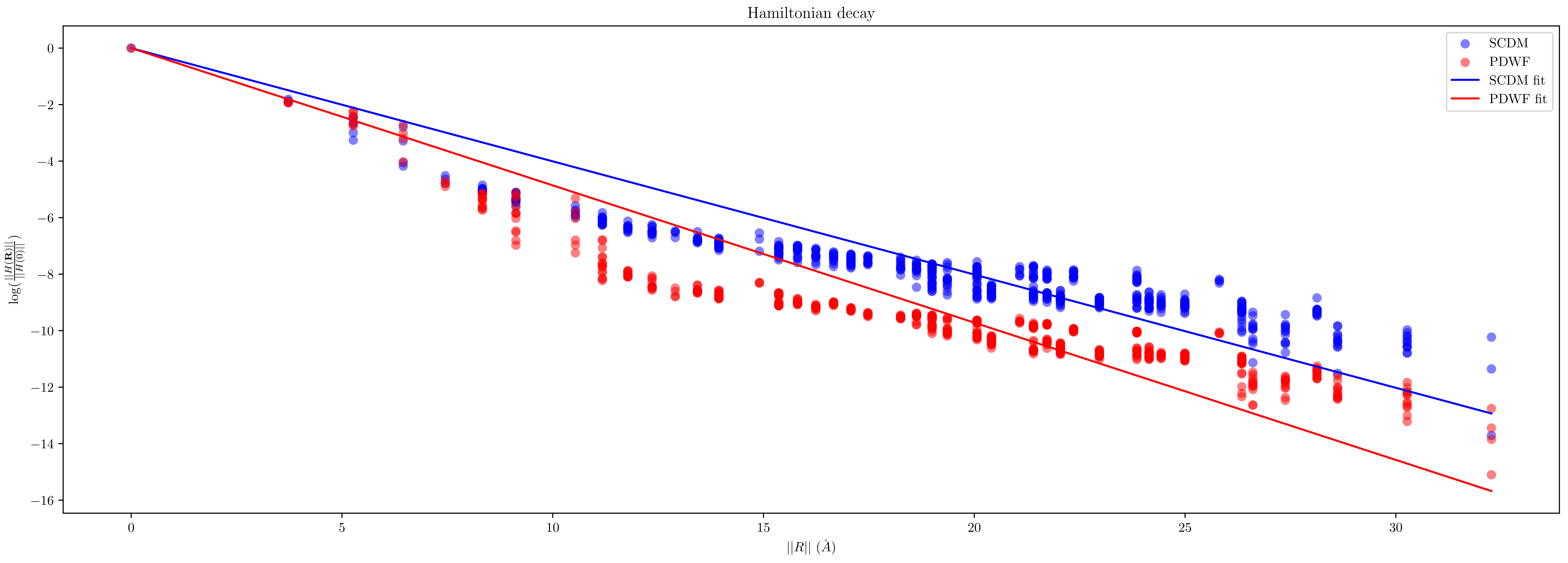

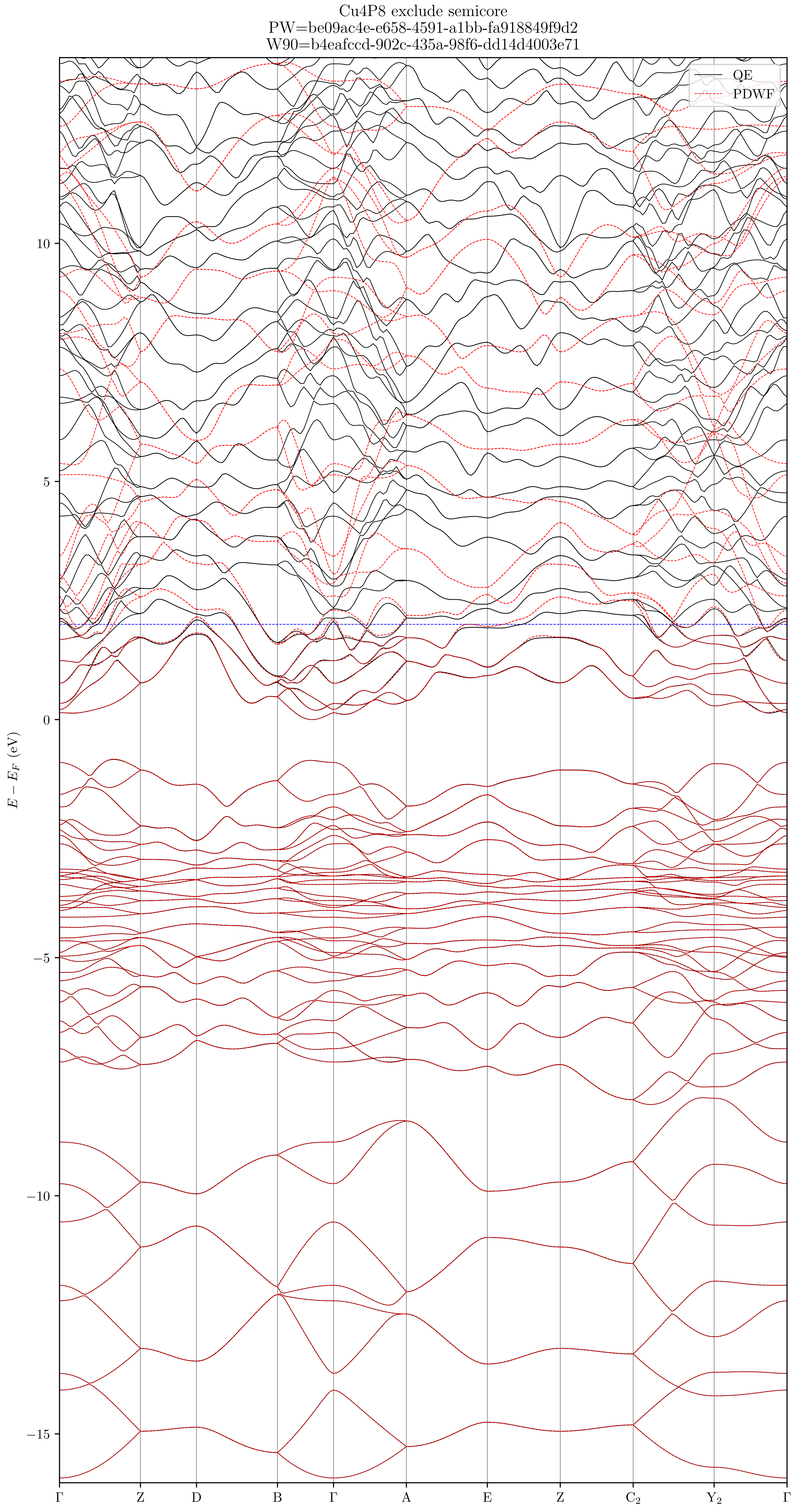

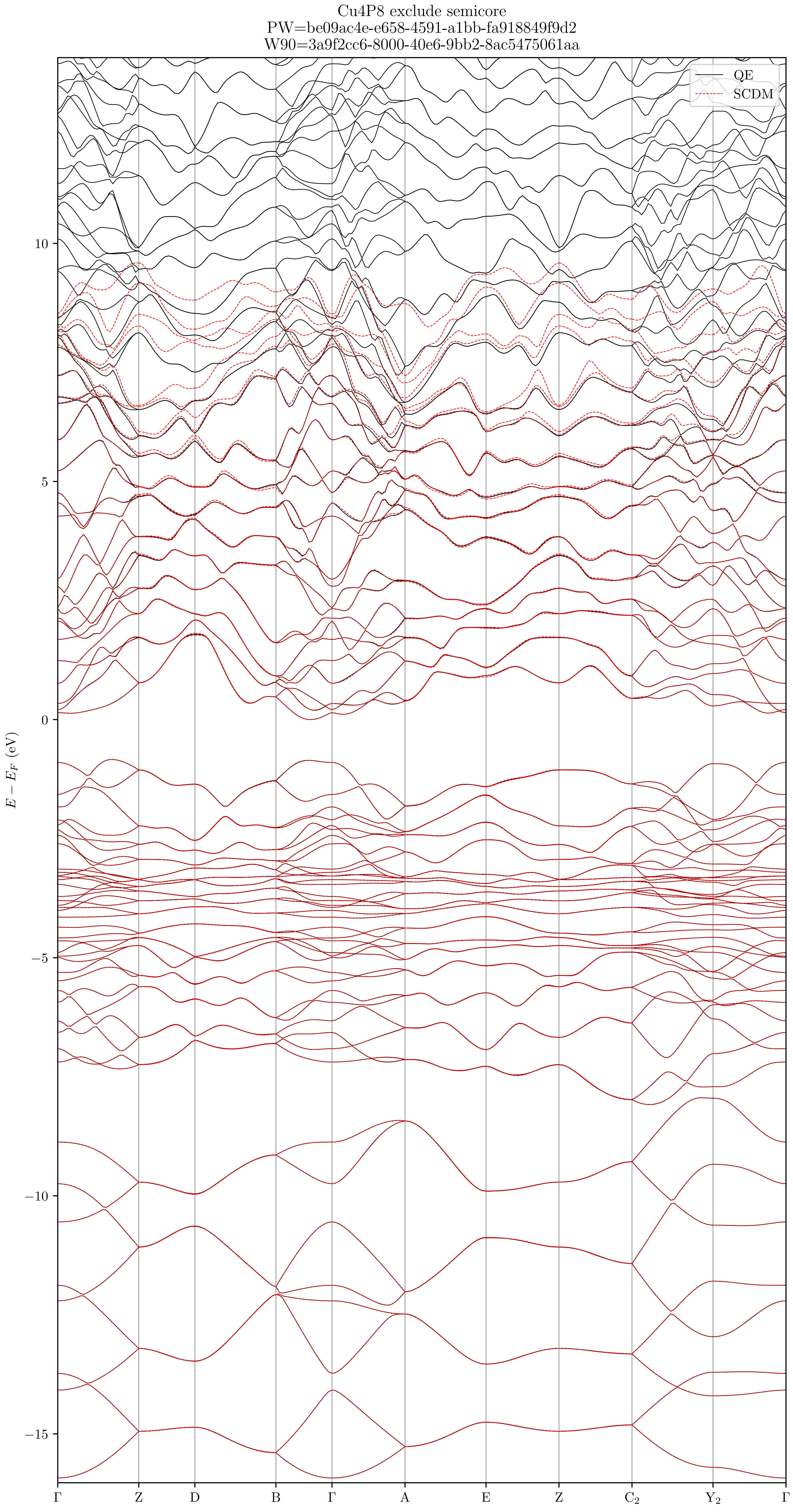

PDWF band distance (meV) and τ ($\mathring{A}$)

η_0	η_2	η_4	$\eta_0^{\max}$	$\eta_2^{\max}$	$\eta_4^{\max}$	τ
1.4	4.0	112.0	8.9	47.3	684.3	2.682

SCDM band distance (meV) and τ ($\mathring{A}$)

η_0	η_2	η_4	$\eta_0^{\max}$	$\eta_2^{\max}$	$\eta_4^{\max}$	τ
2.5	3.0	4.2	14.4	29.8	30.5	2.965

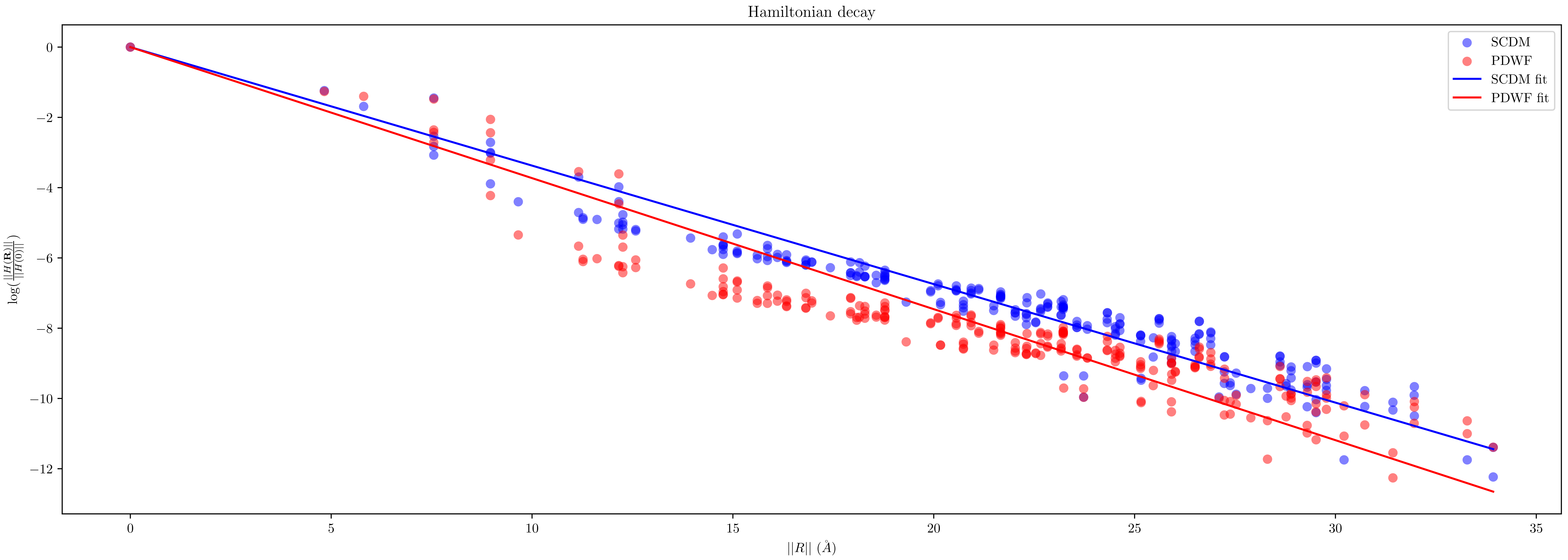

Cu4Sb2 exclude semicore
PW=b7ba5d05-75d3-46fd-b003-a754123188a3
W90=d75a8a21-3543-4770-99b3-040634a070a1

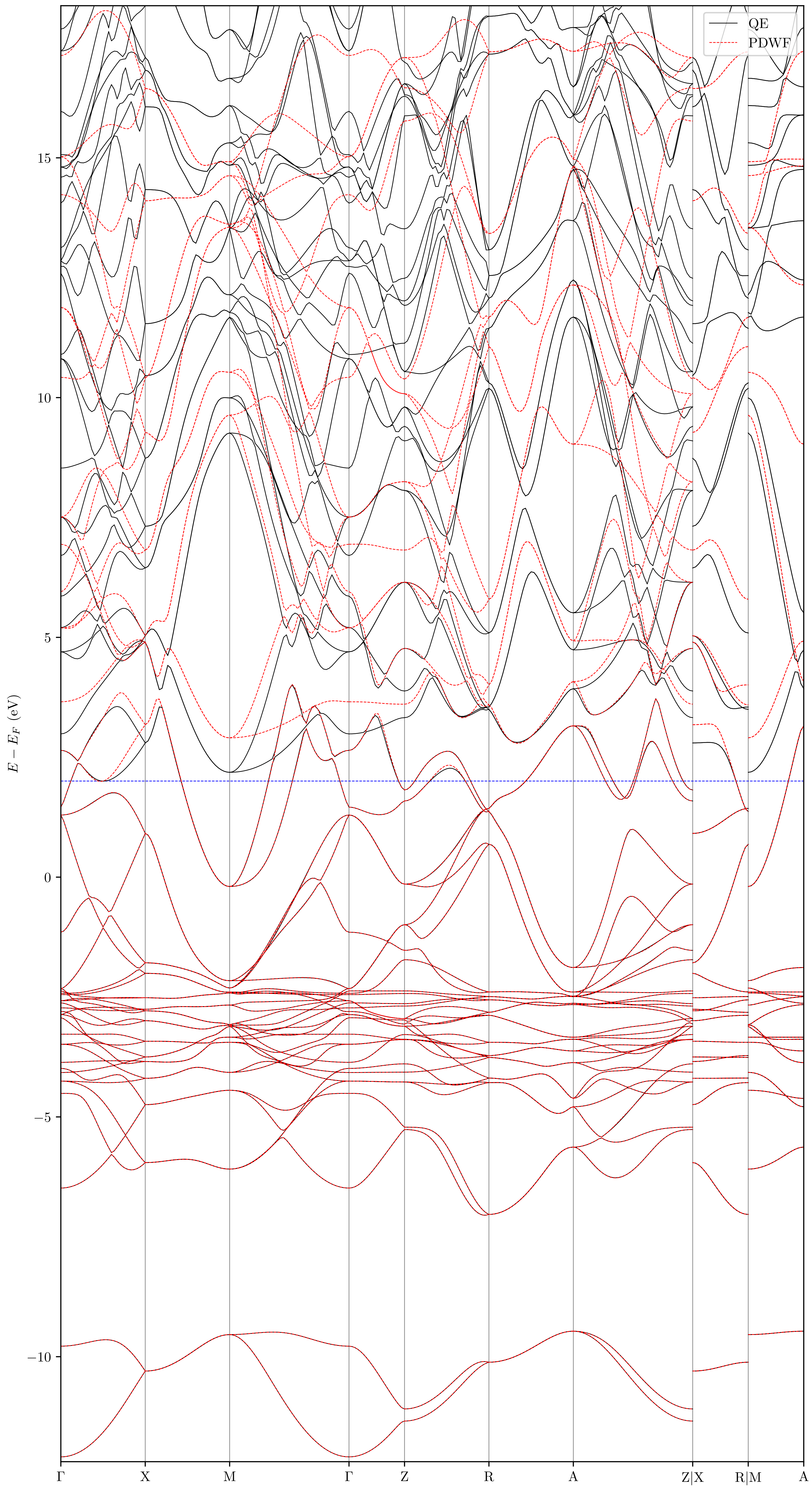

Cu4Sb2 exclude semicore
PW=b7ba5d05-75d3-46fd-b003-a754123188a3
W90=d4b20d7f-dd04-45c4-af48-7f3429874428

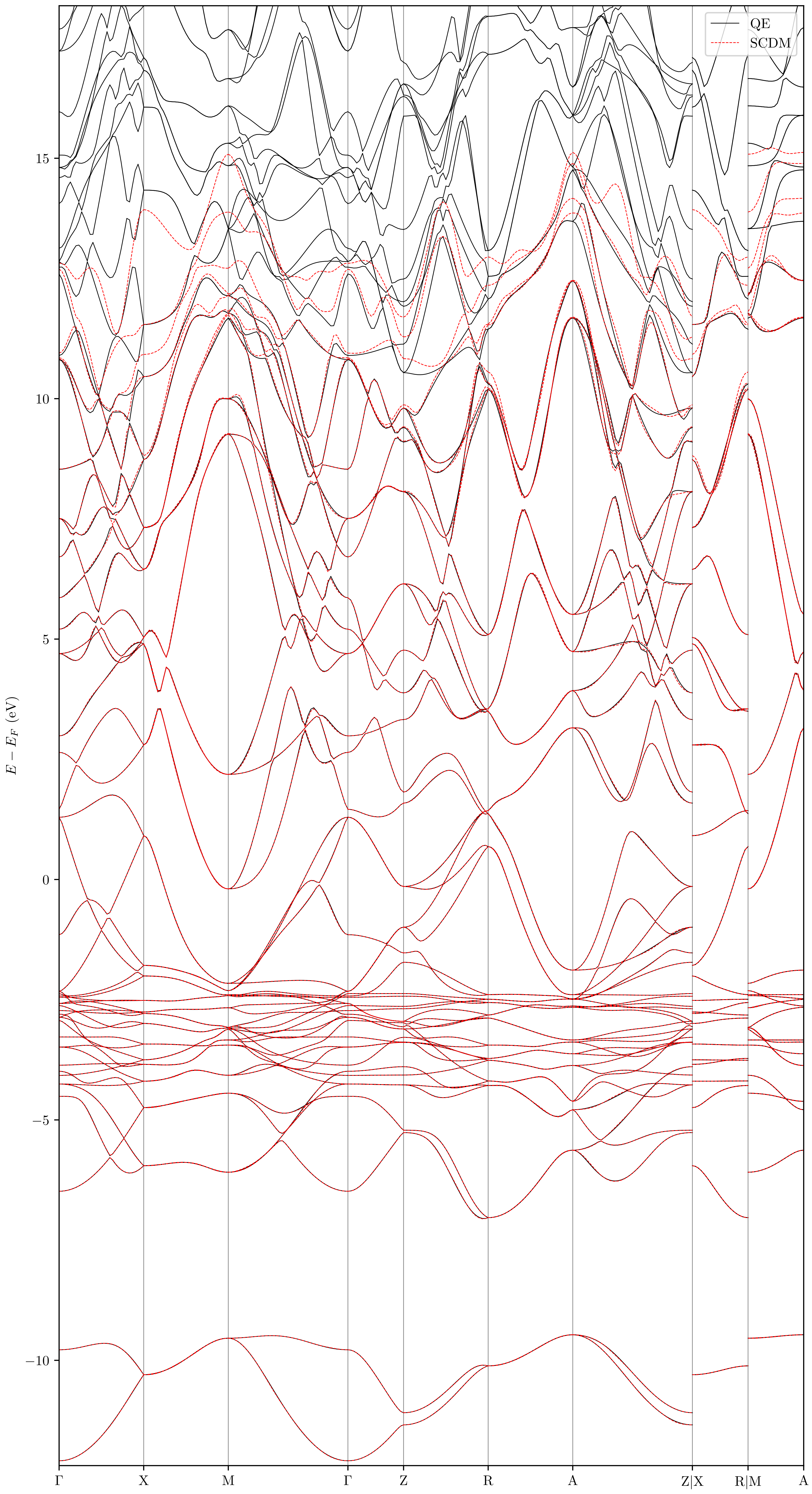

PDWF band distance (meV) and τ ($\AA$)

η_0	η_2	η_4	$\eta_0^{\max}$	$\eta_2^{\max}$	$\eta_4^{\max}$	τ
1.3	3.5	91.9	22.6	23.7	751.8	2.282

SCDM band distance (meV) and τ ($\AA$)

η_0	η_2	η_4	$\eta_0^{\max}$	$\eta_2^{\max}$	$\eta_4^{\max}$	τ
2.6	3.1	4.3	27.4	27.4	54.2	2.755

Hamiltonian decay

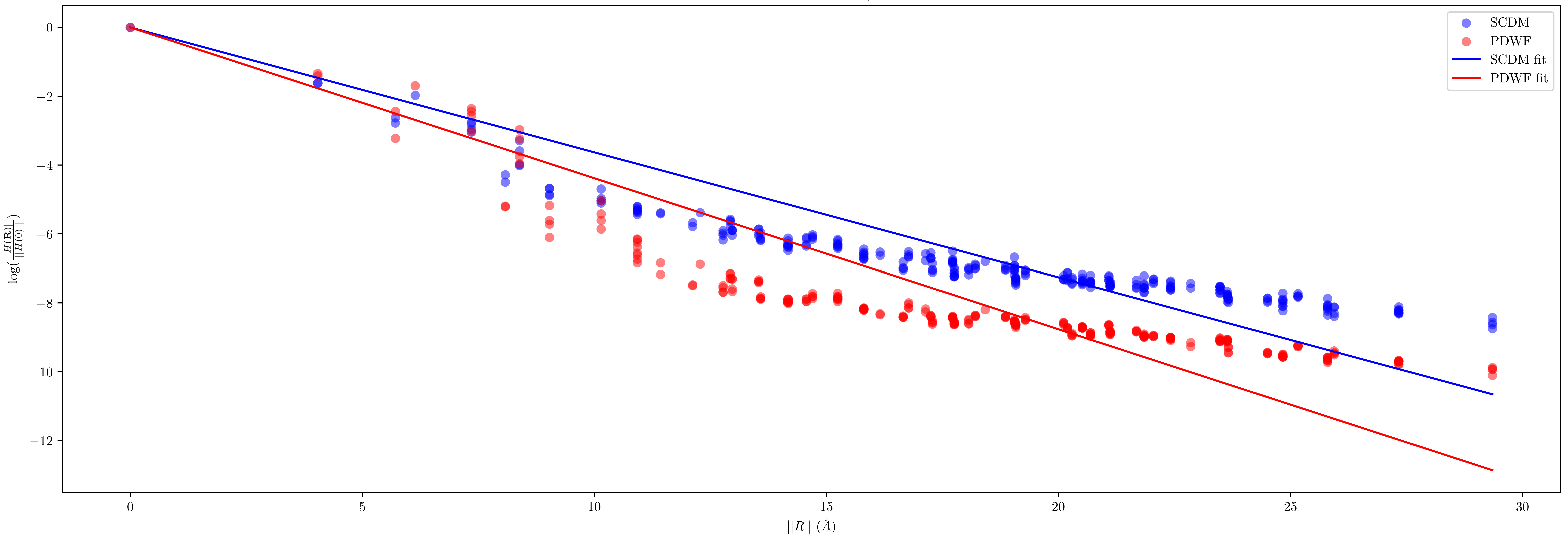

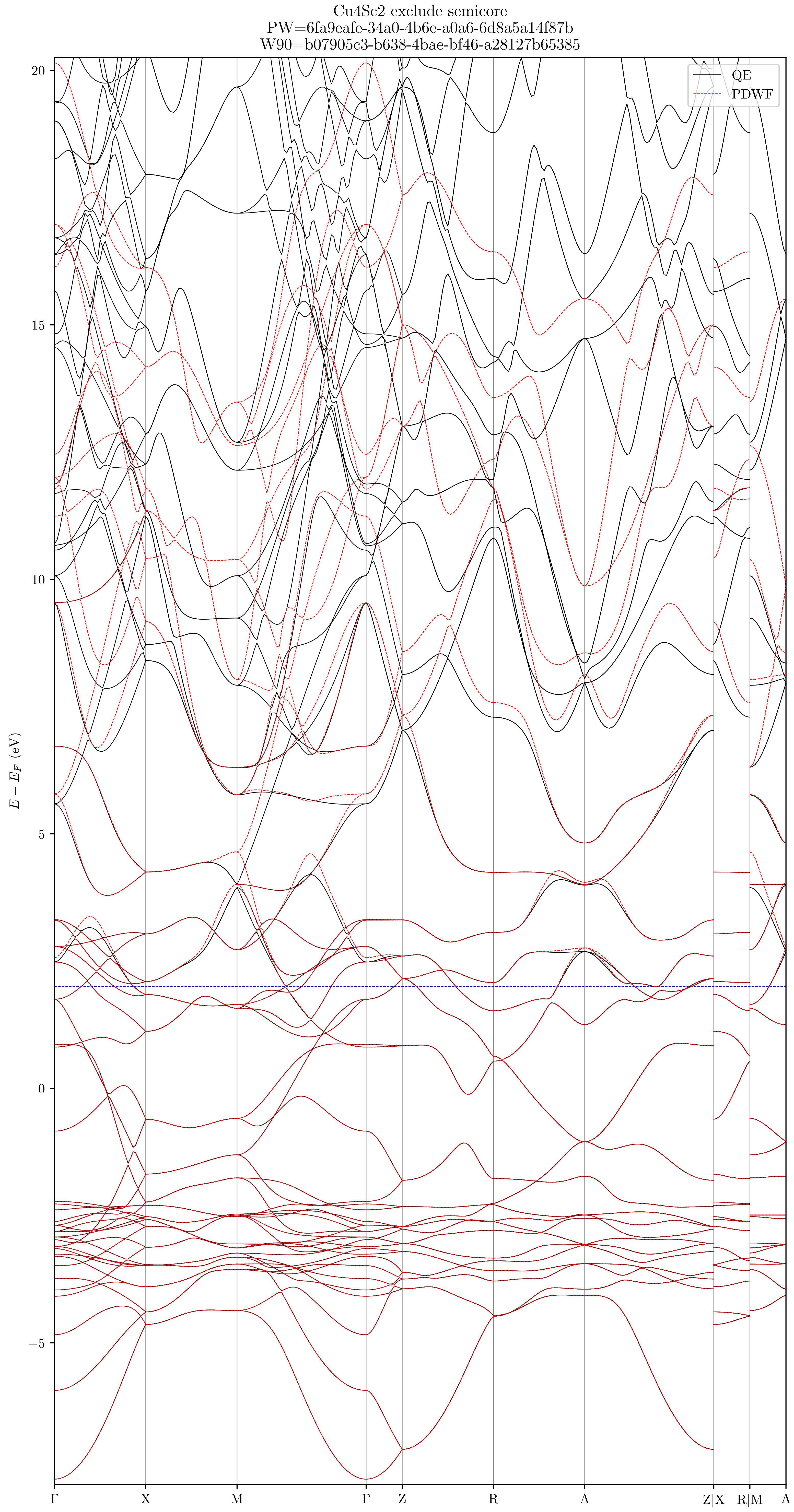

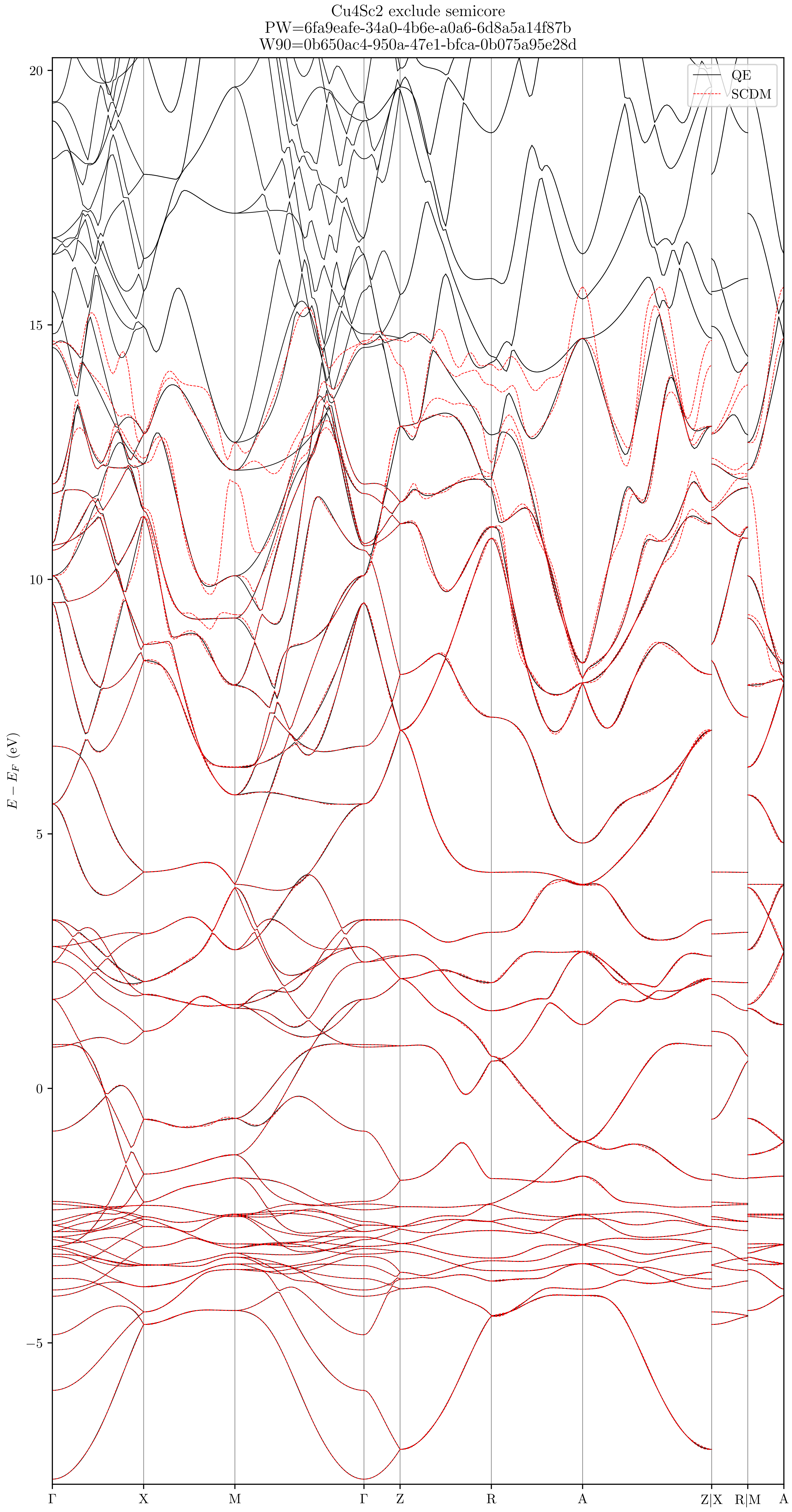

PDWF band distance (meV) and τ ($\AA$)

η_0	η_2	η_4	$\eta_0^{\max}$	$\eta_2^{\max}$	$\eta_4^{\max}$	τ
0.7	1.1	21.2	9.5	18.8	312.0	1.635

SCDM band distance (meV) and τ ($\AA$)

η_0	η_2	η_4	$\eta_0^{\max}$	$\eta_2^{\max}$	$\eta_4^{\max}$	τ
4.1	4.8	5.9	37.2	39.4	71.2	2.754

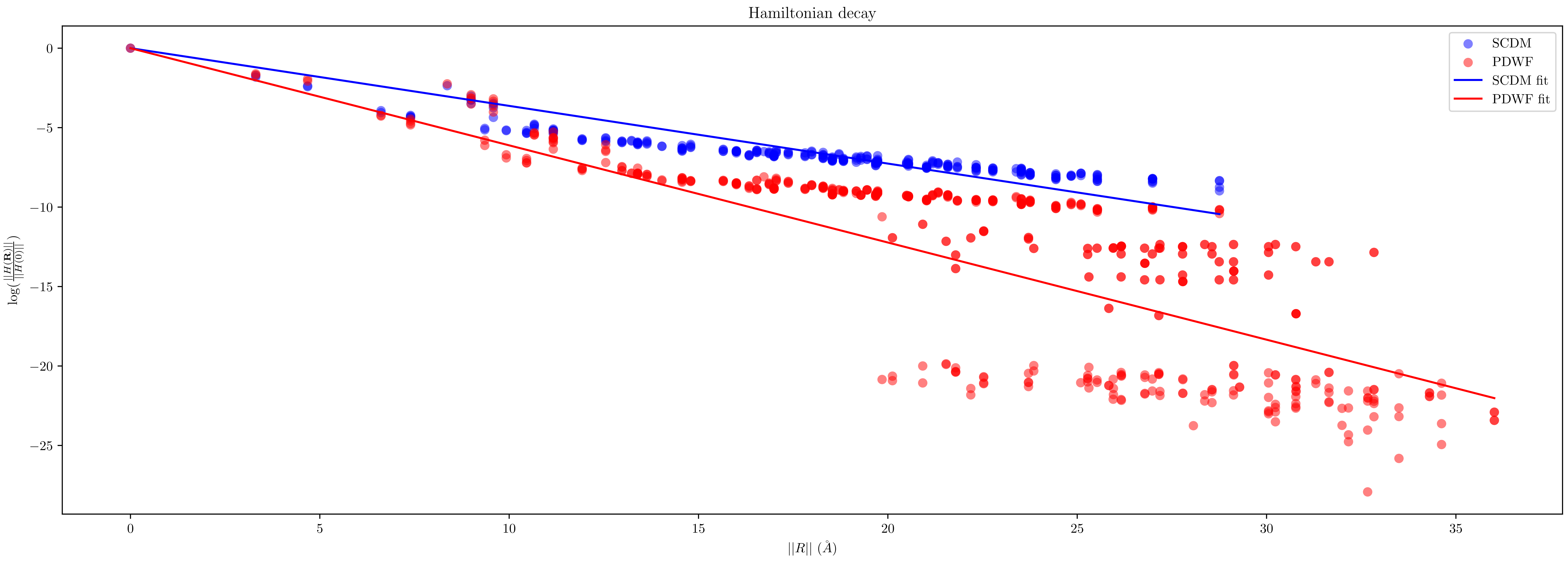

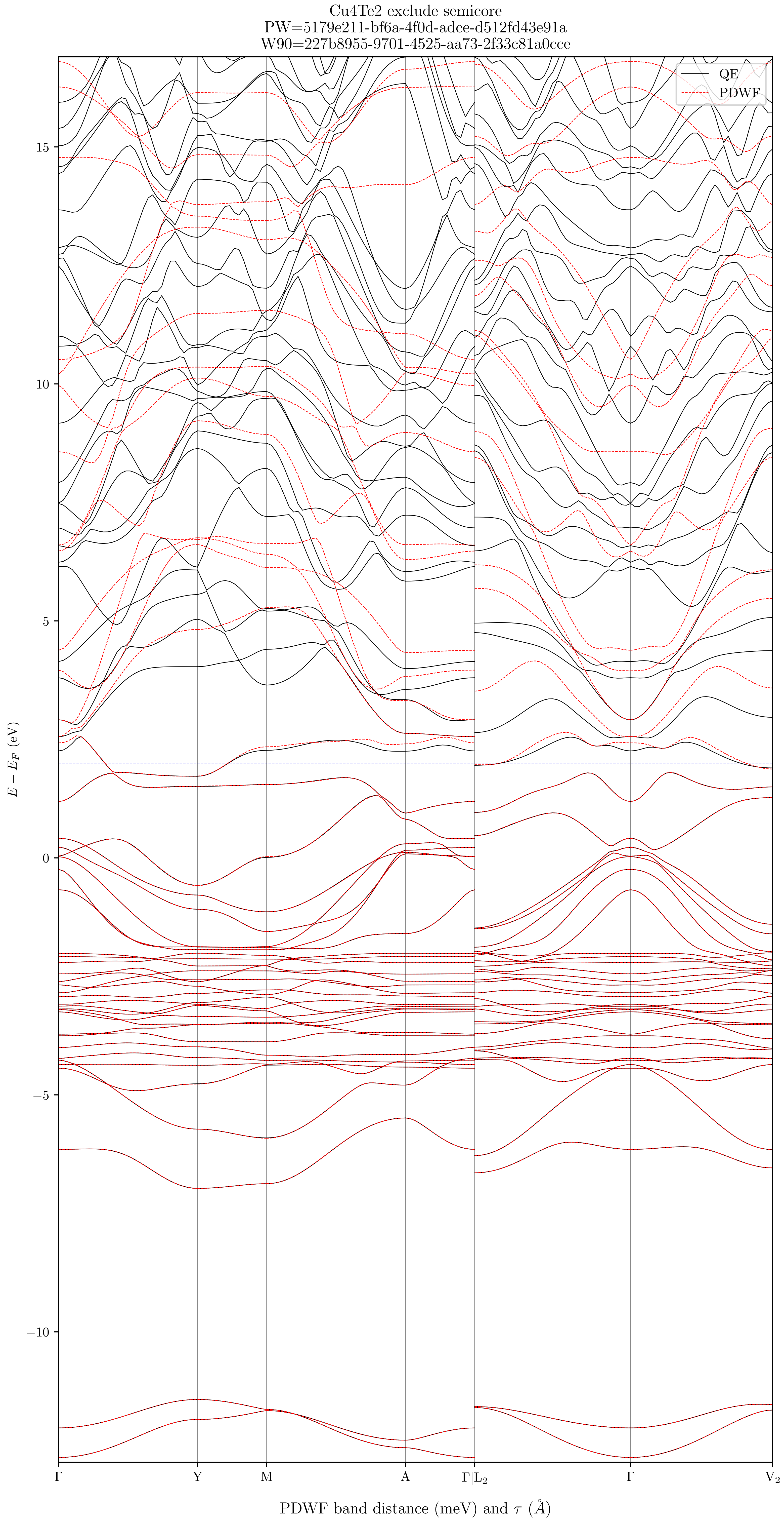

η_0	η_2	η_4	$\eta_0^{\max}$	$\eta_2^{\max}$	$\eta_4^{\max}$	τ
1.1	3.3	69.1	8.3	21.1	931.4	2.565

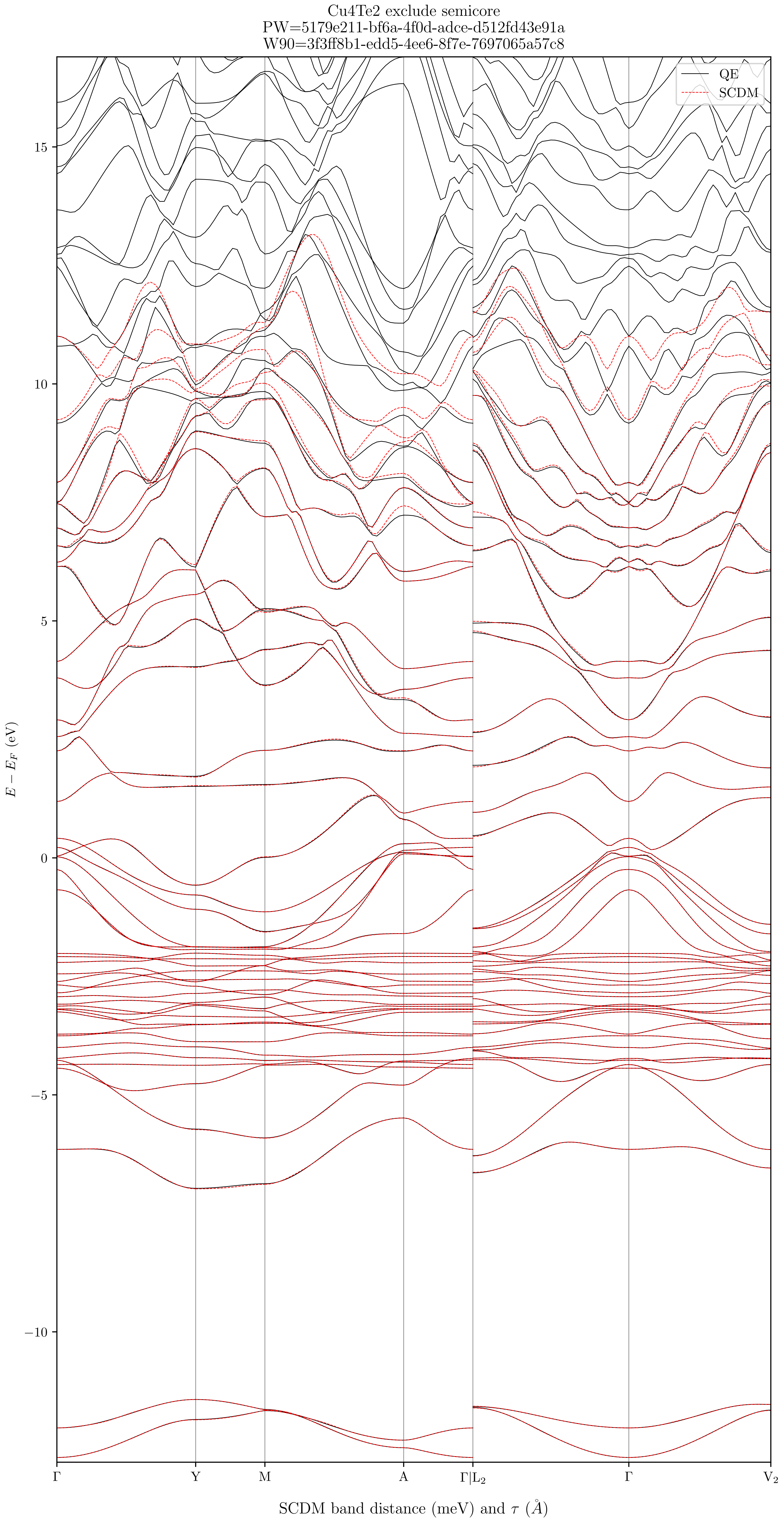

η_0	η_2	η_4	$\eta_0^{\max}$	$\eta_2^{\max}$	$\eta_4^{\max}$	τ
2.5	3.2	4.1	25.4	27.8	38.0	3.062

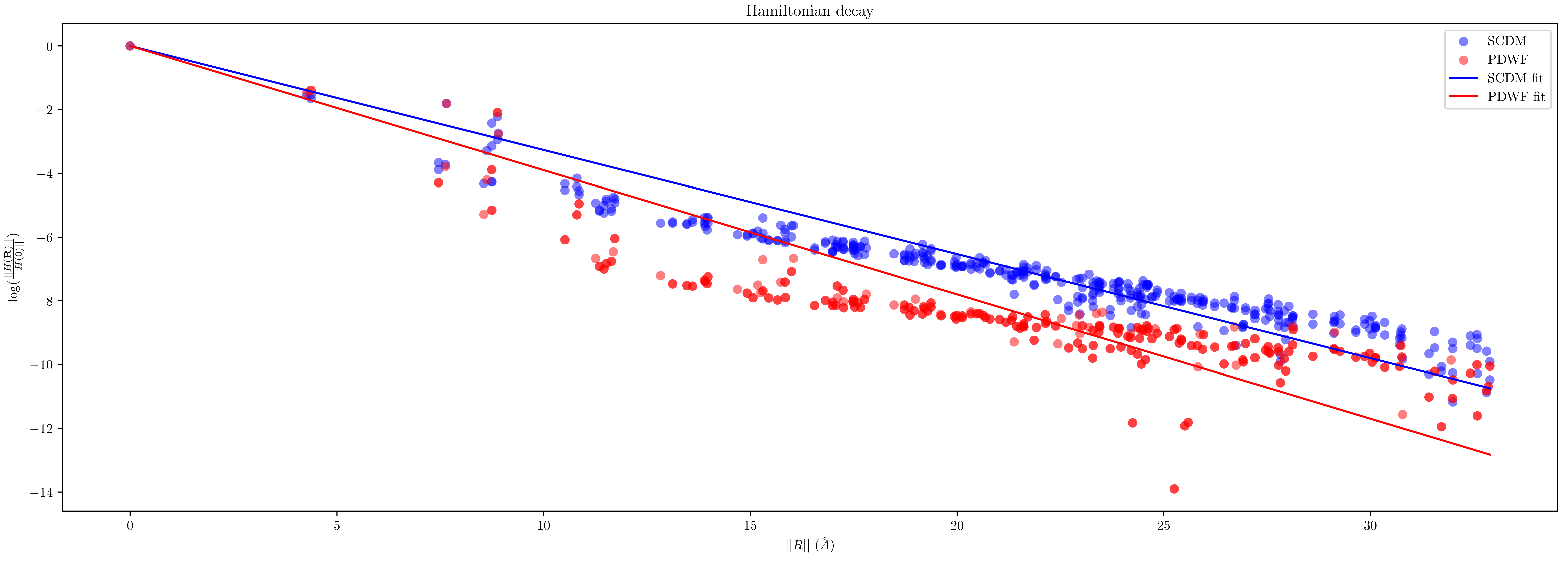

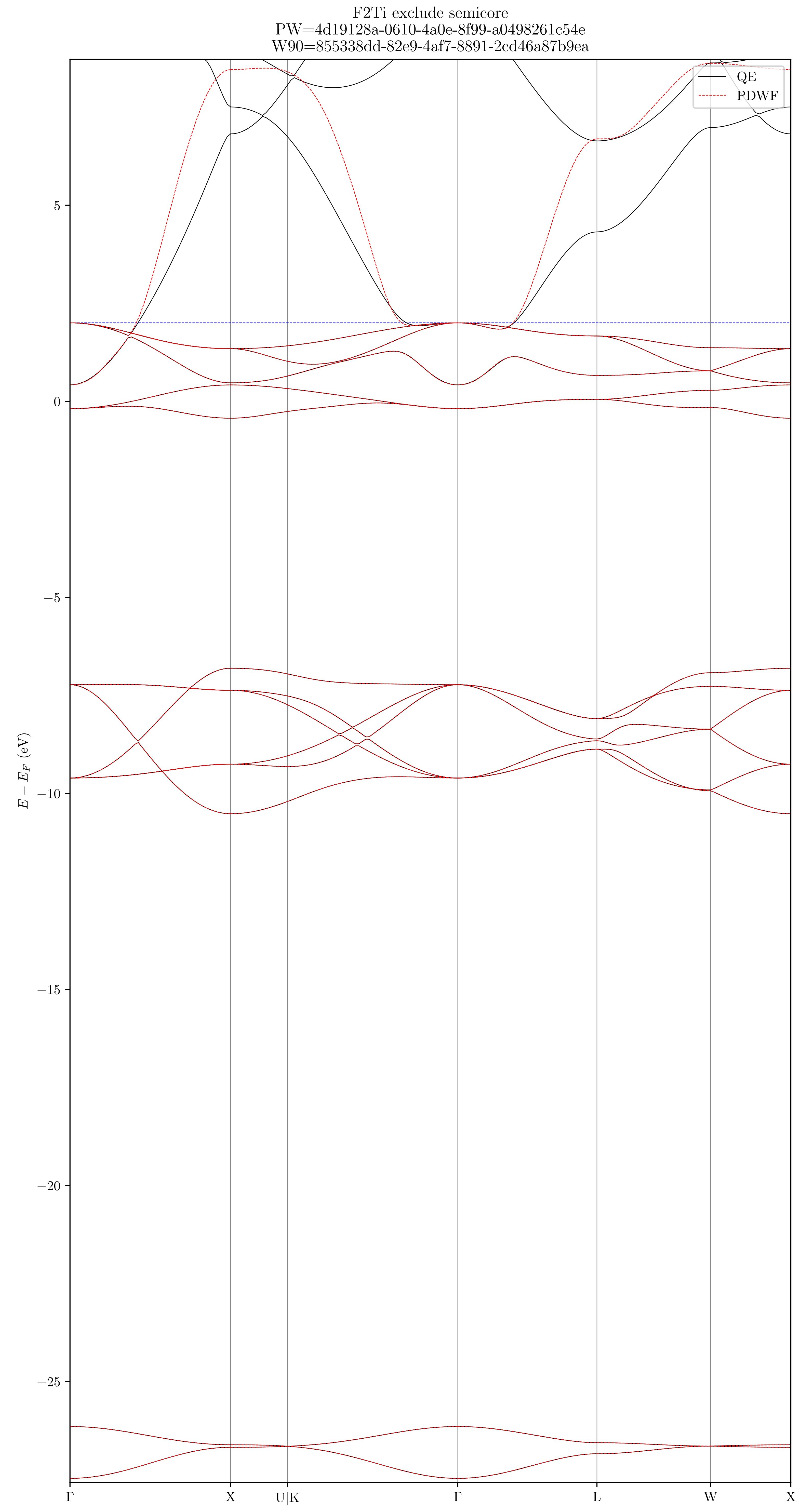

PDWF band distance (meV) and τ ($\AA$)

η_0	η_2	η_4	$\eta_0^{\max}$	$\eta_2^{\max}$	$\eta_4^{\max}$	τ
0.2	3.0	47.8	1.3	34.3	820.7	1.779

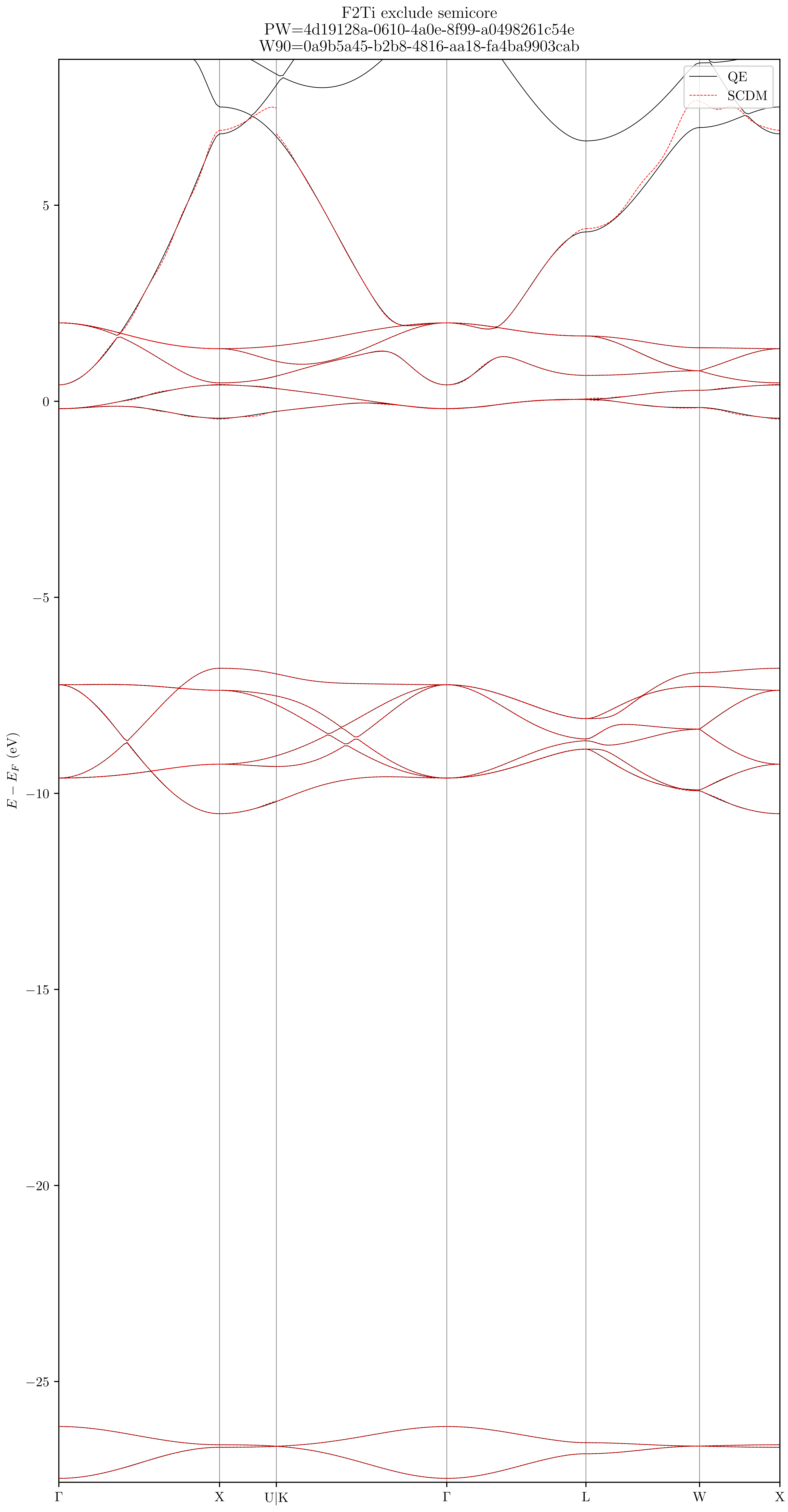

SCDM band distance (meV) and τ ($\AA$)

η_0	η_2	η_4	$\eta_0^{\max}$	$\eta_2^{\max}$	$\eta_4^{\max}$	τ
4.0	4.4	5.6	35.6	36.6	82.6	2.281

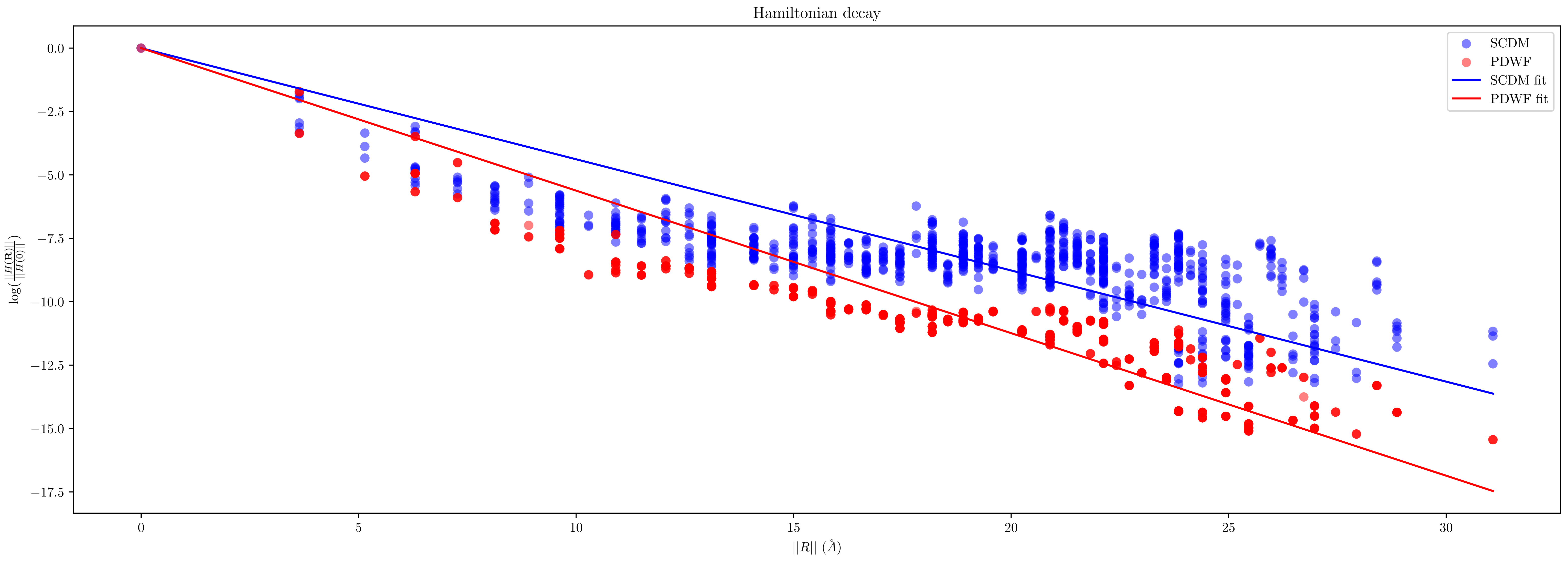

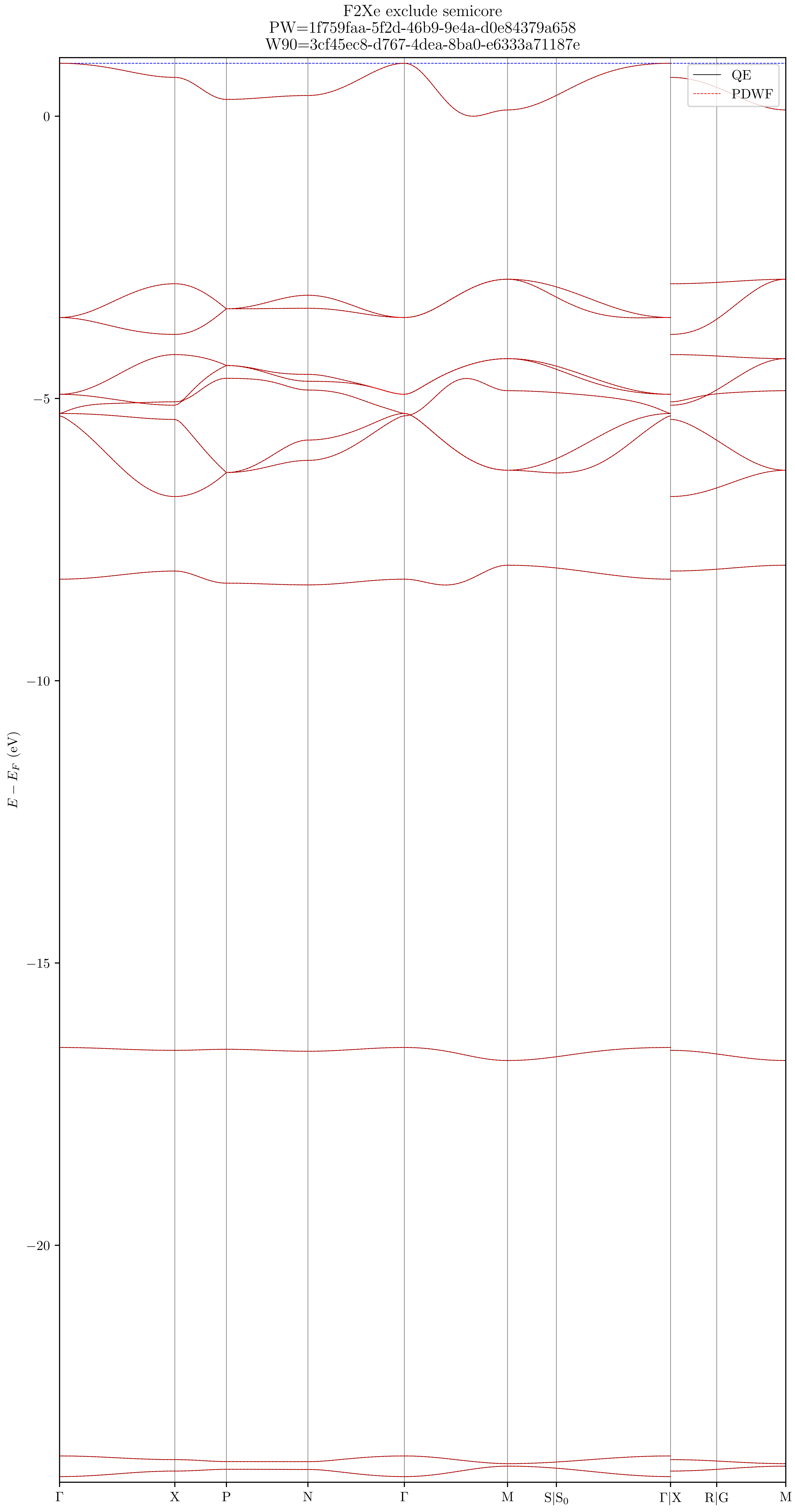

PDWF band distance (meV) and τ ($\AA$)

η_0	η_2	η_4	$\eta_0^{\max}$	$\eta_2^{\max}$	$\eta_4^{\max}$	τ
0.0	0.0	0.0	0.0	0.0	0.1	1.461

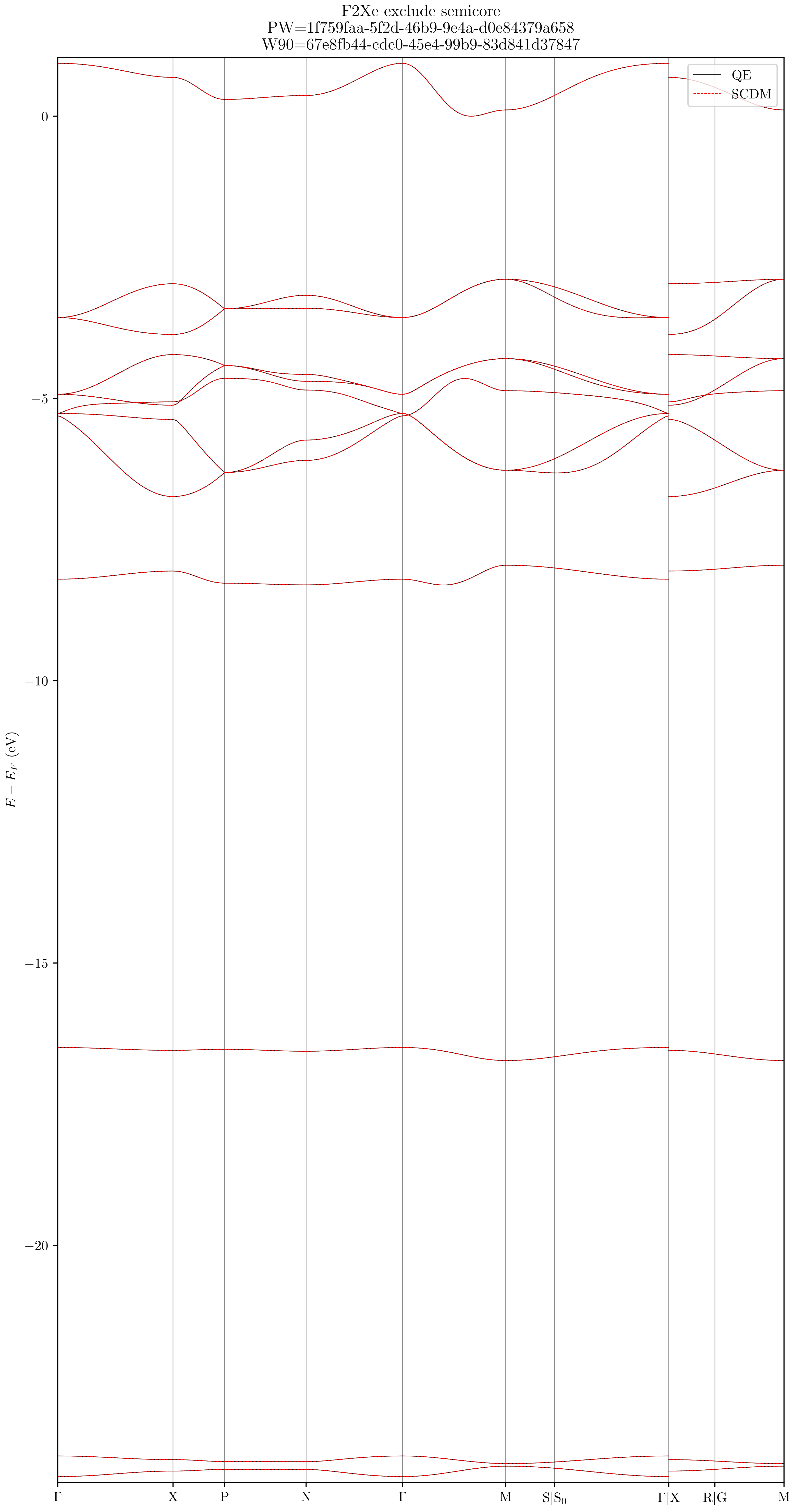

SCDM band distance (meV) and τ ($\AA$)

η_0	η_2	η_4	$\eta_0^{\max}$	$\eta_2^{\max}$	$\eta_4^{\max}$	τ
0.0	0.0	0.0	0.0	0.0	0.1	1.521

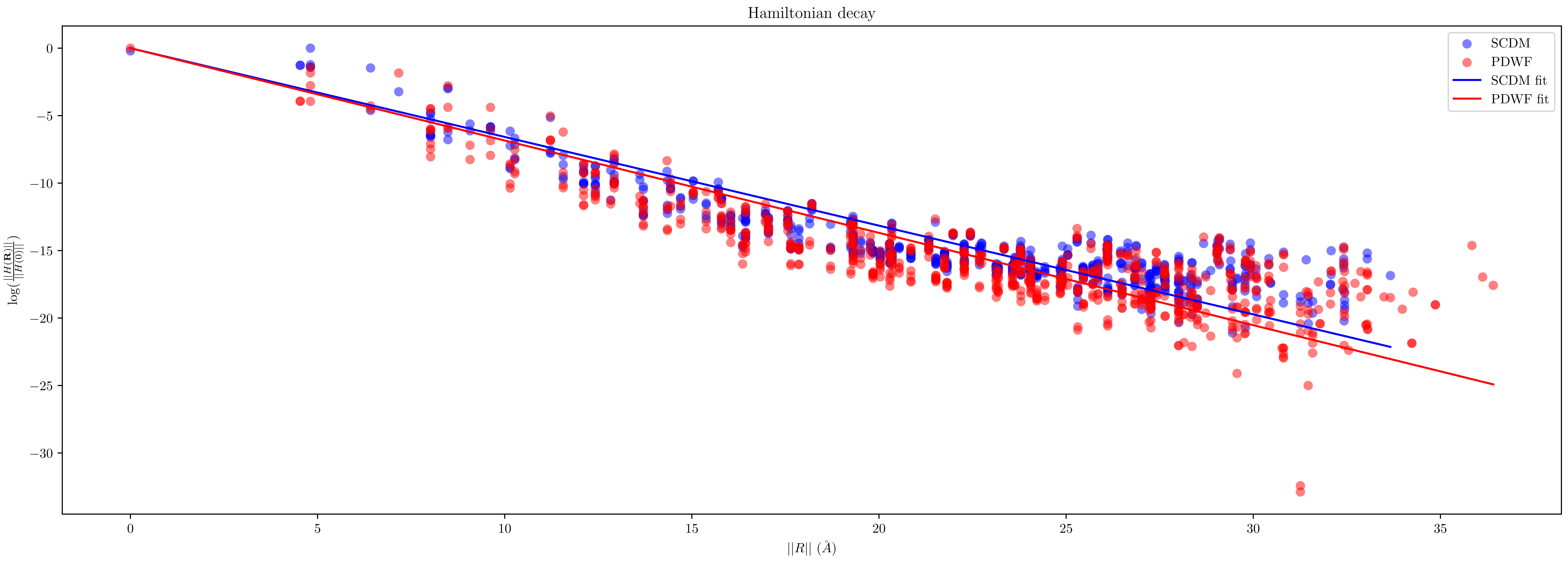

F4H2K2 **INCLUDE** semicore
PW=eed97ba0-21c3-4769-aa94-e6069ca79411
W90=bb379938-fb68-4cfa-ab8e-a0cda8c5605e

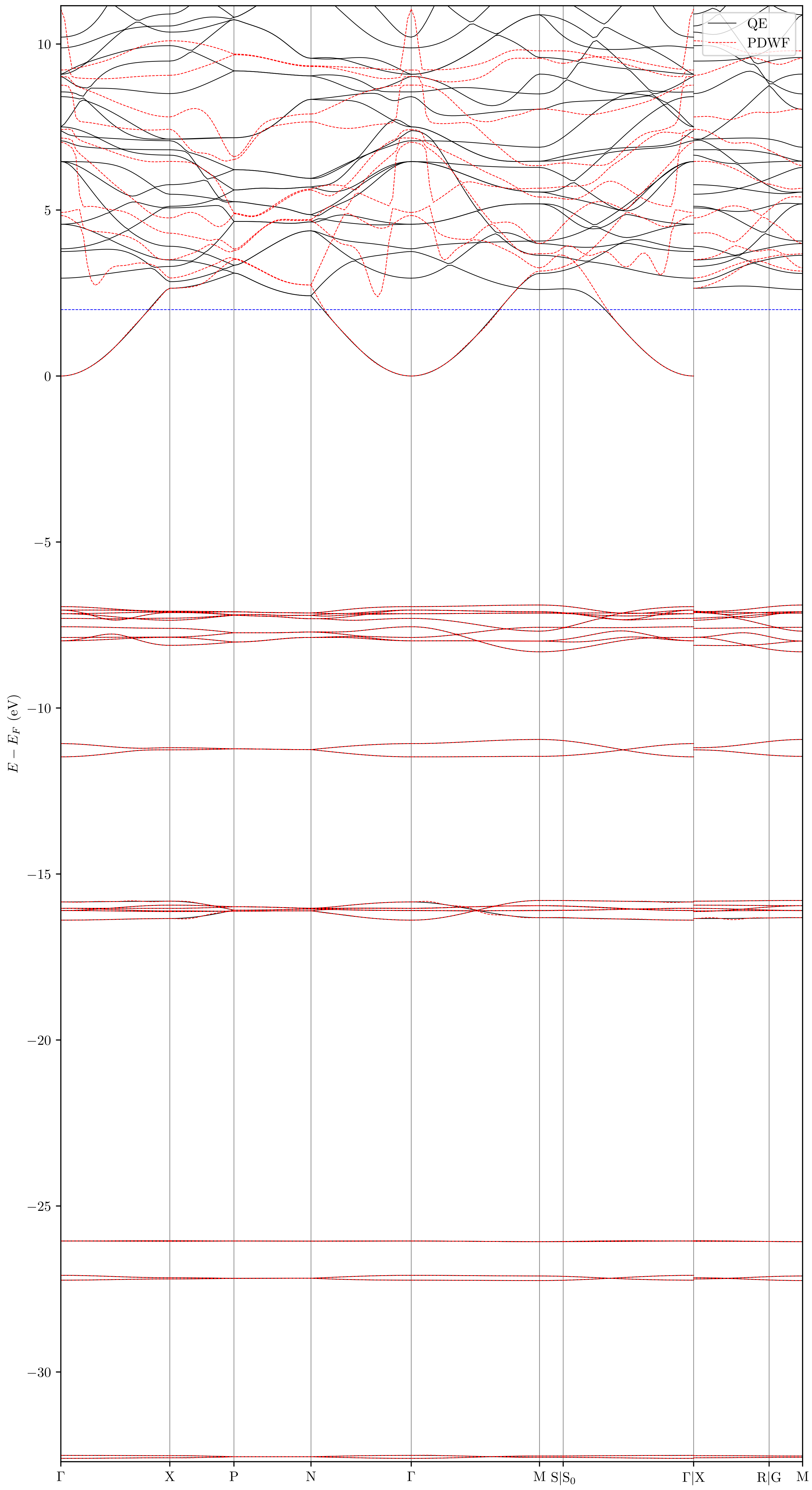

F4H2K2 **INCLUDE** semicore
PW=eed97ba0-21c3-4769-aa94-e6069ca79411
W90=afa80ddd-472c-4eaa-a648-6fedda6f784b

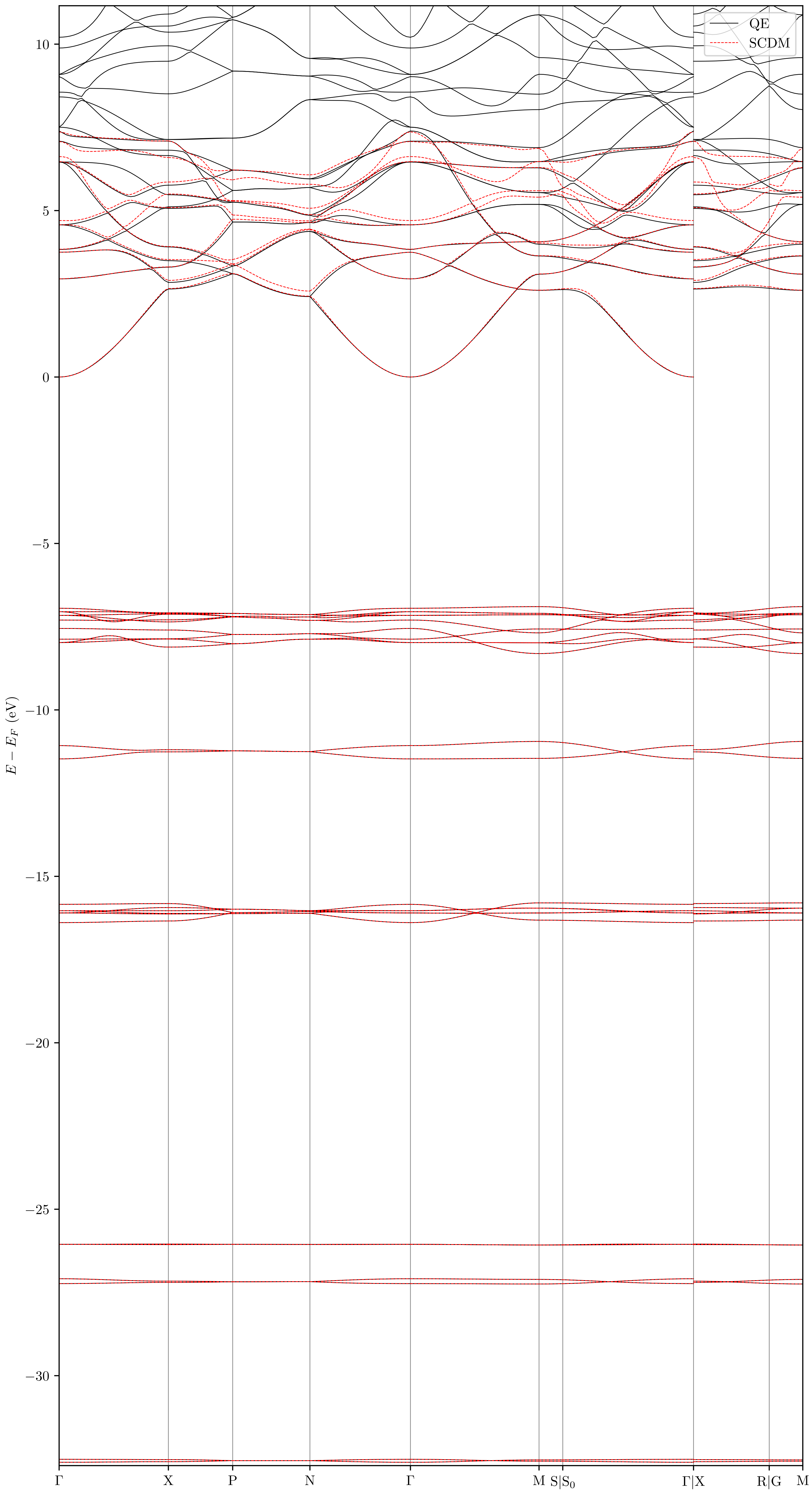

PDWF band distance (meV) and τ ($\AA$)

η_0	η_2	η_4	η_0^{max}	η_2^{max}	η_4^{max}	τ
3.0	3.0	3.0	52.3	52.3	52.3	2.773

SCDM band distance (meV) and τ ($\AA$)

η_0	η_2	η_4	η_0^{max}	η_2^{max}	η_4^{max}	τ
0.6	0.6	0.7	5.8	5.8	6.2	2.523

Hamiltonian decay

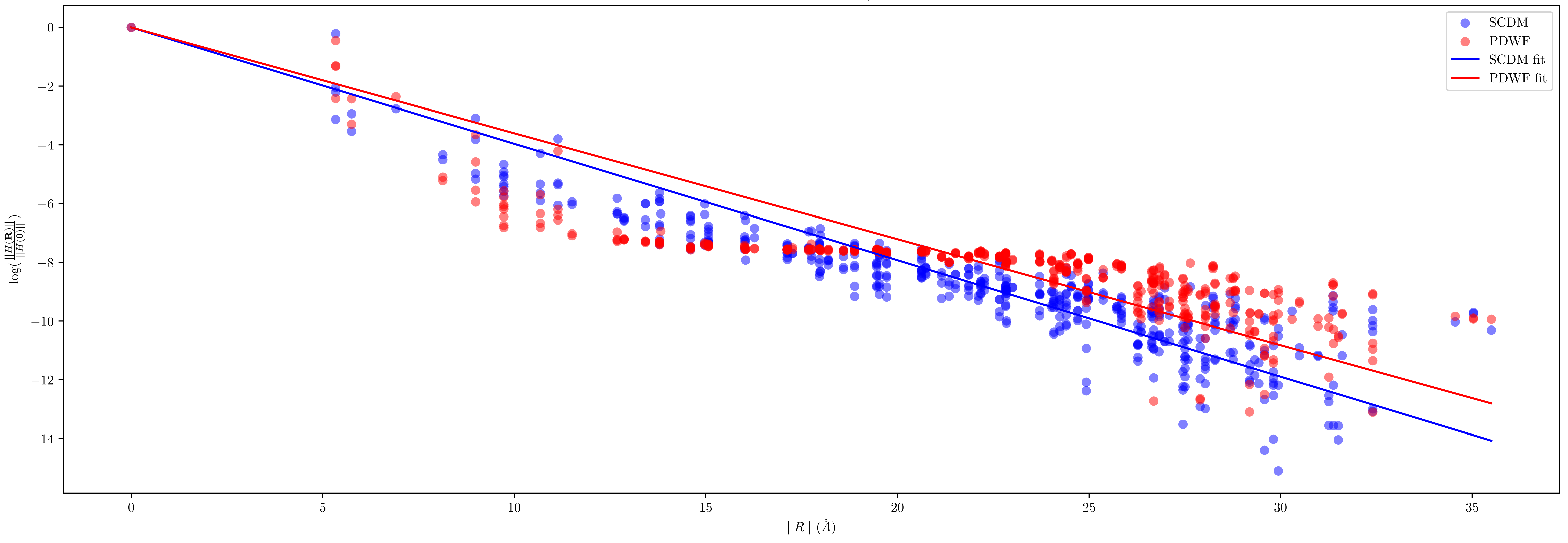

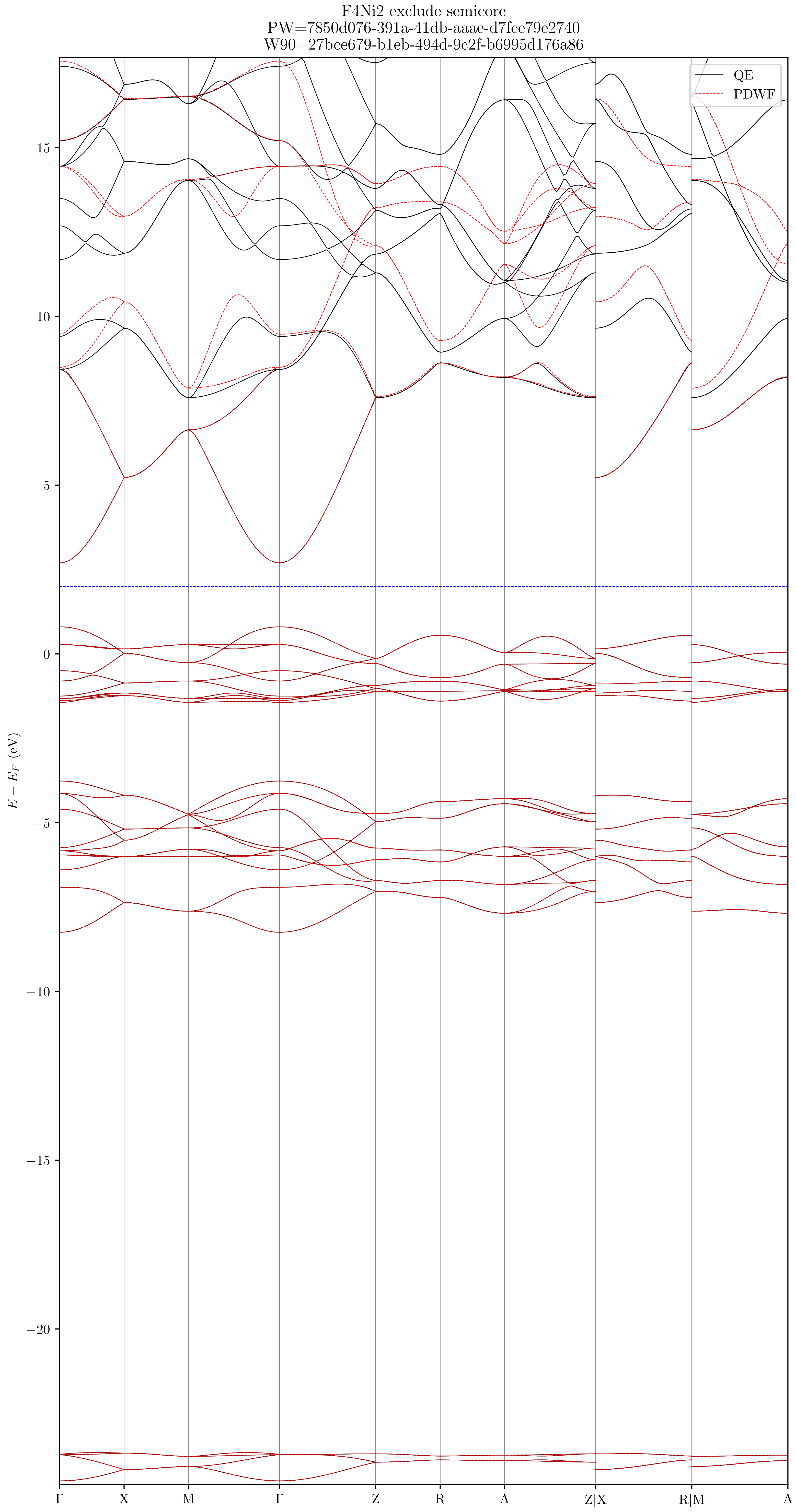

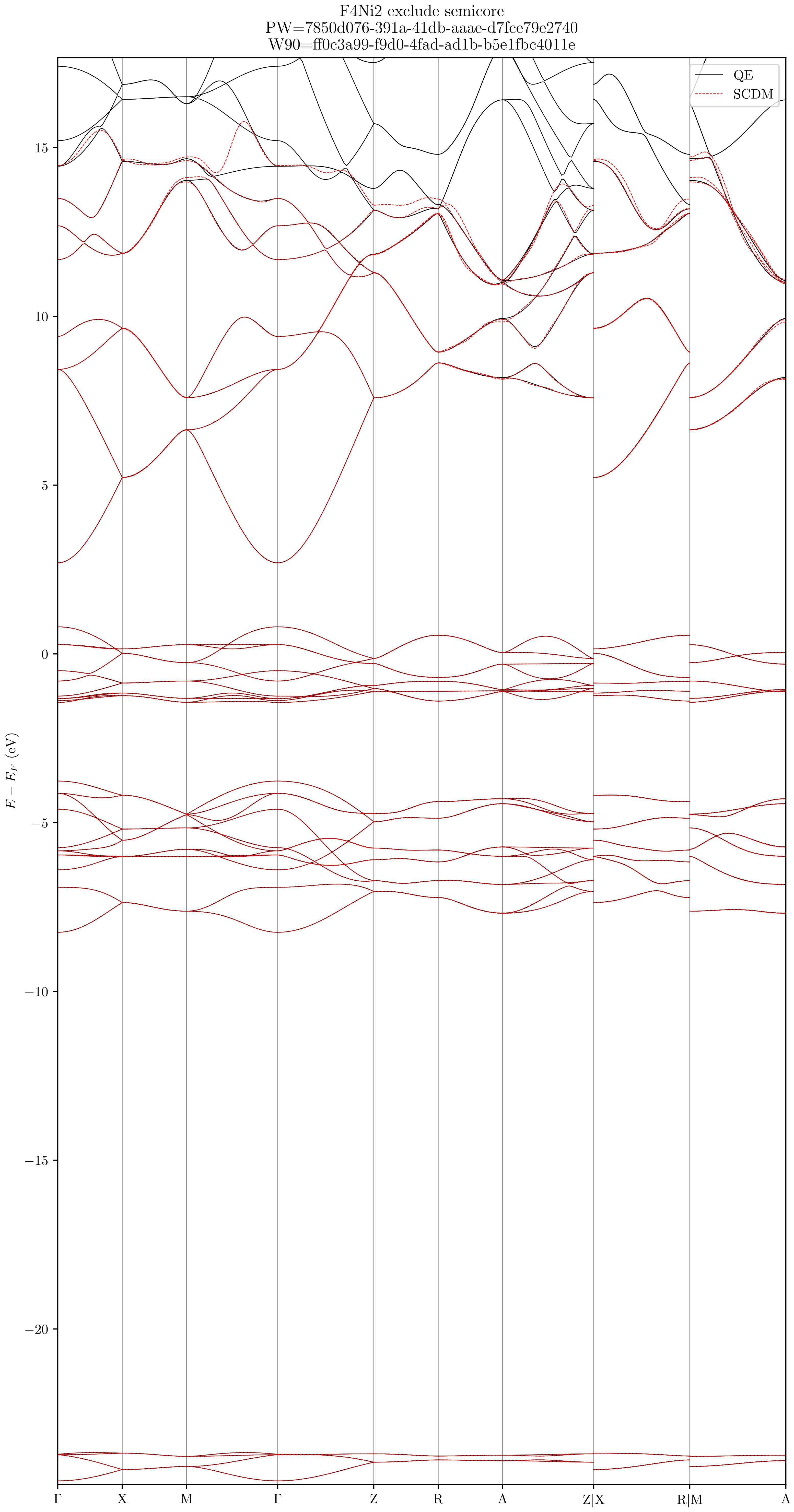

PDWF band distance (meV) and τ ($\AA$)

η_0	η_2	η_4	$\eta_0^{\max}$	$\eta_2^{\max}$	$\eta_4^{\max}$	τ
0.3	0.3	0.3	2.4	2.4	2.4	2.026

SCDM band distance (meV) and τ ($\AA$)

η_0	η_2	η_4	$\eta_0^{\max}$	$\eta_2^{\max}$	$\eta_4^{\max}$	τ
1.3	1.2	1.2	11.5	11.5	11.5	2.401

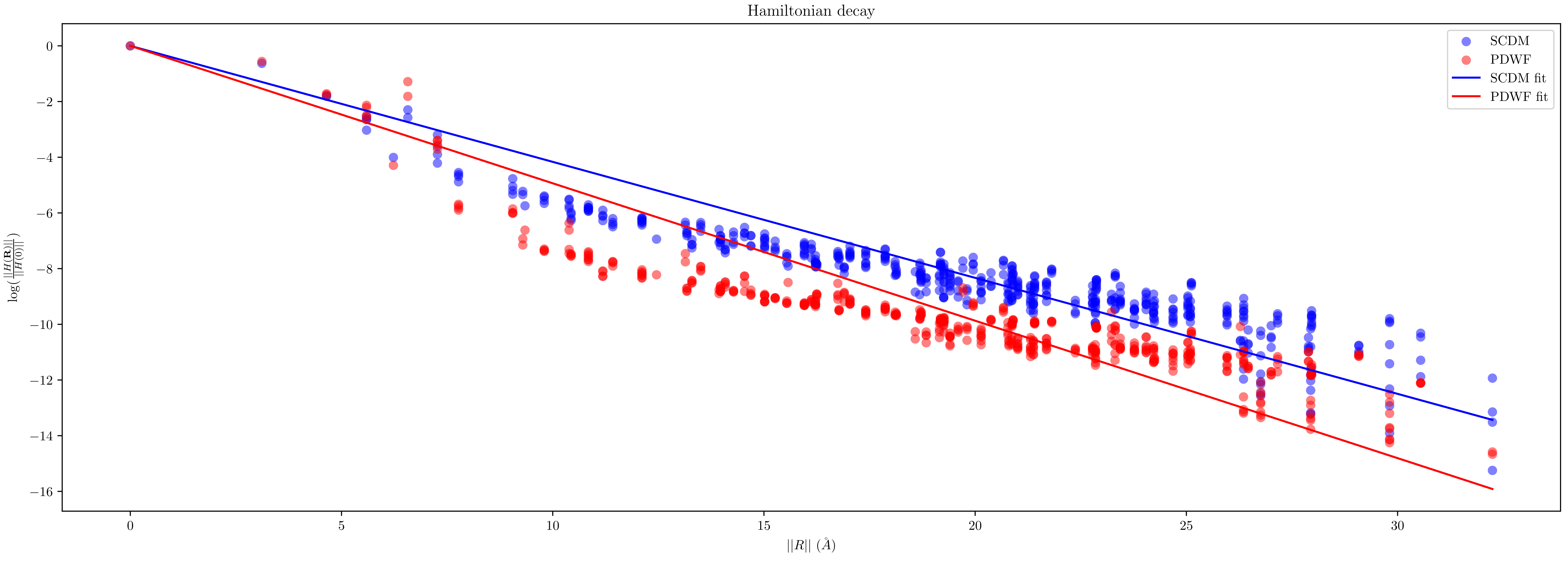

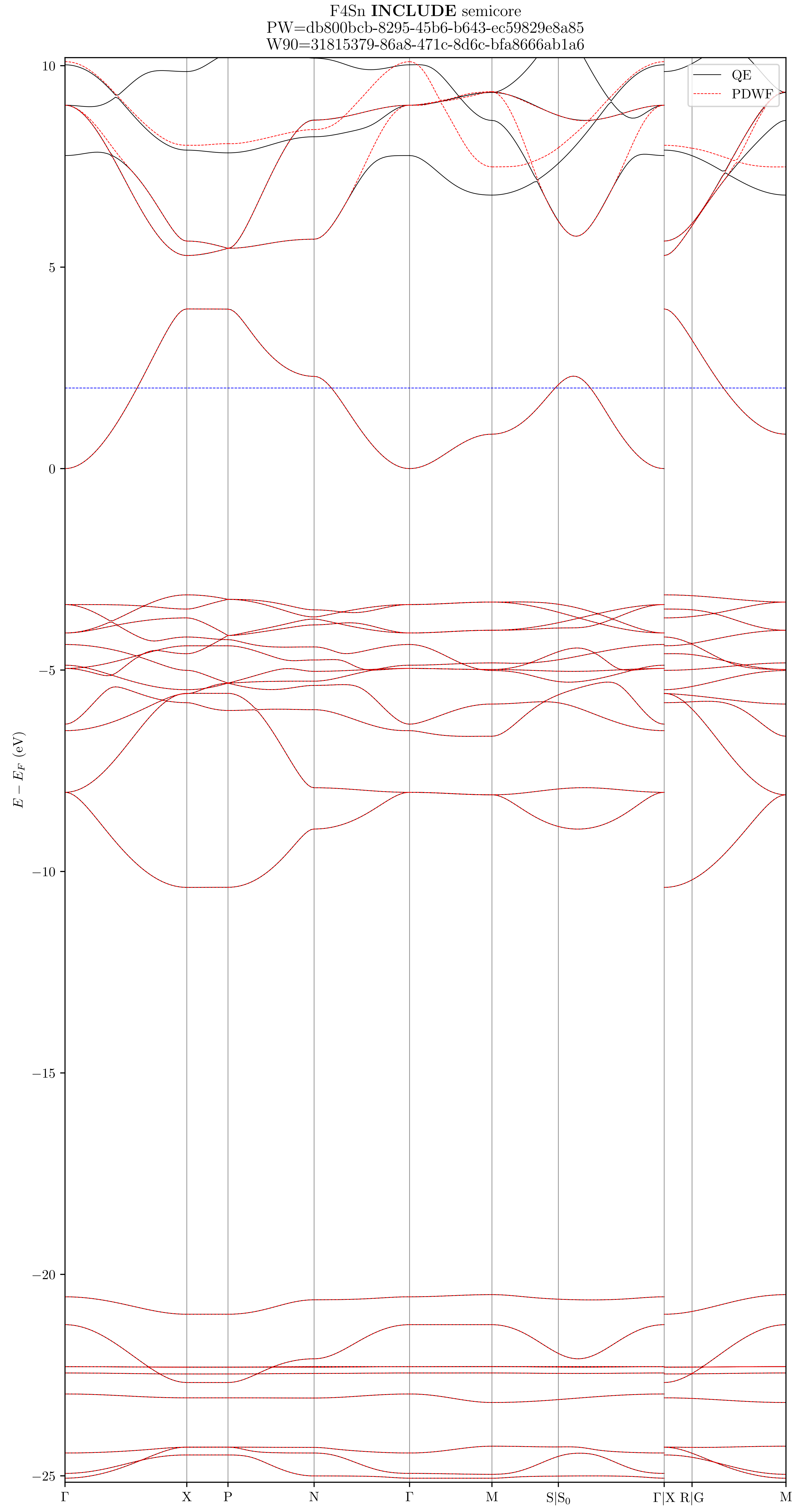

PDWF band distance (meV) and τ ($\AA$)

η_0	η_2	η_4	$\eta_0^{\max}$	$\eta_2^{\max}$	$\eta_4^{\max}$	τ
0.4	0.4	0.4	2.7	2.7	3.8	2.328

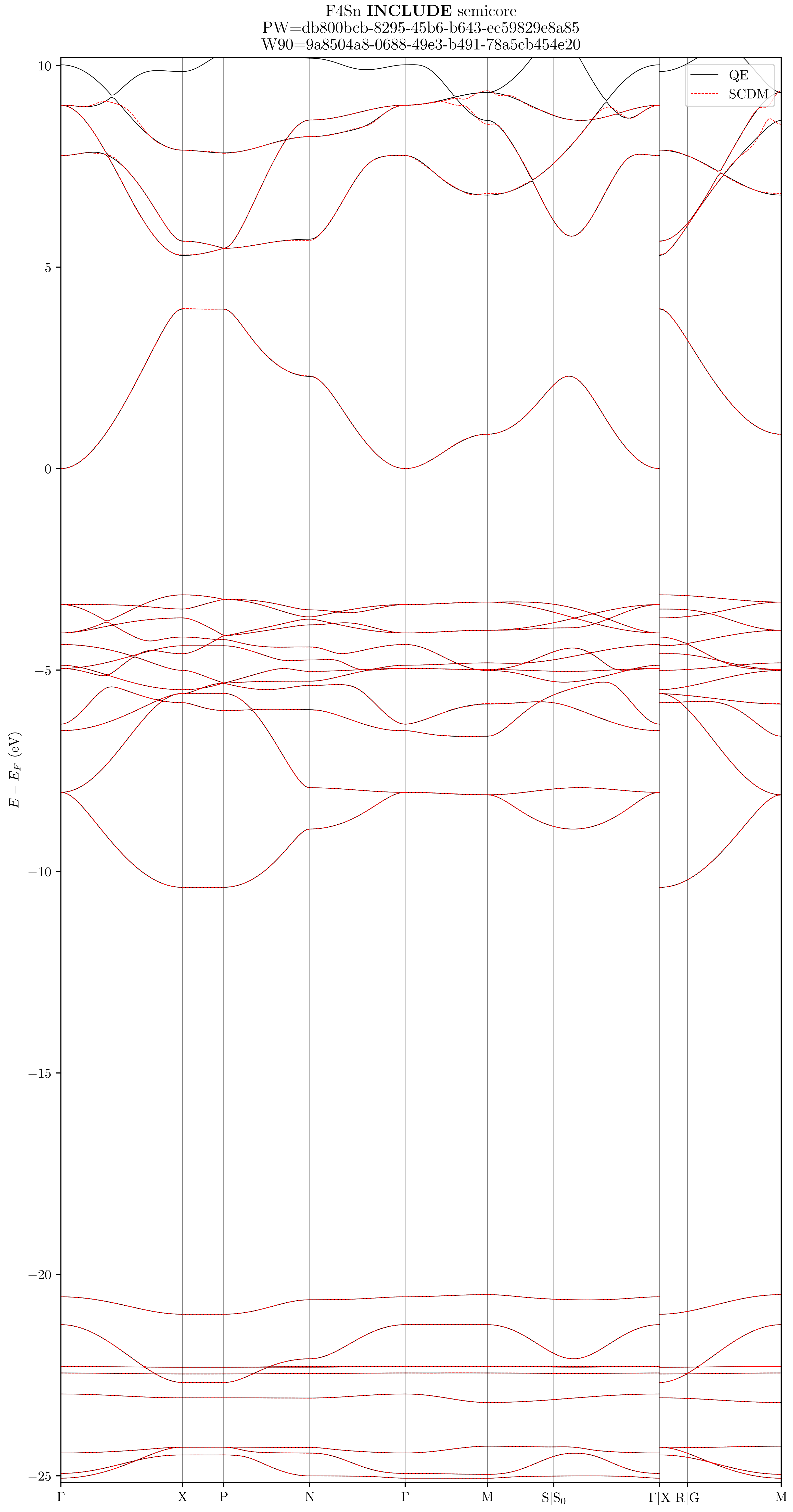

SCDM band distance (meV) and τ ($\AA$)

η_0	η_2	η_4	$\eta_0^{\max}$	$\eta_2^{\max}$	$\eta_4^{\max}$	τ
1.2	1.3	1.4	14.7	14.7	14.7	2.614

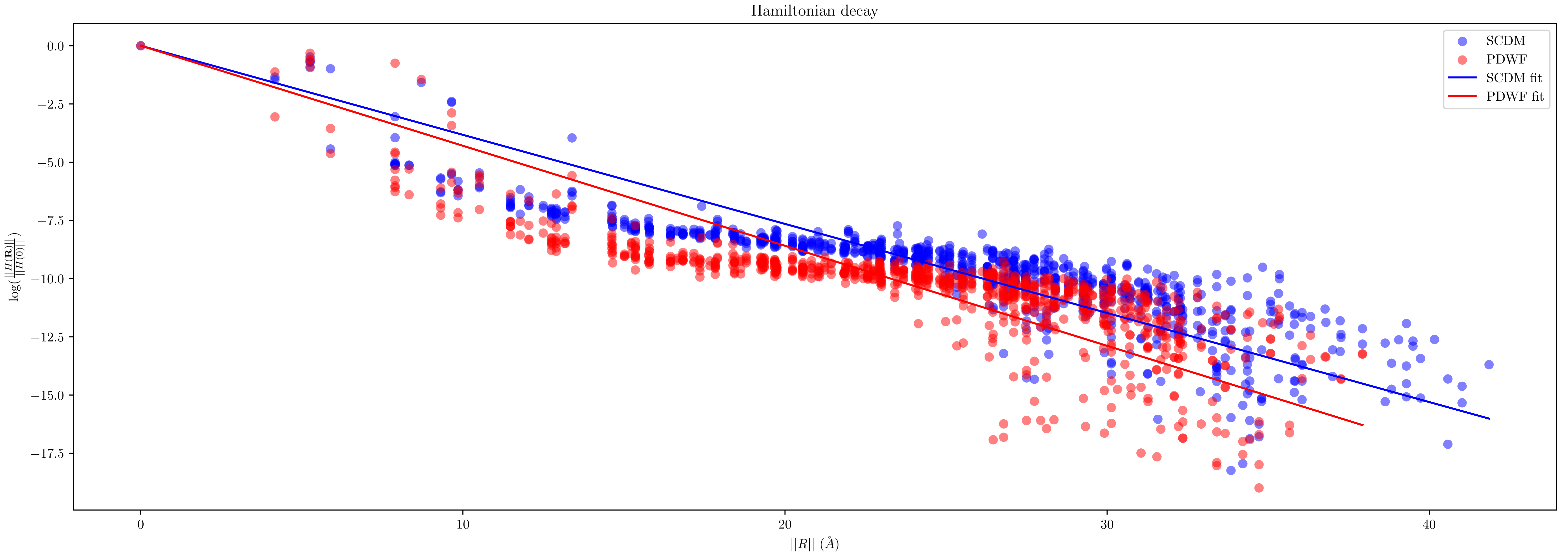

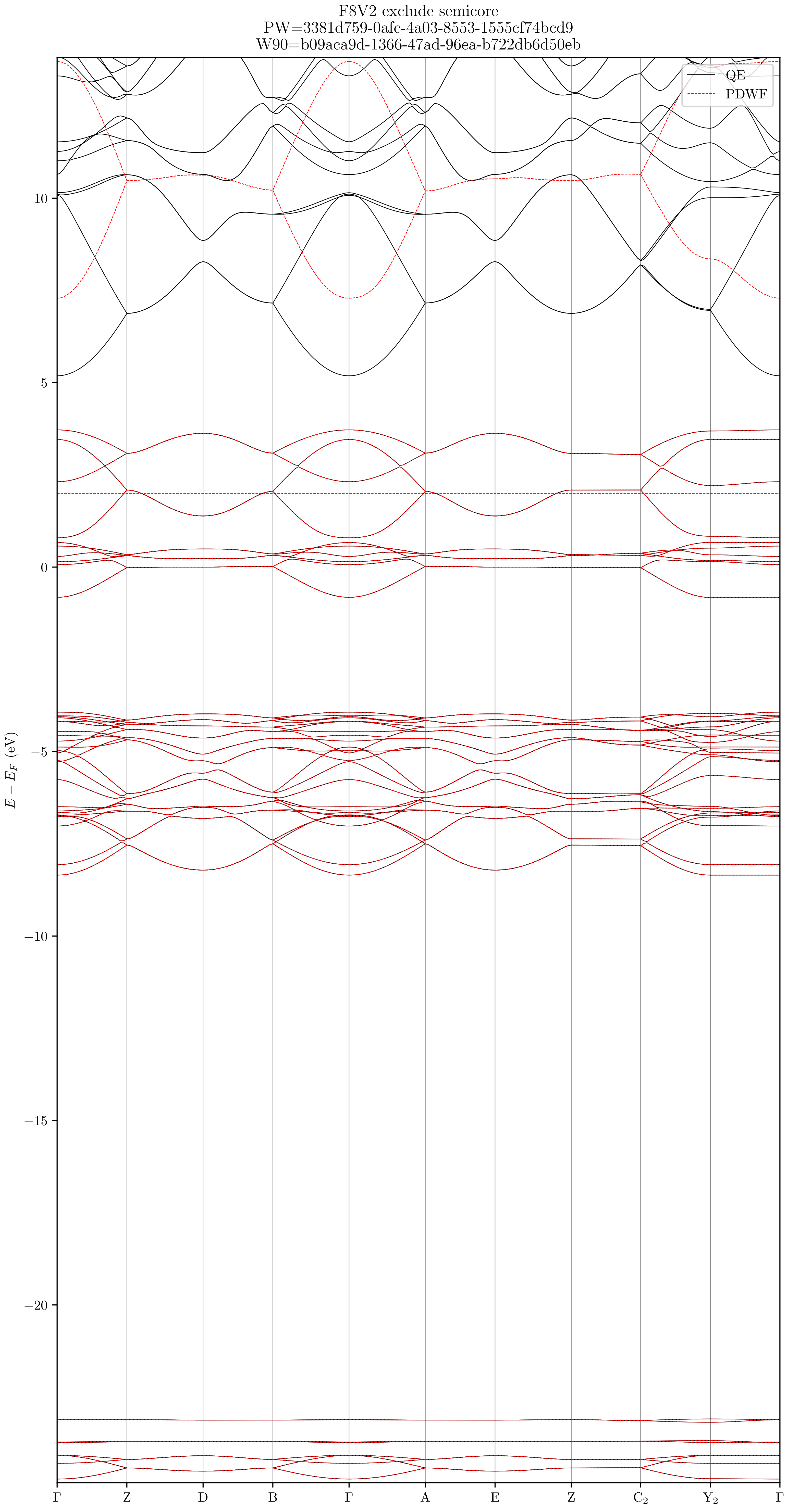

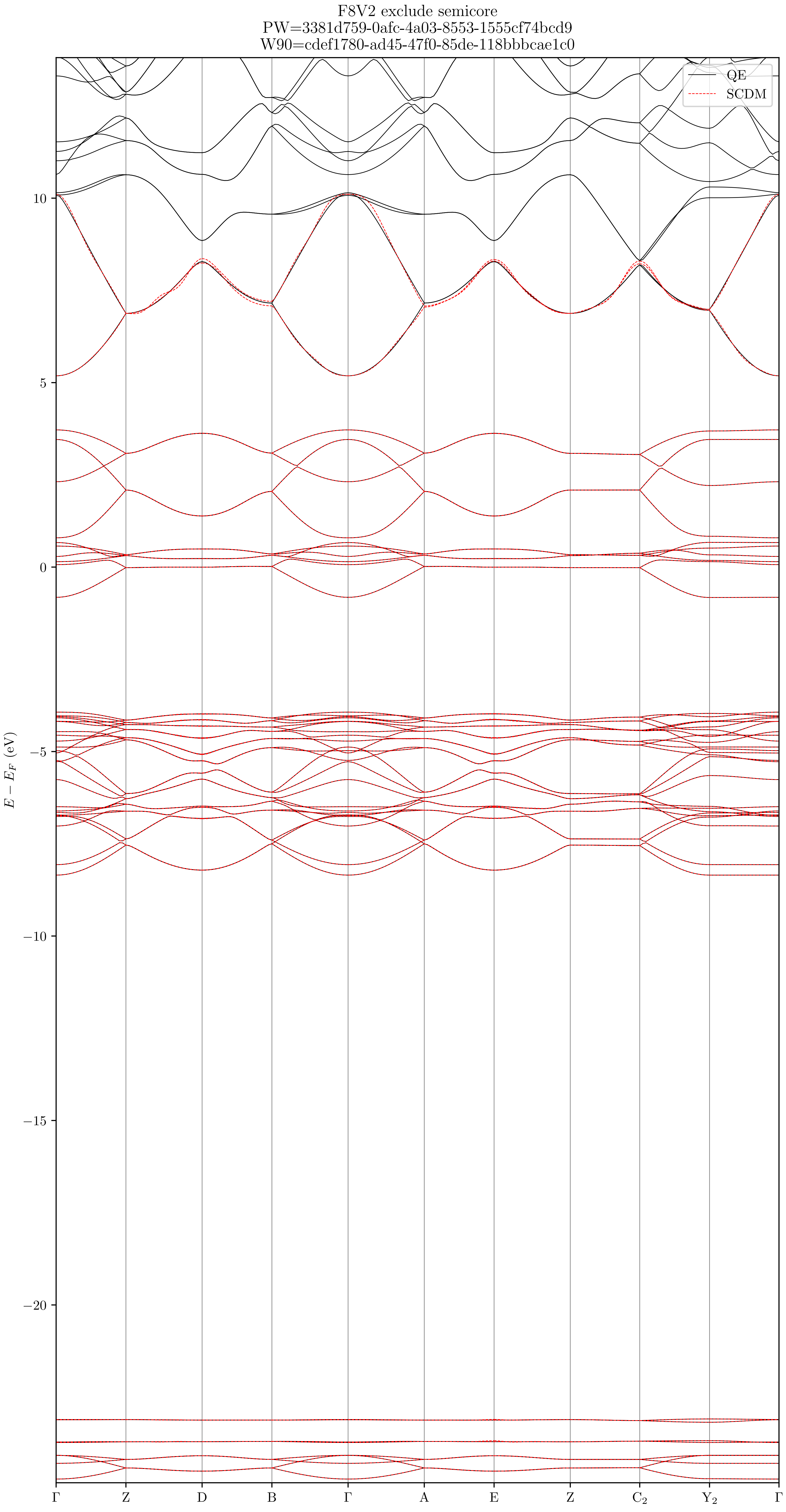

PDWF band distance (meV) and τ (Å)

η_0	η_2	η_4	$\eta_0^{\max}$	$\eta_2^{\max}$	$\eta_4^{\max}$	τ
0.0	0.0	0.0	0.3	0.3	0.3	1.914

SCDM band distance (meV) and τ (Å)

η_0	η_2	η_4	$\eta_0^{\max}$	$\eta_2^{\max}$	$\eta_4^{\max}$	τ
1.8	1.6	1.6	26.1	26.1	26.1	2.391

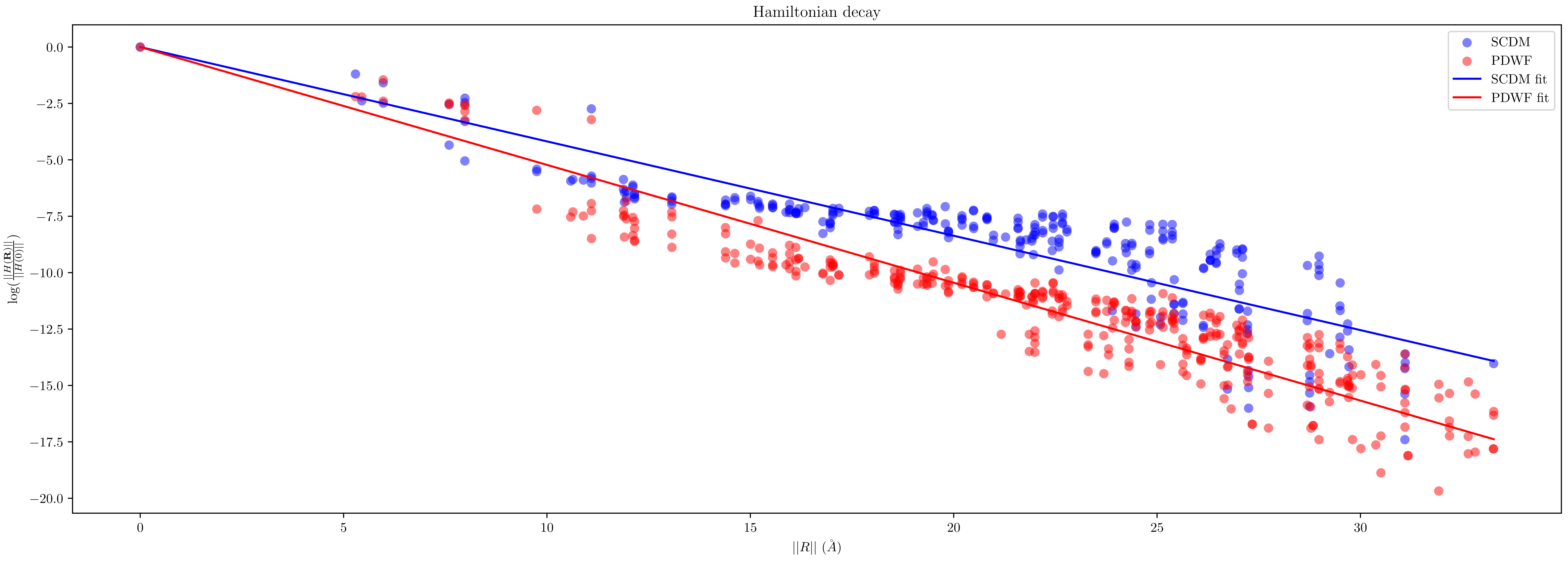

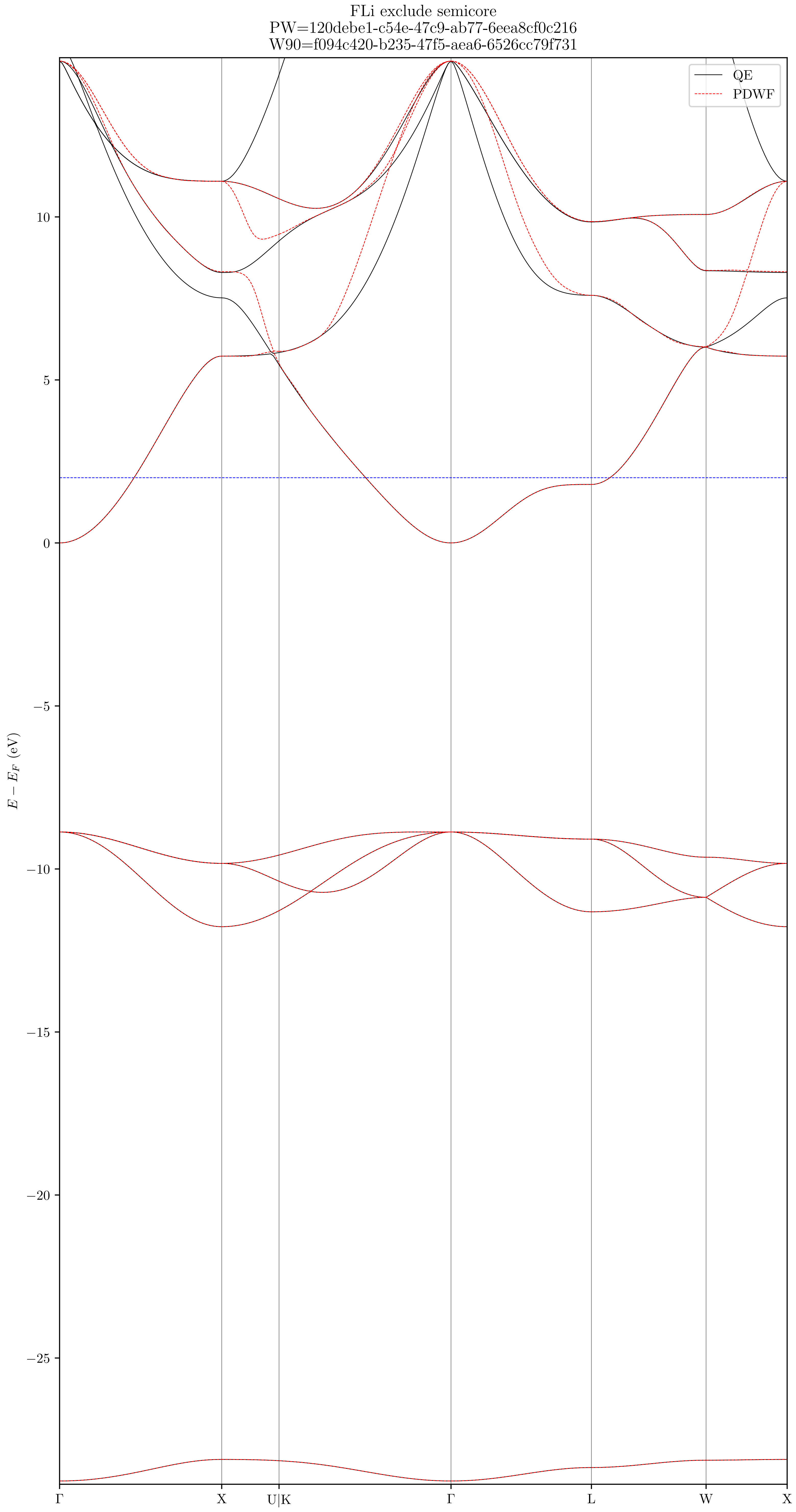

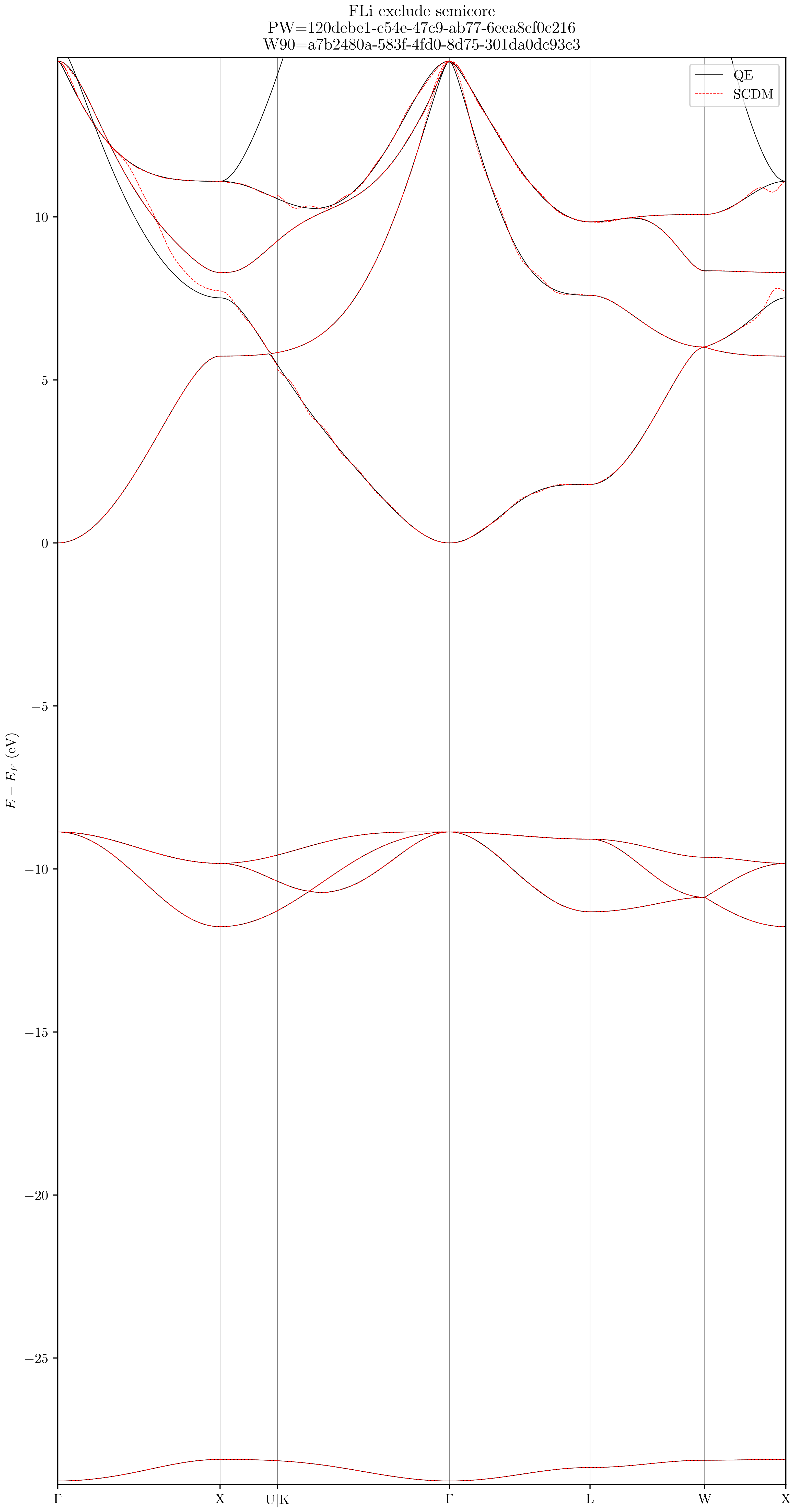

PDWF band distance (meV) and τ ($\AA$)

η_0	η_2	η_4	$\eta_0^{\max}$	$\eta_2^{\max}$	$\eta_4^{\max}$	τ
0.3	0.4	0.7	1.2	2.6	5.5	1.859

SCDM band distance (meV) and τ ($\AA$)

η_0	η_2	η_4	$\eta_0^{\max}$	$\eta_2^{\max}$	$\eta_4^{\max}$	τ
2.4	3.9	6.4	15.3	31.0	49.4	2.001

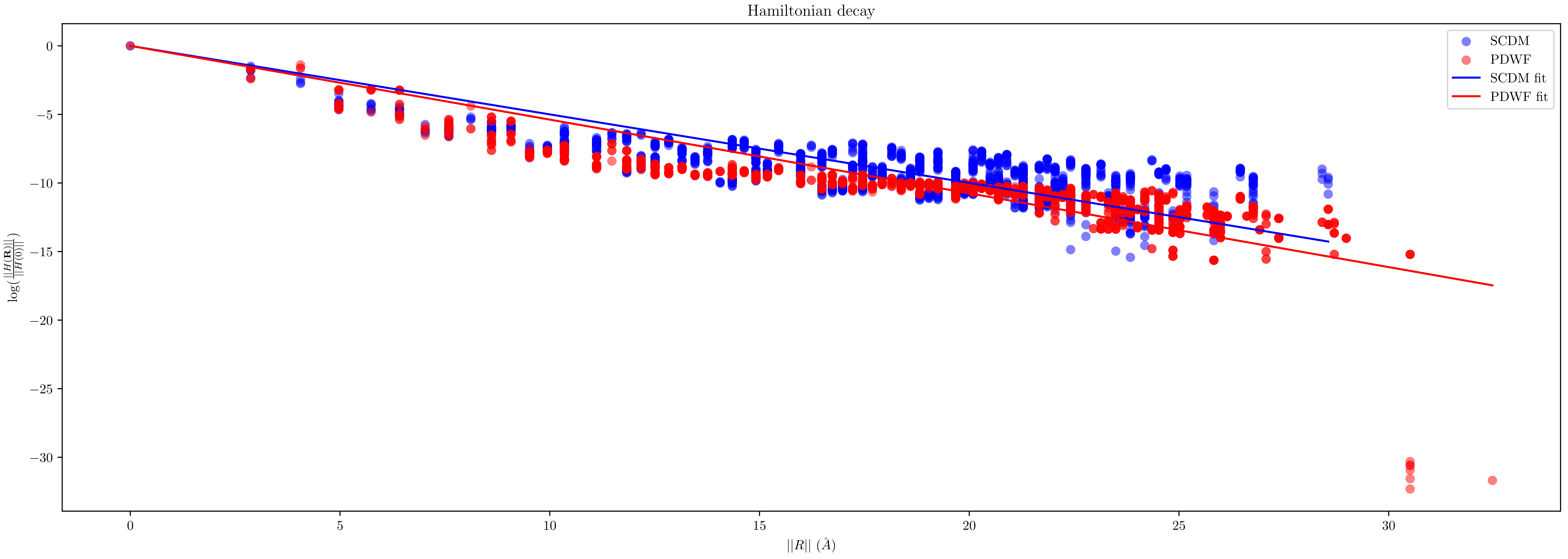

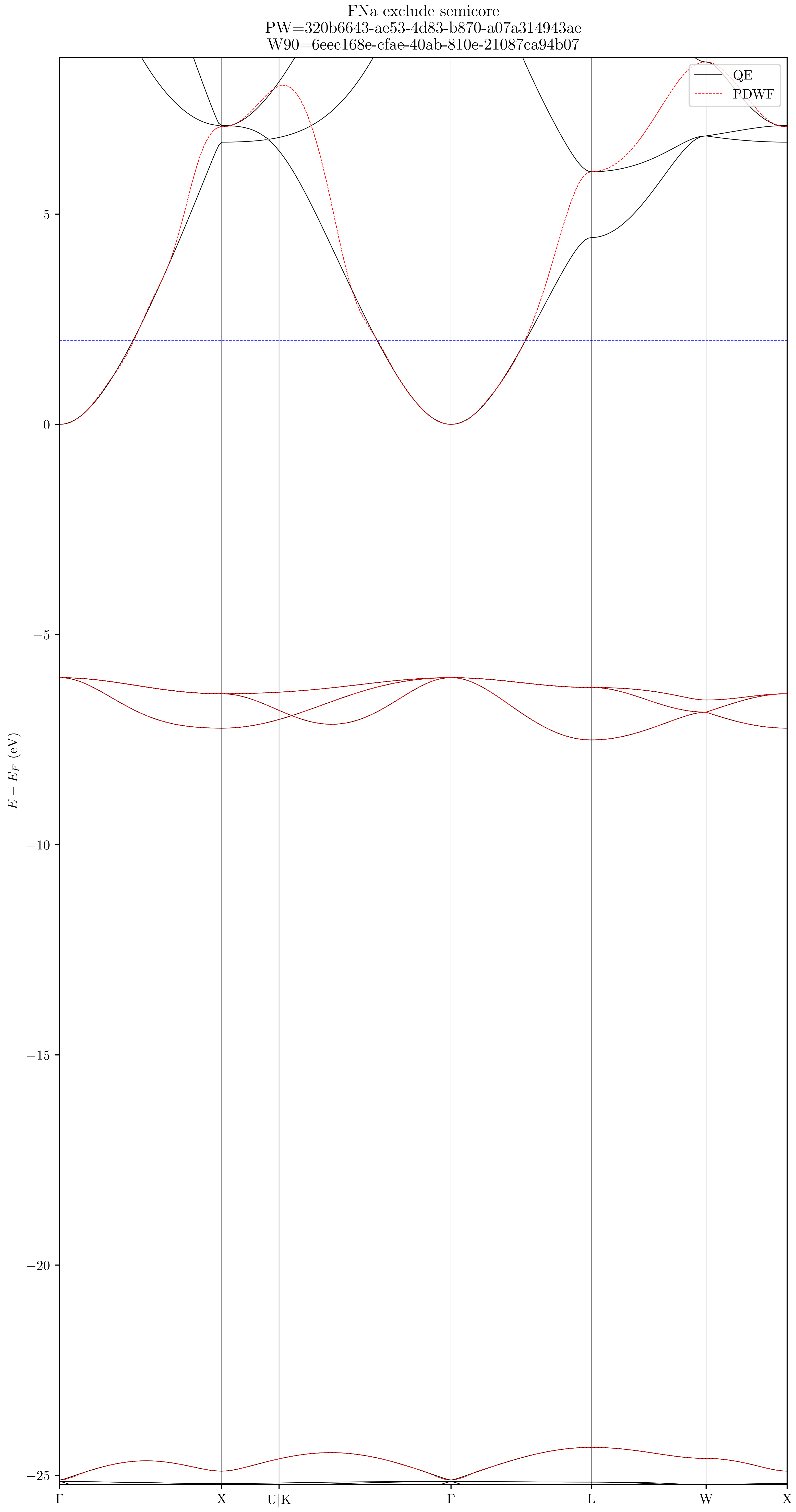

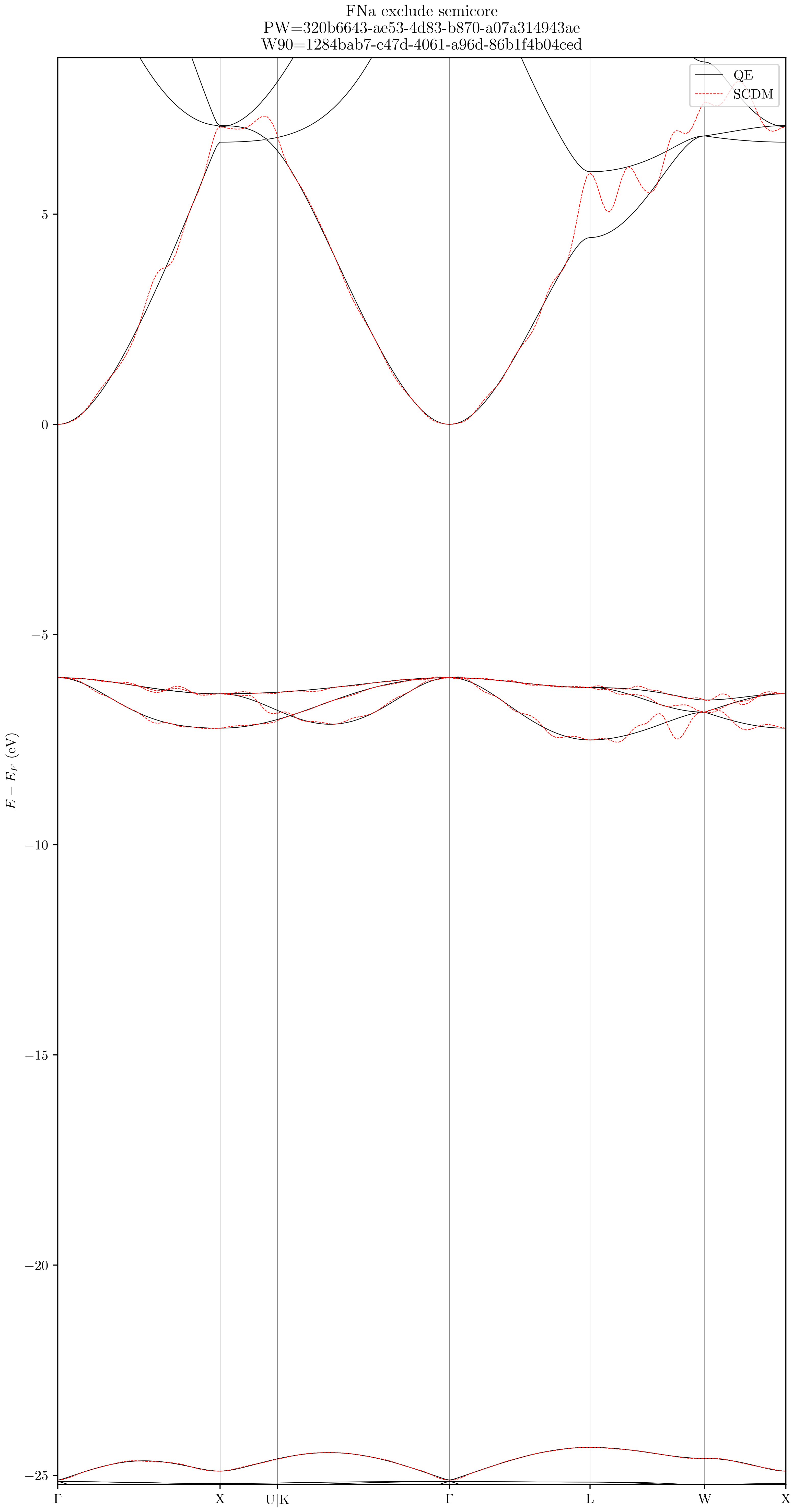

PDWF band distance (meV) and τ ($\AA$)

η_0	η_2	η_4	$\eta_0^{\max}$	$\eta_2^{\max}$	$\eta_4^{\max}$	τ
2.5	2.7	7.0	22.5	22.5	55.9	1.895

SCDM band distance (meV) and τ ($\AA$)

η_0	η_2	η_4	$\eta_0^{\max}$	$\eta_2^{\max}$	$\eta_4^{\max}$	τ
51.9	51.3	51.5	503.1	503.1	503.1	2.885

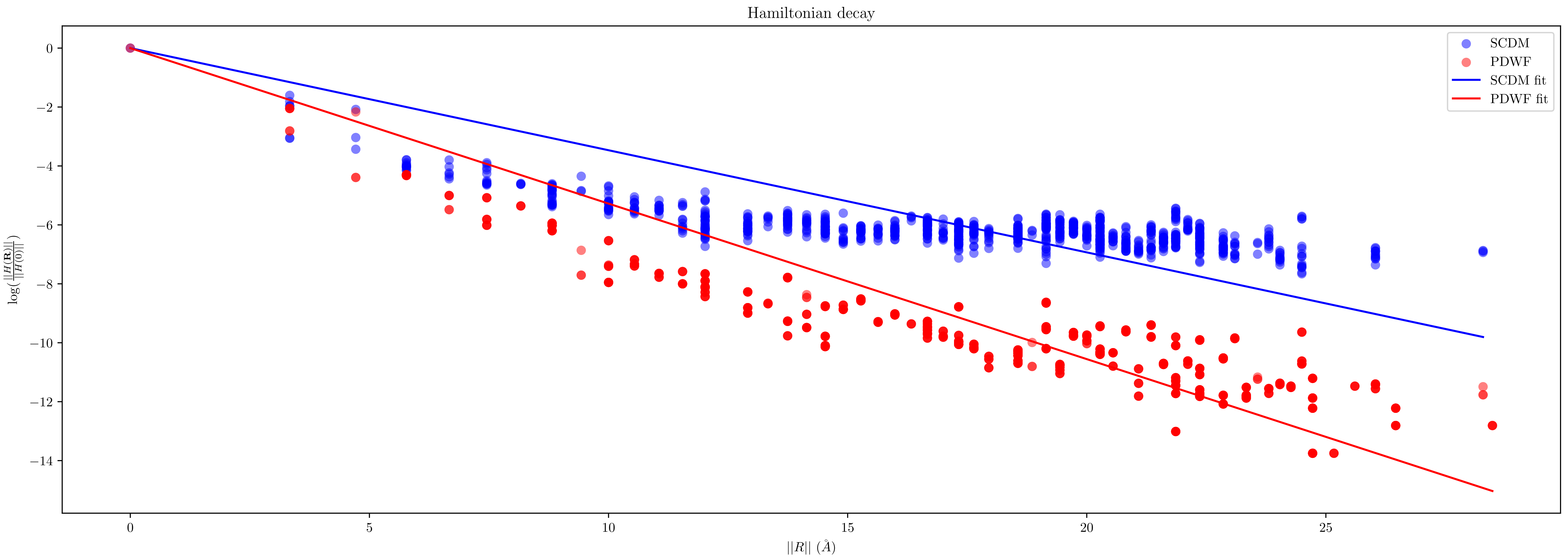

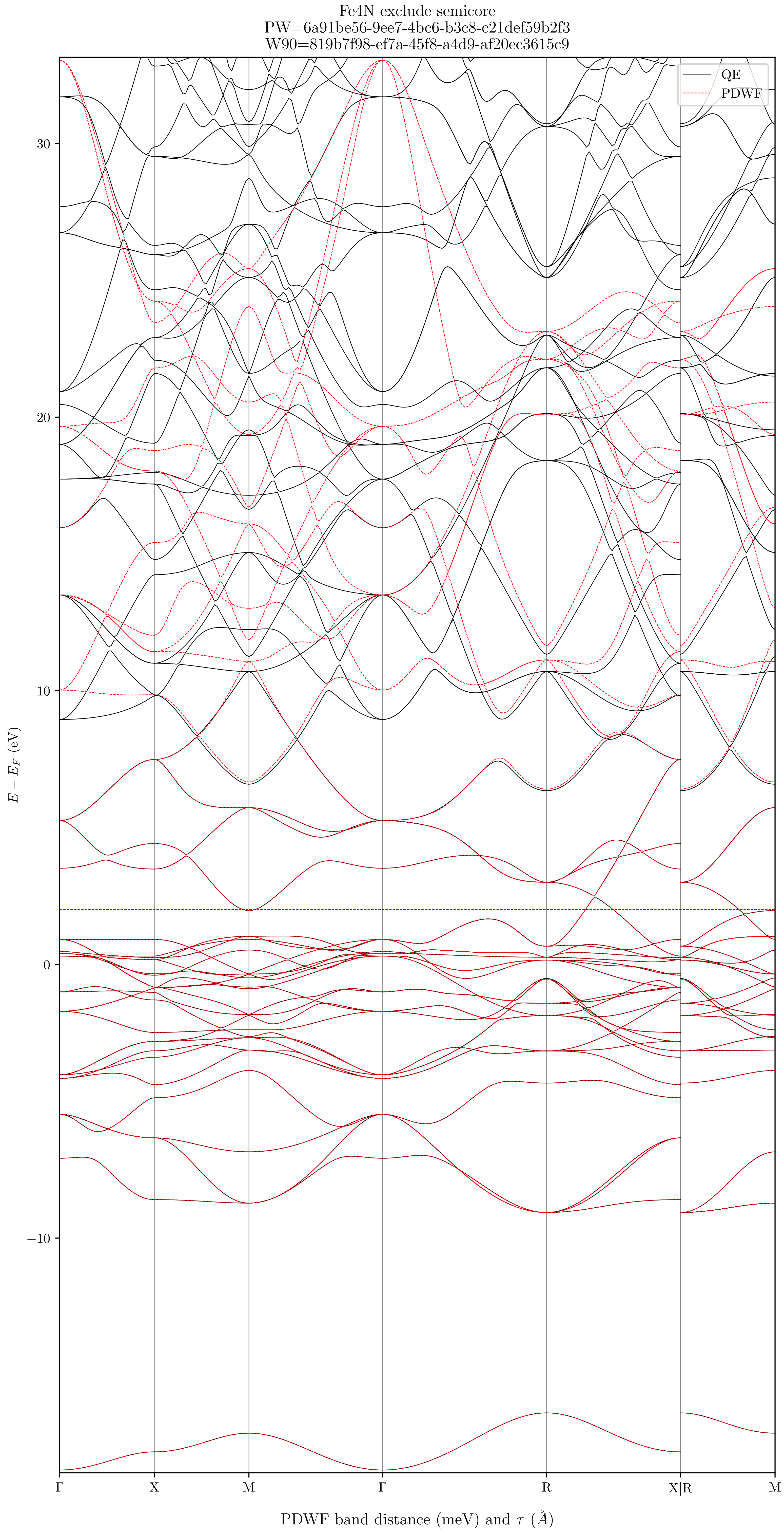

η_0	η_2	η_4	$\eta_0^{\max}$	$\eta_2^{\max}$	$\eta_4^{\max}$	τ
1.7	1.5	1.6	18.9	19.0	19.0	1.928

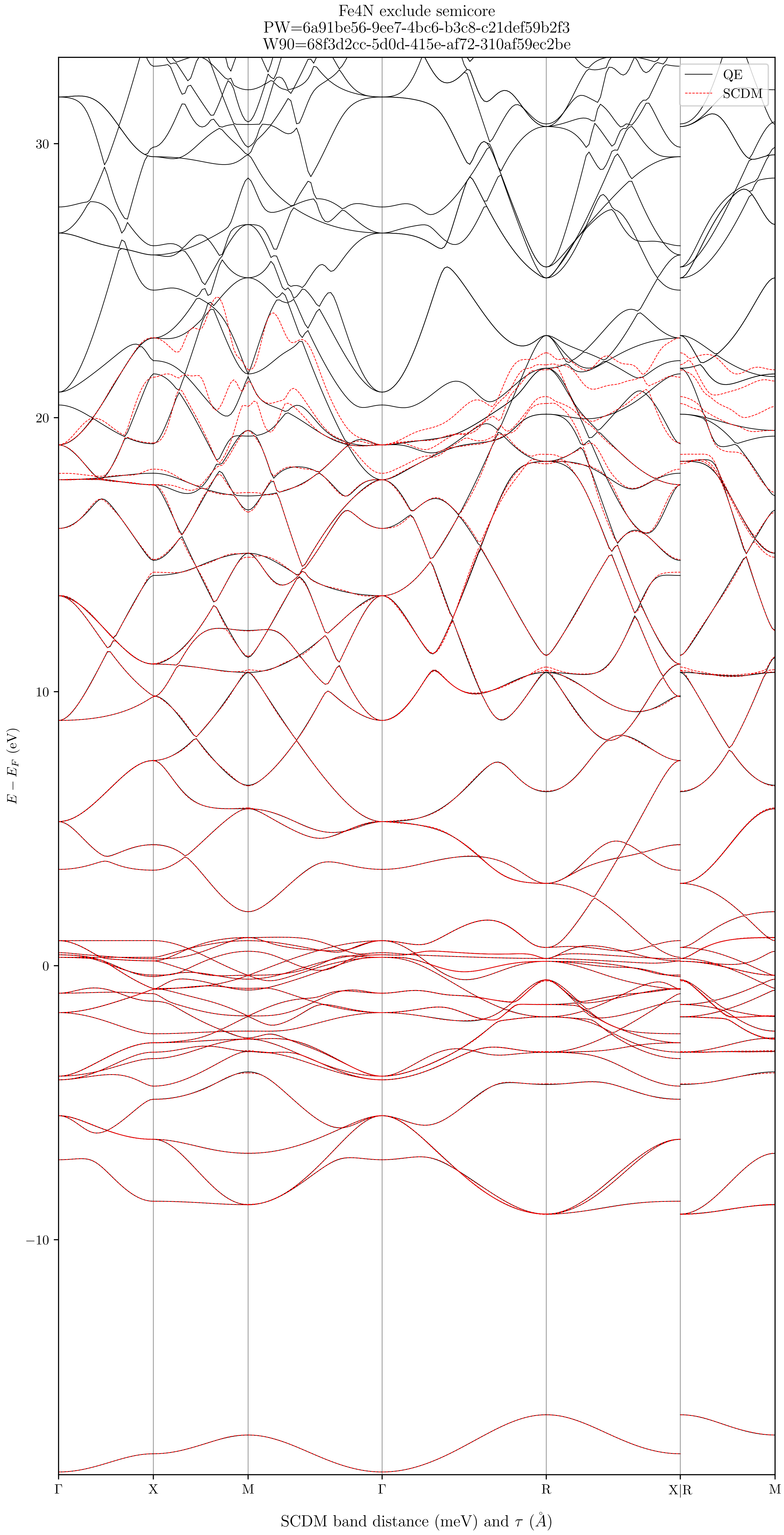

η_0	η_2	η_4	$\eta_0^{\max}$	$\eta_2^{\max}$	$\eta_4^{\max}$	τ
5.4	4.8	4.8	41.0	41.0	41.0	2.476

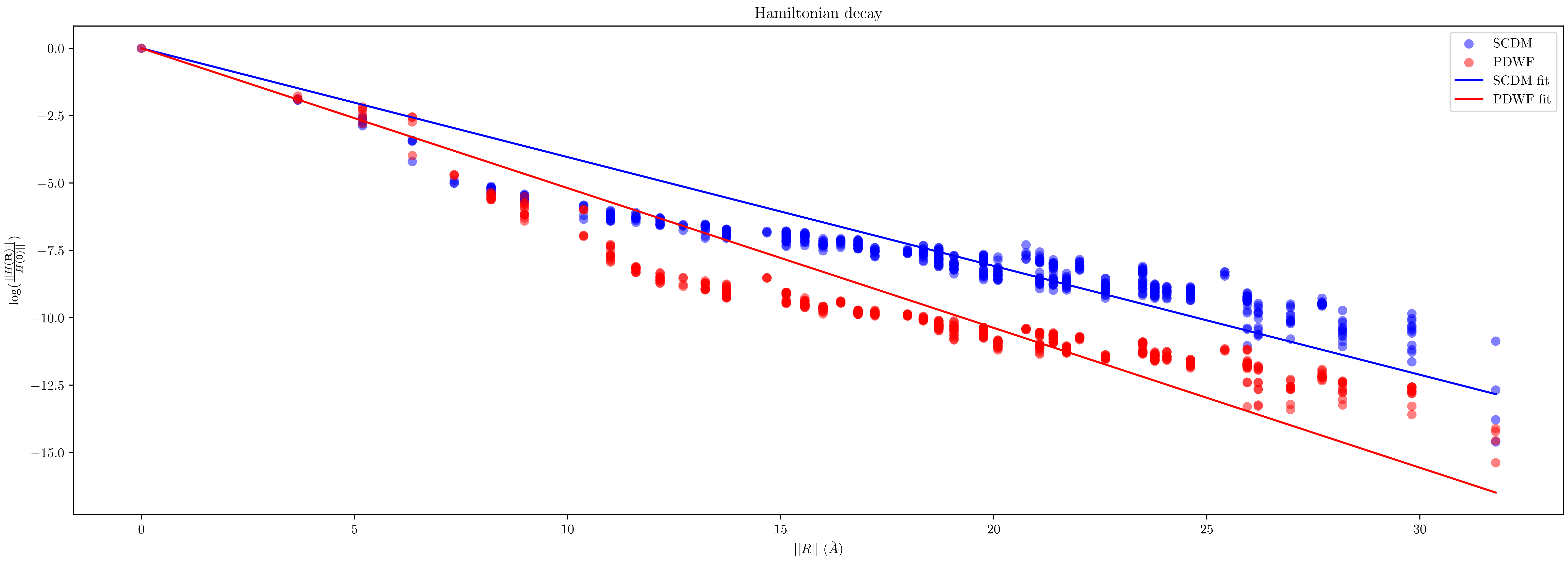

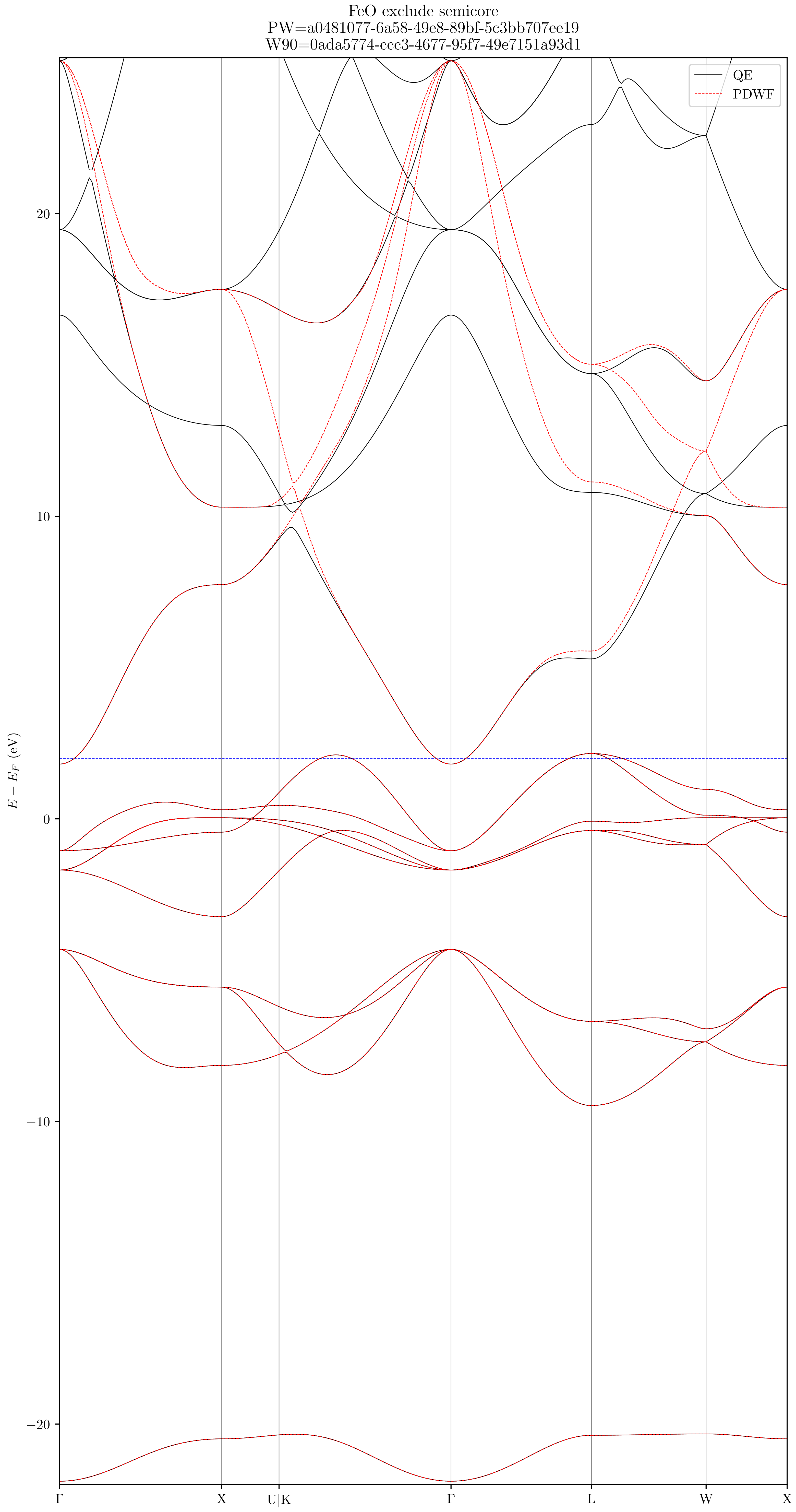

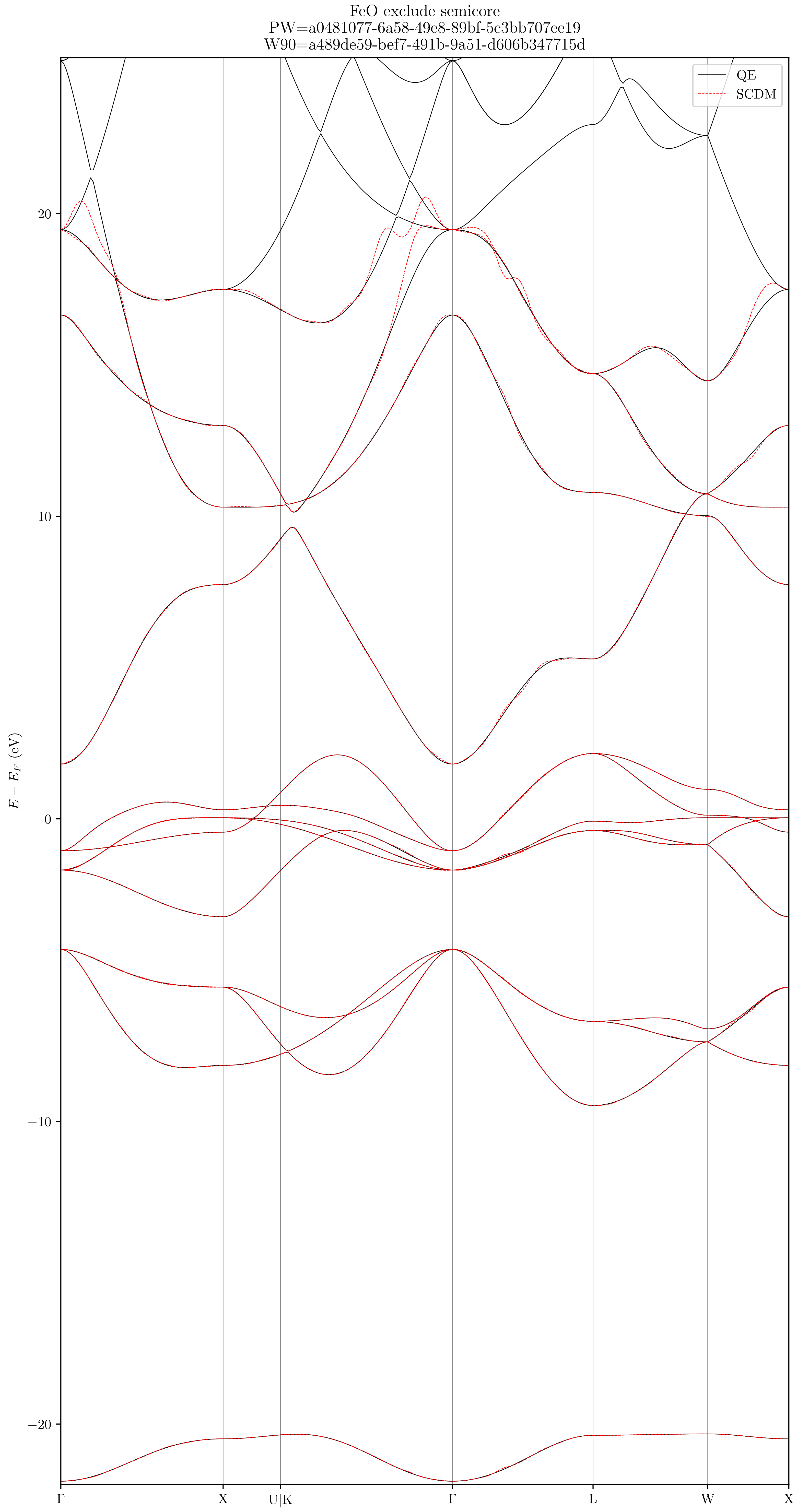

PDWF band distance (meV) and τ ($\AA$)

η_0	η_2	η_4	$\eta_0^{\max}$	$\eta_2^{\max}$	$\eta_4^{\max}$	τ
0.5	0.5	0.6	3.3	3.3	7.8	1.817

SCDM band distance (meV) and τ ($\AA$)

η_0	η_2	η_4	$\eta_0^{\max}$	$\eta_2^{\max}$	$\eta_4^{\max}$	τ
7.4	7.4	8.5	52.9	52.9	86.6	2.304

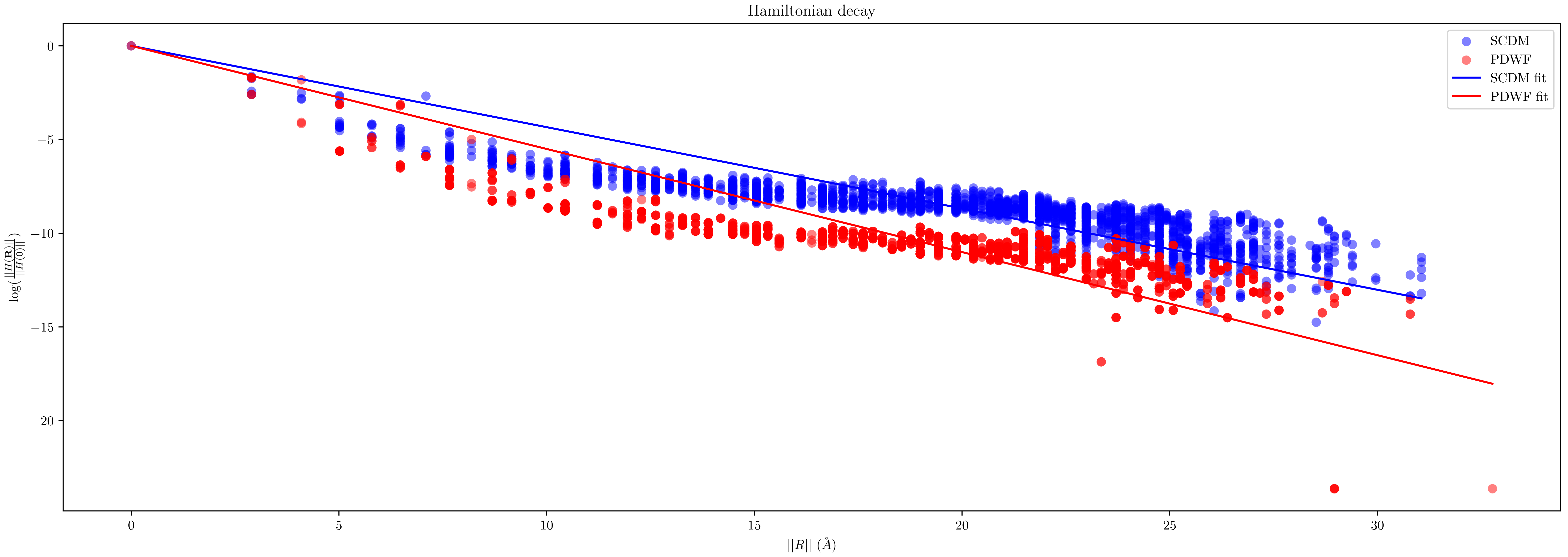

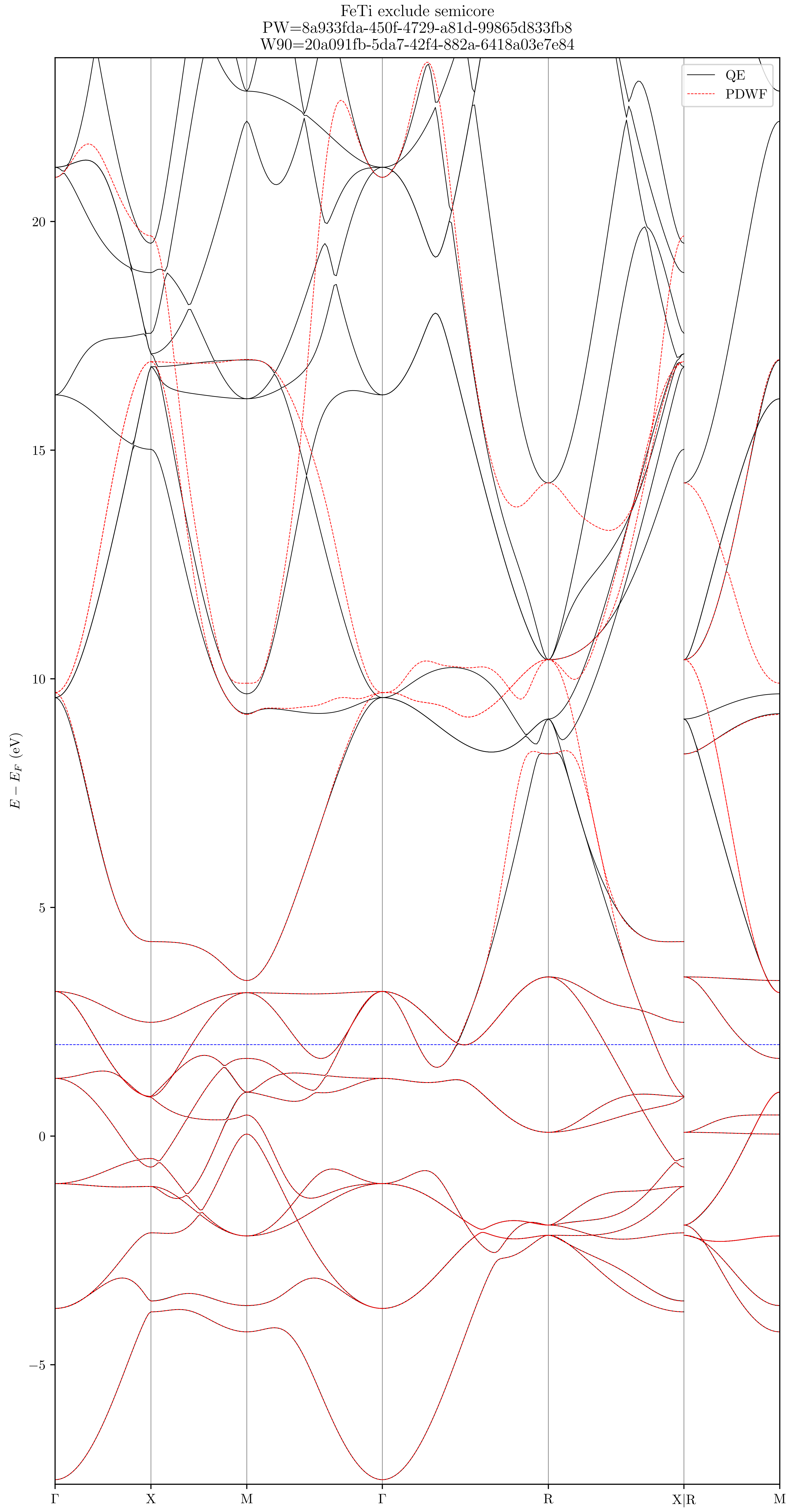

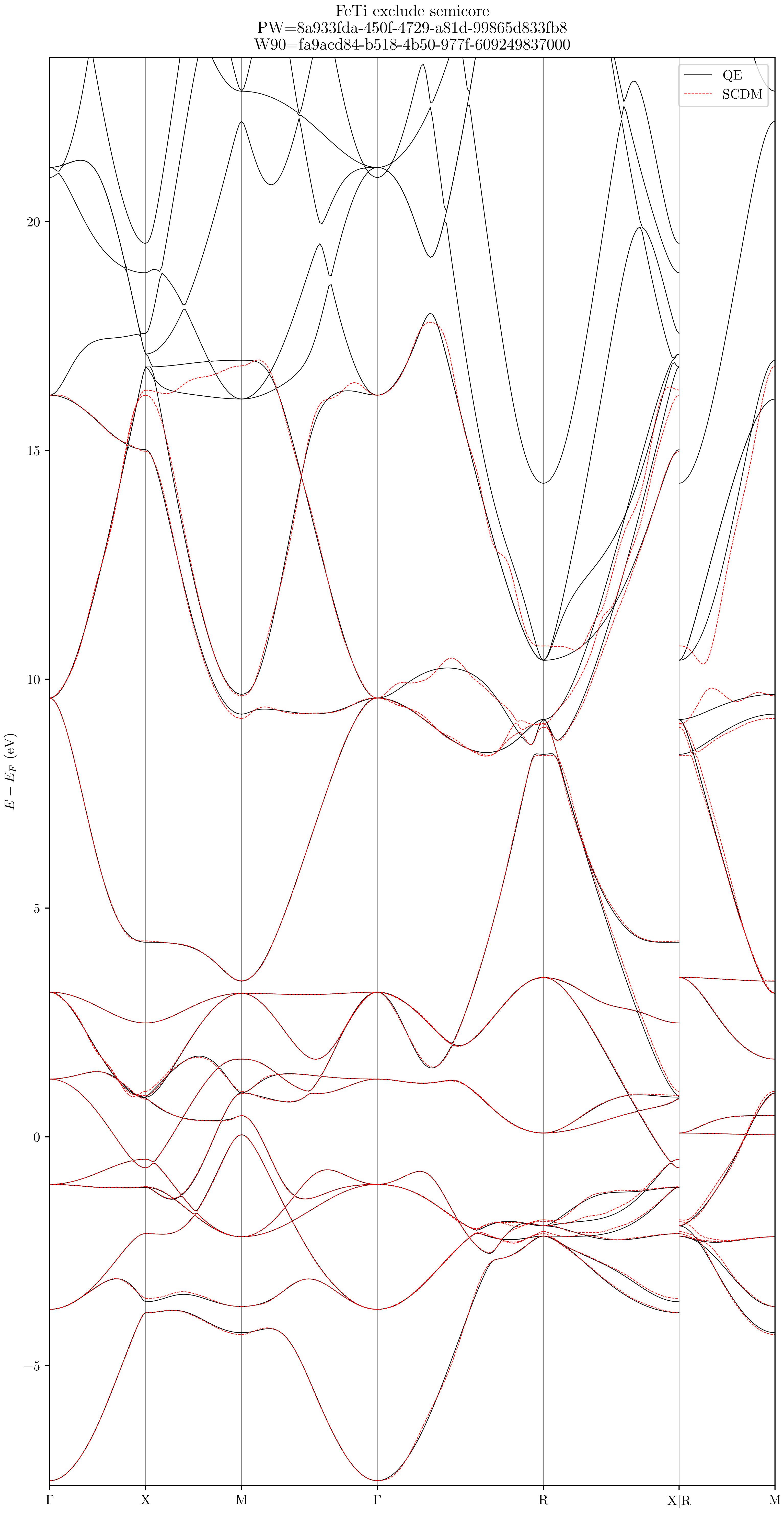

PDWF band distance (meV) and τ ($\AA$)

η_0	η_2	η_4	$\eta_0^{\max}$	$\eta_2^{\max}$	$\eta_4^{\max}$	τ
1.4	1.8	3.1	7.7	19.7	34.9	1.848

SCDM band distance (meV) and τ ($\AA$)

η_0	η_2	η_4	$\eta_0^{\max}$	$\eta_2^{\max}$	$\eta_4^{\max}$	τ
31.8	29.4	28.6	160.1	160.1	160.1	2.261

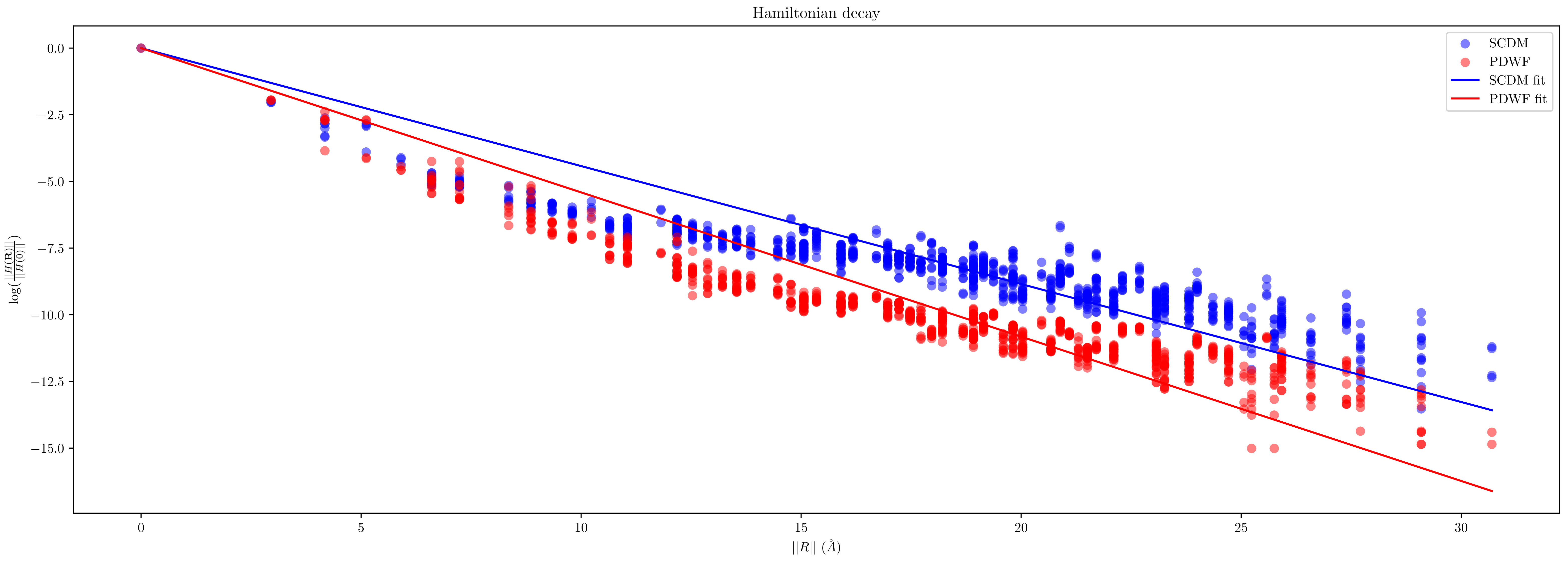

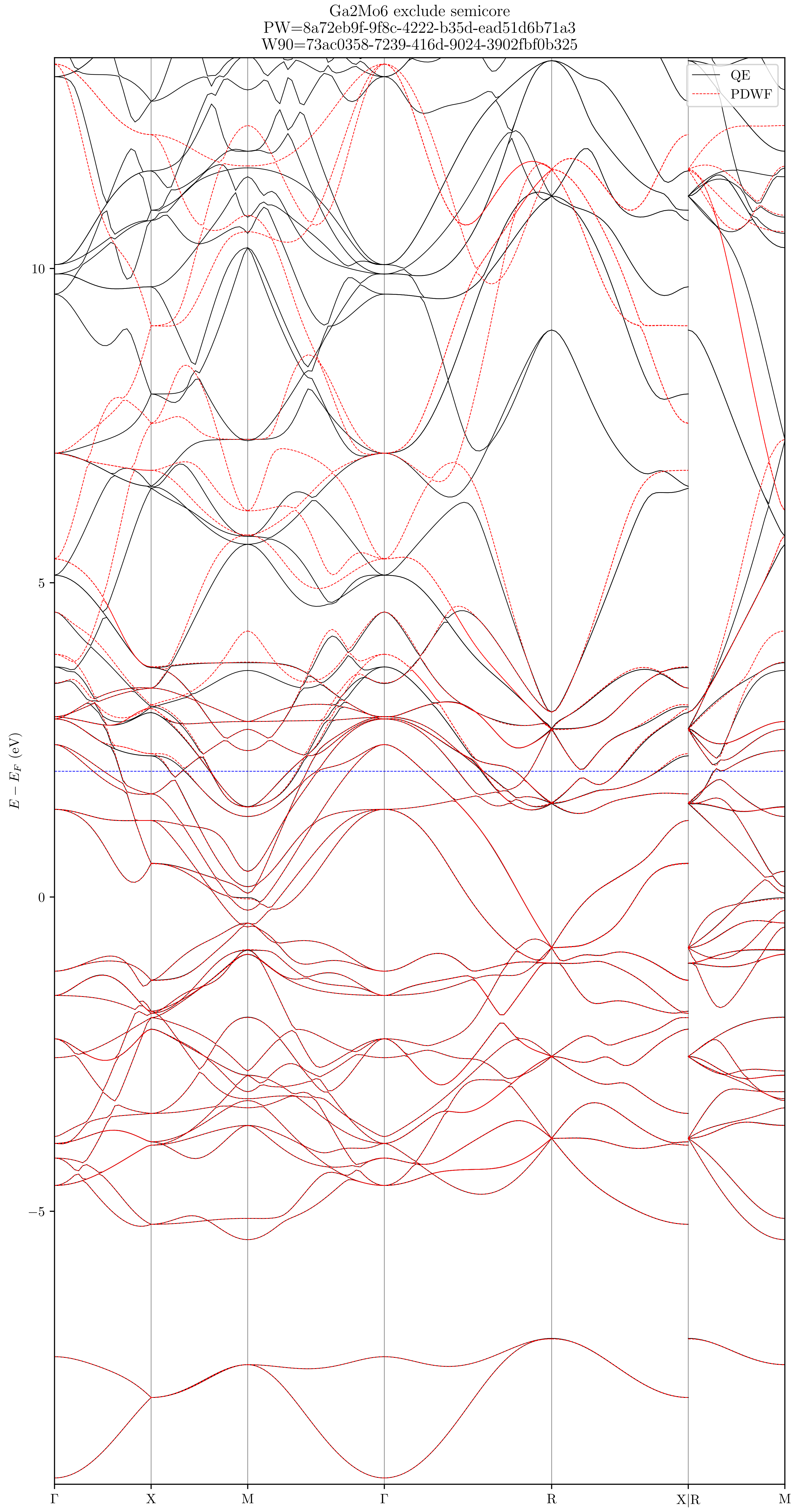

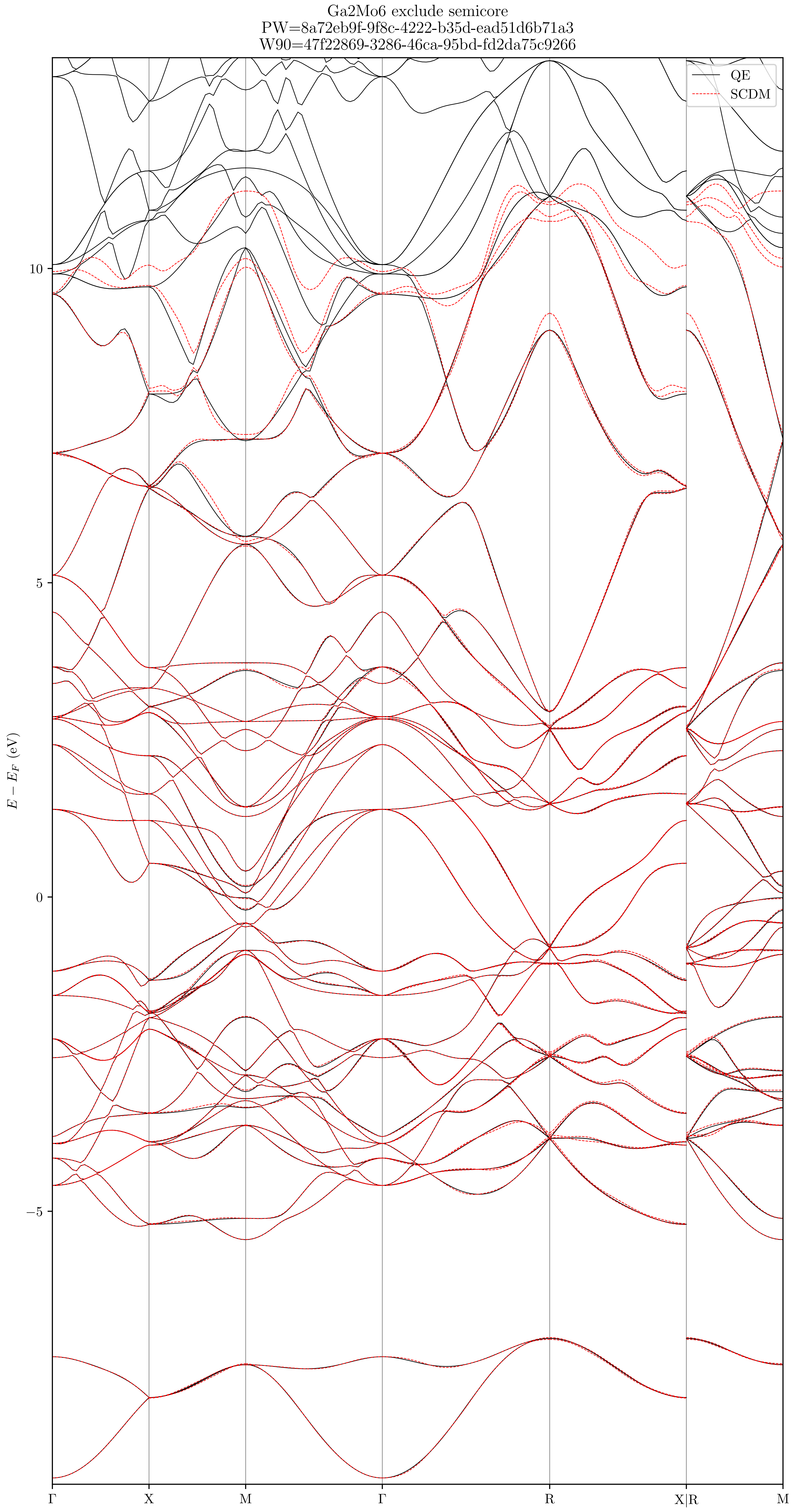

PDWF band distance (meV) and τ ($\AA$)

η_0	η_2	η_4	$\eta_0^{\max}$	$\eta_2^{\max}$	$\eta_4^{\max}$	τ
1.9	3.0	29.1	10.8	19.6	284.2	2.284

SCDM band distance (meV) and τ ($\AA$)

η_0	η_2	η_4	$\eta_0^{\max}$	$\eta_2^{\max}$	$\eta_4^{\max}$	τ
11.2	10.3	9.8	85.2	85.2	85.2	2.719

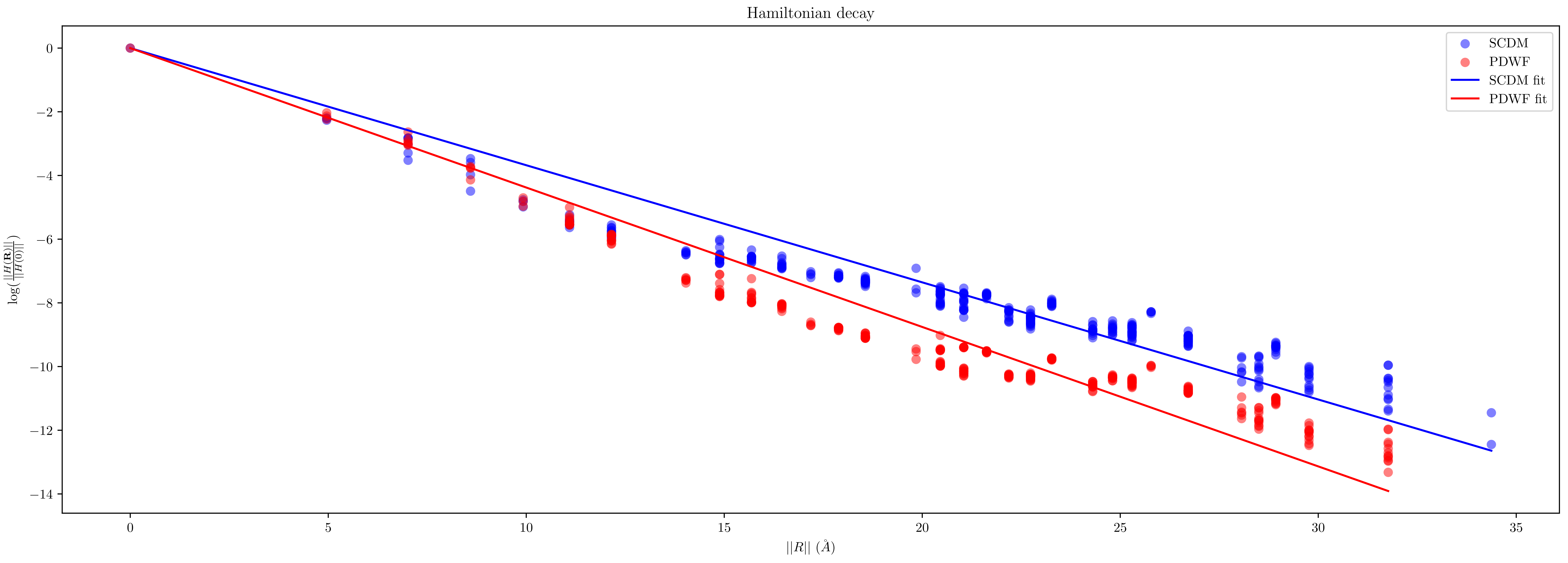

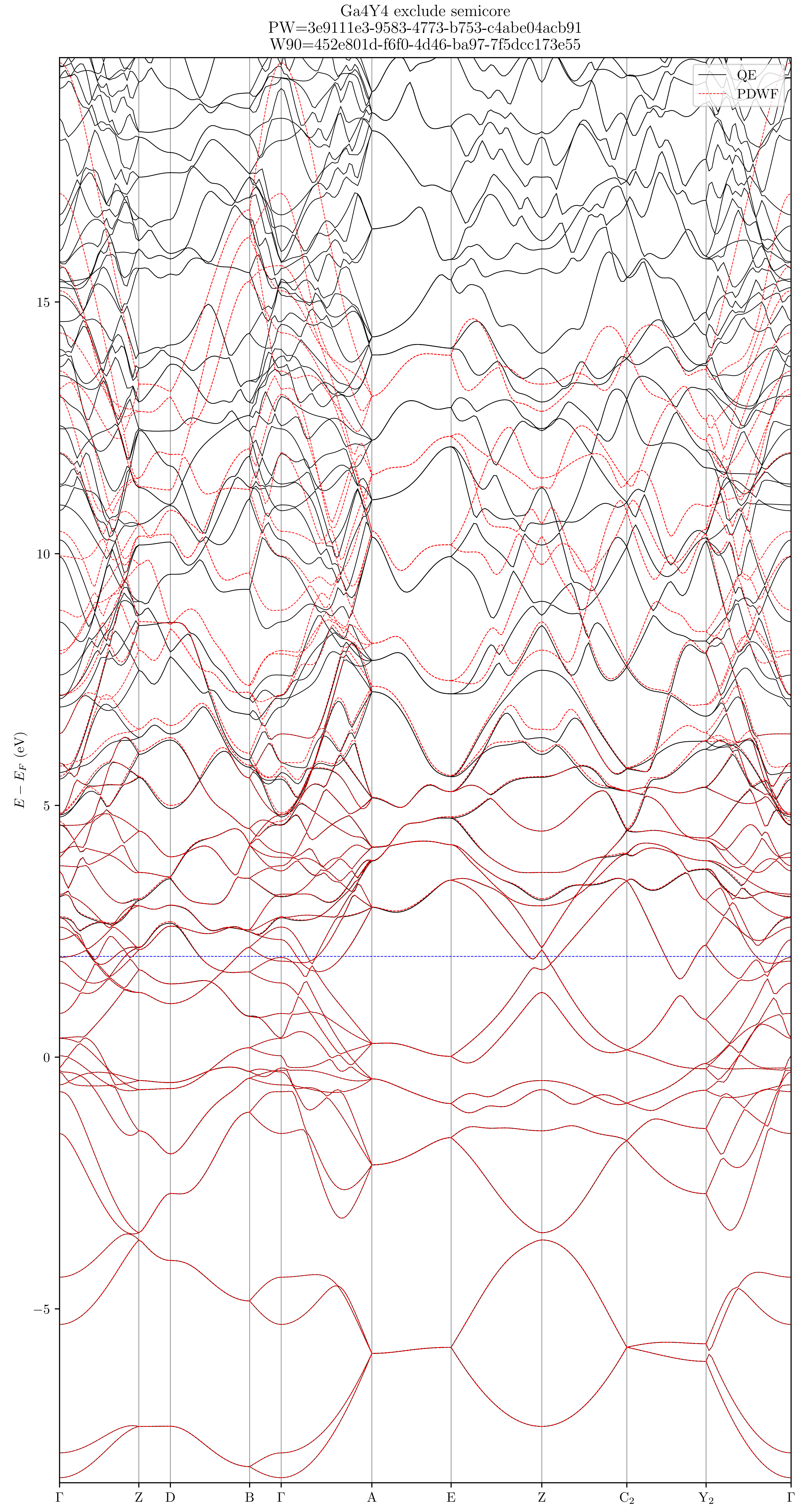

PDWF band distance (meV) and τ ($\AA$)

η_0	η_2	η_4	η_0^{max}	η_2^{max}	η_4^{max}	τ
1.0	1.3	5.5	5.5	8.7	33.0	2.480

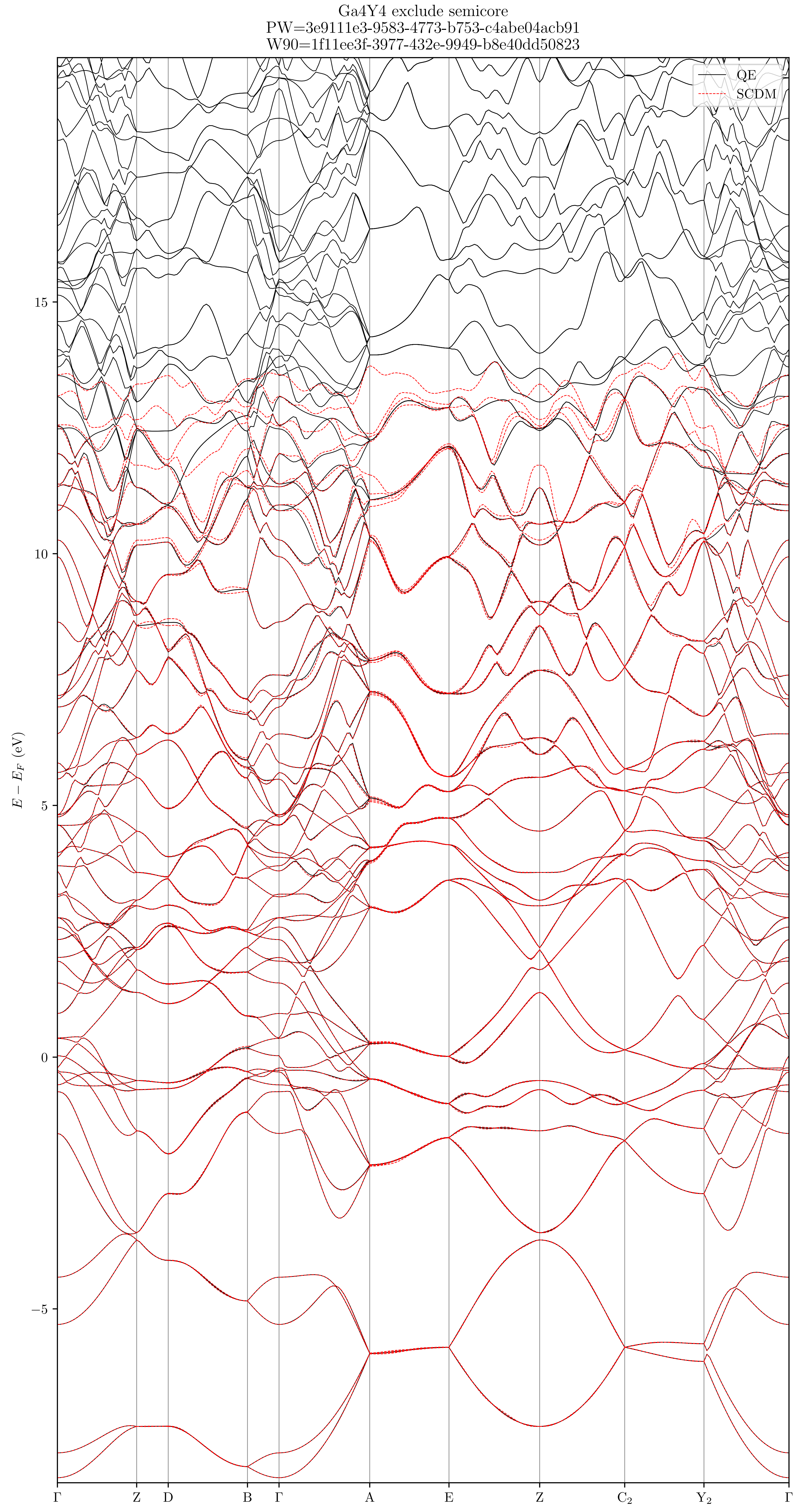

SCDM band distance (meV) and τ ($\AA$)

η_0	η_2	η_4	η_0^{max}	η_2^{max}	η_4^{max}	τ
7.2	7.3	7.2	41.7	41.7	41.7	3.225

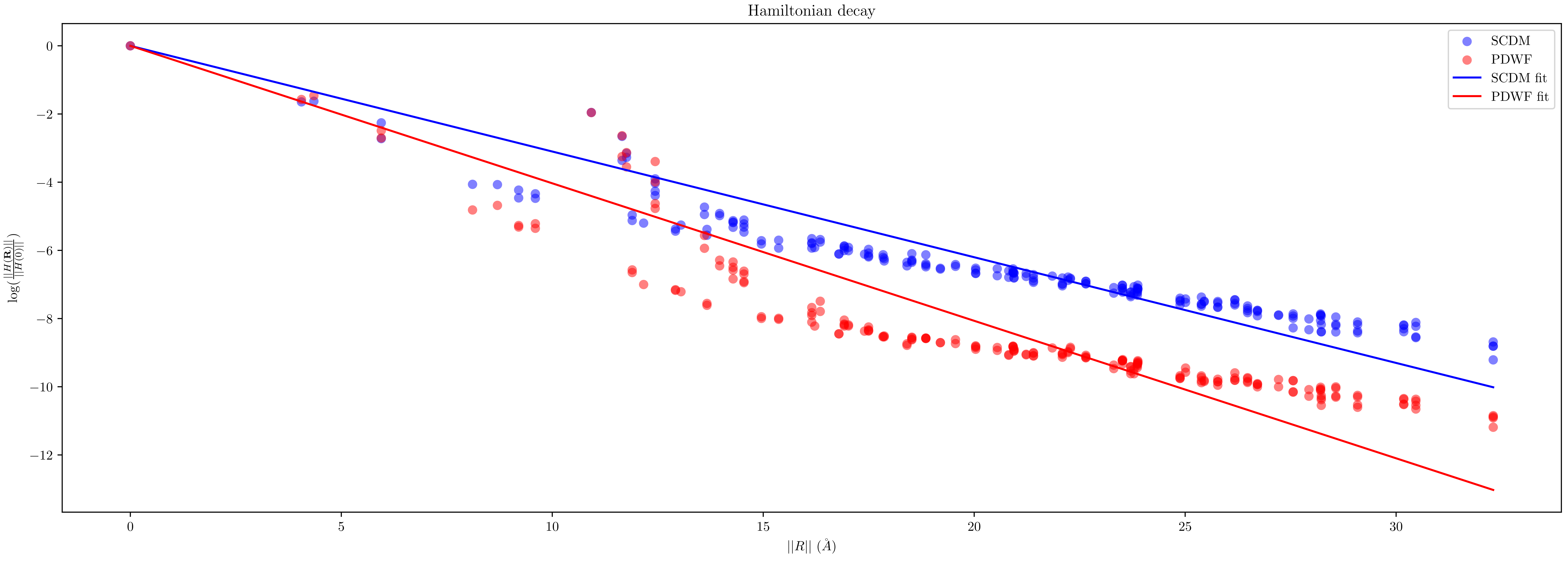

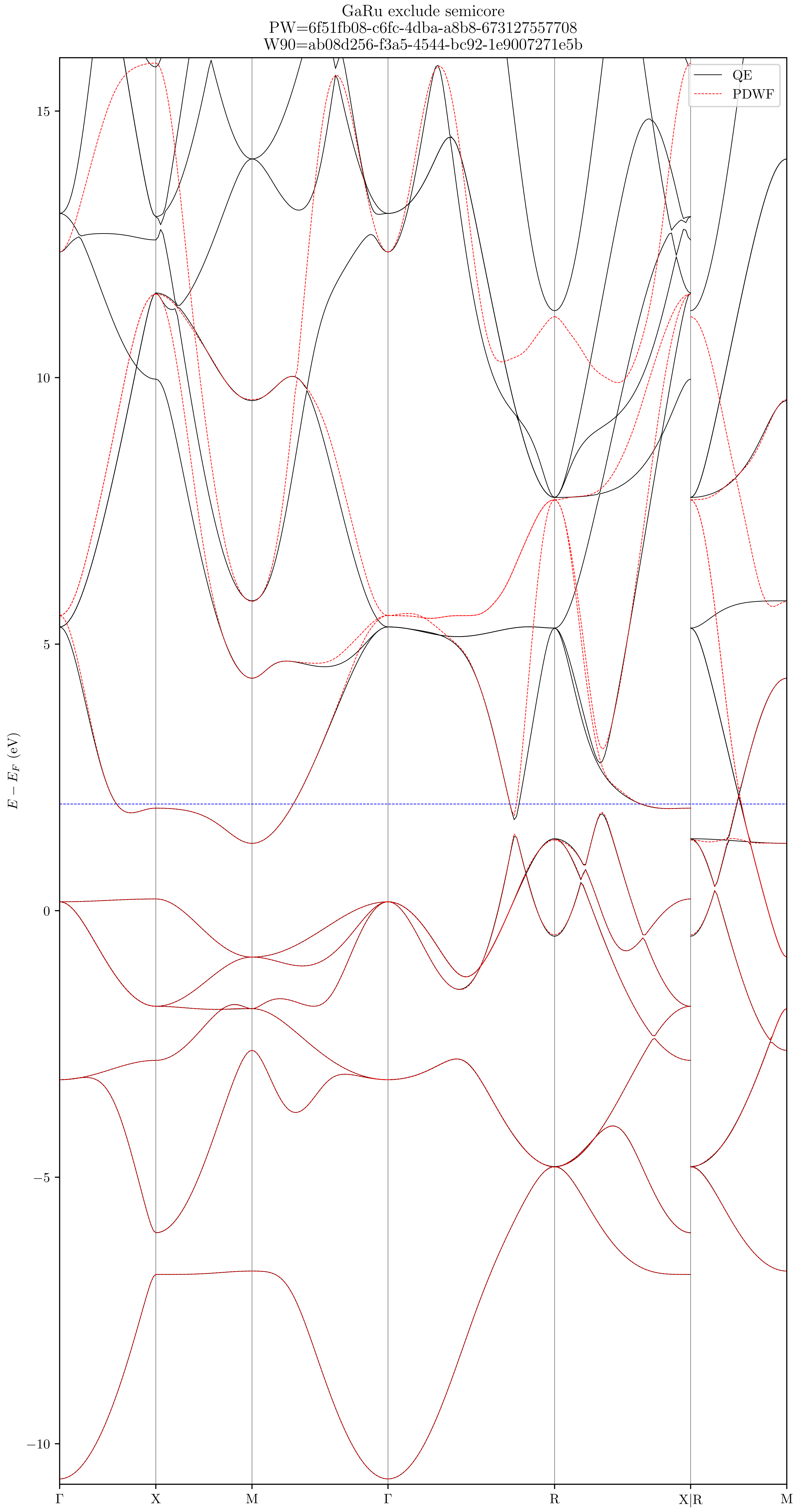

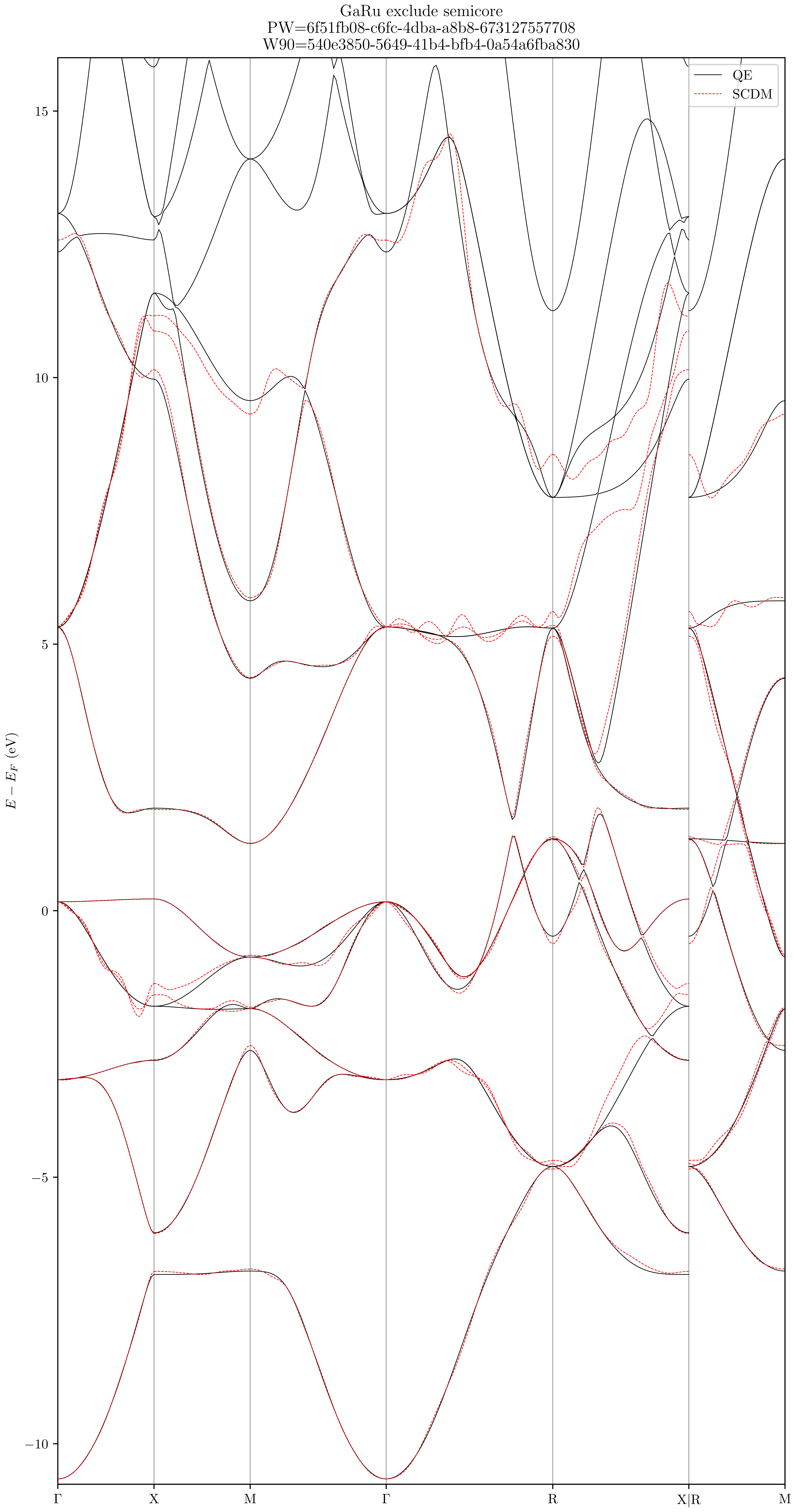

PDWF band distance (meV) and τ ($\AA$)

η_0	η_2	η_4	$\eta_0^{\max}$	$\eta_2^{\max}$	$\eta_4^{\max}$	τ
3.5	9.1	66.7	25.4	158.9	979.2	1.940

SCDM band distance (meV) and τ ($\AA$)

η_0	η_2	η_4	$\eta_0^{\max}$	$\eta_2^{\max}$	$\eta_4^{\max}$	τ
83.6	79.0	81.8	459.3	459.3	459.3	2.493

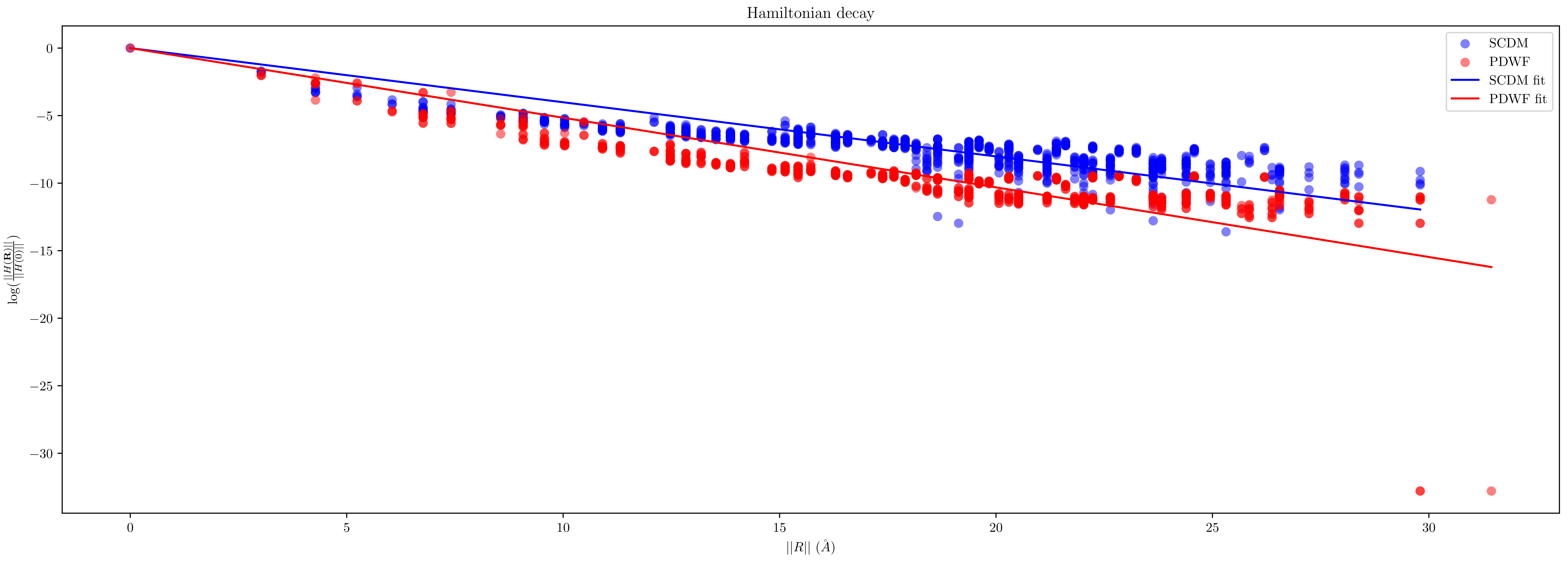

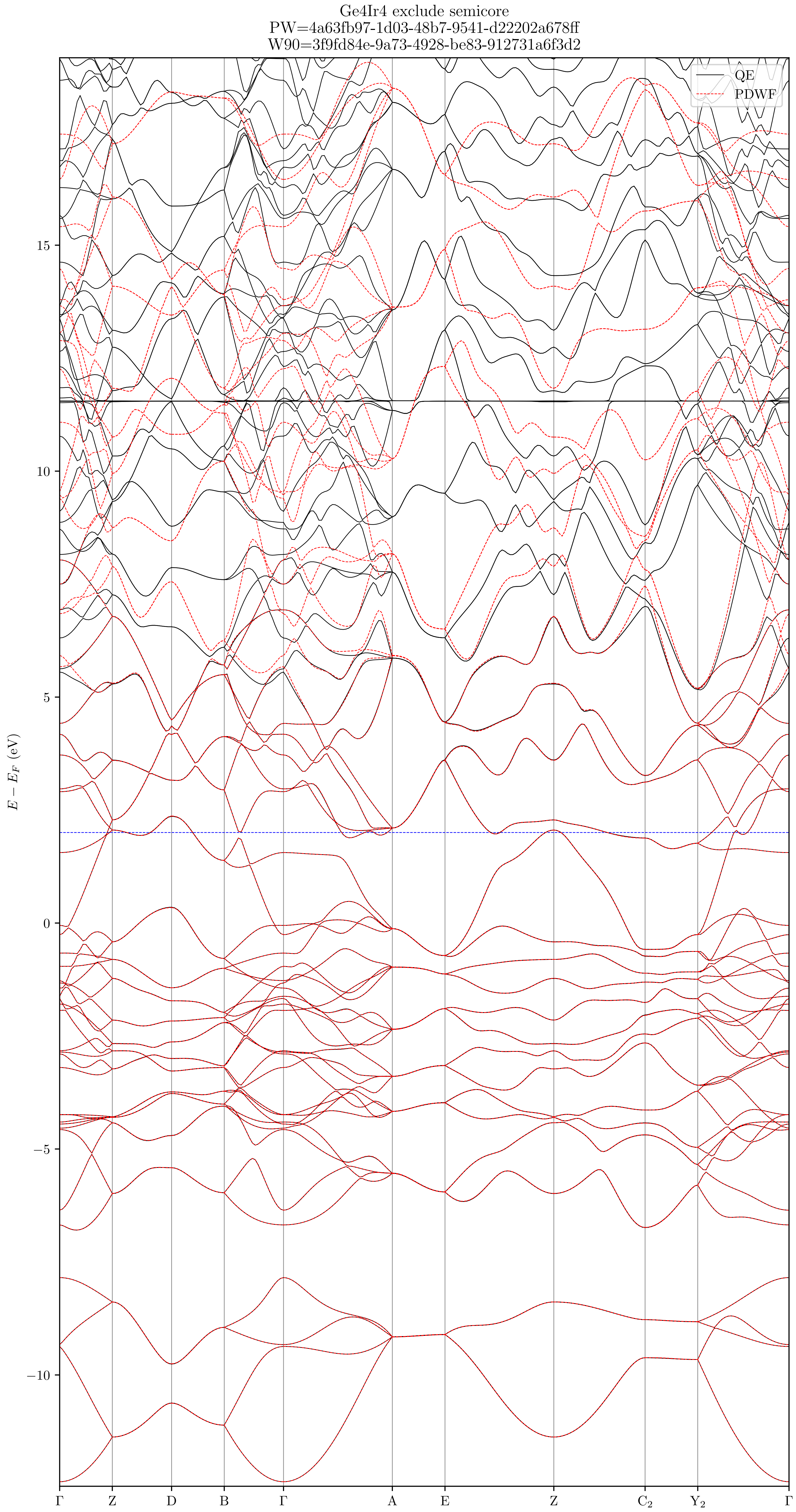

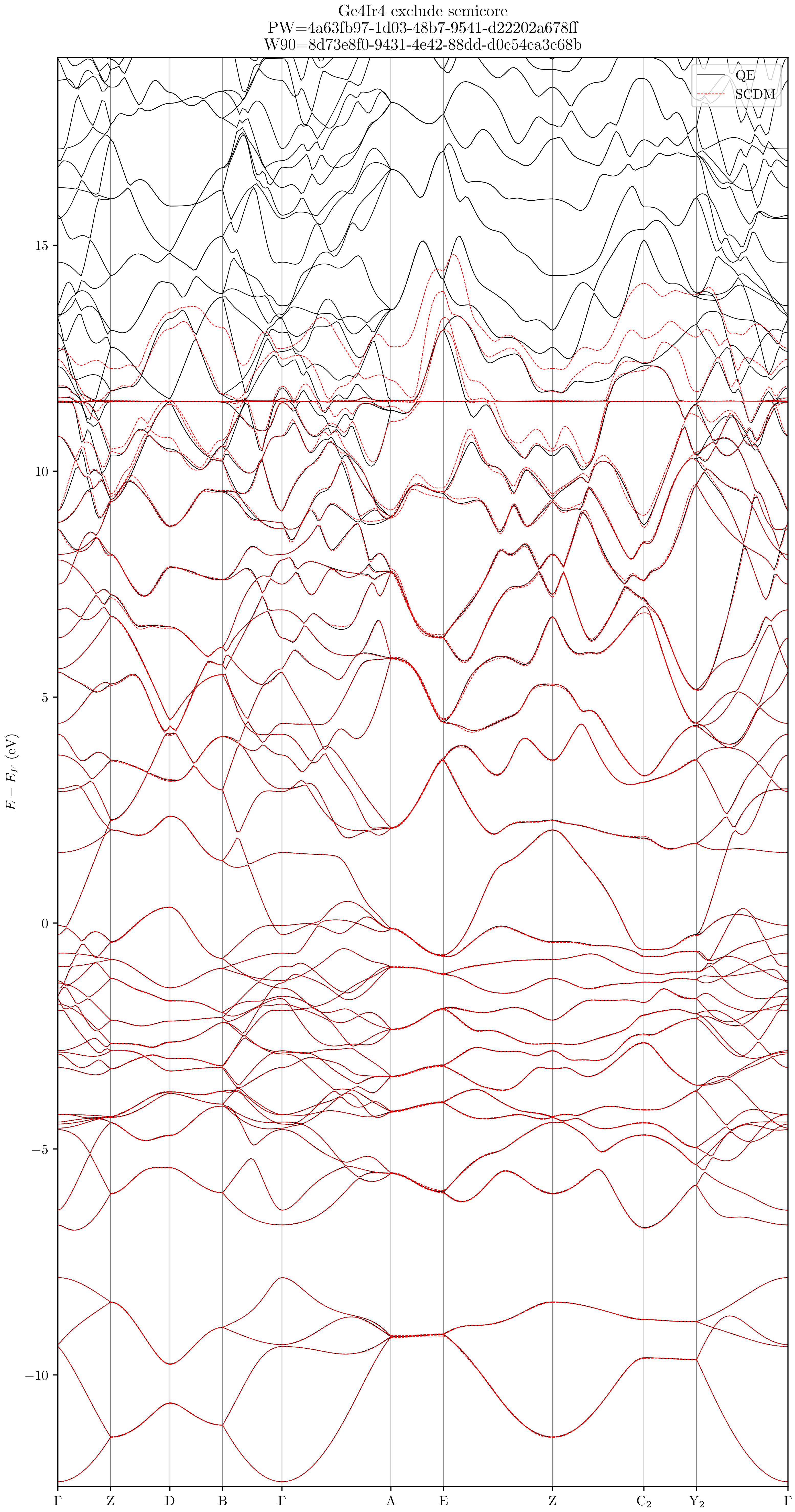

PDWF band distance (meV) and τ ($\AA$)

η_0	η_2	η_4	$\eta_0^{\max}$	$\eta_2^{\max}$	$\eta_4^{\max}$	τ
1.7	1.7	2.0	9.9	9.9	12.3	2.274

SCDM band distance (meV) and τ ($\AA$)

η_0	η_2	η_4	$\eta_0^{\max}$	$\eta_2^{\max}$	$\eta_4^{\max}$	τ
5.8	5.9	6.4	32.9	32.9	41.9	2.700

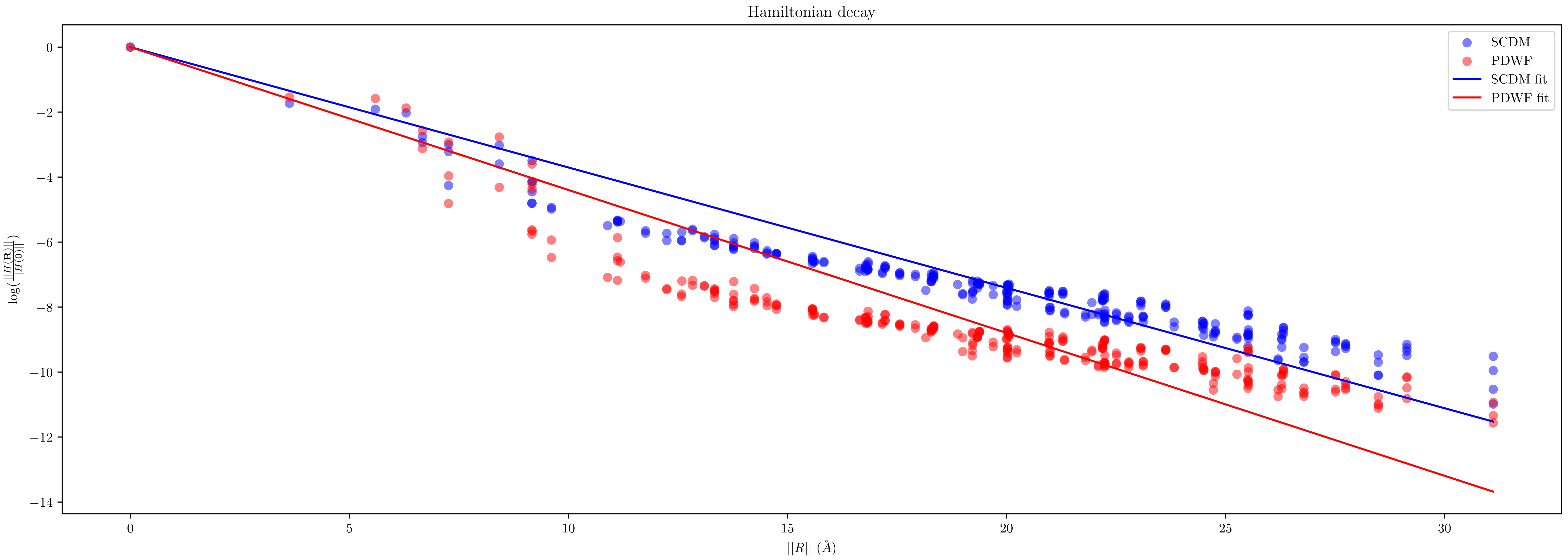

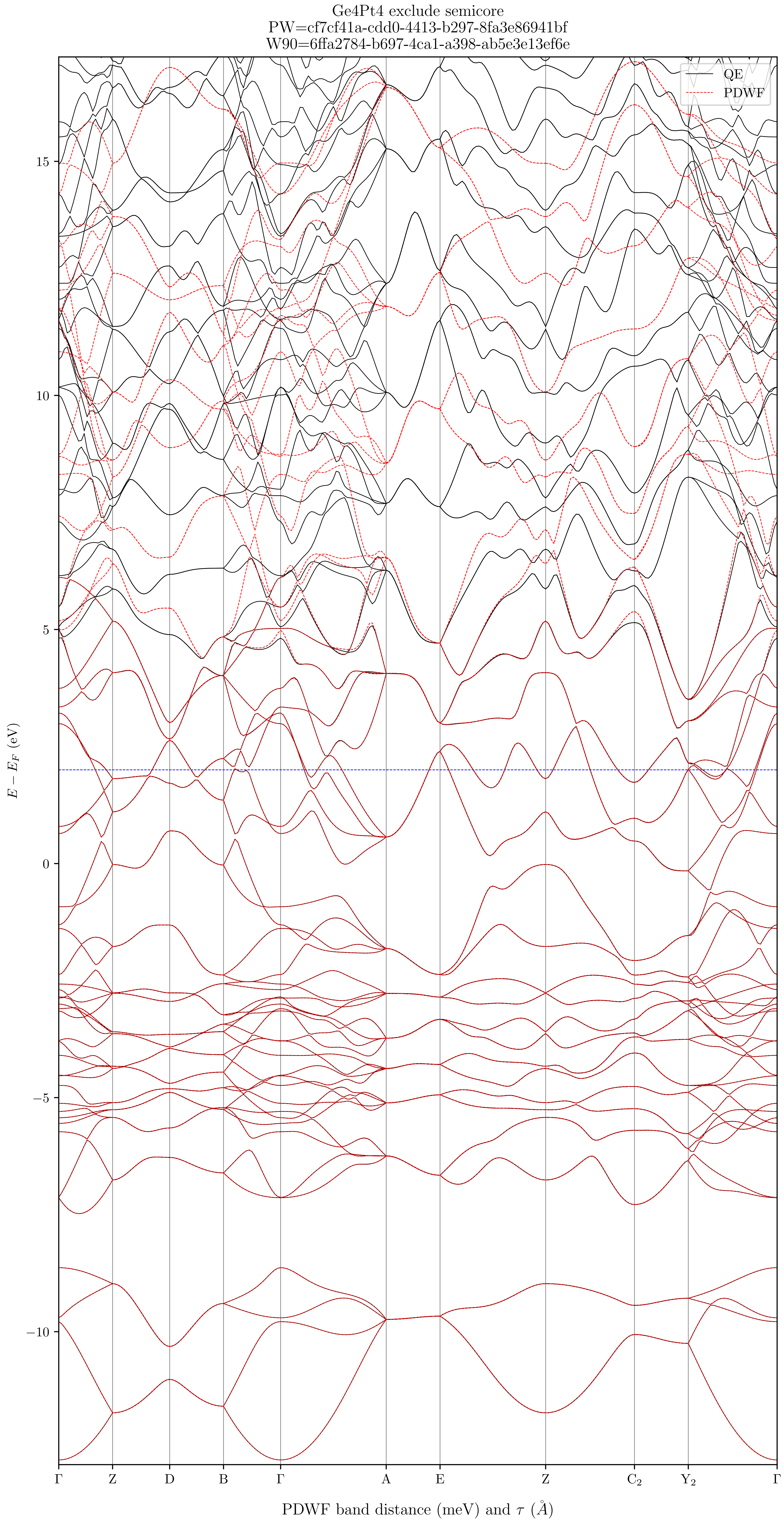

η_0	η_2	η_4	$\eta_0^{\max}$	$\eta_2^{\max}$	$\eta_4^{\max}$	τ
1.1	1.2	1.6	6.5	7.1	13.6	2.372

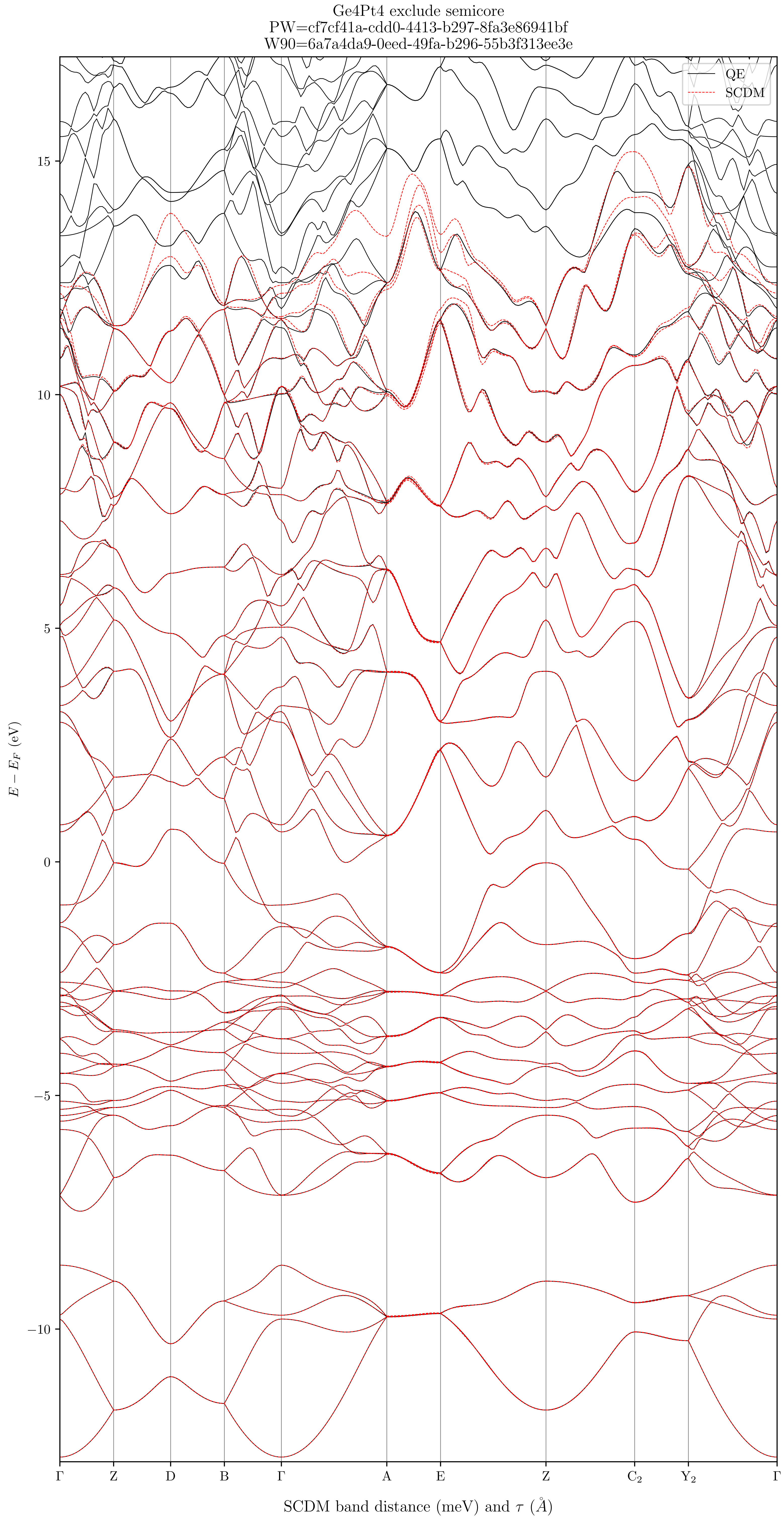

η_0	η_2	η_4	$\eta_0^{\max}$	$\eta_2^{\max}$	$\eta_4^{\max}$	τ
3.3	3.4	3.8	22.5	22.5	33.7	2.731

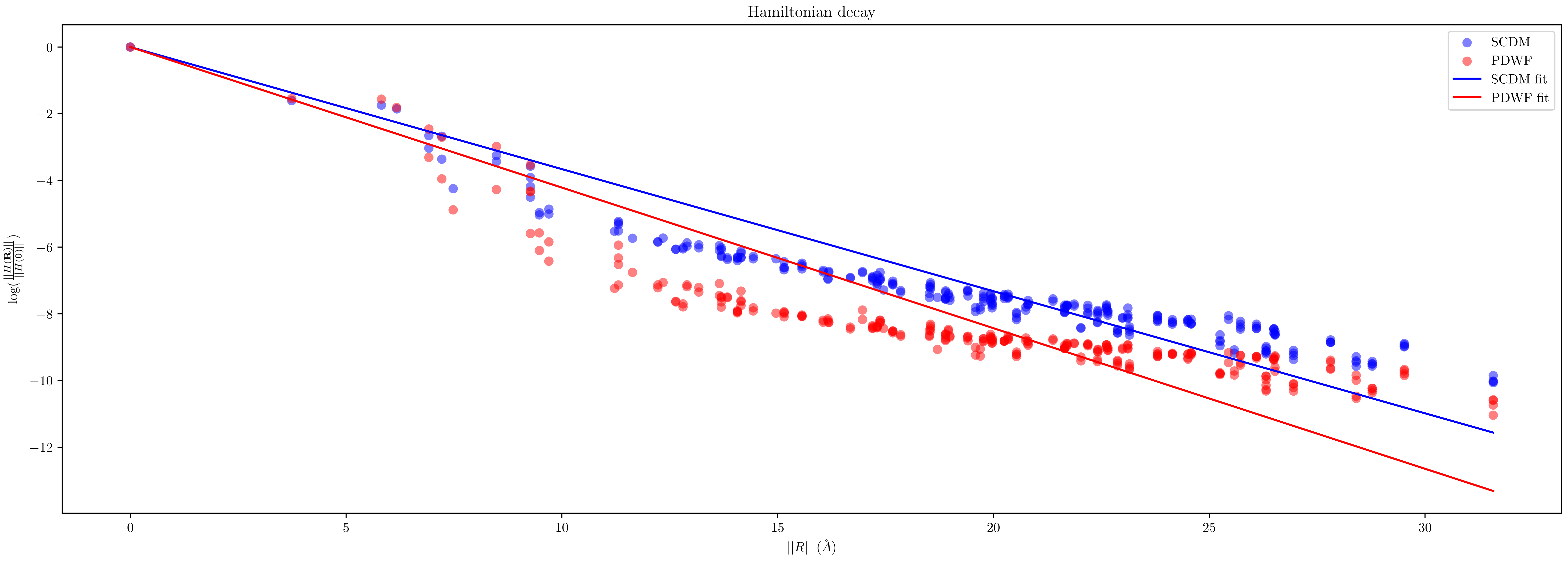

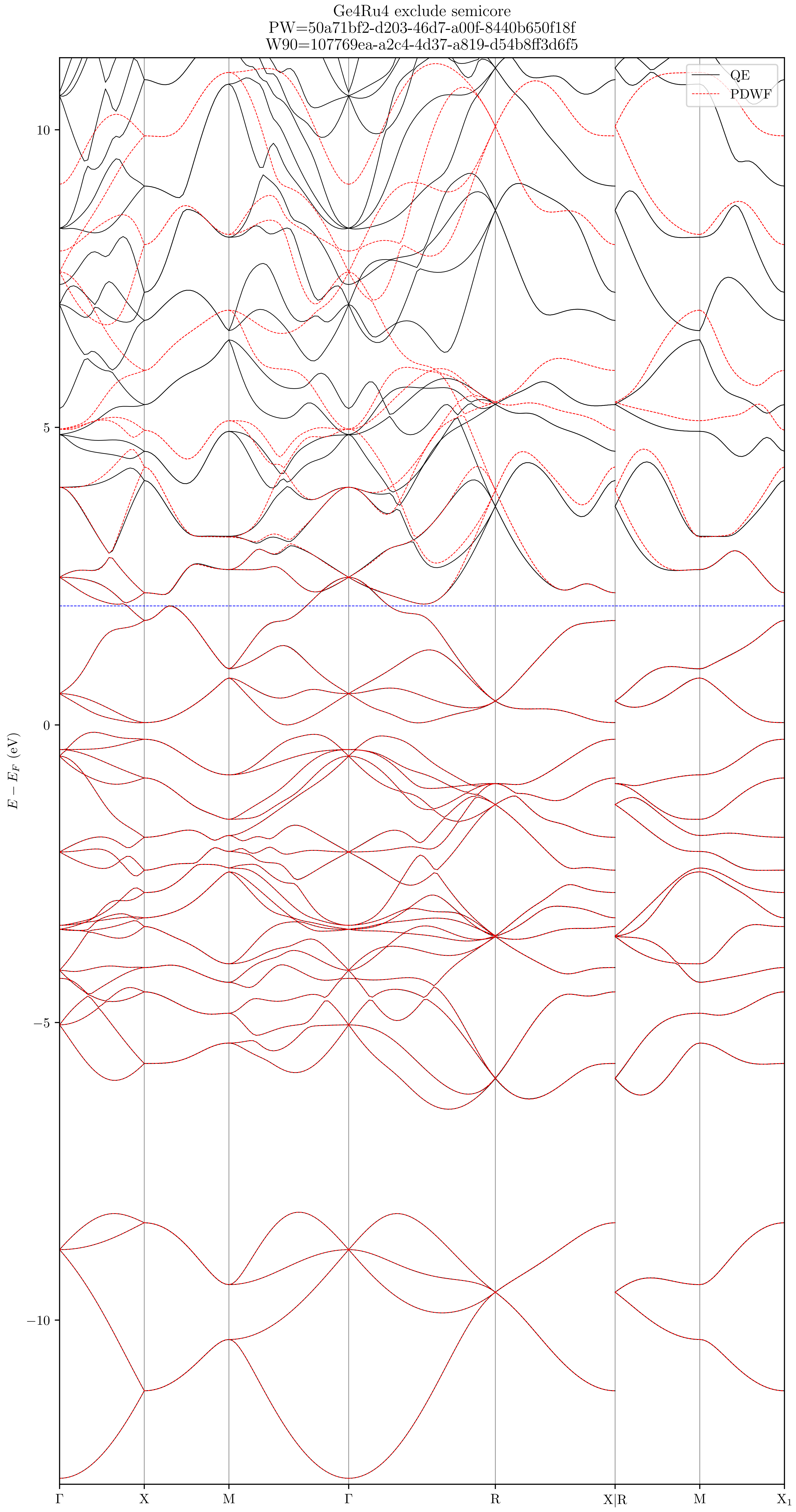

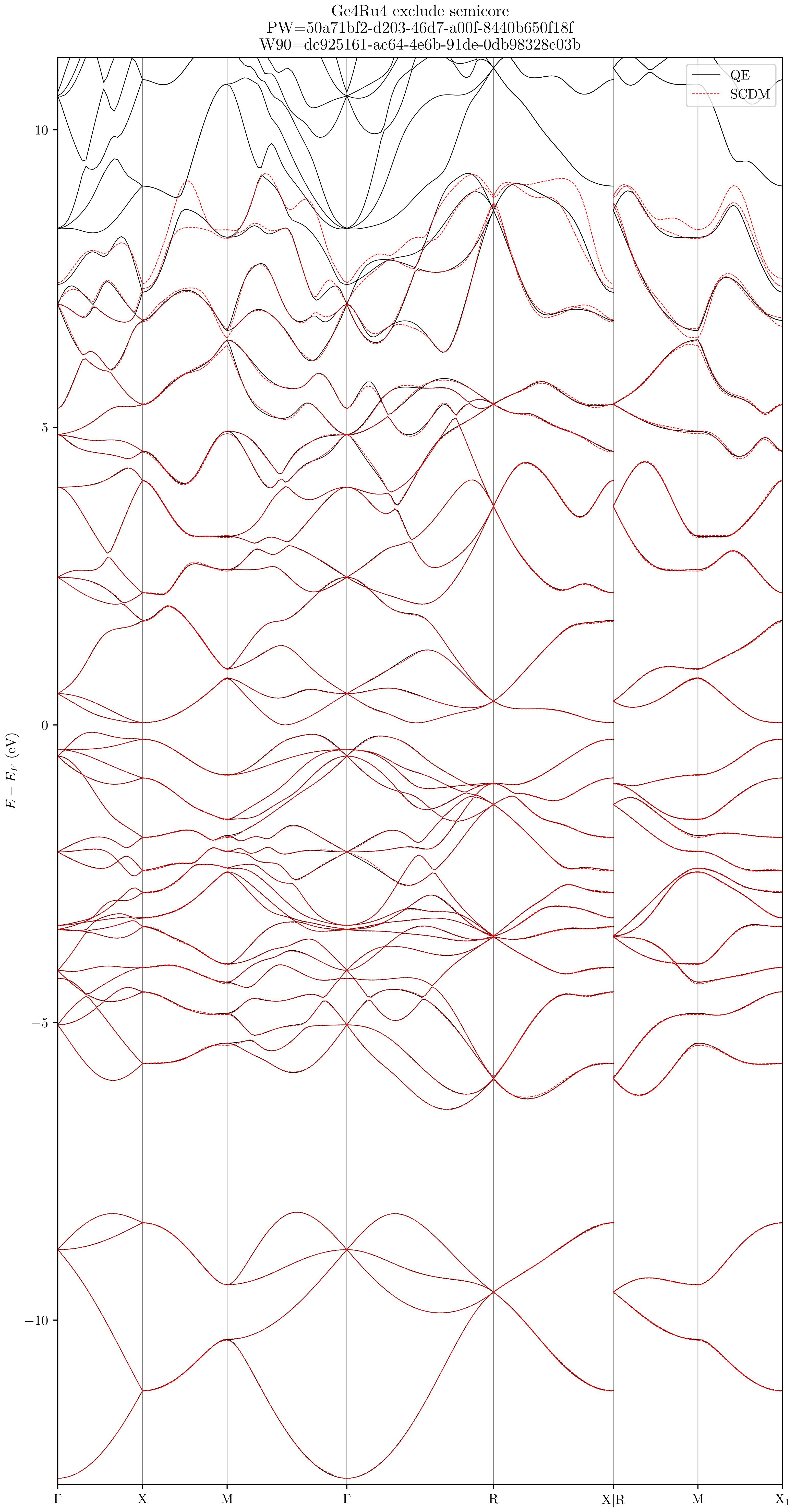

PDWF band distance (meV) and τ ($\AA$)

η_0	η_2	η_4	$\eta_0^{\max}$	$\eta_2^{\max}$	$\eta_4^{\max}$	τ
1.4	1.5	27.9	8.4	8.4	235.8	2.278

SCDM band distance (meV) and τ ($\AA$)

η_0	η_2	η_4	$\eta_0^{\max}$	$\eta_2^{\max}$	$\eta_4^{\max}$	τ
5.9	5.9	6.2	44.5	44.5	44.5	2.691

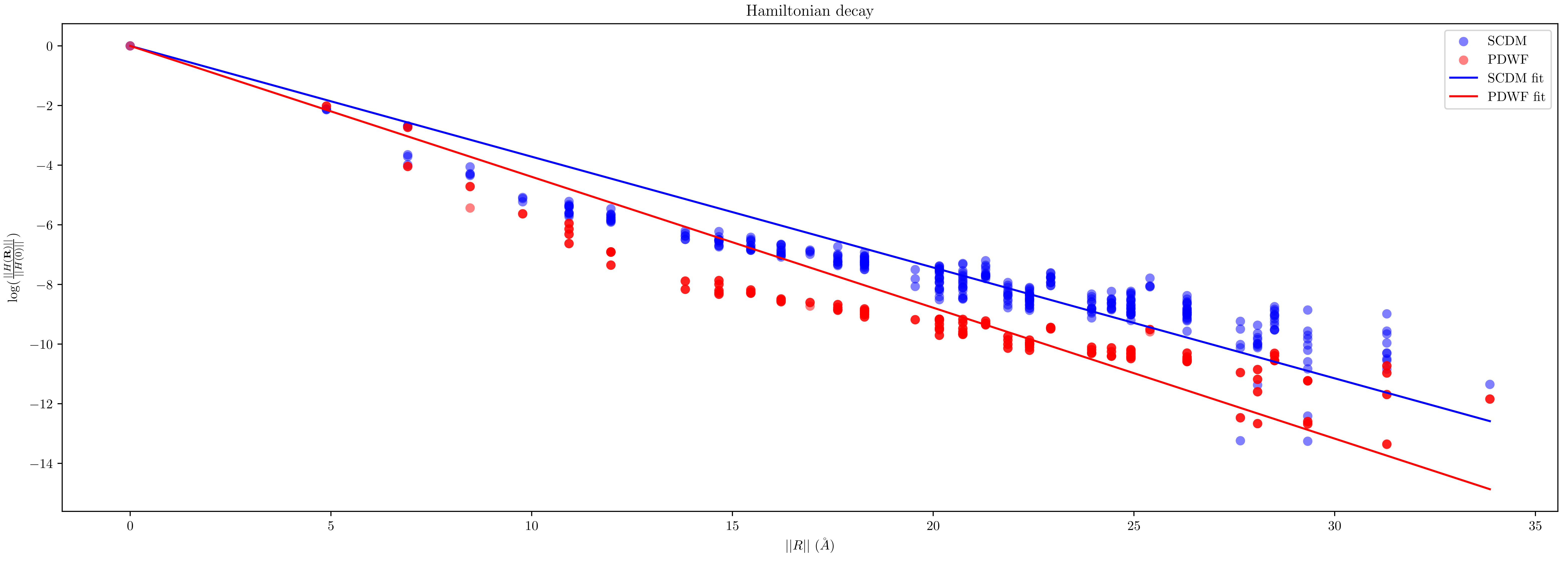

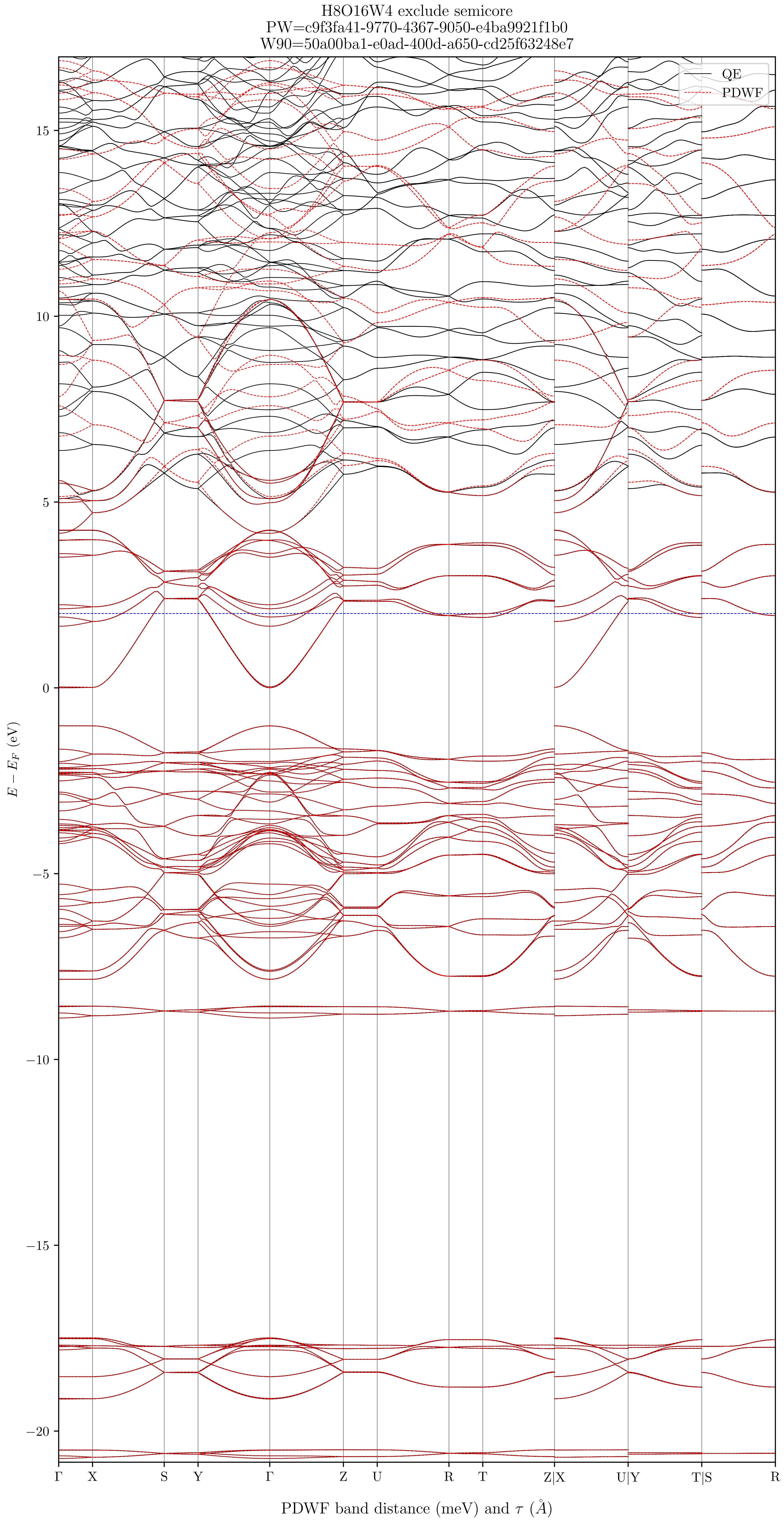

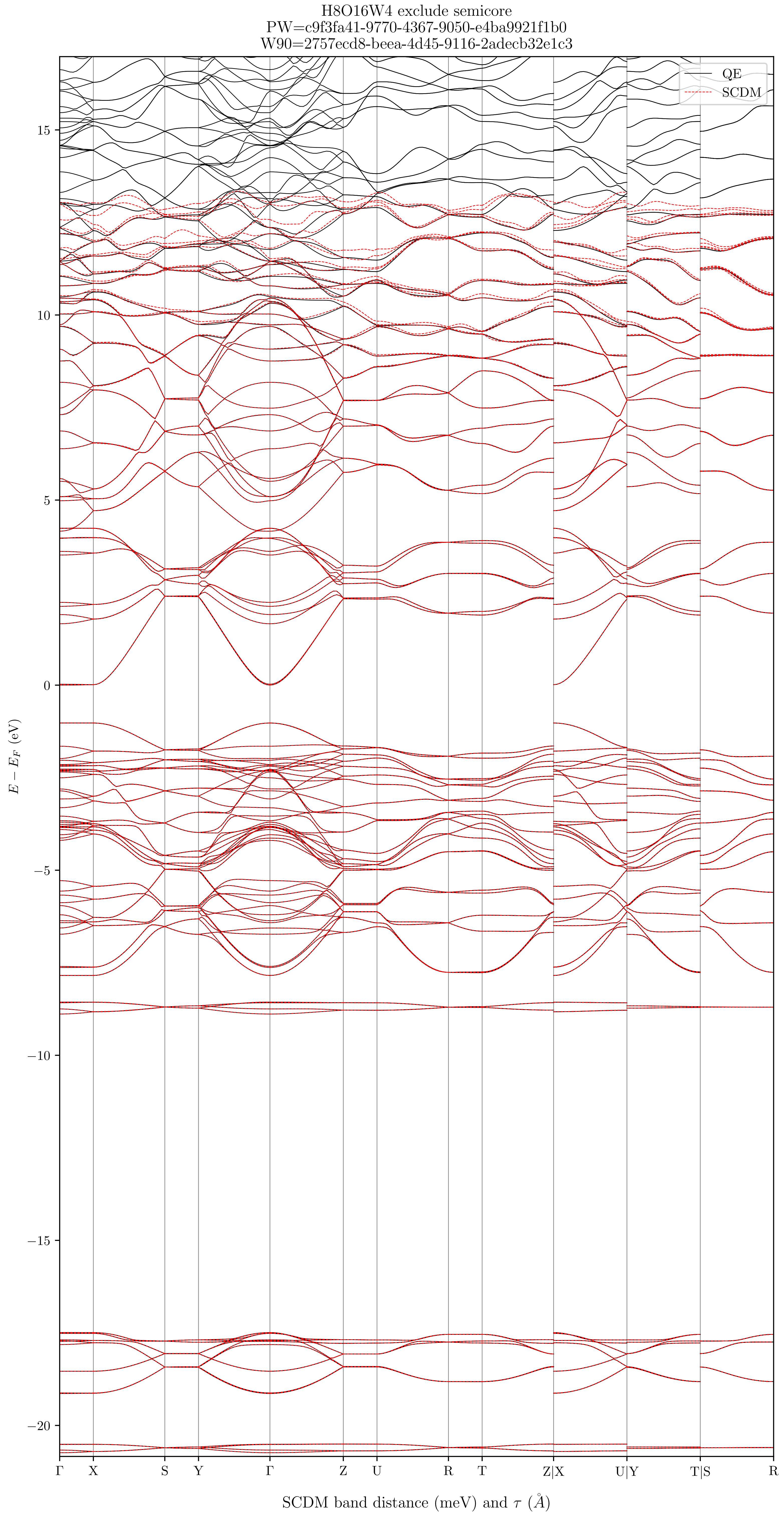

PDWF band distance (meV) and τ ($\AA$)

η_0	η_2	η_4	$\eta_0^{\max}$	$\eta_2^{\max}$	$\eta_4^{\max}$	τ
0.2	0.2	0.3	2.8	2.8	2.9	2.556

SCDM band distance (meV) and τ ($\AA$)

η_0	η_2	η_4	$\eta_0^{\max}$	$\eta_2^{\max}$	$\eta_4^{\max}$	τ
1.2	1.2	1.2	10.5	10.5	10.5	3.230

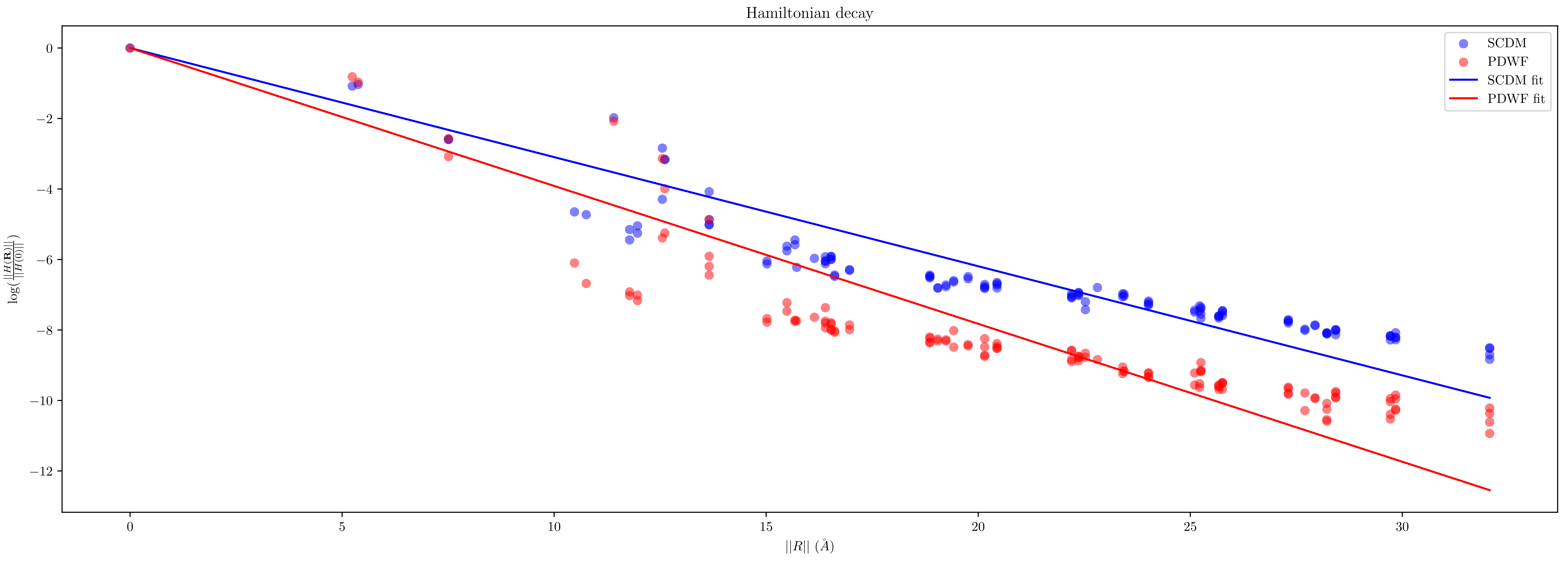

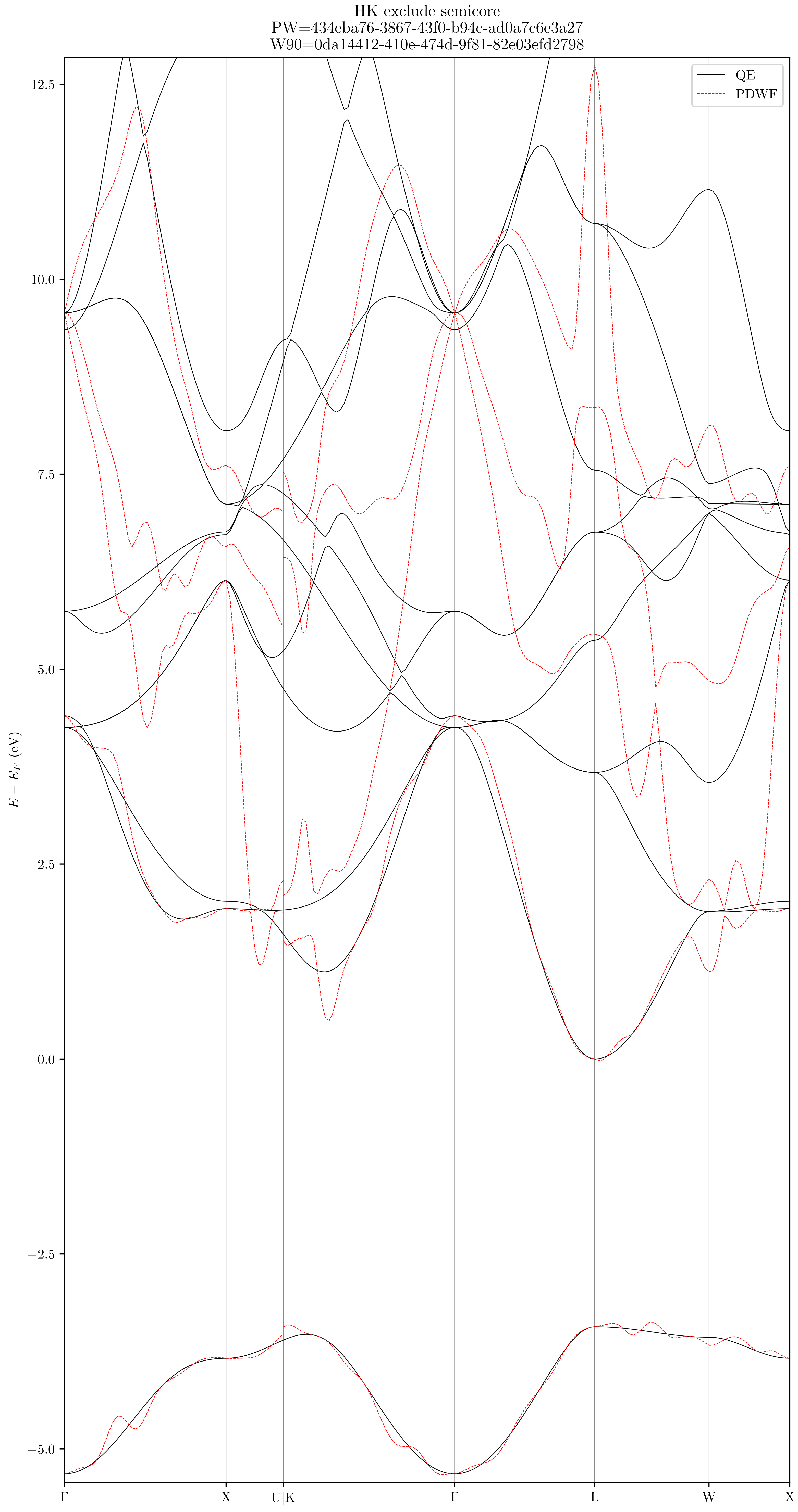

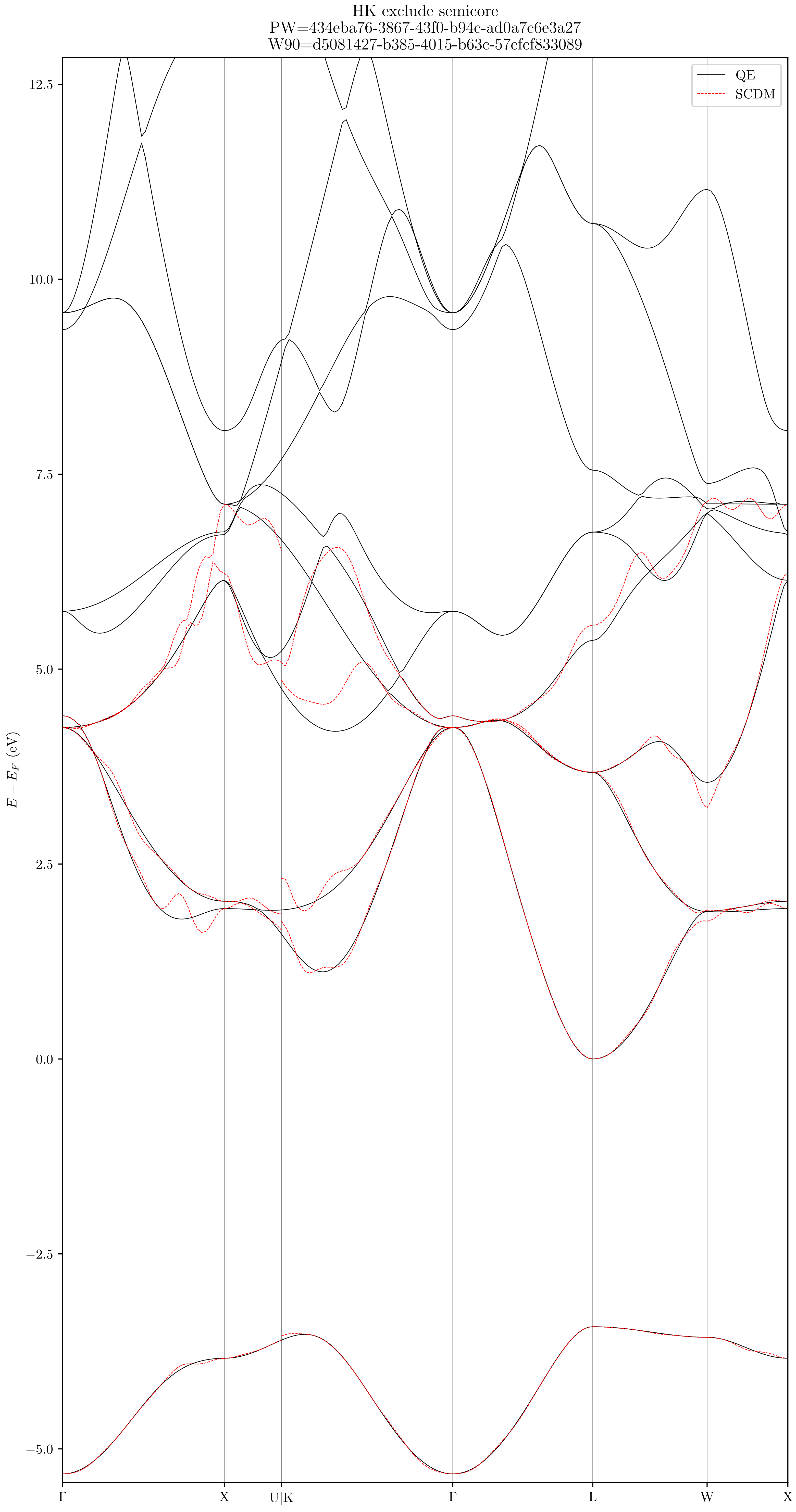

PDWF band distance (meV) and τ ($\AA$)

η_0	η_2	η_4	$\eta_0^{\max}$	$\eta_2^{\max}$	$\eta_4^{\max}$	τ
72.2	72.2	130.0	292.3	292.3	640.5	3.482

SCDM band distance (meV) and τ ($\AA$)

η_0	η_2	η_4	$\eta_0^{\max}$	$\eta_2^{\max}$	$\eta_4^{\max}$	τ
13.9	13.9	31.5	51.9	51.9	102.9	2.927

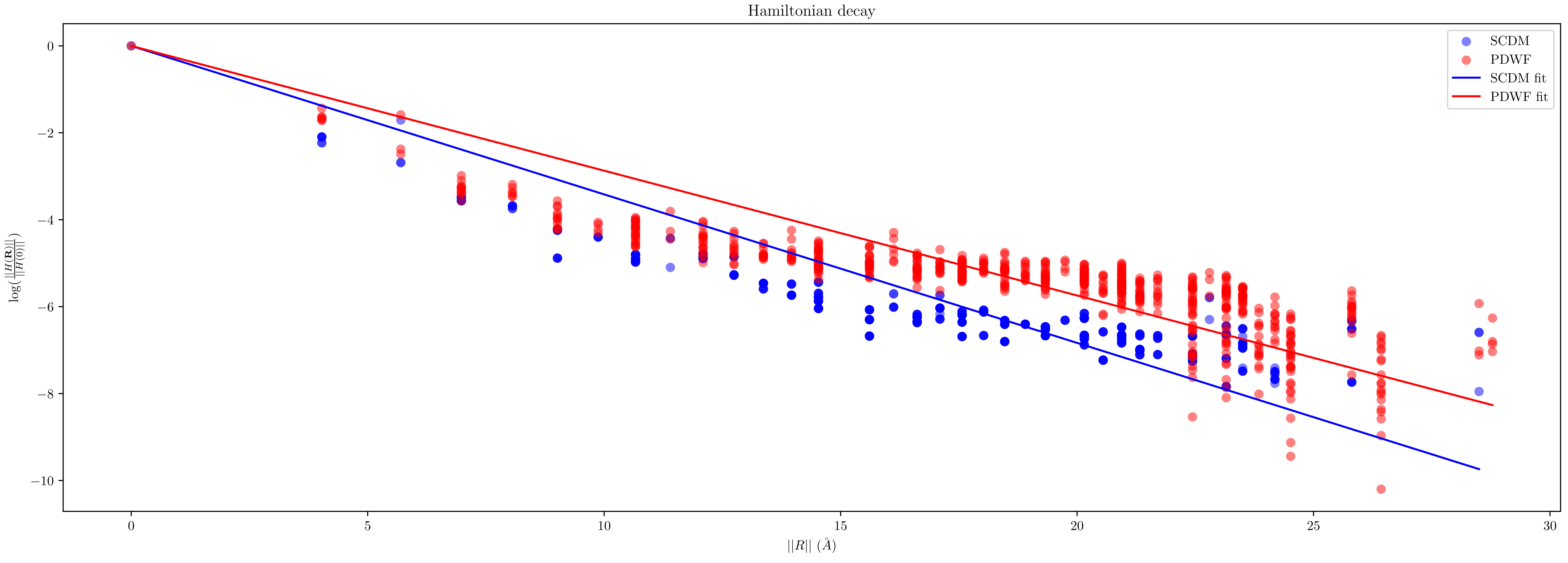

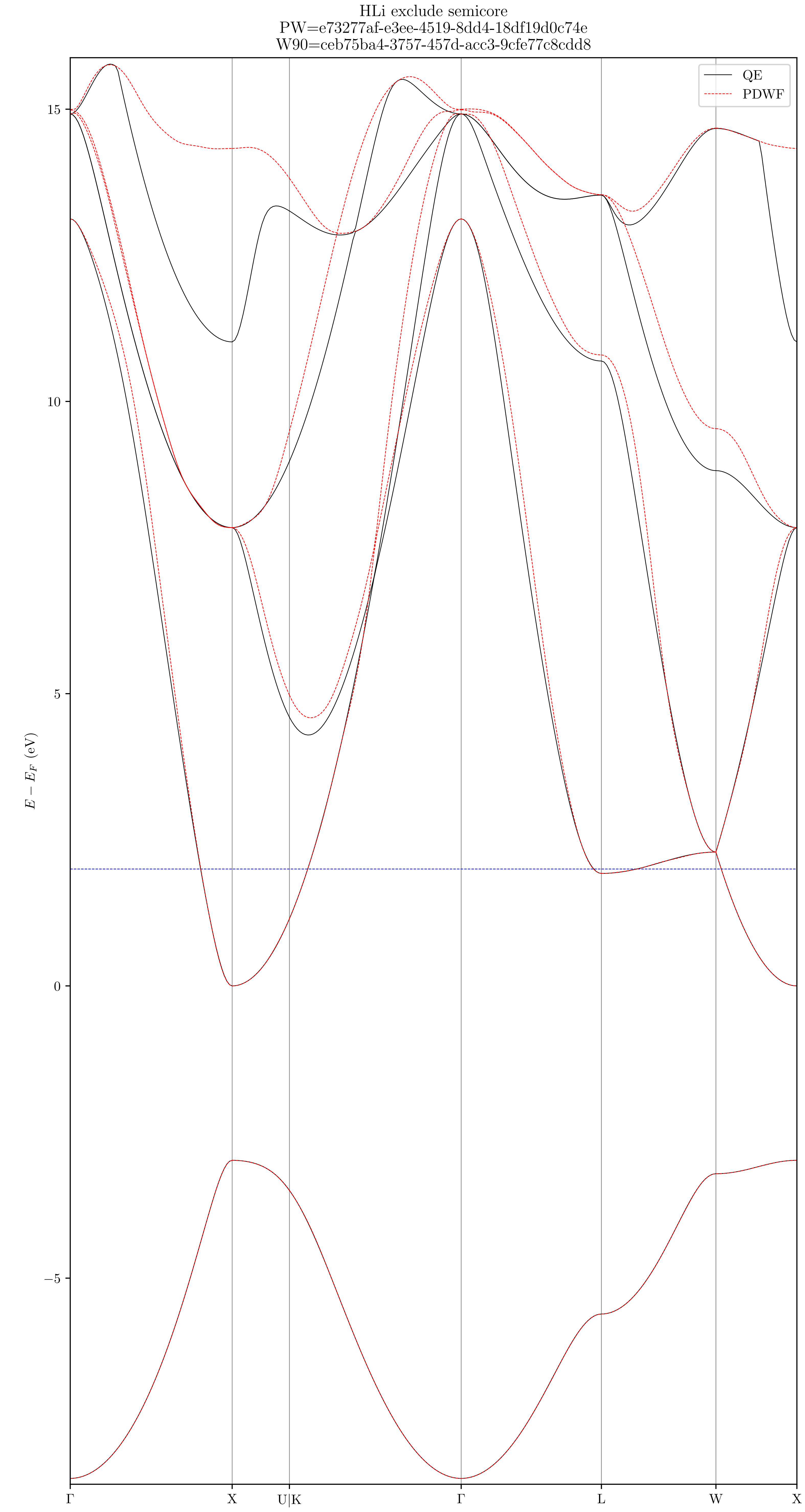

PDWF band distance (meV) and τ ($\AA$)

η_0	η_2	η_4	$\eta_0^{\max}$	$\eta_2^{\max}$	$\eta_4^{\max}$	τ
1.9	2.2	4.0	9.3	10.7	31.2	1.936

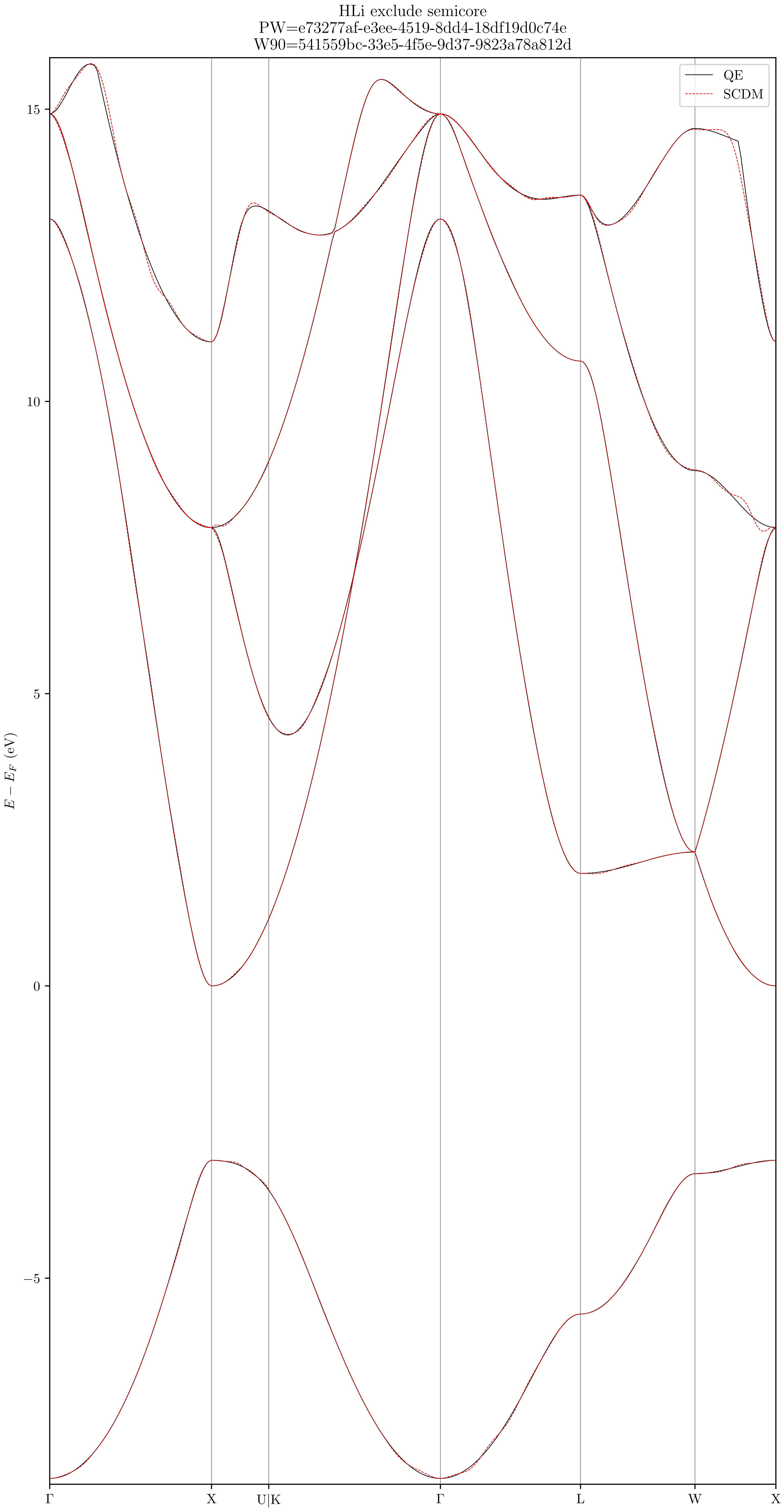

SCDM band distance (meV) and τ ($\AA$)

η_0	η_2	η_4	$\eta_0^{\max}$	$\eta_2^{\max}$	$\eta_4^{\max}$	τ
12.8	12.2	11.8	41.5	41.5	41.5	2.367

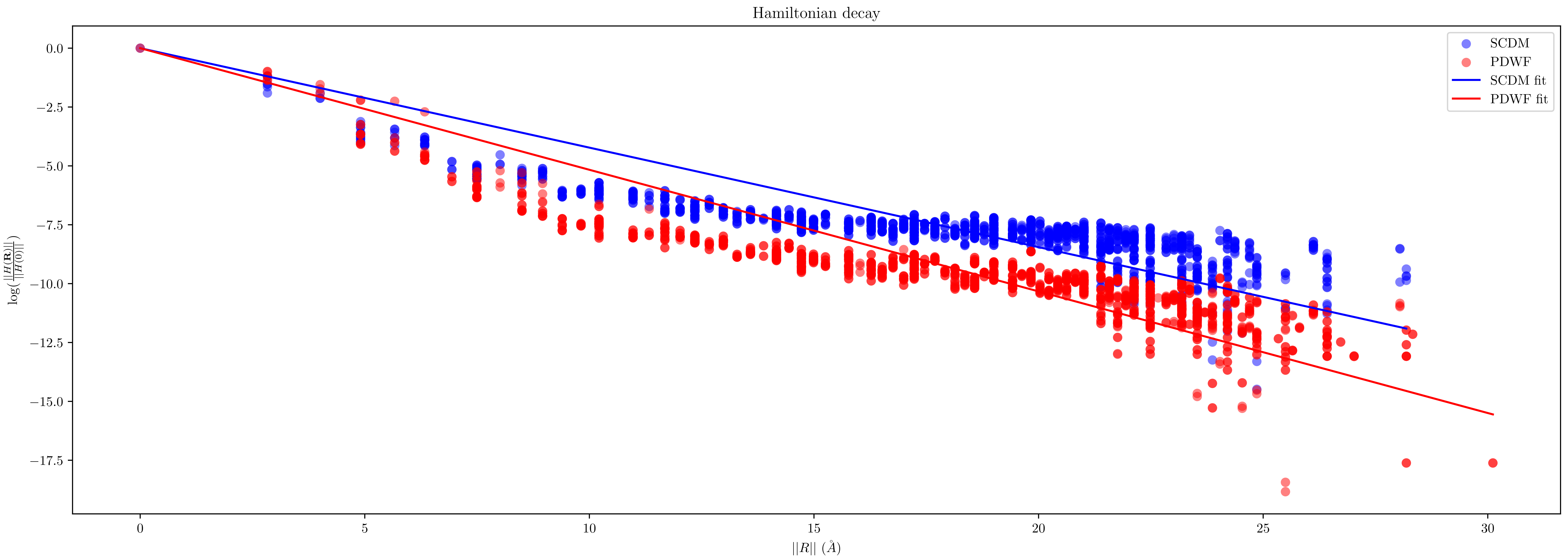

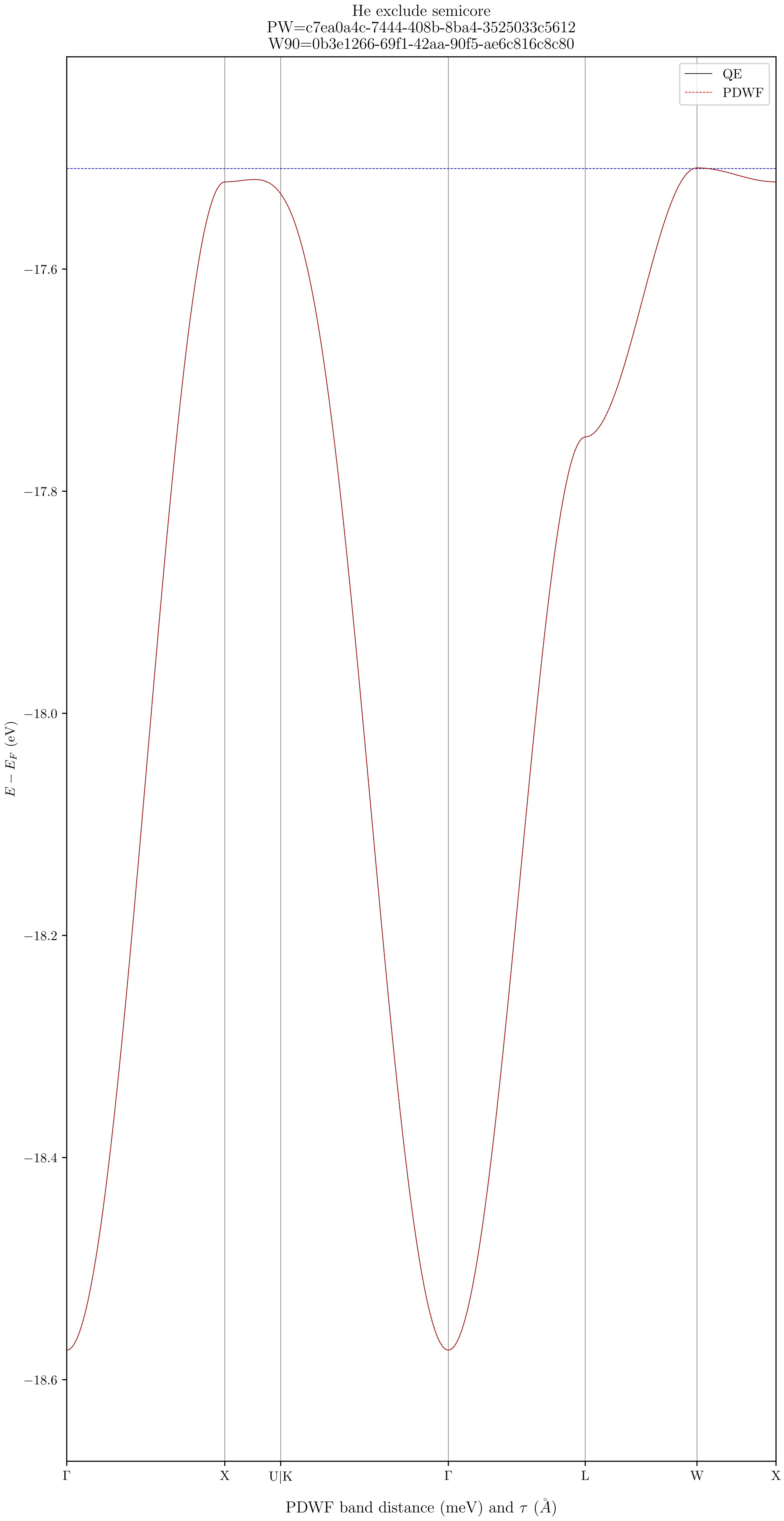

η_0	η_2	η_4	$\eta_0^{\max}$	$\eta_2^{\max}$	$\eta_4^{\max}$	τ
0.0	0.0	0.0	0.0	0.0	0.0	0.880

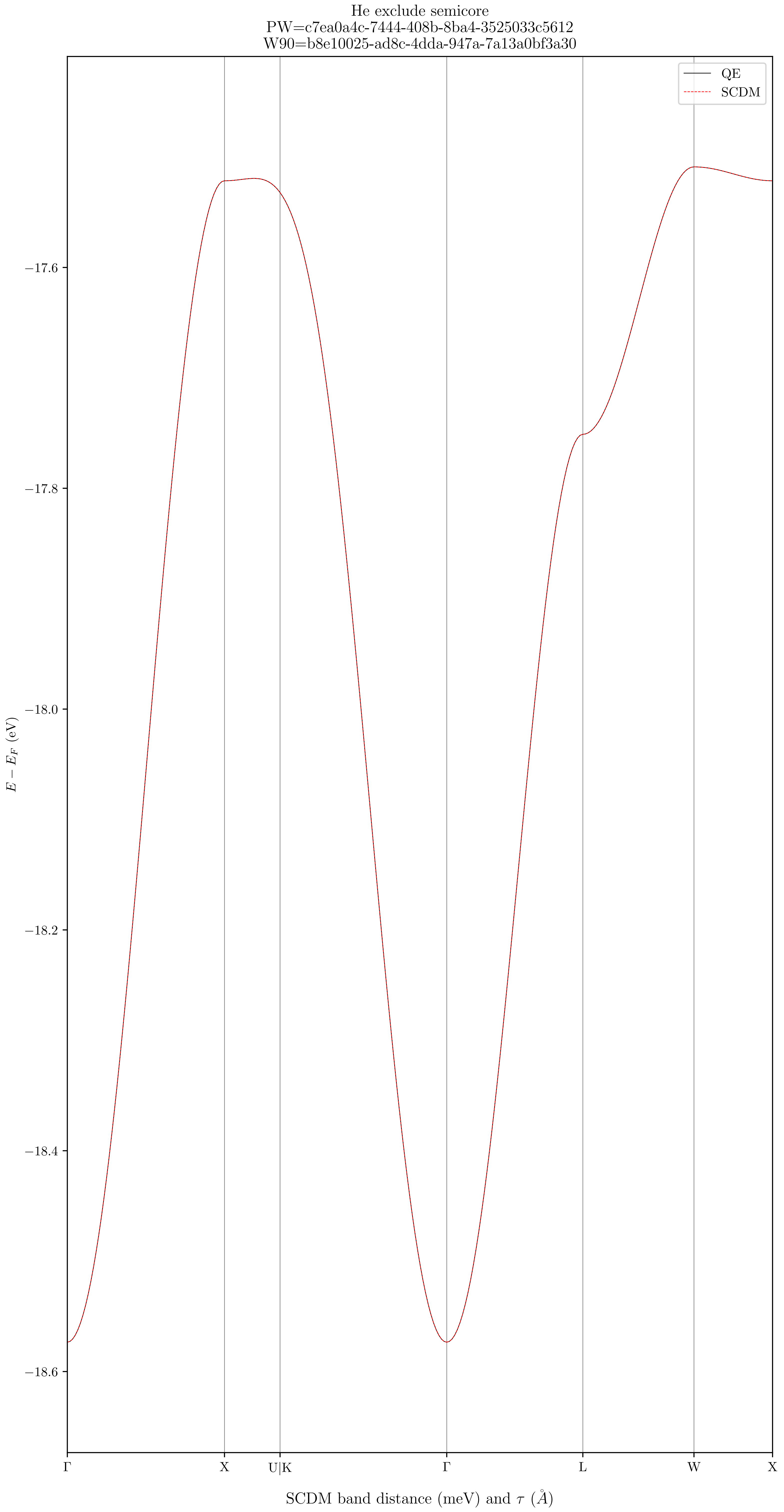

η_0	η_2	η_4	$\eta_0^{\max}$	$\eta_2^{\max}$	$\eta_4^{\max}$	τ
0.0	0.0	0.0	0.0	0.0	0.0	0.880

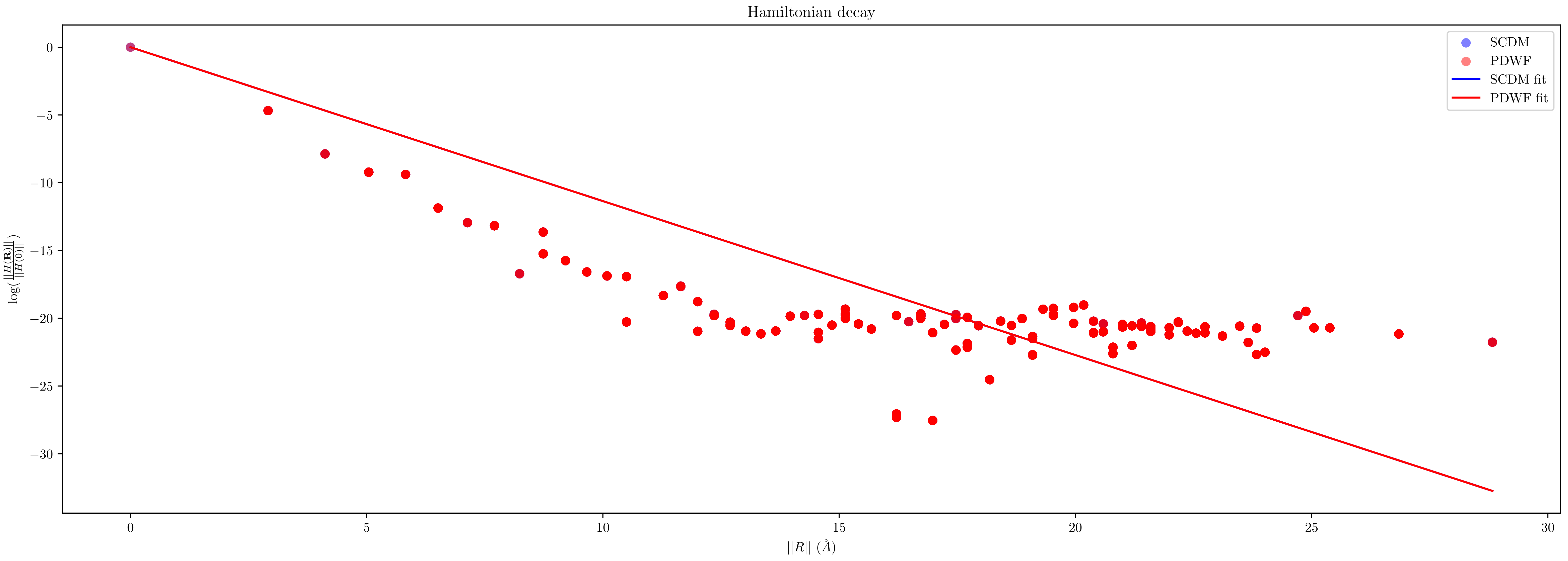

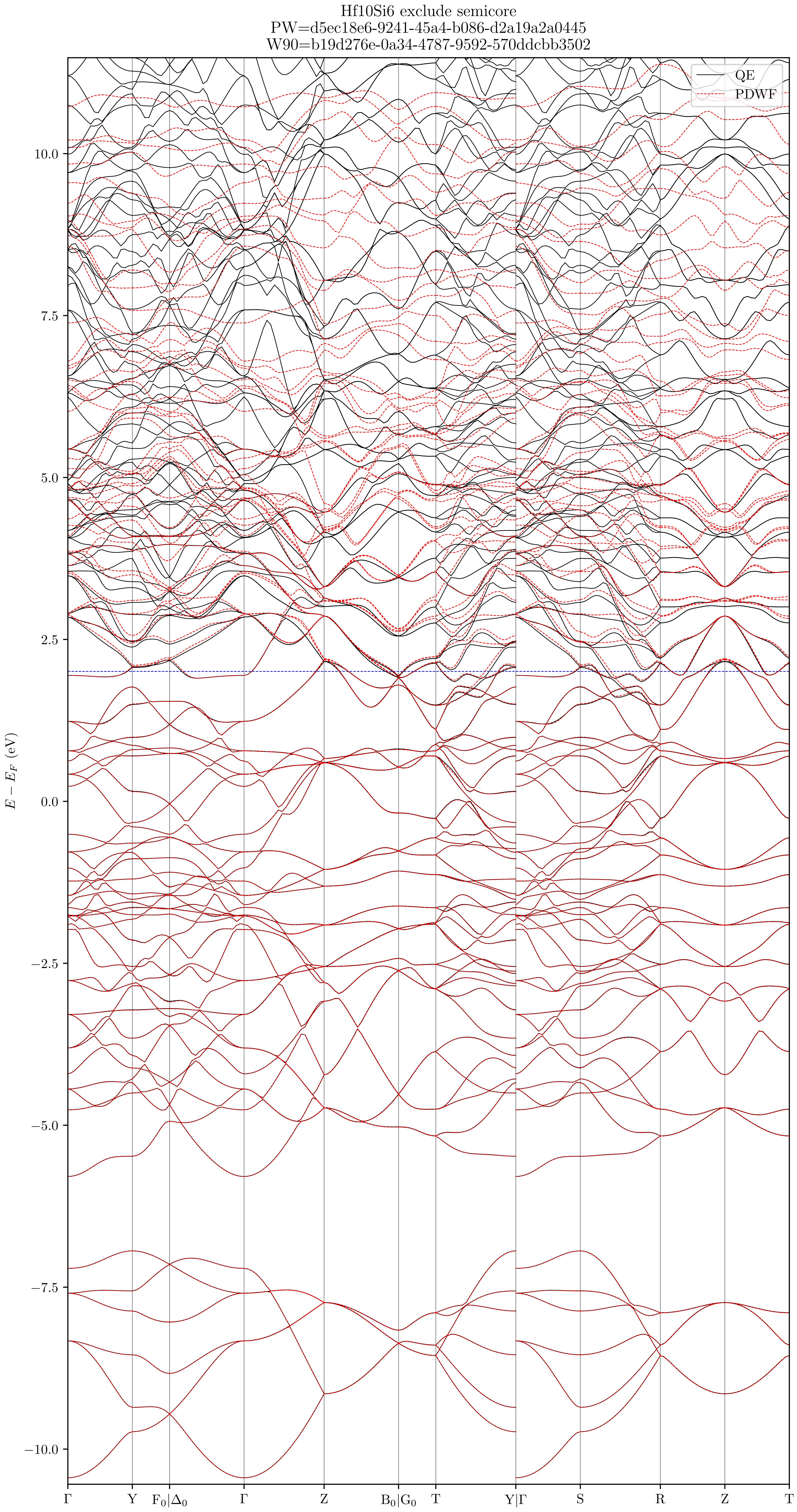

PDWF band distance (meV) and τ ($\AA$)

η_0	η_2	η_4	$\eta_0^{\max}$	$\eta_2^{\max}$	$\eta_4^{\max}$	τ
1.8	3.9	51.9	13.5	52.2	415.7	2.791

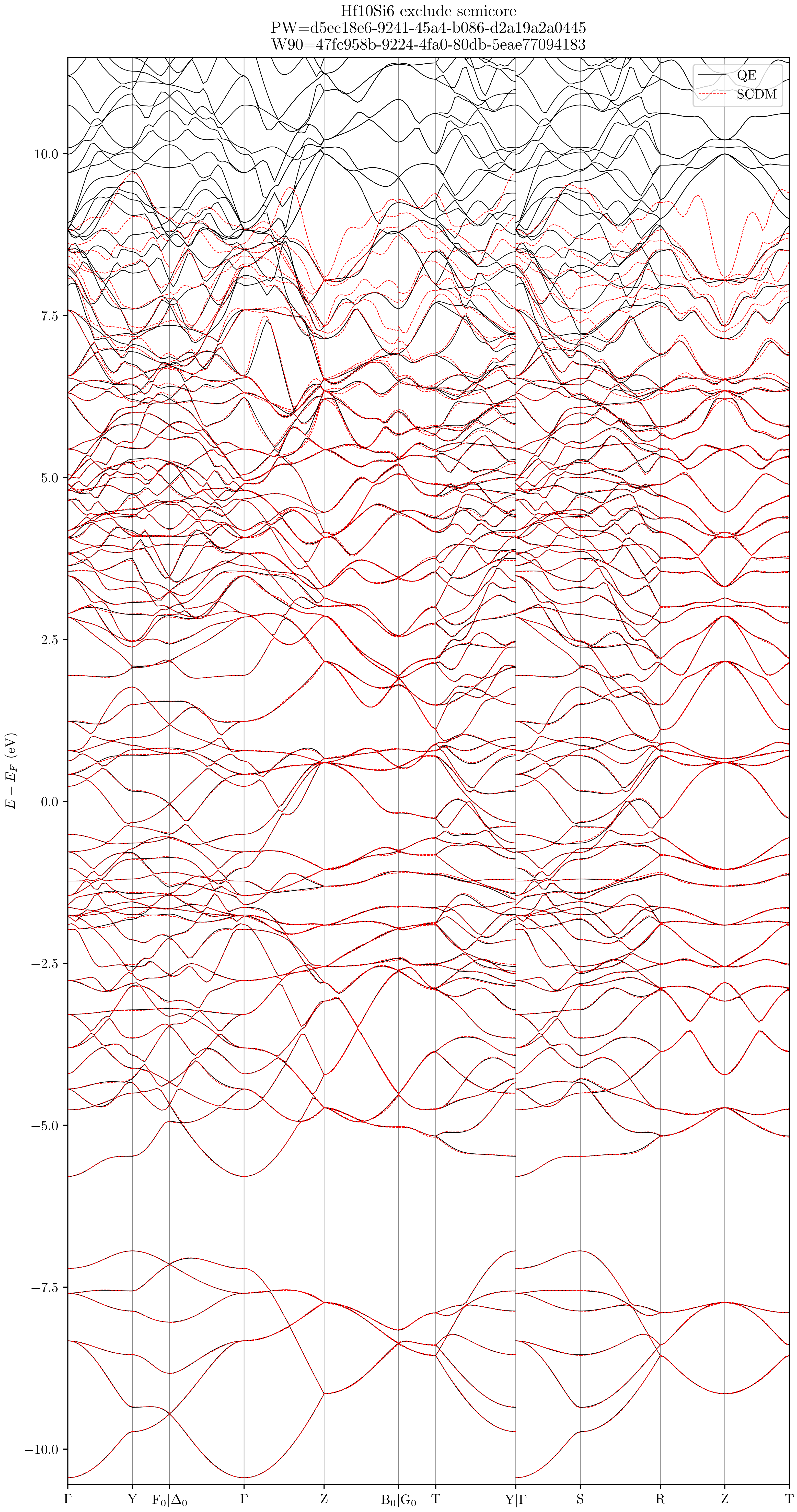

SCDM band distance (meV) and τ ($\AA$)

η_0	η_2	η_4	$\eta_0^{\max}$	$\eta_2^{\max}$	$\eta_4^{\max}$	τ
7.3	7.2	7.5	45.1	45.1	45.1	3.334

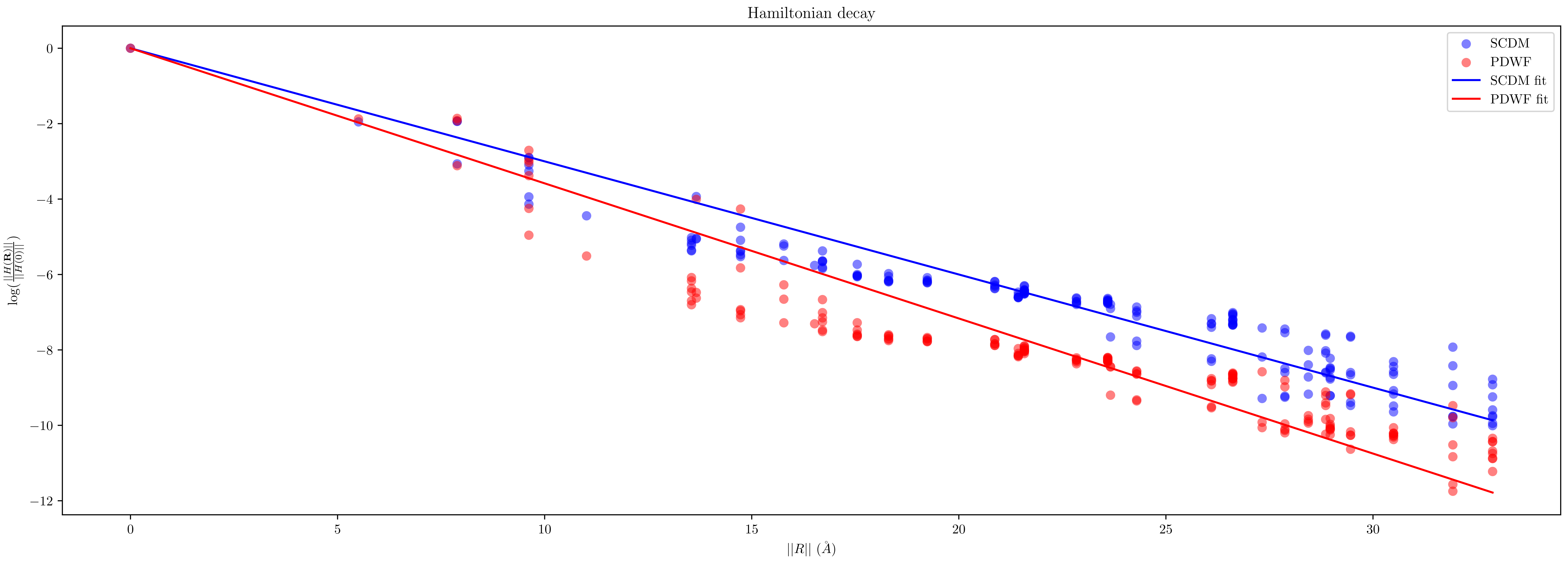

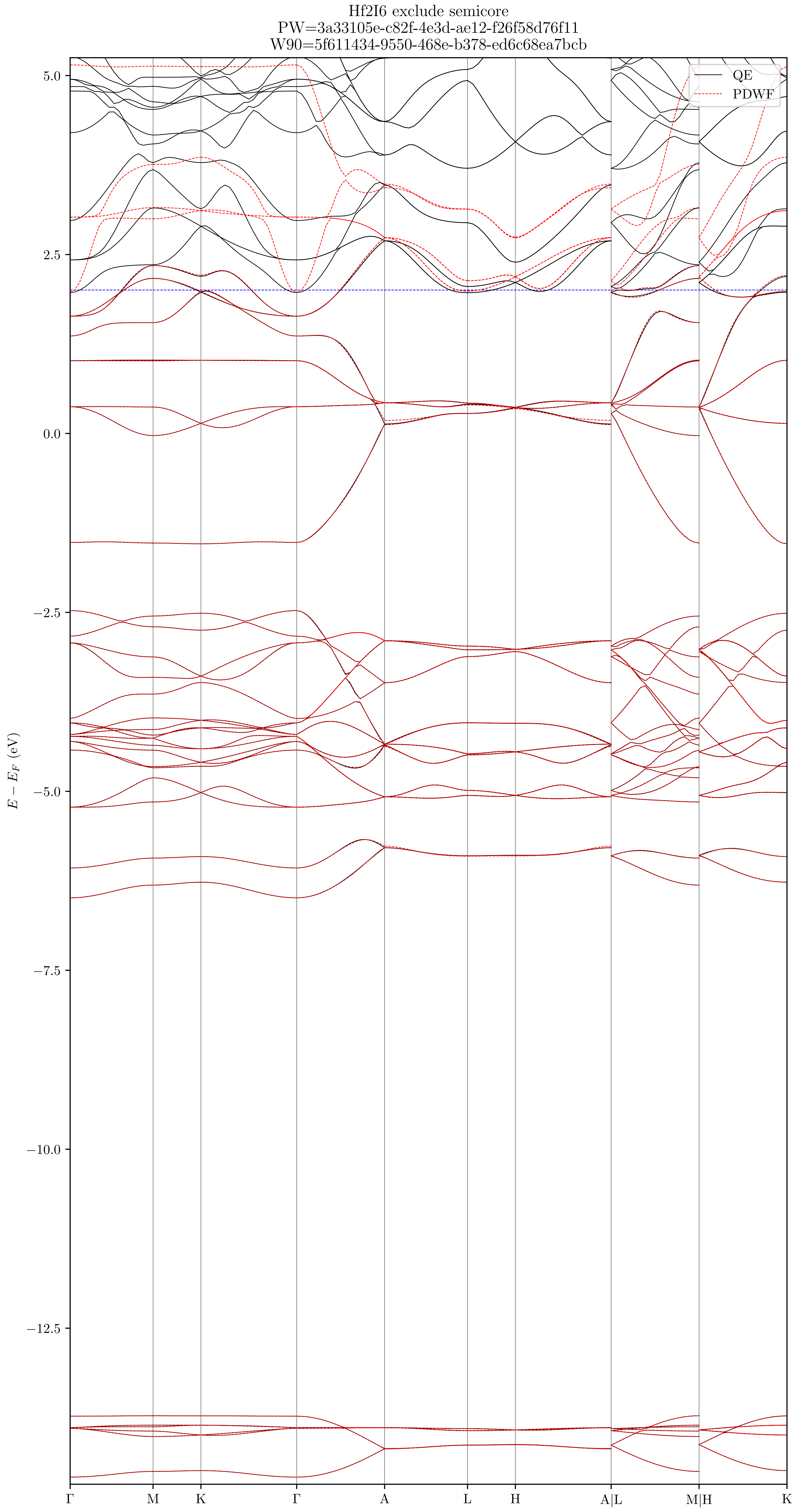

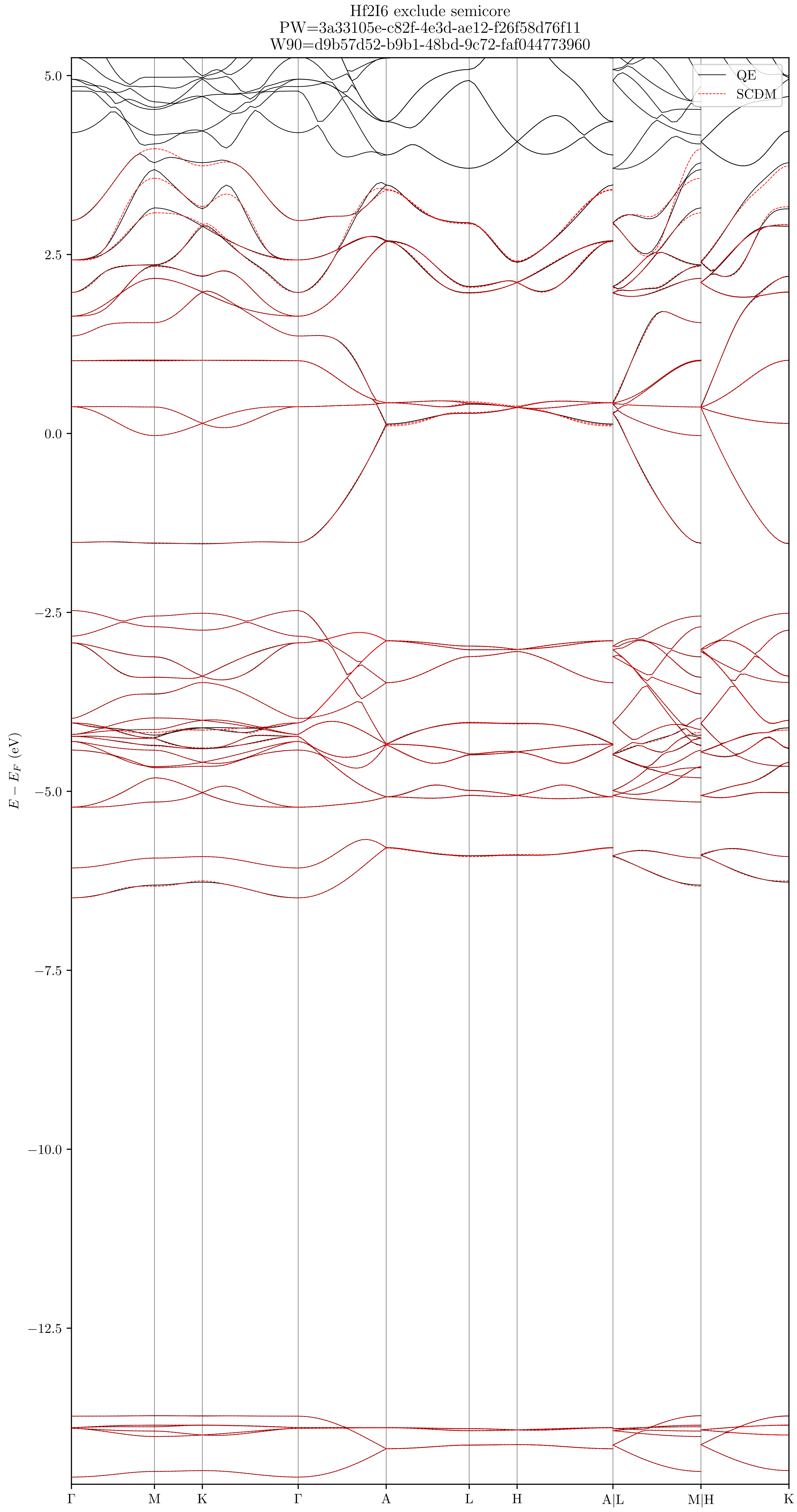

PDWF band distance (meV) and τ ($\AA$)

η_0	η_2	η_4	$\eta_0^{\max}$	$\eta_2^{\max}$	$\eta_4^{\max}$	τ
2.3	8.3	115.6	22.1	51.1	805.7	2.934

SCDM band distance (meV) and τ ($\AA$)

η_0	η_2	η_4	$\eta_0^{\max}$	$\eta_2^{\max}$	$\eta_4^{\max}$	τ
4.1	4.7	11.2	39.0	39.0	152.6	3.097

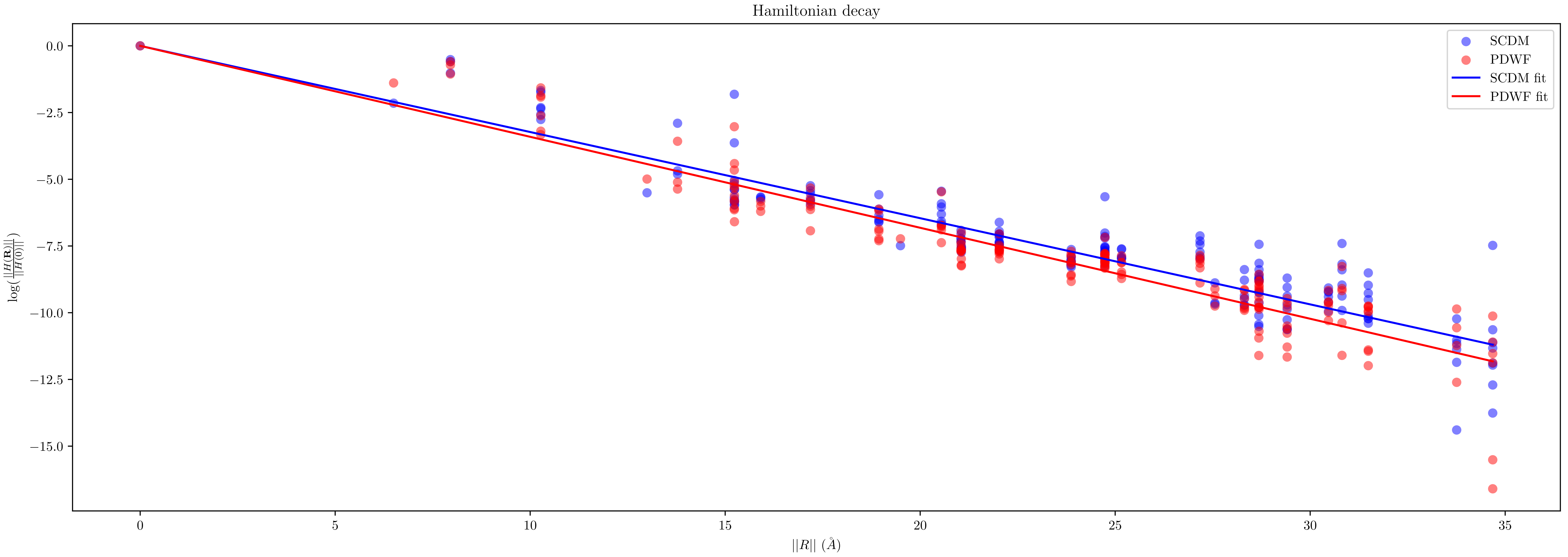

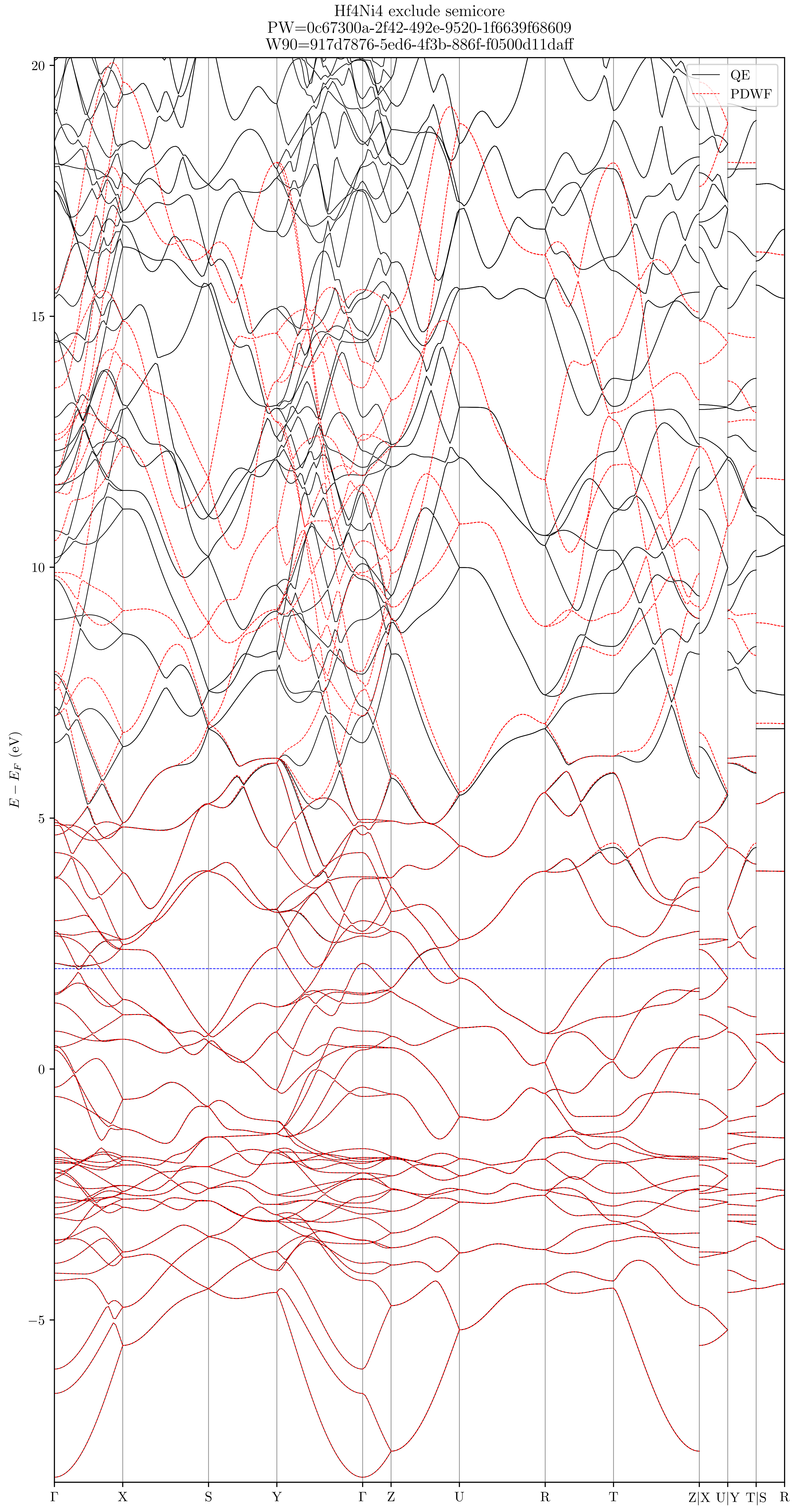

PDWF band distance (meV) and τ ($\AA$)

η_0	η_2	η_4	$\eta_0^{\max}$	$\eta_2^{\max}$	$\eta_4^{\max}$	τ
0.5	0.7	1.6	6.3	6.3	21.0	2.258

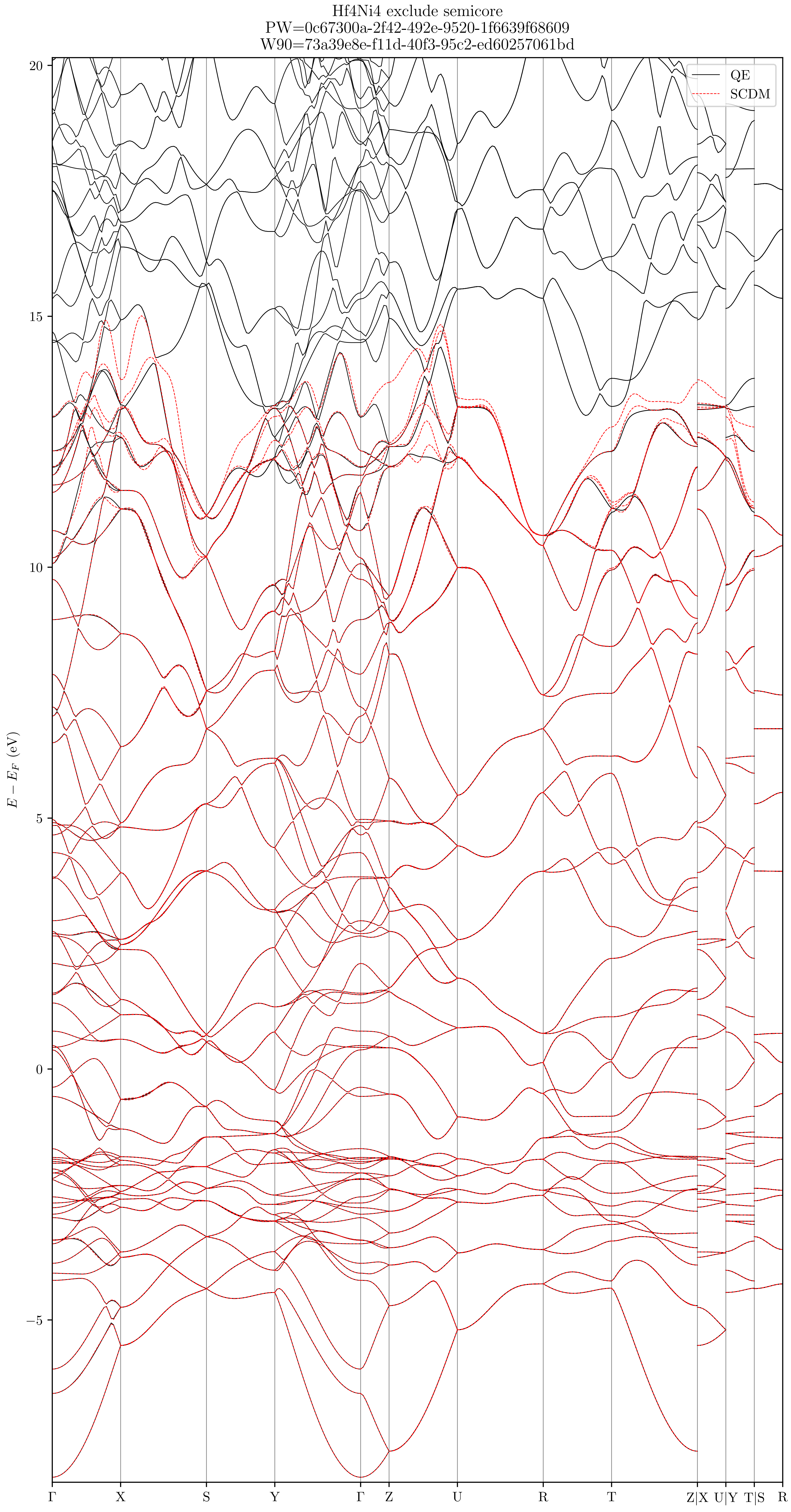

SCDM band distance (meV) and τ ($\AA$)

η_0	η_2	η_4	$\eta_0^{\max}$	$\eta_2^{\max}$	$\eta_4^{\max}$	τ
1.9	2.1	2.3	24.9	24.9	24.9	2.661

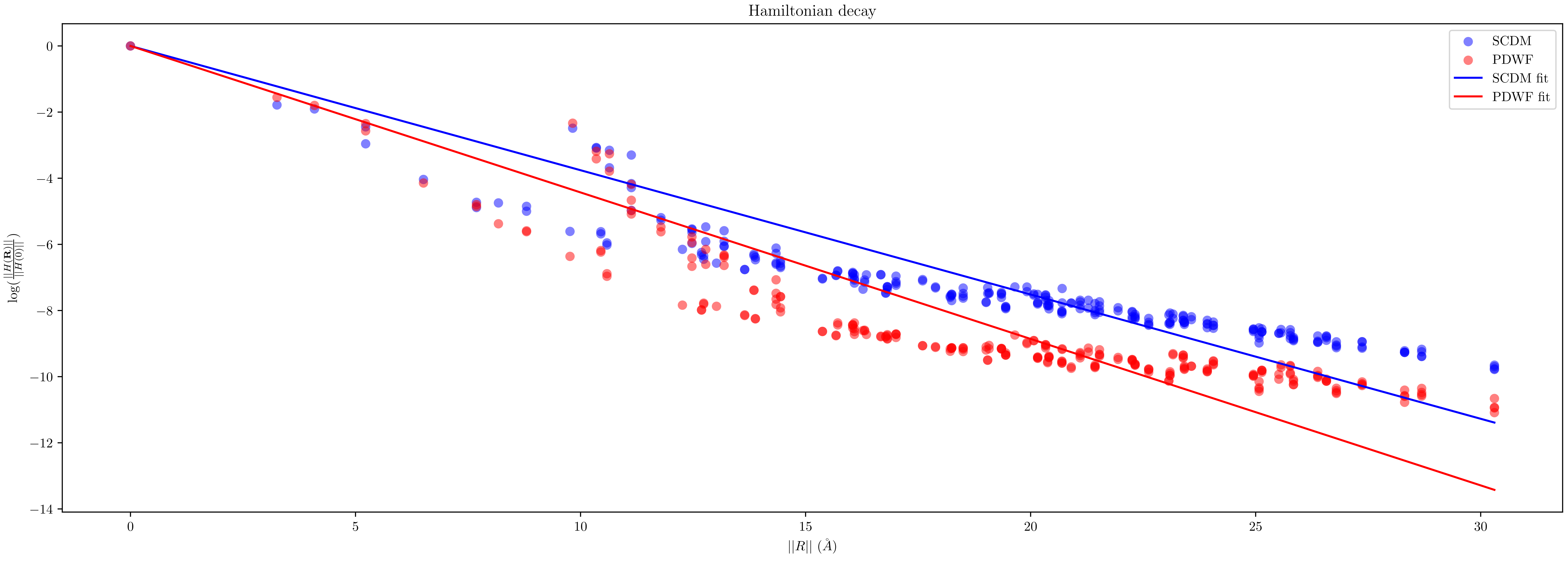

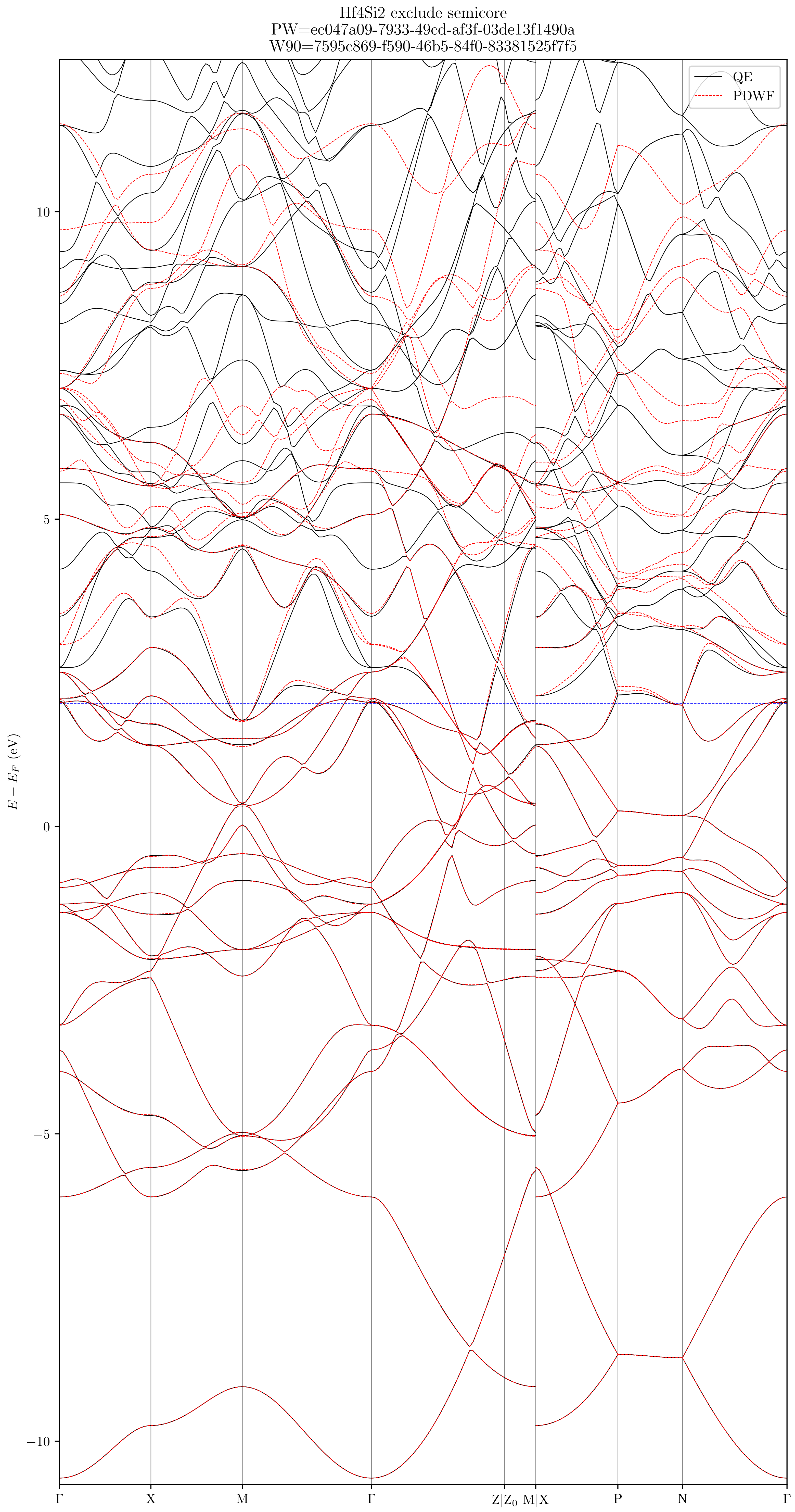

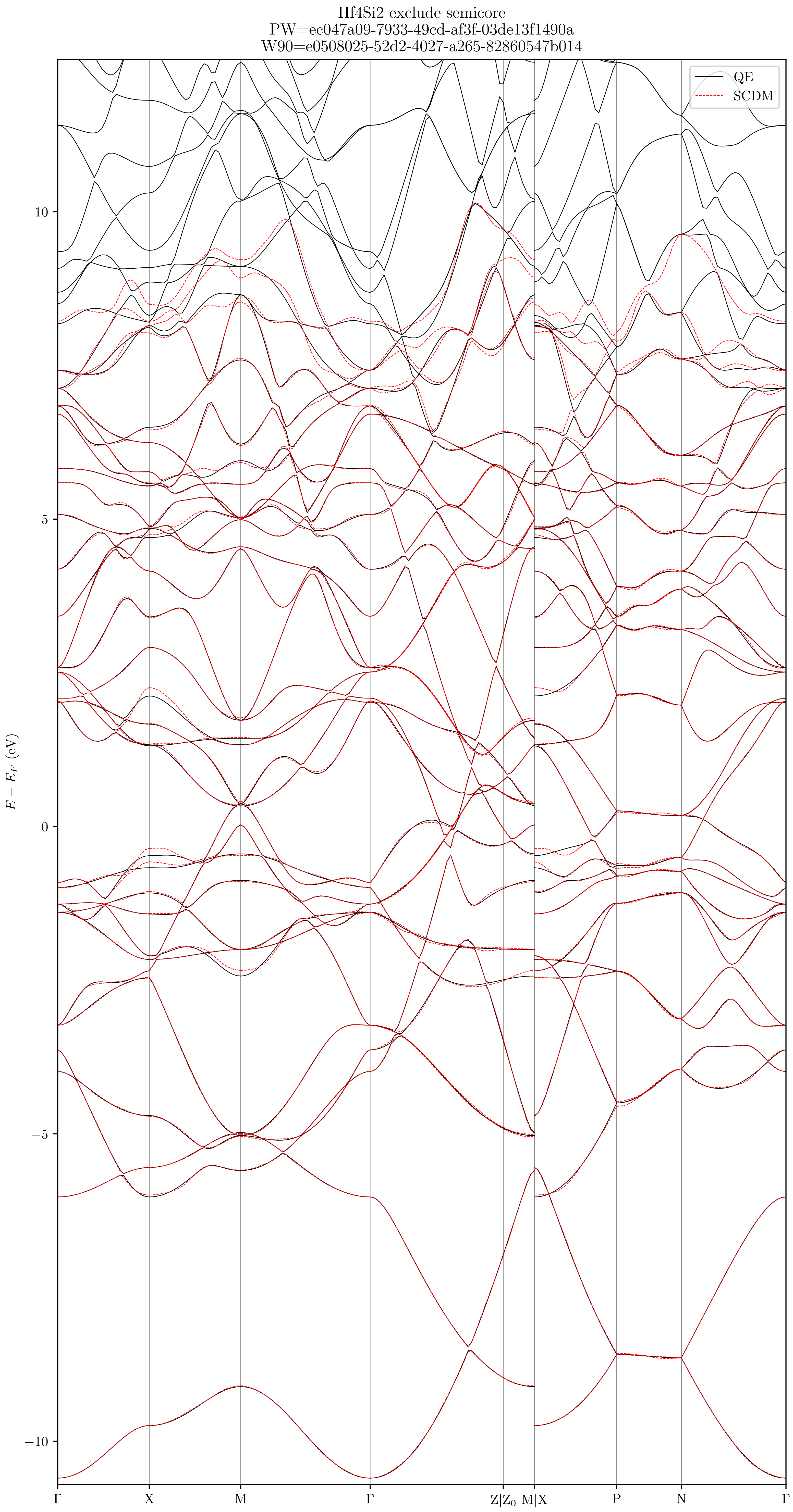

PDWF band distance (meV) and τ ($\AA$)

η_0	η_2	η_4	$\eta_0^{\max}$	$\eta_2^{\max}$	$\eta_4^{\max}$	τ
3.6	6.3	77.1	20.2	50.4	537.7	2.486

SCDM band distance (meV) and τ ($\AA$)

η_0	η_2	η_4	$\eta_0^{\max}$	$\eta_2^{\max}$	$\eta_4^{\max}$	τ
17.7	17.2	17.3	117.7	119.9	134.7	2.894

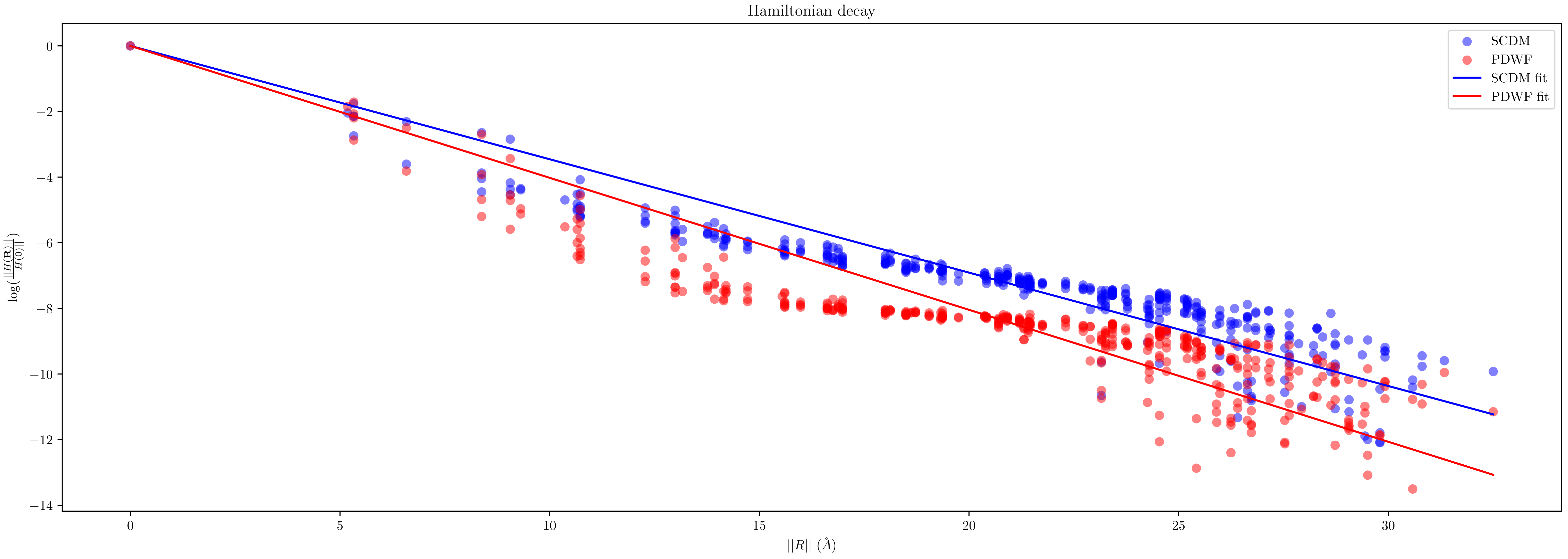

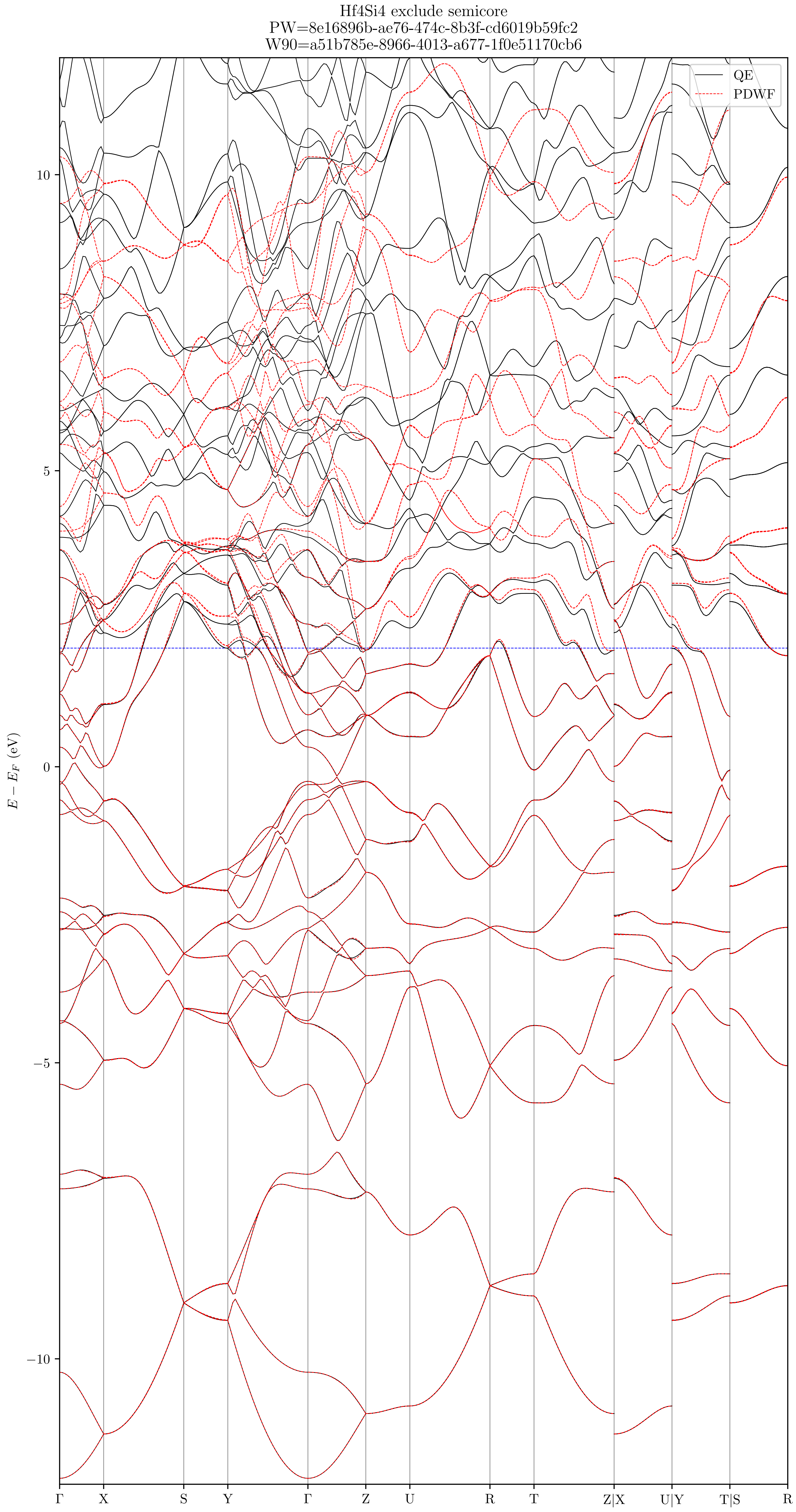

PDWF band distance (meV) and τ ($\AA$)

η_0	η_2	η_4	$\eta_0^{\max}$	$\eta_2^{\max}$	$\eta_4^{\max}$	τ
4.2	8.4	76.6	29.1	75.1	514.6	2.521

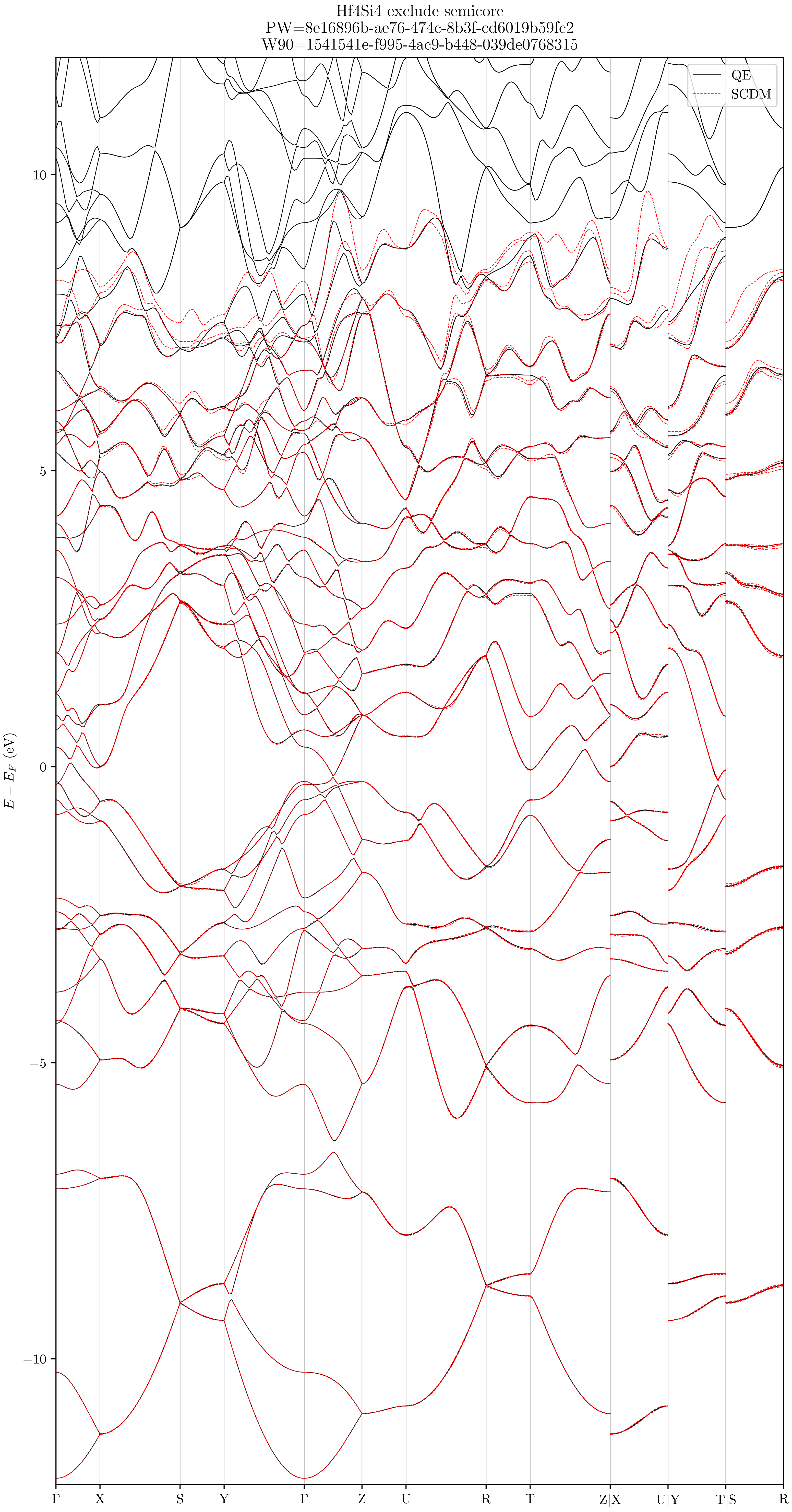

SCDM band distance (meV) and τ ($\AA$)

η_0	η_2	η_4	$\eta_0^{\max}$	$\eta_2^{\max}$	$\eta_4^{\max}$	τ
8.5	8.8	10.0	43.2	43.2	62.2	2.849

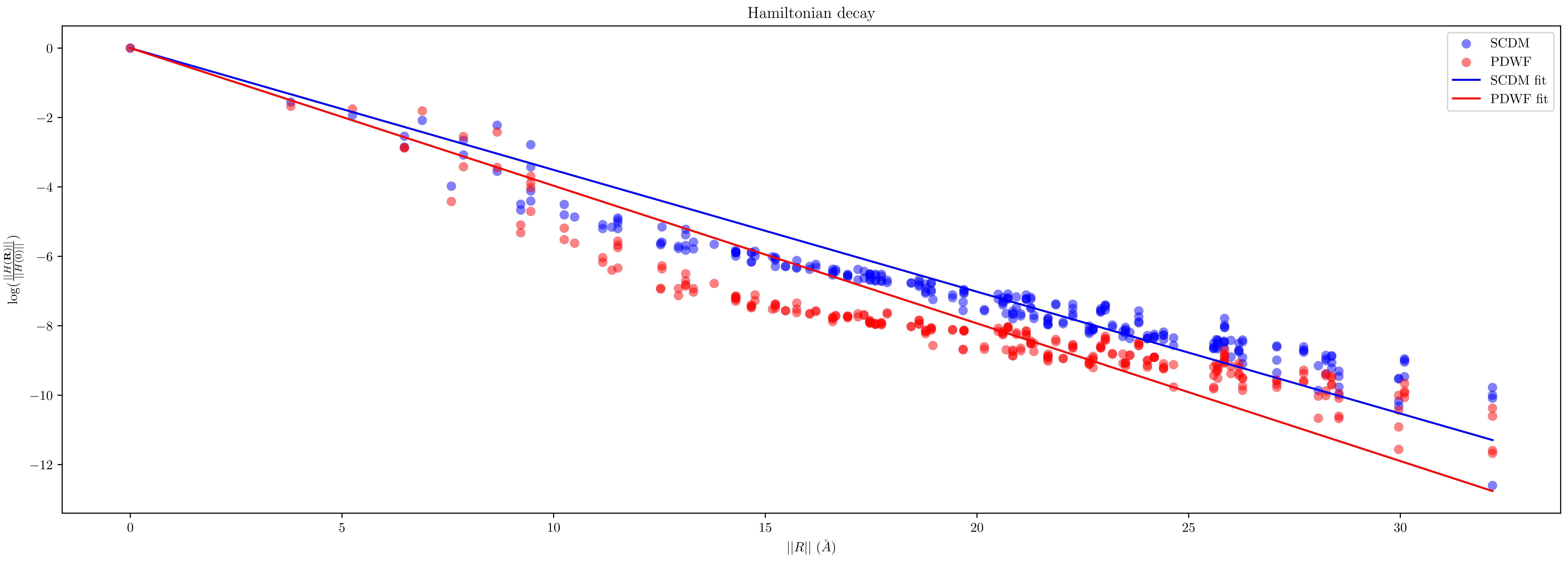

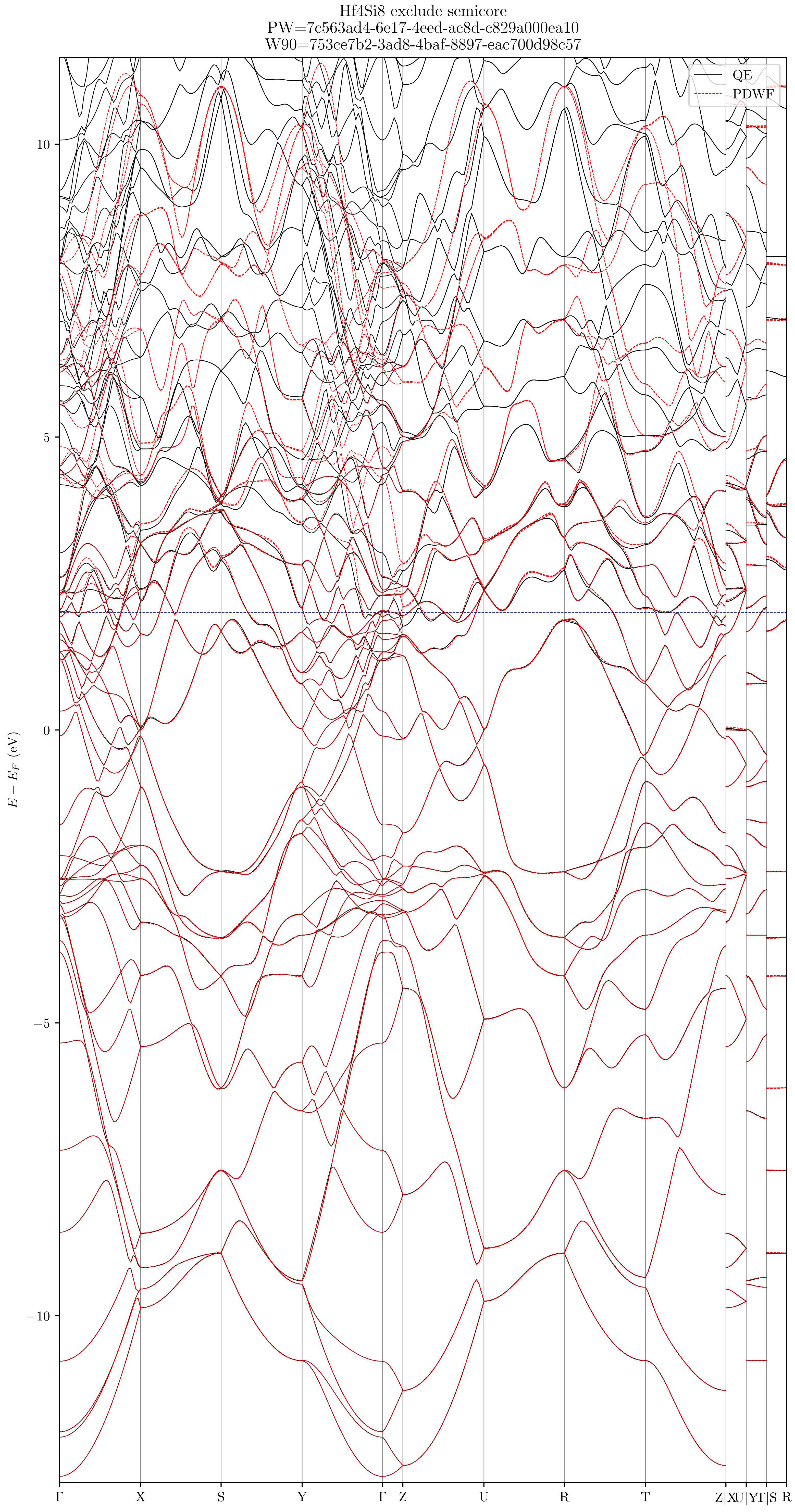

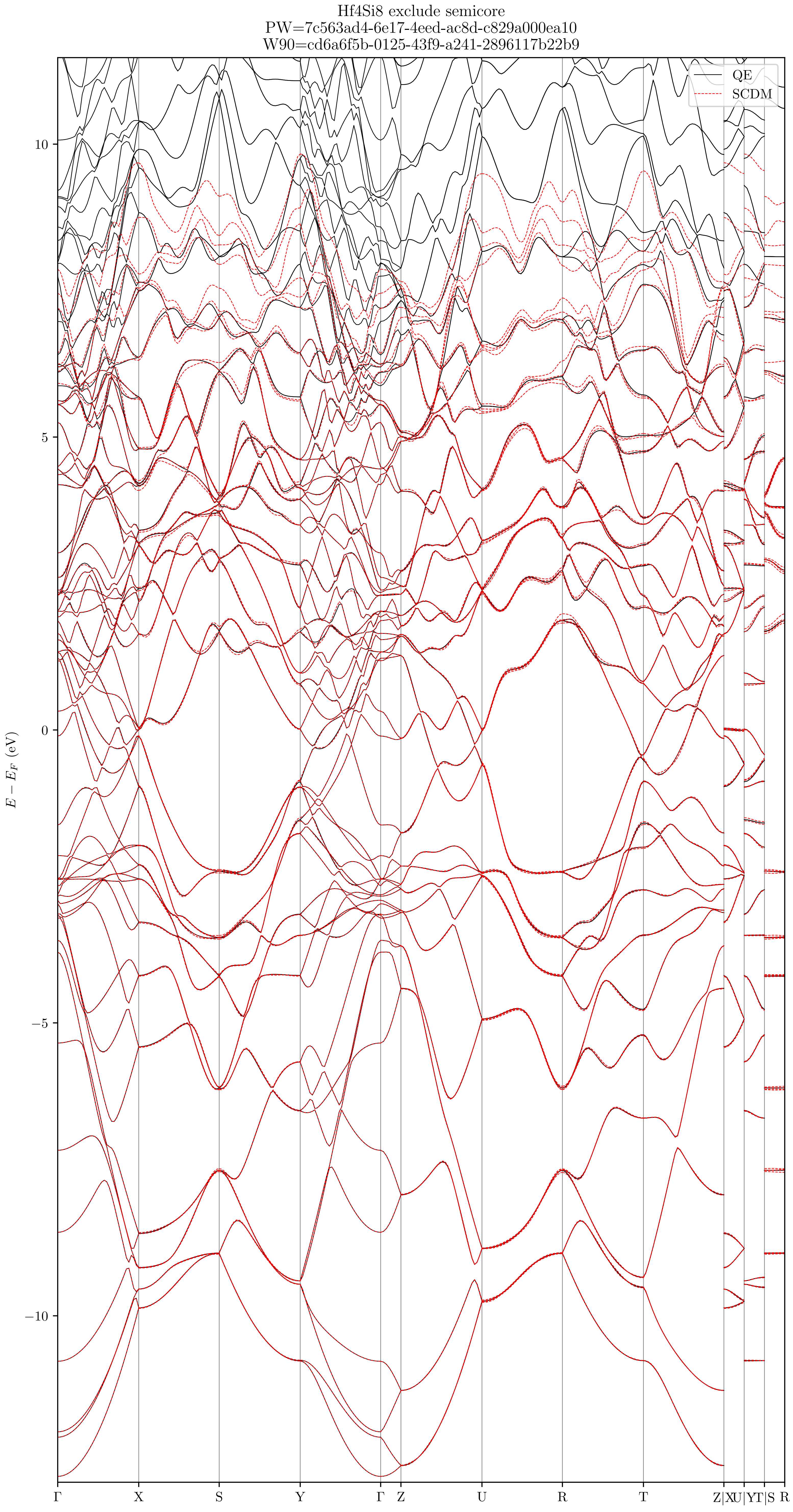

PDWF band distance (meV) and τ ($\AA$)

η_0	η_2	η_4	$\eta_0^{\max}$	$\eta_2^{\max}$	$\eta_4^{\max}$	τ
4.6	10.4	47.3	23.3	136.8	1095.8	3.200

SCDM band distance (meV) and τ ($\AA$)

η_0	η_2	η_4	$\eta_0^{\max}$	$\eta_2^{\max}$	$\eta_4^{\max}$	τ
9.9	11.2	13.4	48.3	79.2	117.9	3.411

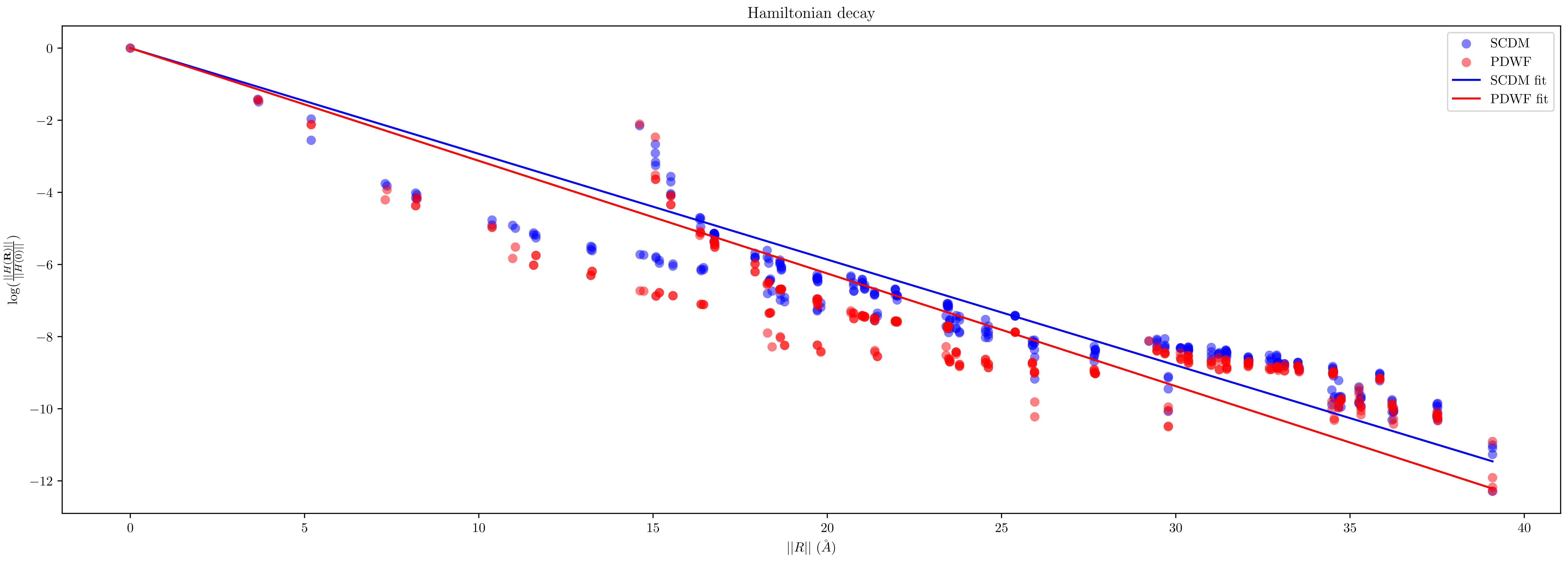

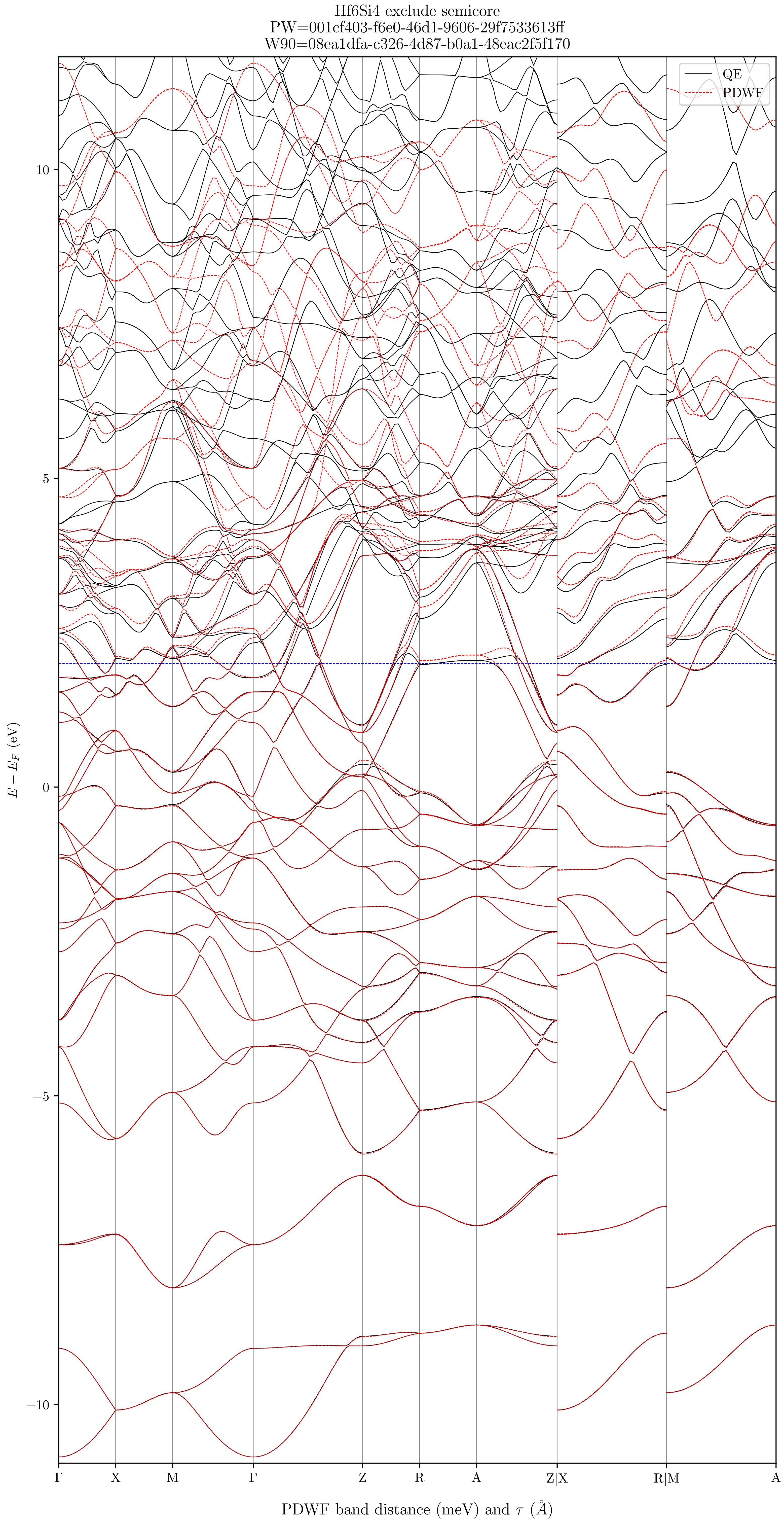

η_0	η_2	η_4	$\eta_0^{\max}$	$\eta_2^{\max}$	$\eta_4^{\max}$	τ
5.5	9.3	52.8	26.8	66.1	425.5	2.749

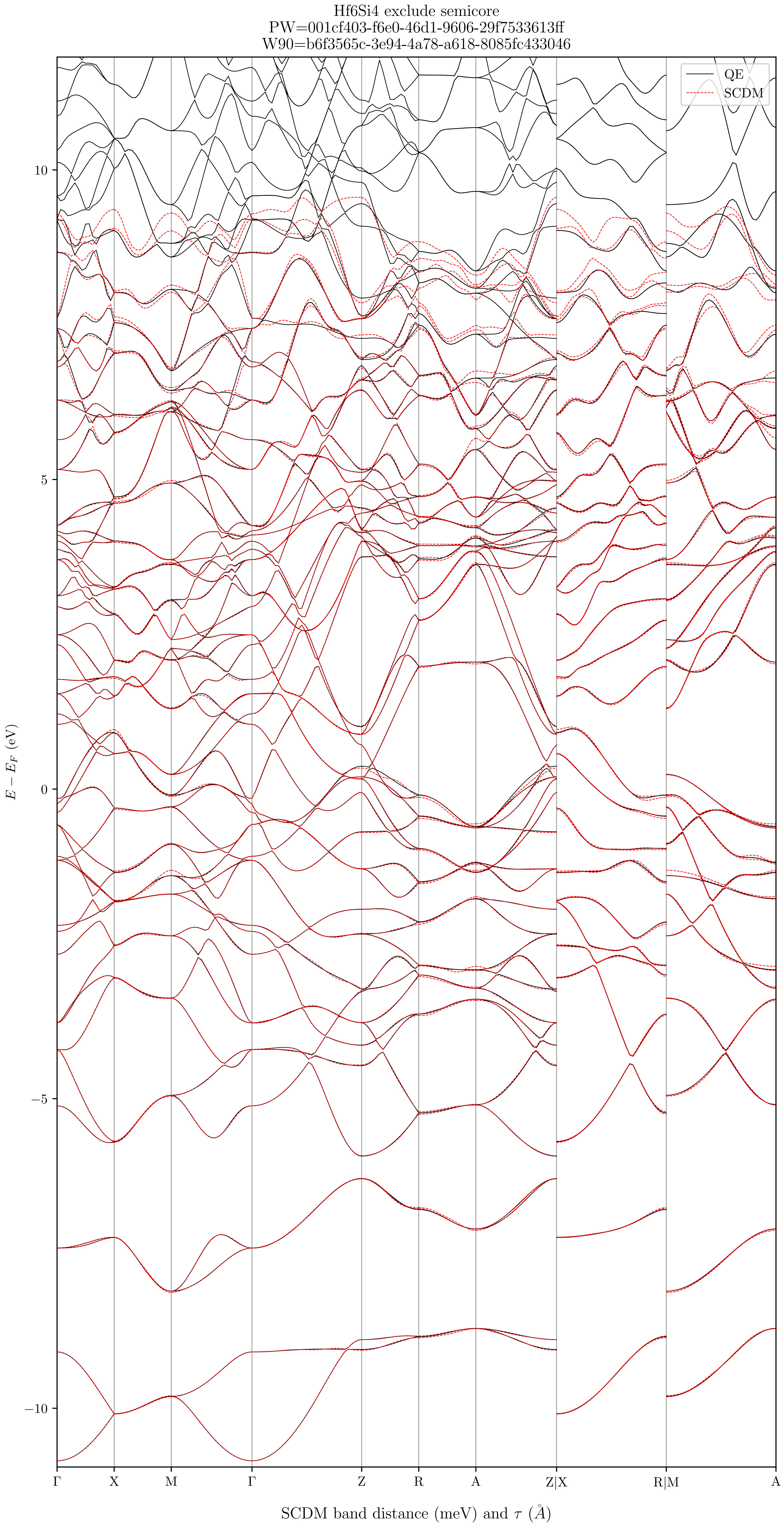

η_0	η_2	η_4	$\eta_0^{\max}$	$\eta_2^{\max}$	$\eta_4^{\max}$	τ
11.9	12.0	11.5	86.4	86.4	86.4	3.031

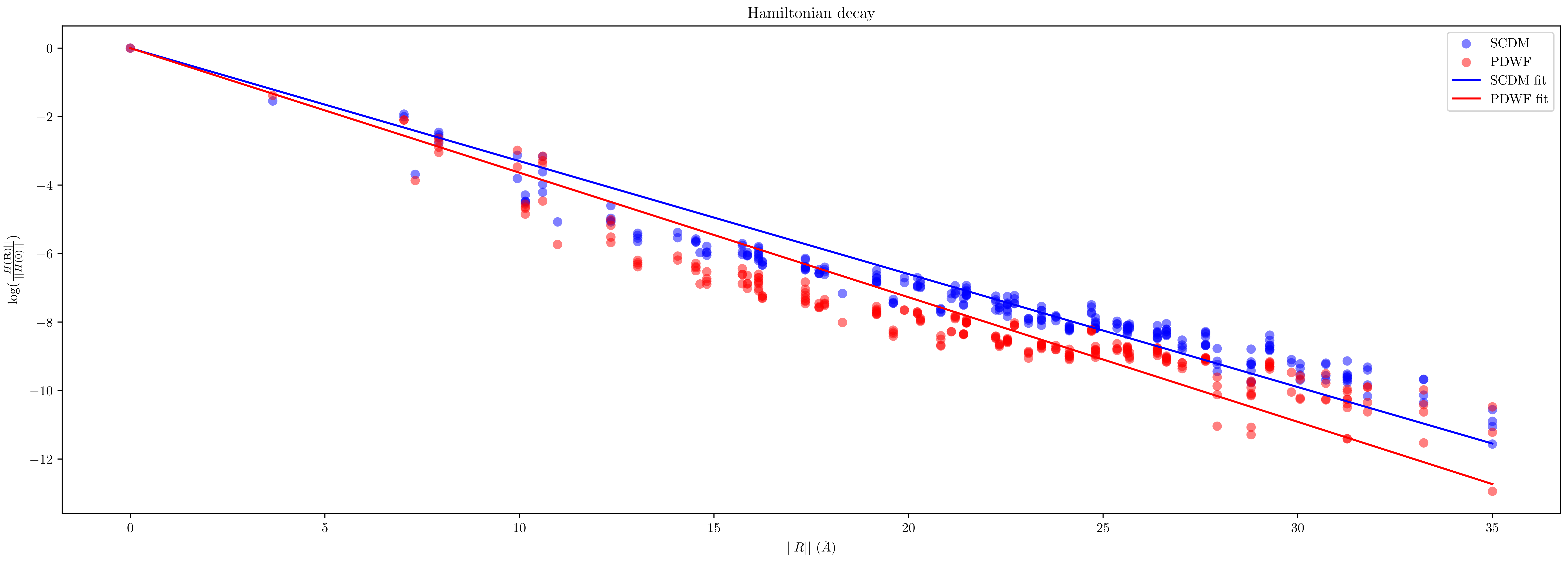

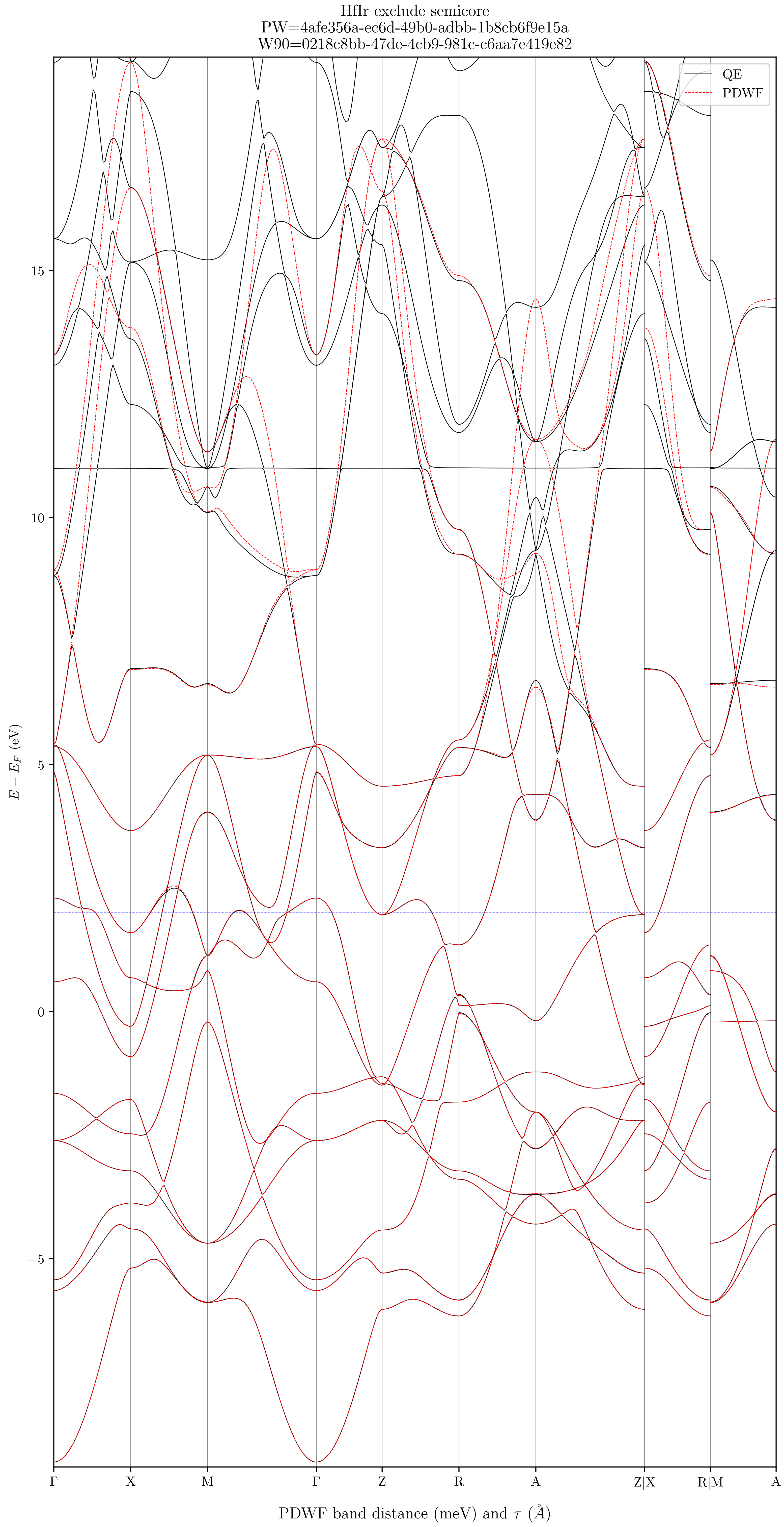

η_0	η_2	η_4	η_0^{max}	η_2^{max}	η_4^{max}	τ
2.7	3.0	4.3	16.5	17.6	51.3	1.971

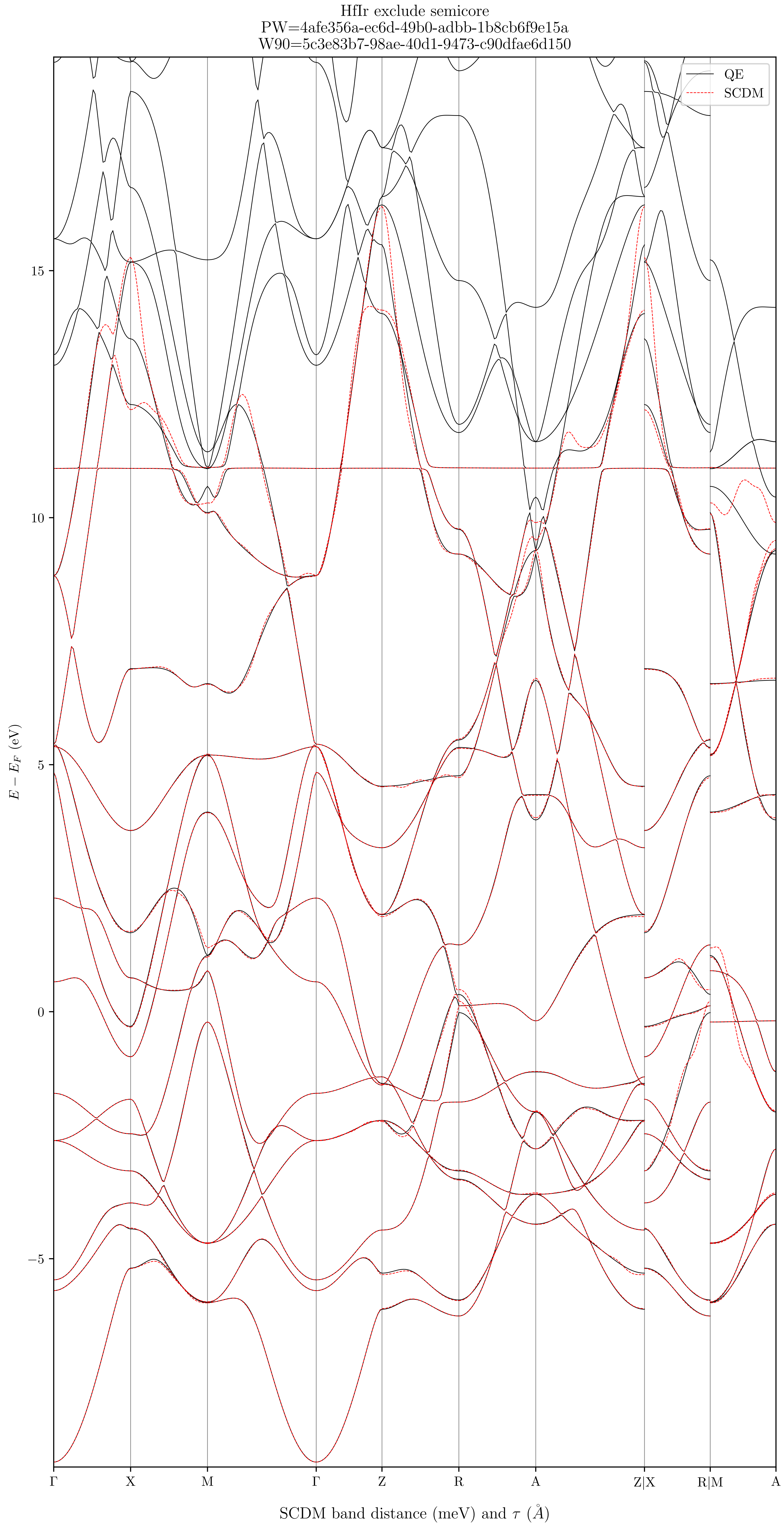

η_0	η_2	η_4	η_0^{max}	η_2^{max}	η_4^{max}	τ
25.6	31.1	29.7	403.5	409.9	409.9	2.348

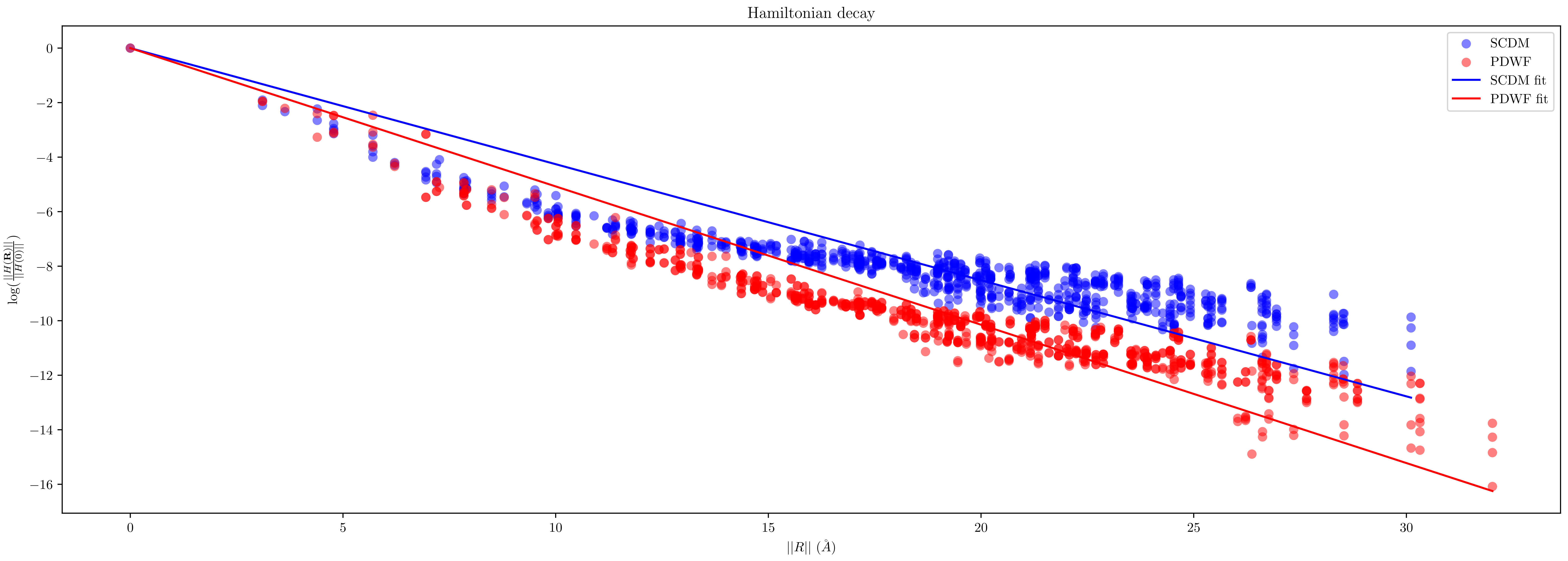

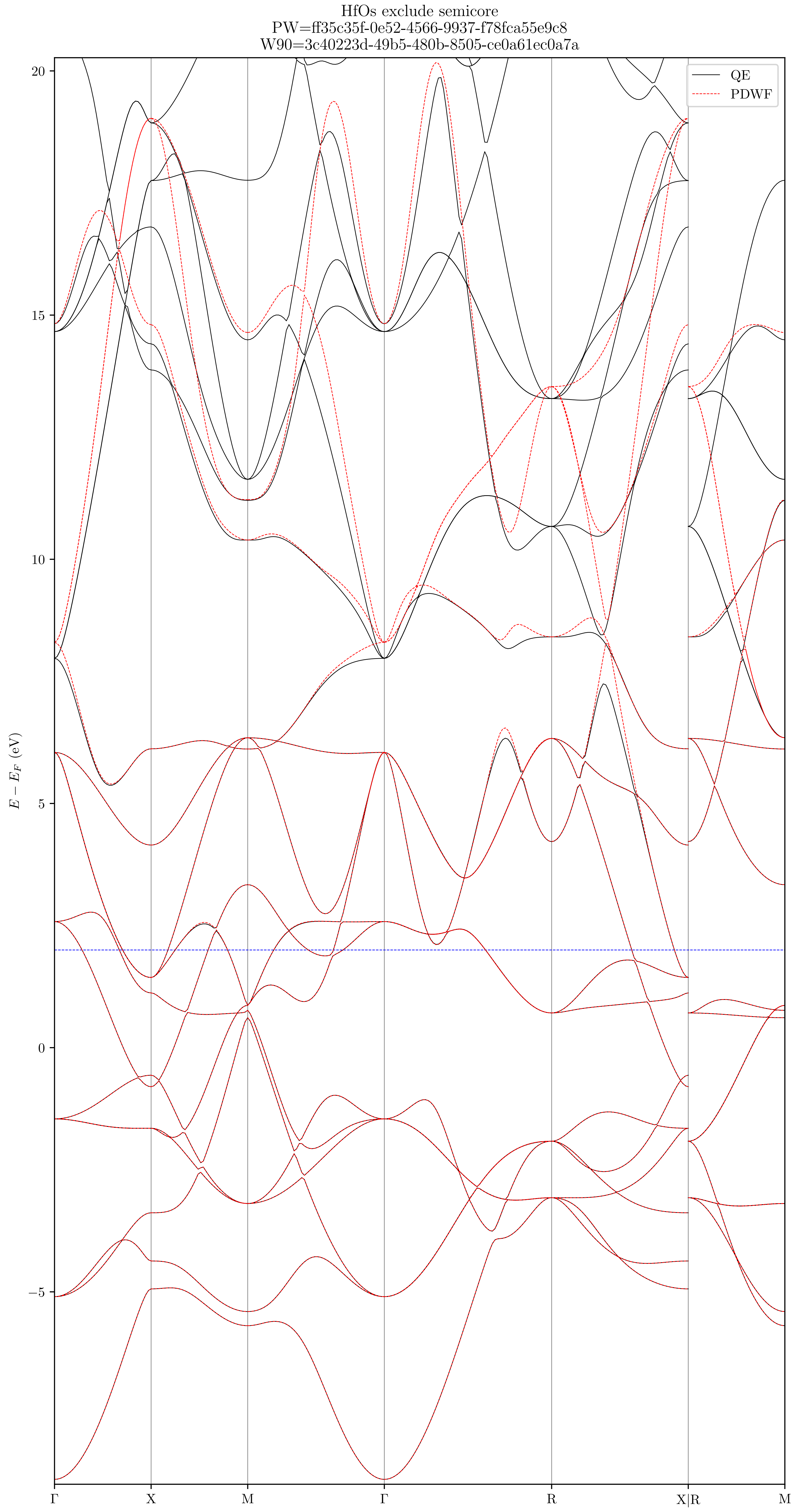

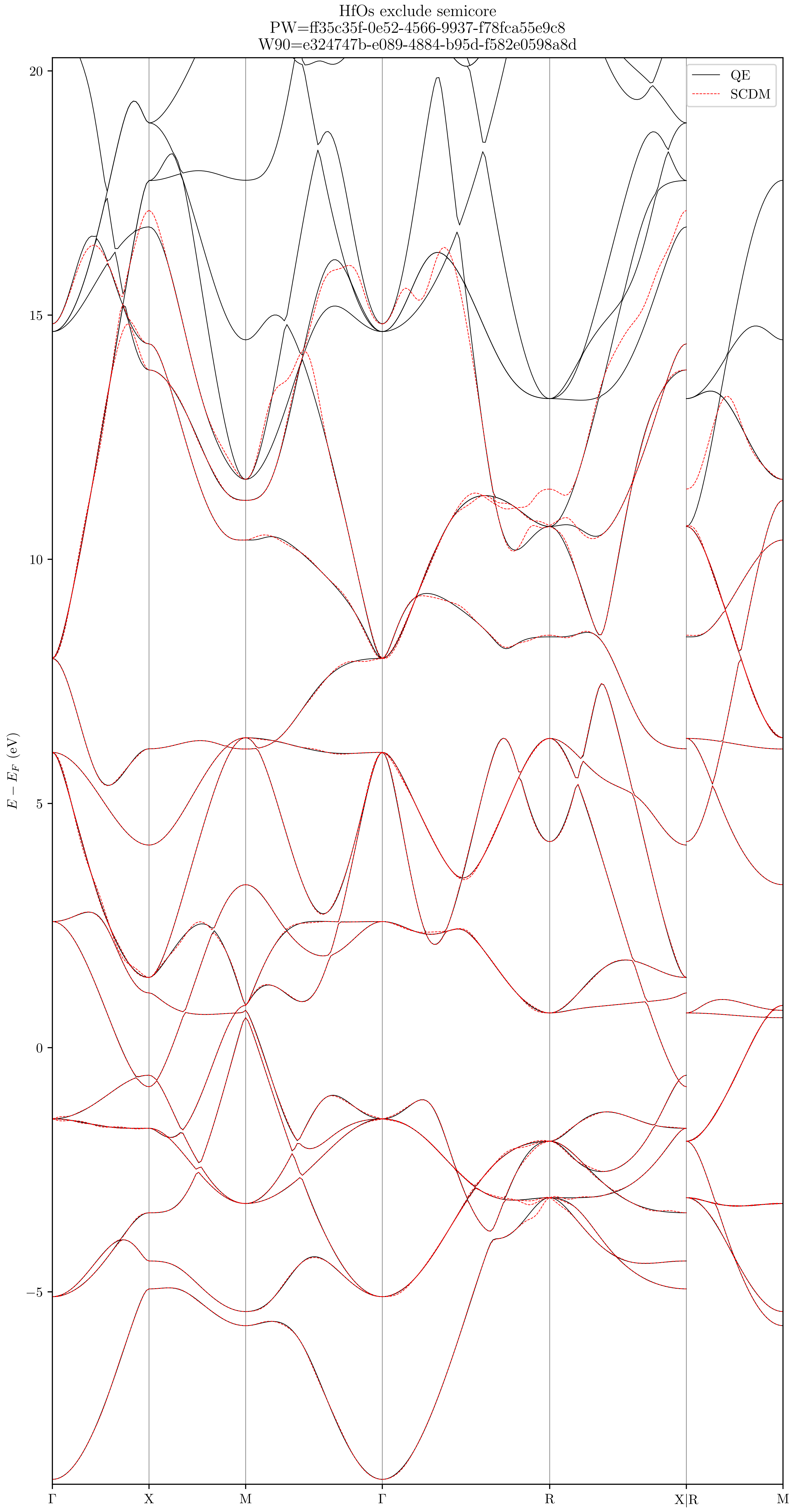

PDWF band distance (meV) and τ ($\AA$)

η_0	η_2	η_4	$\eta_0^{\max}$	$\eta_2^{\max}$	$\eta_4^{\max}$	τ
1.1	1.1	2.2	7.7	7.7	29.7	1.823

SCDM band distance (meV) and τ ($\AA$)

η_0	η_2	η_4	$\eta_0^{\max}$	$\eta_2^{\max}$	$\eta_4^{\max}$	τ
15.9	15.0	15.5	188.3	188.3	188.3	2.312

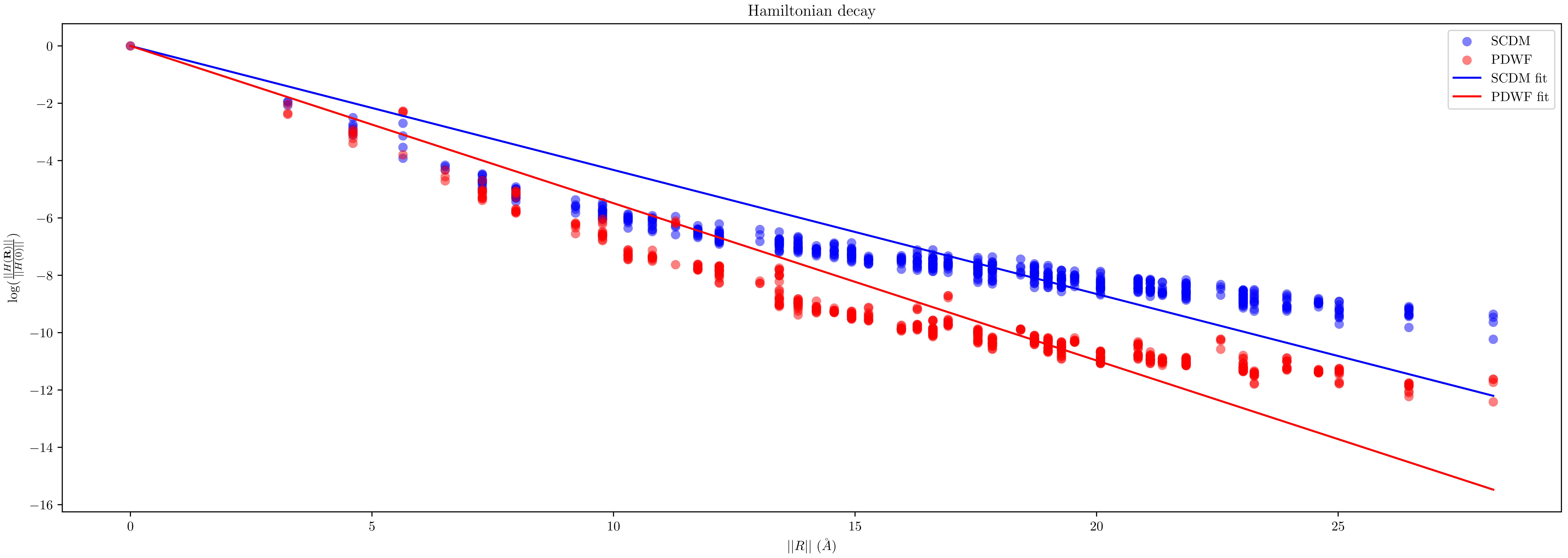

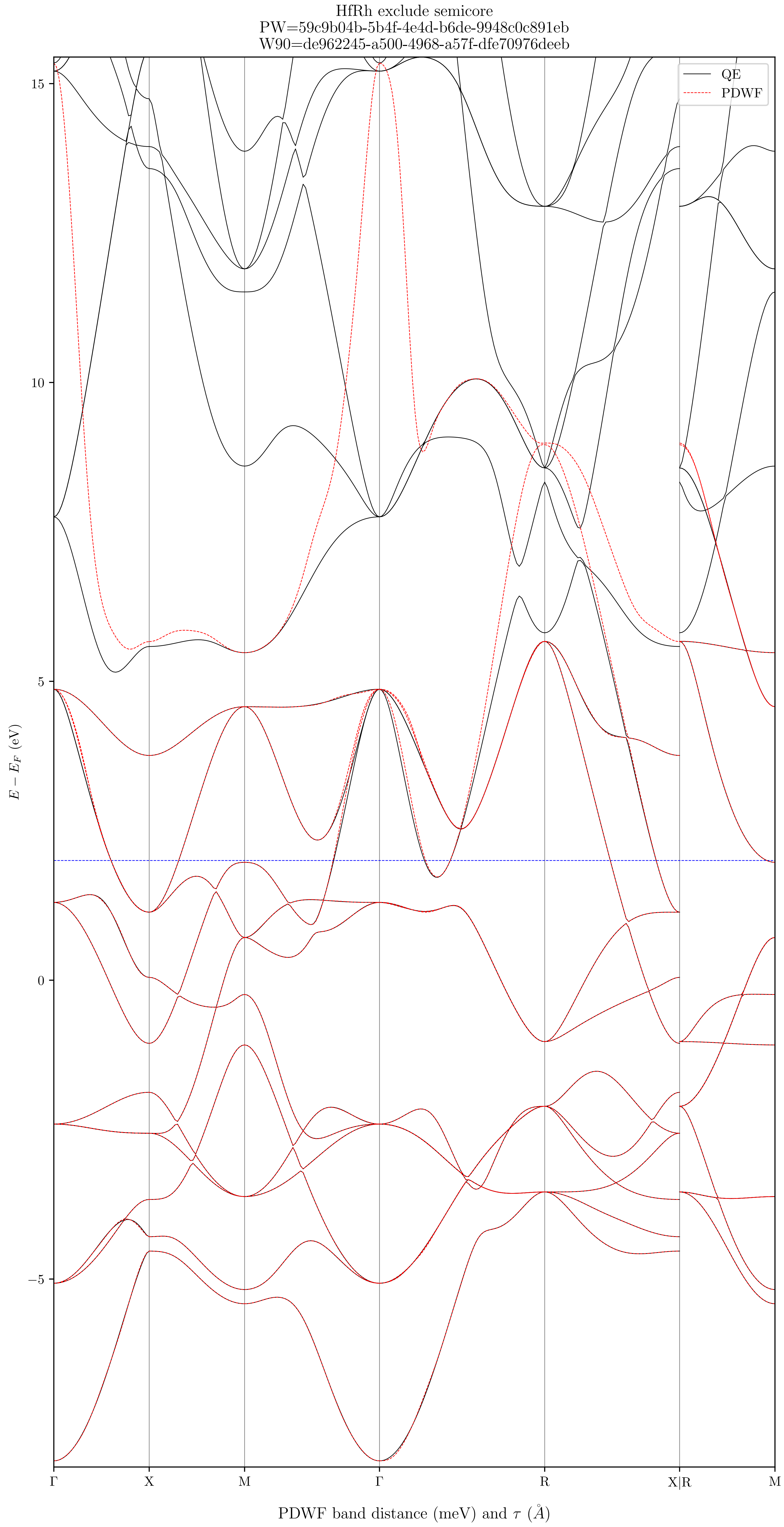

η_0	η_2	η_4	$\eta_0^{\max}$	$\eta_2^{\max}$	$\eta_4^{\max}$	τ
3.6	3.9	22.7	35.9	35.9	227.0	2.083

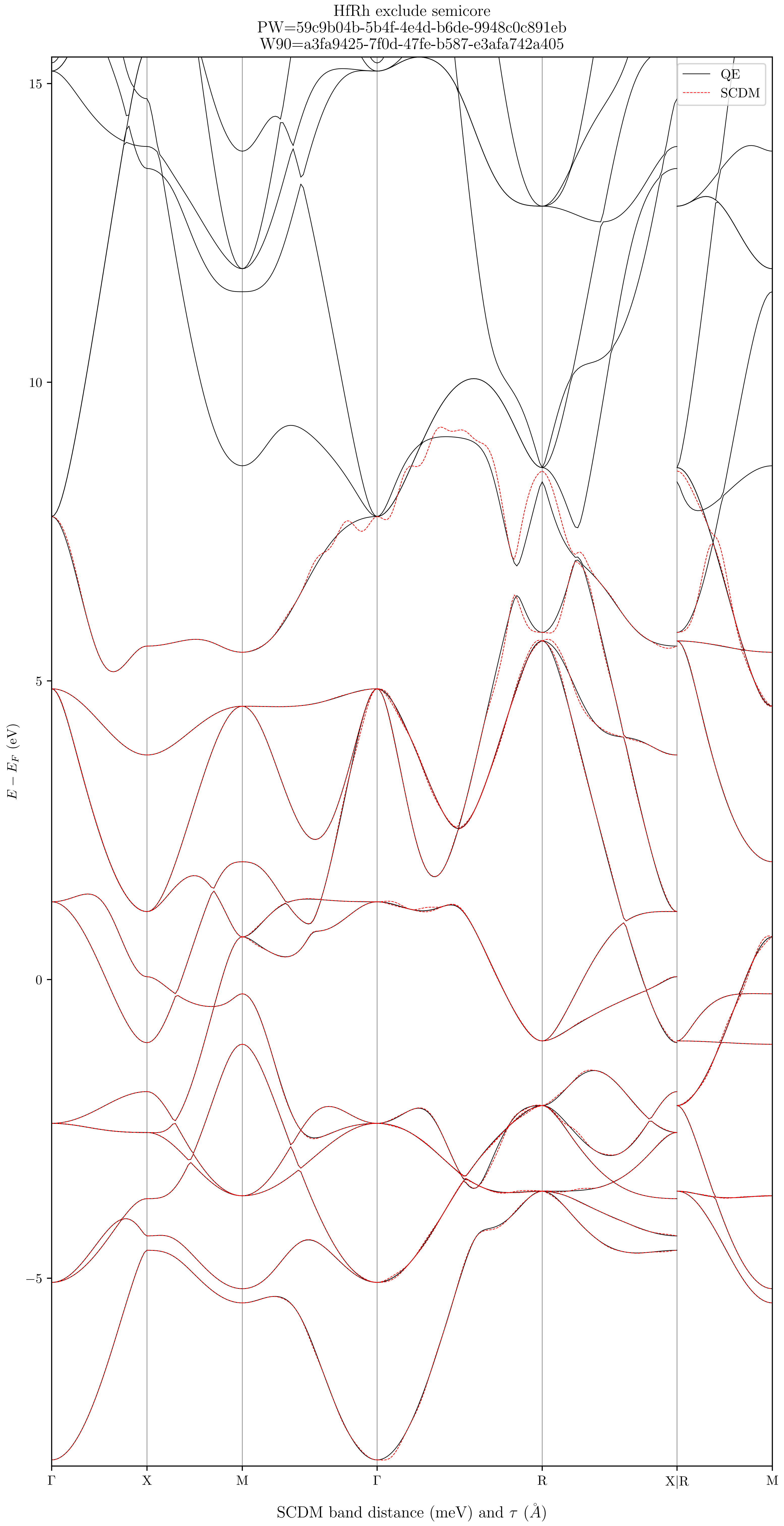

η_0	η_2	η_4	$\eta_0^{\max}$	$\eta_2^{\max}$	$\eta_4^{\max}$	τ
9.9	10.3	10.9	75.1	75.1	75.1	2.353

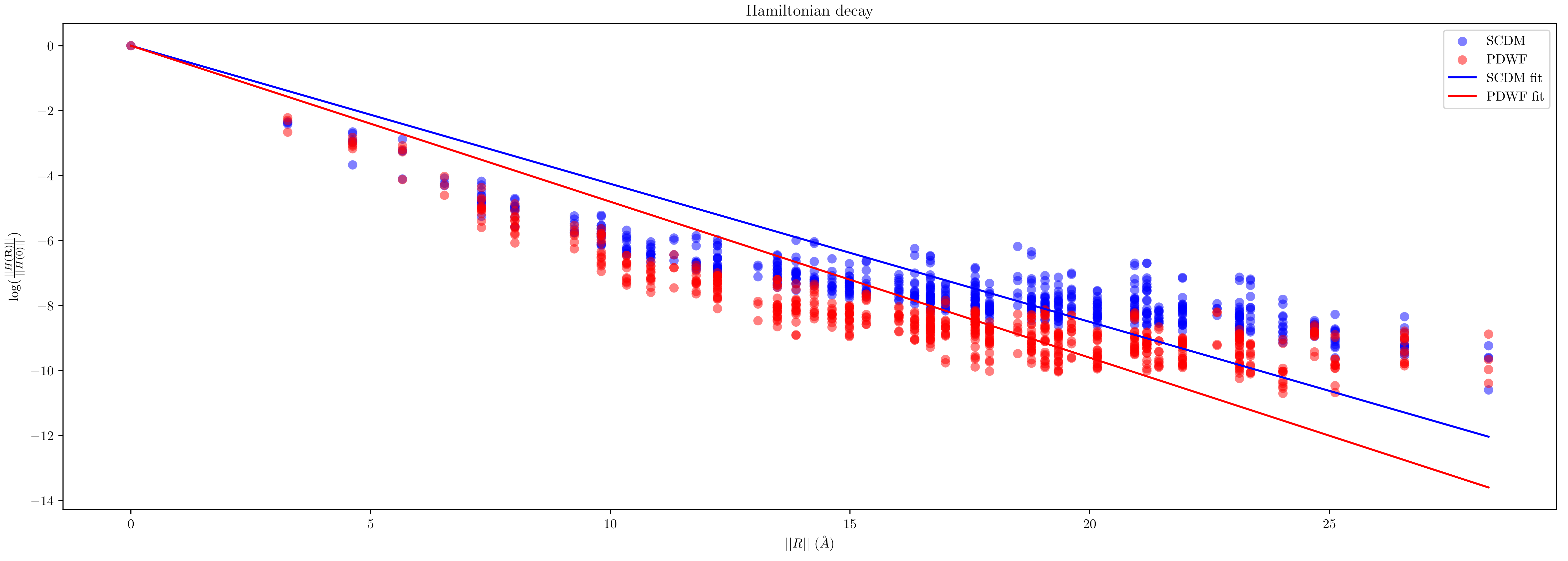

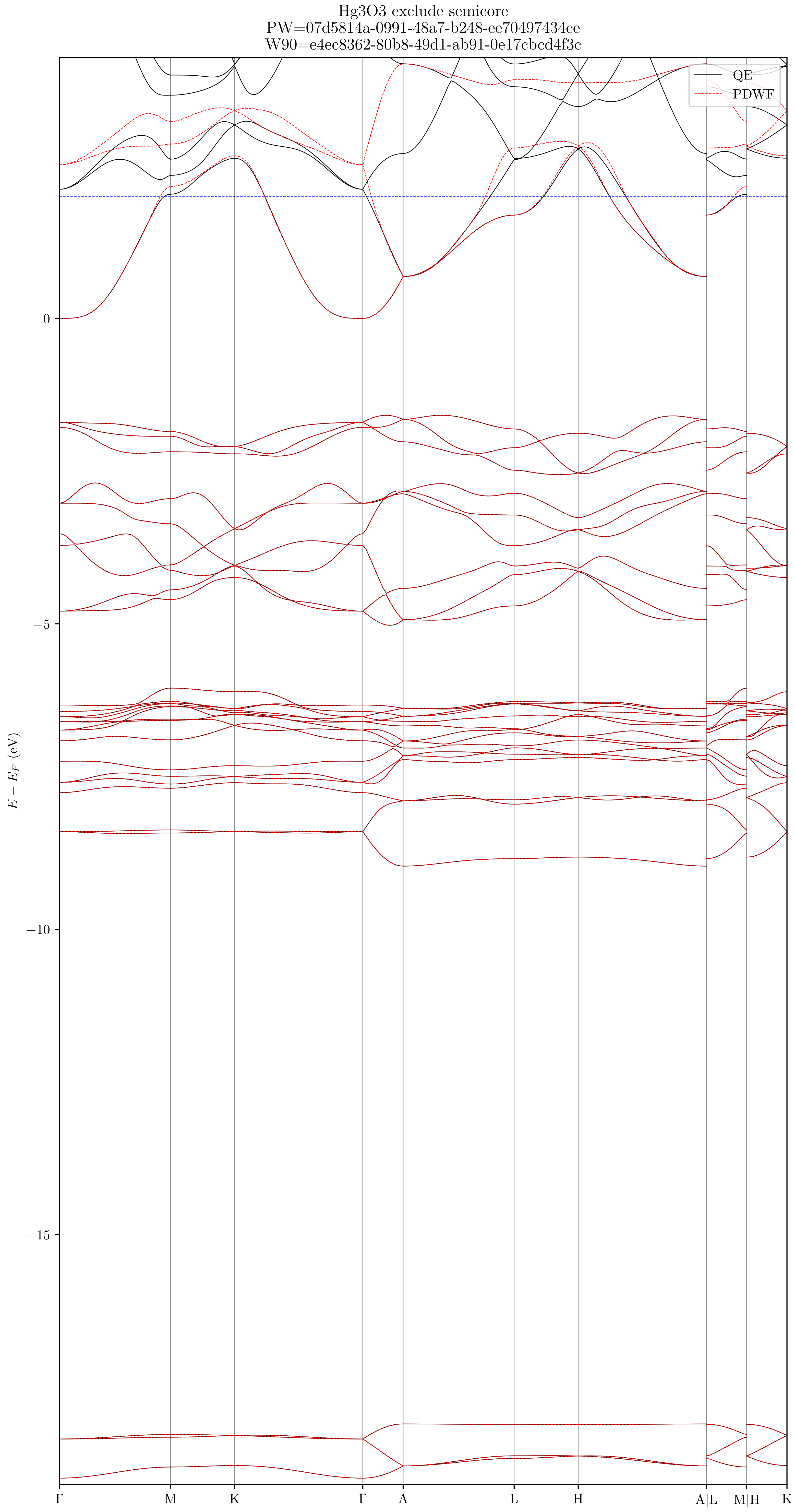

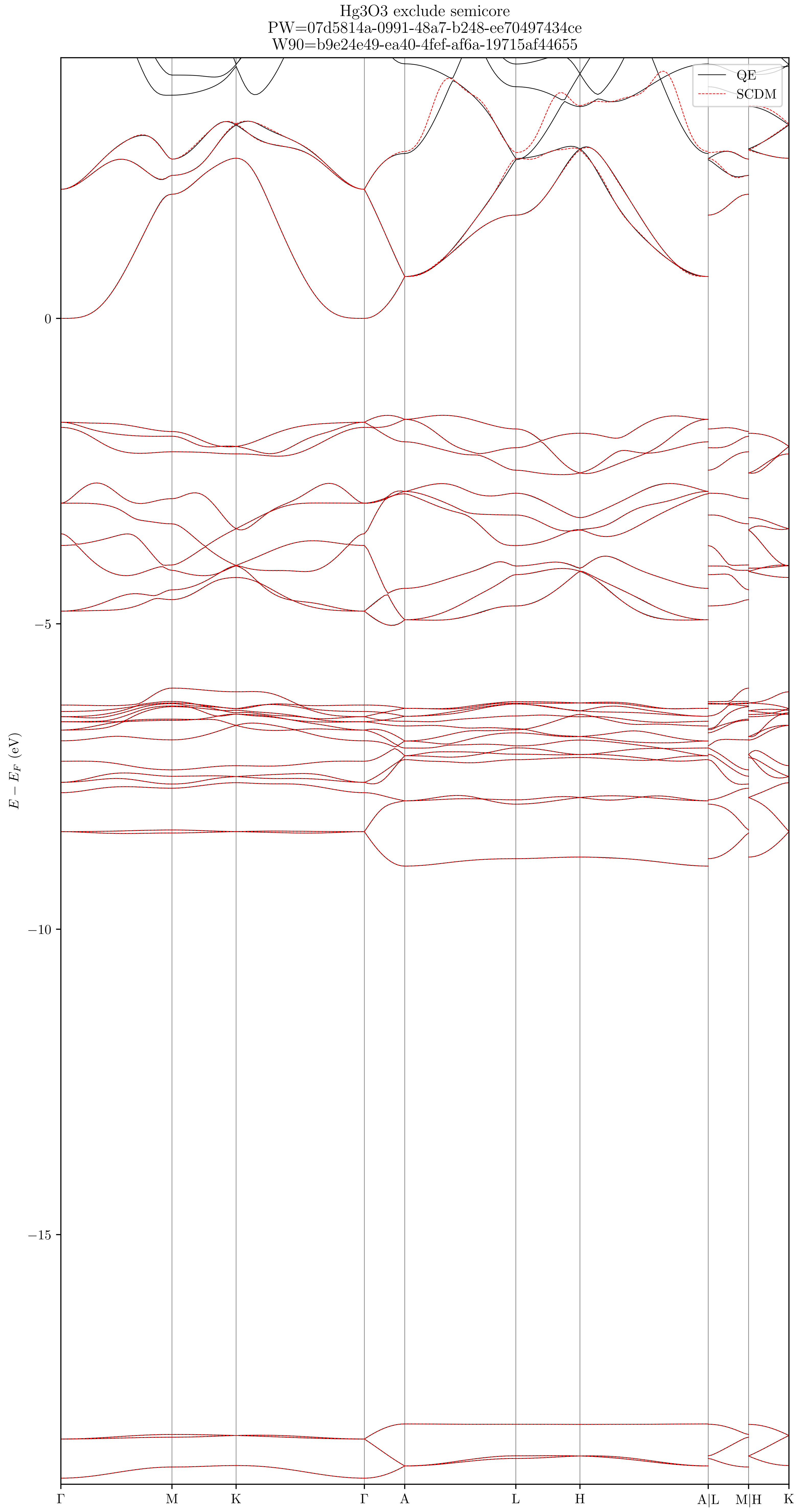

PDWF band distance (meV) and τ ($\AA$)

η_0	η_2	η_4	$\eta_0^{\max}$	$\eta_2^{\max}$	$\eta_4^{\max}$	τ
0.3	0.6	56.6	2.3	12.4	556.3	2.293

SCDM band distance (meV) and τ ($\AA$)

η_0	η_2	η_4	$\eta_0^{\max}$	$\eta_2^{\max}$	$\eta_4^{\max}$	τ
0.9	1.1	7.2	7.3	16.3	125.3	2.599

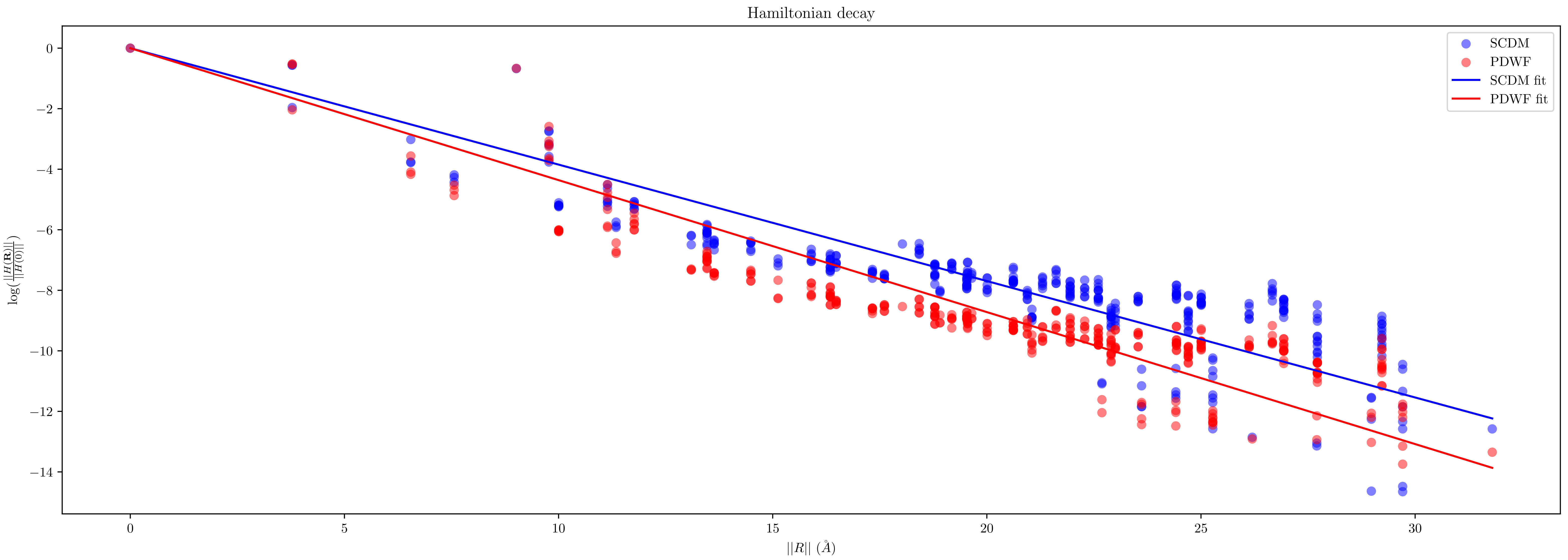

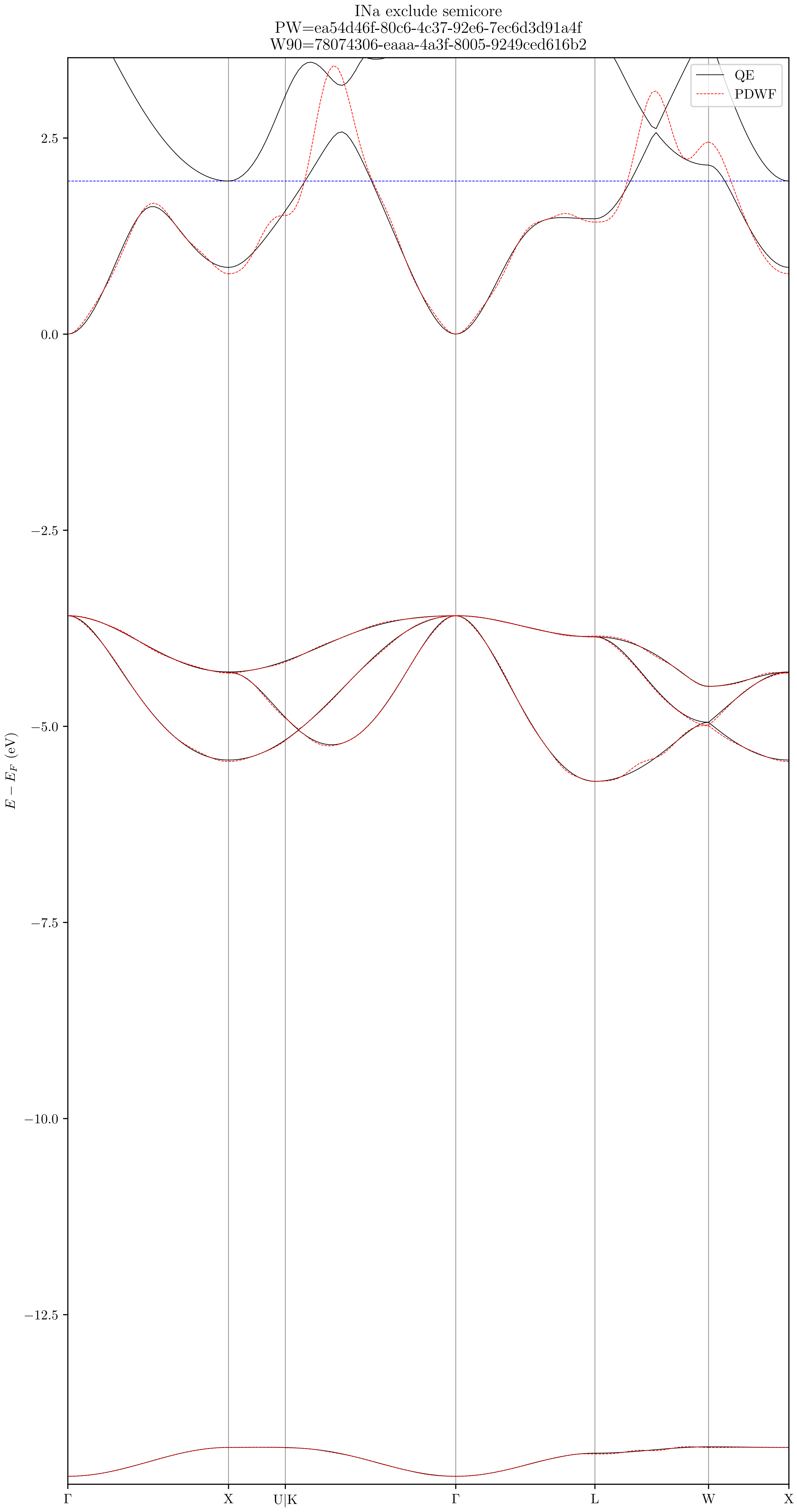

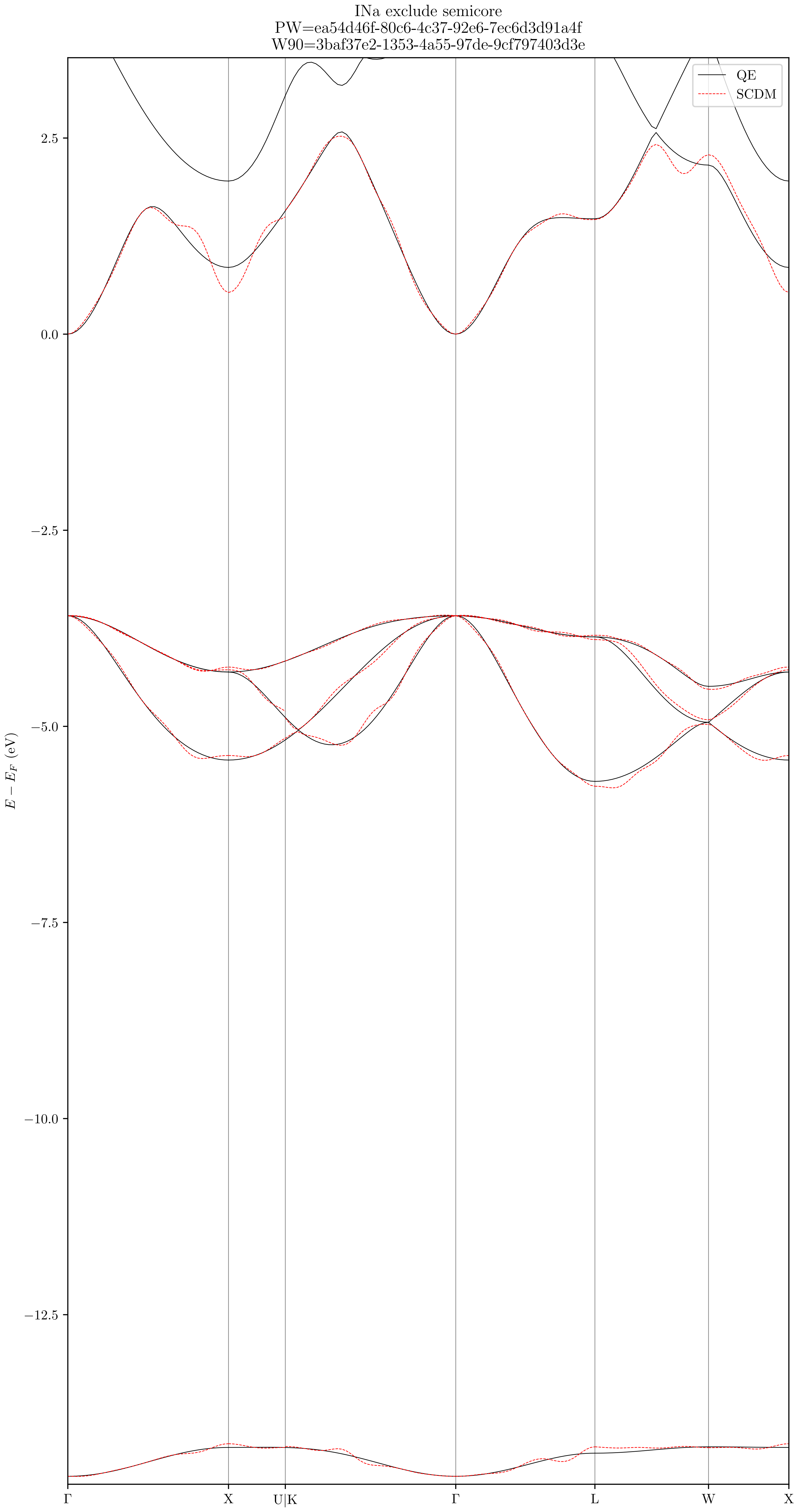

PDWF band distance (meV) and τ ($\AA$)

η_0	η_2	η_4	$\eta_0^{\max}$	$\eta_2^{\max}$	$\eta_4^{\max}$	τ
9.8	9.8	15.3	61.1	61.1	71.9	2.833

SCDM band distance (meV) and τ ($\AA$)

η_0	η_2	η_4	$\eta_0^{\max}$	$\eta_2^{\max}$	$\eta_4^{\max}$	τ
31.5	31.5	44.2	119.8	119.8	292.0	3.271

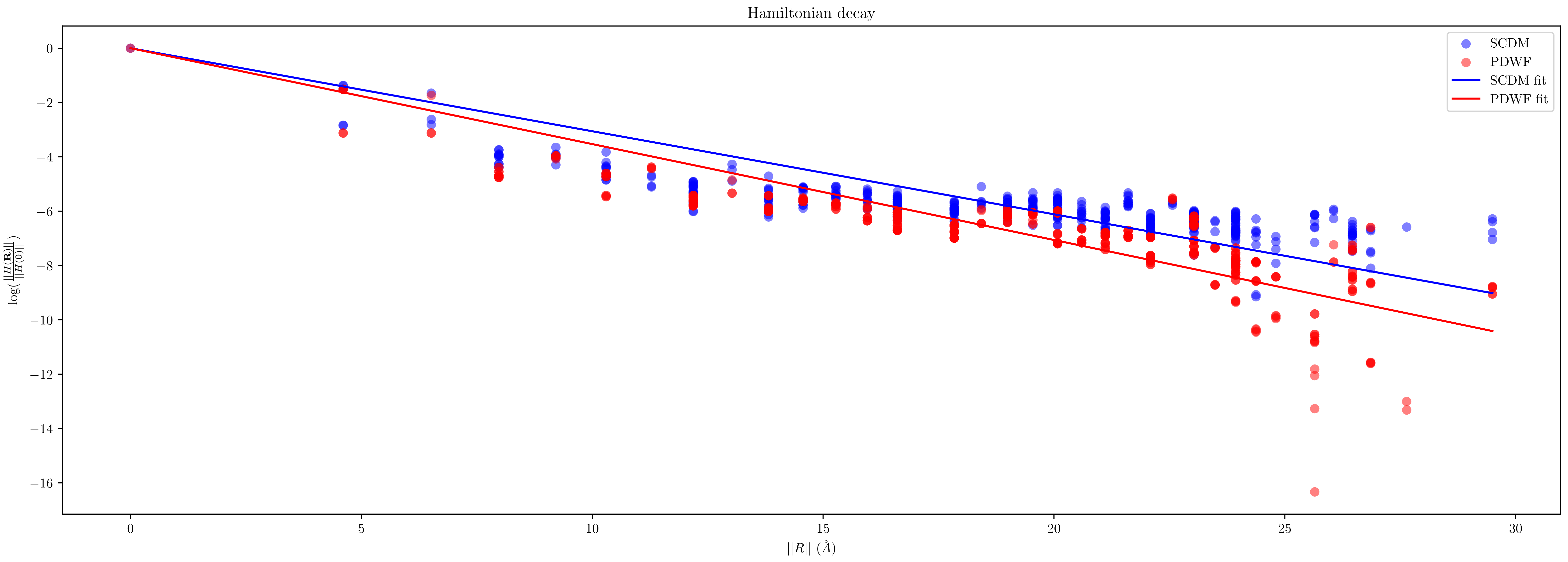

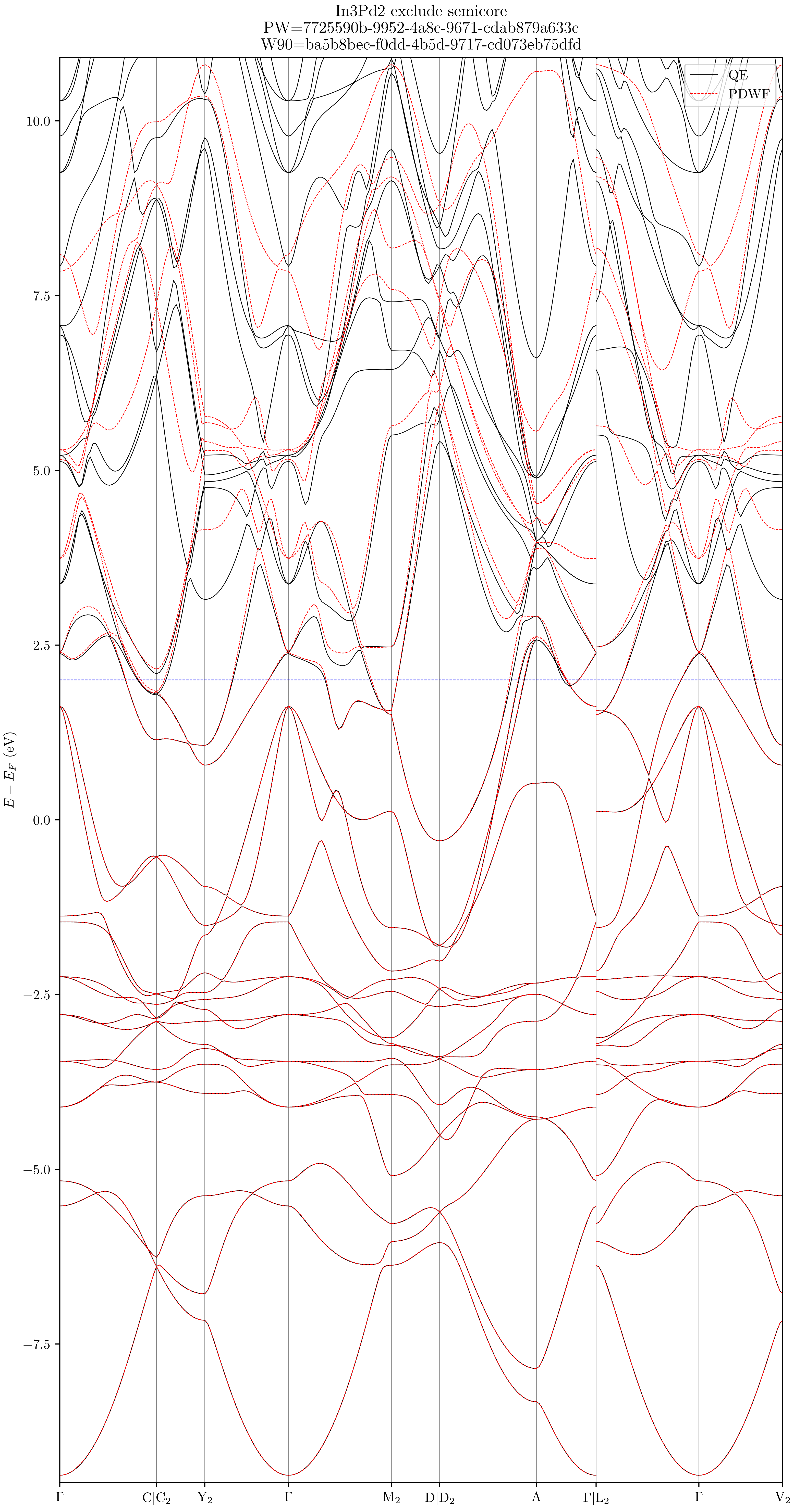

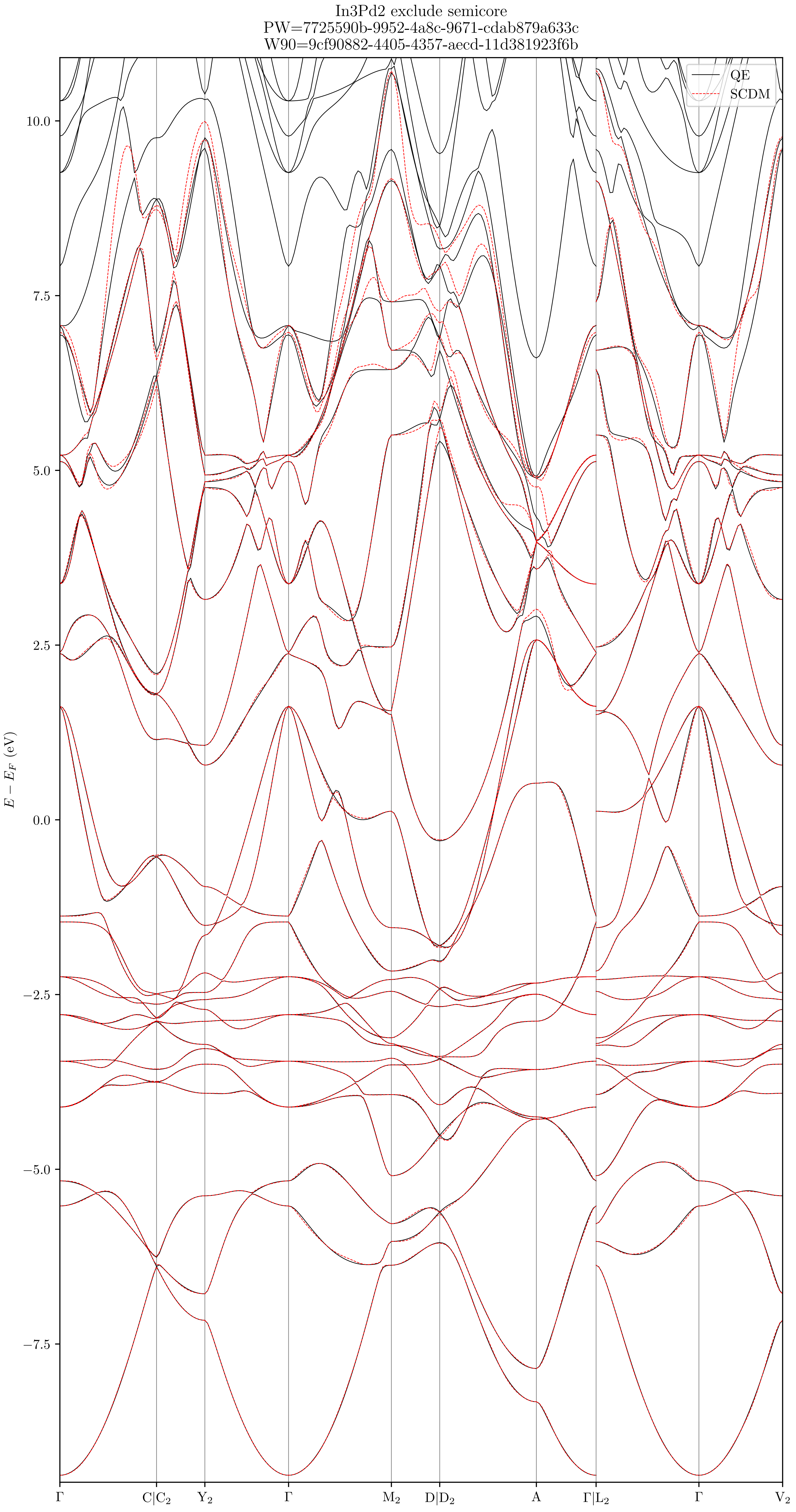

PDWF band distance (meV) and τ ($\AA$)

η_0	η_2	η_4	$\eta_0^{\max}$	$\eta_2^{\max}$	$\eta_4^{\max}$	τ
1.6	5.4	72.8	10.7	82.4	454.0	2.286

SCDM band distance (meV) and τ ($\AA$)

η_0	η_2	η_4	$\eta_0^{\max}$	$\eta_2^{\max}$	$\eta_4^{\max}$	τ
7.3	8.7	12.5	62.4	75.6	131.7	2.788

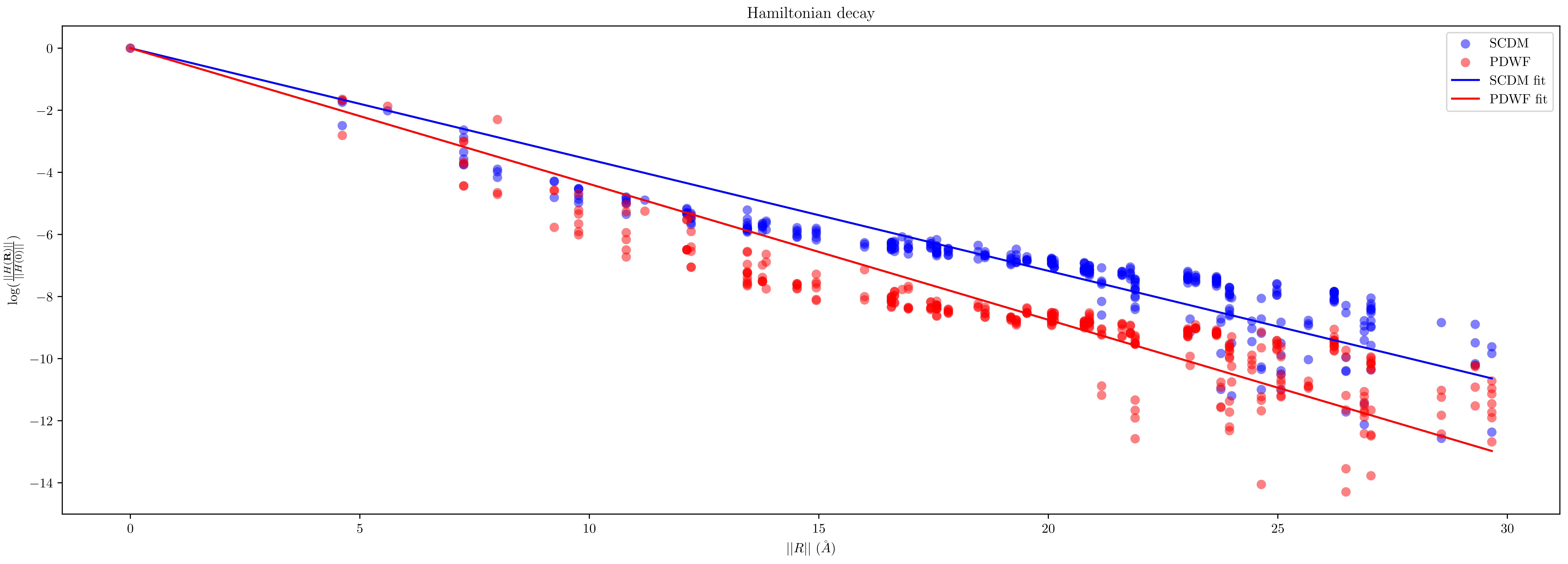

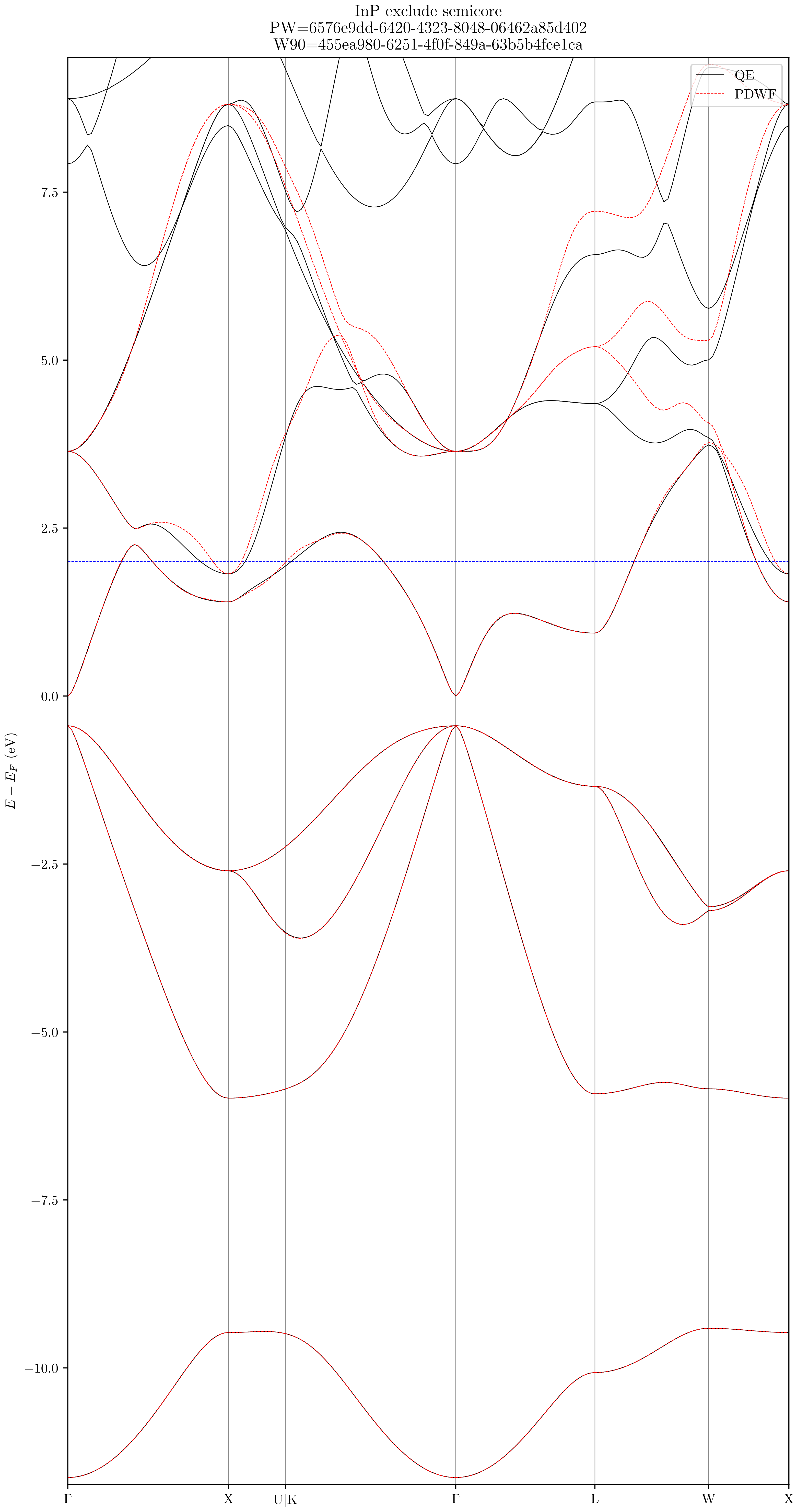

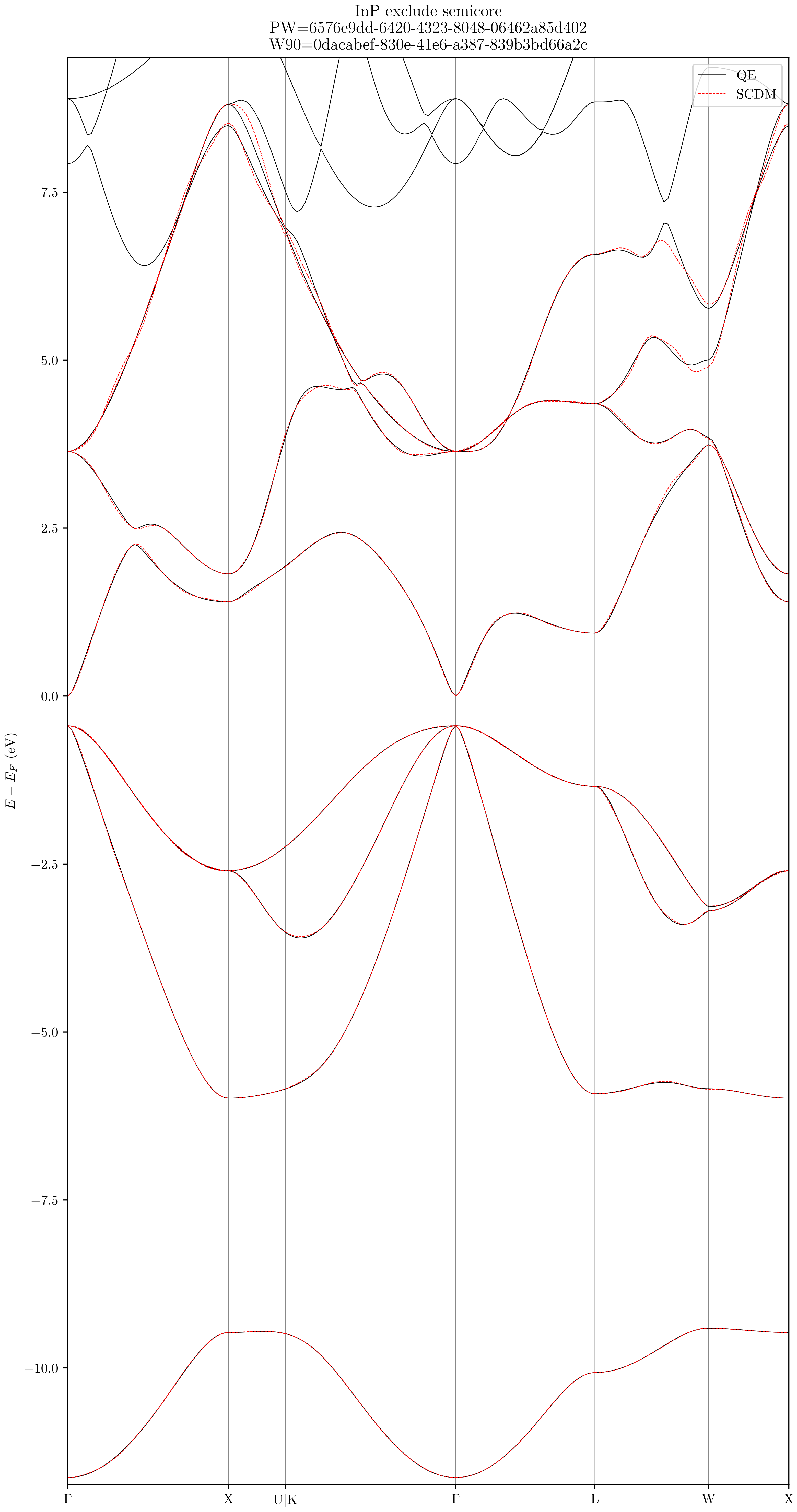

PDWF band distance (meV) and τ ($\AA$)

η_0	η_2	η_4	$\eta_0^{\max}$	$\eta_2^{\max}$	$\eta_4^{\max}$	τ
2.0	9.5	51.3	10.1	47.8	377.1	2.527

SCDM band distance (meV) and τ ($\AA$)

η_0	η_2	η_4	$\eta_0^{\max}$	$\eta_2^{\max}$	$\eta_4^{\max}$	τ
6.7	8.0	11.2	33.5	33.6	78.7	2.832

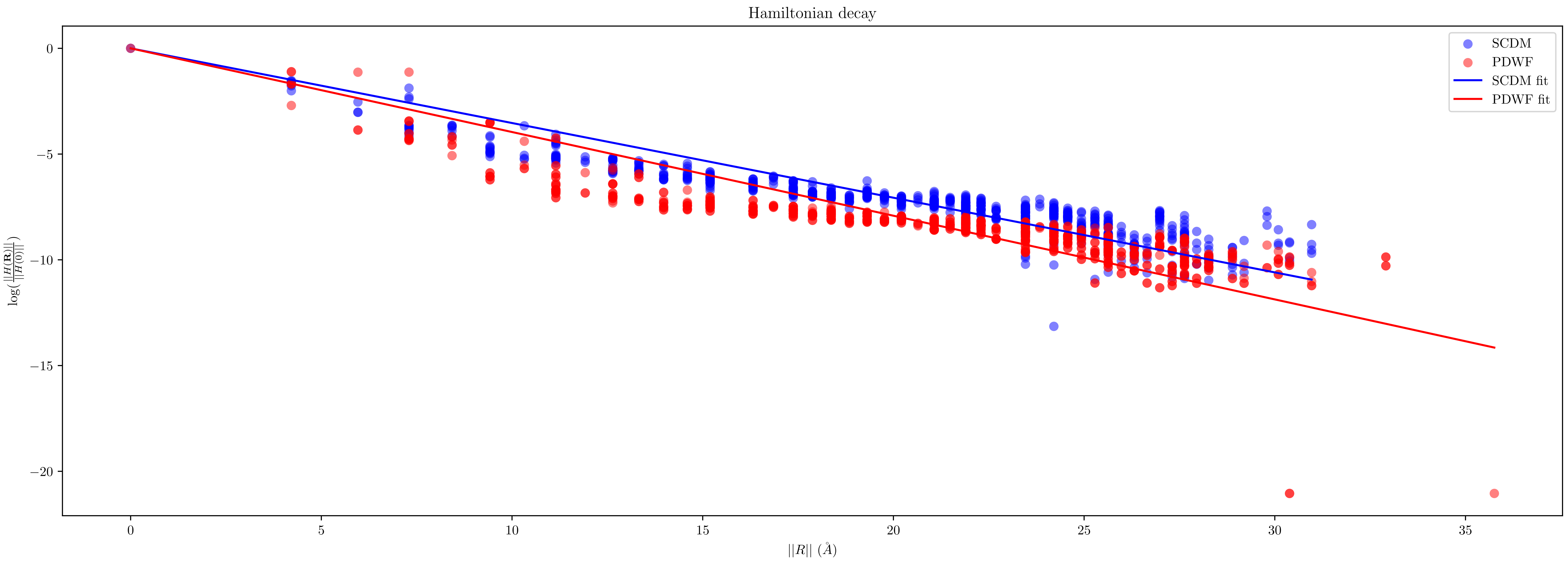

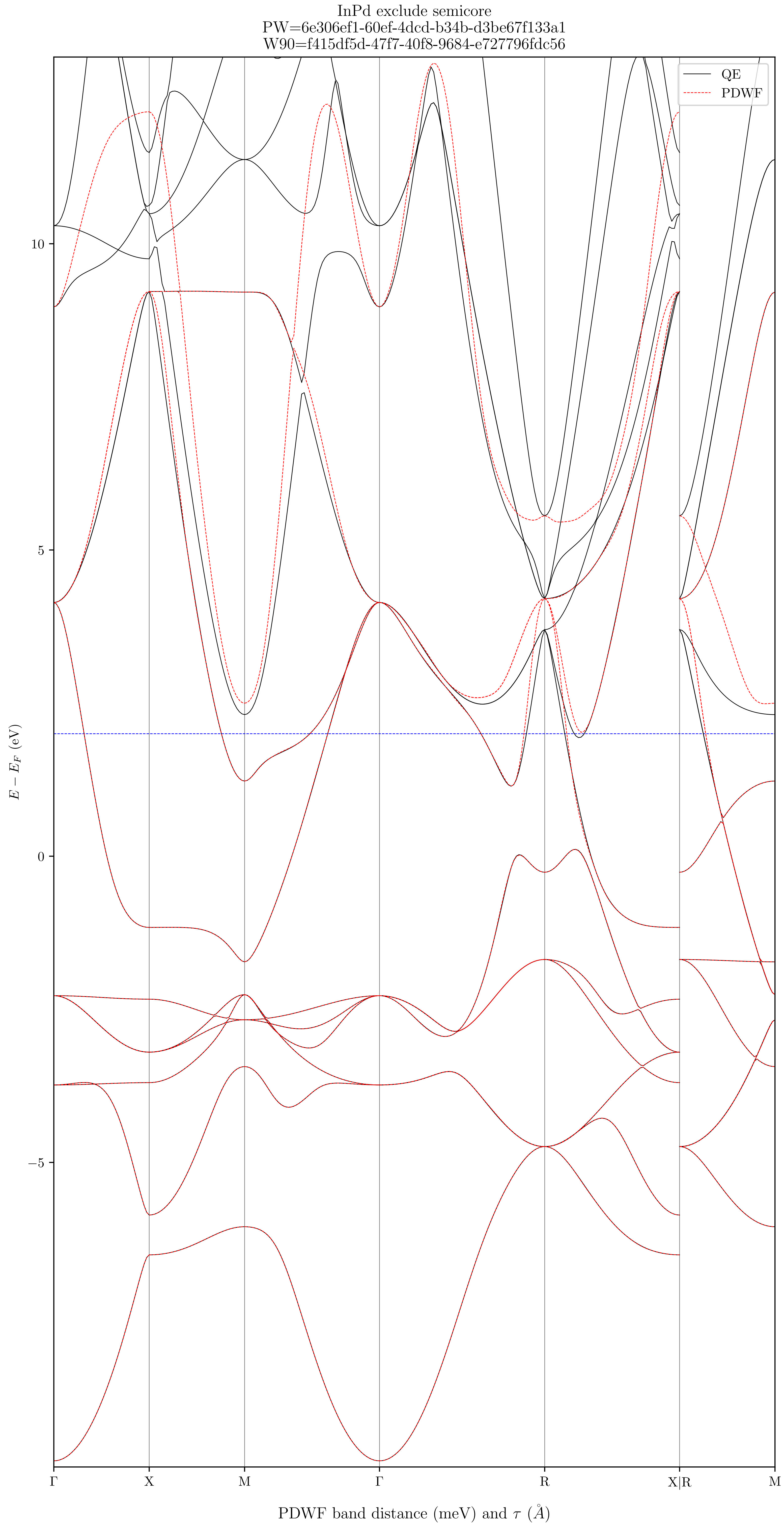

η_0	η_2	η_4	η_0^{max}	η_2^{max}	η_4^{max}	τ
2.3	12.5	122.6	31.1	145.7	1104.5	0.927

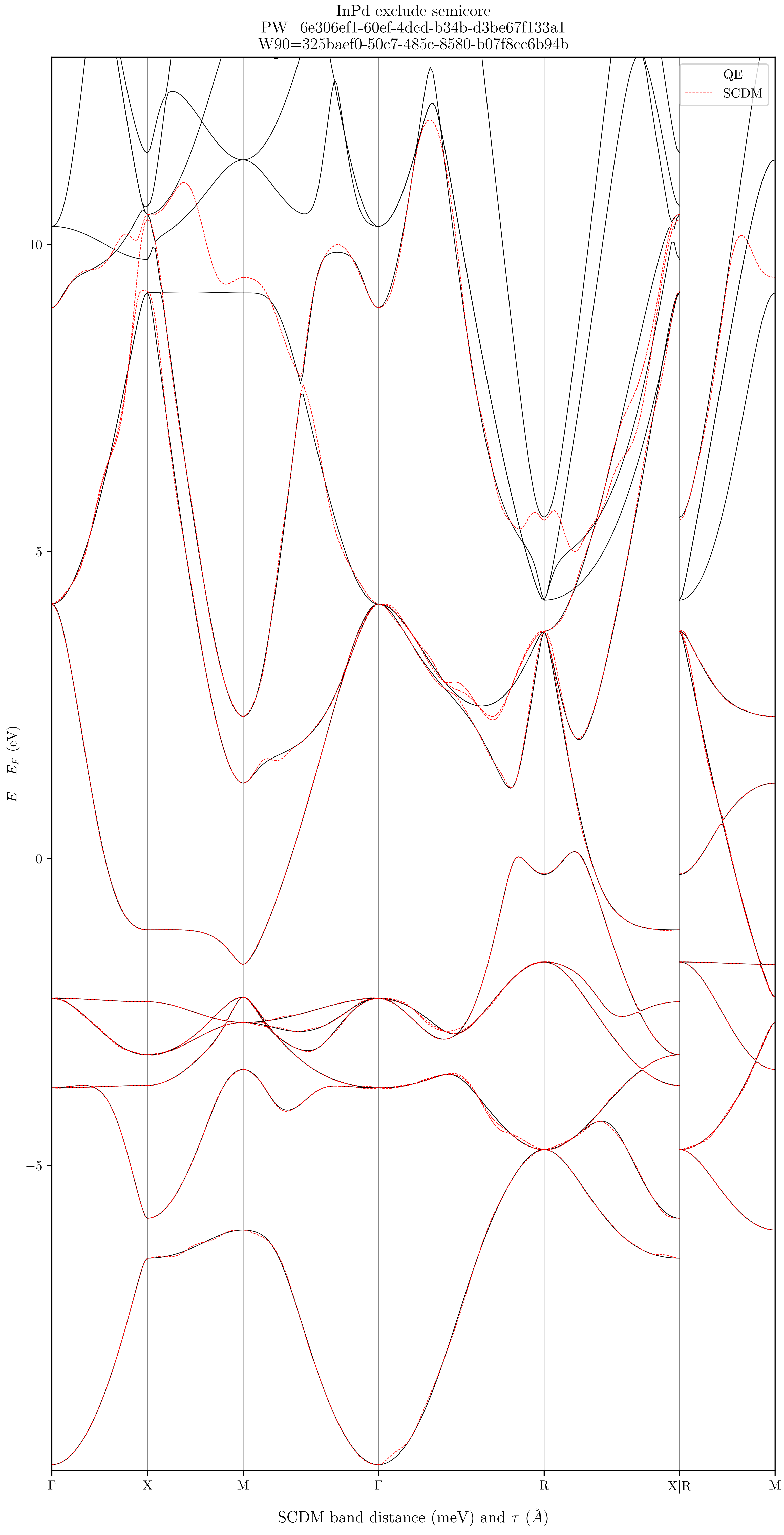

η_0	η_2	η_4	η_0^{max}	η_2^{max}	η_4^{max}	τ
15.6	17.6	34.3	130.0	130.0	278.0	2.539

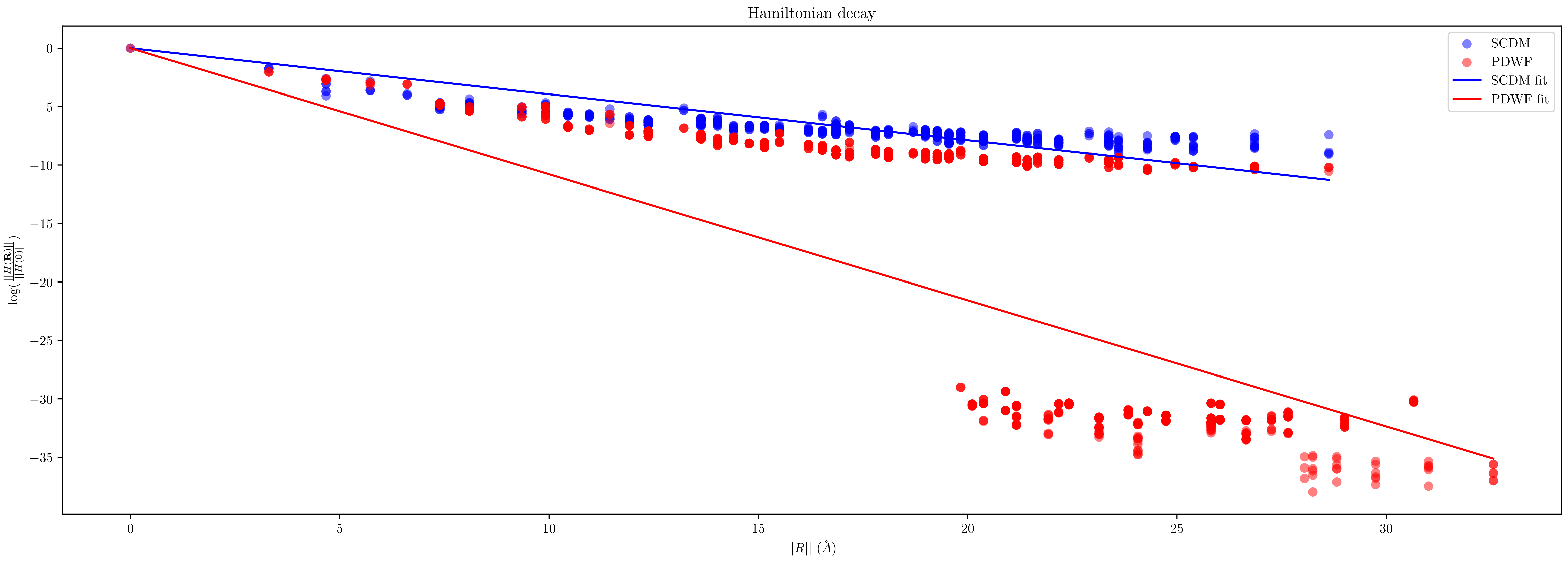

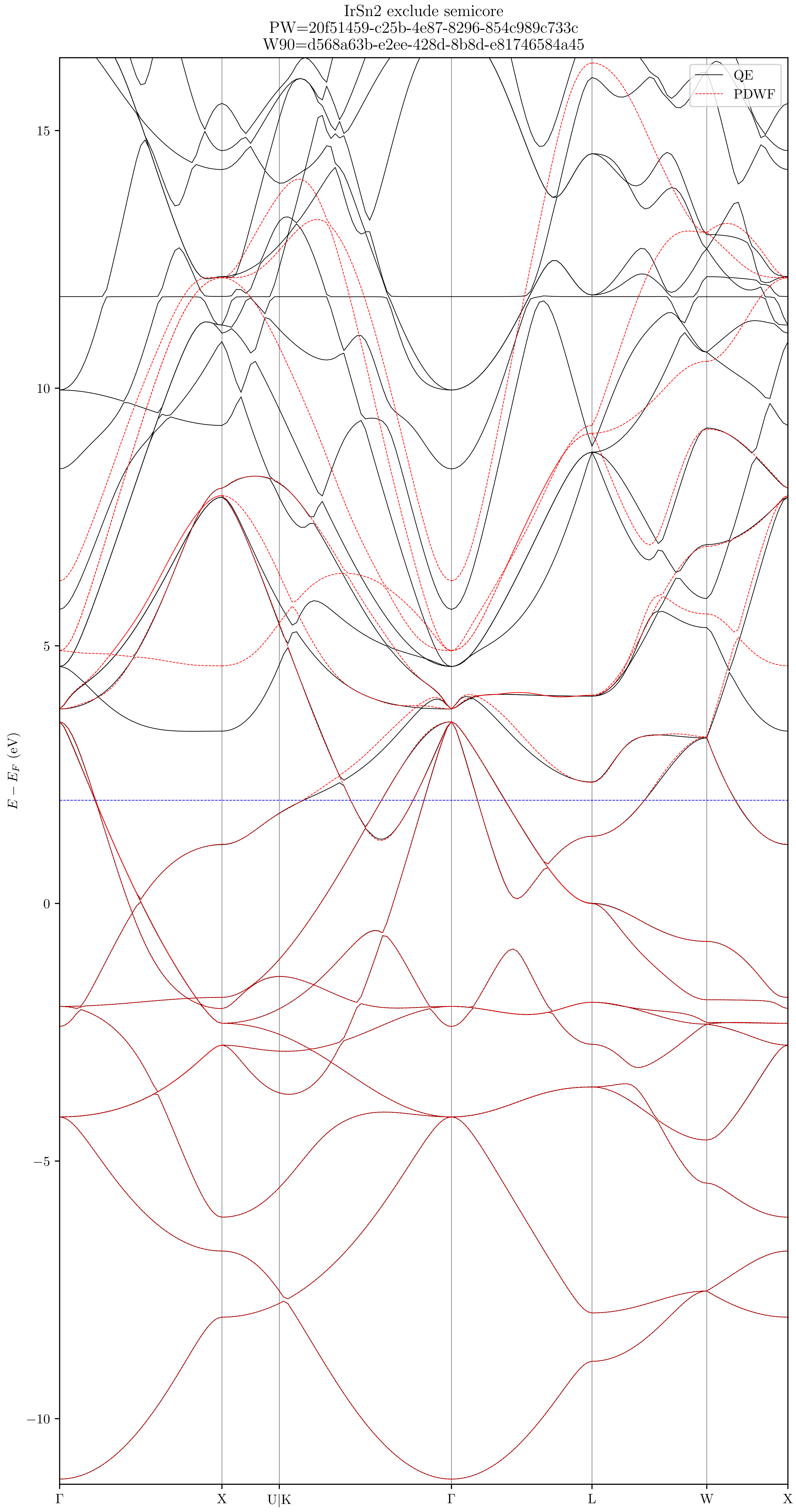

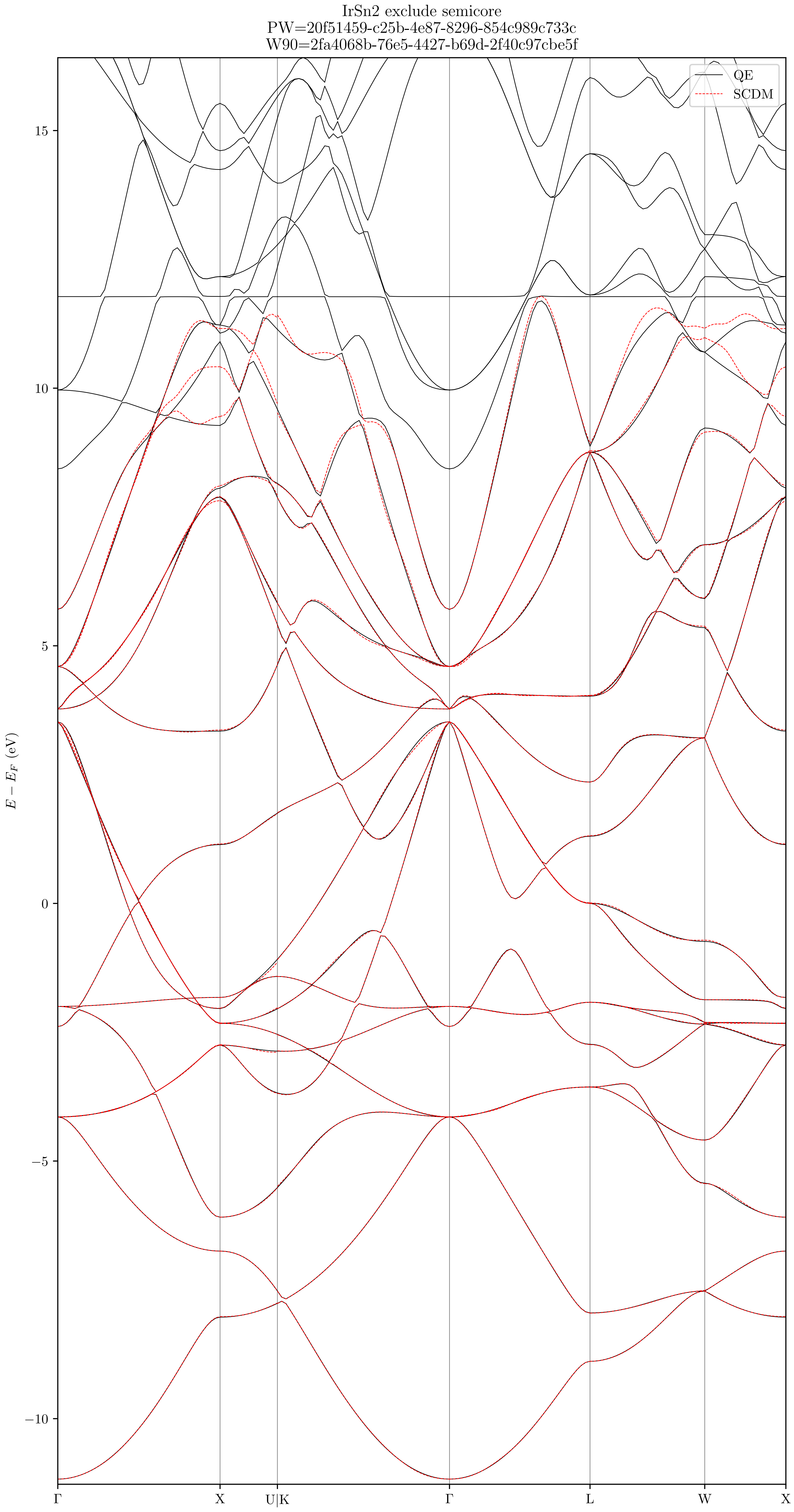

PDWF band distance (meV) and τ ($\AA$)

η_0	η_2	η_4	$\eta_0^{\max}$	$\eta_2^{\max}$	$\eta_4^{\max}$	τ
1.6	3.1	45.3	9.0	25.7	229.6	2.360

SCDM band distance (meV) and τ ($\AA$)

η_0	η_2	η_4	$\eta_0^{\max}$	$\eta_2^{\max}$	$\eta_4^{\max}$	τ
7.0	7.7	9.5	58.9	58.9	65.0	2.761

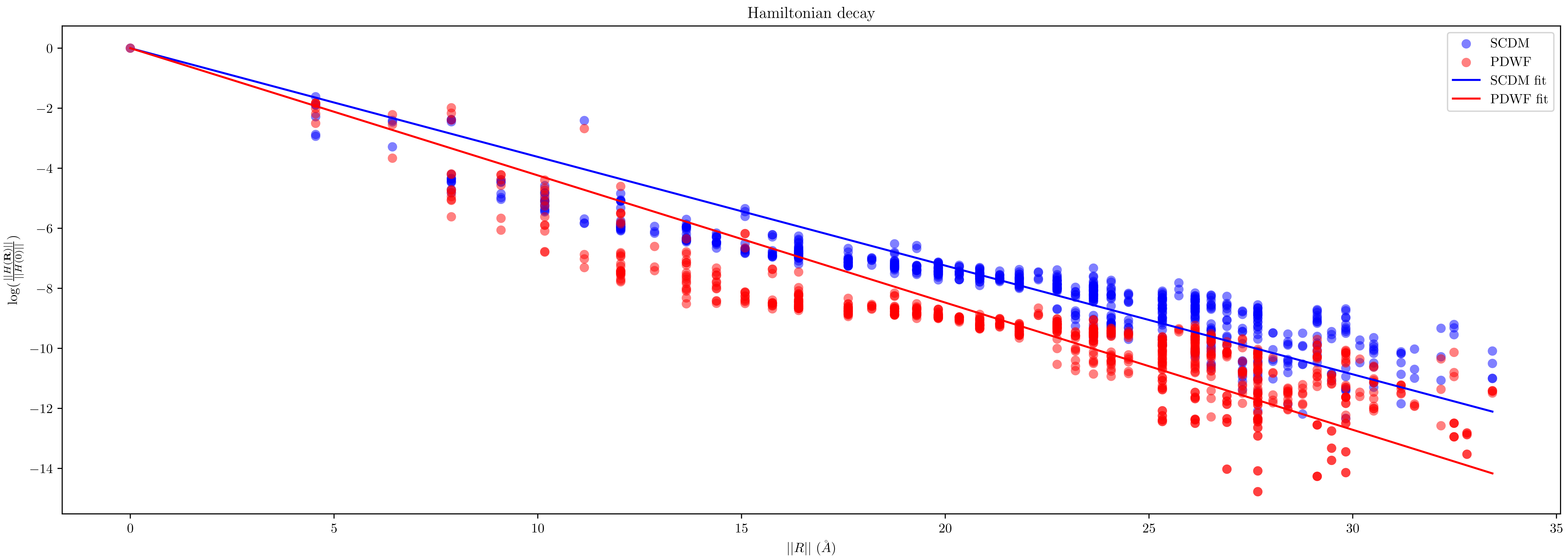

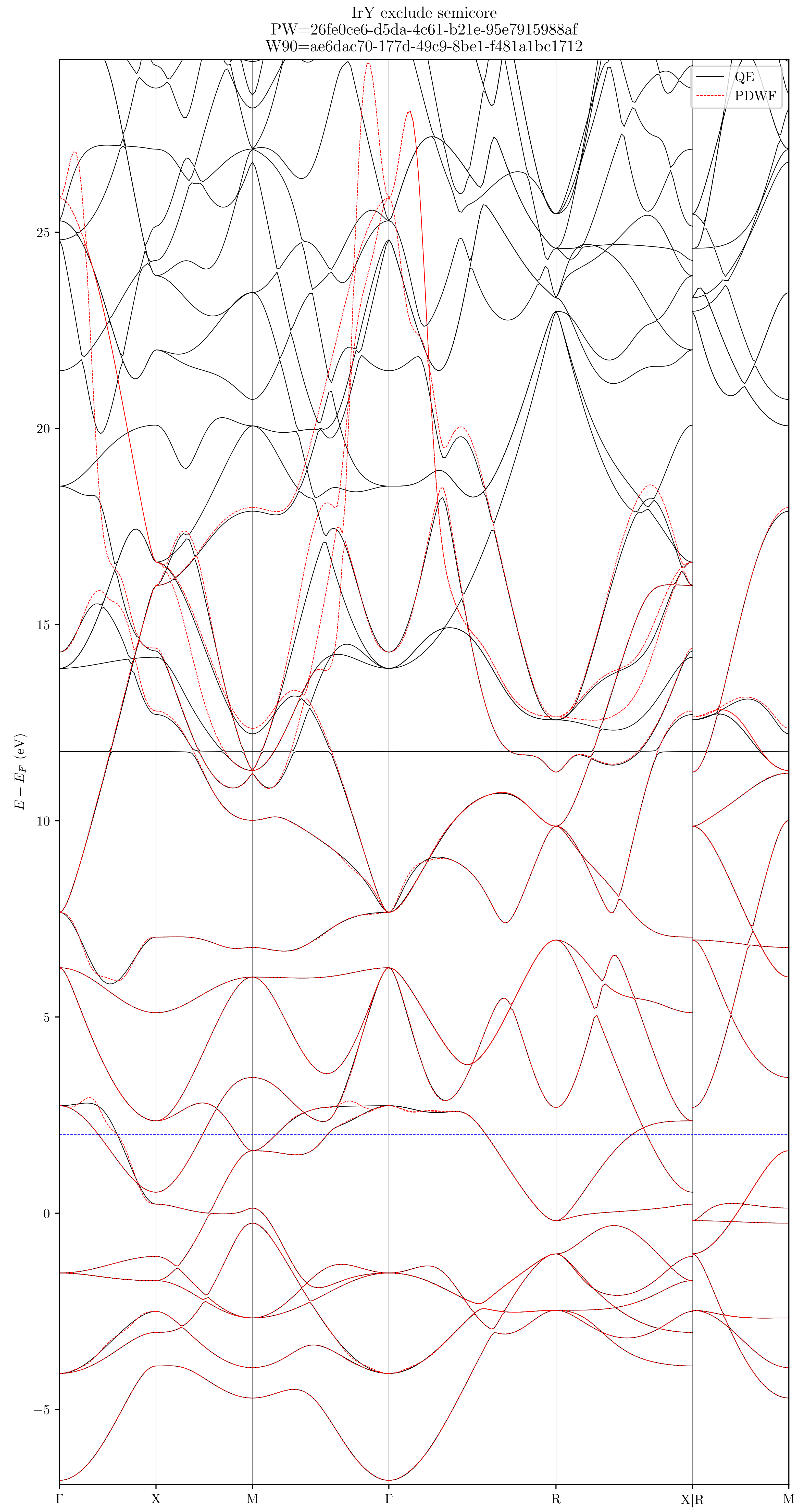

PDWF band distance (meV) and τ ($\AA$)

η_0	η_2	η_4	$\eta_0^{\max}$	$\eta_2^{\max}$	$\eta_4^{\max}$	τ
5.7	7.9	15.4	50.8	111.8	211.8	2.321

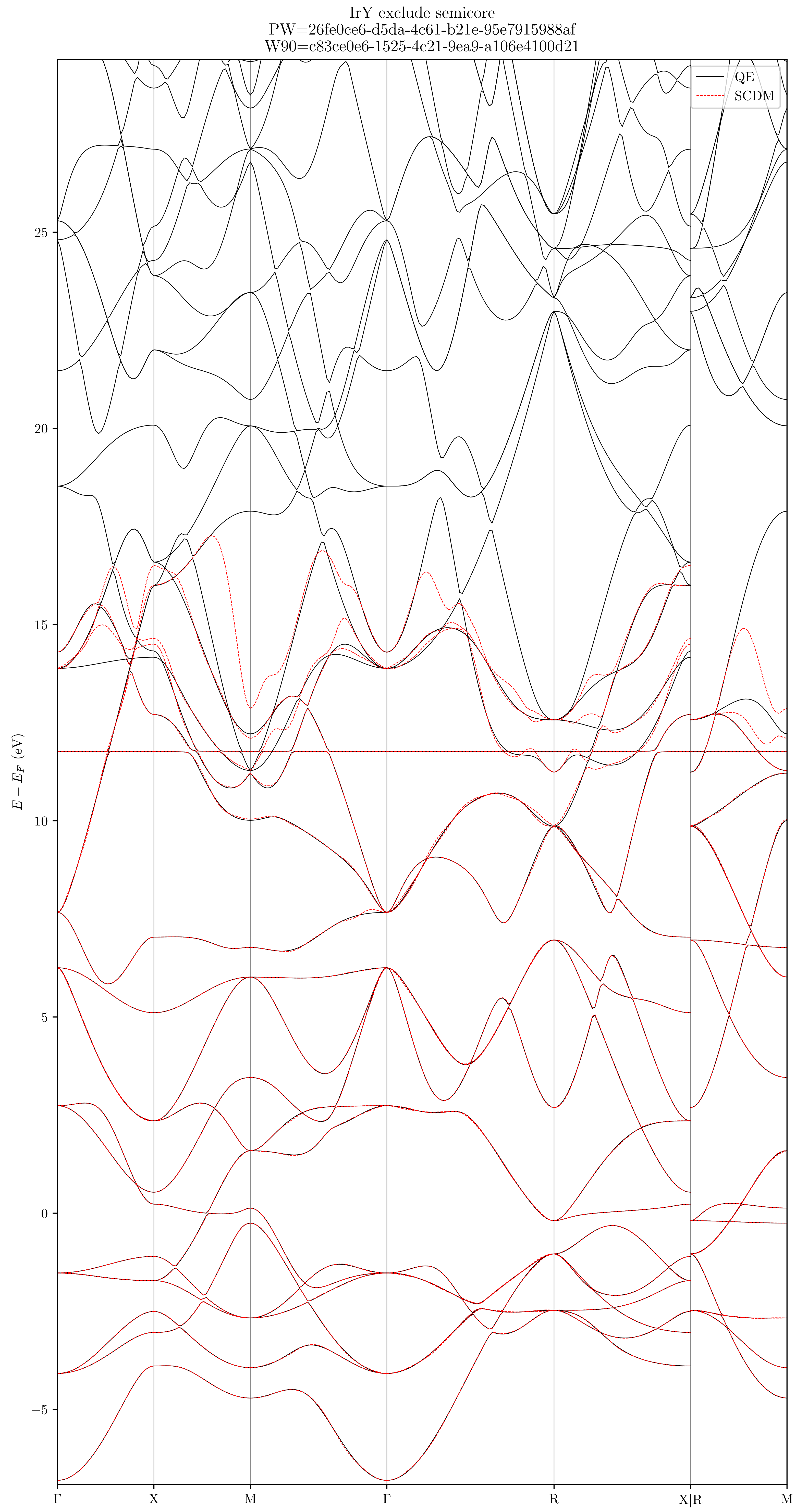

SCDM band distance (meV) and τ ($\AA$)

η_0	η_2	η_4	$\eta_0^{\max}$	$\eta_2^{\max}$	$\eta_4^{\max}$	τ
6.7	6.7	7.3	41.0	41.0	41.0	2.439

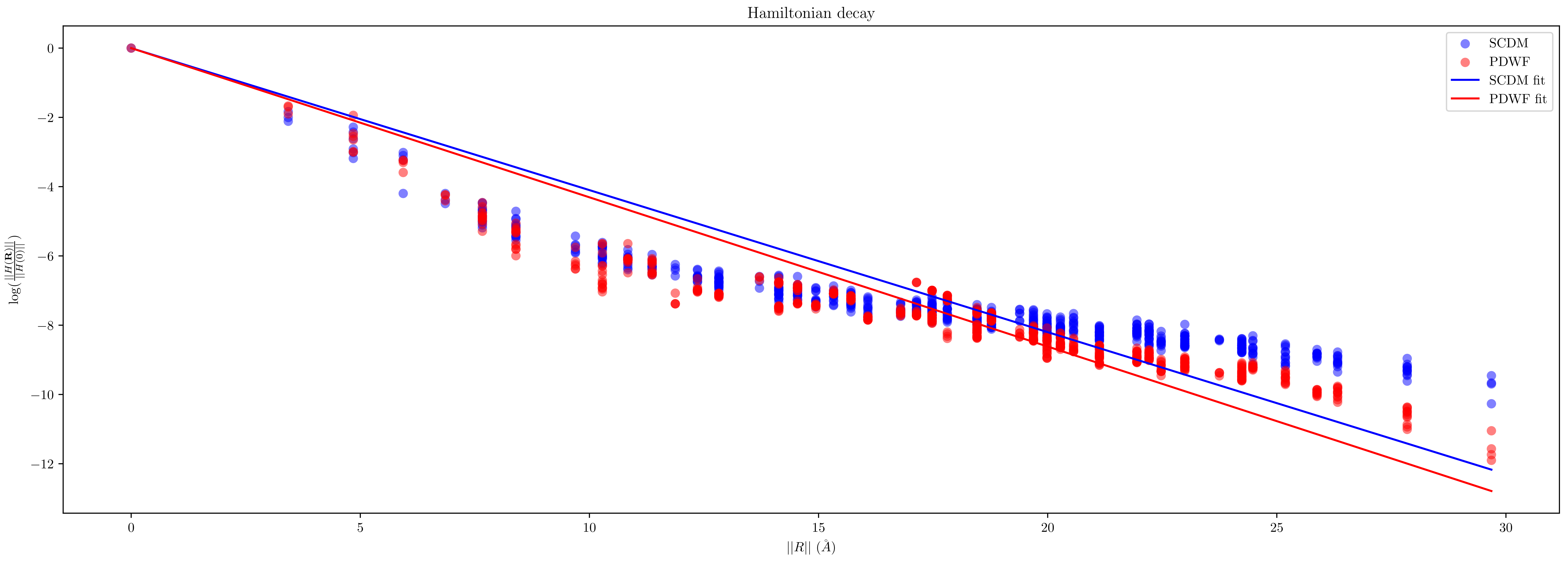

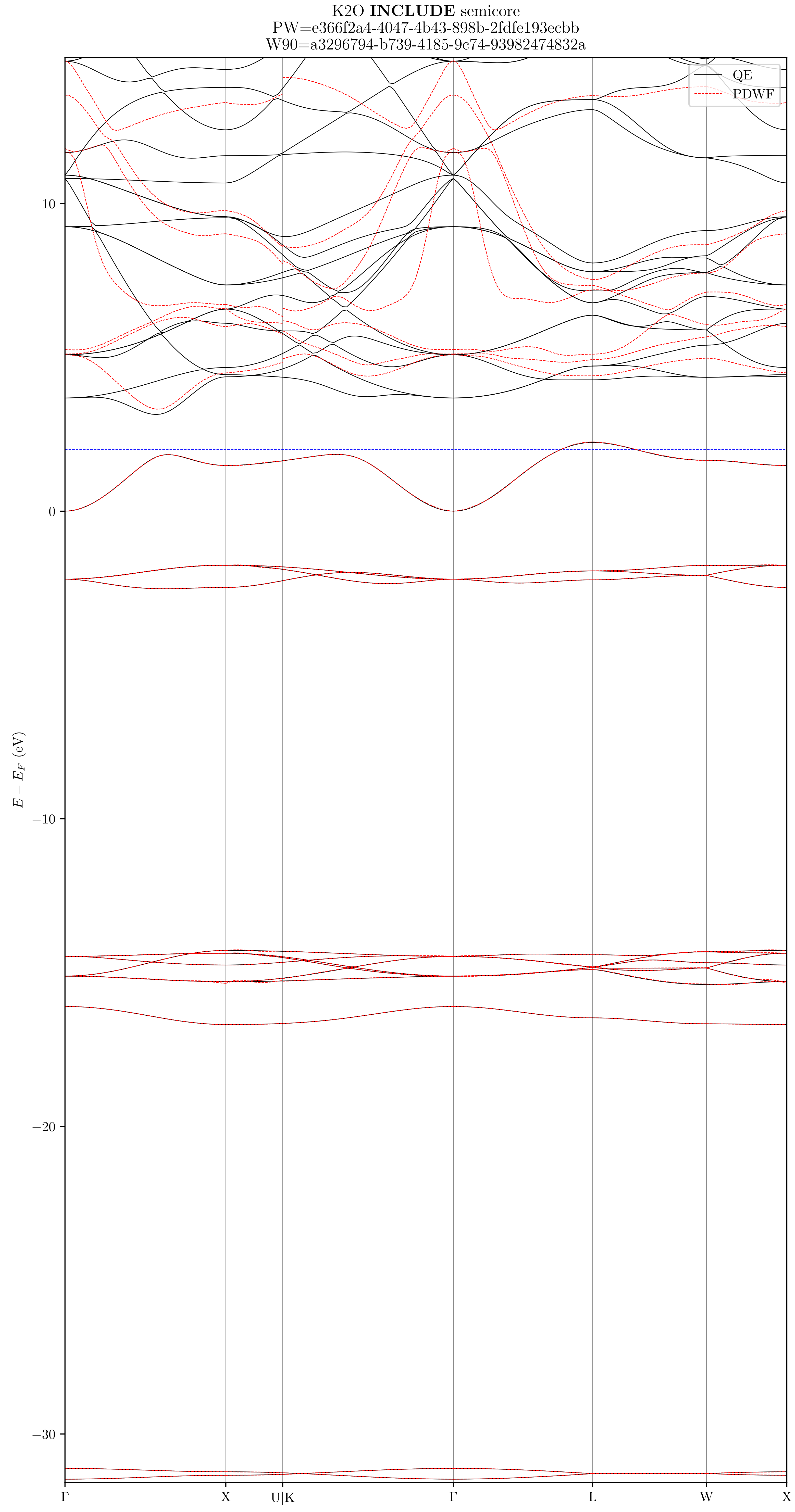

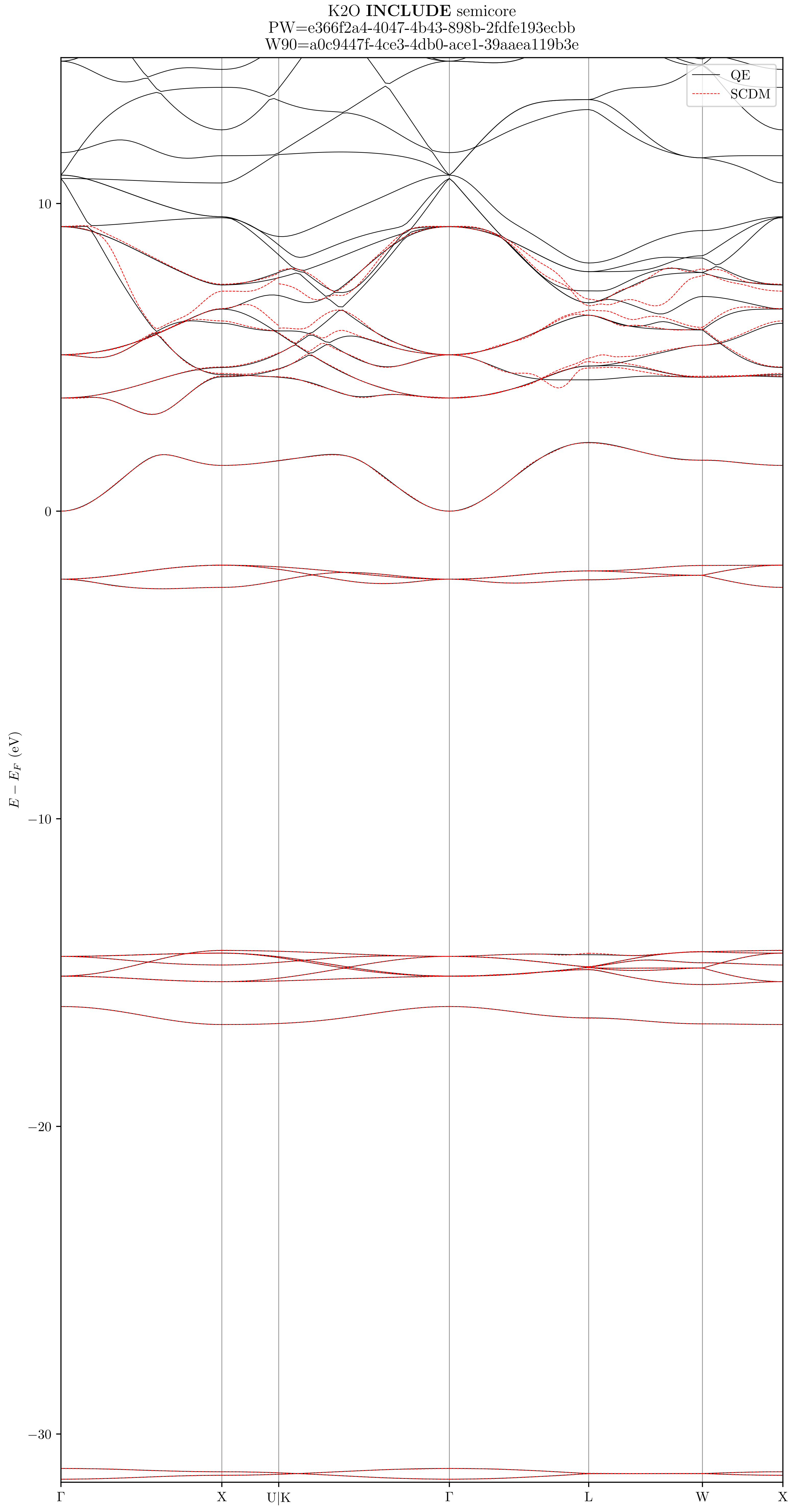

PDWF band distance (meV) and τ ($\AA$)

η_0	η_2	η_4	$\eta_0^{\max}$	$\eta_2^{\max}$	$\eta_4^{\max}$	τ
5.4	5.5	14.9	54.1	54.1	176.8	2.415

SCDM band distance (meV) and τ ($\AA$)

η_0	η_2	η_4	$\eta_0^{\max}$	$\eta_2^{\max}$	$\eta_4^{\max}$	τ
3.9	4.3	4.6	47.2	47.2	47.2	2.573

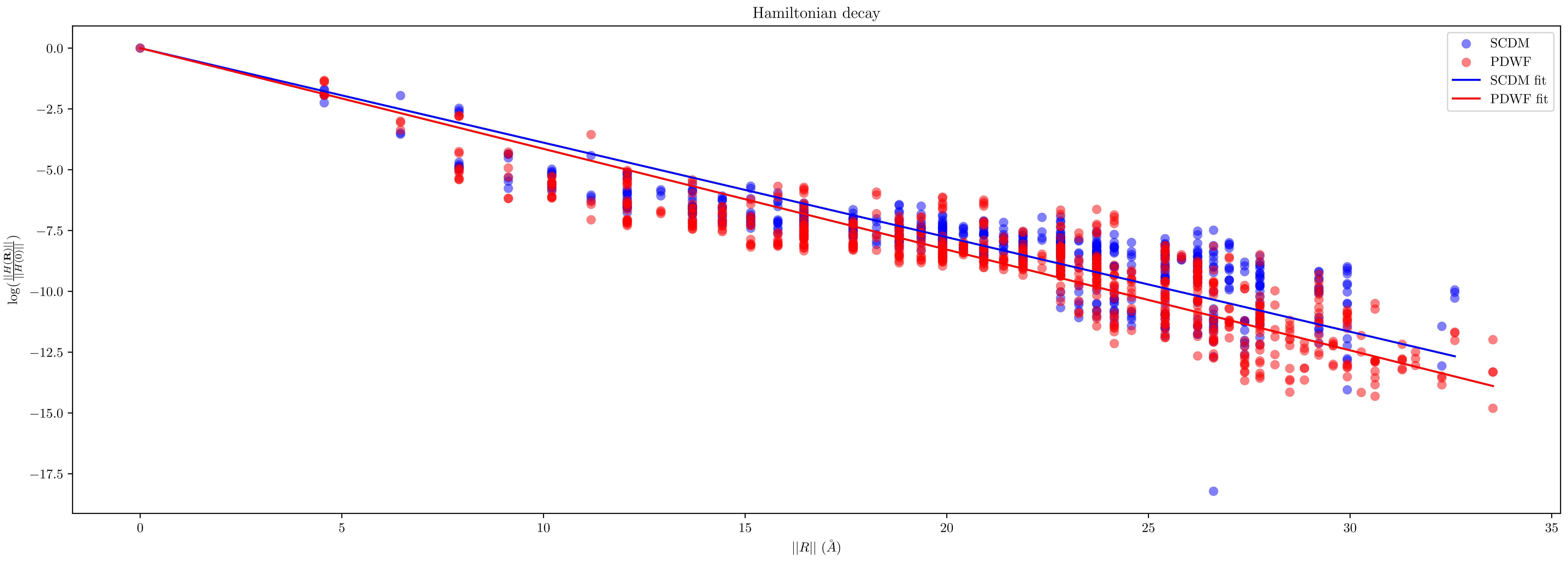

K2O2 **INCLUDE** semicore
PW=0be5eae4-e4c5-466e-bfc9-40a8fe932b36
W90=2bb89531-7ae1-4250-9dfc-3afc398f34e0

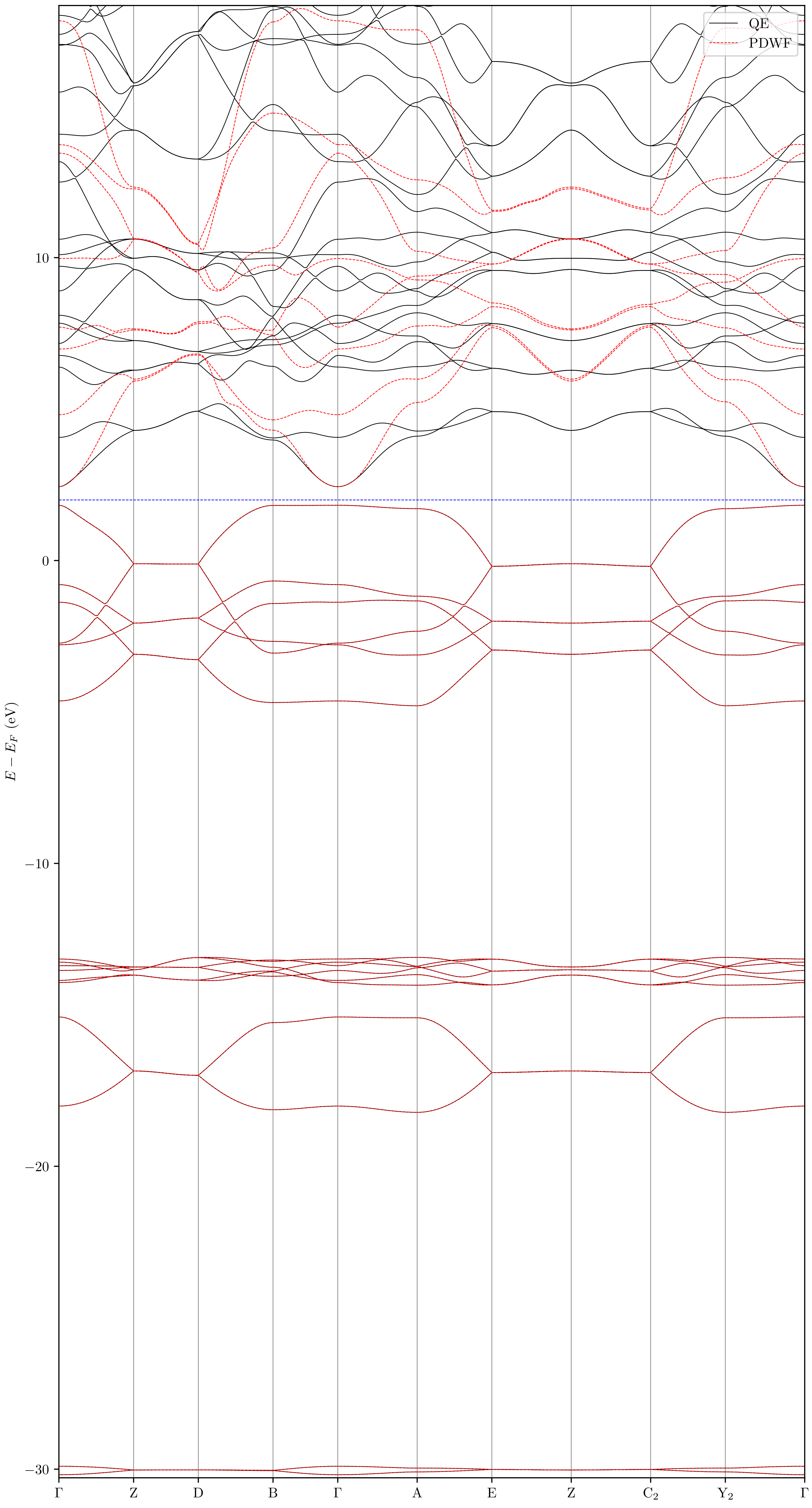

PDWF band distance (meV) and τ ($\AA$)

η_0	η_2	η_4	$\eta_0^{\max}$	$\eta_2^{\max}$	$\eta_4^{\max}$	τ
0.8	0.8	23.9	6.0	6.0	337.5	2.134

K2O2 **INCLUDE** semicore
PW=0be5eae4-e4c5-466e-bfc9-40a8fe932b36
W90=9feccd6a-7a7c-4078-802d-381ad7dbd99b

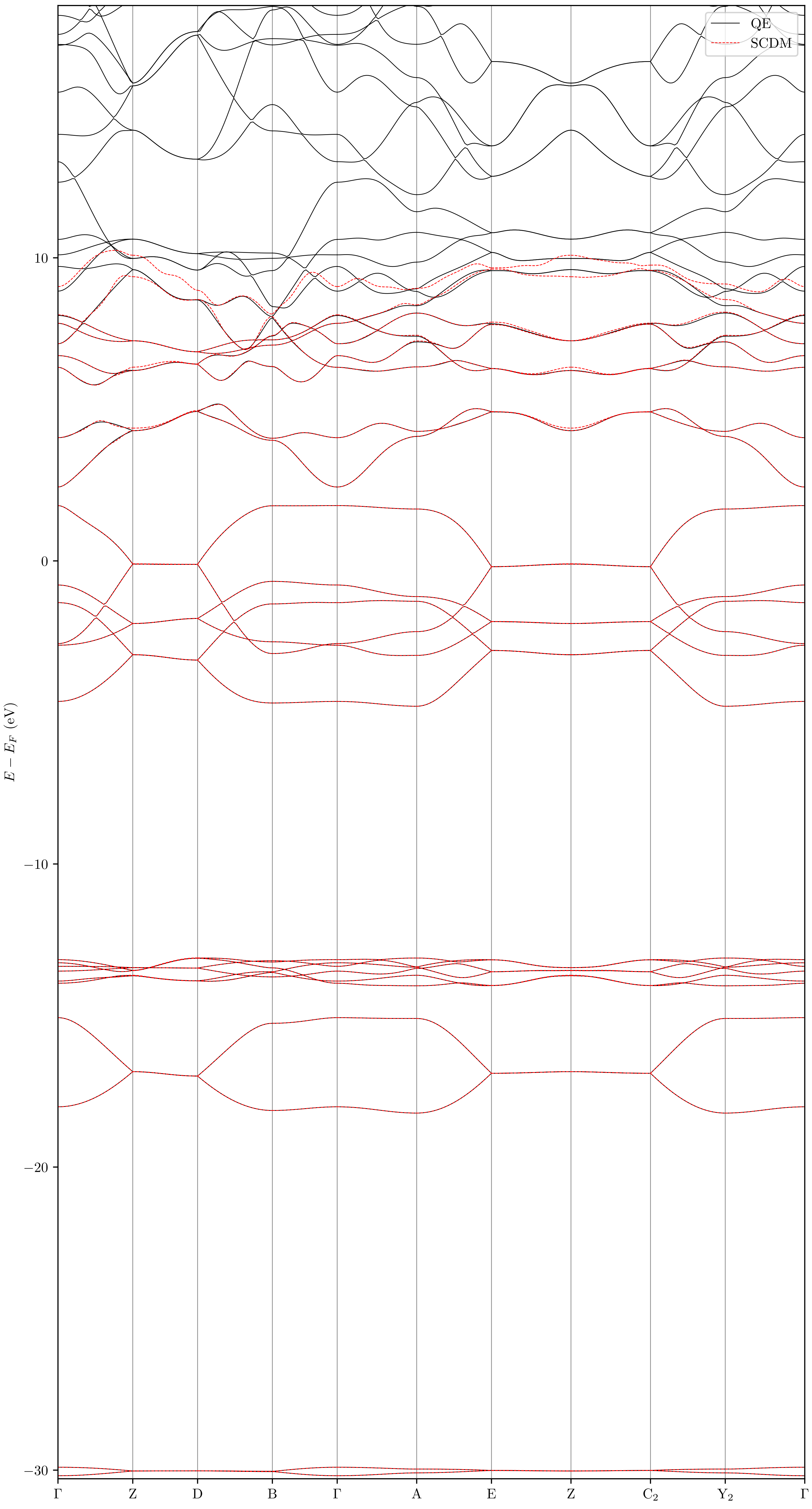

SCDM band distance (meV) and τ ($\AA$)

η_0	η_2	η_4	$\eta_0^{\max}$	$\eta_2^{\max}$	$\eta_4^{\max}$	τ
2.2	2.2	2.4	17.5	17.5	17.5	2.312

Hamiltonian decay

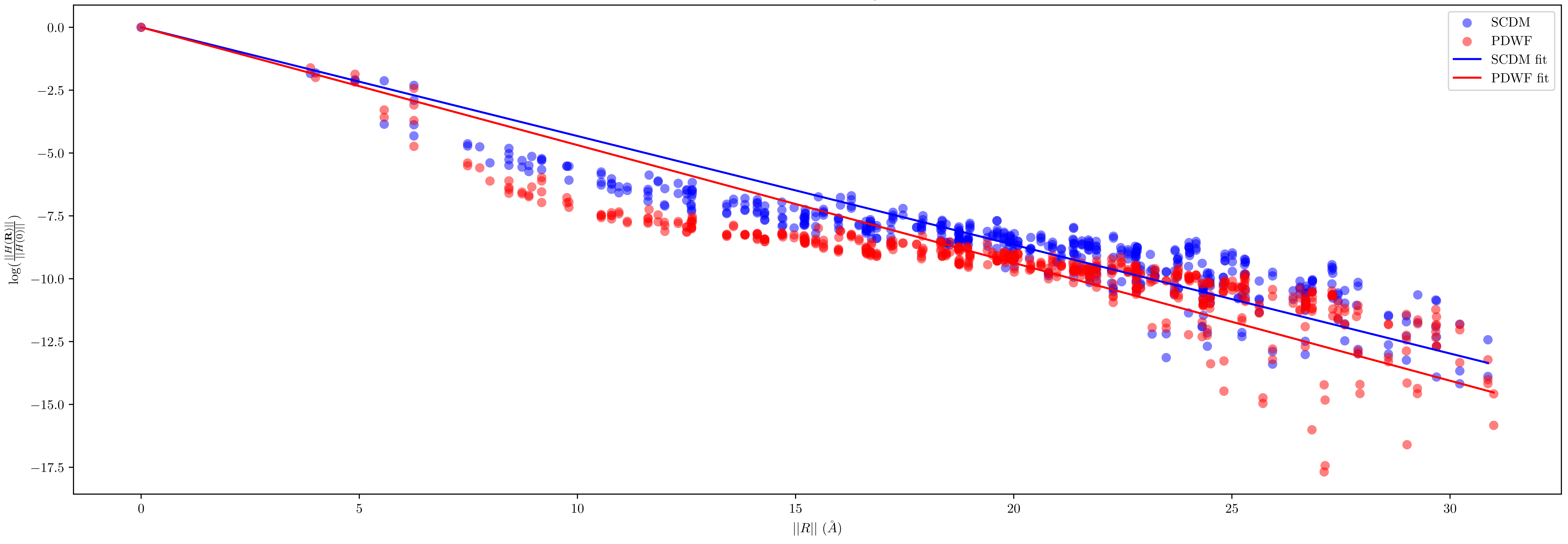

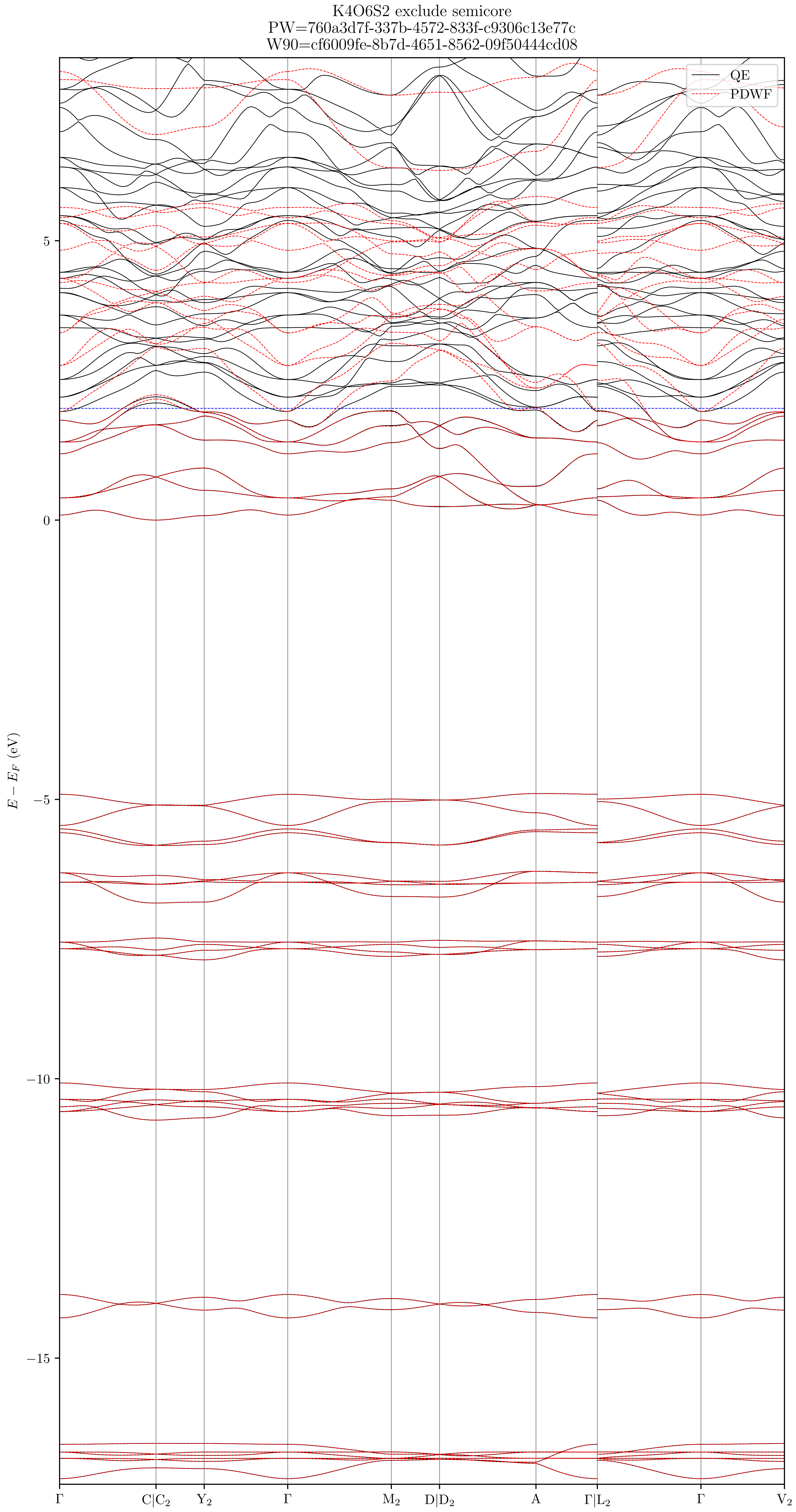

PDWF band distance (meV) and τ ($\AA$)

η_0	η_2	η_4	$\eta_0^{\max}$	$\eta_2^{\max}$	$\eta_4^{\max}$	τ
0.4	0.8	162.5	3.4	9.0	696.5	2.669

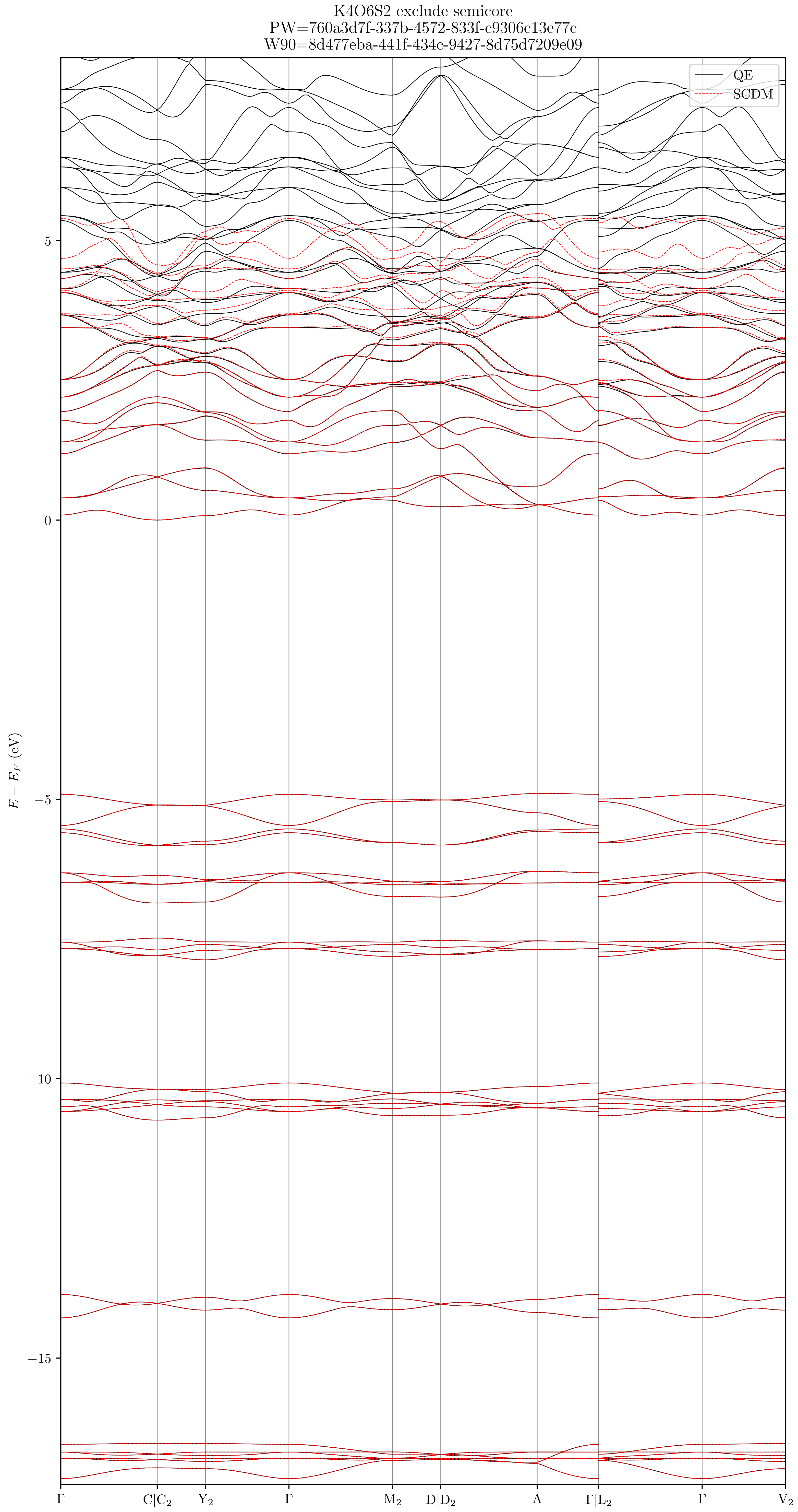

SCDM band distance (meV) and τ ($\AA$)

η_0	η_2	η_4	$\eta_0^{\max}$	$\eta_2^{\max}$	$\eta_4^{\max}$	τ
0.4	0.9	5.8	4.9	12.4	60.7	3.000

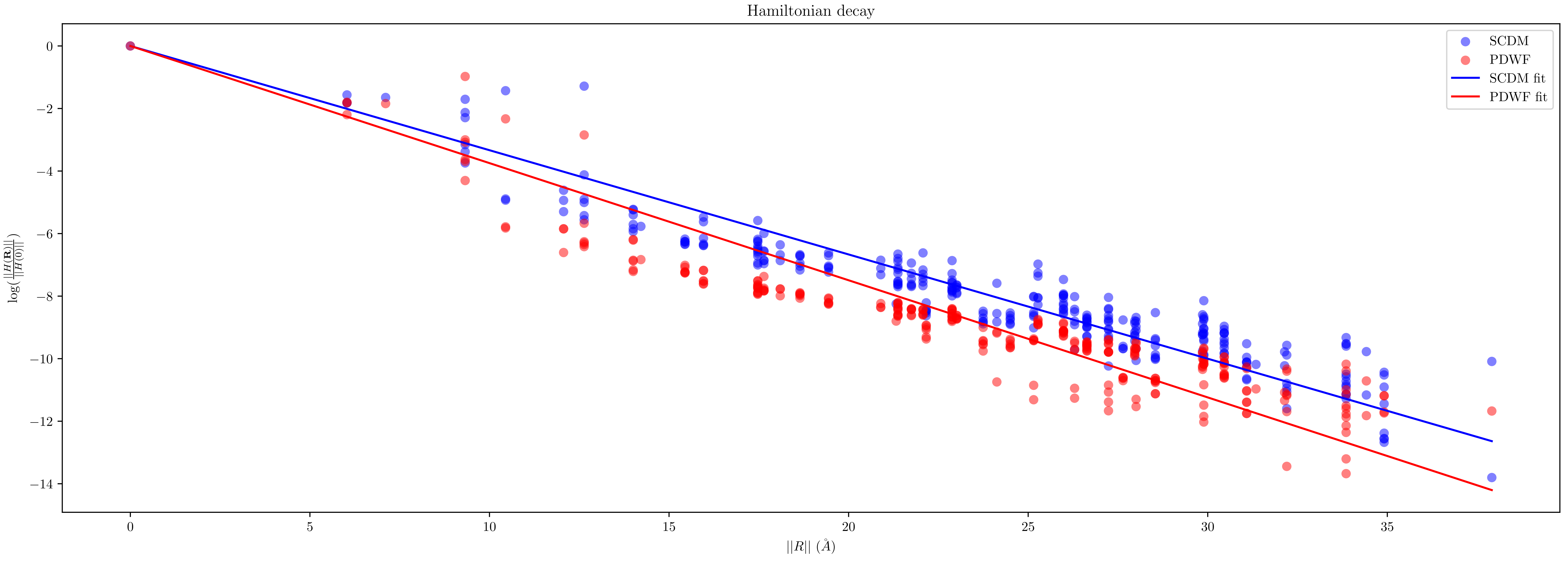

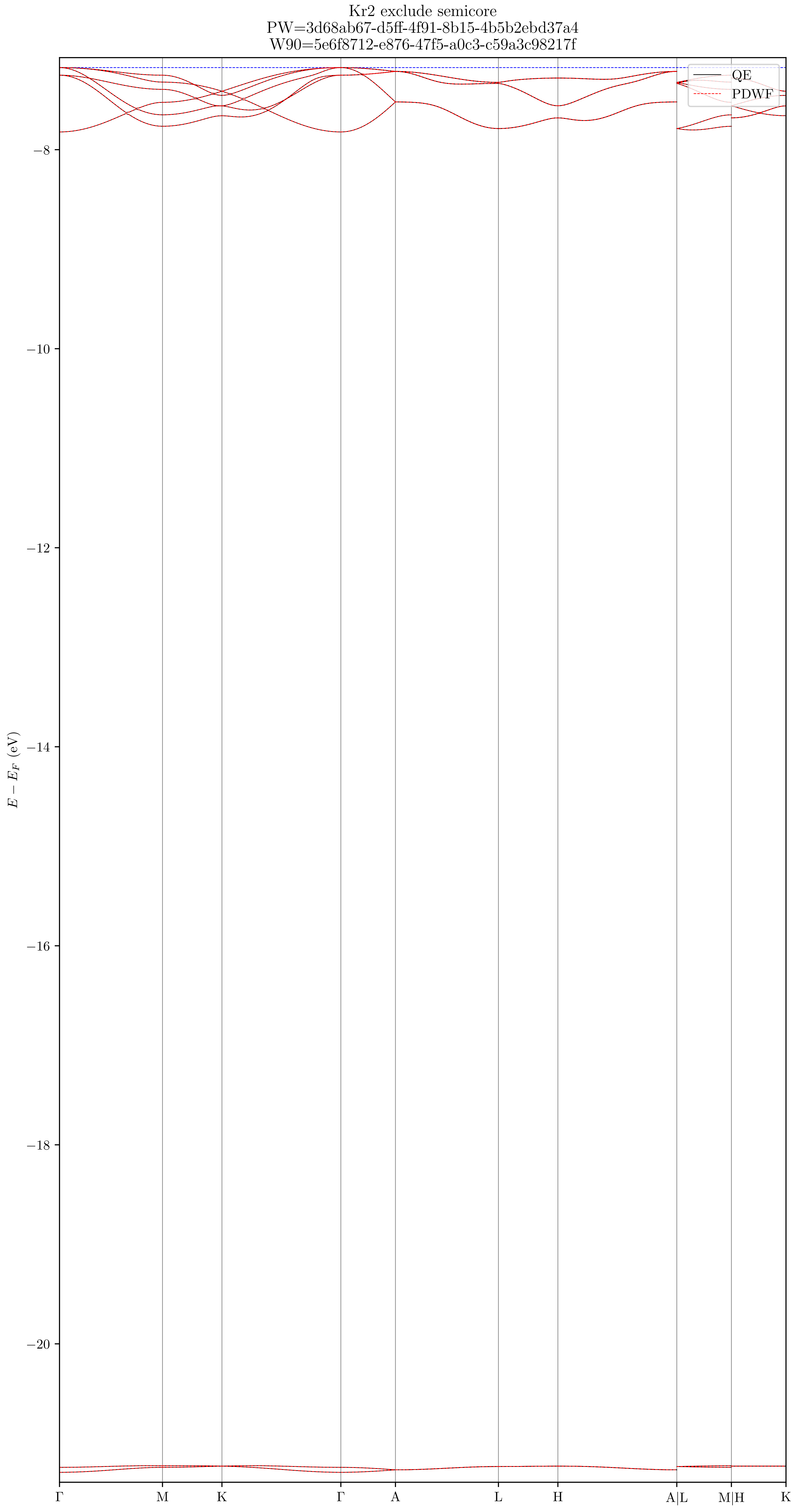

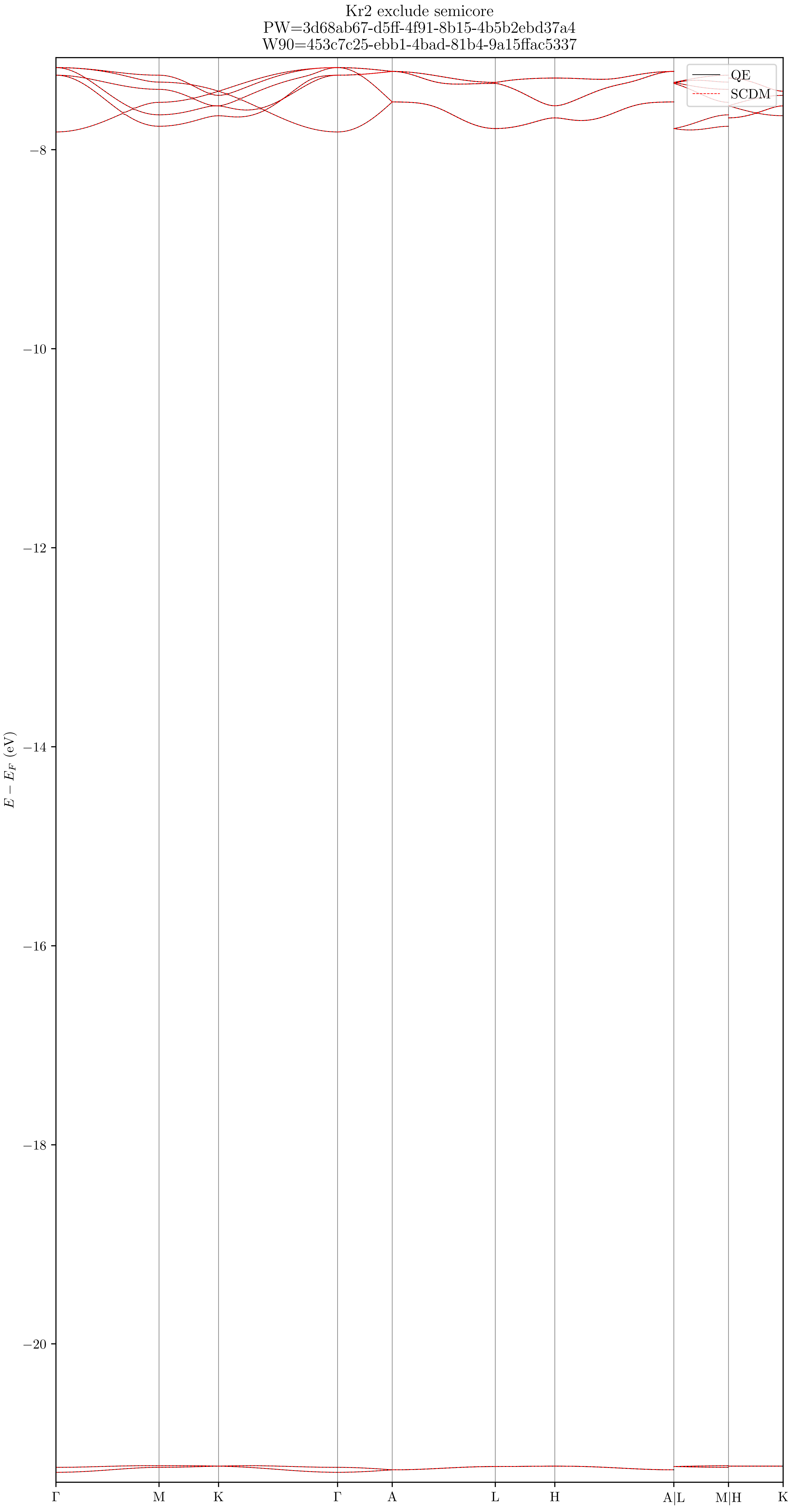

PDWF band distance (meV) and τ ($\AA$)

η_0	η_2	η_4	$\eta_0^{\max}$	$\eta_2^{\max}$	$\eta_4^{\max}$	τ
0.0	0.0	0.0	0.0	0.0	0.0	1.335

SCDM band distance (meV) and τ ($\AA$)

η_0	η_2	η_4	$\eta_0^{\max}$	$\eta_2^{\max}$	$\eta_4^{\max}$	τ
0.0	0.0	0.0	0.0	0.0	0.0	1.418

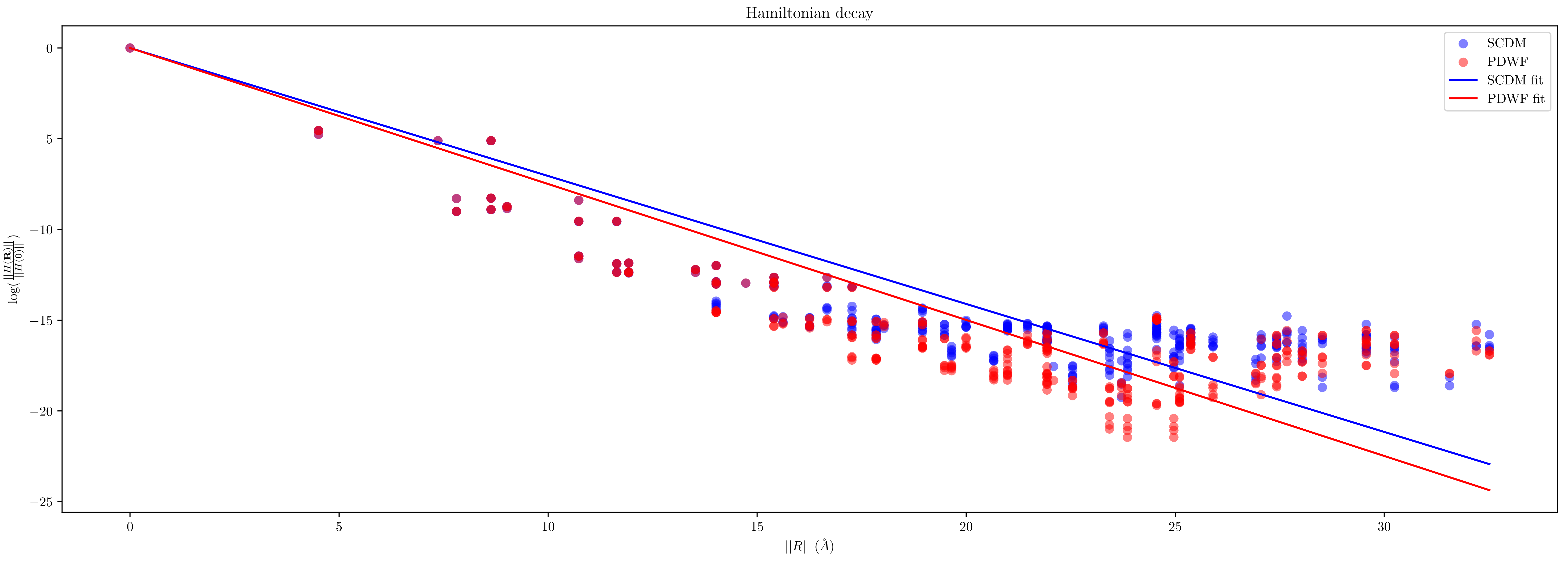

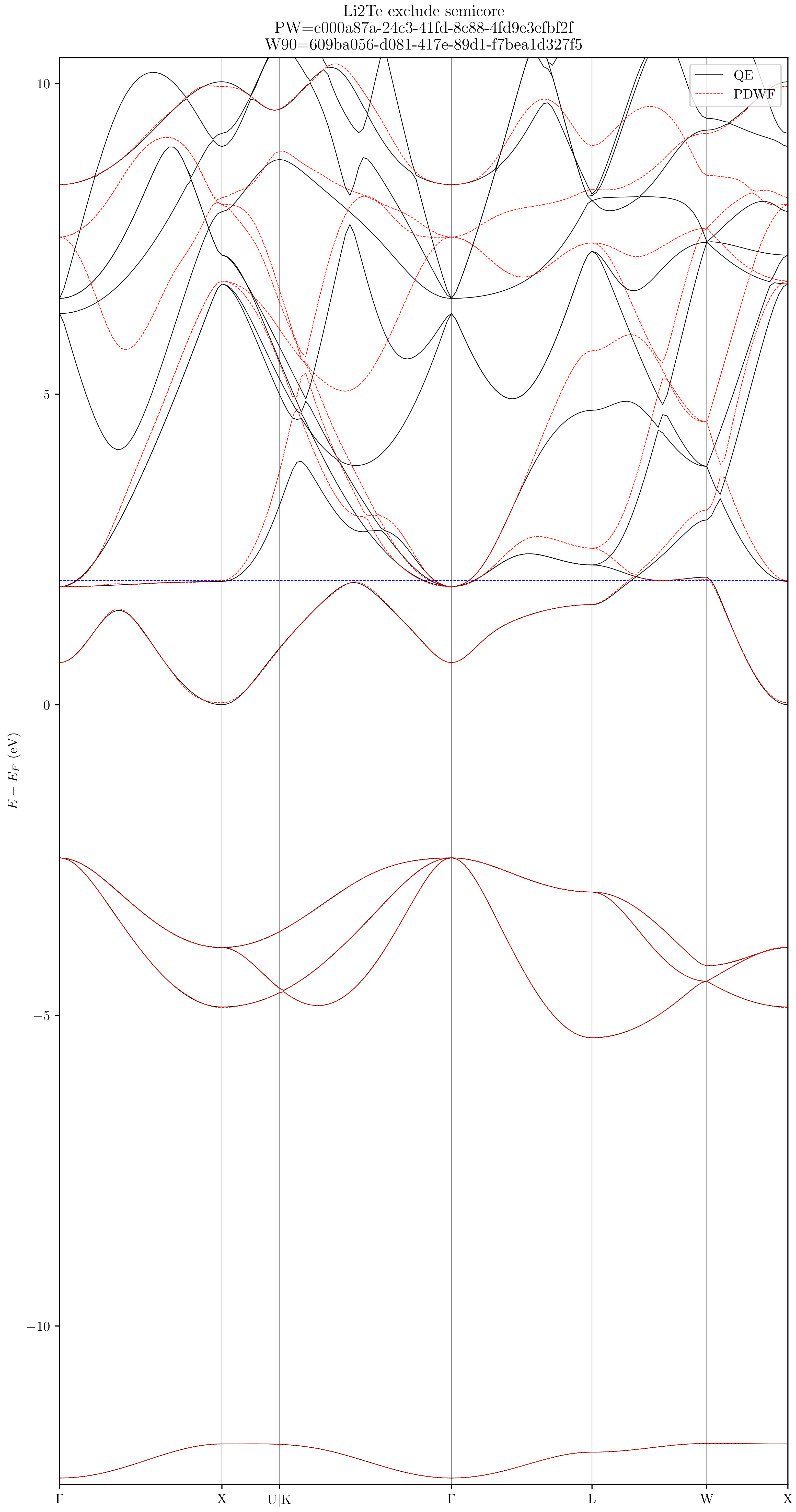

PDWF band distance (meV) and τ ($\AA$)

η_0	η_2	η_4	$\eta_0^{\max}$	$\eta_2^{\max}$	$\eta_4^{\max}$	τ
2.6	5.0	75.8	9.9	31.2	323.0	2.539

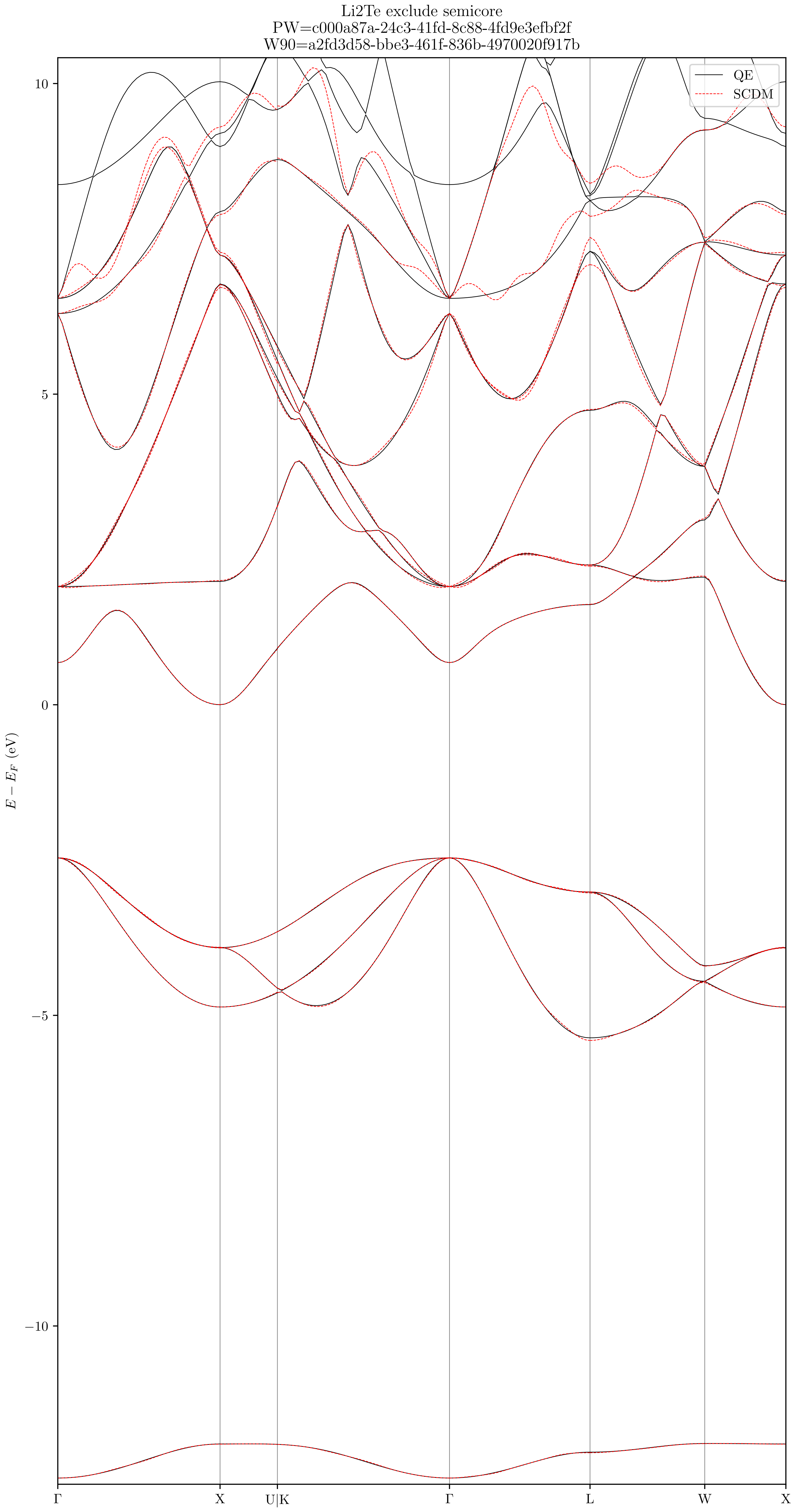

SCDM band distance (meV) and τ ($\AA$)

η_0	η_2	η_4	$\eta_0^{\max}$	$\eta_2^{\max}$	$\eta_4^{\max}$	τ
6.6	6.5	9.4	42.2	42.2	42.2	2.933

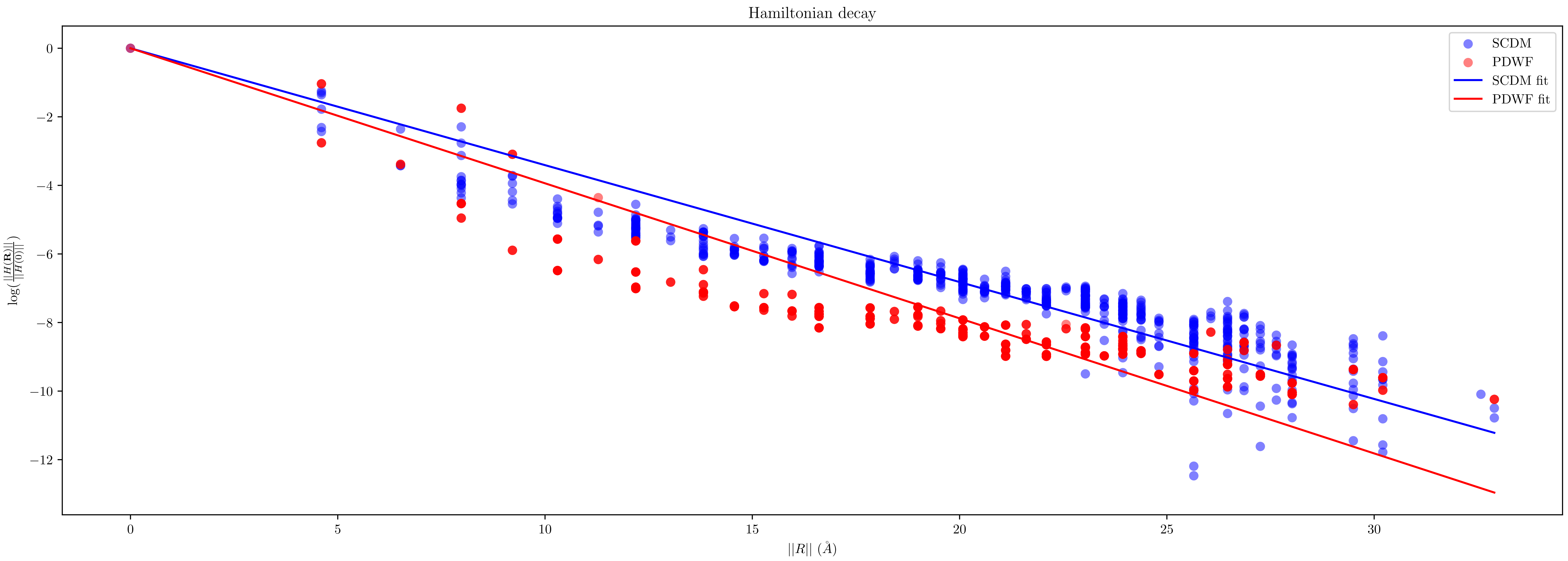

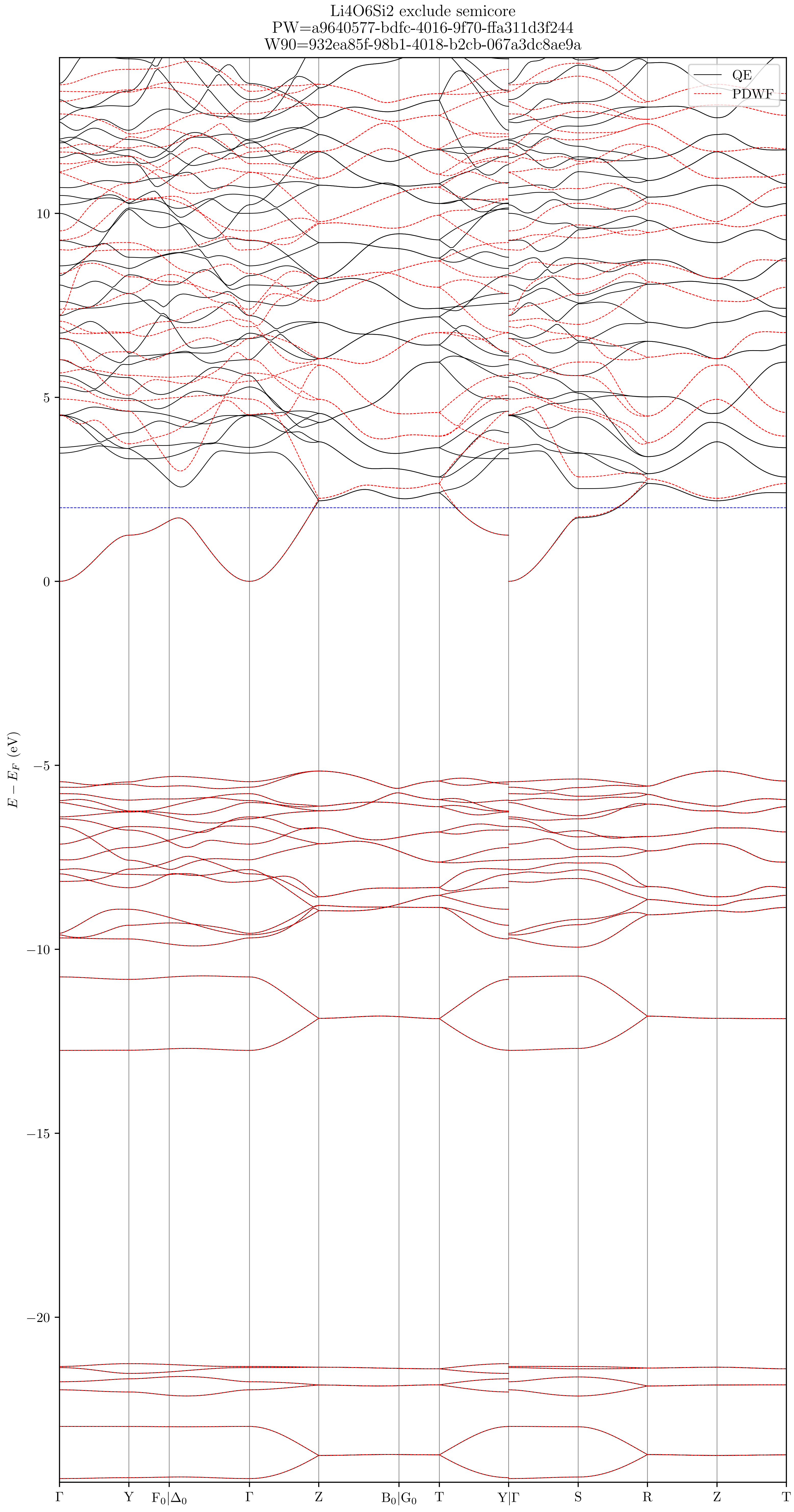

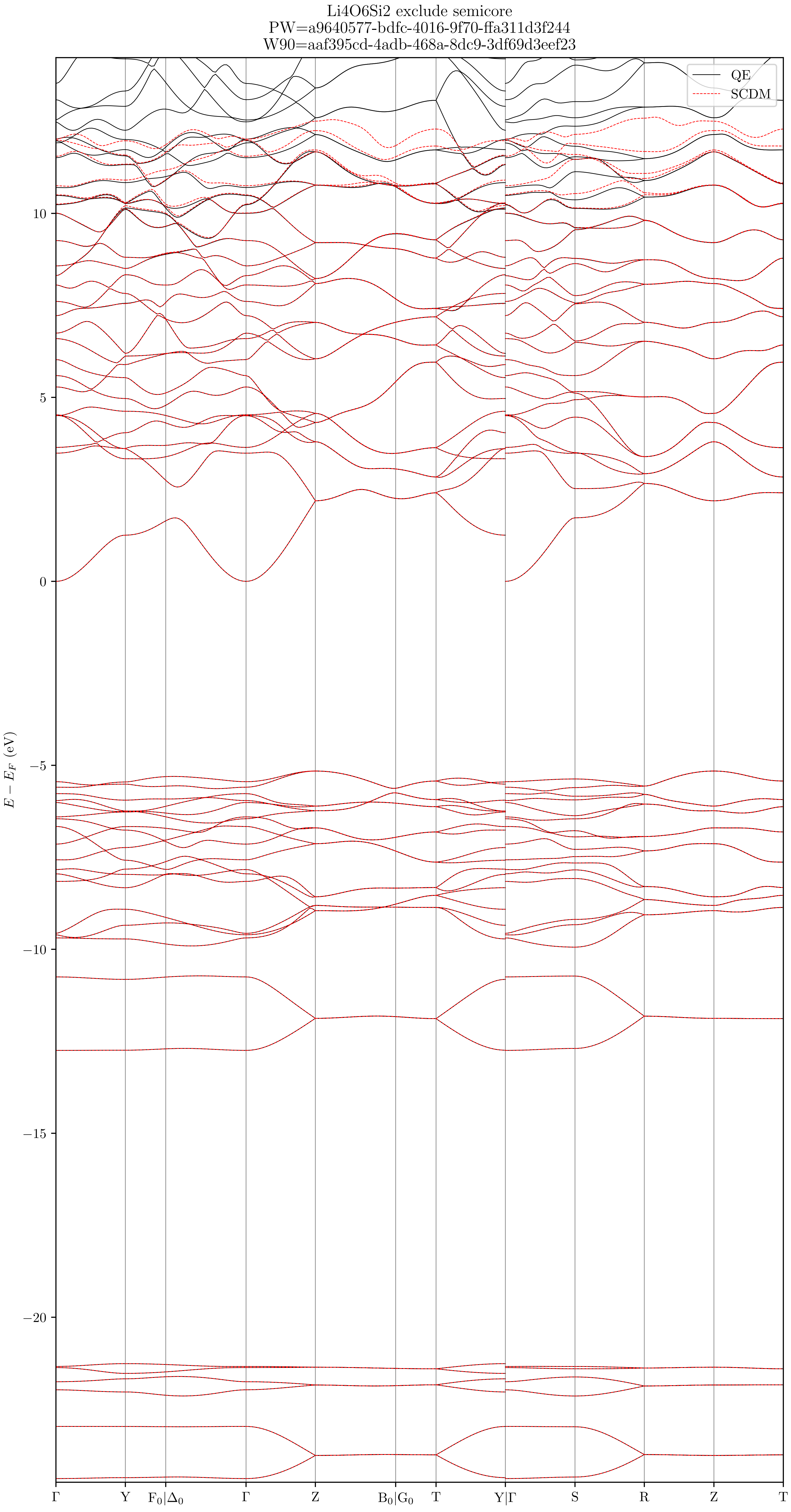

PDWF band distance (meV) and τ ($\AA$)

η_0	η_2	η_4	$\eta_0^{\max}$	$\eta_2^{\max}$	$\eta_4^{\max}$	τ
0.7	0.8	62.7	5.3	11.8	655.6	2.300

SCDM band distance (meV) and τ ($\AA$)

η_0	η_2	η_4	$\eta_0^{\max}$	$\eta_2^{\max}$	$\eta_4^{\max}$	τ
0.3	0.3	0.5	1.5	1.5	3.6	2.334

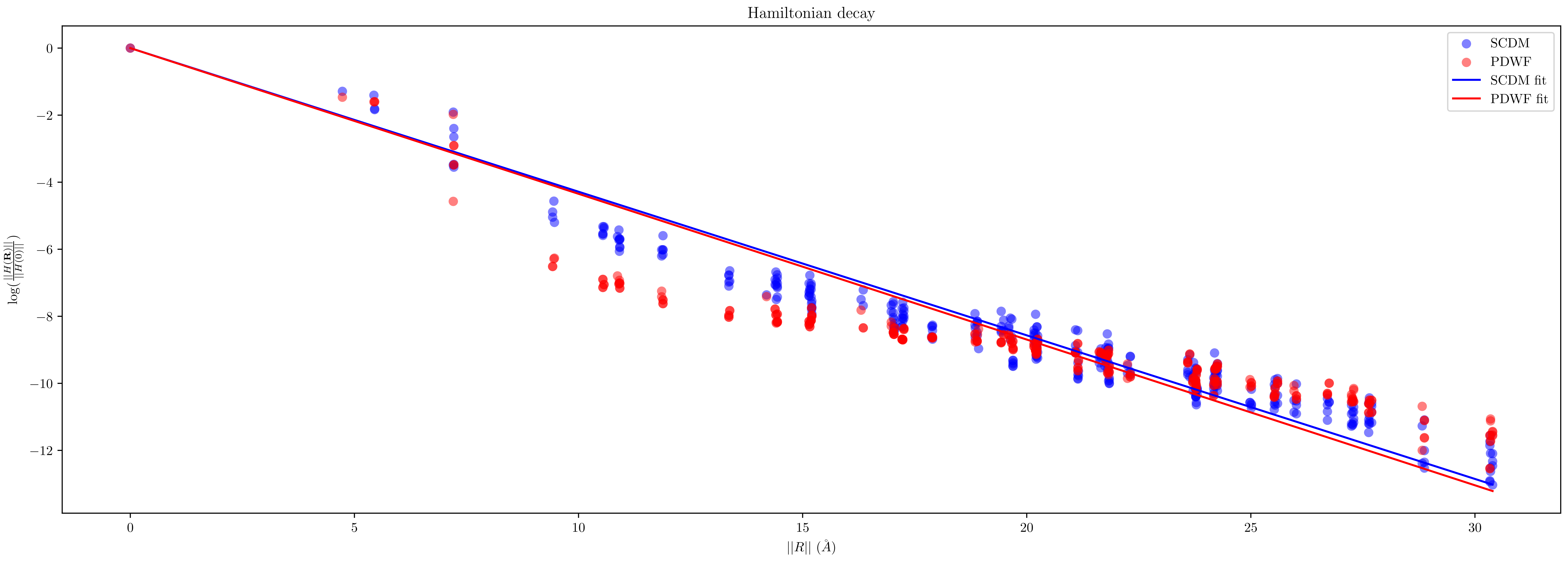

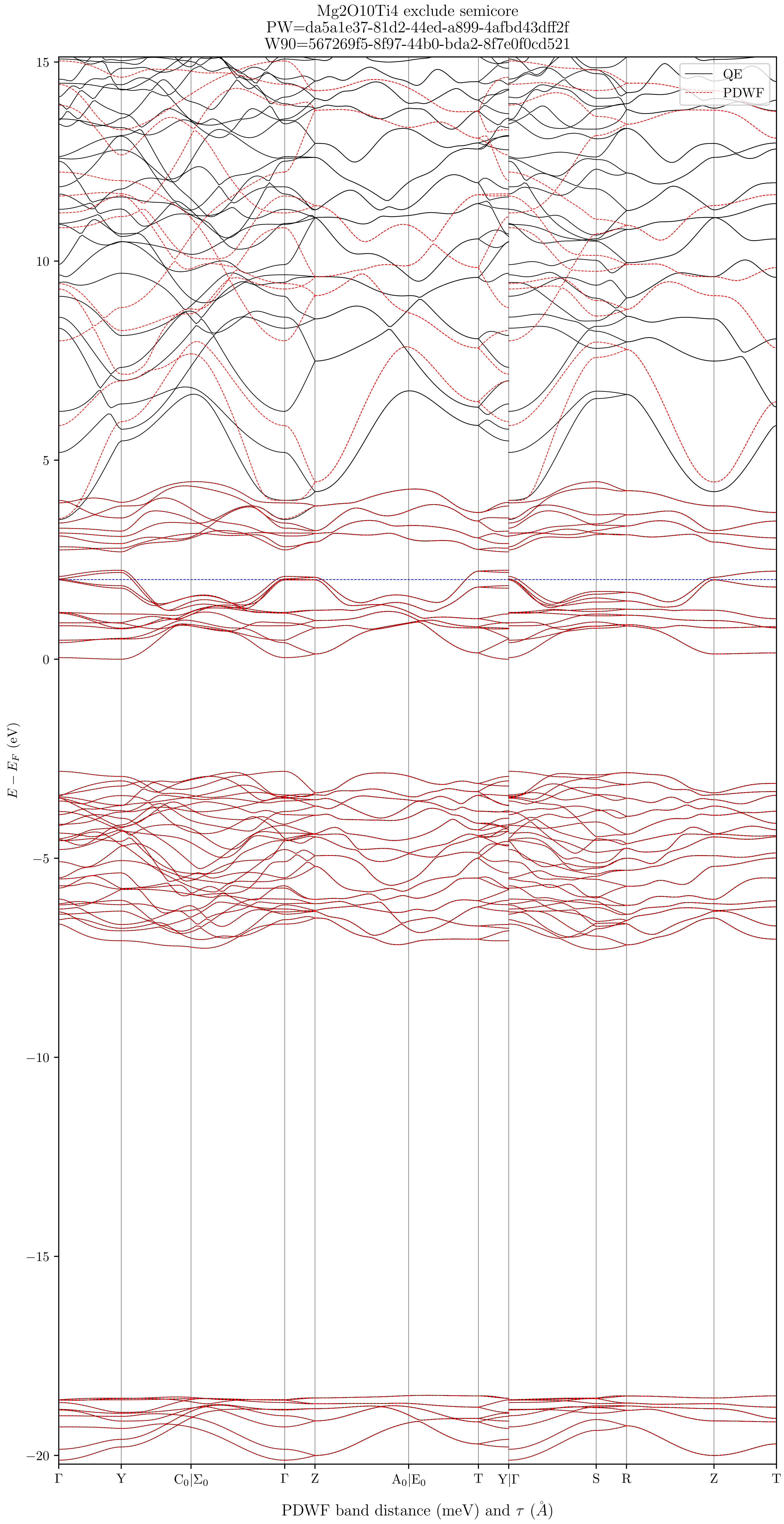

η_0	η_2	η_4	$\eta_0^{\max}$	$\eta_2^{\max}$	$\eta_4^{\max}$	τ
0.1	0.1	0.2	1.8	1.8	4.2	2.422

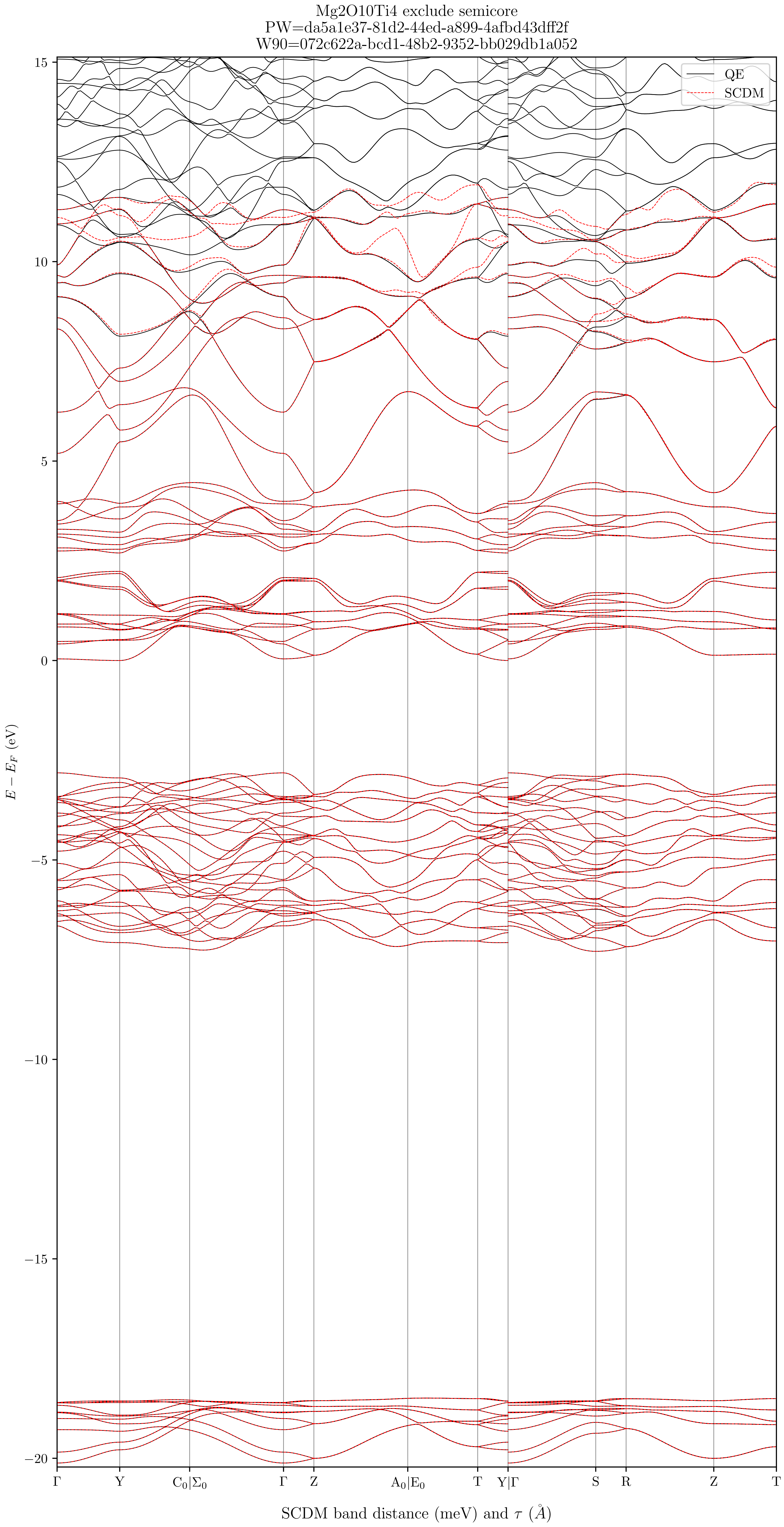

η_0	η_2	η_4	$\eta_0^{\max}$	$\eta_2^{\max}$	$\eta_4^{\max}$	τ
0.6	0.6	0.5	10.4	10.4	10.4	2.887

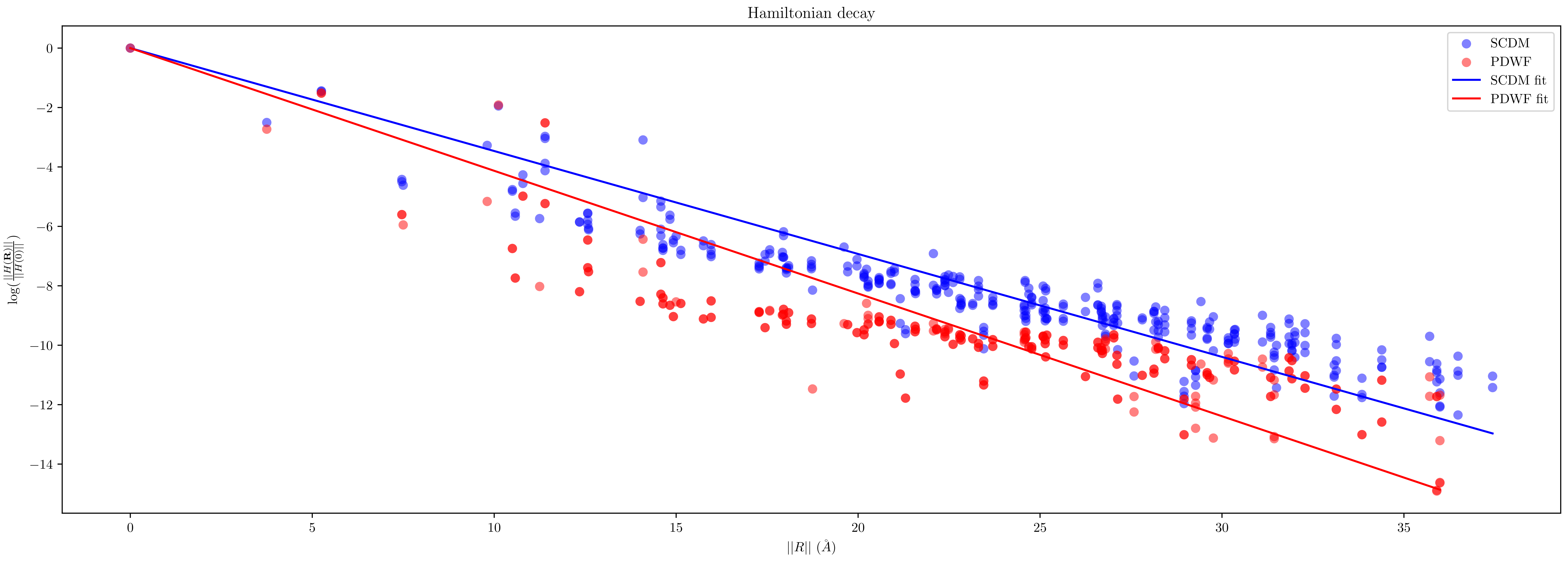

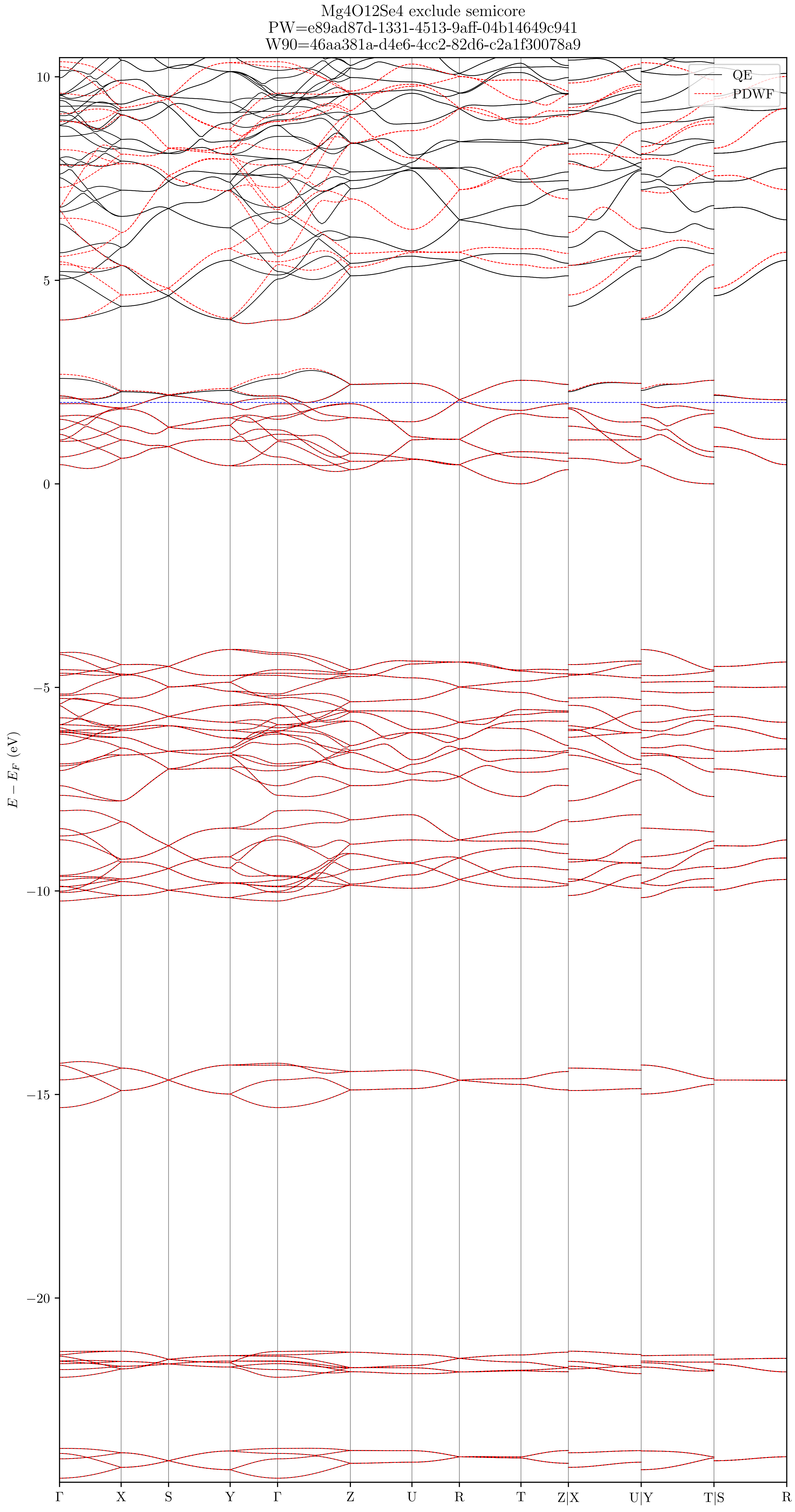

PDWF band distance (meV) and τ ($\AA$)

η_0	η_2	η_4	$\eta_0^{\max}$	$\eta_2^{\max}$	$\eta_4^{\max}$	τ
0.1	0.1	0.9	1.3	1.3	5.5	2.396

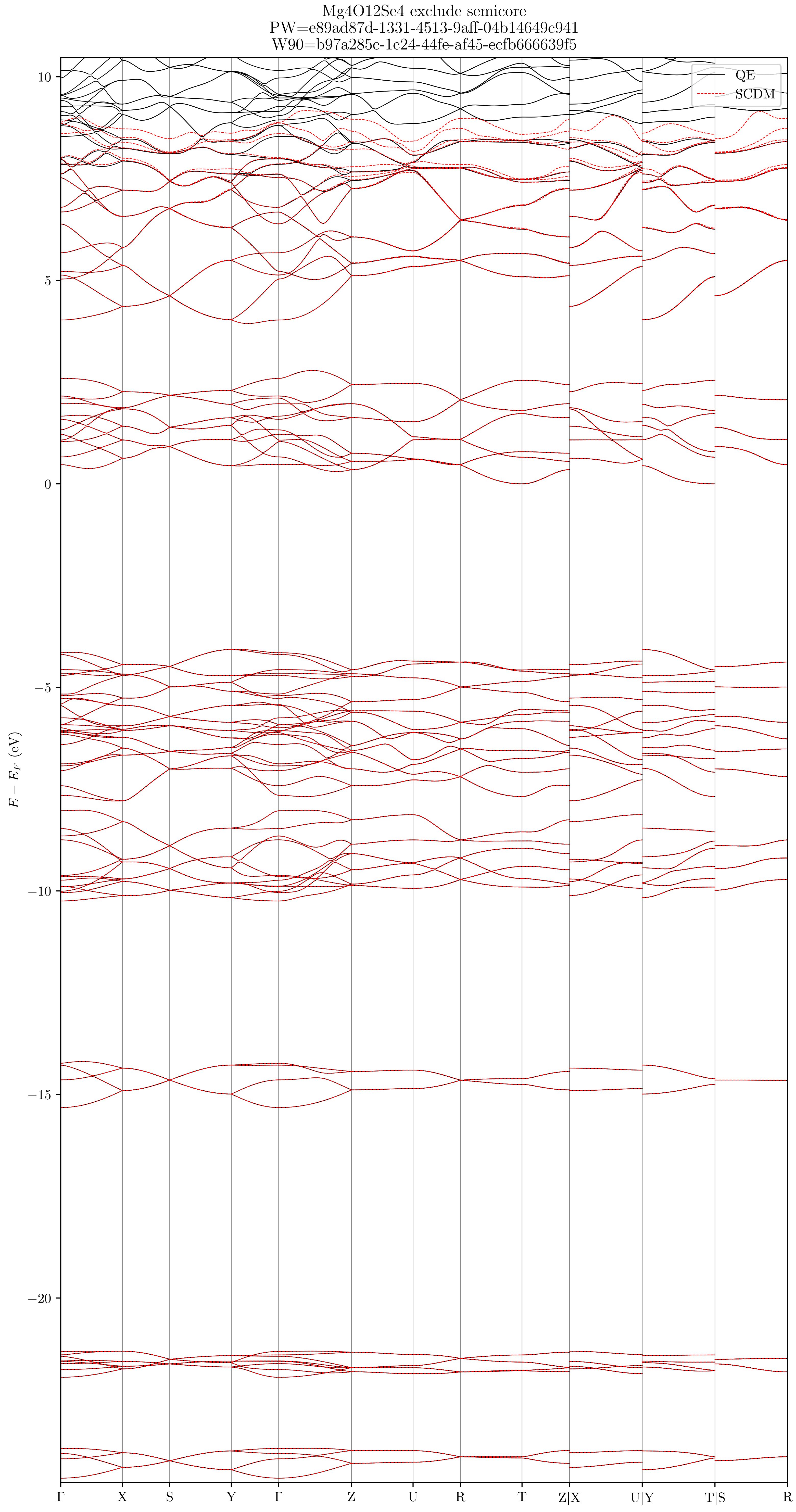

SCDM band distance (meV) and τ ($\AA$)

η_0	η_2	η_4	$\eta_0^{\max}$	$\eta_2^{\max}$	$\eta_4^{\max}$	τ
0.6	0.6	0.7	5.9	5.9	6.0	2.736

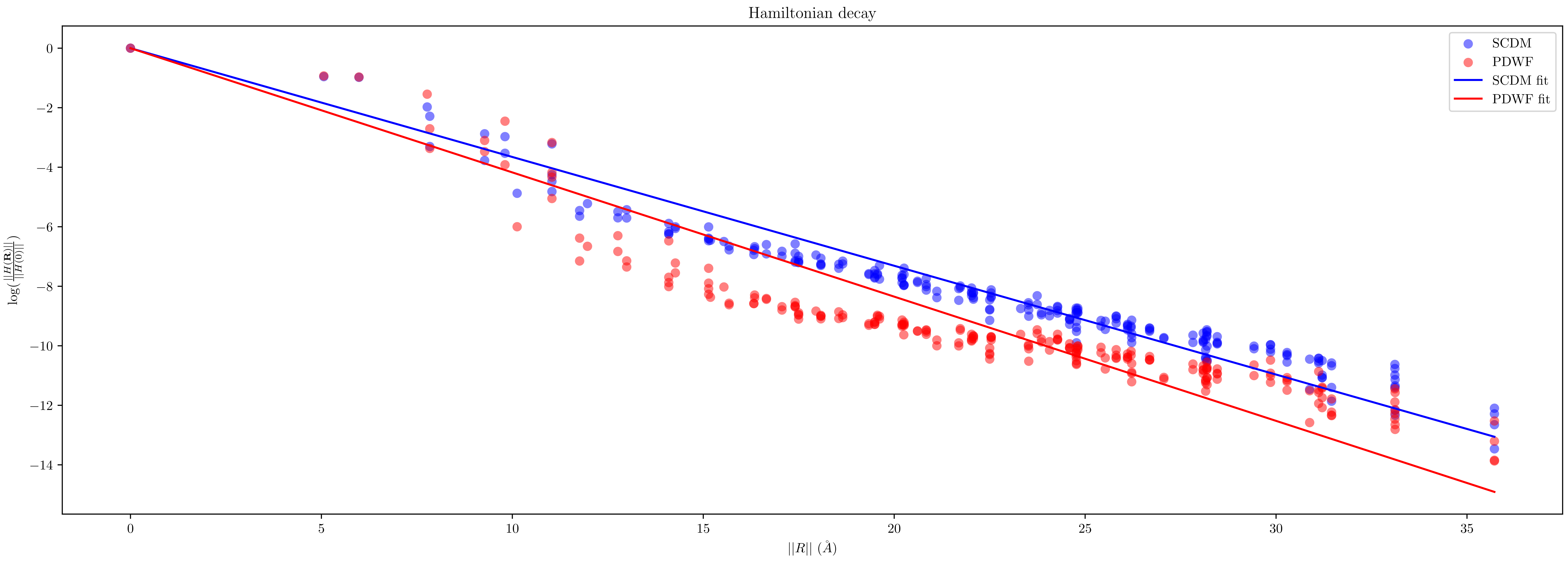

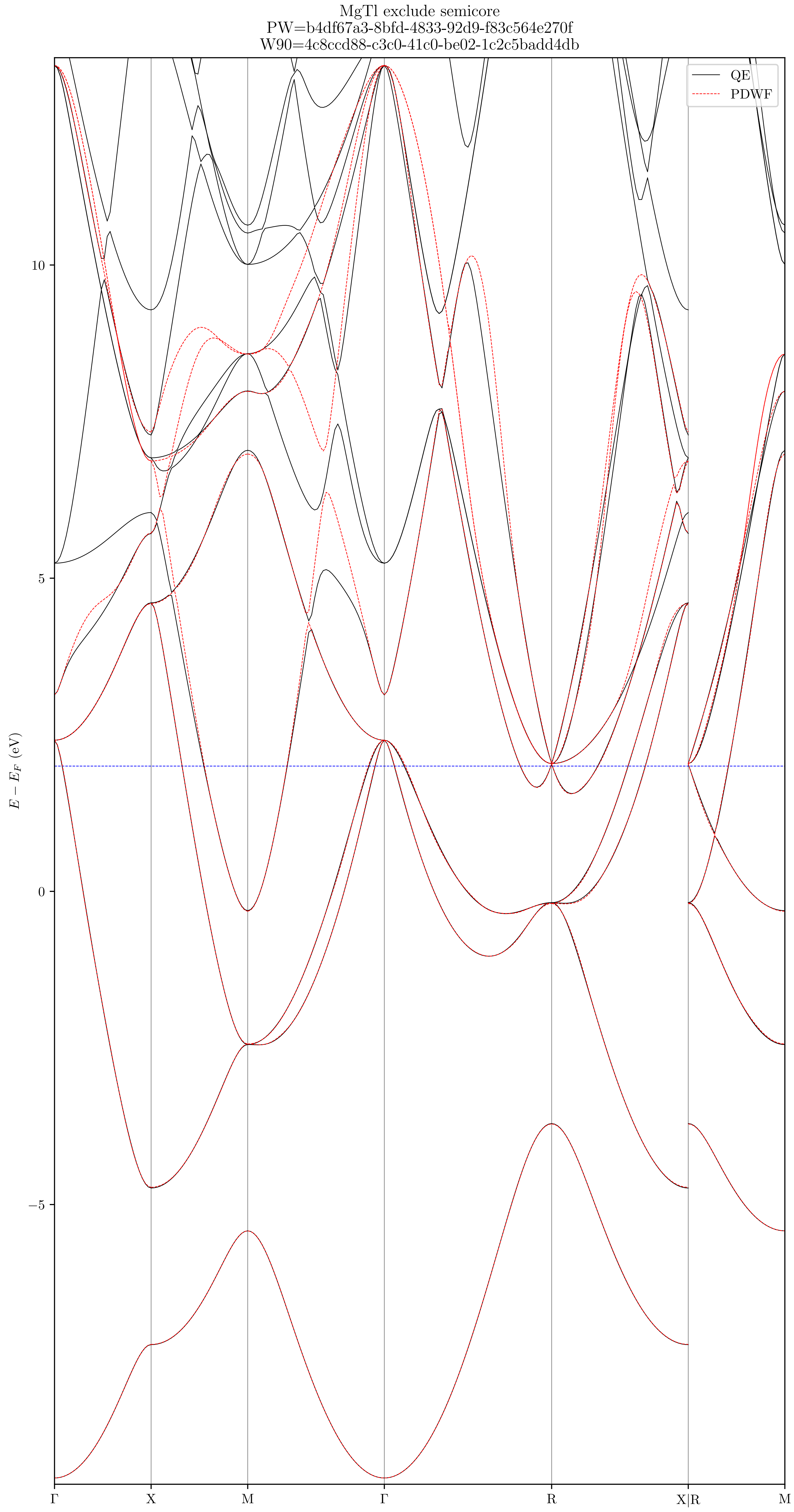

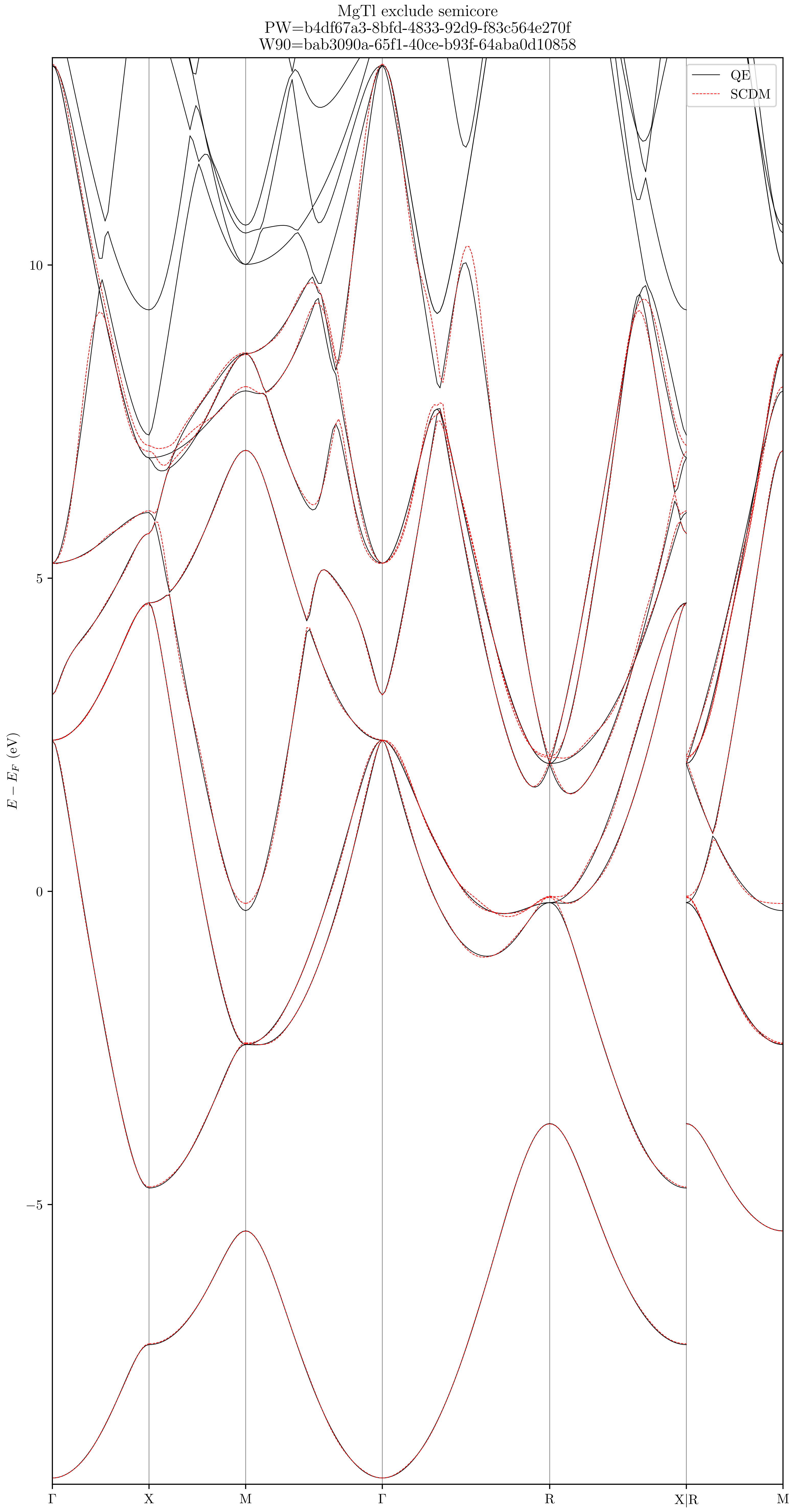

PDWF band distance (meV) and τ ($\AA$)

η_0	η_2	η_4	$\eta_0^{\max}$	$\eta_2^{\max}$	$\eta_4^{\max}$	τ
6.9	9.9	28.3	34.5	40.8	243.4	2.295

SCDM band distance (meV) and τ ($\AA$)

η_0	η_2	η_4	$\eta_0^{\max}$	$\eta_2^{\max}$	$\eta_4^{\max}$	τ
27.0	31.5	35.2	104.2	114.3	133.0	2.658

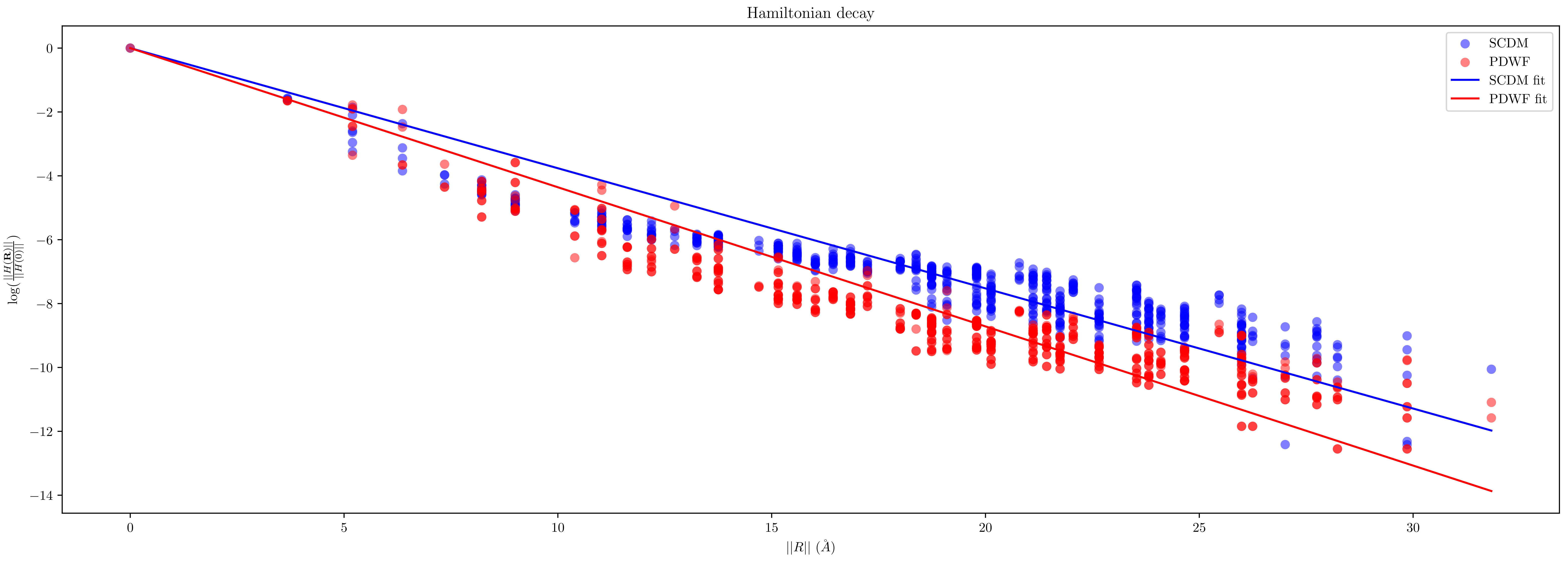

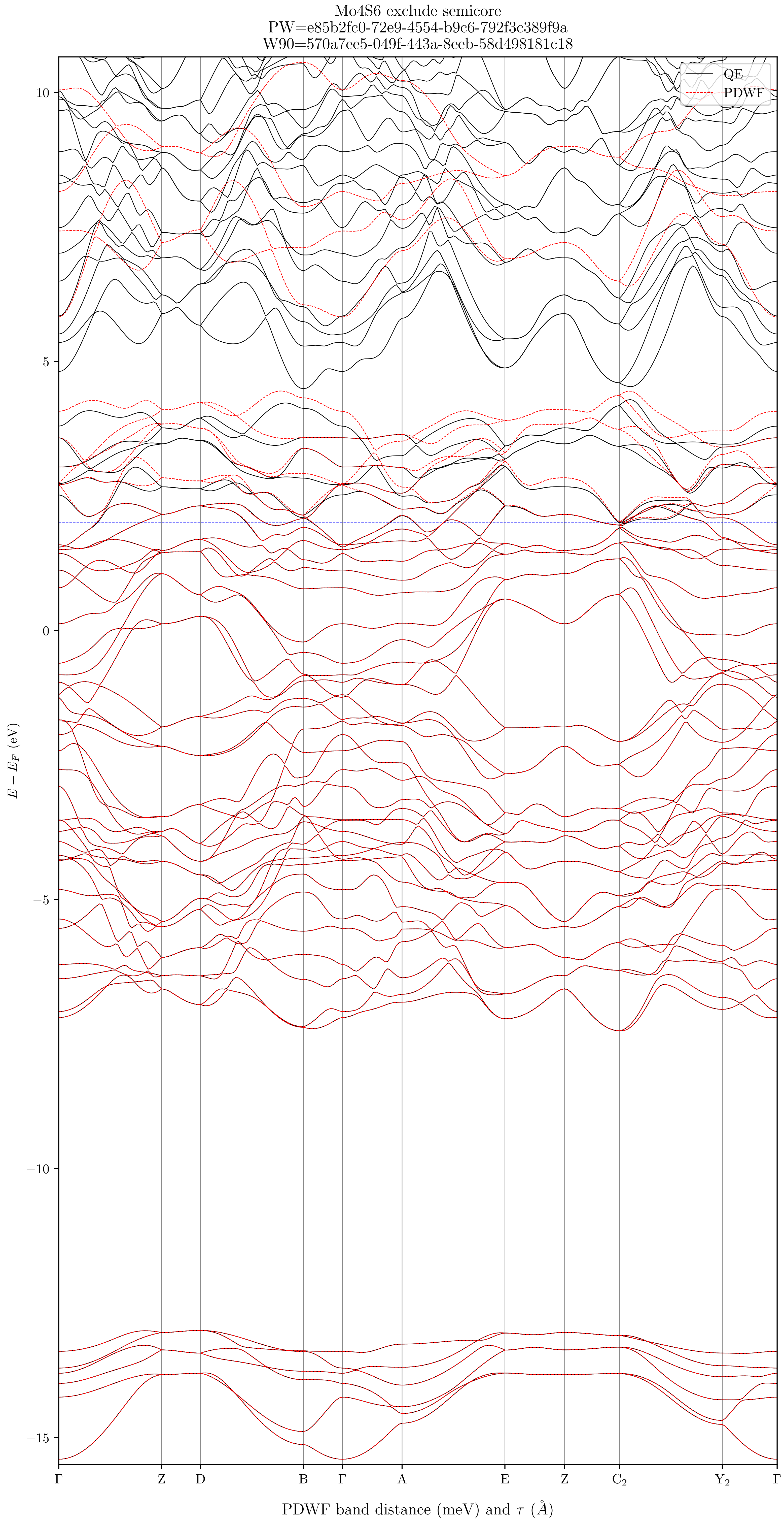

η_0	η_2	η_4	$\eta_0^{\max}$	$\eta_2^{\max}$	$\eta_4^{\max}$	τ
0.4	1.5	63.1	2.6	10.9	479.7	2.383

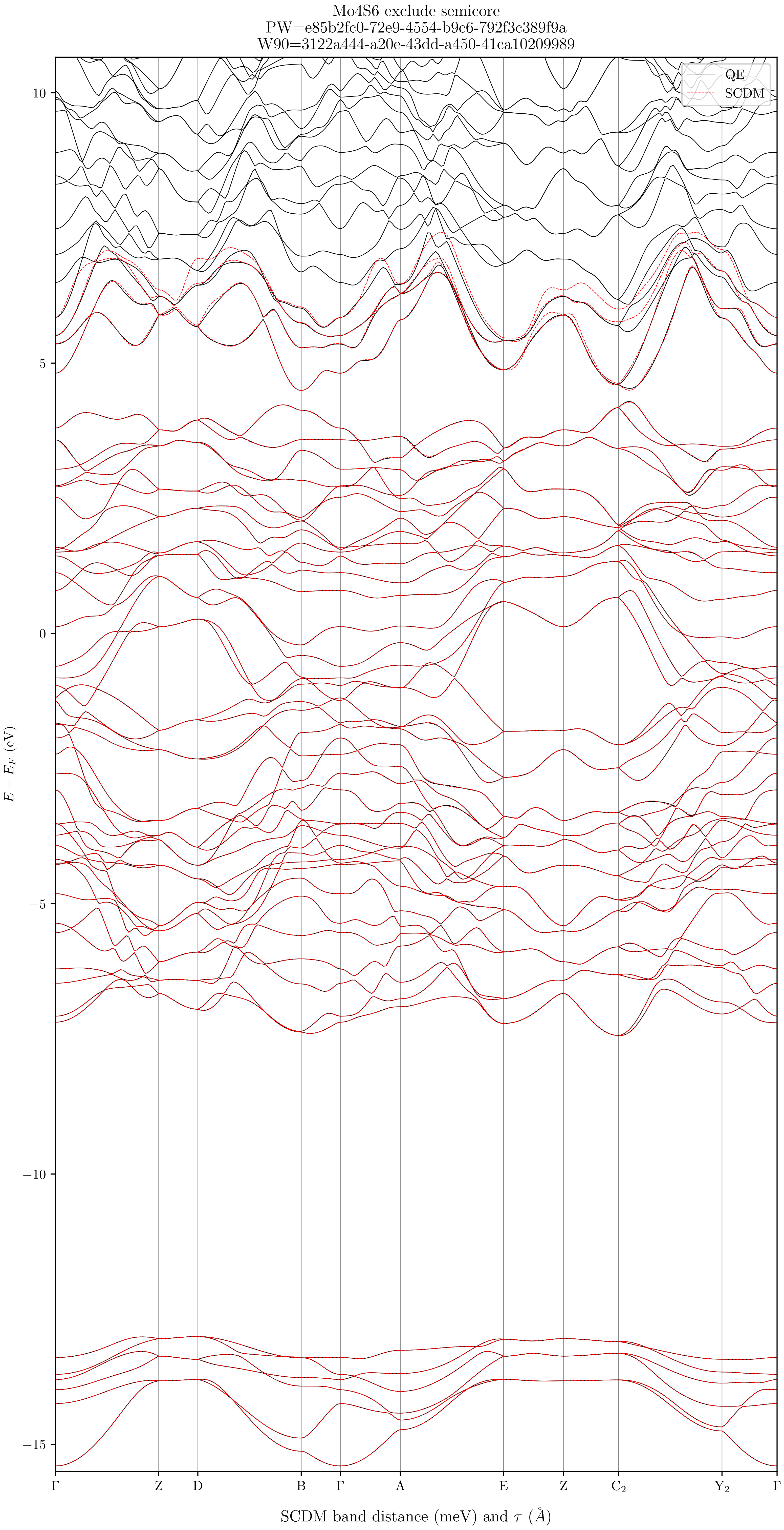

η_0	η_2	η_4	$\eta_0^{\max}$	$\eta_2^{\max}$	$\eta_4^{\max}$	τ
1.6	1.5	1.9	17.4	17.4	31.9	2.802

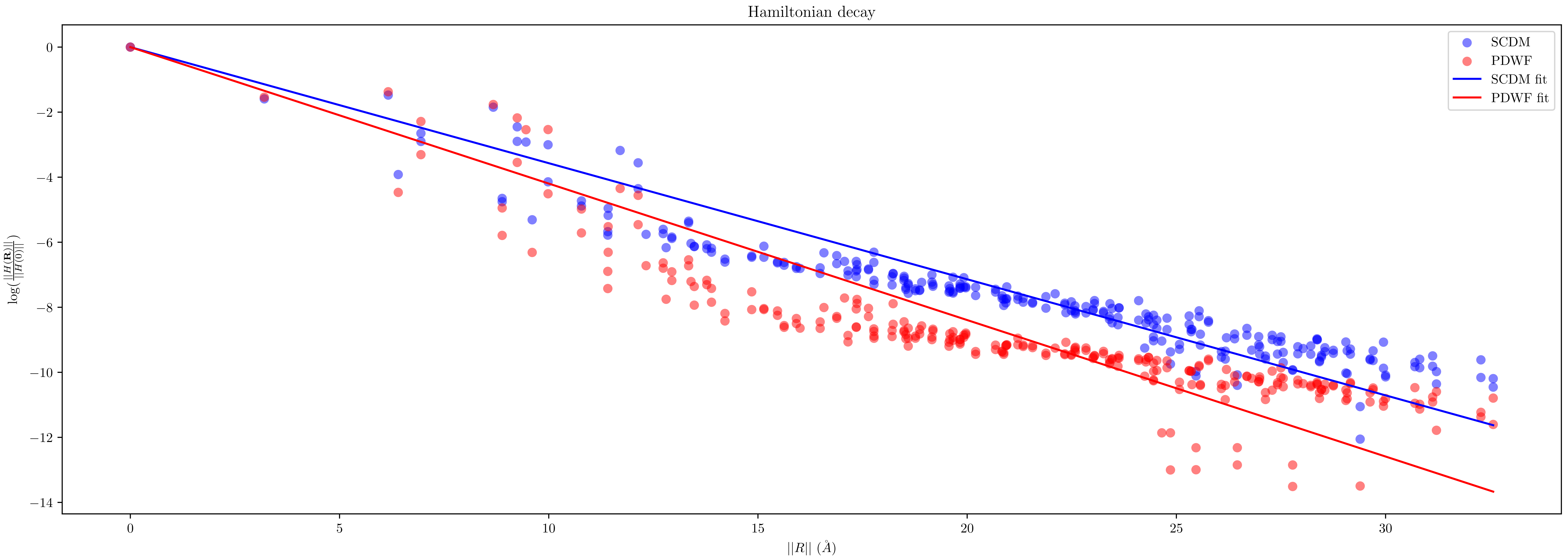

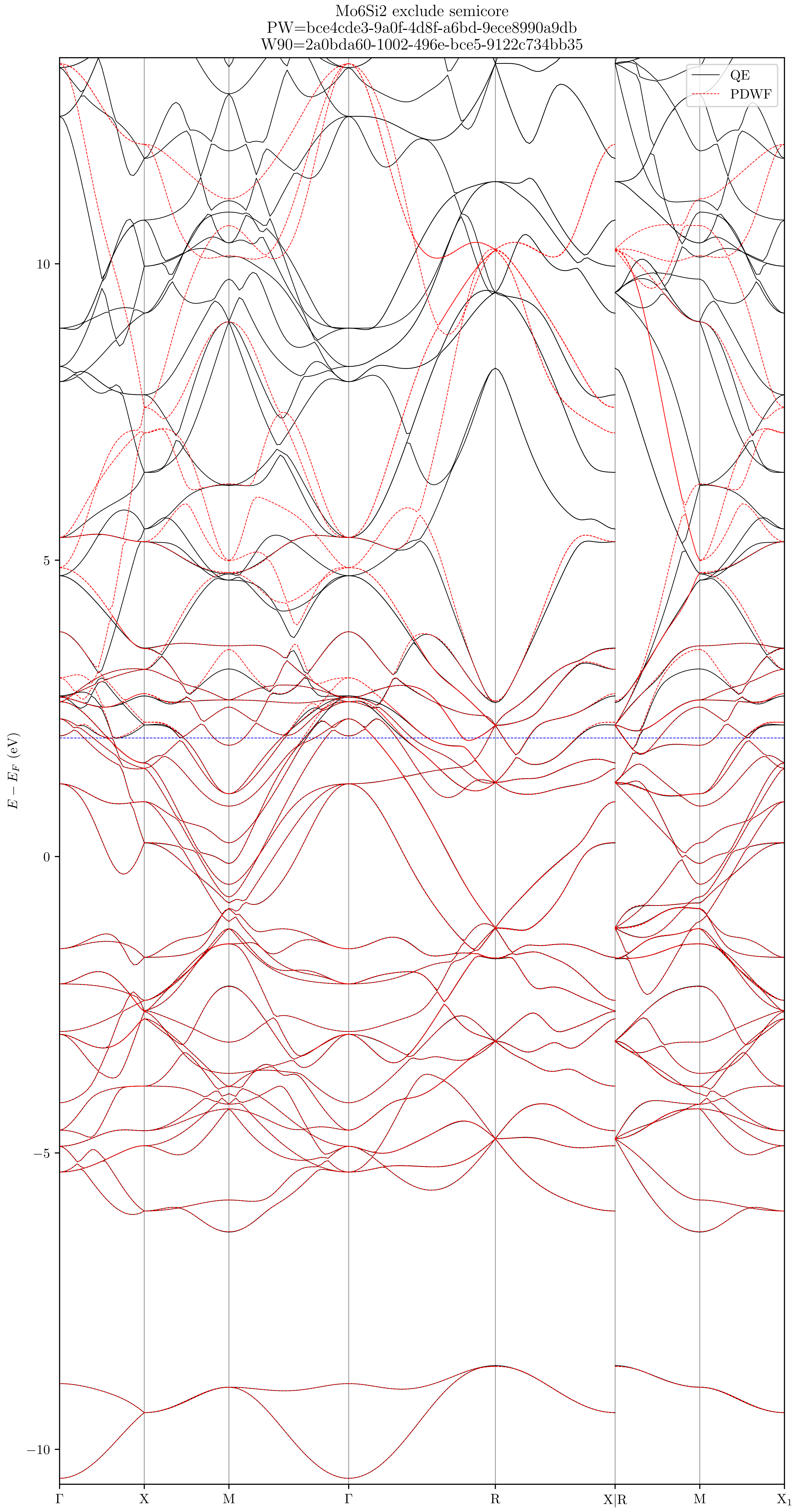

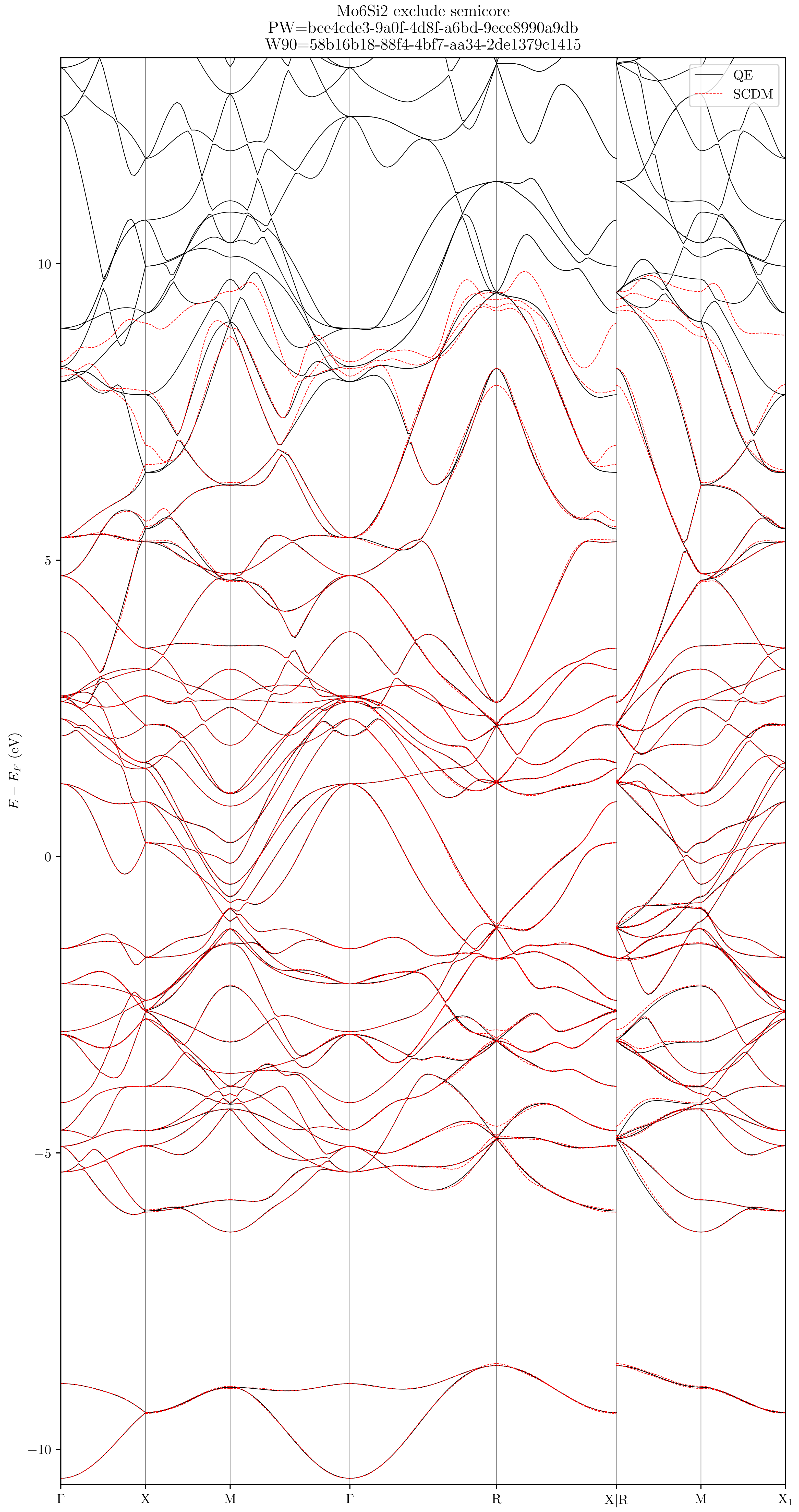

PDWF band distance (meV) and τ ($\AA$)

η_0	η_2	η_4	$\eta_0^{\max}$	$\eta_2^{\max}$	$\eta_4^{\max}$	τ
2.5	3.1	29.5	16.9	17.5	350.5	2.312

SCDM band distance (meV) and τ ($\AA$)

η_0	η_2	η_4	$\eta_0^{\max}$	$\eta_2^{\max}$	$\eta_4^{\max}$	τ
15.4	13.9	12.8	208.9	208.9	208.9	2.636

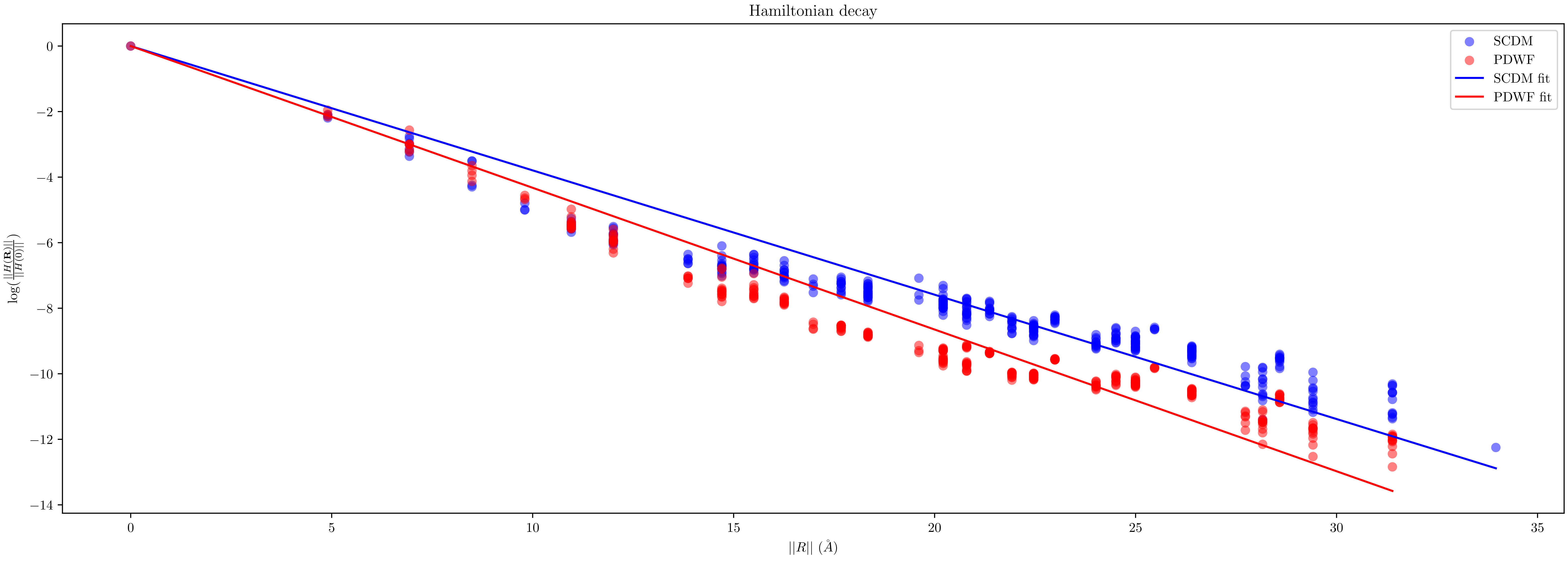

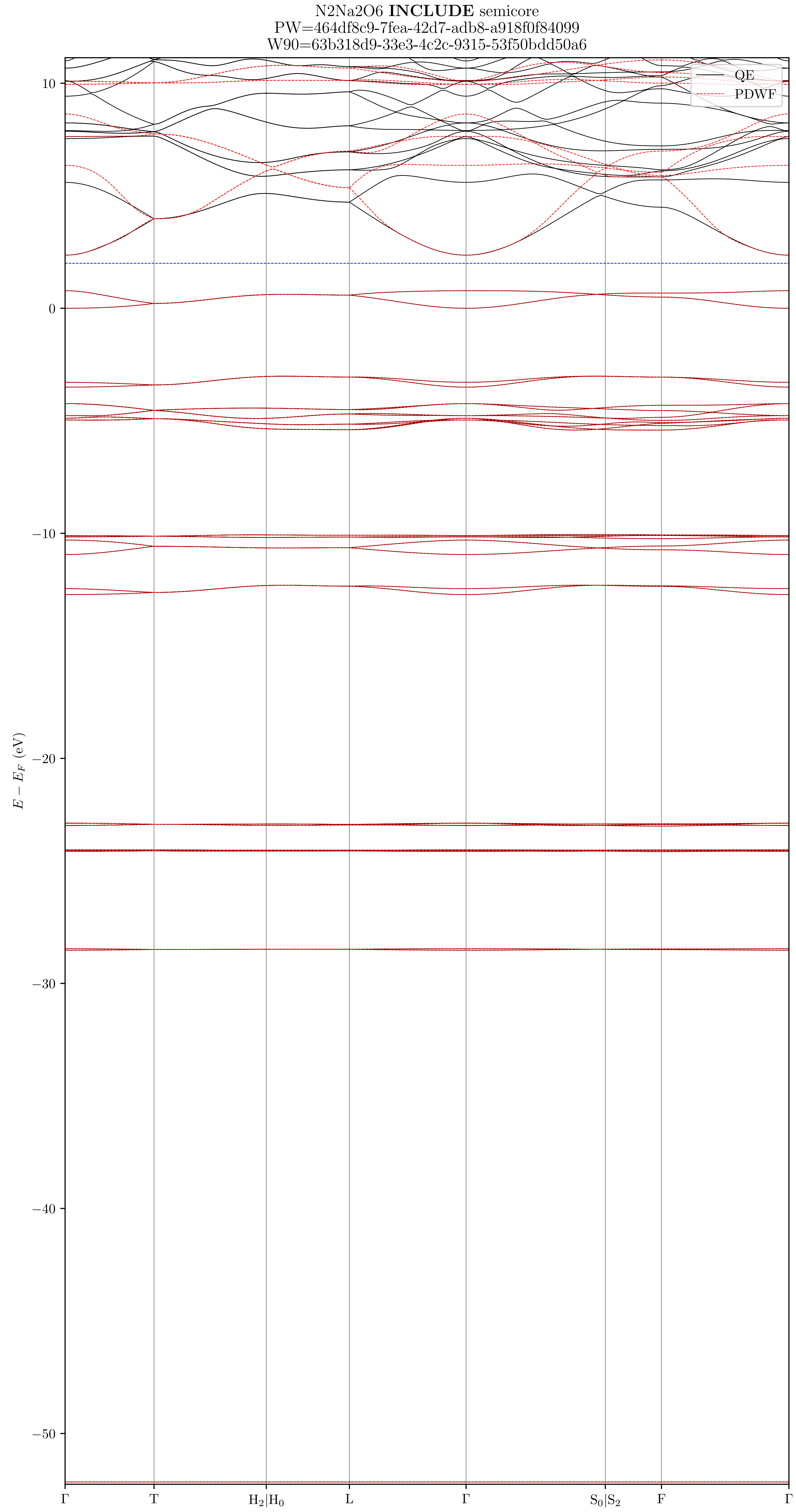

PDWF band distance (meV) and τ ($\AA$)

η_0	η_2	η_4	η_0^{max}	η_2^{max}	η_4^{max}	τ
0.2	0.2	1.7	1.1	1.1	28.8	2.431

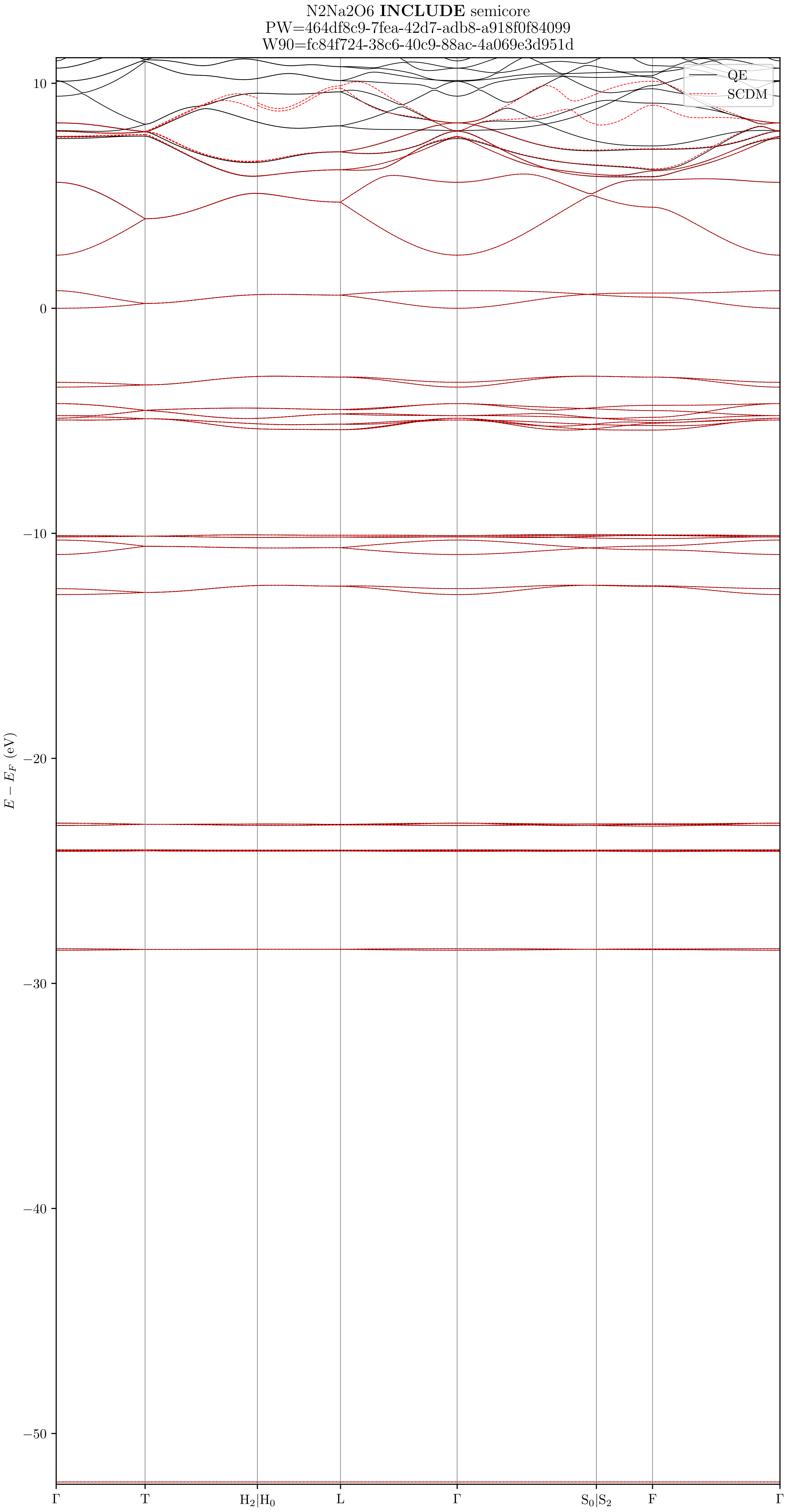

SCDM band distance (meV) and τ ($\AA$)

η_0	η_2	η_4	η_0^{max}	η_2^{max}	η_4^{max}	τ
0.2	0.2	0.2	2.3	2.3	2.3	2.349

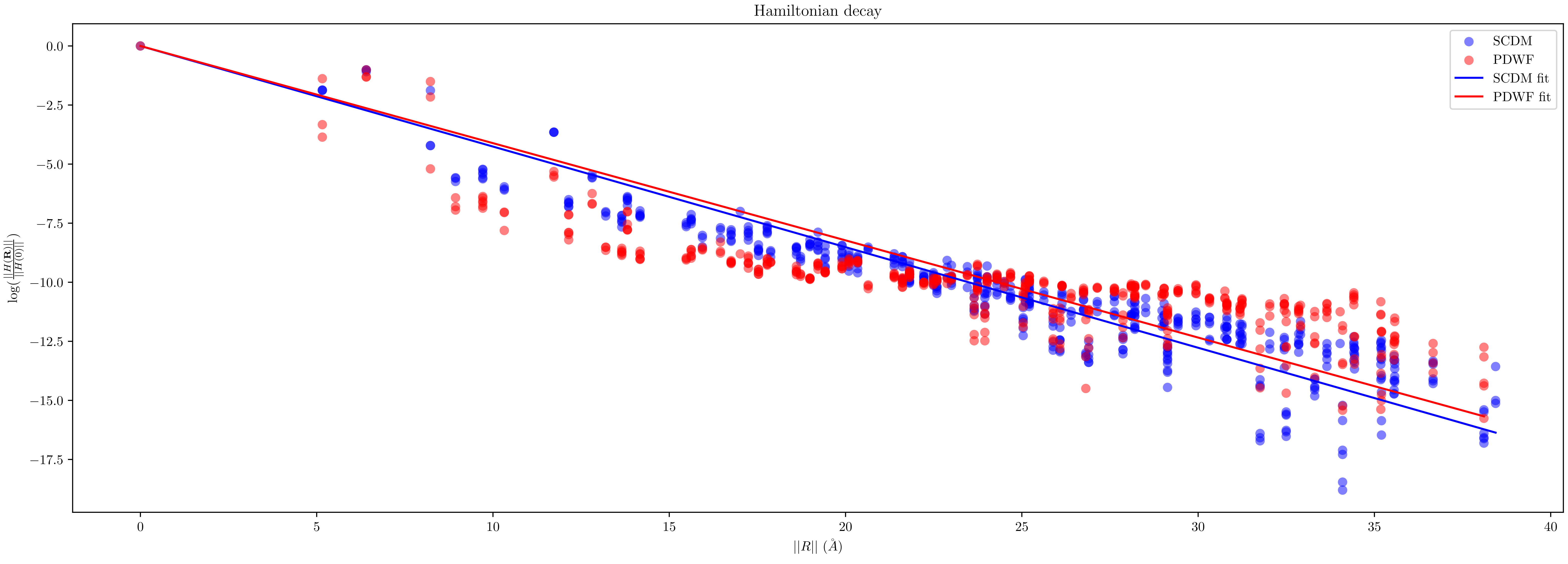

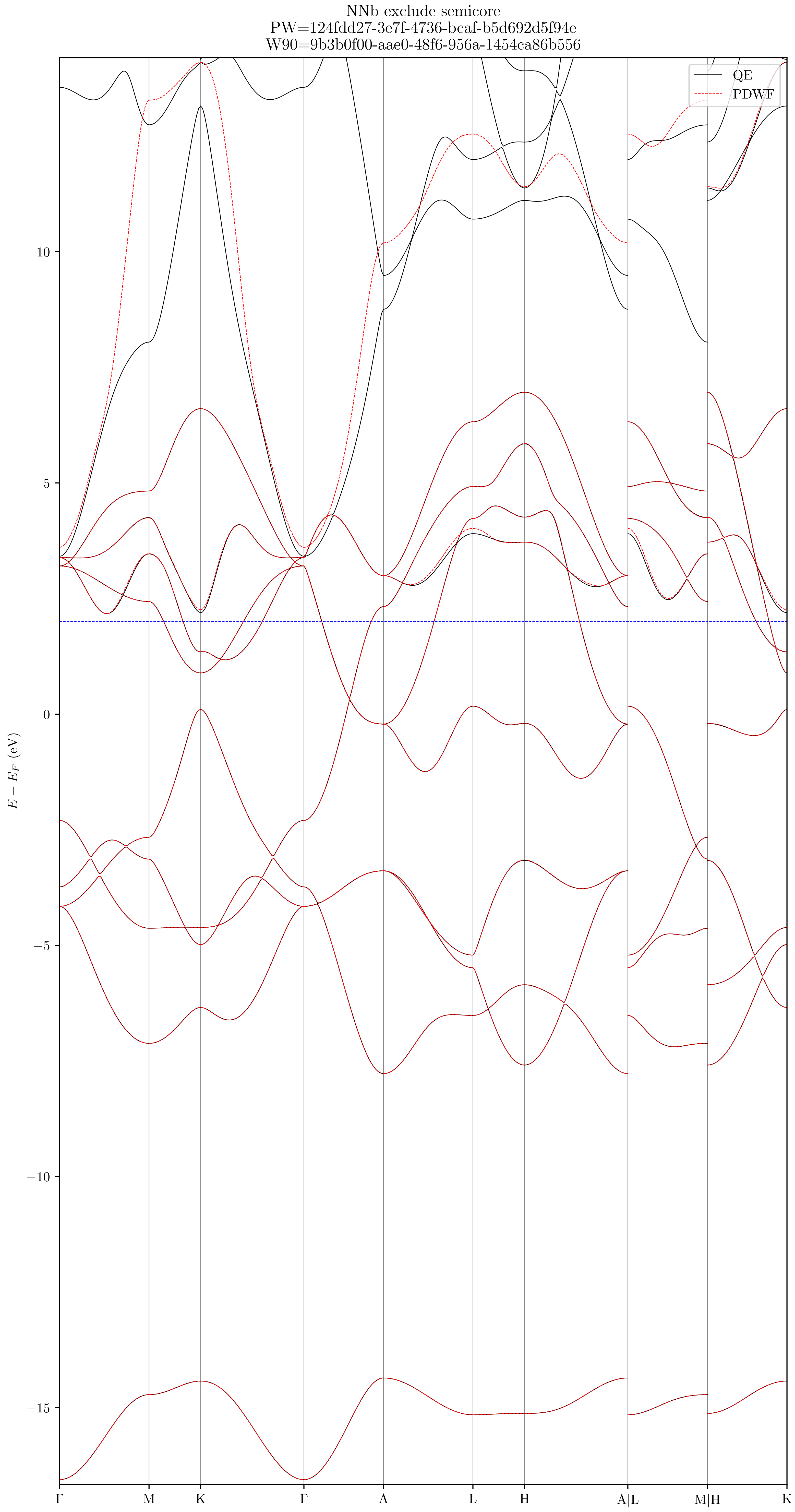

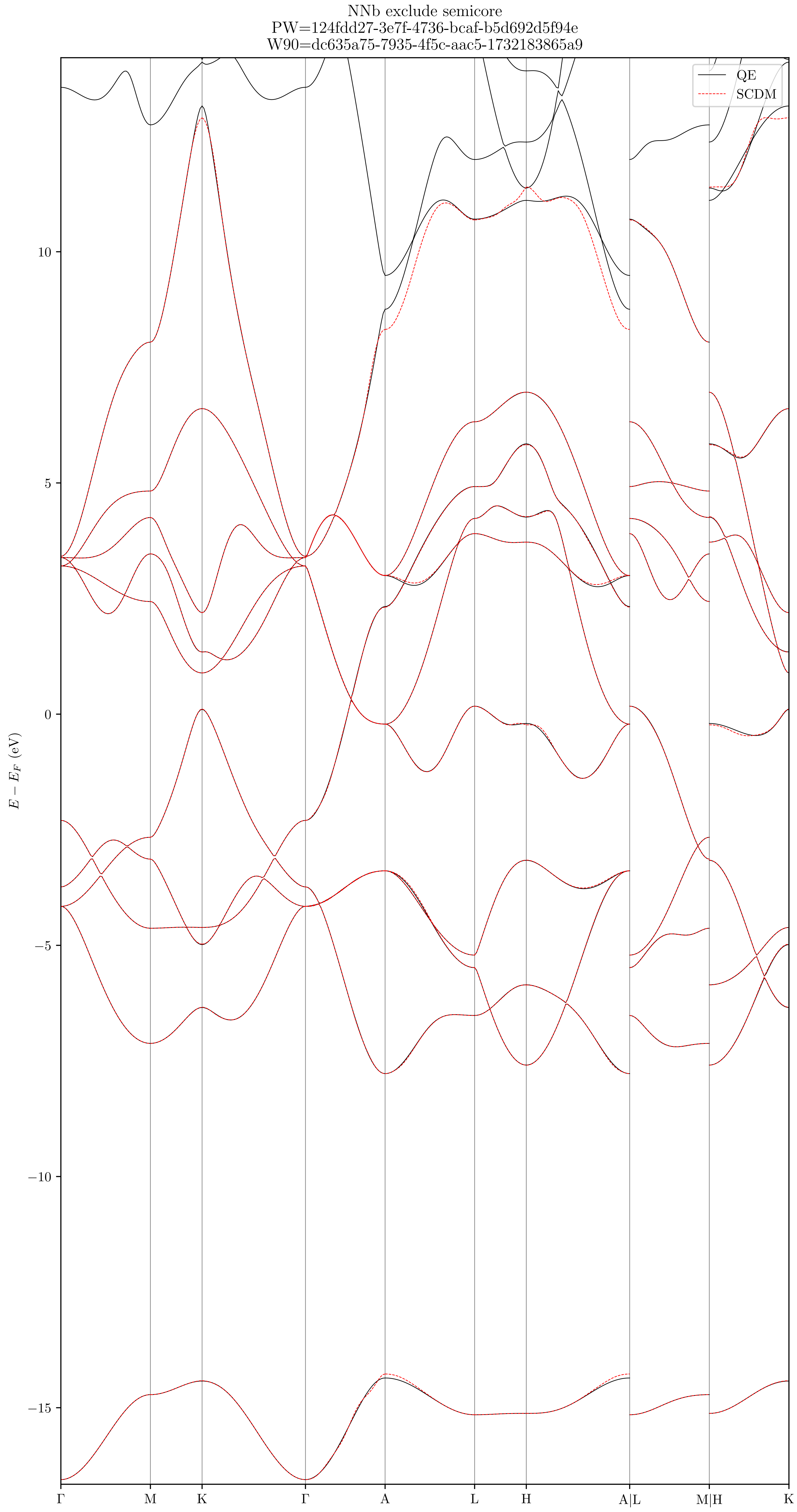

PDWF band distance (meV) and τ ($\AA$)

η_0	η_2	η_4	$\eta_0^{\max}$	$\eta_2^{\max}$	$\eta_4^{\max}$	τ
1.3	1.9	22.9	7.9	7.9	197.9	1.749

SCDM band distance (meV) and τ ($\AA$)

η_0	η_2	η_4	$\eta_0^{\max}$	$\eta_2^{\max}$	$\eta_4^{\max}$	τ
13.4	12.8	12.2	86.2	86.2	86.2	2.084

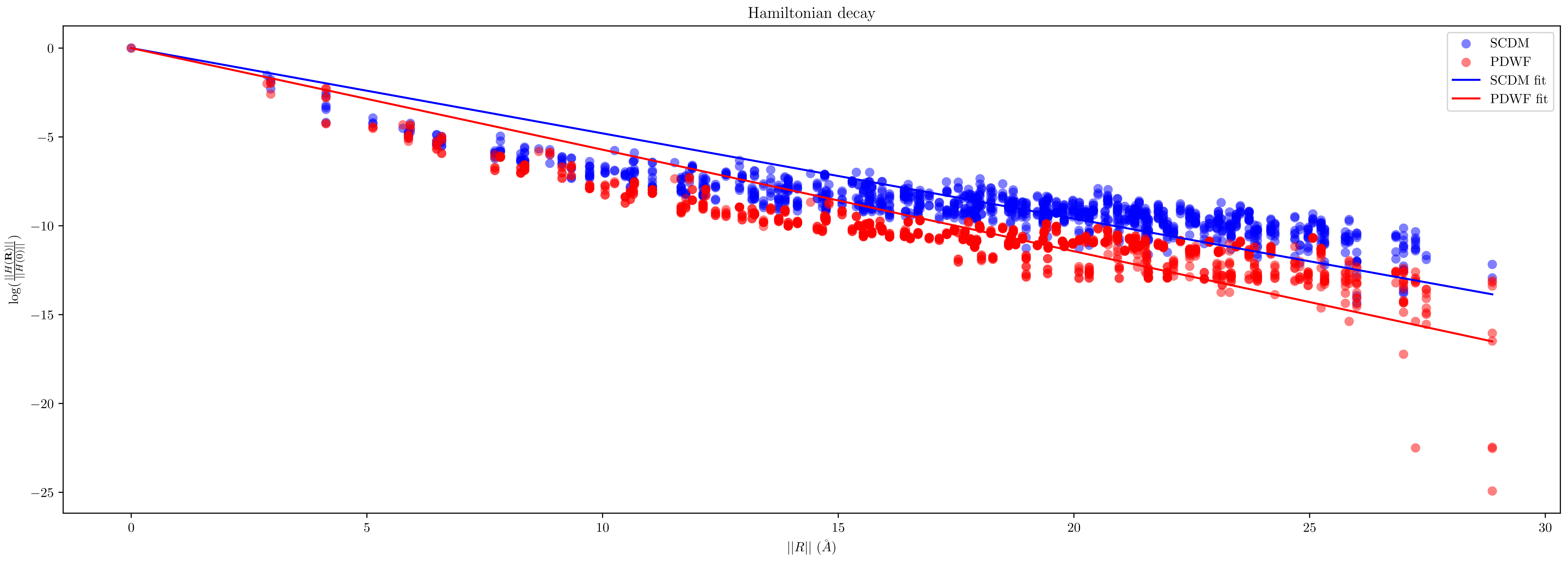

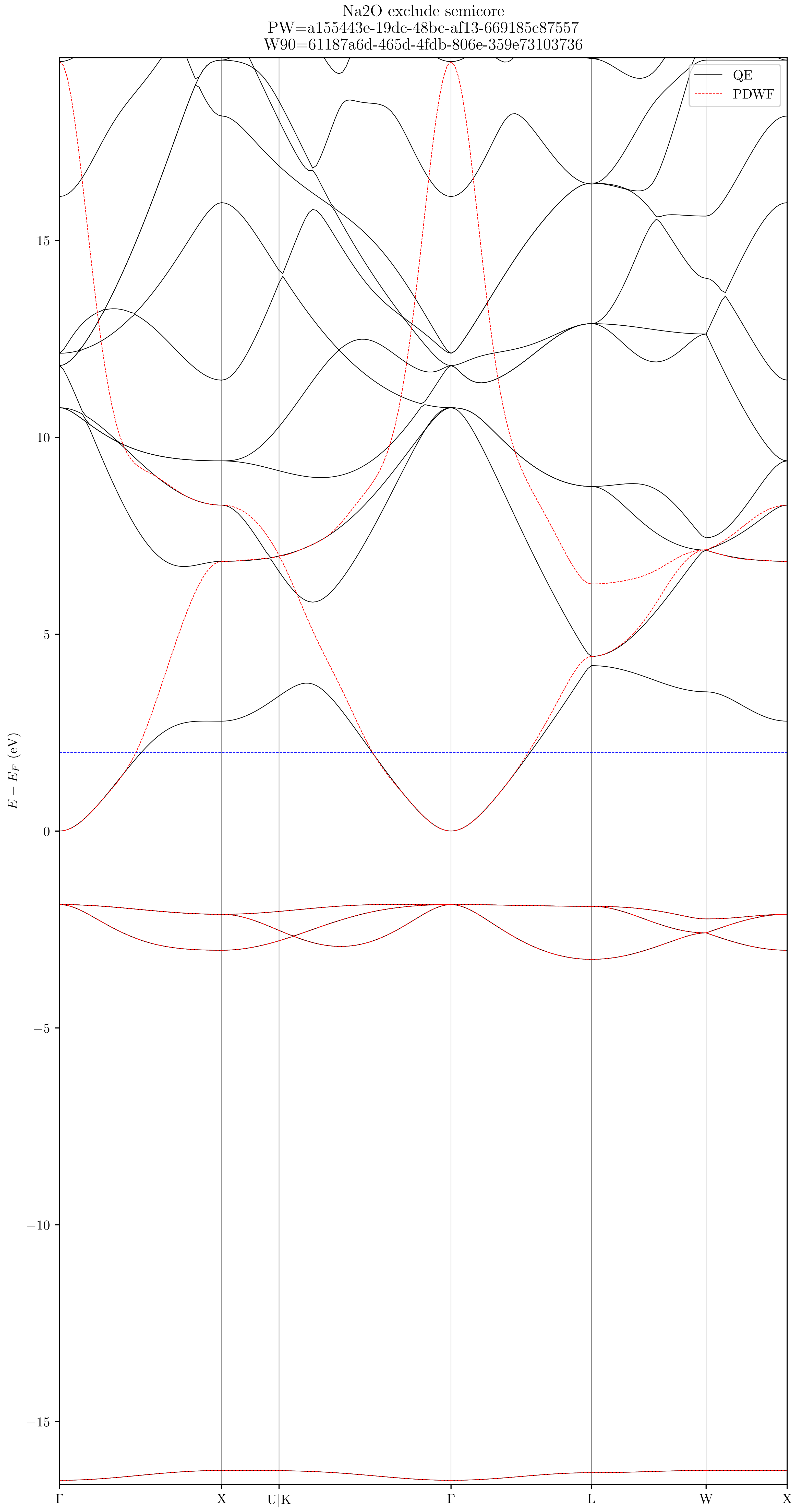

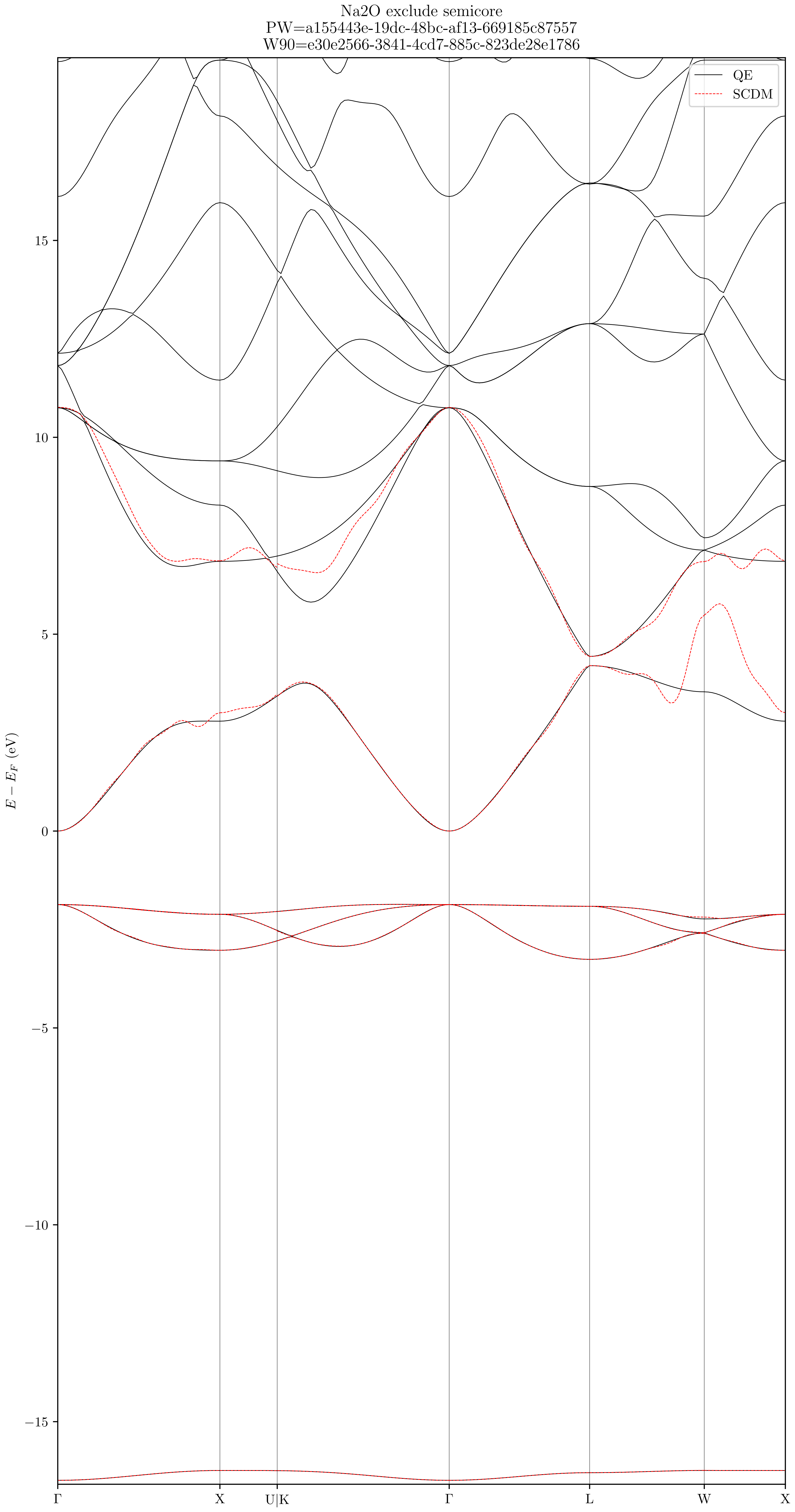

PDWF band distance (meV) and τ ($\AA$)

η_0	η_2	η_4	$\eta_0^{\max}$	$\eta_2^{\max}$	$\eta_4^{\max}$	τ
0.3	0.8	42.5	1.4	5.2	524.5	2.105

SCDM band distance (meV) and τ ($\AA$)

η_0	η_2	η_4	$\eta_0^{\max}$	$\eta_2^{\max}$	$\eta_4^{\max}$	τ
5.6	6.1	23.8	46.1	46.1	121.5	2.785

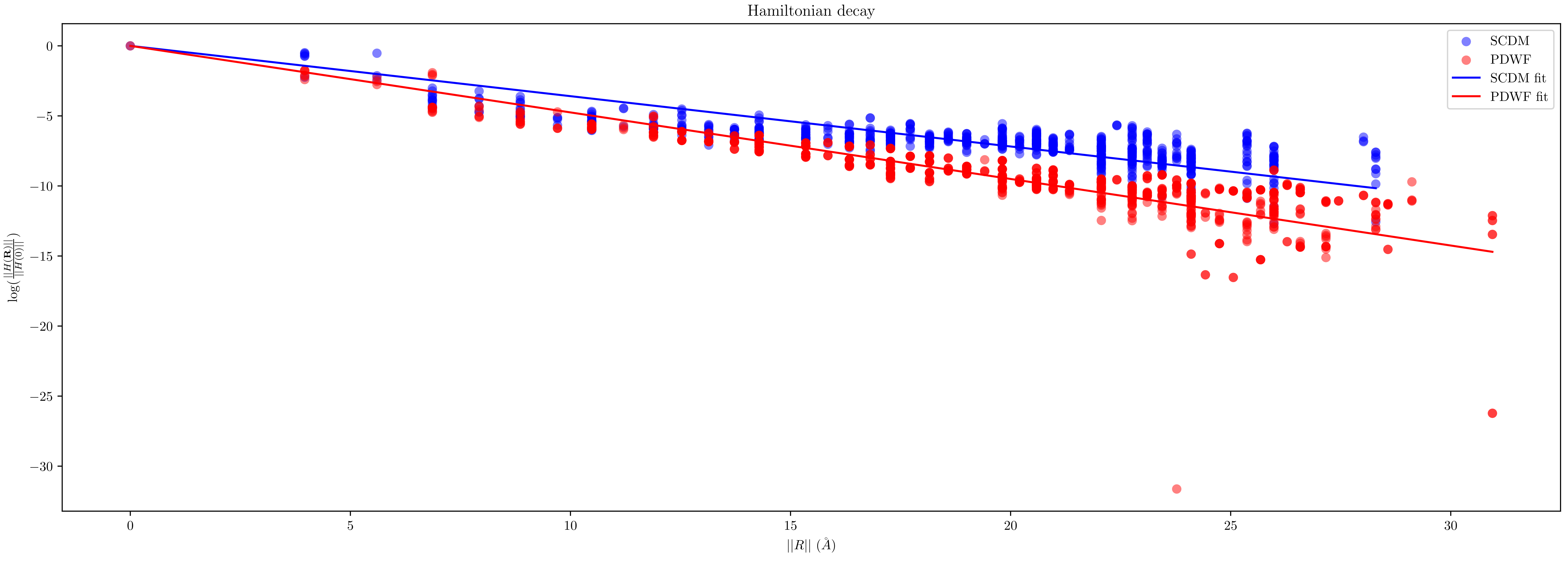

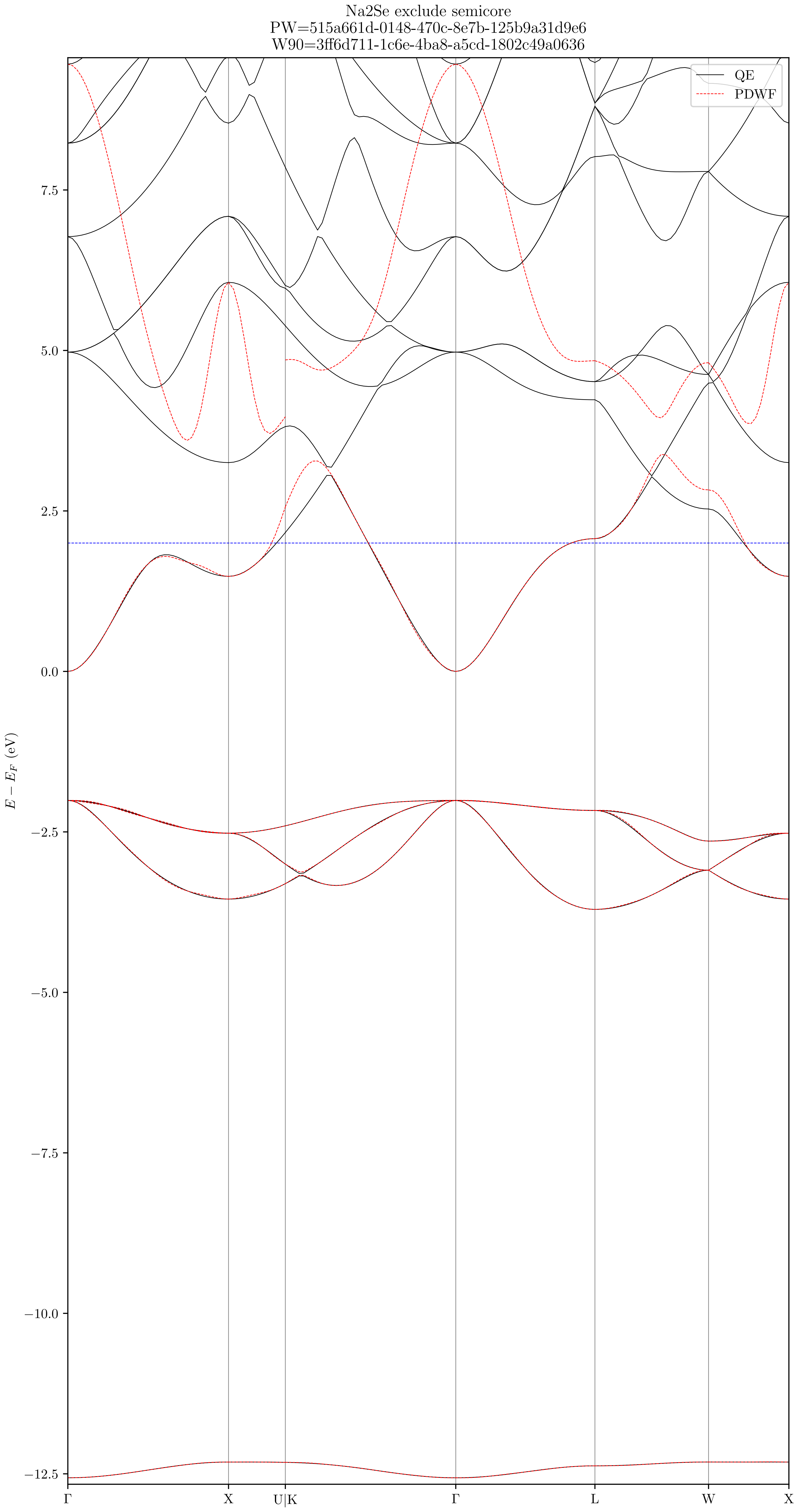

PDWF band distance (meV) and τ ($\AA$)

η_0	η_2	η_4	$\eta_0^{\max}$	$\eta_2^{\max}$	$\eta_4^{\max}$	τ
5.6	6.0	36.5	25.9	29.9	318.0	3.135

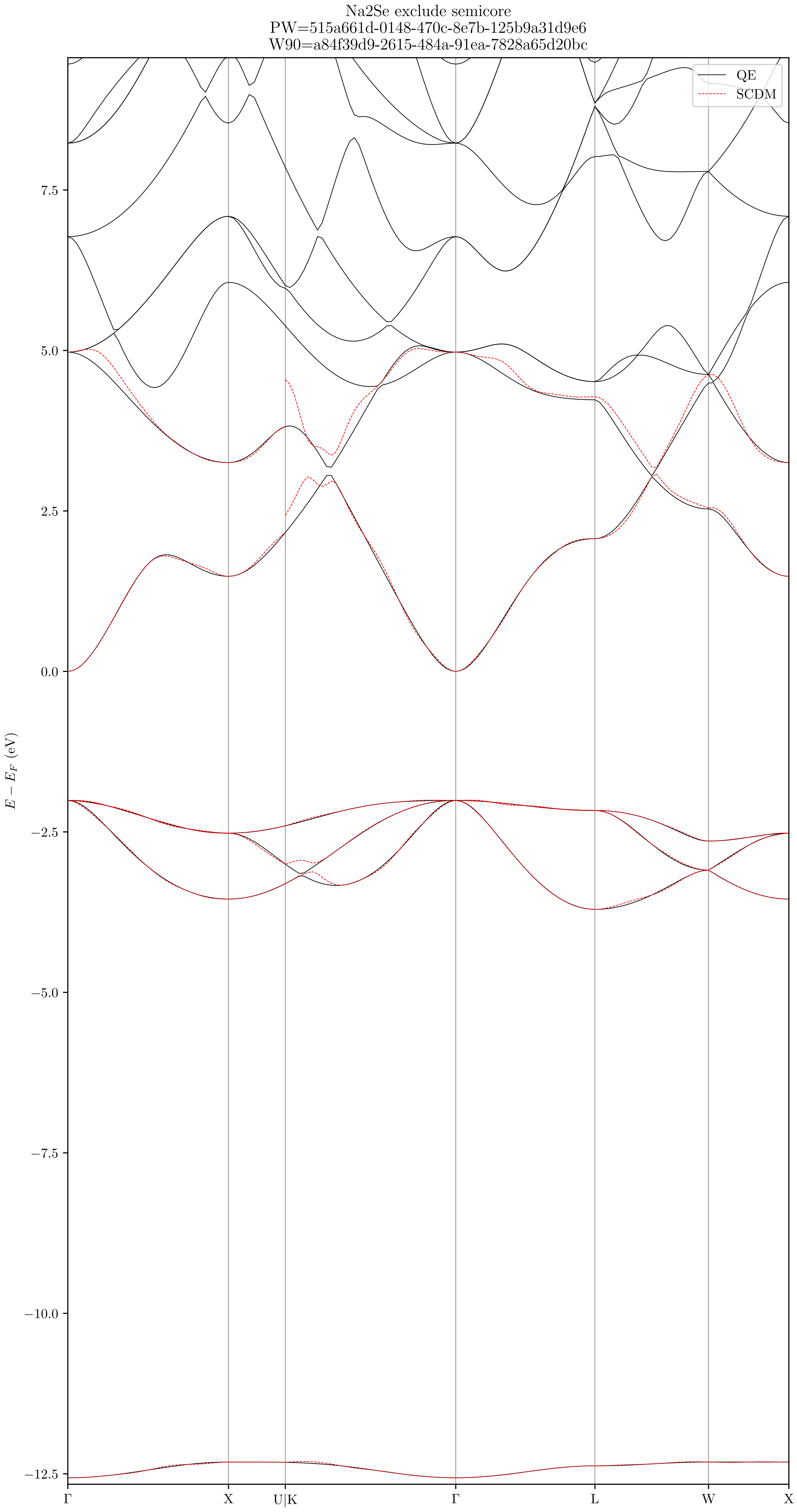

SCDM band distance (meV) and τ ($\AA$)

η_0	η_2	η_4	$\eta_0^{\max}$	$\eta_2^{\max}$	$\eta_4^{\max}$	τ
19.1	18.9	28.7	204.2	204.2	248.4	3.287

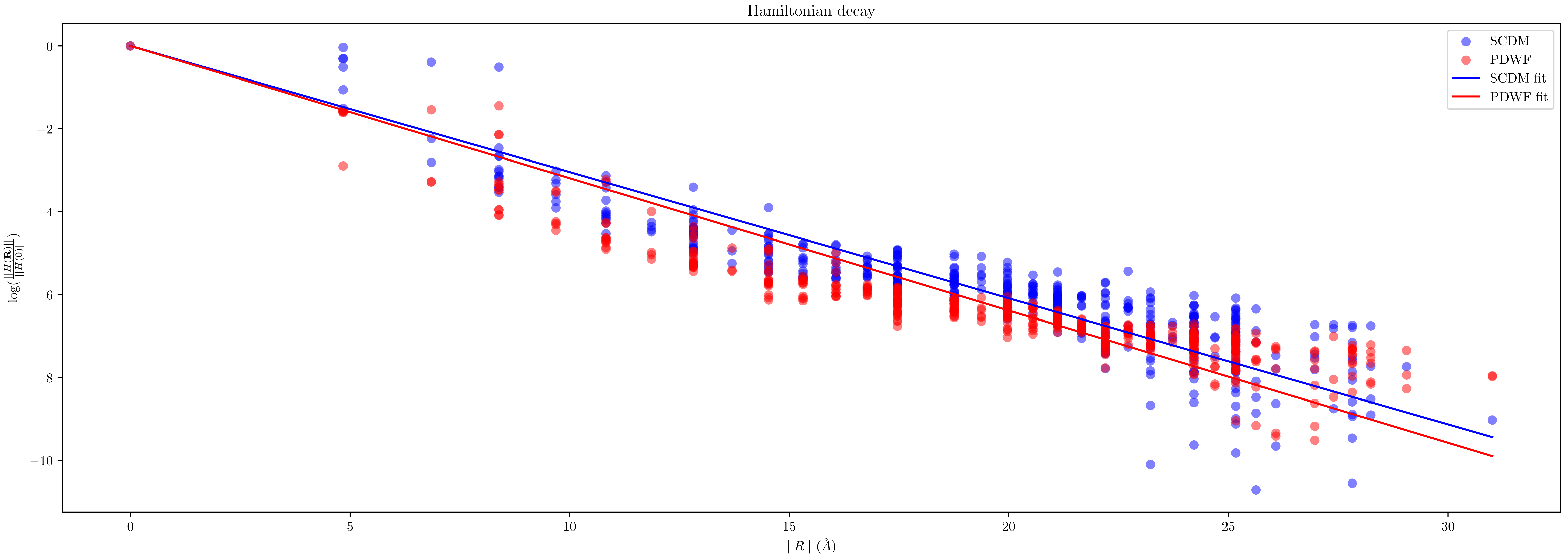

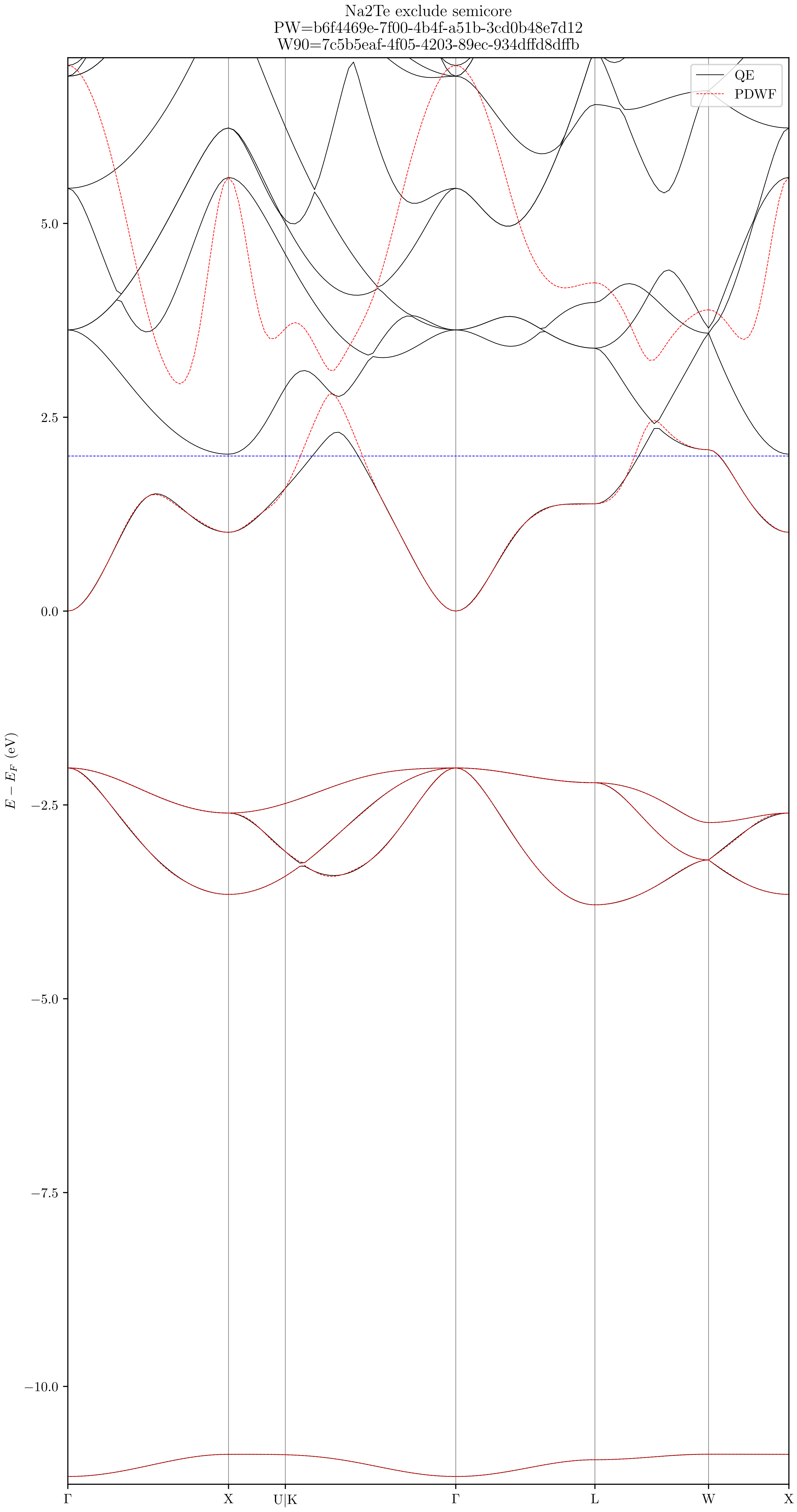

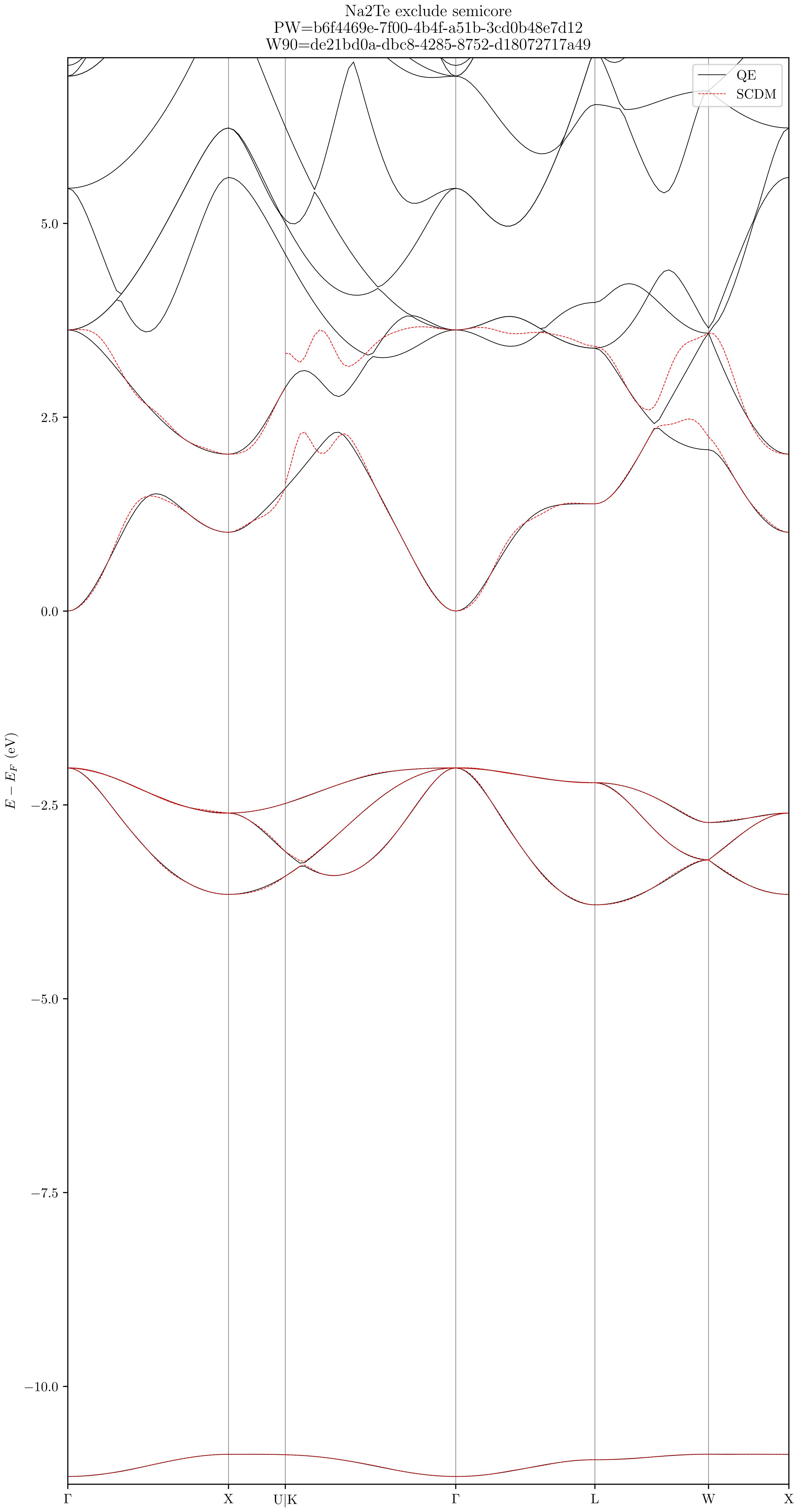

PDWF band distance (meV) and τ ($\AA$)

η_0	η_2	η_4	$\eta_0^{\max}$	$\eta_2^{\max}$	$\eta_4^{\max}$	τ
3.2	3.4	77.1	15.1	15.1	471.7	3.064

SCDM band distance (meV) and τ ($\AA$)

η_0	η_2	η_4	$\eta_0^{\max}$	$\eta_2^{\max}$	$\eta_4^{\max}$	τ
5.0	7.2	51.4	30.9	40.1	477.1	3.435

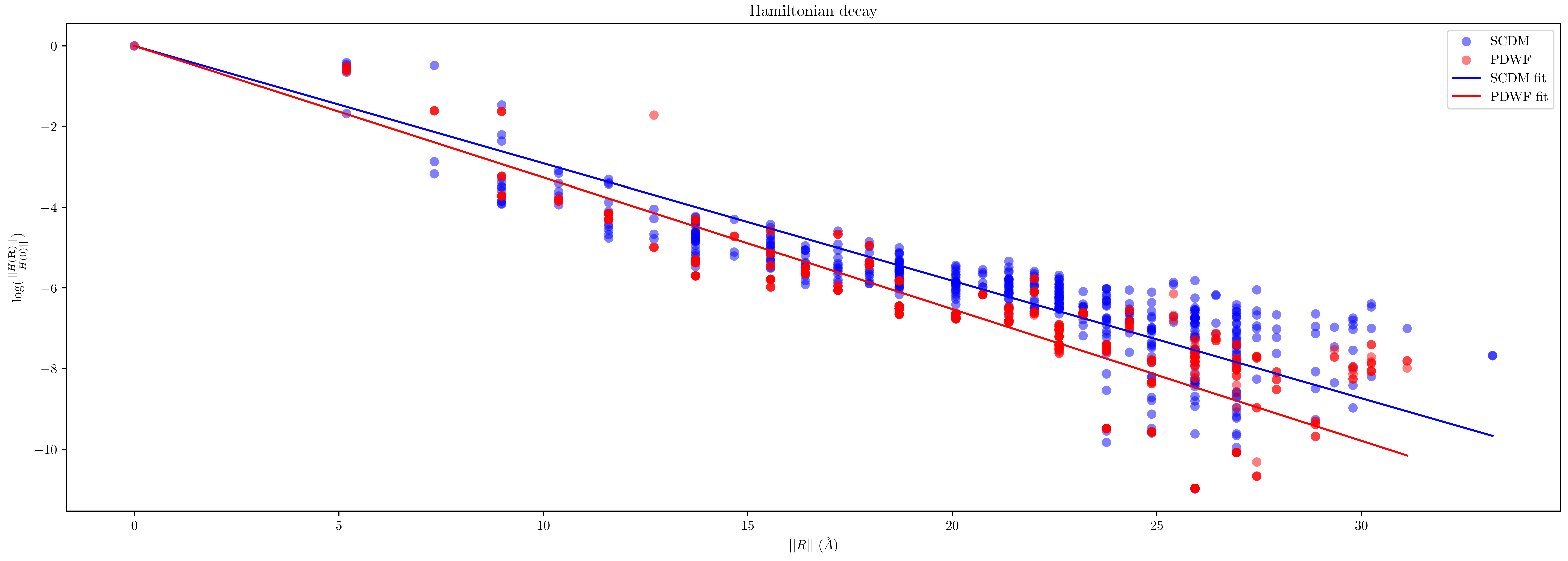

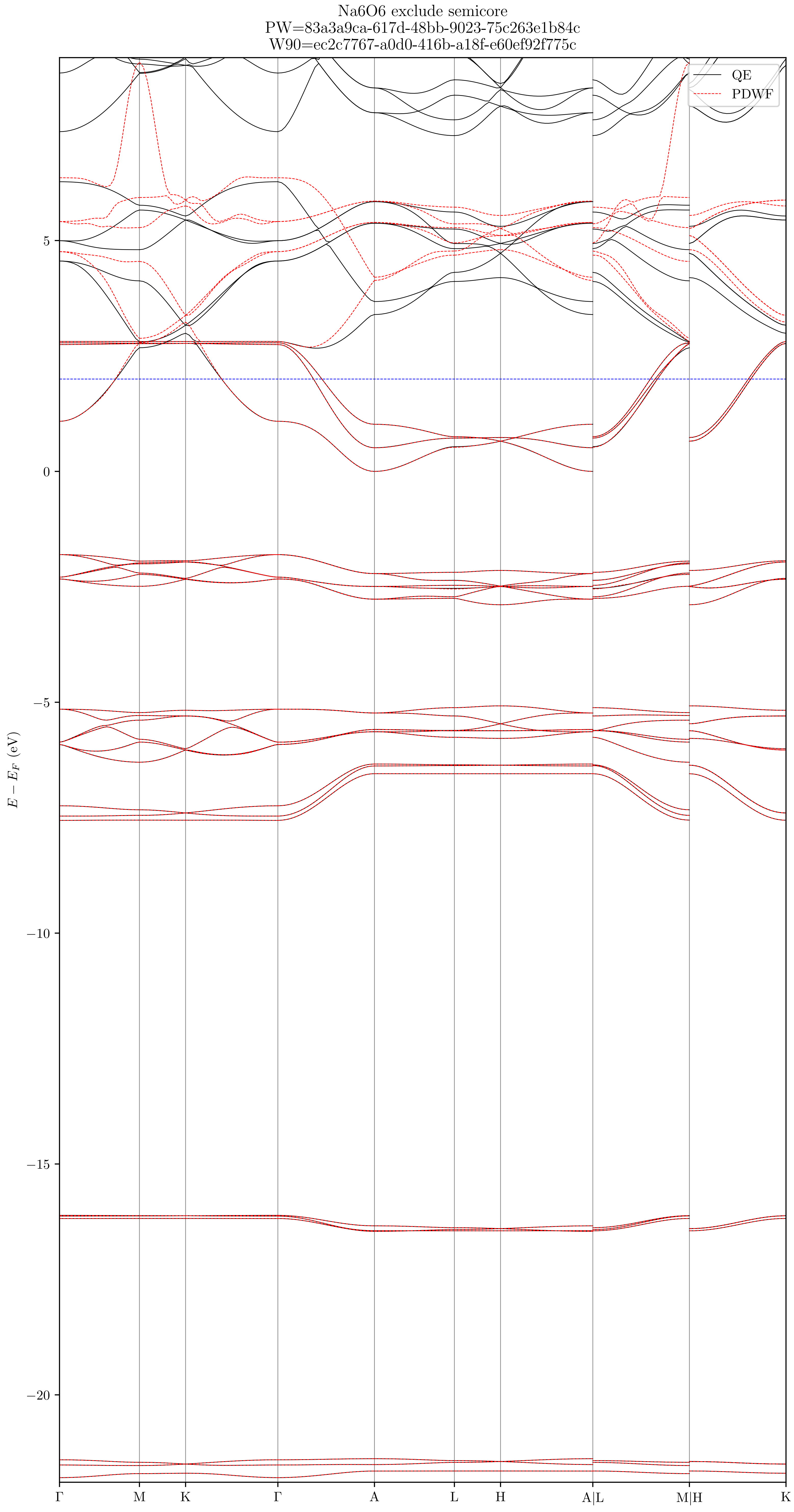

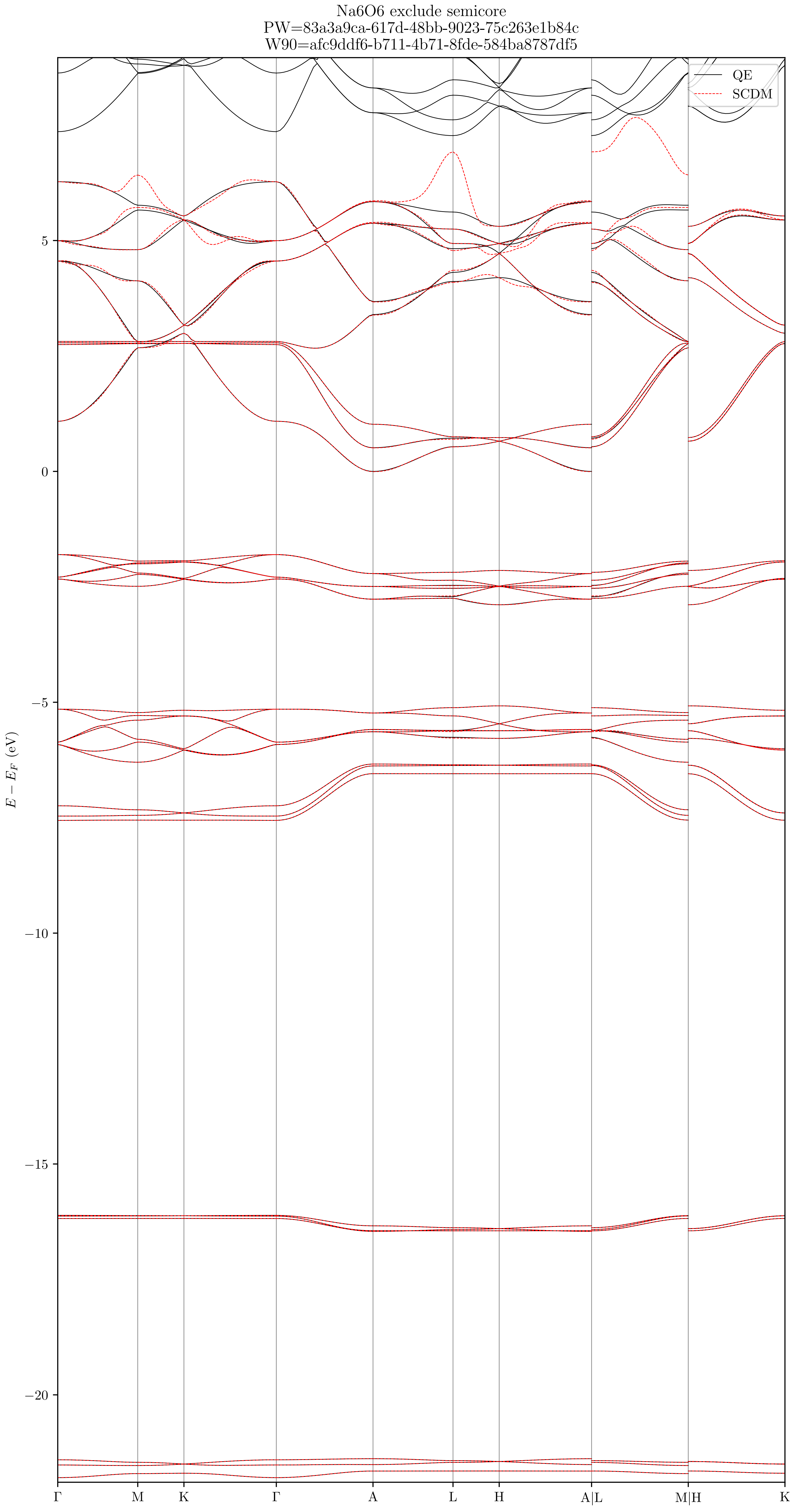

PDWF band distance (meV) and τ ($\AA$)

η_0	η_2	η_4	$\eta_0^{\max}$	$\eta_2^{\max}$	$\eta_4^{\max}$	τ
1.1	1.3	11.4	13.3	13.3	134.2	2.650

SCDM band distance (meV) and τ ($\AA$)

η_0	η_2	η_4	$\eta_0^{\max}$	$\eta_2^{\max}$	$\eta_4^{\max}$	τ
1.9	2.3	2.9	22.9	22.9	35.3	2.787

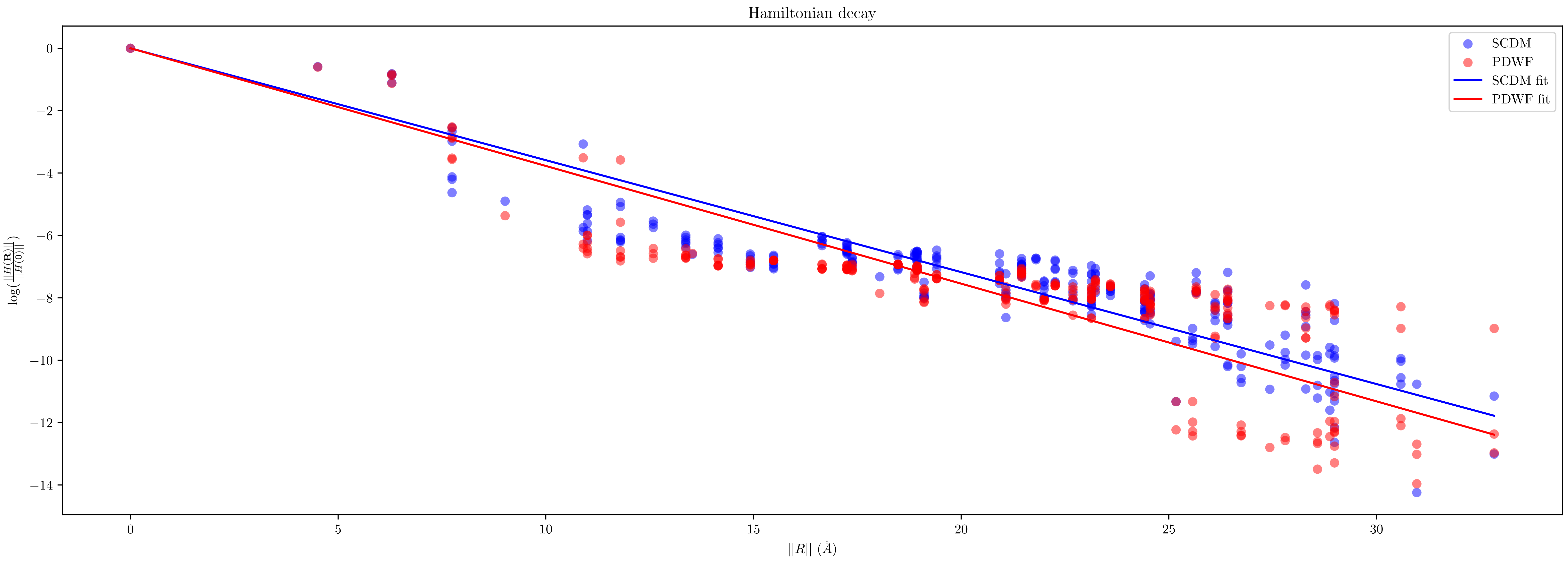

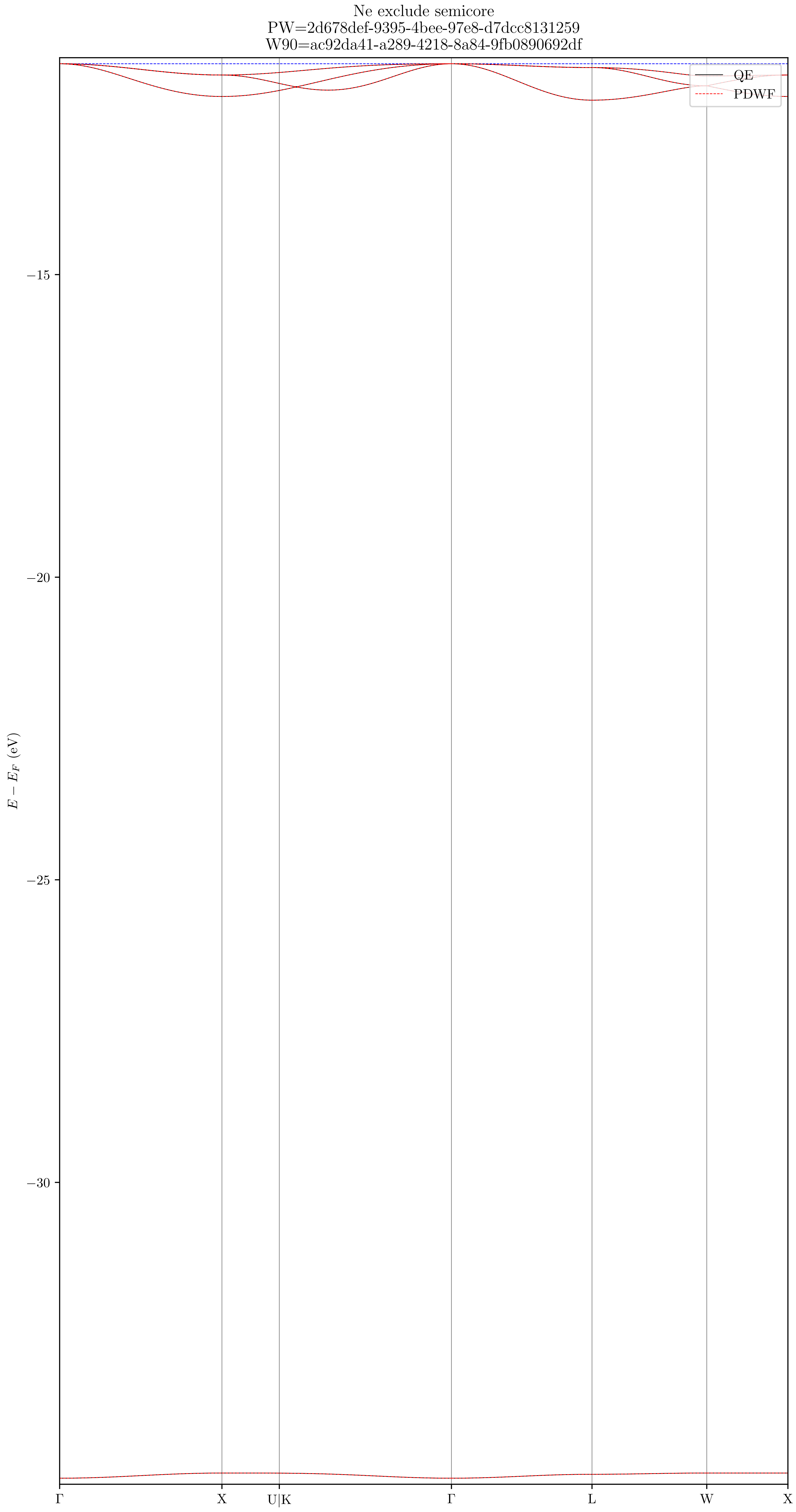

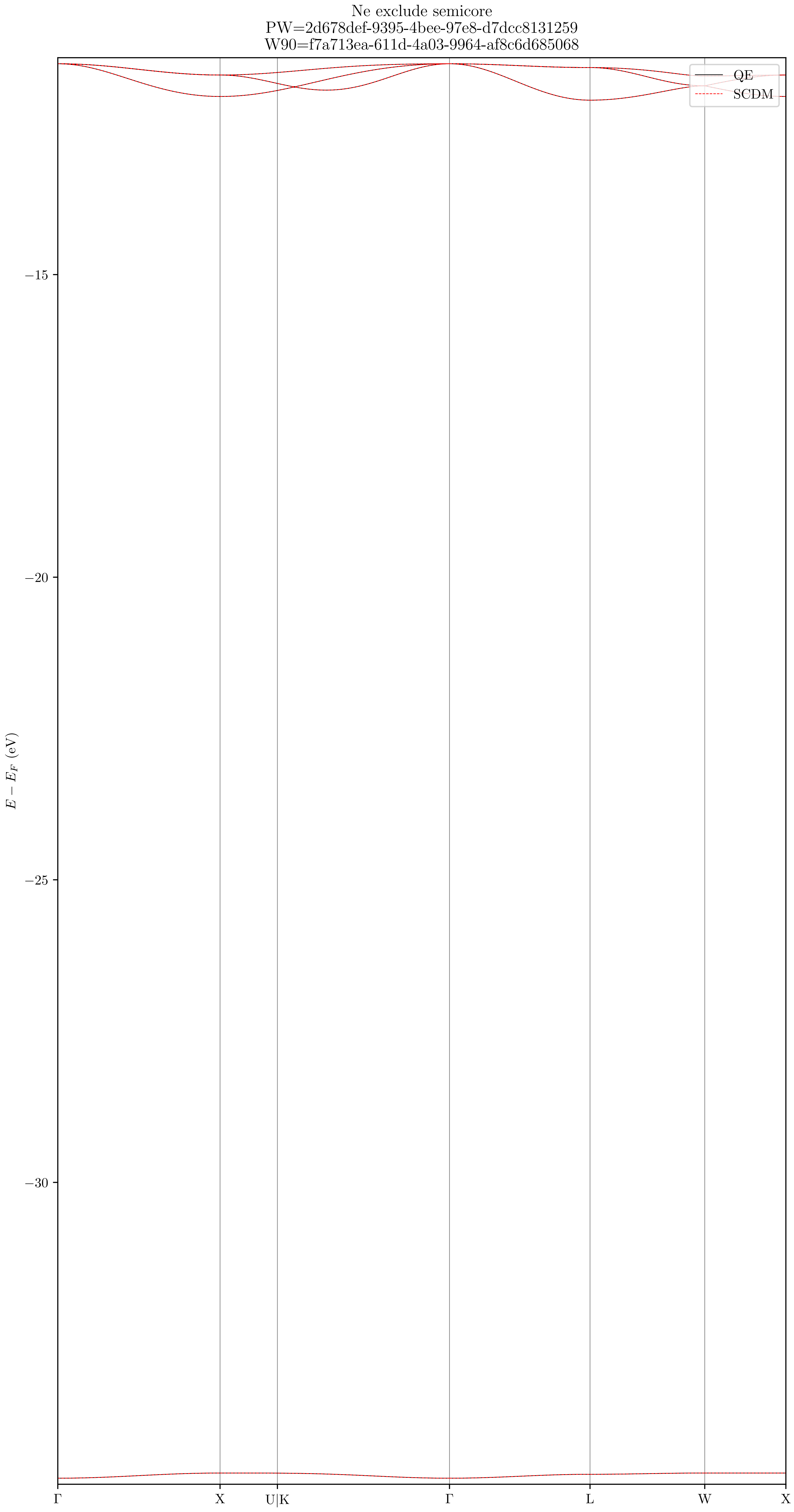

PDWF band distance (meV) and τ ($\AA$)

η_0	η_2	η_4	$\eta_0^{\max}$	$\eta_2^{\max}$	$\eta_4^{\max}$	τ
0.0	0.0	0.0	0.0	0.0	0.0	0.988

SCDM band distance (meV) and τ ($\AA$)

η_0	η_2	η_4	$\eta_0^{\max}$	$\eta_2^{\max}$	$\eta_4^{\max}$	τ
0.0	0.0	0.0	0.0	0.0	0.0	1.116

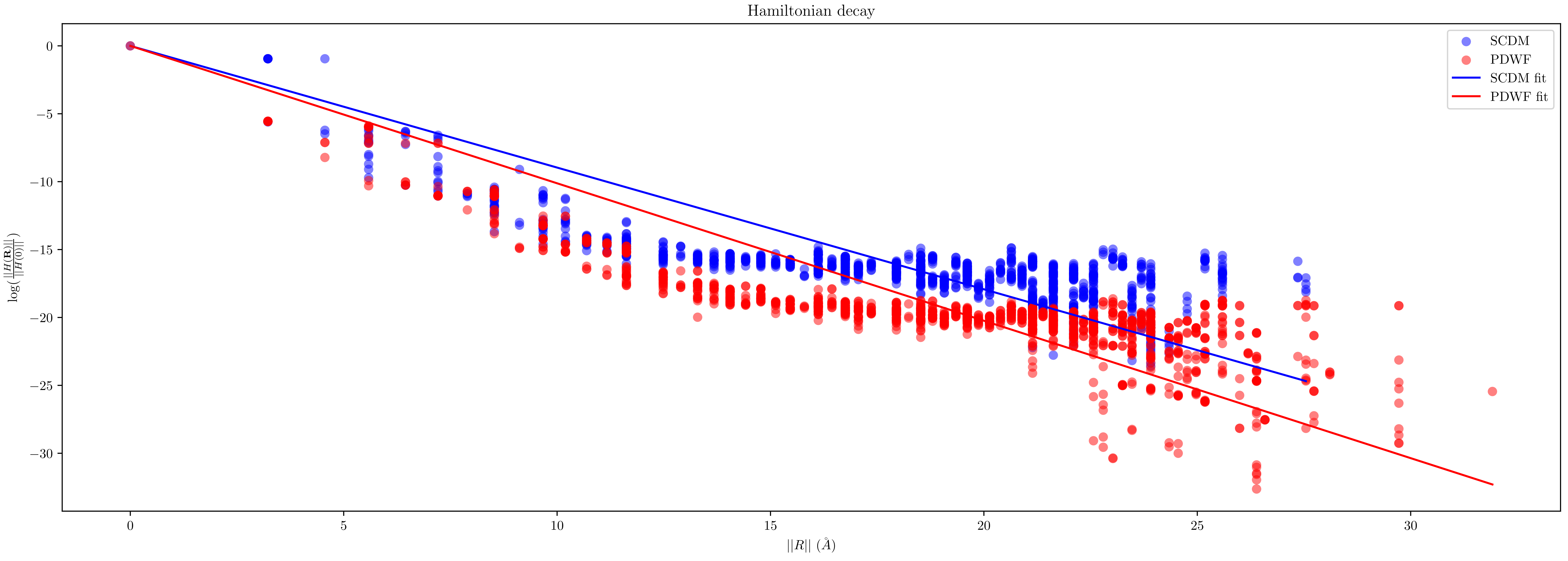

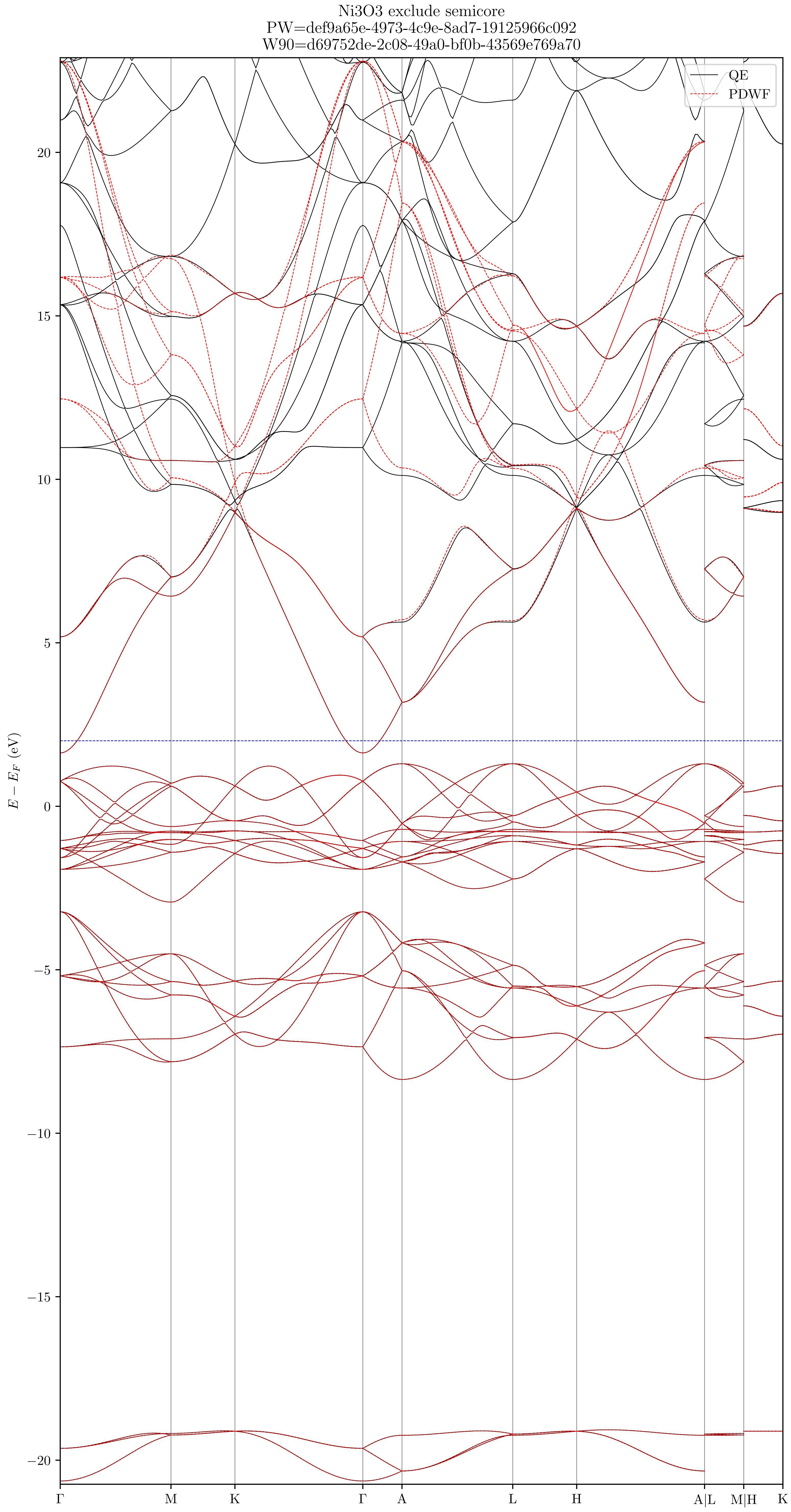

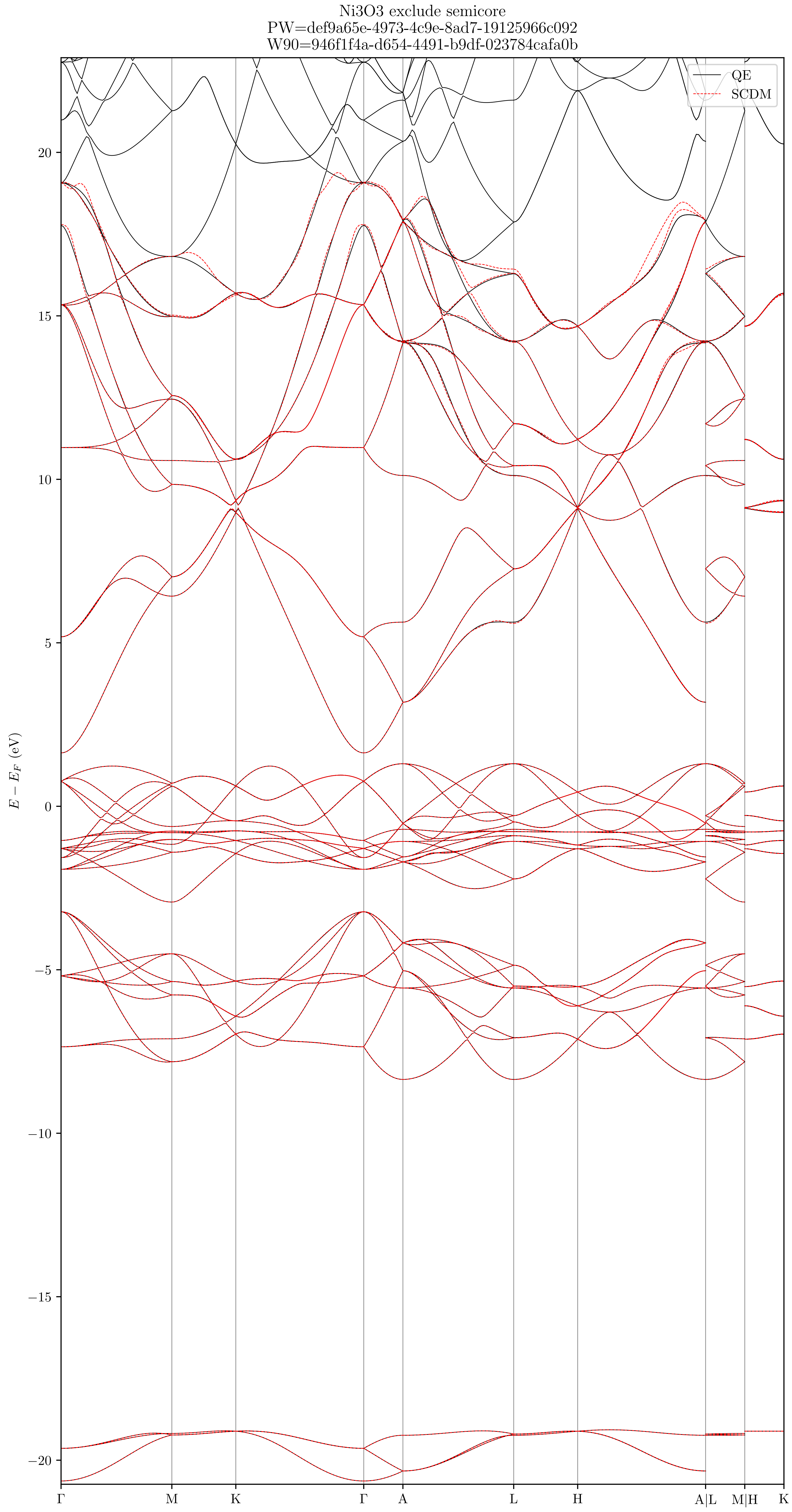

PDWF band distance (meV) and τ ($\AA$)

η_0	η_2	η_4	$\eta_0^{\max}$	$\eta_2^{\max}$	$\eta_4^{\max}$	τ
0.6	0.6	0.7	6.9	6.9	6.9	2.172

SCDM band distance (meV) and τ ($\AA$)

η_0	η_2	η_4	$\eta_0^{\max}$	$\eta_2^{\max}$	$\eta_4^{\max}$	τ
1.9	1.9	1.9	19.8	19.8	19.8	2.475

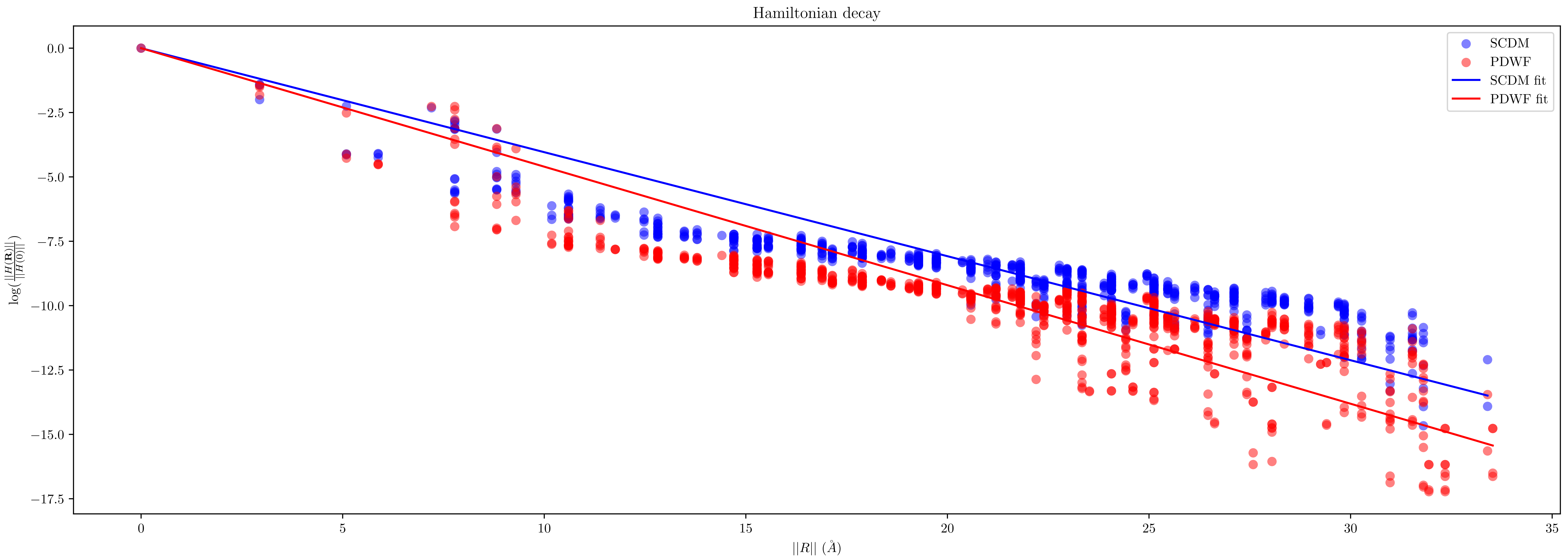

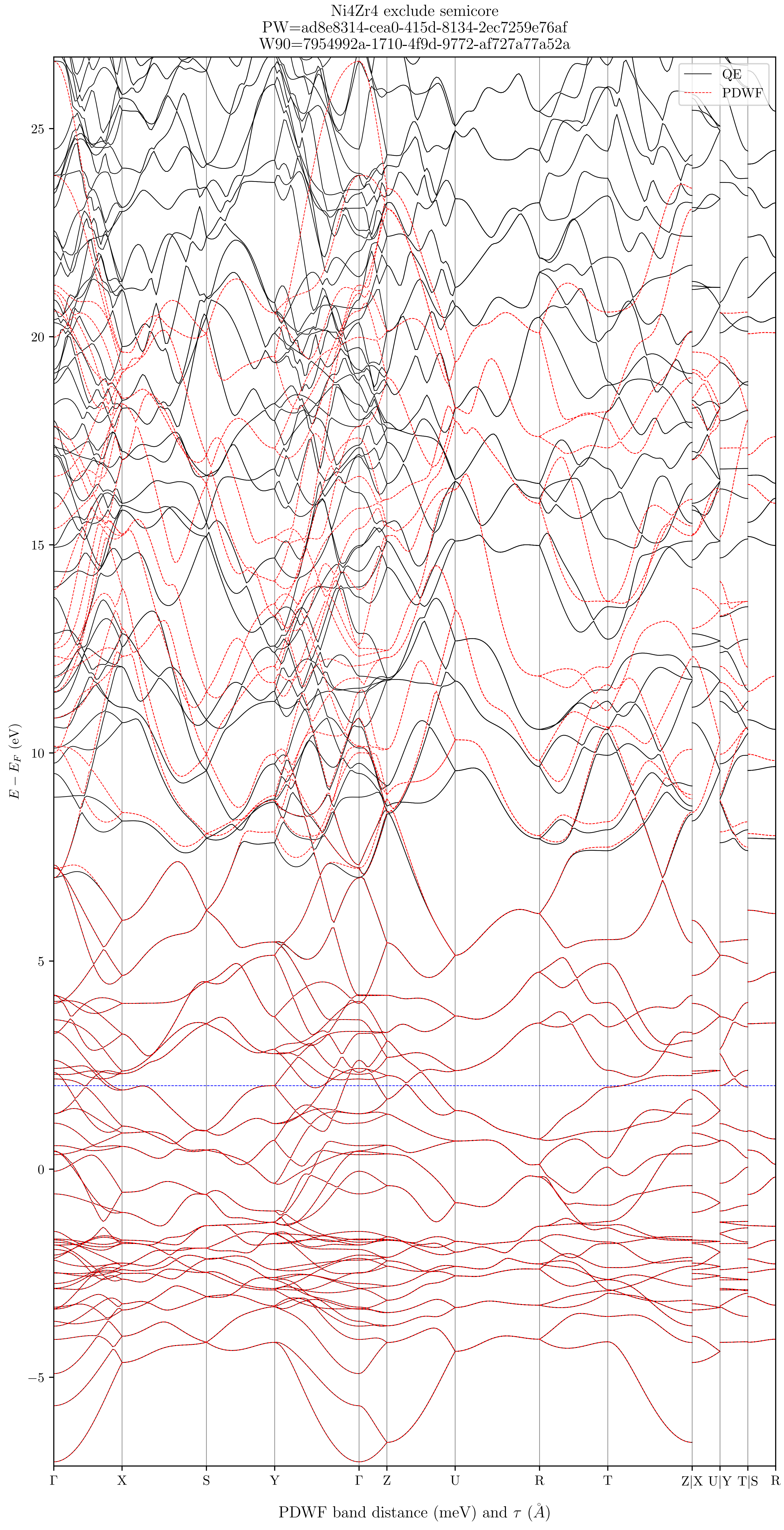

η_0	η_2	η_4	$\eta_0^{\max}$	$\eta_2^{\max}$	$\eta_4^{\max}$	τ
0.4	0.5	0.6	2.7	8.2	11.0	2.256

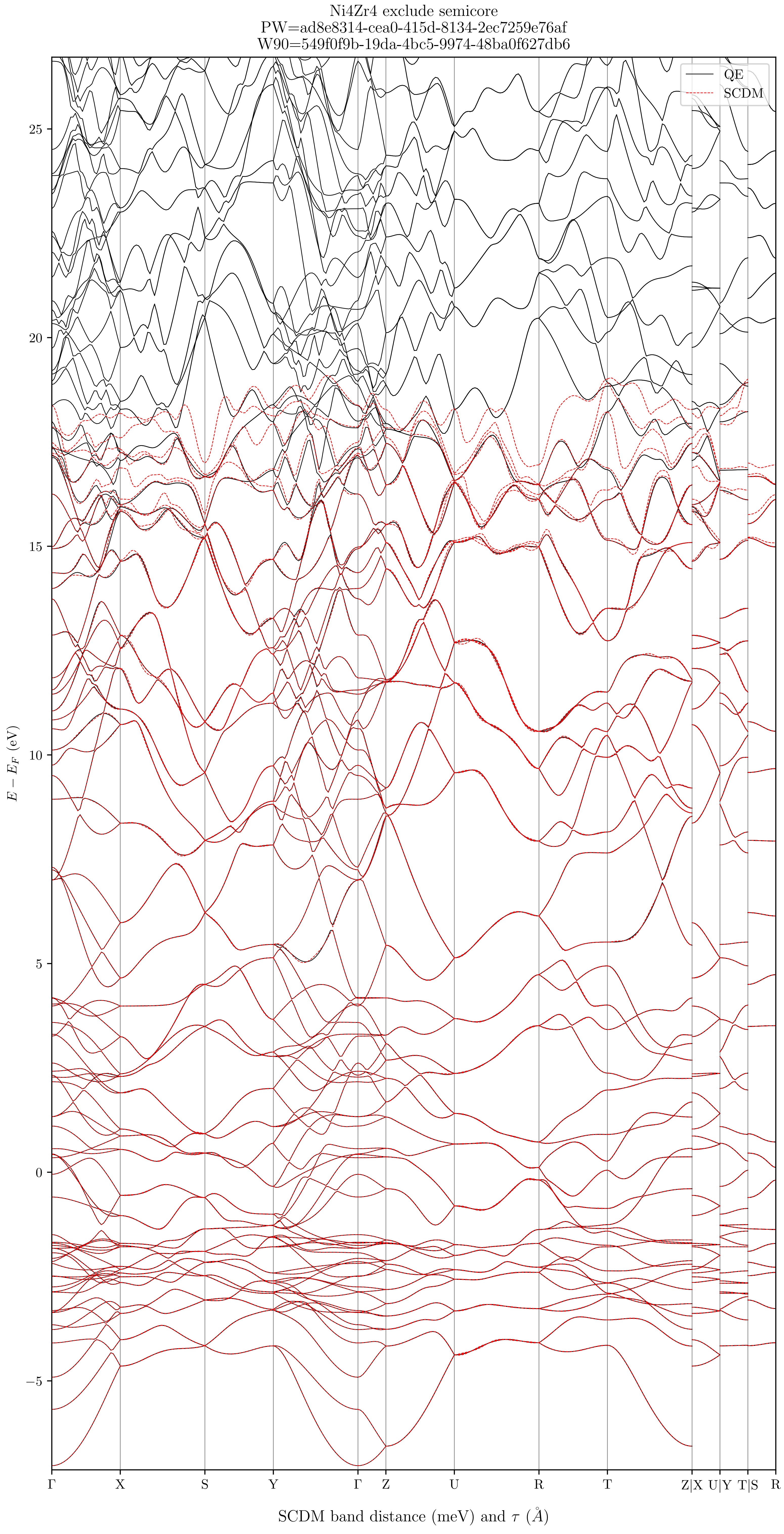

η_0	η_2	η_4	$\eta_0^{\max}$	$\eta_2^{\max}$	$\eta_4^{\max}$	τ
2.2	2.4	2.6	19.8	19.8	19.8	2.819

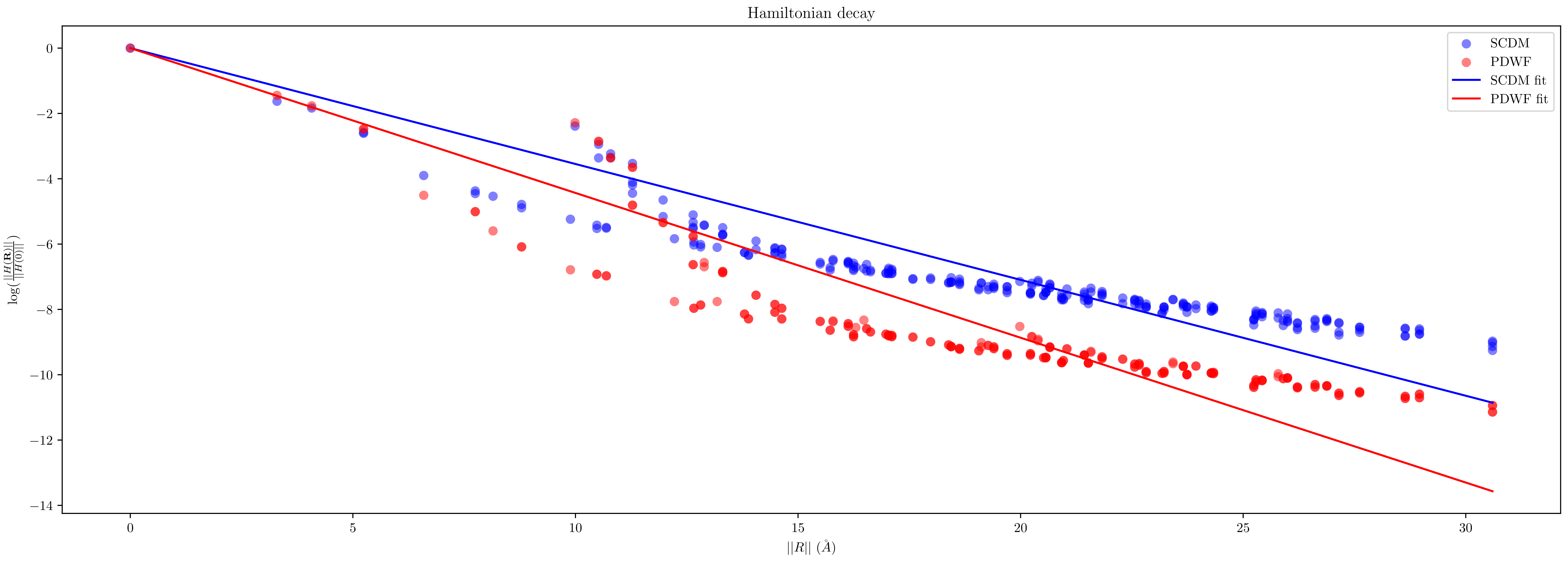

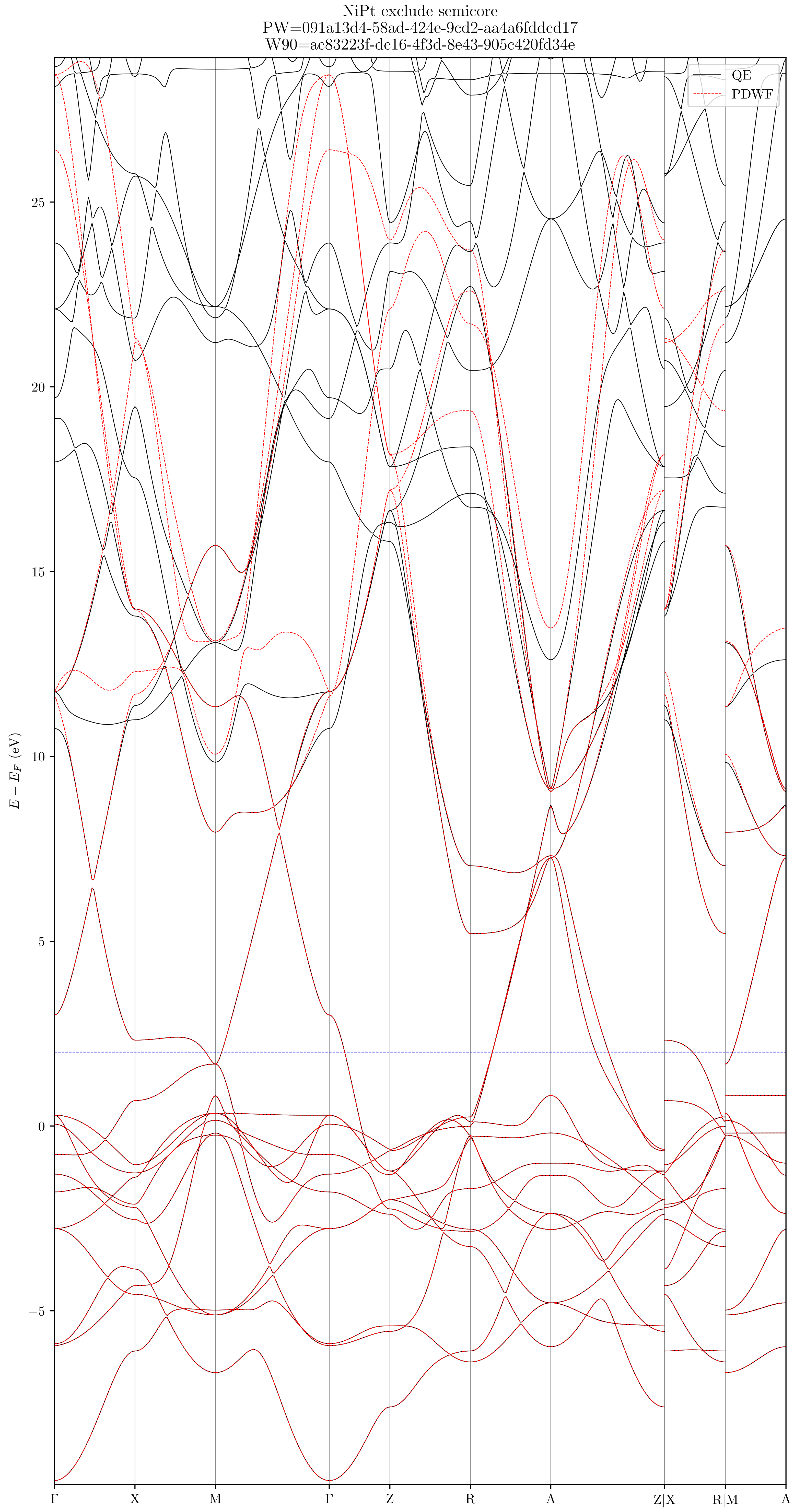

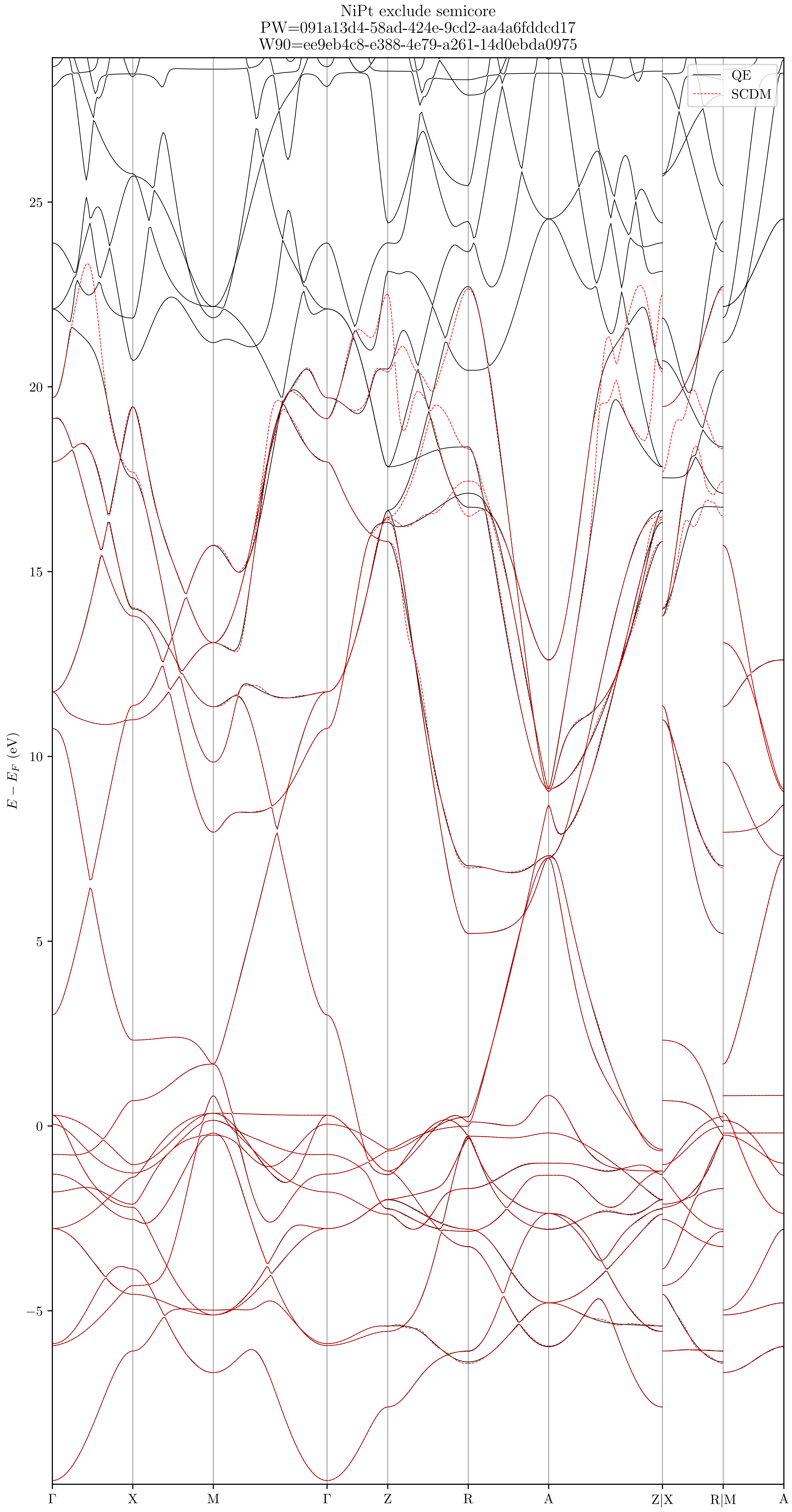

PDWF band distance (meV) and τ ($\AA$)

η_0	η_2	η_4	η_0^{max}	η_2^{max}	η_4^{max}	τ
0.9	0.9	0.9	6.6	6.6	7.9	1.623

SCDM band distance (meV) and τ ($\AA$)

η_0	η_2	η_4	η_0^{max}	η_2^{max}	η_4^{max}	τ
8.5	8.1	8.3	56.6	56.6	61.0	2.336

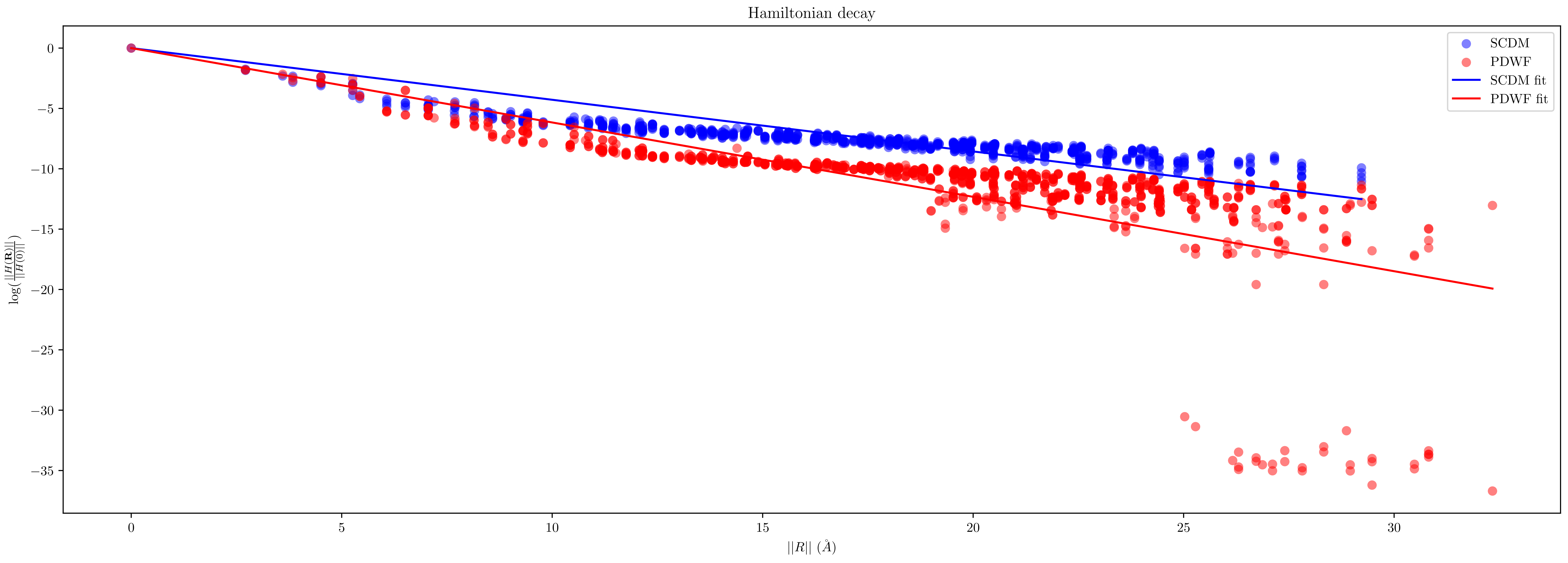

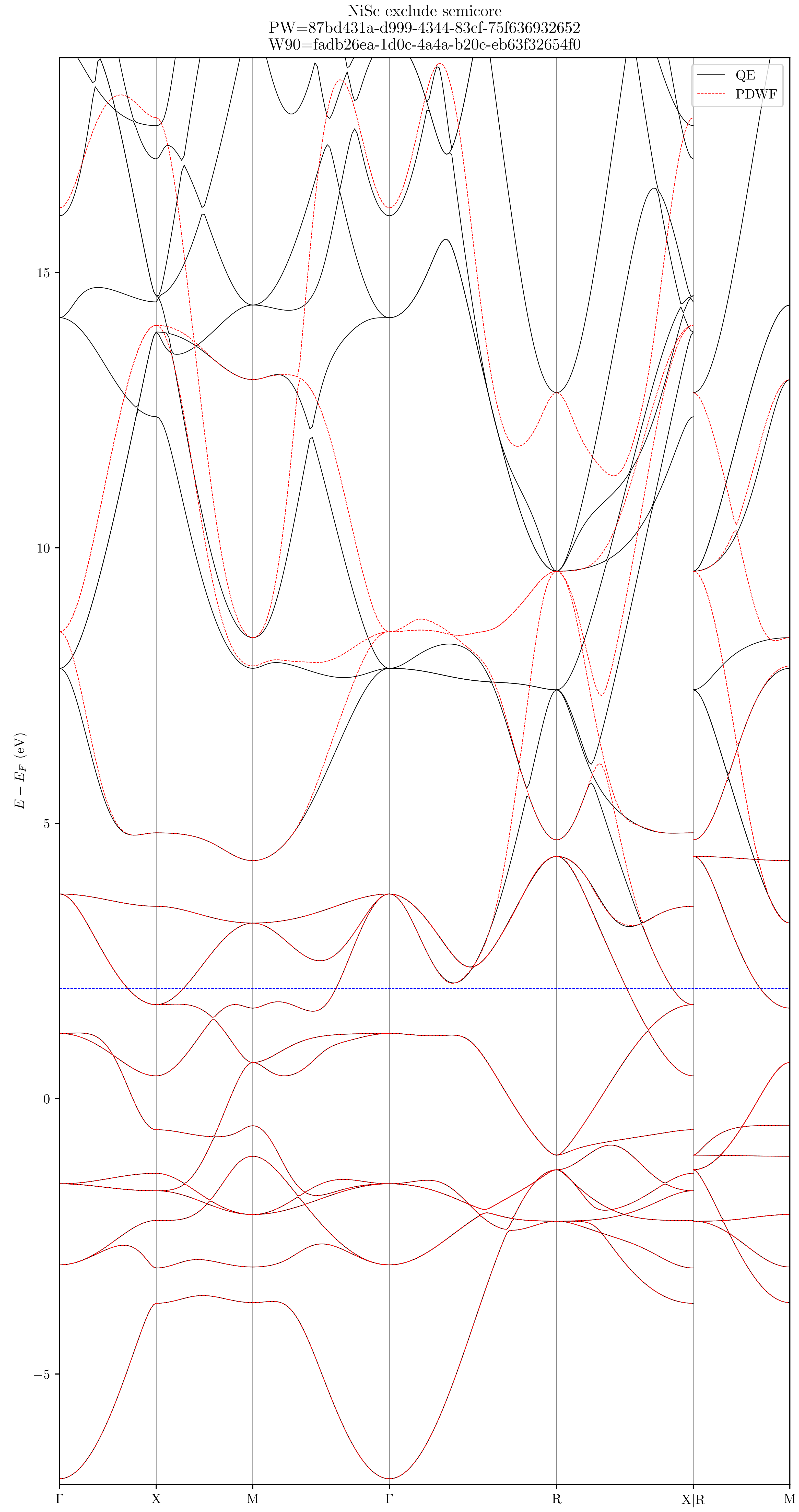

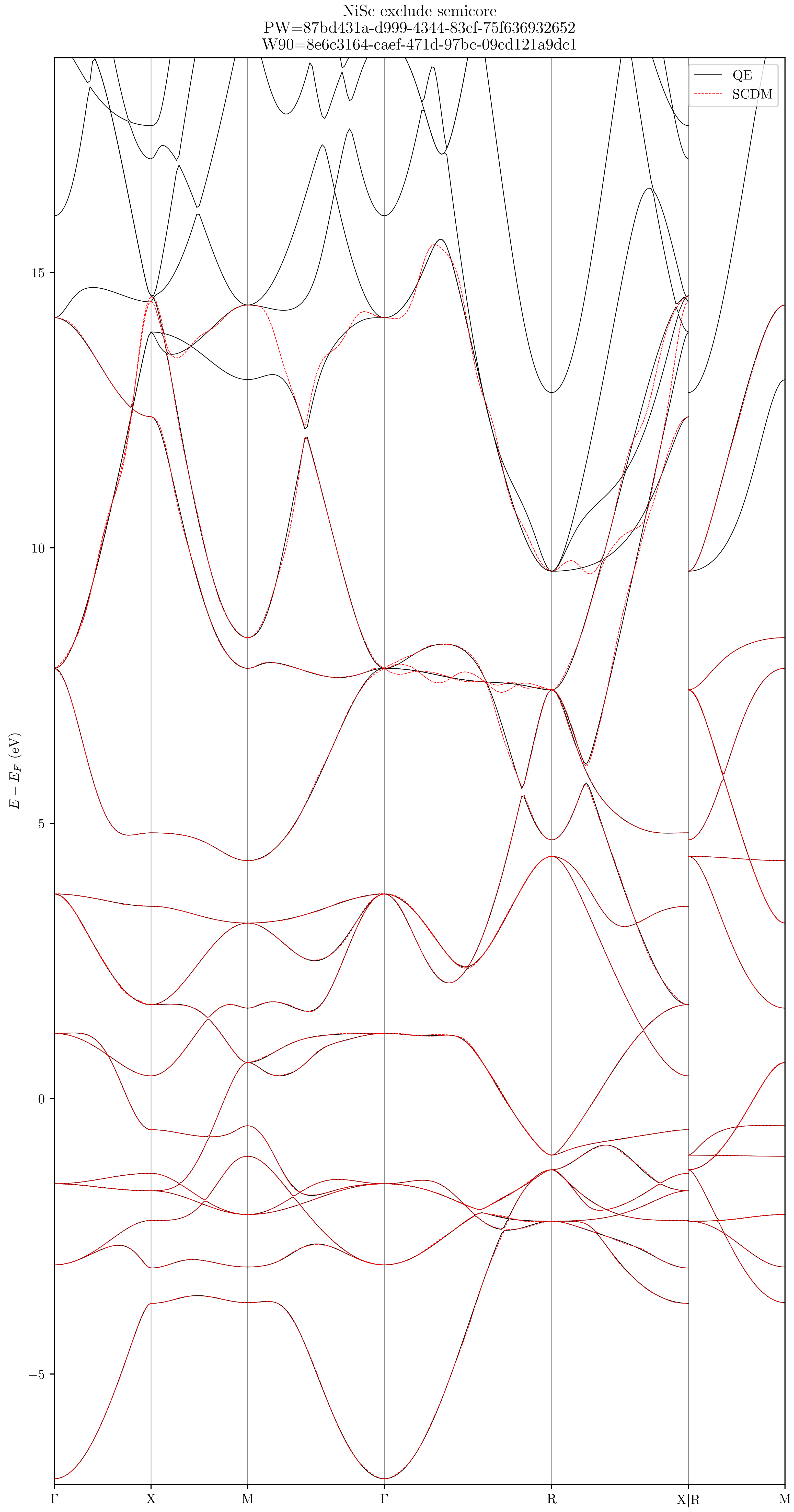

PDWF band distance (meV) and τ ($\AA$)

η_0	η_2	η_4	$\eta_0^{\max}$	$\eta_2^{\max}$	$\eta_4^{\max}$	τ
0.7	0.9	9.9	7.1	7.1	175.9	1.668

SCDM band distance (meV) and τ ($\AA$)

η_0	η_2	η_4	$\eta_0^{\max}$	$\eta_2^{\max}$	$\eta_4^{\max}$	τ
5.7	6.0	6.5	49.5	49.5	49.5	2.320

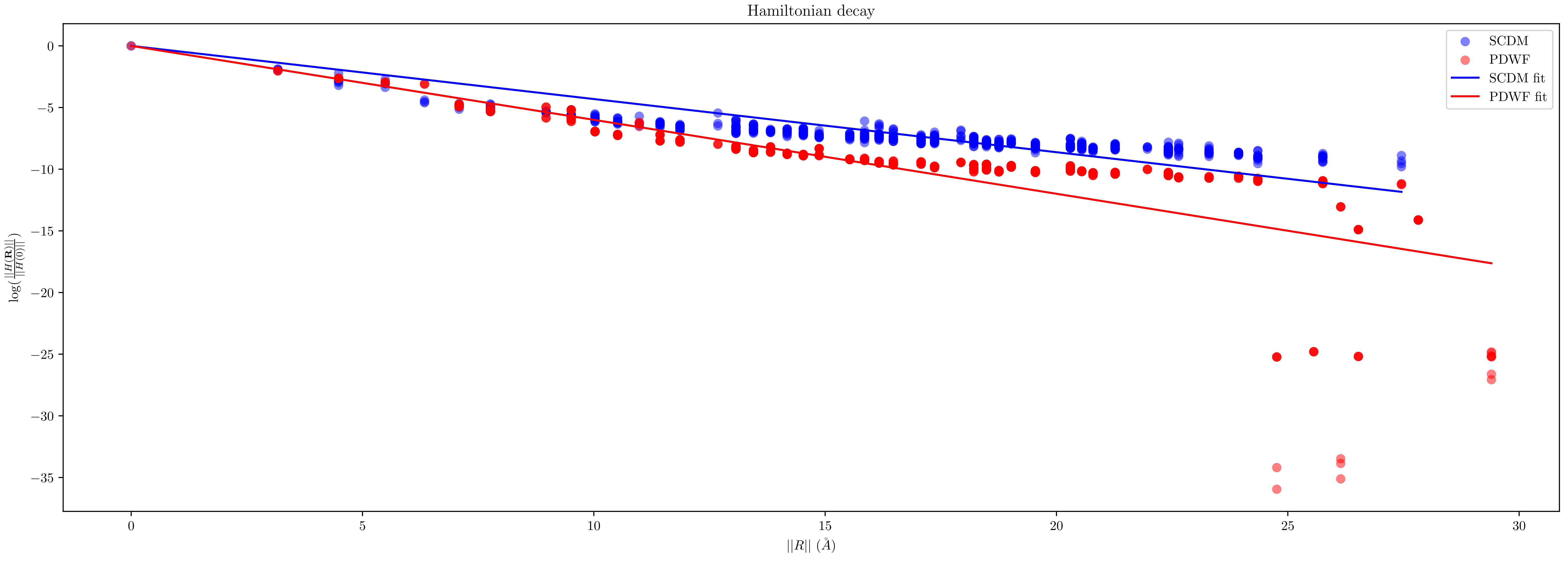

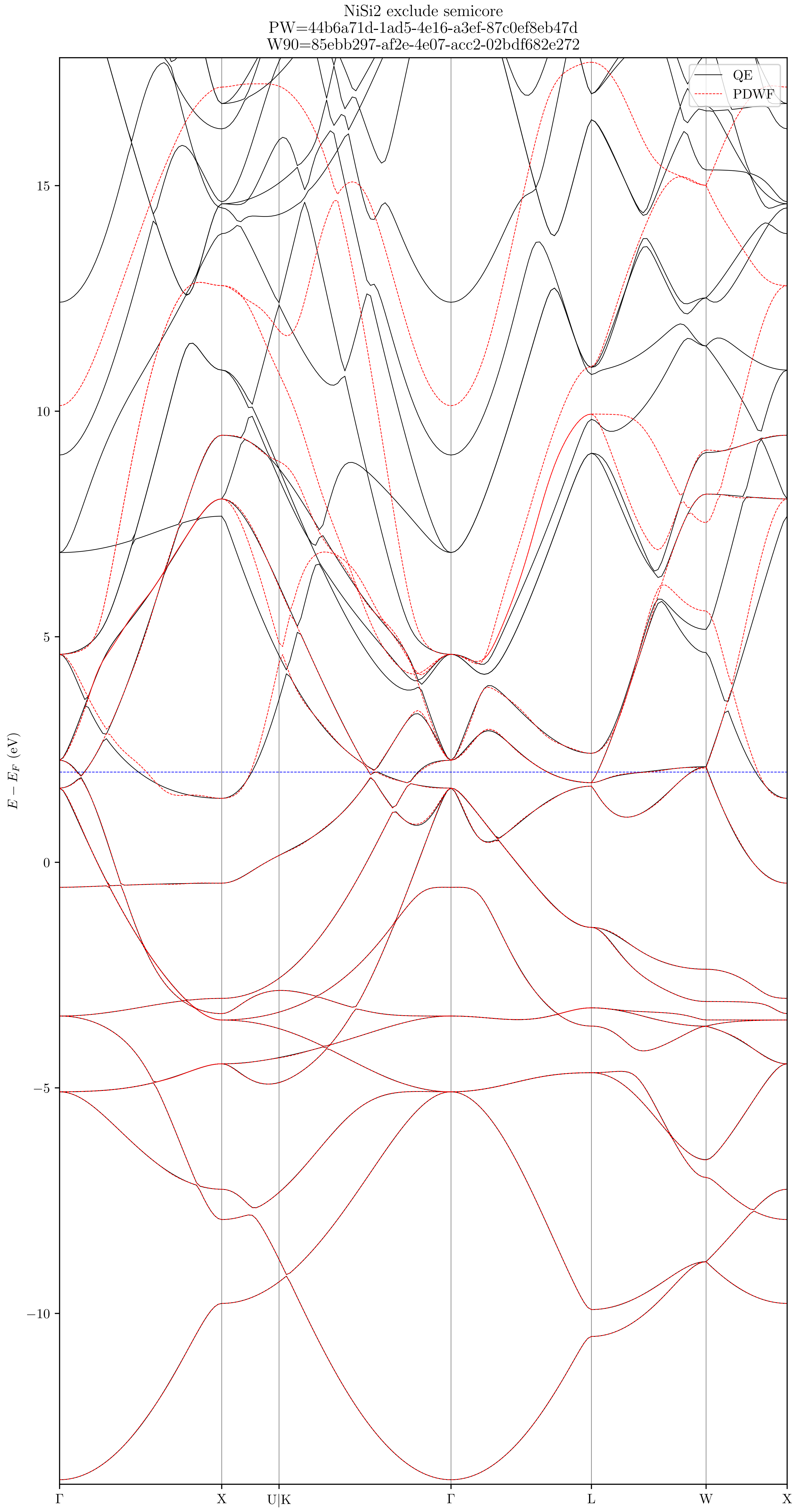

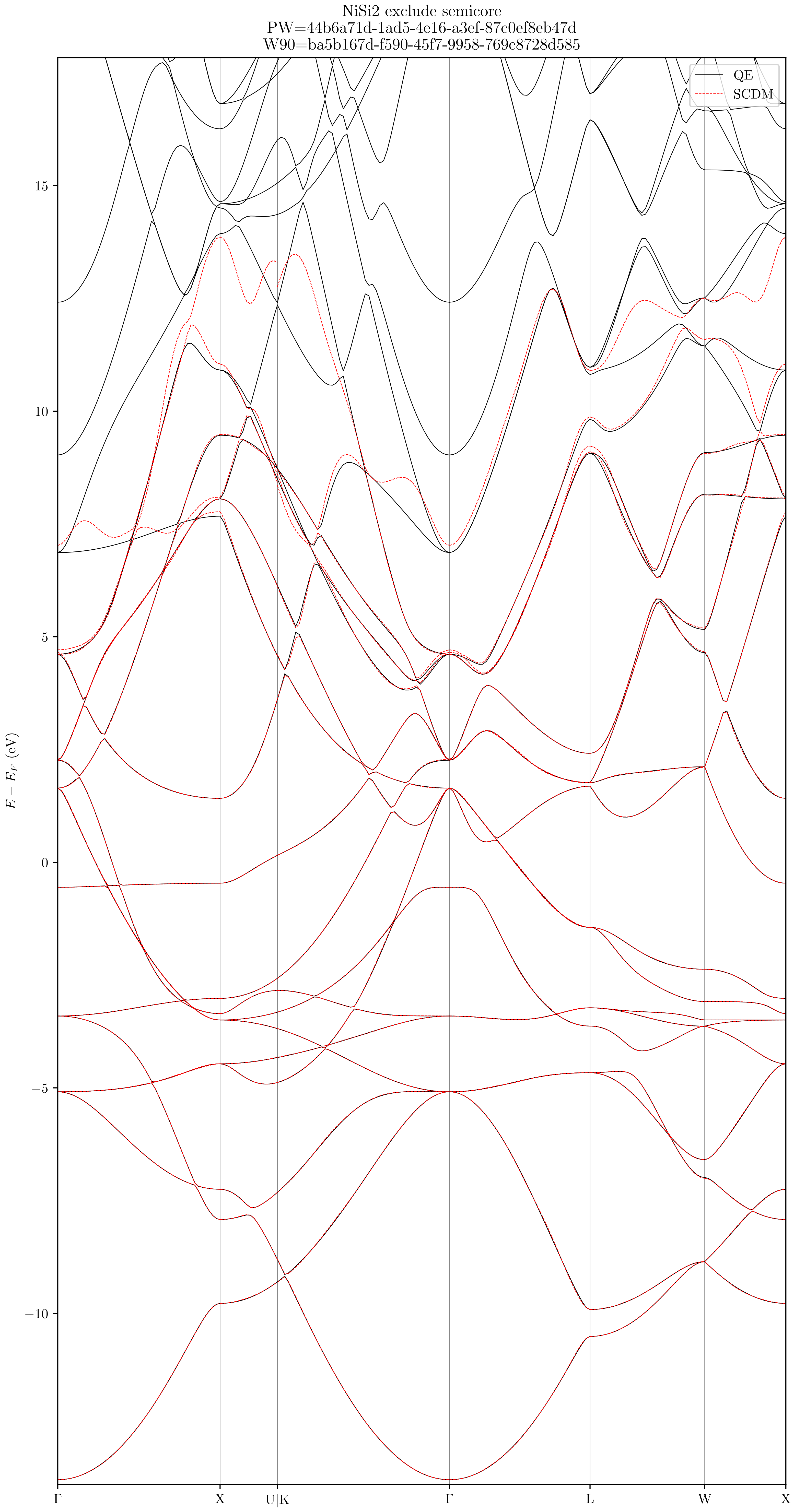

PDWF band distance (meV) and τ ($\AA$)

η_0	η_2	η_4	$\eta_0^{\max}$	$\eta_2^{\max}$	$\eta_4^{\max}$	τ
3.2	9.0	43.7	15.4	91.3	636.3	2.339

SCDM band distance (meV) and τ ($\AA$)

η_0	η_2	η_4	$\eta_0^{\max}$	$\eta_2^{\max}$	$\eta_4^{\max}$	τ
5.4	5.6	6.7	38.0	38.0	49.4	2.489

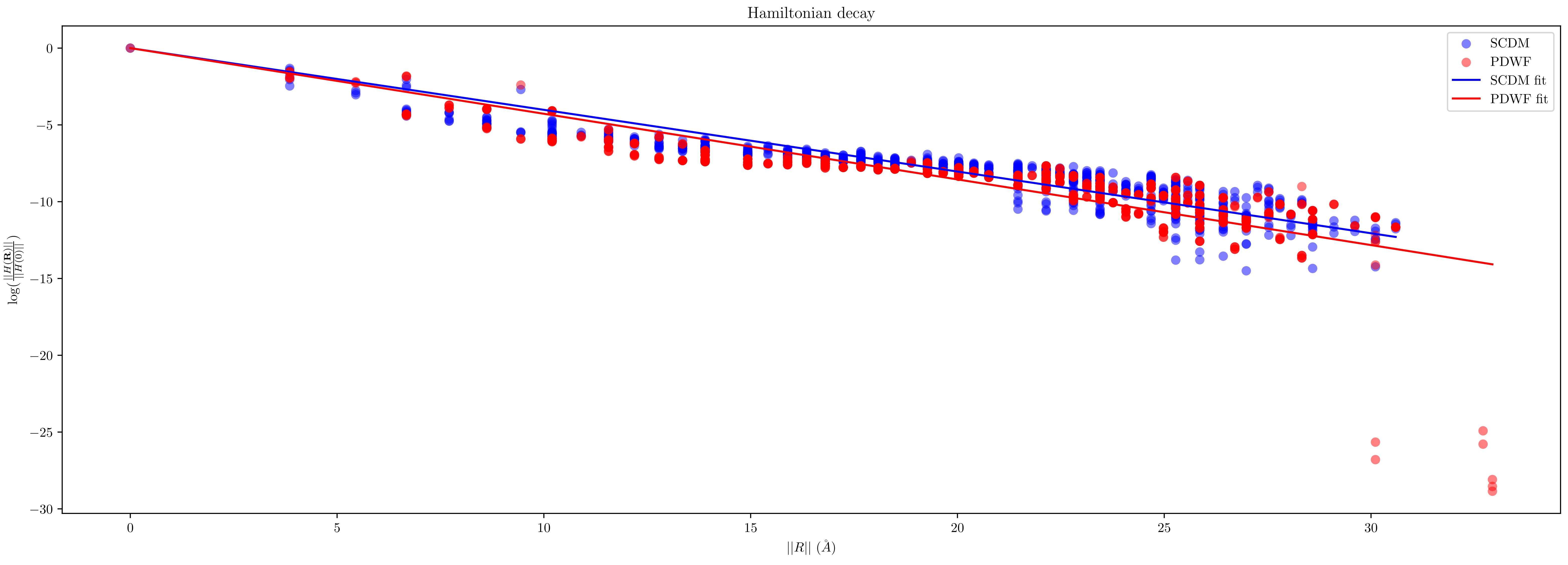

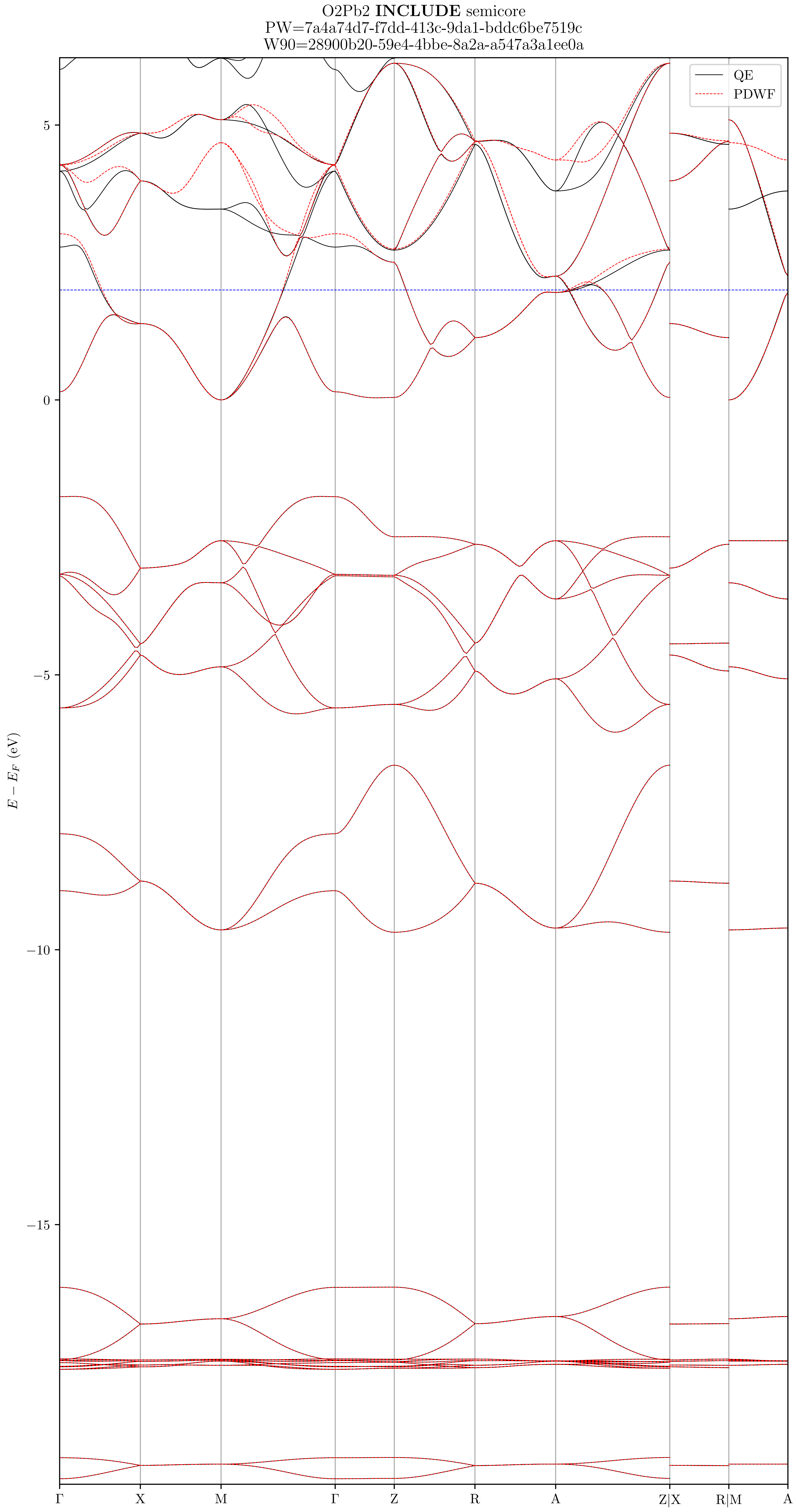

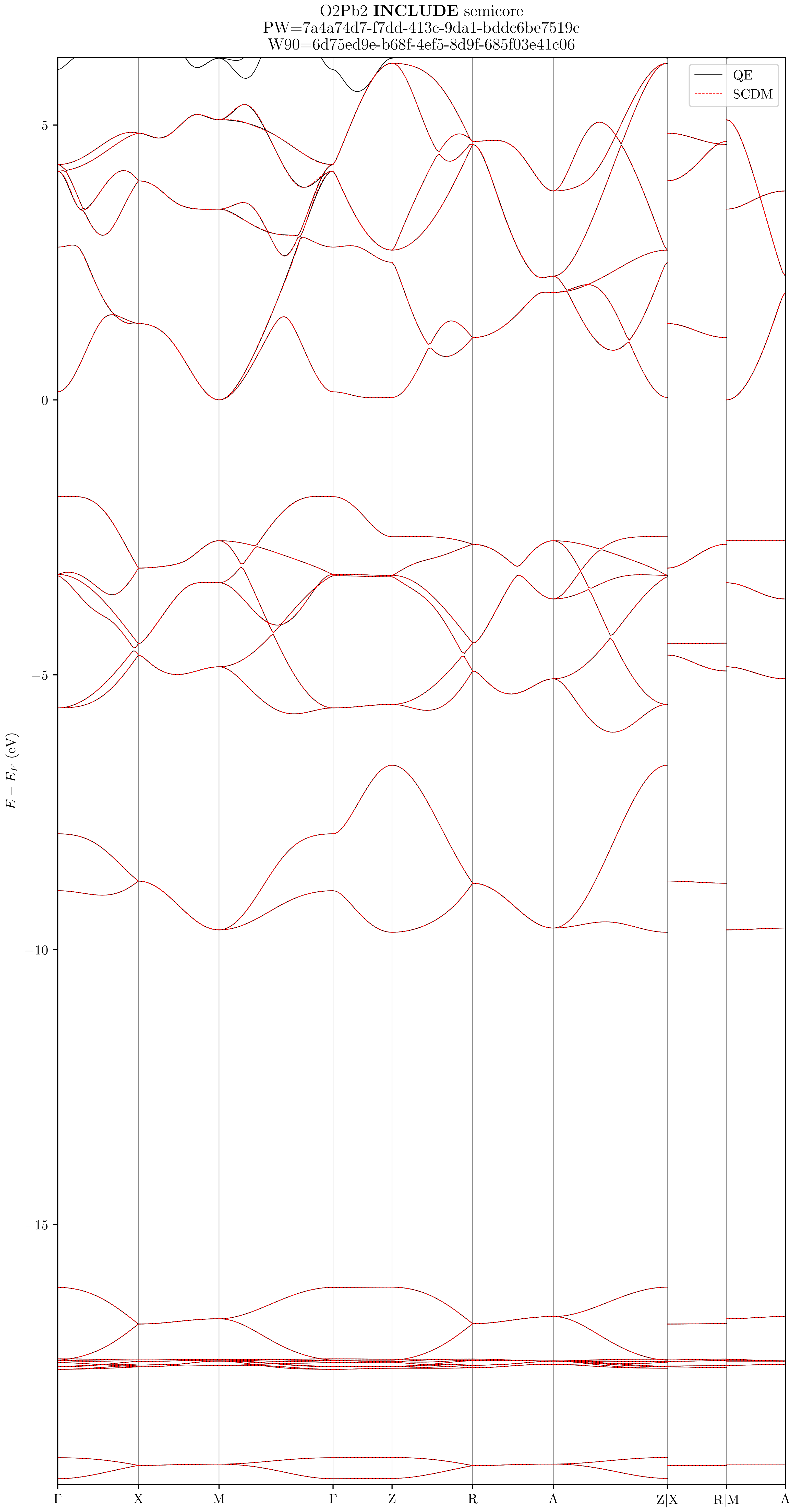

PDWF band distance (meV) and τ ($\AA$)

η_0	η_2	η_4	$\eta_0^{\max}$	$\eta_2^{\max}$	$\eta_4^{\max}$	τ
0.5	0.8	18.8	4.6	10.1	220.5	2.189

SCDM band distance (meV) and τ ($\AA$)

η_0	η_2	η_4	$\eta_0^{\max}$	$\eta_2^{\max}$	$\eta_4^{\max}$	τ
0.6	0.8	1.5	8.0	10.2	30.0	2.056

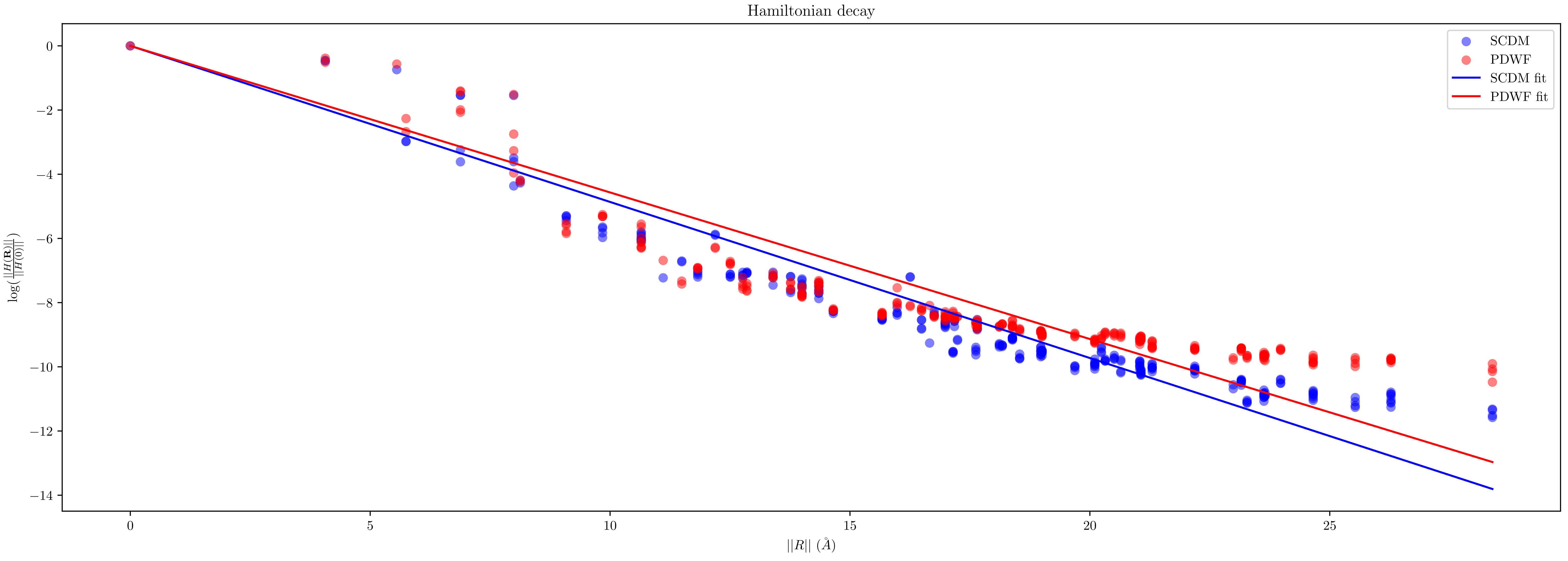

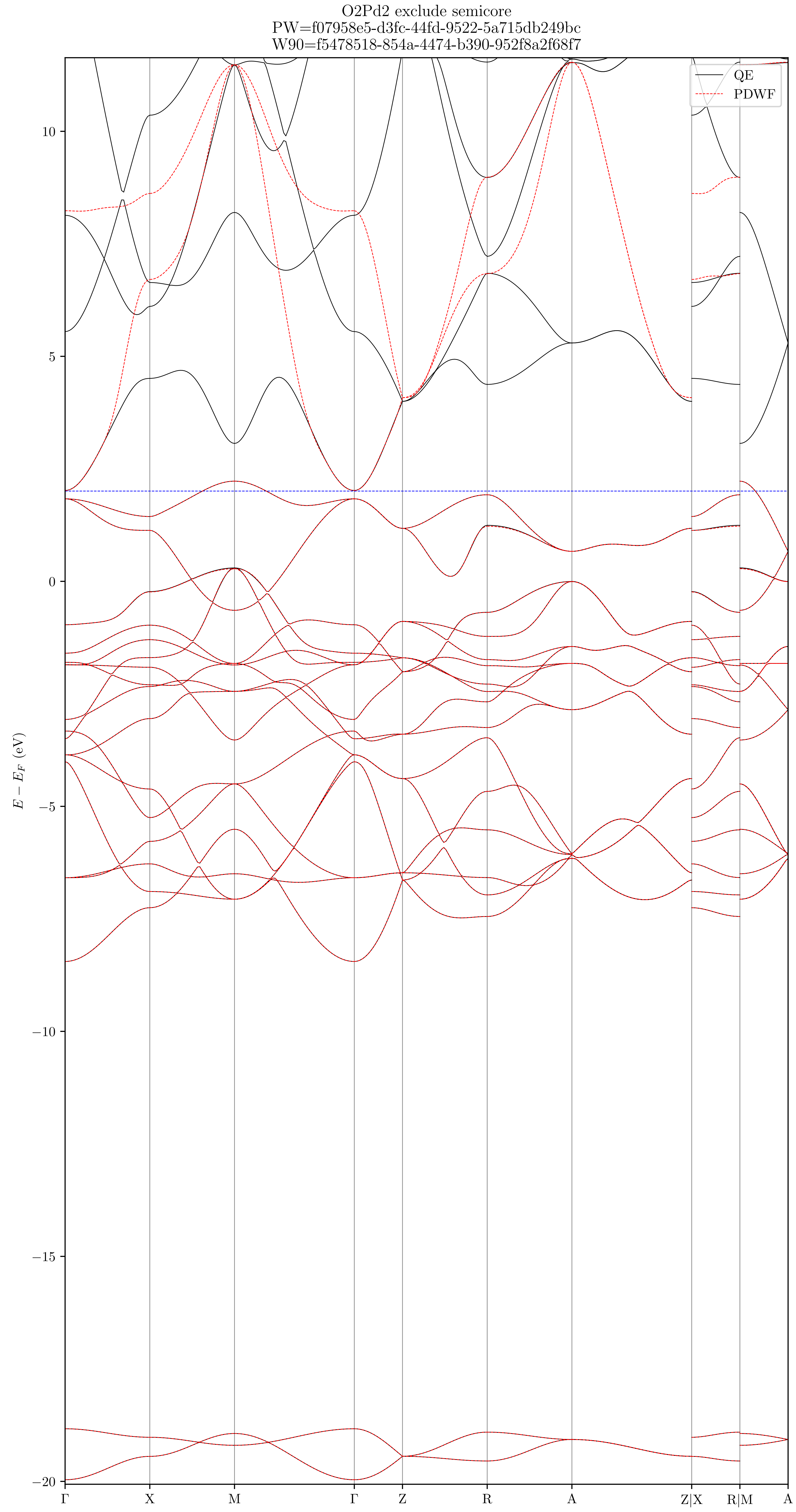

PDWF band distance (meV) and τ ($\AA$)

η_0	η_2	η_4	η_0^{max}	η_2^{max}	η_4^{max}	τ
1.0	2.1	8.8	12.2	20.2	241.2	1.976

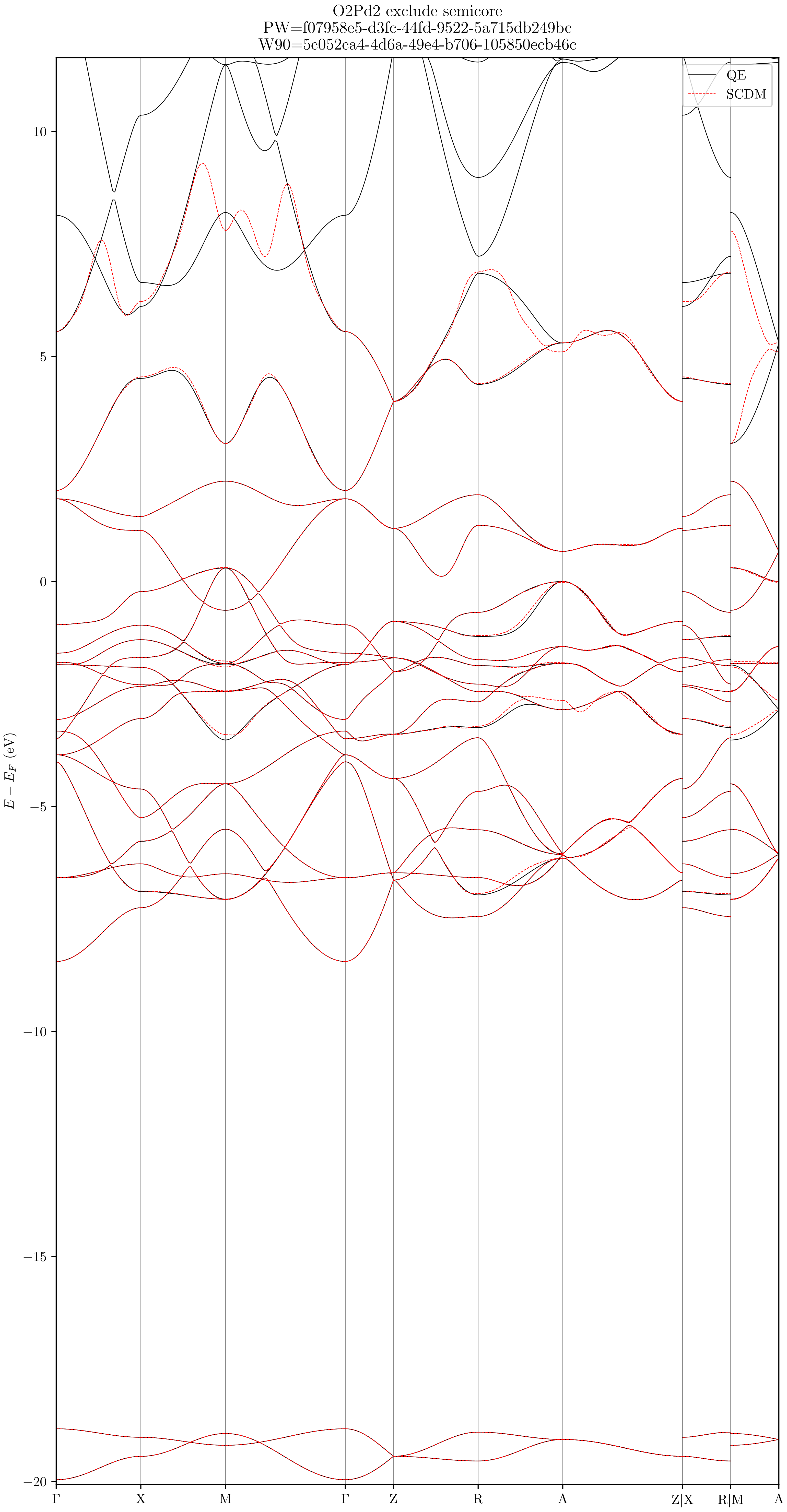

SCDM band distance (meV) and τ ($\AA$)

η_0	η_2	η_4	η_0^{max}	η_2^{max}	η_4^{max}	τ
20.4	19.3	24.4	207.6	207.6	534.9	2.370

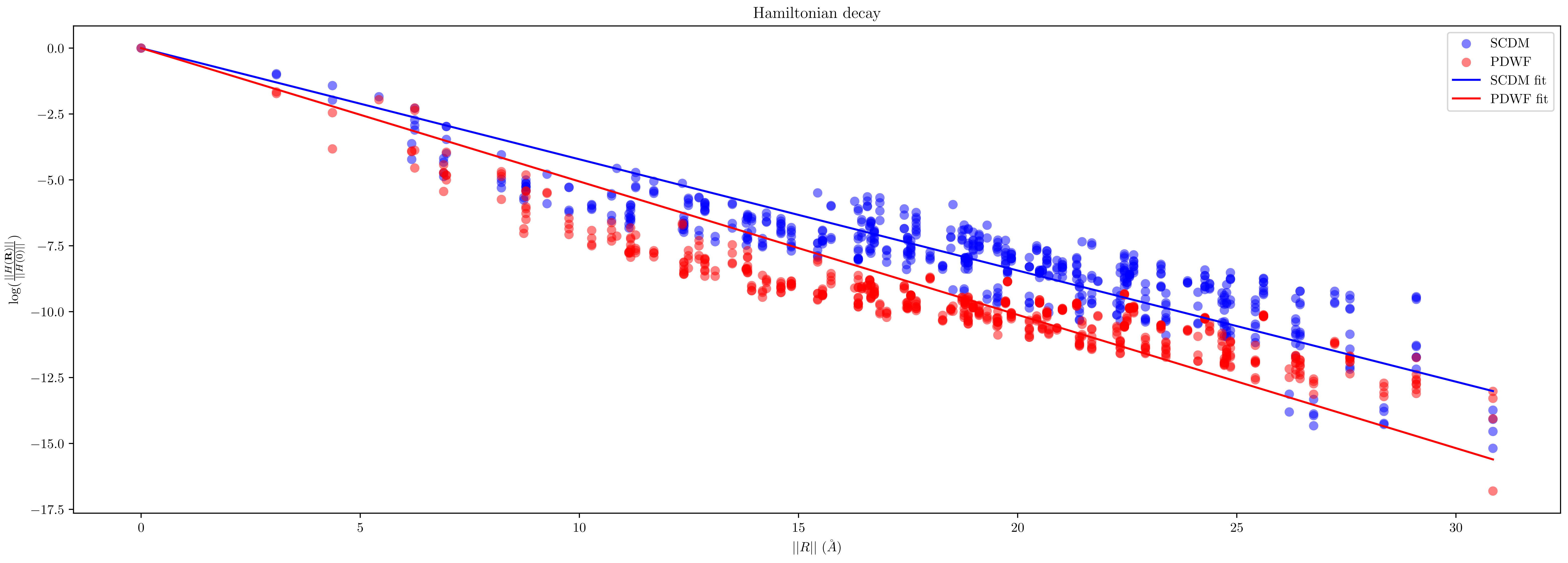

O2Rb **INCLUDE** semicore
PW=f9cdbf71-1b0f-462a-a55d-ecd035676f7c
W90=b9e46f0c-2746-45c4-88d2-7ccb9ca53237

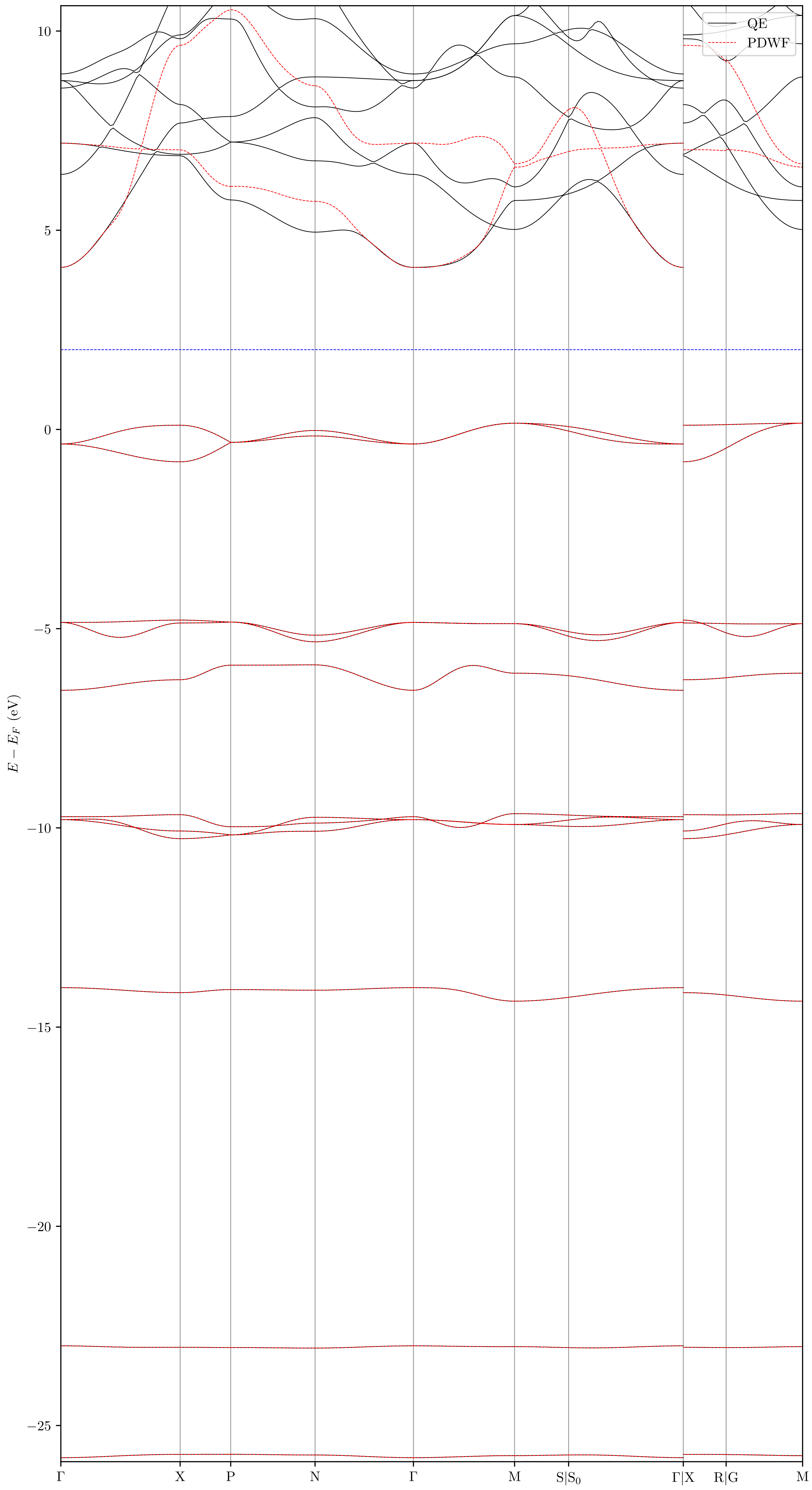

O2Rb **INCLUDE** semicore
PW=f9cdbf71-1b0f-462a-a55d-ecd035676f7c
W90=d6024ccc-3262-444e-ab14-ffecf0c81723

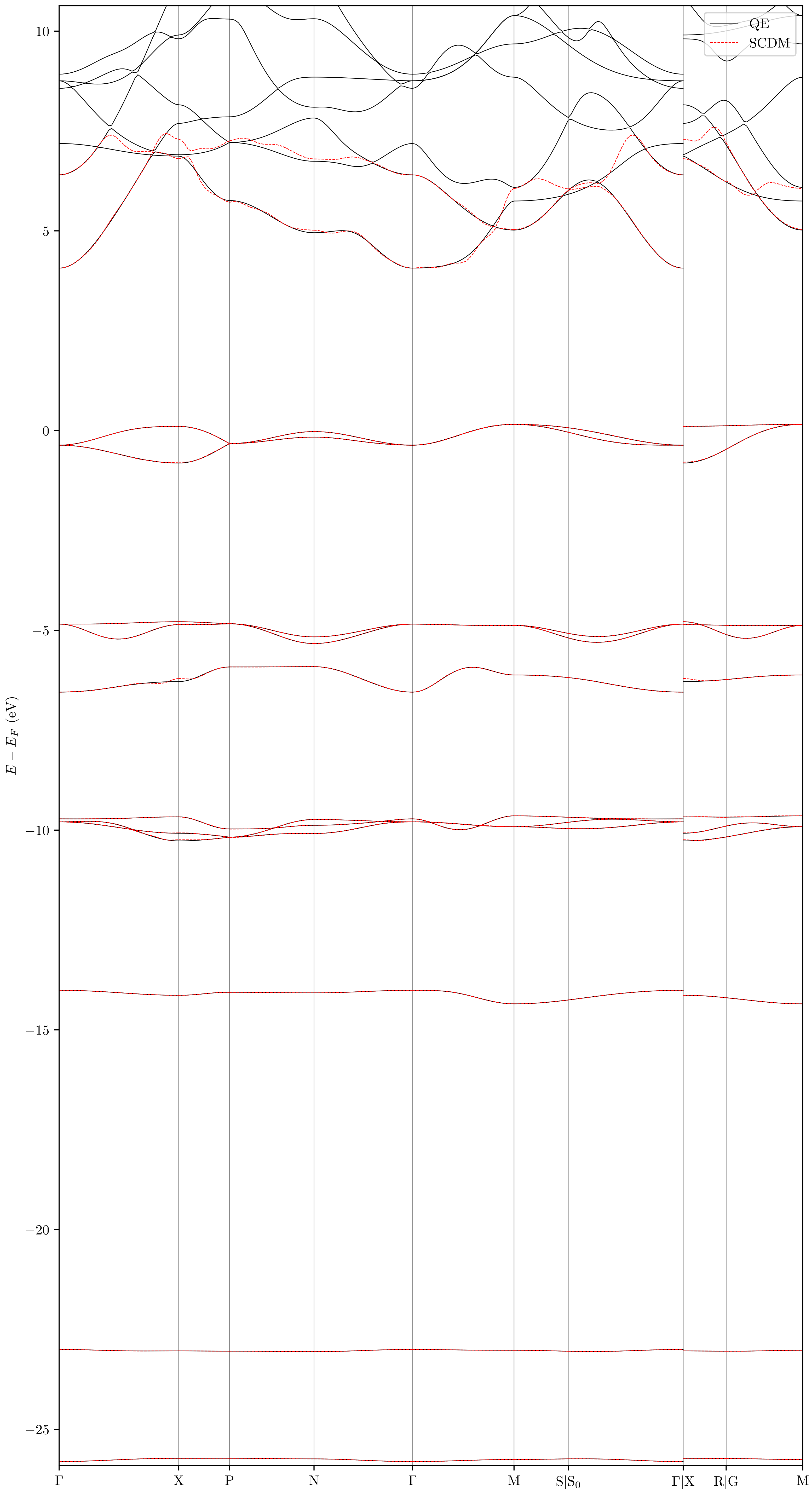

PDWF band distance (meV) and τ ($\AA$)

η_0	η_2	η_4	$\eta_0^{\max}$	$\eta_2^{\max}$	$\eta_4^{\max}$	τ
0.7	0.7	0.9	3.9	3.9	3.9	2.196

SCDM band distance (meV) and τ ($\AA$)

η_0	η_2	η_4	$\eta_0^{\max}$	$\eta_2^{\max}$	$\eta_4^{\max}$	τ
5.1	4.9	5.0	76.0	76.0	76.0	2.661

Hamiltonian decay

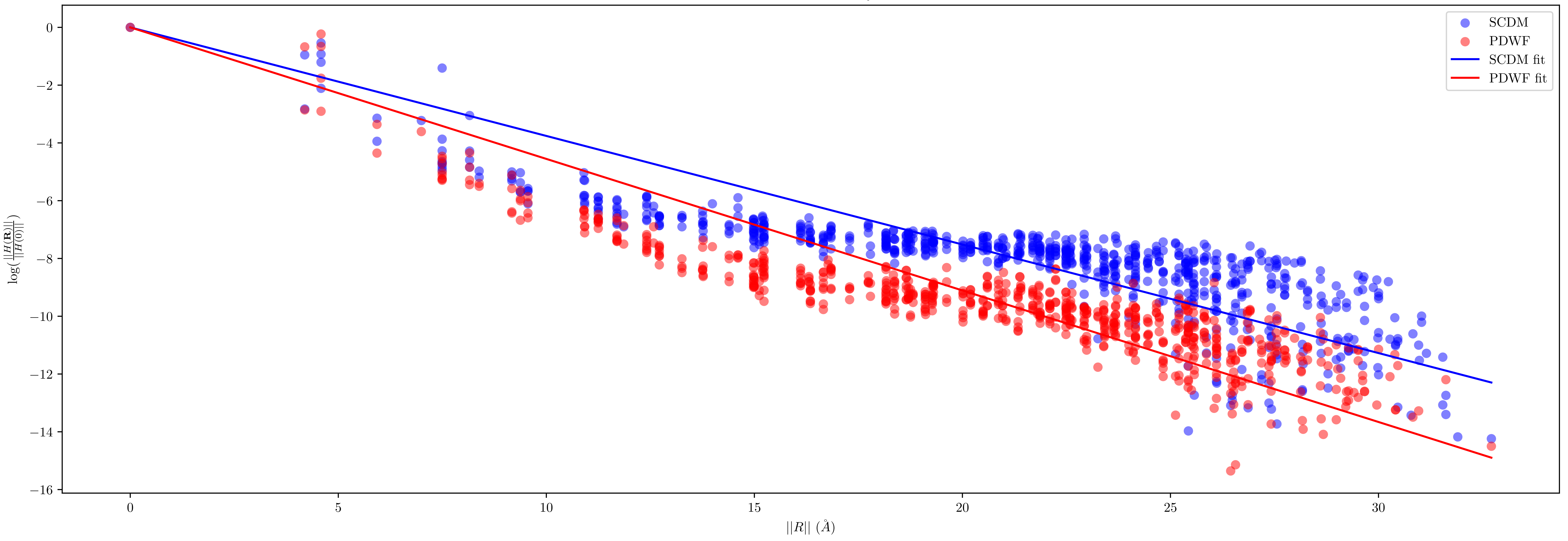

O2Rb2 **INCLUDE** semicore
PW=d7432188-936d-417e-9273-709986f64b5b
W90=1525ee02-8aa6-4f5c-af84-e2998a177843

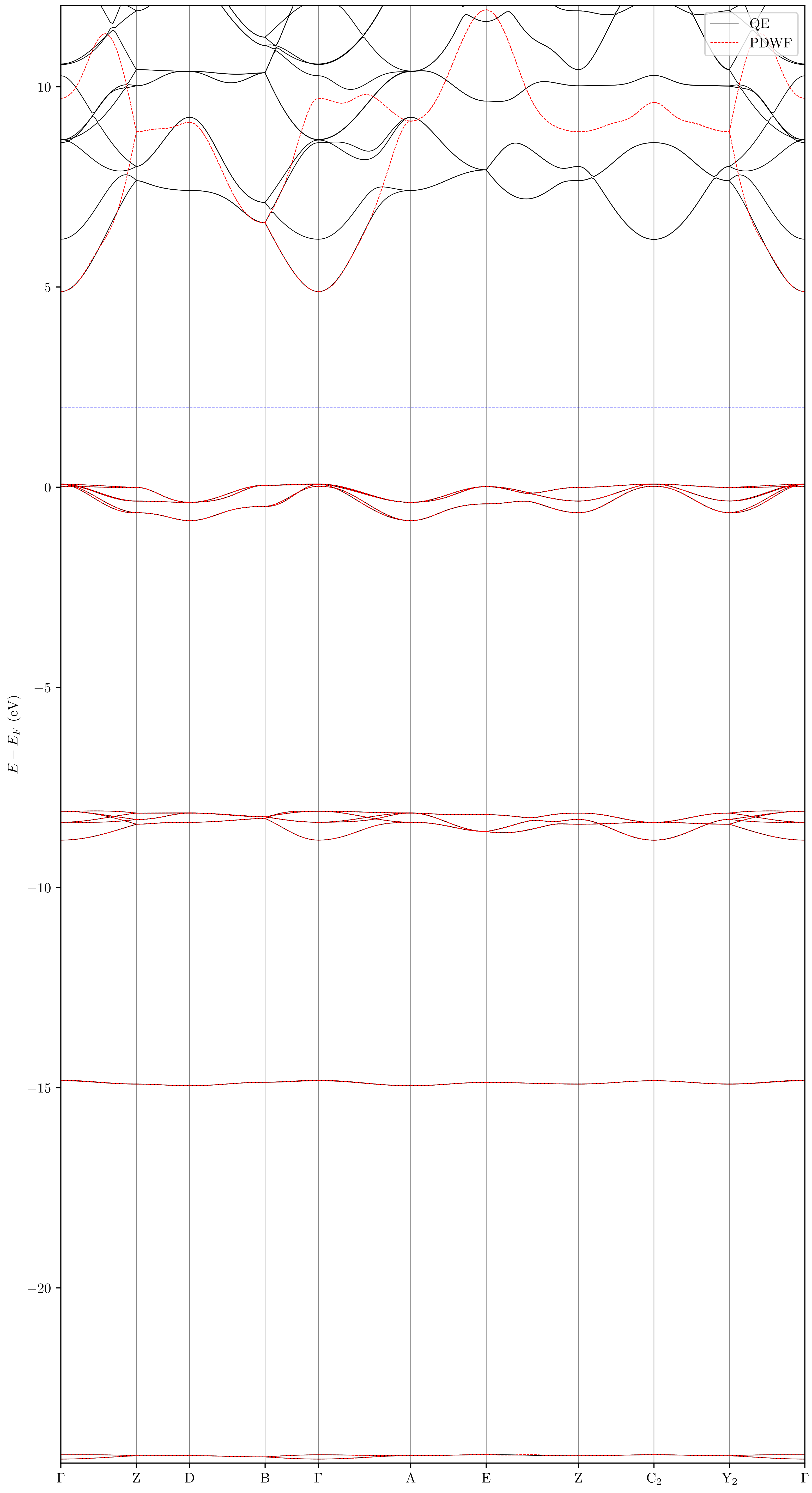

PDWF band distance (meV) and τ ($\AA$)

η_0	η_2	η_4	η_0^{max}	η_2^{max}	η_4^{max}	τ
1.2	1.1	1.1	16.0	16.0	16.0	2.178

O2Rb2 **INCLUDE** semicore
PW=d7432188-936d-417e-9273-709986f64b5b
W90=ece5d91a-a02e-436b-b5c5-b757f970eb29

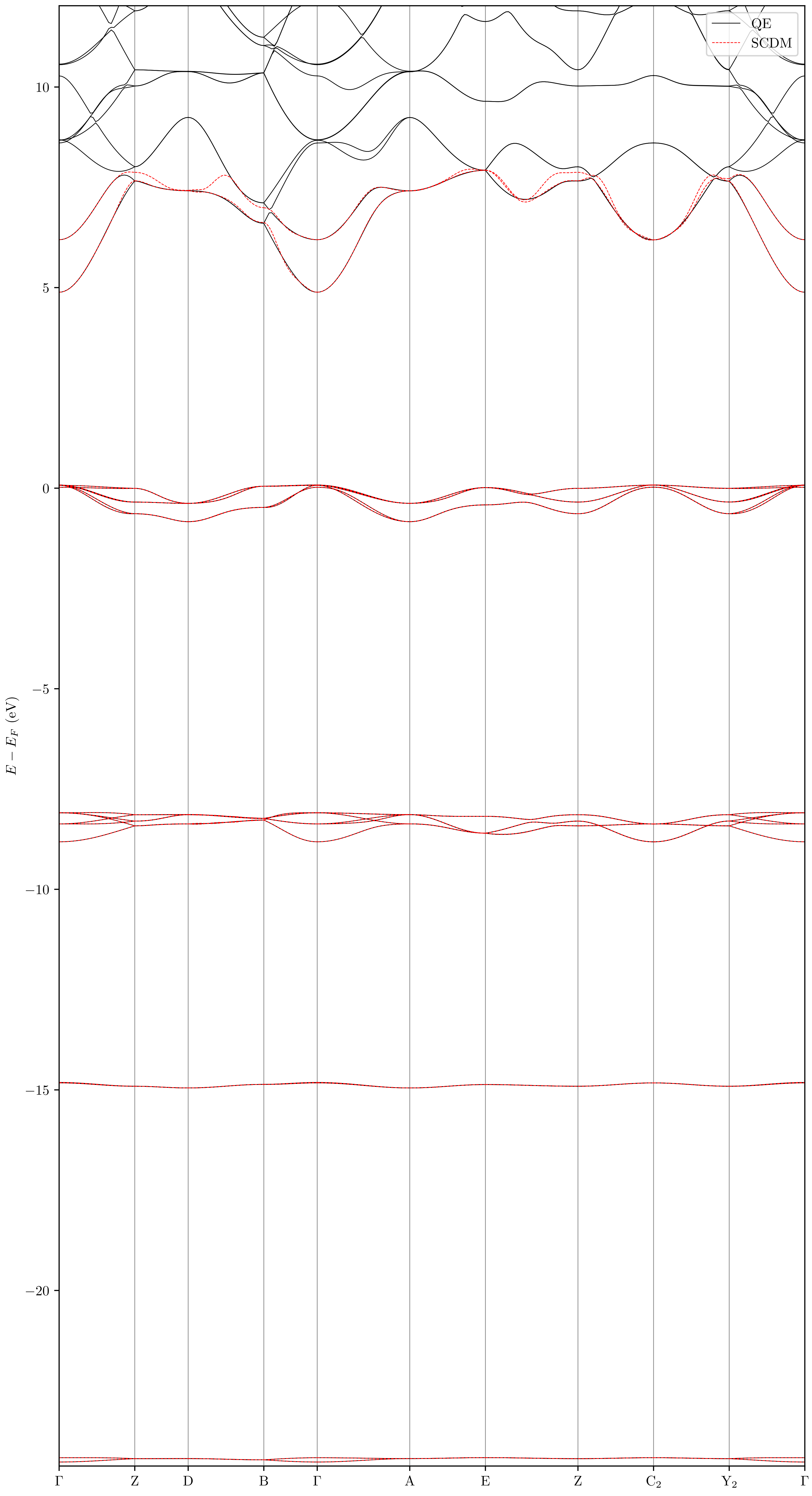

SCDM band distance (meV) and τ ($\AA$)

η_0	η_2	η_4	η_0^{max}	η_2^{max}	η_4^{max}	τ
1.2	1.1	1.1	12.5	12.5	12.5	2.449

Hamiltonian decay

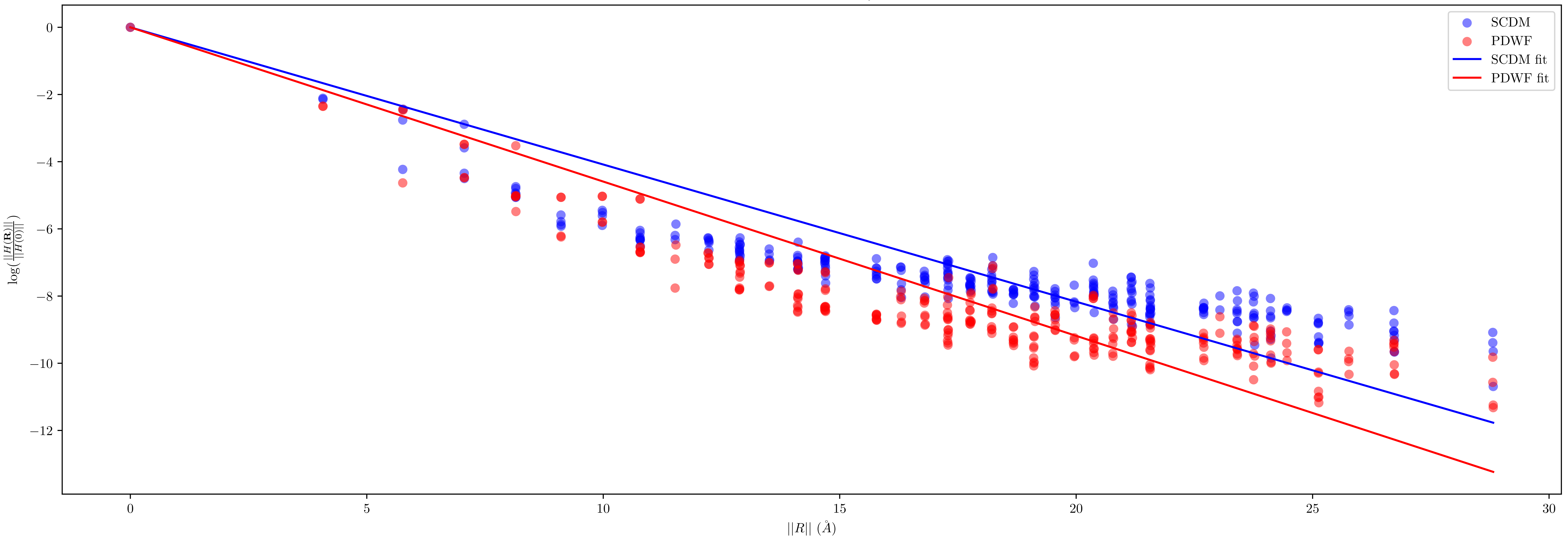

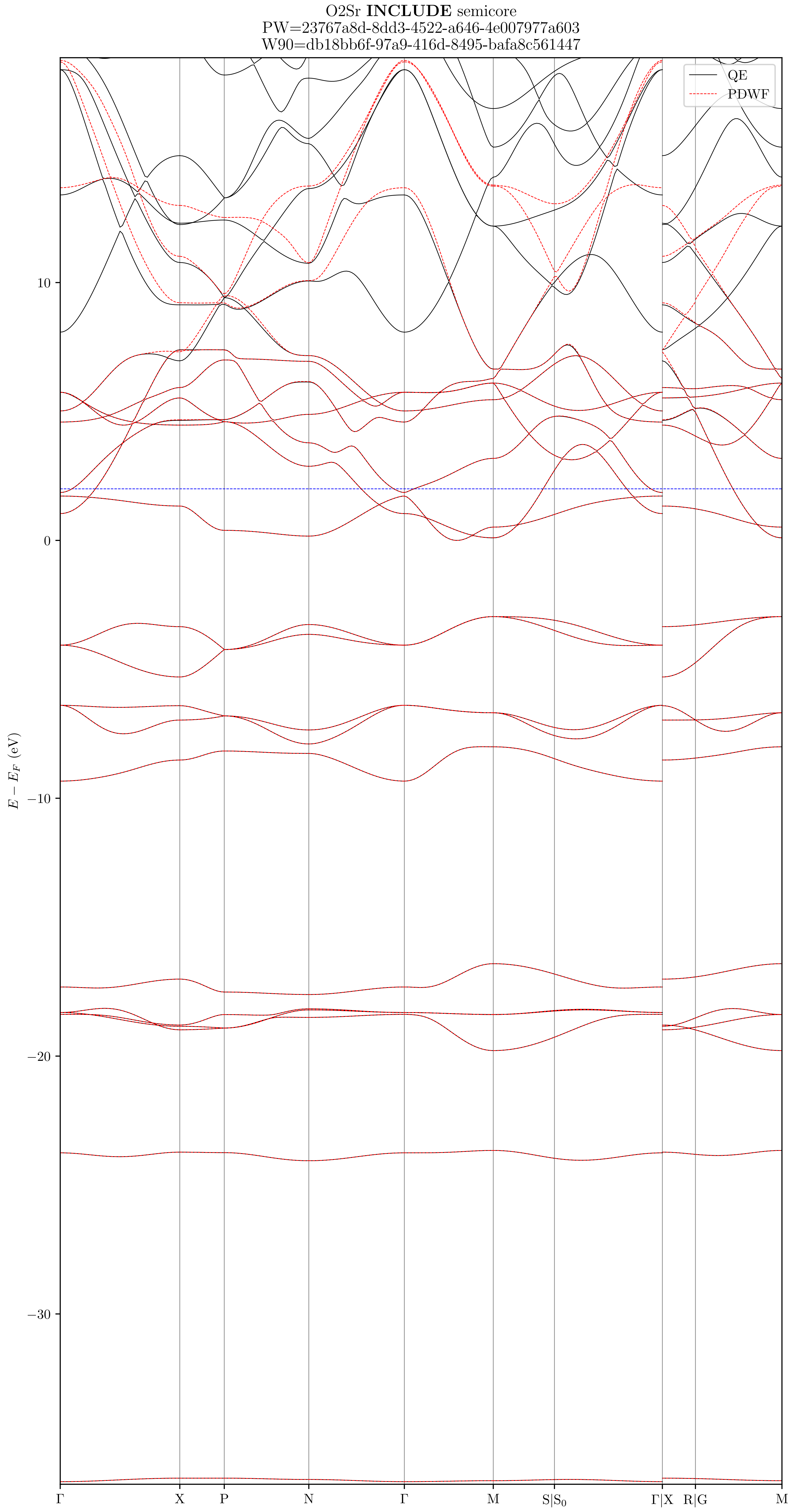

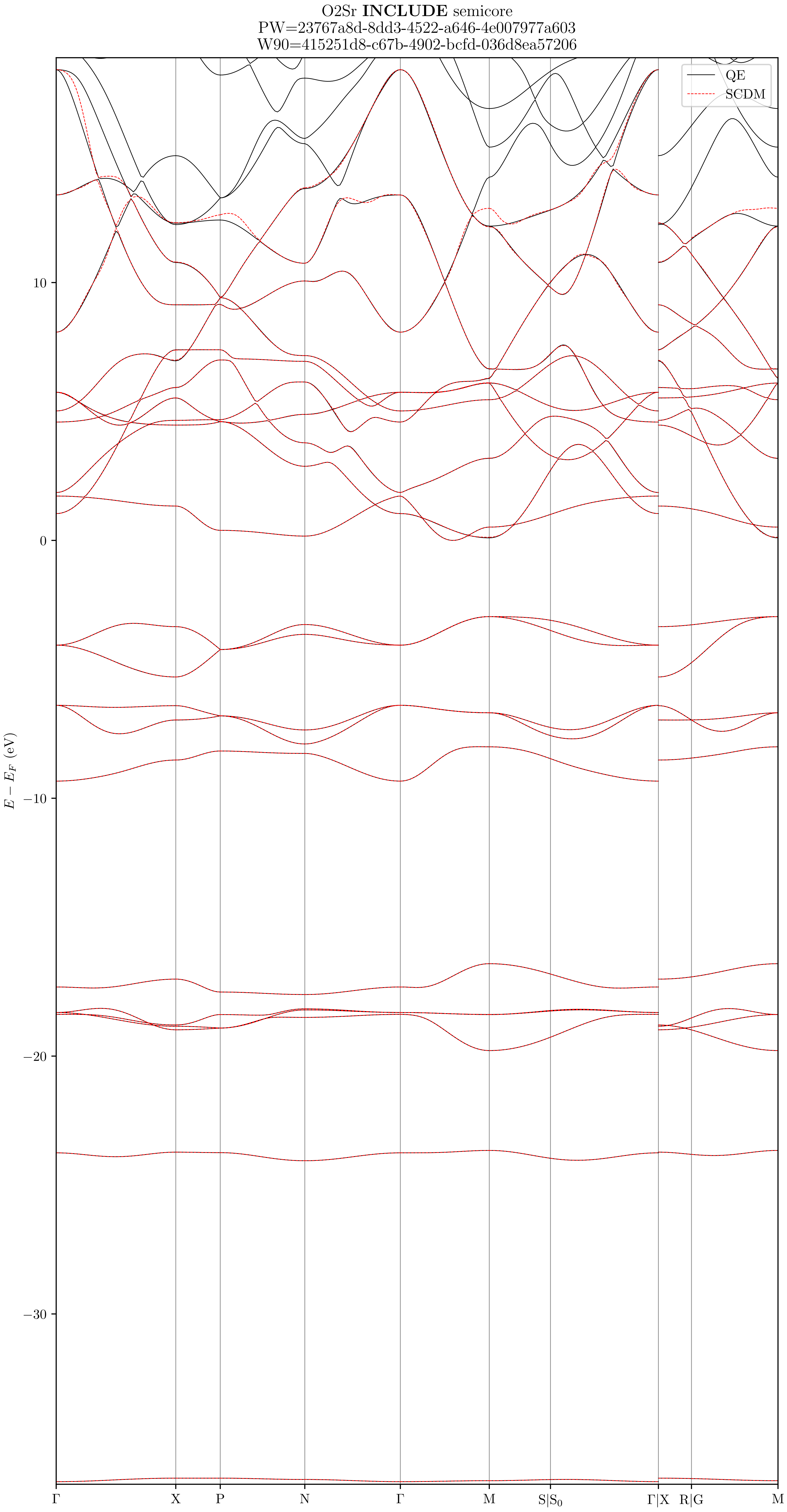

PDWF band distance (meV) and τ ($\AA$)

η_0	η_2	η_4	$\eta_0^{\max}$	$\eta_2^{\max}$	$\eta_4^{\max}$	τ
0.3	0.5	0.6	2.5	7.6	7.6	2.175

SCDM band distance (meV) and τ ($\AA$)

η_0	η_2	η_4	$\eta_0^{\max}$	$\eta_2^{\max}$	$\eta_4^{\max}$	τ
1.1	1.8	2.0	6.5	24.6	24.6	2.462

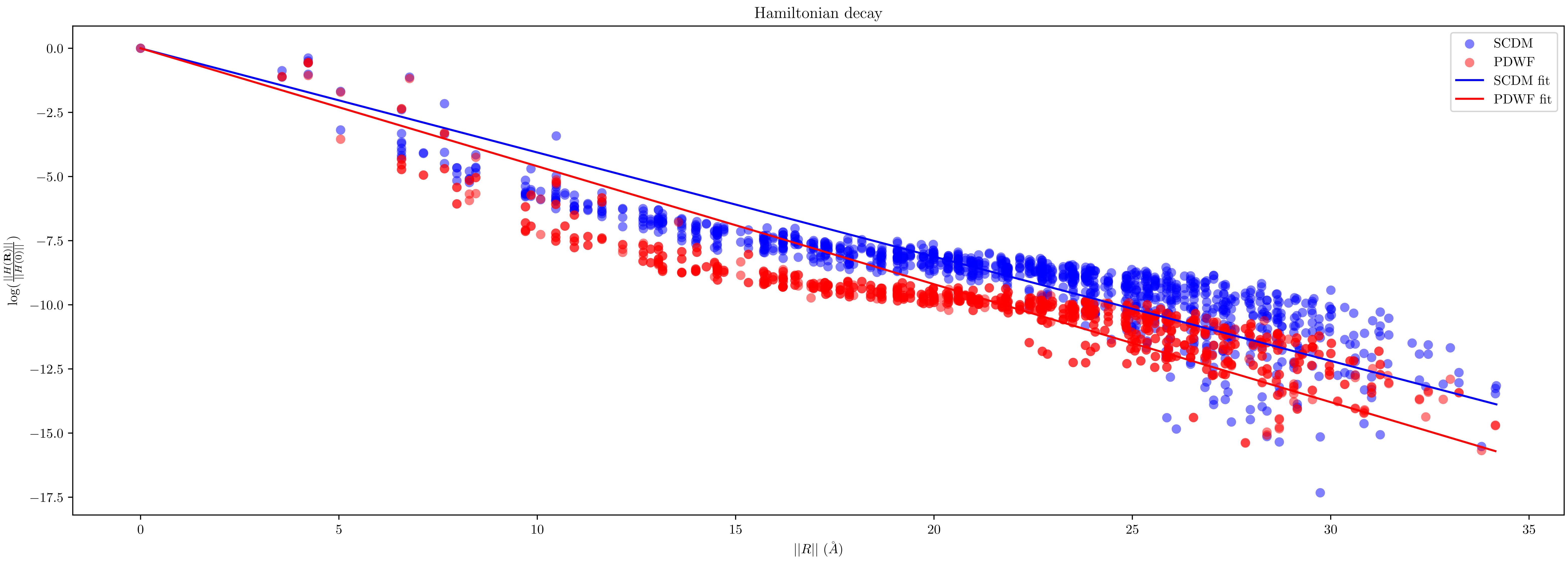

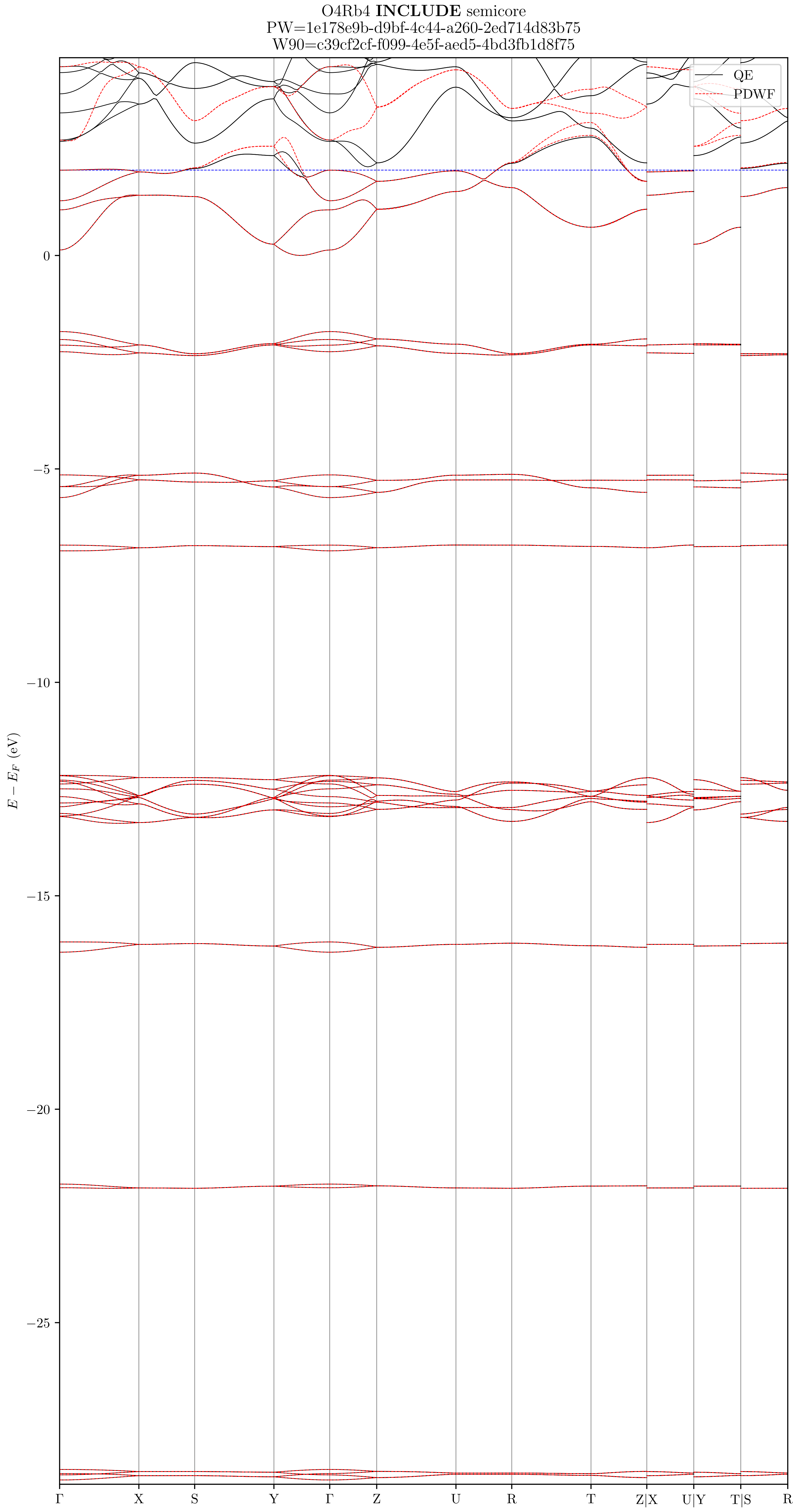

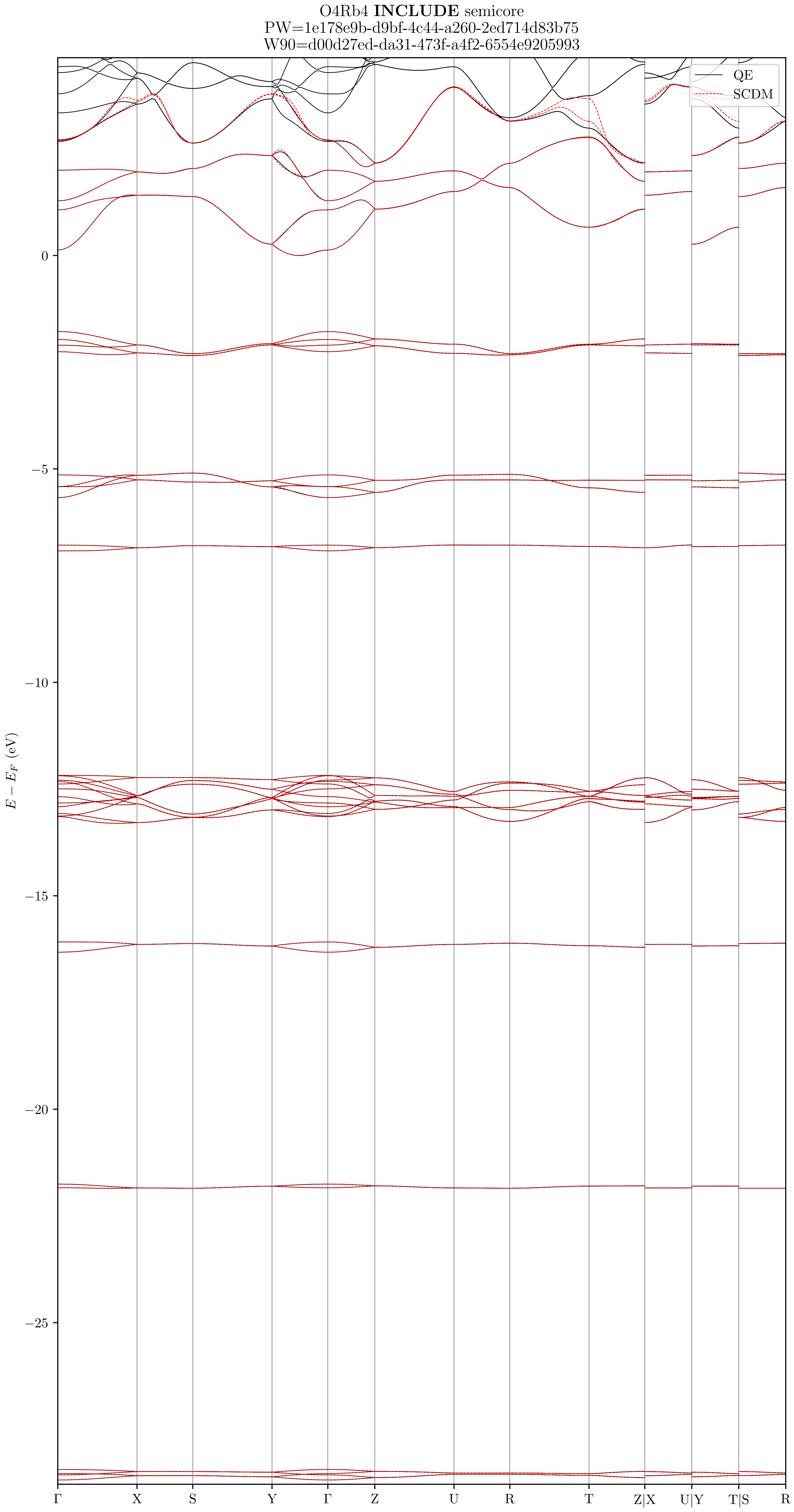

PDWF band distance (meV) and τ ($\AA$)

η_0	η_2	η_4	$\eta_0^{\max}$	$\eta_2^{\max}$	$\eta_4^{\max}$	τ
0.2	0.6	75.4	1.4	10.8	949.5	2.398

SCDM band distance (meV) and τ ($\AA$)

η_0	η_2	η_4	$\eta_0^{\max}$	$\eta_2^{\max}$	$\eta_4^{\max}$	τ
0.8	1.0	14.8	14.2	15.5	268.7	2.579

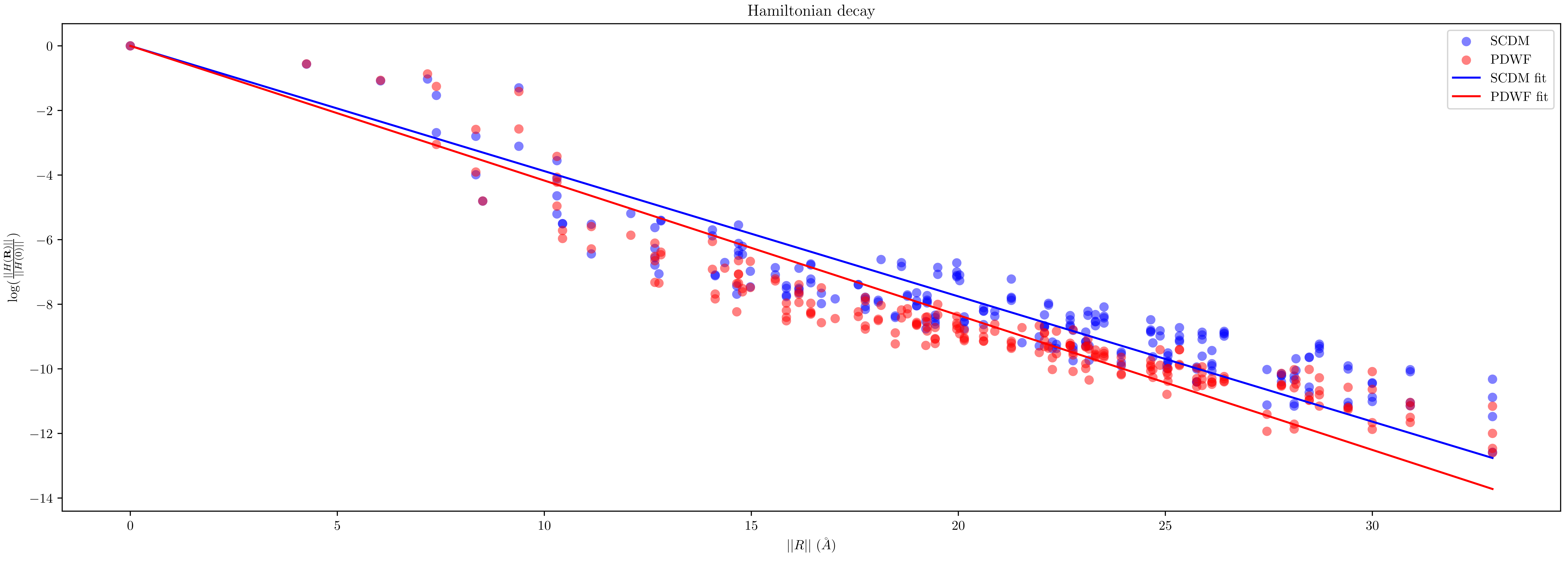

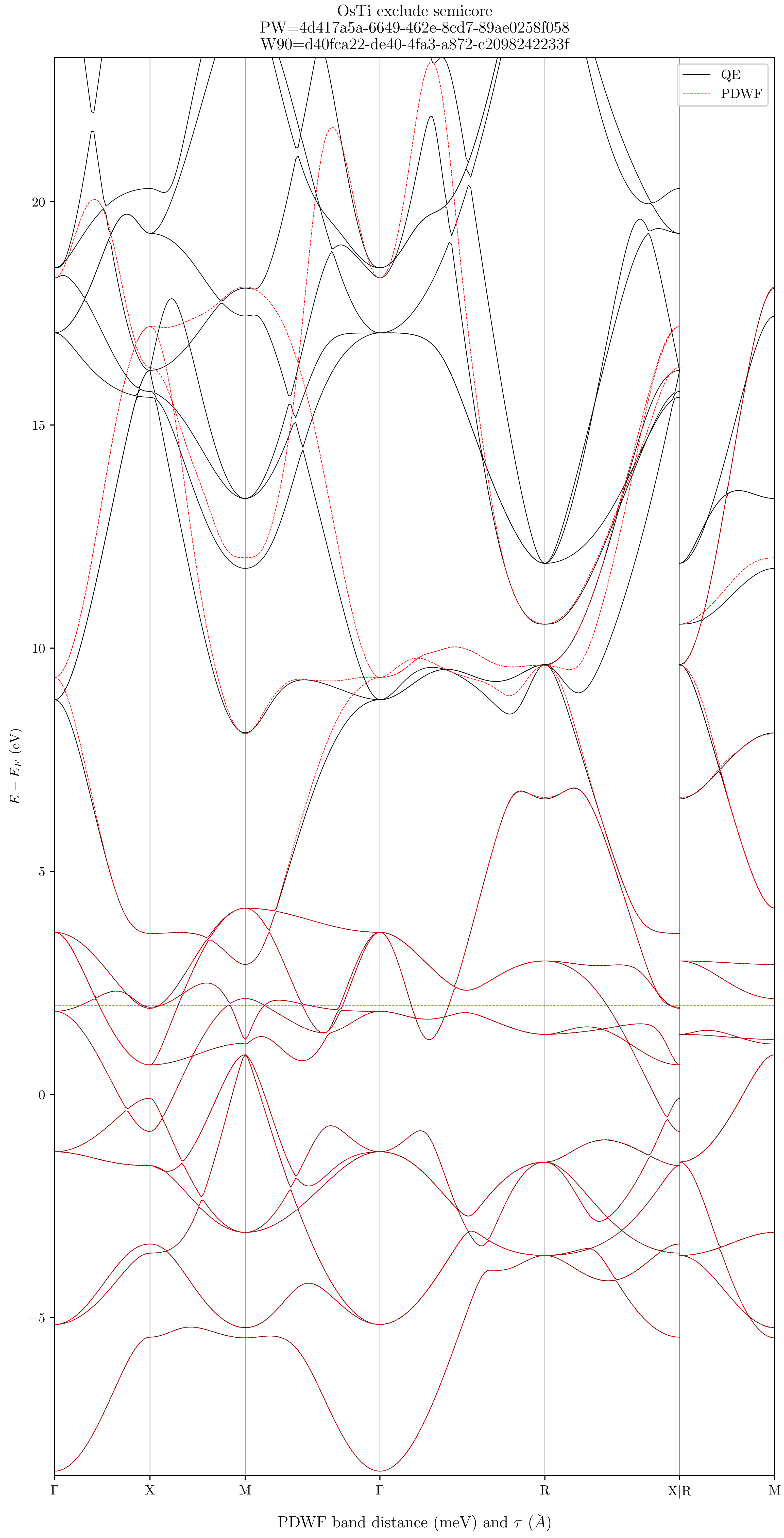

η_0	η_2	η_4	$\eta_0^{\max}$	$\eta_2^{\max}$	$\eta_4^{\max}$	τ
1.8	1.6	1.5	9.7	10.0	10.0	1.829

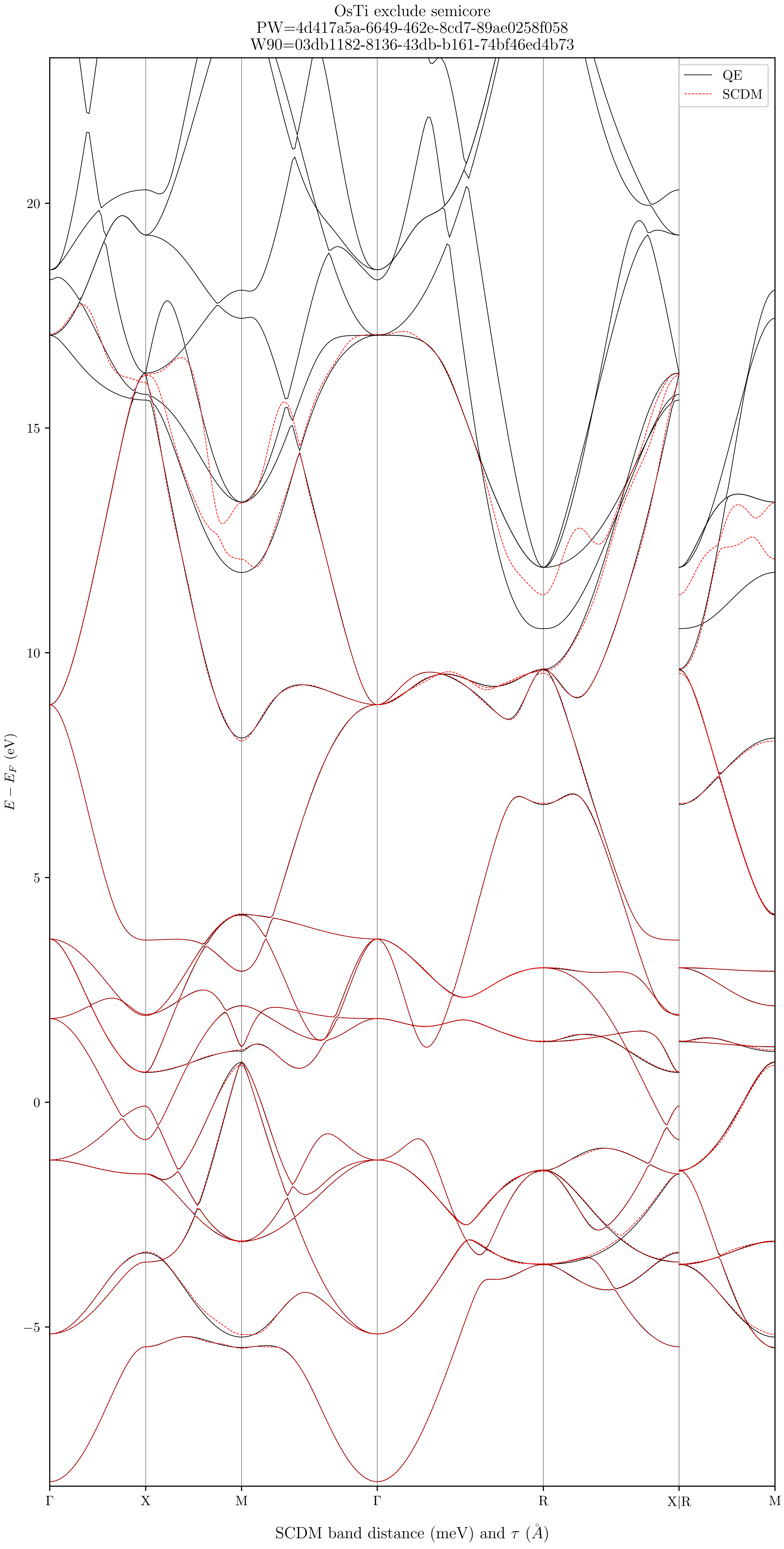

η_0	η_2	η_4	$\eta_0^{\max}$	$\eta_2^{\max}$	$\eta_4^{\max}$	τ
17.8	16.3	14.6	139.8	139.8	139.8	2.242

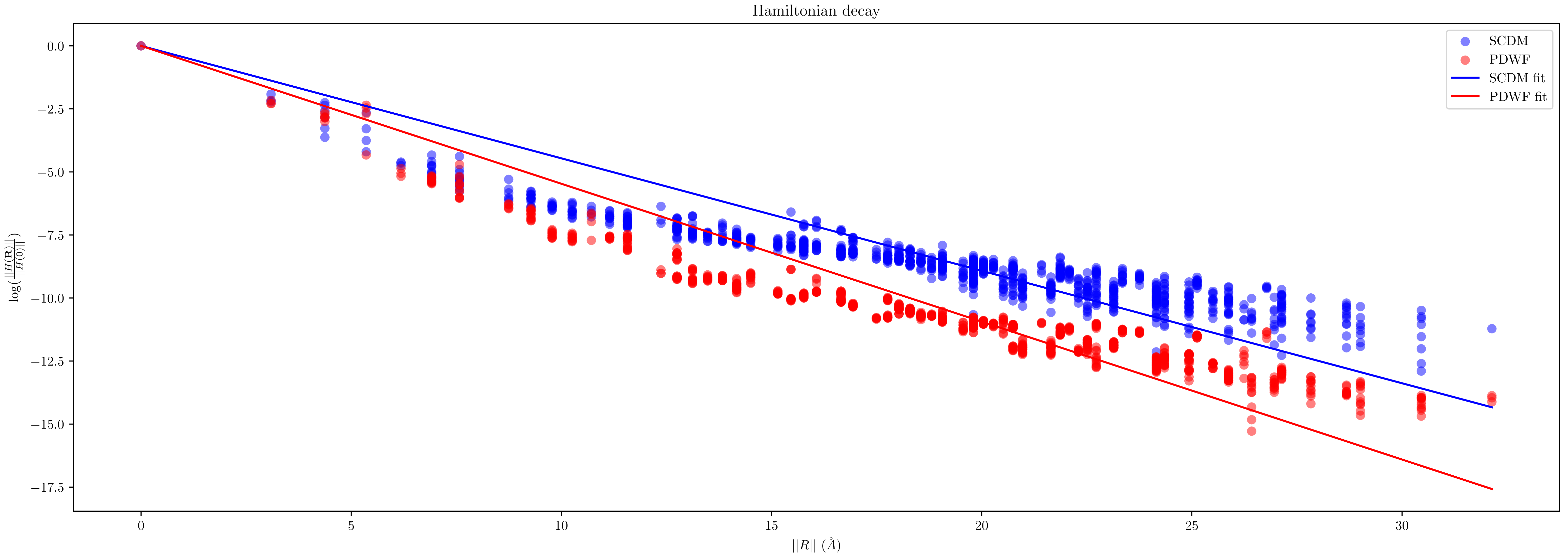

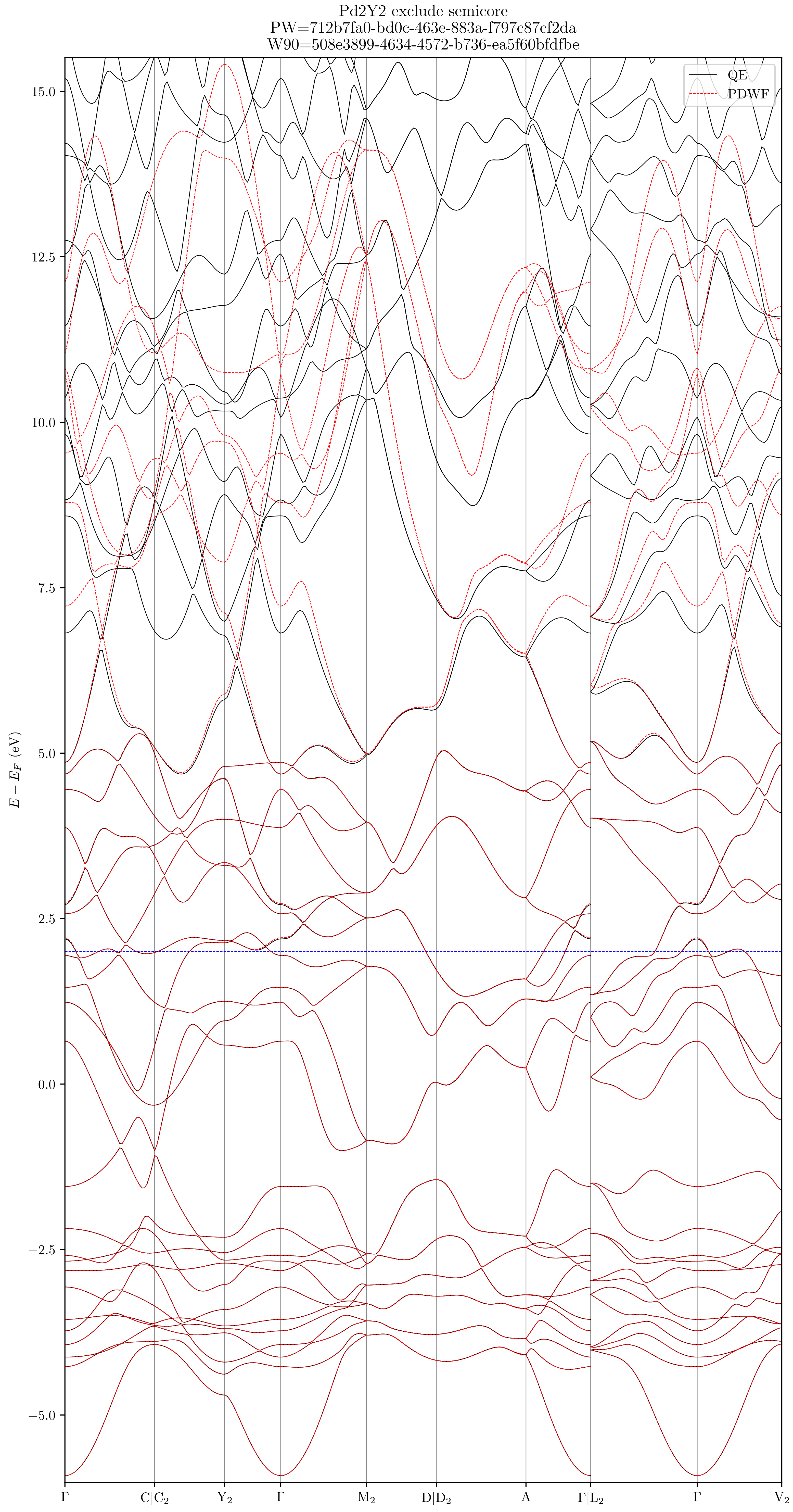

PDWF band distance (meV) and τ (Å)

η_0	η_2	η_4	$\eta_0^{\max}$	$\eta_2^{\max}$	$\eta_4^{\max}$	τ
0.7	1.2	2.3	6.8	8.7	19.9	2.333

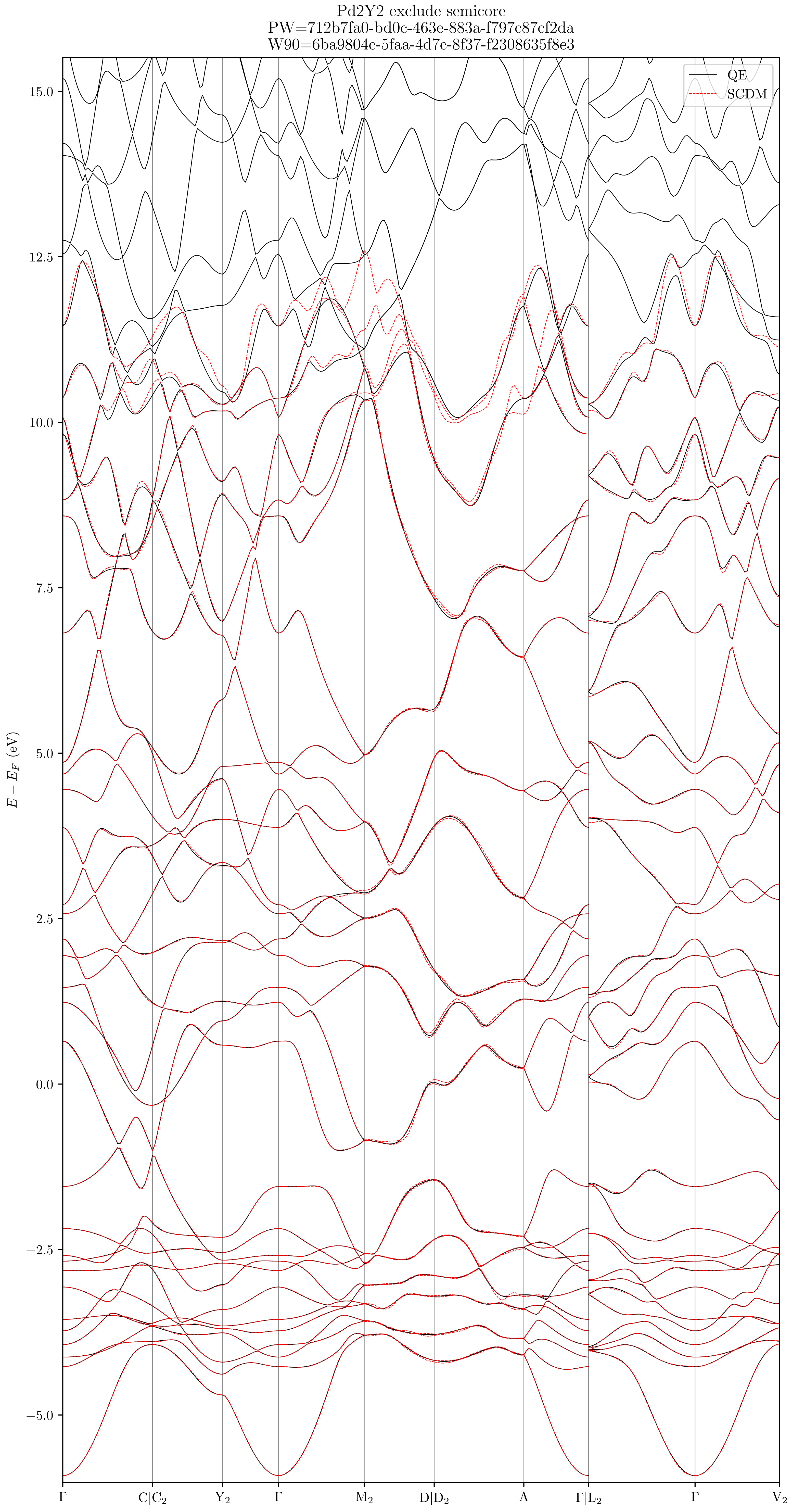

SCDM band distance (meV) and τ (Å)

η_0	η_2	η_4	$\eta_0^{\max}$	$\eta_2^{\max}$	$\eta_4^{\max}$	τ
7.3	9.1	9.6	73.5	76.2	76.2	2.913

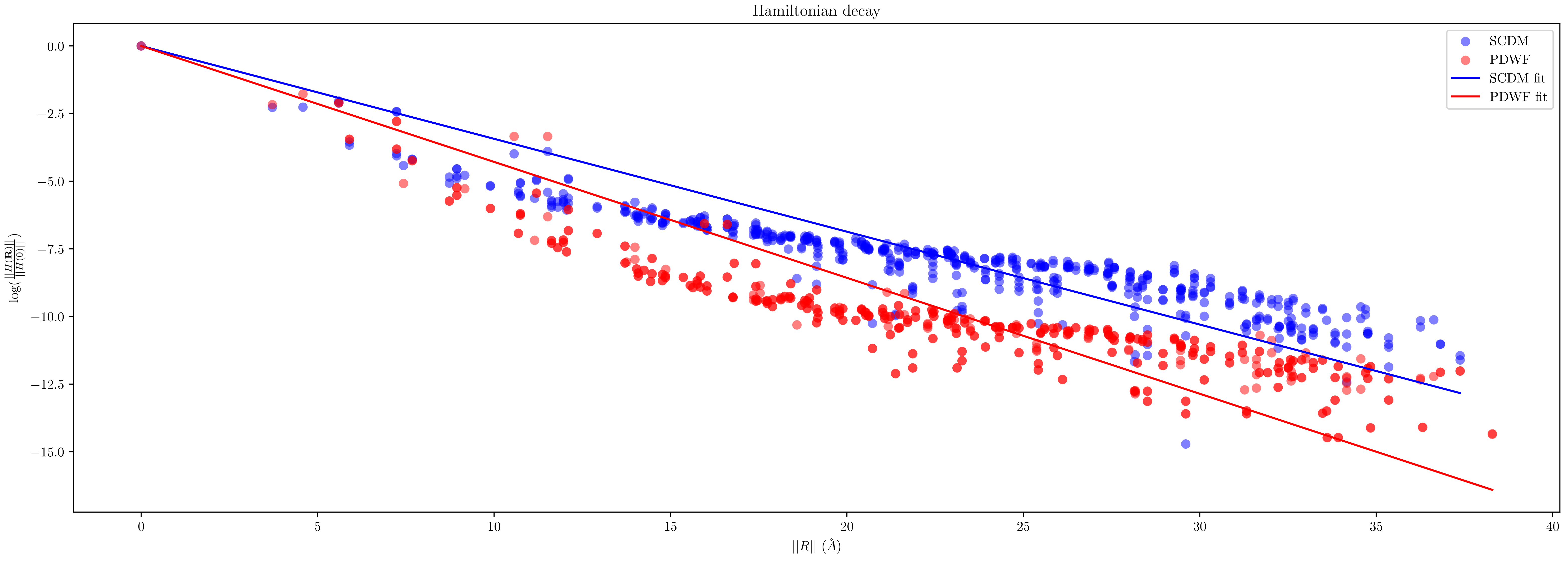

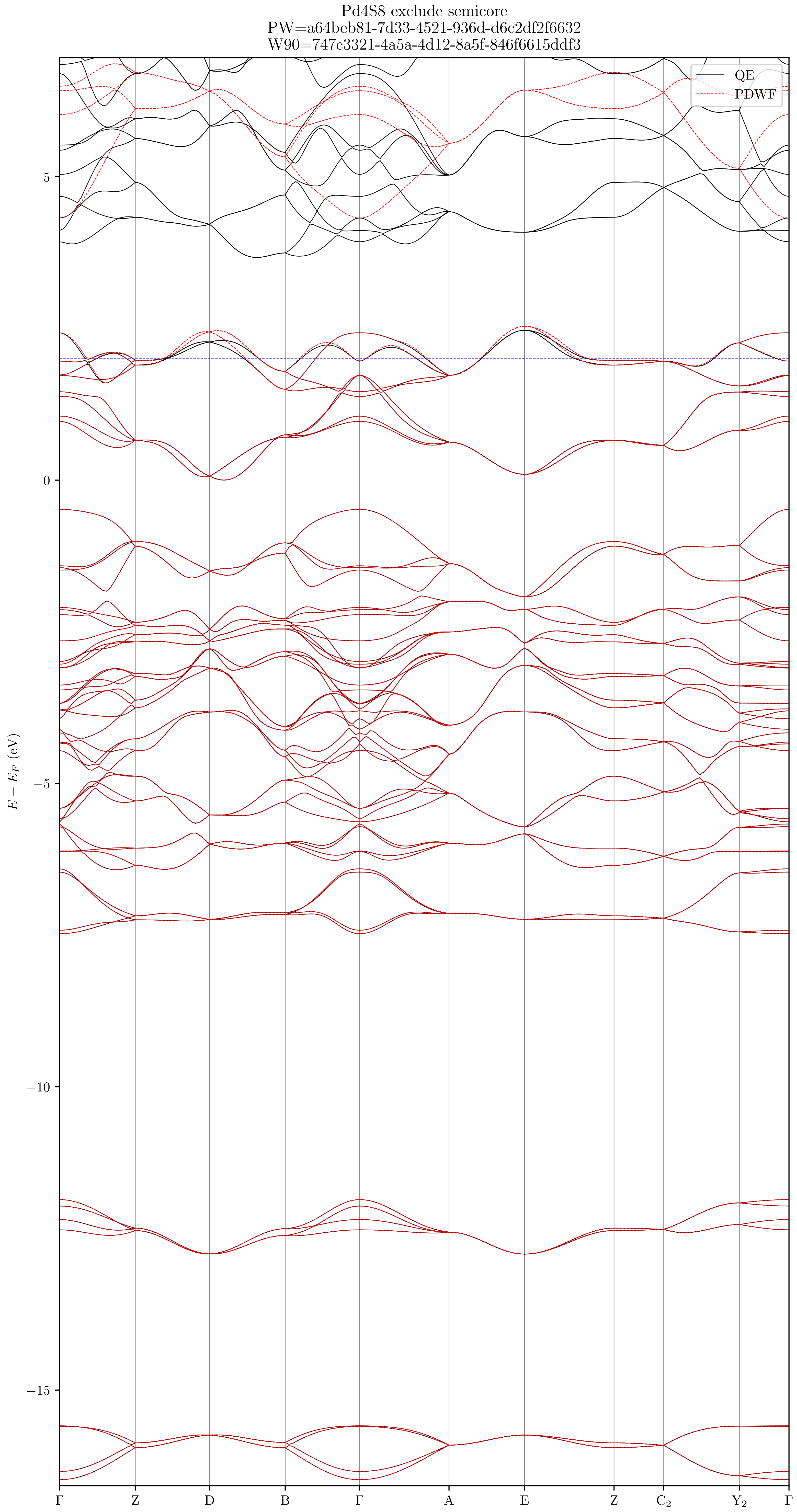

PDWF band distance (meV) and τ ($\mathring{A}$)

η_0	η_2	η_4	$\eta_0^{\max}$	$\eta_2^{\max}$	$\eta_4^{\max}$	τ
0.3	1.5	13.5	2.8	20.7	169.0	2.307

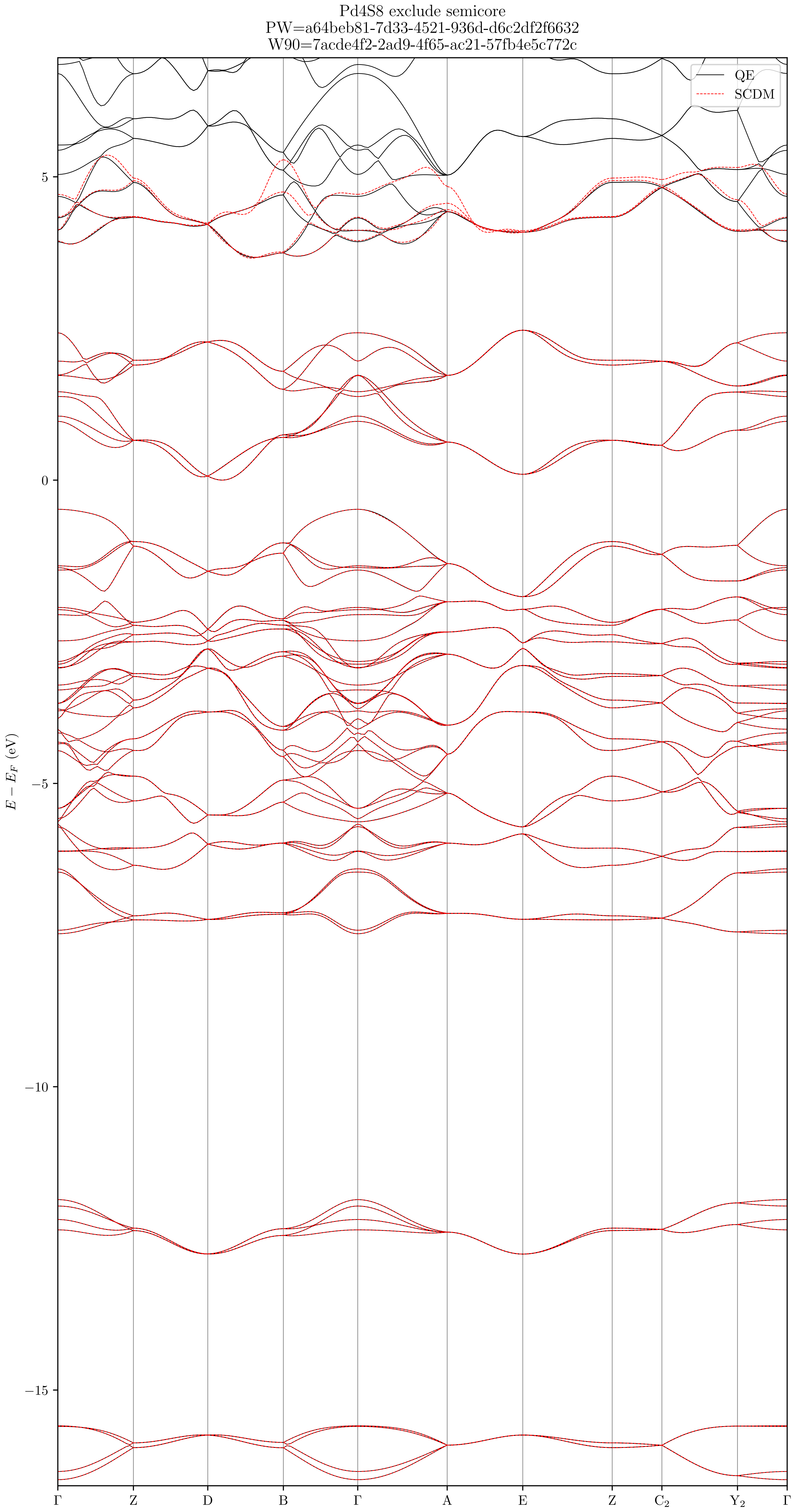

SCDM band distance (meV) and τ ($\mathring{A}$)

η_0	η_2	η_4	$\eta_0^{\max}$	$\eta_2^{\max}$	$\eta_4^{\max}$	τ
0.7	0.8	1.4	11.5	11.7	19.2	2.720

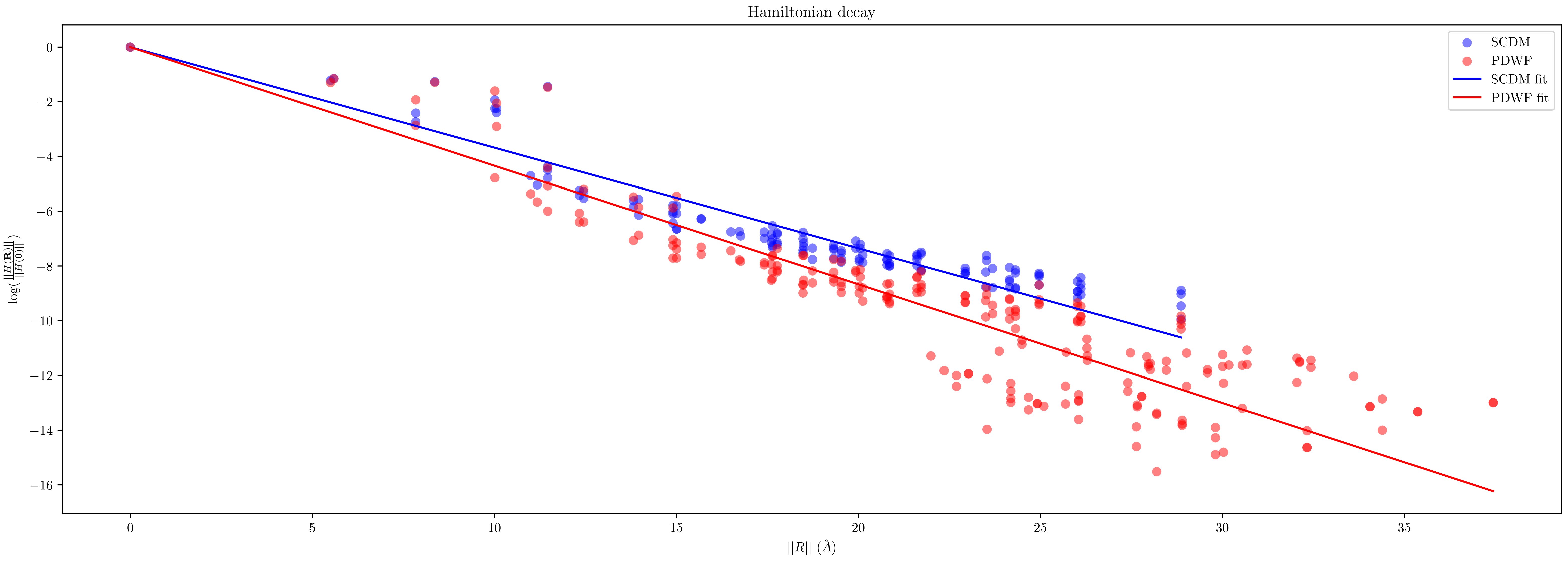

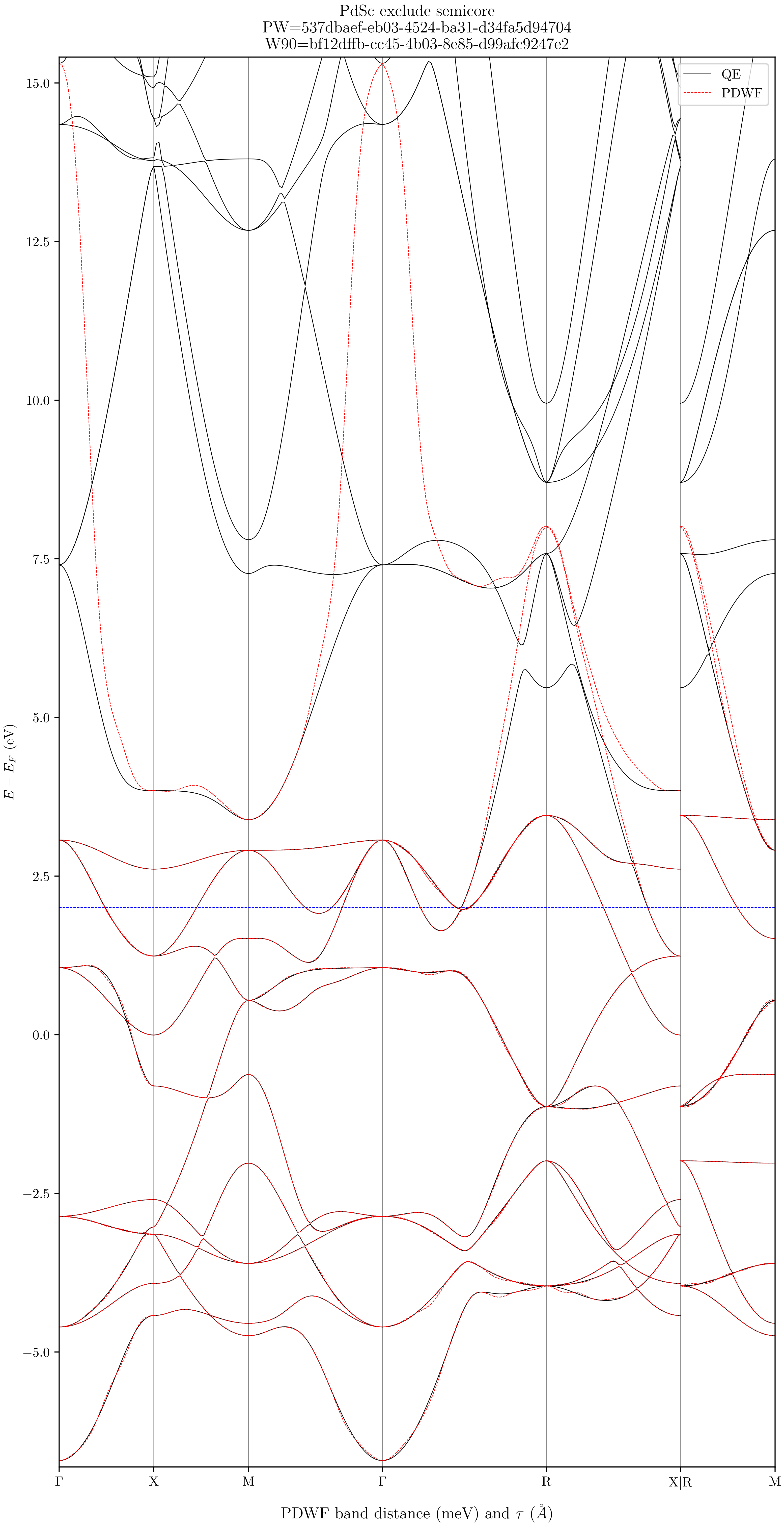

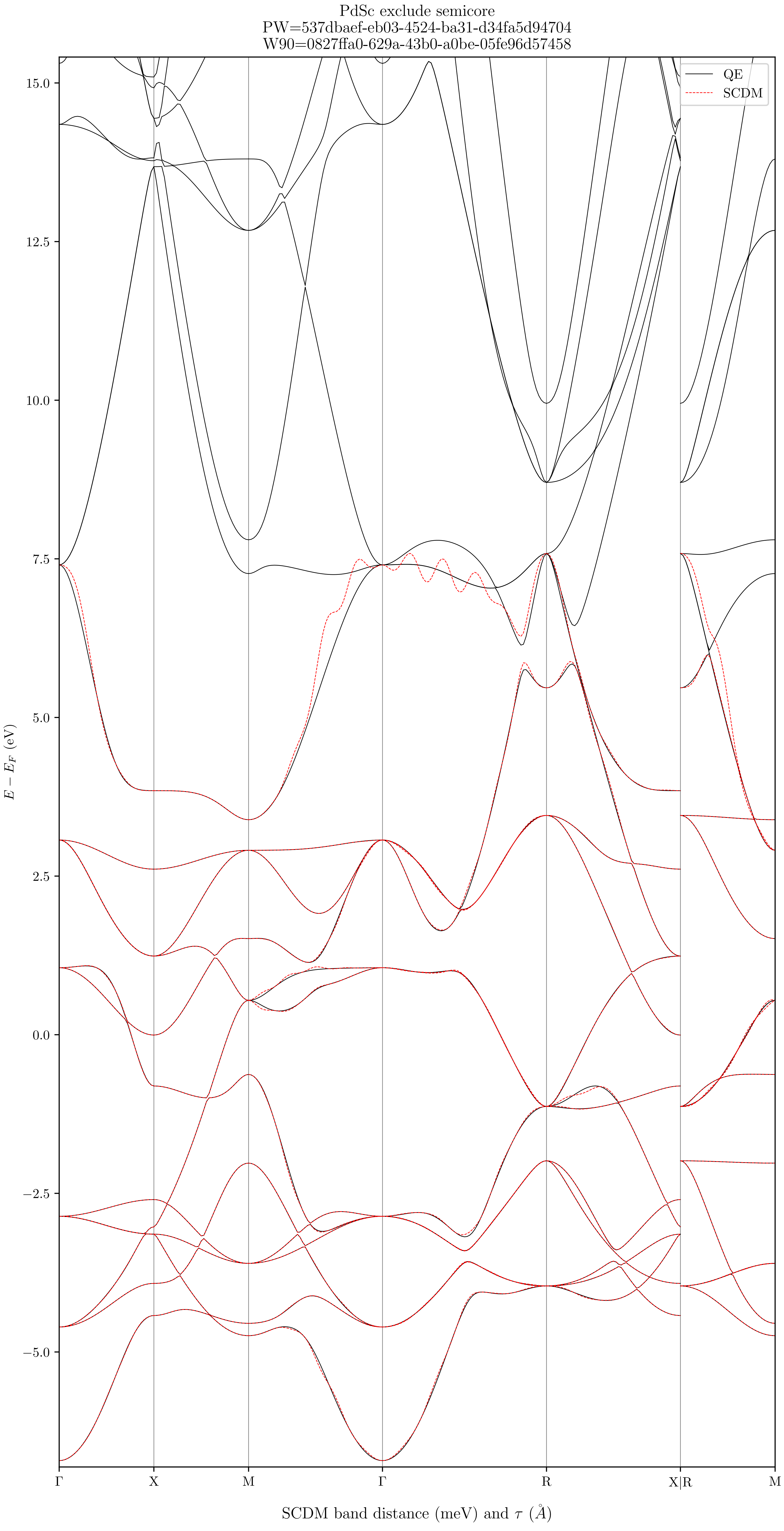

PDWF band distance (meV) and τ ($\AA$)

η_0	η_2	η_4	$\eta_0^{\max}$	$\eta_2^{\max}$	$\eta_4^{\max}$	τ
10.8	10.6	21.4	75.7	75.7	244.3	2.411

SCDM band distance (meV) and τ ($\AA$)

η_0	η_2	η_4	$\eta_0^{\max}$	$\eta_2^{\max}$	$\eta_4^{\max}$	τ
8.5	9.2	9.5	68.2	68.2	77.2	2.243

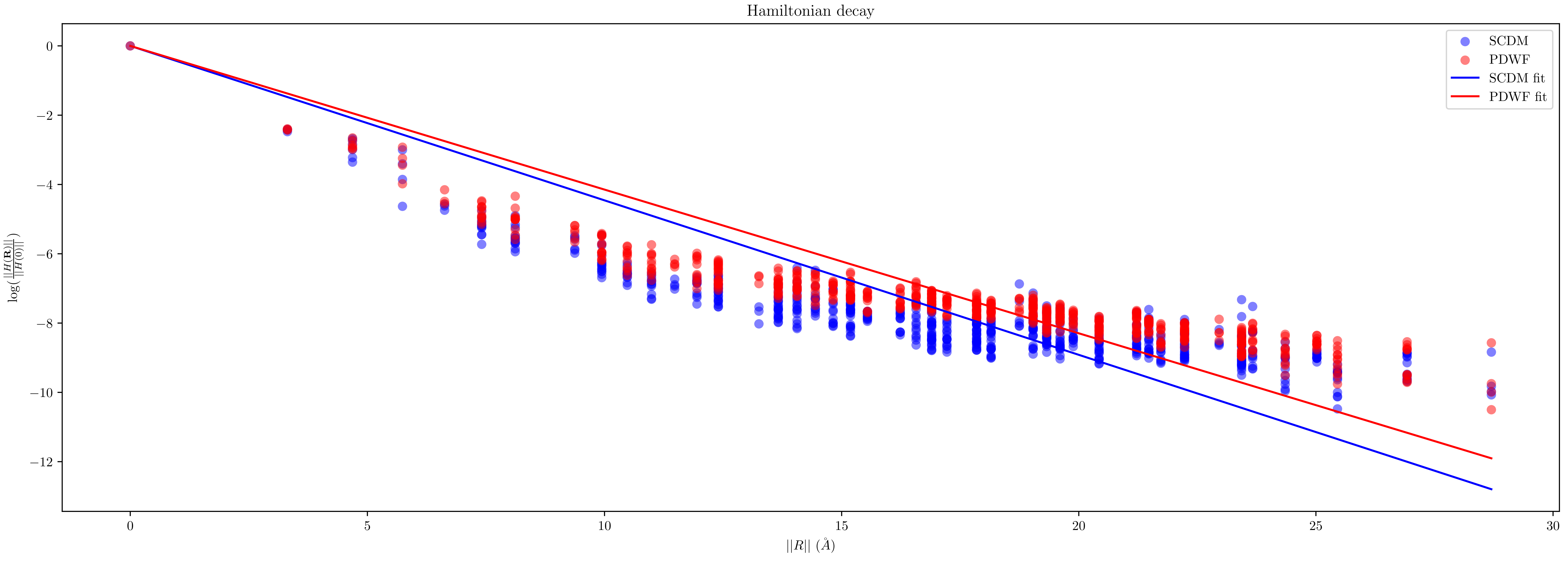

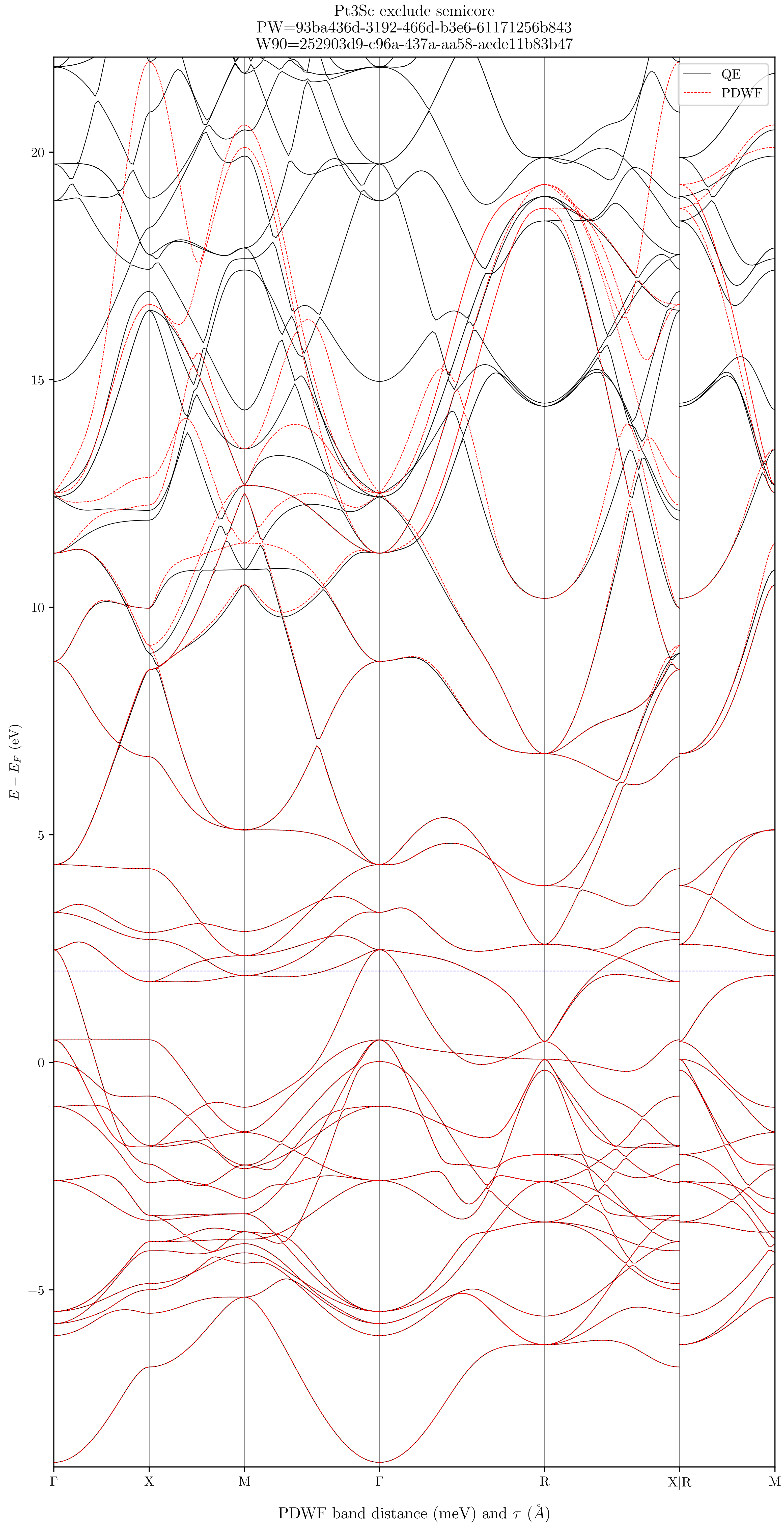

PDWF band distance (meV) and τ ($\AA$)

η_0	η_2	η_4	$\eta_0^{\max}$	$\eta_2^{\max}$	$\eta_4^{\max}$	τ
0.6	0.6	0.7	4.3	4.3	4.3	1.739

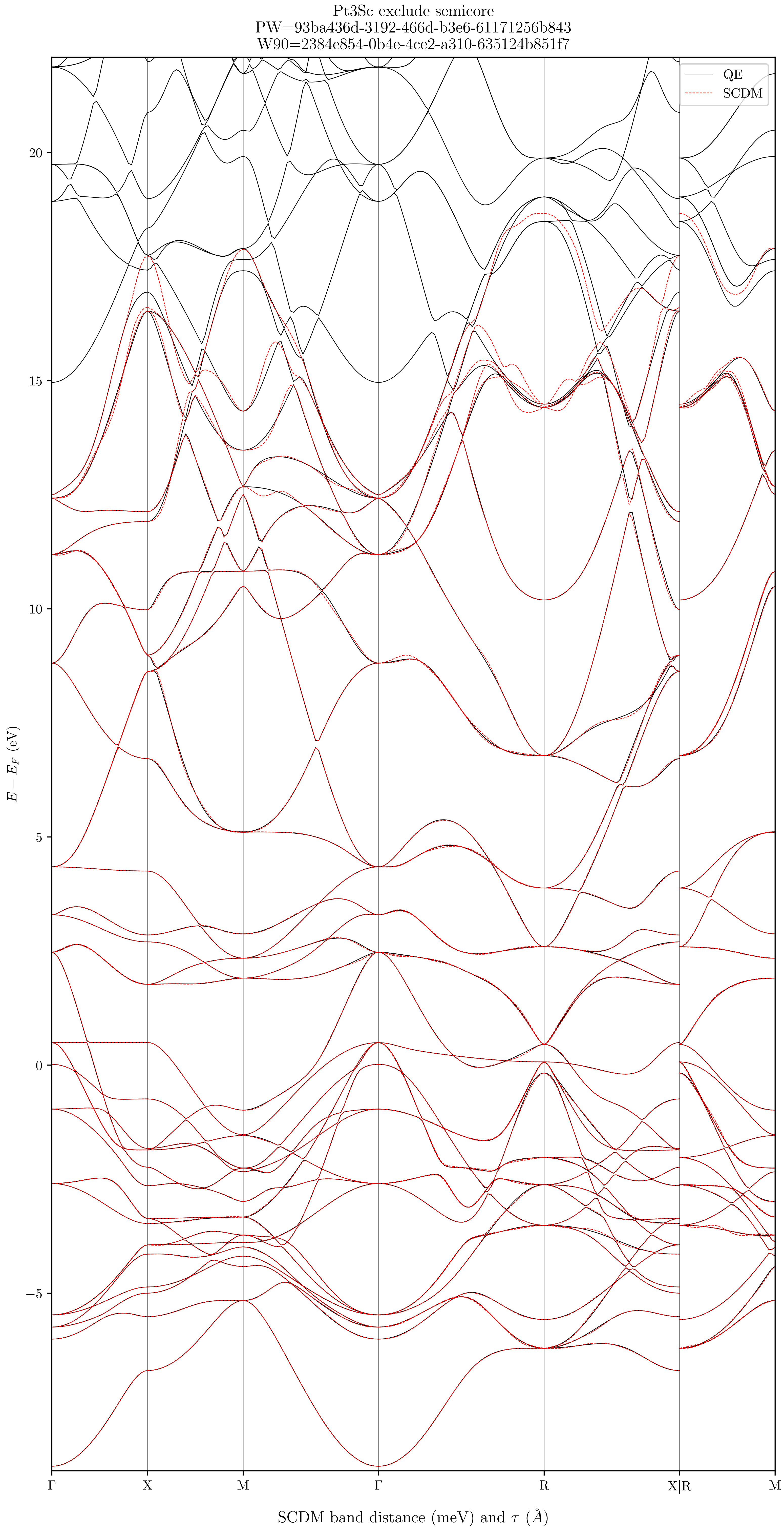

SCDM band distance (meV) and τ ($\AA$)

η_0	η_2	η_4	$\eta_0^{\max}$	$\eta_2^{\max}$	$\eta_4^{\max}$	τ
8.4	8.4	8.3	70.9	70.9	70.9	2.476

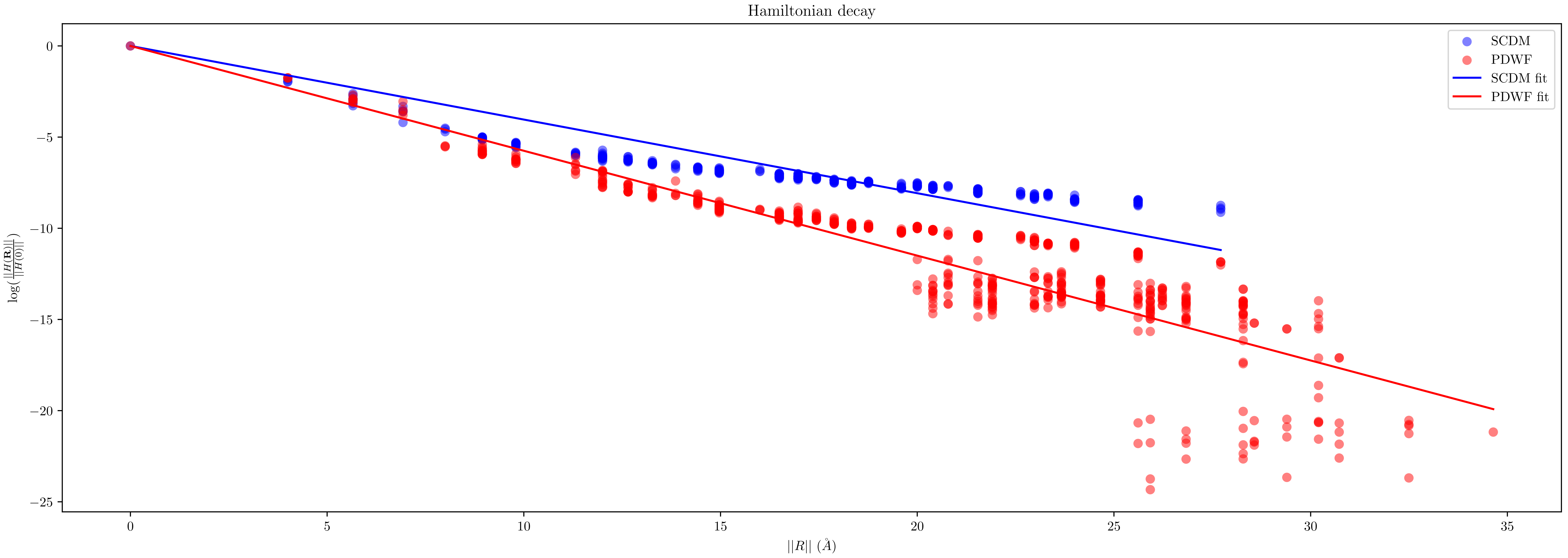

Pt3Y exclude semicore
PW=f22d8788-f913-402d-a789-c93d8e7ae2e6
W90=38df72cf-dbd0-492e-b99b-aa7f24eb38d4

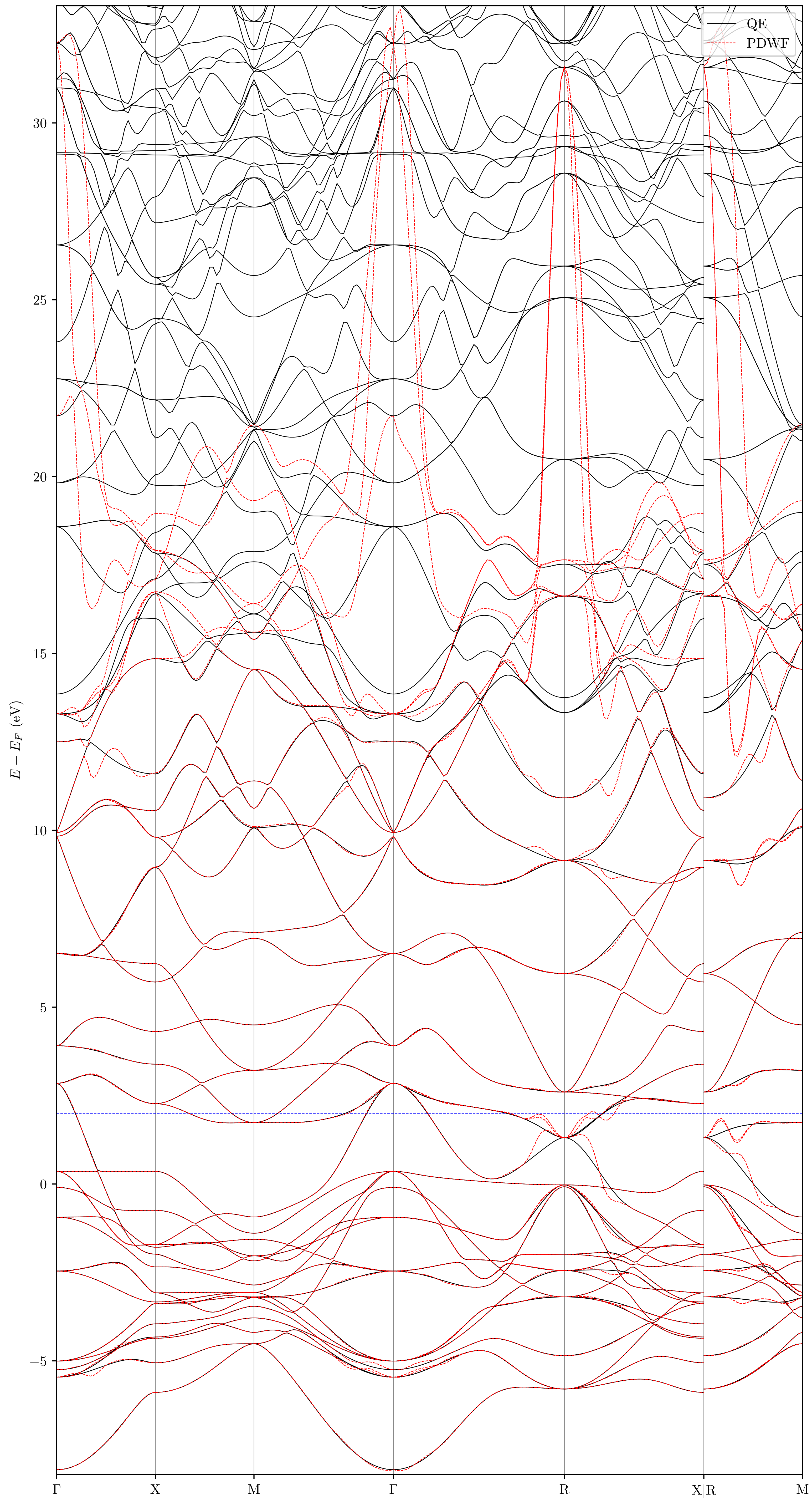

PDWF band distance (meV) and τ ($\AA$)

η_0	η_2	η_4	$\eta_0^{\max}$	$\eta_2^{\max}$	$\eta_4^{\max}$	τ
23.2	45.6	43.9	271.6	503.3	505.2	1.987

Pt3Y exclude semicore
PW=f22d8788-f913-402d-a789-c93d8e7ae2e6
W90=780c7715-4041-46b9-b4fd-f15cffa60222

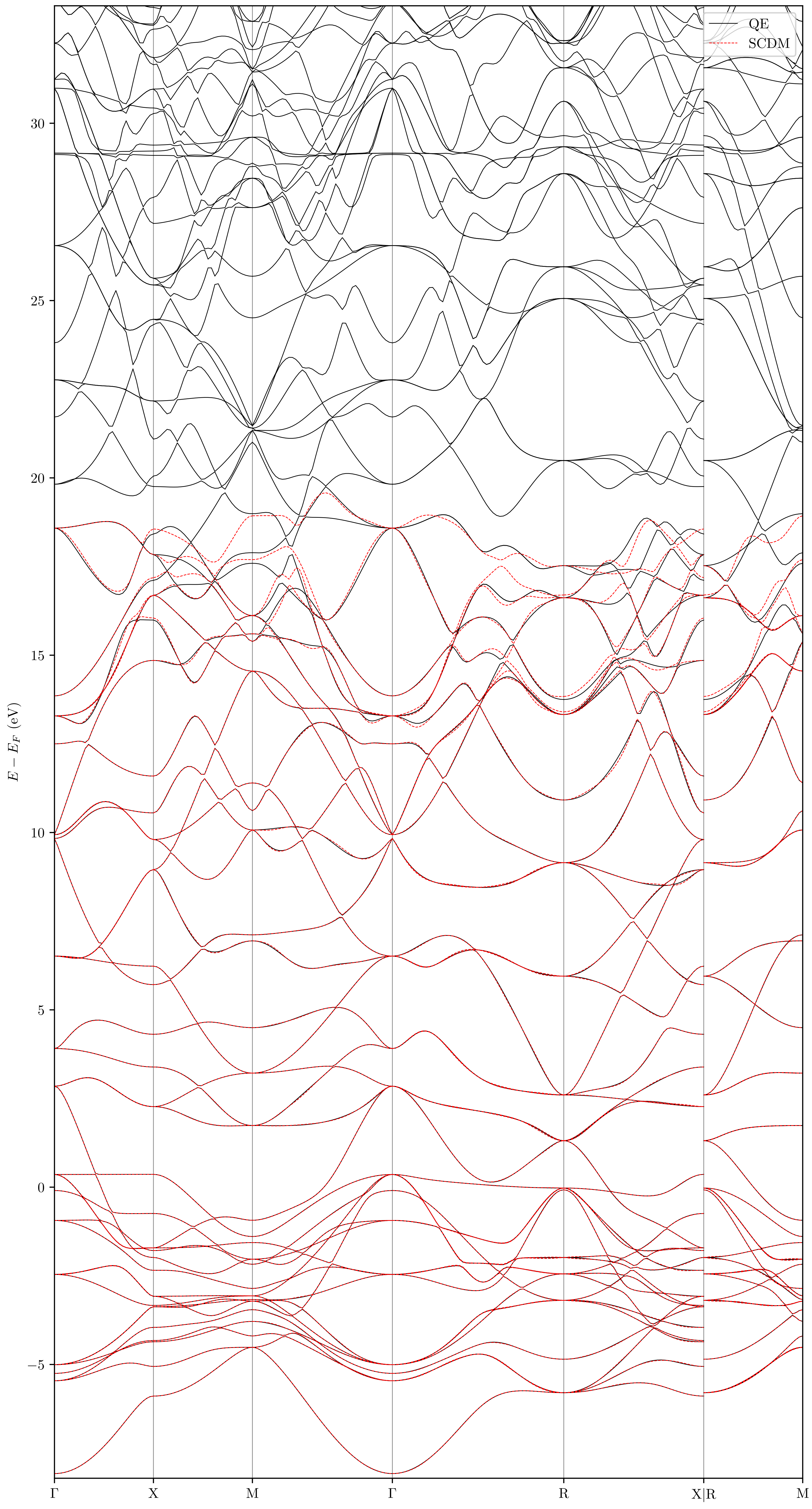

SCDM band distance (meV) and τ ($\AA$)

η_0	η_2	η_4	$\eta_0^{\max}$	$\eta_2^{\max}$	$\eta_4^{\max}$	τ
5.0	5.0	5.3	26.5	31.3	32.7	2.521

Hamiltonian decay

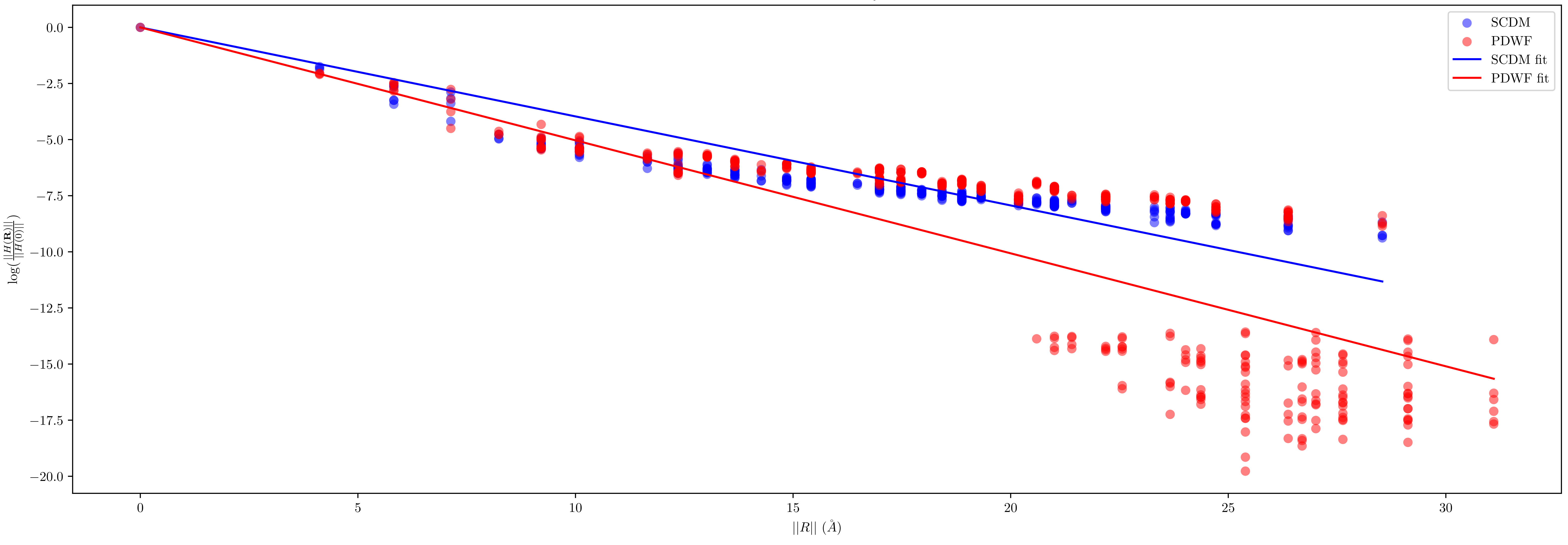

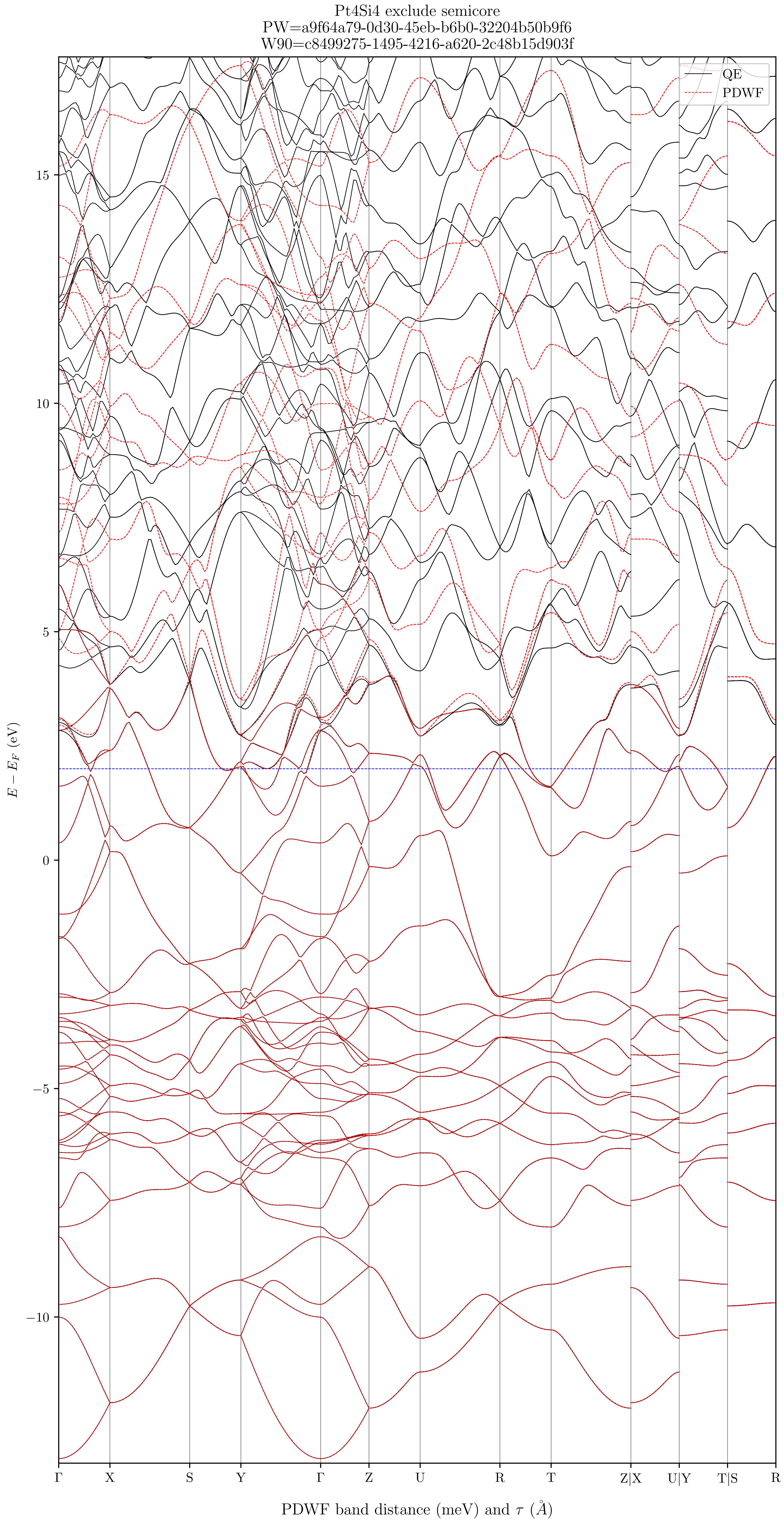

η_0	η_2	η_4	$\eta_0^{\max}$	$\eta_2^{\max}$	$\eta_4^{\max}$	τ
1.2	1.4	25.7	8.6	11.2	256.9	2.322

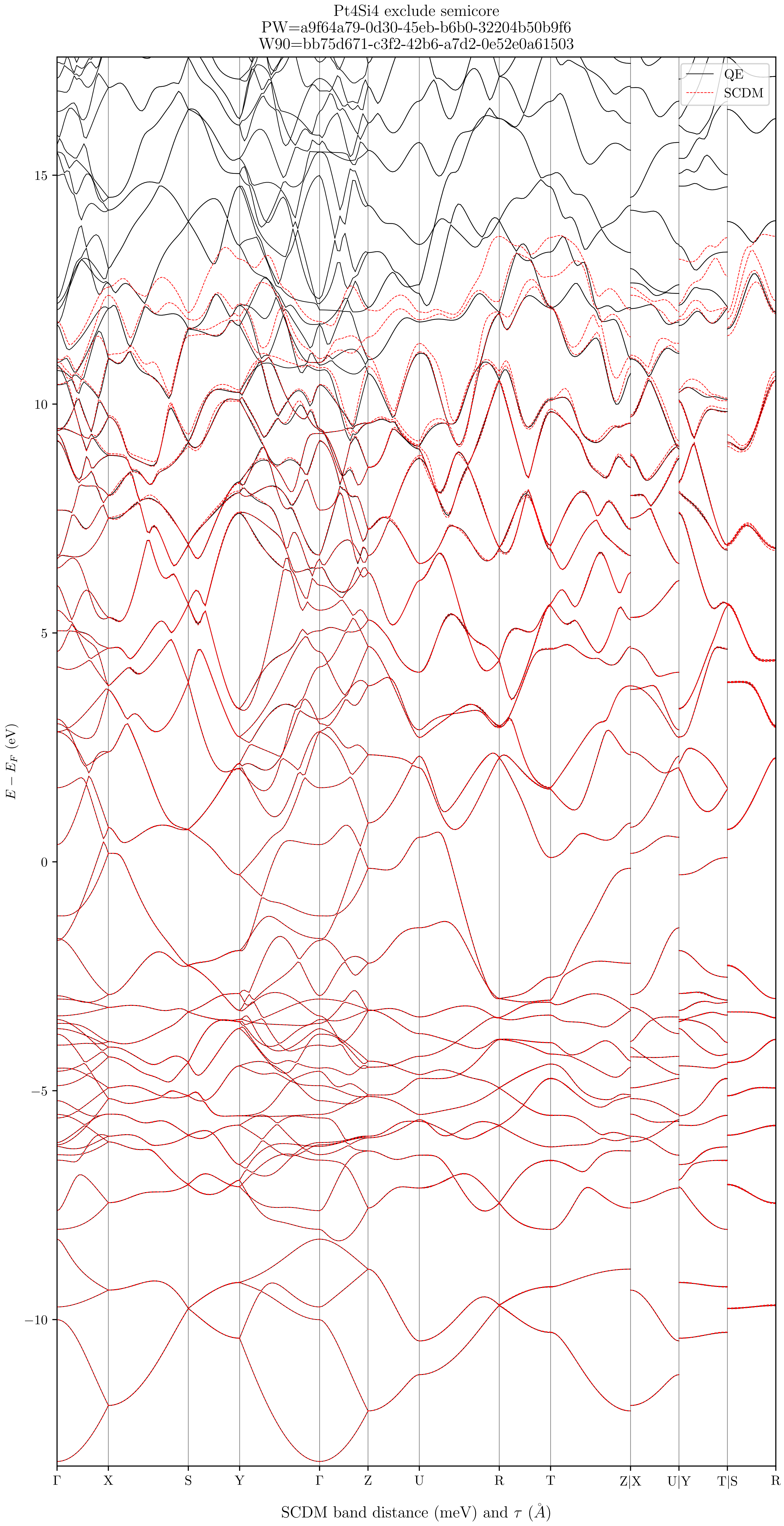

η_0	η_2	η_4	$\eta_0^{\max}$	$\eta_2^{\max}$	$\eta_4^{\max}$	τ
3.3	3.5	4.0	22.3	22.3	34.6	2.727

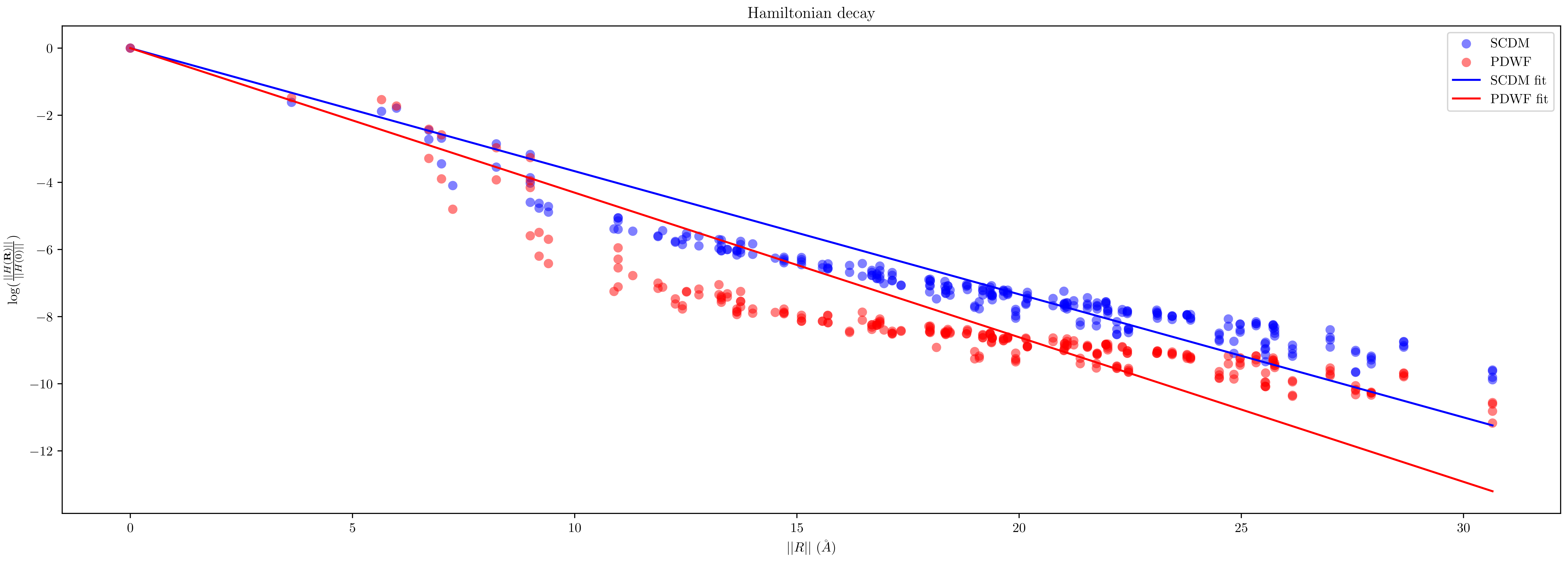

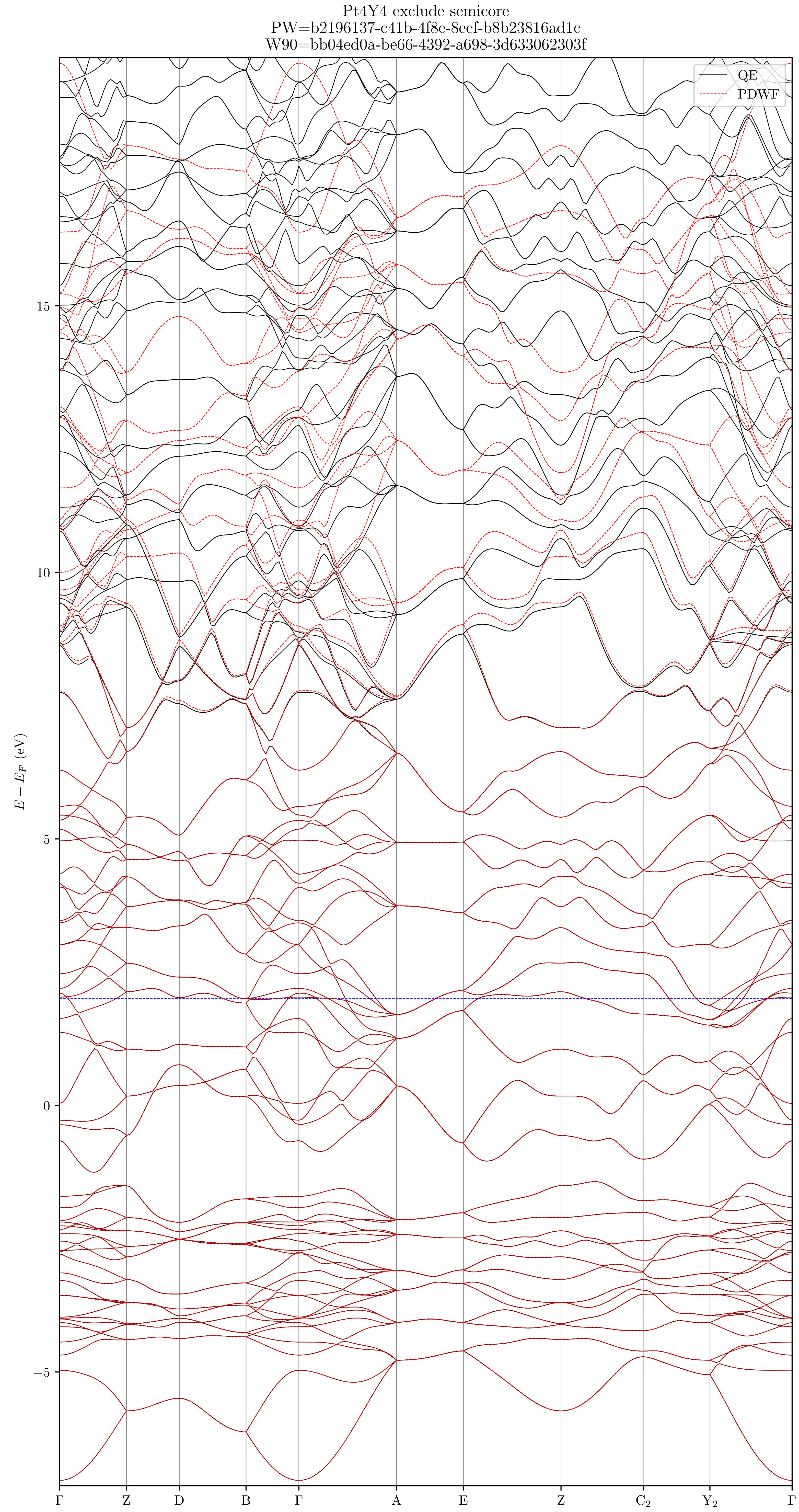

PDWF band distance (meV) and τ (Å)

η_0	η_2	η_4	$\eta_0^{\max}$	$\eta_2^{\max}$	$\eta_4^{\max}$	τ
0.5	0.7	0.7	3.7	5.5	5.5	2.247

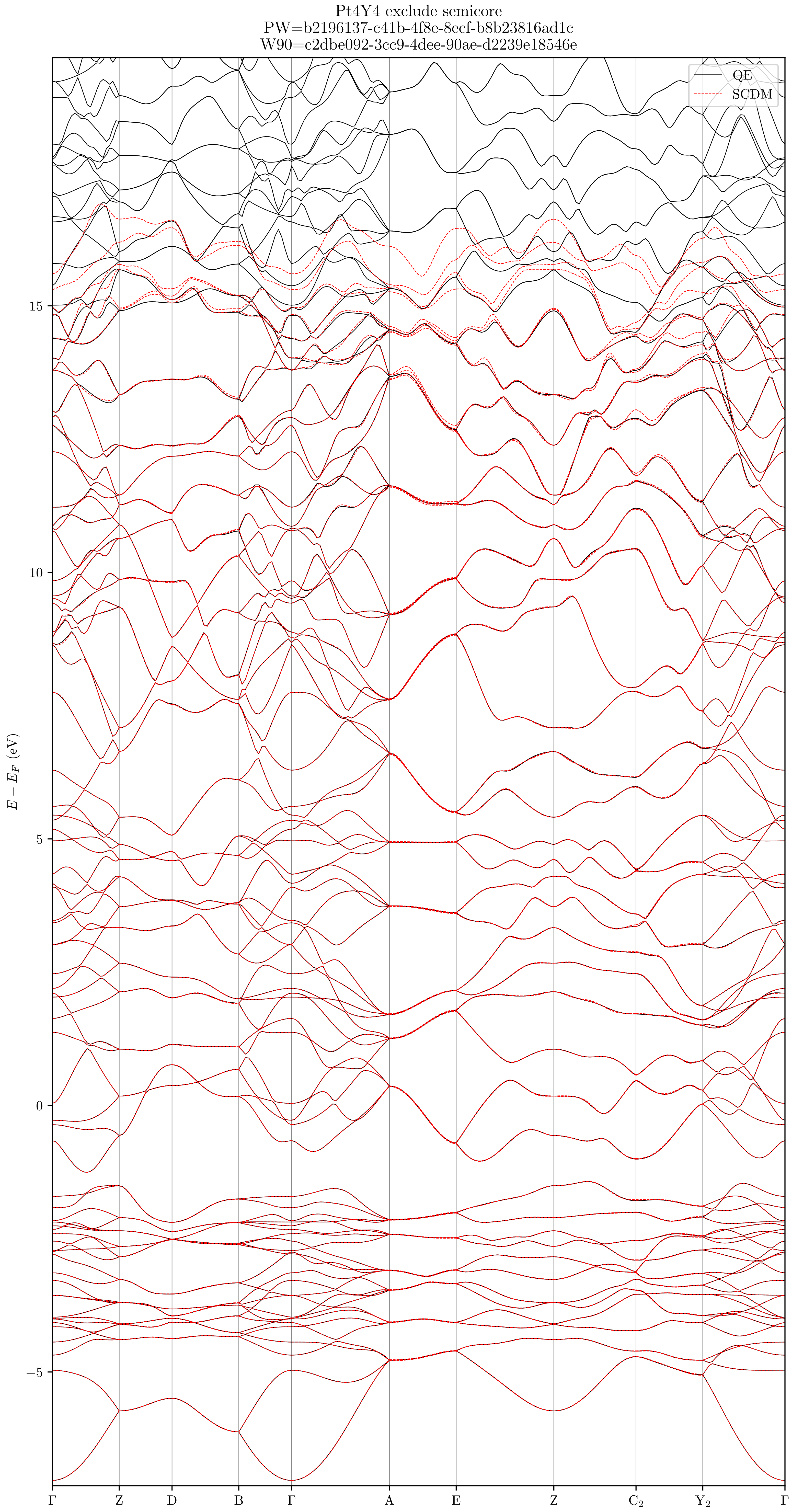

SCDM band distance (meV) and τ (Å)

η_0	η_2	η_4	$\eta_0^{\max}$	$\eta_2^{\max}$	$\eta_4^{\max}$	τ
2.8	3.2	3.5	20.7	20.7	32.3	2.708

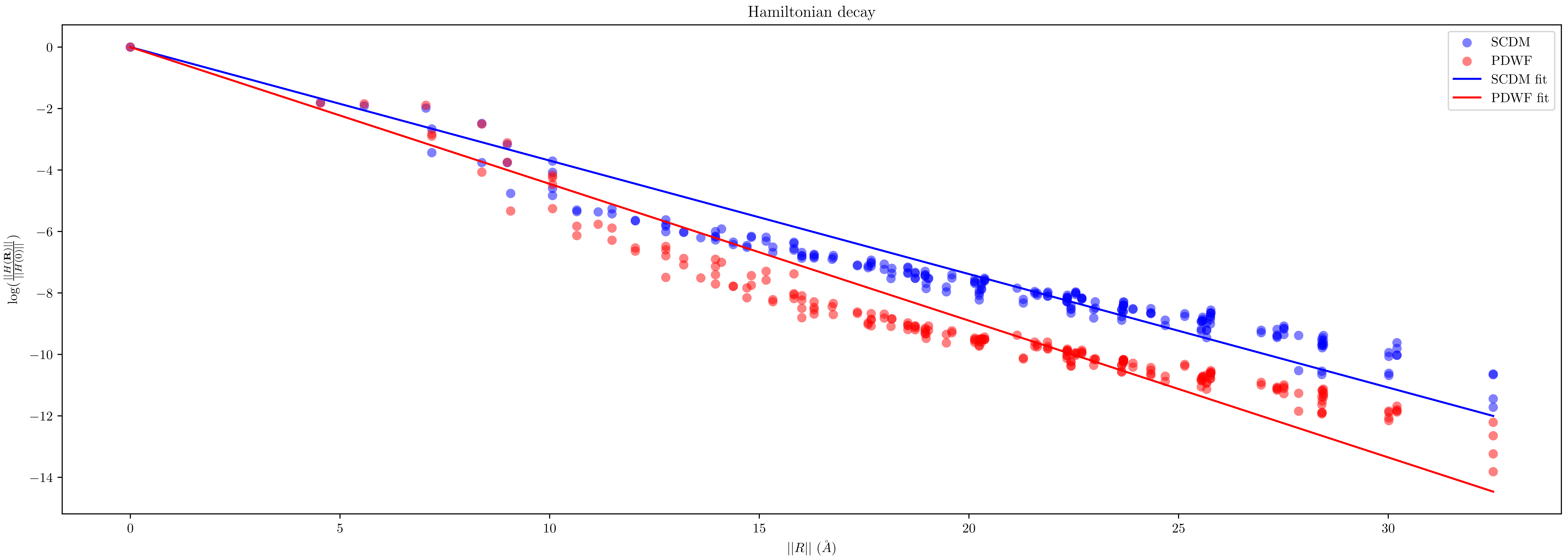

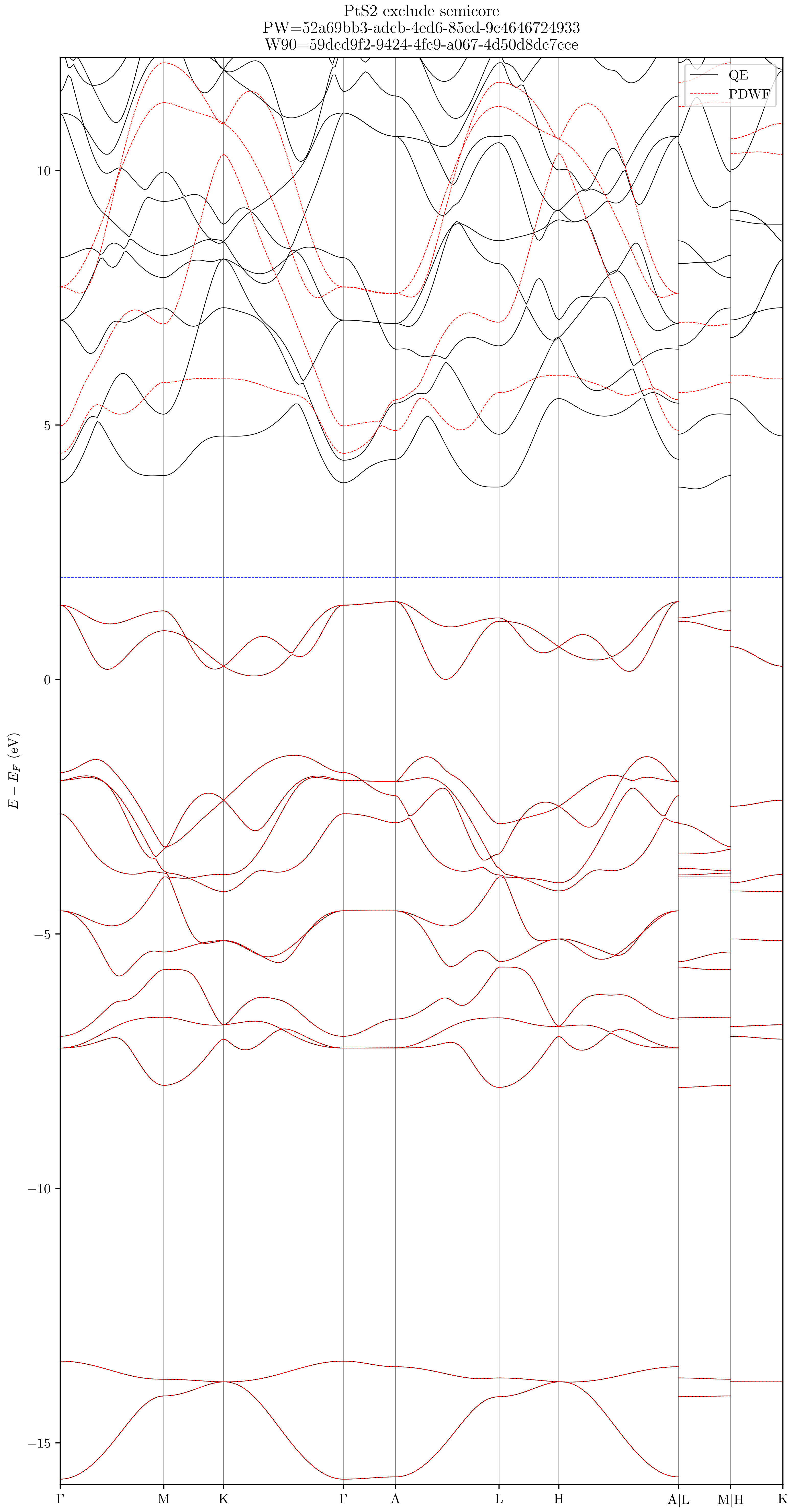

PDWF band distance (meV) and τ ($\AA$)

η_0	η_2	η_4	$\eta_0^{\max}$	$\eta_2^{\max}$	$\eta_4^{\max}$	τ
0.6	0.6	0.8	4.6	4.6	4.6	2.184

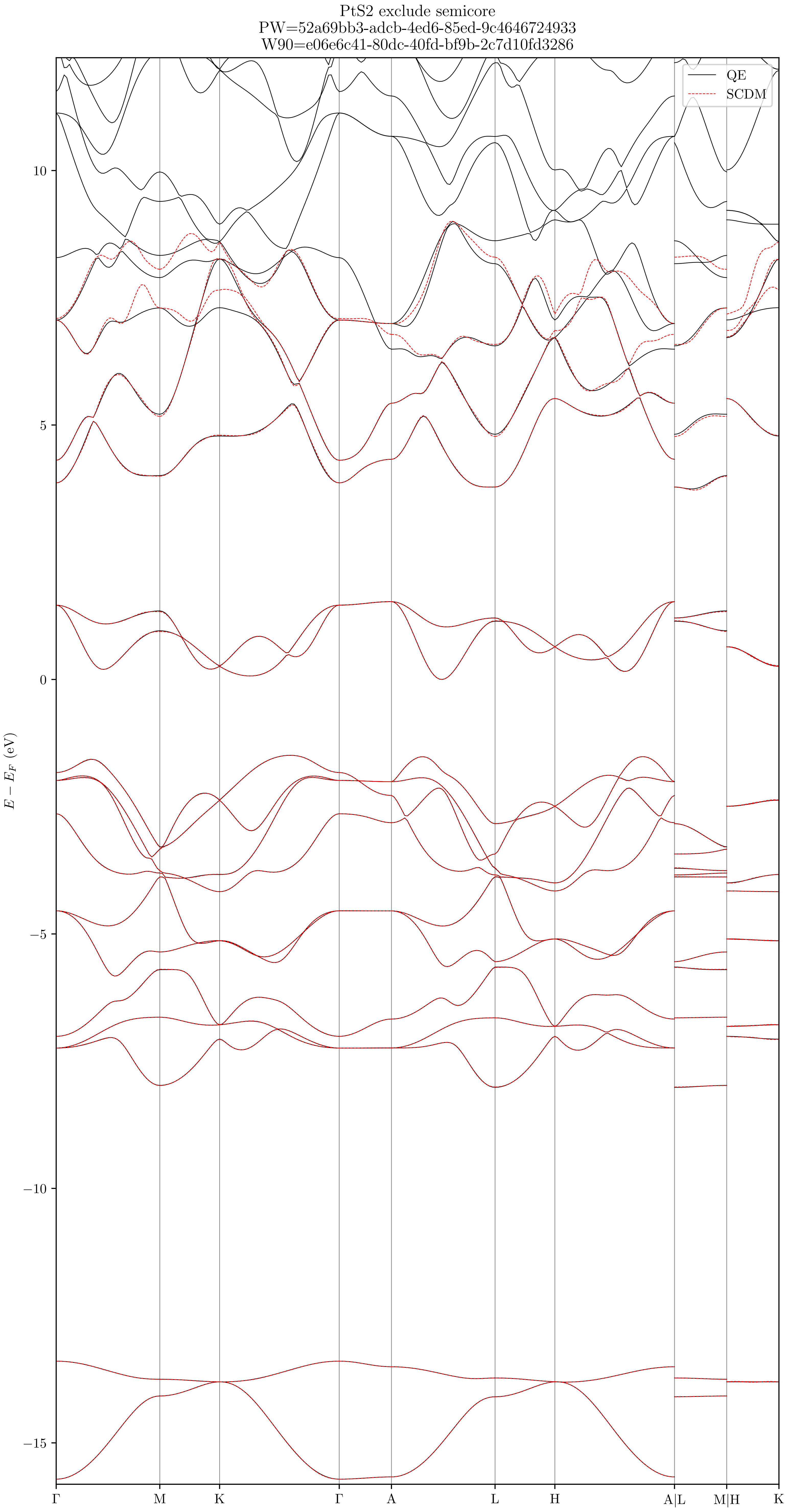

SCDM band distance (meV) and τ ($\AA$)

η_0	η_2	η_4	$\eta_0^{\max}$	$\eta_2^{\max}$	$\eta_4^{\max}$	τ
2.4	2.7	2.7	11.1	14.3	14.8	2.747

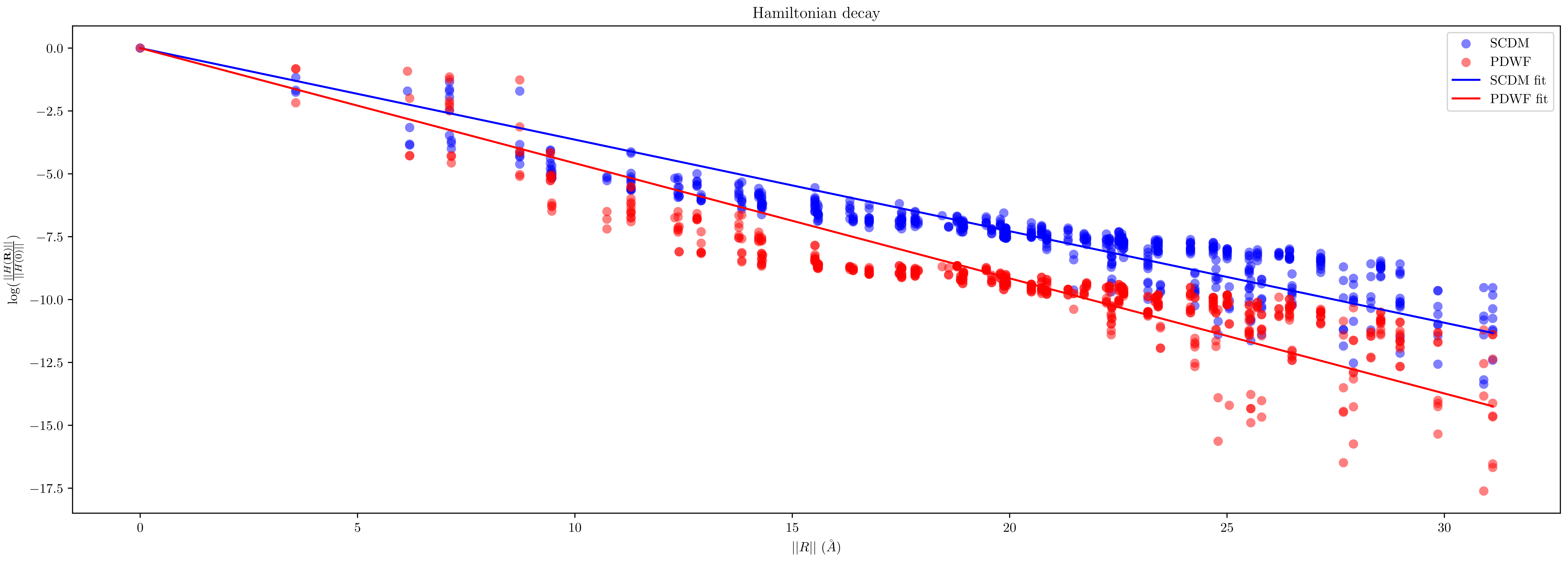

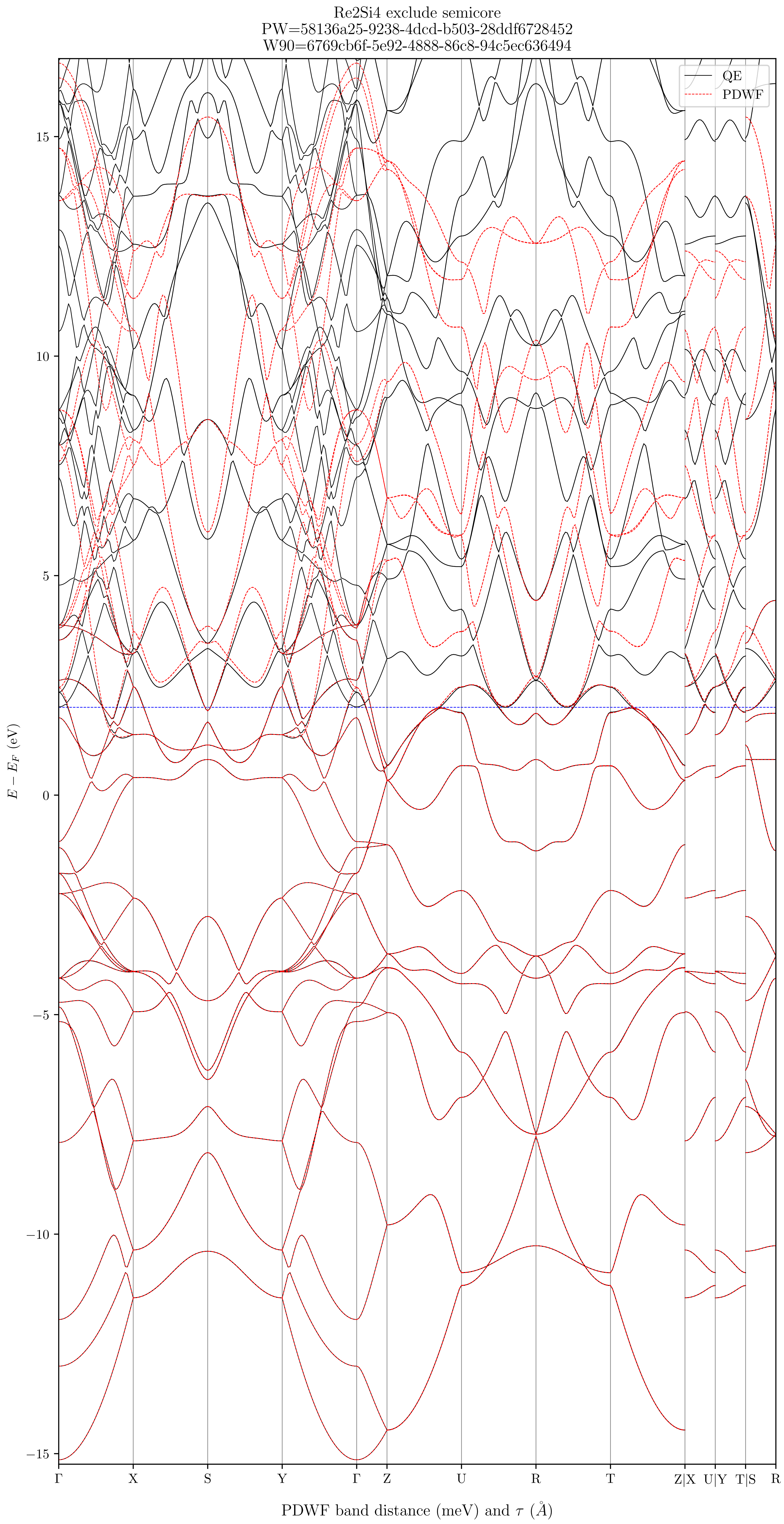

η_0	η_2	η_4	$\eta_0^{\max}$	$\eta_2^{\max}$	$\eta_4^{\max}$	τ
2.5	6.9	122.1	17.0	86.2	1405.3	2.391

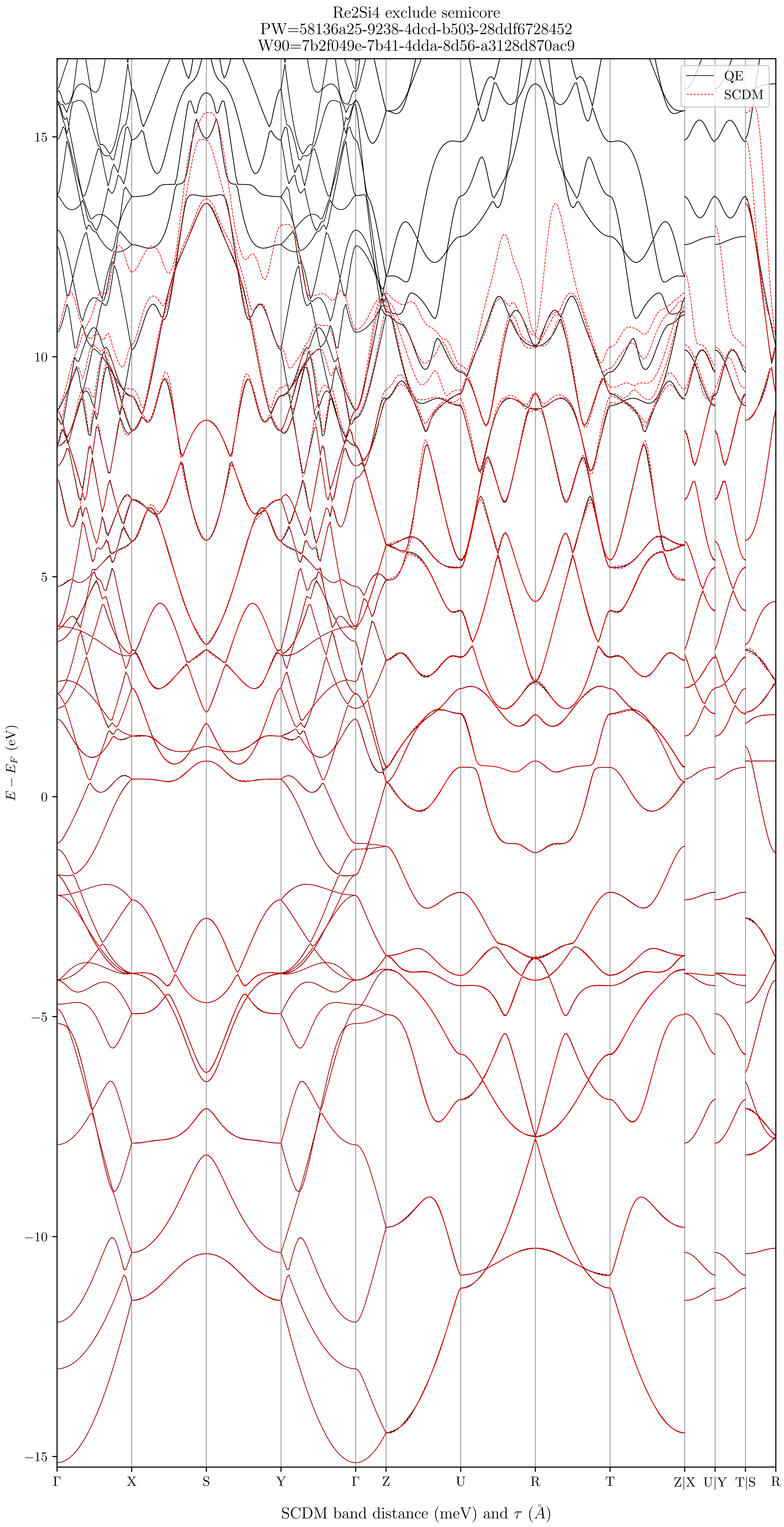

η_0	η_2	η_4	$\eta_0^{\max}$	$\eta_2^{\max}$	$\eta_4^{\max}$	τ
6.3	6.7	7.9	30.4	39.5	53.5	2.807

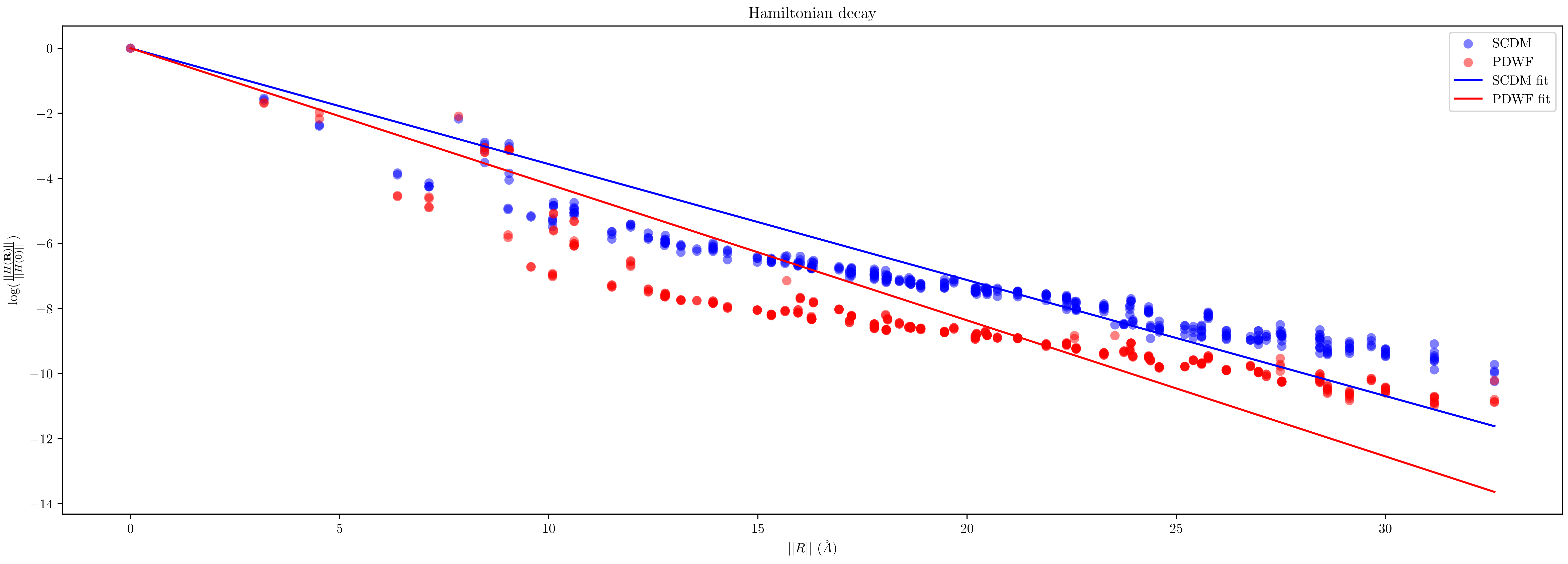

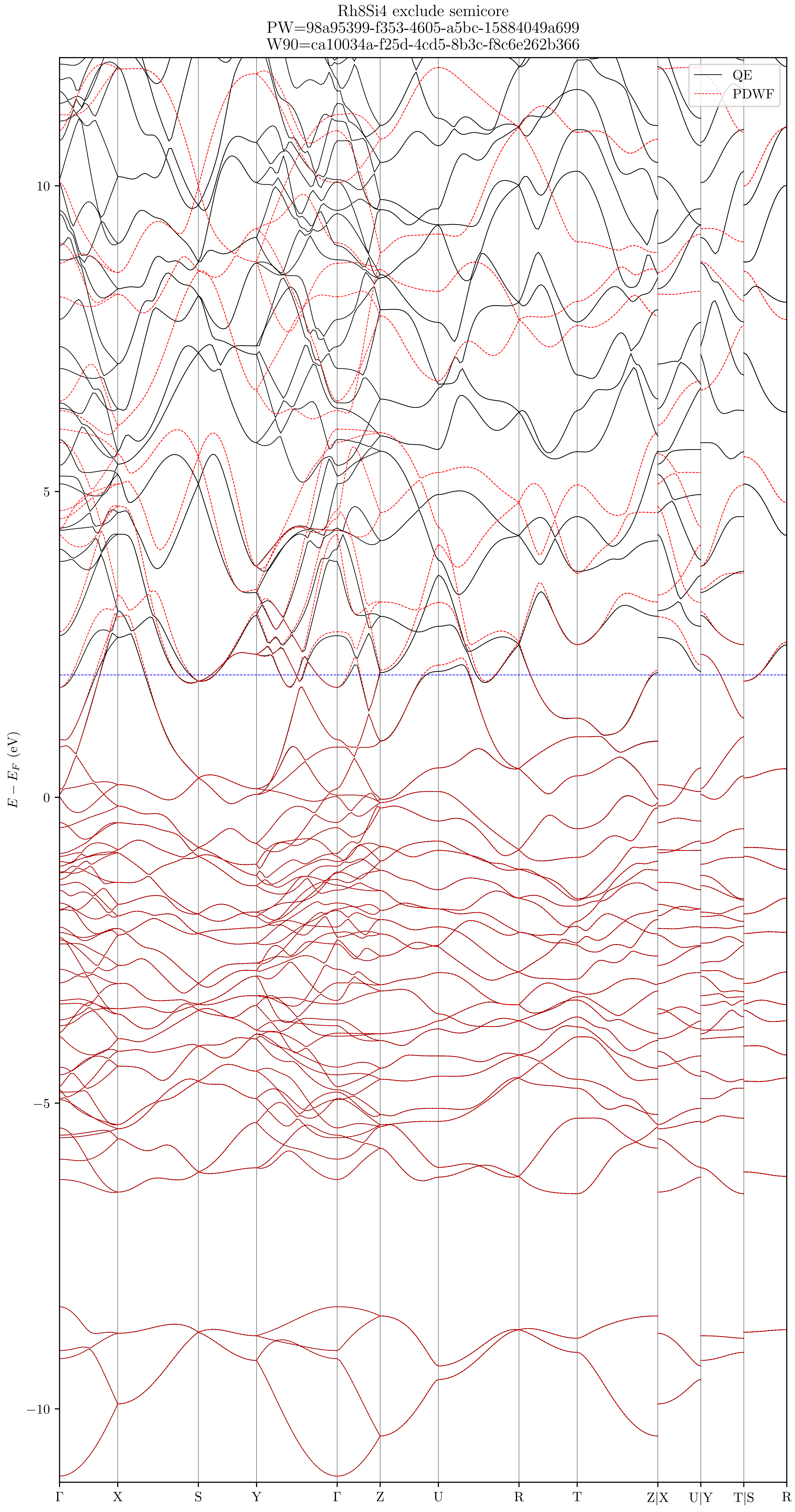

PDWF band distance (meV) and τ ($\AA$)

η_0	η_2	η_4	$\eta_0^{\max}$	$\eta_2^{\max}$	$\eta_4^{\max}$	τ
0.7	4.0	56.1	5.9	43.0	554.3	2.322

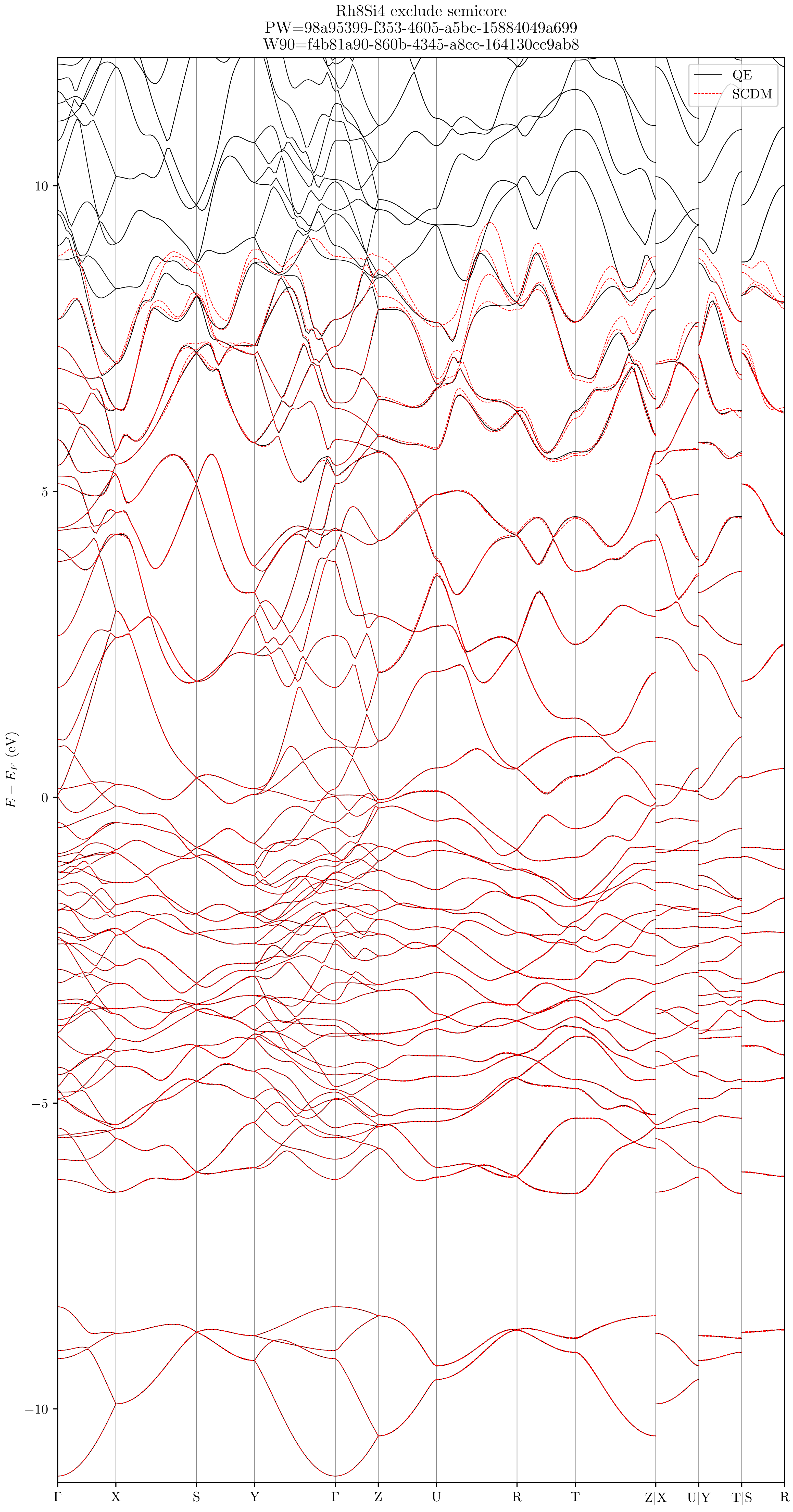

SCDM band distance (meV) and τ ($\AA$)

η_0	η_2	η_4	$\eta_0^{\max}$	$\eta_2^{\max}$	$\eta_4^{\max}$	τ
3.3	3.4	4.1	19.2	22.9	42.7	2.792

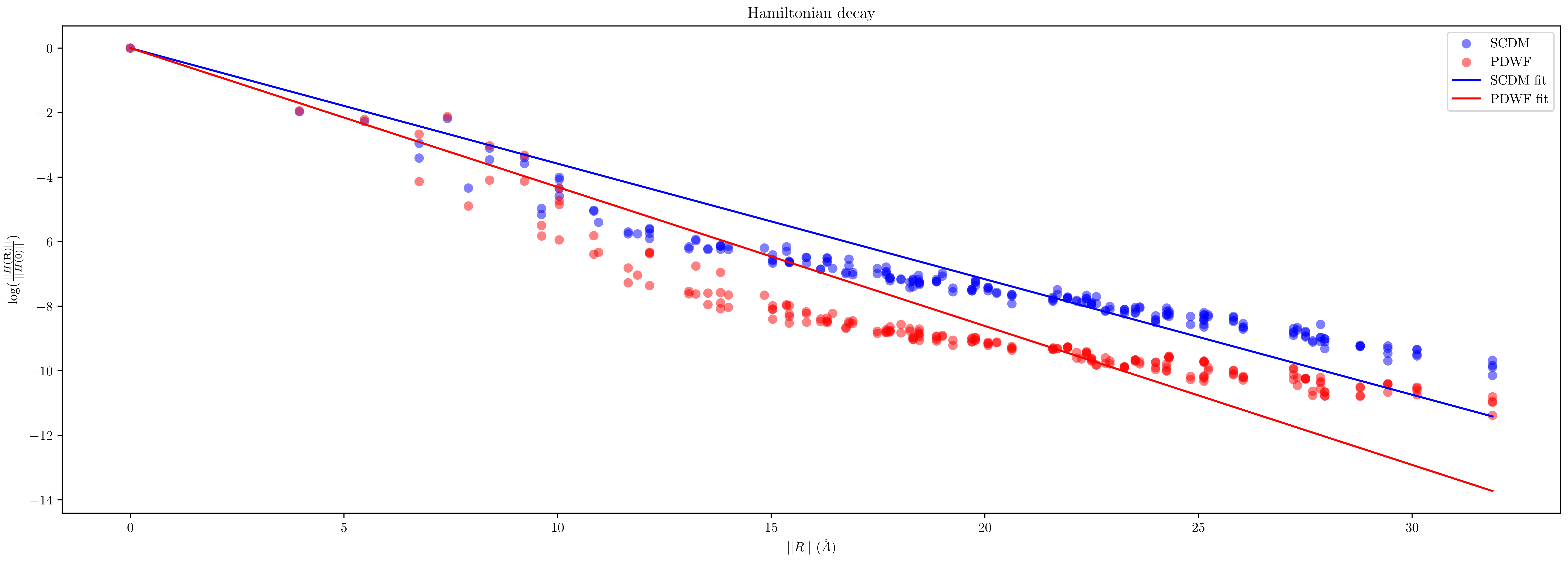

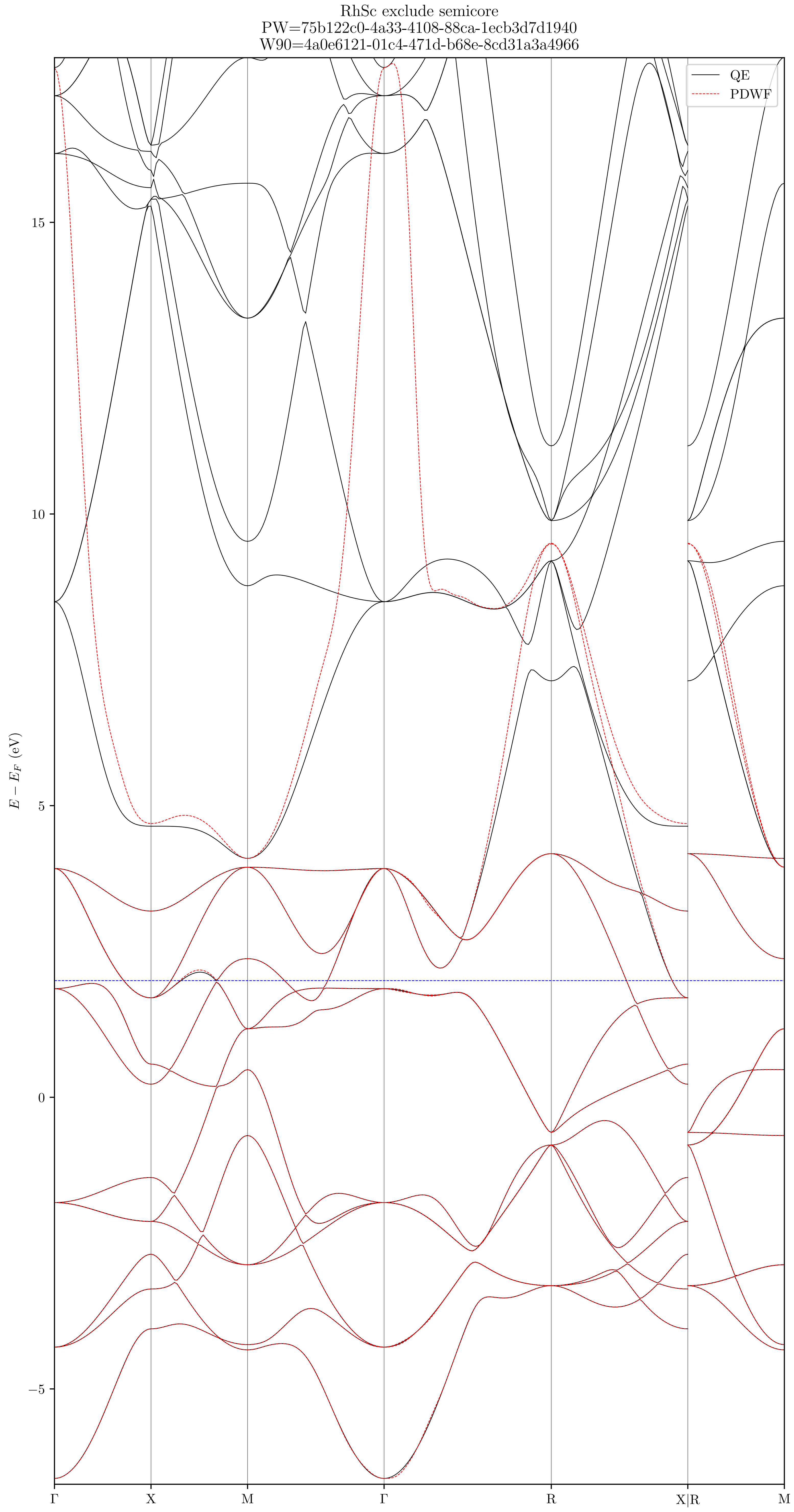

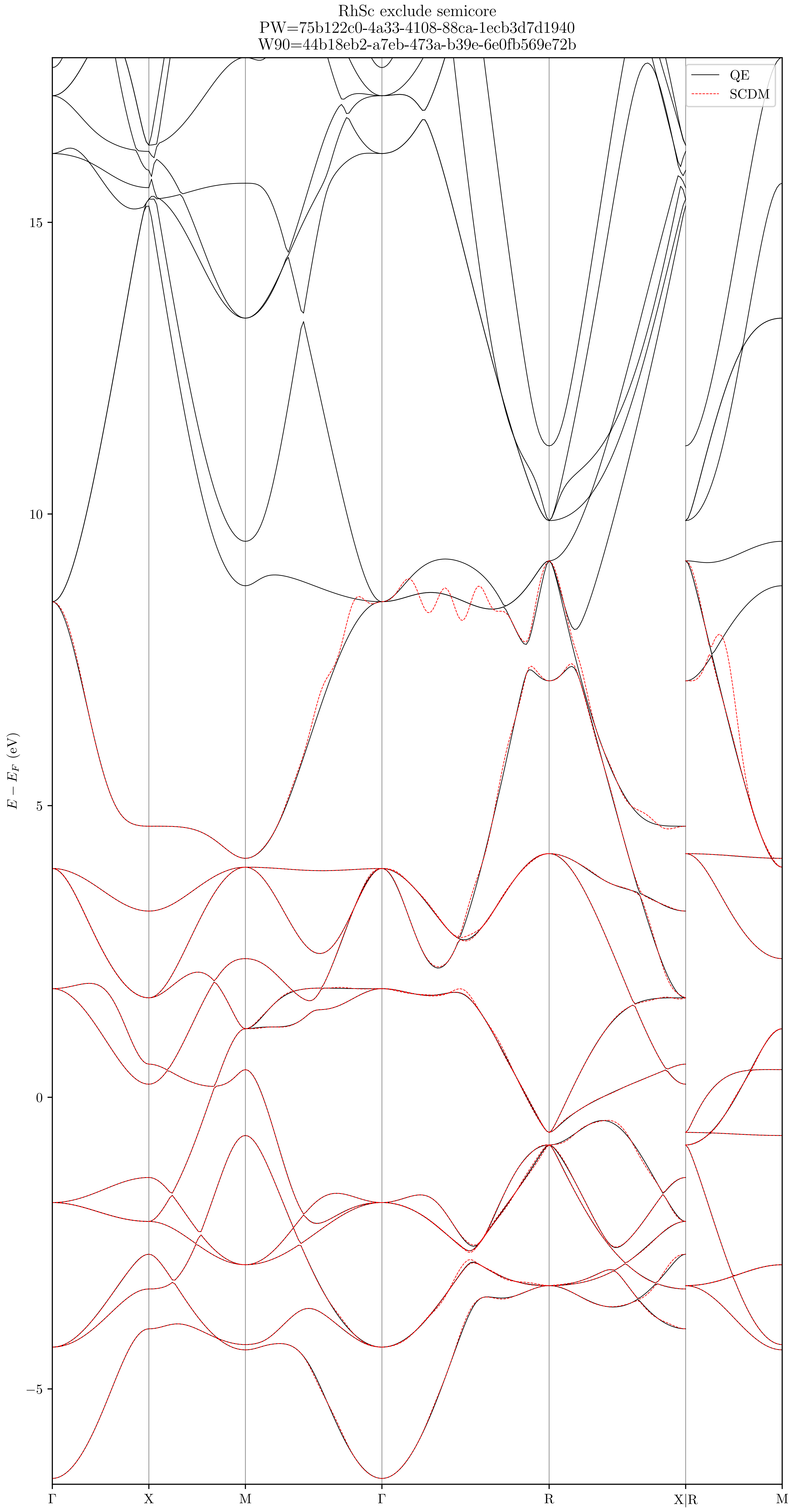

PDWF band distance (meV) and τ ($\AA$)

η_0	η_2	η_4	$\eta_0^{\max}$	$\eta_2^{\max}$	$\eta_4^{\max}$	τ
3.5	3.5	8.2	40.3	40.3	135.2	2.105

SCDM band distance (meV) and τ ($\AA$)

η_0	η_2	η_4	$\eta_0^{\max}$	$\eta_2^{\max}$	$\eta_4^{\max}$	τ
11.1	11.0	12.1	96.0	96.0	124.2	2.304

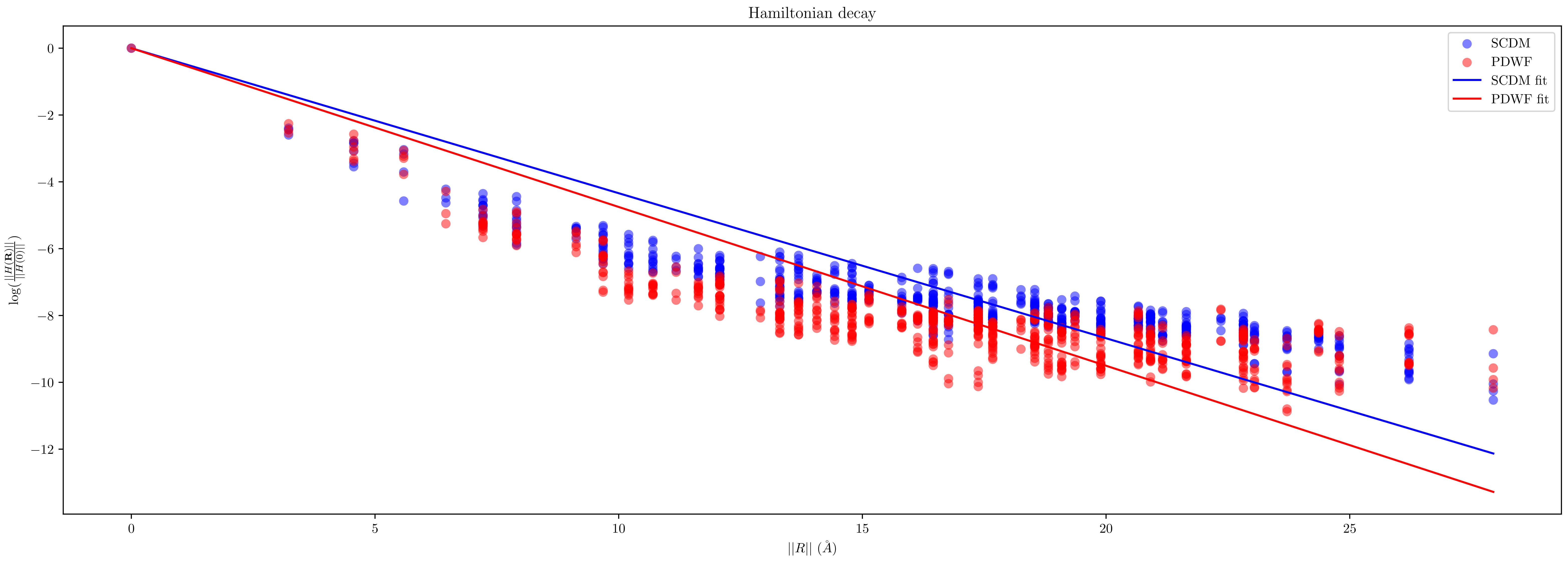

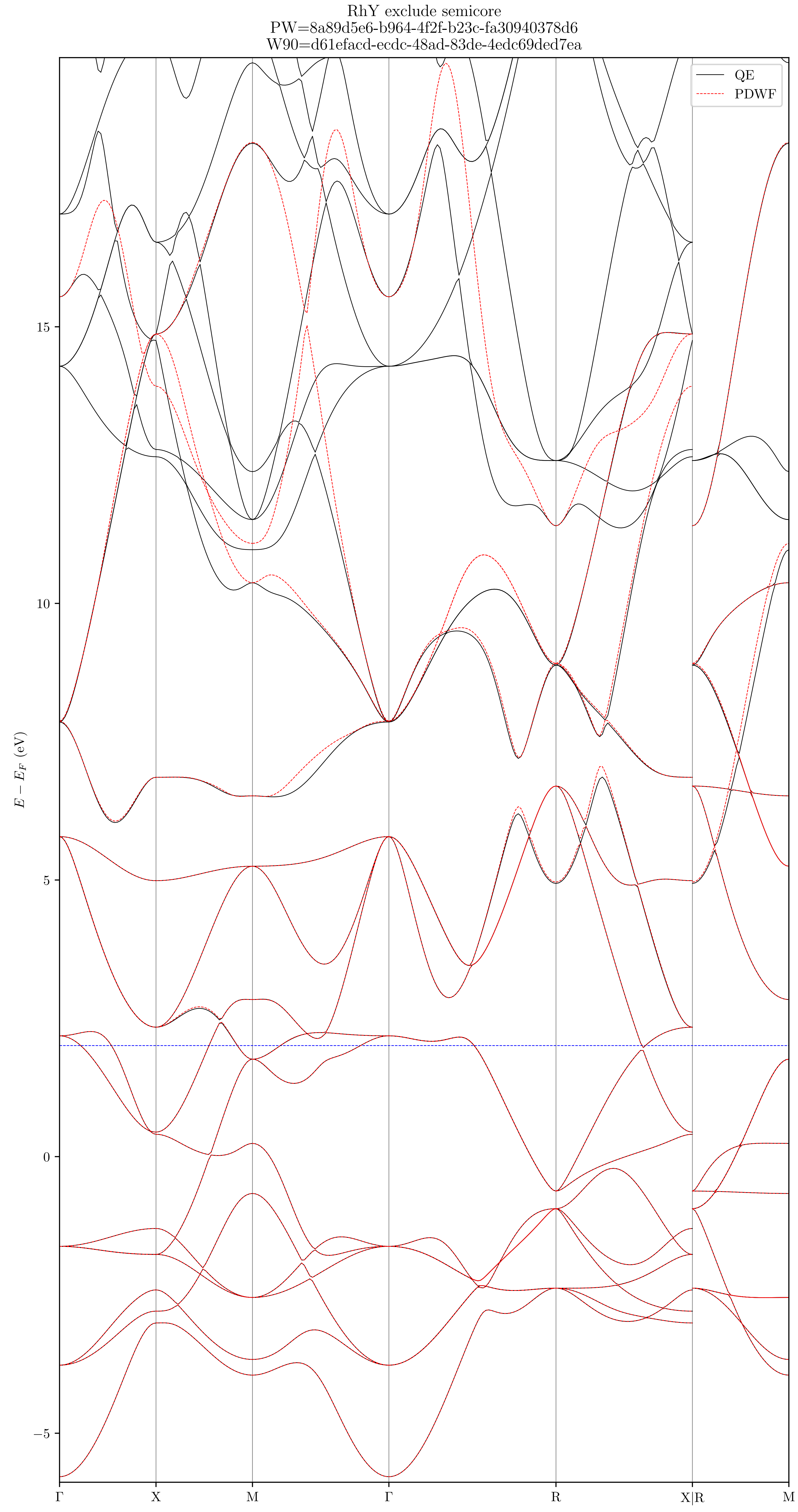

PDWF band distance (meV) and τ ($\AA$)

η_0	η_2	η_4	$\eta_0^{\max}$	$\eta_2^{\max}$	$\eta_4^{\max}$	τ
0.6	0.7	2.7	3.1	3.7	28.9	1.861

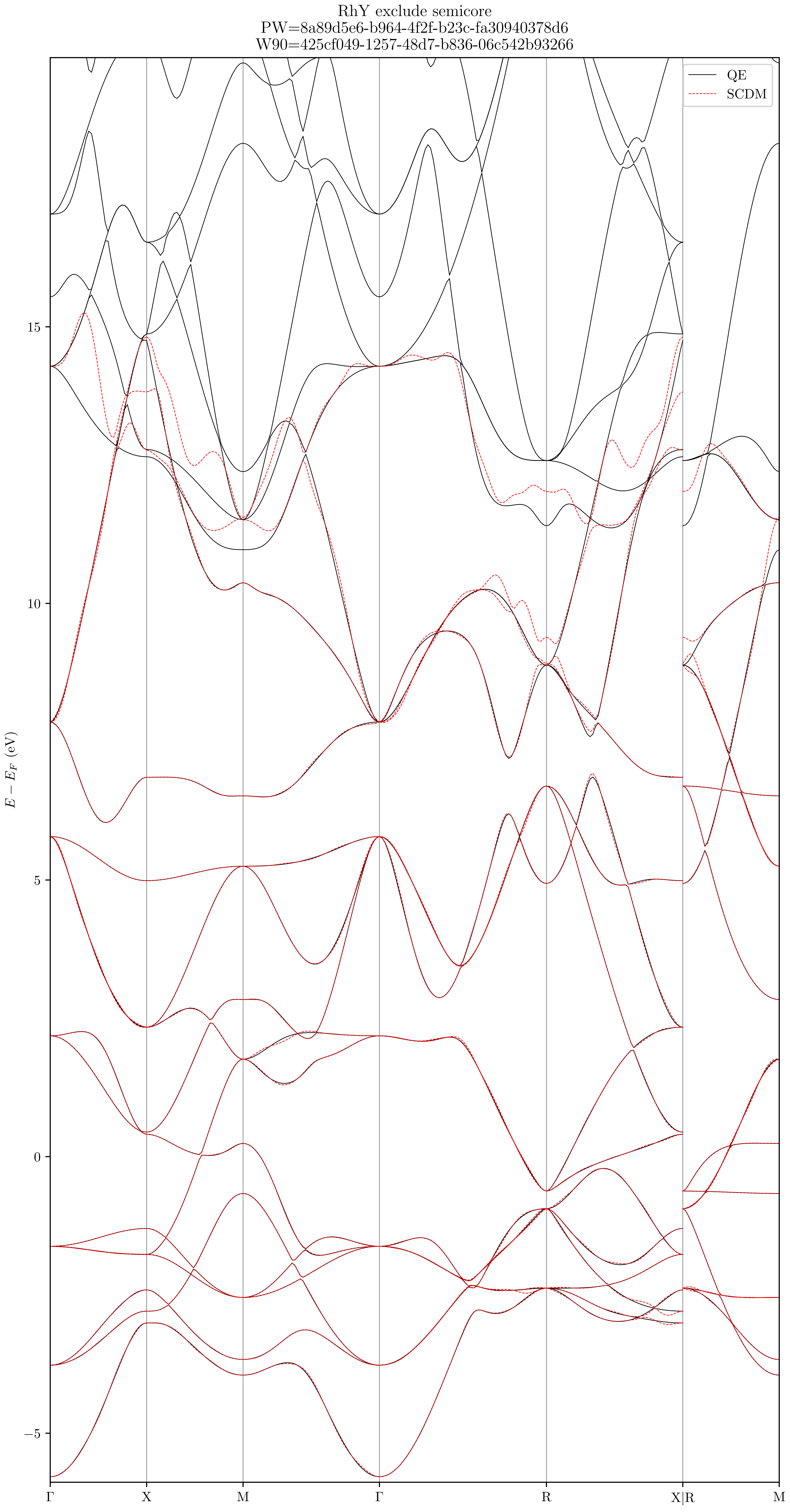

SCDM band distance (meV) and τ ($\AA$)

η_0	η_2	η_4	$\eta_0^{\max}$	$\eta_2^{\max}$	$\eta_4^{\max}$	τ
10.5	10.7	10.9	89.2	89.2	89.2	2.507

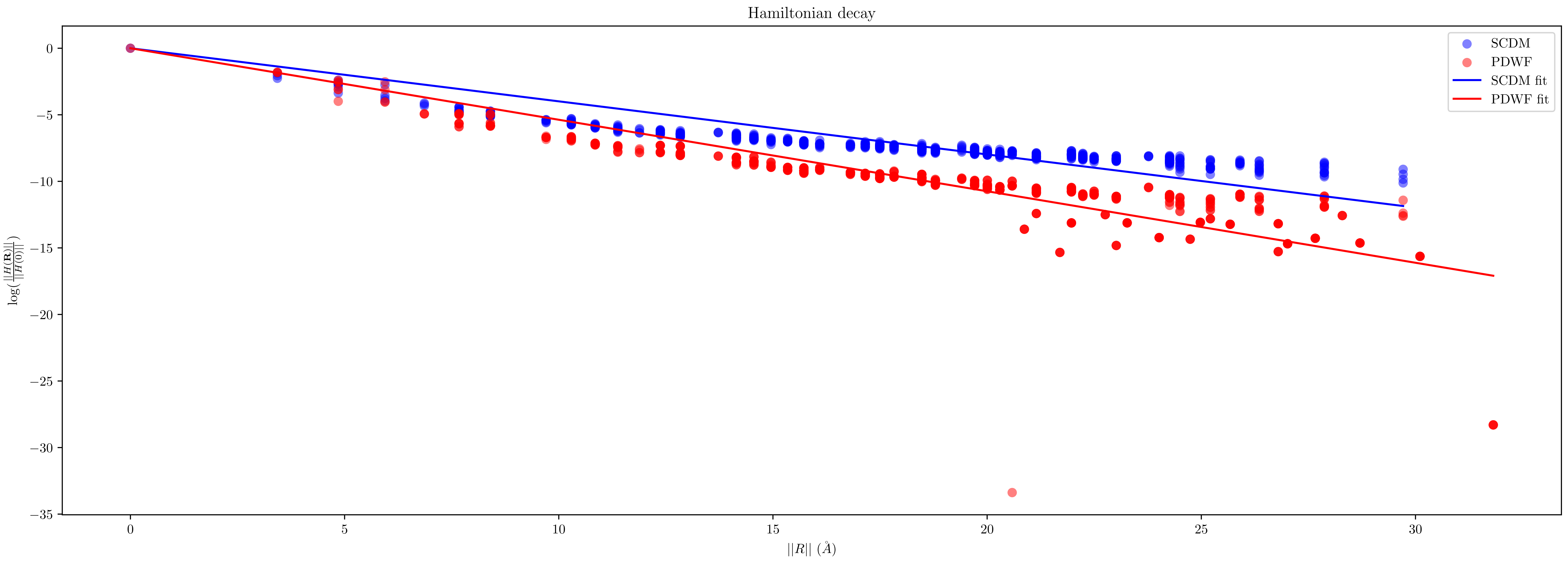

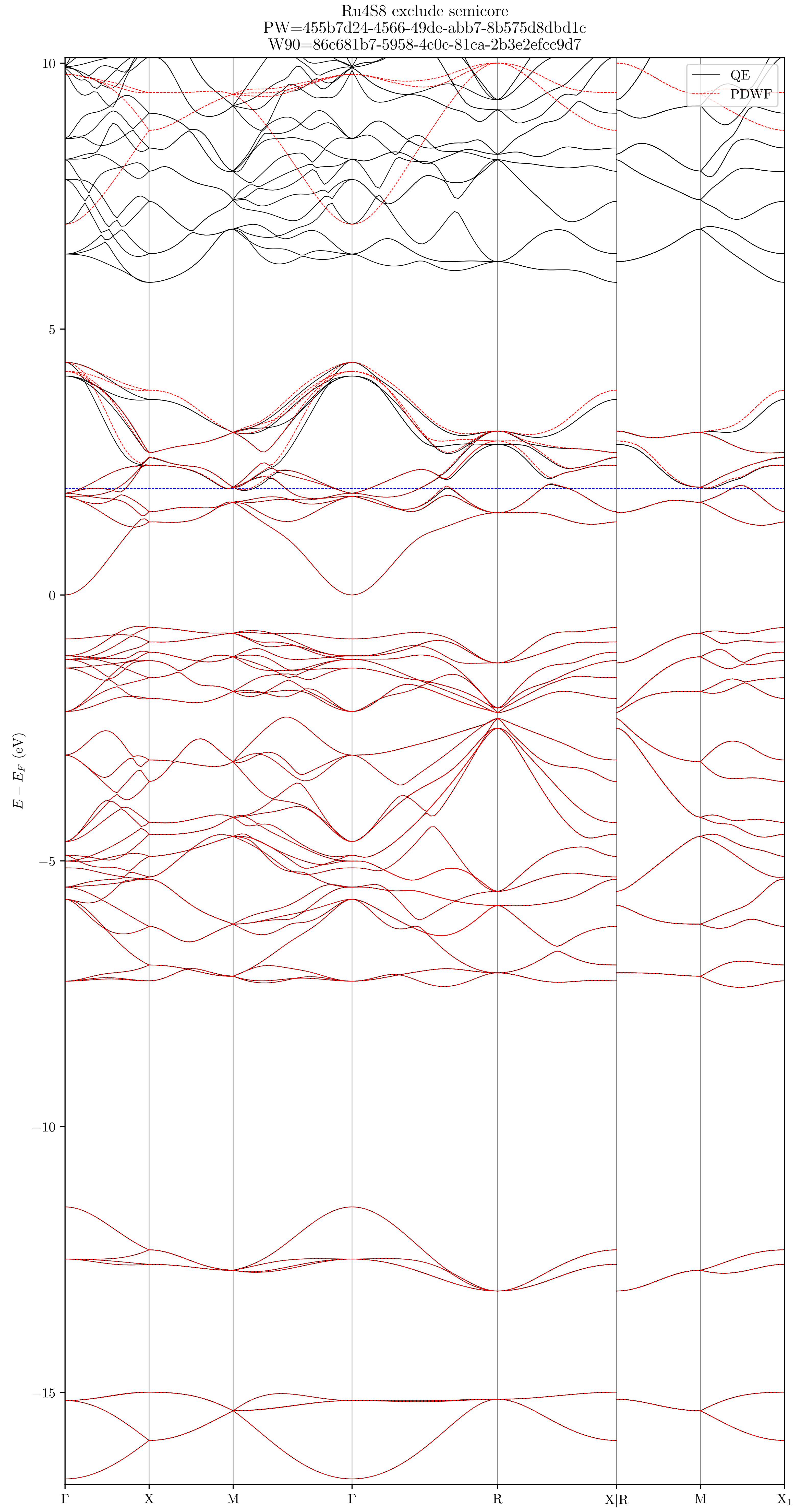

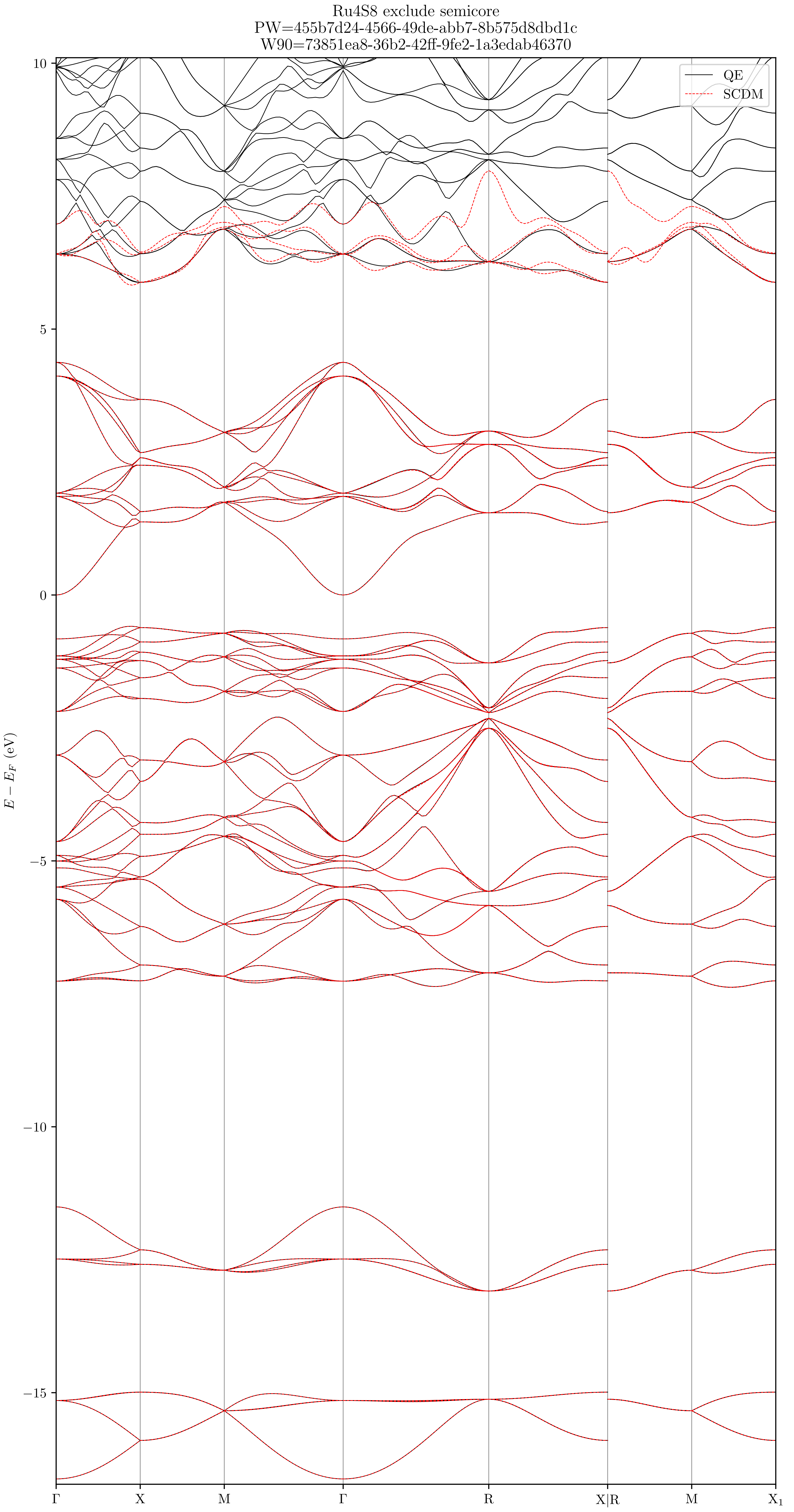

PDWF band distance (meV) and τ ($\AA$)

η_0	η_2	η_4	$\eta_0^{\max}$	$\eta_2^{\max}$	$\eta_4^{\max}$	τ
0.4	1.1	24.1	3.4	12.6	221.3	2.314

SCDM band distance (meV) and τ ($\AA$)

η_0	η_2	η_4	$\eta_0^{\max}$	$\eta_2^{\max}$	$\eta_4^{\max}$	τ
1.6	1.6	1.9	21.2	21.2	21.2	2.750

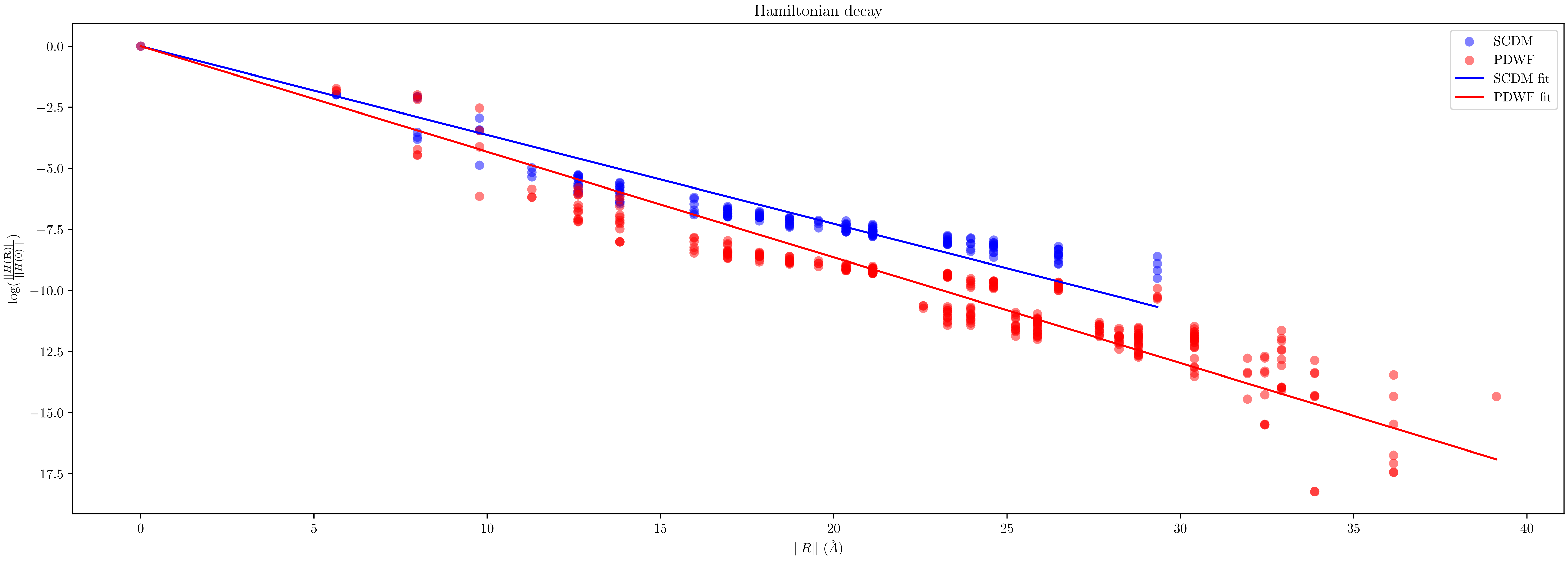

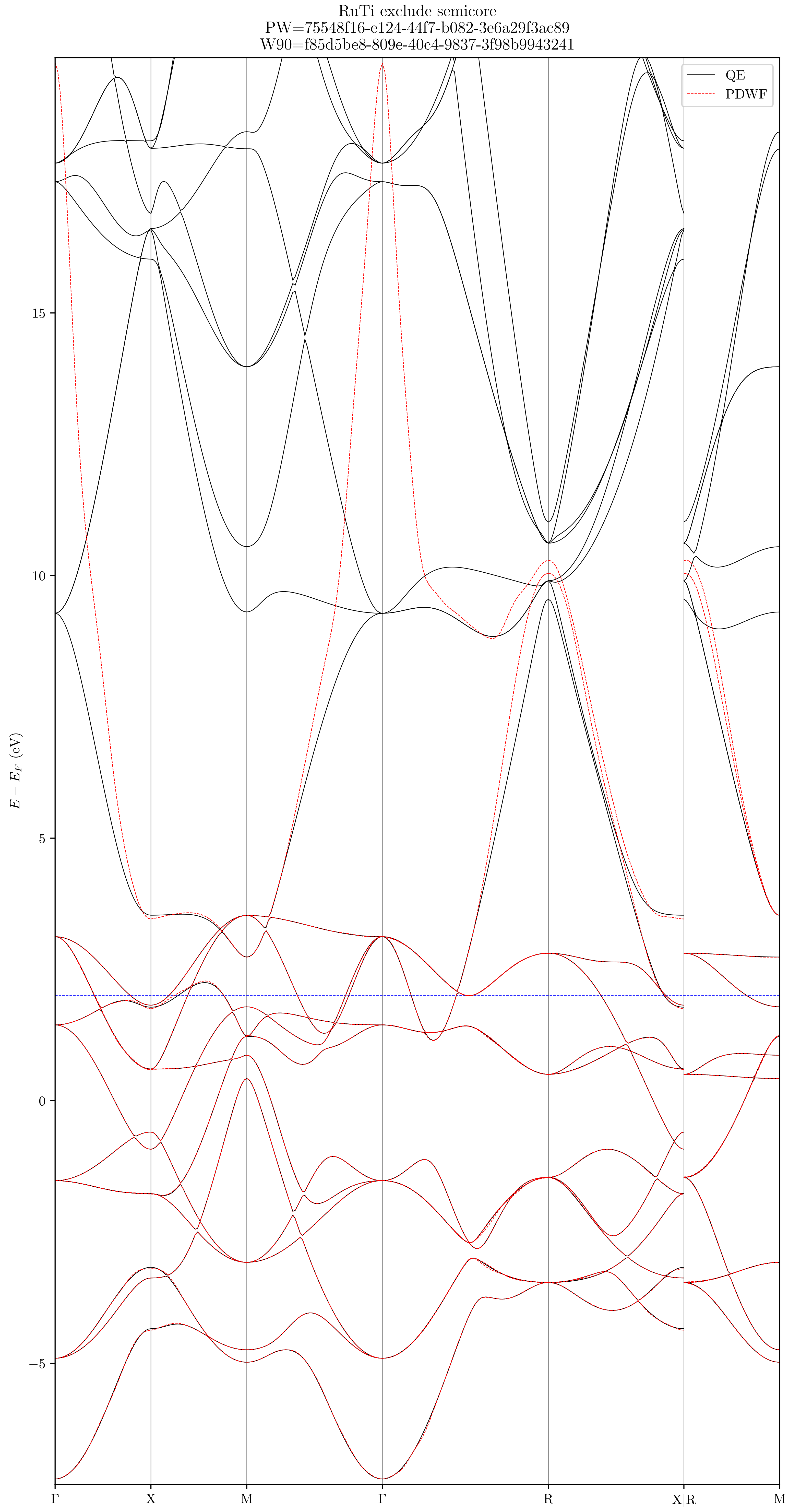

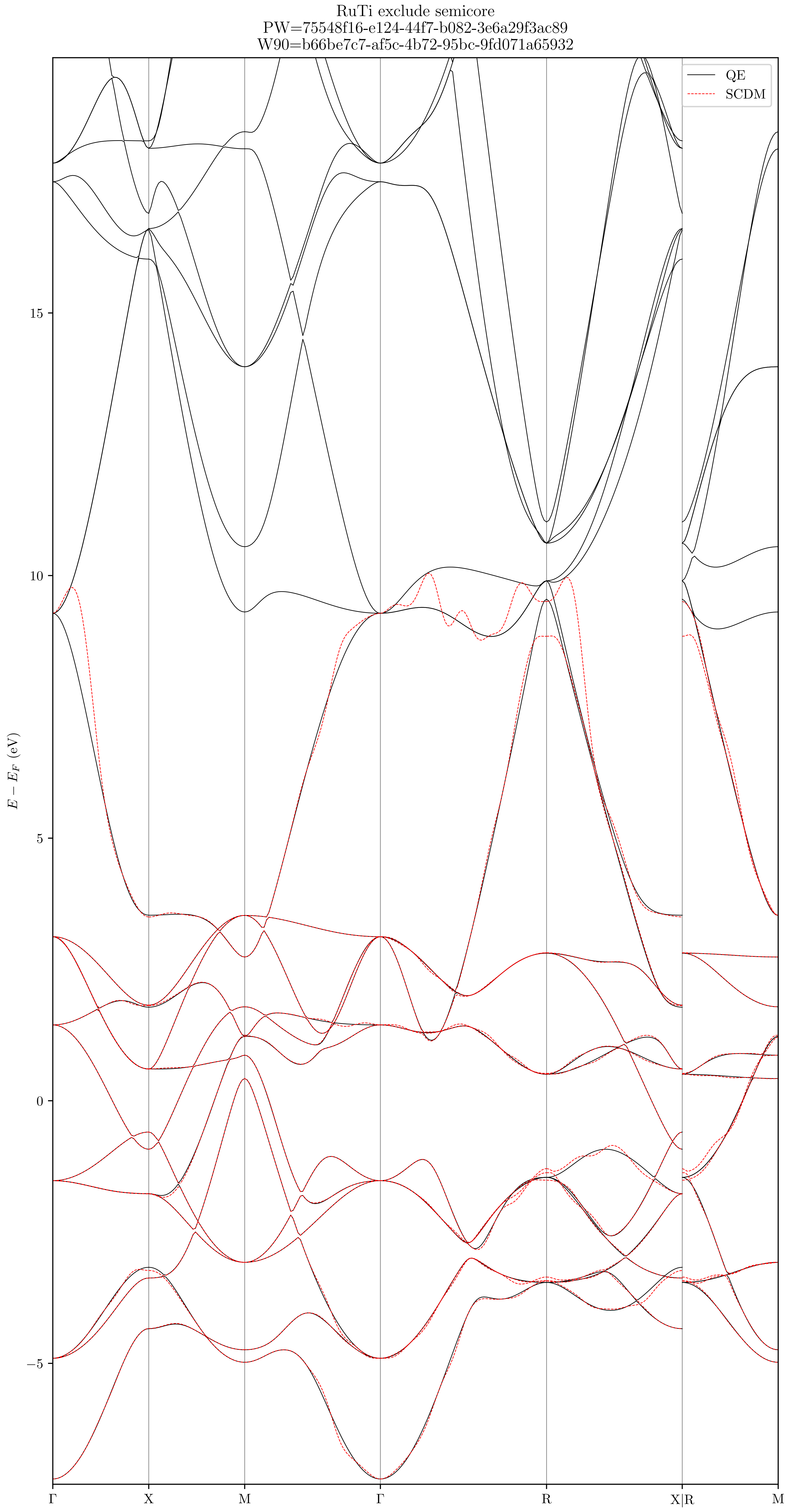

PDWF band distance (meV) and τ ($\AA$)

η_0	η_2	η_4	$\eta_0^{\max}$	$\eta_2^{\max}$	$\eta_4^{\max}$	τ
7.2	6.9	11.0	57.4	57.4	106.5	2.169

SCDM band distance (meV) and τ ($\AA$)

η_0	η_2	η_4	$\eta_0^{\max}$	$\eta_2^{\max}$	$\eta_4^{\max}$	τ
33.4	28.6	26.2	194.5	194.5	194.5	2.449

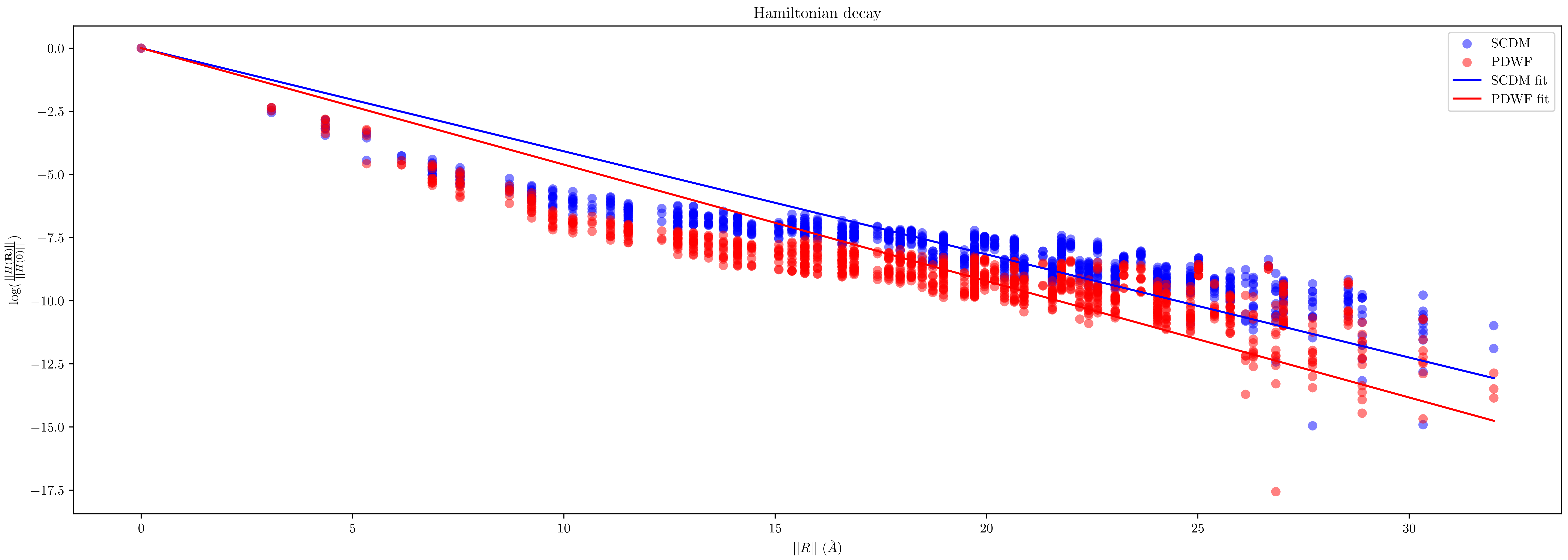

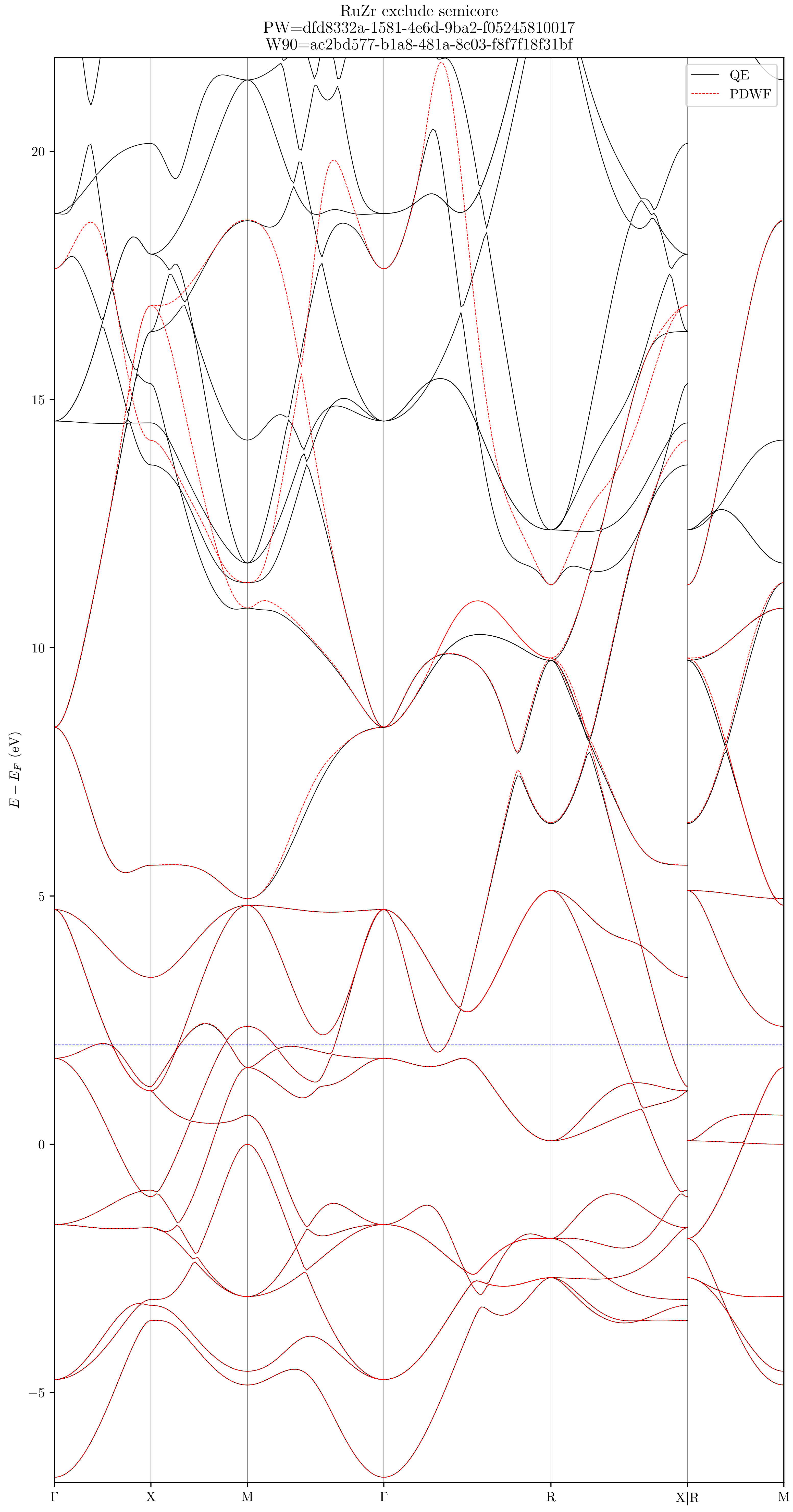

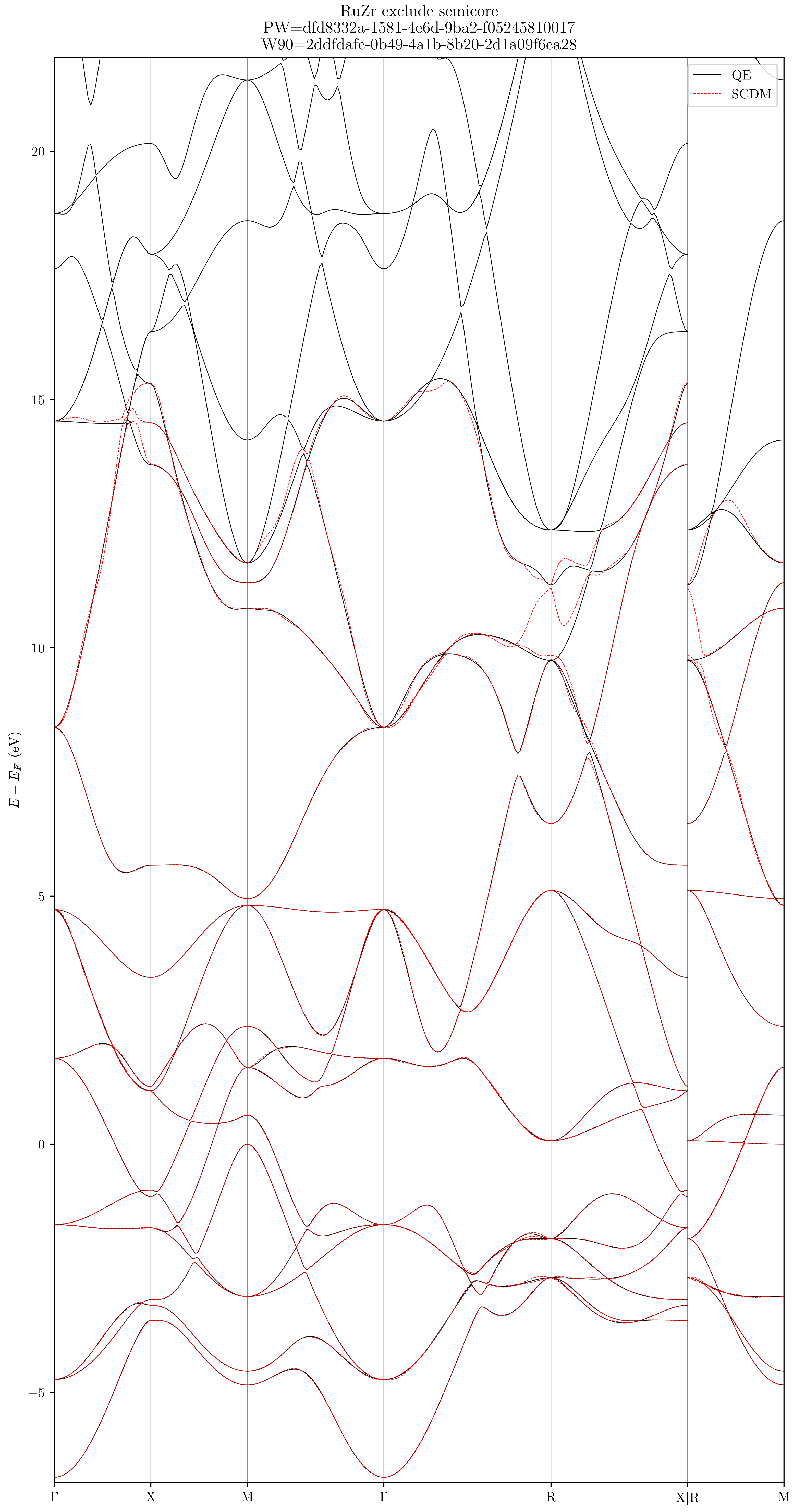

PDWF band distance (meV) and τ ($\AA$)

η_0	η_2	η_4	$\eta_0^{\max}$	$\eta_2^{\max}$	$\eta_4^{\max}$	τ
0.8	0.9	1.4	4.8	5.8	12.8	1.879

SCDM band distance (meV) and τ ($\AA$)

η_0	η_2	η_4	$\eta_0^{\max}$	$\eta_2^{\max}$	$\eta_4^{\max}$	τ
8.9	8.5	8.6	54.8	54.8	54.8	2.265

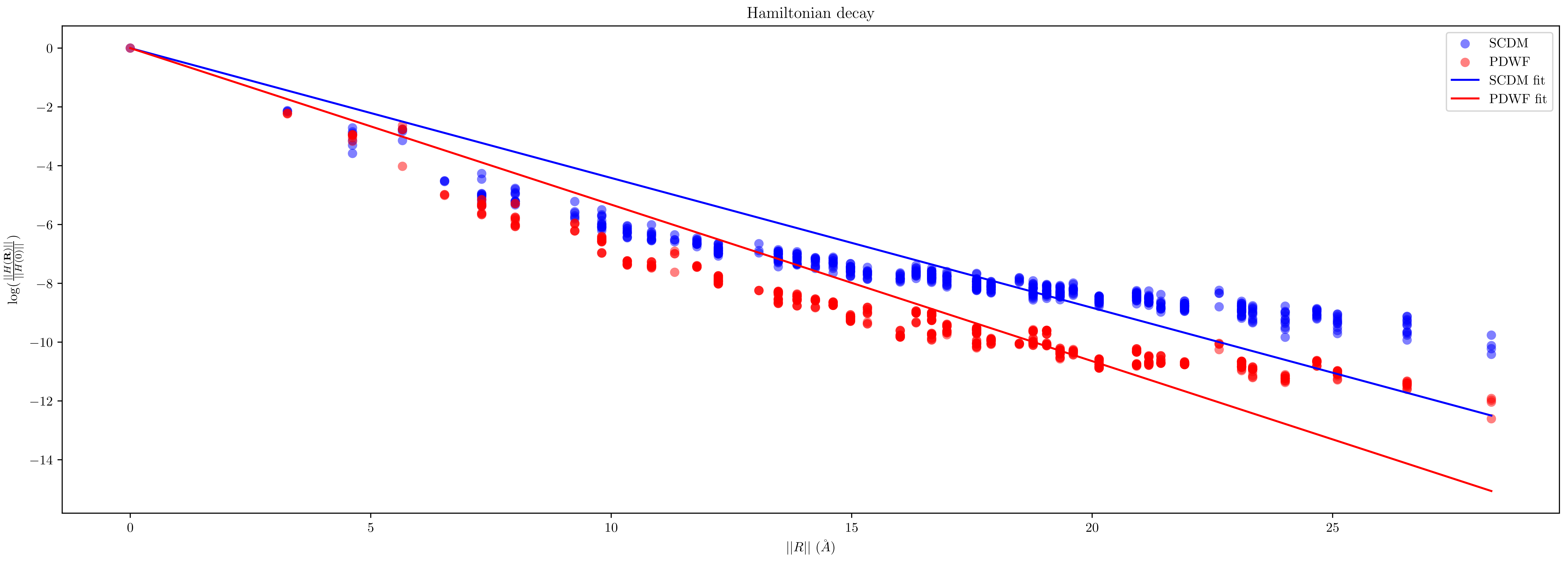

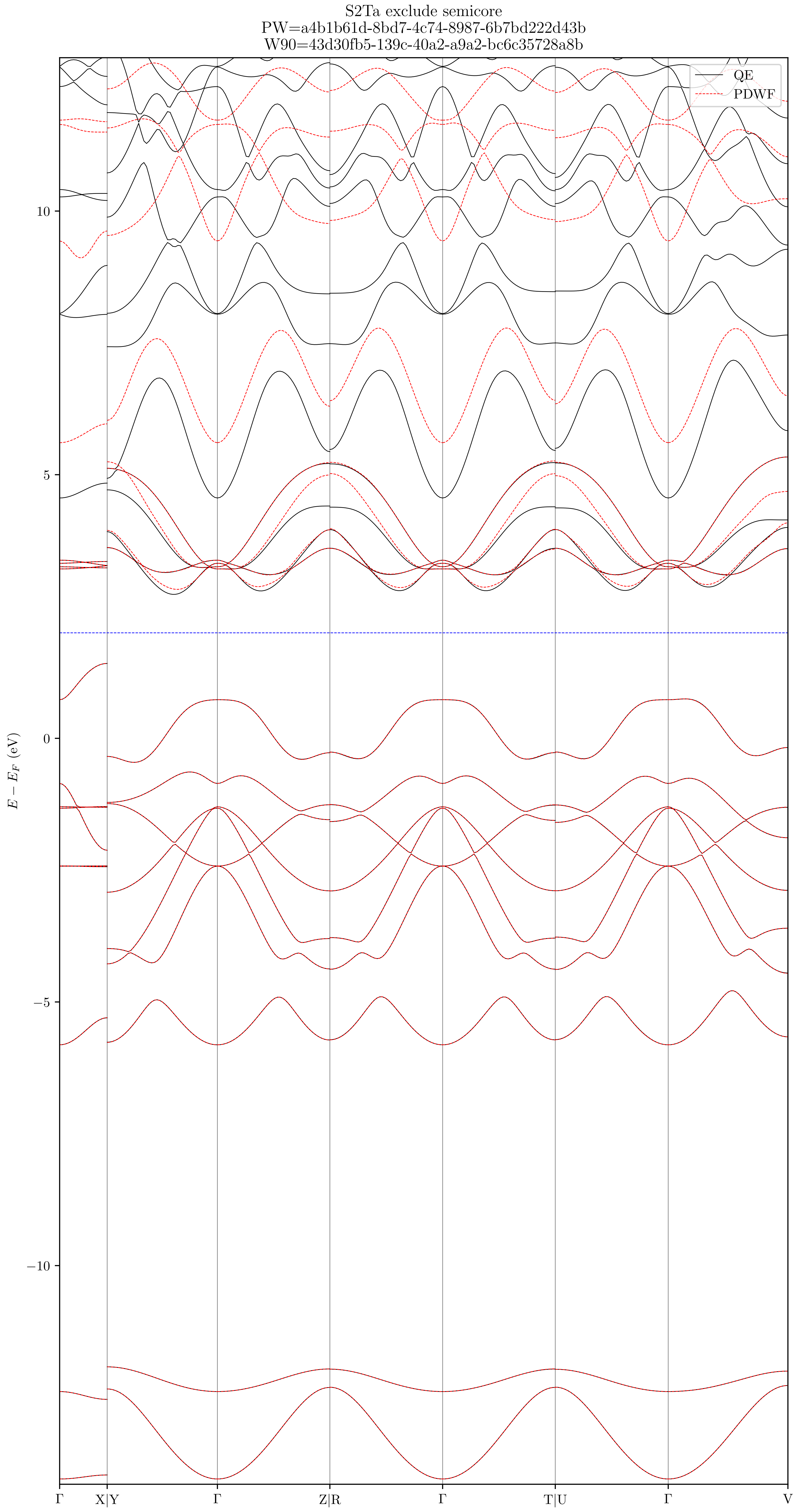

PDWF band distance (meV) and τ ($\AA$)

η_0	η_2	η_4	$\eta_0^{\max}$	$\eta_2^{\max}$	$\eta_4^{\max}$	τ
1.4	1.4	19.1	7.7	8.0	114.2	2.337

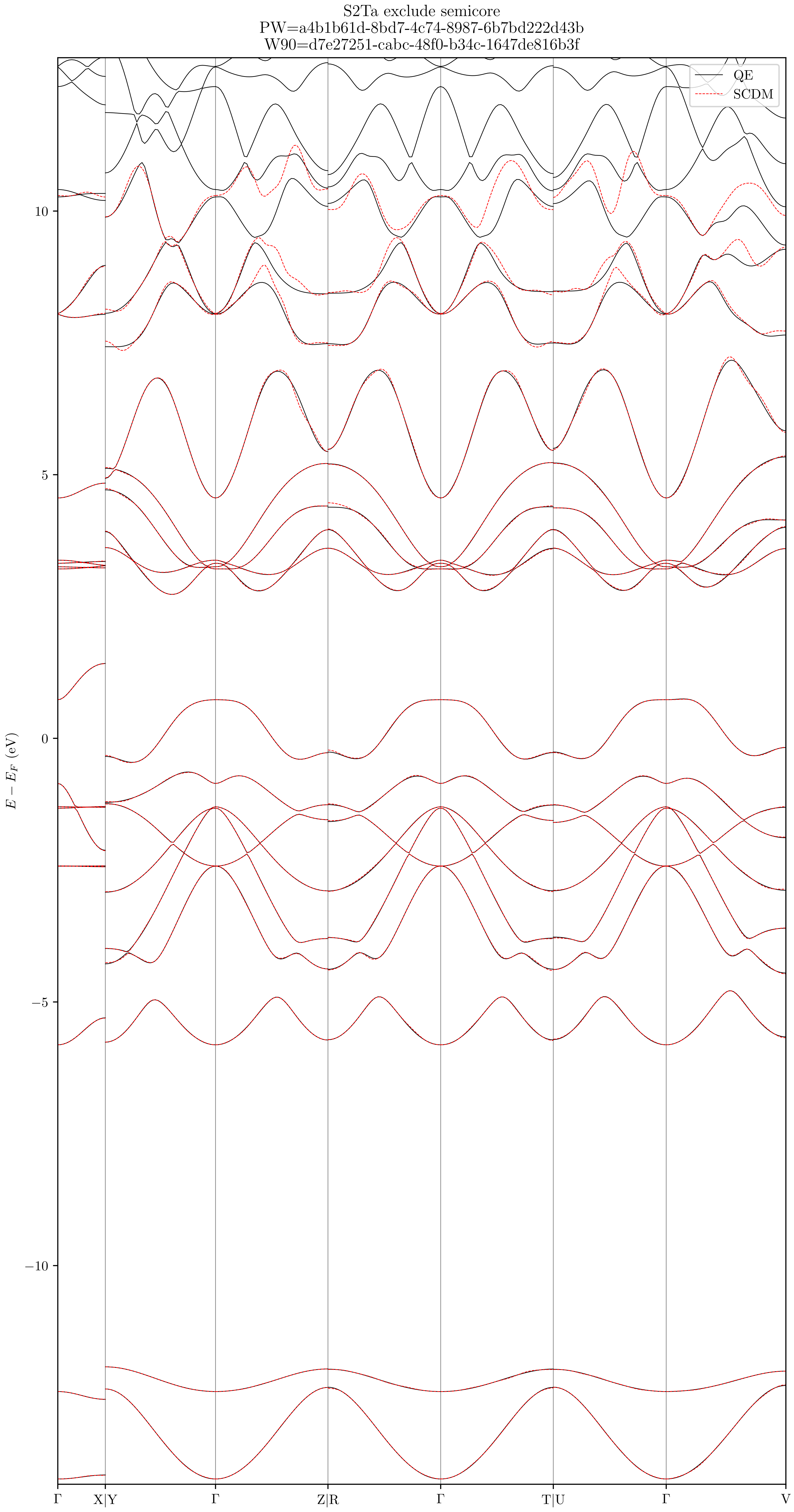

SCDM band distance (meV) and τ ($\AA$)

η_0	η_2	η_4	$\eta_0^{\max}$	$\eta_2^{\max}$	$\eta_4^{\max}$	τ
5.6	5.5	5.6	34.9	38.1	38.1	2.796

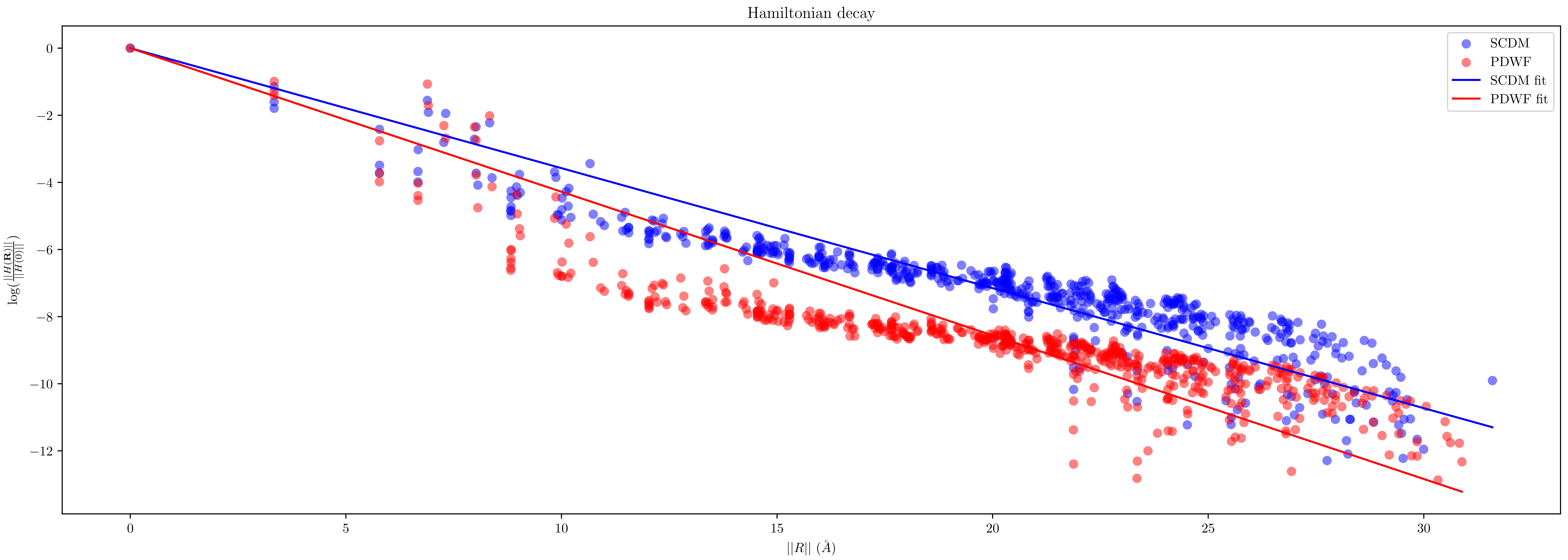

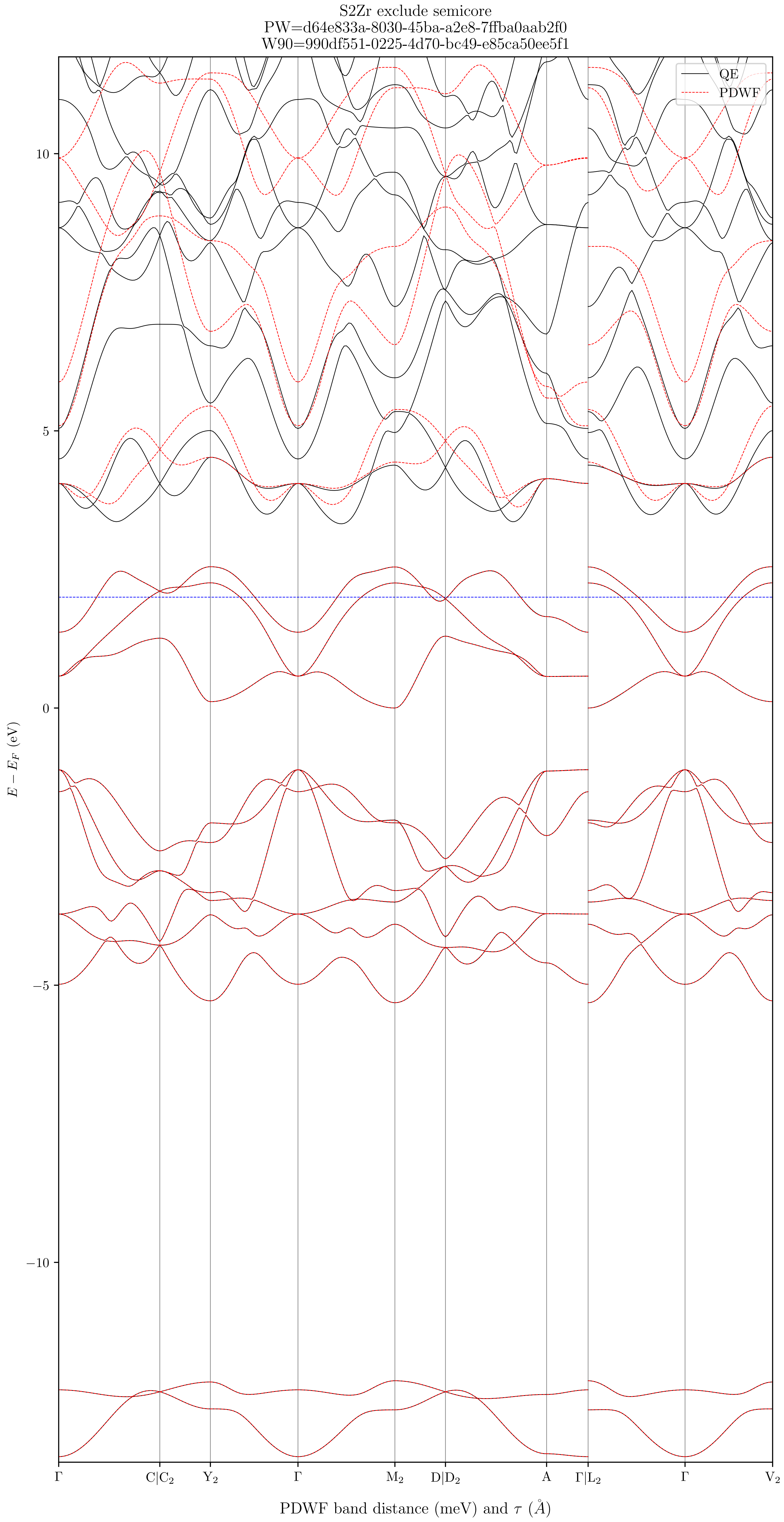

η_0	η_2	η_4	$\eta_0^{\max}$	$\eta_2^{\max}$	$\eta_4^{\max}$	τ
1.2	1.1	20.0	7.9	7.9	107.4	2.361

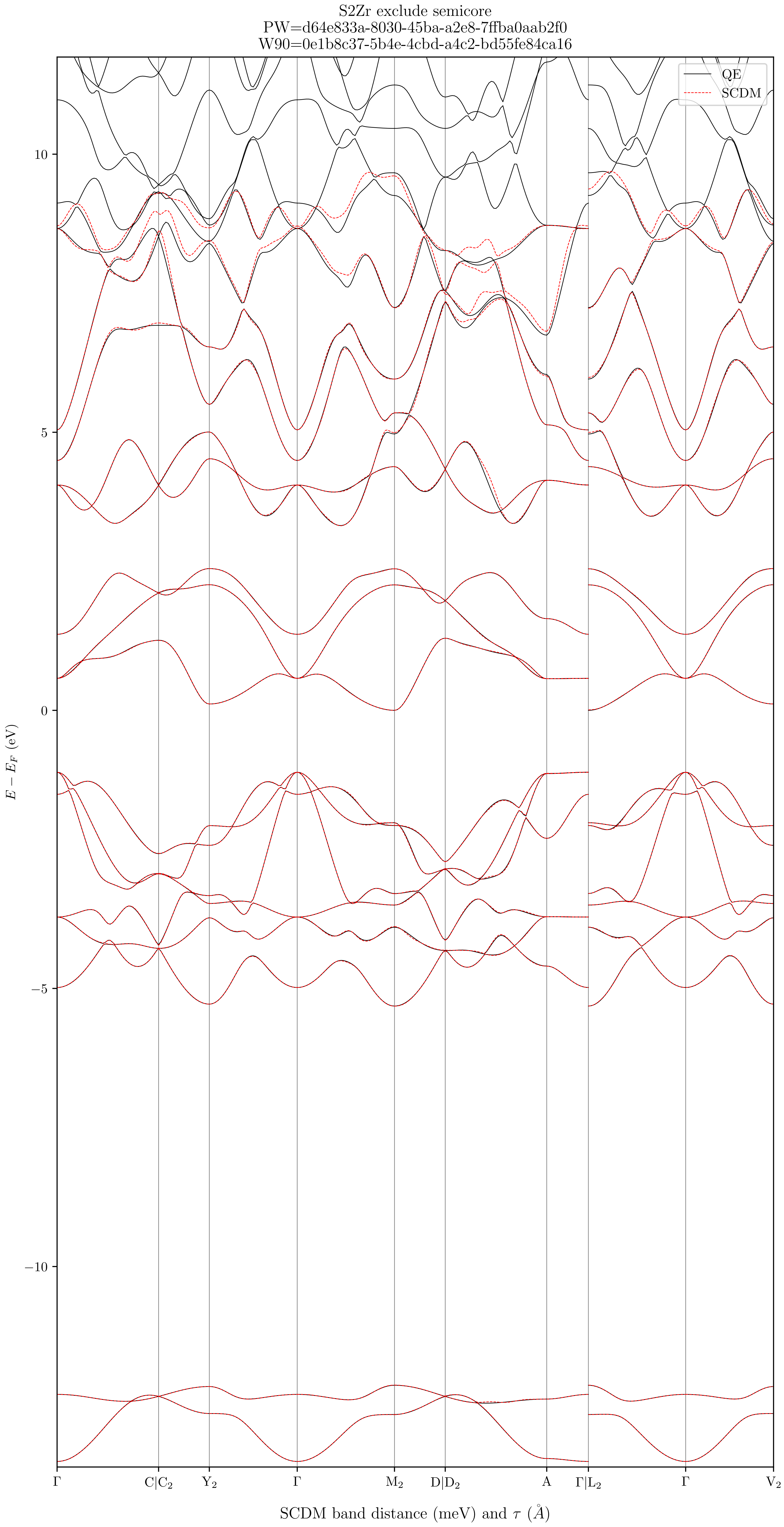

η_0	η_2	η_4	$\eta_0^{\max}$	$\eta_2^{\max}$	$\eta_4^{\max}$	τ
4.3	4.1	4.2	25.3	25.3	25.3	2.853

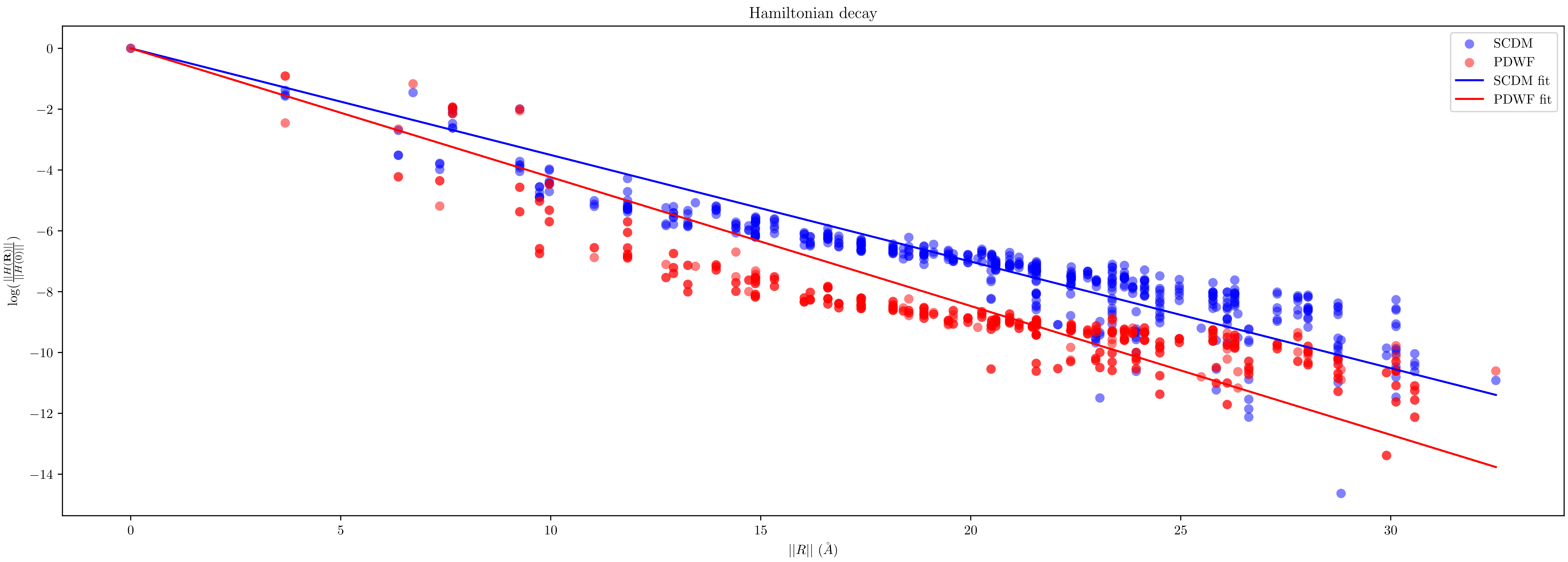

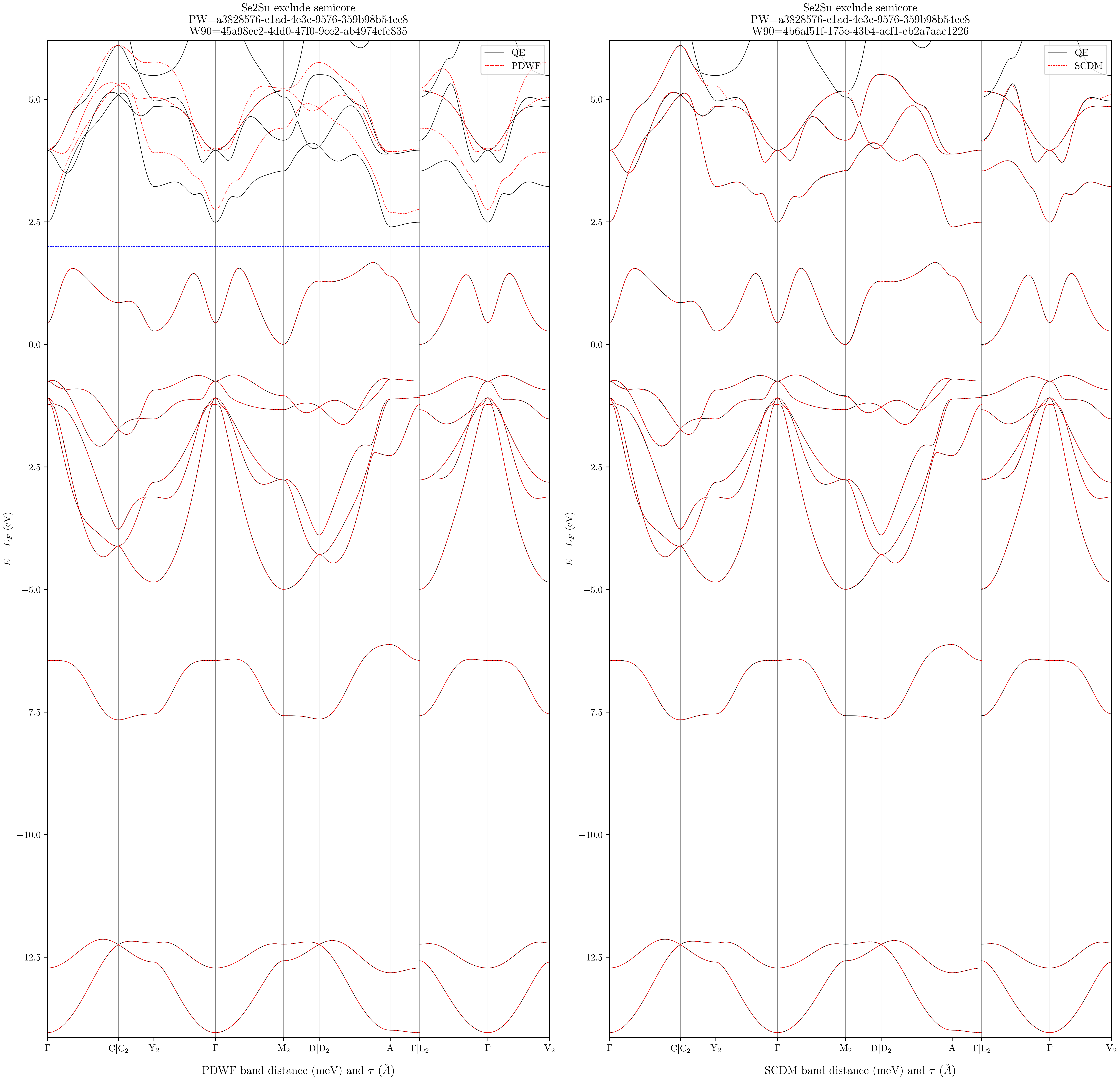

η_0	η_2	η_4	$\eta_0^{\max}$	$\eta_2^{\max}$	$\eta_4^{\max}$	τ
0.6	1.1	88.3	3.3	6.2	425.6	2.232

η_0	η_2	η_4	$\eta_0^{\max}$	$\eta_2^{\max}$	$\eta_4^{\max}$	τ
2.6	2.9	3.0	18.0	18.7	18.7	2.543

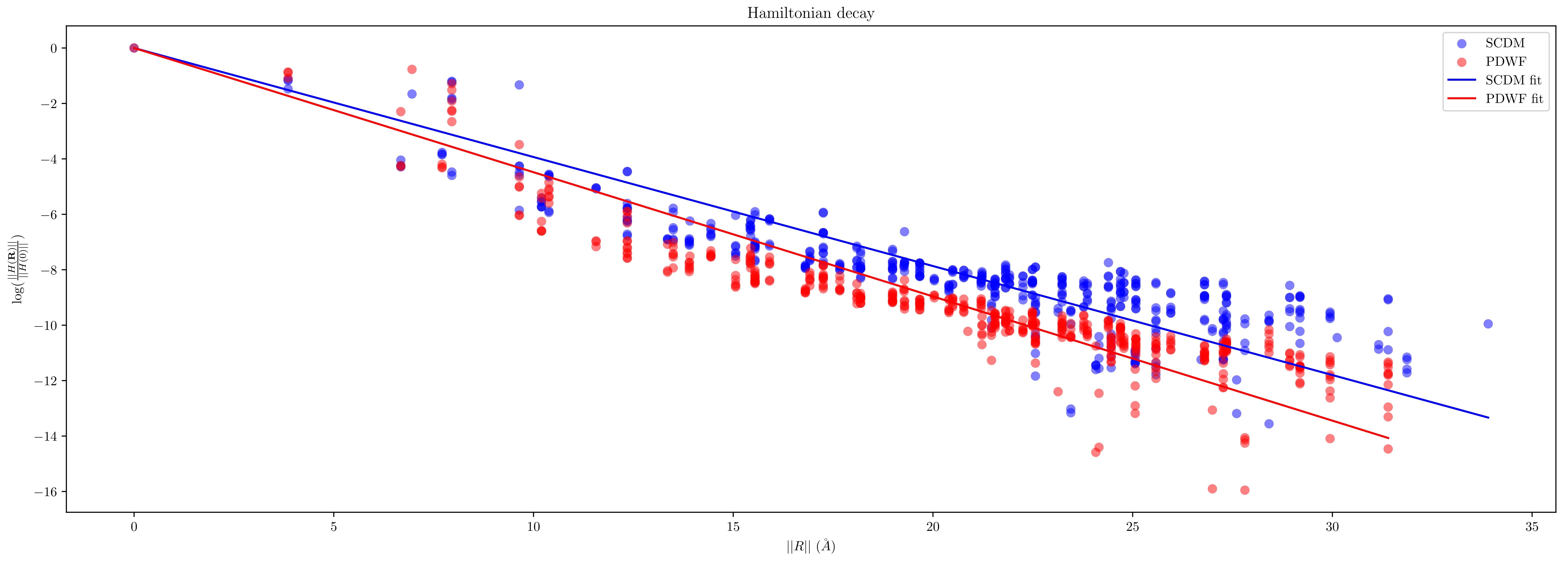

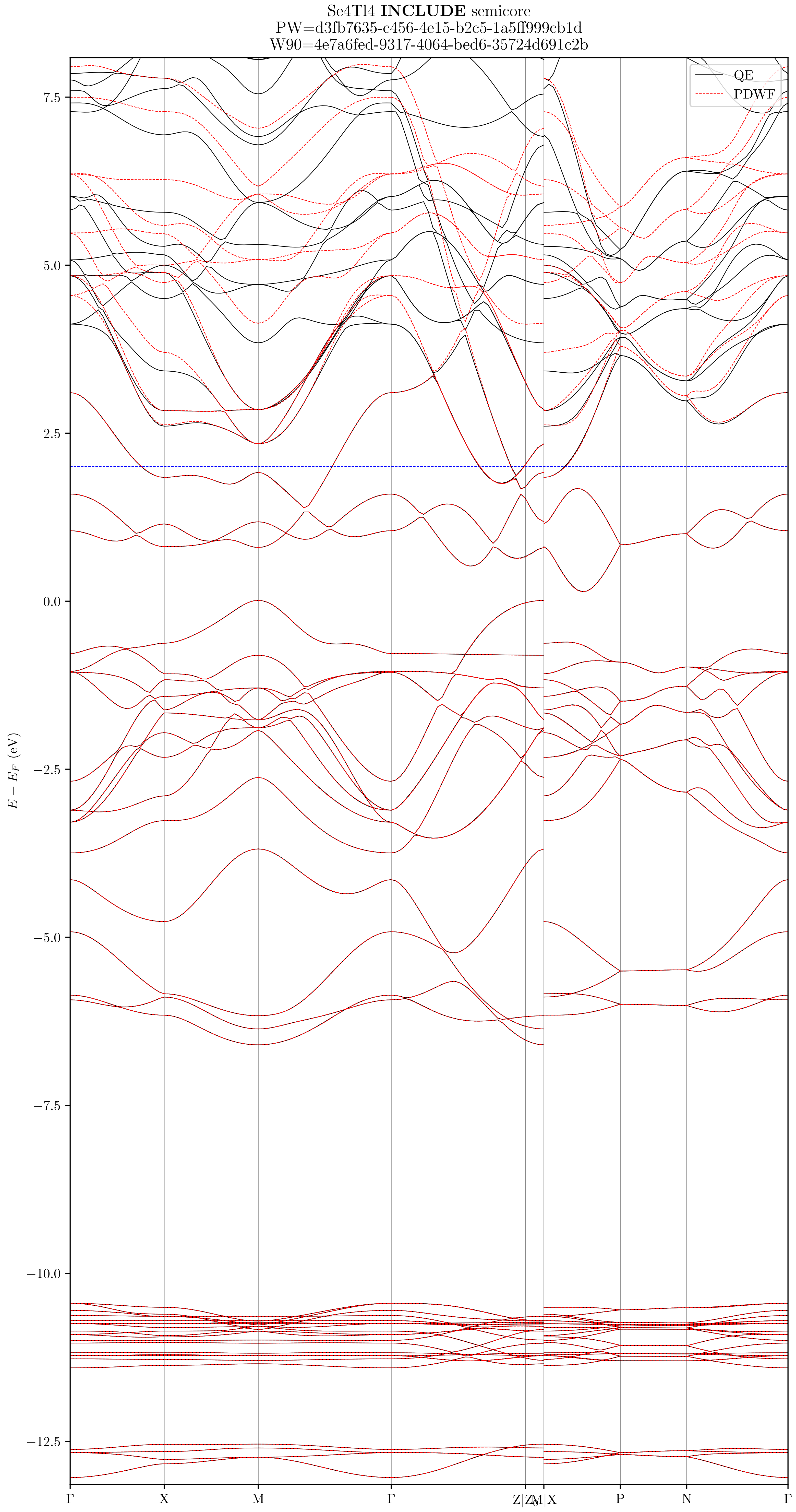

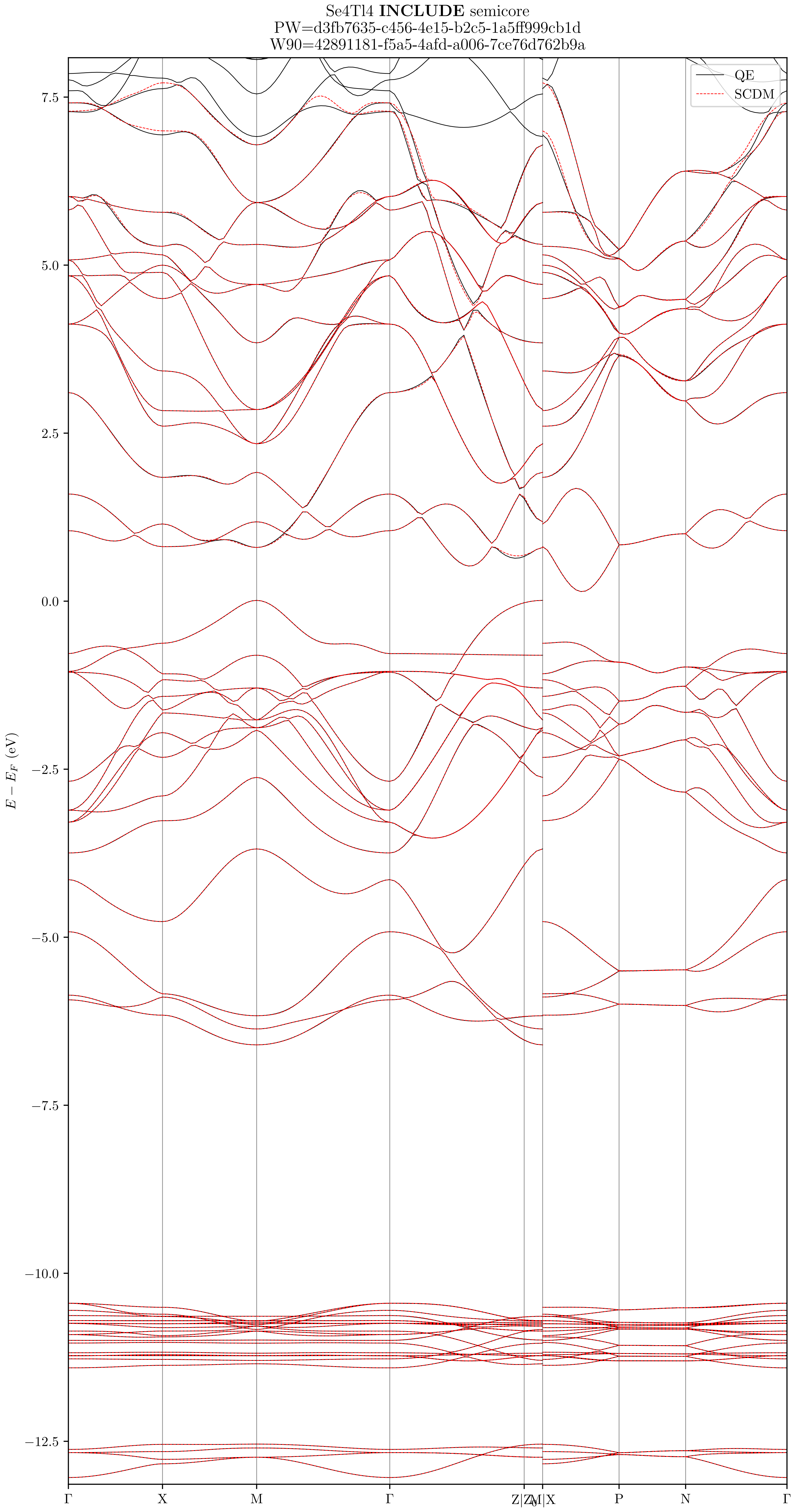

PDWF band distance (meV) and τ ($\AA$)

η_0	η_2	η_4	$\eta_0^{\max}$	$\eta_2^{\max}$	$\eta_4^{\max}$	τ
1.0	1.2	27.4	7.2	9.9	354.8	2.644

SCDM band distance (meV) and τ ($\AA$)

η_0	η_2	η_4	$\eta_0^{\max}$	$\eta_2^{\max}$	$\eta_4^{\max}$	τ
1.2	1.9	2.4	17.7	37.9	41.4	2.851

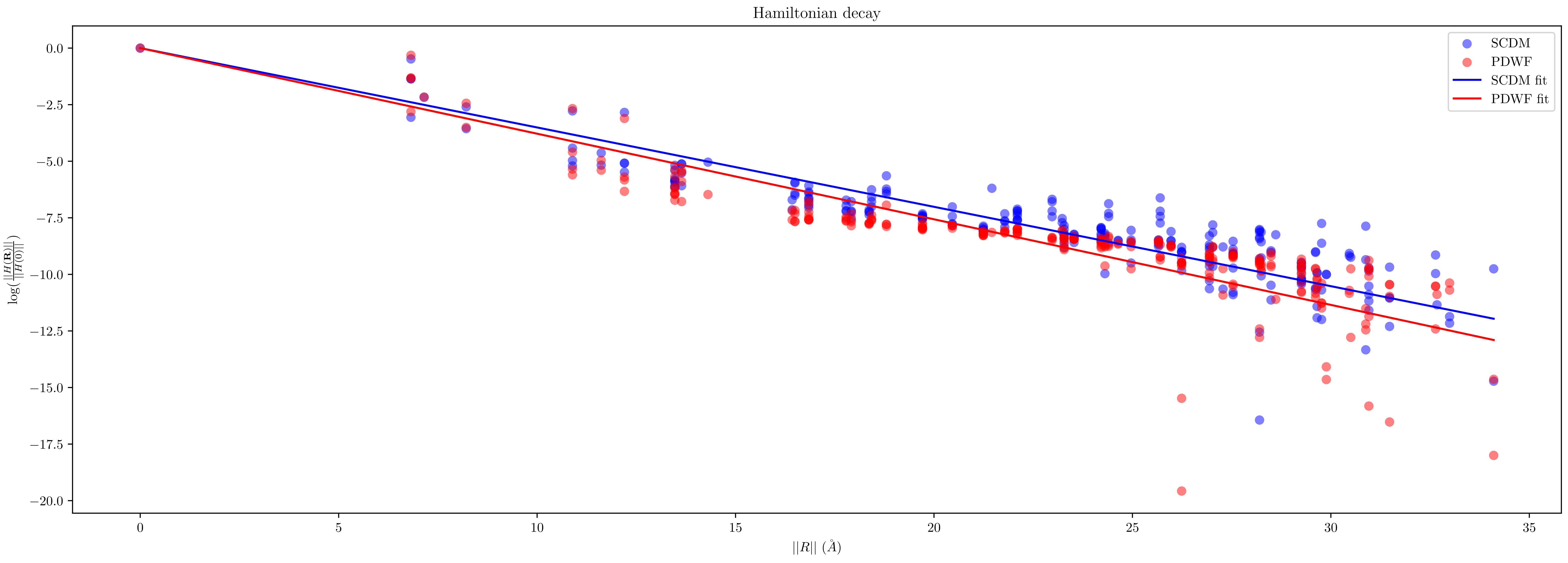

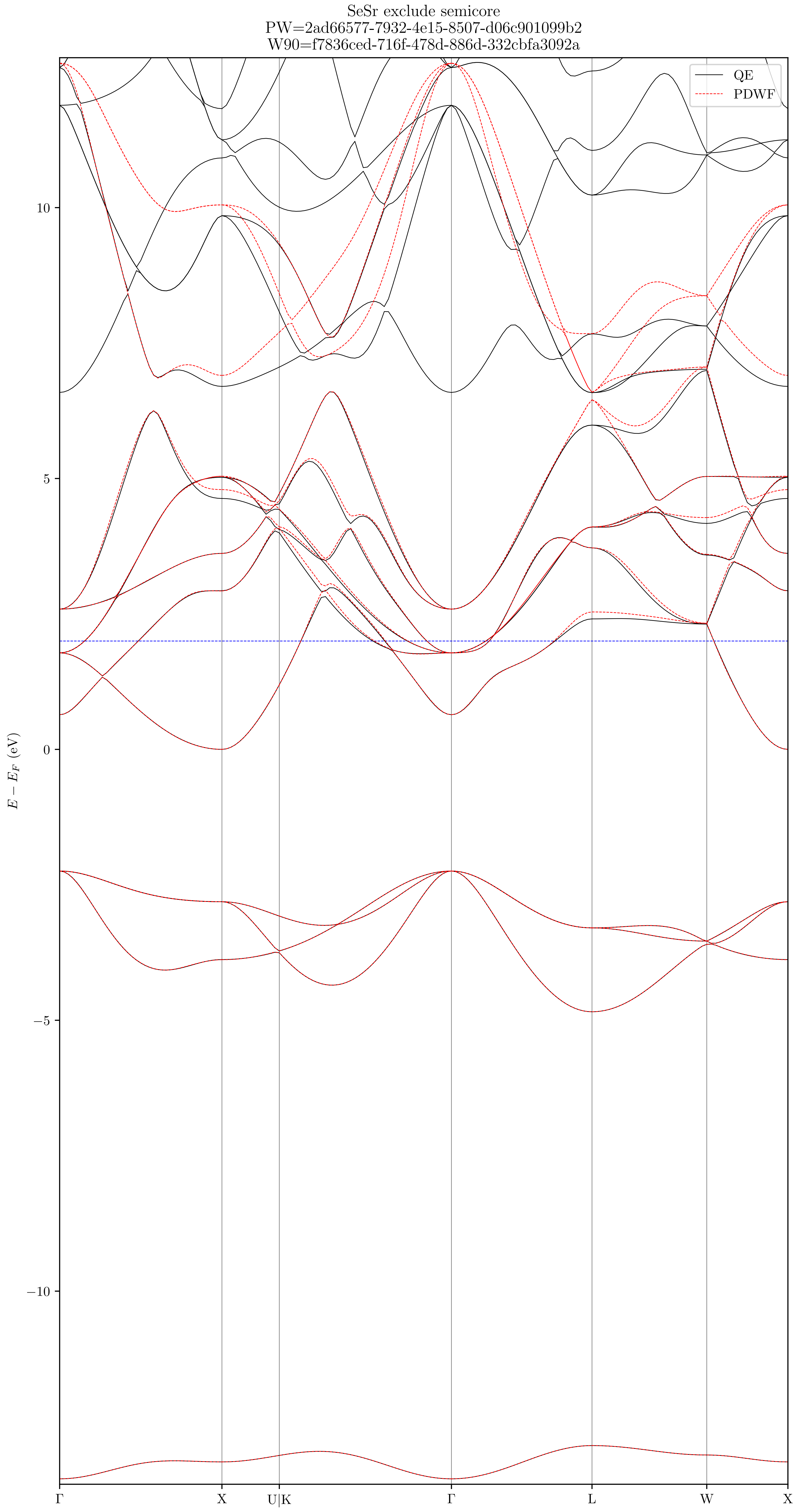

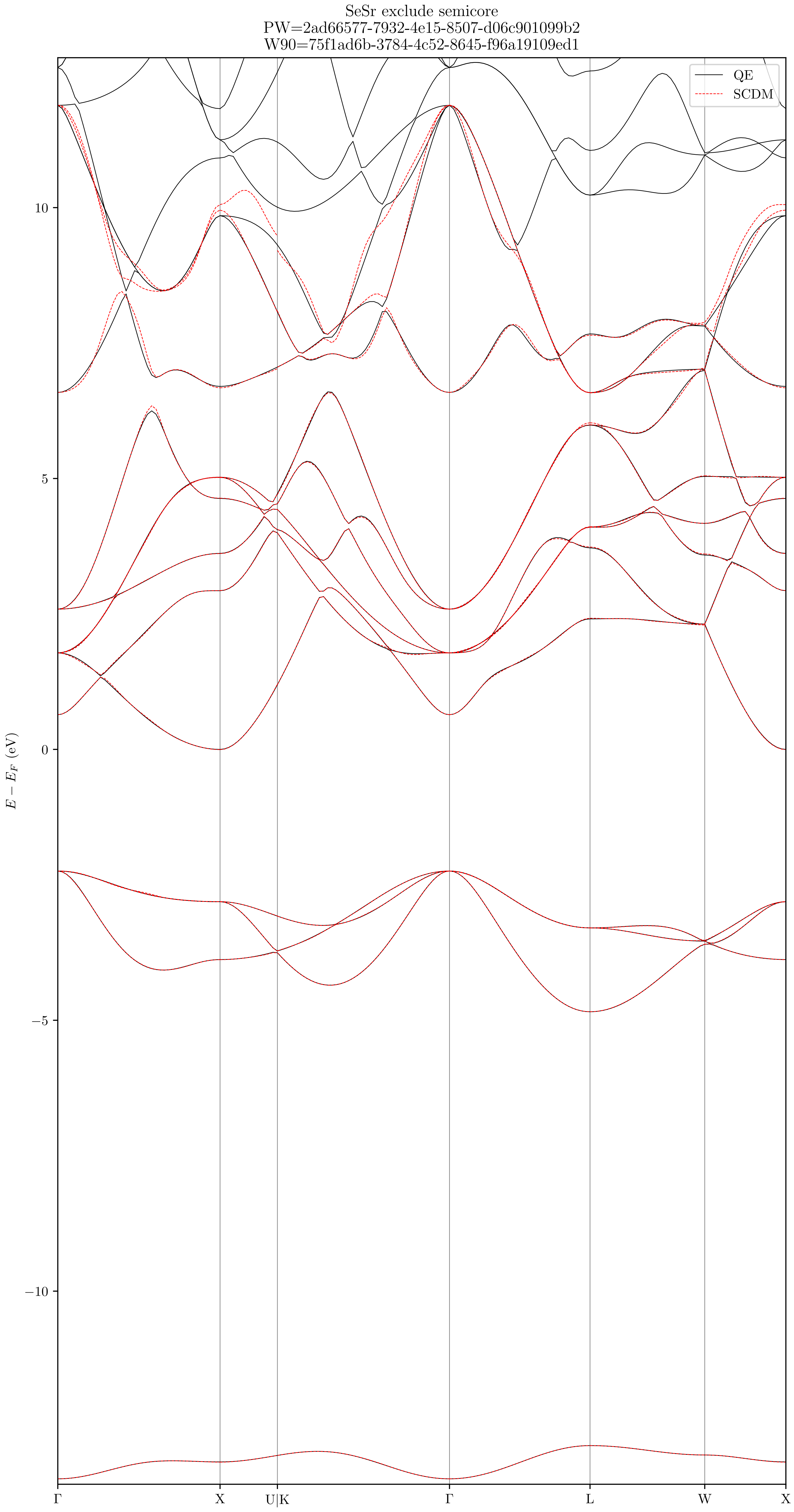

PDWF band distance (meV) and τ ($\AA$)

η_0	η_2	η_4	$\eta_0^{\max}$	$\eta_2^{\max}$	$\eta_4^{\max}$	τ
1.2	1.7	22.9	5.2	11.0	128.4	2.266

SCDM band distance (meV) and τ ($\AA$)

η_0	η_2	η_4	$\eta_0^{\max}$	$\eta_2^{\max}$	$\eta_4^{\max}$	τ
2.7	3.6	5.1	18.4	24.8	27.7	2.671

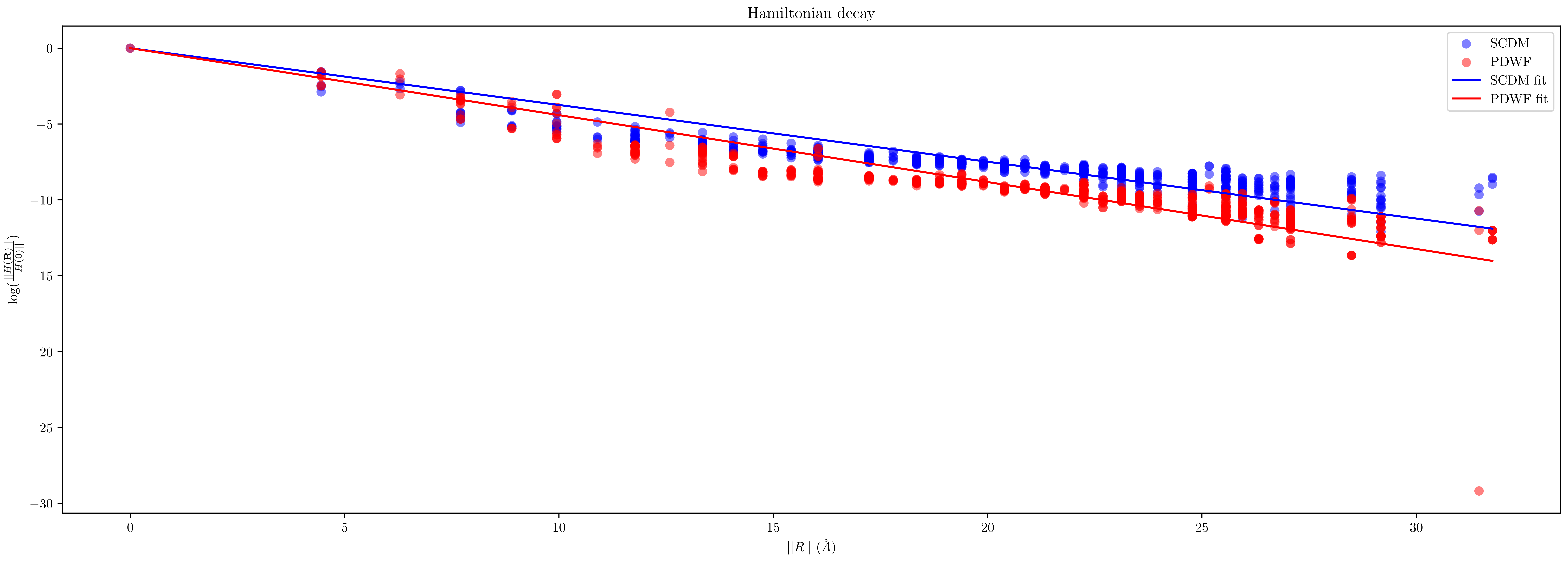

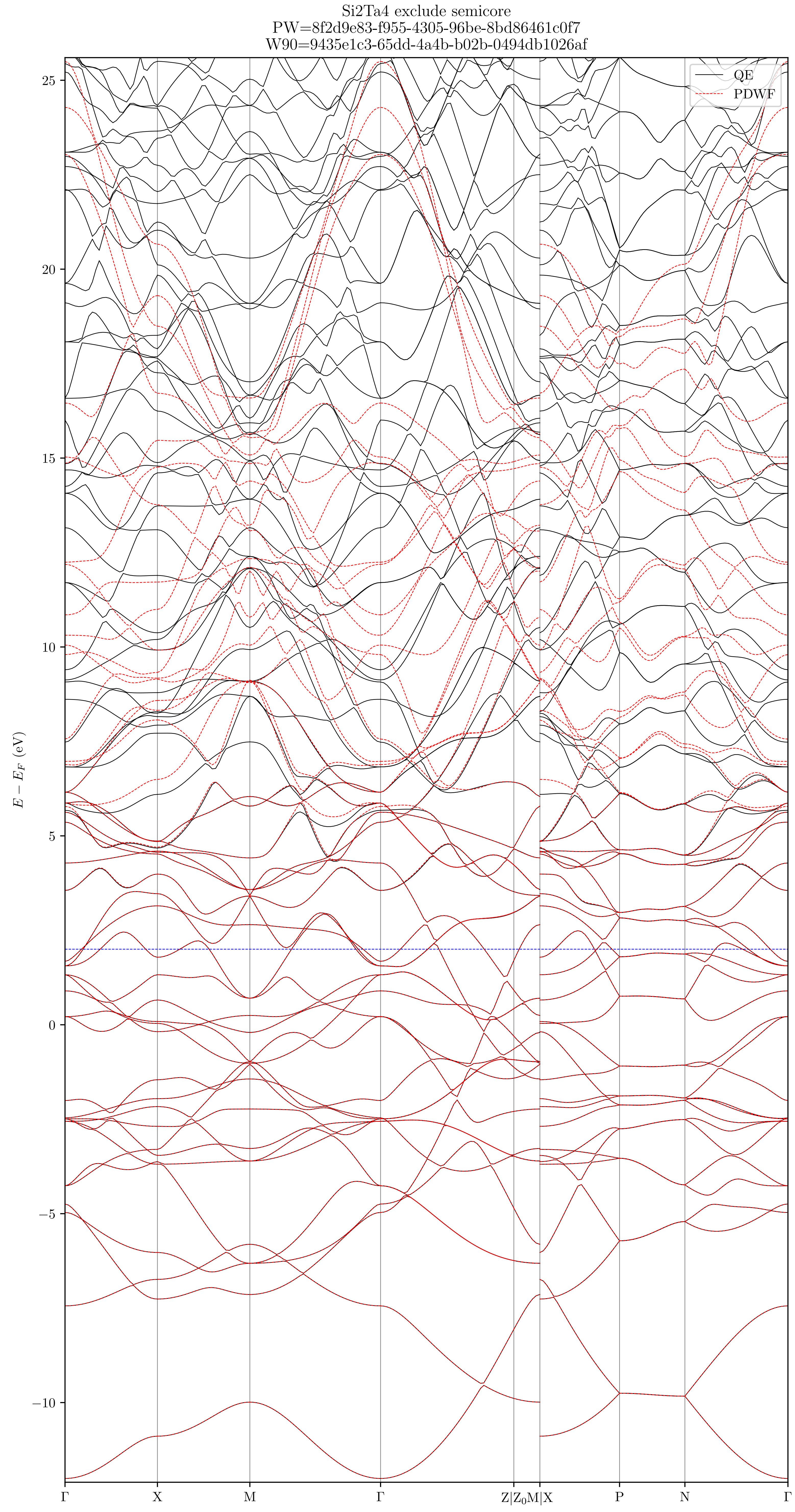

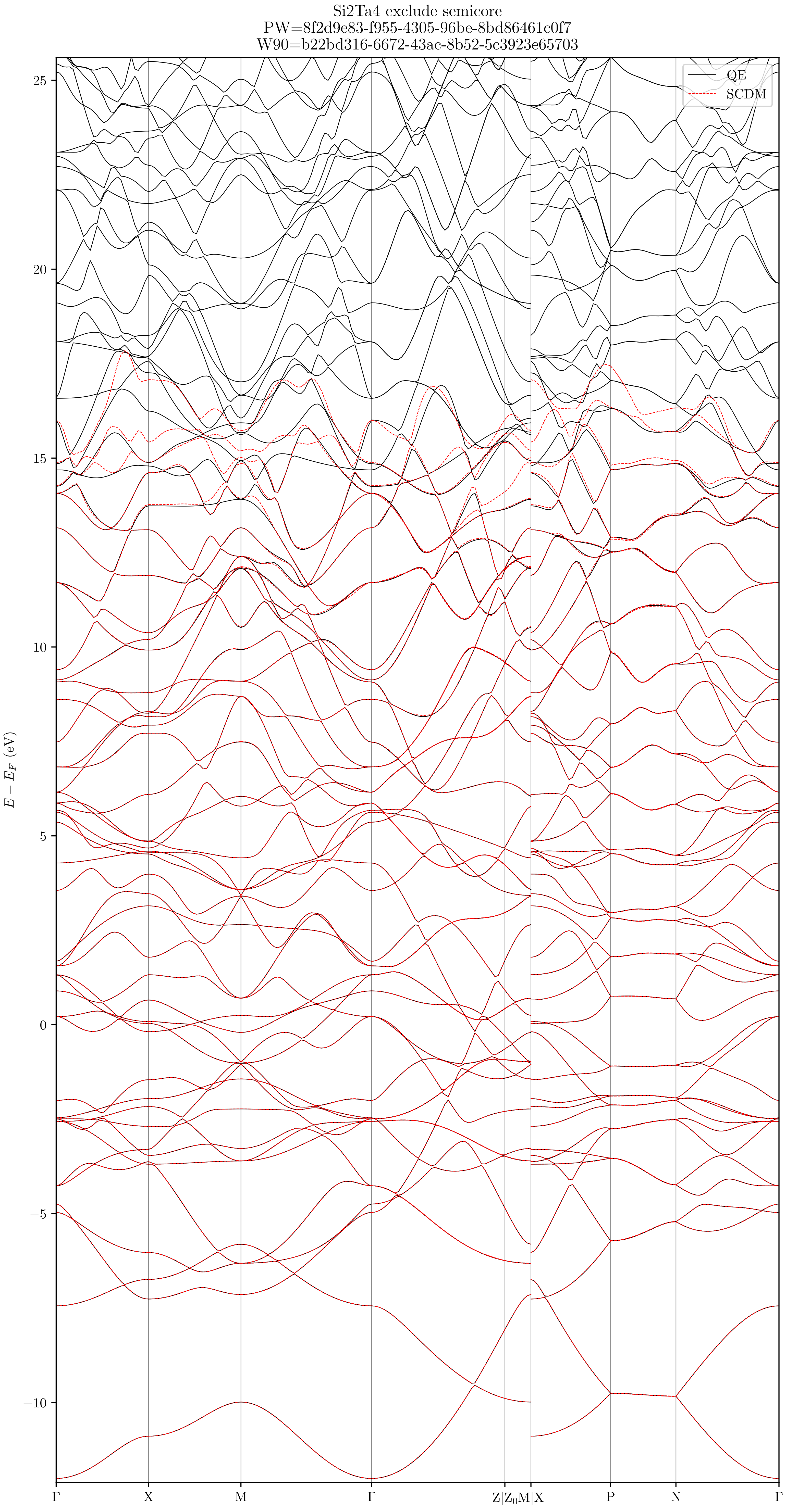

PDWF band distance (meV) and τ ($\AA$)

η_0	η_2	η_4	$\eta_0^{\max}$	$\eta_2^{\max}$	$\eta_4^{\max}$	τ
1.1	1.1	1.9	4.7	5.3	16.4	2.342

SCDM band distance (meV) and τ ($\AA$)

η_0	η_2	η_4	$\eta_0^{\max}$	$\eta_2^{\max}$	$\eta_4^{\max}$	τ
2.7	2.6	2.6	11.3	11.3	11.3	2.729

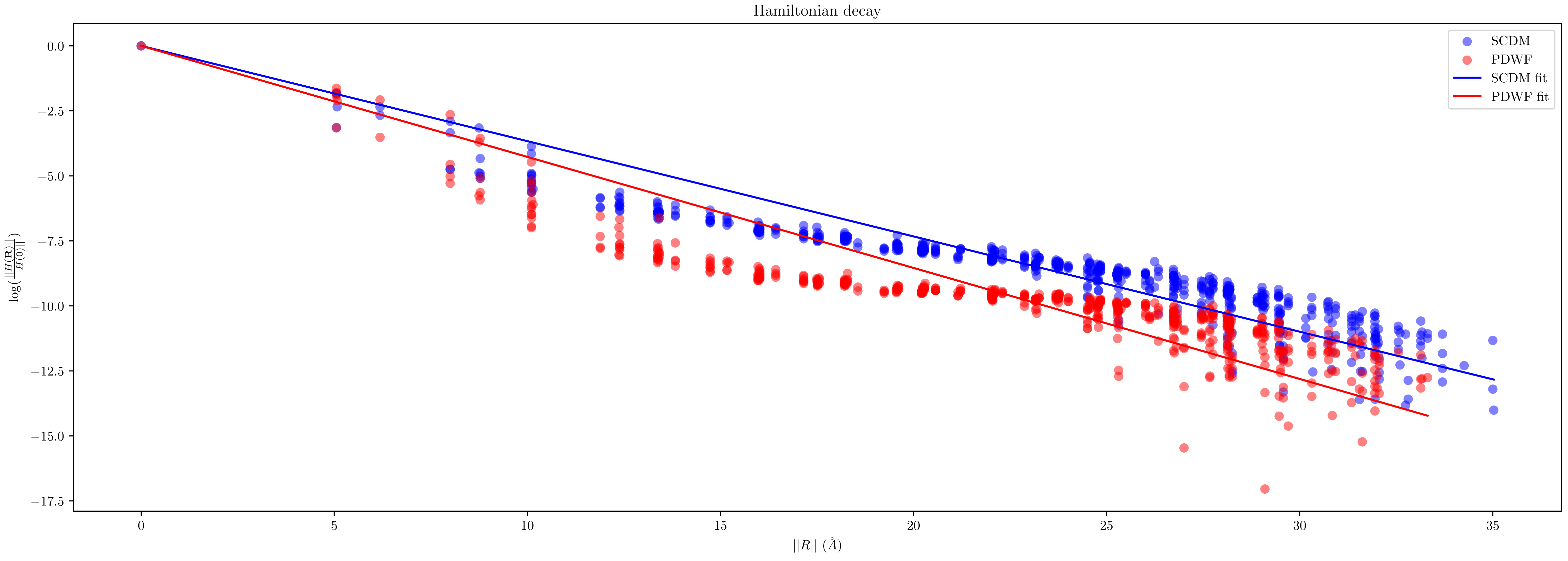

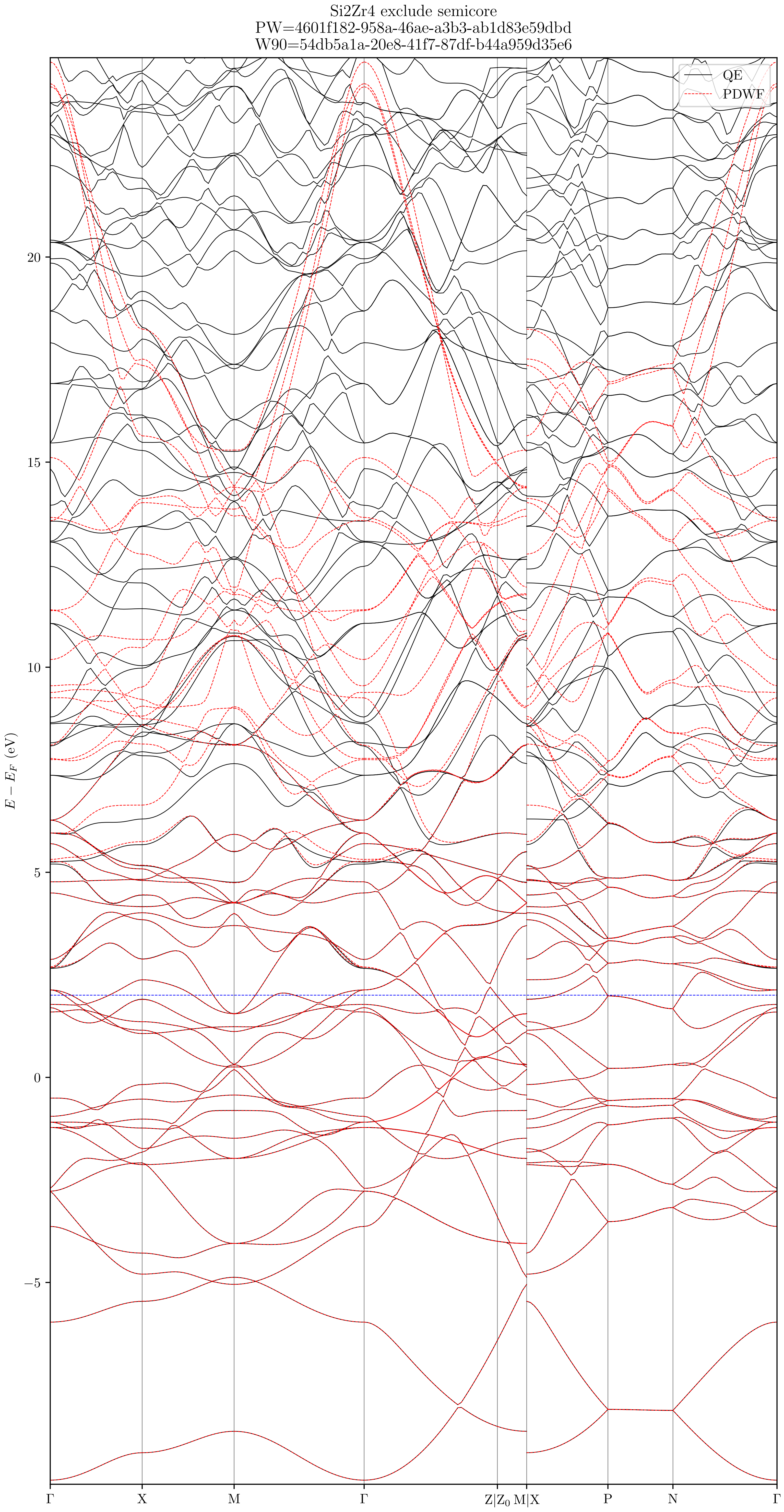

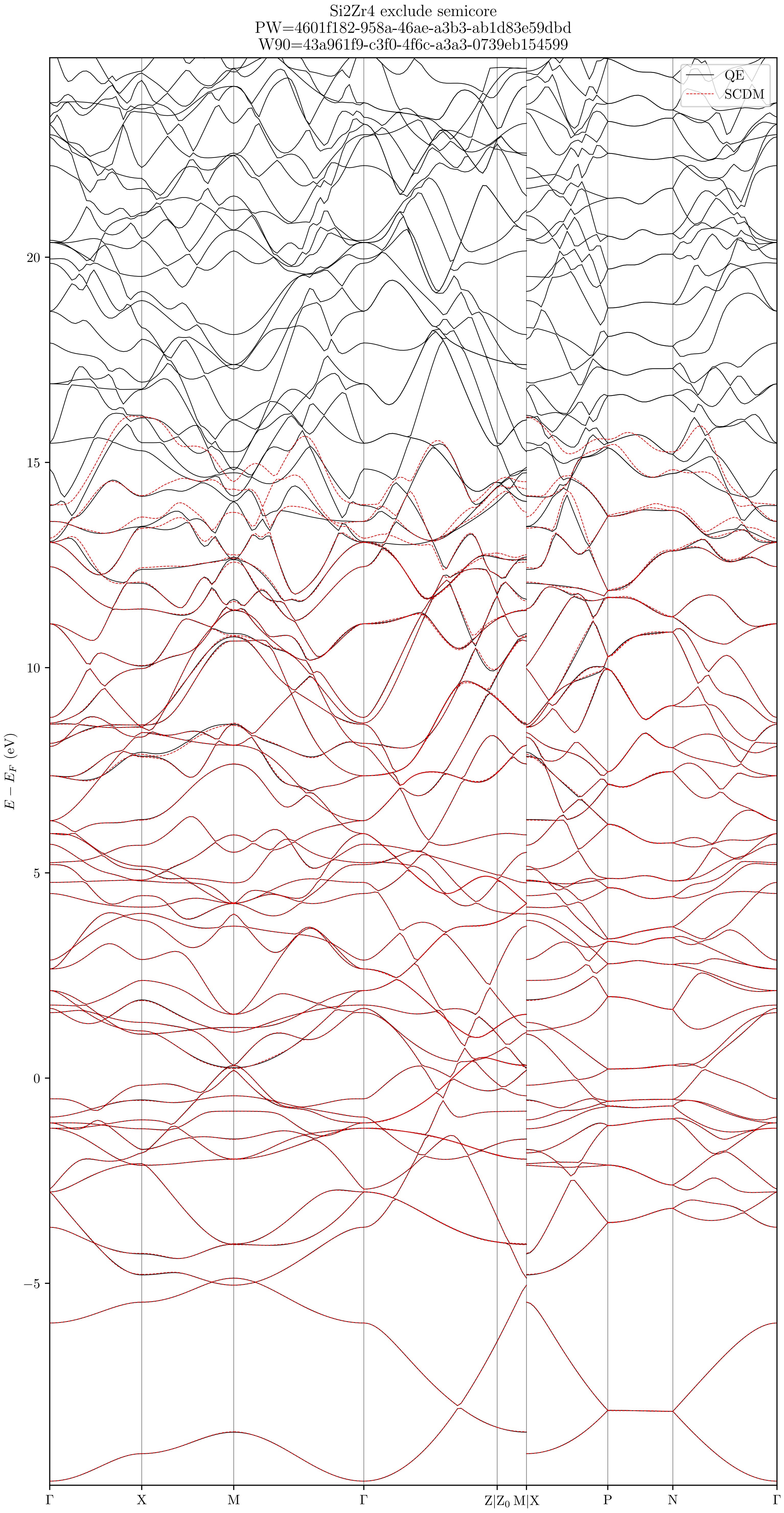

PDWF band distance (meV) and τ ($\AA$)

η_0	η_2	η_4	$\eta_0^{\max}$	$\eta_2^{\max}$	$\eta_4^{\max}$	τ
1.5	1.6	3.9	6.0	7.6	36.7	2.346

SCDM band distance (meV) and τ ($\AA$)

η_0	η_2	η_4	$\eta_0^{\max}$	$\eta_2^{\max}$	$\eta_4^{\max}$	τ
4.2	4.5	4.3	14.7	27.9	27.9	2.757

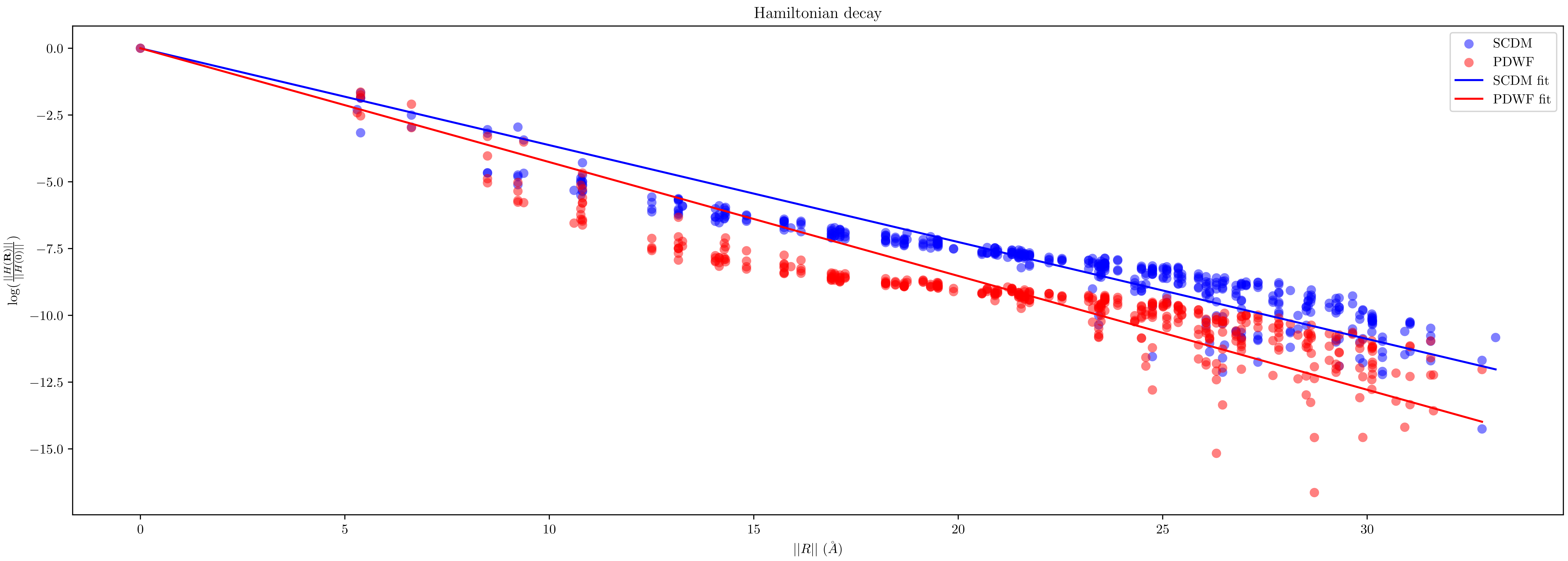

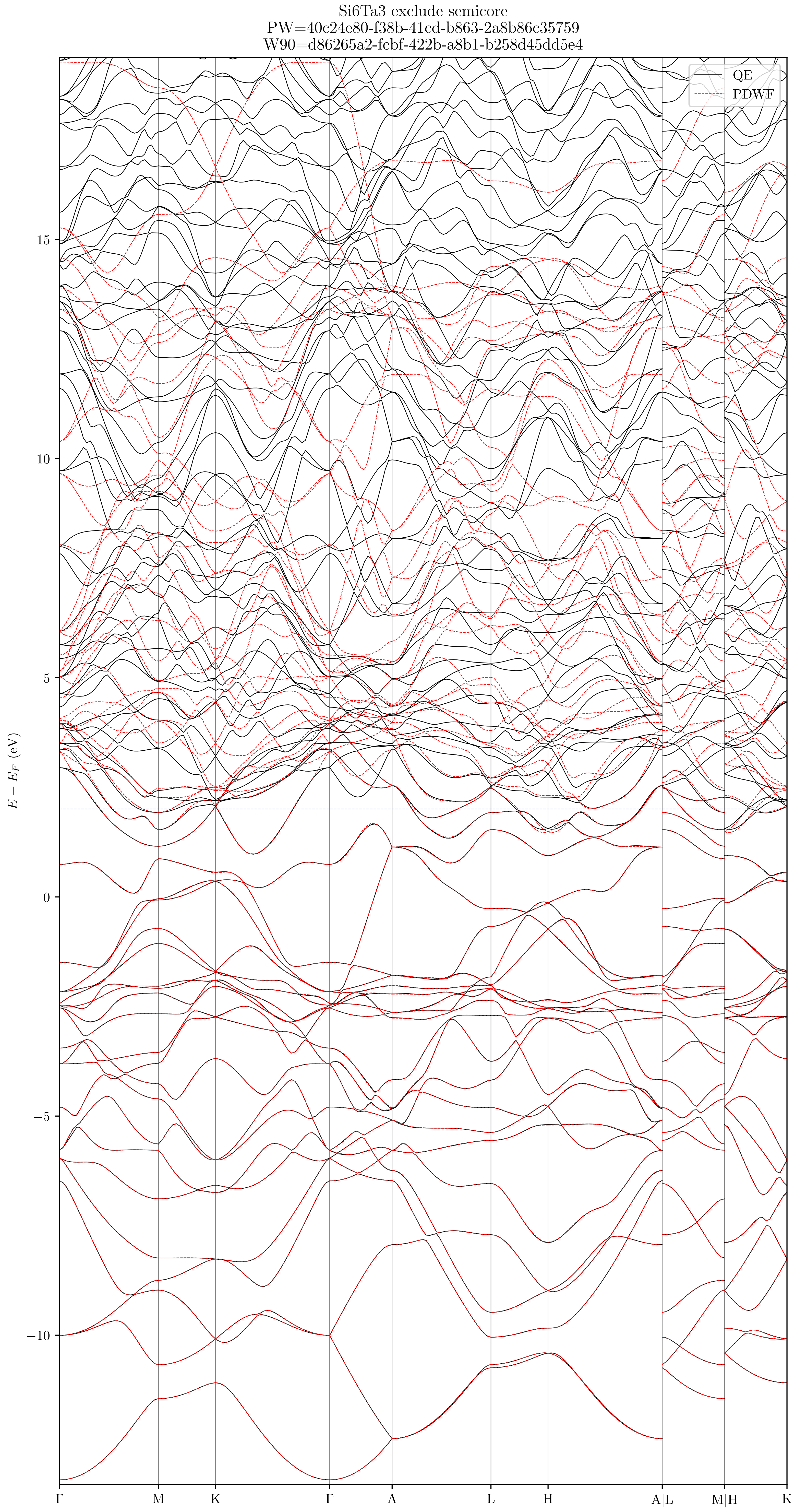

PDWF band distance (meV) and τ ($\AA$)

η_0	η_2	η_4	$\eta_0^{\max}$	$\eta_2^{\max}$	$\eta_4^{\max}$	τ
4.2	8.9	98.3	24.2	73.7	584.8	2.488

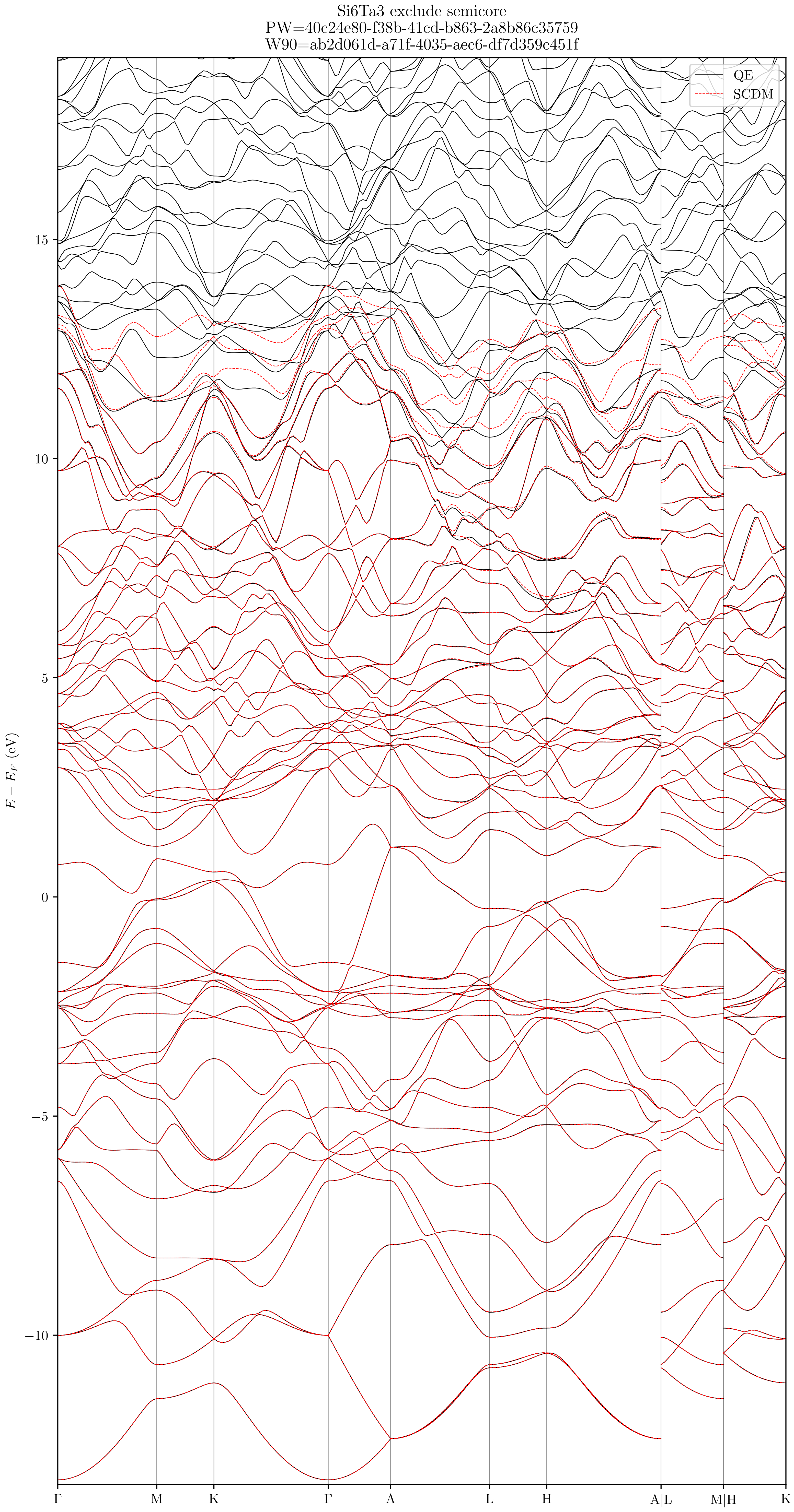

SCDM band distance (meV) and τ ($\AA$)

η_0	η_2	η_4	$\eta_0^{\max}$	$\eta_2^{\max}$	$\eta_4^{\max}$	τ
3.4	3.4	3.7	15.4	19.7	19.7	2.678

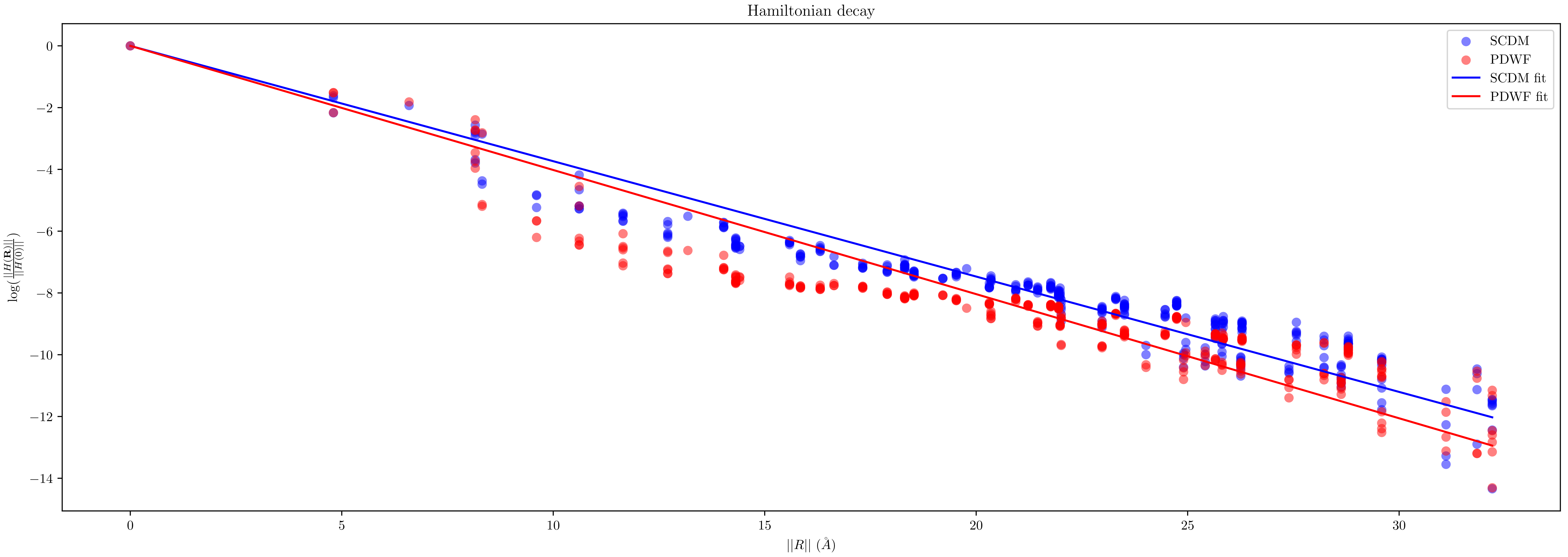

Si6Y10 exclude semicore
PW=d346891d-e361-40f7-86b8-d130f73745f7
W90=8c600b2d-a991-4df5-86a7-c1e78ace10dc

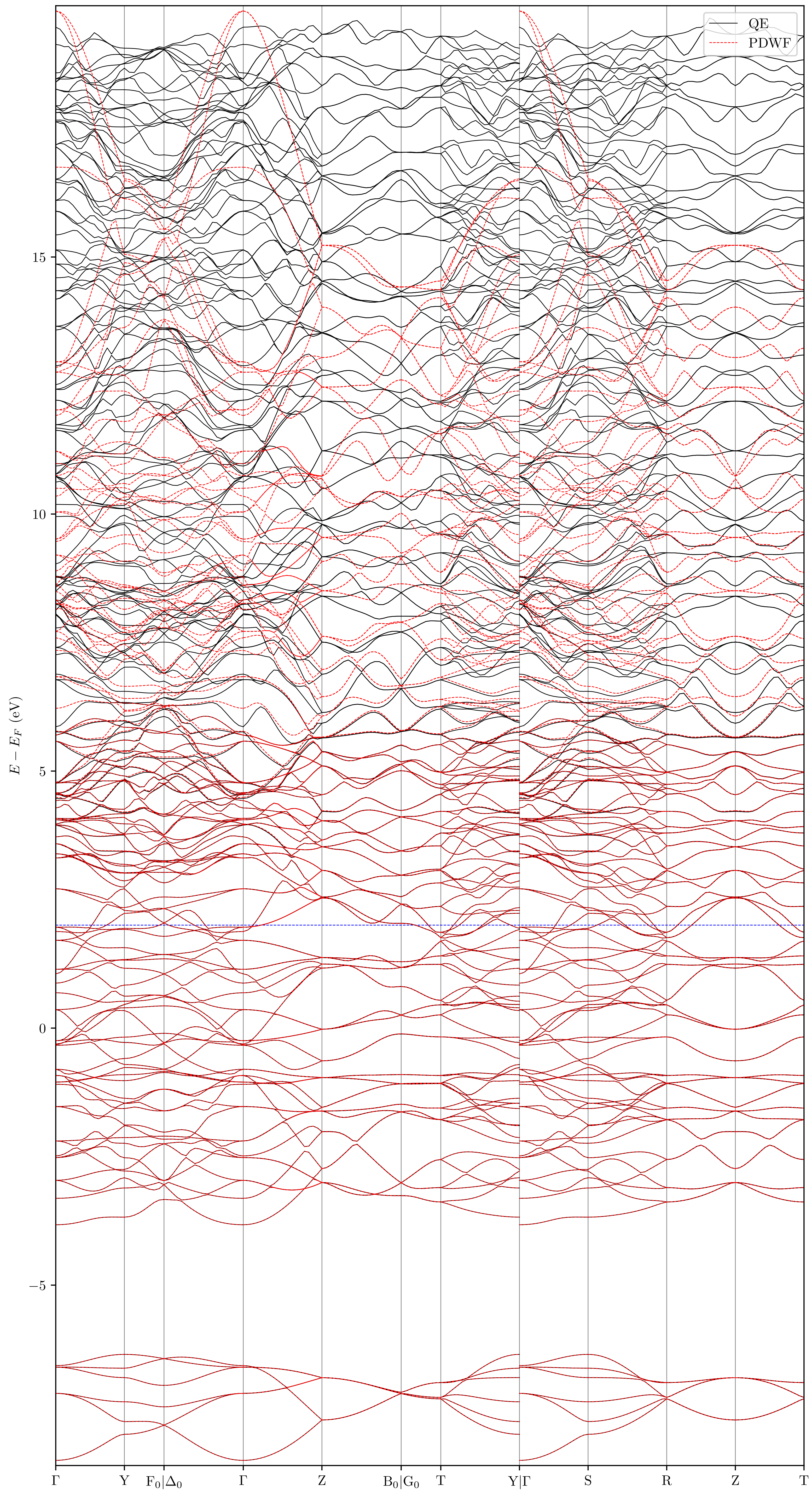

PDWF band distance (meV) and τ (Å)

η_0	η_2	η_4	$\eta_0^{\max}$	$\eta_2^{\max}$	$\eta_4^{\max}$	τ
0.9	0.9	1.4	4.8	4.8	16.0	2.710

Si6Y10 exclude semicore
PW=d346891d-e361-40f7-86b8-d130f73745f7
W90=b3363142-418d-4088-9900-a94029c9d259

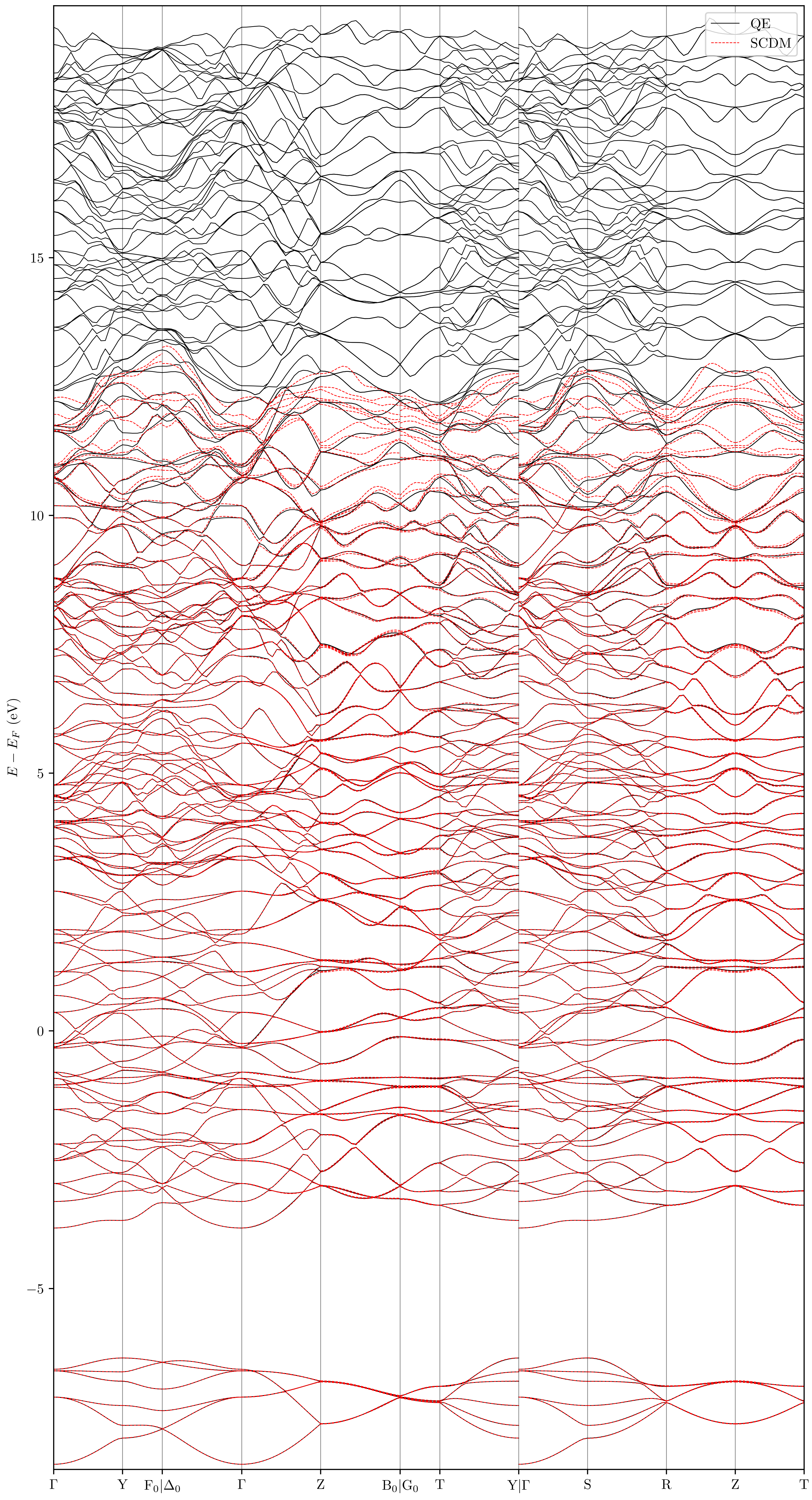

SCDM band distance (meV) and τ (Å)

η_0	η_2	η_4	$\eta_0^{\max}$	$\eta_2^{\max}$	$\eta_4^{\max}$	τ
5.3	5.9	6.0	24.2	37.8	37.8	3.457

Hamiltonian decay

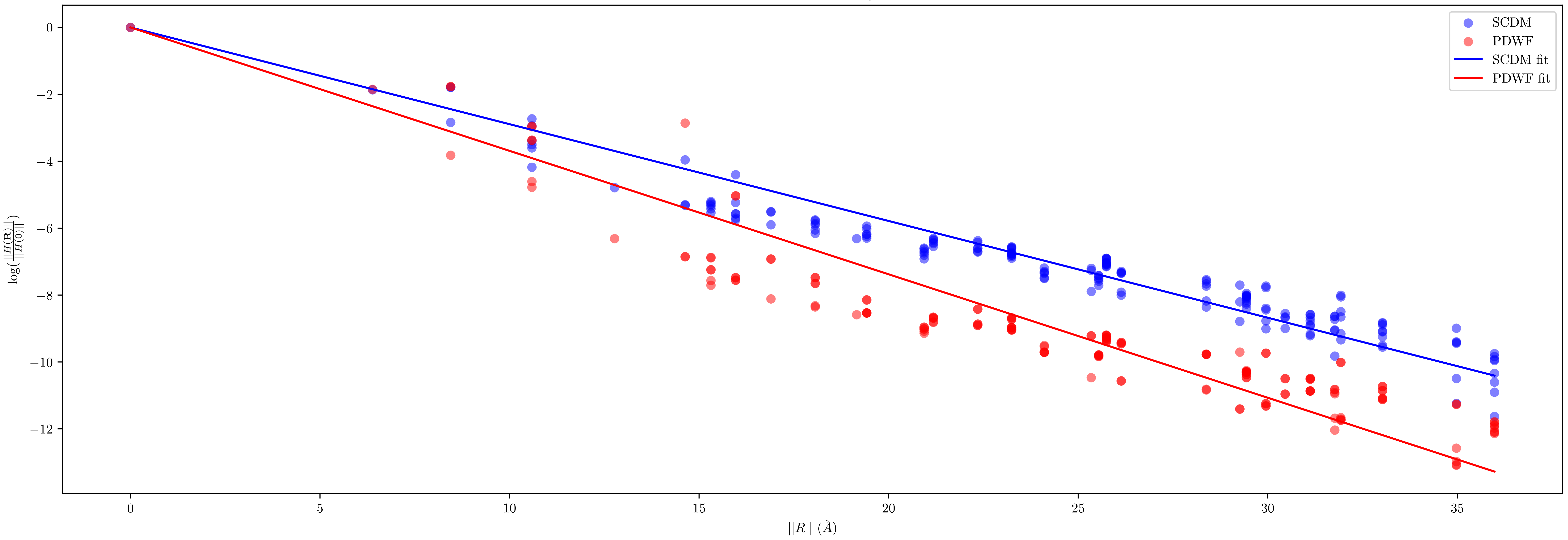

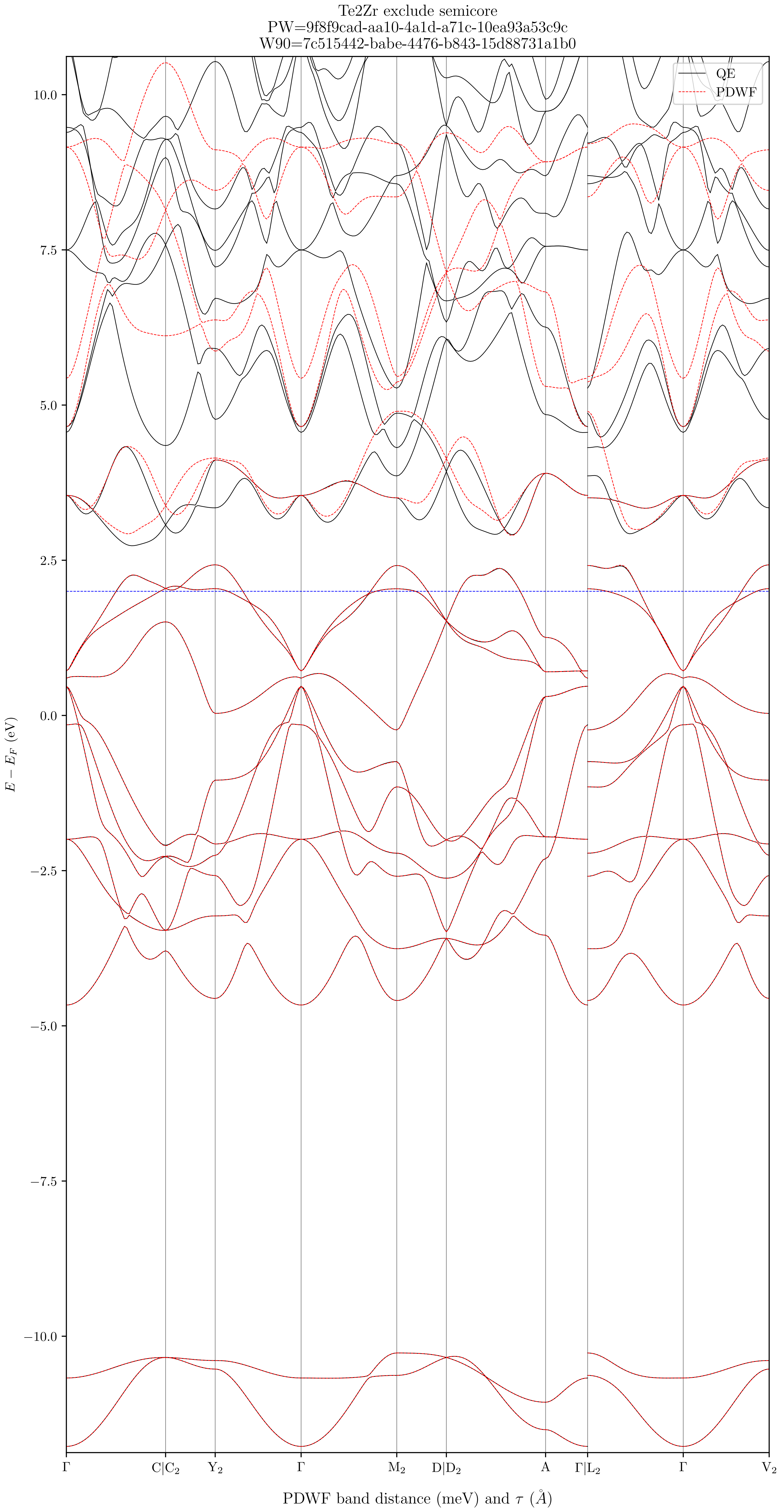

η_0	η_2	η_4	$\eta_0^{\max}$	$\eta_2^{\max}$	$\eta_4^{\max}$	τ
1.5	1.6	66.9	8.7	8.7	450.3	2.541

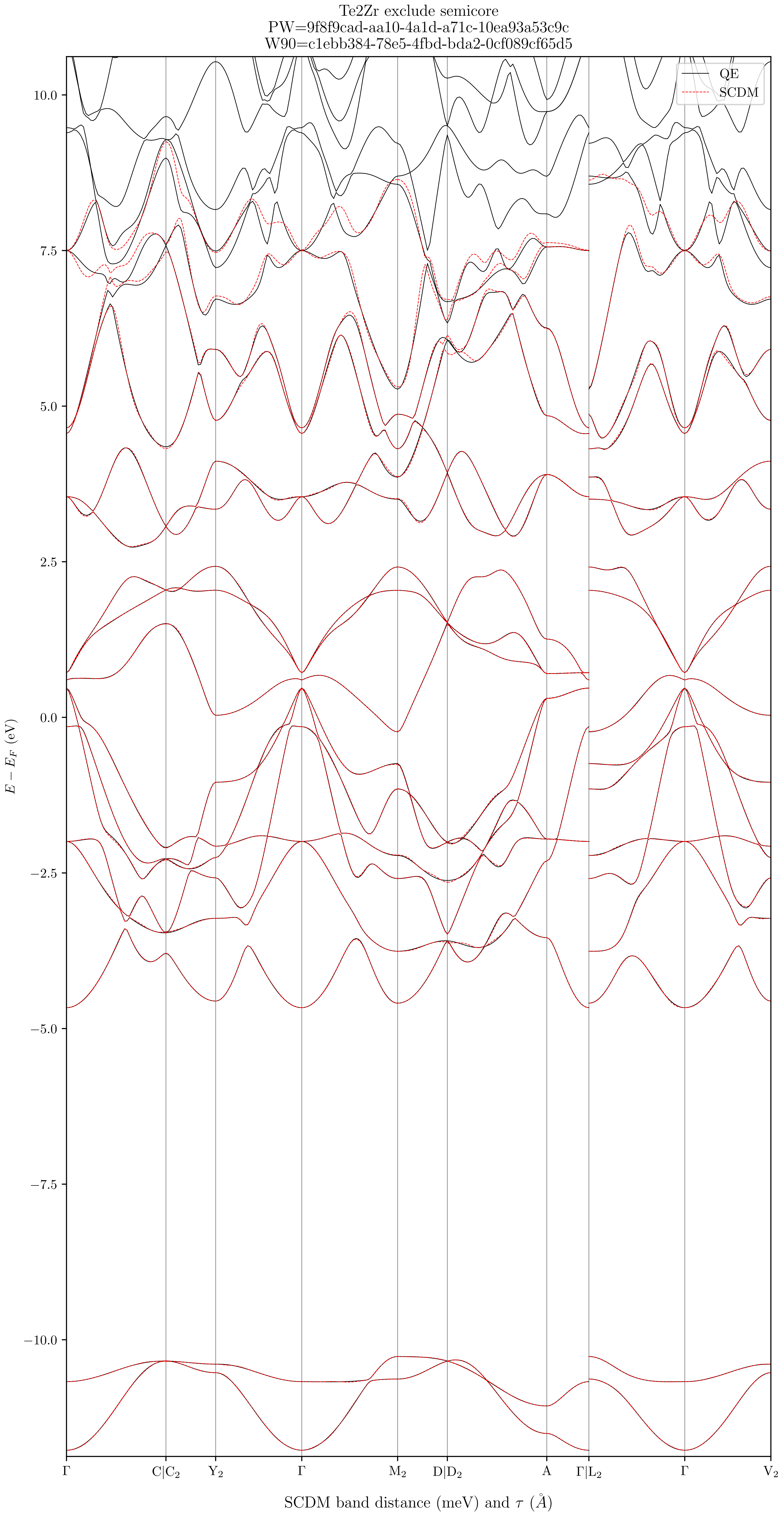

η_0	η_2	η_4	$\eta_0^{\max}$	$\eta_2^{\max}$	$\eta_4^{\max}$	τ
5.8	5.3	5.5	28.9	28.9	35.3	3.026

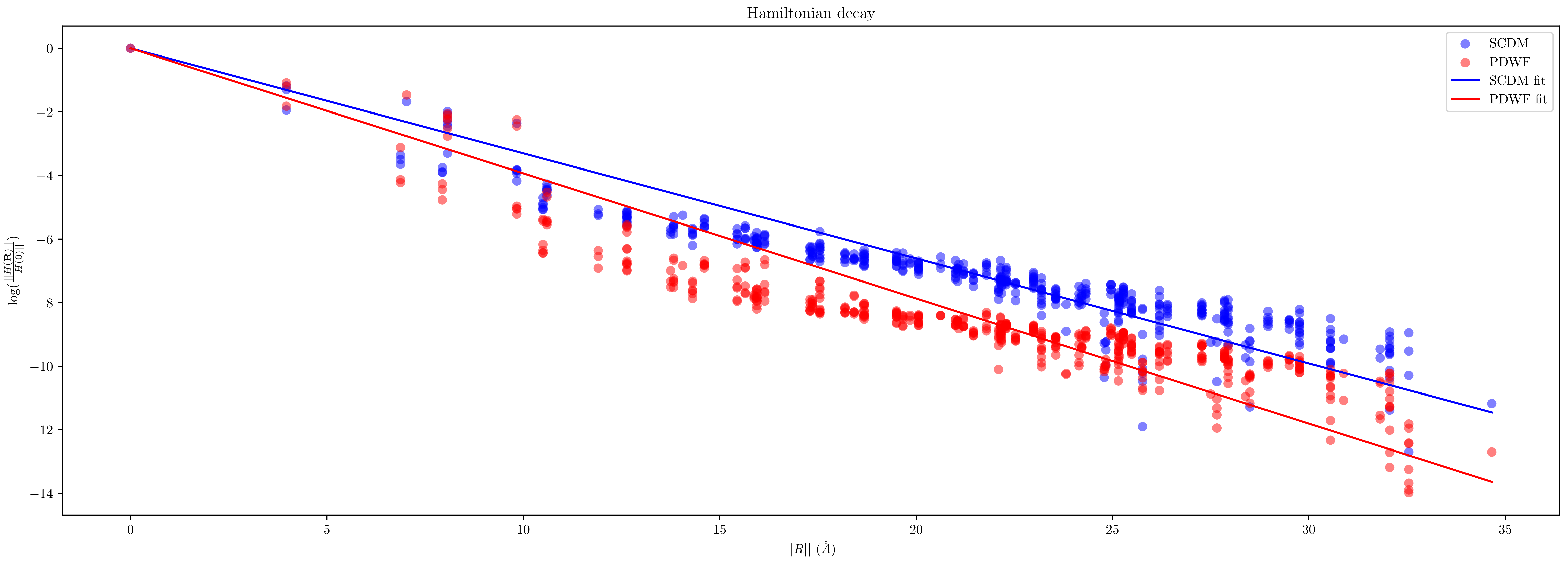

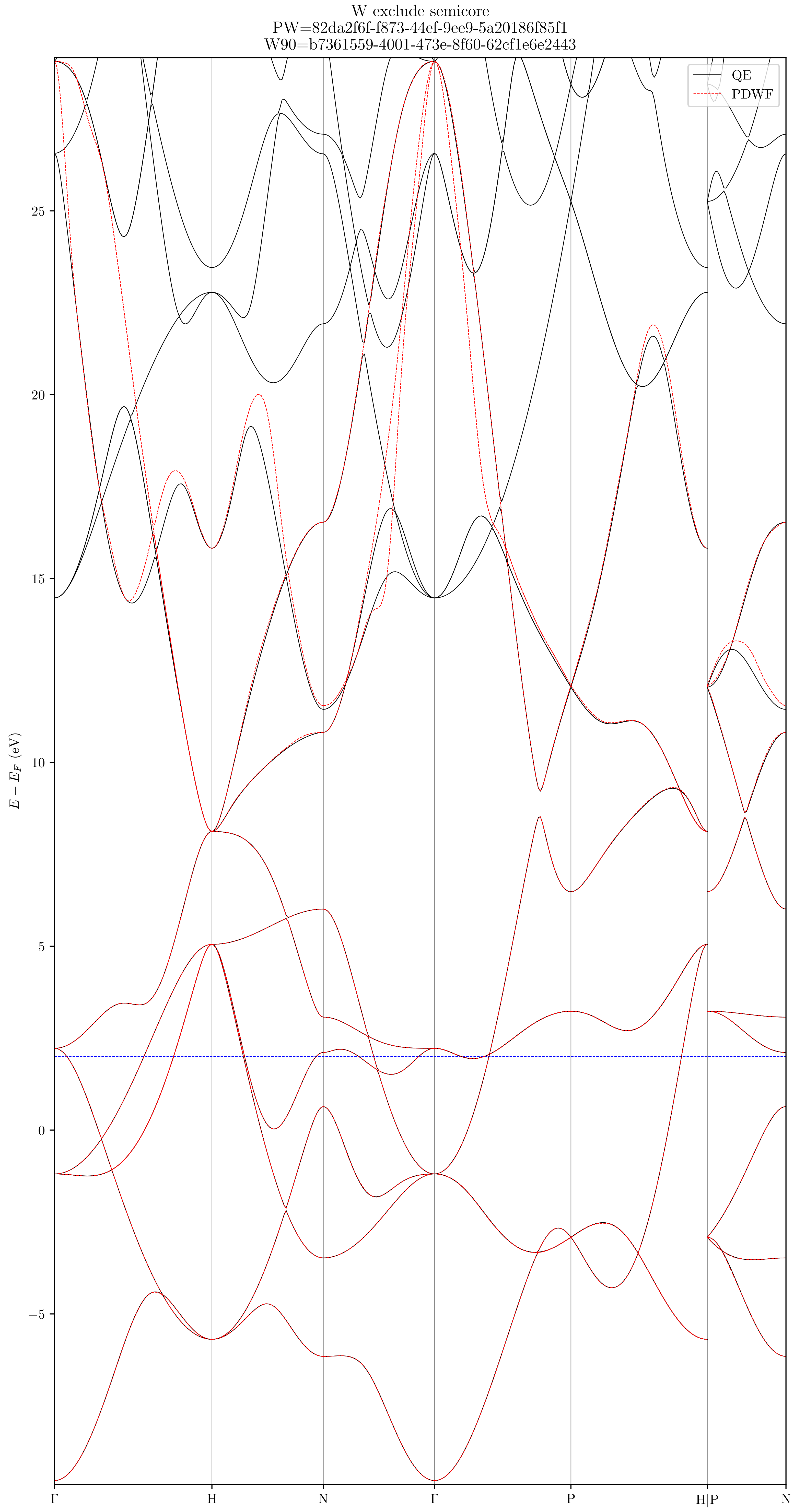

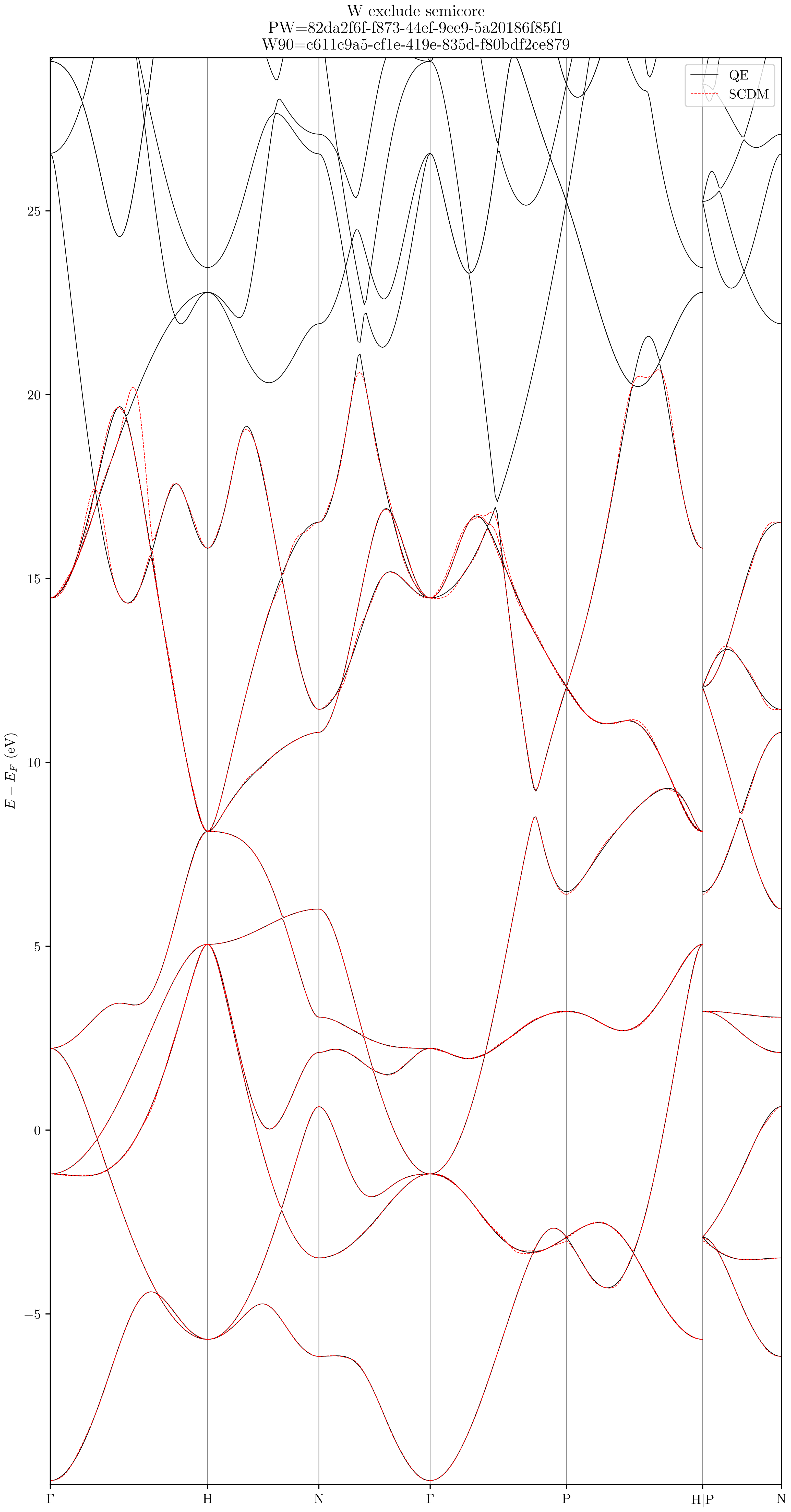

PDWF band distance (meV) and τ ($\AA$)

η_0	η_2	η_4	$\eta_0^{\max}$	$\eta_2^{\max}$	$\eta_4^{\max}$	τ
4.4	4.2	3.6	31.8	31.8	31.8	1.700

SCDM band distance (meV) and τ ($\AA$)

η_0	η_2	η_4	$\eta_0^{\max}$	$\eta_2^{\max}$	$\eta_4^{\max}$	τ
14.5	15.0	13.8	93.2	93.2	93.2	2.270

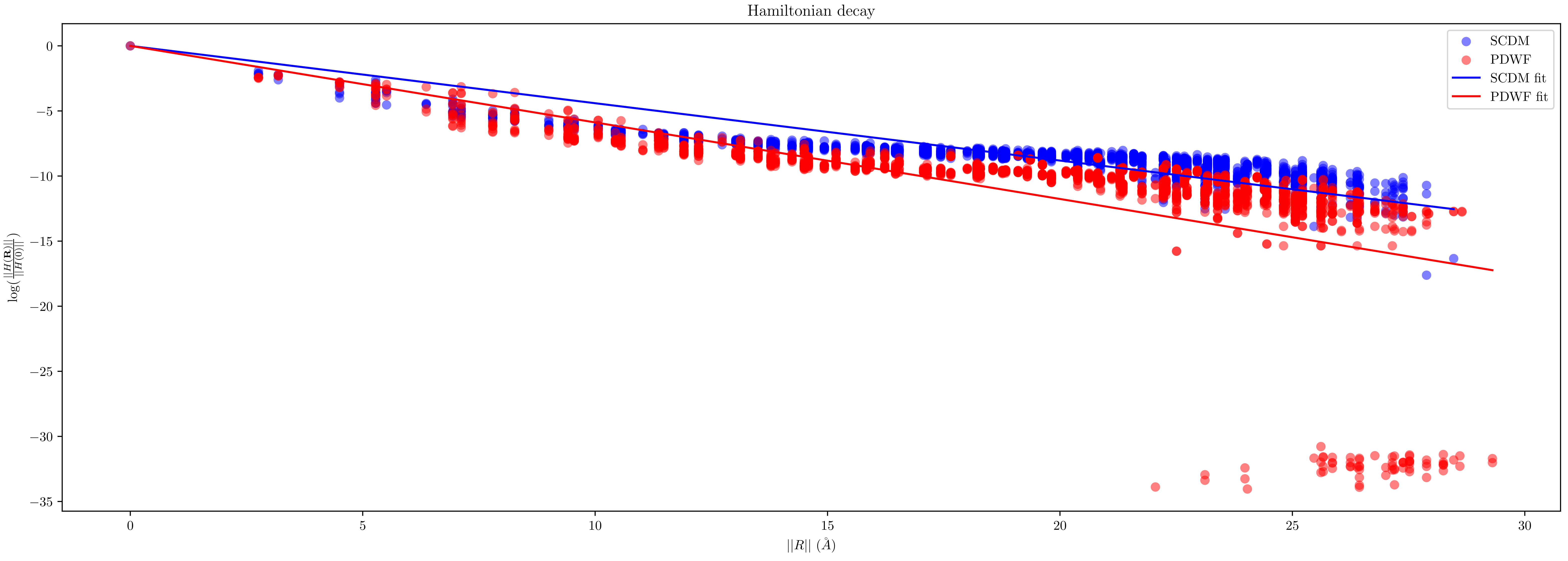

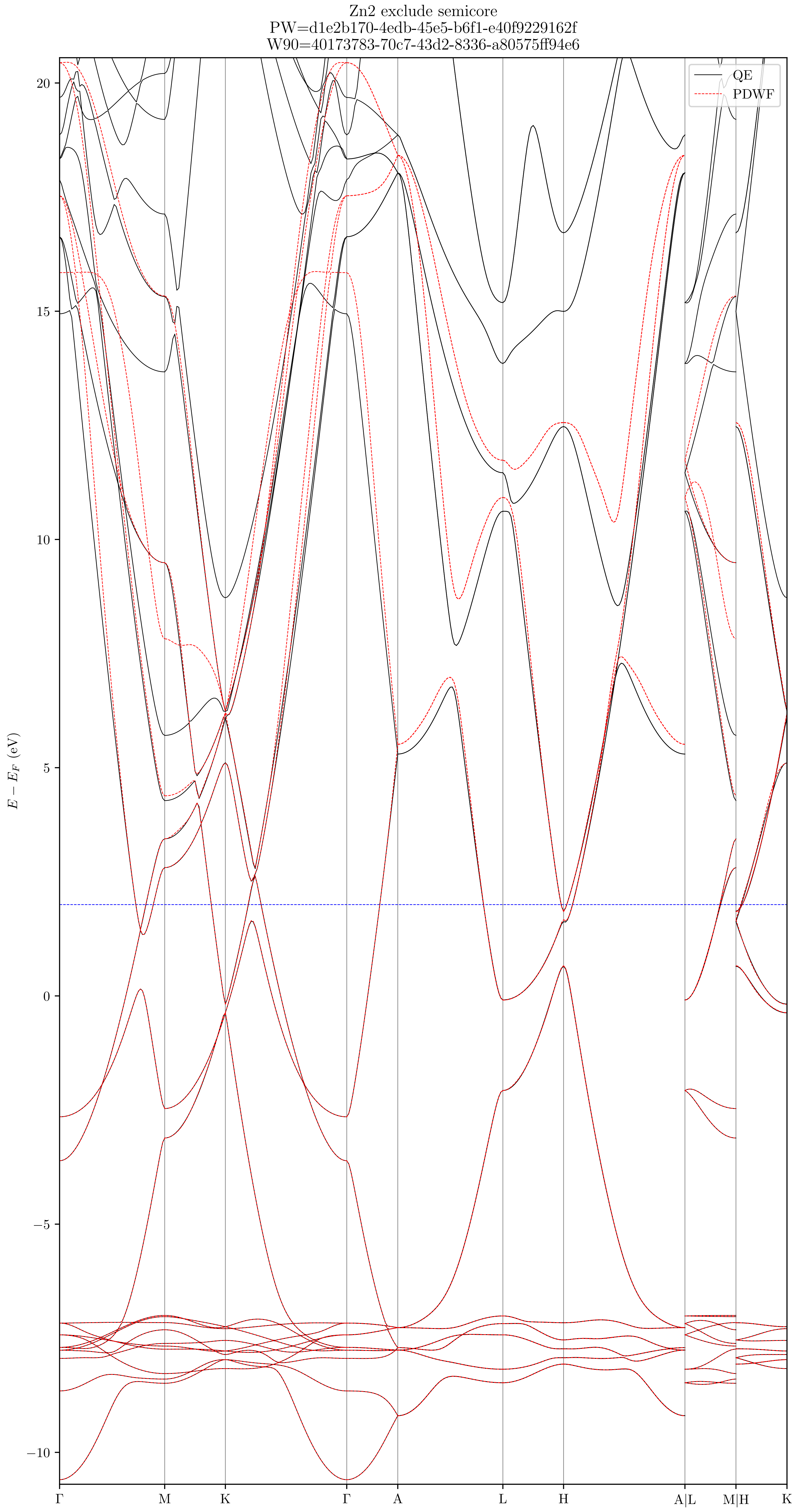

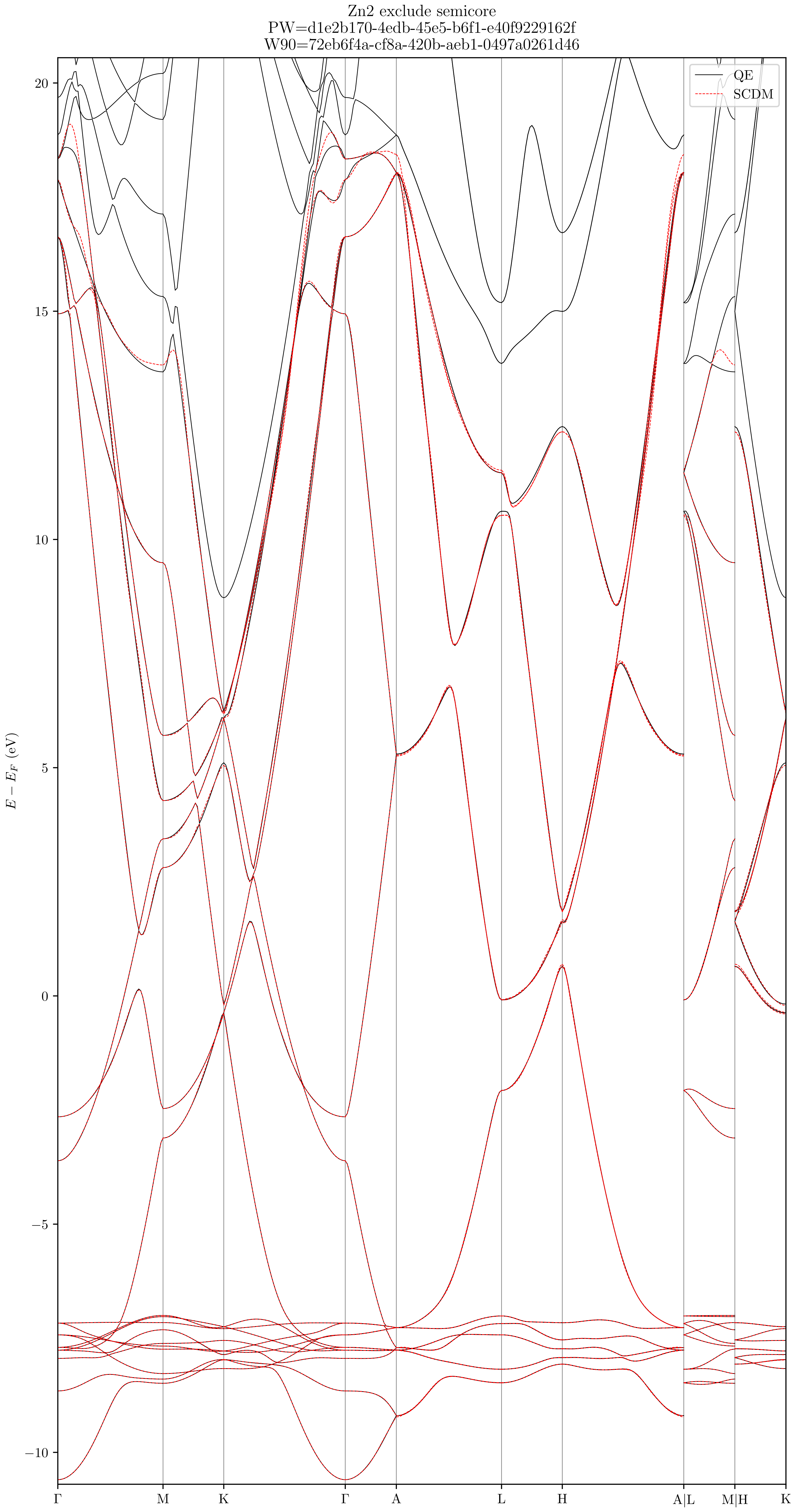

PDWF band distance (meV) and τ ($\AA$)

η_0	η_2	η_4	$\eta_0^{\max}$	$\eta_2^{\max}$	$\eta_4^{\max}$	τ
2.2	3.5	7.5	18.2	43.5	125.6	2.075

SCDM band distance (meV) and τ ($\AA$)

η_0	η_2	η_4	$\eta_0^{\max}$	$\eta_2^{\max}$	$\eta_4^{\max}$	τ
4.9	6.8	8.9	48.5	52.8	75.2	2.330

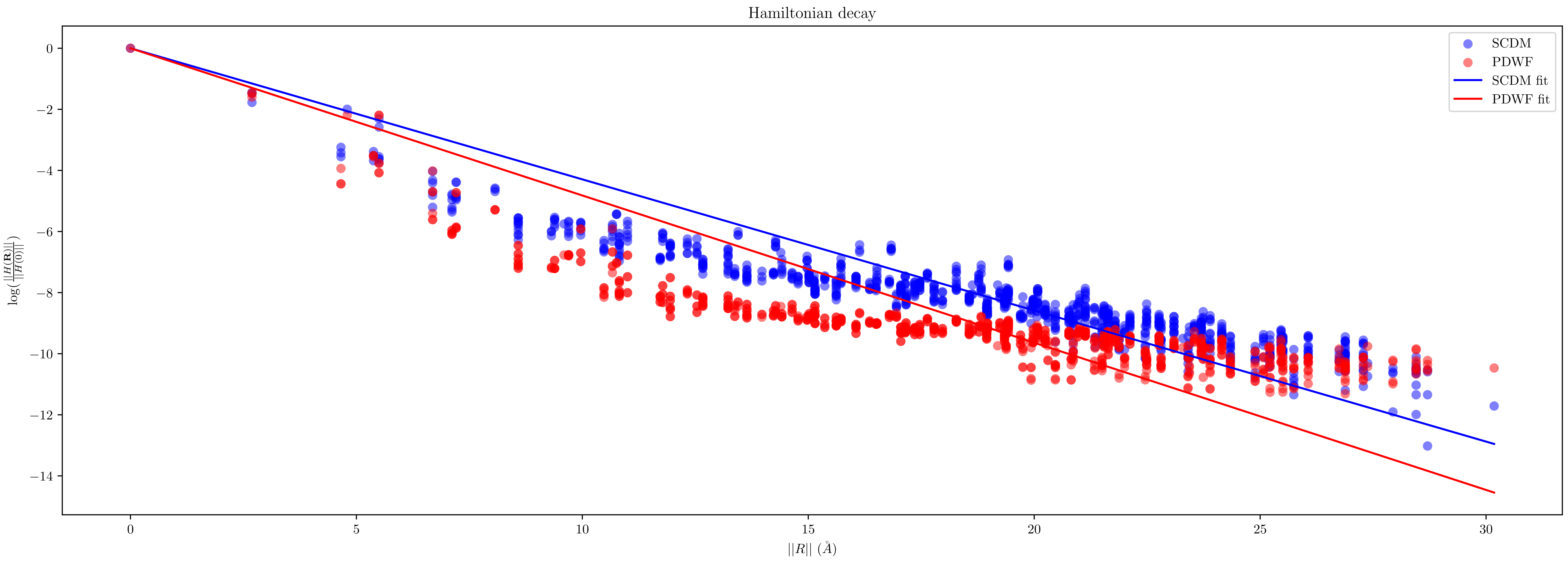

Supplement: Supplementary file 1 — Supplementary Information [file 41524_2023_1146_MOESM1_ESM.pdf]
